# Tracking development assistance for health and for COVID-19: a review of development assistance, government, out-of-pocket, and other private spending on health for 204 countries and territories, 1990–2050

**DOI:** 10.1016/S0140-6736(21)01258-7

**Published:** 2021-10-09

**Authors:** Global Burden of Disease 2020 Health Financing Collaborator Network, Angela E Micah, Ian E Cogswell, Brandon Cunningham, Satoshi Ezoe, Anton C Harle, Emilie R Maddison, Darrah McCracken, Shuhei Nomura, Kyle E Simpson, Hayley N Stutzman, Golsum Tsakalos, Lindsey E Wallace, Yingxi Zhao, Rahul R Zende, Cristiana Abbafati, Michael Abdelmasseh, Aidin Abedi, Kedir Hussein Abegaz, E S Abhilash, Hassan Abolhassani, Michael R M Abrigo, Tara Ballav Adhikari, Saira Afzal, Bright Opoku Ahinkorah, Sepideh Ahmadi, Haroon Ahmed, Muktar Beshir Ahmed, Tarik Ahmed Rashid, Marjan Ajami, Budi Aji, Yonas Akalu, Chisom Joyqueenet Akunna, Hanadi Al Hamad, Khurshid Alam, Fahad Mashhour Alanezi, Turki M Alanzi, Yosef Alemayehu, Robert Kaba Alhassan, Cyrus Alinia, Syed Mohamed Aljunid, Sami Almustanyir Almustanyir, Nelson Alvis-Guzman, Nelson J Alvis-Zakzuk, Saeed Amini, Mostafa Amini-Rarani, Hubert Amu, Robert Ancuceanu, Catalina Liliana Andrei, Tudorel Andrei, Blake Angell, Mina Anjomshoa, Carl Abelardo T Antonio, Catherine M Antony, Muhammad Aqeel, Jalal Arabloo, Morteza Arab-Zozani, Timur Aripov, Alessandro Arrigo, Tahira Ashraf, Desta Debalkie Atnafu, Marcel Ausloos, Leticia Avila-Burgos, Asma Tahir Awan, Getinet Ayano, Martin Amogre Ayanore, Samad Azari, Gulrez Shah Azhar, Tesleem Kayode Babalola, Mohammad Amin Bahrami, Atif Amin Baig, Maciej Banach, Nastaran Barati, Till Winfried Bärnighausen, Amadou Barrow, Sanjay Basu, Bernhard T Baune, Mohsen Bayati, Habib Benzian, Adam E Berman, Akshaya Srikanth Bhagavathula, Nikha Bhardwaj, Pankaj Bhardwaj, Sonu Bhaskar, Sadia Bibi, Ali Bijani, Virginia Bodolica, Nicola Luigi Bragazzi, Dejana Braithwaite, Nicholas J K Breitborde, Alexey V Breusov, Nikolay Ivanovich Briko, Reinhard Busse, Lucero Cahuana-Hurtado, Emily Joy Callander, Luis Alberto Cámera, Carlos A Castañeda-Orjuela, Ferrán Catalá-López, Jaykaran Charan, Souranshu Chatterjee, Soosanna Kumary Chattu, Vijay Kumar Chattu, Simiao Chen, Arrigo Francesco Giuseppe Cicero, Omid Dadras, Saad M A Dahlawi, Xiaochen Dai, Koustuv Dalal, Lalit Dandona, Rakhi Dandona, Dragos Virgil Davitoiu, Jan-Walter De Neve, Antonio Reis de Sá-Junior, Edgar Denova-Gutiérrez, Deepak Dhamnetiya, Samath Dhamminda Dharmaratne, Leila Doshmangir, John Dube, Elham Ehsani-Chimeh, Maysaa El Sayed Zaki, Maha El Tantawi, Sharareh Eskandarieh, Farshad Farzadfar, Tomas Y Ferede, Florian Fischer, Nataliya A Foigt, Alberto Freitas, Sara D Friedman, Takeshi Fukumoto, Nancy Fullman, Peter Andras Gaal, Mohamed M Gad, MA Garcia-Gordillo, Tushar Garg, Mansour Ghafourifard, Ahmad Ghashghaee, Asadollah Gholamian, Ali Gholamrezanezhad, Ghozali Ghozali, Syed Amir Gilani, Ionela-Roxana Glăvan, Ekaterina Vladimirovna Glushkova, Salime Goharinezhad, Mahaveer Golechha, Srinivas Goli, Avirup Guha, Veer Bala Gupta, Vivek Kumar Gupta, Annie Haakenstad, Mohammad Rifat Haider, Alemayehu Hailu, Samer Hamidi, Asif Hanif, Harapan Harapan, Risky Kusuma Hartono, Ahmed I Hasaballah, Shoaib Hassan, Mohamed H Hassanein, Khezar Hayat, Mohamed I Hegazy, Golnaz Heidari, Delia Hendrie, Ileana Heredia-Pi, Claudiu Herteliu, Kamal Hezam, Ramesh Holla, Sheikh Jamal Hossain, Mehdi Hosseinzadeh, Sorin Hostiuc, Tanvir M Huda, Bing-Fang Hwang, Ivo Iavicoli, Bulat Idrisov, Olayinka Stephen Ilesanmi, Seyed Sina Naghibi Irvani, Sheikh Mohammed Shariful Islam, Nahlah Elkudssiah Ismail, Gaetano Isola, Mohammad Ali Jahani, Nader Jahanmehr, Mihajlo Jakovljevic, Manthan Dilipkumar Janodia, Tahereh Javaheri, Sathish Kumar Jayapal, Ranil Jayawardena, Seyed Behzad Jazayeri, Ravi Prakash Jha, Jost B Jonas, Tamas Joo, Farahnaz Joukar, Mikk Jürisson, Billingsley Kaambwa, Rohollah Kalhor, Tanuj Kanchan, Himal Kandel, Behzad Karami Matin, Salah Eddin Karimi, Getinet Kassahun, Gbenga A Kayode, Ali Kazemi Karyani, Leila Keikavoosi-Arani, Yousef Saleh Khader, Himanshu Khajuria, Rovshan Khalilov, Mohammad Khammarnia, Junaid Khan, Jagdish Khubchandani, Neda Kianipour, Gyu Ri Kim, Yun Jin Kim, Adnan Kisa, Sezer Kisa, Stefan Kohler, Soewarta Kosen, Rajasekaran Koteeswaran, Sindhura Lakshmi Koulmane Laxminarayana, Ai Koyanagi, Kewal Krishan, G Anil Kumar, Dian Kusuma, Demetris Lamnisos, Van Charles Lansingh, Anders O Larsson, Savita Lasrado, Long Khanh Dao Le, Shaun Wen Huey Lee, Yeong Yeh Lee, Stephen S Lim, Stany W Lobo, Rafael Lozano, Hassan Magdy Abd El Razek, Muhammed Magdy Abd El Razek, Mokhtar Mahdavi Mahdavi, Azeem Majeed, Alaa Makki, Afshin Maleki, Reza Malekzadeh, Ana Laura Manda, Fariborz Mansour-Ghanaei, Mohammad Ali Mansournia, Carlos Alberto Marrugo Arnedo, Adolfo Martinez-Valle, Seyedeh Zahra Masoumi, Richard James Maude, Martin McKee, Carlo Eduardo Medina-Solís, Ritesh G Menezes, Atte Meretoja, Tuomo J Meretoja, Mohamed Kamal Mesregah, Tomislav Mestrovic, Neda Milevska Kostova, Ted R Miller, GK Mini, Andreea Mirica, Erkin M Mirrakhimov, Bahram Mohajer, Teroj Abdulrahman Mohamed, Mokhtar Mohammadi, Abdollah Mohammadian-Hafshejani, Shafiu Mohammed, Modhurima Moitra, Ali H Mokdad, Mariam Molokhia, Mohammad Ali Moni, Yousef Moradi, Jakub Morze, Seyyed Meysam Mousavi, Christine Mpundu-Kaambwa, Moses K Muriithi, Saravanan Muthupandian, Ahamarshan Jayaraman Nagarajan, Mukhammad David Naimzada, Vinay Nangia, Atta Abbas Naqvi, Aparna Ichalangod Narayana, Bruno Ramos Nascimento, Muhammad Naveed, Biswa Prakash Nayak, Javad Nazari, Rawlance Ndejjo, Ionut Negoi, Sandhya Neupane Kandel, Trang Huyen Nguyen, Justice Nonvignon, Jean Jacques Noubiap, Vincent Ebuka Nwatah, Bogdan Oancea, Foluke Adetola Ogunyemi Ojelabi, Andrew T Olagunju, Babayemi Oluwaseun Olakunde, Stefano Olgiati, Jacob Olusegun Olusanya, Obinna E Onwujekwe, Adrian Otoiu, Nikita Otstavnov, Stanislav S Otstavnov, Mayowa O Owolabi, Jagadish Rao Padubidri, Raffaele Palladino, Songhomitra Panda-Jonas, Eun-Cheol Park, Fatemeh Pashazadeh Kan, Shrikant Pawar, Hamidreza Pazoki Toroudi, David M Pereira, Arokiasamy Perianayagam, Konrad Pesudovs, Cristiano Piccinelli, Maarten J Postma, Sergio I Prada, Mohammad Rabiee, Navid Rabiee, Fakher Rahim, Vafa Rahimi-Movaghar, Mohammad Hifz Ur Rahman, Mosiur Rahman, Amir Masoud Rahmani, Usha Ram, Chhabi Lal Ranabhat, Priyanga Ranasinghe, Chythra R Rao, Priya Rathi, David Laith Rawaf, Salman Rawaf, Lal Rawal, Reza Rawassizadeh, Robert C Reiner Jr, Andre M N Renzaho, Bhageerathy Reshmi, Mavra A Riaz, Rezaul Karim Ripon, Anas M Saad, Mohammad Ali Sahraian, Maitreyi Sahu, Joseph S Salama, Sana Salehi, Abdallah M Samy, Juan Sanabria, Francesco Sanmarchi, João Vasco Santos, Milena M Santric-Milicevic, Brijesh Sathian, Miloje Savic, Deepak Saxena, Mehdi Sayyah, Falk Schwendicke, Subramanian Senthilkumaran, Sadaf G Sepanlou, Allen Seylani, Saeed Shahabi, Masood Ali Shaikh, Aziz Sheikh, Adithi Shetty, Pavanchand H Shetty, Kenji Shibuya, Mark G Shrime, Kanwar Hamza Shuja, Jasvinder A Singh, Valentin Yurievich Skryabin, Anna Aleksandrovna Skryabina, Shahin Soltani, Moslem Soofi, Emma Elizabeth Spurlock, Simona Cătălina Stefan, Viktória Szerencsés, Miklós Szócska, Rafael Tabarés-Seisdedos, Biruk Wogayehu Taddele, Yonas Getaye Tefera, Aravind Thavamani, Ruoyan Tobe-Gai, Roman Topor-Madry, Marcos Roberto Tovani-Palone, Bach Xuan Tran, Lorainne Tudor Car, Anayat Ullah, Saif Ullah, Nasir Umar, Eduardo A Undurraga, Pascual R Valdez, Tommi Juhani Vasankari, Jorge Hugo Villafañe, Francesco S Violante, Vasily Vlassov, Bay Vo, Sebastian Vollmer, Theo Vos, Giang Thu Vu, Linh Gia Vu, Richard G Wamai, Andrea Werdecker, Mesfin Agachew Woldekidan, Befikadu Legesse Wubishet, Gelin Xu, Sanni Yaya, Vahid Yazdi-Feyzabadi, Vahit Yiğit, Paul Yip, Birhanu Wubale Yirdaw, Naohiro Yonemoto, Mustafa Z Younis, Chuanhua Yu, Ismaeel Yunusa, Telma Zahirian Moghadam, Hamed Zandian, Mikhail Sergeevich Zastrozhin, Anasthasia Zastrozhina, Zhi-Jiang Zhang, Arash Ziapour, Yves Miel H Zuniga, Simon I Hay, Christopher J L Murray, Joseph L Dieleman

## Abstract

**Background:**

The rapid spread of COVID-19 renewed the focus on how health systems across the globe are financed, especially during public health emergencies. Development assistance is an important source of health financing in many low-income countries, yet little is known about how much of this funding was disbursed for COVID-19. We aimed to put development assistance for health for COVID-19 in the context of broader trends in global health financing, and to estimate total health spending from 1995 to 2050 and development assistance for COVID-19 in 2020.

**Methods:**

We estimated domestic health spending and development assistance for health to generate total health-sector spending estimates for 204 countries and territories. We leveraged data from the WHO Global Health Expenditure Database to produce estimates of domestic health spending. To generate estimates for development assistance for health, we relied on project-level disbursement data from the major international development agencies' online databases and annual financial statements and reports for information on income sources. To adjust our estimates for 2020 to include disbursements related to COVID-19, we extracted project data on commitments and disbursements from a broader set of databases (because not all of the data sources used to estimate the historical series extend to 2020), including the UN Office of Humanitarian Assistance Financial Tracking Service and the International Aid Transparency Initiative. We reported all the historic and future spending estimates in inflation-adjusted 2020 US$, 2020 US$ per capita, purchasing-power parity-adjusted US$ per capita, and as a proportion of gross domestic product. We used various models to generate future health spending to 2050.

**Findings:**

In 2019, health spending globally reached $8·8 trillion (95% uncertainty interval [UI] 8·7–8·8) or $1132 (1119–1143) per person. Spending on health varied within and across income groups and geographical regions. Of this total, $40·4 billion (0·5%, 95% UI 0·5–0·5) was development assistance for health provided to low-income and middle-income countries, which made up 24·6% (UI 24·0–25·1) of total spending in low-income countries. We estimate that $54·8 billion in development assistance for health was disbursed in 2020. Of this, $13·7 billion was targeted toward the COVID-19 health response. $12·3 billion was newly committed and $1·4 billion was repurposed from existing health projects. $3·1 billion (22·4%) of the funds focused on country-level coordination and $2·4 billion (17·9%) was for supply chain and logistics. Only $714·4 million (7·7%) of COVID-19 development assistance for health went to Latin America, despite this region reporting 34·3% of total recorded COVID-19 deaths in low-income or middle-income countries in 2020. Spending on health is expected to rise to $1519 (1448–1591) per person in 2050, although spending across countries is expected to remain varied.

**Interpretation:**

Global health spending is expected to continue to grow, but remain unequally distributed between countries. We estimate that development organisations substantially increased the amount of development assistance for health provided in 2020. Continued efforts are needed to raise sufficient resources to mitigate the pandemic for the most vulnerable, and to help curtail the pandemic for all.

**Funding:**

Bill & Melinda Gates Foundation.

## Introduction

How much countries spend on health has long been of interest for relevance to a range of social and economic objectives, including the goal of providing essential health services and universal health coverage.^1^, ^2^, ^3^, ^4^, ^5^, ^6^ The COVID-19 pandemic has renewed interest in the past, present, and future of global health financing, in part because responding to the COVID-19 pandemic has been and continues to be tremendously costly. Governments around the world implemented restrictions on travel and mass gatherings; required masks and quarantines; and rolled out and ramped up access to COVID-19 testing, contact tracing, and, when possible, COVID-19 treatment.^7^, ^8^ Governments have fought to secure access to the first round of approved COVID-19 vaccines, with vaccination campaigns beginning in more than 30 countries in 2020, including China, Israel, Russia, Mexico, the USA, and the UK.^9^, ^10^, ^11^

**Research in context**

**Evidence before this study**

The Global Burden of Disease Health Financing Collaborator Network and WHO have each published annual estimates of global health spending, with the former also publishing estimates of future health spending. So far, few research efforts have provided estimates of health spending associated with the COVID-19 pandemic, and these vary in their scope and completeness. The Economist Intelligence Unit's COVID-19 Health Funding Tracker provides visualisations of pledged and disbursed funding for pandemic response, although it does not include international commitments to individual countries or pledges less than US$2 million. The Kaiser Family Foundation Donor Funding for the Global Novel Coronavirus Response tracker is a tabulated database of funding pledged for the global response to COVID-19. This tracker has not been updated since April, 2020, and does not include resources for research and development or in-kind support. The Centre for Disaster Protection's database tracks funding from multilateral agencies and regional development banks for global humanitarian and development needs, but does not capture bilateral or private contributions. Devex's interactive website tracks funding committed toward addressing the health, social, economic, and environmental effects of the pandemic, but does not provide information on the disbursements of these funds. The COVID-19 Research Project tracker from the UK Collaborative on Development Research and the Global Research Collaboration for Infectious Disease Preparedness collates data on all research and development project commitments and maps these to WHO research priorities. Although these existing studies track various aspects of the funding targeted toward the pandemic response, none reports comprehensive tracking for the health sector.

**Added value of this study**

We track spending on health globally, providing estimates from 1990 to 2050. We focus mainly on novel estimates of development assistance for health for COVID-19 in 2020 and the ramifications that follow for funding other essential health areas. These estimates add value to trackers by focusing on 2020 disbursements, being as comprehensive as possible, and placing development assistance for health for COVID-19 in the context of past spending and projected future health spending.

**Implications of all the available evidence**

Since 1995, global health spending has increased constantly, but inequalities in spending remain. Our estimates suggest that although contributions have been forthcoming, there remains a gap in funding needed to fully address the effects of the pandemic in most low-income and middle-income countries, especially as vaccine roll-outs are initiated globally. Continued efforts are needed to raise the required resources to provide health care for the pandemic—prevention and treatment—for the most vulnerable. Beyond COVID-19, projected disparities in future health spending across income groups suggest an ongoing benefit of leveraging development assistance resources in promoting an equitable response to any future health emergencies.

Funding the pandemic response has been complicated by global economic recession, which has not spared low-income and middle-income countries.^12^ It was estimated that the global economy shrank by 3·3% in 2020, and the economies of low-income and middle-income countries (excluding China) shrank by 4·3%.^13^ Uncertainty is expected to linger as third and fourth waves sweep the globe and new COVID-19 variants spread with increasing speed.^14^, ^15^ Unemployment increased globally, and extreme poverty is estimated to have increased by between 88 and 115 million in 2020.^16^ Moreover, the economic consequences of the health crisis are leading to long-standing reductions in economic development in some countries, and further indirect adverse effects on health.^4^, ^17^, ^18^, ^19^, ^20^, ^21^

The response to the dual health and economic crises caused by COVID-19 generated substantial costs across countries. High-income countries and many middle-income countries have been able to finance their government programmes with financial reserves, reallocation of government resources, and by borrowing. The International Monetary Fund (IMF) estimates that globally, government debt increased by $20 trillion between Sept 1, 2019, and Sept 1, 2020.^22^ Although some low-income countries have also borrowed resources (obtaining loans at market rates) to fund their responses to the 2020 crises, development assistance plays a unique role in funding health systems during emergencies in many countries and can be a catalyst for rapidly scaling up novel health services.^23^

We estimated development assistance for health for COVID-19 in 2020, and assessed how that assistance compared with broader trends in health financing. We first present retrospective estimates of domestic spending on health and development assistance for health to provide context on the broader health financing landscape. Then we focus on development assistance for health and how it was used to target COVID-19 in 2020, and ramifications for development assistance for health funding in other essential health areas. We disaggregate estimates for development assistance for health for COVID in 2020 by funding sources, disbursing agencies, recipients, and programme areas to enable comparison with other key focus areas such as HIV/AIDS, health system strengthening, and pandemic preparedness. Finally, we generate estimates of future health spending to enable an assessment of the implications for future health financing.^24^, ^25^ Comprehensive health spending estimates are important for examining potential gaps in resource needs versus available funding.

## Methods

### Overview

Health spending can be disaggregated into four key financing sources: government, out-of-pocket, and prepaid private health spending, which collectively make up domestic health spending; and development assistance for health, which includes international disbursements for health to low-income and middle-income countries. Government health spending includes social health insurance and government public health programmes. Out-of-pocket health spending includes health-care spending by a patient or their household but excludes insurance premiums. Prepaid private health spending includes private insurance spending and spending by non-governmental agencies on health.

To estimate total health spending for 204 countries and territories, we estimated each of the four financing sources separately for the years that underlying data were available, and used past trends and relationships to forecast each financing source from the point where retrospective estimates end to the end of 2050. The primary data source for domestic spending financing streams was the WHO Global Health Expenditure Database (GHED), and retrospective estimates were made from 1995 to 2018. Development assistance for health was estimated by use of a diverse set of project and agency expenditure and revenue statements, and estimates extend from 1990 to 2020, with additional project-level databases used to generate special estimates of development assistance for health for COVID-19 in 2020 (appendix pp 26, 91). Forecasts for each financing source begin in 2019 or 2021. All estimates are inflation-adjusted and are mostly reported in 2020 US$, although some were adjusted for national prices and are thus reported in purchasing-power parity-adjusted US$ or relative to gross domestic product (GDP).

### Estimating domestic health spending, 1995–2018

For government, out-of-pocket, and prepaid private health spending, we downloaded data from the GHED for all available countries in current national currency units.^26^ We adjusted these estimates for inflation, converted to 2020 US$, modelled estimates to ensure consistency over time and comprehensiveness across countries and territories, and estimated uncertainty (appendix p 21). We also converted these estimates into 2020 purchasing-power parity-adjusted US$. We used deflator series and exchange rate data based on data from the IMF World Economic Outlook.^14^

For each of the three domestic financing sources, we used the metadata provided by WHO to qualitatively assess the reliability of data extracted from the GHED. We assigned a weight to each downloaded datapoint according to the documented source information included in the metadata, completeness of metadata, and documented methods of estimation (appendix p 13). We then used a spatiotemporal Gaussian process model to generate a complete time series of data from 1995 until 2018 for each country, and calculated 95% uncertainty intervals (UIs).^27^, ^28^

### Estimating development assistance for health, 1990–2020

Development assistance for health refers to the financial and non-financial resources that are disbursed through international development agencies to maintain or improve health in low-income and middle-income countries. We tracked these disbursements from their originating sources through their disbursing agencies to the health focus areas that these resources were designed to target in recipient countries. Originating sources were typically the national treasuries of donor governments or private philanthropies from which development assistance funds are transferred. The funds from originating sources are channelled through international development agencies, which are here referred to as the disbursing agency, before being disbursed to the recipient country. We relied on project-level disbursement data from major international development agencies' online databases, including the Organisation for Economic Cooperation and Development's Creditor Reporting System (OECD CRS), the Global Fund to Fight AIDS, Tuberculosis and Malaria (the Global Fund), and annual financial statements and reports for information on income sources. Data were not yet available for some more recent years, and we relied on budget data to generate these estimates. Detailed explanation of how the disbursements were estimated for each disbursing agency is provided in the appendix (pp 47–80) and published elsewhere.^5^, ^29^, ^30^, ^31^, ^32^, ^33^, ^34^, ^35^, ^36^ We disaggregated the estimates into ten health focus areas and 53 programme areas. This disaggregation captures the main programmatic areas to which development assistance for health contributions have historically been provided, and facilitates comparison with 2020 contributions and the international funding for the ongoing pandemic. We defined relevant health focus areas and programme areas for projects on the basis of a keyword search of the project descriptions downloaded from international agencies' online project databases. The specific keywords we used and their assigned health focus or programme area are detailed in the appendix (pp 35–46).

We leveraged information from available financial documents on revenue to remove double counting across disbursing agencies, so that each flow of funding is counted only once even if it was moved from one agency to another. Although OECD CRS completeness has improved over time, in earlier years (for this study, 1990 to 1996 especially) the data were less complete; thus, we used adjusted commitment data from the Development Assistance Committee tables to estimate disbursements. We also estimated the expenses associated with administering loans and grants.

### Estimating development assistance for health for COVID-19

We developed a separate method for estimating development assistance for health for COVID-19 because much of the project-level data used as input for the historical development assistance for health estimates do not extend to 2020, and therefore do not include resources disbursed in response to COVID-19. Likewise, budget and commitment data made before 2020 do not include the response to the pandemic. To adjust our 2020 development assistance estimates and include disbursements related to COVID-19, we extracted project data on commitments and disbursements from a diverse set of databases. Our primary focus was on the UN Office of Humanitarian Assistance financial tracking service (UNOCHA) and the International Aid Transparency Initiative (IATI). We relied on UNOCHA for data on non-governmental organisations; UNICEF; the UN Population Fund; and bilateral development agencies for the United Arab Emirates, Switzerland, Italy, and New Zealand. We relied on IATI for data on bilateral development agencies for Australia, Belgium, Canada, Denmark, Finland, France, Germany, Italy, the Netherlands, New Zealand, Norway, South Korea, Spain, Sweden, Switzerland, the UK, the USA, and the European Commission. We obtained data through correspondence for regional banks and other international agencies. For US foundations, we extracted commitment estimates from Candid. We extracted relevant data directly from organisations' online databases for the Bill & Melinda Gates Foundation; the Global Fund; Gavi, the Vaccine Alliance; WHO; the Pan American Health Organization; the World Bank; and regional development banks. For each agency, we extracted data on all 2020 project commitments and disbursements, as available. Data on commitments captured the resource allocation committed towards project activities that were generally available in project budget documents. Disbursements captured the value of project resources that had been transferred to implementing agencies to finance project activities.

We used the information available to determine whether projects were new grants or previous grants repurposed to COVID-19 projects. UNOCHA data designated whether resources were new or not. For IATI, we assumed that COVID-19 projects starting before 2020 were repurposed. For data received from correspondence or extracted from online databases, we contacted the respective agencies and searched their websites for more information. We included all research and development funding that went through international development agencies (appendix pp 91–92).

The approach used for each disbursing agency was specific to the data extracted and is detailed in the appendix (pp 92–106). Briefly, the general method was to use keywords to isolate COVID-19-relevant projects from each agency's database. We then examined these projects for completeness of information and adjusted the data to ensure that the estimate used was as precise as possible and could be compared between agencies. For instance, for projects with commitment estimates but without disbursement estimates, we calculated the average commitment-to-disbursement ratio for projects with complete data (for that development agency if possible) and multiplied the mean of those disbursement ratios by the commitment estimates for the projects without disbursement information. This commitment-to-disbursement ratio captures the proportion of committed funds that were estimated to have been disbursed in 2020. Next, we used a keyword search on project descriptions to disaggregate the estimated COVID-19 disbursements into eight COVID-19 programme areas: country-level coordination (planning, monitoring, and evaluation; risk communication; community engagement; and travel restrictions); surveillance, rapid-response teams, and case investigation; national laboratories and testing; infection prevention and personal protective equipment; treatment; supply chain and logistics; maintaining other essential health services and systems; and research and development for vaccines and other therapeutics (from a development agency). These programme areas were developed based on a review of the data and literature.

Finally, we reviewed the development agencies to identify any instances of double counting of the same resources across agencies. To do this, we reviewed income and recipient agency data for each of the disbursing channels that we tracked and excluded disbursements to recipient agencies that we tracked separately as disbursing agencies. We typically kept the disbursed resources with the development agency that was closest in the process to the final recipient country. This review ensured that we counted each disbursement from the source agency, to ultimate disbursing agency, to the final recipient only once.

### Estimating future health spending, 2019–50

We estimated GDP; general government spending (across all sectors); government, out-of-pocket, and prepaid private health spending; and total development assistance for health provided and received until 2050. The methods used for these projections draw heavily from our previous research, with the key change being the updating of the retrospective estimates on which these projections are based.^5^, ^32^, ^33^ Because this research draws from a diverse set of underlying input data, updates to these data have cascading effects and affect all of the projections.

We generated these projections by use of ensemble modelling techniques, such that the estimates are the mean of 1000 estimated projections from a broad set of models. We defined model selection by out-of-sample validation. This selection was country-specific and year-specific. We completed projections sequentially so that previously projected values could be used as covariates and for bounding other models. We forecasted GDP per working-age adult aged 20–64 years from 2022 to 2050, with 2020 and 2021 estimates drawing on methods that focused on estimating economic growth sensitive to COVID-19 projects (appendix p 8). We forecasted general government spending from 2020 to 2050 (as the retrospective estimates extend to 2019). We modelled development assistance for health as a proportion of the donor country's general government spending, or, for private donors, on the basis of AutoRegressive Integrated Moving Average modelling techniques from 2021 to 2050 (as the retrospective estimates extend to 2020).

We aggregated total development assistance for health provided across sources and used a separate model to project the proportion of total development assistance for health that each recipient was expected to receive from 2019 to 2050 (as these retrospective data extend to 2018). We also modelled when countries are projected to transition to being high-income and are no longer eligible to receive development assistance for health. We projected government health spending as a share of general government spending, prepaid private health spending as a share of GDP, and out-of-pocket health spending as a share of GDP from 2019 to 2050 (as the retrospective data extend to 2018). To capture increased government spending in response to COVID-19, we checked whether our 2020 estimates of government health spending increased by at least the estimates made by the IMF October 2020 Fiscal Monitor of previously unanticipated government health spending. Countries that had year-over-year spending increases in 2020 that were less than estimated increase reported in the Fiscal Monitor were adjusted upward proportionally.

All projections incorporated several types of uncertainty. We used ensemble modelling techniques to propagate model uncertainty. We took draws of the variance-covariance matrix of each estimate's model to propagate parameter uncertainty. Finally, we added a random walk residual to each projection to propagate fundamental uncertainty. We generated 95% UIs by taking the 2·5th and 97·5th percentile of the 1000 estimated random draws.

### Reporting

We report all the historical and future spending estimates in inflation-adjusted 2020 US$ and 2020 US$ per capita, and in purchasing-power parity-adjusted US$ per capita and as a proportion of GDP. For the development assistance for health estimates, we adjusted for inflation by taking disbursements in nominal US$ in the year of disbursements and using US GDP deflators from the IMF World Economic Outlook database to convert the series to constant 2020 US$. For the historical and future global health spending estimates, we used country-specific exchange rate data and deflator series from IMF to convert the series to constant 2020 US$. We report all spending estimates by 2019 Global Burden of Diseases, Injuries, and Risk Factors Study (GBD) super-region and 2020 World Bank income groups.^37^ For these aggregates (and the global aggregate) the reported estimates provide information about a group as a whole, rather than the mean of the countries included in that group. For all tables and figures, the country income classifications were held constant at the 2020 reported level, irrespective of whether they changed groups. The time periods for each of the financing sources differs relative to the availability of the underlying data (development assistance for health, 1990–2020; domestic health spending, 1995–2018; and future health spending, 2019–50). These time periods provide a time series of health spending data that makes sure to leverage all available data. We completed all the analyses using Stata (versions 13 and 15), R (versions 3.6.0 and 3.6.1), and Python (version 3.7.0).

### Role of the funding source

The funder of this study had no role in study design, data collection, data analysis, data interpretation, or writing of the report.

## Results

In 2019, total global health spending reached $8·8 trillion (95% UI 8·7–8·8) and was $1132 per person (1119–1143; table 1), with a wide variation in spending across geographical regions and country income groups. In 2019, high-income countries spent an estimated $5702 (95% UI 5638–5769) per person, with country-specific spending ranging from $523 (443–610) per person in the Northern Mariana Islands to $11 345 (11 114–11 578) per person in the USA. Across this income group, the most spending was from governments, which made up 61·4% (95% UI 60·9–62·0) of health spending, although countries such as the USA and Switzerland also relied substantially on prepaid private spending. Upper-middle-income countries spent $500 (484–517) per person on health, ranging from $94 (84–104) in Venezuela to $1492 (1274–1751) in Tokelau, and relied mostly on governments to finance their health spending. Lower-middle-income countries spent $90 (86–95) per person on health, ranging from $29 (26–32) in Benin to $395 (368–425) in Palestine, and relied mostly on out-of-pocket spending to finance their health spending. In 2019, low-income countries spent $36 (35–37) per person on health, ranging from $7 (6–8) in Somalia to $73 (66–81) in Sierra Leone, and also relied mostly on out-of-pocket spending. In 2019, development assistance for health to low-income and middle-income countries was $40·4 billion, or 0·5% (95% UI 0·5–0·5) of total global health spending. In 2019, development assistance for health made up 24·6% (24·0–25·1) of health spending in low-income countries and 3·2% (3·1–3·4) of health spending in lower-middle-income countries.

**Table 1:** Health spending and development assistance for health in 2019 and 2050 by region

		**Health spending per person, 2019 (US$)**	**Projected health spending per person, 2050 (2020 US$)**	**Health spending per person, 2019 ($PPP)**	**Projected health spending per person, 2050 ($PPP)**	**Total health spending per GDP, 2019**	**Total health spending per GDP, 2050**	**Development assistance for health system strengthening per person, 2020 (US$)**
**Global**								
Total		1132 (1119 to 1143)	1519 (1448 to 1591)	1485 (1470 to 1501)	2053 (1973 to 2135)	10·0% (9·8 to 10·1)	12·9% (11·5 to 14·2)	0·7
**World Bank income groups**								
High income		5702 (5638 to 5769)	8539 (8074 to 9032)	6288 (6226 to 6356)	9322 (8871 to 9812)	12·6% (12·5 to 12·8)	17·6% (15·5 to 19·9)	0·0
Upper-middle income		500 (484 to 517)	1001 (922 to 1083)	984 (959 to 1011)	1915 (1783 to 2051)	5·7% (5·5 to 5·9)	8·3% (6·4 to 10·3)	0·0
Lower-middle income		90 (86 to 95)	150 (141 to 159)	281 (267 to 297)	461 (433 to 493)	4·1% (3·9 to 4·3)	4·6% (3·8 to 5·4)	0·1
Low income		36 (35 to 37)	46 (44 to 47)	124 (121 to 129)	151 (145 to 158)	5·0% (4·7 to 5·3)	4·6% (4·1 to 5·2)	0·1
**GBD super-region**								
Central Europe, eastern Europe, and central Asia		571 (560 to 580)	656 (628 to 684)	1426 (1398 to 1453)	1635 (1564 to 1708)	6·0% (5·9 to 6·1)	7·0% (6·0 to 7·9)	0·2
High income		6282 (6211 to 6356)	9302 (8791 to 9852)	6773 (6701 to 6848)	9949 (9436 to 10 500)	13·1% (12·9 to 13·3)	18·1% (15·8 to 20·5)	0·0
Latin America and Caribbean		514 (497 to 530)	762 (710 to 824)	1163 (1125 to 1200)	1734 (1615 to 1873)	7·4% (7·2 to 7·6)	10·3% (8·7 to 12·0)	0·1
North Africa and Middle East		378 (370 to 386)	469 (444 to 498)	945 (926 to 967)	1225 (1154 to 1296)	5·8% (5·6 to 6·0)	8·6% (7·5 to 9·7)	0·0
South Asia		69 (62 to 77)	134 (119 to 150)	229 (207 to 255)	447 (397 to 502)	3·4% (3·1 to 3·8)	4·1% (2·9 to 5·6)	0·0
Southeast Asia, east Asia, and Oceania		439 (419 to 459)	1025 (915 to 1138)	766 (735 to 800)	1784 (1606 to 1966)	5·1% (4·8 to 5·3)	7·7% (5·6 to 10·3)	0·0
Sub-Saharan Africa		76 (74 to 79)	99 (94 to 106)	193 (187 to 199)	253 (240 to 268)	5·0% (4·8 to 5·2)	5·0% (4·4 to 5·7)	0·1
**Central Europe, eastern Europe, and central Asia**								
Central Asia								
	Armenia	436 (409 to 462)	531 (495 to 568)	1387 (1301 to 1472)	1689 (1576 to 1807)	9·6% (8·6 to 10·6)	10·0% (6·7 to 13·9)	3·3
	Azerbaijan	157 (140 to 174)	185 (161 to 211)	550 (492 to 612)	648 (567 to 741)	3·6% (3·2 to 4·0)	4·1% (2·8 to 6·0)	0·0
	Georgia	330 (311 to 350)	516 (456 to 585)	1133 (1067 to 1201)	1773 (1568 to 2009)	7·4% (6·8 to 8·1)	10·5% (7·4 to 14·4)	0·0
	Kazakhstan	282 (258 to 308)	403 (331 to 484)	855 (782 to 934)	1219 (1002 to 1465)	3·1% (2·8 to 3·4)	3·8% (2·6 to 5·4)	0·0
	Kyrgyzstan	83 (79 to 88)	108 (98 to 121)	350 (333 to 369)	455 (412 to 510)	6·3% (6·0 to 6·7)	7·7% (5·7 to 10·2)	0·0
	Mongolia	203 (187 to 222)	293 (254 to 345)	623 (575 to 683)	900 (781 to 1059)	4·8% (4·4 to 5·3)	5·9% (4·0 to 8·3)	9·4
	Tajikistan	59 (56 to 61)	68 (62 to 77)	251 (240 to 262)	292 (264 to 329)	7·0% (6·4 to 7·6)	7·7% (5·4 to 10·8)	0·0
	Turkmenistan	603 (559 to 648)	745 (680 to 814)	1247 (1157 to 1340)	1543 (1406 to 1685)	7·7% (7·1 to 8·4)	8·6% (6·0 to 12·0)	0·0
	Uzbekistan	93 (85 to 102)	128 (112 to 145)	391 (356 to 427)	537 (469 to 606)	5·3% (4·8 to 5·8)	5·9% (4·1 to 8·4)	0·7
Central Europe								
	Albania	293 (270 to 317)	355 (319 to 396)	816 (753 to 884)	990 (888 to 1103)	5·5% (5·1 to 6·0)	6·0% (4·1 to 8·3)	0·0
	Bosnia and Herzegovina	548 (517 to 582)	951 (872 to 1038)	1416 (1336 to 1503)	2457 (2253 to 2682)	9·0% (8·4 to 9·5)	12·2% (8·3 to 17·4)	0·0
	Bulgaria	785 (745 to 826)	991 (847 to 1162)	1896 (1799 to 1997)	2396 (2047 to 2808)	7·7% (7·3 to 8·2)	8·9% (6·0 to 12·7)	0·0
	Croatia	1024 (964 to 1082)	1299 (1040 to 1619)	2020 (1901 to 2134)	2563 (2052 to 3193)	6·7% (6·3 to 7·1)	8·4% (5·5 to 12·3)	0·0
	Czech Republic	1851 (1789 to 1911)	2547 (2244 to 2869)	3296 (3186 to 3402)	4536 (3996 to 5109)	7·6% (7·1 to 8·3)	9·9% (6·8 to 13·6)	0·0
	Hungary	1100 (1068 to 1133)	1296 (1197 to 1405)	2321 (2253 to 2390)	2734 (2526 to 2964)	6·7% (6·5 to 6·9)	7·2% (5·3 to 9·5)	0·0
	Montenegro	732 (689 to 780)	814 (745 to 892)	1840 (1733 to 1960)	2047 (1873 to 2242)	8·1% (7·7 to 8·7)	9·0% (6·4 to 12·0)	0·0
	North Macedonia	412 (396 to 429)	412 (378 to 449)	1137 (1092 to 1183)	1136 (1042 to 1239)	6·5% (6·0 to 7·0)	6·7% (5·1 to 8·7)	0·0
	Poland	1023 (999 to 1048)	1461 (1338 to 1599)	2256 (2202 to 2310)	3221 (2949 to 3525)	6·4% (6·3 to 6·6)	8·7% (6·0 to 12·3)	0·0
	Romania	773 (733 to 815)	1038 (861 to 1228)	1819 (1723 to 1917)	2440 (2025 to 2888)	5·8% (5·4 to 6·1)	6·7% (4·4 to 9·6)	0·0
	Serbia	518 (505 to 533)	637 (598 to 677)	1302 (1268 to 1339)	1600 (1504 to 1700)	6·8% (6·6 to 7·0)	7·4% (5·4 to 9·7)	0·0
	Slovakia	1375 (1317 to 1435)	1813 (1637 to 2046)	2373 (2272 to 2475)	3128 (2824 to 3530)	6·9% (6·6 to 7·2)	8·6% (5·9 to 11·6)	0·0
	Slovenia	2248 (2186 to 2314)	2691 (2511 to 2883)	3456 (3360 to 3558)	4137 (3860 to 4432)	8·4% (8·1 to 8·6)	10·5% (7·4 to 14·5)	0·0
Eastern Europe								
	Belarus	361 (334 to 387)	459 (364 to 576)	1163 (1075 to 1248)	1480 (1174 to 1858)	5·7% (5·3 to 6·1)	6·6% (4·3 to 10·0)	0·0
	Estonia	1628 (1586 to 1669)	2211 (1973 to 2456)	2623 (2555 to 2689)	3561 (3179 to 3956)	6·8% (6·6 to 7·0)	8·2% (5·7 to 11·4)	0·0
	Latvia	1132 (1094 to 1170)	1384 (1259 to 1517)	2008 (1941 to 2075)	2456 (2233 to 2693)	6·2% (6·0 to 6·4)	7·1% (4·9 to 9·7)	0·0
	Lithuania	1321 (1277 to 1364)	1649 (1519 to 1786)	2562 (2477 to 2646)	3198 (2947 to 3465)	6·6% (6·3 to 6·9)	7·3% (4·9 to 10·3)	0·0
	Moldova	215 (198 to 232)	252 (225 to 285)	667 (6168 to 720)	783 (698 to 884)	5·8% (4·3 to 8·3)	7·1% (4·4 to 11·5)	0·0
	Russia	563 (537 to 586)	611 (554 to 676)	1546 (1475 to 1610)	1679 (1522 to 1858)	5·4% (5·1 to 5·6)	6·2% (4·4 to 8·5)	0·0
	Ukraine	258 (242 to 274)	259 (233 to 288)	957 (899 to 1015)	962 (865 to 1067)	7·4% (6·7 to 8·1)	8·4% (6·1 to 11·2)	0·0
**High income**								
Australasia								
	Australia	5507 (5409 to 5608)	7818 (7303 to 8402)	5398 (5302 to 5498)	7663 (7159 to 8236)	10·3% (9·8 to 11·1)	13·6% (9·4 to 18·6)	0·0
	New Zealand	4175 (4081 to 4269)	6012 (5553 to 6508)	4434 (4334 to 4534)	6386 (5898 to 6912)	9·9% (9·4 to 10·2)	13·2% (9·4 to 17·6)	0·0
High-income Asia Pacific								
	Brunei	544 (501 to 592)	490 (374 to 620)	1454 (1339 to 1583)	1310 (1001 to 1659)	2·3% (2·1 to 2·5)	2·5% (1·6 to 3·6)	0·0
	Japan	4489 (4372 to 4622)	6150 (5583 to 6734)	4787 (4662 to 4929)	6558 (5953 to 7180)	10·9% (10·6 to 11·3)	14·4% (10·0 to 20·0)	0·0
	Singapore	2843 (2719 to 2966)	5002 (4033 to 6083)	4645 (4443 to 4847)	8173 (6590 to 9940)	4·5% (4·3 to 4·8)	7·2% (4·6 to 10·6)	0·0
	South Korea	2442 (2395 to 2487)	4957 (4470 to 5478)	3529 (3461 to 3594)	7165 (6461 to 7917)	7·8% (7·6 to 7·9)	14·1% (9·7 to 20·2)	0·0
High-income North America								
	Canada	5163 (5104 to 5218)	7077 (6531 to 7661)	5837 (5770 to 5899)	8000 (7383 to 8661)	11·2% (11·0 to 11·4)	15·1% (10·8 to 20·4)	0·0
	Greenland	6579 (5966 to 7194)	7651 (6719 to 8606)	5381 (4879 to 5884)	6258 (5595 to 7038)	11·5% (10·1 to 13·0)	12·9% (8·8 to 18·2)	0·0
	USA	11 345 (11 114 to 11 578)	17 300 (15 680 to 18 976)	11 345 (11 114 to 11 578)	17 300 (15 680 to 18 976)	17·2% (16·8 to 17·5)	24·8% (19·0 to 32·5)	0·0
Southern Latin America								
	Argentina	946 (905 to 988)	1239 (1086 to 1428)	2285 (2186 to 2387)	2993 (2624 to 3449)	9·7% (9·3 to 10·2)	13·0% (8·8 to 18·1)	0·0
	Chile	1329 (1304 to 1353)	2025 (1885 to 2184)	2472 (2426 to 2516)	3765 (3505 to 4062)	9·7% (9·5 to 9·9)	14·0% (10·1 to 19·5)	0·0
	Uruguay	1549 (1516 to 1585)	2268 (2009 to 2572)	2156 (2110 to 2205)	3157 (2796 to 3580)	9·5% (9·2 to 9·8)	11·9% (8·2 to 16·8)	0·0
Western Europe								
	Andorra	2948 (2827 to 3079)	2957 (2538 to 3587)	3141 (3012 to 3280)	3150 (2704 to 3822)	6·7% (5·9 to 7·8)	9·7% (6·6 to 13·9)	0·0
	Austria	5383 (5302 to 5463)	6766 (6253 to 7294)	6132 (6039 to 6222)	7707 (7123 to 8308)	10·3% (10·1 to 10·5)	12·8% (8·8 to 17·3)	0·0
	Belgium	4983 (4887 to 5083)	6490 (6019 to 6960)	5698 (5588 to 5813)	7421 (6882 to 7958)	10·4% (10·2 to 10·7)	12·7% (9·5 to 16·6)	0·0
	Cyprus	1262 (1212 to 1313)	1582 (1443 to 1743)	1881 (1806 to 1957)	2358 (2150 to 2598)	5·0% (4·0 to 6·6)	6·2% (4·3 to 9·3)	0·0
	Denmark	6182 (6035 to 6340)	8095 (7433 to 8849)	6112 (5967 to 6269)	8004 (7350 to 8750)	10·0% (9·7 to 10·3)	12·2% (9·2 to 15·8)	0·0
	Finland	4595 (4504 to 4690)	5642 (5261 to 6023)	4680 (4587 to 4776)	5745 (5358 to 6134)	9·1% (8·9 to 9·3)	9·9% (7·3 to 13·1)	0·0
	France	4844 (4792 to 4891)	6245 (5714 to 6811)	5605 (55475 to 5659)	7226 (6612 to 7881)	11·4% (11·0 to 11·7)	14·1% (10·8 to 18·0)	0·0
	Germany	5498 (5428 to 5570)	7518 (6986 to 7987)	6482 (6399 to 6567)	8863 (8236 to 9416)	11·4% (11·2 to 11·5)	14·6% (10·4 to 19·5)	0·0
	Greece	1595 (1515 to 1677)	1620 (1470 to 1785)	2551 (2422 to 2682)	2591 (2351 to 2854)	8·0% (7·6 to 8·4)	9·2% (7·1 to 11·7)	0·0
	Iceland	5486 (5256 to 5722)	6931 (6189 to 7676)	5227 (5007 to 5451)	6603 (5896 to 7313)	8·6% (8·0 to 9·2)	10·3% (7·4 to 13·8)	0·0
	Ireland	5673 (5413 to 5932)	8456 (7758 to 9225)	6362 (6071 to 6653)	9484 (8700 to 10 345)	6·8% (6·5 to 7·1)	9·1% (6·6 to 11·8)	0·0
	Israel	3246 (3184 to 3307)	4174 (3854 to 4522)	3056 (2997 to 3114)	3930 (3629 to 4257)	7·1% (6·6 to 7·4)	8·1% (5·7 to 11·1)	0·0
	Italy	2995 (2931 to 3061)	3160 (2924 to 3424)	3916 (3833 to 4003)	4132 (3823 to 4478)	8·7% (8·6 to 8·9)	10·6% (8·1 to 13·6)	0·0
	Luxembourg	6246 (5877 to 6655)	6893 (6019 to 7849)	6436 (6055 to 6856)	7102 (6202 to 8087)	5·3% (5·0 to 5·6)	6·7% (4·9 to 8·9)	0·0
	Malta	3174 (3081 to 3268)	4837 (4486 to 5211)	4803 (4663 to 4946)	7320 (6788 to 7886)	9·7% (8·6 to 10·6)	10·9% (7·4 to 15·5)	0·0
	Monaco	3560 (3346 to 3779)	4719 (4118 to 5446)	3479 (3270 to 3693)	4612 (4025 to 5322)	1·7% (1·5 to 2·1)	2·0% (1·4 to 2·9)	0·0
	Netherlands	5586 (5449 to 5735)	7722 (6885 to 8669)	6217 (6065 to 6382)	8594 (7662 to 9648)	10·3% (10·0 to 10·6)	13·6% (9·5 to 18·7)	0·0
	Norway	7352 (7176 to 7537)	8329 (7576 to 9223)	7013 (6845 to 7189)	7945 (7227 to 8798)	10·4% (10·2 to 10·7)	12·6% (10·1 to 15·6)	0·0
	Portugal	2187 (2117 to 2260)	2389 (2141 to 2685)	3350 (3243 to 3462)	3659 (3281 to 4113)	9·1% (8·8 to 9·4)	10·6% (8·2 to 13·6)	0·0
	San Marino	3393 (3249 to 3540)	3944 (3644 to 4282)	4612 (4417 to 4812)	5361 (4953 to 5821)	7·2% (6·9 to 7·5)	8·5% (6·6 to 11·1)	0·0
	Spain	2801 (2731 to 2868)	3313 (3063 to 3708)	3985 (3884 to 4079)	4712 (4357 to 5274)	9·1% (8·9 to 9·4)	12·3% (9·5 to 15·8)	0·0
	Sweden	5898 (5692 to 6117)	8016 (7268 to 8874)	6149 (5933 to 6376)	8356 (7577 to 9250)	10·8% (10·4 to 11·3)	12·9% (9·1 to 17·6)	0·0
	Switzerland	10 203 (10 057 to 10 351)	14 239 (12 827 to 15 690)	8521 (8398 to 8644)	11 891 (10 712 to 13 103)	11·6% (11·1 to 11·9)	17·0% (11·9 to 23·5)	0·0
	UK	4392 (4329 to 4457)	5279 (4863 to 5702)	4960 (4888 to 5033)	5962 (5492 to 6439)	10·1% (9·9 to 10·3)	12·3% (9·9 to 14·9)	0·0
**Latin America and Caribbean**								
Andean Latin America								
	Bolivia	233 (213 to 254)	336 (299 to 378)	585 (536 to 639)	845 (752 to 949)	6·4% (5·8 to 7·0)	8·5% (5·9 to 12·1)	0·0
	Ecuador	483 (455 to 512)	684 (600 to 771)	963 (907 to 1022)	1366 (1198 to 1540)	8·0% (7·5 to 8·5)	11·0% (7·6 to 15·4)	0·0
	Peru	346 (324 to 369)	401 (352 to 455)	683 (639 to 728)	790 (694 to 897)	5·0% (4·6 to 5·3)	6·3% (4·5 to 8·6)	0·0
Caribbean								
	Antigua and Barbuda	972 (925 to 1024)	1381 (1160 to 1608)	1281 (1219 to 1349)	1820 (1528 to 2119)	5·6% (5·3 to 5·9)	7·9% (5·8 to 10·7)	0·0
	The Bahamas	2191 (2111 to 2270)	3238 (2932 to 3572)	2468 (2377 to 2557)	3646 (3302 to 4022)	6·2% (6·0 to 6·5)	10·1% (7·4 to 13·5)	0·0
	Barbados	1126 (1074 to 1182)	1104 (1023 to 1205)	1020 (973 to 1071)	1000 (927 to 1091)	6·2% (5·9 to 6·5)	6·4% (5·0 to 8·4)	0·0
	Belize	283 (258 to 309)	450 (346 to 551)	431 (394 to 471)	686 (527 to 841)	6·0% (5·4 to 6·6)	8·1% (5·6 to 11·7)	0·0
	Bermuda	8049 (7007 to 9277)	10 721 (8170 to 13 824)	5686 (4950 to 6553)	7574 (5771 to 9765)	6·7% (5·0 to 9·2)	12·1% (7·6 to 18·3)	0·0
	Cuba	1170 (1090 to 1255)	1745 (1549 to 1936)	2863 (2667 to 3071)	4268 (3790 to 4735)	13·1% (11·7 to 14·7)	20·3% (14·1 to 28·4)	0·0
	Dominica	431 (403 to 460)	466 (391 to 550)	676 (632 to 722)	731 (613 to 863)	5·1% (4·8 to 5·5)	5·1% (3·8 to 6·6)	0·0
	Dominican Republic	446 (416 to 476)	715 (600 to 847)	1124 (1048 to 1200)	1804 (1514 to 2137)	5·7% (5·3 to 6·2)	7·6% (5·1 to 10·9)	0·0
	Grenada	515 (483 to 551)	650 (560 to 766)	863 (808 to 923)	1088 (937 to 1283)	4·7% (4·4 to 5·1)	5·1% (3·6 to 6·9)	0·0
	Guyana	323 (301 to 346)	542 (422 to 692)	648 (604 to 694)	1087 (847 to 1390)	4·4% (3·9 to 5·0)	5·1% (3·6 to 7·5)	0·0
	Haiti	47 (43 to 53)	73 (64 to 84)	111 (100 to 124)	171 (151 to 199)	5·3% (3·0 to 7·5)	7·6% (3·8 to 12·6)	0·0
	Jamaica	365 (337 to 393)	598 (509 to 698)	715 (660 to 769)	1170 (996 to 1366)	6·7% (6·0 to 7·3)	12·0% (8·3 to 16·8)	0·0
	Puerto Rico	1286 (1138 to 1459)	1626 (1385 to 1890)	1496 (1323 to 1697)	1890 (1611 to 2198)	3·9% (3·4 to 4·5)	5·3% (3·8 to 7·2)	0·0
	Saint Kitts and Nevis	1026 (967 to 1086)	1457 (1293 to 1651)	1415 (1332 to 1497)	2008 (1782 to 2276)	5·2% (4·8 to 5·7)	5·7% (4·3 to 7·3)	0·0
	Saint Lucia	531 (496 to 568)	708 (610 to 809)	744 (695 to 797)	992 (855 to 1134)	4·5% (4·2 to 4·9)	6·2% (4·3 to 8·3)	0·0
	Saint Vincent and the Grenadines	359 (334 to 387)	510 (451 to 576)	626 (583 to 675)	890 (788 to 1005)	4·7% (4·4 to 5·1)	6·5% (4·7 to 8·7)	0·0
	Suriname	408 (381 to 438)	610 (501 to 746)	1420 (1324 to 1524)	2121 (1742 to 2595)	8·2% (7·6 to 8·8)	12·0% (8·1 to 17·1)	0·0
	Trinidad and Tobago	1145 (1093 to 1203)	1392 (1262 to 1530)	1836 (1752 to 1929)	2232 (2022 to 2452)	6·6% (6·3 to 7·0)	8·1% (6·1 to 10·6)	0·0
	Virgin Islands	969 (825 to 1133)	1132 (931 to 1375)	969 (825 to 1133)	1132 (931 to 1375)	2·4% (2·0 to 2·9)	2·7% (1·9 to 3·8)	0·0
Central Latin America								
	Colombia	447 (429 to 468)	810 (714 to 917)	1214 (1165 to 1270)	2198 (1939 to 2490)	7·8% (7·5 to 8·2)	12·7% (8·8 to 17·9)	0·0
	Costa Rica	1083 (1061 to 1105)	1682 (1530 to 1869)	1798 (1762 to 1835)	2792 (2541 to 3104)	8·7% (8·6 to 8·9)	13·0% (9·3 to 17·7)	0·0
	El Salvador	322 (299 to 347)	508 (452 to 572)	707 (657 to 764)	1116 (994 to 1258)	7·7% (7·1 to 8·3)	10·9% (8·2 to 14·1)	0·0
	Guatemala	308 (291 to 326)	461 (420 to 501)	601 (567 to 636)	899 (820 to 977)	6·9% (6·3 to 7·4)	8·4% (6·5 to 10·7)	0·0
	Honduras	187 (173 to 203)	283 (245 to 331)	429 (396 to 465)	650 (562 to 759)	7·1% (6·5 to 7·7)	8·3% (5·6 to 11·5)	0·0
	Mexico	502 (482 to 525)	619 (572 to 671)	1171 (1124 to 1223)	1441 (1332 to 1564)	5·6% (5·4 to 5·8)	6·6% (5·0 to 8·2)	0·0
	Nicaragua	169 (153 to 186)	262 (228 to 297)	501 (454 to 551)	777 (678 to 881)	8·7% (7·9 to 9·6)	12·5% (8·9 to 17·2)	1·3
	Panama	1103 (1075 to 1128)	1631 (1464 to 1813)	2353 (2293 to 2406)	3479 (3121 to 3866)	7·1% (6·9 to 7·2)	8·8% (6·1 to 12·3)	0·0
	Venezuela*	94 (84 to 104)	80 (68 to 93)	230 (206 to 255)	196 (166 to 228)	3·7% (3·0 to 4·5)	3·9% (2·5 to 5·6)	0·0
Tropical Latin America								
	Brazil	639 (598 to 682)	1099 (950 to 1266)	1443 (1350 to 1540)	2480 (2144 to 2858)	9·3% (8·7 to 9·9)	14·8% (10·1 to 21·0)	0·2
	Paraguay	380 (357 to 404)	750 (678 to 830)	968 (908 to 1029)	1909 (1727 to 2114)	7·2% (6·8 to 7·7)	11·1% (7·7 to 15·0)	0·0
**North Africa and Middle East**								
	Afghanistan	54 (49 to 58)	58 (53 to 64)	223 (205 to 243)	243 (221 to 265)	10·5% (9·3 to 11·8)	9·9% (6·6 to 14·2)	0·0
	Algeria	231 (210 to 253)	308 (260 to 363)	765 (696 to 838)	1021 (862 to 1202)	6·4% (5·8 to 7·0)	9·0% (6·4 to 12·3)	0·0
	Bahrain	1052 (1001 to 1103)	938 (793 to 1108)	2260 (2151 to 2369)	2015 (1705 to 2380)	4·6% (4·1 to 4·9)	6·2% (4·2 to 8·6)	0·0
	Egypt	173 (162 to 183)	276 (248 to 306)	617 (580 to 655)	984 (884 to 1093)	5·2% (4·6 to 5·7)	5·9% (4·0 to 8·2)	0·0
	Iran	675 (642 to 706)	890 (770 to 1014)	1112 (1059 to 1164)	1467 (1270 to 1671)	8·4% (7·7 to 9·1)	14·2% (10·2 to 19·1)	0·0
	Iraq	185 (168 to 203)	322 (246 to 409)	415 (378 to 455)	723 (552 to 917)	3·6% (3·3 to 4·0)	5·0% (3·1 to 7·9)	0·0
	Jordan	306 (284 to 331)	475 (403 to 560)	734 (679 to 792)	1137 (966 to 1341)	6·9% (6·4 to 7·4)	10·3% (7·3 to 14·2)	0·0
	Kuwait	1453 (1317 to 1607)	1098 (906 to 1332)	2724 (2469 to 3013)	2059 (1699 to 2497)	5·4% (4·7 to 6·3)	7·6% (5·0 to 11·3)	0·0
	Lebanon	395 (367 to 425)	436 (382 to 501)	1663 (1547 to 1789)	1837 (1608 to 2109)	10·7% (10·0 to 11·5)	12·7% (9·0 to 17·3)	0·0
	Libya	798 (683 to 930)	658 (448 to 924)	1155 (988 to 1344)	944 (648 to 1336)	9·0% (3·5 to 18·1)	10·3% (3·2 to 24·4)	0·0
	Morocco	176 (165 to 187)	295 (263 to 331)	429 (401 to 455)	719 (642 to 808)	5·1% (4·7 to 5·6)	7·1% (4·9 to 9·9)	0·0
	Oman	612 (560 to 667)	679 (528 to 860)	1272 (1165 to 1387)	1410 (1098 to 1788)	4·1% (3·5 to 4·7)	5·4% (3·4 to 7·9)	0·0
	Palestine	395 (368 to 425)	742 (638 to 859)	190 (177 to 205)	357 (307 to 414)	10·6% (9·7 to 11·5)	14·3% (9·8 to 19·7)	0·0
	Qatar	1731 (1554 to 1923)	2790 (1869 to 4115)	3017 (2707 to 3351)	4861 (3256 to 7169)	3·2% (2·8 to 3·5)	9·4% (5·5 to 15·6)	0·0
	Saudi Arabia	1364 (1285 to 1444)	2161 (1901 to 2437)	3223 (3035 to 34117)	5107 (4491 to 5758)	6·5% (6·1 to 6·9)	15·4% (10·4 to 22·1)	0·0
	Sudan	49 (44 to 55)	65 (57 to 75)	252 (226 to 280)	334 (289 to 382)	5·1% (3·3 to 6·7)	4·6% (2·6 to 6·7)	0·0
	Syria	44 (39 to 50)	57 (50 to 64)	1046 (925 to 1204)	1353 (1185 to 1542)	3·6% (3·1 to 4·2)	4·4% (3·0 to 6·5)	0·0
	Tunisia	281 (260 to 303)	434 (393 to 477)	884 (820 to 953)	1365 (1237 to 1504)	7·8% (7·2 to 8·4)	10·9% (7·6 to 15·2)	0·0
	Turkey	359 (336 to 382)	552 (448 to 665)	1316 (1231 to 1400)	2026 (1644 to 2438)	4·4% (4·1 to 4·6)	6·3% (4·1 to 9·0)	0·0
	United Arab Emirates	1751 (1675 to 1824)	1353 (839 to 2357)	3203 (3065 to 3338)	2476 (1535 to 4312)	4·7% (4·3 to 5·2)	9·4% (4·9 to 18·6)	0·0
	Yemen	35 (30 to 40)	46 (39 to 54)	105 (91 to 121)	137 (117 to 161)	5·4% (4·0 to 7·4)	5·5% (3·6 to 8·3)	0·0
South Asia								
	Bangladesh	48 (43 to 54)	76 (67 to 86)	129 (115 to 145)	204 (180 to 230)	2·6% (2·3 to 3·0)	2·7% (1·8 to 3·8)	0·3
	Bhutan	90 (82 to 98)	195 (155 to 241)	315 (288 to 345)	684 (545 to 846)	2·6% (2·4 to 2·8)	3·4% (2·2 to 5·1)	12·2
	India	75 (67 to 85)	150 (132 to 173)	253 (223 to 286)	504 (441 to 578)	3·5% (3·1 to 4·0)	4·3% (2·9 to 6·2)	0·0
	Nepal	58 (54 to 64)	105 (93 to 120)	188 (173 to 205)	336 (298 to 386)	5·3% (4·8 to 5·8)	5·9% (4·1 to 8·3)	0·0
	Pakistan	43 (40 to 46)	82 (70 to 94)	158 (146 to 170)	298 (256 to 343)	3·0% (2·8 to 3·3)	3·7% (2·5 to 5·2)	0·0
**Southeast Asia, east Asia, and Oceania**								
East Asia								
	China	563 (531 to 594)	1470 (1289 to 1657)	893 (843 to 943)	2334 (2046 to 2631)	5·3% (5·0 to 5·6)	8·3% (5·6 to 11·7)	0·0
	North Korea	61 (52 to 70)	72 (61 to 83)	35 (30 to 41)	41 (35 to 48)	5·2% (4·5 to 6·1)	7·2% (4·9 to 10·1)	0·0
	Taiwan (province of China)	1377 (1307 to 1464)	2368 (2218 to 2561)	2765 (2624 to 2939)	4754 (4453 to 5142)	5·1% (4·8 to 5·5)	7·7% (5·5 to 10·6)	0·0
Oceania								
	American Samoa	577 (492 to 668)	711 (603 to 828)	577 (492 to 668)	711 (603 to 828)	4·9% (4·0 to 5·8)	5·9% (3·9 to 8·4)	0·0
	Cook Islands	756 (692 to 825)	1287 (918 to 1773)	1056 (967 to 1152)	1797 (1282 to 2476)	3·4% (3·1 to 3·8)	4·1% (2·6 to 6·4)	0·0
	Federated States of Micronesia	147 (131 to 164)	249 (211 to 290)	131 (117 to 147)	222 (189 to 260)	3·9% (3·3 to 4·6)	5·4% (3·7 to 7·5)	0·0
	Fiji	195 (182 to 208)	304 (269 to 340)	498 (466 to 531)	777 (689 to 870)	3·4% (2·9 to 4·0)	4·5% (3·2 to 6·1)	0·0
	Guam	953 (826 to 1099)	1164 (1000 to 1352)	953 (826 to 1099)	1164 (1000 to 1352)	2·6% (2·2 to 3·1)	3·1% (2·3 to 4·2)	0·0
	Kiribati	200 (182 to 219)	255 (209 to 317)	259 (236 to 284)	331 (272 to 411)	12·0% (10·9 to 13·1)	14·0% (9·9 to 19·5)	0·0
	Marshall Islands	536 (499 to 580)	837 (683 to 1036)	499 (464 to 539)	778 (635 to 964)	12·4% (10·4 to 14·2)	15·4% (11·1 to 21·3)	0·0
	Nauru	1237 (1134 to 1342)	840 (653 to 1124)	1387 (12571to 1504)	941 (732 to 1260)	11·9% (6·9 to 17·1)	7·8% (3·7 to 14·1)	0·0
	Niue	2273 (1858 to 27652	2897 (1734 to 4478)	849 (694 to 1028)	1082 (648 to 1673)	11·9% (9·4 to 15·1)	11·9% (6·6 to 20·3)	0·0
	Northern Mariana Islands	523 (443 to 610)	651 (509 to 841)	523 (443 to 610)	651 (509 to 841)	2·2% (1·8 to 2·7)	3·5% (2·3 to 5·2)	0·0
	Palau	1827 (1742 to 1916)	3046 (2651 to 3482)	1817 (1733 to 1907)	3031 (2638 to 3464)	11·3% (10·7 to 11·8)	16·2% (12·0 to 21·4)	0·0
	Papua New Guinea	73 (66 to 82)	104 (87 to 126)	106 (95 to 119)	151 (127 to 183)	2·6% (2·4 to 3·0)	3·0% (2·1 to 4·2)	2·1
	Samoa	235 (217 to 253)	331 (291 to 378)	319 (294 to 344)	450 (396 to 514)	5·3% (4·7 to 5·8)	7·3% (5·2 to 10·2)	0·0
	Solomon Islands	112 (99 to 128)	176 (102 to 300)	116 (102 to 132)	182 (105 to 310)	4·8% (3·9 to 6·1)	5·8% (2·9 to 11·1)	0·0
	Tokelau	1492 (1274 to 1751)	2679 (2229 to 3236)	3074 (2625 to 3608)	5520 (4592 to 6666)	23·1% (19·6 to 27·2)	26·3% (17·8 to 38·5)	0·0
	Tonga	240 (224 to 256)	330 (297 to 367)	290 (272 to 310)	399 (360 to 444)	4·7% (4·3 to 5·1)	4·8% (3·8 to 6·0)	88·3
	Tuvalu	757 (693 to 833)	1010 (799 to 1307)	835 (763 to 918)	1114 (881 to 1441)	19·0% (16·9 to 21·3)	19·8% (13·2 to 28·2)	0·0
	Vanuatu	100 (88 to 114)	148 (104 to 212)	92 (81 to 105)	136 (95 to 195)	3·1% (2·7 to 3·5)	3·3% (2·0 to 5·2)	0·0
Southeast Asia								
	Cambodia	83 (75 to 91)	108 (97 to 120)	233 (212 to 257)	305 (275 to 340)	5·0% (4·6 to 5·6)	4·5% (3·2 to 6·1)	0·0
	Indonesia	122 (115 to 131)	283 (237 to 336)	373 (351 to 399)	864 (724 to 1028)	3·0% (2·8 to 3·2)	4·8% (3·3 to 7·0)	0·0
	Laos	63 (60 to 66)	89 (81 to 98)	202 (193 to 211)	286 (260 to 314)	2·5% (2·1 to 2·9)	2·1% (1·4 to 3·0)	0·2
	Malaysia	448 (422 to 477)	947 (844 to 1070)	1200 (1130 to 1277)	2537 (2261 to 2866)	4·1% (3·8 to 4·3)	6·5% (4·5 to 9·2)	0·0
	Maldives	1164 (1085 to 1245)	1712 (1343 to 2146)	2145 (2000 to 2293)	3153 (2475 to 3953)	9·1% (6·9 to 10·9)	12·2% (7·2 to 18·9)	0·0
	Mauritius	671 (632 to 714)	1232 (1113 to 1362)	1553 (1463 to 1652)	2853 (2576 to 3152)	6·4% (6·0 to 6·8)	9·3% (6·5 to 12·7)	0·0
	Myanmar	60 (56 to 63)	109 (99 to 120)	233 (220 to 246)	424 (385 to 468)	4·4% (4·0 to 4·8)	4·8% (3·2 to 6·8)	0·0
	Philippines	156 (142 to 172)	259 (228 to 295)	396 (362 to 437)	659 (580 to 751)	4·3% (3·8 to 4·8)	5·6% (3·8 to 7·6)	0·0
	Seychelles	673 (642 to 701)	935 (791 to 1103)	1440 (1376 to 1502)	2003 (1694 to 2362)	4·7% (4·4 to 4·9)	6·1% (4·1 to 9·0)	0·0
	Sri Lanka	156 (146 to 167)	234 (211 to 261)	555 (519 to 593)	830 (749 to 926)	4·0% (3·7 to 4·3)	4·4% (3·1 to 6·0)	1·8
	Thailand	307 (286 to 330)	595 (517 to 680)	759 (708 to 818)	1475 (1282 to 1686)	3·9% (3·6 to 4·2)	6·8% (4·7 to 9·6)	0·0
	Timor-Leste	82 (74 to 90)	97 (73 to 132)	226 (205 to 249)	269 (202 to 365)	6·5% (5·6 to 7·4)	5·1% (3·2 to 7·7)	0·0
	Vietnam	167 (157 to 177)	288 (241 to 344)	512 (483 to 545)	884 (741 to 1056)	5·7% (4·4 to 6·9)	7·1% (4·3 to 10·7)	0·1
**Sub-Saharan Africa**								
Central sub-Saharan Africa								
	Angola	57 (50 to 64)	94 (75 to 117)	195 (172 to 222)	326 (260 to 405)	2·7% (2·4 to 3·1)	3·2% (2·1 to 4·7)	0·0
	Central African Republic	30 (28 to 33)	47 (41 to 56)	61 (56 to 66)	95 (83 to 113)	6·1% (5·6 to 6·6)	7·7% (5·6 to 10·4)	0·0
	Congo (Brazzaville)	47 (43 to 51)	70 (58 to 86)	93 (86 to 102)	139 (115 to 172)	2·2% (1·9 to 2·6)	2·5% (1·7 to 3·6)	0·2
	Democratic Republic of the Congo	20 (19 to 22)	25 (22 to 29)	43 (41 to 46)	54 (46 to 62)	4·0% (3·5 to 4·5)	4·3% (2·8 to 6·3)	0·0
	Equatorial Guinea	222 (196 to 252)	507 (431 to 603)	554 (488 to 627)	1265 (1074 to 1504)	2·8% (2·5 to 3·2)	3·5% (2·1 to 5·5)	0·0
	Gabon	245 (228 to 261)	452 (395 to 521)	541 (504 to 576)	997 (872 to 1151)	3·4% (3·1 to 3·6)	5·3% (3·8 to 7·3)	0·0
Eastern sub-Saharan Africa								
	Burundi	29 (27 to 31)	40 (35 to 47)	86 (80 to 93)	120 (103 to 141)	10·0% (8·8 to 11·2)	12·3% (8·7 to 16·8)	0·0
	Comoros	83 (74 to 94)	112 (96 to 133)	187 (166 to 213)	252 (216 to 299)	5·6% (4·7 to 6·6)	6·2% (4·2 to 8·7)	0·0
	Djibouti	54 (47 to 61)	72 (58 to 87)	88 (77 to 100)	119 (96 to 144)	1·6% (1·4 to 1·9)	1·9% (1·2 to 2·8)	0·0
	Eritrea	15 (13 to 17)	20 (17 to 23)	46 (42 to 52)	62 (54 to 72)	2·5% (2·2 to 2·8)	2·4% (1·7 to 3·3)	0·0
	Ethiopia	26 (24 to 28)	38 (33 to 43)	75 (69 to 80)	107 (94 to 122)	3·0% (2·5 to 3·4)	2·6% (1·7 to 3·7)	0·2
	Kenya	103 (95 to 112)	176 (152 to 204)	248 (229 to 270)	423 (367 to 492)	5·2% (4·6 to 5·9)	6·4% (4·4 to 8·8)	0·1
	Madagascar	22 (19 to 25)	31 (26 to 35)	70 (61 to 79)	98 (85 to 113)	4·0% (3·6 to 4·5)	5·4% (3·9 to 7·3)	0·0
	Malawi	43 (40 to 45)	51 (43 to 60)	107 (101 to 113)	126 (108 to 149)	9·8% (8·9 to 11·0)	7·9% (5·4 to 10·9)	0·0
	Mozambique	36 (34 to 38)	43 (35 to 54)	100 (95 to 106)	121 (97 to 151)	7·4% (7·0 to 7·9)	4·9% (3·3 to 7·3)	0·0
	Rwanda	53 (47 to 59)	96 (76 to 119)	153 (137 to 172)	278 (221 to 344)	6·6% (5·8 to 7·5)	8·6% (5·7 to 12·5)	0·0
	Somalia	7 (6 to 8)	9 (8 to 11)	21 (19 to 23)	28 (24 to 33)	5·4% (4·7 to 6·2)	6·4% (4·9 to 8·5)	0·0
	South Sudan	29 (27 to 30)	34 (30 to 41)	83 (79 to 88)	100 (88 to 119)	9·3% (4·3 to 15·8)	11·1% (4·3 to 20·5)	0·0
	Tanzania	40 (37 to 43)	54 (42 to 71)	103 (95 to 111)	140 (108 to 183)	3·7% (3·1 to 4·3)	3·6% (2·3 to 5·4)	0·0
	Uganda	46 (43 to 50)	55 (48 to 62)	131 (121 to 142)	154 (135 to 176)	5·7% (4·5 to 7·6)	4·3% (2·6 to 6·8)	0·0
	Zambia	62 (58 to 67)	76 (59 to 102)	205 (191 to 222)	252 (193 to 336)	5·6% (5·1 to 6·1)	5·1% (3·4 to 7·4)	0·1
Southern sub-Saharan Africa								
	Botswana	464 (436 to 494)	1257 (1124 to 1392)	1144 (1073 to 1218)	3097 (2768 to 3429)	6·2% (5·7 to 6·6)	11·7% (8·0 to 16·4)	0·0
	Eswatini	226 (215 to 238)	340 (296 to 389)	593 (563 to 625)	893 (775 to 1021)	6·4% (6·0 to 6·8)	6·6% (4·4 to 9·0)	0·0
	Lesotho	125 (115 to 136)	220 (188 to 255)	390 (358 to 425)	686 (586 to 795)	13·0% (11·9 to 14·2)	14·7% (10·1 to 20·9)	0·0
	Namibia	410 (382 to 441)	806 (702 to 930)	964 (899 to 1039)	1897 (1652 to 2190)	9·4% (8·7 to 10·1)	15·0% (10·6 to 20·4)	0·0
	South Africa	478 (448 to 513)	766 (666 to 890)	1202 (1128 to 1290)	1927 (1675 to 2238)	9·1% (8·5 to 9·8)	14·2% (10·8 to 18·2)	0·0
	Zimbabwe	75 (69 to 83)	108 (90 to 130)	211 (194 to 231)	303 (253 to 364)	7·1% (6·5 to 7·8)	8·3% (5·8 to 11·5)	0·0
Western sub-Saharan Africa								
	Benin	29 (26 to 32)	42 (37 to 49)	79 (72 to 86)	116 (101 to 133)	2·2% (2·1 to 2·5)	2·2% (1·6 to 3·0)	0·2
	Burkina Faso	41 (39 to 44)	70 (64 to 78)	119 (113 to 125)	202 (183 to 224)	5·1% (4·9 to 5·4)	5·8% (4·1 to 8·2)	0·0
	Cameroon	49 (42 to 56)	77 (65 to 90)	121 (104 to 139)	192 (161 to 223)	3·1% (2·7 to 3·6)	3·2% (2·4 to 4·2)	0·0
	Cape Verde	183 (170 to 196)	325 (261 to 386)	380 (354 to 407)	676 (542 to 802)	5·0% (4·7 to 5·4)	7·1% (5·0 to 9·6)	0·0
	Chad	28 (25 to 33)	33 (28 to 40)	71 (62 to 83)	85 (71 to 101)	4·3% (3·7 to 4·9)	3·8% (2·8 to 5·3)	0·0
	Côte d'Ivoire	72 (66 to 79)	110 (94 to 129)	170 (155 to 186)	259 (221 to 302)	5·3% (2·6 to 10·6)	6·8% (3·0 to 14·0)	0·0
	The Gambia	39 (38 to 41)	49 (44 to 55)	118 (113 to 124)	147 (132 to 166)	5·0% (4·8 to 5·3)	4·6% (3·4 to 6·2)	0·0
	Ghana	72 (66 to 81)	121 (104 to 142)	189 (171 to 210)	316 (271 to 371)	3·7% (2·9 to 5·1)	4·6% (3·0 to 7·3)	0·0
	Guinea	50 (45 to 55)	81 (71 to 93)	124 (112 to 136)	201 (174 to 230)	4·3% (3·6 to 5·3)	4·4% (3·1 to 6·2)	0·0
	Guinea-Bissau	58 (55 to 62)	78 (71 to 85)	178 (167 to 190)	238 (215 to 259)	7·8% (6·9 to 8·6)	7·4% (5·3 to 10·0)	0·0
	Liberia	66 (62 to 70)	94 (81 to 114)	154 (145 to 164)	222 (191 to 267)	11·3% (8·5 to 15·7)	13·7% (8·2 to 23·1)	0·1
	Mali	30 (27 to 32)	39 (34 to 44)	80 (74 to 87)	105 (92 to 118)	3·0% (2·3 to 3·6)	2·5% (1·6 to 3·8)	0·0
	Mauritania	65 (59 to 71)	93 (81 to 106)	210 (192 to 231)	301 (263 to 342)	3·7% (3·2 to 4·2)	3·7% (2·7 to 5·2)	0·0
	Niger	30 (29 to 32)	37 (34 to 42)	71 (67 to 75)	87 (78 to 97)	5·5% (5·2 to 5·8)	5·5% (3·9 to 7·4)	0·0
	Nigeria	81 (73 to 90)	106 (91 to 123)	192 (173 to 213)	249 (215 to 290)	3·5% (3·2 to 3·9)	3·4% (2·5 to 4·8)	0·1
	São Tomé and Príncipe	117 (109 to 125)	183 (134 to 258)	234 (219 to 252)	367 (268 to 518)	5·6% (5·3 to 6·0)	6·3% (4·0 to 9·9)	0·0
	Senegal	66 (60 to 74)	90 (80 to 102)	158 (142 to 176)	215 (191 to 243)	4·4% (4·0 to 4·9)	4·4% (3·2 to 5·9)	0·1
	Sierra Leone	73 (66 to 81)	110 (95 to 128)	241 (217 to 269)	363 (312 to 422)	13·4% (12·0 to 14·9)	14·9% (10·5 to 20·5)	0·4
	Togo	40 (37 to 44)	62 (54 to 71)	96 (88 to 105)	146 (128 to 169)	5·2% (3·9 to 6·4)	5·9% (3·7 to 8·4)	0·0

Estimates in parentheses are 95% uncertainty intervals. GBD=Global Burden of Diseases, Injuries, and Risk Factors Study. GDP=gross domestic product.

* Estimates for Venezuela are presented as 2014 USD. $PPP=2020 purchasing-power parity-adjusted US$.

Between 1995 and 2019, inflation-adjusted health spending per person increased in 182 of the 204 countries and territories considered. For health spending for every country and year from 1995 to 2050, see the appendix (p 142). We estimate that global health spending will reach $14·3 trillion (95% UI 13·7–15·0) by 2050.

**Figure 1:**
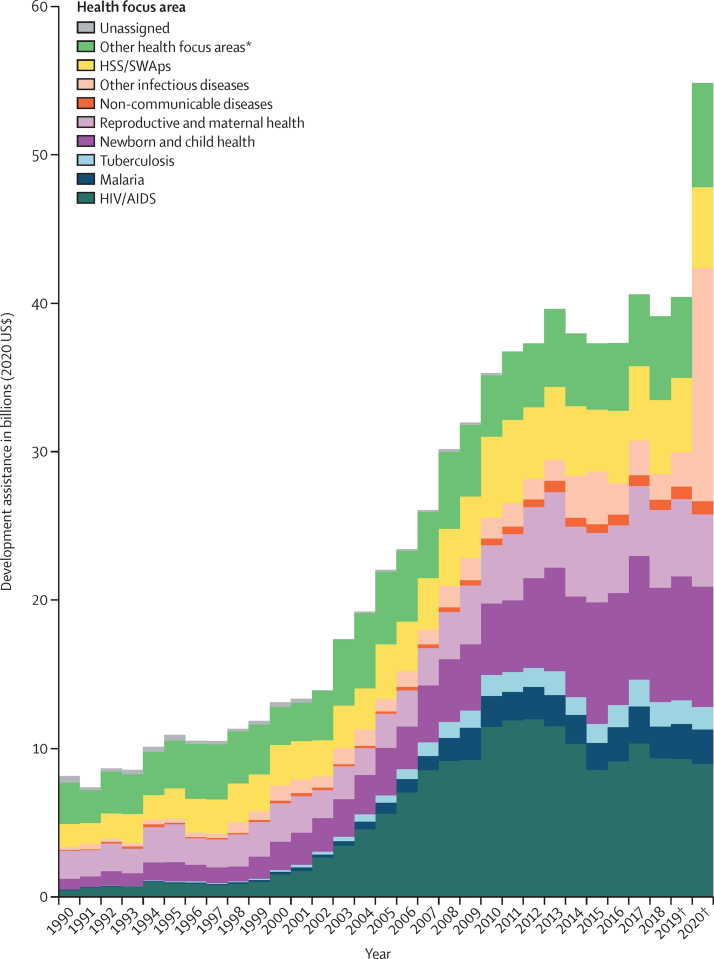
Development assistance for health by health focus area, 1990–2020 HSS/SWAps=health system strengthening and sector-wide approaches. *Other captures development assistance for health for which we have health focus area information but is not identified as being allocated to any of the health focus areas listed. Health assistance for which we have no health focus area information is designated as unassigned. †2019 and 2020 disbursement estimates are preliminary.

Between 2012 and 2019, development assistance for health contributions plateaued at an annualised rate of 1·2%. However, in 2020, total development assistance for health (including development assistance for health for COVID-19) amounted to $54·8 billion, a $14·0 billion (34·6%) increase from 2019. The increase is largely (96·5%) attributable to disbursements for the health response to COVID-19, which amassed $13·7 billion of health assistance (or 24·9% of total development assistance for health) in 2020 (figure 1). For year-on-year comparisons, we observed decreases in assistance targeted toward reproductive and maternal health (−6·8%), tuberculosis (−5·5%), and malaria (−2·2%). Meanwhile, development assistance for sector-wide approaches and health system strengthening increased (8·8%). Of note is the small proportion of health systems strengthening resources (14·9% in 2020) that have been targeted towards pandemic preparedness.

**Figure 2:**
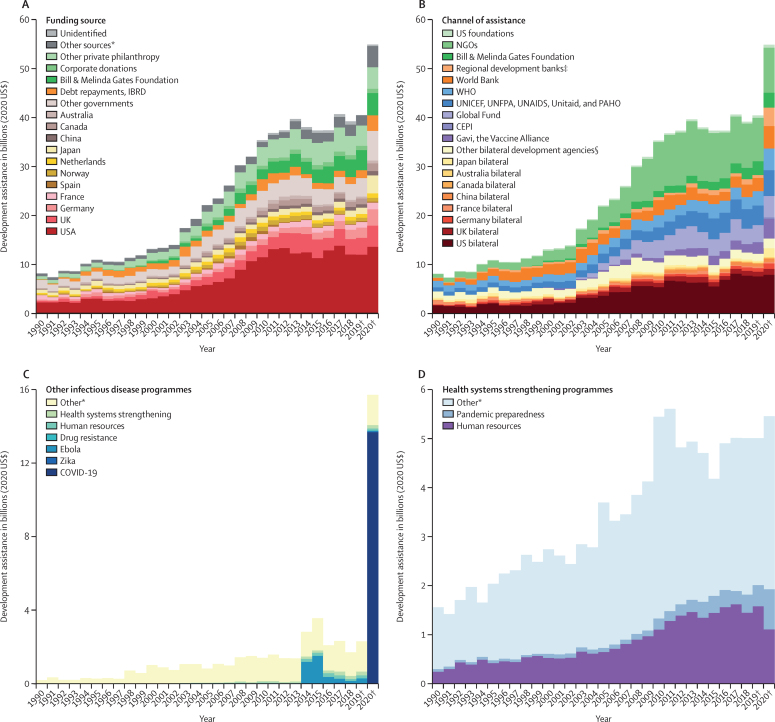
Development assistance for health, 1990–2020 (A) Development assistance for health by source of funding. (B) Development assistance for health by channel of assistance. (C) Development assistance for other infectious disease programme areas. (D) Development assistance for health systems strengthening programmes. CEPI=Coalition for Epidemic Preparedness Innovations. GAVI=Gavi, the Vaccine Alliance. HSS/SWAps=health systems strengthening and sector-wide approaches. IBRD=International Bank for Reconstruction and Development. NGO=non-governmental organisation. PAHO=Pan American Health Organization. UNFPA=UN Population Fund. *Other captures development assistance for health for which we have source information but is not identified as being allocated to any of the health focus areas listed. Health assistance for which we have no health focus area information is designated as unassigned. †2019 and 2020 disbursement estimates are preliminary. ‡Regional development banks include the African Development Bank, the Asian Development Bank, and the Inter-American Development Bank. §Other bilateral development agencies include Austria, Belgium, Denmark, Finland, Greece, Ireland, Italy, Luxembourg, the Netherlands, New Zealand, Norway, South Korea, Spain, Sweden, Switzerland, the United Arab Emirates, the European Commission, and the European Economic Area.

Figure 2A shows the main sources of development assistance for health in 2020. Most of the funding came from the USA, the UK, and the Bill & Melinda Gates Foundation. The key disbursing agencies for these resources were USA bilateral organisations, non-governmental organisations, and the World Bank (figure 2B).

**Figure 3:**
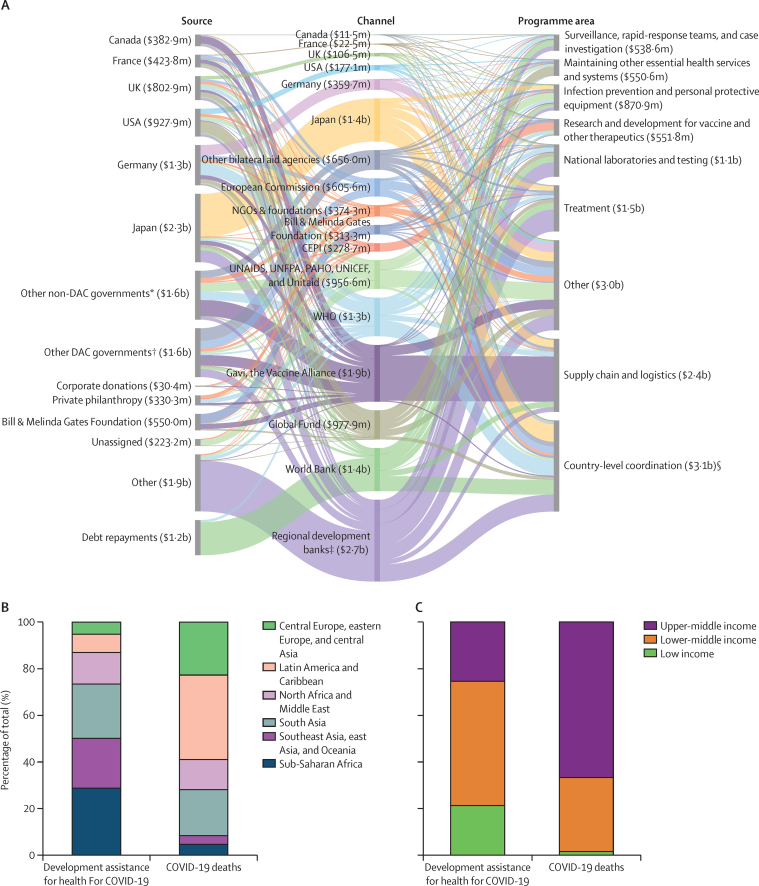
Distribution of development assistance for health for COVID-19 by programme area, recipient region, and income group, 2020 (A) Flow of development assistance for health disbursements from source to channel to programme area for COVID-19. Data are in million (m) or billion (b) 2020 US$. (B) Percentage of disbursed development assistance for health for COVID (excluding global initiatives) and percentage of total deaths from COVID-19 by GBD super-region. (C) Percentage of disbursed development assistance for health for COVID-19 (excluding global initiatives) and percentage of total deaths from COVID-19 by World Bank income group. The COVID-19 burden is represented by the percentage of total COVID-19 deaths in 2020 for lower-middle-income countries only, as high-income countries to not receive development assistance. CEPI=Coalition for Epidemic Preparedness Innovations. DAC=Development Assistance Committee. GAVI=Gavi, the Vaccine Alliance. GBD=Global Burden of Diseases, Injuries, and Risk Factors Study HSS/SWAps=health systems strengthening and sector-wide approaches. IBRD=International Bank for Reconstruction and Development. NGO=non-governmental organisation. PAHO=Pan American Health Organization. UNFPA=UN Population Fund. *Other non-DAC governments include Afghanistan, Angola, Argentina, Azerbaijan, Bangladesh, Bhutan, Brazil, Brunei, Bulgaria, Côte d'Ivoire, Cameroon, Central African Republic, Chad, China, Colombia, Croatia, Democratic Republic of the Congo, Egypt, Estonia, Ethiopia, Gabon, Guinea, India, Indonesia, Iran, Iraq, Jamaica, Jordan, Kenya, Kuwait, Latvia, Lebanon, Libya, Lithuania, Madagascar, Malaysia, Malta, Monaco, Myanmar, Nigeria, Oman, Pakistan, Palestine, Peru, Qatar, Romania, Russia, São Tomé and Príncipe, Saudi Arabia, Serbia, Singapore, South Africa, South Sudan, Sudan, Syria, Taiwan (province of China), Thailand, Togo, Turkey, Uganda, Ukraine, United Arab Emirates, Yemen, and Zimbabwe. †Other DAC governments include Australia, Austria, Belgium, Czechia, Denmark, Finland, Greece, Hungary, Iceland, Ireland, Italy, Luxembourg, the Netherlands, New Zealand, Norway, Poland, Portugal, Slovakia, Slovenia, South Korea, Spain, Sweden, and Switzerland. ‡Development banks include the African Development Bank, the Asian Development Bank, and the Inter-American Development Bank. UN agencies include PAHO, UNAIDS, UNFPA, UNICEF, and Unitaid. §Country-level coordination includes planning, monitoring, and evaluations; risk communication and community engagement; and travel restrictions.

Figure 3A shows the flow of development assistance for health contributions toward COVID-19 from the original source of funds, through the disbursing agency, and to the targeted programme area of focus as available for 2020. A total of $13·7 billion was disbursed in 2020 toward addressing the health-related effects of COVID-19 in low-income and middle-income countries. Of this total, the largest bilateral contributors were Japan ($2·3 billion), Germany ($1·3 billion), and the USA ($0·9 billion). Most of Japan's support ($1·4 billion) was disbursed through its own bilateral agencies, mostly the Japanese International Cooperation Agency, and was targeted to India ($360·6 million), Morocco ($204·7 million), and Indonesia ($194·4 million). Sub-Saharan Africa ($2·7 billion); south Asia ($2·2 billion); and southeast Asia, east Asia, and Oceania ($2·0 billion) were the main geographical super-regions that received COVID-19 funds. Both the UK and Germany primarily supported the Coalition for Epidemic Preparedness Innovations (CEPI; $64·3 million from the UK and $56·9 million from Germany). The Asian Development Bank, Gavi, and the Global Fund are the international development agencies that channelled most of the resources committed or disbursed for COVID-19.

Figure 3B shows the allocation of development assistance for health for COVID-19 in the GBD super-regions. 23·5% of donors' COVID-19 resources were for global initiatives and did not directly target a single country or region. Examples of such investments include vaccine development and procurement, and partner organisations' coordination. The specific region to receive the most development assistance for health for COVID-19 was sub-Saharan Africa (28·8%). This figure also illustrates the poor alignment between COVID-19 assistance for health and COVID-19 burden. In 2020, 34·3% of recorded COVID-19 deaths in low-income and middle-income countries occurred in Latin America, but countries in Latin America received 7·7% of all development assistance for health for COVID-19 allocated to any specific country.

**Figure 4:**
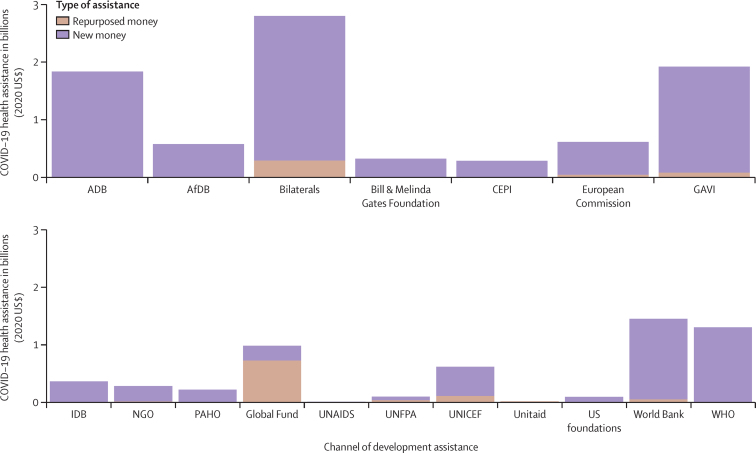
Type of health assistance provided towards the COVID-19 pandemic, 2020 This figure shows the amount of global development assistance for health for the COVID-19 pandemic that was repurposed versus new spending. Overall, 90·1% of global development assistance for health was new money, and 9·9% was repurposed money. ADB=Asian Development Bank. AfDB=African Development Bank. CEPI=Coalition for Epidemic Preparedness Innovations. GAVI=Gavi, the Vaccine Alliance. Global Fund=The Global Fund to Fight AIDS, Tuberculosis and Malaria. IDB=Inter-American Development Bank. NGO=Non-governmental organisation. PAHO=Pan American Health Organization. UNFPA=UN Population Fund.

The proportion of COVID-19 funds that were new resources versus those that were previously budgeted and repurposed to COVID-19 is shown in figure 4. Of the total $13·7 billion in development assistance for health for COVID-19, $1·4 billion (9·9%) was money from already existing projects that was redirected toward COVID-19-related response activities. $12·3 billion (90·1%) of this funding was additional, previously unbudgeted resources. Across the disbursing entities, the Asian Development Bank ($1·8 billion) and Gavi ($1·8 billion) disbursed most of their resources as new money. Relative contributions for health and non-health response are shown in table 2. The health response accounted for 9·8% of the overall response, with the non-health response making up the dominant share ($125·4 billion) of the disbursed response. The types of contributions made and disbursed across the various disbursing agencies as part of the health-related COVID-19 response are shown in table 3. Grants made up most contributions, at $8·7 billion (63·6%), compared with loans at $4·8 billion (34·9%).

**Table 2:** Estimates of global COVID-19-related disbursements and commitments of health and non-health funds, 2020

	**Total**	**Health-related COVID-19 funds**	**Non-health-related COVID-19 funds**
Commitment	243·804	61·5121	182·2919
Disbursement	139·1044	13·66932	125·4351

Units are billion US$. Using IHME's estimate of health-related disbursements, we estimated commitments and disbursements by health and non-health spending using data from the United Nations' Office for the Coordination of Humanitarian Affairs Financial Tracking Service, compiled by the Center for Disaster Protection.^38^ In the case where a grant was allocated to multiple sectors (health, as well as one or more non-health sectors), we calculated the funds attributed to health spending by multiplying the total grant amount by 1 divided by the number of sectors represented. Proportions of global commitments and disbursements by type of spending (health and non-health) were multiplied by IHME's estimate of health-related disbursements. IHME=Institute for Health Metrics and Evaluation.

**Table 3:** Development assistance for health specific to COVID-19 in 2020, by channel and type of assistance (millions of 2020 US$)

	**Total**	**New funds**	**Repurposed funds**	**Commitment**	**Disbursement**	**Grant**	**Loan**
African Development Bank	566·3	566·3	..	589·1	566·3	137·7	428·5
Asian Development Bank	1817·0	1817·0	..	2103·1	1817·0	184·0	1633·0
Bilateral development agencies	2775·5	2493·4	282·0	3057·3	2775·5	1542·1	1032·7
Bill & Melinda Gates Foundation	313·3	313·3	..	313·3	313·3	313·3	..
Coalition for Epidemic Preparedness Innovations	278·7	278·7	..	598·0	278·7	227·8	51·0
European Commission	605·6	565·9	39·6	718·9	605·6	605·6	..
Gavi, the Vaccine Alliance	1903·3	1827·2	76·1	1903·3	..	1903·3	..
The Global Fund	977·9	257·3	720·7	977·9	..	977·9	..
Inter-American Development Bank	363·9	363·9	..	..	363·9	..	363·9
Non-governmental organisation	280·6	267·5	13·1	153·1	280·6	280·6	..
Pan American Health Organization	218·7	218·7	..	..	218·7	218·7	..
UNAIDS	9·0	0·6	8·4	27·3	9·0	9·0	..
United Nations Population Fund	96·9	56·5	40·4	30·3	74·2	96·9	..
UNICEF	614·2	506·3	107·9	140·0	485·9	614·2	..
Unitaid	17·7	..	17·7	..	17·7	17·7	..
USA foundations*	93·7	93·7	..	93·7	..	93·7	..
World Bank International Bank for Reconstruction and Development	913·1	873·1	40·0	694·5	913·1	..	913·1
World Bank International Development Association	528·5	519	9·4	442·4	528·5	181·0	347·4
WHO	1295·5	1295·5	..	7508·4	1295·5	1295·5	..
Total	13 669·4	12 313·9	1355·3	19 350·6	10 543·5	8699	4769·6

Spending data are reported in millions of 2020 US$, where estimates were available. In-kind contributions such as personal protective equipment was assumed to be grants. $200·7 million in bilateral development agencies funds were not able to be identified as either grants or loans.

* Commitments of at least $10 000 from USA foundations internationally and in the USA.

**Figure 5:**
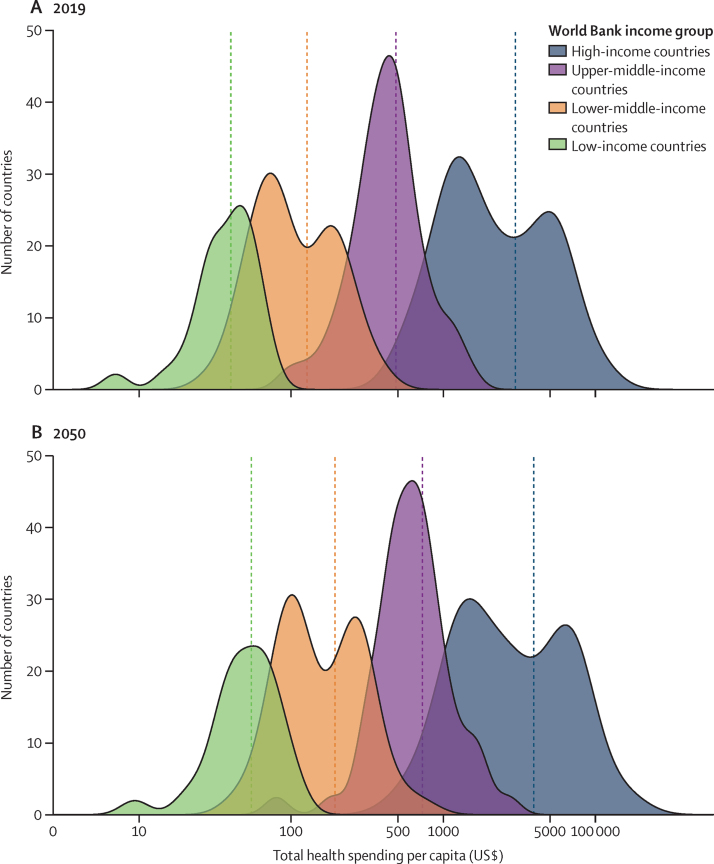
Distribution of total health spending per capita in 2019 and 2050 by income group (A) 2019. (B) 2050. Estimates are reported in 2020 US$. The x-axes are presented in a natural logarithmic scale. Vertical lines represent the mean for each distribution. The World Bank income classification does not include Cook Islands, Niue, and Tokelau. The Cook Islands and Niue were assigned to the high-income group, and Tokelau was assigned to the upper-middle-income group.

Relative to global spending, development assistance for health for COVID-19 remained small in 2020 (0·1%), and it is too early to know how much domestic spending has focused on COVID-19. Although there is a great deal of uncertainly, future spending on health is expected to continue to climb, albeit at a slower pace than anticipated before the pandemic. We estimated that global spending on health will reach $9·9 trillion (95% UI 9·7–10·1) in 2030 and $14·3 trillion (13·7–15·0) in 2050. Health spending over time for each income group is shown in figure 5, which highlights that health spending disparities are expected to persist and that there is a great deal of variation in total spending within income groups. We estimate that in 2050, low-income countries will spend $46 (95% UI 44–47) per person, lower-middle-income countries will spend $150 (141–159) per person, upper-middle-income countries will spend $1001 (922–1083) per person, and high-income countries will spend $8536 (8074–9032) per person.

## Discussion

The context in which COVID-19 has spread globally is one of grave inequality in access to health services.^39^, ^40^ This research highlights how this inequality exists at the national level within the health sector. In 2019, national health spending ranged from $7 to $11 345 per person, with government health spending ranging from $2 to $6578 per person (table 1, appendix p 142). Development assistance for health reached $40·4 billion in 2019 and increased to $54·8 billion in 2020 because of the additional resources provided in response to COVID-19. Although development assistance for health makes up less than 1·0% of health spending in most middle-income countries, development assistance for health was 24·6% (95% UI 24·0–25·1) of total health spending in low-income countries. Health spending per person has grown almost universally between 1995 and 2019 and is expected to rise in 195 of 204 countries and territories to 2050, but expected spending remains highly unequal. The finding of rising global health spending before the onset of the pandemic is aligned with what is reported in the WHO 2020 Global Health Spending Report.^4^ In both our study and WHO spending report, the pandemic is expected to perpetuate already existing vulnerabilities in global health spending, such as inequalities in spending across and within income groups.

Our estimates of development assistance for health for COVID-19 show that in 2020, a total of $13·7 billion was disbursed to aid the health response in low-income and middle-income countries. This figure constituted over a quarter of all development assistance for health, and is evidence of how resources can be scaled quickly when needed. Resources focused on country-level coordination, supply chain and logistics, case management and treatment, surveillance, rapid-response teams, case investigation, maintaining essential health services and systems, and infection prevention and control have received modest contributions.

Previous estimates of development assistance for health for COVID-19 vary greatly, primarily because each set of estimates focused on tracking different aspects of the international response. Our total estimates of resources committed is similar to that reported by the Economist Intelligence Unit COVID-19 Health Funding Tracker, which estimated $13·8 billion pledged as of December, 2020, although it excluded support for individual countries. Our estimates of resources committed are much smaller in magnitude, however, than what is reported by the other trackers. As of its last update in April 2020, the Kaiser Family Foundation estimated $19 billion had been pledged toward the health sector response.^24^ As of Jan 20, 2021, the Centre for Disaster Protection online visualisation tool reported $100·7 billion had been committed in loans (concessional and non-concessional) and $14·24 billion had been committed in grants.^25^ A key difference between these estimates and our own is that most of the other research on this topic has not estimated disbursements, and instead focused on commitments or pledges. In practice, disbursements are generally less than commitments. Moreover, in the case of unexpected crisis, disbursing resources can take much longer than the time span that we have predicted. As of Dec 13, 2020, the Devex database reported 987 programme announcements worth $193·1 billion, 810 grants worth $2·9 billion, 2171 tenders worth $4·7 billion, and 1133 contracts worth $1·7 billion.^26^ Unlike our estimates, these have a much broader focus and include support for the humanitarian response (outside of the health sector).

Only a few studies have estimated the resources needed for an effective pandemic response.^3^, ^12^ Previously, the Commission on a Global Health Risk Framework for the Future, which was set up after the Ebola epidemic, estimated that annually $4·5 billion was needed globally for pandemic preparedness, by contrast with an expected annual loss of more than $60 billion from potential pandemics. One study^12^ on a sample of 73 low-income and middle-income countries estimated that the additional cost of responding to the COVID-19 pandemic was between $33 billion and $62 billion.

One consequence of the COVID-19 pandemic that is different from other recent epidemics, such as the Ebola outbreak in 2014 or MERS-CoV in 2012, is the significant economic toll on high-income countries. Precisely how the economic recession will affect governments' willingness to maintain record development assistance resources for low-income and middle-income countries is not known. Some countries have already restructured their aid budgets to prioritise addressing domestic challenges. For instance, the UK has passed a bill to reduce the foreign aid commitment from 0·7% of GDP to 0·5% of GDP to enable management of the national budget.^41^ In addition to this, Kobayashi and colleagues^42^ suggest that the global scale of the COVID-19 pandemic might have weakened public sentiment about contributing public resources abroad, given the immense challenges at home. In the next decade, development assistance for health disbursements might grow at a substantially slower pace and could rely more heavily on private philanthropy and multilateral institutions.

Nonetheless, as the pandemic has evolved, so have calls for support for low-income and middle-income countries. These calls for support are not only to address immediate health needs but also to provide additional social safety net support and economic relief. Multilateral institutions such as the IMF, the World Bank, and regional development banks including the Asian Development Bank, the Inter-American Development Bank, and the African Development Bank have all responded promptly to these requests with various packages. However, much of this support consists of loans that will add to national debt, with potential for long-term effects on growth and the ability to increase social spending.^43^ Additionally, there are calls for debt repayment suspension and a repurposing of such resources towards bolstering struggling economies.

The pandemic's effect on other essential service provisions has been notable, largely because addressing the urgent demands of the pandemic required a realignment of health resources, including personnel, hospital supplies, services, data, and funding, and has made accessing health services safely substantially more challenging.^44^ At the beginning of the pandemic, when some health-care systems were overwhelmed by the unfolding crisis, the complete refocus of resources seemed to be the most logical response. However, over time, this burden on the health system has made evident the importance of strong health systems worldwide in maintaining global health security, and made it even more obvious that targeting development assistance contributions to the strengthening of the broader health system is a crucial investment for global health security overall.^45^, ^46^ Although contributions toward health system strengthening have improved, the relative proportion of development assistance funds that are targeted toward such activities remains small, with an even smaller amount allocated toward pandemic preparedness.^47^ The pandemic has shown how quickly a local epidemic can develop into a global crisis, and the need for global activity and cooperation to be commensurate with this challenge.

The COVID-19 pandemic has also had ramifications for the Sustainable Development Goals (SDGs) more broadly. According to the SDGs Report 2020,^48^, ^49^ globally, progress has been insufficient and the pandemic has only worsened the precarious progress made on most of the goals. For instance, in several countries in sub-Saharan Africa it seemed the health toll of the pandemic was less than expected in 2020, but the economic fallout due to public health measures such as lockdowns might have had much more severe consequences and pushed an additional 23 million people into poverty.^50^, ^51^ The mitigation strategies used to reduce the impact of the pandemic have also led to a meaningful global response. So far, development assistance contribution that falls outside of the health sector has been much larger than health-related contributions ($125·4 billion *vs* $13·7 billion). This reflects how the effect of the COVID-19 pandemic has reverberated outside of the health sector, affecting the social, economic, and environmental sectors in most countries.

Another challenge associated with raising and disbursing record-setting amounts of development assistance is the crucial task of allocating these scarce resources. Despite the differences in the burden of the pandemic across geographical regions, the patterns in the allocation of development assistance for health for COVID-19 remain largely the same as development assistance for health in the past, with Latin America and the Caribbean receiving little support despite their high burden. In future, key contributions must be aligned with where they are needed the most.

The distribution of the COVID-19 vaccine has generated much public discourse.^52^ Although the initial discussion in popular media and among policy makers focused on national interests, the Access to COVID-19 Tools (ACT) Accelerator partnership was launched in April, 2020, by WHO, the European Commission, and the French Government to ensure pooled and equitable distribution of tools to fight COVID-19. ACT-Accelerator was estimated to need $33 billion for its work. Of ACT-Accelerator's four key pillars, the pillar that focuses on vaccines is the COVID-19 Vaccines Global Access Facility, which is led by Gavi, WHO, and CEPI. Leveraging its previous experience with managing advanced market commitment for immunisation, Gavi was tasked with facilitating the procurement of vaccines for 92 middle-income countries through the COVID advance market commitment. The objective was to raise $2 billion before the end of 2020 to enable it to support access to vaccines irrespective of income.^53^ As of August, 2021, $17·9 billion had been committed to the ACT Accelerator partnership.

COVID-19 has highlighted the nuanced role health systems play in providing health security. Although robust health systems—with capacity to test, track, and treat those with the virus and ability to access and provide vaccines quickly and efficiently—are necessary for keeping deaths from COVID-19 at bay, it has also been made clear that a robust health system alone is not sufficient.^54^ COVID-19 mortality rates have varied greatly across the globe, but there is little consensus on what is driving this variation, with key historical measures of pandemic preparedness highlighting capacity gaps, but not accurately predicting where the outcomes will be the best or worst.^55^ To be successful in fighting the virus and preventing substantial loss, a constellation of characteristics that fall both inside and outside of the traditional health system is necessary. These characteristics include leadership, public health system capacity, social safety nets, and trust in the systems providing information and care.^56^, ^57^ A painful lesson of this pandemic is that global health security is only as strong as its weakest link. It is crucial that those working in the health sector ensure that the entire health system is in fact robust, and that policy makers outside of the health sector also be prepared for many potential crises that can accompany a pandemic.

This research highlights the context in which COVID-19 spread quickly across the globe—one of enormous variation in health-care spending. Robust health systems require more resources, and those working in government and advocacy outside of the health sector are encouraged to see COVID-19 and the staggering loss of life and broader setbacks in global development as indicators of the importance of investing in health in and outside the health system, and building governments and social systems that can also contribute towards health security.

This study has several limitations. The data were extracted from multiple sources with different reporting structures and components included. We kept definitions and methods as consistent as possible across data sources to ensure a reliable estimate of resources. Furthermore, given that the pandemic is ongoing and development projects take some time to reach implementation, disbursement could still be low even where commitments have been made. Moreover, global health resource tracking does not allow comparable tracking of how domestic resources are spent on health and does not allow for comprehensive estimates of domestic health spending on COVID-19. More information is needed to assess how governments, households, and private organisations are spending health resources, and what resources are available for responding to health emergencies. Health financing reporting, especially in many low-income and middle-income countries, but also in high-income countries, is constrained by how timely domestic health spending data are available. Health financing data are not collated in a manner that is shareable or comparable across time and country. As countries deliberate on ways to rebuild systems after the pandemic, the health financing architecture and equity are important aspects to consider. Finally, our quantification of uncertainty captures some types of uncertainty, but not all types of uncertainty. It is included here as a relative quantification of where we have the most confidence in our estimates.

Health spending in 2019, before the COVID-19 pandemic, ranged widely, and marks tremendous variation in the access to essential health services and universal health coverage. Much more money is needed to fully address the effects of the pandemic in most low-income and middle-income countries, along with access to key tools such as vaccines. Moreover, long-term projections suggest that health inequalities are likely to persist and that international efforts to invest in global public goods related to global pandemic preparedness are essential. Resources are needed urgently to mitigate the loss associated with the COVID-19 pandemic and fund systems that can prevent and respond quicker to the next global health crisis.

## Data sharing

Data used for this study were extracted from publicly available sources that are listed in the appendix (pp 7, 28–33, 65, 81–82, 92, 117). Further details are available on the Global Health Data Exchange website at http://ghdx.healthdata.org/series/financing-global-health-fgh

## Declaration of interests

D McCracken's position was supported in part through the Wellcome Trust, and by the Department of Health and Social Care using UK aid funding managed by the Fleming Fund. R Ancuceanu reports consulting fees from AbbVie and AstraZeneca; payment or honoraria for lectures, presentations, speakers bureaus, manuscript writing, or educational events from Sandoz, AbbVie, and Braun Medical; and support for attending meetings or travel from AbbVie and AstraZeneca, all outside the submitted work. M Ausloos and C Herteliu report grants or contracts from the Romanian National Authority for Scientific Research and Innovation (CNDS-UEFISCDI), project number PN-III-P4-ID-PCCF-2016-0084, outside the submitted work. C Herteliu reports grants or contracts from CNDS-UEFISCDI, project number PN-III-P2-2.1-SOL-2020-2-0351, outside the submitted work. S Bhaskar reports an unpaid leadership or fiduciary role in a board, society, committee or advocacy group, with the Rotary Club of Sydney Board of Directors, outside the submitted work. R Busse reports grants or contracts from Berlin University Alliance (COVID pre-exploration project), outside the submitted work. S M S Islam reports grants or contracts from National Health and Medical Research Council (NHMRC) and the National Heart Foundation of Australia, all outside the submitted work. K Krishan reports non-financial support from UGC Centre of Advanced Study phase II, Department of Anthropology, Panjab University, Chandigarh, India, outside the submitted work. M J Postma reports grants or contacts from Merck Sharp & DDohme, GlaxoSmithKline, Pfizer, Boehringer Ingelheim, Novavax, Bayer, Bristol Myers Squibb, AstraZeneca, Sanofi, IQVIA, BioMerieux, WHO, EU, Seqirus, FIND, Antilope, DIKTI, LPDP, and Budi; consulting fees from Merk Sharp & Dohme, GlaxoSmithKline, Pfizer, Boehringer Ingelheim, Novavax, Quintiles, Bristol Myers Squibb, Astra Zeneca, Sanofi, Novartis, Pharmerit, IQVIA, and Seqirus; participation on a Data Safety Monitoring Board or Advisory Board to Asc Academics as Advisor; and stock or stock options in Health-Ecore and PAG, all outside the submitted work. M G Shrime reports grants or contracts from the Iris O'Brien Foundation; payment or honoraria for lectures, presentations, speakers bureaus, manuscript writing or educational events from Brightsight speakers; and leadership or fiduciary role in board, society, committee or advocacy group, paid or unpaid with Pharos Global Health Advisors as a board member. J A Singh reports consulting fees from Crealta/Horizon, Medisys, Fidia, Two labs, Adept Field Solutions, Clinical Care options, Clearview healthcare partners, Putnam associates, Focus forward, Navigant consulting, Spherix, MedIQ, UBM, Trio Health, Medscape, WebMD, and Practice Point communications, and the National Institutes of Health and the American College of Rheumatology; payment or honoraria for lectures, presentations, speakers bureaus, manuscript writing, or educational events from Simply Speaking; support for attending meetings and travel from OMERACT; leadership or fiduciary role in other board, society, committee, or advocacy group, paid or unpaid, with OMERACT as a member of the steering committee, with the US Food and Drug Administration Arthritis Advisory Committee, with the Veterans Affairs Rheumatology Field Advisory Committee as a member, and with the UAB Cochrane Musculoskeletal Group Satellite Center on Network Meta-analysis as a Director and Editor; stock or stock options in TPT Global Tech, Vaxart pharmaceuticals, and Charlotte's Web Holdings; and previously owned stock options in Amarin, Viking, and Moderna pharmaceuticals, all outside the submitted work. All other authors declare no competing interests.
