# Supplementary material for: Tracking development assistance for health and for COVID-19: a review of development assistance, government, out-of-pocket, and other private spending on health for 204 countries and territories, 1990–2050
Source: Lancet. 2021 Oct 9;398(10308):1317–43. doi: 10.1016/S0140-6736(21)01258-7 (PMC8457757; doi:10.1016/S0140-6736(21)01258-7)
Supplement: Supplementary appendix [file mmc1.pdf]

THE LANCET

Supplementary appendix

This appendix formed part of the original submission and has been peer reviewed. We post it as supplied by the authors.

Supplement to: Global Burden of Disease 2020 Health Financing Collaborator Network. Tracking development assistance for health and for COVID-19: a review of development assistance, government, out-of-pocket, and other private spending on health for 204 countries and territories, 1990–2050. *Lancet* 2021; published online Sept 22. http://dx.doi.org/10.1016/S0140-6736(21)01258-7.

SUPPLEMENTARY METHODS ANNEX

Tracking development assistance for health and for the COVID-19 pandemic: a review of development assistance, government, out-of-pocket, and other private spending on health for 204 countries, 1990–2050

Version: August 19, 2021

Global Burden of Disease Health Financing Collaborator Network

Table of Contents

SECTION 1. METHODS OVERVIEW

S1.1 Overview

S1.2 Estimating domestic health spending: 1995-2018

S1.3 Estimating development assistance for health: 1990-2020

S1.4 Estimating gross domestic product for 2020 and 2021 that were COVID-19 sensitive

S1.5 Reporting and uncertainty analysis

SECTION 2. TRACKING TOTAL HEALTH SPENDING AND ITS COMPONENTS

S2.1 Overview

S2.2 Statistical model to fill missingness in health expenditure variables

S2.3 Currency exchange and deflation

SECTION 3. TRACKING DEVELOPMENT ASSISTANCE FOR HEALTH

S3.1 Overview

S3.2 Tracking development assistance for health from bilateral aid agencies and the European Commission

S3.3 Tracking development assistance for health from the development banks

S3.4 Tracking contributions from the Global Fund and Gavi

S3.5 Tracking expenditure by United Nations Agencies active in the health domain

S3.6 Tracking development assistance for health from private foundations

S3.7 Tracking non-governmental organizations

S3.8 Tracking Development Assistance for health from China

S3.9 Calculating the technical assistance and program support component of development assistance for health from loan-and grant-making channels of assistance

S3.10 Regular development assistance for health References

S3.11 Tracking Development Assistance for Health: incorporating COVID-19

SECTION 4. FORECASTING HEALTH SPENDING FROM 2020 THROUGH 2050

S4.1 Data

S4.2 Covariates

S4.2.1 List of Forecasted Variables

S4.2.2 Covariates Used for Forecasting

S4.3 Ensemble Modeling

S4.3.1 Sub-model Setup

S4.3.2 Covariates

S4.3.3 Convergence to Global Growth Rates

S4.3.4 Specifications

S4.4 Inclusion and Exclusion Criteria

S4.5 Creating the Forecasts

S4.5.1 Ranking Sub-Models

S4.5.2 Uncertainty Estimation

S4.6 Ad-hoc Draws Correlation

S6.6.1 Motivation

S6.6.2 Bivariate Correlated Distributions

S4.7 Future Health Scenarios

S6.7.1 Long-term Growth Regressions

S6.7.2 Forecasting Better and Worse Scenarios

S6.7.3 Uncertainty Estimation

S4.8 Package and Architecture

S6.8.1 Architecture

S6.8.2 Template Model Builder

S4.9 Tables and Figures

SECTION 5. AUTHORS' CONTRIBUTIONS STATEMENT

Abbreviations

ADB	Asian Development Bank
AfDB	African Development Bank
BMGF	Bill & Melinda Gates Foundation
CRS	Creditor Reporting System
DAC	Development Assistance Committee
DAH	Development assistance for health
EC	European Commission
GDP	Gross domestic product
Global Fund	Global Fund to fight AIDS, Tuberculosis and Malaria
GGHE	General government health expenditure
GHED	Global Health Expenditure Database
GHES	Government health spending (Aggregate of "Social insurance contributions" and "Transfers from government domestic revenue (allocated to health purposes)", as defined in GHED)
HSS	Health System Strengthening
IBRD	International Bank for Reconstruction and Development
IDA	International Development Association
IDB	Inter-American Development Bank
LCU	Local currency units
NASA	National AIDS Spending Assessments
NGO	Non-governmental organization
NHA	National Health Account
NPISH	Non-profit institutions serving households
ODA	Official development assistance
OECD	Organisation for Economic Co-operation and Development
OOP	Out-of-pocket health spending (defined in GHED: “Other revenues from households n.e.c")
PAHO	Pan American Regional Office for WHO
PI	Private insurance
PPP	Prepaid private health spending (Aggregate of "Voluntary prepayment", "Other revenues from corporations n.e.c.", "Other revenues from NPISH n.e.c.", and "Compulsory prepayment (Other, and unspecified, than FS.3)", as defined in GHED)

SHA 2011	System of Health Accounts 2011
SWAps	Sector Wide Approaches
TB	Tuberculosis
THE	Total health spending (defined in GHED: “Current health expenditure by revenues of health care financing schemes”)
UNICEF	United Nations Children’s Fund
UNAIDS	Joint United Nations Programme on HIV/AIDS
UNFPA	United Nations Population Fund
USD	US dollars
VolAg	Report of Voluntary Agencies
WHO	World Health Organization

SECTION 1. METHODS OVERVIEW

S1.1 Overview

The methods presented here summarize the various components of the estimation process; interested readers are encouraged to consult the appendix for additional details on data sources, methods, and for additional results presented in alternative units. “Health spending” is defined as money spent on services, supplies, and basic infrastructure to deliver health care, using the same definition employed by the System of Health Accounts 2011 and the WHO Global Health Expenditure Database (GHED).[1,2] Health “financing” and “funding” are used interchangeably to refer to the source, as opposed to the utilization, of financial resources. “Economic development” refers to GDP per capita.

We estimated health spending from four main sources – government, out-of-pocket, prepaid private, and development assistance for health (DAH) – for 204 countries and territories. For brevity, “countries and territories” are referred to only as “countries”, all of which are categorized into four World Bank income groups (high-, upper-middle-, lower-middle-, and low-income) and seven Global Burden of Disease (GBD) super-regions (Central Europe, Eastern Europe, and Central Asia; GBD high income; Latin America and Caribbean; North Arica and Middle East; South Asia; Southeast Asia, East Asia, and Oceania; and sub-Saharan Africa). Data tracking government, out-of-pocket, and prepaid private health spending, which together comprise total domestic health spending, were available from 1995 through 2018. Government health spending includes social health insurance and mandated private health insurance, as well as government public health programmes. Out-of-pocket health spending includes health care spending paid by the patient or his or her household, excluding insurance premiums paid in advance of care. Prepaid private health spending includes voluntary private insurance and non-governmental agency spending on health.

Estimates of DAH, defined as the financial and in-kind contributions from major development agencies to low- and middle-income countries for the purpose of maintaining or improving population health, were generated from 1990 through 2020, although transfers to recipient countries can only be estimated until 2018. In other words, the total amount of DAH, by source, is estimated through 2020, but is not allocated by recipient country for 2019 and 2020. The sum of domestic health spending and DAH, net of the administrative costs needed to run the development agencies, form the envelope of total health spending for each country and year.

Domestic health spending from each of the three sources was projected for each country from 2019 to 2050, and DAH was projected from 2019 to 2050, by modelling rates of change across time. These models incorporate country-specific time trends that attenuate across time and converge to the global average, consider a broad set of covariates and time-series modelling techniques, and propagate four types of uncertainty - model, data, parameter, and fundamental uncertainty.

S1.2 Estimating domestic health spending: 1995–2017

We extracted data on gross domestic product (GDP) per capita from five leading sources of these estimates (World Bank, International Monetary Fund [IMF], United Nations, Penn World Tables, and Maddison) and built from methods described by James and colleagues to generate a single series of GDP per capita using Gaussian processes.[3-7] This method incorporated data from all five GDP series and also propagated uncertainty through the estimates.[8] The resulting series spans 204 countries from 1950 to 2019, and larger uncertainty intervals highlight countries where the input data from the five sources was discordant or estimates were missing.

We extracted data from the WHO's Global Health Expenditure Database (GHED) on government domestic revenue transfers allocated for health, compulsory prepayment, voluntary prepayment, social insurance contributions, and other domestic revenue from households, corporations, and non-profit institutions serving households.[9] Data from GHED excludes spending on major investments such as hospital construction, health worker education and training, and research and development. Countries report these data and the data source to WHO; when possible, these are based on National Health Accounts, but alternative sources are used in many cases. Health spending estimates were extracted in current national currency units, deflated 2020 national currency units, and exchanged to 2020 US dollars. Deflator series and exchanges rates used were based on those reported in the IMF World Economic Outlook.[5]

We estimated domestic government spending on health by aggregating transfers from government domestic revenue for health purposes, compulsory prepayment, and social insurance contributions. Then, to estimate domestic prepaid private health spending, we aggregated voluntary prepayment, other domestic revenues from non-profit institutions serving the household, and other domestic revenues from corporations. All payments by households other than social insurance contributions were designated as out-of-pocket spending. To generate domestic health spending estimates in purchasing-power parity-adjusted (PPP) dollars, we multiplied the health spending fractions by GDP per capita measured in 2020 PPP dollars.

The extracted data were evaluated for quality. Some of the extracted data points were not tied to an underlying data source and were estimated using a diverse set or poorly defined methods. Furthermore, for a given country, some data varied substantially across time. To overcome these concerns, we used a spatiotemporal Gaussian process regression model to estimate health spending across time, country, and spending category.[10] To prevent data with insufficiently described estimation methods or without data source identification from substantially influencing our model estimation, each data point was assessed and assigned a weight between one and five using the point-specific metadata provided in the GHED. We based weights on metadata completeness, documented source information, and documented methods for estimation. The distribution of data points assigned to each of the five data quality scores, by country income group, is presented in the appendix. Our guidelines for assessing the metadata, a detailed description of how the weights were created, and additional information about the spatiotemporal Gaussian process regression model can also be found in the appendix.

S1.3 Estimating development assistance for health: 1990–2020

To track DAH, we relied on revenue data in financial statements, annual reports, budget documents, and project disbursement records reported by international development agencies such as the Organisation for Economic Co-operation and Development (OECD) Creditor Reporting System, the World Bank, the Global Fund, and major philanthropic entities like the Bill & Melinda Gates Foundation, Susan Thompson Buffet Foundation, and Rotary International.[11-20] We used budget and commitment data to generate estimates for recent years for which disbursement data were not yet available; for most channels data extends to 2018 or 2019 so that at most two years of estimation were needed.

Our DAH estimates tracked disbursements from the originating donor (called the source); to the development agency responsible for disbursing the funds to the recipient country (called the channel); and to the recipient country. We used revenue and disbursement data to count only once the transfers between development agencies to limit double counting. In addition to reporting the source, channel, and recipient of DAH, we disaggregated spending into nine major health focus areas (as well as more detailed programme areas) by searching project titles and descriptions for keywords, as well as using other indicators in the data. Health focus areas included HIV/AIDS; malaria; tuberculosis; reproductive and maternal health; newborn and child health; non-communicable diseases; other infectious diseases; sector-wide approaches and health

system strengthening; and other. The “Other” category captured projects such as general support for a conference on the Sustainable Development Goals that were not allocated to any of the eight specific health focus areas; remaining funds for which no project descriptions were available were classified as “unallocable.” The list of keywords used to isolate relevant health projects for each of the health focus areas are included in the supplemental appendix Table S3.4.

While the majority of the methods used for tracking DAH have been described previously, the current estimates of DAH incorporated improvements in methodology such as leveraging additional project level descriptions from the Creditor Reporting System for the allocation of disbursements channeled through NGOs and ongoing refinement of our keyword search list.[21-25] For all DAH tracking, we include funds that were transferred through major development agencies included in our research, as well as private foundation and non-governmental agencies for whom we have data. This definition of DAH excludes spending on basic bench science which may have down-stream effects on the development of new drugs.

S1.4 Estimating GDP per capita for 2020 and 2021 that were COVID-19 sensitive

Overview: In light of the COVID-19 pandemic, we used a separate set of methods to estimate GDP per capita in 2020 and 2021. We first estimated the relationship between COVID-19 mortality, mobility, and mandate projections and key proximate economic indicators that were reported on a monthly basis, then forecasted the proximate indicator for each month through the end of 2021. We then aggregated the monthly values of these indicators to reflect annual growth rates, and used these annual changes to estimate change in GDP per capita for 2020 and 2021.

More precisely: While GDP per capita is largely available only at the quarterly or annual level, indicators such as consumer price index, electricity consumption, electricity generation, employed persons, exchange rate, exports, imports, interest rates, loans, retail sales, tax revenue, tourist arrivals were available at a monthly level. Using data from CEIC[30], we had monthly values in 2020 for at least one indicator in 103 countries, with values as recent as August 2020. We regressed each of these indicators on monthly aggregates of IHME’s estimates of COVID death rate, mobility, and number of mandates, as well as nested country-super region random effects; then used these fitted regression values in the ST-GPR framework described elsewhere in this appendix. Using IHMEs reference scenario projections, we were able to generate estimates for each of our economic indicators for all months through December of 2021. We then took mean values from 2019-2021 to generate growth rates in 2020 and 2021.

After generating annual growth rates for these indicators, we used linear mixed effects regressions and the relationship between GDP per capita growth rates from all non-tourism sectors and the tourism sector separately and the key covariates. Historical data fed into the regressions were downloaded from the World Bank World Development Indicators database, the International Monetary Fund World Economic Outlook database, the OECD Main Economic Indicators database[31], and the CEIC World Trend Plus database. We then used model outputs and the 2020 and 2021 growth rates generated in the previous step to generate GDP per capita growth rates for these years.

Tourist GDP per person growth rates were predicted using tourist arrivals growth rates and employed persons growth rates, while non-tourist GDP per person growth rates were predicted using country-level employed persons growth rates, electricity generation and interest rate growth rates by GBD super region, and consumer price index global growth rates. Each model included a country-specific random intercept. All GBD super region and global growth rates were weighted by country-level GDP. Predicted growth rates for each of the two sectors were applied to their retrospective GDP per person values, which were then aggregated to produce total GDP per person predictions for 2020 and 2021. Growth rates for GDP per person for 2020 and 2021 were then calculated using these values.

S 1.5 Reporting and uncertainty analysis

All inflation-adjusted health spending estimates are reported using 2020 prices. Future values are not discounted. We report health spending per capita in US dollars and purchasing-power parity-adjusted dollars and as a fraction of GDP. When not otherwise indicated, estimates are reported in 2020 US dollars. We report country spending estimates using 2019 Global Burden of Disease super-regions and 2019 World Bank income groups, regardless of whether a country changed, or is projected to change, income groups during the study period.[28,29] GBD regions can be seen in Figure S1.4 below. Rates were calculated to reflect each group, rather than the average of countries within the group, such that spending per capita estimates for an income group or region more heavily reflect rates in more populous countries. The uncertainty interval around each estimate was computed using the 2.5th and 97.5th percentiles of the 1,000 draws. All analyses were done using R (version 3.6.3), Stata (version 15.1), and Python (version 3.7.3). Table S1.4 presents all packages used for the analyses in this paper.

Table S1.4 Programming packages used for analyses

**Packages used within R (version 3.6.3)**					**Packages used within Python (version 3.7.3)**		**Packages used within Stata (version 15.1)**	
**AFModel**	**doParallel**	**influence.ME**	**RColorBrewer**	**tidyr**	json	xarray	egenmore	findname
**argparse**	**dplyr**	**iterators**	**readstata13**	**tidyverse**	csv	pandas	xfill	grc1leg
**arm**	**DT**	**lme4**	**readxl**	**TMB**				
**bit64**	**feather**	**lmtest**	**reticulate**	**tools**				
**boot**	**foreach**	**MASS**	**rjson**	**yaml**				
**Cairo**	**forecast**	**Matrix**	**RMySQL**	**zip**				
**car**	**foreign**	**micEcon**	**scales**	**zoo**				
**chron**	**frontier**	**openxlsx**	**shiny**					
**crayon**	**GauProMod**	**pacman**	**shinythemes**					
**cowplot**	**ggplot2**	**parallel**	**stringi**					
**data.table**	**gridExtra**	**plotly**	**stringr**					
	**haven**							

Figure S1.4 Global Burden of Disease super-regions and regions (source www.healthdata.org)

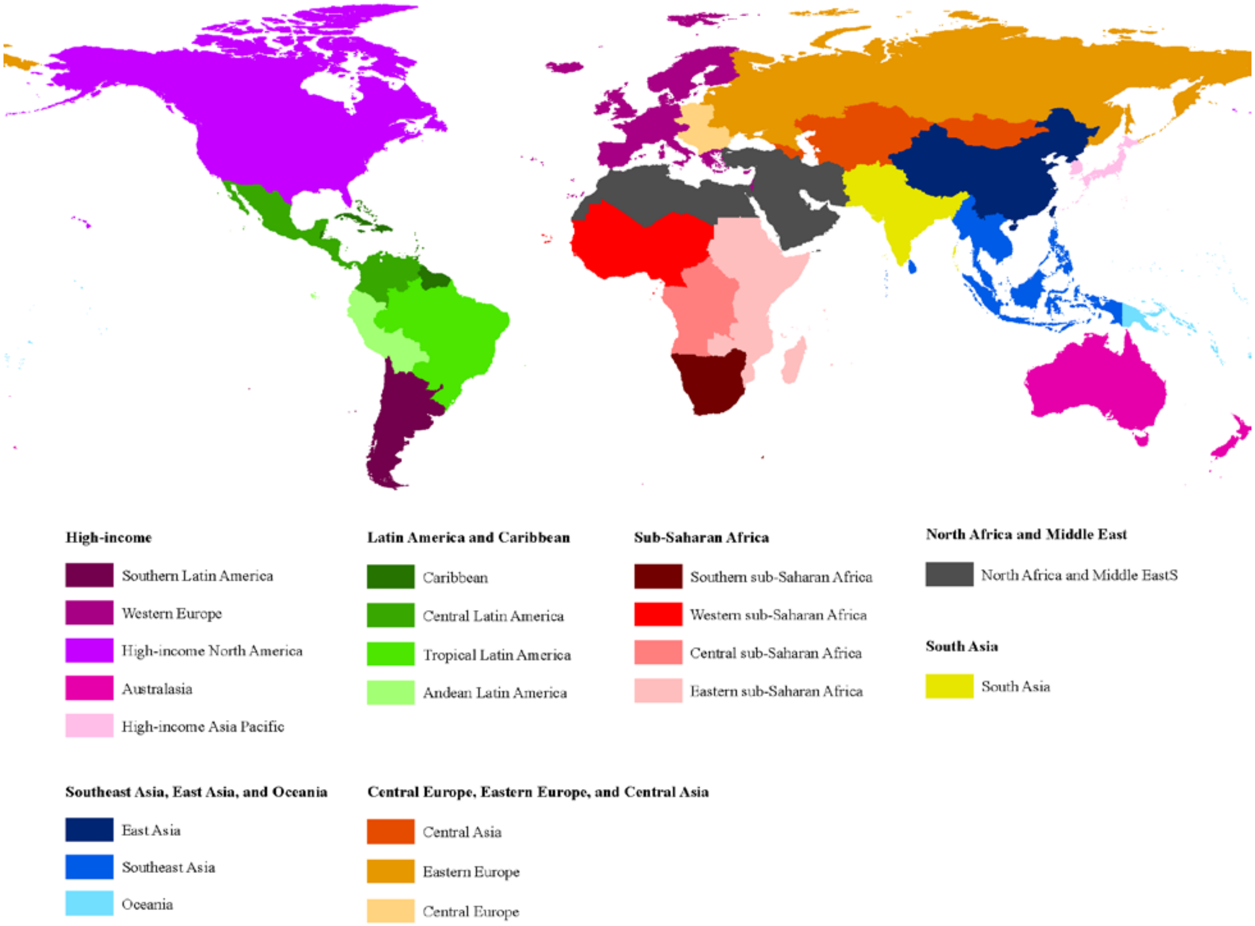

References

1WHO | Global Health Expenditure Database. WHO. http://www.who.int/health-accounts/ghed/en/ (accessed Dec 23, 2017).

2OECD, Eurostat and World Health Organization. A System of Health Accounts 2011: Revised edition. Paris: OECD Publishing, 2017 https://read.oecd-ilibrary.org/social-issues-migration-health/a-system-of-health-accounts-2011_9789264270985-en (accessed March 7, 2019).

3The Database | Penn World Table version 9.1. Groningen Growth and Development Centre: Faculty of Economics and Business. https://www.rug.nl/ggdc/productivity/pwt/ (accessed Apr 14, 2020).

4DataBank | The World Bank. The World Bank. http://databank.worldbank.org/data/home.aspx (accessed Dec 18, 2020).

5International Monetary Fund. World Economic Outlook, October 2020: A Long and Difficult Ascent. International Monetary Fund, 2020 https://www.imf.org/en/Publications/WEO/Issues/2020/09/30/world-economic-outlook-october-2020 (accessed Oct 13, 2020).

6Maddison Project Database 2020. Groningen Growth and Development Centre: Faculty of Economics and Business. https://www.rug.nl/ggdc/historicaldevelopment/maddison/releases/maddison-project-database-20 (accessed Dec 8, 2020).

7National Accounts Main Aggregates Database. United Nations Statistics Division - National Health Accounts. https://unstats.un.org/unsd/snaama/Introduction.asp (accessed Dec 17, 2020).

8James SL, Gubbins P, Murray CJ, Gakidou E. Developing a comprehensive time series of GDP per capita for 210 countries from 1950 to 2015. *Population Health Metrics* 2012; **10**: 12.

9Global Health Expenditure Database. World Health Organization. http://apps.who.int/nha/database (accessed Dec 21, 2018).

10GBD 2015 Risk Factors Collaborators. Global, regional, and national comparative risk assessment of 79 behavioural, environmental and occupational, and metabolic risks or clusters of risks, 1990–2015: a systematic analysis for the Global Burden of Disease Study 2015. *The Lancet* 2016; **388**: 1659–724.

11Find a Grant. https%3a%2f%2fwww.theglobalfund.org%2fen%2fportfolio%2ffind%2f (accessed Dec 22, 2018).

12OECD Statistics. https://stats.oecd.org/ (accessed Dec 22, 2018).

13Malaria Operational Plans (MOPs) | PMI. https://www.pmi.gov/resource-library/mops/ (accessed Dec 22, 2018).

14Key figures: donor contributions & pledges. https://www.gavi.org/investing/funding/donor-contributions-pledges/ (accessed Dec 22, 2018).

15Trustees reports and financial statements - International Finance Facility for Immunisation. https://www.iffim.org/finance/trustees-reports-and-financial-statements/ (accessed Dec 22, 2018).

16Financial reports. https://www.gavi.org/investing/funding/financial-reports/ (accessed Dec 22, 2018).

17Disbursements and commitments. https://www.gavi.org/results/disbursements/ (accessed Dec 22, 2018).

18 UNAIDS. PCB Archive. http://www.unaids.org/en/aboutunaids/unaidsprogrammecoordinatingboard/pcbmeetingarchive (accessed Nov 9, 2020).

19Executive Board. /executive-board (accessed Dec 22, 2018).

20 GB | Governing Body Documentation. http://apps.who.int/gb/ (accessed Dec 22, 2018).

21Ravishankar N, Gubbins P, Cooley RJ, *et al.* Financing of global health: tracking development assistance for health from 1990 to 2007. *The Lancet* 2009; **373**: 2113–24.

22Leach-Kemon K, Chou DP, Schneider MT, *et al.* The Global Financial Crisis Has Led To A Slowdown In Growth Of Funding To Improve Health In Many Developing Countries. *Health Affairs* 2012; **31**: 228–35.

23Dieleman JL, Graves C, Johnson E, *et al.* Sources and Focus of Health Development Assistance, 1990–2014. *JAMA* 2015; **313**: 2359–68.

24Dieleman JL, Graves CM, Templin T, *et al.* Global health development assistance remained steady in 2013 but did not align with recipients' disease burden. *Health Aff (Millwood)* 2014; **33**: 878–86.

25Dieleman JL, Templin T, Sadat N, *et al.* National spending on health by source for 184 countries between 2013 and 2040. *The Lancet* 2016; **387**: 2521–35.

26Micah AE, Zhao Y, Chen CS, *et al.* Tracking development assistance for health from China, 2000–2018. *BMJ Global Health* under review.

27Das Gupta P. Standardization and Decompostion of Rates: A User's Manual. U.S. Bureau of the Census, 1993 https://www.census.gov/content/dam/Census/library/publications/1993/demo/p23-186.pdf (accessed Dec 21, 2018).

28World Bank Country and Lending Groups – World Bank Data Help Desk. The World Bank. https://datahelpdesk.worldbank.org/knowledgebase/articles/906519-world-bank-country-and-lending-groups (accessed Dec 21, 2018).

29Abate KH, Abay SM, Abbafati C, *et al.* Global, regional, and national age-sex-specific mortality and life expectancy, 1950–2017: a systematic analysis for the Global Burden of Disease Study 2017. *The Lancet* 2018; **392**: 1684–735.

30CEIC World Trend Plus database https://insights.ceicdata.com/node/WORLD (accessed August 07, 2020).

31OECD Main Economic Indicators https://www.oecd.org/sdd/oecdmaineconomicindicatorsmei.htm (accessed August 07, 2020).

SECTION 2. TRACKING TOTAL HEALTH SPENDING AND ITS COMPONENTS

List of Tables and Figures

Table S2.1 Rules for assigning weight values to metadata

Table S2.2 Countries and income groups, number of assigned weights

Figure S2.1 Comparison of WHO GHED extracted and IHME ST-GPR THE in per capita space, 2000-2018

Figure S2.2 Comparison of WHO GHED extracted and IHME ST-GPR THE in per GDP space, 2000-2018

Figure S2.3 Currency conversion process diagram

S 2.1 Overview of GHED data cleaning process

We used Global Health Expenditure Database (GHED) data from the World Health Organization (WHO) to generate our estimates.[1] From the GHED, we extracted "Current health expenditure by revenues of health care financing schemes" for total health expenditure (THE), "Other revenues from households n.e.c" for out-of-pocket (OOP), "Gross Domestic Product" for GDP. We summed "Social insurance contributions" and "Transfers from government domestic revenue (allocated to health purposes)" for government health spending (GHE). We summed "Voluntary prepayment", "Other revenues from corporations n.e.c.", "Compulsory prepayment (Other, and unspecified, than FS.3)", and "Other revenues from NPISH n.e.c." for pre-paid private (PPP).

To ensure we used the best possible data from the GHED, we evaluated the metadata also provided by GHED to establish the reliability of the data. To do so, we downloaded the metadata from the GHED website for each data point for the five indicators. We used the metadata to decide how each given data point should be weighted, from 0 to 5, with 0 meaning drop, and 1 through 5 meaning keep and treated these weights as inverse variance weights.

To assign the weights, we established guidelines for the metadata that informed how the underlying data points should be weighted. We gave priority to factors such as complete, documented source information and penalized factors such as having been derived or estimated. Table S2.1 below describes the guidelines we created; any metadata that did not meet any of the disqualifying factors were given a value of 5 to reflect highest reliability.

In order to assign weights to the latest 2021 GHED estimates, we trained a linear SVM (Support Vector Machine) model supported by an NLP (Natural Language Processing) pipeline that learned from the weights that were manually assigned (based on the aforementioned guidelines) to metadata downloaded in the year 2017. The NLP pipeline consisted of a count vectorizer to transform the text metadata matrix into a matrix of token counts that can easily be interpreted by any machine learning algorithm. The count vectorizer also helped us bring in the possibility of simple words as well as combinations of simple words (i.e., word n-grams) to help with predicting the optimum reliability weight for each data point. The final model was tuned to achieve close resemblance for predicted weights when juxtaposed against the manually assigned weights for data points downloaded in the year 2017; and then used to predict weights for data points downloaded in 2021.

We used the four primary metadata variables from the GHED database: data type, method of estimation, comments, and sources. We applied the guidelines to each unique set of metadata across these four variables. For a subset of data points, the metadata indicated that the data point was the sum of other data points. In these cases, if the indicator was a sub-indicator of GHES, PPP, or OOP, we assigned the data

point a value of 2 to reflect that even though we could not determine if the sub-components existed, as they are not reported in GHED, we did not feel that being a sum warranted dropping the data. We assigned the summed GHES, PPP, and OOP indicators the lowest value of its sub-indicators. If the summed data point was THE, however, we assigned the data point the lowest value of its sub-components, the summed GHES, PPP, and OOP indicators.

After designating each of the 4,427 unique sets of metadata a value weight, we applied these weights to the underlying data points. In total, we had 32,509 data points, as multiple data points shared the same unique set of metadata. Once the weights were applied to the data, we reassigned all high-income country data points that were a 0 based on the metadata to 3. We made this change to reflect that high-income countries have higher quality data and thus should not be dropped, but should also not be given the highest weight value. The high-income classification comes from the World Bank.[2] For all countries across all years of data their relative weighting scores can be seen in Table S2.2.

Each component of health expenditure was aggregated for all component-year-country combinations by taking component values (downloaded in current national unit); dividing by GHED reported GDP for the corresponding year-country (also in current national unit) to convert each component into its fraction of GDP; and finally multiplying those fractions by IHME GDP series measured in USD 2020. (Note: though we harvested and cleaned metadata for THE and GDP, we do not model THE (we model GHE, PPP, OOP and then aggregate) or use the WHO's GDP data since we have our own methods with multiple sources for determination of our value).

Each component of health expenditure was then converted into three spending metrics for the time period between 2000-2018, measured in purchasing power parity (PPP): health spending per capita ($), annualized rate of change in health spending per capita 2000-2018 (%), and annualized rate of change in health spending 2000-2018 (%). Then, each component of health expenditure was aggregated and converted into six spending metrics for the time period between 2019-2050, measured in PPP: health spending per capita ($), annualized rate of change in health spending per capita 2019-2050 (%), annualized rate of change in health spending 2019-2050 (%), Government health spending per capita ($), difference between GHEpc (government health expenditure per capita) reference scenario and better scenario 1, 2050 ($), and difference between GHEpc reference scenario and better scenario 2, 2050 ($).

Formula for calculating AROC (Annualized rate of change):

If you apply this growth rate n times (where n is the number of steps from starting to ending year) to the value in the starting year, you will get the value in the ending year. For example –

$$\underset{1990 \rightarrow 2019}{\text{AROC}} = \left(\frac{value\ in\ 2019}{value\ in\ 1990}\right)^{\frac{1}{2019-1990}} - 1$$

Table S2.1 Rules for assigning weight values to metadata

Data type	Methods of estimation	Sources and/or Comments	Weight
**Blank**			0
**Estimated**			0
	Derived by applying the sum of the components		Lowest weight of the components
	Interpolated but with additional information		2
	Method description is unclear or provides very little information		0
	Time trend interpolation		1
	Uses data from other countries		0
		Abstract that's not from something documented	1 or method (whichever is bigger)
		Adjusted	0 or method (whichever is bigger)
		Adjusted using something	2 or method (whichever is bigger)
		Any suggestion that the WHO is unclear or unsure about some aspect of the data point's metadata	0 or method (whichever is bigger)
		Approximation	0 or method (whichever is bigger)
		Assumption	0 or method (whichever is bigger)
		Both blank	0 or method (whichever is bigger)
		Both with no intelligible information	0 or method (whichever is bigger)
		Budget address	1 or method (whichever is smaller)
		Calculation was used to generate the estimate	1 or method (whichever is bigger)
		Consultation/contact (without an additional documented source)	1 or method (whichever is smaller)
		Consultations with additional source, but no specifics and just consult is documented	1 or method (whichever is bigger)
		Currency conversion	1 or method (whichever is bigger)
		Data delivered/provided/reported by (a non-documented source)	1 or method (whichever is smaller)
		Data provided but not clear by whom, with an additional source if additional source is not documented	1 or method (whichever is smaller)
		Derived	0 or method (whichever is bigger)
		Estimated based on	1 or method (whichever is bigger)

Data type	Methods of estimation	Sources and/or Comments	Weight
		Estimation	0 or method (whichever is bigger)
		Excludes (if it excludes what we do want)	0 (supersedes method)
		Extrapolated	0 or method (whichever is bigger)
		Forecasted	0 or method (whichever is bigger)
		Government department, no explicit documented source	1 or method (whichever is bigger)
		Government ministry, but no explicit documented source	1 or method (whichever is bigger)
		Includes (if it includes what we don't want)	0 (supersedes method)
		Inferred	0 or method (whichever is bigger)
		Interpolation	0 or method (whichever is bigger)
		Missing (if missing something that should be included)	0 (supersedes method)
		Modified	0 or method (whichever is bigger)
		Modified from something/modified using something	2 or method (whichever is bigger)
		Needs assessment	1 or method (whichever is smaller)
		Needs discussion	1 or method (whichever is smaller)
		Needs validation	1 or method (whichever is smaller)
		Needs verification	1 or method (whichever is smaller)
		Only provides hint of a source	1 or method (whichever is smaller)
		Projected	0 or method (whichever is bigger)
		Provides only a vague term that does not provide adequate information to infer or determine what the source is	0 or method (whichever is bigger)
		Reply	1 or method (whichever is smaller)
		Response	1 or method (whichever is smaller)
		Speech	1 or method (whichever is smaller)
		Sum of	2 (except for THE, which is lowest weight of components)
		Total of	3 (except for THE, which is lowest weight of components)
		Underestimated	0 (supersedes method)
		Unpublished	1 or method (whichever is smaller)
		Validated figures, but without specifics	2 or method (whichever is bigger)
		Weights	0 or method (whichever is bigger)

Table S2.2 Countries and income groups, number of assigned weights

Country	0	1	2	3	4	5
Afghanistan	88	63	0	0	0	20
Albania	114	0	0	0	0	57
Algeria	128	16	6	0	0	21
Andorra	0	0	0	114	0	0
Angola	152	0	0	0	0	19
Antigua and Barbuda	50	0	3	108	0	10
Argentina	77	0	58	19	0	17
Armenia	73	16	36	1	0	45
Australia	0	0	0	171	0	0
Austria	0	0	0	171	0	0
Azerbaijan	76	18	19	0	0	20
Bahamas	0	0	0	171	0	0
Bahrain	6	1	0	162	0	2
Bangladesh	39	2	14	0	0	116
Barbados	28	0	4	135	0	4
Belarus	94	7	1	0	4	65
Belgium	0	0	0	171	0	0
Belize	95	17	38	0	0	21
Benin	123	0	0	0	3	45
Bhutan	110	0	0	0	0	61
Bolivia Plurinational States of	141	0	1	0	0	29
Bosnia and Herzegovina	120	0	0	0	0	51
Botswana	116	0	0	0	0	55
Brazil	134	0	18	0	0	19
Brunei Darussalam	0	0	0	171	0	0
Bulgaria	100	10	0	0	9	52
Burkina Faso	96	0	0	0	0	75
Burundi	110	3	0	0	0	58
Cabo Verde Republic of	84	19	4	0	0	64
Cambodia	96	3	1	0	0	71
Cameroon	144	0	0	0	0	27
Canada	0	0	0	171	0	0
Central African Republic	92	34	0	0	1	44
Chad	127	0	6	0	0	38
Chile	3	0	6	63	0	99
China	68	84	0	0	0	19
Colombia	28	41	32	0	1	69
Comoros	151	0	0	0	0	20
Congo	94	15	0	0	0	62
Cook Islands	151	0	1	0	0	19

**Costa Rica**	107	0	11	0	19	34
**Cote d'Ivoire**	78	19	0	0	0	74
**Croatia**	64	0	0	90	0	17
**Cuba**	95	0	19	0	0	57
**Cyprus**	0	0	0	171	0	0
**Czech Republic**	45	3	0	117	0	6
**Democratic Republic of the Congo**	103	0	0	0	0	68
**Denmark**	0	0	0	171	0	0
**Djibouti**	127	0	0	0	0	44
**Dominica**	120	0	23	0	0	28
**Dominican Republic**	132	0	0	0	0	39
**Ecuador**	72	32	42	0	0	25
**Egypt**	46	80	2	0	3	40
**El Salvador**	113	7	19	0	0	32
**Equatorial Guinea**	66	11	0	64	0	11
**Eritrea**	113	1	0	0	0	57
**Estonia**	9	5	9	117	3	28
**Eswatini**	140	5	0	0	0	26
**Ethiopia**	127	8	0	0	0	36
**Fiji**	64	0	2	0	0	105
**Finland**	0	0	0	171	0	0
**France**	0	0	0	171	0	0
**Gabon**	97	0	0	0	0	74
**Gambia**	109	18	0	0	0	44
**Georgia**	54	10	33	0	0	74
**Germany**	0	0	0	171	0	0
**Ghana**	124	0	11	0	0	36
**Greece**	0	0	0	171	0	0
**Grenada**	111	4	18	0	0	38
**Guatemala**	147	0	0	0	0	24
**Guinea**	109	7	0	0	0	55
**Guinea-Bissau**	140	1	0	0	0	30
**Guyana**	102	0	29	0	1	39
**Haiti**	139	0	12	0	0	20
**Honduras**	74	0	32	0	2	63
**Hungary**	15	0	0	90	0	66
**Iceland**	0	0	0	171	0	0
**India**	118	1	0	0	0	52
**Indonesia**	81	20	0	0	10	60
**Iran (Islamic Republic of)**	112	35	0	0	0	24
**Iraq**	100	46	1	0	2	22
**Ireland**	0	0	0	171	0	0

**Israel**	0	0	0	171	0	0
**Italy**	0	0	0	152	0	0
**Jamaica**	94	5	26	0	0	46
**Japan**	0	0	0	171	0	0
**Jordan**	67	72	3	1	3	25
**Kazakhstan**	75	20	0	0	0	76
**Kenya**	120	0	0	0	0	51
**Kiribati**	145	0	1	0	0	25
**Kuwait**	0	0	0	171	0	0
**Kyrgyzstan**	77	28	0	0	0	47
**Lao People's Democratic Republic**	105	2	1	0	0	63
**Latvia**	88	0	0	72	0	11
**Lebanon**	145	7	0	0	0	19
**Lesotho**	113	0	0	0	0	20
**Liberia**	120	19	1	0	0	31
**Libya**	139	11	1	0	1	19
**Lithuania**	54	0	0	63	0	54
**Luxembourg**	0	0	0	171	0	0
**Madagascar**	150	2	0	0	0	19
**Malawi**	48	15	0	0	0	108
**Malaysia**	98	54	0	0	0	19
**Maldives**	91	21	15	0	0	44
**Mali**	130	3	0	0	0	38
**Malta**	7	1	0	162	0	1
**Marshall Islands**	95	0	37	0	0	39
**Mauritania**	61	79	0	0	0	31
**Mauritius**	118	23	0	0	0	30
**Mexico**	24	0	0	0	0	147
**Micronesia (Federated States of)**	106	0	31	0	0	34
**Monaco**	0	0	0	114	0	0
**Mongolia**	117	5	30	0	0	19
**Montenegro**	136	0	0	5	0	30
**Morocco**	141	8	0	0	0	22
**Mozambique**	132	0	0	0	0	39
**Myanmar**	103	33	0	1	0	34
**Namibia**	99	4	0	0	0	68
**Nauru**	146	1	0	9	0	15
**Nepal**	26	51	24	0	3	67
**Netherlands**	0	0	0	171	0	0
**New Zealand**	0	0	0	171	0	0
**Nicaragua**	98	34	20	0	0	19
**Niger**	103	2	0	0	0	66

**Nigeria**	85	0	0	0	0	86
**Niue**	136	0	1	0	1	33
**Norway**	0	0	0	171	0	0
**Oman**	56	0	0	108	0	7
**Pakistan**	107	8	5	0	6	45
**Palau**	96	0	16	27	0	32
**Panama**	80	16	34	18	0	23
**Papua New Guinea**	121	3	24	0	0	23
**Paraguay**	57	21	23	0	0	70
**Peru**	124	13	4	0	0	30
**Philippines**	144	5	3	0	0	19
**Poland**	72	0	0	90	0	9
**Portugal**	0	0	0	171	0	0
**Qatar**	0	0	0	171	0	0
**Republic of Korea**	1	0	0	162	0	8
**Republic of Moldova**	104	18	0	0	1	48
**Romania**	92	28	0	0	0	51
**Russian Federation**	47	60	0	27	0	37
**Rwanda**	114	3	0	0	0	54
**Saint Kitts and Nevis**	75	0	11	72	0	13
**Saint Lucia**	114	19	19	0	0	19
**Saint Vincent and the Grenadines**	141	0	7	0	0	23
**Samoa**	112	0	18	0	0	41
**San Marino**	0	0	0	114	0	0
**Sao Tome and Principe**	74	5	0	0	0	92
**Saudi Arabia**	32	0	0	135	0	4
**Senegal**	125	22	0	0	0	24
**Serbia**	94	10	0	0	0	67
**Seychelles**	93	15	0	45	0	18
**Sierra Leone**	104	35	0	0	1	31
**Singapore**	0	0	0	171	0	0
**Slovakia**	56	0	0	108	0	7
**Slovenia**	0	0	0	171	0	0
**Solomon Islands**	152	0	0	0	0	19
**South Africa**	88	0	16	0	0	67
**South Sudan**	150	6	0	0	0	15
**Spain**	0	0	0	171	0	0
**Sri Lanka**	16	46	29	0	0	80
**Sudan**	45	82	1	0	14	29
**Suriname**	132	0	1	0	0	38
**Sweden**	0	0	0	171	0	0
**Switzerland**	0	0	0	171	0	0

**Syrian Arab Republic**	135	3	2	0	10	21
**Tajikistan**	58	1	0	0	0	112
**Thailand**	33	0	0	0	0	138
**The Republic of North Macedonia**	39	65	0	0	11	37
**Timor-Leste**	132	0	10	0	0	29
**Togo**	106	19	2	0	0	44
**Tonga**	108	1	18	0	0	44
**Trinidad and Tobago**	42	0	6	117	0	6
**Tunisia**	124	0	0	0	2	45
**Turkey**	93	27	0	0	0	51
**Turkmenistan**	152	0	0	0	0	19
**Tuvalu**	144	0	0	0	0	27
**Uganda**	82	13	2	0	0	74
**Ukraine**	116	5	18	0	2	30
**United Arab Emirates**	0	0	0	171	0	0
**United Kingdom**	0	0	0	171	0	0
**United Republic of Tanzania**	90	19	0	0	0	62
**United States of America**	0	0	0	171	0	0
**Uruguay**	60	0	21	63	0	27
**Uzbekistan**	130	0	0	0	0	41
**Vanuatu**	152	0	0	0	0	19
**Venezuela (Bolivarian Republic of)**	98	3	32	9	11	18
**Viet Nam**	17	0	1	0	0	153
**Yemen**	104	7	0	0	38	22
**Zambia**	114	38	0	0	0	19
**Zimbabwe**	130	9	0	0	0	32
**High income**	0	0	0	8046	0	0
**Low income**	4618	579	101	0	41	1946
**Lower-middle income**	5152	695	455	2	69	2311
**Upper-middle income**	4583	509	467	7	51	2103
**GBD High-income**	148	1	85	5409	0	0

S2.2 Statistical model to fill missingness in health expenditure variables

After the cleaning up of the data previously described above, we used Spatiotemporal Gaussian process regression (ST-GPR) in order to predict and fill out the missingness that existed in the resulting health expenditure dataset. ST-GPR is a stochastic modeling technique designed to detect signals amidst noisy data. It also serves as a powerful tool for interpolating non-linear trends. Unlike classical linear models that assume that the trend underlying data follows a definitive functional form, GPR assumes that the specific trend of interest follows a Gaussian process, where each point can be estimated with a mean and covariance function.

The first step to implementing ST-GPR is to identify relevant covariates that would be helpful in predicting each health expenditure variable of interest. Using the following set of covariates, we estimated the first stage of the process (space-time) in order to predict and fill up the dependent variables. The covariates used are:

a) All-sector government expenditure per capita, logged
b) Lag Distributed Income, logged
c) Healthcare Access and Quality Index, logged
d) Proportion of total population above the age of 65, logit transformed.

The dependent variables were logs of GHE per capita, PPP per capita and OOP per capita. We also run these models sequentially so that the following model can include the previous component as a covariate. GHE per capita uses only the above covariates, while PPP per capita additionally included GHE per capita as a covariate, and OOP per capita additionally uses GHE per capita and OOP per capita as covariates.

Given the weight of data, we were able to adjust the weight of each data point that contributed to the likelihood function of the Gaussian process, by inflating the pointwise variance for data points with lower weights. For missing data points, the resulting uncertainty was determined by region specific estimates. The final resulting dataset was a complete set of GHE, PPP and OOP per capita estimates for 204 countries from 1995 through 2018, where the uncertainty around each point was constructed by simulating from a normal distribution. Goodness-of-fit was determined using out-of-sample validation and choosing the models with the best performing set of hyperparameters. Overall, ST-GPR is a stochastic modeling technique that performs well at detecting signals among datasets that are noisy. It deviates from classic linear models that assume trend of its underlying data must follow a definitive functional form. Instead, GPR assumes the trend must follow a Gaussian Process form, having a covariance function and a pointwise mean. For the distribution of the Gaussian process, we used a Matérn covariance matrix. GPRs were run repeatedly to ensure that every country-year estimate per metric had 1,000 draws. This ST-GPR process follows the same form of that found in the Global Burden of Disease Study 2017 appendix.[3] This analysis was conducted in the following programs: Stata (version 13.1) and R (version 3.6.0).

Figure S2.1 below shows the comparison of WHO GHED extracted data and IHME ST-GPR THE in GDP per capita space from 2000-2018. Figure S2.2 shows the comparison of WHO GHED extracted data and IHME ST-GPR THE in per GDP space from 2000-2018.

Figure S2.1 Comparison of WHO GHED extracted and IHME ST-GPR THE *in per capita* space, 2000-2018

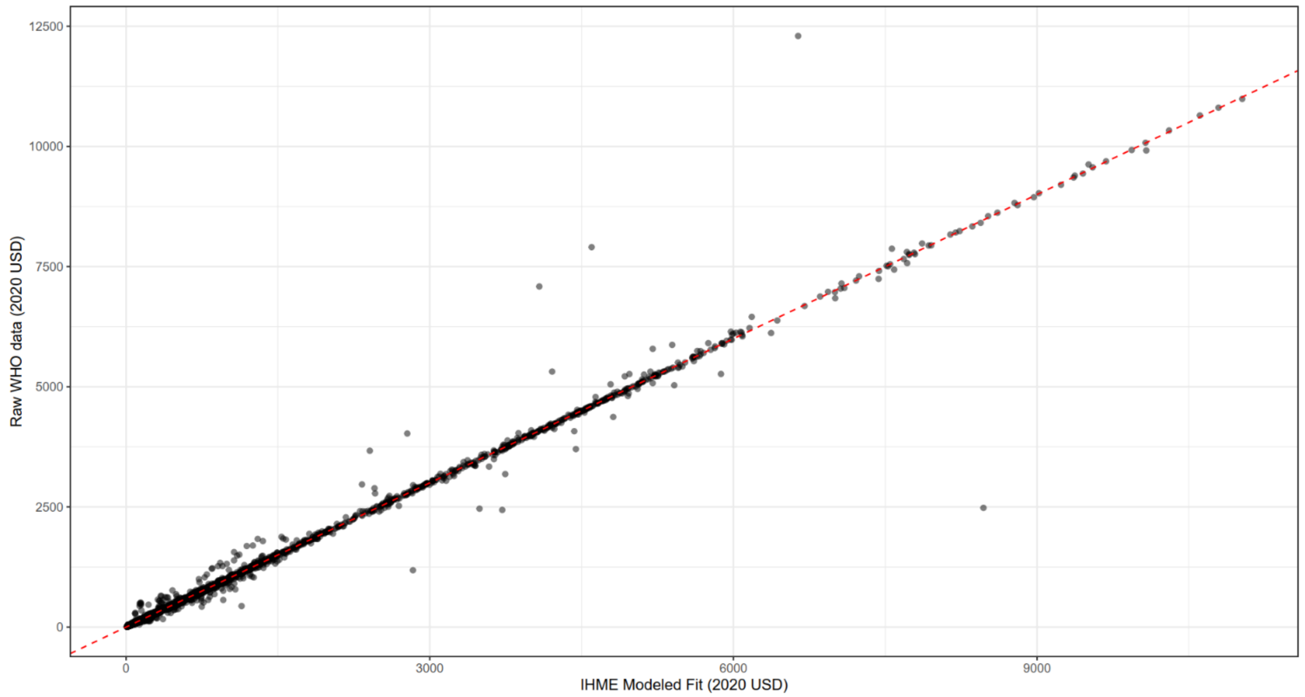

Figure S2.2 Comparison of WHO GHED extracted and IHME ST-GPR THE *in per GDP* space, 2000-2018

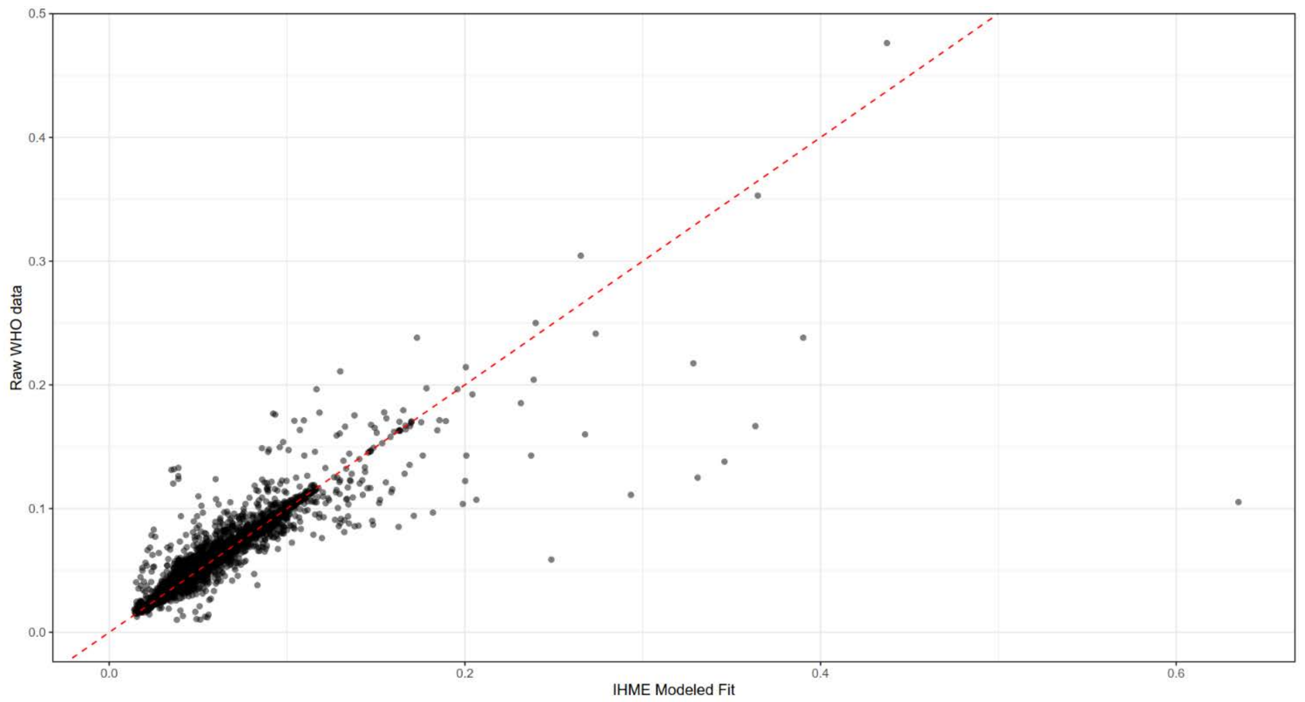

S2.3 Currency exchange and deflation

To convert a metric from real 2020 USD to real 2020 PPP, the following steps were taken. Real USD was exchanged to real LCU (local currency units) by multiplying with country-year specific USD to LCU exchange rates. Then, values were converted from real LCU to real PPP (purchasing-power parity-adjusted dollars) using the country-year specific LCU to PPP conversion series. Any currency deflation from nominal to real currency units or between real currency units in different base years were performed after converting either USD or PPP to LCU, and used country-specific deflator series. All deflators and exchange rates were extracted from the World Bank[4], International Monetary Fund[5], Penn World Tables[6], the United Nations National Accounts[7], and the World Health Organization[1], and were imputed to provide a complete series for each of the variables between 1950 and 2020. We then used several models including ordinary least-squares regression and mixed effects models, to complete each source series from 1950 to 2020.

Figure S2.3 shows the currency conversion process used in this work.

Figure S2.3 Currency conversion process diagram

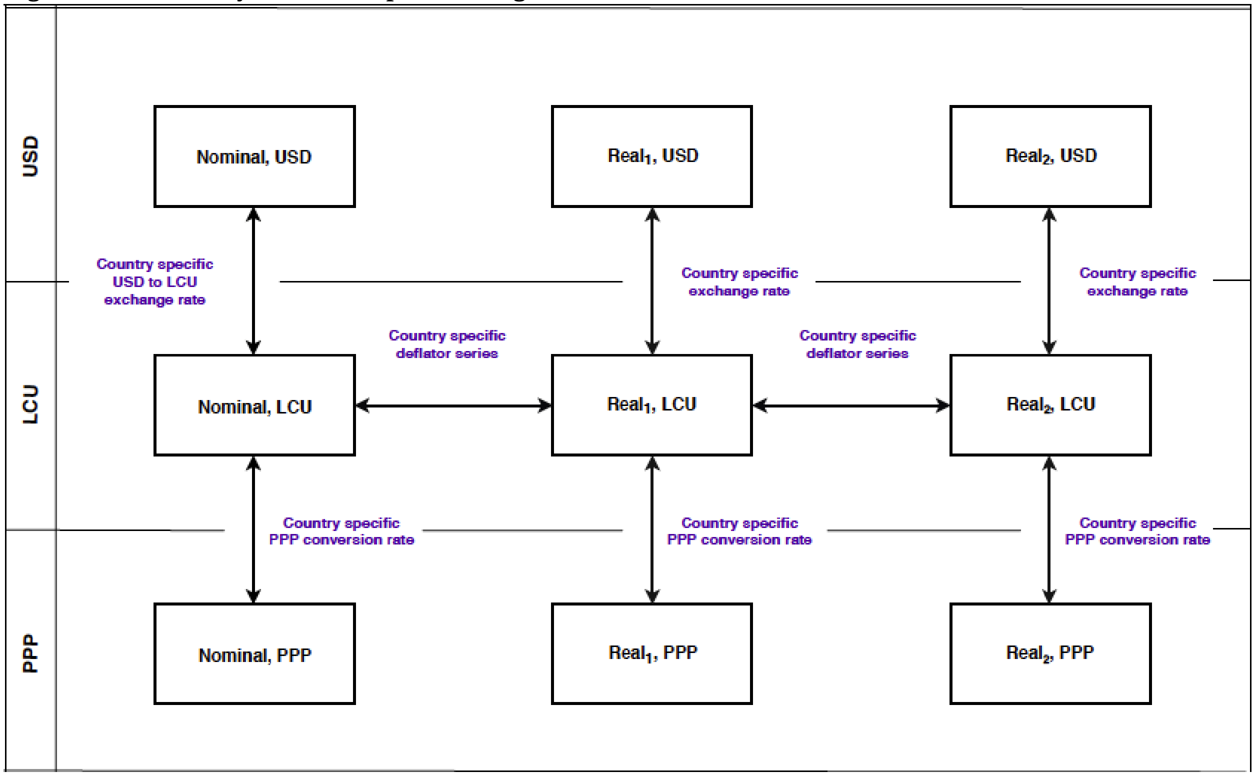

References

1 Global Health Expenditure Database. World Health Organization. http://apps.who.int/nha/database (accessed Jan 2, 2021).

2 World Bank Country and Lending Groups – World Bank Data Help Desk. World Bank. https://datahelpdesk.worldbank.org/knowledgebase/articles/906519-world-bank-country-and-lending-groups (accessed September 28, 2020).

3 Stanaway JD, Afshin A, Gakidou E, *et al.* Global, regional, and national comparative risk assessment of 84 behavioural, environmental and occupational, and metabolic risks or clusters of risks for 195 countries and territories, 1990–2017: a systematic analysis for the Global Burden of Disease Study 2017. *The Lancet* 2018; **392**: 1923–94.

4 World Development Indicators (WDI) | Data Catalog. https://datacatalog.worldbank.org/dataset/world-development-indicators (accessed Jan 4, 2021).

5 World Economic Outlook Database October 2020. https://www.imf.org/en/Publications/WEO/weo-database/2020/October (accessed October 13, 2020).

6 Feenstra, Robert C., Robert Inklaar and Marcel P. Timmer (2015), "The Next Generation of the Penn World Table" American Economic Review, 105(10), 3150-3182, available for download at www.ggdc.net/pwt DOI:10.15141/S5Q94M.

7 National Accounts - Analysis of Main Aggregates (AMA). https://unstats.un.org/unsd/snaama/Basic (accessed Jan 04, 2021).

SECTION 3. TRACKING DEVELOPMENT ASSISTANCE FOR HEALTH FROM TRADITIONAL DONORS

List of Figures

Figure S3.1 Comparing CRS commitments, CRS disbursements, and DAC commitments

Figure S3.2 CRS disbursement to commitment ratio and cutoff points by donor agency

Figure S3.3 One- to six-year disbursement schedules for bilateral channels

Figure S3.4 DAH as a percentage of corresponding budget data by bilateral agency

Figure S3.5 Malaria DAH to program areas as assigned by keyword search and PMI reports

Figure S3.6 Comparison of world Bank IDA Replenishment and DAC

Figure S3.7 Disbursements by the African Development Bank

Figure S3.8 Disbursements by Asian Development Bank

Figure S3.9 Disbursements by Inter-American Development Bank

Figure S3.10 Contributions received by the Global Fund to Fight AIDS, Tuberculosis and Malaria

Figure S3.11 The Global Fund to Fight AIDS, Tuberculosis and Malaria's commitments and disbursements

Figure S3.12 Gavi's income and disbursements

Figure S3.13 Total revenue received by non-governmental organizations

Figure S3.14 Expenditure by non-governmental organizations

Figure S3.15 In-kind contributions by loan- and grant-making DAH channels of assistance

List of Tables

Table S3.1. Summary of primary data sources and databases

Table S3.2. Data sources received via personal correspondence

Table S3.3. Additional data sources, databases and model choices used for preliminary estimates of DAH

Table S3.4. Terms for keyword searches

Table S3.5. Additional health focus area categorizations

Table S3.6. World Bank's health sector and theme codes

Table S3.7 Summary of data sources for the regional development banks

Table S3.8 Summary of US non-governmental organizations in the study

Table S3.9 Summary of data sources for calculating in-kind contributions

Box 3.1. EXAMPLE - Australia's primary and additional data sources

Box 3.2. EXAMPLE. Post-keyword search weighting

Box 3.3. EXAMPLE. Australia's CRS disbursement to commitment ratio and cutoff year

Box 3.4. EXAMPLE. Australia's one- to six-year disbursement schedules

Box 3.5. EXAMPLE. Australia's DAH as a percentage of corresponding budget data

S3.1 Overview

We summarize the original methodology as well as updates for this year's estimates below. A more detailed description of the original methodology used to obtain the estimates can be found in Dieleman et al.[1] All known, systematically reported, available data on health-related disbursements and expenditures were extracted, as well as income and revenue from existing project databases, annual reports, and audited financial statements. The channels included and the corresponding data sources are summarized in Table S3.1. Data sources obtained via personal correspondence are summarized in Table S3.2.

DAH for bilateral agencies included all health-related disbursements from bilateral donor agencies, excluding funds that they transferred to any of the other channels we tracked in order to avoid double-counting. This information was extracted from the Creditor Reporting System (CRS) and Development Assistance Committee (DAC) databases of the Development Assistance Committee of the Organisation for Economic Co-operation and Development (OECD-DAC). In some cases, donor agencies did not report disbursement data to the CRS. A method for predicting disbursements from commitment data was implemented to address this challenge. For detailed description of this method see Tracking Development Assistance for Health from Bilateral Aid Agencies and the European Commission section below as well as in Dieleman et al. [1]

For other grant- and loan-making institutions, annual disbursements on health grants and loans were similarly included, excluding transfers to any other channels and ignoring any repayments on outstanding debts. For a more detailed description of this process see Dieleman et al.[1] The annual disbursements for grant- and loan-making institutions only reflect the financial transfers made by these agencies. Therefore, in-kind transfers from these institutions in the form of staff time for providing technical assistance and the costs of managing programs were estimated separately.[1]

Estimates of DAH for the United Nations (UN) agencies included annual expenditures on health both from their core budgets and from voluntary contributions. Calculating DAH for the United Nations Children's Fund (UNICEF) involved estimating the fraction of its total expenditure spent on health prior to 2001.[1]

Non-governmental organizations' (NGOs) DAH estimates utilized data from US government sources, GuideStar Research Fundamental Plus dataset and a survey of health expenditure for a sample of NGOs to estimate DAH from US-based and internationally based NGOs receiving support from the US government. We were unable to include other NGOs due to the lack of audited and comparable data.

The database also included an analysis of the composition of health funding by recipient country, health focus area and program area. In this round of updates to the database we incorporated improvements in methodology such as leveraging additional project level descriptions from the Creditor Reporting System for the allocation of disbursements channeled through NGOs and ongoing refinement of our keyword search list. All methodological updates made are detailed in their relevant channel sub-section below. The improvements to our health focus area keyword search terms are detailed in Table S3.4.

For many channels, reporting-time lags prevent primary disbursement data for the most recent year(s). For those years, the values of DAH were predicted, using channel-specific time trends. The methods employed to obtain these predictions are summarized in Table S3.3. In general, these methods depend on data availability. The estimates are based on channel-specific budget, commitment, and appropriations data, and in many cases assume the most recent disbursement patterns persist. Due to the lack of more detailed disaggregated data, estimates for the most recent year are not provided for recipient countries.

We predicted ebola funding in 2019-2020 for bilateral sources and the European Commission by assuming that 2019 and 2020 ebola funding was equal to 2018 ebola funding.

In addition, for SWAp/HSS funding, we allocated SWAP/HSS projects with multiple health focus areas identified by a proportional allocation based on the relative proportions of the project going to the various health focus areas.

For projects that list a recipient as a region, we split DAH among the low-middle income countries that are eligible to receive DAH within that region based on known proportion of DAH. We do not split projects targeted at global initiatives and label the recipient as "global". For example, a project for vaccine development targeted at no specified recipient country would be classified as global.

For countries that only began existing in certain years, we backcasted DAH in years before their existence as follows. For countries that split off from parent countries, we calculated a three-year average ratio of child country DAH received to parent country DAH received. In years before the child country split off, DAH received by the parent country would have included DAH received in the region that would split off to become the child country. Therefore, we reallocated funding from the parent country, in all years before the child country split off, adding this proportion of the parent country's DAH to the child country's DAH and subtracting out this value from the parent country's DAH. By this method, total annual DAH between the parent and child country do not change, but the allocation of funding between the parent country and child country change. For any country that ceased to exist (such as former Yugoslavia and former USSR) and that had observed DAH received in certain years, we split the funding equally among its new constituent countries.

S3.1.1 Currency exchange and deflation

All results are presented in real 2020 US dollars. All disbursement sequences are converted into real 2020 US dollars by taking disbursements in nominal US dollars in the year of disbursement and adjusting these sequences into real 2020 US dollars using US gross domestic product (GDP) deflators[99]. All disbursement sequences in nominal currencies other than US dollars are converted into real 2020 US dollars by adjustment using country-year specific rates from the OECD's exchange rate series from OECD.Stat. If the current year is not yet available, the previous year's deflator value is utilized. OECD does not include an exchange series for the United Arab Emirates. IHME deflator estimates for the United Arab Emirates are leveraged for deflation. Analyses are conducted in Stata (versions 13.1 or 15.1) or R (version 3.6.3).

Table S3.1. Summary of primary data sources and databases

Channel	Source
**Bilateral agencies**	OECD-DAC and CRS databases[2]
**European Commission**	OECD-DAC and CRS databases[3]
**Joint United Nations Programme on HIV/AIDS (UNAIDS)**	Financial reports, audited financial statements, and budget[4]
**United Nations Children's Fund (UNICEF)**	Financial reports and audited financial statements[5–7]
**United Nations Population Fund (UNFPA)**	Financial reports and audited financial statements[8]
**UNITAID**	Project level data from IATI database shared through correspondence, financial reports and audited financial statements[9]
**Pan American Health Organization (PAHO)**	Financial reports and audited financial statements[10]
**World Health Organization (WHO)**	Financial reports and audited financial statements[11]
**World Bank**	Online project database and correspondence[12,13]
**African Development Bank (AfDB)**	Correspondence, replenishment reports, and compendium of statistics[14,15]
**Asian Development Bank (ADB)**	Online project database and correspondence[81,98]

**Inter-American Development Bank (IDB)**	Online project database and correspondence[16,17]
**Gavi, the Vaccine Alliance**	Online project database, cash received database, International Finance Facility for Immunisation (IFFIm) annual reports, Advance Market Commitment for Pneumococcal Vaccines (AMC) annual reports, and annual reports[18–21]
**The Global Fund to Fight AIDS, Tuberculosis and Malaria (The Global Fund)**	Online grant database, contributions report and annual reports[22–24]
**Coalition for Epidemic Preparedness and Innovation (CEPI)**	Project level data through correspondence
**NGOs registered in the US**	United States Agency for International Development (USAID) Report of Voluntary Agencies (VolAg), tax filings, annual reports, financial statements, RED BOOK Expanded Database, and WHO's Model List of Essential Medicines, Guidestar Research Fundamentals Plus dataset[25–29]
**Bill & Melinda Gates Foundation (BMGF)**	Online grant database, IRS 990 tax forms, and correspondence[30,31]
**Other private US foundations**	Foundation Center's grants database[32]
**Wellcome Trust**	Project level data through correspondence
**European Economic Area**	Online project database[33] and correspondence[34]
**China**	Financial reports and yearbooks

Table S3.2. Data sources received via personal correspondence

Channel	Data received
**World Bank**	Health project-level disbursement data, 1990 – 2020[13]
**BMGF**	Health disbursement data, 2019[31]
**CEPI**	Health disbursement data, 2020
**IDB**	Health project-level loan disbursement data, 2020[17]
**AfDB**	Health project-level disbursement data, 1990 – December 2020[35]
**ADB**	Health project-level disbursement data, 1990 – 9 December 2020[98]
**UNITAID**	Health project-level disbursement data, 2007 – 2020[36]
**UAE bilateral**	UAE Foreign Assistance in Health 1990 - 2008[37]
**European Economic Area**	Health project-level disbursement data, 2007-2020

Table S3.3. Additional data sources, databases and model choices used for preliminary estimates of DAH

Channel	Data source	Variables used	Years of budget data used for modeling*	Years underlying DAH data not available; thus modeled*	Model used
**National agencies**					

**Australia**	Australia's International Development Assistance (2008-2020); Australia's Overseas Aid Program (1998-2008)[38,39]	Health official development assistance (ODA): International development assistance budget	1998-2019	2020	Weighted average of actual DAH/budgeted DAH
**Austria**	Austria Federal Ministry of Finance budget[40]	General ODA: Federal ODA budget	2007-2019	2020	Weighted average of DAH/budgeted ODA
**Belgium**	Project Budget General – general expenses[41]	General ODA: Foreign affairs, foreign trade development and cooperation	2000-2019	2020	Weighted average of DAH/budgeted ODA
**Canada**	Canadian International Development Agency – Report on Plans and Priorities[42]	General ODA: Financial summary – planned spending	1996-2019	2020	Weighted average of DAH/budgeted ODA
**Denmark**	Danish Ministry of Foreign Affairs Budget[43]	General ODA: Budgeted expenditures on overseas development assistance	2000-2019	2020	Weighted average of DAH/budgeted ODA
**European Commission**	General budget[44]	Data not used as they were inconsistent with disbursements	–	2020	Based on weighted average of trends in member countries
**Finland**	Document Assembly in budget years 1998-2020[45]	General ODA: Ministry of Foreign Affairs' administrative appropriations, international development	2002-2019	2020	Weighted average of DAH/budgeted ODA
**France**	Budget and Financial documents[46,47]	General ODA: aggregated project data; Total ODA	2009-2019	2020	Weighted average of DAH/budgeted ODA
**Germany**	Plan of the Federal Budget[48]	General ODA: Development expenditure	2001-2019	2020	Weighted average of DAH/budgeted ODA

**Greece**	Ministry of Finance Budget (2013-2018); OECD Data (1996-2020)[2,49,50]	General ODA; ODA commitments	1996-2019	2020	Weighted average of DAH/budgeted ODA
**Ireland**	Department of Finance – budget 2000-2004; Estimates for Public Services and Summary Public Capital Programme, 2005-2020[51]	General ODA: Summary of adjustments to gross current estimates – international co-operation	2002-2019	2020	Weighted average of DAH/budgeted ODA
**Italy**	The Italian Agency for Development Cooperation[52]	General ODA: Net development corporation	2007-2019	2020	Weighted average of DAH/budgeted ODA
**Japan**	Highlights of the Budget for FY1999-2020[53]	General ODA: Major budget expenditures	2003-2019	2020	Weighted average of DAH/budgeted ODA
**Korea, South**	ODA Korea comprehensive implementation plan[54]	General ODA: Plan for international development cooperation	2008-2019	2020	Weighted average of DAH/budgeted ODA
**Luxembourg**	State Budget[55]	General ODA: Ministry of Foreign Affairs – budgeted international development cooperation and humanitarian aid	2001-2019	2020	Weighted average of DAH/budgeted ODA
**Netherlands**	Netherlands International Cooperation Budget (2001-2020)	General ODA: Total annual official development assistance expenditure	2001-2019	2020	Weighted average of DAH/budgeted ODA
**New Zealand**	Vote Foreign Affairs and Trade (1998-2001); VOTE Official Development Assistance (2002-2020)[56]	General ODA: Total annual official development assistance expenditure	1998-2019	2020	Weighted average of DAH/budgeted ODA
**Norway**	Norwegian Ministry of Finance National Budget (2014-2020); Correspondence (2000-2013)[57,58]	General ODA: ODA budget	2000-2019	2020	Weighted average of DAH/budgeted ODA

**Portugal**	Ministry of Finance and Public Administration State Budget 2003-2020[59]	General ODA: Integrated service expenditure – external cooperation budget	2003-2019	2020	Weighted average of DAH/budgeted ODA
**Spain**	Annual Plans of Spanish International Cooperation[60]	General ODA: Spanish total development cooperation	2003-2019	2020	Weighted average of DAH/budgeted ODA
**Sweden**	Correspondence (2000-2010); Ministry of Foreign Affairs Budget (2010-2020)[61]	General ODA: Ministry for Foreign Affairs budgets for expenditure – international development cooperation	2000-2019	2020	Weighted average of DAH/budgeted ODA
**Switzerland**	Foreign Affairs (2000-2006); Budget – Further Explanations and Statistics (2007-2020)	General ODA: Direction of development and cooperation (2000-2006); foreign affairs – international cooperation, development aid (in the South and East) (2007-2016)	2000-2019	2020	Weighted average of DAH/budgeted ODA
**United Kingdom**	IATA (Department for International Development (DFID)) [62,63]	General ODA: assistance for international development; Sum (revised) - aggregated project data	1998-2019	2020	Weighted average of DAH/budgeted ODA
**United States**	Foreign Assistance Dashboard (2006-2020); Budget of the US Government (2005-2020)[64,65]	Global health ODA: Planned foreign assistance for health; Department of Health and Human Services global health budget	2005-2018	2019-2020	Weighted average of actual DAH/budgeted DAH
**UN agencies**					

**WHO**	Programme budget[66]	DAH budget: Programme budget	2002-2019	2020	Weighted average of DAH/budget
**UNAIDS**	Unified Budget and Workplan, bienniums 2002-2017, 2018-2019, 2020-2021[67,68,69]	DAH budget: Unified Budget and Workplan	2002-2020	2020	Weighted average of DAH/Core Budget
**UNICEF**	Financial report and audited financial statements[7,69,70]	Total expenditure; Total health expenditure	1990-2019	2020	Weighted average of DAH/budget
**UNFPA**	Audited Financial report, statistical and financial review, contributions report, annual report, and Structured Funding dialogue [71,72]	Total health expenditure	2002-2019	2020	2019 DAH/budget
**PAHO**	Proposed program budget[10]	Total regular budget, estimated voluntary contributions	2000-2019	2020	Weighted average of DAH/budget
**NGOs**	VolAg (1990-2014), GuideStar (2015-2017), sample of top NGOs [25,26]	Revenue breakdowns for: US public, non-US public, private, in-kind, BMGF; total overseas expenditures	1990-2017	2018-2020	Regression on DAH, US GDP, and USAID and private voluntary organization (PVO) revenue

* Years of budget data used for modeling versus years underlying DAH data unavailable thus modeled: The data used to estimate DAH by channel vary across channels. Table S3.1 reports our primary data used for each channel. Due to reporting lags there are some years we need to estimate disbursement using additional data sources. These additional data sources, the years in which the primary data is modeled, the years the additional data is available, and the methods for this estimating these modeled years are reported in Table S3.3. Years of budget data used for modeling are the years of additional data available to us. We rely on historic trends to inform our estimates so we rely on many years of additional data despite only modeling a few years of primary data. Years underlying DAH data unavailable thus modeled are the years the primary data is incomplete and thus estimated using additional data. See example below for more details for Australia.

**Box 3.1. EXAMPLE - Australia's primary and additional data sources**
**Project-level data for health-related projects funded by Australia's bilateral aid agencies are available from the OECD's CRS database through 2018. This is the primary data source used to estimate DAH channeled by Australian aid agencies, as described in Table S3.1. 2019 and 2020 are incomplete because of lags in reporting. To estimate DAH disbursed for 2019-2020, additional data are available from Australia's International Development Assistance budget (2008-2020) and Australia's Overseas Aid Program budget**

(1998-2008), as described in Table S3.3. These sources provide health-specific official development assistance (ODA) budgeted by Australia, 1998-2020. We convert countries' budgeted ODA, as given in nominal local currency units, to nominal US dollars using the OECD's currency exchange rate series based on USD monthly averages. To estimate DAH disbursed in 2019-2020, we calculated the ratio of disbursed DAH (from the CRS database) relative to budgeted DAH (from the International Development Assistance and Overseas Aid Program budgets) for 1998-2018. We combine the most recent three ratios into a single estimate by taking a weighted average, weighting substantially higher the most recent year. We multiply this ratio – the estimated disbursed DAH to budgeted DAH – by the 2019 or 2020 budgeted DAH to estimate disbursed DAH in those years. These methods are described more fully in Dieleman et al.[1]

S3.1.2 DISAGGREGATING BY HEALTH FOCUS AREA

Similar to our previous work, the analysis of health focus areas included assessments of development assistance for HIV/AIDS, tuberculosis (TB), malaria, reproductive and maternal health, newborn and child health, other infectious diseases (including COVID-19), non-communicable diseases, and SWAps and health system strengthening by using keyword searches within descriptive fields. These were chosen as the areas of focus because of their relevance to current policy debates about global health financing and data availability.

In effect, DAH was disaggregated into nine health focus areas: HIV/AIDS, tuberculosis, malaria, reproductive and maternal health, newborn and child health, non-communicable diseases, SWAps/health sector support, other infectious diseases, and other. For most data sources, project-level data were available only through 2019. Methods to estimate health focus area allocations for 2020 are described in more detail below. Keyword searches were performed for a subset of global health channels that provide project-level data with project titles or descriptions. These sources include the bilateral development assistance agencies from 24 DAC member countries, one DAC participant country, THE GLOBAL FUND, the World Bank, Asian Development Bank, African Development Bank, Inter-American Development Bank, Bill & Melinda Gates Foundations, Non-government Organizations, US foundations, Wellcome Trust and the European Economic Area. The keywords used are outlined in Table S3.4 below. Descriptive fields were adjusted so that they were all in capitalized letters, special characters were modified, and search terms with multiple words were put between quotation marks. All keywords were translated into nine major languages (English, Spanish, French, Portuguese, Italian, Dutch, German, Norwegian, and Swedish) used in the OECD CRS, checked for double meanings across all languages, and adjusted accordingly.

Total DAH was split across the health focus areas using weighted averages based on the number of keywords present in each project's descriptive variables. If, for example, three keywords suggested the project focused on HIV/AIDS and two keywords related to tuberculosis, three-fifths of the project's total DAH was allocated to HIV/AIDS and two-fifths was allocated to tuberculosis. To account for the sensitivity of this method, several checks were implemented after the keyword searches to ensure the project was accurately categorized. First, projects that were tagged as child and newborn vaccines and other infectious diseases were categorized as child and newborn vaccines only. Second, projects that were tagged as one of the three major infectious diseases (HIV/AIDS, tuberculosis, or malaria) and other infectious diseases were categorized under only HIV/AIDS, tuberculosis, or malaria.

Box 3.2. EXAMPLE. Post-keyword search weighting

A project in the CRS database had a value of $1,000 of DAH. A keyword search conducted on this project's title and description tagged five keywords: 3 keywords related to HIV/AIDs and 2 keywords related to tuberculosis. Therefore, $600, or 3/5 of total DAH, was allocated to HIV/AIDS, while $400, or 2/5 of total DAH, was allocated to tuberculosis.

In addition to keyword searches, funds were allocated to health focus areas based on characteristics of the channel or additional channel variables. For the bilateral agencies and the EC, purpose codes from the CRS were used to

supplement keyword searches. For the World Bank-IDA and -IBRD, health focus areas were also determined by the project sector codes and theme codes, which included percentages of health funds that targeted each theme. All funds from Gavi were allocated to child and newborn vaccines, health system strengthening and non-communicable diseases and all funds from UNICEF to HIV/AIDS prevention, newborn and child health, ebola, and reproductive and maternal health – other (plus a very small amount being unallocable). Funds from THE GLOBAL FUND were distributed to malaria, HIV/AIDS, TB, and health sector support based on disease components. Within each disease component, keyword searches on programmatic budget data and project descriptions were conducted to distribute among program areas. Funds from UNAIDS were allocated to HIV/AIDS and TB with specific program areas determined by budget information. UNITAID funds were allocated based on assignments made by correspondence. UNFPA funds were allocated to specific health focus areas based on project expenditure data from its financial review report. PAHO and WHO funds were allocated to specific health focus areas based on project expenditure data from correspondence budget data and their proposed programme budgets. For all channels, projects listed as HIV/TB were distributed evenly among the two health focus categories. See Table S3.5 below for more details on these categorizations.

Table S3.4. Terms for keyword searches

Health focus area level	Program area	Keywords
**HIV/AIDS**	HIV envelope/other	" HUMANIMMUNODEFVIRUS " " SIDA " " OVC " " H I V " " HIV " " AIDS " " HUMAN IMMUNODEFICIENCY " " REVERSE TRANSCRIPTASE INHIBITOR " " ACQUIRED IMMUNE DEFICIENCY SYNDROME " " ACQUIRED IMMUNNODEFICIENCY " "RETROVIRAL " " VCT " " MALE CIRCUMCISION" " ART " " ARV " " CD4 COUNT " " HAART " " PMTCT " " MOTHER TO CHILD TRANSMISSION" " MOTHER TO CHILD AIDS TRANSMISSION" " PARENT TO CHILD TRANSMISSION" " PRESIDENT S EMERGENCY PLAN FOR AIDS RELIEF " " PEPFAR " " THREE DISEASES FUND " " 3 DISEASES FUND " " EMTCT " " 90 90 90 " " MOTHERTOCHILD"
	Care and Support	" CAREANDSUPPORT " " CARE ACTIVIT" " PAIN RELIEF " " SYMPTOM RELIEF " " PSYCHO SOCIAL SUPPORT " " CHRONICALLY ILL " " CLINICAL MONITORING " " CARE AND SUPPORT " " PSYCHOLOGICAL SERVICE" " PSYCHOLOGICAL SUPPORT " " PSYCHOSOCIAL SUPPORT " " PSYCHOSOCIAL SERVICE" " MATERIAL SUPPORT " " HOME BASED CARE " " PALLIATIVE CARE "
	Counseling and Testing	" COUNSELING " " TESTING " " VCT " " COUNSELLING " " COUNSELINGANDTESTING " " DIAGNOS"
	Orphans and Vulnerable Children	" VULNERABLECHILD" " OVC " " ORPHAN" " VULNERABLE CHILD" " INFECTED CHILD"
	Prevention of mother-to-child transmission (PMTCT)	" MOTHERTOCHILD" " MOTHER TO CHILD" " PARENT TO CHILD" " PMTCT " " EMTCT " " OPTION B "
	Prevention	" CONDOM" " PREVENT" " HIV EDUCATION " " AIDS EDUCATION " " REDUCING THE TRANSMISSION OF HIV " " REDUCE THE TRANSMISSION OF HIV " " MALE CIRCUMCISION" " SAFE BLOOD SUPPL" " SAFE INJECTION" " ABSTINENCE " " BLOOD SAFETY " " MICROBICIDE" " HARM REDUCTION " " PREP " " PEP " " PROPHYLAXIS " " ABCD " " BE FAITHFUL " " EARLY DETECTION "
	Treatment	"RETROVIRAL " " TREAT" " ART " " ARV " " CD4 COUNT " " HAART " " VIRAL LOAD " " VIRAL BURDEN " " VIRAL TITER

Health focus area level	Program area	Keywords
		" " DRUG REGIMEN" " FIRST LINE " " REVERSE TRANSCRIPTASE INHIBITOR "
	Antimicrobial resistance	" ANTIMICROBIAL RESISTAN" " ANTI MICROBIAL RESISTAN" " ANTIBIOTIC RESISTAN" " AMR " "DRUG RESISTAN" " MDR " " XDR " " RESISTANCE TESTING " " DRUG SUSCEPTIBILITY TESTING " " DST " " SECOND LINE "
**Tuberculosis**	Tuberculosis envelope/other	" TUBERCULOSIS " " TB " " TBC " " TUBERCULAR" " DOTS " " DIRECTLY OBSERVED TREATMENT " " RIFAMPICIN " " ISONIAZID " " THREE DISEASES FUND " " 3 DISEASES FUND " " RIFAMPIN "
	Treatment	" TREAT" " DOTS " " FIRST LINE " " DRUGS " " RIFAMPICIN " " RIFAMPIN " " ISONIAZID " " INH " " PYRAZINAMIDE " " PZA " " ETHAMBUTOL " " EMB " " STREPTOMYCIN " " SM " " STM " " PATIENT KIT " " INJECTABLE AGENT" " FLUOROQUINOLONES " " REGIMEN" " CASE MANAGEMENT " " ANTIMICROBIAL THERAPY " " DRUG SUSCEPTIBLE " " DRUG SENSITIVE "
	Diagnosis	" MICROSCOPY " " SPUTUM " " SMEAR " " CULTURE " " BACTERIOLOGICALLY CONFIRMED " " LABORATORY CONFIRMED " " GENEXPERT " " XPERT MTB RIF " " CHEST X RAY" " DIAGNOS" " LINE PROBE ASSAY "
	Antimicrobial resistance	" ANTIMICROBIAL RESISTAN" " ANTI MICROBIAL RESISTAN" " ANTIBIOTIC RESISTAN" " AMR " "DRUG RESISTAN" " MDR " " XDR " " RESISTANCE TESTING " " DRUG SUSCEPTIBILITY TESTING " " DST " " SECOND LINE "
**Malaria**	Malaria envelope/other	" MALARIA " " FALCIPARUM " " ANOPHELES " " ARTEMISININ " " PRIMAQUINE " " INDOOR RESIDUAL SPRAY" " INDOORRESIDUALSPRAY" " IRS " "VIVAX " " BEDNET" " BED NET" " SMITN " " ITN " " LLIN " " INSECTICIDAL NET" " INSECTICIDE TREATED NET" " THREE DISEASES FUND " " 3 DISEASES FUND " " CHLOROQUINE " " ANTI MALARIAL " " ANTIMALARIAL "
	Diagnosis	" DIAGNOS" " CASE DETECTION " " MICROSCOPY " " BLOOD SURVEY" " BIOLOGICAL TESTING " " EDT " " LAMP " " RDT "
	Community outreach	" COMMUNITYOUTREACH " " OUTREACH " " COMMUNITY MOBILIZATION" " AWARE" " COMMUNICATION STRATEGY " " SOCIAL COMMUNICATION " " PARTNERSHIP" " ACTIVITIES NEAR COMMUNITIES " " BCC " " BEHAVIORAL CHANGE COMMUNICATION " " BEHAVIOURAL CHANGE COMMUNICATION " " BEHAVIOR CHANGE COMMUNICATION " " BEHAVIOUR CHANGE COMMUNICATION " " SOCIAL MOBILIZATION "
	Vector control: bednets	" BEDNET" " BED NET" " SMITN " " ITN " " LLIN " " INSECTICIDAL NET" " INSECTICIDE TREAT"
	Vector control: irs	" INDOORRESIDUALSPRAY" " IRS " " REDUCE THE PARASITE RESERVOIR " " FOGGING " " COILS " " LARVICID" " LARVACID" " VECTOR CONTROL" "RESIDUAL SPRAY" " RESIDUALSSPRAY " "INDOOR SPRAY" " INDOORSPRAY "
	Vector control: other than bednets and irs	" PREVENT" " IPT " " SMC " " SEASONAL MALARIA CHEMOPREVENTION "
	Treatment	" ARTEMISININ " " PRIMAQUINE " " ACT " " DRUG" " TREAT" " CASE MANAGEMENT " " COMBINATION THERAPY " " ANTI MALARIAL " " ANTIMALARIAL " " CHLOROQUINE "

Health focus area level	Program area	Keywords
	Antimicrobial resistance	" ANTIMICROBIAL RESISTAN" " ANTI MICROBIAL RESISTAN" " ANTIBIOTIC RESISTAN" " AMR " "DRUG RESISTAN" " MDR " " XDR " " RESISTANCE TESTING " " DRUG SUSCEPTIBILITY TESTING " " DST " " SECOND LINE "
**Reproductive and maternal health**	envelope/other	" FERTILITY " " FAMILY PLANNING " " FP " " BIRTH" " WOMEN HEALTH " " WOMEN S HEALTH " " WOMENS HEALTH " " CONTRACEP" " IPPF " " PLANNED PARENTHOOD " " ABORTION" " UNFPA " " POSTPARTUM " " POST PARTUM " " MATERNAL " " MATERNITY " " MOTHER" " SBA " " ANTENATAL " " PRENATAL " " PERINATAL " " POSTNATAL " " FETUS" " FETAL" " IPTP " " REPRODUCTIVE HEALTH " " OBSTETRIC" " PREGNANC" " RH " " REPROD " " RHCS " " SEXUAL HEALTH " " SYPHILIS " " FISTULA " " ANEMI" " ANAEMI" " FOETUS" " FOETAL " " FGM " " FEMALE GENITAL MUTILATION " " FEMALE GENITAL CUTTING " " FEMALE CIRCUMCISION " " SBAS " " OBSTRUCTED LABOR " " OBSTRUCTED LABOUR " " MNCH" " RNCH " " RCH " " RNH " " MNH " " MCH " " EMAS " " MCNH " " PMNCH " "ECLAMPSIA " " PRETERM " " ANC " " WCAH " " TBA " " TBAS " " BIRTH ATTENDANT" " CESAREAN" " CAESAREAN" " C SECTION" " STI " " STD " " SEXUALLY TRANSMITTED " " CHILD MARRIAGE " " EARLY MARRIAGE " " FORCED MARRIAGE " " INFORMAL MARRIAGE "
	Family planning	" FERTILITY " " FAMILY PLANNING " " FP " " BIRTH SPACING " " CONTRACEPT" " FAMILY SIZE" " IPPF " " PLANNED PARENTHOOD " " ABORTION" " BIRTH CONTROL " " CONDOM" " IUD " " VASECTOMY " " TUBULAR LIGATION"
	Maternal health	" POSTPARTUM " " POST PARTUM " " MATERNAL HEALTH " " MATERNAL MORTALITY " " MATERNAL DEATH" " SAFE MOTHERHOOD " " BIRTH ATTENDANT" " SBA " " ANTENATAL " " PRENATAL " " PERINATAL " " POSTNATAL " " FETUS" " FETAL" " IPTP " " MATERNITY " " OBSTETRIC" " PREGNANC" " FISTULA " " SEPSIS " " SEPTICEMIA " " ANEMI" " ANAEMI" " FOETUS" " FOETAL " " SBAS " " OBSTRUCTED LABOR " " OBSTRUCTURED LABOUR " " DELIVERY ROOM" " CHILD DELIVERY " " MIDWIV" " MIDWIFE" "ECLAMPSIA " " PRETERM " " ANC " " TBA " " TBAS " " CESAREAN" " CAESAREAN" " C SECTION" " MNH " " MCH " " MNCH " " MCNH "
**Newborn and child health**	envelope/other	" NEONATAL " " PERINATAL " " POSTNATAL " " MALNUTRITION " " VITAMIN A " " BREAST FE" " BREASTFE" " MICRONUTRIENT" " FORTIFICATION " " STUNT" " WASTING " " BABY FRIENDLY HOSPITAL INITIATIVE " " BREASTMILK " " BREAST MILK " " IODINE " " IODIZED " " IODIZATION " " VAD " " LACTAT" " FOLIC ACID " " FOLAT" " VACCIN" " IMMUNIZ" " POLIO " " DIPHTHERIA " " TETANUS " " PERTUSSIS " " DTP " " HIB " " ROTAVIRUS " " MEASLES " " IMMUNIS" " HEPB " " INJECTION SAFETY " " RUBELLA " " MENINGITIS " " PENTA " " PENTAVALENT " " PNEUMONIA " " PNEUMOCOCC" " HAEMOPHILUS INFLUENZAE " " TETRA " " GAVI " " CHILDHEALTH " " CHILD HEALTH " " CHILDREN " " INFANT" " NEWBORN" " CHILD MORTALITY " " UNDER FIVE MORTALITY " " CHILD SURVIVAL " " CHILDHOOD ILLNESS" " LRI " " RESPIRATORY INFECTION" " DIARRHEA"

Health focus area level	Program area	Keywords
		" DIARRHOEA" " ORAL REHYDRATION " " ORT " " ORS " " UNICEF " " MNCH" " RNCH " " RCH " " RNH " " MNH " " MCH " " EMAS " " MCNH " " POLIOVIRUS " " LRTI " " GPEI " " GLOBAL POLIO ERADICATION INITIATIVE "
	Child/newborn nutrition	" NUTRITION" " MALNUTRITION " " BIRTH WEIGHT " " BIRTHWEIGHT " " VITAMIN A " " BREAST FE" " BREASTFE" " FEEDING " " MICRONUTRIENT" " ZINC " " FORTIFICATION " " STUNT" " WASTING " " UNDERWEIGHT " " BABY FRIENDLY HOSPITAL INITIATIVE " " BREASTMILK " " BREAST MILK " " IODINE " " IODIZED " " IODIZATION " " VAD " " LACTAT" " FOLIC ACID " " FOLAT" " IRON " " DEWORMING "
	Child/newborn vaccines	" POLIO " " VACCIN" " IMMUNIZ" " DIPHTHERIA " " TETANUS " " PERTUSSIS " " DTP " " HIB " " ROTAVIRUS " " MEASLES " " IMMUNIS" " HEPB " " INJECTION SAFETY " " RUBELLA " " MENINGITIS " " PENTA " " PENTAVALENT " " PNEUMONIA " " PNEUMOCOCC" " HAEMOPHILUS INFLUENZAE " " TETRA " " GAVI " " POLIOVIRUS " " DPT " " PCV " " GVAP " " VACCINE ACTION PLAN " " IA2030 " " GPEI " " GLOBAL POLIO ERADICATION INITIATIVE "
**Non-communicable diseases**	envelope/other	" TOBACCO" " SMOK" "CIGAR" " FCTC " " TFI " " SCHIZOPHRENIA " " MENTAL HEALTH " " NEUROTIC " " NEUROSIS " " NEUROSES " " NEUROLOGICAL" " PSYCHOLOG" " PSYCHIATR" " EMOTIONAL DISORDER" " OBSESSIVE COMPULSIVE " " OCD " " PTSD " " POST TRAUMATIC " " POSTTRAUMATIC " " ALCOHOL DEPENDEN" " ALCOHOL ABUSE " " ADDICTION " " BEHAVIORAL DISORDER" " DRUG ABUSE " " SUBSTANCE ABUSE " " OPIOID " " COCAINE " " AMPHETAMIN" " DEPRESSIVE DISORDER" " DEPRESSION " " DYSTHYMIA " " BIPOLAR " " ANXIETY " " EATING DISORDER " " PHOBIA " " DRUG DEPENDEN" " ATTENTION DEFICIT HYPERACTIVITY DISORDER " " ADHD " " PANIC DISORDER" " SELF HARM " " STRESS DISORDER" " SUBSTANCE USE DISORDER" " DRUG USE DISORDER" " MENTAL ILLNESS" " MENTAL DISORDER" " PSYCHOSOCIAL " " PSYCHO SOCIAL " " HEROIN " " OXYCODONE " " ATTENTION DEFICIT DISORDER " " SUICIDE PREVENTION" " HEADACHE " " ANOREXIA " " BULIMIA " " HYPERKINETIC DISORDER" " PERSONALITY DISORDER" " FETAL ALCOHOL SYNDROME" " PSYCHOSIS " " DOWN SYNDROME " " DOWN S SYNDROME " " DOWNS SYNDROME " " AUTISM " " ASPERGER " " DEVELOPMENTAL DISORDER" " CONDUCT DISORDER" " INTELLECTUAL DISABILIT" " MENTAL DISAB" " MENTAL RETARDATION " " ALZHEIMER" " DEMENTIA" " EPILEPSY " " MIGRAINE " " PARKINSON" " MENTALLY DISAB" " NERVOUS SYSTEM" " SYNAPSE" " NON COMMUNICABLE " " NONCOMMUNICABLE " " CANCER" " CHEMOTHERAPY " " RADIATION " " NEOPLAS" " TUMOR " " LEUKEMIA " " LYMPHOMA " " MYELOMA " " HPV " " HUMAN PAPILLOMA VIRUS " " HEP C " " HEPATITIS C " " DIABET" " INSULIN " " ENDOCRINE " " RHEUMAT" " ISCHAEMIC " " ISCHEMIC " " CIRCULATORY " " CIRRHOSIS " " DIGESTIVE DISEASE" "

Health focus area level	Program area	Keywords
		OTHER DIGESTIVE " " PEPTIC " " APPENDICITIS " " GASTRITIS " " GENITOURINARY " " UROGENITAL " " MUSCULOSKELETAL " " GOUT " " BACK PAIN " " MACULAR " " HEARING " " AUDIOLOG" " PERIODONTAL " " CARIES " " CONGENITAL " " OBESITY " " OVERWEIGHT " " GLAUCOMA " " HYPERTENSI" " HERNIA " "ARTHRITIS " " CLEFT LIP" " CLEFT PALATE" " PHENYLKETONURIA " " SICKLE CELL" " DREPANOCYTOSIS " " HEMOPHILIA " " HAEMOPHILIA " " THALASSEMIA " " GENETIC DISORDER" " HEART DISEASE" " CHRONIC RESPIRATORY " " COPD " " STROKE " " CATARACT" " CHRONIC OBSTRUCTIVE PULMONARY DISEASE" " ASTHMA " " SKIN DISEASE" " DERMATITIS " " PSORIASIS " " SCABIES " " PHYSICAL DISAB" " DENTAL " " ORAL HEALTH " " CVD " " IHD " " CKD " " KIDNEY DISEASE" " MSK " " EYE " " CEREBROVASCULAR " " VASCULAR " " BLOOD PRESSURE " " ACUTE GLOMERULONEPHRITIS " " ALOPECIA AREATA " " ANEURYSM " " ANGINA " " ARTERY " " ATHEROSCLEROSIS " " ATRIAL FIBRILLATION" " ATRIAL FLUTTER " " BENIGN PROSTATIC HYPERPLASIA " "BLASTOMA" " BLIND " " PREVENTABLE BLINDNESS " " AVOIDABLE BLINDNESS " " BLOOD DISORDER" " BRONCHITI" " CARCINOMA " " CARDIAC " " CARDIO" " CELLULITIS " " CEREBRAL " " CORONARY " " DEAF" " DECUBITUS ULCER " "DIALYSIS" " DUODENITIS " " ECZEMA " " EKZEMA " " EDENTULISM " " ENDOCARDITIS " " FIBROSIS " " G6PD DEFICIENCY " " GALL BLADDER " " BILE DUCT " " GLYCEMI" " GLYCAEMI" " HEMOGLOBINOPATH" " HEMOLYTIC ANEMIA " " HODGKIN" " INSOMNIA " " INTERSTITIAL LUNG DISEASE" " INTESTINAL OBSTRUCTION" " LEUKAEMIA " " MELANOMA" " MULTIPLE SCLEROSIS " " MYOCARD" " NCD " " NECK PAIN " " NEPHRITIS " " NEPHROSIS " " NEURAL TUBE DEFECT" " NEURODEGENERATIVE " " INFLAMMATORY BOWEL" " ONCOLOG" " OPTICAL " " OSTEOMYELITIS " " OTITIS MEDIA " " PANCREATITIS " " PARALYTIC ILEUS " " PERITONEAL " " PNEUMOCONIOSIS " " PROSTATE " " PRURITUS " " SARCOIDOSIS " " PYELONEPHRITIS " " REFRACTIVE ERROR" " RENAL " " RETINA " " SARCOMA " " SUBCUTANEOUS DISEASE" " URINARY DISEASE" " URINARY TRACT INFECTION" " UROLITHIASIS " " URTICARIA " " VENTRICULAR " " VISION LOSS " " ACCOMODATION DISORDER" " SENSE ORGAN " " GUILLAIN BARRE SYNDROME" " IMPETIGO " " LOSE WEIGHT " " BIRTH DEFECT" "PAPILLOMAVIRUS" " GENE DEFECT" " PHYSICALLY DISAB" " TUMOUR" " BRAIN INJUR" " MAMMOGRA" " ANTITUMOR " " ANTITUMOUR " " BARIATRIC" " FATTY LIVER" " IMMUNOTHERAPY " " CHROMOSOMAL ABERRATION" " PERIODONTITIS " " OSTEOPOROSIS " "MALIGNANC" " NEURON"

Health focus area level	Program area	Keywords
	Mental health	" SCHIZOPHRENIA " " MENTAL HEALTH " " NEUROTIC " " NEUROSIS " " NEUROSES " " NEUROLOGICAL" " PSYCHOLOG" " PSYCHIATR" " EMOTIONAL DISORDER" " OBSESSIVE COMPULSIVE " " OCD " " PTSD " " POST TRAUMATIC " " POSTTRAUMATIC " " ALCOHOL DEPENDEN" " ALCOHOL ABUSE " " ADDICTION " " BEHAVIORAL DISORDER" " DRUG ABUSE " " SUBSTANCE ABUSE " " OPIOID " " COCAINE " " AMPHETAMIN" " DEPRESSIVE DISORDER" " DEPRESSION " " DYSTHYMIA " " BIPOLAR " " ANXIETY " " EATING DISORDER " " PHOBIA " " DRUG DEPENDEN" " ATTENTION DEFICIT HYPERACTIVITY DISORDER " " ADHD " " PANIC DISORDER" " SELF HARM " " STRESS DISORDER" " SUBSTANCE USE DISORDER" " DRUG USE DISORDER" " MENTAL ILLNESS" " MENTAL DISORDER" " PSYCHOSOCIAL " " PSYCHO SOCIAL " " HEROIN " " OXYCODONE " " ATTENTION DEFICIT DISORDER " " SUICIDE PREVENTION" " HEADACHE " " ANOREXIA " " BULIMIA " " HYPERKINETIC DISORDER" " PERSONALITY DISORDER" " FETAL ALCOHOL SYNDROME" " PSYCHOSIS " " DOWN SYNDROME " " DOWN S SYNDROME " " DOWNS SYNDROME " " AUTISM " " ASPERGER " " DEVELOPMENTAL DISORDER" " CONDUCT DISORDER" " INTELLECTUAL DISABILIT" " MENTAL DISAB" " MENTAL RETARDATION " " ALZHEIMER" " DEMENTIA" " EPILEPSY " " MIGRAINE " " PARKINSON" " MENTALLY DISAB" " NERVOUS SYSTEM" " SYNAPSE"
	Tobacco	" TOBACCO" " SMOK" "CIGAR" " FCTC " " TFI "
**SWAps/ Health sector support**	envelope/other	" SWAP" " TRAINING " " CAPACIT" " DATA SYSTEM" " SECTOR WIDE APPROACH" " HEALTH SYSTEM" " SECTOR PROGRAM" " BUDGET SUPPORT" " SECTOR SUPPORT " " HSS " " TRACKING PROGRESS " " SKILLED WORKER" " HEALTH WORKER" " SKILLED STAFF " " HEALTH PROFESSIONAL" " FACILITIES " " ESSENTIAL MEDICINES " " POLICY DEVELOPMENT" " MEDICAL EQUIPMENT" " SURGICAL EQUIPMENT" " HOSPITAL EQUIPMENT" " HOSPITAL EQMT " " INSTITUTIONAL STRENGTHENING " " HSPSP " " M&E " " M & E " " MONITORING " " SURVEILLANCE " " GOVERNANCE " " HUMAN RESOURCE" " HUMAN CAPITAL " " SCALING UP " " REALLOCATE RESOURCES " " STRATEGIES AND PROGRAM" " HIV STRATEG" " PROGRAM IN COUNTRY ACTIVITIES " " STRATEGIC INFORMATION " " PROCUREMENT " " EVIDENCE BASED " " CASE REPORTING " " MEDICAL WORKER" " HEALTH CARE PERSONNEL " " OPERATIONAL RESEARCH " " SUPPORTIVE ENVIRONMENT " " INFORMATION SYSTEM" " WORKFORCE " " INFRASTRUCTUR" " MEDICAL EDUCATION " " CASE NOTIFICATION " " CASE FINDING " " LABORATORY

Health focus area level	Program area	Keywords
		STRENGTHENING " " LABORATORY QUALITY " " LABORATORY NETWORK" " CONTROL SERVICES " " INFECTION CONTROL " " CONTROL PROGRAM" " SCALE UP" " STOP TB STRATEGY " " HEALTH EDUCATION " " CONTINUING EDUCATION " " SUPPLY " " HEALTH MANAGEMENT" " HEALTH POLICY " " MANAGEMENT AND COORDINATION " " ADMINISTRATIVE MANAGEMENT " " MANAGEMENT AND ADMINISTRATION " " COLD CHAIN" " HEALTH PROMOTION " " TECHNICAL ASSISTANCE " " DSS " " DISTRIBUTION SYSTEMS " " SERVICE DELIVERY " " BUILDINGS " " HEALTH FACILIT" " CONSTRUCT" " MEDICAL SCHOOL" "CENTERS OF EXCELLENCE" "NURSE" "DOCTOR" "PHYSICIAN" "MEDICAL LABORATORY SCIENTIST" "SURGEON" "SPECIALIST" "PHARMACIST" "HEALTH LABOR" "LABOR MARKET" "PERSONNEL" "MEDICAL PRACTIONER" "DENTAL PRACTIONER" "TASK SHIFTING"
	Human resources	" INFRASTRUCTUR" " MEDICAL EQUIPMENT" " SURGICAL EQUIPMENT" " HOSPITAL EQUIPMENT" " HOSPITAL EQMT " " BUILDINGS " " HEALTH FACILIT" " CONSTRUCT" " MEDICAL SCHOOL" "CENTERS OF EXCELLENCE" " TRAINING " " CAPACIT" " SKILLED WORKER" " HEALTH WORKER" " SKILLED STAFF " " HEALTH PROFESSIONAL" " HUMAN RESOURCE" " HUMAN CAPITAL " " MEDICAL WORKER" " WORKFORCE " " MEDICAL EDUCATION " " HEALTH EDUCATION " " CONTINUING EDUCATION " " HEALTH MANAGEMENT" " MANAGEMENT AND COORDINATION " " ADMINISTRATIVE MANAGEMENT " " MANAGEMENT AND ADMINISTRATION " "NURSE" "DOCTOR" "PHYSICIAN" "MIDWIFE" "MIDWIVES" "MEDICAL LABORATORY SCIENTIST" "SURGEON" "SPECIALIST" "PHARMACIST" "HEALTH LABOR" "LABOR MARKET" "PERSONNEL" "MEDICAL PRACTIONER" "DENTAL PRACTIONER" "TASK SHIFTING" " TECHNICAL ASSISTANCE "
	Pandemic preparedness	" PANDEMIC PREPAREDNESS " " PANDEMIC RESPONSE" " PANDEMIC ALERT" " EPIDEMIC ALERT" " EPIDEMIC RESPONSE" " EPIDEMIC PREPAREDNESS " " OUTBREAK RESPONSE" " OUTBREAK ALERT" " OUTBREAK PREPAREDNESS " " PANDEMIC INFLUENZA " " EPIDEMIOLOGICAL INVESTIGATION" " CONTACT MANAGEMENT " " PREPAREDNESS AND RESPONSE PLAN" " PREPAREDNESS & RESPONSE PLAN" " BIOSAFETY MEASURE" "EARLY WARNING " " HEALTH SECURITY PREPAREDNESS " " HEALTH SECURITY RISK ASSESSMENT " " RAPID RESPONSE STRATEG" " CONTACT TRAC"
**Other infectious diseases**	envelope/other	" INFECTIOUS " " COMMUNICABLE " " TRICHURIASIS " " YELLOW FEVER " " WHIPWORM " " TRACHOMA " " SCHISTOSOMIASIS " " BILHARZIA " " SNAIL FEVER " " KAYAYAMA FEVER " " RABIES " " ONCHOCERCIASIS " " RIVER BLINDNESS " " ROBLES DISEASE" " LYMPHATIC FILARIASIS " " ELEPHANTIASIS " " LEISHMANIASIS " " LEISHMANIOSIS " " HOOKWORM " " FOOD BORNE " " FOODBORNE " " ECHINOCOCCOSIS " " HYDATID DISEASE" " HYDATIDOSIS " " DENGUE " " CYSTICERCOSIS " " CHAGAS "

Health focus area level	Program area	Keywords
		" TRYPANOSOMIASIS " " SLEEPING SICKNESS " " ASCARIASIS " " TROPICAL DISEASE" " CHOLERA " " DYSENTERY " " PARASITE DISEASE" " FAO " " NEGLECTED TROPICAL DISEASE" "TYPHOID" " LEPROSY " " BURULI ULCER " " EBOLA" " EBOV " " EVD " " ZIKA " " ZIKV " " GUINEA WORM " " DRACUNCULIASIS " " FILARIASIS " " HEPATITIS E" " ENCEPHALITIS " " VARICELLA" " INFLUENZA" " FLU " " NTD " " HEPATITIS A " " HEPATITIS D " " HEP A " " HEP D " " ROUNDWORM" " RINGWORM" " TAPEWORM" " FLATWORM" " CHIKUNGUNYA " " LASSA " " MERS " " NIPAH " " MARBURG " " SARS " " CORONAVIRUS " " COVID" " SARS COV 2 " " NCOV "
	Antimicrobial resistance	" ANTIMICROBIAL RESISTAN" " ANTI MICROBIAL RESISTAN" " ANTIBIOTIC RESISTAN" " AMR " "DRUG RESISTAN" " MDR " " XDR " " RESISTANCE TESTING " " DRUG SUSCEPTIBILITY TESTING " " DST " " SECOND LINE "

Table S3.5. Additional health focus area categorizations

Channel	Allocation criteria	Health focus area
**Bilaterals and the EC**	CRS purpose code 13030, family planning	Family planning
	CRS purpose code 13020, reproductive health care	Maternal health, non-family planning
	CRS purpose code 12240, basic nutrition	Child and newborn nutrition
	CRS purpose code 12250, infectious disease control and the keywords “child” or “vaccine” present in descriptive variables	Child and newborn vaccines
	CRS purpose code 13040, STD control including HIV/AIDS	HIV/AIDS
	CRS purpose code 12262, malaria control	Malaria, unspecified
	CRS purpose code 12250, infectious disease control and no other keywords present in the descriptive variables	Other infectious diseases
	CRS purpose code 12263, tuberculosis control	Tuberculosis
	CRS purpose code 12230, basic health infrastructure	SWAPs/health system strengthening - other
	CRS purpose code 12281, health personnel development	SWAPs/health system strengthening - Human resources
	CRS purpose code 12281, medical education/training	SWAPs/health system strengthening - Human resources
**World Bank IDA and IBRD**	Theme code population and reproductive health	Reproductive and maternal health, other
	Theme code tuberculosis	Tuberculosis, other
	Theme code child health	Newborn and child health, other
	Theme code HIV/AIDS	HIV/AIDS, other
	Theme code malaria	Malaria, other
	Theme code injuries and non-communicable diseases	Non-communicable diseases, other
	Theme code nutrition and food security	Newborn and child health, nutrition
	Theme code other communicable diseases	Other infectious diseases, other

Channel	Allocation criteria	Health focus area
	Theme code health system performance	SWAPs/health systems strengthening, other
	Theme code social analysis and monitoring	SWAPs/health systems strengthening, other
**UNFPA**	Family planning, population and development strategies, population and development, population dynamics<br>Output 02. Integrated sexual and reproductive health services	Family planning
	Reproductive health, maternal and newborn health, young people's SRH and sexuality education, HIV and STI prevention services, sexual and reproductive health, sexuality education<br>Output 02. Integrated sexual and reproductive health services	Maternal health
	Output 02. Integrated sexual and reproductive health services	Split equally between Family planning and Maternal health, unspecified, and HIV other
	HIV and STI prevention services, HIV and AIDS	HIV prevention
	Advocacy, Multisector, Gender equality and women's empowerment, reproductive rights, program coordination and assistance, adolescents and youth, civil society and rights for all, ending harmful practices, marginalized girls, protection rights.<br>Output 01. Sexual and reproductive health policies for those furthest behind<br>Output 02. Integrated sexual and reproductive health services<br>Output 06. Adolescents and youth skills and capabilities<br>Output 07. Adolescents and youth policies<br>Output 08. Youth leadership and participation<br>Output 12. Elimination of harmful practices	Reproductive and maternal health - other
	Health workforce capacity	Reproductive and maternal health HSS - human resources
	Data and policies, Data availability and analysis, Data production and dissemination, Monitoring and evaluation, National population data system, Organizational adaptability, Organizational effectiveness and efficiency, Population data analysis, Population dynamics, Program coordination and assistance, Programme effectiveness, Resources management.<br>Output 04. Delivery of sexual and reproductive health commodities<br>Output 05. Accountability for sexual and reproductive health<br>Output 09. Gender equality laws and policies<br>Output 10. Gender and sociocultural norms	Reproductive and maternal health HSS - other

Channel	Allocation criteria	Health focus area
	Output 11. Prevention and addressing of gender-based violence<br>Output 13. Population data systems<br>Output 14. Demographic intelligence<br>OEE1. Improved programming for results<br>OEE2. Optimized management of resources<br>OEE3. United Nationals coordination and coherence<br>OEE4. Communication, resources mobiliztion and partnerships	
**UNICEF**	HIV/AIDS results	HIV/AIDS – prevention, treatment, care, PMTCT, OVC, and other
	Nutrition results	Newborn and child health – nutrition
	Health results	Reproductive and maternal health – other<br>Newborn and child health – HSS, vaccines, other
	Ebola (starting in 2014)	Other infectious diseases - Ebola
	The keyword search was run on budget information for years 2008-2019<br>Program components in budget documents from 1998 to 2007	All program areas under HIV/AIDS and TB
**UNAIDS**	Assigned in database received through correspondence	HIV/AIDS (care and strengthening, counseling and testing, orphans and vulnerable children, PMTCT, prevention, treatment, other) , TB (diagnosis, treatment and other), HSS
**GAVI**	Disease components for Malaria, HIV/AIDS, TB, TB/HIV, and Other (health systems strengthening)<br>Keyword search on program service delivery areas	All program areas under Malaria, TB, HIV and Swap/HSS
**GLOBAL FUND**	Reproductive, maternal, newborn, child, and adolescent health (divided by 2); Research in human reproduction	Maternal health, unspecified
**WHO**	Nutrition	Child and newborn nutrition
	Vaccine-preventable diseases	Child and newborn vaccines
	Reproductive, maternal, newborn, child and adolescent health (divided by 2)	Child and newborn health, unspecified
	Aging and health;	Maternal, newborn, and child health, unspecified
	HIV/AIDS	HIV/AIDS
	Malaria	Malaria
	Tuberculosis	Tuberculosis
	Mental health and substance abuse	Non-communicable diseases, mental health
	Disabilities and rehabilitation; Non-communicable diseases; Violence and injuries	Non-communicable diseases, unspecified
	Neglected tropical diseases; Tropical disease research; Outbreak and crisis response (50%); Alert and response capacities (50%); Polio eradication	Other infectious diseases
	Health system information and evidence;<br>Integrated people-centered health services;<br>National health policies, strategies and plans;	SWAps/health system strengthening

Channel	Allocation criteria	Health focus area
	Access to medicines and health technologies and strengthening regulatory capacity; health emergency information and risk assessment (50%); Improved access to quality essential health services; Reduced number of people suffering financial hardships; Improved access to essential medicines, vaccines, diagnostics and devices for primary health care; Determinants of health addressed; Risk factors reduced through multisectoral action; Healthy settings and Health in All Policies promoted; Strengthen country capacity in data and innovation; Strengthen leadership, governance and advocacy for health; Financial, human and administrative resources managed in an efficient, effective, results-oriented and transparent manner	
	Country health emergency preparedness and the International Health Regulations; health emergency information and risk assessment(50%); Emergency operations; Emergency core services; Outbreak and crisis response(50%); Epidemic- and pandemic-prone diseases; Alert and response capacities(50%); Infectious hazard management; Countries prepared for health emergencies; Epidemics anf pandemics prevented; Health emergencies rapidly detected and responded to	SWAps/health system strengthening, pandemic preparedness
	Social determinants for health; Health and the environment; Food safety; Antimicrobial resistance; gender, equity and human rights mainstreaming	Other
	HIV/AIDS and STls; HIV/AIDS, TB and malaria (33%)	HIV/AIDS, unspecified
**PAHO**	Tuberculosis; HIV/AIDS, TB and malaria (33%)	Tuberculosis, unspecified
	HIV/AIDS, TB and malaria (33%); Malaria and other Vector-Borne Diseases (50%);	Malaria, unspecified
	Communicable diseases; Malaria and other Vector-Borne Diseases (50%); Neglected Tropical and zoonotic diseases; Response capacity for communicable diseases; Risk factors for communicable diseases; Elimination of communicable diseases	Other infectious diseases
	Nutrition; Nutrition food safety and food security; Malnutrition	Child and newborn nutrition
	Vaccíne-Preventable Diseases	Child and newborn vaccines
	Women, maternal, newborn, child, and adolescent and adult health	Maternal and child health, unspecified
	Mental Health and psychoactive substance use disorders; Intersectoral action on mental health; Access to services for NCDs and mental health conditions (50%)	Non-communicable diseases, mental health

Channel	Allocation criteria	Health focus area
	Non Communicable Diseases and Risk Factors; Chronic noncommunicable diseases; Access to services for NCDs and mental health conditions (50%); Risk factors for NCDs	Non-communicable diseases, unspecified
	Health systems leadership and governance; Human resources for health; Social protection and financing; Health systems information and evidence; Health services; People-centered integrated health services; Access to medical products and strengthening regulatory capacity; Health governance and financing, national health policies, strategies and plans; Access to comprehensive and quality health services; Access to health technologies; Strengthened stewardship and governance; Increased public financing for health; Strengthened financial protection; Health promotion and intersectoral action; Integrated information systems for health; Data, information, knowledge, and evidence; Research, ethics, and innovation for health; Leadership and governance; Management and administration	Swap/health system strengthening, unspecified
	Health emergencies preparedness and risk reduction;<br>Epidemic and pandemic prevention and control; Health Emergencies Detection and Response	Swap/health system strengthening, pandemic preparedness
	Violence and Injuries; Disabilities and Rehabilitation; Antimicrobial resistance; Aging and health; Gender, equity, human rights and ethnicity; Social determinants of health; Health and the environment; Strategic communications; Management and administration; Flexible and learning organization; Health throughout the life course; Quality care for older people; Response capacity for violence and injuries	Other

S3.1.3 Disaggregating preliminary estimates by health focus area

Estimates by health focus area for years in which descriptive data were not available (usually 2020) were obtained by modeling channel-specific DAH per health focus area as a function of time. Out-of-sample validation was used to test the predictive accuracy of a large suite of models, estimating the models using 1990-2010 data and predicting 2011 and 2012. The potential models included fractional multinomial logit regression, OLS regression, autoregressive integrated moving average (ARIMA) models, Epanechnikov kernel-weighted local polynomial smoothing, and multivariable fractional polynomial models. For each model, time was modeled linearly, with splines, and by including lag-dependent variables. Other methodologies considered included modeling health-focus-area-specific DAH as a dollar amount and as a fraction of the channel-specific total DAH. Lastly, models that involved transforming the dependent variable in natural log and logit transformed space were considered. In order to accommodate zero values in the logit transformation, the transformation described in Smithson and Verkuilen were applied.[73] Over 40 models and specifications were evaluated in total.

Each of the potential model and specification described above were estimated using data from 1990 through 2010, and then the estimated model was used to predict DAH by health focus area for 2011 and 2012. Since we have DAH

estimates for 2011 and 2012, we compared the modeled estimates and the observed estimates and calculated average percent deviation and average total absolute deviation for each model and specification across all the channels and health focus areas. A variant of the Epanechnikov kernel-weighted local polynomial smoothing had the smallest average percent deviations and average total absolute error. In this model and specification, health focus area-specific DAH fractions were independently estimated at the channel level after they were logit transformed. Time was the only independent variable included in the model. The health focus area-specific DAH estimates were adjusted so the sum of the channel's health focus area disbursements totaled channel-specific DAH envelope. Our preferred model, the Epanechnikov kernel-weighted local polynomial smoothing, minimized both the average percent deviation and the total absolute error out of sample, predicting two years ahead. See Dieleman et al. for a table that demonstrates the performance of four models, each with their optimal specification (as determined by the out-of-sample average percent deviation and total absolute error).[1]

S3.2 Tracking development assistance for health from bilateral aid agencies and the European Commission

OECD-DAC maintains two databases on aid flows: 1) the DAC annual aggregates database, which provides summaries of the total volume of flows from different donor countries and institutions, and 2) the CRS, which contains project- or activity-level data.[3] This year, we used the DAC databases to track health ODA from 24 OECD-DAC members (Austria, Australia, Belgium, Canada, Denmark, Finland, France, Germany, Greece, Ireland, Italy, Japan, Luxembourg, the Netherlands, New Zealand, Norway, Portugal, South Korea, Spain, Sweden, Switzerland, the United Kingdom, the United States, and the EC), and one DAC Participant country, United Arab Emirates, for the years 1990 to 2020. Observed data for the DAC members was available from 1990 to 2019, and observed data for the United Arab Emirates was available from 2009 to 2019. United Arab Emirates bilateral health ODA from 1990 to 2008 was obtained through personal correspondence. We determined the observed 2019 data for the United States was incomplete after consulting several USAID data tools and other data experts in the field. Therefore, we predicted 2019-2020 for the United States.

These two DAC databases track the following types of resource flows:

Official development assistance (ODA), defined as "flows of official financing administered with the promotion of the economic development and welfare of developing countries as the main objective"[2] is tracked from its 30 members (Austria, Australia, Belgium, Canada, Czech Republic, Denmark, Finland, France, Germany, Greece, Hungary, Iceland, Ireland, Italy, Japan, Luxembourg, the Netherlands, New Zealand, Norway, Poland, Portugal, Slovakia, Slovenia, South Korea, Spain, Sweden, Switzerland, the United Kingdom, the United States, and the EC). The CRS also now includes some private ODA, such as that funded by BMGF and the Global Fund to Fight AIDS, Tuberculosis and Malaria (THE GLOBAL FUND), as well as assistance from a number of non-DAC countries such as the United Arab Emirates and Kuwait.

ODA includes:

• Bilateral ODA, which is given directly by DAC members as aid to recipient governments, core contributions to NGOs and public-private partnerships, and earmarked funding to international organizations.

• Multilateral ODA, which includes core contributions to multilateral agencies such as WHO, UNFPA, THE GLOBAL FUND, Gavi, UNAIDS, UNICEF, PAHO, the World Bank, and other regional development banks. Only regular budgetary contributions to these institutions can be reported to the OECD-DAC; hence, extrabudgetary funds, including earmarked contributions that donors can report as bilateral ODA, are not included as multilateral ODA. Only 70% of core contributions to WHO can be counted as multilateral ODA.

a. Official development finance (ODF), which includes grants and loans made by multilateral agencies.

b. Other official flows (OOF), which refers to transactions that “do not meet the conditions for eligibility as Official Development Assistance or Official Aid, either because they are not primarily aimed at development, or because they have a Grant Element of less than 25 percent.”

The DAC aggregate tables include all multilateral development banks, THE GLOBAL FUND, operational activities of UN agencies and funds, and a few other multilateral agencies. The project-level data in the CRS cover a smaller subset of multilateral institutions, including UNAIDS, UNFPA, UNICEF, public-private partnerships including Gavi and THE GLOBAL FUND, some development banks, and BMGF, but do not reflect the core-funded operational activities of WHO prior to 2009, disbursements by Gavi prior to 2007 and BMGF prior to 2009, or all loans from the World Bank.

This research utilized the CRS as the principal source for tracking bilateral DAH. This is because the DAC aggregate tables do not report detailed project-level information about the recipient country and health focus area. The OECD sector codes for general health (121), basic health (122), and population programs (130) were used to identify health flows in the CRS. Only ODA related flows are used in our analysis, including OECD flow codes corresponding to ODA grants (11), ODA grant-like (12), ODA loans (13), and equity investment (19). In addition, we included select projects with OECD sector code for emergency aid (720) in order to capture additional funding for ebola and zika outbreaks. Projects with purpose code emergency food aid (72040) were excluded, and the remaining project descriptive variables were parsed for the keywords “EBOLA” and “ZIKA” to identify emergency aid projects targeting these diseases. Based on our definition of DAH, we also excluded any remaining projects containing any of the following keywords: " WATER " " SANITATION " " FOOD " " EARTHQUAKE " " HURRICANE " " TERREMOTO " " HURACAN " " FAMILY CARE STRUCTURES ".

To avoid double-counting, all identifiable earmarked commitments and disbursements between one disbursing agency to another were tagged and removed before calculating final DAH estimates. Income statements, expenditure statements, and keyword searches in the descriptive project fields were used to identify potential sources of double-counting.

In multilateral agencies, public-private partnerships, development banks, and foundations, when expenditure by recipient agency information was available, double counting was tagged and dropped in the disbursing agency from whom the resources was transferred. In the absence of detailed expenditure data in the disbursing agency that transferred resources, the income statement of the receiving disbursing agency that reflected the disbursement was used to flag the disbursement in the initial disbursing agency. Preliminary estimates were generated based on the annual disbursed envelope exclusive of resources flagged as transfers to other agencies.

Channel codes in the CRS data were used to track DAH to international and donor-country-based non-government organizations. The names of NGOs that were captured in IHME’s NGO data (as detailed in section 3.7 titled “Tracking non-governmental organizations”) were searched for in the CRS descriptive variables and tagged as double-counting. Research funds for HIV/AIDS channeled by the US government through the National Institutes for Health (NIH) were also removed from the total since they do not meet the definition of DAH as contributions from institutions whose primary purpose is development assistance. Official development finance (ODF) from the CRS was not counted because these expenditures were included elsewhere, either in the analysis of multilateral institutions relevant to the study or in the assessment of health spending by BMGF, the data for which were obtained via correspondence and from their annual reports, audited financial statements, and project databases. To avoid double-counting, only health assistance flows from multilateral institutions to low- and middle-income countries were counted, and not transfers to multilateral institutions. Also, for regional projects the disbursements are split amongst all countries in the specified OECD region. For example, a project allocated to recipient “North of Sahara, regional” would have its disbursements split equally between all the countries in the corresponding OECD region: Algeria, Egypt, Libya, Morocco, and Tunisia.

Allocation of funding to health focus areas was assigned as described in the section “DISAGGREGATING BY HEALTH FOCUS AREA”, based on a keyword search of five descriptive variables in the CRS: project title, short

description, long description, channel name, and channel reported name. Additional adjustments were made based on CRS purpose codes, as detailed in Table S3.5, in order to ensure that the specified purpose corresponded to the highest-weighted **bilateral** health focus area.

S3.2.1 Estimating disbursements for the 24 channels and the EC

Both the DAC tables and the CRS rely on information reported by DAC members and other institutions to the OECD-DAC. Hence, the quality of the data varies considerably over time and across donors. Three variables were used to estimate yearly donor disbursements: CRS commitments, CRS disbursements, and DAC commitments. There were two main challenges in using the data from the CRS for this research:

1. underreporting of aid activity to the CRS compared to what is reported to the DAC, and

2. underreporting of disbursement data to the CRS compared to commitment data reported to the CRS.

These issues are highlighted in Figure S3.1. Methods developed to account for both these challenges are discussed below. Details on how we estimated the cost of providing technical assistance and program support for these institutions are highlighted below in the section titled calculating the 'Technical assistance and program support component of development assistance for health from loan-and grant-making channels of assistance'.

To address these two challenges, we determined a cutoff point for each channel. We defined this channel-specific cutoff year as when the ratio of total CRS disbursements to commitments was greater than 50% and did not drop subsequently below 30%. Figure S3.2 below shows each donor's CRS disbursement to commitment ratio in green, and the estimated cutoff year is marked with a vertical red line. For years after the cutoff year, DAH is measured using the unadjusted disbursement data. For the time prior to the cutoff year, it was determined that the disbursement data are not of high enough quality, and adjusted commitments were used instead.

Two adjustments were made to commitments to estimate disbursements before each donor-specific cutoff point:

I. The first adjustment addressed underreporting of aid activity to the CRS (relative to the DAC). To address this challenge, all CRS commitments for the health sector were adjusted upward using the DAC commitment to CRS commitment coverage ratio. The coverage ratio of the CRS was well below 10% before 1996 but has improved steadily over time.

II. The second adjustment addressed underreporting of disbursements data to the CRS (relative to commitments reported to the CRS). To address this challenge, we pooled completed projects in the CRS that have disbursement data for each channel and computed yearly project disbursement rates (the fraction of total commitments disbursed for each year of a project) and overall project disbursement rates (the fraction of total commitments disbursed over the life of each project) by project length. Yearly disbursement schedules were calculated for projects with lengths of one, two, three, four, five, and six years. When an observed project length was more than six years, all expenditure after the sixth year was aggregated and assumed to be expended in the sixth year. This does not happen often. Yearly disbursement rates were the median of these shares, averaged across projects for every donor in each project year. The sum of these averages equals one, so that all the disbursements were expended over the lifetime of a project. We also adjusted donor disbursement schedules by the ratio of disbursements to commitments in the CRS in order to reflect the fact that not all commitments are typically disbursed. The product of these donor-specific yearly disbursement rates, the donor-specific overall disbursement rates, and the donor-specific disbursement to commitment ratios produced the donor-specific disbursement schedules. The donor-specific disbursement schedules were applied to project-level DAC-adjusted commitments reported in the CRS. Figure S3.3 shows the yearly disbursement rates and overall disbursement rates for projects with one- to six-year lifespans for each of the 24 member countries and the EC.

Lastly, to address the challenge of underreporting of aid activity to the CRS compared to the DAC for all years, the difference between each donor's aggregate DAC health commitments and CRS health disbursements was added to

each donor's yearly DAH. Since only aggregate commitments are reported to the DAC, several adjustments were made, based on more detailed CRS data:

I. First, each donor's yearly median project length was calculated by applying the donor-specific disbursement schedules described above to CRS projects that had disbursement in order to get adjusted DAC commitments.
II. Commitments for projects that have not opened yet were then subtracted, based on the open date reporting in the CRS. This ensured that future disbursements were not captured.
III. The donor-specific ratio of disbursements to commitments was applied to adjusted DAC commitments in order to account for the fact that not all commitments are usually disbursed.
IV. Lastly, these DAC-adjusted commitments were compared to CRS disbursements, inclusive of transfers that were later dropped as double-counting.

In addition to tracking disbursements from the EC, gross disbursements from the DAC were used to compile data on the sources of funding for the EC.

Figure S3.1 Comparing CRS commitments, CRS disbursements, and DAC commitments

This figure compares commitments and disbursements from the Creditor Reporting System (CRS) and Development Assistance Committee (DAC) databases of the Development Assistance Committee of the Organisation for Economic Co-operation and Development (OECD-DAC) from 1990 to 2019. CRS disbursements are usually underreported when compared to both CRS and DAC commitments data, especially in earlier years. Because of this gap between CRS and DAC, CRS disbursements data were adjusted to fit DAC commitments data. Drop in 2019 across all commitments and disbursements is due to incomplete USA bilateral data for that year.

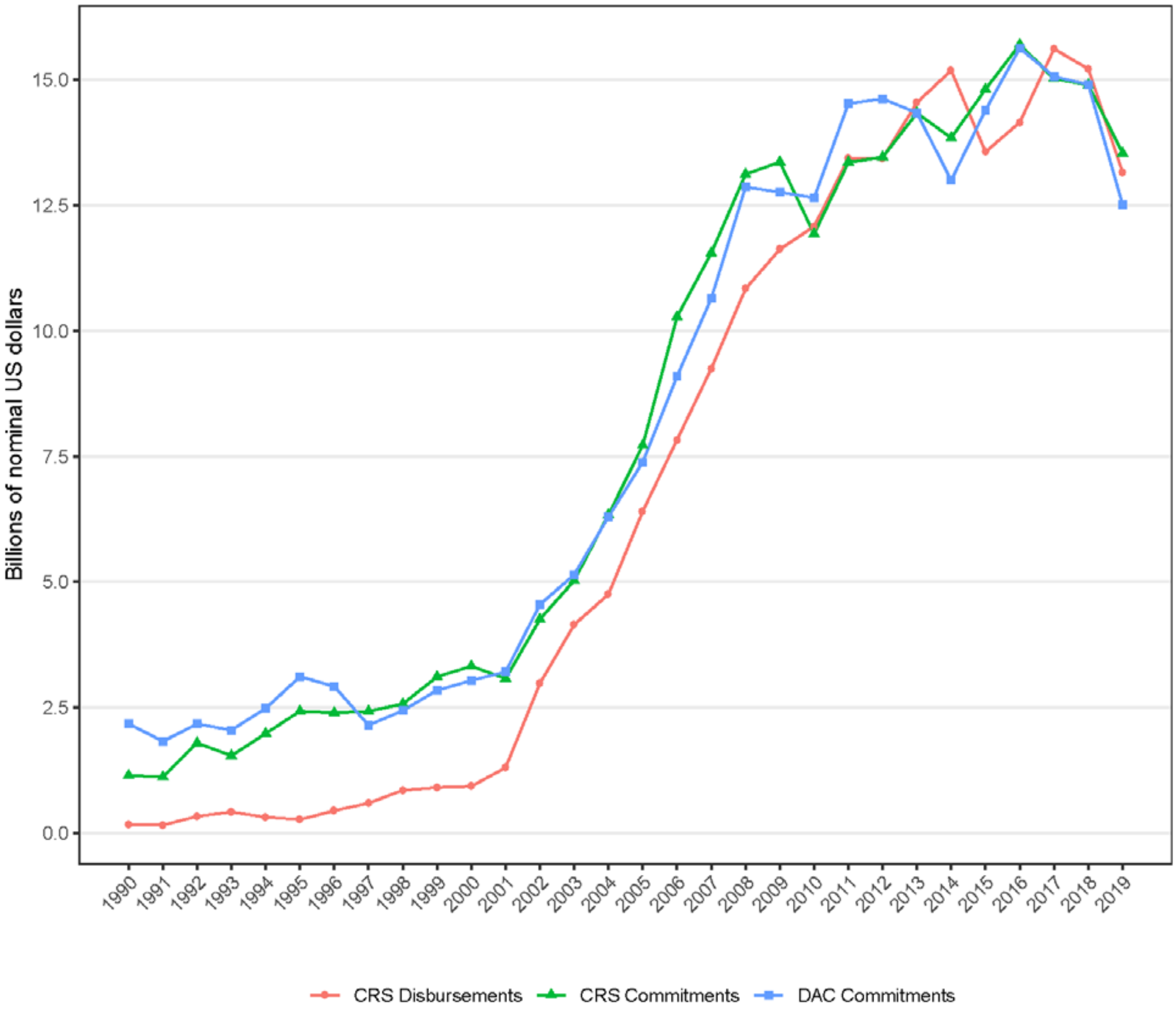

Source: OECD-DAC and OECD Creditor Reporting System

Figure S3.2 CRS disbursement to commitment ratio and cutoff points by donor agency

This figure shows the channel-specific cutoff year. Before this year, we adjust CRS commitments using disbursement schedules. After this cutoff we rely on CRS-reported disbursements. The total CRS disbursements to commitments ratio is in black, and the cutoff year is marked with a vertical red line. The cutoff year is determined to be when the ratio goes above 50% and does not fall back below 30%. The vertical axis represents the CRS disbursement to commitment ratio as a percentage. ARE = United Arab Emirates, AUS = Australia, AUT = Austria, BEL = Belgium, CAN = Canada, CHE = Switzerland, DEU = Germany, DNK = Denmark, EC = European Commission, ESP = Spain, FIN = Finland, FRA = France, GBR = Great Britain, GRC = Greece, IRL = Ireland, ITA = Italy, JPN = Japan, KOR = South Korea, LUX = Luxembourg, NLD = the Netherlands, NOR = Norway, NZL = New Zealand, PRT = Portugal, SWE = Sweden, USA = United States of America.

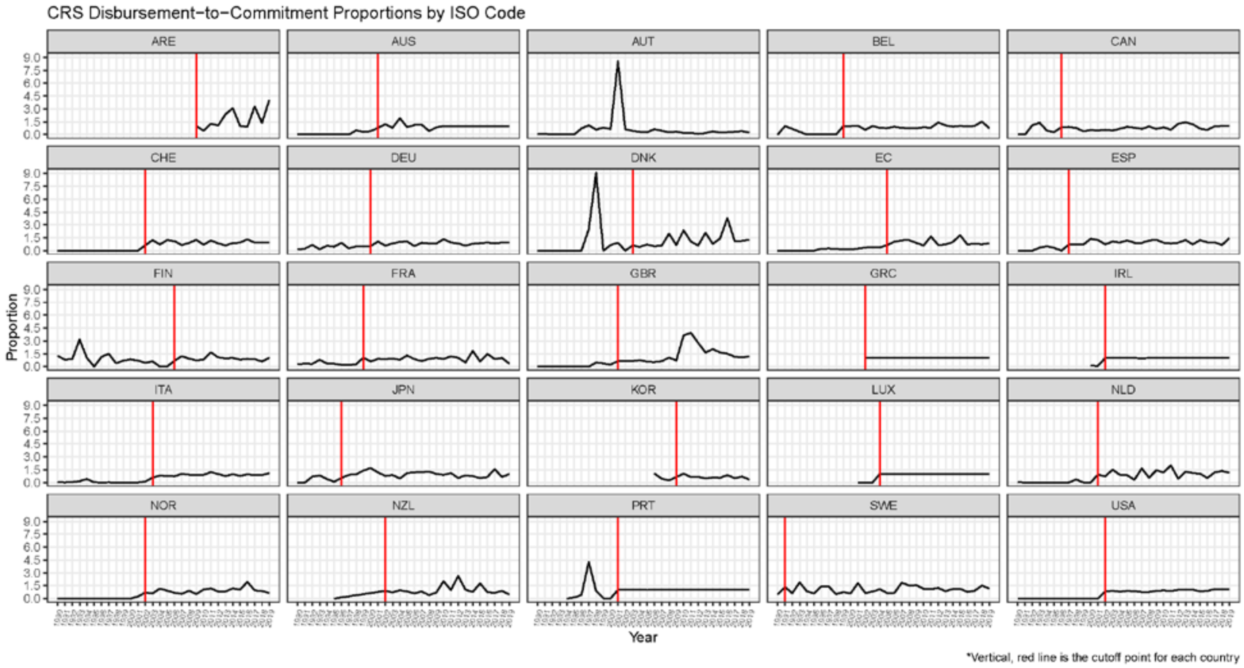

Source: OECD Creditor Reporting System

**Box 3.3. EXAMPLE. Australia's CRS disbursement to commitment ratio and cutoff year**
**The black line shows the ratio of Australia's disbursements to commitments, as reported in the CRS. Prior to 2001, the ratio was always below 50%. In 2001, the ratio rose above 50%; it did not fall below 30% in subsequent years, thereby defining 2001 as the cutoff year. Thus, for Australia, before 2001 DAH is based on adjusted CRS commitment data. These data are adjusted using disbursements schedules (Figure S3.3) and data from the DAC. After 2001, Australia's DAH is based on the disbursements reported in the CRS.**

Figure S3.3 One- to six-year disbursement schedules for bilateral channels

This figure shows the estimated disbursement schedules for bilateral channels. Before the channel-specific cutoff year, we rely on commitment data to inform our estimates of DAH. Commitment data are adjusted to reflect disbursements over time using schedules estimated from projects in the CRS that have both commitment and disbursement data. The vertical axis represents the percentage of the commitment disbursed. ARE = United Arab Emirates, AUS = Australia, AUT = Austria, BEL = Belgium, CAN = Canada, CHE = Switzerland, DEU = Germany, DNK = Denmark, EC = European Commission, ESP = Spain, FIN = Finland, FRA = France, GBR = Great Britain, GRC = Greece, IRL = Ireland, ITA = Italy, JPN = Japan, KOR = South Korea, LUX = Luxembourg, NLD = the Netherlands, NOR = Norway, NZL = New Zealand, PRT = Portugal, SWE = Sweden, USA = United States of America

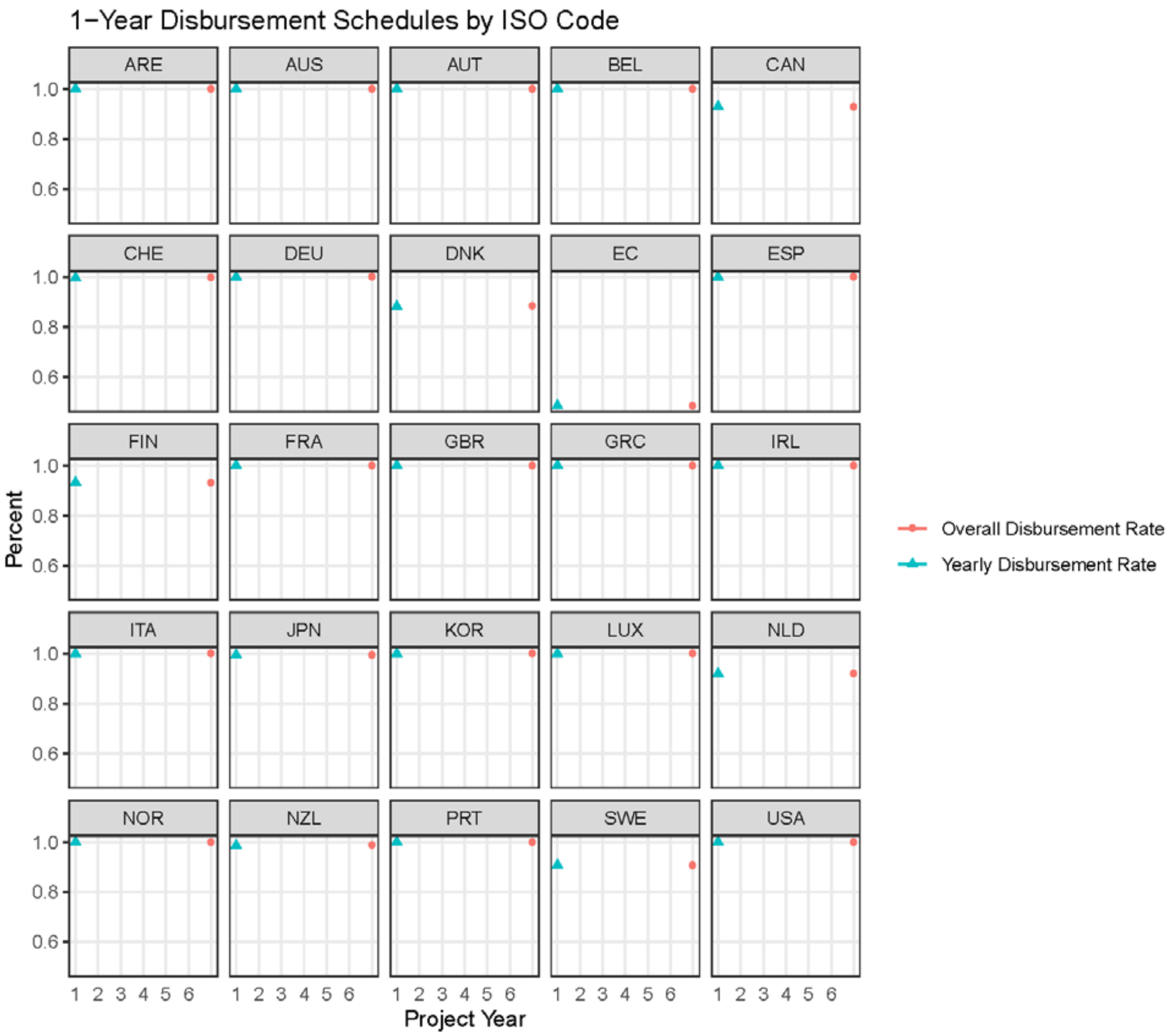

1−Year Disbursement Schedules by ISO Code
ARE
AUS
AUT
BEL
CAN
CHE
DEU
DNK
EC
ESP
FIN
FRA
GBR
GRC
IRL
ITA
JPN
KOR
LUX
NLD
NOR
NZL
PRT
SWE
USA
1.0
0.8
0.6
Percent
1 2 3 4 5 6
Project Year
Overall Disbursement Rate
Yearly Disbursement Rate

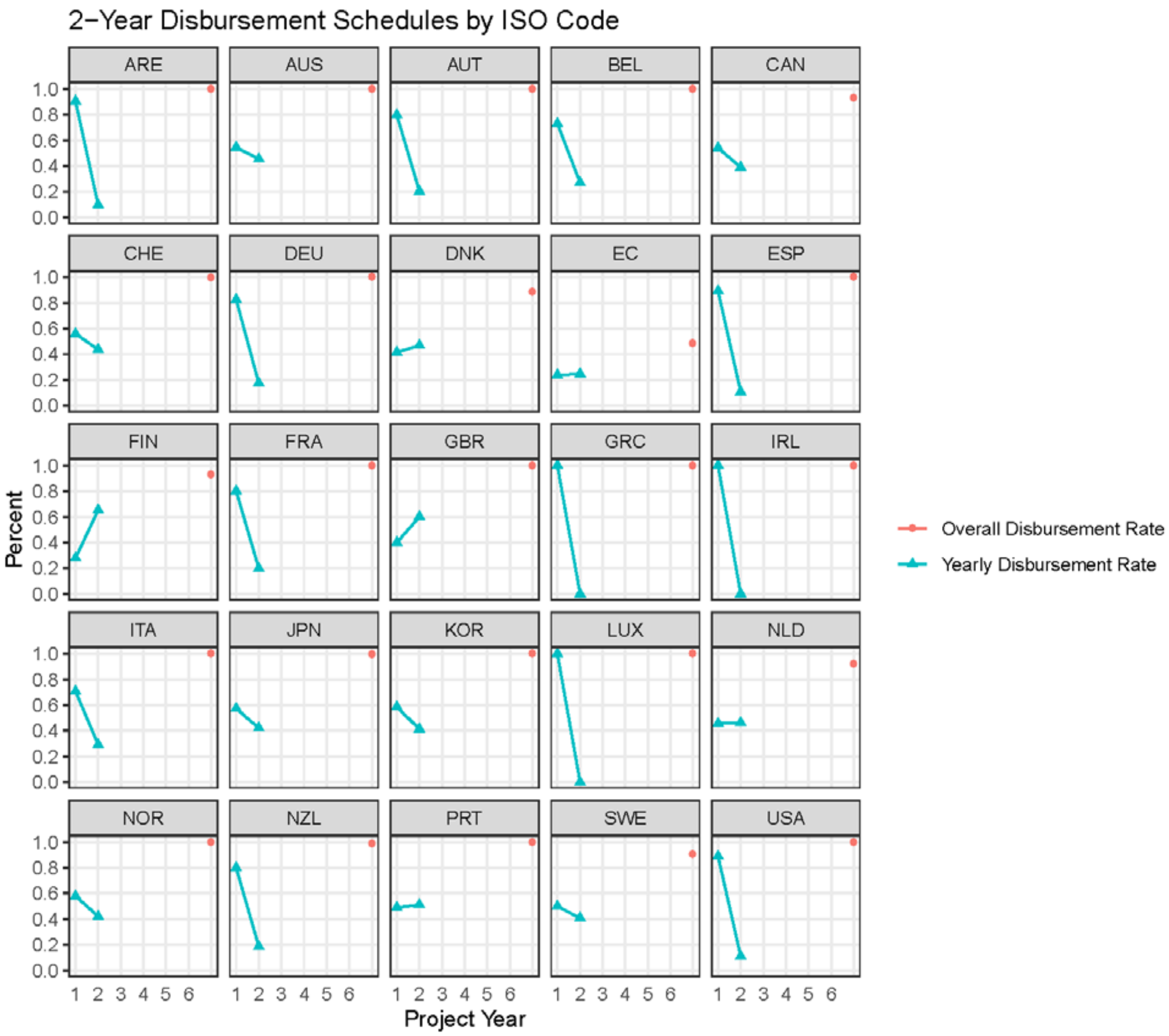

2−Year Disbursement Schedules by ISO Code
ARE
AUS
AUT
BEL
CAN
CHE
DEU
DNK
EC
ESP
FIN
FRA
GBR
GRC
IRL
ITA
JPN
KOR
LUX
NLD
NOR
NZL
PRT
SWE
USA
Percent
1.0
0.8
0.6
0.4
0.2
0.0
1 2 3 4 5 6
Project Year
Overall Disbursement Rate
Yearly Disbursement Rate

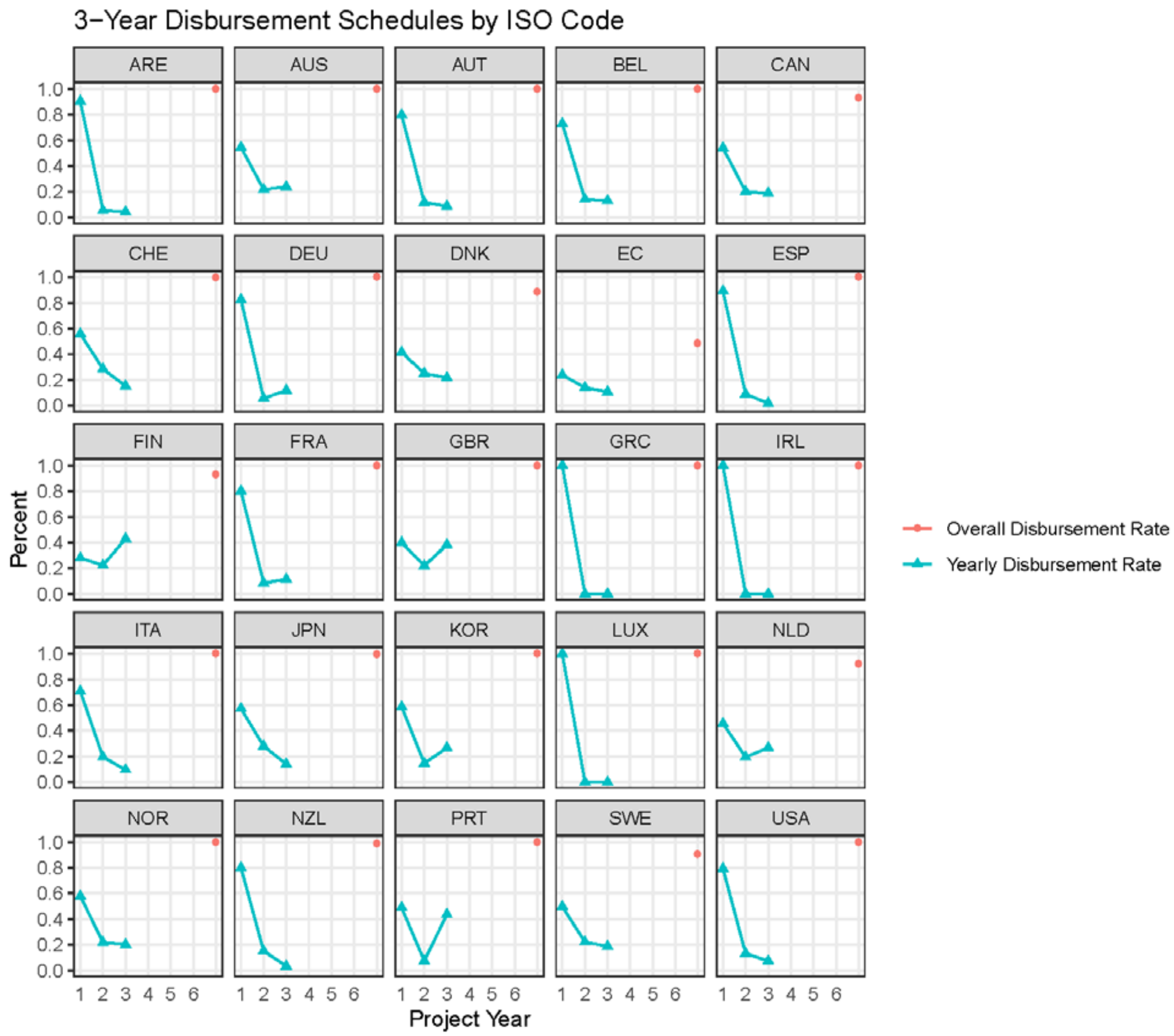
3−Year Disbursement Schedules by ISO Code
ARE
AUS
AUT
BEL
CAN
CHE
DEU
DNK
EC
ESP
FIN
FRA
GBR
GRC
IRL
ITA
JPN
KOR
LUX
NLD
NOR
NZL
PRT
SWE
USA
Percent
1.0
0.8
0.6
0.4
0.2
0.0
1 2 3 4 5 6
Project Year
Overall Disbursement Rate
Yearly Disbursement Rate

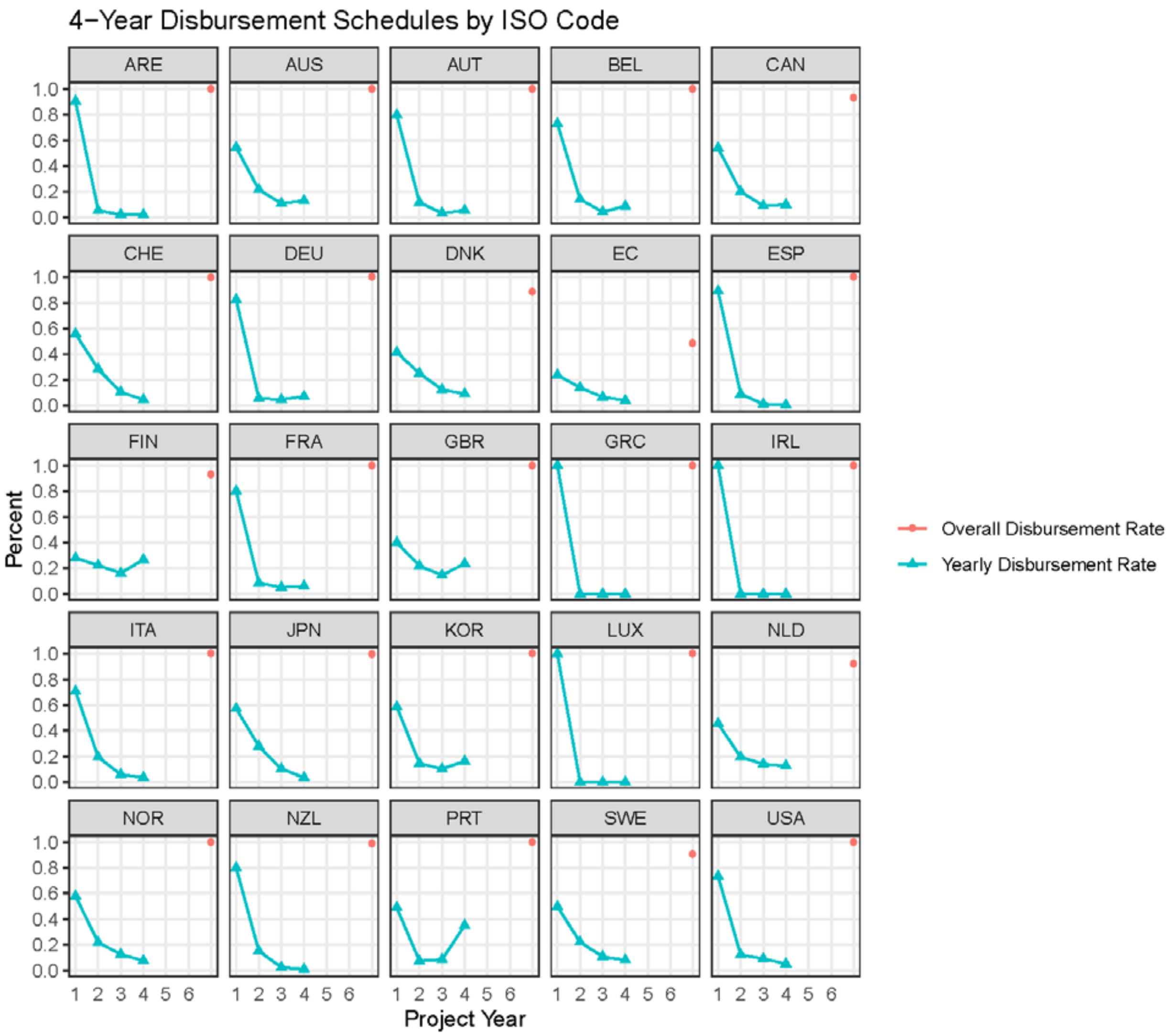

4−Year Disbursement Schedules by ISO Code
ARE
AUS
AUT
BEL
CAN
CHE
DEU
DNK
EC
ESP
FIN
FRA
GBR
GRC
IRL
ITA
JPN
KOR
LUX
NLD
NOR
NZL
PRT
SWE
USA
Percent
0.0
0.2
0.4
0.6
0.8
1.0
1 2 3 4 5 6
Project Year
Overall Disbursement Rate
Yearly Disbursement Rate

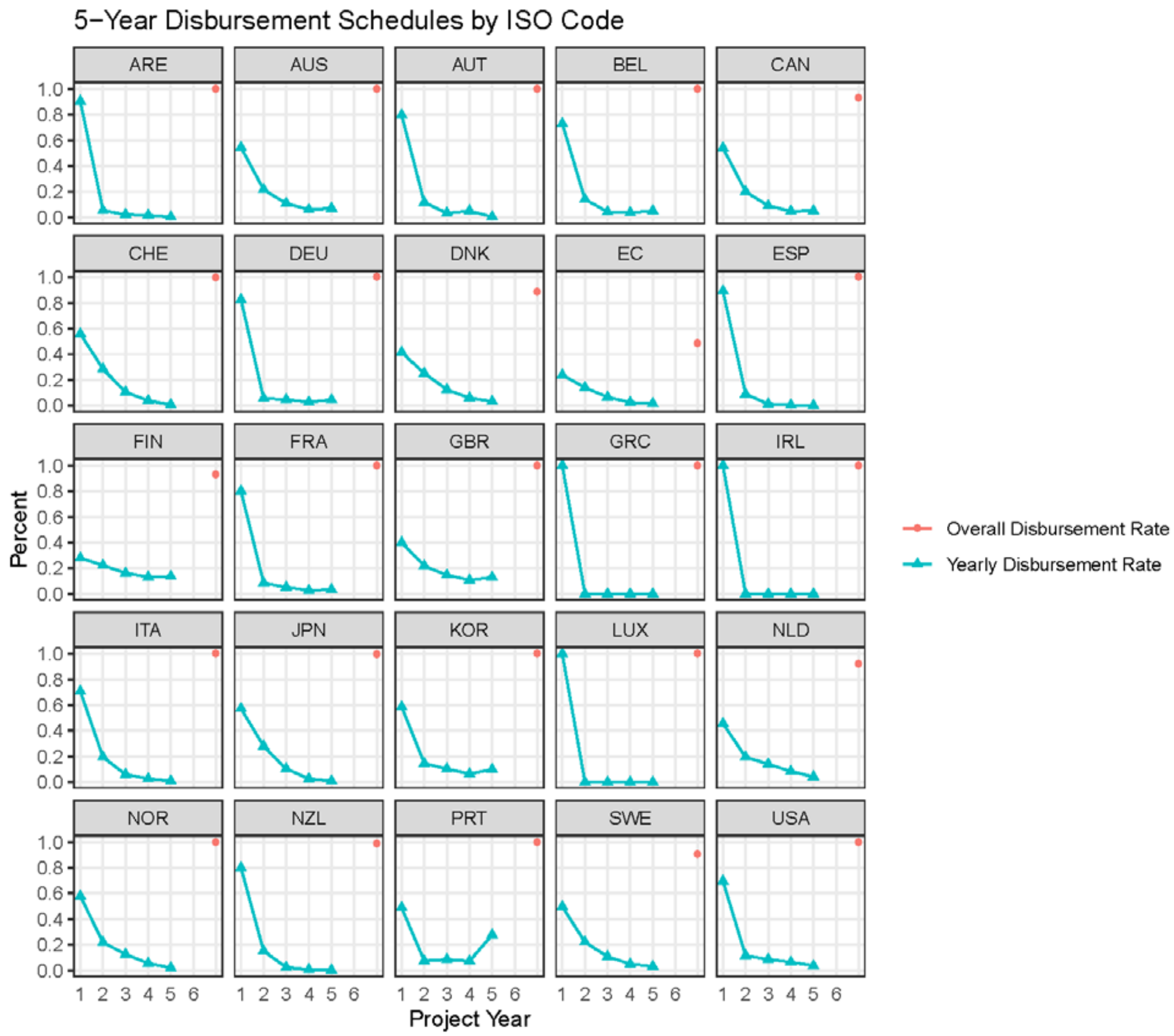
5−Year Disbursement Schedules by ISO Code
ARE
AUS
AUT
BEL
CAN
CHE
DEU
DNK
EC
ESP
FIN
FRA
GBR
GRC
IRL
ITA
JPN
KOR
LUX
NLD
NOR
NZL
PRT
SWE
USA
Percent
Project Year
Overall Disbursement Rate
Yearly Disbursement Rate

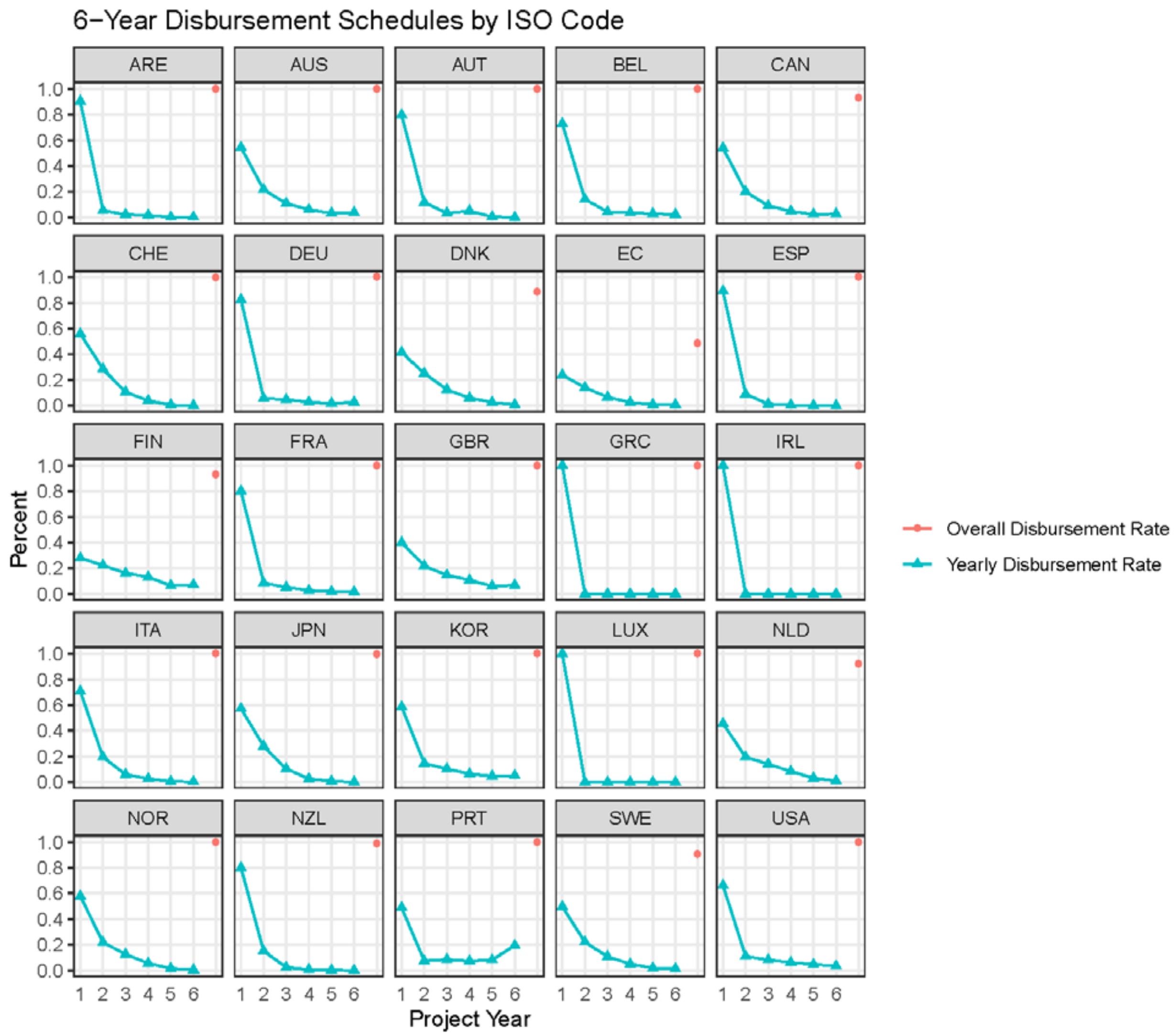
6–Year Disbursement Schedules by ISO Code
ARE
AUS
AUT
BEL
CAN
CHE
DEU
DNK
EC
ESP
FIN
FRA
GBR
GRC
IRL
ITA
JPN
KOR
LUX
NLD
NOR
NZL
PRT
SWE
USA
Percent
Project Year
Overall Disbursement Rate
Yearly Disbursement Rate

Box 3.4. EXAMPLE. Australia's one- to six-year disbursement schedules
**To estimate disbursements using commitment data, we rely on disbursement schedules derived from CRS data that include both commitments and disbursements. Disbursement schedules are specific for each channel and the length of a project. These schedules also take into consideration the average amount of commitments for each channel that lead to disbursements. Across all Australian projects in the CRS with complete disbursements data, Australia disbursed 100% of the funds that it committed, as shown by the solid red dot on the right-hand side of the Australia panel (upper left corner of the first panel of Figure S3.3). In projects with a length of one year, Australia disbursed 100% of the funds that it committed in that year. For two-year projects, Australia disbursed 50% of total disbursements in year one and 50% of total disbursements in year two. In projects with lengths of three years, Australia disbursed about 50% of total disbursements in year one and 23% and 27% of total disbursements in years two and three, respectively. This is estimated for projects ranging from one to six years. The disbursement schedules were applied to commitment data from the CRS to estimate disbursements for years prior to the cutoff year, which is 2001 for Australia.**

To predict DAH for the recent years not reported in the CRS, budget data were extracted from a variety of sources. These data are listed in Table S3.3. Global health budgetary data were utilized whenever possible, but these detailed data were available as a complete time series only for Australia and the United States. For all other bilateral channels, general ODA budgets were used. In order to predict DAH for 2019-2020 for 24 bilateral agencies, the budget ratio for each donor was calculated by dividing DAH estimates by the corresponding budget data (ODA or global health). Budget ratios were projected using a weighted average of the previous three years (placing one-half weight on the one-year lagged ratio, one-third weight on the two-year lagged ratio, and one-sixth weight on the three-year lagged ratio), and this ratio was multiplied by the observed budgeted DAH for those same years. Figure S3.4 plots the budget ratio for each bilateral channel. Budget data for the EC were inconsistent and did not match the disbursement series. Instead, DAH for 2019-2020 was estimated based on trends in DAH for EC member countries. A weighted average was applied to the percent change in DAH from 2016-2018 for all EC member countries. The weighting was based on each country's total national contributions to the EC. These data were collected from the EC's financial statements.[74] The weighted average was then applied to the EC's 2018 DAH to forecast 2019-2020.

Figure S3.4 DAH as a percentage of corresponding budget data by bilateral agency

This figure shows the trend of the ratio of DAH measured as a share of budget data. Green dots indicate that a donor provided global-health-specific budget data, so in these cases the denominator is all global-health-specific budgeted data. The numerator is estimated DAH. Red dots indicate that a donor did not have global-health-specific budget data, so overall ODA budget data were used in calculating the DAH to budget ratios. The vertical axis represents estimated DAH as a fraction of corresponding budget data. ARE = United Arab Emirates, AUS = Australia, AUT = Austria, BEL = Belgium, CAN = Canada, CHE = Switzerland, DEU = Germany, DNK = Denmark, ESP = Spain, FIN = Finland, FRA = France, GBR = Great Britain, GRC = Greece, IRL = Ireland, ITA = Italy, JPN = Japan, KOR = South Korea, LUX = Luxembourg, NLD = the Netherlands, NOR = Norway, NZL = New Zealand, PRT = Portugal, SWE = Sweden, USA = United States of America.

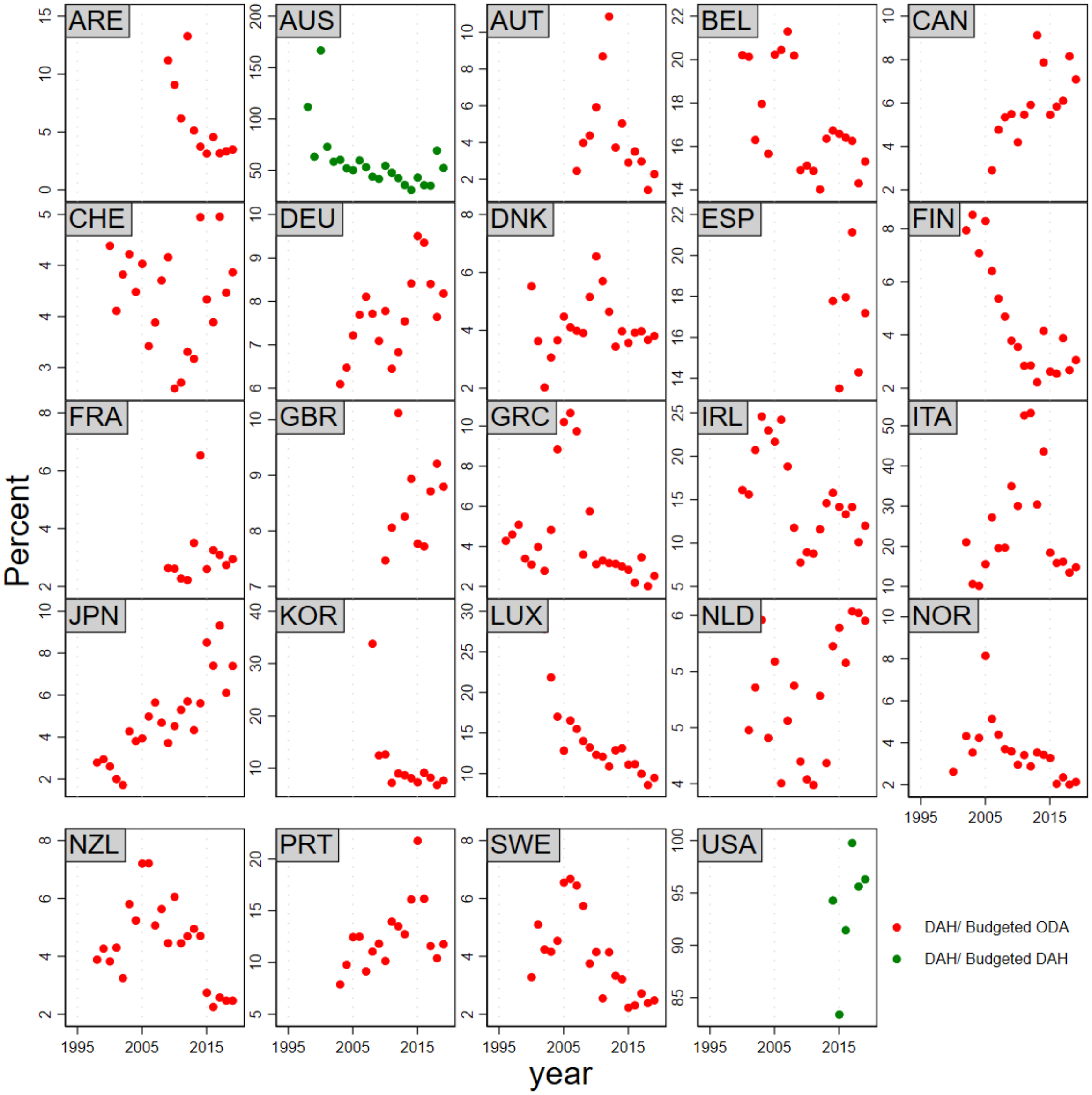

Source: IHME DAH Database (2020) and corresponding bilateral ODA/DAH budget documents outlined in Table S3.1 and 3.3.

**Box 3.5. EXAMPLE. Australia's DAH as a percentage of corresponding budget data**
Australia provided global-health-specific budget data for 1998-2020 through its International Development Assistance and Overseas Aid Program budgets. For 1998-2018, health ODA and observed DAH were used to create DAH to budget ratios. These budget ratios were then applied to 2019-2020 health ODA budget data to project DAH in 2019-2020, using a weighted average: $(Total\ DAH_t) = \left(\frac{1}{2}\right)(Budget\ ratio_{t-1})\ (Budgeted\ GHE_t)\ + \left(\frac{1}{3}\right)(Budget\ ratio_{t-2})(Budgeted\ GHE_t) + \left(\frac{1}{6}\right)(Budget\ ratio_{t-3})(Budgeted\ GHE_t)$ where t = year to be modeled.

To supplement our estimates of development assistance for health to HIV/AIDS and malaria program areas for the United States, we used additional available data from the President's Emergency Plan for AIDS Relief (PEPFAR) and the President's Malaria Initiative (PMI). We downloaded data on all planned funding by PEPFAR by recipient country, year, and program area from 2004 to 2020.[75] All PEPFAR projects were assigned to our eight HIV/AIDS program areas using PEPFAR budget codes, splitting out overhead costs equally to all other program areas. We then created country-year specific HIV/AIDS program area fractions out of total annual HIV/AIDS DAH, which we applied to all United States HIV/AIDS projects in the CRS from 2004 to 2018 by country-year. To inform malaria funding by program areas, we downloaded the most recently available malaria funding tables from malaria operational plans for all countries and years.[76] We used a keyword search to assign each line item in these tables to our eight malaria program areas, and then created fractions for the malaria program areas out of the total annual malaria DAH specific to each country-year. These fractions were applied to all United States malaria projects in the CRS from 2006 to 2018 by country-year.

Figure S3.5 Malaria DAH to program areas as assigned by keyword search and PMI reports

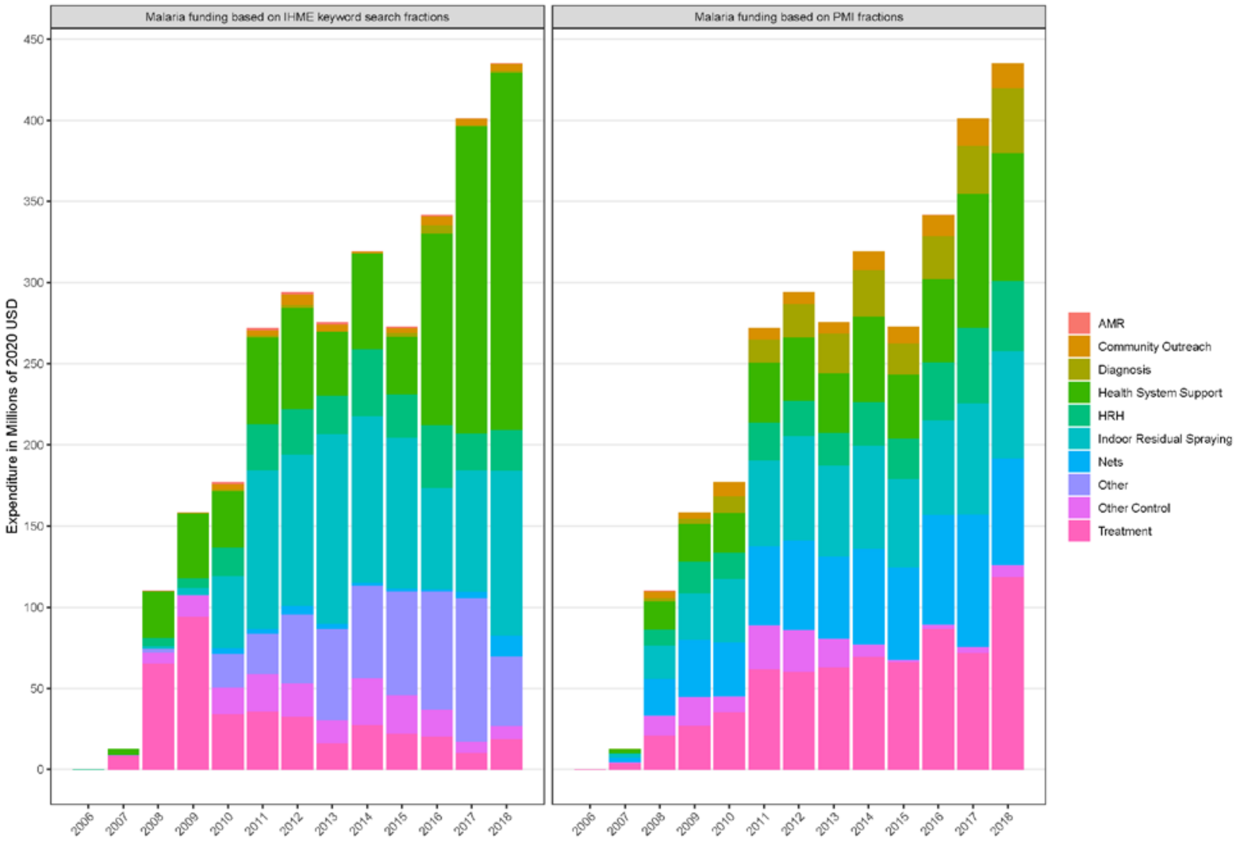

Source: IHME DAH Database (2020) and PMI malaria operational plans

This figure outlines the assignment of funding to malaria program areas for United States projects from the OECD CRS from 2006 onwards. The figure on the left shows how malaria funding is broken out based on keyword search. The figure on the right shows the breakdown of funding to malaria program areas based on PMI malaria funding tables. Using the data from PMI reduces the amount of funding allocated to the "other" program area. As such, in as often as more disaggregated information on project allocation is available, IHME uses such project information available in project budget documents or other project documents to disaggregate into program areas.

S3.3 Tracking development assistance for health from the development banks

The World Bank project-level health disbursement data for 1990 through December 2020 were obtained through correspondence with Kathleen E. Krackenberger and Miyuki T. Parris, Operations Analysts.[77] The World Bank recently underwent a recoding process for their disbursements. This recoding affected health disbursements, however the recoding was not completed for projects with disbursements prior to 2001. To create a comparable dataset adjustments had to be made. Regression analysis to predict health disbursements were explored, however, in the end the average percent change between project-level health disbursements before and after recoding was used to adjust health disbursements prior to 2001. It was observed that on average, between 2001 and 2005 (inclusive) the recoding process decreased health disbursements by 0.22%. This number was used to adjust all project-level health disbursements prior to 2001.[78] Health disbursements included all health projects as well as other sector projects with a health sector code. In addition, data were collected from the World Bank online loans database in order to fill in descriptive information for loans from the two arms of the World Bank: the International Development Association (IDA) and the International Bank for Reconstruction and Development (IBRD).[79] Along with keyword searches, health theme codes were used to allocate disbursements by health focus area. The online database contains up to five sector codes and five theme codes that can be assigned to each project. Sector codes represent economic, political, and social subdivisions, while theme codes represent the goals or objectives of World Bank activities. The codes are summarized in Table S3.6. Emergency recovery loans were excluded since they do not fit the definition of DAH.

Table S3.6 World Bank's health sector and theme codes

**Health sector codes**<br>**Sector codes represent economic, political, or social subdivisions within society. World Bank projects are classified by up to five sectors.**	**Health theme codes**<br>**Theme codes represent the goals or objectives of World Bank activities.**
**Historic (prior to 2001):**<br>(1)**Basic health**<br>(2)**Other population health and nutrition**<br>(3)**Targeted health**<br>(4)**Primary health, including reproductive health, child health, and health promotion**<br><br>**Current (as of 2001):**<br>**(1) Health**<br>**(2) Compulsory health finance**<br>**(3) Public administration – health**<br>**(4) Noncompulsory health finance**<br>**(5) Health facilities and construction**	Current:<br>(1) HIV/AIDS<br>(2) Malaria<br>(3) Tuberculosis<br>(4) Other communicable diseases<br>(5) Population and reproductive health<br>(6) Child health<br>(7) Nutrition and food security<br>(8) Injuries and non-communicable diseases<br>(9) Health system performance<br>(10) Social analysis and monitoring

Data on yearly government contributions were obtained from the DAC statistics in order to disaggregate IDA flows by source. Because China does not report to DAC, we generated contribution estimates using replenishment data. We split the 3-year replenishment amount over the three years of the 16th-19th replenishment, to obtain China's contributions to the World Bank from 2008-2018. We validated our use of replenishment data by extracting China's and other countries' contributions to the World Bank International Development Association (IDA) 16th, 17th and 18th replenishment and comparing the number with the contribution we extracted from the OECD Creditor's Reporting System (CRS)[80]. The trend of contribution from major donors was similar, we therefore used China's replenishment contribution as a proxy of the contribution to World Bank IDA. We further disaggregated the DAH contribution to World Bank IDA from China by multiplying China's contributing proportion over World Bank IDA's total health envelope.

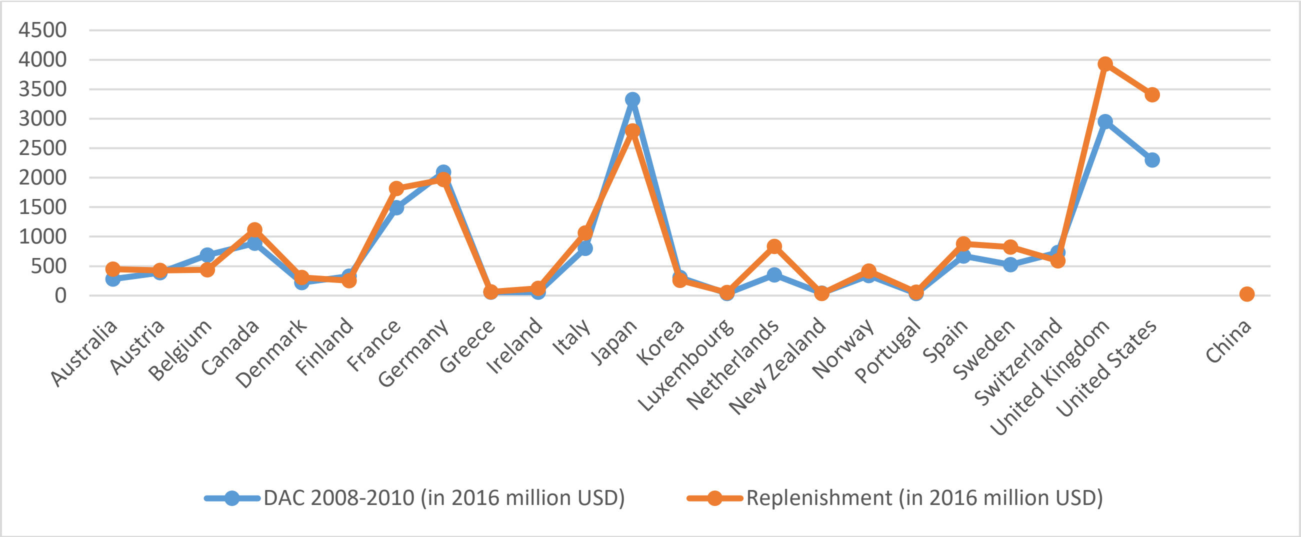

Panel a. Comparison of World Bank International Development Association 16th Replenishment

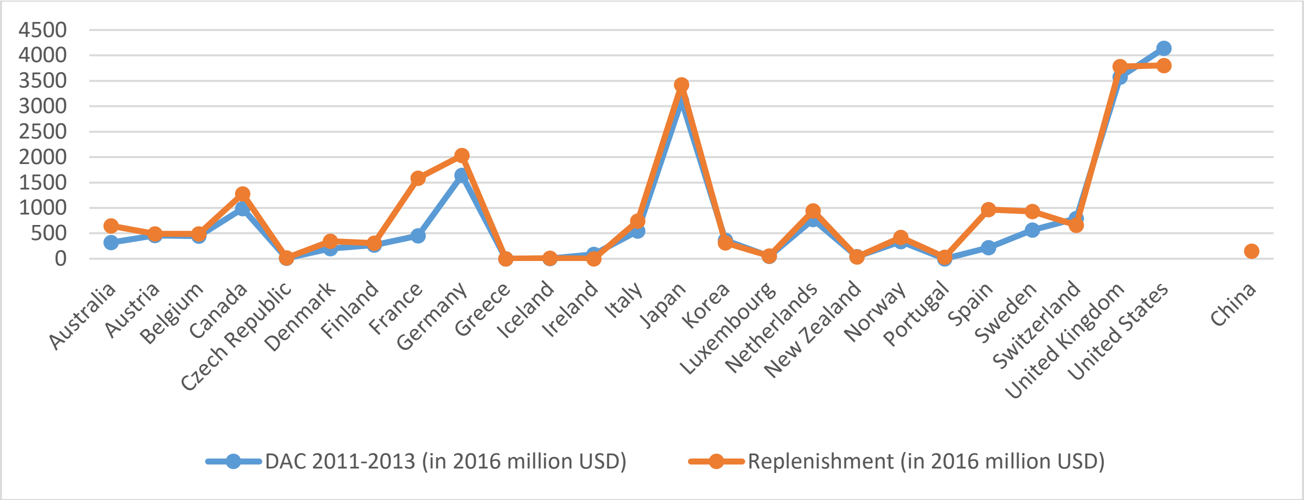

Panel b. Comparison of World Bank International Development Association 17th Replenishment

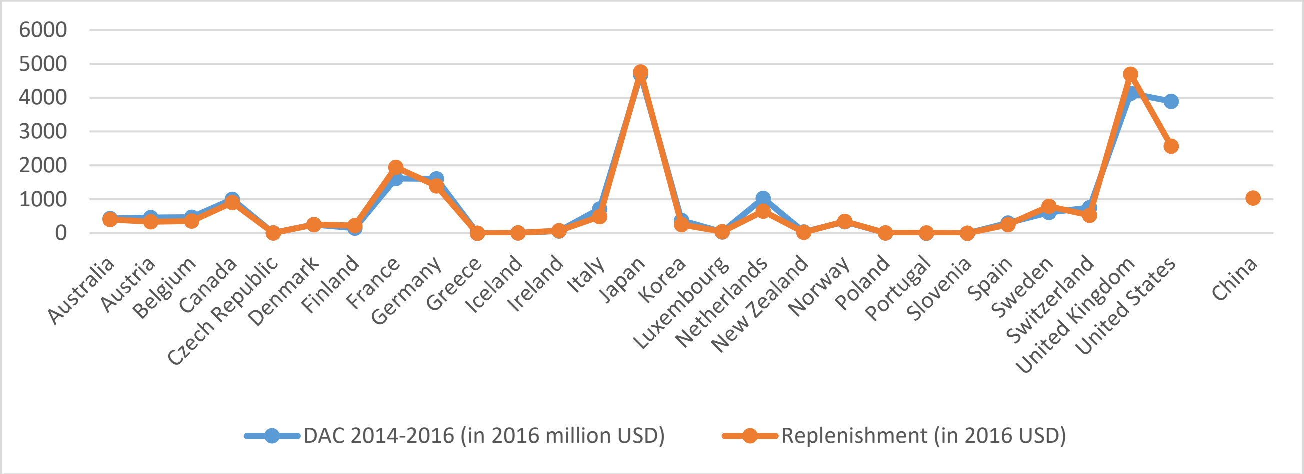

Panel c. Comparison of World Bank International Development Association 18th Replenishment

Figure S3.6 Comparison of World Bank IDA Replenishment and DAC

The three figures show the comparison of country commitment to World Bank IDA replenishment rounds, and yearly government contributions reported to DAC statistics. The orange line shows the country commitment to each IDA replenishment round. The blue line shows the sum of yearly reported contribution during the three-year replenishment round. Panel a is contributions and commitments from replenishment round 16; panel b is contributions and commitments from replenishment round 17; panel c is contributions and commitments from replenishment round 18.

Details on how we estimated the cost of providing technical assistance and program support for these institutions are highlighted below in the section titled "Calculating the technical assistance and program support component of development assistance for health from loan-and grant-making channels of assistance."

S3.3.1 Regional development banks

The Asian Development Bank (ADB) and Inter-American Development Bank (IDB) maintain their own loan databases, which we used to estimate disbursements.[15,16,81] To estimate health disbursements from the African Development Bank (AfDB), data was received via correspondence with Mr. Yomi Ayodabo (Division Manager, Loan accounting) .[35] To estimate health disbursement from the Asian Development Bank (ADB), data were received via correspondence with Dr. Rouselle F. Lavado. Table S3.7 provides a summary of the data sources used across the regional banks. Furthermore, Figure S3.6 displays the disbursements for AfDB from 1990 to 2020 and eFigures 3.7, and 3.8 display commitments and disbursements from 1990 to 2020 for ADB and IDB.

For AfDB, we received project level disbursement data from 2001 through 2020. For pre-2001 estimates, data from the Compendium of Statistics were used. Donor information for projects funded by the African Development Fund was based on subscription information from the 9th to 14th replenishment reports[82]. The sole donor to projects implemented by the Nigeria Trust Fund was Nigeria, and all income for African Development Bank projects was attributable to other sources.

For ADB, we received project-level disbursement data from 1990 through 2020, including recipient, project title and description and annual disbursement. A keyword search was performed to identify health focus areas of the projects. For income source of ADB, data for country donations were extracted from the replenishment reports of Asian Development Fund and we manually divide the four or five years' replenishment into annual contributions[83]. We extracted the total revenue of Ordinary Capital Resources and Asian Development Fund from the Financial reports of ADB to generate the total envelope of income sources and subtracted the country contribution to provide the estimate for resources coming from the bank itself. Using the fraction for the Bank's own resources and country contribution, we provided the estimate of income source for each project.

The IDB's project database also provided commitments and disbursements for all projects. The same methods were used for estimating annual disbursements from the IDB as were used for the ADB. Through correspondence, 2020 IDB health loan disbursements were obtained in October 2020. These were used for the 2020 estimates. However, since these channels have so few new projects each year, we assume that smoothing disbursements over time for reported projects captures the majority of total disbursements for 2020. The contribution quotas by donor for the Fund for Special Operations from 1990-2016 were extracted from Annual Financial reports for the IDB. The ratio of each donor's contribution to total contribution was applied to total disbursements to assign income sources. In 2017, the Fund for Special Operations was transferred to the Ordinary Capital. Donor assignments from 2017 onwards are unallocable.

Table S3.7 Summary of data sources for the regional development banks

This figure indicates the data available and used to estimate DAH. (X) indicates that project-level data are present in the dataset. (-) indicates that project-level data are not present in the dataset.

Institution	Data source	Commitments	Cumulative disbursements	Yearly disbursement	Notes
**African Development Bank (AfDB)**	Compendium of Statistics	X	-	(Aggregate - not at the project level)	The Compendium of Statistics was not available for 1990-1993, 1995, 1998-1999, or after 2002; we estimated yearly disbursements using the average of neighboring disbursements
	Correspondence	-	-	X	Annual loan disbursements from 2001 through December 2020
**Asian Development Bank (ADB)**	Correspondence	X	X	X	Annual grant loan disbursements from 1990 through 9 December 2020
**InterAmerican Development Bank (IDB)**	Online projects database	-	X	-	As yearly disbursement amounts are not provided in the online database, we estimated yearly disbursements by allocating cumulative disbursements over each year of the project.
	Correspondence	-	-	X	Loan disbursements from January through 5 October 2020

Figure S3.7 Disbursements by the African Development Bank

The orange line with squares shows estimated disbursements based on the Compendium of Statistics from 1990 through 2001 and actual disbursements received from 2001 onwards.

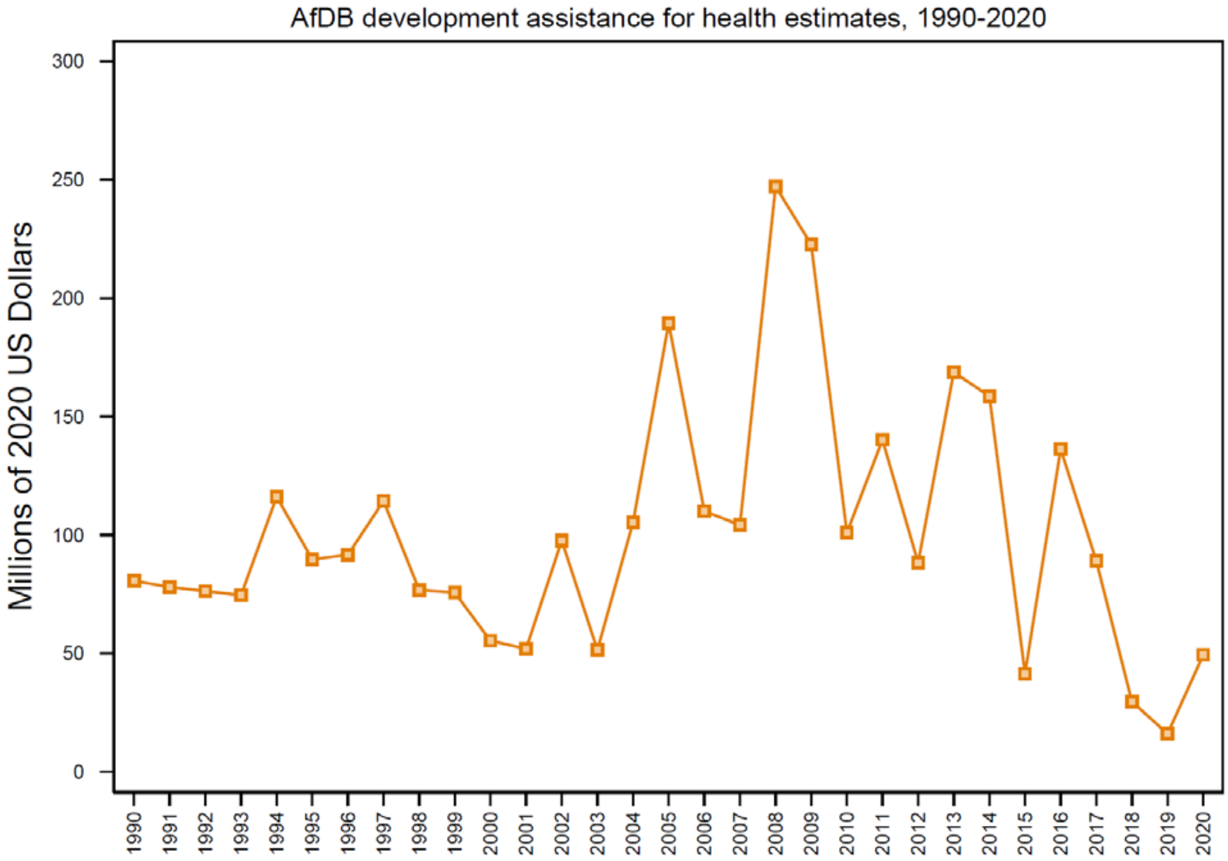

Source: IHME DAH Database (2020) and African Development Bank Compendium of Statistics.

Figure S3.8 Disbursements by Asian Development Bank

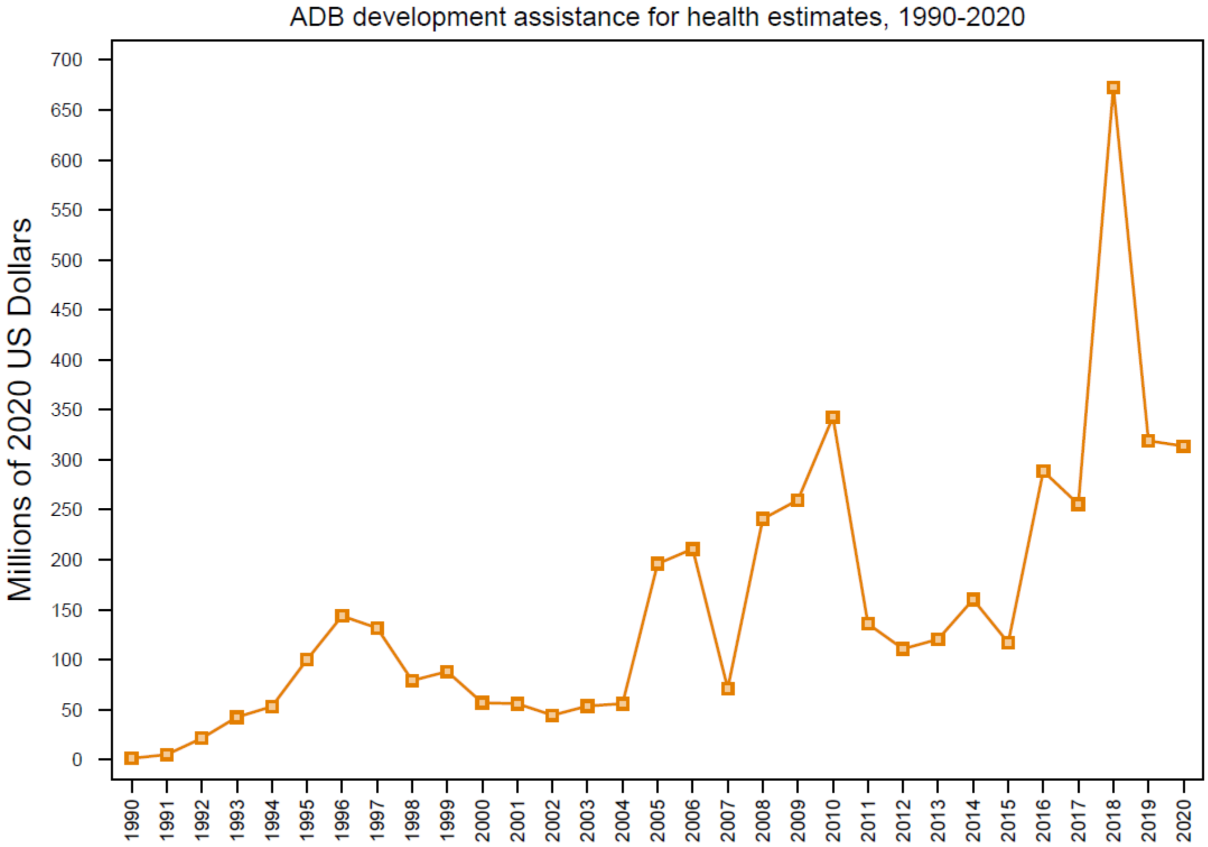

Source: IHME DAH Database (2020)

Figure S3.9 Disbursements by Inter-American Development Bank

The black line shows total disbursements from the Inter-American Development Bank's (IDB) online projects database. The blue line shows disbursements excluding administrative expenses. This data comes from the online projects database and from correspondence for 2020.

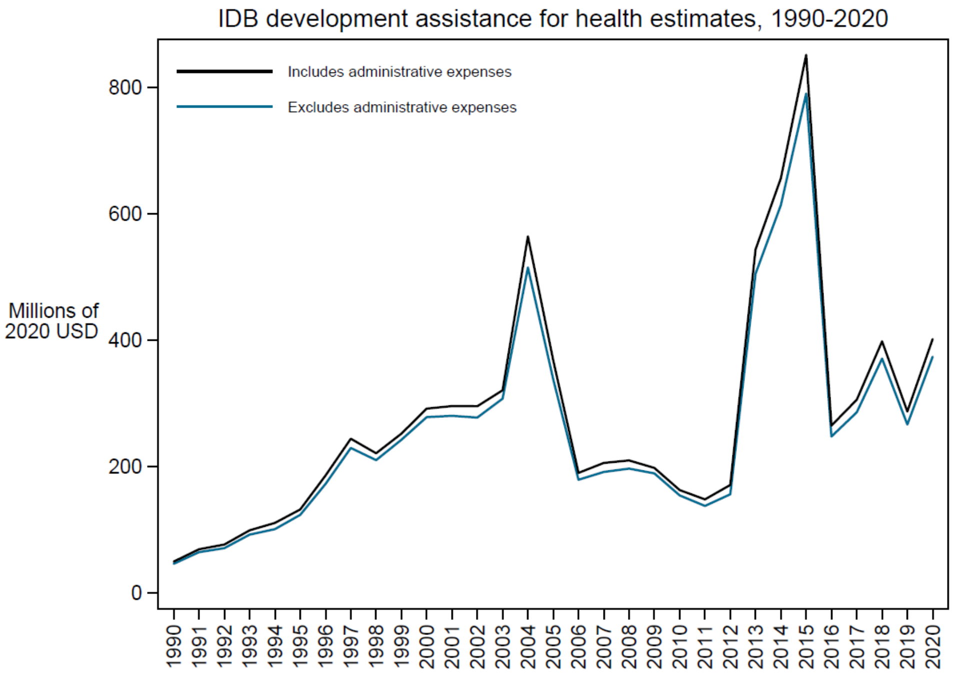

Source: IHME DAH Database (2020) and correspondence

S3.4 Tracking contributions from THE GLOBAL FUND and GAVI

The Global Fund to Fight AIDS, Tuberculosis and Malaria

The grants database made available online by the Global Fund to Fight AIDS, Tuberculosis and Malaria (THE GLOBAL FUND) provides grant-level commitments and annual disbursements from its inception in 2002 to the present year.[22] In addition, sources of funding were compiled from the THE GLOBAL FUND contributions dataset and annual reports, all downloaded from the THE GLOBAL FUND website.[23,24] Regional grants were split evenly between all countries identified in the regional grant documents found on the THE GLOBAL FUND website. Figure S3.10 shows THE GLOBAL FUND's annual contributions received from public and private sources. Figure S3.10 shows THE GLOBAL FUND's annual commitments and disbursements from its project database from 2002 through 2020.

Figure S3.10 Contributions received by the Global Fund to Fight AIDS, Tuberculosis and Malaria

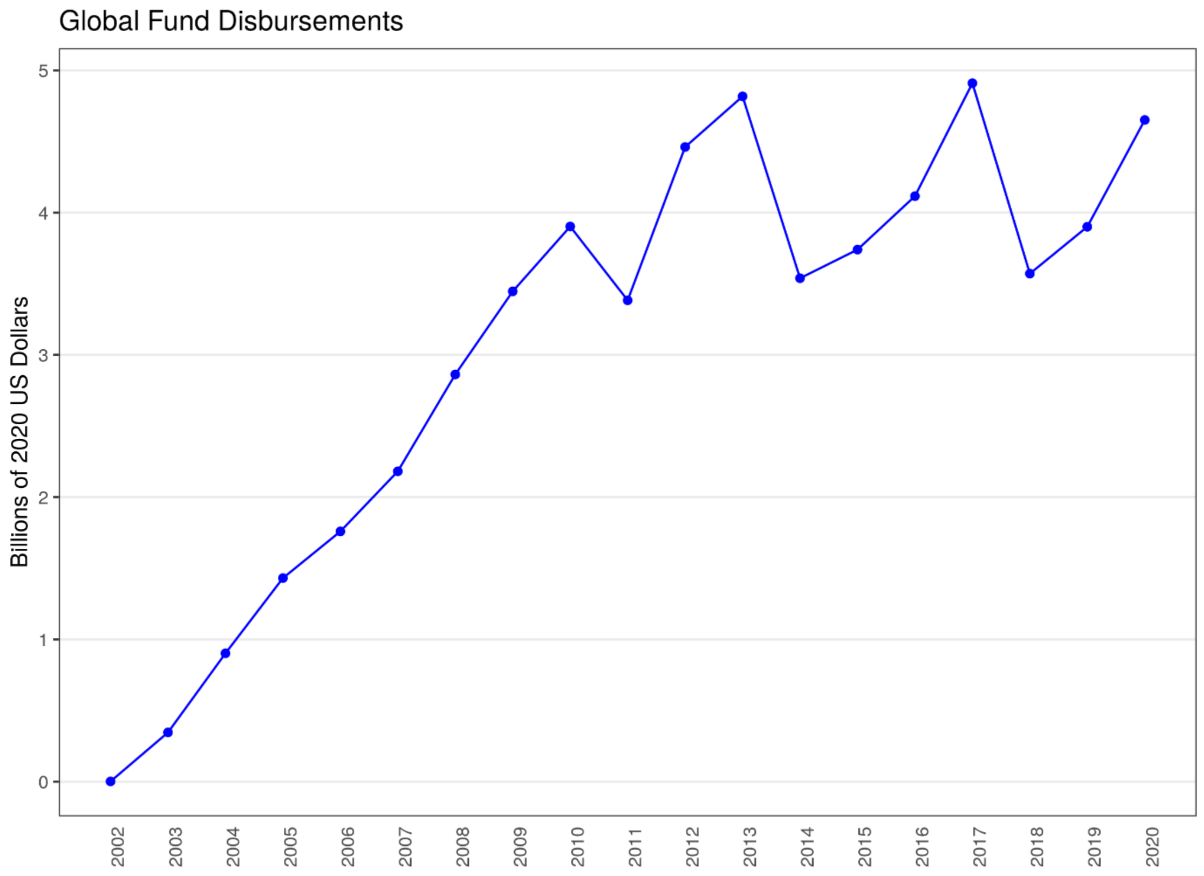

Source: THE GLOBAL FUND pledges and contributions 2020

Figure S3.11 The Global Fund to Fight AIDS, Tuberculosis and Malaria's commitments and disbursements

The dashed green line shows commitments from the Global Fund to Fight AIDS, Tuberculosis and Malaria's (THE GLOBAL FUND) online grants database. The orange line shows disbursements from the online grants database.

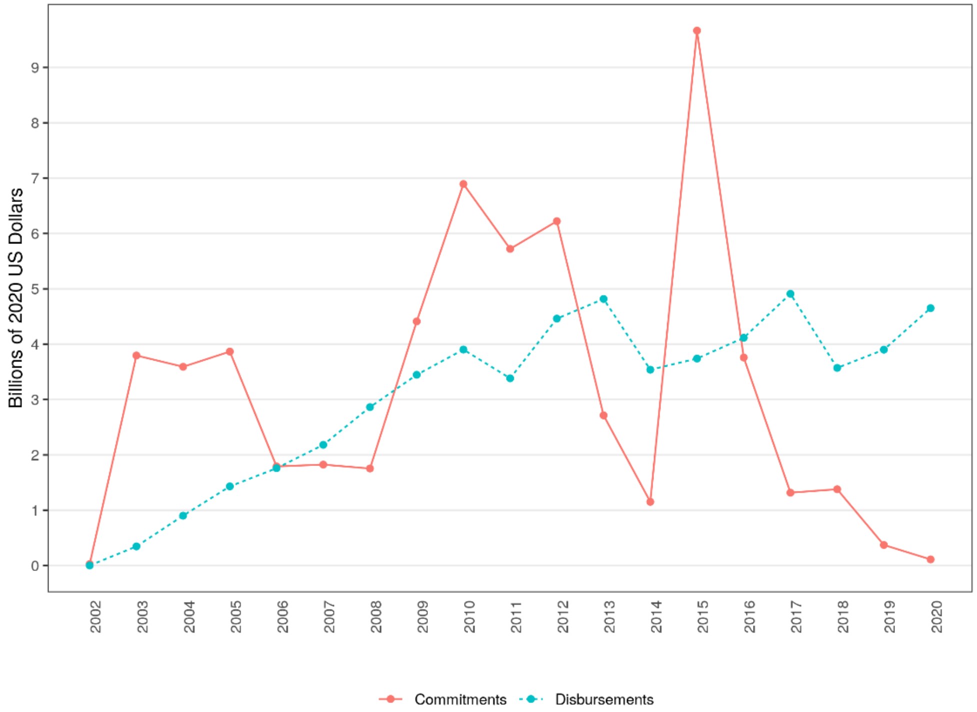

Source: IHME DAH Database (2020)

S3.4.1 Gavi, the Vaccine Alliance

Gavi provided publicly available project-level data on commitments, disbursements, and investment cases from 2000 through the present.[18,21] Gavi's annual DAH was defined as the sum of (1) project-level disbursements by year paid; (2) investment cases (one-time investments in disease prevention and control); and (3) administrative and work plan costs. Data from Gavi's online databases include expenditure for (1) and (2), but not (3). However, project level data from the CRS for 2007-2012 did include administrative and work plan costs, so disbursements data from the online database were adjusted to match the CRS in those years. The average fraction of administrative and work plan costs was added to total disbursements in 2000-2006 and 2013-2015, the years in which the CRS did not include these data. Contributions data from Gavi's website as well as annual reports from the International Finance Facility for Immunisation (IFFIm) and Advance Market Commitment for Pneumococcal Vaccines were used to determine Gavi's annual income.[19,20,84]

All of the data sources used for Gavi estimates were complete through 2019. Donor contributions received and outstanding pledges data were available on Gavi's website. A linear model of annual disbursements to pledges was used as total disbursements for 2020. Gavi disbursements were assigned to health focus areas including child and newborn vaccines, HSS, and non-communicable disease as documented in Table S3.7 above. Of note, we reclassified all GAVI health system strengthening projects as maternal, newborn, child health specific health system strengthening disbursements

Figure S3.12 Gavi's income and disbursements

The dashed green line shows commitments from Gavi's online database. The dashed orange line shows the disbursements from Gavi's online database, which are the sum of project-level disbursements and investment cases.

These data are adjusted using Gavi expenditure data reported to the Creditor Reporting System (CRS) to add administrative and work plan costs to the total. Adjusted disbursements are shown by the solid orange line.

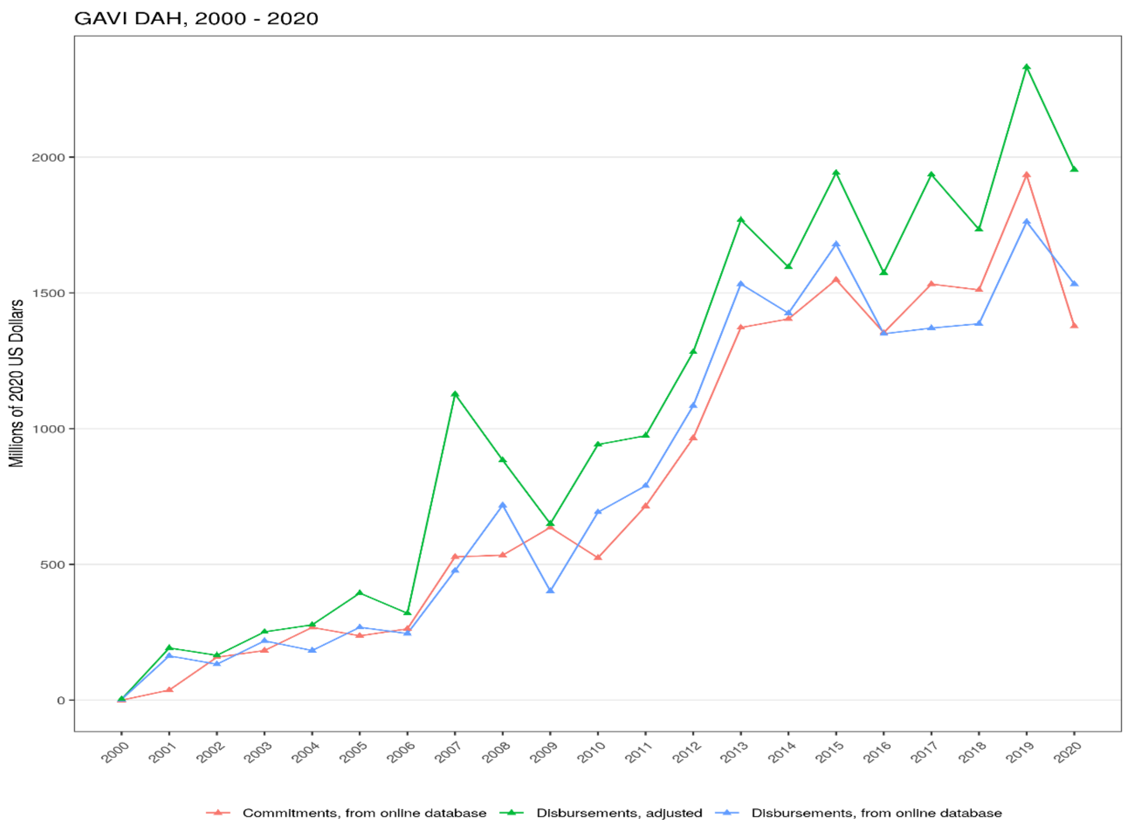

Source: IHME DAH Database (2020)

S3.4.2 Coalition for Epidemic Preparedness and Innovation

CEPI provided a complete dataset of all disbursed funding by disease from 2017-2020 and all income received from donors from 2018-2020. Funding data was provided per target vaccine and were classified between the other infectious diseases health focus area and the health systems strengthening other infectious diseases health focus area.

S3.5 Tracking expenditure by United Nations Agencies active in the health domain

Data on income and expenditures were collected for six UN agencies: WHO, UNICEF, UNFPA, UNAIDS, UNITAID and PAHO. The data sources and calculations for each are described in detail below. Similar to the bilateral channels, we extracted budget data for the UN agencies to predict DAH for years for which we did not have health expenditure data. Model choices and budget measures for UN agencies are presented in Table S3.3.

S3.5.1 World Health Organization

Data on WHO's budgetary and extrabudgetary income and expenditure were compiled from annual reports and audited financial statements released by WHO.[85] Income data were extracted from WHO's assessed and voluntary contributions, while expenditure data were extracted from both budgetary and extrabudgetary spending reports. For financial statements representing activities over a two-year period, both income and expenditure data were divided by two, in order to approximate yearly amounts, and dollars were deflated using the US GDP deflator specific to the

reporting year. Expenditures from trust funds, regional offices tracked separately, and associated entities not part of WHO's program of activities, such as UNAIDS and THE GLOBAL FUND trust funds were excluded. Expenditures from supply
services funds were also excluded, as these expenditures pertain to services provided by WHO but paid for by recipient countries. Additionally, WHO projects tracked as paid Ebola expenditure were extracted from the UNOCHA Financial Tracking System database and included as WHO health expenditure.

Disbursement data were not available for WHO in 2020. Much like the bilateral agencies, the ratio of DAH to the total program budget was estimated for 1990-2019 and then predicted for 2020 using a three year weighted average of 2017-2019. The predicted ratio was then multiplied by the observed program budget for 2020 to get the estimates of DAH.

S3.5.2 United Nations Population Fund

Data on income and expenditure were extracted for UNFPA from its audited financial statements.[72] As the 1990-2005 statements represent activities over a two-year period, income and expenditure data were divided by two in order to approximate yearly amounts.

Income and expenditures associated with procurement and cost-sharing activities were excluded from estimates of health assistance because UNFPA uses cost-sharing accounts when a donor contributes to UNFPA for a project to be conducted in the donor's own country. Since this money can be considered domestic spending that goes through UNFPA before being returned to the country in the form of a UNFPA program, it is not included in calculations of total DAH. UNFPA's additional expenditures for these projects come from trust funds or regular resources and are therefore captured in our estimates. To estimate disbursements by health focus areas, UNFPA's total health expenditure was multiplied by the proportion of funding reported for each program area from annual reports from 1997 through 2013, from the UNFPA transparency portal for 2014 through 2018, and from the 2018-2019 Statistical and Financial Review for 2019-2020. Maternal and child health spending classified as "other" was split equally between the maternal and child health program areas. Health spending in 2018 that was classified as towards "Integrated sexual and reproductive health services" was split equally between maternal health, family planning, other reproductive health and other HIV/AIDS program areas. Additionally, UNFPA projects tracked as paid Ebola expenditure were extracted from the UNOCHA Financial Tracking System database and added to UNFPA health expenditure.

The disbursement data for UNFPA were available through 2018. For year 2019-2020, the ratio of DAH and income was estimated to be the same as that for 2018. This approach was used rather than implementing a three-year weighted average of ratios because the change in funding structure was assumed to persist for the near future. The predicted ratio was then multiplied by observed income to estimate DAH for 2019-2020.

S3.5.3 United Nations Children's Fund

Data on income and expenditure for UNICEF were extracted from its audited financial statements.[69] As these statements represent activities over a two-year period from 1990-2011, income and expenditure
data were divided by two in order to approximate yearly amounts. The audited financial statements from 2012 onwards are produced on an annual basis.

Since UNICEF's activities are not limited to the health sector, the fraction of UNICEF's expenditure that was for health was estimated using either financial data from correspondence (2001-2013 observed data was used to estimate 1990 through 2000 expenditure) or a combination of annual reports and annual results reports from 2014 through 2019. The annual results reports provide the proportion of funding for each program area and the average of 2014 through 2016 proportions was used to estimate the spending proportion for the years 1990 through 2013. In the annual results report, HIV/AIDS funding and Nutrition funding (as of 2014) was reported separately from health funding so the percentages spent on each health program were proportioned based on total spending for Health.

Furthermore, UNICEF projects tracked as paid Ebola expenditure were extracted from the UNOCHA Financial Tracking System and added to estimates for UNICEF's health expenditure.

The product of observed program budget and the three-year weighted average of the DAH to budget was used to predict DAH in 2020.

S3.5.4 Joint United Nations Programme on HIV/AIDS

UNAIDS income and expenditure data for both its core and noncore budgets were extracted from its audited financial statements.[67] As financial data are provided on a biennial basis in all years prior to 2012, those values were divided by two to obtain yearly amounts. Dollars were deflated using the US GDP deflator specific to the reporting year.

For UNAIDS, budget measures were available only for a subset of reported total disbursements. UNAIDS reported total expenditure, which combined Unified Budget and Workplan (UBW) and non-UWB components, but only UBW budget data were available.[68] To predict DAH for UNAIDS in 2019-2020, disbursements for that year were estimated by multiplying the observed UBW budget by the three-year weighted average of the ratio of DAH to the UWB budget. UNAIDS disbursements were assigned to HIV/AIDS and TB program areas as documented in Table S3.7 above.

S3.5.5 UNITAID

Data on project level disbursement was obtained through correspondence with UNITAID. Income data was extracted from the annual financial statements downloaded from UNITAID's website. The project level data provided covered project disbursements from 2007 through 2020 and project budget commitments was provided for 2020.

S3.5.6 Pan American Health Organization

The Pan American Regional Office for WHO, or PAHO, reports its income and expenditure in its biennial financial report.[10,86] The funds transferred through the "Rotating Fund" were excluded because developing countries fund this procurement of health commodities which are then used within that funding country , and it therefore does not fit the definition of DAH.

As the financial data are provided on a biennial basis (with the exception of 2010 through 2016, where single-year financial reports were available), the quantities were divided by two to obtain yearly amounts. Dollars were deflated using the US GDP deflator specific to the reporting year.

Correspondence with PAHO revealed that data from the financial statements include both Program and nonProgram funds. The latter include funds that countries provide PAHO, so that PAHO can reinvest these funds into the countries' national health systems. These funds should not be included as development assistance for health, and PAHO provided corrected disbursement numbers for 2008 to 2013. The corresponding disbursement numbers for 2014 and 2015 were identified in the PAHO End-of-Biennium Assessment 2014-2015. These funds were provided as biennial disbursements, so they were divided by two to obtain yearly disbursements. The ratio of Program disbursements numbers provided by PAHO and the sum of Program and non-Program funds collected from financial statements was taken for the years 2008 to 2015. The average ratio was calculated, and this ratio was multiplied through disbursement numbers collected from financial statements from earlier years. In this way, Program and non-Program funds collected from audited statements from earlier years were adjusted to estimate DAH.

For PAHO, disbursement data were not available for 2020. PAHO provided budget information along with

disbursements for 2008 to 2019. The average ratio between spending and budget was calculated over the years 2008 to 2019, and this ratio was used to estimate 2020 disbursements.

S3.6 Tracking development assistance for health from private foundations

Previous studies on foundations outside the US have documented the severe paucity of reliable time series data and lack of comparability across countries.[87] Hence, this research focused efforts on tracking only US foundations.

S3.6.1 US Foundations

The Foundation Center maintains a database of all grants of $10,000 or more awarded by over 1,000 US foundations. The Foundation Center has coded each grant by sector and international focus and therefore is able to identify global health grants. IHME purchased a customized dataset with cross-border health grants and health grants to US-based international programs from 1992 to 2018 from the Foundation Center.[32] Grants from BMGF, which were tracked separately, were excluded. Additionally, grants to channels that this research already tracks were excluded.

The Foundation Center adopted a new classification methodology as of FGH 2016. The Foundation Center was able to provide historic data based on the new classification system from 2002 to 2012. In order to obtain the series from 1992 to 2001, we multiplied a weighted fraction calculated based on both old and new classification data values from 2002 through 2004 by the old data series (1992 to 2001) we had previously obtained.

$$
\begin{aligned}
(Weighted\ fraction) \\
&= \left(\frac{1}{2}\right)\left(DAH_{new\ classification}\right)/\left(DAH_{old\ classification}\right)2002 \\
&+ \left(\frac{1}{3}\right)\left(DAH_{new\ classification}\right)/\left(DAH_{old\ classification}\right)2003 \\
&+ \left(\frac{1}{6}\right)\left(DAH_{new\ classification}\right)/\left(DAH_{old\ classification}\right)2004
\end{aligned}
$$

$$(DAH\ Estimate_t) = (Weighted\ fraction)(DAH\ Observed_t)$$

where DAH Observed is the old data values for the series 1992 through 2001

To estimate total health grants in 1990-1991 and 2019-2020, natural log of US foundation DAH was regressed on the lagged natural log of US GDP per capita and year using ordinary least squares estimation. The missing years of data were predicted based on estimated regression coefficients from the equation. Exponents of the predicted values were used as final estimates

$$(ln\ Foundation_t) = \alpha + 1.\beta_1(\ln US\ GDP\ per\ capita_t) + \beta_2(year_t) + \varepsilon$$

Details on how we estimated the cost of providing technical assistance and program support for these US foundations are highlighted below in the section titled calculating the technical assistance and program support component of development assistance for health from loan-and grant-making channels of assistance.

S3.6.2 Bill & Melinda Gates Foundation

BMGF has been the single largest grant-making institution in the health domain since 2000; hence, additional research was undertaken to accurately capture its annual disbursements. BMGF's IRS 990PF filings for years 1999-2008, which report all global health grants disbursed per year, were downloaded from the BMGF website.

Additionally, disbursement data for years 2009-2019 were collected from the BMGF online grants database, the OECD CRS and personal correspondence. The OECD CRS data was used to identify NGOs that are double-counted from other data sources.

An ordinary least squares linear regression model was used to predict the disbursement for BMGF for 2020. Since there is a strong correlation between market trends and BMGF annual disbursements, market data including lagged US GDP, lagged yearly average of Berkshire stock returns, lagged yearly average of the Russell Index, and lagged total assets of the BMGF Trust were utilized to predict the total disbursement for year 2020.

$$(BMGF\ total\ disbursement_t) = \alpha + \beta_1(US\ GDP\ per\ capita_{t-2}) + \beta_2(Berkshire\ stock\ returns_{t-2}) + \beta_3(Russell\ Index_{t-2}) + \beta_4(BMGF\ total\ asset_{t-2}) + \varepsilon$$

BMGF's predicted DAH was adjusted to account for in-kind DAH and double-counting. The difference between BMGF's final DAH and DAH without in-kind added and double-counting removed from 2003-2019 was regressed using ordinary least squares on DAH without in-kind added and double-counting removed and year. The predicted difference was then subtracted from the predicted DAH from the previous regression for 2020.

S3.6.3 European Economic Area

DAH estimates for the European Economic Area were based on two downloadable project datasets available online. IHME DAH data for 2014 to 2018 was obtained from projects in the 2009-2014 grant period dataset with programme area "public health initiatives", and 2007-2012 data was obtained from projects in the 2004-2009 grant period dataset with sector "health and childcare". Childcare projects not related to health were identified and excluded based on manual screening and keyword search for the following terms: "NURSERY" "ORPHANAGE" "RECREATION" "FOSTER" " CARE HOME " " CHILDREN S HOME " " CHILD DEVELOPMENT CENTRE " " YOUTH CENTER" " YOUTH CAMPS " " RESIDENTIAL CENTRE" " RESIDENTIAL CARE FACILITIES " "CHILDCARE" " INFORMAL EDUCATION FOR CHILDREN " " PREVENT INSTITUTIONALISATION " " SPORTS FIELD" " SPORT FIELD" " SPORTS FACILIT" " SPORTS ACTIVIT" " SPORTS INFRASTRUCTURE" " REINTEGRATION OF JUVENILE OFFENDERS "

We determined that no health projects to low- and middle-income countries were implemented by the EEA prior to the 2004-2009 grant period, based on the report on the Financial Instrument 1999-2003 and the Financial Mechanism 1994-1998.[88,89] Project summary variables were obtained through correspondence, and were used together with project titles to assign DAH to health focus areas using a keyword search. To assign annual funding for projects that were implemented across multiple years, project grants were split based on the average daily spending across the duration of the project and the proportion of the year that the project was in implementation. The relative contributions of donors to the EEA grants (Norway, Iceland, and Liechtenstein) were obtained as constant ratios based on the 2016-2017 Annual Report.[90]

An open call for projects in the EEA's third grant period occurred in 2014, the first projects of which began disbursing in 2019 and are set to conclude in 2021. Data for the 2019-2020 grants came from personal correspondence.

S3.7 Tracking non-governmental organisations

Currently, there are no centralized, easily accessible databases for tracking program expenses of the thousands of NGOs based in high-income countries that are active in providing development assistance and humanitarian relief worldwide. This study relied on CRS data and the only comprehensive data source identified for a large subset of

these NGOs, namely the United States Agency for International Development's Report of Voluntary Agencies (USAID's VolAg report).[27] The report, which includes both US-based and international NGOs that received funding from the US government, provides data from 1990 to 2014 on domestic and overseas expenditures for these NGOs as well as their revenue from US and other public sources, private contributions, and in-kind. Total revenue and expenditure data obtained from the NGOs' IRS tax forms, accessed through the GuideStar online database, were also used in tracking NGOs incorporated in the US.[26] The GuideStar Research Fundamentals Plus dataset was used to obtain information for US-based NGOs in fiscal years 2015-2017, including domestic, overseas, and total expenses, and revenues from all public and private sources.

First, in order to track disbursements from OECD donor countries to NGOs, we utilized channel codes present in the CRS database. The code 21000 identified international NGOs and the code 22000 identified donor-country-based NGOs. In order to remove double-counting, we conducted a keyword search on channels where the donor country was the United States to exclude NGOs present in the USAID VolAg report and the GuideStar dataset. Allocation of funding to health focus areas for NGOs tracked through the CRS was assigned as described in the section "DISAGGREGATING BY HEALTH FOCUS AREA", based on a keyword search of five descriptive variables in the CRS: project title, short description, long description, channel name, and channel reported name. For NGOs tracked in the USAID VolAg report and the GuideStar dataset, allocation of funding to health focus areas was assigned as described in the section "DISAGGREGATING BY HEALTH FOCUS AREA", based on a keyword search of the NGO's description given in the VolAg report. Due to CRS projects containing more complete descriptions than the VolAg report descriptions, the health focus area allocations from CRS were prioritized over those from the VolAg report for US NGO agencies that appeared in both sources.

In order to use the USAID VolAg data and the GuideStar dataset, several challenges were overcome. We outline these challenges here and discuss below the methods employed to estimate a consistent series of DAH channeled through NGOs despite these challenges. First, with the exception of BMGF, it was impossible to track the amount of funding from US foundations routed through US NGOs, which may have led to double-counting in estimates of total health assistance. The second challenge relates to the incompleteness of the universe of NGOs captured through the VolAg report and the GuideStar Research Fundamentals dataset. The VolAg report provides data on NGOs that received funding from the US government. While this covers many of the largest NGOs, it is not a comprehensive list. A related problem is that the VolAg report and GuideStar dataset only include NGOs that received funds in a given year. While many of the largest NGOs are consistently funded by the US government and are therefore in the dataset every year, not all NGOs are reported across all years. Third, health sector-specific expenditure is not reported in the VolAg or systematically reported in IRS tax forms. The VolAg does report overseas expenditure but does not disaggregate this expenditure by sector. Fourth, complete data are lacking in several time periods. The 2016 VolAg provided data through 2014. For NGOs incorporated in the US, IRS tax forms were obtained. Furthermore, prior to 1998 the VolAg report did not include international NGOs. Attempts were made to compile other data on the health expenditures of the top international NGOs, in terms of overseas expenditure, by searching other websites for financial documents and contacting these organizations directly. Getting reliable time series data before 2000 proved to be extremely difficult for even this small sample of international NGOs.

Estimates of the share of overseas expenditure spent on health-related projects drew upon a sample of NGOs for which such data were available. Collecting financial data on health expenditures for each NGO would have been prohibitively time-consuming. Therefore, a sample of NGOs was drawn from the list for each year; the sample included the top 30 NGOs in terms of overseas expenditure and 20 randomly selected US-based NGOs from the remaining pool, with the probability of being selected set proportional to overseas expenditure. Next, health expenditure data were collected for each NGO in this sample by seeking out annual reports, audited financial statements, 990 tax forms, and data from NGO websites. Health expenditure was carefully reviewed to ensure that expenditures on food aid, food security, disaster relief, and water and sanitation projects were not included. Table S3.8 summarizes the number of NGOs included each year in the USAID report, the number of NGOs in the sample by year, and the number of NGOs for which health expenditure data were successfully compiled.

Table S3.8 Summary of US non-governmental organizations in the study

Year	Number of US NGOs in VolAG report	Number of international NGOs in VolAG report	Number of US NGOs in IHME sample	Number of US NGOs from sample for which data on health expenditure were found
**1990**	267	-	16	9
**1991**	334	-	19	14
**1992**	385	-	18	15
**1993**	411	-	17	12
**1994**	424	-	17	10
**1995**	416	-	16	12
**1996**	423	-	21	14
**1997**	425	-	23	18
**1998**	435	42	24	22
**1999**	438	-	33	28
**2000**	433	50	34	28
**2001**	442	51	33	26
**2002**	486	58	33	27
**2003**	507	54	42	32
**2004**	508	55	47	33
**2005**	494	59	45	36
**2006**	536	67	50	38
**2007**	556	68	50	40
**2008**	565	78	58	48
**2009**	580	90	57	45
**2010**	579	94	69	57
**2011**	595	112	73	63
**2012**	579	94	69	60
**2013**	519	113	69	52
**2014**	485	106	73	54
**2015**	-	-	67	53
**2016**	-	-	115	98
**2017**	-	-	81	69

A random effects regression model was fit to predict health expenditure as a fraction of total expenditure using the data for the sampled NGOs. A random effects model was chosen because the sample included observations for several NGOs for multiple years. A random effects model allows for the effect of each type of NGO to be captured distinctly. This model was used to predict the fraction of expenditure spent on health for the remaining NGOs. To ensure that the predicted health fractions were bounded between zero and one, the regression utilized the logit-transformed health fraction as the dependent variable. Since several NGOs in the sample were observed for multiple years, the regression included a random effect that varied by NGO. Five of the nine variables used to predict the health fraction were drawn from the VolAg reports and the GuideStar dataset. They were (1) fraction of revenue from in-kind donations, (2) fraction of revenue from the US government, (3) fraction of revenue from private financial contributions, (4) overseas expenditure as a fraction of total expenditure, and (5) calendar year. The remaining four variables used to predict the health fraction were binary indicators that were constructed based on

keyword searches on the NGO name and NGO description found in the VolAg. For both the NGO name and description, a keyword search was conducted to indicate whether the name or description was sufficiently health-related. Another keyword search was conducted independently on the NGO names and descriptions for keywords that indicated if the NGOs might focus on something other than health. These four indicators proved excellent predictors of health fractions.

$$\begin{aligned} logit(\,NGO - specific\; DAH_{it}) &= \alpha + \beta_1(Inkind\; contributions\; fraction_{it}) \\ &+ \beta_2(US\; government\; contributions\; fraction\;_{it}) \\ &+ \beta_3(Private\; financial\; contributions\; fractions_{it}) \\ &+ \beta_4(Overseas\; expenditure\; as\; a\; fraction\; of\; total\; expenditure_{it}) \\ &+ \beta_5(Health - related\; name_{it}) + \beta_6(Non - health - related\; name_{it}) \\ &+ \beta_7(Health - related\; description_{it}) + \beta_8(Non - health - related\; desciption_{it}) + U_i + \varepsilon \end{aligned}$$

Overseas health expenditure was calculated for individual NGOs in each year by multiplying the estimated health fraction and total overseas expenditure. For the NGOs that were sampled, the observed health fraction acquired through data collection was used. For the unsampled NGOs, the fitted fraction from the previously described random effects regression was used. Total overseas expenditure, reported in the VolAg, was not available for 2015-2020. For US-based NGOs, the 2017 NGO overseas fraction was calculated by regressing the logit transformed observed overseas fraction on a linear time trend using ordinary least squares, for each NGO independently. For these cases, the overseas health fraction was calculated as the product of estimated overseas fraction, estimated health fraction, and total expenditure found in the IRS 990 forms.

$$logit(Observed\; overseas\; health\; expenditure_i) = \alpha + \beta_i(year_t) + U_i + \varepsilon$$

At this point three reasons remained why the overseas health expenditure for some NGOs remained unknown. First, if an observation was non-US-based, then IRS tax forms were not available and total overseas expenditure could not be calculated. Second, for 2018 to 2020, no data were available. Finally, if an NGO was reported in the VolAg in multiple years but not for an intermittent year, no NGO-specific data were available for the gap year. This would be the case if an NGO received support from the US government one year and then again in a nonconsecutive year. For all three of these scenarios, a panel-based hierarchical linear regression model was used to fill in the overseas health expenditure gaps. Total overseas health expenditure (measured at the NGO-year level) was regressed on US GDP per capita and US bilateral DAH disbursed. Because the US government funds many of these NGOs, US bilateral DAH was an excellent predictor of NGO DAH. A flexible model was employed to allow both the GDP and US government DAH coefficients to vary randomly across NGOs, such that each NGO employed a unique (but not independent) relationship between overseas health expenditure, GDP, and US government DAH. A random intercept was also included to capture the significant unobserved heterogeneity present in our set of NGOs. Once fit, this model was used to predict overseas health expenditure for all remaining gaps.

$$(\,NGO\; DAH_{it}) = \alpha + \beta_{1i}(US\; GDP\; per\; capita_t) + \beta_{2i}(US\; bilateral\; DAH\; per\; capita_t) + U_i + \varepsilon$$

Expenditures financed from each revenue source were then calculated by multiplying overseas health expenditure by NGO-specific revenue fractions. Expenditures from in-kind sources were deflated by a constant fraction. This was determined by comparing the federal upper limit and average wholesale price valuations of drugs on the WHO's Model List of Essential Medicines from the RED BOOK Expanded Database.[28,29] Figure S3.13 and Figure S3.14 show the income and estimated overseas health expenditure, respectively, of the NGOs in the universe of US- and non-US-based NGOs that were tracked in this study from 1990 to 2017 in constant 2020 US dollars.

Figure S3.13 Total revenue received by non-governmental organizations

The orange line shows total revenue for all sources, both public and private, received by NGOs. The green line shows estimates of private financial contributions to NGOs, while the blue line shows private in-kind donations to NGOs.

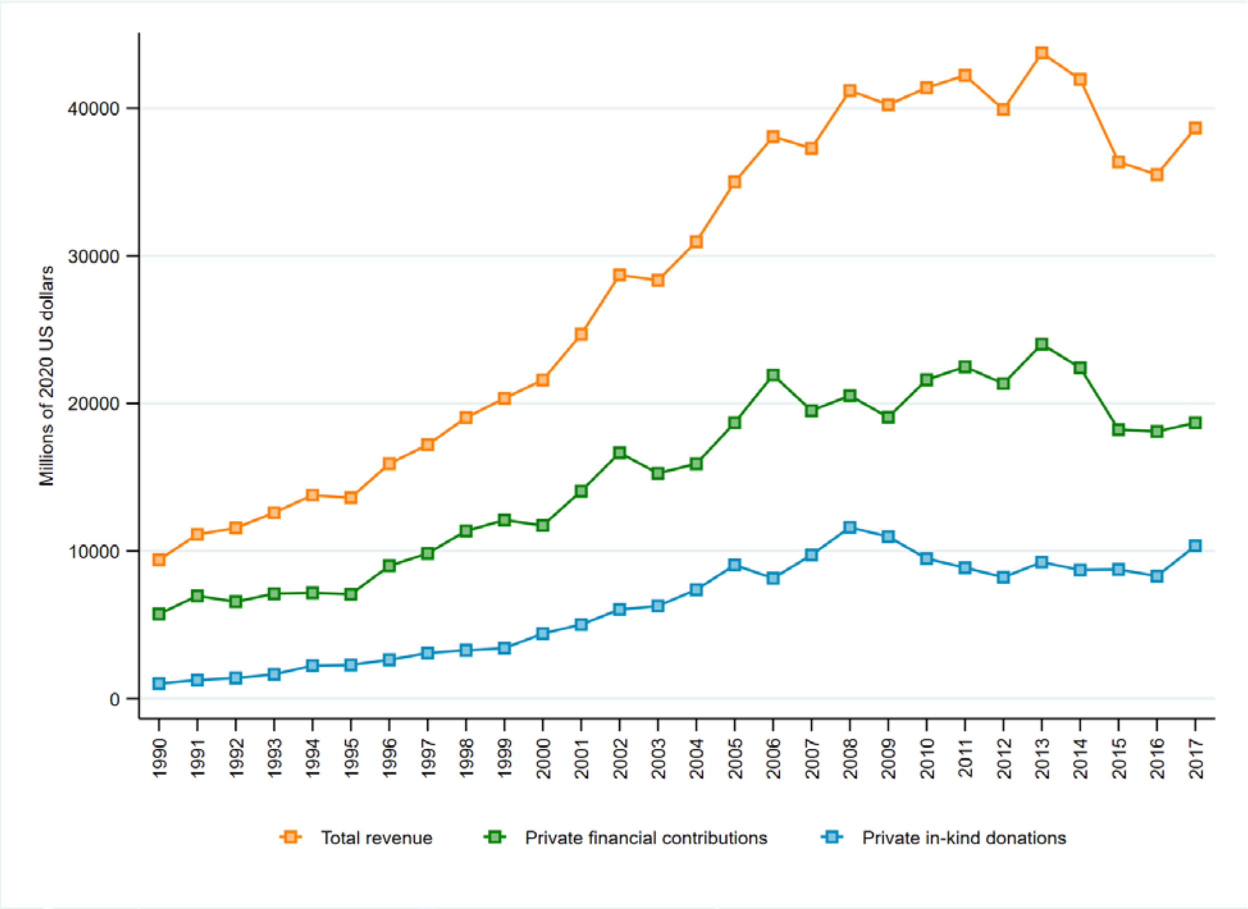

Source: IHME DAH Database (2020)

Figure S3.14 Expenditure by non-governmental organizations

The orange line illustrates total overseas expenditure by NGOs, regardless of sector. The green line shows overseas expenditure by NGOs to health-specific recipients, or DAH.

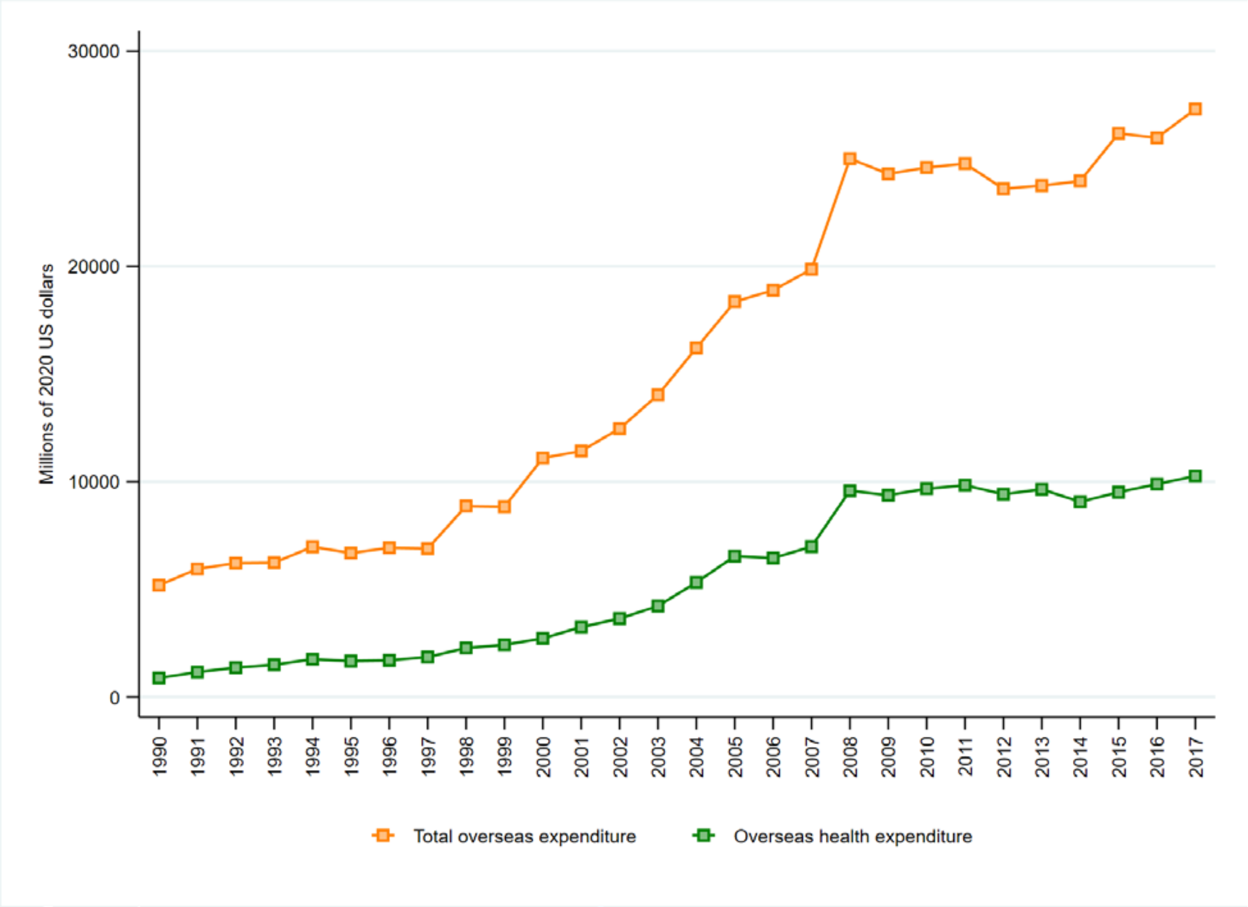

Source: IHME DAH Database (2020)

S3.8 Tracking development assistance for health from China

The estimates of DAH contributions from China is based on data from diverse sources including government reports and international development agencies databases. We generate estimates for bilateral and multilateral contributions from China separately and aggregate to obtain the total DAH contributions from China. We use project descriptions and reports where available to determine health focus areas of the contributions. For years with limited data, we use various methods inclusive of interpolation and modeling to generate estimates.

Following the addition of the China International Development Cooperation Agency (CIDCA) into our estimates last year, we now include five Chinese agencies in our bilateral DAH envelope: the National Health Commission, the Ministry of Commerce, the Ministry of Education, the Export-Import Bank of China, and CIDCA.

Detailed description of the methods for generating the estimates are reported in Micah AE, Zhao Y, Chen CS, et al[97].

S3.9 Calculating the technical assistance and program support component of development assistance for health from loan-and grant-making channels of assistance

The following methods were used to estimate the costs incurred by loan- and grant-making institutions for administering and supporting health sector loans and grants, which includes costs related to staffing and program management.

Data on the total administrative costs were compiled for institutions in our universe for which these data were readily available: IDA, IBRD, ADB, AfDB, IDB, BMGF, the Wellcome Trust, US Foundations, CEPI, THE GLOBAL FUND, Gavi, UNICEF, UNFPA, WHO, UNITAID, PAHO, UNAIDS, USAID, and the UK Department for International Development (DFID), Japan International Cooperation Agency (JICA), Norwegian Aid Agency (NORAD) and the Swedish International Development Agency (SIDA). The sources of data for the institutions in this sample are summarized in Table S3.9. The ratio of total administrative costs to total grants and loans was calculated for each source by year. It was assumed that the percentage of operating and administrative costs devoted to health would be equal to the percentage of grants and loans that were for health. In other words, if 20% of a foundation's grants were for health, the model assumed that 20% of administrative costs of the foundation were spent on facilitating these health grants. Given this assumption, the ratios of the observed administrative costs to grants/loans were used to estimate the in-kind contribution made by each of these organizations toward maintaining their health grants and loans. For the institutions not in this sample, the ratio from the institution most similar to it was used to arrive at an estimate of in-kind contributions. For example, for US foundations we selected the top 10 and bottom 10 US foundations based on total disbursements across the entire time period. For each foundation, we calculated the ratio of the "cash basis" column of total operating and administrative expenses to grants paid. We then used the average of these top 10 and bottom 10 foundations ratio as the inkind ratio across time. Total in-kind contributions from all grant- and loan-making global health institutions are shown in Figure S3.14.

Table S3.9 Summary of data sources for calculating in-kind contributions

Organization	Source	Notes
**BMGF**	990 tax returns (1999-2006)<br>BMGF Trust financial statements (2007-2019)	Used "cash basis" column to calculate ratio of total operating and administrative expenses to grants paid.<br>Used "grants expenditure" statement to calculate ratio of administrative expenditure to grants/program expenditure.
**US Foundations**	990 tax returns	Calculated the average across time of the ratio of the "cash basis" column of total operating and administrative expenses to grants paid.
**Wellcome Trust**	Annual report	Calculated ratio of support costs to grants awarded by the trust
**CEPI**	Board of directors report, 2016-2019	Calculated ratio of operating expenses to total expenditure.
**THE GLOBAL FUND**	Annual report financial statements	Calculated ratio of operating expenses to grants disbursed.
**Gavi**	Annual report financial statements	Calculated ratio of management, general, and fundraising expenses to program expenses.
**UNICEF**	Annual report financial statements	Calculated ratio of programme support to total expenditure (1990-2011)<br>Calculated ratio of institutional budget to total programme and emergency budgets (2012-2019)
**UNFPA**	Annual financial review	Calculated ratio of institutional budget to total expenses

**WHO**	Financial report and annual financial statements	Calculated ratio of general operating expenses to total expenses
**UNITAID**	Financial statement	Calculated ratio of operating expenses to program expenses
**USAID**	US government budget database	Used outlays spreadsheet to calculate ratio of total outlays for USAID operating account to sum of outlays for bilateral accounts.
**DFID**	Annual report expense summary	Calculated ratio of DFID's administration expenses to DFID's bilateral program expenses from 2002 onward.
**JICA**	Statement of income in annual report	Calculated ratio of general administrative expenses to operating expenses less depreciation
**SIDA and other Swedish agencies disbursing aid**	Swedish OpenAid website[91]	Calculated ratio of administrative costs to the sum of all program expenses
**NORAD and other Norwegian agencies disbursing aid**	Norwegian Aid statistics website[92]	Calculated ratio of general administrative expenses to operating expenses less depreciation
**IDA**	World Bank audited financial statements	Calculated ratio of management fee charged by IBRD to development credit disbursements.
**IBRD**	World Bank audited financial statements	Calculated ratio of administrative expenses to loan disbursements.
**AfDB**	Income statements in annual reports	Calculated ratio of administrative expenses to the sum of loan and grant disbursements
**ADB**	Annual report	Calculated ratio of administrative expenses to the sum of loan and grant disbursements
**IDB**	Annual report and financial statement	Calculated the ratio of administrative expenses to total loan disbursements
**EEA**	Administrative budget data[93]	Calculated ratio of donor states' management budget and donor programme partners' participation to Net allocation to beneficiary countries
**PAHO**	Audited financial statements	Calculated ratio of general operating expenses and contractual services to total expenses.
**UNAIDS**	Audited financial statements	Calculated ratio of general operating expenses to total expenses.

Figure S3.15 In-kind contributions by loan- and grant-making DAH channels of assistance

This figure illustrates the proportions of financial and in-kind DAH disbursed by loan- and grant-making institutions. The proportion of in-kind DAH varies, based on the channel. The overall proportion of in-kind DAH received across all channels has grown over time.

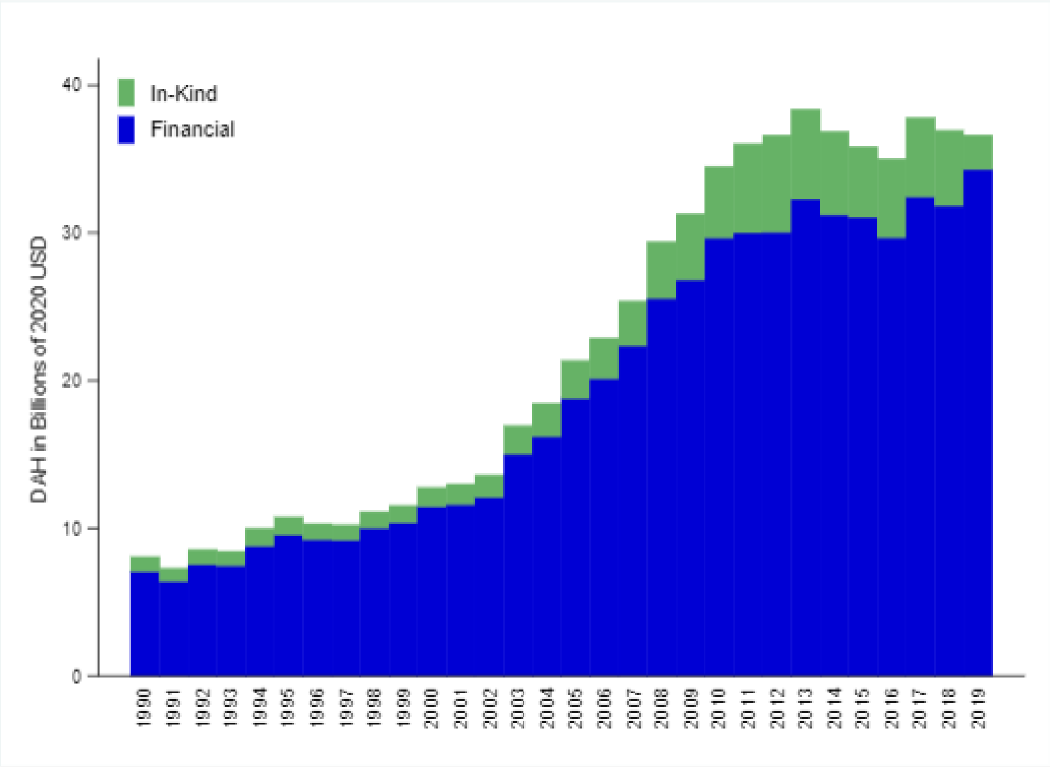

Source: IHME DAH Database (2020)

S3.11 REFERENCES

1 Dieleman JL, Schneider MT, Haakenstad A, *et al*. Development assistance for health: past trends, associations, and the future of international financial flows for health. *The Lancet* 2016; **387**: 2536–44.

2 OECD. International Development Statistics (IDS) online databases. https://www.oecd.org/development/stats/idsonline.htm (accessed October 10, 2020).

3 European Commission. Annual reports - international cooperation and development. Int. Coop. Dev. /europeaid/annual-reports_en (accessed November 2, 2020).

4 UNAIDS. PCB Archive. http://www.unaids.org/en/aboutunaids/unaidsprogrammecoordinatingboard/pcbmeetingarchive (accessed Nov 9, 2020).

5 UNICEF. UNICEF Strategic Plan: updated financial estimates, 2019-2022. 2020. https://www.unicef.org/about/execboard/files/2019-ABL5-SP_financial_estimates_2019-2022-EN-ODS.pdf

6 UNICEF. United Nations Children's Fund Financial report and audited financial statements for the year ended 31 December 2017 and Report of the Board of Auditors. United Nations: New York, 2018. https://www.unicef.org/about/execboard/files/A-73-5-Add3-BOA_report-ODS-EN.pdf

7 UNICEF. Annual Report 2019. UNICEF. https://www.unicef.org/reports/annual-report-2019 (accessed Nov, 23, 2020).

8 United Nations Population Fund. Annual Report 2019. http://www.unfpa.org/annual-report (accessed Sept 30, 2020).

9 UNITAID. Audited Financial Statements (2007-2019). Unitaid. https://unitaid.eu/publications/ (accessed Nov 17, 2020).

10 Pan American Health Organization, World Health Organization. Executive Committee Session. 2019; published online Dec 20. http://iris.paho.org/xmlui/handle/123456789/34210 (accessed Nov 10, 2020).

11 World Health Organization. Financial report and audited financial statement, 2019. World Health Organization, 2019 http://apps.who.int/gb/ebwha/pdf_files/WHA68/A68_38-en.pdf.

12 The World Bank. Projects & Operations. http://projects.worldbank.org/ (accessed Jan 13, 2021).

13 Paris M. Project database 1990-2020 obtained through personal correspondence. 2021.

14 African Development Bank. Compendium of statistics on bank group operations. Tunis, Tunisia: Statistics Department, African Development Bank.

15 African Development Bank. Financial report – Financial Management and Financial Statements Year Ended 31 December 2019. (accessed Nov 9, 2020).

15 African Development Bank. Online project database. https://www.afdb.org/en/projects-and-operations/project-portfolio/ (accessed Jan 30, 2017).

16 Inter-American Development Bank. Annual Report – The Year in Review 2019. https://www.iadb.org/en/about-us/annual-reports (accessed Oct 26, 2020).

16 Inter-American Development Bank. Projects database. https://www.iadb.org/en/projects-search?query%5Bcountry%5D=&query%5Bsector%5D=SA&query%5Bstatus%5D=&query%5Bquery%5D= (accessed Oct 21, 2020).

17 Neret M. health disbursement data. 2019; accessed Dec 19.

18 Gavi, the Vaccine Alliance. Disbursements and commitments. http://www.gavi.org/results/disbursements/ (accessed Oct 9, 2020).

19 Gavi, the Vaccine Alliance. Pneumococcal AMC. https://www.gavi.org/investing-gavi/innovative-financing/pneumococcal-amc / (accessed Oct 9, 2020).

20 Gavi, the Vaccine Alliance. Financial reports. http://www.gavi.org/funding/financial-reports/ (accessed Oct 9, 2020).

21 Gavi, the Vaccine Alliance. Cash Received database. http://www.gavi.org/funding/donor-contributions-pledges/cash-receipts/ (accessed Oct 9, 2020).

22 The Global Fund to fight AIDS, Tuberculosis and Malaria. Grants in detail and Disbursements. https://data-service.theglobalfund.org/downloads (accessed Dec 1, 2020).

23 The Global Fund to fight AIDS, Tuberculosis and Malaria. THE GLOBAL FUND annual reports. http://www.theglobalfund.org/en/archive/annualreports/ (accessed Dec 1, 2020).

24 The Global Fund to fight AIDS, Tuberculosis and Malaria. THE GLOBAL FUND pledges & contributions report. https://data-service.theglobalfund.org/downloads (accessed Dec 1, 2020).

25 GuideStar USA. Income tax filings. http://www2.guidestar.org/Home.aspx (accessed Nov 25, 2020).

26 United States Agency for International Development. VolAg report: report of voluntary agencies engaged in overseas relief and development. https://www.usaid.gov/pvo/volag-report (accessed Jan 30, 2017).

27 Thomson Reuters. Red book expanded database. New York: Thomson Reuters, 2009.

28 World Health Organization. WHO | Essential medicines. WHO. http://www.who.int/topics/essential_medicines/en/ (accessed Feb 15, 2017).

29 GuideStar. Data Sets for Research. https://learn.guidestar.org/products/data-sets-for-research (accessed November 24, 2020).

30 Bill & Melinda Gates Foundation. Audited Financial statements. Seattle, WA: Bill & Melinda Gates Foundation http://www.gatesfoundation.org/Who-We-Are/General-Information/Financials (accessed Oct 12, 2020).

31 Chan S. Foundation Awards and Payments. 2016; published online Aug 16.

32 Foundation Center. Grants database. Candid. https://candid.org/ (accessed Jan 13, 2021).

33 EEA Grants-Norway Grants, Financial Mechanism Office. EEA Grants project portal. https://eeagrants.org/resources?title=&field_resource_type_target_id=109 (accessed Sept 17, 2020).

34 Popic A. EEA project data obtained through personal correspondence. Brussels, Belgium. EEA and Norway Grants. 2020.

35 Fofana A. AfDB development support for health projects enquiry. 2019; accessed Dec 19.

36 Rouzinova R. Incorporating UNITAID into Financing Global Health Landscape. 2017; published online Aug 21.

37 Al Anood Al Abdool., United Arab Emirates Ministry of Foreign Affairs and International Cooperation. UAE Foreign Assistance in Health 1990-2008 through personal correspondence. 2018; published online Jan 24.

38 Australian Government. Department of Foreign Affairs and Trade. Aid budget and statistical information. Dep. Foreign Aff. Trade. http://dfat.gov.au/aid/aid-budgets-statistics/Pages/default.aspx (accessed Dec 11, 2020).

39 Department of Foreign Affairs and Trade. Annual reports. Dep. Foreign Aff. Trade. http://dfat.gov.au/about-us/publications/corporate/annual-reports/pages/annual-reports.aspx (accessed Nov 15, 2020).

40 Austria Federal Ministry of Finance. Federal budget. https://www.bmf.gv.at/budget/das-budget/das-budget.html (accessed Nov 24, 2020).

41 Belgium House of Representatives. Project budgets. https://www.lachambre.be/kvvcr/showpage.cfm?section=/pri/budget&language=fr&rightmenu=right_pri&story=2019-budget.xml (accessed Nov 15, 2020).

42 Government of Canada. Planning and performance. GAC. http://international.gc.ca/gac-amc/publications/plans/index.aspx?lang=eng#rpp (accessed Nov 15, 2020).

43 Danish Ministry of Foreign Affairs. Foreign affairs budget. http://www.oes-cs.dk/bevillingslove/ (accessed Nov 15, 2020).

44 European Commission. General Budget - budget on-line. https://ec.europa.eu/budget/graphs/revenue_expediture.html (accessed Nov 15, 2020).

45 Ministry of Finance Finland. State budget bills [in Finnish]. http://budjetti.vm.fi/indox/index.jsp (accessed Nov 15, 2020).

46 Ministry of Foreign Affairs and International Development. Politique francaise en faveur du developpement. Ministry of Foreign Affairs and International Development, 2019 https://www.performance-publique.budget.gouv.fr/sites/performance_publique/files/files/documents/dpt-2019/DPT2019_politique_developpement-W.pdf(accessed Nov 15, 2020).

47 Legifrance. Republique Francaise. Budget and financial documents. https://www.legifrance.gouv.fr/initRechTexte.do (accessed Nov 15, 2020).

48 German Federal Ministry of Economic Cooperation and Development. Plan of the federal budget. Fed. Minist. Econ. Coop. Dev. http://www.bmz.de/en/ministry/InDetail/budget/index.html (accessed Nov 15, 2020).

49 Greece Standing Committee on Economic Affairs. The state budget and budgets for certain special funds and services, 2013 and 2014 [in Greek]. 2014. http://www.hellenicparliament.gr/UserFiles/7b24652e-78eb-4807-9d68-e9a5d4576eff/Proyp2014-prak.pdf.

50 Greece Standing Committee on Economic Affairs. Ratification of the State Budget for the financial year 2017. http://www.hellenicparliament.gr/Nomothetiko-Ergo/Anazitisi-Nomothetikou-Ergou?law_id=76e87dd3-8cae-433f-963f-a6c50141de97 (accessed Feb 13, 2018).

51 Department of Finance, Government of Ireland. The Budget. http://www.budget.gov.ie/Budgets/2019/2019.aspx (accessed Nov 15, 2020).

52 Ministry of Foreign Affairs and International Cooperation. Stato di previsione del ministero degli affari esteri e della cooperazione internazionale. 2020. https://www.esteri.it/mae/resource/doc/2019/01/tabella_6_del_2019.pdf (accessed Nov 15, 2020).

53 Ministry of Finance Japan. Budget. http://www.mof.go.jp/english/budget/budget/index.html (accessed Nov 15, 2020).

54 Korea Official Development Assistance. Comprehensive Implementation plan for international development cooperation by year. http://www.odakorea.go.kr/hz.blltn2.YearPlanSlPL.do?brd_seq=3&blltn_div=oda (accessed Nov 15, 2020).

55 Ministry of Finance Luxembourg. State Budget[in French]. http://www.igf.etat.lu/ (accessed Nov 15, 2020).

56 New Zealand Treasury. Vote budget data - budgets of the New Zealand Government. 2019; published online May 30. http://www.treasury.govt.nz/budget (accessed Nov 15, 2020).

57 Ministry of Foreign Affairs Norway. Email correspondences. April 18, 2011, February 13, 2012, and August 14, 2013.

58 Norwegian Ministry of Finance. The National Budget 2019: a summary. https://www.statsbudsjettet.no/english/ (accessed Nov 15, 2020).

59 Ministry of Finance Portugal. State budget report. http://www.dgo.pt/Paginas/default.aspx (accessed Nov 15, 2020).

60 Ministry of Finance and Public Function Spain. State General Budget [in Spanish] http://www.congreso.es/docu/pge2019/pge_2019-tomos/PGE-ROM/doc/L_19_A_A4.PDF (accessed Nov 15, 2020).

61 Ministry of Foreign Affairs Sweden. International aid budget. http://www.regeringen.se/ (accessed Nov 15, 2020).

62 UK - Department for International Development. IATI Dashboard. http://dashboard.iatistandard.org/publisher/dfid.html (accessed Nov 15, 2020).

63 Her Majesty's Treasury United Kingdom. Budget. https://www.gov.uk/government/publications/spring-budget-2018-documents (accessed Nov 15, 2020).

64 Executive Office of the President of the United States. Budget of the United States Government. https://www.gpo.gov/fdsys/browse/collection.action?collectionCode=BUDGET&browsePath=Fiscal+

Year+2014&isCollapsed=true&leafLevelBrowse=false&isDocumentResults=true&ycord=0 (accessed Nov 20, 2020).

65 US Foreign Assistance Dashboard. Foreign assistance by category, health-planned stage. https://www.foreignassistance.gov/explore (accessed Nov 20, 2020).

66 World Health Organization. Proposed programme budget 2020-2021. https://apps.who.int/iris/handle/10665/274693 (accessed October 23, 2020).

67 Joint United Nations Programme on HIV/AIDS. Financial report and audited financial statement. https://www.unaids.org/en/aboutunaids/unaidsprogrammecoordinatingboard/pcbmeetingarchive (accessed Nov 9, 2020).

68 Joint United Nations Programme on HIV/AIDS. Unified budget and workplan. https://www.unaids.org/en/aboutunaids/unaidsprogrammecoordinatingboard/pcbmeetingarchive/ (accessed Nov 9, 2020).

69 UNICEF. United Nations Children's Fund Financial report and audited financial statements for the year ended 31 December 2018 and Report of the Board of Auditors. United Nations: New York, 2019. https://digitallibrary.un.org/record/3824598?ln=en

70 Sabbah L. Health expenditure data - UNICEF. 2015; published online Sept 29.

71 UNFPA. Report on contributions by member states and others to UNFPA and revenue projections. https://executiveboard.unfpa.org/ (accessed Aug 23, 2018).

72 UNFPA. Statistical and financial review, 2019. https://executiveboard.unfpa.org/ (accessed Oct 28, 2020).

73 Smithson M, Verkuilen J. A better lemon squeezer? Maximum-likelihood regression with beta-distributed dependent variables. *Psychol Methods* 2006; **11**: 54–71.

74 Office of the European Union. EU budget 2018 Financial report. Luxembourg: Office of the European Union, 2015 https://ec.europa.eu/info/publications/eu-budget-2018-financial-report_en (accessed Nov 15, 2020).

75 President's Emergency Plan for AIDS Relief/ US Government. PEPFAR Dashboards- PEPFAR OU Budgets by Budget code FY04-FY19. 2019. https://data.pepfar.gov/dashboards (accessed October 11, 2020).

76 President's Malaria Initiative/US Government. Malaria Operational Plans (MOPs). 2019. https://www.pmi.gov/resource-library/mops (accessed December, 2020).

77 Krackenberger K. IHME-WB FGH 2020 data request. 2021.

78 Projects & Operations - Sectors. http://projects.worldbank.org/sector (accessed Jan 13, 2021).

79 Miyuki Parris. Project database 1990-2020 obtained through personal correspondence. Washington D.C.: The World Bank, 2021.

80 World Bank Group - International Development Association. IDA18 Replenishment. Int. Dev. Assoc. - World Bank. 2016; published online Feb 16. http://ida.worldbank.org/financing/replenishments/ida18-replenishment (accessed Oct 10, 2020).

81 Asian Development Bank. Online project database. https://www.adb.org/projects (accessed Nov 23, 2020).

82 African Development Bank Group. Resource Mobilization and Partnerships. https://frmb.afdb.org/?page=adf&subpage=adf-ar (accessed Dec 21, 2018).

83 Asian Development Bank. Asian Development Fund 12 Donors' Report; published online May 2016. https://www.adb.org/site/adf/replenishments (accessed Oct 12, 2018).

84 Gavi, the Vaccine Alliance. Annual financial reports. https://www.gavi.org/search?s=financial&f%5B0%5D=type%3Adocument&page=0 (accessed Oct 9, 2020).

85 World Health Organization. WHO Audited Financial Statements for the year ended 31 December 2019. Geneva, Switzerland: World Health Organization, 2019 http://apps.who.int/gb/ebwha/pdf_files/WHA72/A72_36-en.pdf (accessed October, 2020).

86 Pan American Health Organization. Financial Report of the Director and Report of the External Auditor for 2019. 1 January 2019 - 31 December 2019 https://www.paho.org/hq/index.php?option=com_content&view=article&id=15194:official-document-357&Itemid=40453&lang=en er 2018. Washington D.C.: Pan American Health Organization, 2019. (accessed October, 2020)

87 Schlutter A, Volker T, Walkenhorst P. Foundations in Europe: International Reference Book on Society, Management and Law. Gutersloh, Germany; Washington, DC: Bertelsmann Stiftung; Brookings Institution Press, 2001.

88 European Economic Area. EEA grants report. Financial Instrument 1999-2003. Final report. http://eeagrants.org/content/download/10037/139187/version/3/file/141219+FI99-03+final+report.pdf.

89 European Economic Area. EEA report - Financial Mechanism 1994-1998. Final report. http://eeagrants.org/content/download/5915/65422/version/1/file/Financial+Mechanism+1994-1998+Final+Report.pdf.

90 European Economic Area. EEA Annual Report - 2016-2017. https://eeagrants.org/content/download/13045/175890/version/1/file/Annual+Report+2016-2017.pdf.

91 Swedish International Development Agency. Sweden's aid to the world for all sectors in. Openaid.se. https://openaid.se/aid/ (accessed December, 2020).

92 NORAD. Norwegian Aid Statistics. NoradDev. https://norad.no/en/front/toolspublications/norwegian-aid-statistics/ (accessed Dec 21, 2020).

93 European Economic Area. Administrative budget - EEA Grants. https://eeagrants.org/Who-we-are/How-we-work/Administrative-budget (accessed Oct 27, 2020).

94 UNFPA. UNFPA Report on the Structured Funding Dialogue 2019-2020. https://www.unfpa.org/pcm/node/19087 (accessed Oct 28, 2020).

95 World Health Organization. Status of collection of assessed contributions, including Member States in arrears in the payment of their contributions to an extent that would justify invoking Article 7 of the Constitution. Geneva, Switzerland: World Health Organization, 2020 http://apps.who.int/gb/ebwha/pdf_files/WHA72/A72_37-en.pdf (accessed October, 2020).

96 World Health Organization. Voluntary contributions by fund and by contributor, 2019. Geneva, Switzerland: World Health Organization, 2020. http://apps.who.int/gb/ebwha/pdf_files/WHA72/A72_INF5-en.pdf (accessed Oct 26, 2020).

97 Micah AE, Zhao Y, Chen CS, et al. Tracking development assistance for health from China, 2007–2017BMJ Global Health 2019;4:e001513.

98 Lavado R.F. ADB development support for health projects enquiry. 2019; accessed Dec 19.

99 International Monetary Fund. World Economic Outlook Database: October 2019. https://www.imf.org/external/pubs/ft/weo/2019/02/weodata/index.aspx (accessed Oct 20, 2019).

S3.11 Tracking Development Assistance for Health: incorporating COVID-19

COVID Keyword Search

To further our COVID DAH analysis, we developed a COVID-specific keyword search. This keyword search takes fine grain information supplied by grants' project descriptions and categorize COVID spending into one of the following nine program areas: 1) Country-level coordination, , including planning, monitoring, and evaluation, risk communication, and community engagement and travel restrictions, 2) Surveillance, rapid-response teams, and case investigation, 3) National labs and testing, 4) Infection prevention and personal protective equipment, 5) Treatment, 6) Supply chain and logistics, 7) Maintaining other essential health services and systems, 8) Research & Development for vaccines and other therapeutics, and 9) Other. Each program area is defined by a set of keywords (in English, Spanish, Portuguese, French, Italian, Dutch, German, Norwegian, and Swedish) which identify words and phrases in each grants' project description and proportion the grant total amount into each respective bucket. Grants which do not tag any keywords are automatically placed into the Other program area. Grant project descriptions are cleaned of special characters, made uppercase, void of excessive mid-line spacing, and padded with beginning and ending spaces, before passing through our keyword search. Table S3.11.1. presents the keywords in English for the ten defined program areas.

Table S3.11.1. List of keywords used to define program areas

Program Area	Keywords
Country-level coordination, , including planning, monitoring, and evaluation, risk communication, and community engagement and travel restrictions	" COORDINAT", " MONITOR", " PREPARED", " EMERGENCY RESPONSE", " NATIONAL PLAN", " EMERGENCY OPERATIONS CENT", " EOC ", " RISK ASSESS", " EVALUAT", " COMMUNICAT", " CALL CENT", " COMMUNIT", " VULNERABLE ", " AWARENESS ", " BEHAVIOR", " MARGINALIZED ", " RISK", " BORDER", " PORT", " AIRPORT"," TRAVEL ", " POINT OF ENTRY "
Surveillance, rapid-response teams, and case investigation	" SURVEIL", " RAPID RESPONSE TEAM", " CASE INVESTIGATION ", " CONTACT TRAC", " DETECT", " CLOSE CONTACT", "PHONE"
National labs and testing	" LAB", " TEST", " SEROLOGICAL ", " PCR ", " ANTIBODY "
Infection prevention and personal protective equipment	" INFECTION PREVENT", " IPC ", " PERSONAL PROTECTIVE EQUIP", " PPE ", " MASK", " HAND SANITIZ", " HYGIENE ", " WASH ", " SANITAT", " ISOLATION CENT", " NOSOCOMIAL ", " DISINFECT", " SOAP ", " FACE COVER", " SHIELD "
Treatment	" CASE MANAG", " INTENSIVE CARE ", " CLINIC", " DIAGNOSIS "," DIAGNOSES ", " TREAT", " TRIAGE ", " REFERRAL ", " THERAPY "," THERAPIES ", " VENTILAT", " OXYGEN ", " GUIDELINE", " REMDESIVIR ", " DEXAMETHASONE ", " CONVALESCENT PLASMA ", " TOCILIZUMAB ", " ACTEMRA "
Supply chain and logistics[1]	" OPERATIONAL SUPPORT ", " LOGISTIC", " STOCK ", " SUPPL", " PROCURE", " MED\\w+ EQUIP", " DEPLOY", " DELIVERY SYSTEM"

Maintaining other essential health services and systems	" ESSENTIAL HEALTH SERVICE", " HEALTH WORKFORCE PLAN", " BASIC HEALTH SERVICE", " BASIC SERVICE", " ROUTINE HEALTH SERVICE", " ROUTINE SERVICE", " HIV ", " AIDS ", " TUBERCULOSIS ", " IMMUNIZATION ", " MALARIA ", " MATERNAL ", " CHILD HEALTH ", " NEWBORN ", " REPRODUCTIVE HEALTH ", " SRH ", " NON COMMUNICABLE DISEASE", " NCD ", " NONCOMMUNICABLE DISEASE", " ESSENTIAL HEALTHCARE SERVICE", " TB "
Research & Development for vaccines and other therapeutics	" DIAGNOSTICS MANUFACTUR", " THERAPEUTICS MANUFACTUR", " DIAGNOSTICS DEVELOP", " THERAPEUTICS DEVELOP", " VACCINE DEVELOP", " MODEL", " INNOVAT", " RESEARCH", " R D ", " R AND D "
Other	Anything not categorized by the above keywords

Notes:

1. In the Supply chain and logistics category, we use the regular expression "\\w+" to string match any phrases which start with " MED" and end with "EQUIP". This gives us flexibility to catch phrases like medical equipment and medicines equipment without specifying an exhaustive list of possible permutations of the word medical.

**Example Project Going Through COVID Keyword Search**
Example project description: "COVID 19 Response: Distributing PPE and PCR Tests to Frontline Workers"<br>Example project amount: $900<br><br>Passing through our COVID keyword search, this project would be tagged by National labs and testing twice (for " PCR " and " TEST") and once by Infection prevention and personal protective equipment (for " PPE "). The resulting program area keyword counts would then proportion the grant amount 2/3rd into National labs and testing and 1/3rd into Infection prevention and personal protective equipment, attributing $600 to the former and $300 to the latter.

Table S3.11.2. Summary of COVID data sources by channel

**Channel**	**IATI**	**UNOCHA**	**Correspondence**	**channel Website**	**Other website**
AfDB			X	X	
AsDB			X	X	
Bilateral, Australia	X				
Bilateral, Austria				X	
Bilateral, Belgium	X				
Bilateral, Canada	X				
Bilateral, Switzerland	X	X			
Bilateral, Germany	X				
Bilateral, Denmark	X				
Bilateral, Spain	X				
Bilateral, Finland	X				

Bilateral, France	X				
Bilateral, UK	X				
Bilateral, USA	X				
Bilateral, UAE		X			
Bilateral, South Korea	X				
Bilateral, Italy	X	X			
Bilateral, Netherlands	X				
Bilateral, Norway	X				
Bilateral, New Zealand	X	X			
Bilateral, Sweden	X				
Bilateral, China		X			X
NGOs		X			
US foundations					X
UNICEF		X			
UNFPA		X			
UNAIDs			X		
UNITAID			X		
PAHO				X	
WHO				X	
IDB			X	X	
Gavi			X	X	
The Global Fund				X	
Gates Foundation				X	
World Bank			X	X	
CEPI			X		
EC	X				

Bilateral agencies and European Commission

To track bilateral agencies and the European Commission we used IATI as the main source and supplemented information for bilaterals that do not report to IATI by using UNOCHA, correspondence for Japan, and the data retrieved from the Austrian Development Agency website[1-3]. The bilaterals not reporting COVID-19 projects to IATI include Austria, Greece, Ireland, Japan, Luxembourg, Portugal, and the UAE.

For IATI, we accessed data through the d-portal by first selecting their COVID-19 filter followed by selecting sector groups “Basic health (122)”, “Health, general (121)”, “Population policies / programmes and reproductive health (130)”, and “Emergency response (720)”. IATI’s COVID-19 filter includes projects where “COVID-19” is included in the activity title, activity description, or transaction description or where COVID-19-specific codes are included in humanitarian scope (globally recognised codes) or tag (“COVID-19” code) data fields[4]. To keep only bilateral government and EC projects we kept only the reporting organizations listed in the table above. Not all of the reporting organizations were present in the resulting downloaded dataset. From the d-portal we downloaded the csv files of “Ended projects” and the csv of “Projects ending soon”. These csv files contained project title, reporting organization, total

commitment, total disbursement, and a link to the more detailed project page on the d-portal. To get recipient country, date information, and project descriptions we used a web-scraper and the unique project links to download the information.

Table S3.11.3. Bilateral agency and EC data sources and commitment disbursement ratio breakdown.

Country	Data Source	Reporting organizations	Disbursement commitment median ratio
Australia	IATI	Australia – Department of Foreign Affairs and Trade	1
Austria	ADA website		N/A (no missing disbursement data)
Belgium	IATI	Belgian development agency; Belgian Development Cooperation	1
Canada	IATI	Canada – Global Affairs Canada; Canada – International Development Research Centre; Canada – Department of Finance	1
Denmark	IATI	Denmark Ministry of Foreign Affairs, Danida	1
Finland	IATI	Finland Ministry of Foreign Affairs	1
France	IATI	Agence Francaise de Developpement; France – Ministry for Europe and Foreign Affairs	0.698
Germany	IATI	Germany – Federal Foreign Office; Germany – Ministry for Economic Cooperation and Development	1
Greece	No data		N/A
Ireland	IATI	Ireland – Department of Foreign Affairs and Trade	N/A
Italy	IATI, UNOCHA	AICS – Agenzia Italiana per la Cooperazione allo Sviluppo / Italian Agency for Cooperation and Development	1
Japan	correspondence	Japan International Cooperation Agency; Ministry of Foreign Affairs of Japan	1
Luxembourg	No data		N/A
Netherlands	IATI	Netherlands – Ministry of Foreign Affairs	1
New Zealand	IATI, UNOCHA	New Zealand – Ministry of Foreign Affairs and Trade	1
Norway	IATI	Norad – Norwegian Agency for Development Cooperation	1
Portugal	No data		N/A
South Korea	IATI	Republic of Korea	1
Spain	IATI	Spain – Ministry of Foreign Affairs and Cooperation; Spanish Agency for International Development Cooperation (AECID)	1
Sweden	IATI	Sweden, through Swedish International Development Cooperation	0.939
Switzerland	IATI, UNOCHA	Switzerland – Swiss Agency for Development and Cooperation	1
United Arab Emirates	UNOCHA	Ministry of Foreign Affairs – UAE	1
United Kingdom	IATI	UK – Foreign, Commonwealth and Development Office; UK – Department for Business, Energy and Industrial Strategy	1

		(BEIS); UK – Department for Environment, Food and Rural Affairs	
United States	IATI	United States Agency for International Development (USAID)	0.447
European Commission	IATI	European Commission – Directorate-General for International Cooperation and Development; European Commission – Humanitarian Aid and Civil Protection; European Commission – Neighborhood and enlargement negotiations; European Commission – Service for foreign policy instruments	1

We processed IATI data by removing non-DAH projects, filling in missing disbursement information, disaggregating multi-year projects, and tagging repurposed projects. To remove non-health projects we searched project titles and descriptions for non-health and health keywords. We dropped any projects that contained a non-health keyword (education, food assistance, economic relief/recovery, cash transfer, etc.) and no health keywords. To account for projects missing disbursement information, we calculated disbursement to commitment ratios and used the median for each bilateral donor to calculate project disbursement. The median was chosen since several donors had outlier ratios that resulted in means above 1. Multiple year projects were divided by using a start and end date to calculate the total number of days in a project and determine the disbursement per day. We then multiplied this by the days in each year. The data does not contain a variable specifying repurposed or new money. We assumed that all projects starting prior to 2020 were repurposed in 2020. For the repurposed projects, we determined breaking down total disbursement by year was producing too large of an estimate for USAID and EC projects. For each of these sources, we manually extracted individual disbursement data for 30 projects from IATI, which could not be extracted using the web-scraper. We used the extracted actual COVID-19 disbursements to determine a fraction of the 2020 disbursement that goes to COVID-19. We used the mean of these fractions to scale down all USAID and EC 2020 repurposed projects. Repurposed projects were assigned to a COVID program area of “other” since the project descriptions described the purpose of the original project.

To supplement this data we downloaded all columns and rows available in the UNOCHA dataset and isolated health sector-specific projects where the donor agency was one of the 24 bilateral governments we track. To avoid double counting we dropped UNOCHA projects if the bilateral agency and recipient country were the same as any IATI projects since we believe IATI to be a more comprehensive data source. The data contains separate rows for commitments, disbursements, and pledges from the same project. We match these projects with multiple rows to avoid double counting. We used a 1:1 commitment to disbursement ratio to fill in missing disbursement information. Projects that went to recipient agencies classified in the data as NGOs or UN agencies were tagged as double counting and therefore removed from final dataset.

We received correspondence data for Japan bilateral projects from Shuhei Nomura, Associate Profession at the Graduate School of Medicine, The University of Tokyo, Japan, which included project title, links to project descriptions, date, recipient country, aid type, and commitment. We copied over project descriptions from the Japan MOFA website. Several loan descriptions included social and economic relief in addition to COVID-19 relief. For these loans, a manual keyword search was performed to determine a

health fraction that the loans were multiplied by. For example, the description "This ODA loan is expected to contribute to Bangladesh's social and economic recovery(1), stability (2) and sustainable development(3), as well as curbing the spread of infection(4) in Bangladesh" was assigned as one quarter health as only one of the four key phrases relates to health. We used a 1:1 disbursement to commitment ratio to determine disbursement. Lastly, we downloaded project level data from the Austrian Development Agency website, which included project title, description, recipient country, CRS sector, date, and disbursement information. We searched these projects for COVID-19 keywords to pull out projects for COVID-19. These supplementary projects for Japan and Austria bilateral were all classified as new money based on the date and descriptions of the projects.

We combined this data and ran our keyword search on project descriptions and titles. In order to avoid double-counting spending we have reallocated any money identified as repurposed from our traditional CRS data at the most granular level possible by donor country. After the reallocation we added our COVID data to the top of our reduced traditional envelope in order to obtain the full envelope.

UNAIDS

For UNAIDS, we received data from our contact Jose Antonio, at the agency, on repurposed spending for COVID-19 for recipient countries. These data were used to inform COVID-19 related spending for the agency, and the repurposed money was removed from the total DAH envelope we estimated for 2020 to avoid double-counting. No keyword search was used on this data; all money was manually assigned to 'Maintaining other essential health services and systems', since UNAIDS COVID spending was on maintaining existing programming in the face of the COVID-19 pandemic.

We did not have source information for this channel, and so categorized the source as "Unallocable".

UNICEF

For the UNICEF channel, we identified UNOCHA as the most comprehensive location for UNICEF grant-specific data related to Covid-19[2]. We downloaded all columns and rows available in the database where UNICEF is the destination organization (Figure S3.10.1). To process these data, we subset on the Sector column to isolate "Health". From there, we checked for grants reporting multiple commitments or disbursements. If a grant had multiple rows reporting commitments and disbursements, we merged the rows and preferred disbursements over commitments. We then cleaned up the donor and recipient country names to merge before passing the cleaned and formatted data through our COVID keyword search.

In UNOCHA's data there is a column reporting whether the grant is funded through new or repurposed money – repurposed money being "left-over" amounts intended for another use that has been re-directed to support the pandemic. In our existing data we assume that we have already tracked all repurposed money, so in order to avoid double-counting we reduced the envelope of our traditional UNICEF data by reallocating the health focus area assignments at the most granular level possible – in this case by donor country. After this adjustment, we are able to directly add the UNICEF COVID total amount onto our

existing estimates where the repurposed-tagged money now exactly fills the reduction we made by donor country.

Figure S3.10.1: Sample of UNOCHA's UNICEF Health Data[2]

	Boundary	Source org.	Destination org.	Sector	Amount (US$)
85	Incoming	Asian Development Bank	United Nations Children's Fund	Health	$10,000,000
89	Incoming	Canada, Government of	United Nations Children's Fund	Health	$73,684
90	Incoming	Canada, Government of	United Nations Children's Fund	Health	$74,072
101	Incoming	World Bank	United Nations Children's Fund	Health	$3,869,071
102	Incoming	World Bank	United Nations Children's Fund	Health	$1,500,090
104	Incoming	World Bank	United Nations Children's Fund	Health	$195,300
105	Incoming	World Bank	United Nations Children's Fund	Health	$4,715,074
106	Incoming	World Bank	United Nations Children's Fund	Health	$884,258
109	Incoming	Ireland, Government of	United Nations Children's Fund	Health	$396,902
161	Incoming	United States of America, Government of	United Nations Children's Fund	Health	$389,194
250	Incoming	United States of America, Government of	United Nations Children's Fund	Health	$243,409
270	Incoming	Saudi Arabia (Kingdom of), Government of	United Nations Children's Fund	Health	$4,000,000
288	Incoming	World Bank	United Nations Children's Fund	Health	$384,894
315	Incoming	Yemen Humanitarian Fund	United Nations Children's Fund	Health	$15,999,547
317	Incoming	Japan, Government of	United Nations Children's Fund	Health	$1,084,166
320	Incoming	Ethiopia Humanitarian Fund	United Nations Children's Fund	Health	$25,920
329	Incoming	Canada, Government of	United Nations Children's Fund	Health	$102,799
398	Incoming	Ukraine Humanitarian Fund	United Nations Children's Fund	Health	$139,997
420	Incoming	United States of America, Government of	United Nations Children's Fund	Health	$1,000,000
[illegible]	Incoming	Japan, Government of	United Nations Children's Fund	Health	[illegible]

UNFPA

For the channel UNFPA, we also used the UNOCHA Financial Tracking Service database to access data on the 'COVID-19 Global Humanitarian Response Plan' for grant-specific data related to COVID-19[2]. We downloaded all the grants available in the database, selecting only those in the health sector where UNFPA is the destination organization. Both commitment and disbursement data were available – where there was grant overlap between the two, disbursement data only has been included, to avoid double-counting any grants. Where only commitment data was available, a 1:1 commitment to disbursement ratio was used to estimate disbursement spending. Source information was also available in the dataset.

Following the same strategy as for UNICEF, the value of the repurposed COVID-19 has been removed from our estimates for general UNFPA spending for 2020.

We then used the keyword search described above to categorize each of the grants into the 9 COVID-19 subcategories.

UNITAID

For UNITAID, we received data from correspondence with Nargiza, Mazhidova, a Manager, Impact and Value for Money. This dataset included UNITAID grants, which had a variable tagging those that were COVID related. We followed the same data processing as our regular Unitaid channel pipeline. We split COVID funding into three program areas based on disease area assignments in the data and information from the Unitaid website regarding their contributions to the Access to COVID-19 Tools Accelerator: "Maintaining essential health services and systems", "Case management and treatment", and "National labs and testing"[5]. Grants were counted as new money.

PAHO

For PAHO, we downloaded the latest situation reports from their website and pulled the cumulative numbers of personal protective equipment and testing kits that PAHO had distributed[6]. This included

gloves, surgical mask and respirators, goggles, gowns and COVID-19 PCR tests. We used UNICEF's supply catalog to determine the unit cost for each item by taking the mean of the first four prices listed when we searched for that item[7]. To get our final estimate we multiplied the amount of an item procured by our estimated unit cost. We assigned the total PAHO envelope to "Infection prevention and control" and assumed the disbursement was all new money.

**PPE supply**	**Cost 1**	**Cost 2**	**Cost 3**	**Cost 4**
Per-test cost of first 4 kits listed	$6.75	$4.416	$12.00	$6.00
Per-pair cost of first 4 non-heavy duty, non-child-specific gloves	$ 0.090	$0.35	$0.1008	$0.1211
Per-mask cost of first 4 surgical/respirator masks	$0.161	$2.26	$1.14	$2.2565
Per-gown cost of first 4 surgical gowns	$1.49	$3.71	$15.13	$8.14
Safety goggles	$3.06	$6.9	$1.85	$3.95

WHO

For WHO, we downloaded the underlying data for the Contributions to WHO for COVID-19 visualizations, which reports detailed information on the donors and partners of WHO's COVID-19 response (Figure S3.11.2.)[8]. This data contained donor information, distributed organization, and assigned Strategic Preparedness and Response Plans (SPRPs) that we used for assigning program areas. We pulled recipient country information from the distributed organization variable, which combines the code for the WHO regional office as well as the ISO code of the recipient country. We dropped contributions where the source country was the same as the recipient country as we considered those domestic disbursements. We assigned funds going to headquarters and specific offices that did not indicate a recipient country as "Global" projects. To avoid double counting WHO disbursements, we subtracted disbursements where the source was another one of the channels we track in the aggregation step of our COVID pipeline. For example, the African Development Bank provided money to WHO, which we later took out of our African development bank channel. We used "distributed" variable as our commitment value and "award budget" as our disbursement value. We matched commitments and disbursements by donor, contributor, award ID, award type, and distributed organization since the data contained individual rows for commitments and disbursements. Once we cleaned the data, we ran our COVID keyword search on the SPRP variable. We manually adjusted several keyword assignments. The SPRP "Pandemic supply chain coordination" was changed from being split between "Supply chain and logistics" and "Country-level coordination" to "Supply chain and logistics". "Technical expertise and guidance" was manually assigned to go to "Country-level coordination". "Epidemiological analysis and forecasting" was manually assigned to "Surveillance, rapid-response teams, and case investigation". "Points of entry" was manually assigned to "Country-level coordination". All WHO COVID disbursements was treated as new money.

S3.11.2: Sample of WHO's underlying COVID contributions data

Parent Donor	Contributor	Award	SPRP
All	All	All	All

Underlying Data for COVID Awards and Implementation

Parent Donor	Contributor	Award	Pandemic Flag	Form of Award	Award Classification	Distributed Office	Distributed Organization	SPRP Code	SPRP
Afghanistan	Afghanistan	70360	COVID	In Cash	Standard				N/A
Afghanistan	Afghanistan	70360	COVID	In Cash	Standard				
Afghanistan	Afghanistan	70360	COVID	In Cash	Standard	HQ	HQ/OCC		N/A
Afghanistan	Afghanistan	70360	COVID	In Cash	Standard	HQ	HQ/OCC	A1	A1 - Partner coordi
Afghanistan	Afghanistan	70360	COVID	In Cash	Standard	HQ	HQ/OCC	A1	A1 - Partner coordi
Afghanistan	Afghanistan	70360	COVID	In Cash	Standard	EM	EM_AFG		N/A
Afghanistan	Afghanistan	70360	COVID	In Cash	Standard	EM	EM_AFG		N/A
Afghanistan	Afghanistan	70360	COVID	In Cash	Standard	EM	EM_AFG	B5	B5 - National labor
Afghanistan	Afghanistan	70360	COVID	In Cash	Standard	EM	EM_AFG	B5	B5 - National labor
Afghanistan	Afghanistan	70360	COVID	In Cash	Standard	EM	EM_AFG	B5	B5 - National labor
Afghanistan	Afghanistan	70360	COVID	In Cash	Standard	EM	EM_AFG	B5	B5 - National labor
Afghanistan	Afghanistan	70360	COVID	In Cash	Standard	EM	EM_AFG	B5	B5 - National labor
Afghanistan	Afghanistan	70360	COVID	In Cash	Standard	EM	EM_AFG	B8	B8 - Operational su
Afghanistan	Afghanistan	70360	COVID	In Cash	Standard	EM	EM_AFG	B8	B8 - Operational su
Afghanistan	Afghanistan	70360	COVID	In Cash	Standard	EM	EM_AFG	B8	B8 - Operational su
Afghanistan	Afghanistan	70360	COVID	In Cash	Standard	EM	EM_AFG	B8	B8 - Operational su
Afghanistan	Afghanistan	70360	COVID	In Cash	Standard	EM	EM_AFG	B8	B8 - Operational su
Afghanistan	Afghanistan	70697	COVID	In Cash	Standard				N/A
Afghanistan	Afghanistan	70697	COVID	In Cash	Standard				
Afghanistan	Afghanistan	70697	COVID	In Cash	Standard	HQ	HQ/BOS		N/A
Afghanistan	Afghanistan	70697	COVID	In Cash	Standard	EM	EM/BOS		N/A
Afghanistan	Afghanistan	70697	COVID	In Cash	Standard	EM	EM/BOS		N/A
Afghanistan	Afghanistan	70697	COVID	In Cash	Standard	EM	EM/BOS	A7	A7 - Pandemic sup
Total									

Contributors | Financial Flow (SPRP) | SPRP Pillars | Country SPRP Funding | Pooled Funds Report | Raw Data

World Bank

We received health-related disbursement data from correspondence with Miyuki Parris at the World Bank. This dataset included funding from both the IDA and IBRD branches for COVID-19 specific projects. We followed the same initial data processing as the general World Bank channel workflow. This included disaggregating projects with multiple recipients equally, dropping high-income countries, and disaggregating funding from projects with commitments from IBRD and IDA proportionately. COVID-19 related projects were isolated for separate data processing.

We applied the keyword search described previously to each project's respective individual component descriptions obtained from the World Bank Project Database where available[9]. We extracted the component descriptions from Project Appraisal Documents (PAD) from the World Bank Project Database (Figure S3.11.3). We allocated funding to these components according to the proportion of the project's funding committed to each component. After disaggregating money by program area for each component, we aggregated the components together for each project. This process ensured that the disaggregation by program area best reflected the funding priorities in each project. Where individual component descriptions were not available, we ran the keyword search on the project development objective. We obtained information regarding commitments, and grant and loan disaggregation for IDA projects from Project Appraisal Documents as well (Figure X2.). We assigned all IBRD funding as loans. We used this webpage to disaggregate between new and repurposed money[10]. We assigned projects listed as, "benefiting from the dedicated COVID-19 Fast-Track Facility" as new money. We assigned projects listed as "benefiting from other forms of finance/redeploying of existing projects" as repurposed money.

To assign donor countries for IDA, we used a weighted average of historic contributions (2016 to 2018) to IDA from countries and from IBRD. This is unnecessary for IBRD as the branch receives its income entirely from debt repayments. Through information from correspondence, we were unable to disaggregate funding as new or repurposed money.

Figure S3.11.3

38. **The project will comprise the following components**:

39. **Component 1. Case detection and management [US$8.5 million]:** Activities supported by this component include: establishing and upgrading laboratory, isolation and treatment centers and equipping them with medical supplies and furniture and network installation[8]. NIPH will be upgraded; diagnostic capacity of the four provincial laboratories as well as laboratories attached to the 21 provincial referral hospitals will be built[9]; and isolation and treatment centers in all 25 municipal/provincial referral hospitals will be upgraded.

40. **Component 2. Medical Supplies and Equipment [US$6.5 million]**: This component will finance the procurement of medical supplies and equipment needed for activities outlined in the COVID-19 Master Plan, including business continuity of essential services, such as (i) case management; and (ii) infection prevention and control. Specifically, items procured will include drugs and medical supplies for case management and infection prevention. This component will also allow for flexibility to allocate resources for the purchasing of essential pharmaceutical (medicines and vaccines) and medical supplies as the availability in the country becomes reduced due to the economic impact of the pandemic and the existing mechanisms are insufficient to address the critical health system needs.

Figure S3.11.4.

IDA Resources (in US$, Millions)

	Credit Amount	Grant Amount	Guarantee Amount	Total Amount
**Cambodia**	20.00	0.00	0.00	20.00
National PBA	20.00	0.00	0.00	20.00
**Total**	**20.00**	**0.00**	**0.00**	**20.00**

Expected Disbursements (in US$, Millions)

WB Fiscal Year	2020	2021	2022	2023
Annual	3.00	14.00	2.70	0.30
Cumulative	3.00	17.00	19.70	20.00

AfDB

For the African Development Bank (AfDB), we received data from a contact, Yomi Ayodabo, Chief Fiscal supervisor at AfDB, on all loans and grants distributed in 2020. Any loans or grants which reference COVID-19 in their title or description were selected. All of these loans and grants were classified by AfDb as “Multi-sector” and had both commitment and disbursement information. Reviewing the project description for each project (available on the website)[11,12], we attributed between 0% and 66% of each project’s spending to health spending. Based on detailed project descriptions on their COVID-related projects website, we classified each project as a loan or grant, and extracted descriptions of the health programs to use in the keyword search. [11,12]

We then used the keyword search described above to categorize each of the grants into the nine COVID-19 program areas.
For the source information, we used replenishment data from the general DAH pipeline to inform proportions, and then applied those proportions to the reported COVID-19 health spending to estimate country-specific donations.

ADB

For the Asian Development Bank (ADB), we received data from our contact Rouselle F. Lavado, at the bank, on all health-related loans and grants they distributed. Any loans or grants which reference COVID-19 in their title or description are removed from the general ADB spending pipeline, and a separate dataset is created using only COVID-19 loans and grants. For these grants and loans, we evaluated the proportion of each which was designated for health sector spending in the "Report and Recommendation of the President" for each project on the ADB website[13], and scaled the total loan or grant quantity down by this proportion. This is so that we only include health spending in our COVID-19 estimates. Both commitment and 2020 disbursement data is available for this dataset. Both commitment and 2020 disbursement data is available for all projects in this dataset. For the purposes of estimating 2020 COVID-19 spending, we relied on the disbursement information.

We then use health program descriptions in the "Report and Recommendation of the President" for each grant and loan to run the keyword search described above. This categorizes each of the grants/loans into the nine COVID-19 program areas.

For the source information, we used data from the general DAH pipeline to inform proportions, and then applied those proportions to the reported COVID-19 health spending to estimate country-specific donations.

IDB

For the Inter-American Development Bank (IDB), we received data from our contact Matilde Neret, Contract and Budget Officer at Inter-American Development Bank, on all health-related loans and grants disbursed. Any loans or grants which reference COVID-19 in their title or description are removed from the general IDB spending pipeline, and a separate dataset is created using only COVID-19 loans and grants.

We then used the keyword search described above to categorize each of the grants into the nine COVID-19 subcategories.

GAVI

For the GAVI channel, we used a three-pronged approach to estimating COVID DAH contributions. First, we utilized data we received through correspondence with Alain McLaren, a Senior Analyst at GAVI, on prepayments and firm order commitments (FOCs) to estimate COVID Health System Strengthening and COVAX non-country specific totals. This dataset includes the two aforementioned programs, a note about their global nature, and the amount of money going towards each. Second, we manually extracted country-specific repurposed money totals from GAVI Situation Reports published monthly on their website[14]. Third, we manually extracted country-specific COVAX commitments from a

regularly published document on GAVI's website titled "COVAX AMC Donors Table"[15]. For each of these sources we used distinct methods based on available information on each.

For the prepayments and FOCs data the dataset attests that the reported funds are new money and that the recipients of these prepayments have yet to be determined – which we classify as global. For the GAVI Situation Reports, the Situation Report documents note that the "amount approved for support" column which we extract is entirely repurposed money so we treated it as such and reallocated as necessary (details below). For the COVAX Advanced Market Commitment table, we tagged all reported spending as new money according to COVAX not reporting otherwise. Finally, we appended these three datasets and passed the data through our COVID keyword search.

Similar to the UNICEF channel and others, in order avoid double-counting spending we have reallocated any money identified as repurposed from our traditional GAVI data at the most granular level possible – in this case by donor country. After the reallocation, we added our COVID data to the top of our reduced traditional envelope in order to obtain the full envelope.

The Global Fund

For the Global Fund, we directly pulled country-specific disbursements and repurposed resources information from the "Funding Approved for COVID-19 Response" report published on the GFATM website[16]. This dataset contains tables on country-specific new money disbursements awarded as well as country-specific repurposed money utilized.

Little formatting was needed for this source other than splitting regional and multi-country observations, where money was divided evenly between all countries represented in each region – country lists in each region were found using the Global Fund's data explorer[17]. For example, the Multicountry Americas (Andean) region was evenly split between Colombia, Ecuador, Peru, and Venezuela.

Like other channels, we made adjustments to our traditional Global Fund data to account for the repurposed money identified in our data source. The reallocation occurred at the recipient country level, and was again filled by the tagged repurposed money after appending our Global Fund COVID dataset.

CEPI

For the CEPI channel, we received COVID-19 data through correspondence with Tuva Stolen and Mads Hogholen, the Head of Controlling and Director of Finance and Operations at CEPI, respectively. The dataset we received includes donor-specific COVID disbursement amounts which we confirmed with our contacts are all new money observations. After thoroughly reviewing CEPI's reported activities in 2020 we deemed all COVID-19 spending as going to our R&D COVID program area because CEPI as an organization is focused on R&D, and lead on the COVAX facility on development of the vaccine. We then run our COVID-19 keyword search.

NGOs

For non-governmental organizations, we compiled data from UNOCHA's Financial Tracking System (FTS), which reports continuously updated data on humanitarian funding flows[2]. They receive report

contributions from government donors, UN agencies, NGOs and other humanitarian actors[2]. The data includes project level information, which included source organization, source type, recipient country, and whether the money was new or repurposed. We kept records where the listed recipient agency type included "NGO" and sectors that included "health", "multi-sector", "gender", "covid", or "child protection" in the sector title after reviewing projects from these sectors and determining they were DAH. The data contains separate rows for commitments, disbursements, and pledges from the same project. We match these projects with multiple rows to avoid double counting. We used a 1:1 commitment to disbursement ratio to fill in missing disbursement information.

We then used the keyword search described above to categorize NGO projects into one of the eight COVID program areas.

In order to avoid double-counting spending we have reallocated any money identified as repurposed from our traditional data at the most granular level possible by NGO agency. After the reallocation, we added our COVID data to the top of our reduced traditional envelope in order to obtain the full envelope.

Gates Foundation

For the Bill and Melinda Gates Foundation, we compiled our data using a web scraper applied to their online Grant Database and extracted grants in 2020, which are returned using the search terms: CORONAVIRUS, COVID, SARS, SARS COV 2, NCOV[18]. The web scraper returned a little under 500 unique grants, the data for which contained only commitment information, but had rich project descriptions and duration information (Figure S3.11.5.). Upon reviewing the documentation of their grant database we identified all extracted projects as new money according to how they sort year-specific results in the tool.

To process the scraped data, we passed the data through our COVID keyword search.

Figure S3.11.5.: Snapshot of Project from BMGF Online Grant Database

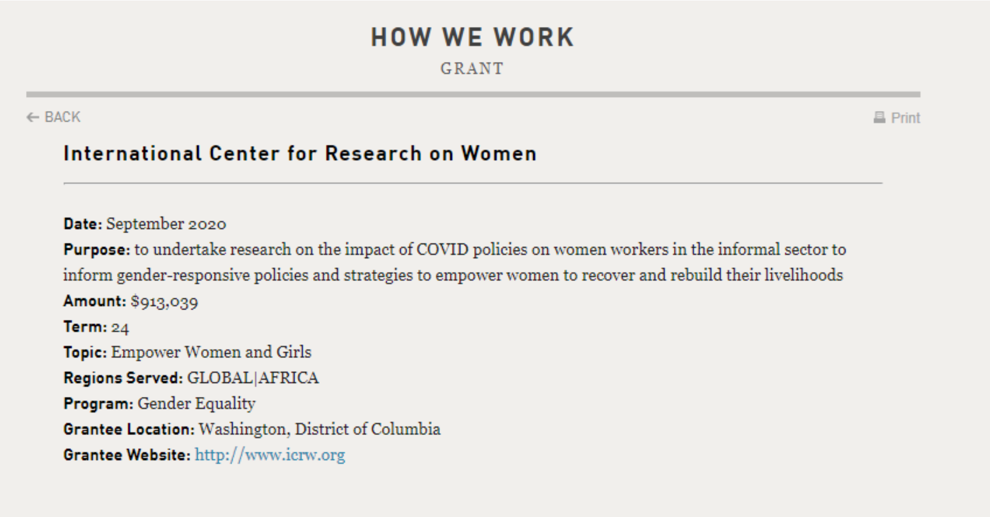

China

For the China Bilateral channel, we used a three-tiered approach to estimating COVID contributions. First, we pull data from UNOCHA where the Source is the Chinese Government[2]. UNOCHA tags new money and repurposed commitment and disbursement observations, and grants with multiple observations of commitments or disbursements have been matched in an identical fashion to the UNCIEF channel. Second, we identified our regular channel National Health Commission estimates as being entirely repurposed for COVID relief; this assumption based on the "White paper on China's action combating COVID-19 which said 56 medical teams that are abroad have assisted in covid-19 response, including providing technical assistance and health education, and delivered over 400 training sessions[19]. recommendation coming from an external FGH collaborator, Yingxi Zhao, who has worked with us previously on our estimation of China's bilateral contributions[20]. Finally, we calculated a back of the envelope estimate of the Chinese government's global PPE, medical equipment and additional medical team contributions, which we consider to be new money.

To format the UNOCHA data, we began by subsetting on the Sector column to isolate "Health". From there, we reformatted column data types to make manipulations simpler, and checked for grants reporting multiple commitments or disbursements. If a grant had multiple rows reporting commitments and disbursements, we merged the rows and preferred disbursements over commitments as to not double-count spending. We then run our COVID keyword search.

To process the NHC data, we extracted the value of DAH contribution from our regular channel data and append it to our COVID data, and then passed it through our COVID keyword search. All NHC contributions were assumed to be medical workforce support hence the reallocation as COVID-related contributions.

To calculate our back of the envelope estimate of the Chinese government's global PPE, medical equipment and medical team contributions, we began by reviewing press releases from various Chinese ministries to gauge the number of batches of PPE and the number of medical teams sent globally. We estimated that 34 additional medical teams were dispatched and 200 out of 283 batches of PPE were send to LMICs[21, 22]. We then estimated a round unit cost per PPE/medical team batch, assuming that each batch contained roughly the same equipment, volume, and staffing. Finally, we multiplied the estimated number of batches by the estimated unit cost and report $60,000,000 as the total PPE, medical equipment and medical team contributions.

To account for repurposed money, we performed a similar reallocation of traditional channel money as noted in other channels – here by Chinese agency. We completely reallocated the 2020 NHC traditional channel amount to COVID, and otherwise reallocated UNOCHA's reported repurposed money also by donor agency.

US Foundations

We utilized purchased dataset from CANDID[23] and isolated USA originating grants funding for COVID-19 related purposes. We removed grants without health-related codes from the data processing. If a grant had an intended beneficiary, we labeled this as the recipient country or countries. If there was no intended beneficiary, we labeled the direct recipient country of the grant as the recipient country. If the grant identified more than one country as the recipient, we divided the funding equally between the recipients. We excluded high-income recipient countries.

We flagged recipient agencies that we track through other channels to avoid double counting this funding. We flagged descriptions that specify any repurposing of funding as repurposed money. We applied the keyword search to descriptions included with each individual grant.

Table S3.11.4. Top recipients of DAH for COVID-19

Recipient	World Bank income group	GBD super-region	Development assistance received for COVID-19 (millions 2020 USD)	Percent of DAH for COVID-19
India	LM	South Asia	1402·5	10·3
Indonesia	UM	Southeast Asia, East Asia, and Oceania	694·2	5·1
Philippines	LM	Southeast Asia, East Asia, and Oceania	430·5	3·1
Pakistan	LM	South Asia	321·9	2·4
Bangladesh	LM	South Asia	260·4	1·9
Argentina	UM	Latin America and Caribbean	242·4	1·8
Nigeria	LM	Sub-Saharan Africa	236·0	1·7
Morocco	LM	North Africa and Middle East	215·7	1·6
South Africa	UM	Sub-Saharan Africa	211·6	1·5
Kenya	LM	Sub-Saharan Africa	200·8	1·5
Ethiopia	L	Sub-Saharan Africa	195·8	1·4
Afghanistan	L	North Africa and Middle East	176·5	1·3
Ecuador	UM	Latin America and Caribbean	167·5	1·2
Democratic Republic of the Congo	L	Sub-Saharan Africa	166·4	1·2
Cambodia	LM	Southeast Asia, East Asia, and Oceania	162·4	1·2
Nepal	LM	South Asia	158·4	1·2
Yemen	L	North Africa and Middle East	155·0	1·1
Papua New Guinea	LM	Southeast Asia, East Asia, and Oceania	126·6	0·9
Kazakhstan	UM	Central Europe, Eastern Europe, and Central Asia	114·4	0·8
Syrian Arab Republic	L	North Africa and Middle East	107·6	0·8
Niger	L	Sub-Saharan Africa	105·7	0·8
Mozambique	L	Sub-Saharan Africa	101·3	0·7
Uganda	L	Sub-Saharan Africa	94·5	0·7
Sri Lanka	LM	Southeast Asia, East Asia, and Oceania	93·1	0·7
Tunisia	LM	North Africa and Middle East	92·2	0·7

Myanmar	LM	Southeast Asia, East Asia, and Oceania	88·8	0·6
Ghana	LM	Sub-Saharan Africa	85·6	0·6
Sudan	L	North Africa and Middle East	85·5	0·6
Mongolia	LM	Central Europe, Eastern Europe, and Central Asia	84·7	0·6
Iran (Islamic Republic of)	UM	North Africa and Middle East	80·8	0·6
Zimbabwe	LM	Sub-Saharan Africa	79·7	0·6
Burkina Faso	L	Sub-Saharan Africa	79·1	0·6
Côte d'Ivoire	LM	Sub-Saharan Africa	78·4	0·6
South Sudan	L	Sub-Saharan Africa	76·4	0·6
Somalia	L	Sub-Saharan Africa	74·3	0·5
Malawi	L	Sub-Saharan Africa	70·3	0·5
Colombia	UM	Latin America and Caribbean	68·7	0·5
Senegal	LM	Sub-Saharan Africa	68·2	0·5
Iraq	UM	North Africa and Middle East	65·0	0·5
Cameroon	LM	Sub-Saharan Africa	63·8	0·5
Turkey	UM	North Africa and Middle East	60·9	0·4
Mali	L	Sub-Saharan Africa	58·5	0·4
Palestine	LM	North Africa and Middle East	57·9	0·4
China	UM	Southeast Asia, East Asia, and Oceania	57·0	0·4
Central African Republic	L	Sub-Saharan Africa	55·0	0·4
Jordan	UM	North Africa and Middle East	52·6	0·4
Lebanon	UM	North Africa and Middle East	47·9	0·4
Ukraine	LM	Central Europe, Eastern Europe, and Central Asia	46·0	0·3
Viet Nam	LM	Southeast Asia, East Asia, and Oceania	43·1	0·3
Chad	L	Sub-Saharan Africa	41·4	0·3
Rwanda	L	Sub-Saharan Africa	40·4	0·3
Haiti	L	Latin America and Caribbean	38·6	0·3
United Republic of Tanzania	LM	Sub-Saharan Africa	38·4	0·3
Zambia	LM	Sub-Saharan Africa	36·8	0·3
Tajikistan	L	Central Europe, Eastern Europe, and Central Asia	34·0	0·2
Sierra Leone	L	Sub-Saharan Africa	31·9	0·2
Lao People's Democratic Republic	LM	Southeast Asia, East Asia, and Oceania	30·9	0·2

Georgia	UM	Central Europe, Eastern Europe, and Central Asia	30·6	0·2
Guinea	L	Sub-Saharan Africa	30·5	0·2
Uzbekistan	LM	Central Europe, Eastern Europe, and Central Asia	29·8	0·2
Djibouti	LM	Sub-Saharan Africa	26·7	0·2
Libya	UM	North Africa and Middle East	26·0	0·2
Benin	LM	Sub-Saharan Africa	25·8	0·2
Bosnia and Herzegovina	UM	Central Europe, Eastern Europe, and Central Asia	25·5	0·2
Togo	L	Sub-Saharan Africa	25·4	0·2
Maldives	UM	Southeast Asia, East Asia, and Oceania	25·0	0·2
Paraguay	UM	Latin America and Caribbean	24·5	0·2
Congo	LM	Sub-Saharan Africa	23·9	0·2
Serbia	UM	Central Europe, Eastern Europe, and Central Asia	22·5	0·2
Mauritania	LM	Sub-Saharan Africa	21·3	0·2
Madagascar	L	Sub-Saharan Africa	21·1	0·2
Peru	UM	Latin America and Caribbean	20·9	0·2
Venezuela (Bolivarian Republic of)	UM	Latin America and Caribbean	20·8	0·2
Liberia	L	Sub-Saharan Africa	20·7	0·2
Nicaragua	LM	Latin America and Caribbean	20·6	0·2
Egypt	LM	North Africa and Middle East	20·4	0·1
Brazil	UM	Latin America and Caribbean	18·4	0·1
Thailand	UM	Southeast Asia, East Asia, and Oceania	18·3	0·1
Malaysia	UM	Southeast Asia, East Asia, and Oceania	17·4	0·1
Gambia	L	Sub-Saharan Africa	16·6	0·1
Angola	LM	Sub-Saharan Africa	16·5	0·1
Mexico	UM	Latin America and Caribbean	16·5	0·1
Burundi	L	Sub-Saharan Africa	15·9	0·1
Guatemala	UM	Latin America and Caribbean	15·9	0·1
Lesotho	LM	Sub-Saharan Africa	15·6	0·1
Cabo Verde	LM	Sub-Saharan Africa	15·3	0·1
Kyrgyzstan	LM	Central Europe, Eastern Europe, and Central Asia	14·7	0·1
Fiji	UM	Southeast Asia, East Asia, and Oceania	14·5	0·1
Honduras	LM	Latin America and Caribbean	14·3	0·1

Republic of Moldova	LM	Central Europe, Eastern Europe, and Central Asia	13·2	0·1
Gabon	UM	Sub-Saharan Africa	13·1	0·1
Belarus	UM	Central Europe, Eastern Europe, and Central Asia	13·0	0·1
Vanuatu	LM	Southeast Asia, East Asia, and Oceania	13·0	0·1
eSwatini	LM	Sub-Saharan Africa	12·8	0·1
Samoa	UM	Southeast Asia, East Asia, and Oceania	12·7	0·1
Armenia	UM	Central Europe, Eastern Europe, and Central Asia	12·6	0·1
Bhutan	LM	South Asia	12·6	0·1
Solomon Islands	LM	Southeast Asia, East Asia, and Oceania	12·3	0·1
Timor-Leste	LM	Southeast Asia, East Asia, and Oceania	12·2	0·1
North Macedonia	UM	Central Europe, Eastern Europe, and Central Asia	11·4	0·1
Namibia	UM	Sub-Saharan Africa	10·5	0·1
Guinea-Bissau	L	Sub-Saharan Africa	10·0	0·1
Azerbaijan	UM	Central Europe, Eastern Europe, and Central Asia	9·1	0·1
Cuba	UM	Latin America and Caribbean	8·7	0·1
Bolivia (Plurinational State of)	LM	Latin America and Caribbean	8·4	0·1
Dominican Republic	UM	Latin America and Caribbean	8·2	0·1
Botswana	UM	Sub-Saharan Africa	7·5	0·1
Kiribati	LM	Southeast Asia, East Asia, and Oceania	7·5	0·1
El Salvador	LM	Latin America and Caribbean	7·5	0·1
Turkmenistan	UM	Central Europe, Eastern Europe, and Central Asia	7·5	0·1
Tonga	UM	Southeast Asia, East Asia, and Oceania	6·8	0·0
Sao Tome and Principe	LM	Sub-Saharan Africa	6·7	0·0
Montenegro	UM	Central Europe, Eastern Europe, and Central Asia	6·2	0·0
Algeria	LM	North Africa and Middle East	6·1	0·0
Tuvalu	UM	Southeast Asia, East Asia, and Oceania	5·5	0·0
Albania	UM	Central Europe, Eastern Europe, and Central Asia	5·4	0·0

Marshall Islands	UM	Southeast Asia, East Asia, and Oceania	5·2	0·0
Costa Rica	UM	Latin America and Caribbean	4·9	0·0
Micronesia (Federated States of)	LM	Southeast Asia, East Asia, and Oceania	4·2	0·0
Jamaica	UM	Latin America and Caribbean	4·0	0·0
Comoros	LM	Sub-Saharan Africa	3·6	0·0
Eritrea	L	Sub-Saharan Africa	2·7	0·0
Democratic People's Republic of Korea	L	Southeast Asia, East Asia, and Oceania	2·7	0·0
Equatorial Guinea	UM	Sub-Saharan Africa	2·6	0·0
Niue	H	Southeast Asia, East Asia, and Oceania	1·6	0·0
Suriname	UM	Latin America and Caribbean	1·0	0·0
Guyana	UM	Latin America and Caribbean	0·9	0·0
Belize	UM	Latin America and Caribbean	0·7	0·0
Tokelau	UM	Southeast Asia, East Asia, and Oceania	0·5	0·0
Bulgaria	UM	Central Europe, Eastern Europe, and Central Asia	0·3	0·0
Grenada	UM	Latin America and Caribbean	0·3	0·0
Saint Lucia	UM	Latin America and Caribbean	0·3	0·0
Saint Vincent and the Grenadines	UM	Latin America and Caribbean	0·3	0·0
Russian Federation	UM	Central Europe, Eastern Europe, and Central Asia	0·2	0·0
Dominica	UM	Latin America and Caribbean	0·004	0·0
Global		Global	3215·5	23·5
Unallocable		Unallocable	1331·7	9·7

References:

1. development-portal.org. http://d-portal.org/ctrack.html#view=search (accessed Jan 22, 2021).
2. Financial Tracking Service. https://fts.unocha.org/ (accessed Jan 22, 2021).
3. Austrian Development Agency. https://www.entwicklung.at/en/projects/all-projects (accessed Jan 22, 2021).
4. Version 1 Guidance Accessing and Using COVID-19 Data. Google Docs. https://docs.google.com/document/u/1/d/1hV_Bk2_VEQmrbSPbp3vE0Rho-gZtOJBSpLkOYInTnOY/edit?usp=embed_facebook (accessed Jan 22, 2021).
5. UNITAID. Unitaid and COVID-19 - Unitaid. https://unitaid.org/covid-19/#en (accessed Jan 22, 2021).

6. PAHO/WHO. COVID-19 Situation Reports. https://www.paho.org/en/covid-19-situation-reports (accessed Jan 22, 2021).
7. UNICEF. UNICEF Supply Catalogue. https://supply.unicef.org/ (accessed Jan 22, 2021).
8. WHO. Contributions to WHO for COVID-19. https://app.powerbi.com/view?r=eyJrIjoiNzNmNTRkMWEtNmZjMS00NzdjLWEyMDYtYWExYzA4NzVhZGQwIiwidCI6ImY2MTBjMGI3LWJkMjQtNGIzOS04MTBiLTNkYzI4MGFmYjU5MCIsImMiOjh9 (accessed Jan 22, 2021).
9. The World Bank. Projects & Operations. World Bank. https://projects.worldbank.org/en/projects-operations/projects-home (accessed Jan 22, 2021).
10. The World Bank. World Bank Group's Operational Response to COVID-19 (coronavirus) – Projects List. World Bank. https://www.worldbank.org/en/about/what-we-do/brief/world-bank-group-operational-response-covid-19-coronavirus-projects-list (accessed Jan 22, 2021).
11. African Development Bank. AfDB Data Portal. https://projectsportal.afdb.org/dataportal/ (accessed Jan 22, 2021).
12. African Development Bank. Our COVID-19 response to date. African Development Bank - Building today, a better Africa tomorrow. 2020; published online June 24. https://www.afdb.org/en/news-events/our-covid-19-response-date (accessed Jan 22, 2021).
13. Asian Development Bank. Projects and Tenders. Asian Development Bank. https://www.adb.org/projects (accessed Jan 22, 2021).
14. Gavi, The Vaccine Alliance. COVID-19 situation reports. https://www.gavi.org/news-resources/document-library/covid-19-situation-reports (accessed Jan 22, 2021).
15. Gavi, The Vaccine Alliance. COVAX Facility. https://www.gavi.org/covax-facility (accessed Jan 22, 2021).
16. The Global Fund. COVID-19 Operational Response. https://www.theglobalfund.org/en/covid-19/ (accessed Jan 22, 2021).
17. The Global Fund. Map - The Global Fund Data Explorer. https://data.theglobalfund.org/investments/home (accessed Jan 22, 2021).
18. Bill & Melinda Gates Foundation. Grantmaking - Awarded Grants. https://www.gatesfoundation.org/How-We-Work/Quick-Links/Grants-Database (accessed Jan 22, 2021).
19. The State Council, The People's Republic of China. China's action against the new coronavirus epidemic. http://www.gov.cn/zhengce/2020-06/07/content_5517737.htm (accessed Jan 22, 2021).
20. Micah AE, Zhao Y, Chen CS, et al. Tracking development assistance for health from China, 2007–2017BMJ Global Health 2019;4:e001513.
21. Xinhuanet. Xinhua International Current Review: The World Reverberation of the Great Anti-epidemic Spirit. http://www.xinhuanet.com/2020-09/09/c_1126473118.htm (accessed Jan 22, 2021).
22. People's Daily. Fully demonstrate China's conscious responsibility as a large responsible country (by everyone). http://paper.people.com.cn/rmrb/html/2020-09/24/nw.D110000renmrb_20200924_2-09.htm (accessed Jan 22, 2021).
23. Candid. Candid. https://candid.org/ (accessed Jan 22, 2021).

Forecasting Health Spending from 2019 through 2050

Supplementary Appendix

Global Burden of Disease Financing Global Health Collaborator Network

Updated: Thursday 15th April, 2021

Contents

S4.1 Data **2**

S4.1.1 Summary of Data Sources 2

S4.1.2 World Health Organization's Global Health Expenditure Database 2

S4.1.3 WB WDI, IMF WEO, PWT, and Maddison 3

S4.1.4 Institute for Health Metrics and Evaluation's Development Assistance for Health Database 4

S4.1.5 Institute for Health Metrics and Evaluation's Global Burden of Disease Study 2017 4

S4.2 Covariates **6**

S4.2.1 List of Forecasted Variables 6

S4.2.2 Covariates Used for Forecasting 6

S4.3 Ensemble Modeling **8**

S4.3.1 Sub-model Setup 8

S4.3.2 Covariates 8

S4.3.3 Convergence to Global Growth Rates 8

S4.3.4 Specifications 9

S4.4 Inclusion and Exclusion Criteria **11**

S4.5 Creating the Forecasts **12**

S4.5.1 Ranking Sub-Models 12

S4.5.2 Uncertainty Estimation 12

S4.6 Ad-hoc Draws Correlation **14**

S4.6.1 Motivation 14

S4.6.2 Bivariate Correlated Distributions 14

S4.7 Future Health Scenarios **14**

S4.7.1 Long-term Growth Regressions 15

S4.7.2 Forecasting Better and Worse Scenarios 15

S4.7.3 Uncertainty Estimation 16

S4.8 Package and Architecture **17**

S4.8.1 Architecture 17

S4.8.2 Template Model Builder 17

References **18**

S4.9 Tables and Figures **20**

Introduction

The objective of this study is to provide data on future health spending patterns that can guide decision-makers. These analyses produced comprehensive and comparable set of gross domestic product and all-sector government spending estimates, followed by all the components of a country's total health expenditure. Additionally, using observed past trends, we predicted the possible trajectories of the covariates of interest conditional on following an optimistic and pessimistic pattern based on global rates of change.

The purpose of this appendix is to describe in detail the methodology used in our analyses. Subsequent sections contain information on all data sources, ensemble forecasting strategies, inclusion criteria, and uncertainty estimation used to generate our estimates, as well as our guidelines on how our future health scenarios analyses were conducted.

SECTION 4. FORECASTING HEALTH SPENDING FROM 2019 through 2050

S4.1 Data

S4.1.1 Summary of Data Sources

We used data from seven sources for the analyses:

- World Health Organization's (WHO) Global Health Expenditure Database
- World Bank (WB) World Development Indicators database (WDI)
- International Monetary Fund's (IMF) World Economic Outlook (WEO) October 2020 database
- Penn World Tables 9.1 (PWT)
- Angus Maddison Project Database 2020
- Institute for Health Metrics and Evaluation's Development Assistance for Health Database 2020 (IHME)
- Institute for Health Metrics and Evaluation's Global Burden of Disease Study 2017 (IHME GBD 2017)

Specifically, we collected health expenditure information on all available sources that is comparable across countries and complete for most countries from WHO and IHME, and demographic data from the WPP, while the underlying data for producing gross domestic product (GDP) and general government expenditure (GGE) were extracted from the IMF, WB, and PWT. Table S4.1 presents the definitions for the various health expenditure sources.

S4.1.2 World Health Organization's Global Health Expenditure Database

We used Global Health Expenditure Database (GHED) data from the WHO to generate our estimates, which spans 192 countries between 2000 and 2018 (in the January 2nd, 2021 version). From the GHED, we pulled the following variables:

(i) Compulsory prepayment (Other, and unspecified, than FS.3)

(ii) Other revenues from NPISH n.e.c.

(iii) Other revenues from corporations n.e.c.

(iv) Other revenues from households n.e.c.

(v) Social insurance contributions

(vi) Transfers from government domestic revenue (allocated to health purposes)

(vii) Gross Domestic Product

(viii) Voluntary prepayment

To ensure we were using the best possible data, we downloaded the metadata for each data point for all of the indicators from the GHED website. We used the metadata to decide how each given data point should be weighted, from 1 to 5, being applied as inverse variance weights. We established guidelines for the metadata that informed how the underlying data points should be weighted, giving priority to factors such as complete, documented source information and penalizing factors such as having been derived or estimated. We adjusted these data by converting them from current local currency to 2020 US dollars. Details of the weighting guidelines and classification are explained in the supplementary appendix our retrospective analysis paper.

Once we have an incomplete set of data points for the health expenditure variables we are interested in forecasting, we used Spatiotemporal Gaussian process regression (ST-GPR) to model the full time-series for each variable across a total of 204 countries. ST-GPR is a stochastic modeling technique that is designed to detect signals amidst noisy data. Unlike classical linear models that assume that the trend underlying data follows a definitive functional form, GPR assumes that the specific trend of interest follows a Gaussian Process, existing with some pointwise mean and covariance function [1]. The covariates that were used in order to determine the initial fit of our health expenditure variables are: lag-distributed income, all-sector government expenditure per capita, and proportion of total population over the age of 64; using a Matérn covariance function for the distribution of the Gaussian process. Once we determined an initial prediction of our dependent variables, using the variability of data across regions, Gaussian process regressions (GPR) were run in order to estimate 1,000 draws of each country-year estimate per metric.

S4.1.3 WB WDI, IMF WEO, PWT, and Maddison

The WDI Database provides data on a wide range of development related variables, including data on GDP and GDP per capita. Data series in this database begin in 1960 and span

through the present. The IMF's WEO Database provides data on various macroeconomic indicators. Macroeconomic series data are available from 1980 to present. The PWT is a database that provides real national accounts data for 182 countries and territories from 1950 to 2017. The Maddison Project database provides historical GDP, GDP per capita, and population data dating as far back as Roman times. We utilized GDP per capita as a primary covariate to produce forecasts. GDP per capita from 1950 through 2021 was constructed using the method described in *James et al* [2]. The method utilized extracted data from a number of sources (IMF, WB, PWT and Maddison), and used multiple random effects models to estimate a mean GDP per capita series to be used in our analysis. Similarly, we used the same methodology to produce a mean general government expenditure (GGE) per GDP series, from 1980 through 2020.

S4.1.4 Institute for Health Metrics and Evaluation's Development Assistance for Health Database

Development assistance for health estimates were obtained from the Institute for Health Metrics and Evaluation's Development Assistance for Health Database. To generate these estimates, IHME collected audited budgets, annual reports, and project records from the primary development agencies providing assistance for the health sector. These records are augmented by information acquired via correspondence, and are standardized and compiled to provide a comprehensive perspective on international financial flows for health. These estimates are tracked backward to the source of the funds and forward to the country recipient, and are available from 1990 through 2020 and 2018, respectively.

S4.1.5 Institute for Health Metrics and Evaluation's Global Burden of Disease Study 2017

Covariates used in forecasting health spending, such as populations and educational attainment per capita [3, 4], were obtained from the Institute for Health Metrics and Evaluation's Global Burden of Disease Study 2017. Population forecasts used were from IHME's Future Health Scenarios work, which uses a cohort component method of projection (CCMP [5]), and takes forecasts of age-specific fertility rates, mortality, migration, and the sex ratio at birth as inputs [6]. Age-specific fertility forecasts were projected by modeling completed cohort fertility as a function of met need for contraception and maternal education. Completed cohort fertility forecasts were then converted back to age-specific fertility rates. Net migration rates were forecasted as a function of the sociodemographic index, the crude popu-

lation growth rate, and mortality due to conflict, war and natural disasters. Total (all-cause) mortality was derived from forecasting cause-specific mortality from GBD 2017, using the three-component model described in Foreman et al [4]. Cause-specific mortality was then aggregated up the GBD 2017 cause hierarchy to generate total mortality forecasts, which were utilized to compute future life tables. Using past population from GBD 2017, the CCMP model advanced populations forward and produced country-age-sex specific estimates for single future calendar years.

S4.2 Covariates

S4.2.1 List of Forecasted Variables

The following are the list of variables which are forecasted in the manuscript:

- GDP: Gross Domestic Product (national income of a country)
- GGE: General Government Expenditure (all sector government expenditure in a country)
- DAH_d: Development Assistance for Health donated
- DAH_r: Development Assistance for Health received
- GHE: Government Health Expenditure
- OOP: Out-of-pocket Private Expenditure
- PPP: Prepaid Private Expenditure
- THE: Total Health Expenditure

S4.2.2 Covariates Used for Forecasting

The following covariates are used as predictors, or independent variables, in our models:

- Total population of a country (1950 - 2050)
- Proportion of total population below the age of 15 (1950 - 2050)
- Proportion of total population above the age of 64 (1950 - 2050)
- Educational attainment per capita (1980 - 2050)
- An indicator variable used to denote the anomalous disbursement of DAH in our DAH_d forecasts (1 for years in 2000 - 2010, 0 otherwise)
- An indicator variable used to denote the Ebola crisis in Guinea, Sierra Leone and Liberia in our DAH_r forecasts (1 for years in 2014 and 2015 for the three aforementioned countries, 0 otherwise).

Additionally, we also use the forecasted GDP and GGE per capita as covariates to predict the health expenditure variables. Figure S4.1 in the Tables and Figures section shows our full pathway for forecasting all our endogenous variables and how each of those variable fed into a succeeding model.

In order to forecast GDP per capita, we first convert GDP per capita to GDP per working population in the following manner:

$$\text{GDP}_{\text{working population}} = \text{GDP}_{\text{total population}} \times \frac{\text{Working population (20-64 years old)}}{\text{Total Population}}$$

We forecast GDP per working population from 2021-2050 and convert back to GDP per capita for further analysis. The out-of-sample root-mean-squared-error (RMSE) based on using GDP per working population was smaller than forecasting with GDP per capita.

S4.3 Ensemble Modeling

The purpose of ensemble modeling is to make sure that we capture the most out of what we have in our arsenal in terms of covariates and model specifications. We are agnostic about one model being the sole predictor of the future, and allow an ensemble of beliefs about predicting off of the past trends. 'Ensembling', in simple terms, is a way of pooling a number of sub-models, where the space of sub-models span different inclusions and combinations of predictors, and/or different econometric specifications.

S4.3.1 Sub-model Setup

Our basic sub-model is a linear mixed effect model of the following form, for country i and time t:

$$\Delta_t Y_{i,t} = (\alpha + \alpha_i) + \left(\sum_{p=1}^{3} \rho_p \Delta_t Y_{i,t-p} \right) + \Delta_t X' \beta + \varepsilon_{i,t} \quad (1)$$

where

$$\left(\alpha, \vec{\beta}\right) \quad \text{(Fixed effects)} \quad (2)$$

$$\alpha_i \sim N(0, \sigma_a^2) \quad \text{(Country specific random intercept)} \quad (3)$$

$$\varepsilon_{i,t} \sim N\left(\sum_{m=1}^{3} \phi_m \varepsilon_{i,t-m}, \sigma_i^2 \right) \quad \text{(Autocorrelated residuals as time random effects)} \quad (4)$$

'Fixed effect' is the equivalent of a non-random coefficient in a linear regression setting, and is estimated globally across all countries and time periods. The likelihood function for the data were all set to be Gaussian distributions for all of our metrics and models.

S4.3.2 Covariates

Using our linear baseline model as defined in section S4.3.1, we created our set of ensemble sub-models by using all combinations of each of the covariates in Table S4.2. For example, if we were predicting GGE per GDP with Educational attainment and Population as the predictors, then we would get a possible combination of four specifications to use (including one with no fixed effects). All covariates were included as fixed effects, and all specifications had the random effect on country intercepts. The proportion of submodels used that contain particular submodel specifications are described in Table S4.3.

S4.3.3 Convergence to Global Growth Rates

We believe that the very-long-term trajectory of a variable forecasted for each country will gradually taper to the global growth rates. The way that we implemented this growth rate convergence is in the following manner:

1) Suppose that we are forecasting $Y_{i,t}$, which includes an intercept at the global level and the country level. Let us also assume that there were $\widetilde{T}$ time periods of Y observed in the past for each country i.

2) We start the tapering of the growth rate to start from 10 years before $\widetilde{T}$ into the future starting from the present, converging to the global value 10 years after $\widetilde{T}$. Therefore, if the present time period is H, then our decyaing scheme will start off from the year $h + \widetilde{T} - 10$ and end at $h + \widetilde{T} + 10$.

3) Our main objective is to slowly taper all the countries' random intercepts to decay to zero, so that they are all following a global intercept. In a first-difference model, since the intercepts represent the linear time trend, this scheme will slowly converge the linear time trends of all countries to decay to the global time trend.

4) Starting from the first year of decay, we will transform the country specific intercepts as such:
$$\hat{\alpha}_i = (\hat{\alpha}_i) \times \frac{20 - t}{20}$$
where t are all the time periods between $h + \widetilde{T} - 10$ and $h + \widetilde{T} + 10$.

5) By the time t reaches $h + \widetilde{T} + 10$, the value of $\hat{\alpha}_i$ will have decayed to zero, and therefore, all the countries will have reached a global linear time trend estimate.

S4.3.4 Specifications

1) **ARIMA (Autoregressive Integrated Moving Average) terms**: We allowed up to three degrees of lags in the model (traditional auto-regressive terms), where each degree of AR term will include itself and all other lower degrees of lags. For example, a GDP per capita model with AR(3) specification (predicting log of GDP per capita) will include once, twice and thrice lagged log GDP per capita term. These were included as fixed effects.

   Additionally, in order to predict the best set of fixed effect coefficients, we test and include auto-correlated residuals in our models (traditional moving-average terms in an ARIMA

setup). This basically means that we allow our models to estimate the residuals with an autoregressive process of their own. These were included as random effects, and we allowed this to exist at the country-year level.

All of the variables we forecasted used the first-differences transformation (across time) as the dependent variable, except DAH received which was forecasted in non-differenced space, because of the presence of very high noise, thereby making the differenced series very unstable to forecast.

2) **Recency weights**: One of the other specifications we included in our sub-models was the option of weighting the recent years higher. This is particularly helpful for countries like Ethiopia and Nigeria, where they had rapid economic growth in recent years, and we believe that is a better predictor of the GDP forecasts than the further past. The weighting function was defined as such:

$$Weight = f(\tau, t) = (T - t + 1)^{\tau} \tag{5}$$

where T is the final year of in-sample data we have, and t is the year at that data point. This is an exponential decaying weighting function, where the degree of decaying is determined by τ, and we test and include a set of values of τ ranging between 0 and 0.5, where $\tau = 0$ refers to equal weights (all time periods are weighed equally at 100). Given that $\left.\frac{df(\tau, t)}{dt}\right|_{\tau>0} < 0$, this allows to weight our data with higher contributions to the likelihood from the recent past, and slowly decaying as we move further into the past towards the first observed time period. These recency weights were multiplied with the pointwise data variance parameter in order to weight the data points.

3) **Convergence Term**: We also allowed for the inclusion of a 'convergence term' in the list of sub-models. A convergence term is the one-year lag of the non-differenced dependent variable, and gets updated as each year is forecasted in the future. If a convergence term was considered in a sub-model, then we only included that sub-model if the coefficient on the convergence term was estimated to be negative (and statistically significant at 10% level).

S4.4 Inclusion and Exclusion Criteria

After we ran all possible combination of our sub-models and created a mean set of forecasts, we only want to keep the best possible set of sub-models. Hence, we implied the following set of inclusion and exclusion criteria in order to filter out the 'unrealistic' sub-models:

(1) All of the estimated coefficients must be **statistically significant at 10% level of significance**, with the exception of the model for PPP, in which all of the estimated coefficients must be **statistically significant at 20% level of significance**. For the fixed effects, we took the mean and the standard deviation of the posterior estimates, and filtered out the sub-model if the absolute z-score is below 1.645 (the absolute value of the one-sided 95th quantile of a standard normal distribution).

For the random effects, we look at the whether the measure of variance is statistically significant or not. The model outputs the mean and standard deviation of the *precision of the random effects*, and therefore allowed us to exclude the specification if the precisions are not statistically significant at 10% level.

(2) If there were any estimated coefficient that **defied a prior belief we have on the direction of the value**, then we dropped that sub-model from consideration. For example, we strongly believe that as a donor country's (high income countries) income (GDP per capita) grows, they will be able to donate more DAH to lower income countries, and so, if we ran a sub-model predicting DAH_d and get a negative coefficient on GDP per capita, we dropped that sub-model from consideration.

Our prior beliefs on the covariates for each dependent variable are listed in Table S4.2.

(3) The forecasted trajectory growth **must not exceed observed growth rates**. We believe that a country will not grow faster than how much it has grown in the past trend. In order to come up with the bounds, we run a stochastic frontier analysis (SFA) of the change in the predicted variable against the level value of the predicted variable. SFA is just like an ordinary least squares specification, except with the addition of an additional 'inefficiency' term with a half-normal distribution. This allows us to estimate (for example, for GDP per capita): conditional of a country's income, how much growth rate did we see in the country's GDP. We ran this analysis across all of the observed data points, and derived a relationship binding the growth rates of GDP against the absolute values of GDP.

S4.5 Creating the Forecasts

S4.5.1 Ranking Sub-Models

In order to find out with sub-models would be able to predict a country's future the best, we ran out-of-sample predictive validity (OOS-PV) tests [7]. Simply put: we took each sub-model that passed the criteria in section S4.4, and instead of running it on all of the past data, we left out some number of recent most years. Following that, we ran the sub-model on the truncated past, and forecasted those years left out. For example, our GDP data extends from 1970 through 2021; we left out 15 years of data, and reran a sub-model from 1970 - 2006, and use the results of that sub-model to forecast GDP for the out-of-sample years (2007 - 2021).

This gave us essentially two trajectories between 2007 through 2021: the truth and the out-of-sample predictions. For each year, we computed the squared error (the difference) between these two lines, and averaged these errors for each of the neighboring years. So for example, we had squared errors for 15 data points between 2006 and 2021, and so the first mean squared error sum was just the squared error at 2007 and 2008, the second was the mean of the sum of squared errors for 2007, 2008 and 2009, and so on. We then took the square root of this new series to get the running root mean squared errors (RMSE) for a given country.

We looked at a country's 2007 RMSE values for each sub-models ran, and listed out the best 10% of the sub-models (that is, the lowest 10% RMSE values), and we did so for every year out-of-sample. For a single country, we may potentially have completely different set of sub-models for each of the OOS years. Then, for the 10% of the sub-models selected in the *first* year OOS, we only used those models to predict the *first* year of forecast for each country; the set of 10% of the sub-models selected in the *second* year OOS were used to predict the *second* year of forecast for each country, and so on, until the last year OOS model selections are used to compute the forecast the remaining years. This allowed us to narrow down every country's trajectory with the best performing OOS-PV sub-models for each year.

S4.5.2 Uncertainty Estimation

To estimate the uncertainty intervals (UI), we reran the selected, ranked sub-models from section S4.5.1 and simulated draws instead of just getting a mean estimate of the future. There are four types of uncertainty we implemented in forecasting:

(i) **Model Uncertainty** : This type of uncertainty comes from having more than one

type of specification to create forecasts, and therefore we included a set of sub-models in the ensemble to incorporate for this uncertainty (which are ranked within each country-year).

(ii) **Data Uncertainty** : If our covariates themselves had draws of the future data (for example, when we forecasted GDP as a covariate to forecast PPP), then we picked randomly from the draws of the independent variable when predicting a sub-model's trajectory, if that covariate was included.

(iii) **Parameter Uncertainty** : This type of uncertainty is due to the variance for the posterior distributions. Once we have run a sub-model, we simulated from each of the estimated posterior distribution to create a set of simulated coefficients. This is done by simulating from the joint precision matrix of all the parameters estimated, and therefore produces a correlated set of coefficients.

(iv) **Fundamental Uncertainty** : The in-sample data and the fitted line in the past will never line up perfectly: there will always be errors from the model fit. We needed to reflect this level of uncertainty in our forecasts as well. By extracting these empirical residuals produced by a sub-model, we forecasted future country-specific residuals by using a random walk process, where the variance of the process is the variance of the residuals from the model fit σ^2_ε:

$$\hat{\varepsilon}_{i,t} \sim N(\varepsilon_{i,\hat{t}-1}, \sigma^2_{\varepsilon,t}) \tag{6}$$

A random walk is an AR(1) process with the coefficient equal to 1: in other words, the current value is independent of last year's value (except for the starting position), and will propagate forward with a random Gaussian noise of $\sigma^2_{\varepsilon,t}$ variance.

All of the above were used to simulate 1,000 forecasts (or draws), and so in order to construct our UIs, we took the mean and the values of the 2.5th and 97.5th quantiles to estimate 95% UIs.

Table S4.4 shows all of the retrospective and prospective estimates of our health spending outcomes in 2020 US dollars and 2020 purchasing-power parity-adjusted dollars in per capita space.

S4.6 Ad-hoc Draws Correlation

S4.6.1 Motivation

Given our current setup of compiling draws for each single year, there is no way of enforcing a temporal correlation across the draws right from the ensemble architecture. For example, the sub-models used in the first 15 years of GDP per capita forecasts were independently constructed and only depended on OOS-PV fits, while the 16th year (2036) onwards all draw from the same set of sub-models. This section details on the method used to generate the same correlation in the first 15 years of that example, drawing from the existing correlation from the 16th year onwards.

S4.6.2 Bivariate Correlated Distributions

Using the GDP per capita example: following from uncertainty estimation, once we generated approximately 1,000 forecast draws for a country and year for any of the covariates, we used the following strategy to achieve consistent temporal correlation across all time periods in the future:

(a) For each country, we recorded country-specific Spearman's correlation coefficient across all draws between 2036 and 2050, which gave us a country-specific correlation vector.

(b) For each value of correlation in step (a), we simulated a bivariate uniform distribution for each country and year (2022 through 2036). This simulated distribution was ranked in such a way that the marginal distributions in the joint distribution were correlated with that value of correlation coefficient we supplied (this joint distribution is known as a *copula*).

(c) We recorded the ranks of the copula, and sorted our draws (within each country) using those temporal ranks, and therefore we ended up with a complete time-series data for all draws, such that each country and year will follow the same rank correlation structure that exists between 2036 and 2050.

(d) Finally, we calculated our final set of uncertainty intervals by taking the 2.5th and 97.5th percentiles of these correlated draws.

We used this method at the end of forecasting every metric, since one metric fed into the other sequentially.

S4.7 Future Health Scenarios

We established the trajectories that our health expenditures are expected to take in the next 33 years using our ensemble models (from hereby referred to as the 'reference' case). The reference forecasts were built upon the basis of each country's past trends and expected future trends from covariates. We additionally also predicted what the possible trajectories for each country would look like if they were to follow the possible optimistic and pessimistic growth rates observed globally (referred to as 'better' and 'worse' cases, respectively).

S4.7.1 Long-term Growth Regressions

In order to determine what the possible better and worse growth rates for each country would be, we ran long-term growth regressions with the following specification:

$$Y_{i,T} - Y_{i,t} = \alpha + \beta Y_{i.t} + \epsilon_{i,t} \tag{7}$$

where the dependent variable represented the long term growth rate of Y for country i, which was computed either as logarithmic or logistic growth rates (for fractions).

The only independent variable we used ($Y_{i,t}$) was the value of Y_i at time t, and it served as a convergence term in this regression. This allowed us to predict the long-term growth rates of Y, conditional on a country's level of Y at time t.

S4.7.2 Forecasting Better and Worse Scenarios

In order to estimate what the future better and worse trajectories would be for each country, we followed these steps (assuming that we are forecasting from 2019 through 2050, with observed data between 1995 and 2018):

(i) We computed the 85th and 15th percentiles of the empirical residuals ϵ, as $Q_{0.85}(\hat{\epsilon_{i,t}})$ and $Q_{0.15}(\hat{\epsilon_{i,t}})$ respectively, where $Q_p(\cdot)$ is a quantile function for a percentile p.

(ii) We computed the starting annualized growth rate from the fitted scenario regression, such that, for country i:

$$\text{Better growth rate} = \exp(\hat{\alpha}) \times \exp(Q_{0.85}(\hat{\epsilon_{i,t}})) \times (Y_{i,2018}^{\hat{\beta}})^{(1/(2018-1995))} \tag{8}$$

$$\text{Worse growth rate} = \exp(\hat{\alpha}) \times \exp(Q_{0.15}(\hat{\epsilon_{i,t}})) \times (Y_{i,2018}^{\hat{\beta}})^{(1/(2018-1995))} \tag{9}$$

(iii) Finally, once we have established the growth rates as a function of the convergence term, we recursively created better and worse trajectories, conditional on the updated

growth rates every year, such that:

$$Y_{i,t+1} = Y_{i,t} \times \exp(\hat{\alpha}) \times \exp(Q_{0.85}(\hat{\epsilon_{i,t}})) \times (Y_{i,t}^{\hat{\beta}}) \tag{10}$$

where $\exp(\hat{\alpha}) \times \exp(Q_{0.85}(\hat{\epsilon_{i,t}})) \times (Y_{i,t}^{\hat{\beta}})$ was the conditional growth rate for a single year.

One condition that we imposed for the computed scenarios is: the better projection cannot be lower than the reference projection, and the worse projection cannot be higher than the reference projection. For countries with wide forecasts where this case did happen, we moved the better and worse forecasts down and up to overlap on top of the reference line respectively.

S4.7.3 Uncertainty Estimation

The uncertainty intervals around a scenario were expected to take the same shape as the uncertainty around our reference forecasts. Therefore, once we have propagated a mean set of better and worse forecasts in section S4.7.2, we created the draws around our scenarios in the following way:

(i) We took our reference forecast's mean line and the 1,000 draws around that line.

(ii) We computed the deviation of the mean from each of the draws (in logarithmic or logistic transformation, depending on the space of the covariate).

(iii) We took each of the scenario mean lines and added the deviations from the previous step to the mean lines, giving us 1,000 draws of the scenario projections.

Figure set S4.3 visualizes the future health scenarios of our health outcomes in per capita space (in 2020 US dollars).

S4.8 Package and Architecture

S4.8.1 Architecture

All analysis and forecasting were done on a parallel computing cluster with 26,000 cores with a CentOS interface. We compiled R [8] version 3.6.0 from source code on a Docker based on Debian OS, which was deployed as a Singularity container with all the necessary compilers and binaries (GCC, G++ and Fortran 8.2.0).

S4.8.2 Template Model Builder

We used the R library **TMB** [9] to run our baseline mixed effects models. TMB stands for Template Model Builder, and it's a powerful method of approximating the integral of the Gaussian probability distribution function (which doesn't exist in closed form) by using a Laplace Approximation, which gives very precise results, and is relatively faster than other approximation packages which exist like Stan or Bugs. TMB uses automatic differentiation on the negative log-likelihood function in order to optimize the model parameters, and also returns a sparse joint precision matrix (inverse of the variance-covariance matrix), from which we can simulate posterior parameter draws.

References

[1] GBD 2017 Risk Factor Collaborators. Global, regional, and national comparative risk assessment of 84 behavioural, environmental and occupational, and metabolic risks or clusters of risks for 195 countries and territories, 1990–2017: a systematic analysis for the Global Burden of Disease Study 2017. *The Lancet*, 392(10159):1923–1994, nov 2018. doi: 10.1016/S0140-6736(18)32225-6. URL https://doi.org/10.1016/S0140-6736(18)32225-6.

[2] Spencer L James, Paul Gubbins, Christopher JL Murray, and Emmanuela Gakidou. Developing a comprehensive time series of gdp per capita for 210 countries from 1950 to 2015. *Population health metrics*, 10(1):12, 2012. URL https://pophealthmetrics.biomedcentral.com/articles/10.1186/1478-7954-10-12.

[3] Emmanuela Gakidou, Krycia Cowling, Rafael Lozano, and Christopher JL Murray. Increased educational attainment and its effect on child mortality in 175 countries between 1970 and 2009: a systematic analysis. *The Lancet*, 376(9745):959–974, 2010. URL https://doi.org/10.1016/S0140-6736(10)61257-3.

[4] Kyle J Foreman, Neal Marquez, Andrew Dolgert, Kai Fukutaki, Nancy Fullman, Madeline McGaughey, Martin A Pletcher, Amanda E Smith, Kendrick Tang, Chun-Wei Yuan, et al. Forecasting life expectancy, years of life lost, and all-cause and cause-specific mortality for 250 causes of death: reference and alternative scenarios for 2016–40 for 195 countries and territories. *The Lancet*, 392(10159):2052–2090, 2018.

[5] Spencer Preston, Patrick Heuveline, and Michel Guillot. *Demography: measuring and modeling population processes*. Wiley-Blackwell, 1 edition. Malden, MA, USA, 2000.

[6] Stein Emil Vollset et al. Widespread population decline: robust fertility, mortality, migration, and population scenarios for 195 countries and territories from 2017 to 2100. *The Lancet*, 2019.

[7] Kyle J Foreman, Rafael Lozano, Alan D Lopez, and Christopher JL Murray. Modeling causes of death: an integrated approach using codem. *Population health metrics*, 10(1): 1, 2012.

[8] R Core Team. *R: A Language and Environment for Statistical Computing*. R Foundation for Statistical Computing, Vienna, Austria, 2017. URL https://www.R-project.org/. Accessed: 2017-12-20.

[9] Kasper Kristensen, Anders Nielsen, Casper W Berg, Hans Skaug, and Brad Bell. Tmb: automatic differentiation and laplace approximation. *arXiv preprint arXiv:1509.00660*, 2015.

S4.9 Tables and Figures

Table S4.1: Definitions of health expenditure sources

Health Expenditure Type	Definition
Development assistance for health	Financial and in-kind contributions from global health channels that aim to improve or maintain health in low- or middle-income countries.
Government health expenditure as source	Government health expenditure as source only includes domestically financed government expenditure on health, including public health spending.
Out-of-pocket expenditure	Paid by individuals for health services; considered catastrophic if exceeding 40% of a household's annual income.
Prepaid private health expenditure	Private risk pooling against catastrophic health expenditure; includes private insurance and non-governmental organizations.

Figure S4.1: State Space Diagram of Forecasting Components

This diagram details the order in which forecasting components are estimated, and which forecasting components are used

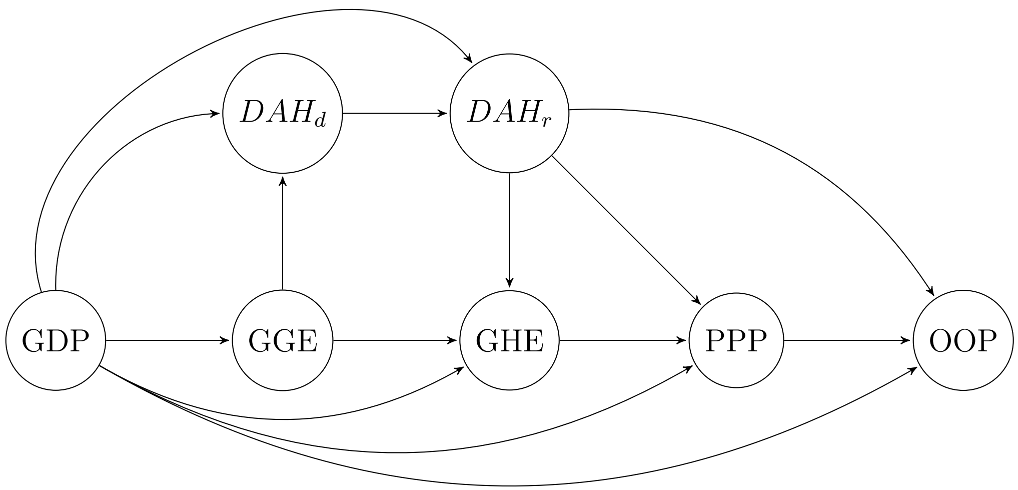

as inputs in others.

Table S4.2: Prediction and Covariates Map with Transformations

	**Predicted Variables**	**Covariates**	**Extra Specifications**
1	GDP per labour (log)		ARIMA(2-3, 1, 0)
2	GGE per GDP (logit)	GDPpc^{+}, Pop >64^{+}, Education^{+}	ARIMA(0-3, 1, 0-3)
3	DAH donated per GGE (logit)	GDPpc^{+}, GGE/GDP^{+}, Bush Era Dummy^{+}	ARIMA(0-3, 1, 0-3)
4	GHE per GGE (logit)	GDPpc^{+}, Pop >64^{+}, GGE/GDP^{+}, DAH/GDP^{-}	ARIMA(0-3, 1, 0-3)
5	PPP per GDP (logit)	GDPpc^{+}, Pop >64^{+}, GGE/GDP. DAH/GDP, GHE/GDP,	ARIMA(0-3, 1, 0-3)
6	OOP per GDP (logit)	GDPpc^{-}, Pop >64^{+}, GGE/GDP, DAH/GDP, GHE/GDP, PPP/GDP	ARIMA(0-3, 1, 0-3)

Figure S4.2: Process diagram for estimating future GDP, all-sector government spending, and health spending by source

This diagram details the inputs and outputs of the ensemble modeling process.

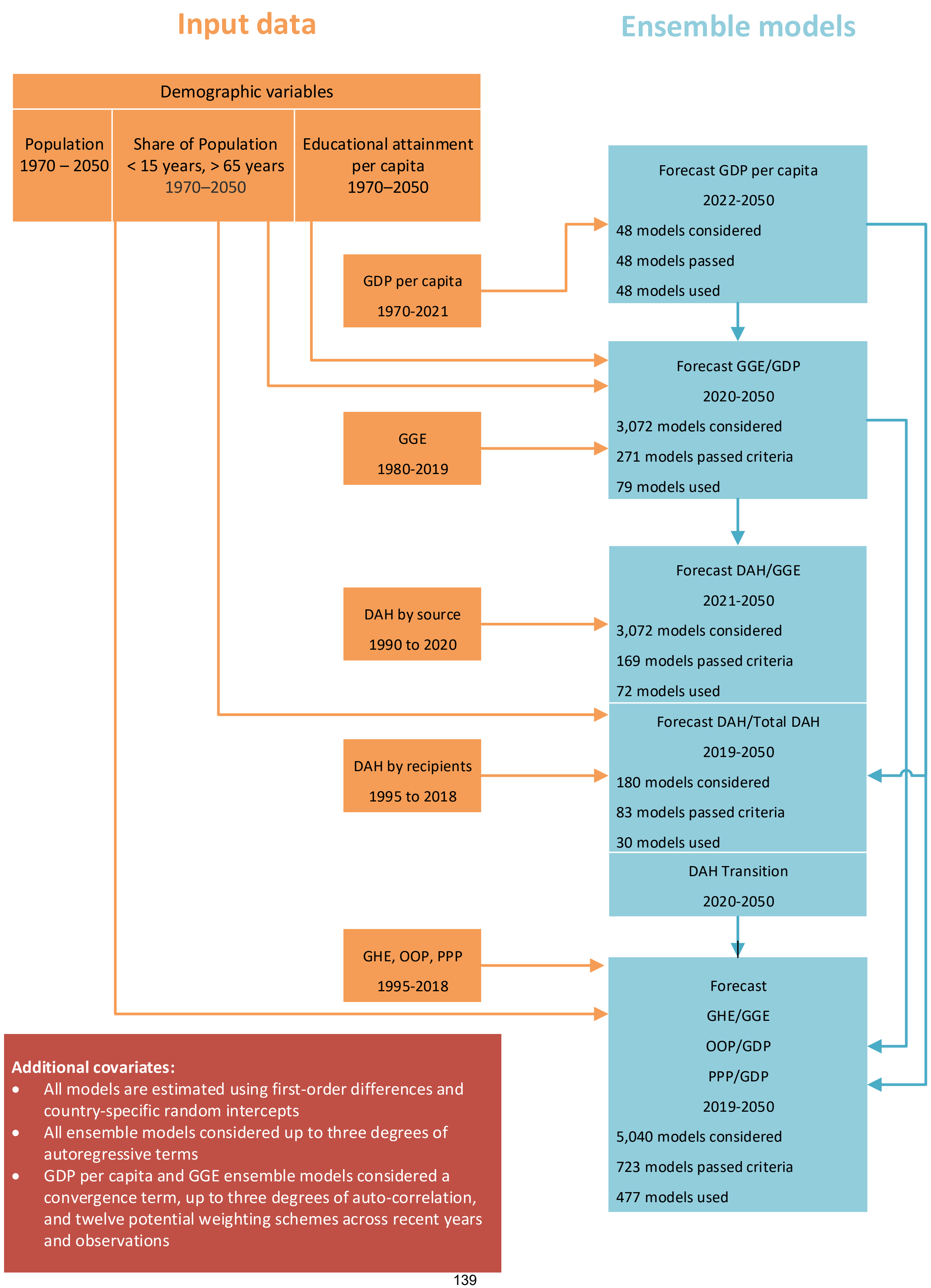

Input data
Ensemble models
Demographic variables
Population
1970 – 2050
Share of Population
< 15 years, > 65 years
1970–2050
Educational attainment
per capita
1970–2050
GDP per capita
1970-2021
GGE
1980-2019
DAH by source
1990 to 2020
DAH by recipients
1995 to 2018
GHE, OOP, PPP
1995-2018
Forecast GDP per capita
2022-2050
48 models considered
48 models passed
48 models used
Forecast GGE/GDP
2020-2050
3,072 models considered
271 models passed criteria
79 models used
Forecast DAH/GGE
2021-2050
3,072 models considered
169 models passed criteria
72 models used
Forecast DAH/Total DAH
2019-2050
180 models considered
83 models passed criteria
30 models used
DAH Transition
2020-2050
Forecast
GHE/GGE
OOP/GDP
PPP/GDP
2019-2050
5,040 models considered
723 models passed criteria
477 models used
Additional covariates:
All models are estimated using first-order differences and country-specific random intercepts
All ensemble models considered up to three degrees of autoregressive terms
GDP per capita and GGE ensemble models considered a convergence term, up to three degrees of auto-correlation, and twelve potential weighting schemes across recent years and observations

Table S4.3: Ensemble submodel specifications used in forecasts

This table shows all submodel specifications considered in the ensemble, and the percentage of models with given specifications used in the forecasts.

	GDP		GGE		DAH Provided		DAH Received		GHE		PPP		OOP	
Number of out-of-sample years included	15		15		15		10		15		15		15	
Number of countries included	204		204		24		139		204		204		204	
Number of top models used per country and year	15		15		15		15		15		15		55	
Total number of models used across all countries and yea	45900		45900		5400		20850		45900		45900		168300	
Covariate(s)	Value	Percentage of models	Value	Percentage of models	Value	Percentage of models	Value	Percentage of models	Value	Percentage of models	Value	Percentage of models	Value	Percentage of models
Autoregressive terms	3	38.7%	3	10.0%	3	0.0%	0	100.0%	2	27.5%	2	100.0%	3	38.8%
	2	61.3%	2	5.2%	2	8.1%			1	34.7%			2	61.2%
			1	7.7%	1	10.8%			0	37.9%				
			0	77.1%	0	81.1%								
Moving average terms	0	100.0%	3	22.5%	3	0.0%	0	100.0%	0	100.0%	0	100.0%	0	100.0%
			2	23.4%	2	42.7%								
			1	24.4%	1	28.4%								
			0	29.6%	0	28.9%								
Convergence term	Included	61.4%	Included	25.7%	Included	86.1%	Not included	100.0%	Not included	100.0%	Not included	100.0%	Not included	100.0%
	Not included	38.6%	Not included	74.3%	Not included	13.9%								
Recency weights (Values represent the tau value used in our recency weighting function described on page 10 of this appendix. Higher values indicate a stronger weight placed on recent years' data.)	0.00000	100.0%	0.70000	6.9%	0.70000	21.3%	0.80000	5.1%	0.20000	7.0%	0.20000	5.8%	0.20000	3.7%
			0.63636	6.3%	0.63636	17.1%	0.77241	5.3%	0.18571	6.9%	0.18571	5.8%	0.18571	3.8%
			0.57273	7.7%	0.57273	12.1%	0.74483	5.5%	0.17143	6.8%	0.17143	5.8%	0.17143	4.6%
			0.50909	12.4%	0.50909	5.6%	0.71724	5.5%	0.15714	6.8%	0.15714	5.7%	0.15714	5.0%
			0.44545	13.7%	0.44545	7.5%	0.68966	5.5%	0.14286	6.7%	0.14286	7.8%	0.14286	5.2%
			0.38182	11.1%	0.38182	8.0%	0.66207	5.6%	0.12857	6.7%	0.12857	7.6%	0.12857	5.6%
			0.31818	7.0%	0.31818	4.3%	0.63448	5.6%	0.11429	6.7%	0.11429	7.7%	0.11429	6.5%
			0.25455	6.2%	0.25455	4.6%	0.60690	5.7%	0.10000	6.6%	0.10000	8.4%	0.10000	6.2%
			0.19091	6.6%	0.19091	4.9%	0.55172	5.8%	0.08571	6.6%	0.08571	8.4%	0.08571	6.9%
			0.12727	7.1%	0.12727	4.8%	0.52414	5.8%	0.07143	6.6%	0.07143	7.4%	0.07143	7.5%
			0.06364	7.4%	0.06364	4.9%	0.49655	5.8%	0.05714	6.5%	0.05714	6.1%	0.05714	8.4%
			0.00000	7.6%	0.00000	5.0%	0.46897	5.9%	0.04286	6.6%	0.04286	6.0%	0.04286	8.9%
							0.44138	5.9%	0.02857	6.6%	0.02857	4.7%	0.02857	9.1%
							0.41379	5.9%	0.01429	6.5%	0.01429	6.2%	0.01429	9.3%
							0.38621	6.6%	0.00000	6.5%	0	6.5%	0	9.2%
							0.35862	1.7%						
							0.33103	1.5%						
							0.30345	1.2%						
							0.27586	1.1%						
							0.24828	1.1%						
							0.22069	1.0%						
							0.1931	1.0%						
							0.16552	1.0%						
							0.13793	0.9%						
							0.11034	0.9%						
							0.08276	0.8%						
							0.05517	0.8%						
							0.02759	0.8%						
							0	0.7%						
Proportion of population under age 15							Included	0.0%						
							Not included	100.0%						
Proportion of population over age 65			Included	0.0%					Included	55.2%	Included	51.8%	Included	42.9%
			Not included	100.0%					Not included	44.8%	Not included	48.2%	Not included	57.1%
GDP per capita			Included	0.0%	Included	0.0%	Included	100.0%	Included	0.0%	Included	0.0%		
			Not included	100.0%	Not included	100.0%	Not included	0.0%	Not included	100.0%	Not included	100.0%		
GGE per GDP					Included	35.1%			Included	0.0%				
					Not included	64.9%			Not included	100.0%				
DAH Received per GDP									Included	0.0%	Included	54.0%	Included	50.9%
									Not included	100.0%	Not included	46.0%	Not included	49.1%
GHE per GDP											Included	0.0%	Included	0.0%
											Not included	100.0%	Not included	100.0%
PPP per GDP													Included	45.3%
													Not included	54.7%

Table S4.4: Health Spending from 1995-2050

This table shows all of our health spending outcomes for every country and year from 1995 through 2050. The variables are in per capita measures, and for currencies 2020 US dollars and 2020 purchasing-power parity-adjusted dollars .Estimates in parentheses are 95% uncertainty intervals. Asterisks indicate countries who are in lower- and middle-income groups.

Figure S4.3: Future Health Scenarios

This set of figures included show fix panels of the dependent variables that we forecasted, starting with DAH, GHE, PPP, OOP and THE per capita. Each variable contains the respective reference, better and worse scenarios. The currency is 2020 US dollars.

Country	Year	2020 US dollars per capita					2020 US purchasing-power parity-adjusted dollars per capita				
		Total health spending	Government health spending	Out-of-pocket health spending	Prepaid private health spending	Development assistance for health	Total health spending	Government health spending	Out-of-pocket health spending	Prepaid private health spending	Development assistance for health
Afghanistan*	1995	30·2 (23·8 to 38·0)	0·8 (0·7 to 1·0)	29·1 (22·8 to 37·0)	0·0 (0·0 to 0·0)	0·2 (0·2 to 0·2)	125·4 (99·0 to 157·9)	3·5 (2·8 to 4·2)	120·9 (94·5 to 153·6)	0·0 (0·0 to 0·0)	1·0 (1·0 to 1·0)
Afghanistan*	1996	28·4 (22·6 to 35·7)	0·8 (0·6 to 0·9)	27·3 (21·5 to 34·7)	0·0 (0·0 to 0·0)	0·3 (0·3 to 0·3)	117·7 (93·8 to 148·2)	3·1 (2·5 to 3·8)	113·2 (89·1 to 143·9)	0·0 (0·0 to 0·0)	1·3 (1·3 to 1·3)
Afghanistan*	1997	27·0 (21·9 to 33·7)	0·7 (0·6 to 0·8)	25·7 (20·6 to 32·4)	0·0 (0·0 to 0·0)	0·6 (0·6 to 0·6)	112·1 (90·9 to 140·0)	2·8 (2·3 to 3·5)	106·7 (85·3 to 134·3)	0·0 (0·0 to 0·0)	2·6 (2·6 to 2·6)
Afghanistan*	1998	25·3 (20·6 to 31·6)	0·6 (0·5 to 0·8)	24·5 (19·7 to 30·7)	0·0 (0·0 to 0·0)	0·3 (0·3 to 0·3)	105·2 (85·5 to 131·0)	2·6 (2·1 to 3·2)	101·5 (81·7 to 127·2)	0·0 (0·0 to 0·0)	1·0 (1·0 to 1·0)
Afghanistan*	1999	24·1 (19·9 to 29·3)	0·6 (0·5 to 0·7)	23·1 (18·9 to 28·4)	0·0 (0·0 to 0·0)	0·4 (0·4 to 0·4)	100·1 (82·6 to 121·8)	2·4 (1·9 to 2·9)	95·9 (78·3 to 117·7)	0·0 (0·0 to 0·0)	1·8 (1·8 to 1·8)
Afghanistan*	2000	22·9 (19·1 to 27·6)	0·5 (0·4 to 0·7)	21·9 (18·1 to 26·5)	0·0 (0·0 to 0·0)	0·5 (0·5 to 0·5)	95·2 (79·5 to 114·4)	2·2 (1·8 to 2·7)	90·9 (75·2 to 110·0)	0·0 (0·0 to 0·0)	2·1 (2·1 to 2·1)
Afghanistan*	2001	22·1 (18·9 to 25·9)	0·5 (0·4 to 0·6)	20·8 (17·5 to 24·6)	0·0 (0·0 to 0·0)	0·8 (0·8 to 0·8)	91·7 (78·3 to 107·5)	2·1 (1·7 to 2·6)	86·4 (72·7 to 102·3)	0·0 (0·0 to 0·0)	3·2 (3·2 to 3·2)
Afghanistan*	2002	24·9 (21·6 to 28·5)	0·6 (0·5 to 0·8)	22·8 (19·5 to 26·4)	0·0 (0·0 to 0·0)	1·5 (1·5 to 1·5)	103·1 (89·6 to 118·2)	2·6 (2·1 to 3·2)	94·5 (80·9 to 109·5)	0·0 (0·0 to 0·0)	6·0 (6·0 to 6·0)
Afghanistan*	2003	30·3 (26·7 to 34·2)	1·3 (1·1 to 1·6)	26·9 (23·3 to 30·8)	0·0 (0·0 to 0·0)	2·1 (2·1 to 2·1)	125·8 (110·9 to 141·9)	5·4 (4·4 to 6·7)	111·8 (96·6 to 127·8)	0·0 (0·0 to 0·0)	8·6 (8·6 to 8·6)
Afghanistan*	2004	34·4 (31·0 to 38·1)	1·6 (1·3 to 1·9)	27·6 (24·2 to 31·4)	0·0 (0·0 to 0·0)	5·2 (5·2 to 5·2)	142·8 (128·8 to 158·1)	6·5 (5·3 to 8·0)	114·7 (100·5 to 130·2)	0·0 (0·0 to 0·0)	21·6 (21·6 to 21·6)
Afghanistan*	2005	35·7 (32·5 to 39·4)	1·7 (1·4 to 2·1)	27·8 (24·6 to 31·5)	0·0 (0·0 to 0·0)	6·2 (6·2 to 6·2)	148·1 (134·9 to 163·5)	7·0 (5·6 to 8·6)	115·3 (102·2 to 130·6)	0·0 (0·0 to 0·0)	25·7 (25·7 to 25·7)
Afghanistan*	2006	36·9 (33·8 to 40·6)	1·9 (1·5 to 2·3)	28·7 (25·5 to 32·3)	0·0 (0·0 to 0·0)	6·4 (6·4 to 6·4)	153·3 (140·2 to 168·5)	7·7 (6·2 to 9·6)	119·0 (105·8 to 134·3)	0·0 (0·0 to 0·0)	26·5 (26·5 to 26·5)

Afghanistan*	2007	40·5 (37·2 to 44·5)	2·2 (1·8 to 2·7)	30·5 (27·3 to 34·3)	0·0 (0·0 to 0·0)	7·8 (7·8 to 7·8)	168·2 (154·5 to 184·5)	9·2 (7·3 to 11·4)	126·7 (113·2 to 142·2)	0·0 (0·0 to 0·0)	32·3 (32·3 to 32·3)
Afghanistan*	2008	40·1 (36·9 to 43·6)	2·2 (1·7 to 2·7)	30·3 (27·2 to 33·7)	0·0 (0·0 to 0·0)	7·6 (7·6 to 7·6)	166·3 (153·2 to 181·1)	9·1 (7·2 to 11·2)	125·7 (113·1 to 140·1)	0·0 (0·0 to 0·0)	31·6 (31·6 to 31·6)
Afghanistan*	2009	44·9 (41·7 to 48·6)	2·3 (1·9 to 2·9)	31·7 (28·5 to 35·3)	0·0 (0·0 to 0·0)	10·9 (10·9 to 10·9)	186·5 (173·2 to 201·6)	9·7 (7·8 to 11·9)	131·5 (118·2 to 146·3)	0·0 (0·0 to 0·0)	45·2 (45·2 to 45·2)
Afghanistan*	2010	44·1 (40·8 to 47·7)	2·3 (1·8 to 2·8)	31·6 (28·2 to 35·2)	0·0 (0·0 to 0·0)	10·2 (10·2 to 10·2)	183·1 (169·2 to 197·9)	9·5 (7·6 to 11·6)	131·3 (117·1 to 146·0)	0·0 (0·0 to 0·0)	42·3 (42·3 to 42·3)
Afghanistan*	2011	46·2 (42·9 to 49·9)	2·3 (1·8 to 2·8)	31·7 (28·5 to 35·3)	0·0 (0·0 to 0·0)	12·2 (12·2 to 12·2)	191·8 (178·0 to 207·1)	9·4 (7·6 to 11·6)	131·8 (118·3 to 146·5)	0·0 (0·0 to 0·0)	50·6 (50·6 to 50·6)
Afghanistan*	2012	45·8 (42·2 to 49·5)	2·4 (2·0 to 3·0)	33·7 (30·3 to 37·2)	0·0 (0·0 to 0·0)	9·7 (9·7 to 9·7)	190·0 (175·1 to 205·4)	10·1 (8·2 to 12·3)	139·7 (125·7 to 154·6)	0·0 (0·0 to 0·0)	40·2 (40·2 to 40·2)
Afghanistan*	2013	48·7 (45·0 to 52·5)	2·5 (2·1 to 3·1)	35·9 (32·4 to 39·6)	0·0 (0·0 to 0·0)	10·3 (10·3 to 10·3)	202·2 (186·7 to 217·8)	10·5 (8·6 to 13·0)	149·0 (134·4 to 164·2)	0·0 (0·0 to 0·0)	42·6 (42·6 to 42·6)
Afghanistan*	2014	50·8 (47·1 to 55·0)	2·7 (2·2 to 3·3)	39·2 (35·5 to 43·4)	0·0 (0·0 to 0·0)	9·0 (9·0 to 9·0)	211·0 (195·3 to 228·1)	11·1 (9·0 to 13·6)	162·6 (147·5 to 180·0)	0·0 (0·0 to 0·0)	37·3 (37·3 to 37·3)
Afghanistan*	2015	54·3 (50·4 to 58·4)	2·8 (2·2 to 3·4)	42·7 (38·9 to 47·0)	0·0 (0·0 to 0·0)	8·8 (8·8 to 8·8)	225·2 (209·3 to 242·3)	11·4 (9·3 to 14·0)	177·3 (161·5 to 195·1)	0·0 (0·0 to 0·0)	36·5 (36·5 to 36·5)
Afghanistan*	2016	54·2 (50·3 to 58·4)	2·9 (2·4 to 3·6)	46·4 (42·6 to 50·5)	0·0 (0·0 to 0·0)	4·9 (4·9 to 4·9)	225·1 (208·9 to 242·5)	12·2 (10·0 to 14·8)	192·5 (176·7 to 209·7)	0·0 (0·0 to 0·0)	20·3 (20·3 to 20·3)
Afghanistan*	2017	55·5 (51·7 to 59·7)	3·0 (2·4 to 3·6)	47·8 (44·1 to 51·7)	0·0 (0·0 to 0·0)	4·7 (4·7 to 4·7)	230·3 (214·7 to 247·6)	12·4 (10·1 to 15·1)	198·3 (182·9 to 214·7)	0·0 (0·0 to 0·0)	19·6 (19·6 to 19·6)
Afghanistan*	2018	55·0 (50·6 to 59·6)	2·9 (2·3 to 3·5)	45·3 (40·9 to 49·9)	0·0 (0·0 to 0·0)	6·8 (6·8 to 6·8)	228·1 (209·9 to 247·2)	11·9 (9·7 to 14·4)	188·2 (169·6 to 206·9)	0·0 (0·0 to 0·0)	28·0 (28·0 to 28·0)
Afghanistan*	2019	53·8 (49·4 to 58·5)	2·8 (2·3 to 3·4)	45·7 (41·2 to 50·2)	0·0 (0·0 to 0·0)	5·4 (5·4 to 5·5)	223·4 (205·0 to 242·7)	11·5 (9·4 to 14·0)	189·5 (170·9 to 208·2)	0·0 (0·0 to 0·0)	22·4 (22·2 to 22·7)
Afghanistan*	2020	54·8 (50·7 to 59·1)	5·2 (4·7 to 5·8)	42·0 (37·9 to 46·2)	0·0 (0·0 to 0·0)	7·6 (7·5 to 7·6)	227·6 (210·6 to 245·2)	21·7 (19·6 to 24·1)	174·4 (157·2 to 191·8)	0·0 (0·0 to 0·0)	31·4 (31·1 to 31·7)

Afghanistan*	2021	49·9 (46·0 to 53·9)	2·7 (2·2 to 3·3)	39·5 (35·6 to 43·4)	0·0 (0·0 to 0·0)	7·7 (7·5 to 7·8)	207·0 (191·0 to 223·6)	11·3 (9·2 to 13·8)	163·9 (147·7 to 180·1)	0·0 (0·0 to 0·0)	31·8 (31·2 to 32·4)
Afghanistan*	2022	47·8 (43·8 to 51·7)	2·3 (1·9 to 2·8)	39·6 (35·6 to 43·5)	0·0 (0·0 to 0·0)	5·9 (5·6 to 6·2)	198·4 (181·9 to 214·8)	9·6 (7·8 to 11·7)	164·4 (147·8 to 180·7)	0·0 (0·0 to 0·0)	24·4 (23·2 to 25·7)
Afghanistan*	2023	48·0 (44·0 to 52·0)	2·3 (1·8 to 2·8)	39·8 (35·8 to 43·7)	0·0 (0·0 to 0·0)	5·9 (5·6 to 6·3)	199·2 (182·5 to 215·7)	9·5 (7·7 to 11·7)	165·1 (148·5 to 181·3)	0·0 (0·0 to 0·0)	24·6 (23·2 to 26·2)
Afghanistan*	2024	48·1 (44·1 to 52·2)	2·3 (1·8 to 2·8)	39·9 (35·9 to 43·8)	0·0 (0·0 to 0·0)	5·9 (5·5 to 6·4)	199·7 (182·9 to 216·7)	9·4 (7·5 to 11·6)	165·7 (149·0 to 181·9)	0·0 (0·0 to 0·0)	24·6 (23·0 to 26·4)
Afghanistan*	2025	48·2 (44·1 to 52·3)	2·2 (1·8 to 2·8)	40·0 (36·0 to 44·0)	0·0 (0·0 to 0·0)	6·0 (5·6 to 6·4)	200·1 (183·1 to 217·1)	9·3 (7·5 to 11·5)	166·1 (149·2 to 182·5)	0·0 (0·0 to 0·0)	24·8 (23·1 to 26·5)
Afghanistan*	2026	48·3 (44·3 to 52·4)	2·2 (1·8 to 2·7)	40·1 (36·0 to 44·2)	0·0 (0·0 to 0·0)	6·0 (5·5 to 6·4)	200·6 (183·9 to 217·5)	9·2 (7·4 to 11·4)	166·6 (149·4 to 183·3)	0·0 (0·0 to 0·0)	24·8 (23·0 to 26·8)
Afghanistan*	2027	48·5 (44·4 to 52·6)	2·2 (1·8 to 2·7)	40·3 (36·1 to 44·4)	0·0 (0·0 to 0·0)	6·0 (5·6 to 6·5)	201·3 (184·2 to 218·4)	9·2 (7·4 to 11·4)	167·1 (149·7 to 184·1)	0·0 (0·0 to 0·0)	24·9 (23·0 to 26·9)
Afghanistan*	2028	48·7 (44·6 to 52·8)	2·2 (1·8 to 2·8)	40·4 (36·2 to 44·6)	0·0 (0·0 to 0·0)	6·0 (5·6 to 6·6)	202·2 (184·9 to 219·0)	9·3 (7·5 to 11·6)	167·8 (150·2 to 185·2)	0·0 (0·0 to 0·0)	25·1 (23·1 to 27·2)
Afghanistan*	2029	49·0 (44·8 to 53·1)	2·3 (1·8 to 2·9)	40·6 (36·4 to 44·8)	0·0 (0·0 to 0·0)	6·1 (5·6 to 6·7)	203·5 (186·0 to 220·6)	9·5 (7·6 to 11·8)	168·7 (151·1 to 186·1)	0·0 (0·0 to 0·0)	25·3 (23·2 to 27·6)
Afghanistan*	2030	49·3 (45·0 to 53·4)	2·4 (1·9 to 3·0)	40·7 (36·4 to 45·0)	0·0 (0·0 to 0·0)	6·2 (5·6 to 6·8)	204·5 (186·8 to 221·8)	9·8 (7·8 to 12·3)	169·0 (151·2 to 186·7)	0·0 (0·0 to 0·0)	25·6 (23·4 to 28·1)
Afghanistan*	2031	49·5 (45·3 to 53·8)	2·4 (1·9 to 3·1)	40·8 (36·5 to 45·1)	0·0 (0·0 to 0·0)	6·3 (5·7 to 6·9)	205·6 (188·0 to 223·1)	10·1 (8·1 to 12·7)	169·5 (151·6 to 187·1)	0·0 (0·0 to 0·0)	26·0 (23·5 to 28·8)
Afghanistan*	2032	49·8 (45·6 to 54·1)	2·5 (2·0 to 3·2)	41·0 (36·6 to 45·3)	0·0 (0·0 to 0·0)	6·3 (5·7 to 7·0)	206·8 (189·5 to 224·4)	10·5 (8·3 to 13·1)	170·0 (152·0 to 187·8)	0·0 (0·0 to 0·0)	26·3 (23·7 to 29·2)
Afghanistan*	2033	50·1 (45·9 to 54·4)	2·6 (2·1 to 3·3)	41·1 (36·7 to 45·4)	0·0 (0·0 to 0·0)	6·4 (5·8 to 7·2)	208·0 (190·3 to 225·7)	10·9 (8·6 to 13·6)	170·5 (152·3 to 188·4)	0·0 (0·0 to 0·0)	26·6 (23·9 to 29·7)
Afghanistan*	2034	50·5 (46·2 to 54·8)	2·7 (2·2 to 3·4)	41·2 (36·8 to 45·5)	0·0 (0·0 to 0·0)	6·5 (5·8 to 7·2)	209·4 (191·7 to 227·3)	11·4 (8·9 to 14·3)	171·1 (152·9 to 189·0)	0·0 (0·0 to 0·0)	26·9 (24·2 to 30·1)

Afghanistan*	2035	50·8 (46·6 to 55·2)	2·9 (2·3 to 3·6)	41·4 (37·0 to 45·7)	0·0 (0·0 to 0·0)	6·6 (5·9 to 7·4)	210·9 (193·4 to 228·9)	12·0 (9·4 to 15·0)	171·7 (153·5 to 189·6)	0·0 (0·0 to 0·0)	27·2 (24·3 to 30·6)
Afghanistan*	2036	51·2 (46·8 to 55·5)	3·0 (2·4 to 3·8)	41·5 (37·1 to 45·8)	0·0 (0·0 to 0·0)	6·6 (5·9 to 7·5)	212·4 (194·4 to 230·4)	12·5 (9·8 to 15·7)	172·4 (154·1 to 190·3)	0·0 (0·0 to 0·0)	27·5 (24·4 to 31·2)
Afghanistan*	2037	51·5 (47·2 to 56·0)	3·1 (2·4 to 3·9)	41·8 (37·4 to 46·1)	0·0 (0·0 to 0·0)	6·6 (5·9 to 7·6)	213·9 (195·8 to 232·2)	12·9 (10·1 to 16·3)	173·4 (155·0 to 191·3)	0·0 (0·0 to 0·0)	27·6 (24·5 to 31·5)
Afghanistan*	2038	51·9 (47·5 to 56·3)	3·2 (2·5 to 4·1)	42·0 (37·6 to 46·3)	0·0 (0·0 to 0·0)	6·7 (5·9 to 7·7)	215·4 (197·1 to 233·8)	13·3 (10·4 to 16·8)	174·4 (155·9 to 192·3)	0·0 (0·0 to 0·0)	27·7 (24·6 to 31·8)
Afghanistan*	2039	52·3 (47·8 to 56·8)	3·3 (2·6 to 4·2)	42·3 (37·8 to 46·6)	0·0 (0·0 to 0·0)	6·7 (5·9 to 7·7)	217·1 (198·5 to 235·8)	13·7 (10·7 to 17·3)	175·6 (157·0 to 193·6)	0·0 (0·0 to 0·0)	27·7 (24·6 to 31·8)
Afghanistan*	2040	52·7 (48·2 to 57·3)	3·4 (2·6 to 4·3)	42·7 (38·2 to 47·0)	0·0 (0·0 to 0·0)	6·7 (5·8 to 7·7)	218·9 (200·1 to 237·7)	14·1 (10·9 to 17·8)	177·2 (158·5 to 195·2)	0·0 (0·0 to 0·1)	27·6 (24·3 to 31·9)
Afghanistan*	2041	53·2 (48·6 to 57·7)	3·5 (2·7 to 4·4)	43·1 (38·6 to 47·5)	0·0 (0·0 to 0·0)	6·6 (5·8 to 7·7)	220·8 (201·6 to 239·4)	14·4 (11·2 to 18·3)	179·0 (160·1 to 197·2)	0·0 (0·0 to 0·1)	27·4 (24·0 to 31·8)
Afghanistan*	2042	53·7 (49·0 to 58·3)	3·6 (2·7 to 4·5)	43·6 (39·0 to 48·1)	0·0 (0·0 to 0·0)	6·5 (5·7 to 7·6)	222·9 (203·5 to 242·2)	14·7 (11·4 to 18·8)	181·0 (161·9 to 199·4)	0·0 (0·0 to 0·1)	27·1 (23·6 to 31·5)
Afghanistan*	2043	54·2 (49·5 to 59·0)	3·6 (2·8 to 4·6)	44·1 (39·5 to 48·7)	0·0 (0·0 to 0·0)	6·4 (5·6 to 7·5)	225·0 (205·4 to 244·7)	15·0 (11·6 to 19·2)	183·2 (164·0 to 202·0)	0·0 (0·0 to 0·1)	26·7 (23·2 to 31·1)
Afghanistan*	2044	54·8 (50·0 to 59·6)	3·7 (2·8 to 4·7)	44·8 (40·1 to 49·4)	0·0 (0·0 to 0·0)	6·3 (5·5 to 7·4)	227·3 (207·7 to 247·6)	15·3 (11·8 to 19·7)	185·7 (166·4 to 204·9)	0·0 (0·0 to 0·1)	26·3 (22·7 to 30·8)
Afghanistan*	2045	55·3 (50·5 to 60·3)	3·7 (2·9 to 4·8)	45·3 (40·6 to 50·0)	0·0 (0·0 to 0·0)	6·2 (5·4 to 7·3)	229·6 (209·5 to 250·2)	15·5 (12·0 to 19·9)	188·2 (168·5 to 207·6)	0·0 (0·0 to 0·1)	25·8 (22·3 to 30·1)
Afghanistan*	2046	55·9 (51·0 to 60·9)	3·8 (2·9 to 4·9)	46·0 (41·2 to 50·8)	0·0 (0·0 to 0·0)	6·1 (5·2 to 7·2)	232·1 (211·8 to 252·8)	15·8 (12·2 to 20·3)	190·9 (170·9 to 210·7)	0·0 (0·0 to 0·1)	25·3 (21·7 to 29·7)
Afghanistan*	2047	56·5 (51·5 to 61·7)	3·9 (3·0 to 5·0)	46·7 (41·8 to 51·5)	0·0 (0·0 to 0·0)	6·0 (5·1 to 7·1)	234·6 (213·9 to 256·0)	16·1 (12·4 to 20·6)	193·6 (173·3 to 213·6)	0·0 (0·0 to 0·1)	24·8 (21·2 to 29·4)
Afghanistan*	2048	57·1 (52·1 to 62·3)	3·9 (3·0 to 5·0)	47·3 (42·4 to 52·2)	0·0 (0·0 to 0·0)	5·9 (5·0 to 6·9)	237·2 (216·4 to 258·6)	16·4 (12·6 to 20·9)	196·5 (175·9 to 216·7)	0·0 (0·0 to 0·1)	24·3 (20·7 to 28·7)

Afghanistan*	2049	57·8 (52·7 to 63·1)	4·0 (3·1 to 5·1)	48·0 (43·0 to 52·9)	0·0 (0·0 to 0·0)	5·7 (4·9 to 6·9)	239·8 (218·6 to 261·8)	16·6 (12·8 to 21·3)	199·3 (178·4 to 219·6)	0·0 (0·0 to 0·1)	23·8 (20·2 to 28·5)
Afghanistan*	2050	58·5 (53·3 to 63·9)	4·1 (3·1 to 5·2)	48·8 (43·7 to 53·7)	0·0 (0·0 to 0·0)	5·6 (4·8 to 6·7)	242·7 (221·3 to 265·1)	17·0 (13·1 to 21·7)	202·4 (181·2 to 223·1)	0·0 (0·0 to 0·1)	23·3 (19·8 to 27·8)
Angola*	1995	71·0 (62·1 to 80·4)	29·0 (24·0 to 34·7)	21·8 (17·5 to 27·2)	16·1 (11·0 to 22·5)	4·0 (4·0 to 4·0)	245·0 (214·5 to 277·6)	100·2 (82·8 to 119·9)	75·4 (60·4 to 93·8)	55·7 (38·1 to 77·6)	13·7 (13·7 to 13·7)
Angola*	1996	54·7 (48·5 to 61·3)	22·7 (18·9 to 27·0)	14·4 (11·5 to 18·0)	10·1 (6·9 to 14·1)	7·5 (7·5 to 7·5)	189·0 (167·5 to 211·7)	78·5 (65·1 to 93·3)	49·6 (39·6 to 62·3)	34·9 (23·9 to 48·7)	26·0 (26·0 to 26·0)
Angola*	1997	49·1 (43·4 to 55·1)	22·9 (19·0 to 27·4)	14·4 (11·5 to 17·9)	8·3 (5·7 to 11·5)	3·5 (3·5 to 3·5)	169·5 (149·9 to 190·2)	79·2 (65·5 to 94·7)	49·6 (39·7 to 61·8)	28·5 (19·6 to 39·6)	12·1 (12·1 to 12·1)
Angola*	1998	44·8 (39·4 to 50·4)	21·2 (17·5 to 25·3)	14·6 (11·7 to 18·1)	7·1 (4·8 to 9·8)	2·0 (2·0 to 2·0)	154·6 (136·1 to 173·9)	73·0 (60·5 to 87·2)	50·3 (40·3 to 62·4)	24·4 (16·7 to 33·9)	6·9 (6·9 to 6·9)
Angola*	1999	50·9 (44·6 to 57·6)	26·4 (21·8 to 31·3)	15·2 (12·1 to 18·8)	6·8 (4·8 to 9·6)	2·5 (2·5 to 2·5)	175·8 (154·1 to 198·9)	91·0 (75·4 to 107·9)	52·5 (41·7 to 64·9)	23·6 (16·4 to 33·2)	8·8 (8·8 to 8·8)
Angola*	2000	47·6 (41·9 to 53·9)	23·6 (19·7 to 28·1)	15·9 (12·8 to 19·7)	5·4 (3·7 to 7·7)	2·6 (2·6 to 2·6)	164·2 (144·7 to 186·0)	81·6 (68·1 to 96·9)	55·0 (44·2 to 68·1)	18·8 (12·9 to 26·6)	8·9 (8·9 to 8·9)
Angola*	2001	54·5 (48·1 to 61·5)	24·3 (20·3 to 28·9)	21·5 (17·3 to 26·6)	5·6 (3·8 to 7·9)	3·1 (3·1 to 3·1)	188·2 (166·2 to 212·4)	83·9 (70·2 to 99·8)	74·3 (59·7 to 91·7)	19·3 (13·3 to 27·2)	10·7 (10·7 to 10·7)
Angola*	2002	57·6 (51·0 to 65·2)	24·7 (20·7 to 29·3)	24·7 (19·9 to 30·6)	5·7 (3·9 to 8·0)	2·5 (2·5 to 2·5)	199·0 (176·2 to 225·1)	85·3 (71·6 to 101·3)	85·3 (68·8 to 105·6)	19·7 (13·6 to 27·7)	8·7 (8·7 to 8·7)
Angola*	2003	62·6 (55·3 to 71·3)	27·2 (22·8 to 32·2)	26·8 (21·5 to 33·2)	5·9 (4·0 to 8·3)	2·7 (2·7 to 2·7)	216·0 (190·9 to 246·0)	93·7 (78·7 to 111·3)	92·5 (74·2 to 114·5)	20·5 (13·9 to 28·5)	9·2 (9·2 to 9·2)
Angola*	2004	63·4 (56·0 to 71·9)	27·5 (23·1 to 32·7)	26·3 (21·2 to 32·5)	6·0 (4·1 to 8·3)	3·5 (3·5 to 3·5)	218·8 (193·3 to 248·3)	95·0 (79·9 to 112·8)	90·9 (73·0 to 112·2)	20·9 (14·3 to 28·7)	11·9 (11·9 to 11·9)
Angola*	2005	64·1 (57·0 to 71·9)	27·9 (23·4 to 33·1)	23·2 (18·6 to 28·7)	5·9 (4·1 to 8·1)	7·1 (7·1 to 7·1)	221·4 (196·8 to 248·2)	96·4 (80·9 to 114·3)	80·2 (64·3 to 99·1)	20·4 (14·1 to 27·8)	24·4 (24·4 to 24·4)
Angola*	2006	64·5 (57·0 to 72·8)	33·7 (28·7 to 39·9)	21·1 (16·9 to 26·2)	6·5 (4·5 to 9·2)	3·2 (3·2 to 3·2)	222·5 (196·7 to 251·5)	116·2 (99·1 to 137·9)	72·8 (58·3 to 90·5)	22·4 (15·5 to 31·6)	11·1 (11·1 to 11·1)

Angola*	2007	75·7 (67·3 to 84·8)	42·9 (36·6 to 50·1)	20·5 (16·4 to 25·3)	8·1 (5·6 to 11·5)	4·2 (4·2 to 4·2)	261·5 (232·3 to 292·9)	148·2 (126·3 to 172·9)	70·8 (56·6 to 87·5)	28·1 (19·4 to 39·6)	14·4 (14·4 to 14·4)
Angola*	2008	94·0 (83·6 to 105·3)	57·9 (49·7 to 67·2)	20·2 (16·0 to 24·9)	10·6 (7·3 to 15·0)	5·3 (5·3 to 5·3)	324·5 (288·8 to 363·5)	199·9 (171·5 to 232·2)	69·7 (55·2 to 86·1)	36·5 (25·2 to 51·9)	18·3 (18·3 to 18·3)
Angola*	2009	84·7 (75·7 to 95·9)	52·4 (44·8 to 61·1)	17·0 (13·4 to 20·9)	11·1 (7·7 to 15·9)	4·2 (4·2 to 4·2)	292·5 (261·2 to 330·9)	180·8 (154·7 to 210·9)	58·7 (46·1 to 72·3)	38·5 (26·7 to 54·8)	14·5 (14·5 to 14·5)
Angola*	2010	75·8 (67·5 to 86·1)	46·9 (39·8 to 55·0)	15·1 (11·8 to 18·9)	10·5 (7·2 to 15·0)	3·3 (3·3 to 3·3)	261·8 (233·1 to 297·1)	162·0 (137·3 to 189·8)	52·1 (40·8 to 65·1)	36·1 (25·0 to 51·9)	11·5 (11·5 to 11·5)
Angola*	2011	72·4 (64·2 to 82·1)	45·3 (38·3 to 53·0)	14·4 (11·3 to 18·2)	10·1 (6·9 to 14·4)	2·5 (2·5 to 2·5)	249·9 (221·7 to 283·5)	156·4 (132·3 to 183·1)	49·8 (39·2 to 62·7)	34·9 (23·8 to 49·8)	8·8 (8·8 to 8·8)
Angola*	2012	72·4 (63·9 to 81·6)	44·3 (37·1 to 51·9)	14·7 (11·6 to 18·7)	10·2 (7·0 to 14·6)	3·2 (3·2 to 3·2)	249·8 (220·5 to 281·8)	152·9 (128·2 to 179·0)	50·9 (40·1 to 64·5)	35·0 (24·2 to 50·3)	11·0 (11·0 to 11·0)
Angola*	2013	74·5 (66·0 to 84·0)	43·5 (36·4 to 50·7)	16·6 (13·3 to 20·9)	10·6 (7·4 to 15·0)	3·8 (3·8 to 3·8)	257·2 (227·7 to 289·8)	150·2 (125·8 to 175·0)	57·2 (45·8 to 72·2)	36·7 (25·7 to 51·9)	13·1 (13·1 to 13·1)
Angola*	2014	73·0 (64·7 to 82·7)	41·0 (34·3 to 48·3)	18·6 (14·9 to 23·4)	11·0 (7·6 to 15·5)	2·4 (2·4 to 2·4)	252·1 (223·3 to 285·5)	141·4 (118·3 to 166·8)	64·3 (51·5 to 80·8)	38·0 (26·3 to 53·4)	8·3 (8·3 to 8·3)
Angola*	2015	66·9 (59·1 to 75·9)	33·3 (27·7 to 39·3)	19·9 (16·0 to 25·2)	10·9 (7·5 to 15·3)	2·8 (2·8 to 2·8)	230·9 (204·0 to 262·1)	114·9 (95·7 to 135·7)	68·6 (55·4 to 86·9)	37·7 (26·0 to 52·8)	9·7 (9·7 to 9·7)
Angola*	2016	63·8 (56·4 to 72·0)	28·9 (24·2 to 34·2)	20·5 (16·5 to 25·5)	10·8 (7·5 to 15·2)	3·6 (3·6 to 3·6)	220·3 (194·8 to 248·4)	99·8 (83·4 to 118·1)	70·8 (56·9 to 88·2)	37·3 (25·8 to 52·4)	12·5 (12·5 to 12·5)
Angola*	2017	65·8 (57·9 to 73·4)	29·7 (24·7 to 35·3)	21·4 (17·3 to 26·5)	11·1 (7·6 to 15·5)	3·6 (3·6 to 3·6)	227·3 (200·1 to 253·3)	102·6 (85·4 to 121·9)	74·0 (59·6 to 91·6)	38·4 (26·3 to 53·5)	12·3 (12·3 to 12·3)
Angola*	2018	58·5 (51·7 to 66·4)	25·6 (21·3 to 30·4)	20·3 (16·3 to 25·1)	10·4 (7·1 to 14·6)	2·3 (2·3 to 2·3)	202·1 (178·4 to 229·3)	88·3 (73·5 to 105·1)	70·2 (56·2 to 86·6)	35·8 (24·6 to 50·3)	7·8 (7·8 to 7·8)
Angola*	2019	56·5 (49·8 to 64·4)	24·1 (19·8 to 29·1)	19·5 (15·6 to 24·1)	10·2 (7·0 to 14·3)	2·7 (2·7 to 2·8)	195·1 (172·0 to 222·3)	83·3 (68·4 to 100·5)	67·3 (53·9 to 83·1)	35·1 (24·1 to 49·4)	9·4 (9·2 to 9·7)
Angola*	2020	57·3 (50·4 to 65·8)	25·0 (20·6 to 30·6)	18·8 (15·0 to 23·1)	10·0 (6·9 to 14·1)	3·5 (3·4 to 3·6)	197·8 (173·9 to 227·3)	86·4 (71·1 to 105·5)	64·8 (51·9 to 79·9)	34·7 (23·7 to 48·7)	11·9 (11·7 to 12·3)

Angola*	2021	55·3 (48·7 to 63·5)	24·0 (19·8 to 29·4)	18·0 (14·4 to 22·2)	9·9 (6·8 to 13·9)	3·4 (3·3 to 3·6)	190·9 (168·0 to 219·2)	83·0 (68·3 to 101·4)	62·1 (49·7 to 76·6)	34·0 (23·4 to 47·8)	11·8 (11·5 to 12·5)
Angola*	2022	53·4 (46·6 to 61·8)	22·5 (18·0 to 28·4)	18·1 (14·5 to 22·3)	10·2 (7·0 to 14·3)	2·6 (2·5 to 2·9)	184·3 (160·9 to 213·4)	77·6 (62·0 to 98·2)	62·6 (50·1 to 77·1)	35·1 (24·2 to 49·3)	9·0 (8·5 to 9·9)
Angola*	2023	54·1 (47·1 to 62·8)	22·7 (17·9 to 28·8)	18·3 (14·6 to 22·5)	10·5 (7·2 to 14·8)	2·6 (2·5 to 2·9)	186·9 (162·6 to 216·8)	78·5 (61·7 to 99·4)	63·1 (50·5 to 77·8)	36·2 (24·9 to 51·0)	9·1 (8·5 to 10·0)
Angola*	2024	54·9 (47·7 to 63·6)	23·0 (17·9 to 29·7)	18·5 (14·8 to 22·8)	10·8 (7·4 to 15·2)	2·6 (2·4 to 2·9)	189·7 (164·5 to 219·7)	79·5 (61·6 to 102·5)	63·7 (51·1 to 78·6)	37·4 (25·7 to 52·6)	9·1 (8·4 to 10·1)
Angola*	2025	55·8 (48·1 to 65·1)	23·3 (17·9 to 30·1)	18·7 (15·0 to 23·0)	11·2 (7·7 to 15·8)	2·6 (2·4 to 3·0)	192·5 (166·1 to 224·6)	80·3 (61·9 to 103·9)	64·4 (51·6 to 79·5)	38·6 (26·5 to 54·4)	9·1 (8·4 to 10·2)
Angola*	2026	56·7 (48·7 to 66·4)	23·6 (18·1 to 31·0)	18·9 (15·1 to 23·3)	11·6 (8·0 to 16·3)	2·6 (2·4 to 3·0)	195·7 (168·0 to 229·1)	81·4 (62·6 to 107·0)	65·3 (52·3 to 80·5)	40·0 (27·5 to 56·2)	9·0 (8·2 to 10·2)
Angola*	2027	57·8 (49·6 to 67·7)	24·1 (18·2 to 31·7)	19·2 (15·3 to 23·7)	12·0 (8·3 to 16·9)	2·6 (2·4 to 2·9)	199·5 (171·1 to 233·6)	83·0 (62·9 to 109·3)	66·2 (53·0 to 81·7)	41·4 (28·5 to 58·2)	8·9 (8·1 to 10·1)
Angola*	2028	59·1 (50·6 to 69·5)	24·7 (18·6 to 32·5)	19·5 (15·6 to 24·1)	12·5 (8·6 to 17·5)	2·5 (2·3 to 2·9)	204·1 (174·8 to 240·0)	85·2 (64·1 to 112·3)	67·2 (53·8 to 83·0)	43·0 (29·6 to 60·5)	8·7 (7·9 to 9·9)
Angola*	2029	60·6 (51·8 to 71·3)	25·4 (18·9 to 33·5)	19·8 (15·8 to 24·5)	13·0 (8·9 to 18·3)	2·5 (2·2 to 2·8)	209·3 (178·9 to 246·3)	87·7 (65·2 to 115·6)	68·4 (54·7 to 84·5)	44·7 (30·8 to 63·0)	8·5 (7·7 to 9·8)
Angola*	2030	62·0 (52·7 to 73·1)	26·0 (19·3 to 34·2)	20·2 (16·1 to 24·9)	13·5 (9·3 to 19·0)	2·4 (2·2 to 2·8)	214·1 (181·9 to 252·2)	89·6 (66·8 to 118·1)	69·7 (55·7 to 86·1)	46·6 (32·2 to 65·7)	8·3 (7·4 to 9·5)
Angola*	2031	63·5 (53·8 to 74·7)	26·6 (19·5 to 34·9)	20·6 (16·4 to 25·4)	14·1 (9·7 to 19·8)	2·3 (2·1 to 2·7)	219·3 (185·8 to 258·0)	91·8 (67·2 to 120·6)	71·0 (56·8 to 87·7)	48·5 (33·4 to 68·4)	8·0 (7·2 to 9·3)
Angola*	2032	64·8 (55·1 to 76·4)	27·1 (20·0 to 36·3)	20·9 (16·7 to 25·8)	14·6 (10·0 to 20·5)	2·3 (2·0 to 2·6)	223·9 (190·1 to 263·7)	93·5 (69·2 to 125·4)	72·2 (57·7 to 89·1)	50·4 (34·7 to 70·9)	7·8 (7·0 to 9·1)
Angola*	2033	66·2 (55·8 to 78·3)	27·6 (20·2 to 36·9)	21·2 (17·0 to 26·2)	15·2 (10·4 to 21·3)	2·2 (2·0 to 2·6)	228·5 (192·6 to 270·3)	95·1 (69·9 to 127·4)	73·3 (58·6 to 90·5)	52·4 (35·9 to 73·7)	7·7 (6·9 to 8·9)
Angola*	2034	67·6 (56·7 to 80·2)	28·1 (20·3 to 37·5)	21·6 (17·3 to 26·6)	15·8 (10·8 to 22·2)	2·2 (1·9 to 2·5)	233·3 (195·9 to 277·0)	96·9 (70·2 to 129·5)	74·5 (59·6 to 92·0)	54·4 (37·4 to 76·6)	7·5 (6·7 to 8·8)

Angola*	2035	68·8 (57·7 to 81·8)	28·5 (20·5 to 38·4)	21·9 (17·5 to 27·0)	16·4 (11·2 to 23·0)	2·2 (1·9 to 2·5)	237·7 (199·2 to 282·3)	98·2 (70·7 to 132·6)	75·6 (60·4 to 93·3)	56·4 (38·5 to 79·4)	7·4 (6·6 to 8·7)
Angola*	2036	70·2 (58·5 to 83·5)	28·9 (20·3 to 39·1)	22·2 (17·8 to 27·4)	17·0 (11·5 to 23·9)	2·1 (1·9 to 2·5)	242·3 (201·8 to 288·3)	99·8 (70·1 to 134·9)	76·6 (61·4 to 94·6)	58·5 (39·8 to 82·5)	7·3 (6·5 to 8·7)
Angola*	2037	71·5 (59·3 to 85·6)	29·4 (20·4 to 40·5)	22·5 (18·0 to 27·8)	17·6 (11·9 to 24·9)	2·1 (1·9 to 2·5)	246·9 (204·7 to 295·6)	101·3 (70·4 to 139·8)	77·6 (62·3 to 95·9)	60·7 (41·2 to 85·9)	7·3 (6·4 to 8·6)
Angola*	2038	72·9 (60·1 to 87·5)	29·8 (20·6 to 41·3)	22·8 (18·3 to 28·2)	18·2 (12·3 to 25·8)	2·1 (1·8 to 2·5)	251·6 (207·7 to 302·2)	102·8 (71·0 to 142·6)	78·7 (63·2 to 97·3)	62·9 (42·6 to 89·1)	7·2 (6·4 to 8·6)
Angola*	2039	74·3 (61·1 to 89·3)	30·2 (20·4 to 42·2)	23·1 (18·6 to 28·6)	18·9 (12·8 to 26·8)	2·1 (1·8 to 2·5)	256·6 (211·0 to 308·4)	104·4 (70·6 to 145·6)	79·9 (64·1 to 98·7)	65·2 (44·0 to 92·5)	7·2 (6·3 to 8·6)
Angola*	2040	75·9 (62·3 to 91·5)	30·7 (20·9 to 43·5)	23·5 (18·8 to 29·0)	19·6 (13·2 to 27·8)	2·1 (1·8 to 2·5)	261·9 (215·1 to 315·9)	106·1 (72·0 to 150·1)	81·1 (65·0 to 100·2)	67·6 (45·6 to 96·0)	7·1 (6·2 to 8·6)
Angola*	2041	77·5 (63·7 to 93·6)	31·3 (21·0 to 44·5)	23·8 (19·1 to 29·5)	20·3 (13·7 to 28·9)	2·0 (1·8 to 2·5)	267·6 (219·8 to 323·0)	108·1 (72·6 to 153·7)	82·3 (66·0 to 101·8)	70·2 (47·2 to 99·7)	7·1 (6·1 to 8·6)
Angola*	2042	79·2 (64·8 to 96·1)	31·8 (21·0 to 45·3)	24·2 (19·4 to 29·9)	21·1 (14·2 to 30·0)	2·0 (1·7 to 2·5)	273·4 (223·8 to 331·7)	109·9 (72·4 to 156·4)	83·6 (67·1 to 103·4)	72·9 (48·9 to 103·6)	7·0 (6·0 to 8·6)
Angola*	2043	80·9 (66·0 to 98·9)	32·4 (21·3 to 46·3)	24·6 (19·7 to 30·4)	21·9 (14·7 to 31·2)	2·0 (1·7 to 2·5)	279·3 (228·0 to 341·5)	111·8 (73·6 to 159·9)	84·9 (68·1 to 105·0)	75·6 (50·6 to 107·7)	6·9 (6·0 to 8·6)
Angola*	2044	82·6 (67·3 to 101·3)	33·0 (21·6 to 47·3)	24·9 (20·0 to 30·8)	22·7 (15·1 to 32·6)	2·0 (1·7 to 2·5)	285·2 (232·2 to 349·8)	113·8 (74·7 to 163·2)	86·0 (69·1 to 106·4)	78·4 (52·2 to 112·5)	6·9 (6·0 to 8·5)
Angola*	2045	84·5 (68·7 to 103·9)	33·6 (22·0 to 49·1)	25·3 (20·3 to 31·3)	23·6 (15·6 to 34·1)	2·0 (1·7 to 2·5)	291·7 (237·2 to 358·6)	116·2 (76·1 to 169·5)	87·3 (70·1 to 108·1)	81·4 (53·8 to 117·6)	6·9 (5·9 to 8·5)
Angola*	2046	86·4 (69·8 to 106·8)	34·3 (22·2 to 51·0)	25·6 (20·6 to 31·7)	24·4 (16·2 to 35·5)	2·0 (1·7 to 2·5)	298·3 (240·9 to 368·7)	118·5 (76·8 to 175·9)	88·5 (71·1 to 109·6)	84·3 (55·9 to 122·7)	6·9 (5·9 to 8·7)
Angola*	2047	88·4 (71·3 to 109·6)	35·0 (22·9 to 52·3)	26·0 (20·9 to 32·2)	25·3 (16·8 to 37·0)	2·0 (1·7 to 2·5)	305·1 (246·3 to 378·4)	120·9 (79·0 to 180·5)	89·8 (72·2 to 111·2)	87·5 (57·8 to 127·7)	6·9 (5·8 to 8·7)
Angola*	2048	90·4 (72·6 to 111·9)	35·7 (23·0 to 53·0)	26·4 (21·2 to 32·7)	26·3 (17·2 to 38·4)	2·0 (1·7 to 2·6)	312·0 (250·6 to 386·2)	123·4 (79·3 to 182·9)	91·0 (73·1 to 112·8)	90·7 (59·4 to 132·5)	6·9 (5·8 to 8·9)

Angola*	2049	92·3 (74·0 to 113·8)	36·4 (22·9 to 54·0)	26·7 (21·5 to 33·1)	27·2 (17·8 to 40·0)	2·0 (1·7 to 2·6)	318·8 (255·4 to 393·0)	125·7 (79·2 to 186·4)	92·2 (74·1 to 114·3)	93·9 (61·3 to 138·0)	6·9 (5·8 to 9·0)
Angola*	2050	94·5 (75·4 to 117·4)	37·2 (23·2 to 56·5)	27·1 (21·7 to 33·6)	28·2 (18·4 to 41·6)	2·0 (1·7 to 2·7)	326·1 (260·5 to 405·2)	128·4 (80·0 to 195·2)	93·5 (75·0 to 115·9)	97·3 (63·6 to 143·6)	7·0 (5·8 to 9·2)
Albania*	1995	149·0 (135·6 to 164·4)	45·7 (38·7 to 54·1)	98·6 (85·9 to 112·5)	0·0 (0·0 to 0·0)	4·7 (4·7 to 4·7)	415·3 (377·8 to 458·2)	127·3 (107·8 to 150·6)	274·8 (239·4 to 313·6)	0·0 (0·0 to 0·0)	13·2 (13·2 to 13·2)
Albania*	1996	152·3 (139·1 to 166·5)	47·6 (40·7 to 55·9)	100·7 (89·3 to 113·7)	0·0 (0·0 to 0·0)	4·0 (4·0 to 4·0)	424·5 (387·7 to 463·9)	132·6 (113·5 to 155·9)	280·7 (248·9 to 316·9)	0·0 (0·0 to 0·0)	11·2 (11·2 to 11·2)
Albania*	1997	145·1 (133·2 to 158·3)	47·1 (40·5 to 55·1)	96·5 (86·4 to 108·4)	0·0 (0·0 to 0·0)	1·5 (1·5 to 1·5)	404·3 (371·2 to 441·1)	131·2 (112·9 to 153·6)	268·9 (240·8 to 302·2)	0·0 (0·0 to 0·0)	4·1 (4·1 to 4·1)
Albania*	1998	156·1 (143·6 to 169·4)	54·1 (47·0 to 62·7)	98·4 (87·9 to 110·4)	0·0 (0·0 to 0·0)	3·6 (3·6 to 3·6)	435·0 (400·3 to 472·2)	150·8 (131·1 to 174·6)	274·1 (244·9 to 307·6)	0·0 (0·0 to 0·0)	10·0 (10·0 to 10·0)
Albania*	1999	169·6 (157·4 to 183·3)	61·2 (53·8 to 70·4)	101·3 (91·6 to 112·2)	0·0 (0·0 to 0·0)	7·2 (7·2 to 7·2)	472·8 (438·8 to 510·8)	170·5 (149·9 to 196·2)	282·4 (255·2 to 312·8)	0·0 (0·0 to 0·0)	19·9 (19·9 to 19·9)
Albania*	2000	172·3 (160·9 to 184·1)	62·9 (55·5 to 71·6)	101·6 (93·3 to 109·8)	0·0 (0·0 to 0·0)	7·8 (7·8 to 7·8)	480·1 (448·3 to 513·1)	175·3 (154·7 to 199·6)	283·0 (260·0 to 306·1)	0·0 (0·0 to 0·0)	21·7 (21·7 to 21·7)
Albania*	2001	178·7 (169·3 to 189·0)	66·9 (59·3 to 75·6)	104·0 (98·5 to 109·5)	0·0 (0·0 to 0·0)	7·8 (7·8 to 7·8)	498·1 (471·7 to 526·8)	186·3 (165·3 to 210·8)	289·9 (274·6 to 305·3)	0·0 (0·0 to 0·0)	21·9 (21·9 to 21·9)
Albania*	2002	181·0 (172·6 to 190·3)	69·4 (61·6 to 78·4)	105·3 (102·6 to 107·7)	0·0 (0·0 to 0·0)	6·3 (6·3 to 6·3)	504·5 (480·9 to 530·4)	193·5 (171·7 to 218·4)	293·4 (285·8 to 300·3)	0·0 (0·0 to 0·0)	17·6 (17·6 to 17·6)
Albania*	2003	185·7 (176·8 to 195·4)	72·6 (64·0 to 81·3)	104·7 (102·0 to 107·3)	0·0 (0·0 to 0·0)	8·5 (8·5 to 8·5)	517·7 (492·7 to 544·6)	202·2 (178·4 to 226·6)	291·7 (284·4 to 299·1)	0·0 (0·0 to 0·0)	23·7 (23·7 to 23·7)
Albania*	2004	192·6 (181·4 to 203·9)	78·7 (69·6 to 88·0)	105·6 (100·3 to 111·0)	0·0 (0·0 to 0·0)	8·3 (8·3 to 8·3)	536·8 (505·6 to 568·2)	219·4 (194·1 to 245·3)	294·3 (279·4 to 309·3)	0·0 (0·0 to 0·0)	23·1 (23·1 to 23·1)
Albania*	2005	195·3 (183·5 to 208·5)	80·2 (71·4 to 89·5)	105·0 (96·9 to 113·4)	0·0 (0·0 to 0·0)	10·1 (10·1 to 10·1)	544·4 (511·4 to 581·2)	223·5 (198·9 to 249·5)	292·6 (270·1 to 316·2)	0·0 (0·0 to 0·0)	28·3 (28·3 to 28·3)
Albania*	2006	200·9 (187·6 to 215·2)	83·5 (74·3 to 93·7)	106·2 (96·8 to 116·8)	0·0 (0·0 to 0·0)	11·2 (11·2 to 11·2)	559·8 (522·9 to 599·9)	232·8 (207·2 to 261·2)	295·9 (269·8 to 325·5)	0·0 (0·0 to 0·0)	31·1 (31·1 to 31·1)

Albania*	2007	200·1 (185·9 to 215·5)	86·0 (77·0 to 96·2)	108·0 (98·0 to 119·1)	0·0 (0·0 to 0·0)	6·1 (6·1 to 6·1)	557·8 (518·0 to 600·6)	239·8 (214·5 to 268·2)	301·1 (273·0 to 331·9)	0·0 (0·0 to 0·0)	16·9 (16·9 to 16·9)
Albania*	2008	209·1 (194·5 to 225·2)	93·7 (84·3 to 104·7)	106·1 (95·7 to 118·4)	0·0 (0·0 to 0·0)	9·3 (9·3 to 9·3)	582·7 (542·0 to 627·6)	261·0 (234·8 to 291·8)	295·6 (266·8 to 329·9)	0·0 (0·0 to 0·1)	26·0 (26·0 to 26·0)
Albania*	2009	208·7 (193·3 to 225·2)	99·3 (89·6 to 110·7)	102·3 (92·0 to 114·4)	0·0 (0·0 to 0·0)	7·1 (7·1 to 7·1)	581·7 (538·8 to 627·5)	276·7 (249·7 to 308·5)	285·1 (256·5 to 318·8)	0·1 (0·0 to 0·1)	19·8 (19·8 to 19·8)
Albania*	2010	207·8 (191·8 to 223·7)	99·5 (89·4 to 110·8)	102·2 (91·2 to 113·9)	0·0 (0·0 to 0·0)	6·0 (6·0 to 6·0)	579·0 (534·5 to 623·5)	277·3 (249·1 to 308·7)	284·8 (254·1 to 317·4)	0·1 (0·1 to 0·1)	16·8 (16·8 to 16·8)
Albania*	2011	222·9 (207·7 to 239·8)	104·0 (93·5 to 115·2)	107·1 (96·1 to 118·9)	0·0 (0·0 to 0·1)	11·8 (11·8 to 11·8)	621·3 (578·8 to 668·2)	289·8 (260·6 to 321·0)	298·5 (267·8 to 331·4)	0·1 (0·1 to 0·1)	32·9 (32·9 to 32·9)
Albania*	2012	225·2 (209·6 to 242·4)	108·1 (97·0 to 119·6)	112·8 (102·3 to 124·6)	0·0 (0·0 to 0·0)	4·3 (4·3 to 4·3)	627·6 (584·0 to 675·6)	301·2 (270·3 to 333·2)	314·4 (285·2 to 347·2)	0·1 (0·1 to 0·1)	11·9 (11·9 to 11·9)
Albania*	2013	234·0 (218·0 to 251·6)	114·9 (103·8 to 126·8)	118·1 (107·2 to 130·0)	0·0 (0·0 to 0·0)	0·9 (0·9 to 0·9)	652·1 (607·6 to 701·2)	320·3 (289·3 to 353·4)	329·2 (298·7 to 362·4)	0·1 (0·0 to 0·1)	2·5 (2·5 to 2·5)
Albania*	2014	253·4 (237·9 to 272·0)	126·9 (115·7 to 140·3)	121·1 (109·8 to 134·0)	0·0 (0·0 to 0·0)	5·4 (5·4 to 5·4)	706·3 (663·1 to 758·1)	353·6 (322·5 to 391·0)	337·5 (306·0 to 373·4)	0·1 (0·0 to 0·1)	15·1 (15·1 to 15·1)
Albania*	2015	248·3 (232·6 to 265·9)	131·3 (119·7 to 144·1)	114·2 (102·7 to 125·9)	0·0 (0·0 to 0·0)	2·8 (2·8 to 2·8)	692·0 (648·4 to 741·0)	366·0 (333·6 to 401·6)	318·3 (286·1 to 350·8)	0·1 (0·0 to 0·1)	7·7 (7·7 to 7·7)
Albania*	2016	249·6 (232·9 to 267·0)	135·5 (123·3 to 148·4)	112·3 (100·9 to 124·7)	0·0 (0·0 to 0·0)	1·8 (1·8 to 1·8)	695·7 (649·0 to 744·3)	377·7 (343·8 to 413·6)	313·0 (281·1 to 347·6)	0·1 (0·1 to 0·1)	5·0 (5·0 to 5·0)
Albania*	2017	263·7 (244·3 to 283·5)	143·6 (130·5 to 157·8)	118·1 (105·9 to 131·4)	0·1 (0·1 to 0·1)	1·9 (1·9 to 1·9)	734·8 (681·0 to 790·2)	400·1 (363·7 to 439·8)	329·2 (295·0 to 366·3)	0·3 (0·2 to 0·4)	5·3 (5·3 to 5·3)
Albania*	2018	283·0 (261·3 to 306·6)	152·4 (136·6 to 169·2)	124·3 (108·9 to 141·2)	0·2 (0·1 to 0·2)	6·1 (6·1 to 6·1)	788·8 (728·3 to 854·5)	424·8 (380·7 to 471·4)	346·5 (303·6 to 393·4)	0·4 (0·3 to 0·6)	17·0 (17·0 to 17·0)
Albania*	2019	292·8 (270·3 to 317·1)	159·6 (142·7 to 177·1)	126·9 (111·3 to 144·0)	0·2 (0·1 to 0·2)	6·2 (6·1 to 6·2)	816·1 (753·3 to 883·8)	444·7 (397·6 to 493·5)	353·7 (310·1 to 401·5)	0·4 (0·3 to 0·7)	17·2 (17·1 to 17·4)
Albania*	2020	300·0 (276·0 to 325·2)	170·4 (152·5 to 190·9)	121·2 (106·2 to 137·5)	0·2 (0·1 to 0·2)	8·2 (8·0 to 8·8)	836·0 (769·1 to 906·3)	475·0 (425·0 to 532·0)	337·7 (296·0 to 383·3)	0·4 (0·3 to 0·6)	22·9 (22·3 to 24·7)

Albania*	2021	290·6 (267·3 to 315·0)	165·3 (147·8 to 185·1)	117·1 (102·7 to 132·9)	0·2 (0·1 to 0·2)	8·0 (7·7 to 8·7)	809·9 (744·9 to 877·9)	460·6 (412·1 to 515·8)	326·4 (286·2 to 370·5)	0·4 (0·3 to 0·6)	22·4 (21·5 to 24·2)
Albania*	2022	274·9 (252·0 to 299·2)	151·1 (133·7 to 169·9)	117·2 (102·8 to 133·0)	0·2 (0·1 to 0·2)	6·4 (6·0 to 7·1)	766·1 (702·3 to 833·9)	421·0 (372·6 to 473·5)	326·8 (286·5 to 370·6)	0·5 (0·3 to 0·7)	17·9 (16·6 to 19·8)
Albania*	2023	276·1 (253·4 to 300·8)	152·0 (134·9 to 171·3)	117·1 (102·8 to 133·0)	0·2 (0·1 to 0·2)	6·8 (6·3 to 7·6)	769·6 (706·2 to 838·4)	423·6 (376·0 to 477·5)	326·5 (286·4 to 370·6)	0·5 (0·3 to 0·7)	19·0 (17·4 to 21·1)
Albania*	2024	277·0 (254·2 to 302·0)	152·8 (135·1 to 172·7)	116·8 (102·6 to 132·5)	0·2 (0·1 to 0·3)	7·3 (6·6 to 8·2)	772·1 (708·3 to 841·7)	425·7 (376·6 to 481·2)	325·6 (285·8 to 369·3)	0·5 (0·3 to 0·7)	20·3 (18·5 to 23·0)
Albania*	2025	277·7 (254·2 to 301·7)	153·3 (135·7 to 174·3)	116·4 (102·3 to 132·1)	0·2 (0·1 to 0·3)	7·8 (7·0 to 8·9)	773·8 (708·6 to 840·7)	427·1 (378·3 to 485·9)	324·5 (285·1 to 368·3)	0·5 (0·4 to 0·7)	21·7 (19·6 to 24·9)
Albania*	2026	278·4 (255·0 to 302·8)	153·8 (136·1 to 175·9)	116·1 (102·1 to 131·8)	0·2 (0·1 to 0·3)	8·3 (7·4 to 9·6)	776·0 (710·7 to 843·9)	428·7 (379·4 to 490·2)	323·6 (284·5 to 367·2)	0·5 (0·4 to 0·7)	23·1 (20·7 to 26·9)
Albania*	2027	279·4 (255·1 to 304·7)	154·6 (135·6 to 176·5)	115·9 (101·9 to 131·5)	0·2 (0·1 to 0·3)	8·8 (7·8 to 10·4)	778·8 (710·9 to 849·3)	430·8 (377·9 to 492·0)	323·0 (284·1 to 366·4)	0·5 (0·4 to 0·8)	24·4 (21·7 to 29·0)
Albania*	2028	280·6 (255·5 to 307·0)	155·4 (136·5 to 177·4)	115·7 (101·8 to 131·0)	0·2 (0·1 to 0·3)	9·3 (8·2 to 11·3)	782·0 (712·0 to 855·6)	433·2 (380·4 to 494·3)	322·4 (283·7 to 365·0)	0·6 (0·4 to 0·8)	25·9 (22·8 to 31·4)
Albania*	2029	282·2 (257·1 to 309·1)	156·7 (137·5 to 179·4)	115·4 (101·6 to 130·7)	0·2 (0·1 to 0·3)	9·8 (8·6 to 12·2)	786·4 (716·5 to 861·3)	436·7 (383·1 to 500·0)	321·7 (283·2 to 364·3)	0·6 (0·4 to 0·8)	27·4 (24·0 to 34·0)
Albania*	2030	284·1 (259·3 to 311·0)	158·3 (138·9 to 180·9)	115·2 (101·4 to 130·5)	0·2 (0·2 to 0·3)	10·4 (9·1 to 13·1)	791·8 (722·6 to 866·7)	441·1 (387·2 to 504·2)	321·1 (282·6 to 363·7)	0·6 (0·4 to 0·8)	29·0 (25·3 to 36·6)
Albania*	2031	286·4 (261·6 to 314·3)	160·0 (140·1 to 183·2)	115·2 (101·4 to 130·6)	0·2 (0·2 to 0·3)	11·0 (9·4 to 14·1)	798·1 (729·0 to 875·9)	445·8 (390·5 to 510·6)	321·1 (282·7 to 363·9)	0·6 (0·4 to 0·9)	30·5 (26·3 to 39·2)
Albania*	2032	288·5 (263·1 to 317·1)	161·5 (141·4 to 184·6)	115·3 (101·6 to 130·6)	0·2 (0·2 to 0·3)	11·5 (9·8 to 15·1)	804·1 (733·1 to 883·8)	450·1 (394·1 to 514·6)	321·4 (283·1 to 363·9)	0·6 (0·5 to 0·9)	32·1 (27·4 to 42·0)
Albania*	2033	291·1 (265·8 to 319·7)	163·2 (142·8 to 187·4)	115·6 (101·8 to 130·8)	0·2 (0·2 to 0·3)	12·0 (10·2 to 16·1)	811·2 (740·7 to 891·1)	454·8 (398·0 to 522·3)	322·1 (283·8 to 364·5)	0·7 (0·5 to 0·9)	33·6 (28·4 to 44·8)
Albania*	2034	293·8 (267·3 to 322·2)	165·0 (144·2 to 189·9)	116·0 (102·2 to 131·1)	0·2 (0·2 to 0·3)	12·6 (10·6 to 17·1)	818·8 (744·9 to 897·9)	459·9 (401·7 to 529·1)	323·2 (284·7 to 365·5)	0·7 (0·5 to 1·0)	35·0 (29·7 to 47·5)

Albania*	2035	296·6 (269·8 to 326·7)	166·9 (145·7 to 192·1)	116·4 (102·6 to 131·7)	0·2 (0·2 to 0·4)	13·1 (11·0 to 18·0)	826·7 (752·1 to 910·4)	465·0 (406·0 to 535·5)	324·5 (285·9 to 366·9)	0·7 (0·5 to 1·0)	36·5 (30·7 to 50·3)
Albania*	2036	299·8 (272·2 to 330·2)	168·9 (147·0 to 194·2)	117·1 (103·2 to 132·4)	0·3 (0·2 to 0·4)	13·6 (11·3 to 18·9)	835·5 (758·7 to 920·1)	470·6 (409·6 to 541·3)	326·4 (287·5 to 369·1)	0·7 (0·5 to 1·0)	37·8 (31·5 to 52·6)
Albania*	2037	303·1 (275·6 to 333·0)	170·9 (149·0 to 196·7)	117·9 (103·8 to 133·3)	0·3 (0·2 to 0·4)	14·0 (11·7 to 19·9)	844·8 (768·0 to 928·2)	476·3 (415·4 to 548·2)	328·5 (289·4 to 371·6)	0·7 (0·5 to 1·1)	39·1 (32·5 to 55·3)
Albania*	2038	306·7 (277·9 to 336·9)	173·1 (150·5 to 199·3)	118·9 (104·7 to 134·5)	0·3 (0·2 to 0·4)	14·4 (11·8 to 20·7)	854·8 (774·4 to 939·0)	482·5 (419·5 to 555·5)	331·3 (291·7 to 374·9)	0·8 (0·5 to 1·1)	40·2 (33·0 to 57·7)
Albania*	2039	310·7 (282·1 to 341·3)	175·5 (153·2 to 202·3)	120·1 (105·7 to 135·9)	0·3 (0·2 to 0·4)	14·8 (12·0 to 21·4)	866·0 (786·3 to 951·1)	489·2 (426·9 to 563·9)	334·8 (294·5 to 378·8)	0·8 (0·6 to 1·2)	41·2 (33·6 to 59·7)
Albania*	2040	314·6 (284·9 to 345·2)	177·8 (154·6 to 205·4)	121·4 (106·7 to 137·3)	0·3 (0·2 to 0·4)	15·2 (12·3 to 22·4)	876·8 (794·0 to 962·2)	495·5 (430·9 to 572·4)	338·2 (297·3 to 382·8)	0·8 (0·6 to 1·2)	42·2 (34·4 to 62·3)
Albania*	2041	318·7 (288·4 to 350·6)	180·2 (156·3 to 208·0)	122·7 (107·8 to 138·9)	0·3 (0·2 to 0·4)	15·5 (12·5 to 23·0)	888·2 (803·9 to 977·2)	502·2 (435·7 to 579·6)	341·9 (300·4 to 387·1)	0·9 (0·6 to 1·2)	43·2 (34·7 to 64·2)
Albania*	2042	322·7 (291·5 to 355·7)	182·5 (158·6 to 210·9)	124·0 (108·9 to 140·4)	0·3 (0·2 to 0·5)	15·9 (12·6 to 23·7)	899·4 (812·3 to 991·5)	508·7 (442·0 to 587·7)	345·6 (303·4 to 391·4)	0·9 (0·6 to 1·3)	44·2 (35·2 to 66·1)
Albania*	2043	326·8 (295·1 to 360·7)	184·8 (160·2 to 214·7)	125·4 (110·1 to 142·0)	0·3 (0·2 to 0·5)	16·3 (12·8 to 24·6)	910·8 (822·3 to 1005·3)	515·2 (446·6 to 598·5)	349·4 (306·8 to 395·7)	0·9 (0·6 to 1·3)	45·3 (35·7 to 68·7)
Albania*	2044	331·1 (299·4 to 365·1)	187·3 (162·1 to 217·5)	126·9 (111·5 to 143·8)	0·3 (0·2 to 0·5)	16·6 (13·1 to 25·5)	922·9 (834·5 to 1017·6)	521·9 (451·7 to 606·1)	353·8 (310·8 to 400·9)	1·0 (0·7 to 1·4)	46·3 (36·5 to 71·0)
Albania*	2045	335·1 (302·6 to 369·9)	189·5 (163·4 to 221·6)	128·3 (112·8 to 145·6)	0·4 (0·2 to 0·5)	17·0 (13·3 to 26·4)	934·0 (843·4 to 1031·0)	528·0 (455·5 to 617·7)	357·6 (314·3 to 405·7)	1·0 (0·7 to 1·4)	47·4 (37·0 to 73·5)
Albania*	2046	339·3 (305·9 to 375·0)	191·7 (165·2 to 224·6)	129·7 (114·1 to 147·3)	0·4 (0·3 to 0·5)	17·4 (13·6 to 27·6)	945·5 (852·5 to 1045·1)	534·3 (460·5 to 625·8)	361·5 (317·9 to 410·6)	1·0 (0·7 to 1·5)	48·6 (37·9 to 76·9)
Albania*	2047	343·3 (308·4 to 379·9)	194·0 (166·5 to 227·6)	131·0 (115·2 to 148·8)	0·4 (0·3 to 0·6)	18·0 (13·8 to 28·6)	956·8 (859·6 to 1058·7)	540·6 (463·9 to 634·2)	365·1 (321·2 to 414·6)	1·1 (0·7 to 1·5)	50·0 (38·5 to 79·7)
Albania*	2048	347·3 (311·9 to 385·8)	196·2 (167·5 to 229·4)	132·1 (116·2 to 150·0)	0·4 (0·3 to 0·6)	18·6 (14·2 to 29·7)	967·9 (869·2 to 1075·2)	546·9 (466·9 to 639·3)	368·1 (323·7 to 418·1)	1·1 (0·7 to 1·6)	51·8 (39·6 to 82·7)

Albania*	2049	351·3 (315·1 to 391·2)	198·5 (168·2 to 232·2)	133·1 (117·0 to 151·2)	0·4 (0·3 to 0·6)	19·3 (14·7 to 31·1)	979·1 (878·1 to 1090·2)	553·2 (468·9 to 647·2)	371·0 (326·2 to 421·3)	1·1 (0·8 to 1·7)	53·7 (40·8 to 86·6)
Albania*	2050	355·3 (318·5 to 395·9)	200·8 (170·7 to 236·1)	134·0 (117·7 to 152·2)	0·4 (0·3 to 0·6)	20·1 (15·2 to 33·0)	990·1 (887·8 to 1103·4)	559·5 (475·7 to 658·0)	373·4 (328·2 to 424·1)	1·2 (0·8 to 1·7)	56·0 (42·4 to 92·0)
Andorra	1995	2384·3 (2270·2 to 2509·8)	1627·0 (1520·9 to 1740·1)	329·7 (302·5 to 360·4)	427·6 (396·8 to 459·9)	0·0 (0·0 to 0·0)	2540·1 (2418·6 to 2673·8)	1733·3 (1620·3 to 1853·8)	351·2 (322·3 to 383·9)	455·6 (422·7 to 489·9)	0·0 (0·0 to 0·0)
Andorra	1996	2402·0 (2312·7 to 2493·2)	1614·5 (1529·3 to 1702·3)	334·1 (312·6 to 355·1)	453·4 (429·2 to 481·7)	0·0 (0·0 to 0·0)	2559·0 (2463·8 to 2656·1)	1720·0 (1629·2 to 1813·5)	355·9 (333·0 to 378·3)	483·0 (457·2 to 513·1)	0·0 (0·0 to 0·0)
Andorra	1997	2511·6 (2423·4 to 2598·5)	1669·3 (1589·3 to 1755·4)	349·8 (329·0 to 371·9)	492·5 (466·6 to 519·3)	0·0 (0·0 to 0·0)	2675·7 (2581·7 to 2768·3)	1778·4 (1693·1 to 1870·0)	372·6 (350·5 to 396·2)	524·7 (497·1 to 553·2)	0·0 (0·0 to 0·0)
Andorra	1998	2562·8 (2478·9 to 2652·5)	1679·9 (1606·5 to 1761·4)	360·0 (340·7 to 383·0)	522·9 (497·1 to 549·0)	0·0 (0·0 to 0·0)	2730·2 (2640·8 to 2825·7)	1789·7 (1711·4 to 1876·5)	383·5 (363·0 to 408·0)	557·0 (529·6 to 584·8)	0·0 (0·0 to 0·0)
Andorra	1999	2509·1 (2424·9 to 2596·3)	1659·0 (1581·1 to 1743·9)	365·8 (347·5 to 387·0)	484·3 (461·8 to 508·8)	0·0 (0·0 to 0·0)	2673·1 (2583·3 to 2765·9)	1767·4 (1684·4 to 1857·8)	389·7 (370·2 to 412·3)	515·9 (491·9 to 542·0)	0·0 (0·0 to 0·0)
Andorra	2000	2432·4 (2347·0 to 2514·4)	1617·5 (1536·3 to 1697·4)	366·0 (348·2 to 385·5)	448·9 (428·9 to 470·9)	0·0 (0·0 to 0·0)	2591·3 (2500·3 to 2678·6)	1723·2 (1636·7 to 1808·3)	390·0 (371·0 to 410·7)	478·2 (456·9 to 501·7)	0·0 (0·0 to 0·0)
Andorra	2001	2502·4 (2414·3 to 2588·9)	1671·6 (1586·3 to 1754·9)	377·5 (361·2 to 395·7)	453·3 (434·9 to 474·3)	0·0 (0·0 to 0·0)	2665·9 (2572·0 to 2758·0)	1780·8 (1689·9 to 1869·5)	402·1 (384·8 to 421·6)	483·0 (463·4 to 505·3)	0·0 (0·0 to 0·0)
Andorra	2002	2589·7 (2495·4 to 2674·5)	1717·4 (1629·6 to 1802·3)	391·9 (374·7 to 409·9)	480·5 (460·7 to 500·0)	0·0 (0·0 to 0·0)	2758·9 (2658·4 to 2849·2)	1829·6 (1736·0 to 1920·1)	417·5 (399·1 to 436·6)	511·9 (490·7 to 532·6)	0·0 (0·0 to 0·0)
Andorra	2003	2599·4 (2509·5 to 2690·0)	1692·2 (1609·5 to 1773·5)	408·0 (390·4 to 424·6)	499·2 (479·6 to 518·6)	0·0 (0·0 to 0·0)	2769·3 (2673·5 to 2865·8)	1802·8 (1714·7 to 1889·4)	434·7 (415·9 to 452·3)	531·8 (510·9 to 552·4)	0·0 (0·0 to 0·0)
Andorra	2004	2552·9 (2467·6 to 2641·2)	1625·7 (1547·8 to 1706·0)	418·0 (401·7 to 434·5)	509·2 (490·5 to 529·0)	0·0 (0·0 to 0·0)	2719·7 (2628·8 to 2813·8)	1731·9 (1648·9 to 1817·5)	445·4 (427·9 to 462·9)	542·5 (522·5 to 563·5)	0·0 (0·0 to 0·0)
Andorra	2005	2601·5 (2519·5 to 2689·1)	1638·2 (1556·3 to 1718·1)	433·0 (415·7 to 450·8)	530·2 (509·6 to 550·6)	0·0 (0·0 to 0·0)	2771·4 (2684·1 to 2864·7)	1745·3 (1658·0 to 1830·3)	461·3 (442·8 to 480·3)	564·9 (542·9 to 586·6)	0·0 (0·0 to 0·0)
Andorra	2006	2518·7 (2435·2 to 2607·3)	1565·0 (1486·4 to 1651·5)	430·9 (413·3 to 448·2)	522·9 (503·8 to 542·7)	0·0 (0·0 to 0·0)	2683·3 (2594·3 to 2777·6)	1667·3 (1583·5 to 1759·4)	459·0 (440·3 to 477·4)	557·0 (536·7 to 578·1)	0·0 (0·0 to 0·0)

Andorra	2007	2500·5 (2416·9 to 2587·6)	1578·1 (1497·7 to 1664·7)	417·7 (400·6 to 435·6)	504·7 (484·0 to 525·0)	0·0 (0·0 to 0·0)	2663·9 (2574·8 to 2756·7)	1681·2 (1595·6 to 1773·5)	445·0 (426·7 to 464·1)	537·7 (515·6 to 559·3)	0·0 (0·0 to 0·0)
Andorra	2008	2531·5 (2447·1 to 2624·6)	1671·7 (1590·5 to 1757·2)	391·7 (375·6 to 409·9)	468·2 (448·2 to 487·5)	0·0 (0·0 to 0·0)	2696·9 (2607·0 to 2796·1)	1780·9 (1694·4 to 1872·0)	417·2 (400·1 to 436·7)	498·8 (477·5 to 519·3)	0·0 (0·0 to 0·0)
Andorra	2009	2594·3 (2502·4 to 2686·3)	1774·1 (1686·4 to 1863·9)	372·4 (355·0 to 389·3)	447·7 (428·4 to 466·5)	0·0 (0·0 to 0·0)	2763·8 (2665·9 to 2861·8)	1890·0 (1796·6 to 1985·6)	396·8 (378·2 to 414·7)	477·0 (456·4 to 496·9)	0·0 (0·0 to 0·0)
Andorra	2010	2650·4 (2557·0 to 2744·7)	1845·2 (1756·8 to 1931·8)	364·3 (348·1 to 381·4)	440·9 (422·1 to 460·3)	0·0 (0·0 to 0·0)	2823·5 (2724·0 to 2924·0)	1965·7 (1871·6 to 2058·0)	388·1 (370·8 to 406·3)	469·7 (449·6 to 490·3)	0·0 (0·0 to 0·0)
Andorra	2011	2615·3 (2530·6 to 2708·3)	1801·0 (1716·8 to 1888·9)	365·7 (348·7 to 383·3)	448·5 (428·3 to 467·8)	0·0 (0·0 to 0·0)	2786·1 (2695·9 to 2885·3)	1918·7 (1828·9 to 2012·4)	389·6 (371·5 to 408·3)	477·8 (456·3 to 498·3)	0·0 (0·0 to 0·0)
Andorra	2012	2583·8 (2497·5 to 2667·9)	1754·8 (1670·4 to 1836·9)	370·5 (354·2 to 386·9)	458·5 (439·5 to 479·7)	0·0 (0·0 to 0·0)	2752·6 (2660·6 to 2842·2)	1869·5 (1779·5 to 1957·0)	394·7 (377·4 to 412·2)	488·5 (468·2 to 511·1)	0·0 (0·0 to 0·0)
Andorra	2013	2585·0 (2503·1 to 2671·9)	1745·1 (1666·3 to 1826·2)	374·0 (357·3 to 391·1)	465·9 (447·1 to 486·5)	0·0 (0·0 to 0·0)	2753·9 (2666·6 to 2846·5)	1859·1 (1775·1 to 1945·5)	398·5 (380·7 to 416·6)	496·4 (476·3 to 518·3)	0·0 (0·0 to 0·0)
Andorra	2014	2562·5 (2480·4 to 2652·9)	1735·9 (1659·1 to 1821·0)	371·0 (354·5 to 388·6)	455·6 (437·5 to 474·8)	0·0 (0·0 to 0·0)	2729·9 (2642·5 to 2826·3)	1849·3 (1767·5 to 1940·0)	395·2 (377·7 to 414·0)	485·3 (466·1 to 505·8)	0·0 (0·0 to 0·0)
Andorra	2015	2582·2 (2498·2 to 2670·8)	1765·5 (1687·9 to 1852·4)	373·8 (357·0 to 392·3)	442·9 (424·8 to 461·0)	0·0 (0·0 to 0·0)	2750·9 (2661·5 to 2845·3)	1880·9 (1798·2 to 1973·4)	398·2 (380·4 to 418·0)	471·8 (452·5 to 491·2)	0·0 (0·0 to 0·0)
Andorra	2016	2761·0 (2679·0 to 2856·3)	1914·9 (1833·1 to 2001·8)	381·6 (365·0 to 399·4)	464·5 (445·5 to 483·8)	0·0 (0·0 to 0·0)	2941·4 (2854·1 to 3042·9)	2040·0 (1952·8 to 2132·6)	406·5 (388·8 to 425·5)	494·9 (474·7 to 515·5)	0·0 (0·0 to 0·0)
Andorra	2017	2821·2 (2725·5 to 2919·1)	1954·4 (1863·9 to 2048·5)	380·3 (365·1 to 398·0)	486·6 (467·4 to 507·0)	0·0 (0·0 to 0·0)	3005·5 (2903·6 to 3109·8)	2082·1 (1985·7 to 2182·4)	405·1 (389·0 to 424·0)	518·3 (497·9 to 540·1)	0·0 (0·0 to 0·0)
Andorra	2018	2876·2 (2759·7 to 2998·3)	1967·1 (1851·4 to 2086·1)	385·5 (365·6 to 410·5)	523·6 (498·1 to 549·3)	0·0 (0·0 to 0·0)	3064·1 (2940·0 to 3194·2)	2095·7 (1972·4 to 2222·3)	410·7 (389·5 to 437·3)	557·8 (530·6 to 585·2)	0·0 (0·0 to 0·0)
Andorra	2019	2948·2 (2826·9 to 3078·9)	2013·3 (1888·9 to 2143·7)	391·5 (371·1 to 416·7)	543·3 (516·4 to 571·0)	0·0 (0·0 to 0·0)	3140·8 (3011·6 to 3280·1)	2144·9 (2012·3 to 2283·7)	417·1 (395·4 to 443·9)	578·8 (550·2 to 608·3)	0·0 (0·0 to 0·0)
Andorra	2020	2934·0 (2809·7 to 3071·4)	2043·7 (1915·3 to 2180·4)	367·0 (348·0 to 390·3)	523·3 (497·6 to 550·1)	0·0 (0·0 to 0·0)	3125·7 (2993·3 to 3272·0)	2177·2 (2040·5 to 2322·9)	390·9 (370·7 to 415·8)	557·5 (530·1 to 586·0)	0·0 (0·0 to 0·0)

Andorra	2021	2875·1 (2754·2 to 3010·0)	1992·7 (1867·6 to 2126·1)	357·9 (339·3 to 380·6)	524·5 (498·0 to 552·5)	0·0 (0·0 to 0·0)	3063·0 (2934·1 to 3206·6)	2122·9 (1989·6 to 2265·0)	381·3 (361·4 to 405·5)	558·8 (530·5 to 588·6)	0·0 (0·0 to 0·0)
Andorra	2022	2789·9 (2662·0 to 2924·3)	1892·5 (1764·7 to 2025·2)	358·0 (339·5 to 380·8)	539·4 (508·6 to 570·6)	0·0 (0·0 to 0·0)	2972·2 (2835·9 to 3115·4)	2016·2 (1880·0 to 2157·6)	381·3 (361·7 to 405·7)	574·7 (541·8 to 607·9)	0·0 (0·0 to 0·0)
Andorra	2023	2841·3 (2702·4 to 2986·3)	1928·8 (1786·6 to 2069·8)	357·8 (339·3 to 380·7)	554·7 (519·6 to 590·6)	0·0 (0·0 to 0·0)	3026·9 (2879·0 to 3181·4)	2054·9 (1903·4 to 2205·0)	381·1 (361·5 to 405·6)	591·0 (553·6 to 629·2)	0·0 (0·0 to 0·0)
Andorra	2024	2887·8 (2727·4 to 3051·8)	1962·2 (1801·5 to 2122·0)	356·5 (337·6 to 379·2)	569·2 (526·6 to 612·4)	0·0 (0·0 to 0·0)	3076·5 (2905·6 to 3251·2)	2090·4 (1919·2 to 2260·7)	379·8 (359·7 to 404·0)	606·4 (561·0 to 652·5)	0·0 (0·0 to 0·0)
Andorra	2025	2886·9 (2725·5 to 3051·3)	1949·1 (1792·2 to 2109·5)	354·7 (336·0 to 377·8)	583·1 (533·7 to 634·2)	0·0 (0·0 to 0·0)	3075·5 (2903·6 to 3250·6)	2076·4 (1909·2 to 2247·3)	377·8 (358·0 to 402·4)	621·2 (568·6 to 675·6)	0·0 (0·0 to 0·0)
Andorra	2026	2917·2 (2739·2 to 3114·4)	1968·2 (1797·7 to 2151·3)	352·6 (333·7 to 376·0)	596·4 (540·8 to 653·9)	0·0 (0·0 to 0·0)	3107·7 (2918·2 to 3317·9)	2096·8 (1915·2 to 2291·9)	375·6 (355·5 to 400·6)	635·3 (576·2 to 696·6)	0·0 (0·0 to 0·0)
Andorra	2027	2962·2 (2772·7 to 3172·5)	2002·7 (1818·4 to 2199·4)	350·4 (332·1 to 373·6)	609·1 (549·1 to 676·2)	0·0 (0·0 to 0·0)	3155·8 (2953·9 to 3379·8)	2133·6 (1937·2 to 2343·1)	373·3 (353·8 to 398·0)	648·8 (585·0 to 720·4)	0·0 (0·0 to 0·0)
Andorra	2028	3004·3 (2798·9 to 3228·9)	2037·9 (1835·0 to 2245·0)	347·9 (329·3 to 370·8)	618·5 (556·0 to 686·8)	0·0 (0·0 to 0·0)	3200·6 (2981·7 to 3439·8)	2171·1 (1954·9 to 2391·7)	370·7 (350·8 to 395·0)	658·9 (592·3 to 731·6)	0·0 (0·0 to 0·0)
Andorra	2029	3032·1 (2817·2 to 3269·2)	2060·7 (1843·8 to 2273·3)	345·0 (326·5 to 367·4)	626·3 (562·4 to 697·0)	0·0 (0·0 to 0·0)	3230·2 (3001·3 to 3482·8)	2195·4 (1964·2 to 2421·9)	367·6 (347·8 to 391·4)	667·3 (599·1 to 742·6)	0·0 (0·0 to 0·0)
Andorra	2030	3029·3 (2805·9 to 3281·7)	2053·2 (1831·4 to 2289·2)	342·0 (323·5 to 364·1)	634·1 (568·3 to 707·0)	0·0 (0·0 to 0·0)	3227·2 (2989·2 to 3496·1)	2187·4 (1951·1 to 2438·7)	364·3 (344·7 to 387·9)	675·5 (605·5 to 753·1)	0·0 (0·0 to 0·0)
Andorra	2031	3054·3 (2825·9 to 3309·0)	2074·3 (1850·8 to 2333·0)	338·7 (320·5 to 360·4)	641·4 (573·9 to 716·9)	0·0 (0·0 to 0·0)	3253·9 (3010·5 to 3525·2)	2209·8 (1971·7 to 2485·4)	360·8 (341·4 to 384·0)	683·3 (611·3 to 763·7)	0·0 (0·0 to 0·0)
Andorra	2032	3055·5 (2815·6 to 3313·8)	2072·9 (1835·5 to 2322·6)	334·8 (316·7 to 356·2)	647·9 (575·8 to 726·2)	0·0 (0·0 to 0·0)	3255·2 (2999·6 to 3530·3)	2208·3 (1955·4 to 2474·4)	356·6 (337·4 to 379·4)	690·2 (613·4 to 773·6)	0·0 (0·0 to 0·0)
Andorra	2033	3052·2 (2803·5 to 3323·2)	2068·3 (1819·9 to 2333·0)	330·4 (312·6 to 351·5)	653·5 (577·5 to 736·2)	0·0 (0·0 to 0·0)	3251·6 (2986·7 to 3540·4)	2203·4 (1938·8 to 2485·4)	352·0 (333·0 to 374·5)	696·2 (615·2 to 784·3)	0·0 (0·0 to 0·0)
Andorra	2034	3045·8 (2783·2 to 3348·1)	2061·6 (1801·0 to 2339·7)	325·8 (308·2 to 346·8)	658·4 (579·4 to 746·6)	0·0 (0·0 to 0·0)	3244·8 (2965·1 to 3566·9)	2196·3 (1918·7 to 2492·5)	347·1 (328·4 to 369·4)	701·4 (617·2 to 795·3)	0·0 (0·0 to 0·0)

Andorra	2035	3034·1 (2768·0 to 3344·0)	2050·6 (1786·4 to 2361·8)	320·9 (303·2 to 342·5)	662·6 (581·1 to 756·9)	0·0 (0·0 to 0·0)	3232·4 (2948·8 to 3562·5)	2184·6 (1903·1 to 2516·1)	341·8 (323·0 to 364·9)	705·9 (619·1 to 806·4)	0·0 (0·0 to 0·0)
Andorra	2036	3020·1 (2735·8 to 3353·5)	2038·4 (1749·1 to 2387·1)	315·6 (297·9 to 336·9)	666·1 (581·7 to 765·6)	0·0 (0·0 to 0·0)	3217·4 (2914·5 to 3572·6)	2171·6 (1863·4 to 2543·1)	336·2 (317·4 to 358·9)	709·6 (619·8 to 815·7)	0·0 (0·0 to 0·0)
Andorra	2037	2999·9 (2704·1 to 3355·5)	2021·8 (1722·9 to 2390·2)	309·9 (292·2 to 330·9)	668·3 (583·1 to 772·1)	0·0 (0·0 to 0·0)	3195·9 (2880·7 to 3574·7)	2153·8 (1835·5 to 2546·3)	330·1 (311·3 to 352·5)	712·0 (621·2 to 822·6)	0·0 (0·0 to 0·0)
Andorra	2038	2991·2 (2687·8 to 3373·9)	2012·2 (1716·1 to 2398·8)	305·5 (288·0 to 326·6)	673·4 (585·0 to 783·2)	0·0 (0·0 to 0·0)	3186·6 (2863·4 to 3594·4)	2143·7 (1828·2 to 2555·5)	325·5 (306·8 to 348·0)	717·4 (623·2 to 834·3)	0·0 (0·0 to 0·0)
Andorra	2039	2979·7 (2662·0 to 3391·0)	2001·2 (1688·6 to 2415·5)	300·8 (283·5 to 321·8)	677·7 (586·1 to 791·4)	0·0 (0·0 to 0·0)	3174·4 (2835·9 to 3612·6)	2132·0 (1798·9 to 2573·3)	320·5 (302·0 to 342·8)	721·9 (624·4 to 843·2)	0·0 (0·0 to 0·0)
Andorra	2040	2966·1 (2637·1 to 3409·1)	1988·7 (1663·3 to 2430·8)	296·0 (278·8 to 316·6)	681·4 (586·3 to 802·9)	0·0 (0·0 to 0·0)	3159·9 (2809·4 to 3631·8)	2118·7 (1772·0 to 2589·6)	315·3 (297·0 to 337·3)	725·9 (624·7 to 855·4)	0·0 (0·0 to 0·0)
Andorra	2041	2955·4 (2614·3 to 3421·1)	1978·0 (1651·5 to 2438·3)	291·5 (274·5 to 312·1)	685·9 (585·8 to 813·2)	0·0 (0·0 to 0·0)	3148·4 (2785·1 to 3644·6)	2107·2 (1759·3 to 2597·6)	310·6 (292·4 to 332·5)	730·7 (624·1 to 866·3)	0·0 (0·0 to 0·0)
Andorra	2042	2942·9 (2588·6 to 3444·3)	1965·6 (1626·1 to 2448·2)	287·1 (270·2 to 307·5)	690·3 (585·9 to 823·4)	0·0 (0·0 to 0·0)	3135·2 (2757·7 to 3669·3)	2094·0 (1732·4 to 2608·1)	305·8 (287·8 to 327·6)	735·4 (624·2 to 877·2)	0·0 (0·0 to 0·0)
Andorra	2043	2934·6 (2573·8 to 3461·7)	1956·0 (1603·7 to 2459·9)	283·0 (266·2 to 303·2)	695·6 (586·8 to 834·8)	0·0 (0·0 to 0·0)	3126·3 (2741·9 to 3687·9)	2083·8 (1708·5 to 2620·6)	301·5 (283·6 to 323·0)	741·0 (625·1 to 889·4)	0·0 (0·0 to 0·0)
Andorra	2044	2926·9 (2551·8 to 3472·7)	1946·9 (1584·8 to 2460·0)	279·0 (262·3 to 298·9)	700·9 (588·8 to 846·3)	0·0 (0·0 to 0·0)	3118·1 (2718·6 to 3699·5)	2074·1 (1688·4 to 2620·7)	297·3 (279·4 to 318·4)	746·7 (627·3 to 901·6)	0·0 (0·0 to 0·0)
Andorra	2045	2917·1 (2538·8 to 3459·5)	1935·8 (1581·0 to 2460·1)	275·0 (258·4 to 294·8)	706·2 (589·0 to 856·2)	0·0 (0·0 to 0·0)	3107·6 (2704·7 to 3685·6)	2062·3 (1684·3 to 2620·9)	293·0 (275·3 to 314·1)	752·3 (627·5 to 912·1)	0·0 (0·0 to 0·0)
Andorra	2046	2915·7 (2529·4 to 3481·9)	1930·2 (1569·8 to 2479·9)	271·9 (255·5 to 291·5)	713·6 (588·9 to 872·1)	0·0 (0·0 to 0·0)	3106·2 (2694·6 to 3709·4)	2056·3 (1672·3 to 2642·0)	289·7 (272·2 to 310·6)	760·2 (627·4 to 929·0)	0·0 (0·0 to 0·0)
Andorra	2047	2918·7 (2521·7 to 3505·8)	1926·9 (1555·3 to 2487·6)	269·3 (253·0 to 288·9)	722·5 (592·0 to 888·2)	0·0 (0·0 to 0·0)	3109·4 (2686·5 to 3734·8)	2052·8 (1656·9 to 2650·1)	286·9 (269·6 to 307·8)	769·7 (630·6 to 946·2)	0·0 (0·0 to 0·0)
Andorra	2048	2926·6 (2527·0 to 3518·2)	1926·7 (1549·5 to 2505·9)	267·2 (250·9 to 286·6)	732·7 (596·1 to 908·0)	0·0 (0·0 to 0·0)	3117·8 (2692·1 to 3748·0)	2052·6 (1650·8 to 2669·6)	284·6 (267·3 to 305·3)	780·6 (635·0 to 967·3)	0·0 (0·0 to 0·0)

Andorra	2049	2940·0 (2532·9 to 3546·2)	1930·2 (1542·5 to 2509·0)	265·5 (249·3 to 284·8)	744·4 (601·2 to 928·6)	0·0 (0·0 to 0·0)	3132·1 (2698·3 to 3777·9)	2056·3 (1643·3 to 2672·9)	282·8 (265·5 to 303·4)	793·0 (640·4 to 989·2)	0·0 (0·0 to 0·0)
Andorra	2050	2956·7 (2537·7 to 3587·3)	1934·6 (1549·1 to 2529·0)	264·4 (248·2 to 283·7)	757·7 (607·7 to 950·0)	0·0 (0·0 to 0·0)	3149·9 (2703·6 to 3821·7)	2061·0 (1650·4 to 2694·3)	281·6 (264·4 to 302·2)	807·2 (647·4 to 1012·1)	0·0 (0·0 to 0·0)
United Arab Emirates	1995	1389·2 (1316·5 to 1462·1)	1052·1 (994·4 to 1115·1)	231·3 (199·7 to 269·3)	105·8 (87·8 to 126·3)	0·0 (0·0 to 0·0)	2542·1 (2408·9 to 2675·5)	1925·2 (1819·6 to 2040·4)	423·2 (365·5 to 492·9)	193·7 (160·6 to 231·2)	0·0 (0·0 to 0·0)
United Arab Emirates	1996	1455·3 (1392·6 to 1518·3)	1099·7 (1051·6 to 1149·6)	244·6 (213·4 to 282·4)	110·9 (95·3 to 128·2)	0·0 (0·0 to 0·0)	2662·9 (2548·2 to 2778·3)	2012·3 (1924·2 to 2103·6)	447·6 (390·6 to 516·8)	203·0 (174·3 to 234·5)	0·0 (0·0 to 0·0)
United Arab Emirates	1997	1416·3 (1355·6 to 1478·2)	1036·4 (991·7 to 1085·4)	265·3 (235·1 to 298·5)	114·5 (100·6 to 129·1)	0·0 (0·0 to 0·0)	2591·6 (2480·5 to 2704·9)	1896·5 (1814·6 to 1986·0)	485·5 (430·1 to 546·2)	209·6 (184·2 to 236·3)	0·0 (0·0 to 0·0)
United Arab Emirates	1998	1488·3 (1430·3 to 1548·5)	1053·2 (1006·8 to 1101·4)	307·1 (275·5 to 339·7)	127·9 (114·6 to 141·3)	0·0 (0·0 to 0·0)	2723·3 (2617·3 to 2833·5)	1927·3 (1842·3 to 2015·5)	561·9 (504·1 to 621·6)	234·1 (209·7 to 258·6)	0·0 (0·0 to 0·0)
United Arab Emirates	1999	1429·5 (1374·3 to 1484·8)	989·6 (946·0 to 1033·2)	314·7 (284·0 to 346·7)	125·2 (113·5 to 137·2)	0·0 (0·0 to 0·0)	2615·7 (2514·8 to 2717·0)	1810·7 (1731·1 to 1890·7)	575·8 (519·8 to 634·4)	229·2 (207·6 to 251·0)	0·0 (0·0 to 0·0)
United Arab Emirates	2000	1410·9 (1357·5 to 1466·1)	969·8 (927·9 to 1011·5)	317·7 (288·9 to 348·5)	123·3 (113·5 to 134·6)	0·0 (0·0 to 0·0)	2581·7 (2484·0 to 2682·7)	1774·7 (1697·8 to 1850·9)	581·3 (528·6 to 637·7)	225·7 (207·6 to 246·3)	0·0 (0·0 to 0·0)
United Arab Emirates	2001	1493·1 (1439·6 to 1550·5)	1032·6 (989·6 to 1077·9)	330·6 (300·6 to 363·3)	129·9 (119·9 to 141·3)	0·0 (0·0 to 0·0)	2732·1 (2634·3 to 2837·1)	1889·4 (1810·9 to 1972·4)	604·9 (550·1 to 664·8)	237·8 (219·4 to 258·6)	0·0 (0·0 to 0·0)
United Arab Emirates	2002	1417·5 (1361·6 to 1473·8)	901·6 (861·7 to 942·6)	369·3 (338·0 to 404·3)	146·6 (137·4 to 156·7)	0·0 (0·0 to 0·0)	2593·8 (2491·6 to 2696·8)	1649·8 (1576·8 to 1724·8)	675·9 (618·4 to 739·8)	268·2 (251·5 to 286·8)	0·0 (0·0 to 0·0)
United Arab Emirates	2003	1446·7 (1389·7 to 1502·0)	878·4 (837·9 to 920·6)	403·3 (369·7 to 439·2)	165·0 (155·0 to 175·6)	0·0 (0·0 to 0·0)	2647·2 (2542·9 to 2748·5)	1607·4 (1533·3 to 1684·6)	737·9 (676·5 to 803·7)	301·9 (283·6 to 321·3)	0·0 (0·0 to 0·0)
United Arab Emirates	2004	1416·6 (1363·0 to 1469·5)	823·7 (785·0 to 863·8)	417·8 (381·9 to 454·5)	175·2 (164·9 to 186·5)	0·0 (0·0 to 0·0)	2592·2 (2494·0 to 2689·0)	1507·2 (1436·4 to 1580·5)	764·4 (698·8 to 831·6)	320·6 (301·8 to 341·2)	0·0 (0·0 to 0·0)
United Arab Emirates	2005	1357·4 (1307·4 to 1410·9)	772·2 (735·3 to 808·0)	408·0 (372·2 to 443·2)	177·2 (166·3 to 187·1)	0·0 (0·0 to 0·0)	2483·8 (2392·4 to 2581·7)	1413·0 (1345·5 to 1478·5)	746·5 (681·0 to 810·9)	324·3 (304·4 to 342·3)	0·0 (0·0 to 0·0)
United Arab Emirates	2006	1307·3 (1255·6 to 1362·5)	754·1 (714·9 to 792·7)	383·2 (348·6 to 416·9)	170·0 (159·0 to 180·6)	0·0 (0·0 to 0·0)	2392·2 (2297·5 to 2493·2)	1379·9 (1308·1 to 1450·5)	701·2 (637·8 to 763·0)	311·1 (290·9 to 330·5)	0·0 (0·0 to 0·0)

United Arab Emirates	2007	1226·9 (1176·7 to 1280·9)	738·5 (702·2 to 778·3)	337·7 (306·4 to 369·1)	150·7 (140·7 to 160·9)	0·0 (0·0 to 0·0)	2245·0 (2153·1 to 2343·9)	1351·3 (1285·0 to 1424·2)	618·0 (560·8 to 675·5)	275·7 (257·4 to 294·4)	0·0 (0·0 to 0·0)
United Arab Emirates	2008	1181·9 (1133·8 to 1233·1)	762·7 (727·6 to 803·4)	287·7 (258·9 to 315·8)	131·4 (122·9 to 141·6)	0·0 (0·0 to 0·0)	2162·6 (2074·7 to 2256·4)	1395·7 (1331·4 to 1470·0)	526·5 (473·8 to 577·9)	240·5 (224·8 to 259·1)	0·0 (0·0 to 0·0)
United Arab Emirates	2009	1322·0 (1267·6 to 1377·5)	927·6 (883·4 to 973·3)	258·4 (231·0 to 287·3)	136·0 (127·5 to 146·4)	0·0 (0·0 to 0·0)	2419·0 (2319·5 to 2520·6)	1697·3 (1616·5 to 1781·0)	472·8 (422·7 to 525·8)	248·9 (233·3 to 268·0)	0·0 (0·0 to 0·0)
United Arab Emirates	2010	1248·5 (1199·2 to 1299·9)	883·5 (842·8 to 923·1)	230·9 (205·7 to 259·4)	134·2 (125·2 to 144·1)	0·0 (0·0 to 0·0)	2284·6 (2194·4 to 2378·6)	1616·6 (1542·3 to 1689·2)	422·5 (376·4 to 474·7)	245·5 (229·1 to 263·7)	0·0 (0·0 to 0·0)
United Arab Emirates	2011	1254·8 (1205·9 to 1303·6)	901·2 (858·7 to 941·9)	219·4 (195·3 to 246·7)	134·2 (125·2 to 144·5)	0·0 (0·0 to 0·0)	2296·0 (2206·5 to 2385·4)	1649·1 (1571·3 to 1723·5)	401·4 (357·4 to 451·4)	245·5 (229·1 to 264·4)	0·0 (0·0 to 0·0)
United Arab Emirates	2012	1215·6 (1168·7 to 1264·1)	872·3 (833·6 to 914·5)	211·5 (186·8 to 238·1)	131·7 (123·2 to 140·9)	0·0 (0·0 to 0·0)	2224·3 (2138·5 to 2313·2)	1596·2 (1525·3 to 1673·4)	387·0 (341·8 to 435·7)	241·1 (225·4 to 257·8)	0·0 (0·0 to 0·0)
United Arab Emirates	2013	1267·5 (1215·4 to 1319·2)	909·6 (868·5 to 954·3)	218·6 (192·4 to 246·2)	139·3 (130·5 to 149·4)	0·0 (0·0 to 0·0)	2319·4 (2224·0 to 2413·9)	1664·4 (1589·2 to 1746·3)	400·1 (352·1 to 450·6)	254·9 (238·8 to 273·3)	0·0 (0·0 to 0·0)
United Arab Emirates	2014	1351·2 (1299·1 to 1405·0)	972·6 (930·3 to 1016·5)	230·8 (201·8 to 258·6)	147·8 (138·2 to 158·5)	0·0 (0·0 to 0·0)	2472·5 (2377·2 to 2570·9)	1779·8 (1702·2 to 1860·0)	422·3 (369·3 to 473·2)	270·5 (252·8 to 290·1)	0·0 (0·0 to 0·0)
United Arab Emirates	2015	1342·4 (1291·6 to 1392·3)	963·1 (921·5 to 1005·2)	234·0 (205·3 to 262·5)	145·3 (136·1 to 154·6)	0·0 (0·0 to 0·0)	2456·3 (2363·5 to 2547·8)	1762·2 (1686·3 to 1839·4)	428·1 (375·7 to 480·4)	266·0 (249·0 to 282·8)	0·0 (0·0 to 0·0)
United Arab Emirates	2016	1297·4 (1250·0 to 1352·2)	932·4 (893·2 to 973·8)	230·2 (202·3 to 260·4)	134·9 (128·1 to 141·9)	0·0 (0·0 to 0·0)	2374·1 (2287·3 to 2474·3)	1706·1 (1634·3 to 1781·9)	421·2 (370·2 to 476·6)	246·9 (234·4 to 259·7)	0·0 (0·0 to 0·0)
United Arab Emirates	2017	1667·8 (1610·8 to 1723·2)	903·7 (859·1 to 949·0)	218·5 (190·1 to 249·5)	545·6 (530·5 to 560·7)	0·0 (0·0 to 0·0)	3051·9 (2947·4 to 3153·1)	1653·7 (1572·1 to 1736·6)	399·8 (347·9 to 456·6)	998·4 (970·7 to 1026·0)	0·0 (0·0 to 0·0)
United Arab Emirates	2018	1688·6 (1618·2 to 1754·5)	881·3 (824·5 to 937·6)	217·8 (187·0 to 252·5)	589·5 (571·6 to 607·0)	0·0 (0·0 to 0·0)	3089·9 (2961·0 to 3210·4)	1612·6 (1508·8 to 1715·6)	398·5 (342·2 to 462·1)	1078·7 (1046·0 to 1110·7)	0·0 (0·0 to 0·0)
United Arab Emirates	2019	1750·6 (1675·1 to 1824·2)	933·8 (868·7 to 1000·3)	216·7 (186·0 to 251·2)	600·1 (581·9 to 618·2)	0·0 (0·0 to 0·0)	3203·3 (3065·1 to 3338·0)	1708·8 (1589·6 to 1830·3)	396·5 (340·3 to 459·7)	1098·0 (1064·9 to 1131·3)	0·0 (0·0 to 0·0)
United Arab Emirates	2020	1740·9 (1660·6 to 1818·9)	990·2 (918·3 to 1061·5)	196·0 (167·9 to 227·1)	554·7 (537·0 to 573·4)	0·0 (0·0 to 0·0)	3185·6 (3038·6 to 3328·4)	1812·0 (1680·3 to 1942·4)	358·6 (307·3 to 415·5)	1015·0 (982·7 to 1049·3)	0·0 (0·0 to 0·0)

United Arab Emirates	2021	1606·9 (1532·8 to 1679·8)	908·1 (842·1 to 973·4)	179·5 (153·7 to 208·1)	519·3 (500·3 to 539·3)	0·0 (0·0 to 0·0)	2940·5 (2804·9 to 3073·7)	1661·7 (1541·0 to 1781·3)	328·5 (281·2 to 380·9)	950·3 (915·5 to 986·9)	0·0 (0·0 to 0·0)
United Arab Emirates	2022	1489·0 (1421·0 to 1556·7)	782·6 (725·5 to 838·8)	178·6 (152·9 to 207·2)	527·8 (505·5 to 550·8)	0·0 (0·0 to 0·0)	2724·7 (2600·2 to 2848·5)	1432·1 (1327·6 to 1534·9)	326·8 (279·9 to 379·1)	965·8 (925·0 to 1007·8)	0·0 (0·0 to 0·0)
United Arab Emirates	2023	1496·3 (1424·9 to 1567·6)	782·4 (723·3 to 840·8)	177·6 (151·9 to 206·0)	536·3 (509·9 to 564·2)	0·0 (0·0 to 0·0)	2737·9 (2607·4 to 2868·5)	1431·6 (1323·6 to 1538·5)	324·9 (277·9 to 377·0)	981·3 (933·0 to 1032·4)	0·0 (0·0 to 0·0)
United Arab Emirates	2024	1504·0 (1430·5 to 1578·1)	782·3 (721·1 to 842·1)	176·6 (150·9 to 204·9)	545·1 (514·4 to 576·4)	0·0 (0·0 to 0·0)	2752·1 (2617·6 to 2887·8)	1431·5 (1319·6 to 1541·0)	323·1 (276·0 to 375·0)	997·4 (941·2 to 1054·8)	0·0 (0·0 to 0·0)
United Arab Emirates	2025	1512·5 (1435·9 to 1590·4)	782·5 (719·3 to 843·5)	175·6 (150·1 to 204·0)	554·4 (519·2 to 589·3)	0·0 (0·0 to 0·0)	2767·7 (2627·6 to 2910·1)	1431·9 (1316·2 to 1543·5)	321·3 (274·7 to 373·3)	1014·5 (950·1 to 1078·4)	0·0 (0·0 to 0·0)
United Arab Emirates	2026	1523·1 (1441·5 to 1605·2)	783·1 (716·8 to 846·4)	175·9 (150·3 to 204·1)	564·1 (523·8 to 604·3)	0·0 (0·0 to 0·0)	2787·1 (2637·7 to 2937·3)	1433·0 (1311·6 to 1548·8)	321·9 (275·0 to 373·5)	1032·3 (958·6 to 1105·9)	0·0 (0·0 to 0·0)
United Arab Emirates	2027	1533·1 (1446·8 to 1619·1)	783·1 (714·5 to 850·0)	176·4 (150·5 to 204·4)	573·6 (529·5 to 619·1)	0·0 (0·0 to 0·0)	2805·3 (2647·4 to 2962·6)	1433·0 (1307·5 to 1555·4)	322·7 (275·4 to 374·0)	1049·6 (968·9 to 1132·9)	0·0 (0·0 to 0·0)
United Arab Emirates	2028	1543·7 (1451·6 to 1635·0)	783·3 (712·1 to 852·3)	177·1 (151·1 to 205·7)	583·3 (534·3 to 634·8)	0·0 (0·0 to 0·0)	2824·8 (2656·2 to 2991·8)	1433·3 (1303·1 to 1559·5)	324·1 (276·6 to 376·3)	1067·4 (977·7 to 1161·5)	0·0 (0·0 to 0·0)
United Arab Emirates	2029	1554·4 (1458·0 to 1651·7)	783·4 (709·3 to 855·8)	178·1 (151·9 to 206·5)	593·0 (539·4 to 650·2)	0·0 (0·0 to 0·0)	2844·4 (2667·9 to 3022·4)	1433·4 (1297·8 to 1566·0)	325·9 (278·0 to 377·9)	1085·1 (987·0 to 1189·8)	0·0 (0·0 to 0·0)
United Arab Emirates	2030	1564·3 (1462·5 to 1667·7)	782·9 (705·3 to 858·7)	178·9 (152·8 to 207·3)	602·5 (543·1 to 665·8)	0·0 (0·0 to 0·0)	2862·4 (2676·1 to 3051·7)	1432·6 (1290·6 to 1571·3)	327·4 (279·6 to 379·2)	1102·4 (993·8 to 1218·4)	0·0 (0·0 to 0·0)
United Arab Emirates	2031	1576·1 (1469·7 to 1686·0)	781·9 (701·8 to 859·7)	179·2 (153·6 to 208·0)	615·0 (552·3 to 684·3)	0·0 (0·0 to 0·0)	2884·1 (2689·3 to 3085·2)	1430·8 (1284·3 to 1573·1)	328·0 (281·1 to 380·6)	1125·3 (1010·6 to 1252·2)	0·0 (0·0 to 0·0)
United Arab Emirates	2032	1599·5 (1484·5 to 1720·7)	780·0 (695·9 to 861·2)	178·5 (152·3 to 206·6)	641·0 (567·6 to 721·9)	0·0 (0·0 to 0·0)	2926·9 (2716·4 to 3148·7)	1427·3 (1273·4 to 1575·8)	326·6 (278·7 to 378·0)	1172·9 (1038·6 to 1320·9)	0·0 (0·0 to 0·0)
United Arab Emirates	2033	1620·8 (1494·4 to 1760·2)	776·4 (691·2 to 863·0)	177·4 (151·7 to 205·0)	667·0 (571·1 to 772·4)	0·0 (0·0 to 0·0)	2965·8 (2734·6 to 3220·9)	1420·8 (1264·8 to 1579·1)	324·6 (277·6 to 375·0)	1220·4 (1045·1 to 1413·3)	0·0 (0·0 to 0·0)
United Arab Emirates	2034	1640·8 (1489·3 to 1806·8)	771·3 (685·6 to 862·2)	176·0 (149·9 to 203·6)	693·6 (572·2 to 830·7)	0·0 (0·0 to 0·0)	3002·5 (2725·2 to 3306·3)	1411·3 (1254·6 to 1577·7)	322·1 (274·2 to 372·6)	1269·1 (1047·0 to 1520·0)	0·0 (0·0 to 0·0)

United Arab Emirates	2035	1657·9 (1482·2 to 1867·6)	764·1 (677·3 to 857·3)	174·1 (148·3 to 201·8)	719·7 (569·7 to 897·3)	0·0 (0·0 to 0·0)	3033·8 (2712·1 to 3417·4)	1398·2 (1239·4 to 1568·7)	318·7 (271·5 to 369·2)	1316·9 (1042·5 to 1641·9)	0·0 (0·0 to 0·0)
United Arab Emirates	2036	1675·1 (1470·8 to 1920·9)	756·5 (668·9 to 851·6)	172·2 (146·6 to 199·8)	746·3 (569·7 to 969·8)	0·0 (0·0 to 0·0)	3065·1 (2691·3 to 3515·1)	1384·4 (1223·9 to 1558·3)	315·1 (268·3 to 365·6)	1365·7 (1042·4 to 1774·6)	0·0 (0·0 to 0·0)
United Arab Emirates	2037	1687·0 (1454·5 to 1975·8)	746·6 (658·5 to 844·0)	169·6 (143·6 to 197·3)	770·8 (564·6 to 1047·4)	0·0 (0·0 to 0·0)	3086·9 (2661·6 to 3615·4)	1366·2 (1204·9 to 1544·4)	310·4 (262·8 to 361·1)	1410·4 (1033·1 to 1916·6)	0·0 (0·0 to 0·0)
United Arab Emirates	2038	1693·9 (1433·8 to 2045·2)	733·6 (645·1 to 832·8)	166·5 (140·2 to 194·4)	793·8 (557·9 to 1127·9)	0·0 (0·0 to 0·0)	3099·6 (2623·7 to 3742·4)	1342·5 (1180·4 to 1523·9)	304·6 (256·6 to 355·8)	1452·5 (1020·8 to 2063·9)	0·0 (0·0 to 0·0)
United Arab Emirates	2039	1698·9 (1409·8 to 2108·5)	719·2 (631·6 to 818·8)	163·0 (136·5 to 191·2)	816·7 (548·3 to 1204·7)	0·0 (0·0 to 0·0)	3108·7 (2579·8 to 3858·2)	1316·0 (1155·7 to 1498·3)	298·2 (249·7 to 349·9)	1494·5 (1003·3 to 2204·5)	0·0 (0·0 to 0·0)
United Arab Emirates	2040	1697·2 (1378·3 to 2167·6)	701·5 (614·4 to 800·9)	158·8 (132·4 to 187·7)	836·9 (534·5 to 1277·5)	0·0 (0·0 to 0·0)	3105·6 (2522·0 to 3966·4)	1283·7 (1124·2 to 1465·5)	290·5 (242·3 to 343·4)	1531·4 (978·1 to 2337·6)	0·0 (0·0 to 0·0)
United Arab Emirates	2041	1689·7 (1341·6 to 2234·0)	681·6 (595·2 to 780·1)	154·1 (128·0 to 183·1)	854·1 (519·1 to 1359·8)	0·0 (0·0 to 0·0)	3091·9 (2454·8 to 4088·0)	1247·1 (1089·1 to 1427·4)	281·9 (234·3 to 335·1)	1562·8 (949·9 to 2488·1)	0·0 (0·0 to 0·0)
United Arab Emirates	2042	1679·0 (1297·4 to 2291·0)	660·7 (575·7 to 757·7)	149·1 (123·3 to 178·0)	869·2 (503·1 to 1434·8)	0·0 (0·0 to 0·0)	3072·3 (2374·1 to 4192·2)	1209·0 (1053·5 to 1386·5)	272·8 (225·6 to 325·7)	1590·5 (920·7 to 2625·4)	0·0 (0·0 to 0·0)
United Arab Emirates	2043	1658·9 (1249·4 to 2347·1)	635·6 (553·3 to 729·9)	143·3 (117·8 to 172·0)	880·1 (486·0 to 1509·7)	0·0 (0·0 to 0·0)	3035·6 (2286·2 to 4294·8)	1163·0 (1012·4 to 1335·5)	262·1 (215·5 to 314·7)	1610·5 (889·3 to 2762·6)	0·0 (0·0 to 0·0)
United Arab Emirates	2044	1629·7 (1193·9 to 2382·9)	606·3 (526·9 to 697·0)	136·6 (112·3 to 165·2)	886·9 (464·4 to 1588·3)	0·0 (0·0 to 0·0)	2982·2 (2184·7 to 4360·4)	1109·4 (964·2 to 1275·5)	249·9 (205·5 to 302·2)	1622·9 (849·7 to 2906·3)	0·0 (0·0 to 0·0)
United Arab Emirates	2045	1591·8 (1131·6 to 2417·6)	574·5 (498·5 to 661·2)	129·3 (105·7 to 157·1)	888·0 (438·5 to 1657·2)	0·0 (0·0 to 0·0)	2912·8 (2070·7 to 4423·9)	1051·3 (912·2 to 1209·9)	236·6 (193·4 to 287·4)	1624·9 (802·4 to 3032·4)	0·0 (0·0 to 0·0)
United Arab Emirates	2046	1547·9 (1065·9 to 2425·8)	542·3 (469·9 to 624·7)	121·9 (99·1 to 149·0)	883·7 (414·2 to 1711·9)	0·0 (0·0 to 0·0)	2832·5 (1950·4 to 4438·9)	992·4 (859·9 to 1143·1)	223·1 (181·4 to 272·7)	1617·0 (758·0 to 3132·5)	0·0 (0·0 to 0·0)
United Arab Emirates	2047	1503·6 (1010·8 to 2420·1)	512·6 (443·7 to 591·0)	115·0 (93·1 to 141·5)	876·0 (392·3 to 1751·8)	0·0 (0·0 to 0·0)	2751·4 (1849·7 to 4428·5)	938·0 (811·9 to 1081·5)	210·4 (170·3 to 258·9)	1602·9 (717·8 to 3205·5)	0·0 (0·0 to 0·0)
United Arab Emirates	2048	1457·3 (953·9 to 2407·2)	483·8 (418·4 to 558·7)	108·3 (87·2 to 134·0)	865·2 (370·4 to 1781·1)	0·0 (0·0 to 0·0)	2666·6 (1745·5 to 4404·8)	885·3 (765·6 to 1022·4)	198·1 (159·6 to 245·2)	1583·2 (677·8 to 3259·2)	0·0 (0·0 to 0·0)

United Arab Emirates	2049	1406·3 (895·3 to 2386·8)	454·4 (392·7 to 525·5)	101·4 (81·4 to 126·2)	850·5 (348·0 to 1801·1)	0·0 (0·0 to 0·0)	2573·3 (1638·3 to 4367·6)	831·5 (718·7 to 961·6)	185·6 (148·9 to 230·9)	1556·2 (636·8 to 3295·7)	0·0 (0·0 to 0·0)
United Arab Emirates	2050	1353·0 (838·7 to 2356·5)	425·9 (367·9 to 492·4)	94·8 (75·8 to 118·6)	832·3 (326·7 to 1807·9)	0·0 (0·0 to 0·0)	2475·8 (1534·7 to 4312·1)	779·3 (673·2 to 901·0)	173·5 (138·6 to 217·0)	1522·9 (597·9 to 3308·1)	0·0 (0·0 to 0·0)
Argentina*	1995	724·7 (680·6 to 767·0)	433·0 (399·5 to 465·3)	193·6 (175·4 to 214·0)	92·3 (74·4 to 112·4)	5·8 (5·8 to 5·8)	1750·4 (1643·9 to 1852·6)	1045·9 (965·1 to 1124·0)	467·7 (423·8 to 516·9)	222·8 (179·7 to 271·4)	14·1 (14·1 to 14·1)
Argentina*	1996	744·3 (709·7 to 781·6)	449·4 (421·9 to 475·4)	189·3 (173·9 to 205·4)	97·1 (80·1 to 116·2)	8·5 (8·5 to 8·5)	1797·8 (1714·4 to 1888·0)	1085·4 (1019·0 to 1148·4)	457·2 (420·0 to 496·2)	234·6 (193·6 to 280·8)	20·5 (20·5 to 20·5)
Argentina*	1997	751·5 (718·2 to 788·0)	454·1 (430·6 to 479·6)	183·7 (168·8 to 198·3)	104·8 (87·8 to 124·6)	8·9 (8·9 to 8·9)	1815·3 (1734·8 to 1903·5)	1096·8 (1040·0 to 1158·4)	443·7 (407·7 to 479·1)	253·3 (212·1 to 301·1)	21·6 (21·6 to 21·6)
Argentina*	1998	752·0 (719·2 to 786·4)	452·4 (429·0 to 475·7)	181·9 (168·7 to 195·0)	109·0 (92·4 to 128·0)	8·7 (8·7 to 8·7)	1816·5 (1737·1 to 1899·6)	1092·7 (1036·3 to 1149·0)	439·3 (407·5 to 471·0)	263·4 (223·2 to 309·2)	21·0 (21·0 to 21·0)
Argentina*	1999	732·2 (702·5 to 763·7)	434·7 (412·6 to 458·0)	183·8 (171·7 to 195·9)	109·7 (93·3 to 127·8)	4·0 (4·0 to 4·0)	1768·6 (1697·0 to 1844·8)	1050·1 (996·5 to 1106·4)	443·9 (414·8 to 473·1)	265·1 (225·5 to 308·7)	9·6 (9·6 to 9·6)
Argentina*	2000	683·8 (653·8 to 715·0)	389·1 (369·0 to 410·8)	187·4 (176·2 to 199·6)	104·9 (88·7 to 121·6)	2·3 (2·3 to 2·3)	1651·7 (1579·4 to 1727·1)	939·9 (891·3 to 992·3)	452·7 (425·7 to 482·1)	253·4 (214·2 to 293·8)	5·6 (5·6 to 5·6)
Argentina*	2001	646·6 (615·9 to 680·1)	358·4 (336·9 to 379·8)	184·4 (171·2 to 198·3)	97·4 (81·4 to 115·9)	6·3 (6·3 to 6·3)	1561·7 (1487·6 to 1642·8)	865·7 (813·7 to 917·5)	445·5 (413·5 to 478·9)	235·3 (196·7 to 279·9)	15·3 (15·3 to 15·3)
Argentina*	2002	519·9 (494·9 to 546·5)	278·0 (260·0 to 296·4)	161·6 (148·1 to 174·6)	78·0 (65·1 to 93·6)	2·4 (2·4 to 2·4)	1255·8 (1195·5 to 1320·1)	671·4 (628·0 to 715·9)	390·3 (357·7 to 421·8)	188·4 (157·2 to 226·0)	5·7 (5·7 to 5·7)
Argentina*	2003	546·5 (520·5 to 575·8)	280·8 (262·1 to 301·1)	162·3 (148·7 to 175·9)	81·7 (68·2 to 97·3)	21·6 (21·6 to 21·6)	1320·0 (1257·4 to 1390·8)	678·3 (633·1 to 727·3)	392·1 (359·1 to 425·0)	197·4 (164·8 to 234·9)	52·3 (52·3 to 52·3)
Argentina*	2004	563·7 (535·9 to 592·8)	297·0 (277·7 to 318·5)	175·6 (161·8 to 190·5)	84·9 (71·0 to 100·8)	6·2 (6·2 to 6·2)	1361·7 (1294·4 to 1431·9)	717·4 (670·9 to 769·4)	424·2 (390·9 to 460·2)	205·2 (171·6 to 243·5)	14·9 (14·9 to 14·9)
Argentina*	2005	618·3 (588·3 to 648·3)	324·7 (303·4 to 346·6)	199·0 (184·3 to 214·6)	92·3 (77·9 to 107·7)	2·3 (2·3 to 2·3)	1493·5 (1421·1 to 1566·0)	784·3 (732·9 to 837·2)	480·6 (445·1 to 518·4)	223·0 (188·2 to 260·2)	5·6 (5·6 to 5·6)
Argentina*	2006	686·8 (655·6 to 717·9)	364·4 (342·1 to 387·9)	219·3 (204·5 to 235·1)	101·0 (86·0 to 117·6)	2·1 (2·1 to 2·1)	1658·9 (1583·6 to 1734·0)	880·1 (826·3 to 936·8)	529·7 (493·9 to 567·9)	244·1 (207·8 to 284·1)	5·0 (5·0 to 5·0)

Argentina*	2007	781·6 (748·8 to 813·7)	424·2 (399·5 to 448·6)	238·4 (222·9 to 255·6)	110·8 (95·8 to 128·4)	8·3 (8·3 to 8·3)	1888·0 (1808·8 to 1965·4)	1024·6 (965·0 to 1083·7)	575·9 (538·5 to 617·4)	267·6 (231·4 to 310·2)	20·0 (20·0 to 20·0)
Argentina*	2008	839·1 (806·5 to 873·7)	470·6 (445·7 to 496·0)	247·0 (230·5 to 263·2)	117·0 (101·8 to 134·6)	4·6 (4·6 to 4·6)	2026·9 (1948·1 to 2110·3)	1136·6 (1076·6 to 1198·1)	596·5 (556·7 to 635·7)	282·7 (246·0 to 325·1)	11·0 (11·0 to 11·0)
Argentina*	2009	890·2 (858·1 to 925·9)	515·8 (489·9 to 541·7)	247·7 (231·1 to 264·3)	122·5 (106·5 to 140·5)	4·2 (4·2 to 4·2)	2150·2 (2072·7 to 2236·4)	1245·9 (1183·4 to 1308·5)	598·2 (558·2 to 638·4)	295·8 (257·3 to 339·3)	10·3 (10·3 to 10·3)
Argentina*	2010	948·3 (915·6 to 984·1)	560·4 (535·9 to 586·9)	256·6 (240·9 to 273·3)	126·8 (110·4 to 145·4)	4·5 (4·5 to 4·5)	2290·5 (2211·5 to 2377·2)	1353·6 (1294·5 to 1417·7)	619·8 (581·8 to 660·3)	306·2 (266·7 to 351·3)	10·9 (10·9 to 10·9)
Argentina*	2011	1012·6 (978·7 to 1048·8)	615·2 (590·1 to 642·9)	267·5 (251·6 to 285·6)	122·0 (107·0 to 140·0)	7·9 (7·9 to 7·9)	2445·9 (2364·0 to 2533·4)	1486·1 (1425·3 to 1552·9)	646·1 (607·7 to 689·7)	294·7 (258·4 to 338·2)	19·0 (19·0 to 19·0)
Argentina*	2012	1027·9 (992·5 to 1063·1)	641·1 (614·8 to 669·5)	270·2 (254·3 to 286·4)	110·0 (95·0 to 126·1)	6·5 (6·5 to 6·5)	2482·8 (2397·3 to 2567·8)	1548·6 (1485·1 to 1617·1)	652·8 (614·3 to 691·7)	265·7 (229·5 to 304·5)	15·7 (15·7 to 15·7)
Argentina*	2013	1036·6 (1004·7 to 1071·1)	660·7 (637·2 to 687·7)	273·4 (258·9 to 287·8)	95·4 (81·3 to 111·1)	7·1 (7·1 to 7·1)	2503·9 (2426·7 to 2587·1)	1596·0 (1539·2 to 1661·0)	660·3 (625·4 to 695·2)	230·4 (196·4 to 268·3)	17·2 (17·2 to 17·2)
Argentina*	2014	1007·1 (976·3 to 1039·0)	655·1 (631·1 to 680·7)	270·8 (258·6 to 283·7)	81·3 (67·9 to 95·5)	0·0 (0·0 to 0·0)	2432·7 (2358·2 to 2509·7)	1582·3 (1524·5 to 1644·2)	654·1 (624·8 to 685·2)	196·3 (164·0 to 230·6)	0·0 (0·0 to 0·0)
Argentina*	2015	1038·6 (1004·2 to 1073·3)	676·7 (648·4 to 704·0)	275·1 (261·2 to 288·7)	82·1 (68·9 to 96·8)	4·6 (4·6 to 4·6)	2508·7 (2425·7 to 2592·4)	1634·6 (1566·1 to 1700·6)	664·5 (631·0 to 697·2)	198·4 (166·5 to 233·7)	11·2 (11·2 to 11·2)
Argentina*	2016	999·1 (966·2 to 1030·0)	636·2 (611·2 to 663·2)	270·9 (258·0 to 284·1)	84·8 (72·1 to 99·1)	7·3 (7·3 to 7·3)	2413·3 (2333·9 to 2487·9)	1536·6 (1476·4 to 1601·8)	654·3 (623·3 to 686·2)	204·8 (174·2 to 239·3)	17·6 (17·6 to 17·6)
Argentina*	2017	1025·0 (991·7 to 1057·8)	647·0 (621·5 to 674·7)	275·1 (262·6 to 288·1)	102·8 (87·2 to 119·3)	0·0 (0·0 to 0·0)	2475·8 (2395·5 to 2555·0)	1562·8 (1501·3 to 1629·8)	664·6 (634·4 to 695·8)	248·4 (210·6 to 288·2)	0·0 (0·0 to 0·0)
Argentina*	2018	989·2 (946·4 to 1033·3)	608·3 (577·1 to 641·4)	269·0 (252·6 to 287·2)	104·6 (86·8 to 124·3)	7·3 (7·3 to 7·3)	2389·3 (2286·0 to 2496·0)	1469·4 (1394·0 to 1549·2)	649·7 (610·1 to 693·7)	252·6 (209·7 to 300·1)	17·6 (17·6 to 17·6)
Argentina*	2019	946·1 (904·9 to 988·3)	573·0 (543·2 to 604·3)	260·6 (244·8 to 278·4)	103·9 (86·3 to 123·5)	8·6 (8·5 to 8·8)	2285·4 (2185·8 to 2387·2)	1384·0 (1312·1 to 1459·8)	629·5 (591·4 to 672·5)	251·1 (208·5 to 298·3)	20·8 (20·5 to 21·3)
Argentina*	2020	932·2 (893·5 to 973·2)	589·7 (560·2 to 620·9)	232·9 (218·9 to 249·0)	95·3 (78·7 to 113·4)	14·3 (13·6 to 15·4)	2251·8 (2158·3 to 2350·8)	1424·4 (1353·1 to 1499·7)	562·7 (528·8 to 601·4)	230·3 (190·2 to 273·9)	34·5 (32·9 to 37·2)

Argentina*	2021	870·5 (832·3 to 910·8)	547·1 (518·4 to 577·8)	216·8 (203·8 to 231·7)	90·9 (74·7 to 108·1)	15·8 (14·9 to 17·2)	2102·8 (2010·4 to 2200·0)	1321·4 (1252·1 to 1395·8)	523·7 (492·2 to 559·6)	219·5 (180·4 to 261·2)	38·1 (35·9 to 41·6)
Argentina*	2022	807·2 (771·1 to 846·1)	484·1 (456·4 to 513·0)	217·5 (204·2 to 232·4)	93·2 (76·6 to 111·1)	12·4 (11·3 to 13·9)	1949·7 (1862·7 to 2043·8)	1169·2 (1102·4 to 1239·3)	525·3 (493·3 to 561·4)	225·1 (185·0 to 268·3)	30·1 (27·3 to 33·7)
Argentina*	2023	814·3 (777·3 to 852·7)	487·4 (459·4 to 516·8)	218·2 (205·0 to 233·2)	95·7 (78·8 to 114·1)	12·9 (11·7 to 14·6)	1967·0 (1877·6 to 2059·7)	1177·4 (1109·7 to 1248·2)	527·2 (495·3 to 563·2)	231·2 (190·3 to 275·6)	31·3 (28·3 to 35·3)
Argentina*	2024	825·8 (783·9 to 870·0)	494·9 (461·5 to 528·6)	219·1 (205·7 to 234·1)	98·4 (81·0 to 117·5)	13·4 (12·0 to 15·3)	1994·8 (1893·5 to 2101·4)	1195·5 (1114·7 to 1276·8)	529·3 (496·8 to 565·5)	237·6 (195·7 to 283·7)	32·4 (29·1 to 37·0)
Argentina*	2025	838·8 (791·7 to 886·9)	503·6 (464·9 to 541·3)	220·2 (206·7 to 235·2)	101·1 (83·1 to 120·5)	13·9 (12·4 to 15·9)	2026·0 (1912·3 to 2142·2)	1216·3 (1122·9 to 1307·4)	531·9 (499·2 to 568·2)	244·3 (200·7 to 291·1)	33·5 (29·9 to 38·3)
Argentina*	2026	851·8 (801·7 to 905·1)	512·5 (470·9 to 556·4)	221·2 (207·6 to 236·3)	103·8 (85·0 to 123·8)	14·3 (12·7 to 16·5)	2057·6 (1936·6 to 2186·3)	1237·9 (1137·6 to 1344·0)	534·3 (501·4 to 570·8)	250·8 (205·3 to 299·0)	34·5 (30·7 to 39·8)
Argentina*	2027	864·9 (811·1 to 923·3)	521·5 (474·1 to 570·3)	222·1 (208·5 to 237·4)	106·6 (87·3 to 127·1)	14·7 (13·0 to 17·0)	2089·2 (1959·2 to 2230·2)	1259·7 (1145·2 to 1377·6)	536·4 (503·5 to 573·3)	257·4 (211·0 to 307·0)	35·6 (31·5 to 41·2)
Argentina*	2028	878·3 (821·0 to 941·5)	530·5 (480·7 to 584·7)	223·2 (209·5 to 238·5)	109·5 (90·0 to 130·6)	15·1 (13·3 to 17·6)	2121·5 (1983·0 to 2274·1)	1281·3 (1161·2 to 1412·3)	539·2 (505·9 to 576·2)	264·5 (217·3 to 315·4)	36·5 (32·2 to 42·6)
Argentina*	2029	892·1 (831·1 to 960·1)	539·8 (486·2 to 599·8)	224·4 (210·5 to 239·8)	112·5 (92·0 to 134·2)	15·5 (13·6 to 18·3)	2154·9 (2007·6 to 2319·2)	1303·9 (1174·5 to 1448·8)	541·9 (508·5 to 579·1)	271·6 (222·1 to 324·1)	37·4 (32·7 to 44·1)
Argentina*	2030	905·8 (840·3 to 978·9)	549·0 (492·4 to 613·2)	225·5 (211·6 to 240·9)	115·5 (94·1 to 138·2)	15·9 (13·8 to 18·7)	2188·0 (2029·7 to 2364·4)	1326·1 (1189·3 to 1481·2)	544·6 (511·1 to 581·8)	279·0 (227·2 to 333·8)	38·3 (33·4 to 45·2)
Argentina*	2031	920·7 (852·4 to 998·4)	559·0 (498·2 to 629·7)	226·8 (212·9 to 242·4)	118·7 (96·8 to 142·3)	16·2 (14·1 to 19·2)	2224·0 (2059·0 to 2411·6)	1350·2 (1203·3 to 1521·0)	547·9 (514·2 to 585·4)	286·8 (233·8 to 343·8)	39·1 (34·0 to 46·4)
Argentina*	2032	935·2 (863·8 to 1016·7)	568·6 (503·3 to 641·4)	228·1 (214·1 to 243·7)	122·0 (99·2 to 146·3)	16·5 (14·3 to 19·6)	2259·0 (2086·4 to 2455·7)	1373·5 (1215·6 to 1549·3)	550·9 (517·1 to 588·5)	294·8 (239·7 to 353·3)	39·9 (34·6 to 47·3)
Argentina*	2033	950·6 (873·7 to 1037·6)	578·9 (508·9 to 655·6)	229·4 (215·3 to 245·1)	125·5 (102·0 to 150·3)	16·8 (14·6 to 20·1)	2296·2 (2110·4 to 2506·3)	1398·2 (1229·2 to 1583·7)	554·1 (520·1 to 591·9)	303·1 (246·3 to 363·2)	40·7 (35·3 to 48·5)
Argentina*	2034	967·2 (887·5 to 1057·9)	590·0 (519·6 to 670·2)	230·9 (216·7 to 246·6)	129·1 (105·2 to 154·6)	17·1 (14·8 to 20·5)	2336·2 (2143·8 to 2555·3)	1425·2 (1255·2 to 1618·8)	557·7 (523·5 to 595·6)	311·9 (254·0 to 373·4)	41·4 (35·6 to 49·4)

Argentina*	2035	983·3 (900·8 to 1076·7)	600·6 (524·1 to 682·9)	232·3 (218·1 to 248·1)	132·9 (108·2 to 160·0)	17·5 (15·0 to 20·8)	2375·1 (2175·9 to 2600·7)	1450·8 (1266·0 to 1649·6)	561·2 (526·9 to 599·2)	321·0 (261·2 to 386·6)	42·2 (36·2 to 50·3)
Argentina*	2036	999·4 (913·6 to 1095·6)	611·3 (532·8 to 695·9)	233·6 (219·4 to 249·5)	136·7 (110·7 to 165·6)	17·8 (15·3 to 21·3)	2414·0 (2206·9 to 2646·3)	1476·5 (1286·9 to 1681·0)	564·3 (529·8 to 602·6)	330·2 (267·4 to 400·0)	42·9 (37·0 to 51·4)
Argentina*	2037	1015·8 (927·5 to 1116·9)	622·3 (540·9 to 711·2)	234·9 (220·5 to 250·8)	140·6 (113·6 to 170·3)	18·1 (15·6 to 21·8)	2453·7 (2240·4 to 2697·9)	1503·2 (1306·5 to 1718·0)	567·3 (532·5 to 605·8)	339·5 (274·3 to 411·4)	43·8 (37·8 to 52·5)
Argentina*	2038	1032·8 (939·9 to 1136·2)	633·5 (549·9 to 723·4)	236·2 (221·8 to 252·3)	144·6 (116·8 to 175·6)	18·4 (15·8 to 22·2)	2494·6 (2270·3 to 2744·6)	1530·2 (1328·3 to 1747·4)	570·6 (535·7 to 609·4)	349·4 (282·2 to 424·1)	44·4 (38·2 to 53·6)
Argentina*	2039	1050·4 (952·2 to 1160·1)	645·3 (555·8 to 743·3)	237·6 (223·0 to 253·7)	148·8 (119·7 to 181·9)	18·7 (16·1 to 22·4)	2537·3 (2300·0 to 2802·2)	1558·8 (1342·6 to 1795·5)	573·8 (538·7 to 612·9)	359·5 (289·2 to 439·4)	45·2 (38·8 to 54·1)
Argentina*	2040	1067·5 (967·1 to 1184·3)	656·9 (567·4 to 757·0)	238·6 (224·1 to 254·9)	153·0 (122·5 to 188·2)	19·1 (16·2 to 23·0)	2578·6 (2336·0 to 2860·6)	1586·6 (1370·5 to 1828·5)	576·4 (541·2 to 615·6)	369·6 (296·0 to 454·6)	46·1 (39·2 to 55·5)
Argentina*	2041	1084·3 (976·1 to 1208·6)	667·9 (567·6 to 776·3)	239·7 (225·0 to 256·1)	157·3 (125·4 to 194·4)	19·4 (16·6 to 23·3)	2619·2 (2357·8 to 2919·4)	1613·4 (1371·0 to 1875·0)	579·0 (543·5 to 618·7)	380·0 (303·0 to 469·6)	46·8 (40·1 to 56·4)
Argentina*	2042	1101·0 (987·7 to 1231·3)	678·8 (572·5 to 792·4)	240·8 (226·0 to 257·4)	161·7 (128·0 to 201·1)	19·7 (16·8 to 23·5)	2659·4 (2385·7 to 2974·2)	1639·5 (1382·8 to 1913·9)	581·6 (546·0 to 621·7)	390·7 (309·2 to 485·7)	47·6 (40·6 to 56·9)
Argentina*	2043	1117·8 (999·2 to 1253·8)	689·8 (580·2 to 809·4)	241·8 (226·9 to 258·3)	166·2 (130·5 to 207·9)	20·0 (17·0 to 24·0)	2700·1 (2413·6 to 3028·6)	1666·2 (1401·6 to 1955·1)	584·1 (548·1 to 623·9)	401·5 (315·3 to 502·3)	48·3 (41·0 to 58·0)
Argentina*	2044	1136·1 (1013·1 to 1277·6)	701·9 (585·0 to 830·3)	243·0 (227·8 to 259·7)	171·0 (133·4 to 214·9)	20·2 (17·2 to 24·3)	2744·2 (2447·2 to 3086·0)	1695·5 (1413·2 to 2005·7)	586·9 (550·3 to 627·3)	413·0 (322·3 to 519·2)	48·8 (41·6 to 58·6)
Argentina*	2045	1152·1 (1024·3 to 1296·2)	712·5 (593·5 to 842·1)	243·7 (228·6 to 260·7)	175·5 (136·6 to 221·9)	20·4 (17·3 to 24·4)	2782·9 (2474·1 to 3131·0)	1720·9 (1433·5 to 2034·0)	588·6 (552·1 to 629·6)	424·0 (329·9 to 535·9)	49·3 (41·9 to 58·9)
Argentina*	2046	1170·2 (1037·4 to 1326·0)	724·3 (601·0 to 861·1)	244·8 (229·6 to 262·1)	180·5 (139·7 to 228·4)	20·5 (17·4 to 24·5)	2826·5 (2505·8 to 3203·0)	1749·5 (1451·7 to 2079·9)	591·4 (554·5 to 633·0)	436·1 (337·5 to 551·8)	49·6 (42·0 to 59·2)
Argentina*	2047	1188·0 (1049·0 to 1353·4)	736·4 (610·7 to 885·4)	245·6 (230·1 to 262·9)	185·3 (142·9 to 235·2)	20·6 (17·4 to 24·7)	2869·5 (2533·7 to 3269·1)	1778·8 (1475·2 to 2138·8)	593·2 (555·8 to 635·1)	447·6 (345·1 to 568·2)	49·9 (42·1 to 59·7)
Argentina*	2048	1204·4 (1058·5 to 1380·7)	747·4 (615·6 to 903·8)	246·2 (230·6 to 263·5)	190·1 (145·6 to 242·3)	20·7 (17·6 to 24·7)	2909·2 (2556·9 to 3335·0)	1805·3 (1486·9 to 2183·1)	594·7 (556·9 to 636·5)	459·2 (351·7 to 585·3)	50·0 (42·6 to 59·6)

Argentina*	2049	1221·5 (1072·7 to 1403·7)	758·4 (620·9 to 922·8)	247·2 (231·4 to 264·6)	195·3 (149·0 to 250·5)	20·6 (17·5 to 24·8)	2950·5 (2591·0 to 3390·7)	1831·8 (1499·8 to 2229·0)	597·1 (559·0 to 639·2)	471·8 (359·9 to 605·0)	49·9 (42·2 to 59·9)
Argentina*	2050	1238·9 (1086·4 to 1427·7)	769·7 (624·9 to 935·6)	248·1 (232·2 to 265·7)	200·6 (152·4 to 259·3)	20·5 (17·3 to 24·7)	2992·6 (2624·2 to 3448·7)	1859·2 (1509·5 to 2259·8)	599·4 (560·9 to 641·8)	484·5 (368·2 to 626·4)	49·5 (41·7 to 59·6)
Armenia*	1995	54·0 (46·0 to 63·8)	15·4 (12·8 to 18·7)	37·7 (30·1 to 47·0)	0·7 (0·5 to 0·9)	0·3 (0·3 to 0·3)	171·8 (146·5 to 203·0)	49·1 (40·6 to 59·4)	119·9 (95·9 to 149·7)	2·1 (1·4 to 2·9)	0·8 (0·8 to 0·8)
Armenia*	1996	55·8 (47·6 to 65·0)	15·1 (12·6 to 18·3)	38·6 (31·1 to 47·3)	0·8 (0·6 to 1·2)	1·2 (1·2 to 1·2)	177·6 (151·6 to 206·9)	48·1 (40·2 to 58·2)	122·9 (98·9 to 150·7)	2·7 (1·9 to 3·8)	3·9 (3·9 to 3·9)
Armenia*	1997	56·7 (48·5 to 65·9)	14·7 (12·3 to 17·6)	40·5 (32·9 to 49·2)	1·0 (0·7 to 1·4)	0·6 (0·6 to 0·6)	180·6 (154·3 to 209·6)	46·7 (39·2 to 56·0)	128·9 (104·7 to 156·8)	3·3 (2·3 to 4·6)	1·8 (1·8 to 1·8)
Armenia*	1998	60·8 (52·7 to 69·8)	15·4 (12·8 to 18·3)	42·6 (34·8 to 51·2)	1·1 (0·8 to 1·6)	1·7 (1·7 to 1·7)	193·5 (167·7 to 222·2)	48·9 (40·8 to 58·3)	135·6 (110·8 to 162·9)	3·6 (2·5 to 5·1)	5·4 (5·4 to 5·4)
Armenia*	1999	62·6 (54·5 to 71·8)	15·4 (12·8 to 18·4)	43·5 (35·8 to 52·0)	1·2 (0·8 to 1·7)	2·6 (2·6 to 2·6)	199·4 (173·3 to 228·5)	48·9 (40·8 to 58·6)	138·5 (114·1 to 165·7)	3·8 (2·6 to 5·5)	8·2 (8·2 to 8·2)
Armenia*	2000	65·1 (57·3 to 74·1)	15·2 (12·6 to 18·2)	43·8 (36·5 to 52·0)	1·3 (0·9 to 1·8)	4·8 (4·8 to 4·8)	207·1 (182·4 to 235·8)	48·3 (40·0 to 58·0)	139·4 (116·3 to 165·6)	4·0 (2·8 to 5·6)	15·4 (15·4 to 15·4)
Armenia*	2001	72·1 (63·8 to 81·7)	17·1 (14·3 to 20·5)	50·7 (43·0 to 59·6)	1·4 (1·0 to 1·9)	2·9 (2·9 to 2·9)	229·5 (203·0 to 260·2)	54·4 (45·5 to 65·4)	161·5 (137·0 to 189·8)	4·4 (3·1 to 6·2)	9·1 (9·1 to 9·1)
Armenia*	2002	93·6 (83·3 to 105·4)	19·2 (16·0 to 22·9)	68·2 (58·3 to 79·2)	1·5 (1·0 to 2·1)	4·7 (4·7 to 4·7)	298·0 (265·1 to 335·7)	61·1 (51·0 to 72·8)	217·2 (185·6 to 252·3)	4·7 (3·3 to 6·6)	15·0 (15·0 to 15·0)
Armenia*	2003	108·4 (96·9 to 121·0)	23·0 (19·3 to 27·3)	82·1 (71·3 to 94·0)	1·5 (1·0 to 2·1)	1·9 (1·9 to 1·9)	345·1 (308·5 to 385·0)	73·1 (61·3 to 87·0)	261·2 (226·9 to 299·4)	4·7 (3·3 to 6·6)	6·1 (6·1 to 6·1)
Armenia*	2004	117·5 (105·9 to 129·3)	26·4 (22·2 to 31·1)	87·1 (76·2 to 98·9)	1·1 (0·7 to 1·5)	3·0 (3·0 to 3·0)	374·0 (337·2 to 411·7)	83·9 (70·6 to 99·1)	277·3 (242·7 to 314·9)	3·4 (2·4 to 4·8)	9·5 (9·5 to 9·5)
Armenia*	2005	131·1 (118·2 to 143·5)	31·9 (27·0 to 37·4)	92·8 (82·0 to 104·6)	0·5 (0·3 to 0·7)	6·0 (6·0 to 6·0)	417·2 (376·4 to 456·7)	101·4 (85·8 to 118·9)	295·3 (261·2 to 333·1)	1·5 (1·0 to 2·1)	19·0 (19·0 to 19·0)
Armenia*	2006	149·6 (136·8 to 163·2)	37·8 (32·1 to 44·3)	102·5 (91·2 to 114·6)	0·4 (0·2 to 0·5)	9·0 (9·0 to 9·0)	476·4 (435·5 to 519·5)	120·4 (102·1 to 141·1)	326·2 (290·3 to 364·9)	1·1 (0·8 to 1·6)	28·7 (28·7 to 28·7)

Armenia*	2007	177·4 (162·8 to 191·7)	46·1 (39·2 to 54·2)	120·9 (108·4 to 133·9)	0·4 (0·3 to 0·6)	10·0 (10·0 to 10·0)	564·7 (518·2 to 610·4)	146·8 (124·8 to 172·6)	384·7 (345·2 to 426·4)	1·4 (1·0 to 2·0)	31·8 (31·8 to 31·8)
Armenia*	2008	203·6 (188·3 to 218·7)	48·3 (41·2 to 56·5)	146·9 (133·3 to 160·6)	0·6 (0·4 to 0·8)	7·8 (7·8 to 7·8)	648·0 (599·4 to 696·0)	153·6 (131·1 to 179·7)	467·6 (424·4 to 511·1)	1·9 (1·3 to 2·7)	24·9 (24·9 to 24·9)
Armenia*	2009	240·0 (223·7 to 256·4)	51·0 (43·7 to 59·1)	173·8 (159·6 to 188·2)	0·8 (0·6 to 1·2)	14·3 (14·3 to 14·3)	763·9 (712·2 to 816·3)	162·5 (139·1 to 188·2)	553·2 (508·1 to 599·0)	2·6 (1·8 to 3·7)	45·6 (45·6 to 45·6)
Armenia*	2010	256·4 (239·0 to 273·2)	49·0 (42·1 to 56·4)	196·5 (182·1 to 212·4)	1·2 (0·8 to 1·6)	9·7 (9·7 to 9·7)	816·1 (761·0 to 869·6)	156·1 (134·1 to 179·4)	625·6 (579·7 to 676·1)	3·7 (2·5 to 5·1)	30·7 (30·7 to 30·7)
Armenia*	2011	282·4 (265·2 to 299·5)	49·5 (42·7 to 57·2)	221·0 (205·9 to 237·2)	1·8 (1·2 to 2·5)	10·1 (10·1 to 10·1)	898·9 (844·1 to 953·5)	157·7 (135·9 to 182·0)	703·4 (655·4 to 755·1)	5·7 (3·9 to 8·0)	32·1 (32·1 to 32·1)
Armenia*	2012	304·5 (285·4 to 322·3)	48·3 (41·5 to 55·9)	242·0 (225·5 to 257·9)	2·9 (2·0 to 4·2)	11·2 (11·2 to 11·2)	969·2 (908·5 to 1025·9)	153·7 (132·2 to 177·9)	770·5 (718·0 to 820·9)	9·4 (6·3 to 13·2)	35·6 (35·6 to 35·6)
Armenia*	2013	333·9 (315·8 to 352·1)	49·9 (43·0 to 57·7)	270·7 (254·3 to 287·2)	4·2 (2·9 to 5·9)	9·0 (9·0 to 9·0)	1062·9 (1005·2 to 1120·9)	158·9 (136·8 to 183·6)	861·8 (809·4 to 914·3)	13·4 (9·1 to 18·8)	28·7 (28·7 to 28·7)
Armenia*	2014	348·3 (329·1 to 367·7)	51·7 (44·6 to 59·5)	286·7 (270·4 to 304·3)	4·4 (2·9 to 6·1)	5·5 (5·5 to 5·5)	1108·7 (1047·8 to 1170·6)	164·5 (141·9 to 189·4)	912·7 (860·8 to 968·6)	13·9 (9·3 to 19·5)	17·6 (17·6 to 17·6)
Armenia*	2015	367·1 (347·7 to 388·6)	56·0 (48·3 to 65·1)	302·3 (285·2 to 320·9)	4·7 (3·2 to 6·6)	4·0 (4·0 to 4·0)	1168·5 (1107·0 to 1236·9)	178·2 (153·8 to 207·3)	962·5 (908·0 to 1021·4)	15·0 (10·3 to 21·0)	12·7 (12·7 to 12·7)
Armenia*	2016	377·3 (358·9 to 396·0)	56·5 (48·7 to 65·7)	309·2 (291·8 to 327·2)	4·5 (3·1 to 6·3)	7·1 (7·1 to 7·1)	1201·1 (1142·6 to 1260·5)	179·8 (155·2 to 209·3)	984·4 (929·0 to 1041·6)	14·5 (10·0 to 20·1)	22·5 (22·5 to 22·5)
Armenia*	2017	396·7 (377·7 to 416·7)	56·4 (47·9 to 65·3)	330·3 (312·4 to 347·8)	5·2 (3·5 to 7·4)	4·8 (4·8 to 4·8)	1263·0 (1202·3 to 1326·5)	179·6 (152·5 to 208·0)	1051·6 (994·6 to 1107·2)	16·5 (11·2 to 23·5)	15·3 (15·3 to 15·3)
Armenia*	2018	411·8 (386·6 to 436·7)	55·3 (46·5 to 64·4)	344·6 (322·9 to 368·6)	7·0 (4·7 to 9·8)	5·0 (5·0 to 5·0)	1311·0 (1230·7 to 1390·2)	175·9 (148·2 to 204·9)	1096·9 (1027·9 to 1173·3)	22·1 (15·1 to 31·3)	16·0 (16·0 to 16·0)
Armenia*	2019	435·7 (408·8 to 462·5)	60·7 (51·2 to 70·7)	362·9 (340·2 to 388·7)	7·5 (5·2 to 10·6)	4·5 (4·4 to 4·6)	1386·9 (1301·4 to 1472·2)	193·1 (163·1 to 224·9)	1155·3 (1083·1 to 1237·4)	24·0 (16·4 to 33·7)	14·4 (14·1 to 14·7)
Armenia*	2020	444·4 (419·6 to 469·5)	93·7 (84·3 to 103·7)	336·9 (316·0 to 360·5)	7·2 (4·9 to 10·1)	6·7 (6·6 to 6·7)	1414·7 (1335·6 to 1494·6)	298·3 (268·3 to 330·1)	1072·4 (1006·0 to 1147·6)	22·8 (15·6 to 32·2)	21·2 (21·0 to 21·3)

Armenia*	2021	402·2 (378·6 to 427·0)	64·3 (54·2 to 75·5)	324·2 (303·9 to 346·8)	7·1 (4·8 to 10·0)	6·6 (6·5 to 6·8)	1280·2 (1205·2 to 1359·4)	204·7 (172·4 to 240·3)	1031·9 (967·4 to 1104·0)	22·5 (15·4 to 31·7)	21·1 (20·8 to 21·5)
Armenia*	2022	394·7 (370·9 to 419·3)	57·6 (48·0 to 67·6)	324·5 (303·8 to 346·9)	7·3 (5·0 to 10·2)	5·3 (5·0 to 5·6)	1256·4 (1180·6 to 1334·8)	183·4 (152·8 to 215·3)	1033·0 (967·2 to 1104·4)	23·1 (15·8 to 32·5)	16·9 (16·0 to 17·8)
Armenia*	2023	397·8 (373·7 to 422·6)	59·0 (49·2 to 69·4)	325·7 (305·0 to 348·5)	7·4 (5·1 to 10·5)	5·6 (5·3 to 6·0)	1266·2 (1189·7 to 1345·3)	187·8 (156·8 to 221·0)	1036·9 (970·8 to 1109·2)	23·7 (16·2 to 33·3)	17·8 (16·8 to 18·9)
Armenia*	2024	400·0 (375·9 to 424·9)	59·5 (49·4 to 70·5)	326·9 (305·7 to 349·7)	7·7 (5·3 to 10·8)	5·9 (5·5 to 6·4)	1273·4 (1196·6 to 1352·5)	189·5 (157·1 to 224·6)	1040·5 (973·2 to 1113·3)	24·5 (16·7 to 34·4)	18·8 (17·6 to 20·3)
Armenia*	2025	402·5 (377·9 to 427·7)	60·1 (50·0 to 71·3)	328·2 (307·0 to 351·2)	8·0 (5·4 to 11·2)	6·2 (5·8 to 6·8)	1281·3 (1203·0 to 1361·6)	191·2 (159·0 to 227·0)	1044·9 (977·2 to 1117·9)	25·4 (17·3 to 35·7)	19·8 (18·5 to 21·5)
Armenia*	2026	405·3 (380·5 to 430·8)	60·6 (50·3 to 72·3)	329·9 (308·6 to 353·1)	8·3 (5·6 to 11·6)	6·5 (6·0 to 7·2)	1290·3 (1211·2 to 1371·5)	192·9 (160·1 to 230·1)	1050·3 (982·4 to 1124·0)	26·3 (17·9 to 36·9)	20·8 (19·2 to 22·8)
Armenia*	2027	408·5 (383·4 to 434·4)	61·3 (50·7 to 73·3)	331·9 (310·3 to 355·3)	8·5 (5·8 to 11·9)	6·8 (6·2 to 7·5)	1300·4 (1220·5 to 1382·8)	195·2 (161·3 to 233·3)	1056·6 (987·8 to 1130·9)	27·1 (18·5 to 38·0)	21·6 (19·8 to 24·0)
Armenia*	2028	412·9 (387·7 to 439·1)	62·2 (51·0 to 74·5)	334·9 (313·0 to 358·3)	8·8 (6·0 to 12·3)	7·0 (6·4 to 7·8)	1314·3 (1234·1 to 1397·7)	198·0 (162·4 to 237·2)	1066·2 (996·4 to 1140·5)	27·9 (19·1 to 39·1)	22·2 (20·3 to 24·9)
Armenia*	2029	417·8 (392·3 to 444·0)	63·2 (52·1 to 75·8)	338·4 (316·2 to 362·3)	9·1 (6·2 to 12·7)	7·1 (6·5 to 8·1)	1330·1 (1248·7 to 1413·5)	201·3 (165·9 to 241·2)	1077·2 (1006·4 to 1153·2)	28·9 (19·7 to 40·4)	22·8 (20·7 to 25·8)
Armenia*	2030	423·3 (397·5 to 449·7)	64·3 (53·1 to 76·9)	342·3 (319·6 to 366·6)	9·4 (6·4 to 13·2)	7·3 (6·5 to 8·3)	1347·4 (1265·3 to 1431·5)	204·6 (169·2 to 244·9)	1089·7 (1017·2 to 1166·9)	29·9 (20·3 to 41·9)	23·2 (20·8 to 26·4)
Armenia*	2031	429·5 (402·8 to 456·6)	65·5 (53·9 to 78·4)	346·9 (323·7 to 371·5)	9·7 (6·6 to 13·7)	7·4 (6·6 to 8·5)	1367·2 (1282·3 to 1453·5)	208·6 (171·6 to 249·6)	1104·1 (1030·3 to 1182·7)	31·0 (21·1 to 43·6)	23·4 (21·0 to 26·9)
Armenia*	2032	435·6 (408·7 to 462·9)	66·7 (55·0 to 80·0)	351·3 (327·7 to 376·3)	10·1 (6·8 to 14·2)	7·5 (6·7 to 8·6)	1386·5 (1300·9 to 1473·5)	212·4 (175·0 to 254·5)	1118·1 (1043·2 to 1197·8)	32·1 (21·8 to 45·3)	23·8 (21·3 to 27·5)
Armenia*	2033	442·3 (415·2 to 470·6)	68·1 (55·3 to 81·8)	356·2 (332·3 to 381·6)	10·5 (7·1 to 14·8)	7·5 (6·7 to 8·8)	1408·0 (1321·7 to 1498·2)	216·7 (176·0 to 260·4)	1134·0 (1057·9 to 1214·9)	33·3 (22·6 to 47·0)	24·0 (21·5 to 28·0)
Armenia*	2034	449·8 (422·0 to 478·5)	69·5 (56·9 to 83·6)	361·7 (337·6 to 387·6)	10·9 (7·4 to 15·3)	7·6 (6·8 to 8·9)	1431·8 (1343·3 to 1523·2)	221·4 (181·0 to 266·1)	1151·6 (1074·7 to 1233·7)	34·6 (23·5 to 48·8)	24·2 (21·6 to 28·3)

Armenia*	2035	457·2 (428·5 to 486·2)	71·0 (58·1 to 85·7)	367·2 (342·7 to 393·2)	11·3 (7·6 to 16·0)	7·7 (6·8 to 9·0)	1455·4 (1364·1 to 1547·8)	226·0 (184·9 to 272·9)	1169·0 (1091·0 to 1251·8)	36·0 (24·3 to 51·0)	24·4 (21·7 to 28·8)
Armenia*	2036	464·6 (435·3 to 493·8)	72·5 (58·6 to 87·8)	372·7 (347·6 to 398·8)	11·7 (7·9 to 16·6)	7·7 (6·8 to 9·1)	1478·9 (1385·7 to 1571·8)	230·6 (186·4 to 279·3)	1186·3 (1106·6 to 1269·6)	37·4 (25·2 to 53·0)	24·6 (21·8 to 29·0)
Armenia*	2037	471·3 (441·9 to 501·3)	73·8 (59·4 to 89·2)	377·5 (352·1 to 404·0)	12·2 (8·2 to 17·3)	7·8 (6·9 to 9·3)	1500·3 (1406·6 to 1595·9)	234·9 (189·0 to 283·8)	1201·7 (1120·7 to 1286·0)	38·7 (26·2 to 54·9)	25·0 (22·1 to 29·7)
Armenia*	2038	477·7 (447·2 to 508·1)	75·0 (60·3 to 90·5)	382·0 (356·2 to 409·0)	12·6 (8·5 to 17·9)	8·0 (7·1 to 9·6)	1520·6 (1423·7 to 1617·5)	238·9 (192·0 to 288·1)	1216·1 (1134·0 to 1301·9)	40·1 (27·1 to 56·8)	25·5 (22·5 to 30·4)
Armenia*	2039	484·1 (453·2 to 515·1)	76·4 (61·6 to 93·4)	386·4 (360·3 to 413·8)	13·0 (8·8 to 18·5)	8·2 (7·2 to 9·9)	1541·0 (1442·7 to 1639·7)	243·4 (196·0 to 297·5)	1230·0 (1146·9 to 1317·3)	41·5 (28·0 to 58·8)	26·1 (22·9 to 31·6)
Armenia*	2040	490·3 (459·1 to 521·8)	77·8 (62·5 to 95·4)	390·6 (364·1 to 418·4)	13·5 (9·1 to 19·2)	8·4 (7·3 to 10·2)	1560·8 (1461·5 to 1661·2)	247·7 (198·9 to 303·7)	1243·4 (1159·2 to 1332·0)	43·0 (28·9 to 61·0)	26·8 (23·4 to 32·5)
Armenia*	2041	496·1 (464·5 to 528·0)	79·1 (63·2 to 96·5)	394·4 (367·7 to 422·7)	13·9 (9·3 to 19·8)	8·7 (7·5 to 10·6)	1579·2 (1478·7 to 1680·7)	251·7 (201·1 to 307·3)	1255·5 (1170·5 to 1345·5)	44·4 (29·8 to 63·1)	27·6 (23·9 to 33·7)
Armenia*	2042	501·4 (469·4 to 533·6)	80·2 (64·2 to 99·0)	397·9 (371·2 to 426·5)	14·4 (9·7 to 20·5)	9·0 (7·7 to 11·1)	1596·2 (1494·3 to 1698·5)	255·3 (204·4 to 315·3)	1266·6 (1181·5 to 1357·8)	45·8 (30·7 to 65·4)	28·6 (24·6 to 35·3)
Armenia*	2043	506·5 (473·8 to 539·1)	81·4 (64·2 to 100·6)	401·0 (374·0 to 429·8)	14·9 (10·0 to 21·3)	9·3 (8·0 to 11·7)	1612·4 (1508·2 to 1716·1)	259·0 (204·3 to 320·3)	1276·4 (1190·5 to 1368·2)	47·3 (31·7 to 67·8)	29·7 (25·5 to 37·2)
Armenia*	2044	511·4 (478·0 to 544·9)	82·5 (64·6 to 103·3)	403·9 (376·4 to 433·0)	15·3 (10·2 to 22·1)	9·7 (8·3 to 12·3)	1628·0 (1521·8 to 1734·5)	262·7 (205·6 to 328·9)	1285·6 (1198·2 to 1378·3)	48·8 (32·5 to 70·4)	31·0 (26·4 to 39·3)
Armenia*	2045	515·8 (481·8 to 550·2)	83·6 (65·6 to 103·7)	406·3 (378·4 to 435·7)	15·8 (10·4 to 22·9)	10·2 (8·6 to 13·2)	1642·1 (1533·8 to 1751·4)	266·0 (208·9 to 330·0)	1293·4 (1204·5 to 1386·9)	50·2 (33·2 to 73·0)	32·4 (27·5 to 41·9)
Armenia*	2046	519·6 (485·0 to 554·8)	84·4 (65·9 to 104·9)	408·2 (379·9 to 437·6)	16·2 (10·7 to 23·6)	10·7 (9·0 to 13·9)	1654·0 (1543·8 to 1765·9)	268·8 (209·6 to 333·9)	1299·4 (1209·4 to 1393·1)	51·7 (33·9 to 75·3)	34·1 (28·8 to 44·3)
Armenia*	2047	523·1 (488·3 to 558·3)	85·3 (66·0 to 105·7)	409·7 (381·3 to 439·6)	16·7 (10·9 to 24·4)	11·3 (9·5 to 14·9)	1665·1 (1554·4 to 1777·3)	271·7 (210·1 to 336·5)	1304·2 (1213·9 to 1399·5)	53·1 (34·7 to 77·5)	36·1 (30·1 to 47·3)
Armenia*	2048	525·8 (490·9 to 562·0)	86·0 (66·7 to 107·6)	410·6 (382·3 to 440·4)	17·1 (11·2 to 25·0)	12·1 (10·1 to 16·2)	1673·7 (1562·6 to 1789·0)	273·8 (212·4 to 342·5)	1306·9 (1216·9 to 1401·9)	54·6 (35·5 to 79·7)	38·4 (32·1 to 51·4)

Armenia*	2049	528·2 (493·2 to 564·7)	86·6 (66·9 to 109·1)	411·1 (382·5 to 440·6)	17·6 (11·4 to 25·7)	12·9 (10·6 to 17·4)	1681·5 (1570·1 to 1797·5)	275·8 (213·0 to 347·5)	1308·7 (1217·7 to 1402·4)	56·0 (36·3 to 81·7)	41·0 (33·8 to 55·5)
Armenia*	2050	530·6 (495·1 to 567·6)	87·3 (68·1 to 109·6)	411·5 (382·6 to 441·3)	18·0 (11·6 to 26·5)	13·8 (11·3 to 18·8)	1689·1 (1576·1 to 1806·9)	277·8 (216·6 to 348·8)	1310·1 (1218·0 to 1404·7)	57·4 (36·9 to 84·2)	43·9 (35·8 to 59·8)
American Samoa*	1995	593·6 (504·0 to 701·4)	467·2 (381·2 to 569·8)	120·7 (93·5 to 153·4)	5·8 (3·9 to 8·2)	0·0 (0·0 to 0·0)	593·6 (504·0 to 701·4)	467·2 (381·2 to 569·8)	120·7 (93·5 to 153·4)	5·8 (3·9 to 8·2)	0·0 (0·0 to 0·0)
American Samoa*	1996	596·8 (500·3 to 707·2)	471·0 (383·4 to 575·2)	119·8 (92·9 to 151·5)	5·9 (4·1 to 8·5)	0·0 (0·0 to 0·0)	596·8 (500·3 to 707·2)	471·0 (383·4 to 575·2)	119·8 (92·9 to 151·5)	5·9 (4·1 to 8·5)	0·0 (0·0 to 0·0)
American Samoa*	1997	602·9 (510·2 to 712·7)	477·4 (391·8 to 579·2)	119·6 (92·8 to 149·7)	5·9 (4·2 to 8·5)	0·0 (0·0 to 0·0)	602·9 (510·2 to 712·7)	477·4 (391·8 to 579·2)	119·6 (92·8 to 149·7)	5·9 (4·2 to 8·5)	0·0 (0·0 to 0·0)
American Samoa*	1998	604·6 (514·2 to 710·0)	480·1 (396·5 to 579·0)	119·0 (92·4 to 150·3)	5·4 (3·8 to 7·7)	0·0 (0·0 to 0·0)	604·6 (514·2 to 710·0)	480·1 (396·5 to 579·0)	119·0 (92·4 to 150·3)	5·4 (3·8 to 7·7)	0·0 (0·0 to 0·0)
American Samoa*	1999	607·0 (516·9 to 709·5)	484·0 (399·4 to 580·9)	118·6 (92·5 to 149·6)	4·4 (3·1 to 6·3)	0·0 (0·0 to 0·0)	607·0 (516·9 to 709·5)	484·0 (399·4 to 580·9)	118·6 (92·5 to 149·6)	4·4 (3·1 to 6·3)	0·0 (0·0 to 0·0)
American Samoa*	2000	614·0 (523·3 to 713·4)	492·3 (411·5 to 583·4)	118·5 (93·1 to 149·4)	3·2 (2·2 to 4·5)	0·0 (0·0 to 0·0)	614·0 (523·3 to 713·4)	492·3 (411·5 to 583·4)	118·5 (93·1 to 149·4)	3·2 (2·2 to 4·5)	0·0 (0·0 to 0·0)
American Samoa*	2001	617·8 (529·5 to 708·3)	497·9 (421·3 to 583·9)	118·2 (93·2 to 148·5)	1·7 (1·2 to 2·5)	0·0 (0·0 to 0·0)	617·8 (529·5 to 708·3)	497·9 (421·3 to 583·9)	118·2 (93·2 to 148·5)	1·7 (1·2 to 2·5)	0·0 (0·0 to 0·0)
American Samoa*	2002	622·9 (543·4 to 711·7)	502·1 (429·1 to 582·1)	118·9 (94·3 to 149·4)	1·9 (1·3 to 2·7)	0·0 (0·0 to 0·0)	622·9 (543·4 to 711·7)	502·1 (429·1 to 582·1)	118·9 (94·3 to 149·4)	1·9 (1·3 to 2·7)	0·0 (0·0 to 0·0)
American Samoa*	2003	631·0 (553·5 to 718·9)	508·5 (436·2 to 591·1)	120·1 (95·3 to 151·2)	2·4 (1·7 to 3·4)	0·0 (0·0 to 0·0)	631·0 (553·5 to 718·9)	508·5 (436·2 to 591·1)	120·1 (95·3 to 151·2)	2·4 (1·7 to 3·4)	0·0 (0·0 to 0·0)
American Samoa*	2004	633·3 (549·9 to 725·8)	509·2 (433·1 to 592·5)	121·2 (96·1 to 152·6)	2·8 (2·0 to 4·0)	0·0 (0·0 to 0·0)	633·3 (549·9 to 725·8)	509·2 (433·1 to 592·5)	121·2 (96·1 to 152·6)	2·8 (2·0 to 4·0)	0·0 (0·0 to 0·0)
American Samoa*	2005	632·3 (548·3 to 726·6)	506·7 (427·7 to 595·3)	122·3 (96·7 to 153·4)	3·2 (2·2 to 4·5)	0·0 (0·0 to 0·0)	632·3 (548·3 to 726·6)	506·7 (427·7 to 595·3)	122·3 (96·7 to 153·4)	3·2 (2·2 to 4·5)	0·0 (0·0 to 0·0)
American Samoa*	2006	618·8 (535·2 to 711·7)	493·0 (414·7 to 587·1)	122·0 (96·5 to 153·9)	3·8 (2·6 to 5·4)	0·0 (0·0 to 0·0)	618·8 (535·2 to 711·7)	493·0 (414·7 to 587·1)	122·0 (96·5 to 153·9)	3·8 (2·6 to 5·4)	0·0 (0·0 to 0·0)

American Samoa*	2007	626·0 (538·6 to 723·8)	497·2 (418·5 to 589·3)	123·7 (99·0 to 156·2)	5·1 (3·4 to 7·1)	0·0 (0·0 to 0·0)	626·0 (538·6 to 723·8)	497·2 (418·5 to 589·3)	123·7 (99·0 to 156·2)	5·1 (3·4 to 7·1)	0·0 (0·0 to 0·0)
American Samoa*	2008	617·2 (530·9 to 711·1)	485·1 (404·5 to 577·8)	123·5 (98·1 to 156·7)	8·6 (5·8 to 12·0)	0·0 (0·0 to 0·0)	617·2 (530·9 to 711·1)	485·1 (404·5 to 577·8)	123·5 (98·1 to 156·7)	8·6 (5·8 to 12·0)	0·0 (0·0 to 0·0)
American Samoa*	2009	599·1 (510·2 to 694·0)	466·3 (384·9 to 559·5)	121·8 (96·3 to 153·5)	11·0 (7·4 to 15·4)	0·0 (0·0 to 0·0)	599·1 (510·2 to 694·0)	466·3 (384·9 to 559·5)	121·8 (96·3 to 153·5)	11·0 (7·4 to 15·4)	0·0 (0·0 to 0·0)
American Samoa*	2010	595·2 (505·3 to 689·8)	461·1 (380·3 to 551·2)	121·4 (95·3 to 153·3)	12·7 (8·5 to 17·6)	0·0 (0·0 to 0·0)	595·2 (505·3 to 689·8)	461·1 (380·3 to 551·2)	121·4 (95·3 to 153·3)	12·7 (8·5 to 17·6)	0·0 (0·0 to 0·0)
American Samoa*	2011	593·9 (507·0 to 685·2)	458·9 (381·1 to 548·3)	121·4 (94·8 to 152·4)	13·6 (9·0 to 18·7)	0·0 (0·0 to 0·0)	593·9 (507·0 to 685·2)	458·9 (381·1 to 548·3)	121·4 (94·8 to 152·4)	13·6 (9·0 to 18·7)	0·0 (0·0 to 0·0)
American Samoa*	2012	575·2 (496·1 to 661·2)	442·2 (369·4 to 527·2)	119·0 (93·0 to 151·3)	14·1 (9·2 to 19·7)	0·0 (0·0 to 0·0)	575·2 (496·1 to 661·2)	442·2 (369·4 to 527·2)	119·0 (93·0 to 151·3)	14·1 (9·2 to 19·7)	0·0 (0·0 to 0·0)
American Samoa*	2013	563·7 (488·5 to 646·0)	432·4 (366·8 to 511·1)	116·9 (90·2 to 146·5)	14·4 (9·6 to 20·1)	0·0 (0·0 to 0·0)	563·7 (488·5 to 646·0)	432·4 (366·8 to 511·1)	116·9 (90·2 to 146·5)	14·4 (9·6 to 20·1)	0·0 (0·0 to 0·0)
American Samoa*	2014	567·9 (494·9 to 651·7)	436·9 (370·4 to 517·3)	116·2 (91·3 to 144·9)	14·7 (9·9 to 20·8)	0·0 (0·0 to 0·0)	567·9 (494·9 to 651·7)	436·9 (370·4 to 517·3)	116·2 (91·3 to 144·9)	14·7 (9·9 to 20·8)	0·0 (0·0 to 0·0)
American Samoa*	2015	573·6 (497·2 to 659·9)	443·2 (373·5 to 527·9)	115·4 (90·5 to 143·8)	14·9 (10·0 to 21·2)	0·0 (0·0 to 0·0)	573·6 (497·2 to 659·9)	443·2 (373·5 to 527·9)	115·4 (90·5 to 143·8)	14·9 (10·0 to 21·2)	0·0 (0·0 to 0·0)
American Samoa*	2016	572·0 (493·3 to 657·7)	442·8 (368·5 to 524·6)	114·0 (89·8 to 143·5)	15·1 (10·2 to 21·3)	0·0 (0·0 to 0·0)	572·0 (493·3 to 657·7)	442·8 (368·5 to 524·6)	114·0 (89·8 to 143·5)	15·1 (10·2 to 21·3)	0·0 (0·0 to 0·0)
American Samoa*	2017	561·3 (482·3 to 648·5)	433·5 (356·6 to 514·7)	111·7 (88·9 to 140·7)	16·0 (10·8 to 22·3)	0·0 (0·0 to 0·0)	561·3 (482·3 to 648·5)	433·5 (356·6 to 514·7)	111·7 (88·9 to 140·7)	16·0 (10·8 to 22·3)	0·0 (0·0 to 0·0)
American Samoa*	2018	581·0 (494·3 to 672·3)	447·9 (366·3 to 532·9)	112·7 (89·4 to 142·2)	20·5 (13·8 to 28·4)	0·0 (0·0 to 0·0)	581·0 (494·3 to 672·3)	447·9 (366·3 to 532·9)	112·7 (89·4 to 142·2)	20·5 (13·8 to 28·4)	0·0 (0·0 to 0·0)
American Samoa*	2019	577·3 (491·8 to 667·9)	445·6 (365·3 to 531·0)	110·9 (88·0 to 140·5)	20·8 (14·0 to 28·8)	0·0 (0·0 to 0·0)	577·3 (491·8 to 667·9)	445·6 (365·3 to 531·0)	110·9 (88·0 to 140·5)	20·8 (14·0 to 28·8)	0·0 (0·0 to 0·0)
American Samoa*	2020	573·9 (488·8 to 663·7)	443·4 (363·4 to 527·7)	109·3 (86·7 to 138·4)	21·2 (14·3 to 29·2)	0·0 (0·0 to 0·0)	573·9 (488·8 to 663·7)	443·4 (363·4 to 527·7)	109·3 (86·7 to 138·4)	21·2 (14·3 to 29·2)	0·0 (0·0 to 0·0)

American Samoa*	2021	575·3 (490·2 to 666·0)	444·1 (364·0 to 528·4)	109·4 (86·8 to 138·3)	21·8 (14·7 to 30·1)	0·0 (0·0 to 0·0)	575·3 (490·2 to 666·0)	444·1 (364·0 to 528·4)	109·4 (86·8 to 138·3)	21·8 (14·7 to 30·1)	0·0 (0·0 to 0·0)
American Samoa*	2022	583·7 (497·5 to 675·6)	451·1 (369·4 to 538·1)	110·1 (87·4 to 139·2)	22·5 (15·2 to 31·2)	0·0 (0·0 to 0·0)	583·7 (497·5 to 675·6)	451·1 (369·4 to 538·1)	110·1 (87·4 to 139·2)	22·5 (15·2 to 31·2)	0·0 (0·0 to 0·0)
American Samoa*	2023	590·5 (502·7 to 682·9)	456·6 (375·1 to 545·3)	110·6 (87·8 to 139·8)	23·3 (15·8 to 32·3)	0·0 (0·0 to 0·0)	590·5 (502·7 to 682·9)	456·6 (375·1 to 545·3)	110·6 (87·8 to 139·8)	23·3 (15·8 to 32·3)	0·0 (0·0 to 0·0)
American Samoa*	2024	596·6 (508·5 to 691·2)	461·6 (378·0 to 550·3)	111·0 (88·0 to 140·2)	24·0 (16·2 to 33·4)	0·0 (0·0 to 0·0)	596·6 (508·5 to 691·2)	461·6 (378·0 to 550·3)	111·0 (88·0 to 140·2)	24·0 (16·2 to 33·4)	0·0 (0·0 to 0·0)
American Samoa*	2025	602·5 (511·5 to 698·2)	466·2 (382·6 to 556·6)	111·5 (88·4 to 140·8)	24·8 (16·7 to 34·5)	0·0 (0·0 to 0·0)	602·5 (511·5 to 698·2)	466·2 (382·6 to 556·6)	111·5 (88·4 to 140·8)	24·8 (16·7 to 34·5)	0·0 (0·0 to 0·0)
American Samoa*	2026	607·6 (516·0 to 704·8)	470·2 (384·7 to 561·3)	111·9 (88·6 to 141·2)	25·5 (17·2 to 35·5)	0·0 (0·0 to 0·0)	607·6 (516·0 to 704·8)	470·2 (384·7 to 561·3)	111·9 (88·6 to 141·2)	25·5 (17·2 to 35·5)	0·0 (0·0 to 0·0)
American Samoa*	2027	611·9 (519·8 to 709·2)	473·6 (387·6 to 566·6)	112·0 (88·8 to 141·3)	26·3 (17·7 to 36·6)	0·0 (0·0 to 0·0)	611·9 (519·8 to 709·2)	473·6 (387·6 to 566·6)	112·0 (88·8 to 141·3)	26·3 (17·7 to 36·6)	0·0 (0·0 to 0·0)
American Samoa*	2028	615·4 (523·0 to 714·9)	476·4 (389·8 to 569·3)	112·0 (88·8 to 141·3)	26·9 (18·2 to 37·6)	0·0 (0·0 to 0·0)	615·4 (523·0 to 714·9)	476·4 (389·8 to 569·3)	112·0 (88·8 to 141·3)	26·9 (18·2 to 37·6)	0·0 (0·0 to 0·0)
American Samoa*	2029	618·1 (526·2 to 718·1)	478·6 (391·6 to 572·8)	111·9 (88·7 to 141·1)	27·6 (18·5 to 38·6)	0·0 (0·0 to 0·0)	618·1 (526·2 to 718·1)	478·6 (391·6 to 572·8)	111·9 (88·7 to 141·1)	27·6 (18·5 to 38·6)	0·0 (0·0 to 0·0)
American Samoa*	2030	620·4 (527·7 to 721·2)	480·5 (390·9 to 576·7)	111·6 (88·5 to 140·6)	28·3 (18·9 to 39·6)	0·0 (0·0 to 0·0)	620·4 (527·7 to 721·2)	480·5 (390·9 to 576·7)	111·6 (88·5 to 140·6)	28·3 (18·9 to 39·6)	0·0 (0·0 to 0·0)
American Samoa*	2031	622·2 (529·3 to 726·2)	482·0 (391·5 to 578·6)	111·2 (88·1 to 140·3)	29·0 (19·2 to 40·8)	0·0 (0·0 to 0·0)	622·2 (529·3 to 726·2)	482·0 (391·5 to 578·6)	111·2 (88·1 to 140·3)	29·0 (19·2 to 40·8)	0·0 (0·0 to 0·0)
American Samoa*	2032	623·9 (531·4 to 728·8)	483·5 (394·0 to 581·5)	110·8 (87·8 to 139·8)	29·6 (19·5 to 41·7)	0·0 (0·0 to 0·0)	623·9 (531·4 to 728·8)	483·5 (394·0 to 581·5)	110·8 (87·8 to 139·8)	29·6 (19·5 to 41·7)	0·0 (0·0 to 0·0)
American Samoa*	2033	625·6 (531·2 to 730·6)	484·9 (394·7 to 583·2)	110·4 (87·5 to 139·3)	30·2 (19·8 to 42·6)	0·0 (0·0 to 0·0)	625·6 (531·2 to 730·6)	484·9 (394·7 to 583·2)	110·4 (87·5 to 139·3)	30·2 (19·8 to 42·6)	0·0 (0·0 to 0·0)
American Samoa*	2034	627·1 (533·6 to 732·9)	486·3 (397·1 to 584·7)	110·0 (87·1 to 138·8)	30·9 (20·1 to 43·5)	0·0 (0·0 to 0·0)	627·1 (533·6 to 732·9)	486·3 (397·1 to 584·7)	110·0 (87·1 to 138·8)	30·9 (20·1 to 43·5)	0·0 (0·0 to 0·0)

American Samoa*	2035	628·8 (534·7 to 734·1)	487·7 (397·4 to 585·5)	109·5 (86·8 to 138·2)	31·5 (20·3 to 44·5)	0·0 (0·0 to 0·0)	628·8 (534·7 to 734·1)	487·7 (397·4 to 585·5)	109·5 (86·8 to 138·2)	31·5 (20·3 to 44·5)	0·0 (0·0 to 0·0)
American Samoa*	2036	630·8 (537·6 to 737·7)	489·6 (399·9 to 589·9)	109·1 (86·4 to 137·6)	32·1 (20·5 to 45·5)	0·0 (0·0 to 0·0)	630·8 (537·6 to 737·7)	489·6 (399·9 to 589·9)	109·1 (86·4 to 137·6)	32·1 (20·5 to 45·5)	0·0 (0·0 to 0·0)
American Samoa*	2037	633·0 (539·2 to 739·9)	491·5 (401·8 to 593·2)	108·7 (86·1 to 137·2)	32·7 (20·9 to 46·4)	0·0 (0·0 to 0·0)	633·0 (539·2 to 739·9)	491·5 (401·8 to 593·2)	108·7 (86·1 to 137·2)	32·7 (20·9 to 46·4)	0·0 (0·0 to 0·0)
American Samoa*	2038	636·2 (541·4 to 743·5)	494·3 (405·3 to 595·0)	108·5 (85·9 to 137·0)	33·4 (21·4 to 47·3)	0·0 (0·0 to 0·0)	636·2 (541·4 to 743·5)	494·3 (405·3 to 595·0)	108·5 (85·9 to 137·0)	33·4 (21·4 to 47·3)	0·0 (0·0 to 0·0)
American Samoa*	2039	639·7 (543·7 to 746·8)	497·4 (407·9 to 598·4)	108·3 (85·7 to 136·8)	34·1 (21·9 to 48·3)	0·0 (0·0 to 0·0)	639·7 (543·7 to 746·8)	497·4 (407·9 to 598·4)	108·3 (85·7 to 136·8)	34·1 (21·9 to 48·3)	0·0 (0·0 to 0·0)
American Samoa*	2040	643·5 (546·6 to 751·1)	500·6 (409·1 to 603·8)	108·1 (85·6 to 136·7)	34·8 (22·3 to 49·6)	0·0 (0·0 to 0·0)	643·5 (546·6 to 751·1)	500·6 (409·1 to 603·8)	108·1 (85·6 to 136·7)	34·8 (22·3 to 49·6)	0·0 (0·0 to 0·0)
American Samoa*	2041	648·2 (551·0 to 756·8)	504·5 (412·3 to 607·3)	108·1 (85·5 to 136·7)	35·5 (22·6 to 50·7)	0·0 (0·0 to 0·0)	648·2 (551·0 to 756·8)	504·5 (412·3 to 607·3)	108·1 (85·5 to 136·7)	35·5 (22·6 to 50·7)	0·0 (0·0 to 0·0)
American Samoa*	2042	653·2 (555·4 to 762·6)	508·9 (415·9 to 611·4)	108·1 (85·5 to 136·8)	36·3 (23·1 to 52·3)	0·0 (0·0 to 0·0)	653·2 (555·4 to 762·6)	508·9 (415·9 to 611·4)	108·1 (85·5 to 136·8)	36·3 (23·1 to 52·3)	0·0 (0·0 to 0·0)
American Samoa*	2043	659·0 (560·8 to 766·8)	513·7 (420·0 to 616·8)	108·2 (85·6 to 136·9)	37·1 (23·5 to 53·6)	0·0 (0·0 to 0·0)	659·0 (560·8 to 766·8)	513·7 (420·0 to 616·8)	108·2 (85·6 to 136·9)	37·1 (23·5 to 53·6)	0·0 (0·0 to 0·0)
American Samoa*	2044	665·2 (564·6 to 774·8)	518·9 (423·4 to 624·0)	108·4 (85·8 to 137·2)	37·9 (23·9 to 55·3)	0·0 (0·0 to 0·0)	665·2 (564·6 to 774·8)	518·9 (423·4 to 624·0)	108·4 (85·8 to 137·2)	37·9 (23·9 to 55·3)	0·0 (0·0 to 0·0)
American Samoa*	2045	672·1 (569·9 to 782·8)	524·6 (428·8 to 631·8)	108·7 (86·0 to 137·6)	38·8 (24·5 to 56·7)	0·0 (0·0 to 0·0)	672·1 (569·9 to 782·8)	524·6 (428·8 to 631·8)	108·7 (86·0 to 137·6)	38·8 (24·5 to 56·7)	0·0 (0·0 to 0·0)
American Samoa*	2046	679·2 (576·9 to 792·7)	530·5 (433·3 to 638·7)	109·0 (86·2 to 137·9)	39·7 (25·2 to 58·0)	0·0 (0·0 to 0·0)	679·2 (576·9 to 792·7)	530·5 (433·3 to 638·7)	109·0 (86·2 to 137·9)	39·7 (25·2 to 58·0)	0·0 (0·0 to 0·0)
American Samoa*	2047	686·6 (583·1 to 800·4)	536·7 (438·0 to 645·4)	109·3 (86·4 to 138·3)	40·6 (25·7 to 59·4)	0·0 (0·0 to 0·0)	686·6 (583·1 to 800·4)	536·7 (438·0 to 645·4)	109·3 (86·4 to 138·3)	40·6 (25·7 to 59·4)	0·0 (0·0 to 0·0)
American Samoa*	2048	694·4 (588·4 to 809·0)	543·2 (443·1 to 653·3)	109·7 (86·7 to 138·7)	41·6 (26·1 to 61·3)	0·0 (0·0 to 0·0)	694·4 (588·4 to 809·0)	543·2 (443·1 to 653·3)	109·7 (86·7 to 138·7)	41·6 (26·1 to 61·3)	0·0 (0·0 to 0·0)

American Samoa*	2049	702·5 (595·6 to 818·9)	550·0 (449·7 to 661·5)	110·0 (87·1 to 139·1)	42·5 (26·4 to 62·5)	0·0 (0·0 to 0·0)	702·5 (595·6 to 818·9)	550·0 (449·7 to 661·5)	110·0 (87·1 to 139·1)	42·5 (26·4 to 62·5)	0·0 (0·0 to 0·0)
American Samoa*	2050	710·5 (602·9 to 828·4)	556·7 (451·9 to 670·7)	110·4 (87·3 to 139·6)	43·5 (27·0 to 63·6)	0·0 (0·0 to 0·0)	710·5 (602·9 to 828·4)	556·7 (451·9 to 670·7)	110·4 (87·3 to 139·6)	43·5 (27·0 to 63·6)	0·0 (0·0 to 0·0)
Antigua and Barbuda	1995	712·0 (664·1 to 762·6)	429·6 (390·0 to 469·5)	150·2 (129·5 to 173·2)	132·2 (114·9 to 151·9)	0·0 (0·0 to 0·0)	938·1 (875·0 to 1004·8)	566·0 (513·9 to 618·6)	197·9 (170·6 to 228·2)	174·2 (151·4 to 200·1)	0·0 (0·0 to 0·0)
Antigua and Barbuda	1996	706·8 (664·2 to 747·9)	423·6 (389·0 to 457·7)	151·5 (133·2 to 171·6)	131·6 (118·2 to 147·9)	0·1 (0·1 to 0·1)	931·3 (875·1 to 985·4)	558·2 (512·5 to 603·0)	199·6 (175·5 to 226·1)	173·4 (155·8 to 194·9)	0·1 (0·1 to 0·1)
Antigua and Barbuda	1997	674·0 (638·3 to 708·2)	399·7 (370·8 to 428·5)	146·3 (129·7 to 164·5)	127·9 (115·3 to 143·1)	0·1 (0·1 to 0·1)	888·1 (841·1 to 933·2)	526·7 (488·5 to 564·6)	192·8 (170·9 to 216·8)	168·5 (151·9 to 188·5)	0·1 (0·1 to 0·1)
Antigua and Barbuda	1998	737·8 (702·0 to 774·4)	435·4 (404·7 to 466·2)	148·3 (132·2 to 167·1)	133·1 (120·2 to 148·0)	21·0 (21·0 to 21·0)	972·1 (925·0 to 1020·3)	573·7 (533·2 to 614·2)	195·4 (174·3 to 220·2)	175·4 (158·3 to 195·0)	27·7 (27·7 to 27·7)
Antigua and Barbuda	1999	723·7 (688·2 to 759·9)	419·3 (391·4 to 448·2)	145·9 (129·0 to 164·0)	131·1 (118·6 to 145·6)	27·4 (27·4 to 27·4)	953·5 (906·8 to 1001·3)	552·5 (515·8 to 590·6)	192·2 (170·0 to 216·1)	172·7 (156·3 to 191·9)	36·1 (36·1 to 36·1)
Antigua and Barbuda	2000	719·3 (685·9 to 756·9)	418·7 (390·6 to 448·4)	151·8 (135·4 to 170·8)	132·7 (120·8 to 145·3)	16·1 (16·1 to 16·1)	947·7 (903·8 to 997·3)	551·7 (514·7 to 590·9)	200·0 (178·3 to 225·0)	174·8 (159·2 to 191·5)	21·1 (21·1 to 21·1)
Antigua and Barbuda	2001	730·8 (698·3 to 766·6)	434·9 (405·4 to 465·4)	161·5 (145·6 to 180·3)	132·9 (124·6 to 142·1)	1·5 (1·5 to 1·5)	962·9 (920·0 to 1010·1)	573·0 (534·2 to 613·2)	212·8 (191·9 to 237·6)	175·1 (164·2 to 187·2)	2·0 (2·0 to 2·0)
Antigua and Barbuda	2002	756·7 (722·5 to 793·8)	450·4 (420·4 to 482·9)	170·5 (154·8 to 188·8)	135·7 (130·2 to 141·2)	0·0 (0·0 to 0·0)	997·0 (951·9 to 1045·9)	593·5 (553·9 to 636·2)	224·7 (203·9 to 248·8)	178·8 (171·6 to 186·0)	0·0 (0·0 to 0·0)
Antigua and Barbuda	2003	762·8 (726·9 to 798·8)	441·3 (411·4 to 472·5)	181·8 (165·6 to 200·1)	135·9 (128·5 to 143·9)	3·8 (3·8 to 3·8)	1005·0 (957·8 to 1052·6)	581·4 (542·1 to 622·5)	239·5 (218·1 to 263·6)	179·1 (169·3 to 189·6)	5·0 (5·0 to 5·0)
Antigua and Barbuda	2004	748·3 (714·2 to 781·9)	416·1 (388·7 to 444·3)	192·9 (175·7 to 210·3)	133·7 (126·5 to 141·6)	5·6 (5·6 to 5·6)	986·0 (941·0 to 1030·3)	548·3 (512·2 to 585·4)	254·1 (231·5 to 277·1)	176·2 (166·7 to 186·6)	7·3 (7·3 to 7·3)
Antigua and Barbuda	2005	744·6 (714·7 to 776·3)	402·1 (375·1 to 430·1)	210·6 (193·0 to 228·1)	131·9 (127·5 to 136·4)	0·0 (0·0 to 0·0)	981·1 (941·6 to 1022·9)	529·8 (494·3 to 566·7)	277·5 (254·3 to 300·6)	173·8 (168·0 to 179·7)	0·0 (0·0 to 0·0)
Antigua and Barbuda	2006	828·2 (794·2 to 864·5)	449·8 (419·2 to 482·0)	242·7 (224·8 to 262·0)	135·6 (131·3 to 140·0)	0·0 (0·0 to 0·0)	1091·2 (1046·5 to 1139·1)	592·7 (552·3 to 635·0)	319·8 (296·1 to 345·2)	178·7 (173·0 to 184·5)	0·0 (0·0 to 0·0)

Antigua and Barbuda	2007	817·2 (782·6 to 854·1)	432·9 (404·1 to 464·2)	251·4 (234·9 to 269·2)	132·9 (128·6 to 137·0)	0·0 (0·0 to 0·0)	1076·8 (1031·2 to 1125·4)	570·4 (532·5 to 611·6)	331·2 (309·5 to 354·6)	175·1 (169·5 to 180·6)	0·0 (0·0 to 0·0)
Antigua and Barbuda	2008	797·1 (764·0 to 833·4)	421·8 (393·4 to 452·5)	240·7 (224·3 to 258·9)	134·6 (130·2 to 139·5)	0·0 (0·0 to 0·0)	1050·3 (1006·7 to 1098·1)	555·8 (518·4 to 596·2)	317·2 (295·5 to 341·1)	177·4 (171·6 to 183·8)	0·0 (0·0 to 0·0)
Antigua and Barbuda	2009	872·9 (833·6 to 914·4)	492·1 (458·9 to 526·2)	230·9 (212·8 to 251·5)	146·2 (137·6 to 154·9)	3·7 (3·7 to 3·7)	1150·1 (1098·3 to 1204·8)	648·4 (604·7 to 693·3)	304·2 (280·4 to 331·3)	192·7 (181·3 to 204·2)	4·9 (4·9 to 4·9)
Antigua and Barbuda	2010	744·2 (711·2 to 780·2)	402·6 (376·2 to 431·0)	204·3 (187·4 to 222·7)	132·7 (122·8 to 142·7)	4·6 (4·6 to 4·6)	980·6 (937·1 to 1028·1)	530·5 (495·7 to 567·9)	269·2 (246·9 to 293·5)	174·9 (161·8 to 188·1)	6·0 (6·0 to 6·0)
Antigua and Barbuda	2011	808·3 (773·4 to 844·0)	450·0 (421·0 to 479·0)	211·0 (193·6 to 228·1)	140·2 (131·2 to 148·8)	7·0 (7·0 to 7·0)	1065·1 (1019·1 to 1112·1)	593·0 (554·8 to 631·2)	278·0 (255·1 to 300·5)	184·8 (172·8 to 196·1)	9·3 (9·3 to 9·3)
Antigua and Barbuda	2012	803·3 (770·8 to 838·8)	451·4 (423·8 to 480·8)	213·4 (196·9 to 230·3)	138·6 (133·9 to 143·5)	0·0 (0·0 to 0·0)	1058·5 (1015·7 to 1105·2)	594·8 (558·4 to 633·5)	281·1 (259·5 to 303·4)	182·6 (176·5 to 189·0)	0·0 (0·0 to 0·0)
Antigua and Barbuda	2013	850·3 (817·2 to 886·5)	488·7 (459·6 to 520·8)	220·0 (204·5 to 238·0)	141·6 (137·3 to 145·7)	0·0 (0·0 to 0·0)	1120·3 (1076·7 to 1168·1)	643·9 (605·6 to 686·2)	289·9 (269·4 to 313·6)	186·5 (181·0 to 191·9)	0·0 (0·0 to 0·0)
Antigua and Barbuda	2014	847·5 (814·9 to 881·5)	488·2 (459·4 to 517·9)	219·8 (204·7 to 237·5)	139·5 (135·1 to 143·7)	0·0 (0·0 to 0·0)	1116·7 (1073·7 to 1161·5)	643·3 (605·3 to 682·3)	289·7 (269·7 to 312·9)	183·8 (178·0 to 189·4)	0·0 (0·0 to 0·0)
Antigua and Barbuda	2015	896·7 (862·2 to 933·4)	524·9 (496·2 to 555·3)	228·2 (213·1 to 245·8)	143·5 (139·3 to 148·1)	0·0 (0·0 to 0·0)	1181·5 (1136·1 to 1229·8)	691·7 (653·8 to 731·7)	300·7 (280·8 to 323·9)	189·1 (183·5 to 195·2)	0·0 (0·0 to 0·0)
Antigua and Barbuda	2016	887·0 (854·7 to 922·6)	502·9 (474·1 to 532·0)	241·7 (225·8 to 258·3)	142·4 (137·6 to 147·1)	0·0 (0·0 to 0·0)	1168·7 (1126·2 to 1215·7)	662·6 (624·6 to 701·0)	318·5 (297·6 to 340·4)	187·6 (181·3 to 193·8)	0·0 (0·0 to 0·0)
Antigua and Barbuda	2017	897·1 (860·2 to 938·3)	493·6 (461·4 to 527·9)	261·0 (244·2 to 280·3)	142·5 (138·3 to 146·7)	0·0 (0·0 to 0·0)	1182·1 (1133·4 to 1236·3)	650·4 (607·9 to 695·6)	343·9 (321·8 to 369·3)	187·7 (182·3 to 193·3)	0·0 (0·0 to 0·0)
Antigua and Barbuda	2018	947·4 (901·3 to 996·6)	521·3 (479·0 to 565·9)	280·9 (259·0 to 306·1)	145·2 (140·0 to 150·6)	0·0 (0·0 to 0·0)	1248·4 (1187·5 to 1313·2)	686·9 (631·2 to 745·6)	370·2 (341·3 to 403·3)	191·3 (184·5 to 198·4)	0·0 (0·0 to 0·0)
Antigua and Barbuda	2019	972·5 (925·3 to 1023·6)	532·7 (489·2 to 579·1)	287·5 (265·1 to 313·6)	152·2 (146·8 to 158·1)	0·0 (0·0 to 0·0)	1281·3 (1219·1 to 1348·8)	701·9 (644·6 to 763·0)	378·8 (349·3 to 413·2)	200·6 (193·4 to 208·4)	0·0 (0·0 to 0·0)
Antigua and Barbuda	2020	1049·7 (994·7 to 1109·2)	638·7 (586·0 to 695·8)	266·2 (245·4 to 290·3)	144·8 (139·0 to 151·0)	0·0 (0·0 to 0·0)	1383·1 (1310·6 to 1461·5)	841·6 (772·1 to 916·8)	350·7 (323·4 to 382·6)	190·7 (183·1 to 199·0)	0·0 (0·0 to 0·0)

Antigua and Barbuda	2021	1027·5 (973·7 to 1085·8)	623·2 (571·7 to 678·9)	259·4 (239·1 to 282·8)	144·9 (138·2 to 152·0)	0·0 (0·0 to 0·0)	1353·8 (1282·9 to 1430·7)	821·2 (753·3 to 894·5)	341·8 (315·0 to 372·7)	190·9 (182·1 to 200·3)	0·0 (0·0 to 0·0)
Antigua and Barbuda	2022	904·1 (859·2 to 953·4)	492·0 (450·2 to 539·2)	261·9 (241·2 to 286·0)	150·2 (142·9 to 158·3)	0·0 (0·0 to 0·0)	1191·2 (1132·1 to 1256·2)	648·2 (593·2 to 710·5)	345·1 (317·8 to 376·9)	197·9 (188·3 to 208·5)	0·0 (0·0 to 0·0)
Antigua and Barbuda	2023	918·4 (871·7 to 969·6)	498·7 (455·1 to 545·7)	264·2 (243·1 to 288·4)	155·5 (147·3 to 165·4)	0·0 (0·0 to 0·0)	1210·1 (1148·5 to 1277·5)	657·1 (599·7 to 719·0)	348·1 (320·3 to 380·0)	204·9 (194·1 to 218·0)	0·0 (0·0 to 0·0)
Antigua and Barbuda	2024	932·3 (884·1 to 985·5)	505·3 (460·2 to 554·8)	266·3 (244·9 to 291·1)	160·7 (152·0 to 171·6)	0·0 (0·0 to 0·0)	1228·4 (1165·0 to 1298·5)	665·7 (606·4 to 731·0)	350·9 (322·7 to 383·5)	211·8 (200·2 to 226·1)	0·0 (0·0 to 0·0)
Antigua and Barbuda	2025	953·7 (899·3 to 1016·6)	519·8 (470·0 to 577·1)	268·4 (247·1 to 292·8)	165·6 (156·2 to 176·5)	0·0 (0·0 to 0·0)	1256·7 (1184·9 to 1339·5)	684·9 (619·3 to 760·4)	353·6 (325·6 to 385·7)	218·2 (205·8 to 232·6)	0·0 (0·0 to 0·0)
Antigua and Barbuda	2026	971·0 (907·9 to 1039·9)	531·2 (472·7 to 594·6)	270·0 (248·6 to 294·5)	169·9 (160·2 to 180·9)	0·0 (0·0 to 0·0)	1279·5 (1196·3 to 1370·2)	699·9 (622·8 to 783·4)	355·7 (327·6 to 388·0)	223·9 (211·1 to 238·3)	0·0 (0·0 to 0·0)
Antigua and Barbuda	2027	987·9 (916·7 to 1067·8)	542·4 (476·7 to 615·3)	271·4 (250·1 to 296·1)	174·2 (164·2 to 185·9)	0·0 (0·0 to 0·0)	1301·7 (1207·8 to 1406·9)	714·6 (628·1 to 810·7)	357·5 (329·6 to 390·2)	229·5 (216·4 to 244·9)	0·0 (0·0 to 0·0)
Antigua and Barbuda	2028	1005·3 (927·0 to 1095·3)	554·0 (478·6 to 637·3)	272·7 (251·5 to 297·9)	178·6 (167·9 to 190·9)	0·0 (0·0 to 0·0)	1324·7 (1221·5 to 1443·2)	730·0 (630·6 to 839·8)	359·4 (331·3 to 392·5)	235·3 (221·2 to 251·5)	0·0 (0·0 to 0·0)
Antigua and Barbuda	2029	1021·0 (937·7 to 1116·2)	564·5 (483·7 to 653·6)	273·8 (252·0 to 299·5)	182·7 (171·4 to 195·5)	0·0 (0·0 to 0·0)	1345·3 (1235·5 to 1470·7)	743·8 (637·4 to 861·2)	360·8 (332·0 to 394·7)	240·8 (225·9 to 257·5)	0·0 (0·0 to 0·0)
Antigua and Barbuda	2030	1036·5 (946·2 to 1136·6)	575·0 (485·9 to 671·1)	274·7 (252·8 to 301·0)	186·9 (174·8 to 200·3)	0·0 (0·0 to 0·0)	1365·7 (1246·7 to 1497·6)	757·6 (640·2 to 884·3)	361·9 (333·2 to 396·6)	246·2 (230·4 to 263·9)	0·0 (0·0 to 0·0)
Antigua and Barbuda	2031	1053·0 (958·6 to 1157·4)	586·2 (494·3 to 687·9)	275·7 (253·9 to 302·3)	191·2 (177·9 to 205·3)	0·0 (0·0 to 0·0)	1387·5 (1263·1 to 1525·1)	772·4 (651·3 to 906·3)	363·2 (334·5 to 398·3)	251·9 (234·4 to 270·5)	0·0 (0·0 to 0·0)
Antigua and Barbuda	2032	1070·2 (969·0 to 1181·5)	598·1 (497·5 to 703·7)	276·6 (254·8 to 303·5)	195·5 (181·2 to 211·2)	0·0 (0·0 to 0·0)	1410·1 (1276·7 to 1556·8)	788·1 (655·5 to 927·2)	364·4 (335·7 to 399·9)	257·6 (238·8 to 278·3)	0·0 (0·0 to 0·0)
Antigua and Barbuda	2033	1086·2 (979·9 to 1201·7)	609·3 (503·4 to 715·2)	277·1 (255·3 to 304·1)	199·8 (184·4 to 216·3)	0·0 (0·0 to 0·0)	1431·1 (1291·1 to 1583·4)	802·8 (663·3 to 942·3)	365·1 (336·3 to 400·7)	263·2 (243·0 to 284·9)	0·0 (0·0 to 0·0)
Antigua and Barbuda	2034	1103·0 (991·8 to 1224·3)	621·1 (507·8 to 733·5)	277·7 (255·6 to 304·8)	204·1 (187·6 to 222·2)	0·0 (0·0 to 0·0)	1453·3 (1306·8 to 1613·1)	818·4 (669·0 to 966·4)	365·9 (336·7 to 401·6)	269·0 (247·2 to 292·8)	0·0 (0·0 to 0·0)

Antigua and Barbuda	2035	1118·3 (1002·4 to 1242·7)	631·6 (517·5 to 751·1)	278·2 (255·9 to 305·3)	208·5 (190·6 to 228·9)	0·0 (0·0 to 0·0)	1473·5 (1320·7 to 1637·3)	832·2 (681·9 to 989·7)	366·5 (337·2 to 402·3)	274·8 (251·2 to 301·6)	0·0 (0·0 to 0·0)
Antigua and Barbuda	2036	1133·6 (1007·7 to 1262·3)	641·9 (517·8 to 767·2)	278·7 (256·1 to 305·9)	213·1 (193·9 to 235·1)	0·0 (0·0 to 0·0)	1493·7 (1327·8 to 1663·2)	845·7 (682·2 to 1010·8)	367·2 (337·5 to 403·0)	280·8 (255·5 to 309·8)	0·0 (0·0 to 0·0)
Antigua and Barbuda	2037	1148·2 (1016·0 to 1278·9)	651·3 (522·2 to 776·0)	279·1 (256·5 to 306·6)	217·8 (196·9 to 241·7)	0·0 (0·0 to 0·0)	1512·9 (1338·7 to 1685·0)	858·1 (688·1 to 1022·4)	367·8 (337·9 to 403·9)	287·0 (259·4 to 318·4)	0·0 (0·0 to 0·0)
Antigua and Barbuda	2038	1163·7 (1023·3 to 1298·4)	661·2 (524·2 to 788·0)	279·8 (256·9 to 307·2)	222·8 (199·8 to 249·3)	0·0 (0·0 to 0·0)	1533·3 (1348·4 to 1710·7)	871·2 (690·6 to 1038·3)	368·6 (338·5 to 404·7)	293·5 (263·3 to 328·5)	0·0 (0·0 to 0·0)
Antigua and Barbuda	2039	1180·2 (1034·3 to 1320·7)	671·8 (530·1 to 801·4)	280·5 (257·5 to 307·9)	227·9 (202·9 to 257·4)	0·0 (0·0 to 0·0)	1555·0 (1362·8 to 1740·2)	885·1 (698·5 to 1055·9)	369·6 (339·3 to 405·7)	300·3 (267·3 to 339·2)	0·0 (0·0 to 0·0)
Antigua and Barbuda	2040	1197·9 (1051·7 to 1348·1)	683·1 (538·4 to 825·4)	281·4 (258·1 to 309·0)	233·4 (206·2 to 265·4)	0·0 (0·0 to 0·0)	1578·4 (1385·7 to 1776·2)	900·1 (709·4 to 1087·5)	370·8 (340·1 to 407·2)	307·6 (271·7 to 349·6)	0·0 (0·0 to 0·0)
Antigua and Barbuda	2041	1214·7 (1065·8 to 1376·9)	693·5 (549·3 to 848·7)	282·2 (258·6 to 309·8)	239·0 (209·8 to 273·7)	0·0 (0·0 to 0·0)	1600·4 (1404·3 to 1814·2)	913·8 (723·7 to 1118·3)	371·8 (340·7 to 408·2)	314·9 (276·5 to 360·7)	0·0 (0·0 to 0·0)
Antigua and Barbuda	2042	1232·2 (1074·4 to 1396·2)	704·2 (549·3 to 860·3)	283·2 (259·4 to 310·7)	244·9 (213·0 to 282·4)	0·0 (0·0 to 0·0)	1623·6 (1415·6 to 1839·7)	927·8 (723·8 to 1133·5)	373·1 (341·8 to 409·4)	322·7 (280·7 to 372·1)	0·0 (0·0 to 0·0)
Antigua and Barbuda	2043	1249·3 (1082·9 to 1419·9)	714·1 (553·6 to 872·7)	284·2 (260·1 to 311·7)	251·0 (216·4 to 291·4)	0·0 (0·0 to 0·0)	1646·1 (1426·8 to 1870·8)	941·0 (729·4 to 1149·9)	374·4 (342·7 to 410·8)	330·7 (285·2 to 383·9)	0·0 (0·0 to 0·0)
Antigua and Barbuda	2044	1267·7 (1094·6 to 1446·5)	725·3 (551·0 to 896·6)	285·2 (260·9 to 312·8)	257·3 (220·3 to 300·8)	0·0 (0·0 to 0·0)	1670·4 (1442·3 to 1906·0)	955·6 (726·0 to 1181·3)	375·8 (343·7 to 412·2)	339·0 (290·3 to 396·4)	0·0 (0·0 to 0·0)
Antigua and Barbuda	2045	1286·7 (1105·6 to 1474·3)	736·8 (556·5 to 915·0)	286·2 (261·6 to 313·8)	263·7 (224·1 to 310·6)	0·0 (0·0 to 0·0)	1695·4 (1456·8 to 1942·6)	970·8 (733·3 to 1205·6)	377·1 (344·7 to 413·5)	347·5 (295·3 to 409·3)	0·0 (0·0 to 0·0)
Antigua and Barbuda	2046	1305·1 (1116·4 to 1504·2)	747·7 (563·6 to 941·6)	287·1 (262·3 to 314·8)	270·3 (227·9 to 320·6)	0·0 (0·0 to 0·0)	1719·6 (1471·0 to 1981·9)	985·2 (742·6 to 1240·6)	378·3 (345·6 to 414·8)	356·1 (300·2 to 422·4)	0·0 (0·0 to 0·0)
Antigua and Barbuda	2047	1323·4 (1130·6 to 1527·4)	758·3 (569·7 to 959·4)	288·0 (263·0 to 315·8)	277·0 (231·5 to 331·0)	0·0 (0·0 to 0·0)	1743·7 (1489·7 to 2012·6)	999·2 (750·6 to 1264·1)	379·5 (346·5 to 416·2)	365·0 (305·0 to 436·1)	0·0 (0·0 to 0·0)
Antigua and Barbuda	2048	1342·0 (1142·3 to 1552·4)	769·3 (575·9 to 971·9)	288·9 (263·6 to 317·0)	283·9 (235·2 to 342·2)	0·0 (0·0 to 0·0)	1768·3 (1505·1 to 2045·5)	1013·6 (758·8 to 1280·6)	380·7 (347·4 to 417·7)	374·0 (309·9 to 450·9)	0·0 (0·0 to 0·0)

Antigua and Barbuda	2049	1359·8 (1151·1 to 1573·9)	779·3 (573·1 to 985·2)	289·7 (264·3 to 318·2)	290·8 (238·9 to 353·5)	0·0 (0·0 to 0·0)	1791·7 (1516·8 to 2073·8)	1026·8 (755·1 to 1298·2)	381·7 (348·2 to 419·2)	383·2 (314·7 to 465·8)	0·0 (0·0 to 0·0)
Antigua and Barbuda	2050	1381·4 (1159·5 to 1608·0)	792·4 (578·5 to 1004·0)	290·8 (265·1 to 319·6)	298·2 (242·8 to 365·2)	0·0 (0·0 to 0·0)	1820·1 (1527·8 to 2118·8)	1044·1 (762·3 to 1322·9)	383·1 (349·3 to 421·0)	392·9 (319·9 to 481·1)	0·0 (0·0 to 0·0)
Australia	1995	2763·9 (2682·3 to 2845·0)	1971·3 (1900·1 to 2041·6)	470·6 (440·6 to 502·0)	322·1 (286·8 to 360·6)	0·0 (0·0 to 0·0)	2709·3 (2629·3 to 2788·8)	1932·3 (1862·6 to 2001·2)	461·3 (431·9 to 492·1)	315·7 (281·2 to 353·5)	0·0 (0·0 to 0·0)
Australia	1996	2880·4 (2815·8 to 2947·7)	2057·2 (2000·3 to 2115·0)	507·0 (483·3 to 531·2)	316·2 (287·2 to 345·4)	0·0 (0·0 to 0·0)	2823·4 (2760·1 to 2889·4)	2016·5 (1960·8 to 2073·2)	497·0 (473·8 to 520·7)	309·9 (281·6 to 338·6)	0·0 (0·0 to 0·0)
Australia	1997	2974·0 (2910·8 to 3041·1)	2137·2 (2081·9 to 2196·8)	552·7 (529·8 to 576·3)	284·1 (258·8 to 310·0)	0·0 (0·0 to 0·0)	2915·2 (2853·3 to 2981·0)	2095·0 (2040·7 to 2153·3)	541·8 (519·4 to 564·9)	278·5 (253·6 to 303·8)	0·0 (0·0 to 0·0)
Australia	1998	3149·8 (3084·5 to 3214·7)	2272·2 (2214·9 to 2328·1)	621·5 (597·9 to 644·5)	256·1 (232·9 to 281·1)	0·0 (0·0 to 0·0)	3087·6 (3023·5 to 3151·2)	2227·3 (2171·1 to 2282·1)	609·3 (586·1 to 631·8)	251·1 (228·3 to 275·5)	0·0 (0·0 to 0·0)
Australia	1999	3314·0 (3250·7 to 3383·8)	2402·6 (2347·5 to 2462·1)	669·2 (646·5 to 692·0)	242·2 (217·9 to 267·3)	0·0 (0·0 to 0·0)	3248·5 (3186·4 to 3316·9)	2355·1 (2301·1 to 2413·5)	656·0 (633·7 to 678·3)	237·4 (213·6 to 262·0)	0·0 (0·0 to 0·0)
Australia	2000	3384·5 (3319·2 to 3451·3)	2430·4 (2376·2 to 2483·8)	701·8 (682·4 to 721·8)	252·4 (229·3 to 276·0)	0·0 (0·0 to 0·0)	3317·6 (3253·6 to 3383·1)	2382·3 (2329·2 to 2434·7)	687·9 (668·9 to 707·5)	247·4 (224·7 to 270·5)	0·0 (0·0 to 0·0)
Australia	2001	3512·9 (3448·3 to 3582·1)	2510·6 (2453·7 to 2570·7)	717·8 (699·1 to 736·9)	284·5 (259·4 to 310·4)	0·0 (0·0 to 0·0)	3443·5 (3380·1 to 3511·3)	2461·0 (2405·2 to 2519·9)	703·6 (685·3 to 722·3)	278·9 (254·3 to 304·3)	0·0 (0·0 to 0·0)
Australia	2002	3639·3 (3574·7 to 3705·8)	2604·6 (2546·8 to 2663·4)	723·8 (706·1 to 743·0)	310·8 (285·8 to 337·1)	0·0 (0·0 to 0·0)	3567·3 (3504·0 to 3632·5)	2553·2 (2496·5 to 2610·8)	709·5 (692·1 to 728·3)	304·7 (280·1 to 330·4)	0·0 (0·0 to 0·0)
Australia	2003	3777·6 (3714·8 to 3842·3)	2701·4 (2644·6 to 2758·6)	731·6 (712·2 to 751·7)	344·6 (318·6 to 370·6)	0·0 (0·0 to 0·0)	3702·9 (3641·4 to 3766·4)	2648·0 (2592·3 to 2704·0)	717·2 (698·2 to 736·8)	337·8 (312·3 to 363·3)	0·0 (0·0 to 0·0)
Australia	2004	3924·6 (3859·3 to 3993·5)	2811·9 (2756·4 to 2869·7)	756·9 (737·4 to 776·2)	355·8 (330·2 to 382·2)	0·0 (0·0 to 0·0)	3847·0 (3783·0 to 3914·6)	2756·3 (2701·9 to 2813·0)	742·0 (722·8 to 760·9)	348·7 (323·6 to 374·7)	0·0 (0·0 to 0·0)
Australia	2005	3990·9 (3925·5 to 4060·1)	2864·1 (2807·0 to 2921·5)	778·7 (760·0 to 798·4)	348·2 (323·4 to 376·4)	0·0 (0·0 to 0·0)	3912·1 (3847·9 to 3979·9)	2807·5 (2751·5 to 2863·7)	763·3 (745·0 to 782·6)	341·3 (317·0 to 368·9)	0·0 (0·0 to 0·0)
Australia	2006	4066·2 (4000·0 to 4136·2)	2925·3 (2864·5 to 2987·6)	794·0 (776·2 to 813·5)	347·0 (320·4 to 372·9)	0·0 (0·0 to 0·0)	3985·9 (3920·9 to 4054·5)	2867·4 (2807·8 to 2928·5)	778·3 (760·8 to 797·4)	340·1 (314·0 to 365·6)	0·0 (0·0 to 0·0)

Australia	2007	4171·4 (4104·5 to 4238·8)	3022·6 (2961·5 to 3080·3)	801·6 (782·7 to 820·1)	347·2 (321·8 to 372·4)	0·0 (0·0 to 0·0)	4089·0 (4023·4 to 4155·0)	2962·8 (2903·0 to 3019·4)	785·8 (767·2 to 803·8)	340·4 (315·5 to 365·0)	0·0 (0·0 to 0·0)
Australia	2008	4274·6 (4207·5 to 4338·4)	3104·7 (3042·3 to 3163·7)	816·3 (795·5 to 837·6)	353·6 (329·2 to 378·8)	0·0 (0·0 to 0·0)	4190·1 (4124·3 to 4252·6)	3043·3 (2982·2 to 3101·2)	800·2 (779·8 to 821·0)	346·6 (322·7 to 371·3)	0·0 (0·0 to 0·0)
Australia	2009	4467·6 (4399·7 to 4533·6)	3261·7 (3200·6 to 3319·3)	852·2 (832·4 to 872·4)	353·7 (329·9 to 381·2)	0·0 (0·0 to 0·0)	4379·3 (4312·8 to 4444·0)	3197·2 (3137·3 to 3253·7)	835·3 (815·9 to 855·2)	346·7 (323·4 to 373·7)	0·0 (0·0 to 0·0)
Australia	2010	4488·6 (4415·9 to 4553·3)	3265·5 (3205·6 to 3326·4)	872·0 (851·2 to 892·2)	351·1 (328·0 to 378·3)	0·0 (0·0 to 0·0)	4399·9 (4328·7 to 4463·3)	3201·0 (3142·2 to 3260·7)	854·8 (834·3 to 874·6)	344·2 (321·5 to 370·8)	0·0 (0·0 to 0·0)
Australia	2011	4538·9 (4471·7 to 4602·4)	3285·8 (3225·8 to 3342·0)	884·8 (864·8 to 906·3)	368·3 (343·6 to 395·3)	0·0 (0·0 to 0·0)	4449·2 (4383·3 to 4511·4)	3220·8 (3162·0 to 3276·0)	867·3 (847·7 to 888·3)	361·1 (336·8 to 387·5)	0·0 (0·0 to 0·0)
Australia	2012	4664·7 (4599·6 to 4732·1)	3301·3 (3243·5 to 3358·4)	918·9 (898·5 to 938·1)	444·6 (419·9 to 472·8)	0·0 (0·0 to 0·0)	4572·5 (4508·7 to 4638·5)	3236·0 (3179·3 to 3292·0)	900·7 (880·7 to 919·6)	435·8 (411·6 to 463·5)	0·0 (0·0 to 0·0)
Australia	2013	4748·9 (4684·1 to 4817·8)	3242·6 (3188·9 to 3301·3)	939·3 (920·1 to 958·2)	567·1 (539·4 to 596·6)	0·0 (0·0 to 0·0)	4655·1 (4591·5 to 4722·6)	3178·5 (3125·8 to 3236·1)	920·7 (901·9 to 939·2)	555·9 (528·8 to 584·8)	0·0 (0·0 to 0·0)
Australia	2014	4921·0 (4852·7 to 4990·7)	3320·2 (3263·7 to 3381·4)	960·2 (939·1 to 981·3)	640·7 (611·5 to 668·8)	0·0 (0·0 to 0·0)	4823·8 (4756·8 to 4892·0)	3254·6 (3199·1 to 3314·5)	941·2 (920·6 to 961·9)	628·0 (599·4 to 655·5)	0·0 (0·0 to 0·0)
Australia	2015	5109·0 (5040·1 to 5179·2)	3469·5 (3414·2 to 3526·9)	968·9 (948·5 to 989·0)	670·6 (641·4 to 700·6)	0·0 (0·0 to 0·0)	5008·0 (4940·5 to 5076·8)	3400·9 (3346·7 to 3457·2)	949·7 (929·8 to 969·4)	657·4 (628·7 to 686·7)	0·0 (0·0 to 0·0)
Australia	2016	5225·3 (5154·8 to 5294·4)	3580·0 (3519·7 to 3637·5)	960·0 (940·0 to 979·8)	685·3 (655·7 to 715·1)	0·0 (0·0 to 0·0)	5122·0 (5052·9 to 5189·7)	3509·2 (3450·1 to 3565·6)	941·0 (921·4 to 960·4)	671·8 (642·7 to 701·0)	0·0 (0·0 to 0·0)
Australia	2017	5255·3 (5180·4 to 5324·7)	3619·4 (3558·0 to 3683·2)	944·3 (925·1 to 965·9)	691·6 (662·9 to 721·7)	0·0 (0·0 to 0·0)	5151·4 (5078·0 to 5219·5)	3547·8 (3487·7 to 3610·4)	925·6 (906·8 to 946·8)	678·0 (649·8 to 707·4)	0·0 (0·0 to 0·0)
Australia	2018	5359·8 (5269·2 to 5455·6)	3703·6 (3624·9 to 3787·2)	948·5 (921·6 to 975·9)	707·7 (668·4 to 747·7)	0·0 (0·0 to 0·0)	5253·8 (5165·1 to 5347·7)	3630·3 (3553·2 to 3712·3)	929·7 (903·4 to 956·6)	693·8 (655·2 to 732·9)	0·0 (0·0 to 0·0)
Australia	2019	5506·6 (5408·9 to 5608·4)	3843·2 (3758·8 to 3933·6)	943·7 (916·7 to 971·0)	719·7 (677·6 to 760·6)	0·0 (0·0 to 0·0)	5397·7 (5302·0 to 5497·6)	3767·2 (3684·5 to 3855·8)	925·0 (898·6 to 951·8)	705·5 (664·2 to 745·5)	0·0 (0·0 to 0·0)
Australia	2020	5784·7 (5671·3 to 5900·3)	4201·1 (4099·5 to 4300·8)	890·0 (864·7 to 915·7)	693·6 (652·2 to 734·5)	0·0 (0·0 to 0·0)	5670·4 (5559·2 to 5783·7)	4118·1 (4018·4 to 4215·8)	872·4 (847·6 to 897·6)	679·9 (639·3 to 720·0)	0·0 (0·0 to 0·0)

Australia	2021	5597·7 (5487·3 to 5711·6)	4056·9 (3958·7 to 4153·2)	857·7 (832·6 to 882·3)	683·2 (640·3 to 726·7)	0·0 (0·0 to 0·0)	5487·1 (5378·9 to 5598·7)	3976·7 (3880·4 to 4071·1)	840·8 (816·2 to 864·8)	669·7 (627·7 to 712·3)	0·0 (0·0 to 0·0)
Australia	2022	5166·9 (5041·1 to 5292·7)	3608·6 (3494·1 to 3717·4)	859·0 (834·3 to 883·5)	699·3 (653·2 to 744·9)	0·0 (0·0 to 0·0)	5064·7 (4941·5 to 5188·1)	3537·3 (3425·0 to 3643·9)	842·0 (817·8 to 866·1)	685·5 (640·3 to 730·2)	0·0 (0·0 to 0·0)
Australia	2023	5235·8 (5093·9 to 5376·0)	3659·7 (3523·1 to 3784·2)	860·4 (835·4 to 884·7)	715·8 (665·7 to 767·4)	0·0 (0·0 to 0·0)	5132·3 (4993·2 to 5269·7)	3587·3 (3453·5 to 3709·4)	843·4 (818·8 to 867·2)	701·6 (652·6 to 752·2)	0·0 (0·0 to 0·0)
Australia	2024	5297·6 (5151·7 to 5442·2)	3703·7 (3565·6 to 3836·3)	861·5 (836·4 to 886·4)	732·4 (676·6 to 790·5)	0·0 (0·0 to 0·0)	5192·9 (5049·9 to 5334·7)	3630·5 (3495·2 to 3760·5)	844·5 (819·9 to 868·9)	717·9 (663·3 to 774·8)	0·0 (0·0 to 0·0)
Australia	2025	5360·7 (5209·3 to 5510·9)	3748·6 (3605·6 to 3879·5)	862·7 (837·7 to 887·7)	749·4 (688·3 to 815·3)	0·0 (0·0 to 0·0)	5254·7 (5106·3 to 5402·0)	3674·5 (3534·3 to 3802·8)	845·7 (821·2 to 870·1)	734·6 (674·7 to 799·1)	0·0 (0·0 to 0·0)
Australia	2026	5428·2 (5269·8 to 5584·9)	3796·2 (3652·3 to 3939·8)	864·5 (839·2 to 889·9)	767·6 (705·8 to 833·3)	0·0 (0·0 to 0·0)	5320·9 (5165·6 to 5474·5)	3721·1 (3580·1 to 3861·9)	847·4 (822·6 to 872·3)	752·4 (691·8 to 816·8)	0·0 (0·0 to 0·0)
Australia	2027	5495·6 (5332·5 to 5660·5)	3840·8 (3697·1 to 3988·3)	866·8 (841·2 to 892·1)	788·0 (726·1 to 856·4)	0·0 (0·0 to 0·0)	5386·9 (5227·1 to 5548·6)	3764·8 (3624·0 to 3909·5)	849·7 (824·6 to 874·5)	772·4 (711·7 to 839·5)	0·0 (0·0 to 0·0)
Australia	2028	5563·9 (5390·7 to 5739·8)	3884·7 (3732·9 to 4052·0)	869·8 (844·1 to 895·3)	809·4 (745·5 to 879·8)	0·0 (0·0 to 0·0)	5453·9 (5284·2 to 5626·3)	3807·9 (3659·2 to 3971·9)	852·6 (827·5 to 877·6)	793·4 (730·8 to 862·4)	0·0 (0·0 to 0·0)
Australia	2029	5635·8 (5457·7 to 5821·4)	3931·1 (3769·8 to 4100·8)	873·2 (847·6 to 898·9)	831·5 (762·2 to 907·6)	0·0 (0·0 to 0·0)	5524·4 (5349·8 to 5706·3)	3853·4 (3695·3 to 4019·7)	856·0 (830·8 to 881·1)	815·1 (747·2 to 889·6)	0·0 (0·0 to 0·0)
Australia	2030	5712·8 (5520·3 to 5910·7)	3981·3 (3800·2 to 4164·0)	877·0 (851·0 to 902·5)	854·5 (779·6 to 936·0)	0·0 (0·0 to 0·0)	5599·8 (5411·1 to 5793·9)	3902·6 (3725·1 to 4081·7)	859·7 (834·2 to 884·7)	837·6 (764·2 to 917·5)	0·0 (0·0 to 0·0)
Australia	2031	5791·7 (5591·4 to 5999·5)	4032·5 (3850·4 to 4226·0)	881·0 (854·4 to 906·8)	878·2 (797·1 to 966·1)	0·0 (0·0 to 0·0)	5677·2 (5480·9 to 5880·9)	3952·8 (3774·3 to 4142·4)	863·6 (837·5 to 888·9)	860·8 (781·3 to 947·0)	0·0 (0·0 to 0·0)
Australia	2032	5881·6 (5671·2 to 6104·2)	4094·3 (3897·9 to 4294·5)	884·9 (858·2 to 910·8)	902·4 (815·5 to 996·2)	0·0 (0·0 to 0·0)	5765·4 (5559·1 to 5983·5)	4013·4 (3820·8 to 4209·6)	867·4 (841·2 to 892·8)	884·6 (799·4 to 976·6)	0·0 (0·0 to 0·0)
Australia	2033	5969·3 (5746·7 to 6201·4)	4154·2 (3941·3 to 4361·1)	888·3 (861·8 to 914·2)	926·9 (834·4 to 1026·2)	0·0 (0·0 to 0·0)	5851·3 (5633·1 to 6078·8)	4072·0 (3863·4 to 4274·9)	870·7 (844·7 to 896·1)	908·6 (817·9 to 1005·9)	0·0 (0·0 to 0·0)
Australia	2034	6055·1 (5825·0 to 6303·3)	4212·1 (3984·9 to 4433·0)	891·2 (864·3 to 917·3)	951·8 (853·3 to 1056·7)	0·0 (0·0 to 0·0)	5935·4 (5709·8 to 6178·7)	4128·8 (3906·1 to 4345·4)	873·6 (847·3 to 899·2)	933·0 (836·4 to 1035·8)	0·0 (0·0 to 0·0)

Australia	2035	6143·9 (5896·9 to 6408·8)	4271·8 (4029·0 to 4504·0)	894·5 (867·9 to 920·8)	977·6 (870·7 to 1089·9)	0·0 (0·0 to 0·0)	6022·4 (5780·4 to 6282·2)	4187·4 (3949·3 to 4415·0)	876·8 (850·7 to 902·6)	958·2 (853·4 to 1068·3)	0·0 (0·0 to 0·0)
Australia	2036	6233·5 (5974·4 to 6513·5)	4332·0 (4078·0 to 4579·2)	897·8 (871·6 to 924·4)	1003·7 (887·6 to 1124·8)	0·0 (0·0 to 0·0)	6110·3 (5856·3 to 6384·7)	4246·4 (3997·4 to 4488·7)	880·0 (854·3 to 906·1)	983·9 (870·0 to 1102·5)	0·0 (0·0 to 0·0)
Australia	2037	6330·2 (6055·0 to 6632·1)	4397·1 (4130·9 to 4659·3)	901·9 (875·4 to 928·6)	1031·2 (903·6 to 1162·4)	0·0 (0·0 to 0·0)	6205·1 (5935·3 to 6501·0)	4310·2 (4049·2 to 4567·2)	884·1 (858·1 to 910·3)	1010·8 (885·7 to 1139·4)	0·0 (0·0 to 0·0)
Australia	2038	6435·1 (6143·0 to 6756·7)	4467·9 (4188·0 to 4749·6)	907·1 (880·4 to 934·4)	1060·1 (923·8 to 1203·8)	0·0 (0·0 to 0·0)	6307·9 (6021·5 to 6623·1)	4379·6 (4105·2 to 4655·7)	889·2 (863·0 to 915·9)	1039·1 (905·6 to 1180·0)	0·0 (0·0 to 0·0)
Australia	2039	6546·2 (6234·7 to 6886·9)	4542·9 (4249·4 to 4845·2)	913·1 (886·2 to 941·0)	1090·3 (941·3 to 1245·0)	0·0 (0·0 to 0·0)	6416·8 (6111·4 to 6750·8)	4453·1 (4165·4 to 4749·4)	895·0 (868·7 to 922·4)	1068·7 (922·7 to 1220·4)	0·0 (0·0 to 0·0)
Australia	2040	6658·3 (6329·1 to 7020·5)	4618·3 (4312·2 to 4938·9)	919·0 (891·9 to 947·4)	1121·0 (958·9 to 1287·9)	0·0 (0·0 to 0·0)	6526·7 (6204·0 to 6881·7)	4527·0 (4227·0 to 4841·3)	900·9 (874·2 to 928·7)	1098·9 (939·9 to 1262·4)	0·0 (0·0 to 0·0)
Australia	2041	6772·7 (6425·5 to 7157·2)	4694·9 (4374·9 to 5034·1)	925·1 (897·6 to 953·9)	1152·7 (978·3 to 1335·5)	0·0 (0·0 to 0·0)	6638·8 (6298·5 to 7015·8)	4602·0 (4288·5 to 4934·6)	906·8 (879·9 to 935·0)	1129·9 (958·9 to 1309·1)	0·0 (0·0 to 0·0)
Australia	2042	6887·7 (6520·4 to 7298·8)	4771·3 (4438·7 to 5124·1)	931·2 (903·3 to 960·3)	1185·2 (994·5 to 1382·8)	0·0 (0·0 to 0·0)	6751·5 (6391·5 to 7154·5)	4677·0 (4351·0 to 5022·8)	912·8 (885·4 to 941·3)	1161·7 (974·9 to 1355·4)	0·0 (0·0 to 0·0)
Australia	2043	7002·6 (6618·2 to 7435·0)	4847·1 (4502·3 to 5215·3)	937·1 (908·7 to 966·7)	1218·4 (1014·9 to 1430·0)	0·0 (0·0 to 0·0)	6864·2 (6487·4 to 7288·0)	4751·3 (4413·3 to 5112·2)	918·5 (890·8 to 947·6)	1194·3 (994·8 to 1401·7)	0·0 (0·0 to 0·0)
Australia	2044	7120·0 (6712·5 to 7587·5)	4924·2 (4567·6 to 5308·1)	943·2 (914·1 to 973·0)	1252·7 (1032·9 to 1481·3)	0·0 (0·0 to 0·0)	6979·3 (6579·8 to 7437·5)	4826·8 (4477·3 to 5203·1)	924·5 (896·0 to 953·7)	1227·9 (1012·4 to 1452·0)	0·0 (0·0 to 0·0)
Australia	2045	7238·3 (6812·7 to 7724·2)	5001·2 (4633·1 to 5396·2)	949·2 (919·5 to 979·6)	1287·9 (1051·1 to 1531·3)	0·0 (0·0 to 0·0)	7095·3 (6678·0 to 7571·5)	4902·4 (4541·5 to 5289·6)	930·5 (901·3 to 960·2)	1262·4 (1030·3 to 1501·1)	0·0 (0·0 to 0·0)
Australia	2046	7356·9 (6912·9 to 7868·2)	5077·7 (4699·5 to 5484·2)	955·2 (924·7 to 986·2)	1324·0 (1073·5 to 1585·8)	0·0 (0·0 to 0·0)	7211·5 (6776·2 to 7712·7)	4977·4 (4606·6 to 5375·8)	936·3 (906·5 to 966·7)	1297·8 (1052·3 to 1554·5)	0·0 (0·0 to 0·0)
Australia	2047	7473·6 (7011·0 to 7999·6)	5152·1 (4763·8 to 5570·5)	960·8 (929·9 to 992·1)	1360·7 (1095·5 to 1644·9)	0·0 (0·0 to 0·0)	7325·8 (6872·4 to 7841·5)	5050·3 (4669·6 to 5460·4)	941·8 (911·5 to 972·4)	1333·8 (1073·8 to 1612·4)	0·0 (0·0 to 0·0)
Australia	2048	7590·1 (7109·6 to 8136·0)	5225·3 (4827·5 to 5655·1)	966·2 (935·0 to 997·8)	1398·6 (1116·0 to 1706·2)	0·0 (0·0 to 0·0)	7440·1 (6969·1 to 7975·2)	5122·0 (4732·1 to 5543·3)	947·1 (916·5 to 978·1)	1370·9 (1093·9 to 1672·5)	0·0 (0·0 to 0·0)

Australia	2049	7704·5 (7204·3 to 8266·5)	5296·1 (4889·9 to 5730·8)	971·2 (939·3 to 1003·0)	1437·2 (1136·6 to 1769·0)	0·0 (0·0 to 0·0)	7552·2 (7061·9 to 8103·1)	5191·4 (4793·2 to 5617·5)	952·0 (920·7 to 983·2)	1408·8 (1114·1 to 1734·0)	0·0 (0·0 to 0·0)
Australia	2050	7817·6 (7303·2 to 8401·7)	5365·0 (4952·0 to 5804·2)	976·0 (943·3 to 1008·0)	1476·5 (1157·5 to 1831·6)	0·0 (0·0 to 0·0)	7663·0 (7158·8 to 8235·6)	5259·0 (4854·2 to 5689·5)	956·7 (924·7 to 988·0)	1447·4 (1134·6 to 1795·4)	0·0 (0·0 to 0·0)
Austria	1995	3604·3 (3531·2 to 3678·1)	2496·6 (2435·2 to 2561·6)	603·8 (575·4 to 633·4)	503·8 (475·4 to 530·6)	0·0 (0·0 to 0·0)	4105·3 (4022·1 to 4189·5)	2843·7 (2773·7 to 2917·7)	687·7 (655·4 to 721·5)	573·9 (541·5 to 604·3)	0·0 (0·0 to 0·0)
Austria	1996	3663·1 (3605·4 to 3718·1)	2568·2 (2518·0 to 2616·5)	623·7 (601·0 to 644·2)	471·1 (450·3 to 492·8)	0·0 (0·0 to 0·0)	4172·3 (4106·7 to 4235·0)	2925·2 (2868·0 to 2980·2)	710·4 (684·5 to 733·8)	536·6 (512·9 to 561·3)	0·0 (0·0 to 0·0)
Austria	1997	3659·7 (3604·8 to 3713·9)	2634·5 (2585·7 to 2682·4)	642·4 (619·7 to 663·7)	382·8 (364·4 to 402·4)	0·0 (0·0 to 0·0)	4168·4 (4105·9 to 4230·2)	3000·7 (2945·1 to 3055·3)	731·7 (705·8 to 756·0)	436·0 (415·0 to 458·4)	0·0 (0·0 to 0·0)
Austria	1998	3843·5 (3786·2 to 3901·0)	2822·7 (2768·9 to 2875·1)	672·5 (649·7 to 694·7)	348·3 (329·3 to 369·0)	0·0 (0·0 to 0·0)	4377·9 (4312·6 to 4443·3)	3215·1 (3153·9 to 3274·8)	766·0 (740·1 to 791·3)	396·7 (375·1 to 420·3)	0·0 (0·0 to 0·0)
Austria	1999	3902·6 (3846·4 to 3962·3)	2885·4 (2833·2 to 2937·0)	689·3 (668·9 to 708·8)	328·0 (312·1 to 345·5)	0·0 (0·0 to 0·0)	4445·1 (4381·1 to 4513·2)	3286·5 (3227·1 to 3345·3)	785·1 (761·9 to 807·3)	373·6 (355·5 to 393·6)	0·0 (0·0 to 0·0)
Austria	2000	4000·1 (3946·2 to 4055·0)	2965·6 (2917·5 to 3019·8)	714·6 (698·7 to 731·3)	319·9 (306·7 to 332·6)	0·0 (0·0 to 0·0)	4556·1 (4494·8 to 4618·7)	3377·9 (3323·1 to 3439·6)	814·0 (795·8 to 832·9)	364·3 (349·3 to 378·8)	0·0 (0·0 to 0·0)
Austria	2001	4054·0 (4004·0 to 4106·3)	2991·8 (2944·4 to 3039·9)	742·9 (727·9 to 757·8)	319·3 (308·0 to 330·1)	0·0 (0·0 to 0·0)	4617·5 (4560·6 to 4677·2)	3407·7 (3353·7 to 3462·5)	846·2 (829·1 to 863·2)	363·6 (350·9 to 376·0)	0·0 (0·0 to 0·0)
Austria	2002	4164·3 (4109·8 to 4218·1)	3059·3 (3009·9 to 3108·1)	775·2 (760·2 to 790·8)	329·8 (317·5 to 341·0)	0·0 (0·0 to 0·0)	4743·2 (4681·2 to 4804·5)	3484·6 (3428·3 to 3540·1)	883·0 (865·9 to 900·8)	375·6 (361·6 to 388·5)	0·0 (0·0 to 0·0)
Austria	2003	4194·7 (4140·3 to 4251·8)	3051·4 (3001·7 to 3101·4)	801·2 (785·8 to 816·2)	342·1 (331·5 to 353·2)	0·0 (0·0 to 0·0)	4777·9 (4715·9 to 4842·9)	3475·6 (3419·0 to 3532·5)	912·6 (895·1 to 929·7)	389·7 (377·6 to 402·3)	0·0 (0·0 to 0·0)
Austria	2004	4420·1 (4367·6 to 4475·8)	3205·3 (3157·7 to 3254·7)	843·8 (827·5 to 859·1)	370·9 (359·6 to 383·3)	0·0 (0·0 to 0·0)	5034·5 (4974·8 to 5098·0)	3650·9 (3596·7 to 3707·1)	961·1 (942·5 to 978·5)	422·5 (409·5 to 436·6)	0·0 (0·0 to 0·0)
Austria	2005	4418·8 (4366·3 to 4470·0)	3200·9 (3154·6 to 3245·3)	848·8 (831·7 to 864·3)	369·1 (356·9 to 380·6)	0·0 (0·0 to 0·0)	5033·2 (4973·3 to 5091·4)	3645·9 (3593·2 to 3696·4)	966·8 (947·3 to 984·4)	420·4 (406·5 to 433·5)	0·0 (0·0 to 0·0)
Austria	2006	4531·5 (4477·3 to 4584·0)	3299·3 (3248·4 to 3348·0)	856·2 (840·8 to 871·9)	376·0 (364·3 to 388·3)	0·0 (0·0 to 0·0)	5161·5 (5099·8 to 5221·3)	3757·9 (3700·0 to 3813·4)	975·2 (957·7 to 993·1)	428·3 (414·9 to 442·3)	0·0 (0·0 to 0·0)

Austria	2007	4664·4 (4611·5 to 4719·0)	3408·1 (3358·4 to 3458·0)	870·4 (854·0 to 886·6)	385·9 (373·9 to 397·4)	0·0 (0·0 to 0·0)	5312·8 (5252·6 to 5375·0)	3881·8 (3825·3 to 3938·7)	991·4 (972·7 to 1009·8)	439·5 (425·8 to 452·6)	0·0 (0·0 to 0·0)
Austria	2008	4789·4 (4734·5 to 4842·7)	3514·3 (3464·3 to 3563·9)	877·7 (861·9 to 893·0)	397·4 (385·9 to 409·0)	0·0 (0·0 to 0·0)	5455·3 (5392·7 to 5516·0)	4002·8 (3946·0 to 4059·4)	999·8 (981·7 to 1017·1)	452·7 (439·6 to 465·9)	0·0 (0·0 to 0·0)
Austria	2009	4901·9 (4845·8 to 4956·4)	3579·6 (3528·3 to 3631·4)	887·9 (871·0 to 904·1)	434·4 (421·7 to 446·5)	0·0 (0·0 to 0·0)	5583·4 (5519·4 to 5645·4)	4077·2 (4018·8 to 4136·2)	1011·3 (992·0 to 1029·8)	494·8 (480·3 to 508·6)	0·0 (0·0 to 0·0)
Austria	2010	4929·0 (4872·4 to 4984·9)	3575·4 (3524·3 to 3628·5)	911·1 (895·3 to 927·0)	442·5 (430·3 to 454·2)	0·0 (0·0 to 0·0)	5614·2 (5549·8 to 5677·9)	4072·4 (4014·3 to 4132·9)	1037·7 (1019·8 to 1055·9)	504·0 (490·1 to 517·4)	0·0 (0·0 to 0·0)
Austria	2011	4959·6 (4908·0 to 5012·3)	3600·3 (3550·6 to 3649·1)	929·8 (913·5 to 946·0)	429·5 (417·1 to 441·7)	0·0 (0·0 to 0·0)	5649·1 (5590·4 to 5709·1)	4100·8 (4044·2 to 4156·4)	1059·1 (1040·5 to 1077·5)	489·2 (475·1 to 503·1)	0·0 (0·0 to 0·0)
Austria	2012	5059·9 (5005·9 to 5116·1)	3677·6 (3628·7 to 3728·8)	951·2 (935·3 to 966·9)	431·0 (419·7 to 442·9)	0·0 (0·0 to 0·0)	5763·3 (5701·8 to 5827·3)	4188·9 (4133·2 to 4247·2)	1083·5 (1065·4 to 1101·3)	490·9 (478·0 to 504·5)	0·0 (0·0 to 0·0)
Austria	2013	5072·2 (5021·6 to 5123·7)	3671·2 (3623·9 to 3717·5)	967·0 (950·7 to 983·8)	434·0 (422·8 to 446·4)	0·0 (0·0 to 0·0)	5777·4 (5719·7 to 5836·0)	4181·6 (4127·7 to 4234·4)	1101·5 (1082·9 to 1120·6)	494·3 (481·5 to 508·4)	0·0 (0·0 to 0·0)
Austria	2014	5104·1 (5052·6 to 5160·7)	3698·7 (3653·0 to 3749·3)	974·5 (958·6 to 990·1)	430·9 (419·2 to 443·5)	0·0 (0·0 to 0·0)	5813·7 (5755·0 to 5878·2)	4212·9 (4160·9 to 4270·6)	1110·0 (1091·9 to 1127·8)	490·8 (477·5 to 505·2)	0·0 (0·0 to 0·0)
Austria	2015	5103·6 (5047·6 to 5155·8)	3695·3 (3642·9 to 3743·2)	978·1 (962·1 to 995·2)	430·2 (417·7 to 443·0)	0·0 (0·0 to 0·0)	5813·1 (5749·3 to 5872·5)	4209·1 (4149·4 to 4263·6)	1114·1 (1095·9 to 1133·6)	490·0 (475·8 to 504·5)	0·0 (0·0 to 0·0)
Austria	2016	5160·1 (5106·3 to 5214·7)	3733·4 (3682·1 to 3785·8)	992·6 (976·4 to 1009·0)	434·2 (422·2 to 446·2)	0·0 (0·0 to 0·0)	5877·5 (5816·1 to 5939·7)	4252·4 (4193·9 to 4312·1)	1130·6 (1112·2 to 1149·2)	494·5 (480·9 to 508·3)	0·0 (0·0 to 0·0)
Austria	2017	5234·1 (5178·6 to 5290·0)	3792·9 (3743·1 to 3846·0)	997·2 (978·9 to 1014·2)	444·0 (432·7 to 455·5)	0·0 (0·0 to 0·0)	5961·7 (5898·6 to 6025·4)	4320·2 (4263·5 to 4380·7)	1135·8 (1114·9 to 1155·2)	505·8 (492·9 to 518·8)	0·0 (0·0 to 0·0)
Austria	2018	5324·6 (5253·3 to 5393·0)	3888·6 (3826·8 to 3951·2)	981·2 (962·4 to 1001·0)	454·8 (438·0 to 471·2)	0·0 (0·0 to 0·0)	6064·9 (5983·6 to 6142·7)	4429·2 (4358·8 to 4500·5)	1117·6 (1096·2 to 1140·1)	518·1 (498·8 to 536·7)	0·0 (0·0 to 0·0)
Austria	2019	5383·2 (5301·9 to 5462·8)	3926·5 (3851·7 to 4002·4)	987·1 (968·1 to 1007·3)	469·6 (451·4 to 486·8)	0·0 (0·0 to 0·0)	6131·6 (6038·9 to 6222·2)	4472·4 (4387·2 to 4558·9)	1124·4 (1102·7 to 1147·3)	534·8 (514·2 to 554·5)	0·0 (0·0 to 0·0)
Austria	2020	5865·9 (5760·3 to 5966·3)	4476·6 (4372·9 to 4576·1)	933·9 (915·8 to 953·3)	455·4 (436·5 to 471·8)	0·0 (0·0 to 0·0)	6681·4 (6561·1 to 6795·7)	5098·9 (4980·8 to 5212·3)	1063·7 (1043·1 to 1085·8)	518·7 (497·2 to 537·4)	0·0 (0·0 to 0·0)

Austria	2021	5721·0 (5617·6 to 5818·1)	4360·6 (4259·6 to 4457·5)	907·4 (889·9 to 926·3)	452·9 (433·6 to 470·8)	0·0 (0·0 to 0·0)	6516·3 (6398·5 to 6626·9)	4966·9 (4851·8 to 5077·2)	1033·5 (1013·6 to 1055·0)	515·9 (493·8 to 536·2)	0·0 (0·0 to 0·0)
Austria	2022	5014·0 (4899·5 to 5121·4)	3641·9 (3532·8 to 3748·5)	908·1 (890·6 to 927·3)	464·0 (443·8 to 483·4)	0·0 (0·0 to 0·0)	5711·0 (5580·7 to 5833·4)	4148·2 (4024·0 to 4269·7)	1034·3 (1014·4 to 1056·3)	528·5 (505·5 to 550·6)	0·0 (0·0 to 0·0)
Austria	2023	5053·4 (4922·8 to 5177·9)	3669·4 (3545·5 to 3791·2)	907·9 (890·3 to 927·2)	476·0 (454·3 to 495·8)	0·0 (0·0 to 0·0)	5755·9 (5607·1 to 5897·8)	4179·5 (4038·4 to 4318·3)	1034·2 (1014·1 to 1056·1)	542·2 (517·4 to 564·8)	0·0 (0·0 to 0·0)
Austria	2024	5097·0 (4951·4 to 5241·4)	3701·2 (3563·8 to 3847·5)	907·3 (889·6 to 926·7)	488·5 (462·9 to 511·5)	0·0 (0·0 to 0·0)	5805·6 (5639·8 to 5970·1)	4215·8 (4059·3 to 4382·4)	1033·4 (1013·2 to 1055·6)	556·4 (527·2 to 582·6)	0·0 (0·0 to 0·0)
Austria	2025	5140·4 (4988·7 to 5289·8)	3732·6 (3587·6 to 3881·3)	906·9 (889·2 to 926·8)	500·9 (472·5 to 528·1)	0·0 (0·0 to 0·0)	5855·1 (5682·2 to 6025·1)	4251·5 (4086·4 to 4420·8)	1033·0 (1012·9 to 1055·6)	570·6 (538·1 to 601·5)	0·0 (0·0 to 0·0)
Austria	2026	5183·5 (5025·3 to 5341·3)	3763·3 (3614·4 to 3911·0)	906·5 (889·0 to 925·7)	513·7 (480·7 to 545·2)	0·0 (0·0 to 0·0)	5904·1 (5723·9 to 6083·8)	4286·5 (4116·9 to 4454·8)	1032·5 (1012·6 to 1054·4)	585·1 (547·5 to 621·0)	0·0 (0·0 to 0·0)
Austria	2027	5228·7 (5066·6 to 5396·8)	3796·0 (3646·3 to 3957·0)	905·9 (888·2 to 925·4)	526·8 (488·6 to 563·2)	0·0 (0·0 to 0·0)	5955·6 (5770·9 to 6147·0)	4323·8 (4153·2 to 4507·1)	1031·8 (1011·7 to 1054·0)	600·0 (556·5 to 641·5)	0·0 (0·0 to 0·0)
Austria	2028	5269·7 (5097·6 to 5448·1)	3824·9 (3668·6 to 3991·8)	904·8 (887·2 to 924·7)	540·0 (496·5 to 584·3)	0·0 (0·0 to 0·0)	6002·3 (5806·3 to 6205·6)	4356·6 (4178·7 to 4546·8)	1030·6 (1010·6 to 1053·2)	615·1 (565·5 to 665·6)	0·0 (0·0 to 0·0)
Austria	2029	5309·1 (5127·1 to 5502·3)	3851·8 (3686·4 to 4029·2)	903·6 (886·0 to 923·4)	553·7 (505·0 to 605·2)	0·0 (0·0 to 0·0)	6047·1 (5839·9 to 6267·2)	4387·3 (4198·8 to 4589·3)	1029·2 (1009·2 to 1051·8)	630·7 (575·2 to 689·3)	0·0 (0·0 to 0·0)
Austria	2030	5346·1 (5150·7 to 5556·1)	3876·7 (3697·5 to 4073·4)	901·9 (883·7 to 922·1)	567·5 (512·0 to 626·8)	0·0 (0·0 to 0·0)	6089·3 (5866·8 to 6328·6)	4415·7 (4211·6 to 4639·7)	1027·3 (1006·6 to 1050·3)	646·3 (583·2 to 714·0)	0·0 (0·0 to 0·0)
Austria	2031	5387·0 (5177·8 to 5614·5)	3904·6 (3709·9 to 4113·2)	900·7 (882·3 to 921·0)	581·7 (519·6 to 645·2)	0·0 (0·0 to 0·0)	6135·9 (5897·7 to 6395·1)	4447·4 (4225·6 to 4685·1)	1025·9 (1004·9 to 1049·1)	662·6 (591·8 to 734·9)	0·0 (0·0 to 0·0)
Austria	2032	5437·6 (5207·6 to 5685·4)	3940·8 (3725·0 to 4176·5)	900·3 (881·4 to 920·5)	596·6 (529·9 to 666·0)	0·0 (0·0 to 0·0)	6193·6 (5931·5 to 6475·8)	4488·6 (4242·8 to 4757·2)	1025·4 (1004·0 to 1048·5)	679·5 (603·6 to 758·6)	0·0 (0·0 to 0·0)
Austria	2033	5488·3 (5240·3 to 5752·6)	3976·3 (3741·3 to 4231·0)	900·2 (880·1 to 921·0)	611·9 (537·0 to 685·1)	0·0 (0·0 to 0·0)	6251·3 (5968·9 to 6552·4)	4529·1 (4261·4 to 4819·2)	1025·3 (1002·5 to 1049·1)	696·9 (611·7 to 780·3)	0·0 (0·0 to 0·0)
Austria	2034	5541·8 (5272·6 to 5830·0)	4013·8 (3759·8 to 4285·6)	900·4 (879·5 to 921·6)	627·6 (546·9 to 707·4)	0·0 (0·0 to 0·0)	6312·2 (6005·6 to 6640·5)	4571·8 (4282·5 to 4881·4)	1025·5 (1001·8 to 1049·8)	714·9 (622·9 to 805·8)	0·0 (0·0 to 0·0)

Austria	2035	5598·0 (5307·2 to 5912·5)	4052·9 (3785·9 to 4349·1)	901·1 (879·6 to 923·1)	644·0 (557·8 to 732·2)	0·0 (0·0 to 0·0)	6376·2 (6045·1 to 6734·4)	4616·4 (4312·2 to 4953·7)	1026·3 (1001·9 to 1051·4)	733·5 (635·4 to 834·0)	0·0 (0·0 to 0·0)
Austria	2036	5660·5 (5350·0 to 5993·0)	4096·5 (3808·2 to 4412·5)	902·8 (881·1 to 925·5)	661·3 (569·9 to 755·1)	0·0 (0·0 to 0·0)	6447·5 (6093·8 to 6826·1)	4666·0 (4337·6 to 5025·9)	1028·3 (1003·6 to 1054·1)	753·2 (649·1 to 860·1)	0·0 (0·0 to 0·0)
Austria	2037	5729·1 (5396·8 to 6080·0)	4144·3 (3836·6 to 4478·0)	905·5 (883·7 to 928·7)	679·3 (582·0 to 779·7)	0·0 (0·0 to 0·0)	6525·6 (6147·1 to 6925·2)	4720·5 (4370·0 to 5100·5)	1031·4 (1006·5 to 1057·8)	773·7 (662·9 to 888·1)	0·0 (0·0 to 0·0)
Austria	2038	5802·2 (5452·7 to 6168·2)	4195·2 (3867·4 to 4546·3)	909·0 (886·4 to 932·6)	698·0 (593·6 to 805·7)	0·0 (0·0 to 0·0)	6608·8 (6210·7 to 7025·7)	4778·4 (4405·0 to 5178·4)	1035·4 (1009·7 to 1062·2)	795·0 (676·1 to 917·7)	0·0 (0·0 to 0·0)
Austria	2039	5880·9 (5513·6 to 6268·9)	4249·7 (3901·7 to 4617·4)	913·6 (890·8 to 937·4)	717·6 (605·3 to 830·4)	0·0 (0·0 to 0·0)	6698·5 (6280·1 to 7140·5)	4840·5 (4444·2 to 5259·3)	1040·6 (1014·6 to 1067·7)	817·4 (689·4 to 945·8)	0·0 (0·0 to 0·0)
Austria	2040	5960·7 (5581·0 to 6362·7)	4304·5 (3944·3 to 4685·3)	918·4 (894·9 to 942·5)	737·8 (616·8 to 859·0)	0·0 (0·0 to 0·0)	6789·4 (6356·9 to 7247·2)	4902·9 (4492·7 to 5336·6)	1046·1 (1019·3 to 1073·5)	840·3 (702·5 to 978·4)	0·0 (0·0 to 0·0)
Austria	2041	6042·6 (5645·9 to 6458·4)	4360·4 (3988·6 to 4756·7)	923·6 (899·7 to 947·9)	758·6 (628·6 to 888·5)	0·0 (0·0 to 0·0)	6882·6 (6430·8 to 7356·2)	4966·5 (4543·2 to 5418·0)	1052·0 (1024·8 to 1079·7)	864·1 (716·0 to 1012·0)	0·0 (0·0 to 0·0)
Austria	2042	6126·4 (5712·2 to 6550·3)	4417·1 (4032·6 to 4822·2)	929·0 (904·5 to 953·6)	780·3 (642·5 to 917·8)	0·0 (0·0 to 0·0)	6978·0 (6506·4 to 7460·9)	5031·2 (4593·3 to 5492·6)	1058·2 (1030·2 to 1086·1)	888·7 (731·8 to 1045·4)	0·0 (0·0 to 0·0)
Austria	2043	6207·2 (5780·1 to 6646·5)	4470·9 (4074·0 to 4885·5)	934·0 (908·8 to 958·8)	802·2 (655·1 to 948·6)	0·0 (0·0 to 0·0)	7070·1 (6583·7 to 7570·5)	5092·5 (4640·4 to 5564·6)	1063·9 (1035·2 to 1092·1)	913·7 (746·2 to 1080·5)	0·0 (0·0 to 0·0)
Austria	2044	6290·8 (5850·3 to 6741·3)	4526·5 (4117·8 to 4948·0)	939·4 (913·4 to 964·7)	824·9 (668·2 to 982·6)	0·0 (0·0 to 0·0)	7165·3 (6663·6 to 7678·5)	5155·8 (4690·3 to 5635·9)	1070·0 (1040·4 to 1098·8)	939·6 (761·1 to 1119·1)	0·0 (0·0 to 0·0)
Austria	2045	6374·4 (5921·6 to 6831·9)	4581·3 (4162·6 to 5018·2)	944·9 (918·1 to 971·0)	848·2 (681·5 to 1019·0)	0·0 (0·0 to 0·0)	7260·6 (6744·8 to 7781·7)	5218·2 (4741·3 to 5715·8)	1076·2 (1045·8 to 1106·0)	966·1 (776·3 to 1160·6)	0·0 (0·0 to 0·0)
Austria	2046	6457·5 (5991·4 to 6934·2)	4635·1 (4207·7 to 5079·0)	950·3 (923·0 to 977·1)	872·1 (695·6 to 1056·0)	0·0 (0·0 to 0·0)	7355·2 (6824·4 to 7898·2)	5279·5 (4792·6 to 5785·1)	1082·4 (1051·3 to 1113·0)	993·4 (792·3 to 1202·9)	0·0 (0·0 to 0·0)
Austria	2047	6537·8 (6059·5 to 7024·6)	4686·2 (4251·2 to 5138·5)	955·3 (927·3 to 982·5)	896·4 (708·5 to 1093·9)	0·0 (0·0 to 0·0)	7446·7 (6902·0 to 8001·1)	5337·6 (4842·2 to 5852·9)	1088·1 (1056·2 to 1119·1)	1021·0 (807·0 to 1246·0)	0·0 (0·0 to 0·0)
Austria	2048	6616·7 (6125·8 to 7123·6)	4735·6 (4294·5 to 5193·2)	960·0 (931·3 to 987·9)	921·1 (719·7 to 1132·8)	0·0 (0·0 to 0·0)	7536·5 (6977·4 to 8113·9)	5394·0 (4891·6 to 5915·2)	1093·4 (1060·8 to 1125·2)	1049·1 (819·8 to 1290·3)	0·0 (0·0 to 0·0)

Austria	2049	6693·5 (6191·6 to 7205·5)	4782·7 (4333·2 to 5252·0)	964·5 (935·1 to 993·0)	946·3 (734·1 to 1172·3)	0·0 (0·0 to 0·0)	7624·0 (7052·4 to 8207·2)	5447·6 (4935·6 to 5982·2)	1098·5 (1065·1 to 1131·0)	1077·8 (836·2 to 1335·2)	0·0 (0·0 to 0·0)
Austria	2050	6766·5 (6253·4 to 7293·7)	4826·2 (4376·5 to 5302·0)	968·5 (938·7 to 997·7)	971·8 (748·6 to 1210·6)	0·0 (0·0 to 0·0)	7707·2 (7122·7 to 8307·7)	5497·1 (4984·9 to 6039·1)	1103·2 (1069·2 to 1136·4)	1106·9 (852·6 to 1378·9)	0·0 (0·0 to 0·0)
Azerbaijan*	1995	35·2 (30·0 to 41·9)	12·0 (9·9 to 14·4)	23·2 (18·5 to 29·3)	0·0 (0·0 to 0·0)	0·0 (0·0 to 0·0)	123·7 (105·2 to 147·3)	42·1 (34·6 to 50·6)	81·5 (64·9 to 102·8)	0·0 (0·0 to 0·0)	0·0 (0·0 to 0·0)
Azerbaijan*	1996	34·3 (29·2 to 40·7)	11·7 (9·5 to 14·0)	22·6 (18·0 to 28·3)	0·0 (0·0 to 0·0)	0·1 (0·1 to 0·1)	120·6 (102·5 to 143·0)	41·1 (33·5 to 49·3)	79·2 (63·1 to 99·5)	0·0 (0·0 to 0·0)	0·2 (0·2 to 0·2)
Azerbaijan*	1997	33·0 (28·1 to 39·0)	11·0 (9·0 to 13·1)	22·0 (17·7 to 27·6)	0·0 (0·0 to 0·0)	0·0 (0·0 to 0·0)	115·9 (98·7 to 137·0)	38·7 (31·7 to 46·0)	77·1 (62·0 to 96·9)	0·0 (0·0 to 0·0)	0·1 (0·1 to 0·1)
Azerbaijan*	1998	32·0 (27·2 to 37·9)	10·0 (8·3 to 12·1)	21·9 (17·6 to 27·6)	0·0 (0·0 to 0·0)	0·1 (0·1 to 0·1)	112·3 (95·5 to 133·1)	35·2 (29·0 to 42·5)	76·8 (61·7 to 96·9)	0·0 (0·0 to 0·0)	0·3 (0·3 to 0·3)
Azerbaijan*	1999	34·7 (29·8 to 40·5)	10·3 (8·4 to 12·3)	22·7 (18·3 to 28·5)	0·0 (0·0 to 0·0)	1·7 (1·7 to 1·7)	121·9 (104·6 to 142·2)	36·1 (29·4 to 43·2)	79·6 (64·3 to 100·1)	0·0 (0·0 to 0·0)	6·1 (6·1 to 6·1)
Azerbaijan*	2000	35·9 (30·7 to 41·9)	10·2 (8·3 to 12·3)	23·5 (19·2 to 29·5)	0·0 (0·0 to 0·0)	2·2 (2·2 to 2·2)	126·0 (107·7 to 147·2)	35·8 (29·1 to 43·1)	82·5 (67·3 to 103·7)	0·0 (0·0 to 0·0)	7·7 (7·7 to 7·7)
Azerbaijan*	2001	34·0 (29·1 to 40·0)	9·8 (7·9 to 11·7)	23·8 (19·4 to 29·3)	0·0 (0·0 to 0·0)	0·4 (0·4 to 0·4)	119·6 (102·1 to 140·6)	34·5 (27·8 to 41·2)	83·6 (68·0 to 102·9)	0·0 (0·0 to 0·0)	1·5 (1·5 to 1·5)
Azerbaijan*	2002	39·3 (33·9 to 45·6)	12·1 (9·8 to 14·4)	26·5 (21·6 to 32·1)	0·0 (0·0 to 0·0)	0·8 (0·8 to 0·8)	138·2 (119·0 to 160·1)	42·5 (34·3 to 50·4)	93·1 (75·9 to 112·6)	0·0 (0·0 to 0·1)	2·7 (2·7 to 2·7)
Azerbaijan*	2003	39·9 (34·4 to 46·3)	12·6 (10·2 to 14·9)	26·9 (21·8 to 32·7)	0·0 (0·0 to 0·0)	0·5 (0·5 to 0·5)	140·0 (120·8 to 162·5)	44·1 (35·8 to 52·3)	94·3 (76·4 to 114·8)	0·0 (0·0 to 0·1)	1·6 (1·6 to 1·6)
Azerbaijan*	2004	43·2 (37·5 to 49·7)	14·8 (12·1 to 17·6)	28·0 (22·7 to 33·6)	0·0 (0·0 to 0·0)	0·4 (0·4 to 0·4)	151·7 (131·7 to 174·5)	52·0 (42·4 to 62·0)	98·3 (79·7 to 118·0)	0·0 (0·0 to 0·1)	1·4 (1·4 to 1·4)
Azerbaijan*	2005	48·2 (42·3 to 54·7)	17·6 (14·5 to 21·0)	29·3 (24·0 to 35·2)	0·0 (0·0 to 0·0)	1·3 (1·3 to 1·3)	169·4 (148·7 to 192·3)	62·0 (50·8 to 73·7)	102·8 (84·2 to 123·6)	0·0 (0·0 to 0·1)	4·5 (4·5 to 4·5)
Azerbaijan*	2006	59·9 (52·7 to 67·6)	24·0 (20·0 to 28·6)	34·0 (28·0 to 40·6)	0·0 (0·0 to 0·0)	1·8 (1·8 to 1·8)	210·2 (185·0 to 237·4)	84·4 (70·3 to 100·4)	119·4 (98·4 to 142·6)	0·0 (0·0 to 0·1)	6·4 (6·4 to 6·4)

Azerbaijan*	2007	70·2 (61·7 to 79·0)	29·7 (24·9 to 35·1)	39·0 (32·4 to 46·2)	0·0 (0·0 to 0·0)	1·5 (1·5 to 1·5)	246·5 (216·9 to 277·5)	104·2 (87·6 to 123·3)	137·0 (113·8 to 162·3)	0·1 (0·0 to 0·1)	5·3 (5·3 to 5·3)
Azerbaijan*	2008	84·6 (75·7 to 95·2)	36·7 (31·0 to 43·4)	46·1 (38·6 to 54·4)	0·0 (0·0 to 0·0)	1·9 (1·9 to 1·9)	297·2 (266·0 to 334·4)	128·8 (109·0 to 152·3)	161·9 (135·4 to 191·1)	0·1 (0·0 to 0·1)	6·5 (6·5 to 6·5)
Azerbaijan*	2009	98·9 (88·7 to 110·8)	41·6 (35·1 to 49·1)	54·7 (46·5 to 63·9)	0·0 (0·0 to 0·0)	2·6 (2·6 to 2·6)	347·5 (311·6 to 389·2)	146·2 (123·3 to 172·6)	192·1 (163·3 to 224·6)	0·1 (0·0 to 0·1)	9·1 (9·1 to 9·1)
Azerbaijan*	2010	104·2 (93·4 to 115·9)	41·3 (35·0 to 48·4)	59·8 (51·3 to 69·4)	0·0 (0·0 to 0·0)	3·0 (3·0 to 3·0)	365·8 (328·1 to 407·0)	145·0 (123·0 to 169·9)	210·2 (180·0 to 243·8)	0·1 (0·0 to 0·2)	10·6 (10·6 to 10·6)
Azerbaijan*	2011	113·7 (102·5 to 125·2)	42·8 (36·8 to 50·1)	66·2 (57·2 to 76·5)	0·0 (0·0 to 0·0)	4·6 (4·6 to 4·6)	399·4 (360·1 to 439·9)	150·5 (129·4 to 176·0)	232·5 (200·9 to 268·8)	0·1 (0·0 to 0·1)	16·2 (16·2 to 16·2)
Azerbaijan*	2012	124·4 (112·8 to 136·9)	45·8 (39·4 to 53·4)	76·0 (66·6 to 86·7)	0·0 (0·0 to 0·0)	2·5 (2·5 to 2·5)	436·9 (396·2 to 480·9)	160·9 (138·3 to 187·5)	267·0 (233·8 to 304·6)	0·1 (0·0 to 0·1)	8·9 (8·9 to 8·9)
Azerbaijan*	2013	138·8 (126·5 to 152·0)	48·4 (41·6 to 56·4)	87·3 (77·4 to 99·3)	0·0 (0·0 to 0·0)	3·1 (3·1 to 3·1)	487·6 (444·4 to 533·7)	170·0 (146·2 to 198·2)	306·8 (271·9 to 348·8)	0·1 (0·0 to 0·2)	10·8 (10·8 to 10·8)
Azerbaijan*	2014	149·9 (136·9 to 163·2)	49·2 (42·2 to 56·7)	98·8 (87·8 to 111·3)	0·0 (0·0 to 0·0)	2·0 (2·0 to 2·0)	526·7 (480·9 to 573·1)	172·7 (148·4 to 199·1)	346·9 (308·4 to 390·9)	0·1 (0·0 to 0·2)	7·0 (7·0 to 7·0)
Azerbaijan*	2015	164·4 (150·9 to 179·3)	51·6 (44·1 to 59·9)	111·8 (100·1 to 125·4)	0·0 (0·0 to 0·0)	1·0 (1·0 to 1·0)	577·3 (530·1 to 629·8)	181·1 (155·0 to 210·3)	392·6 (351·8 to 440·4)	0·1 (0·0 to 0·2)	3·5 (3·5 to 3·5)
Azerbaijan*	2016	161·5 (147·0 to 175·9)	46·9 (40·2 to 54·4)	113·7 (101·6 to 127·2)	0·0 (0·0 to 0·0)	0·9 (0·9 to 0·9)	567·1 (516·5 to 617·7)	164·6 (141·1 to 191·2)	399·2 (356·9 to 446·9)	0·1 (0·0 to 0·2)	3·1 (3·1 to 3·1)
Azerbaijan*	2017	160·1 (145·5 to 176·4)	45·0 (38·2 to 53·1)	113·8 (100·5 to 128·6)	0·0 (0·0 to 0·0)	1·3 (1·3 to 1·3)	562·3 (511·1 to 619·6)	158·2 (134·2 to 186·4)	399·5 (353·1 to 451·5)	0·1 (0·0 to 0·1)	4·5 (4·5 to 4·5)
Azerbaijan*	2018	154·5 (138·1 to 171·7)	42·7 (36·1 to 50·8)	111·0 (96·5 to 126·4)	0·0 (0·0 to 0·0)	0·8 (0·8 to 0·8)	542·5 (485·2 to 603·1)	149·8 (126·8 to 178·6)	389·9 (338·8 to 444·1)	0·1 (0·0 to 0·2)	2·7 (2·7 to 2·7)
Azerbaijan*	2019	156·6 (140·1 to 174·2)	43·8 (36·6 to 52·1)	111·9 (97·3 to 127·5)	0·0 (0·0 to 0·0)	0·8 (0·8 to 0·8)	550·1 (492·0 to 611·9)	154·0 (128·6 to 182·9)	393·2 (341·6 to 447·8)	0·1 (0·0 to 0·2)	2·8 (2·8 to 2·8)
Azerbaijan*	2020	172·0 (156·0 to 189·2)	62·8 (55·6 to 70·9)	108·2 (94·0 to 123·3)	0·0 (0·0 to 0·0)	1·0 (1·0 to 1·1)	604·1 (547·9 to 664·6)	220·4 (195·2 to 249·1)	380·0 (330·1 to 433·1)	0·1 (0·0 to 0·2)	3·5 (3·5 to 3·7)

Azerbaijan*	2021	156·6 (140·2 to 174·3)	51·3 (42·9 to 61·1)	104·3 (90·6 to 118·9)	0·0 (0·0 to 0·0)	1·0 (1·0 to 1·1)	550·2 (492·4 to 612·2)	180·3 (150·8 to 214·6)	366·4 (318·1 to 417·6)	0·1 (0·0 to 0·2)	3·5 (3·4 to 3·8)
Azerbaijan*	2022	145·5 (129·9 to 161·8)	40·4 (33·6 to 48·3)	104·3 (90·5 to 118·8)	0·0 (0·0 to 0·1)	0·8 (0·7 to 0·9)	511·0 (456·2 to 568·5)	141·9 (117·9 to 169·8)	366·2 (317·8 to 417·3)	0·1 (0·0 to 0·2)	2·8 (2·6 to 3·1)
Azerbaijan*	2023	145·2 (129·6 to 161·6)	40·0 (33·0 to 47·8)	104·4 (90·5 to 119·0)	0·0 (0·0 to 0·1)	0·8 (0·8 to 0·9)	510·0 (455·1 to 567·7)	140·4 (116·1 to 167·8)	366·5 (317·9 to 418·0)	0·1 (0·0 to 0·2)	3·0 (2·7 to 3·3)
Azerbaijan*	2024	145·1 (129·3 to 161·5)	39·5 (32·7 to 47·4)	104·7 (90·7 to 119·1)	0·0 (0·0 to 0·1)	0·9 (0·8 to 1·0)	509·5 (454·2 to 567·2)	138·7 (114·7 to 166·4)	367·6 (318·7 to 418·3)	0·1 (0·0 to 0·2)	3·2 (2·9 to 3·5)
Azerbaijan*	2025	145·0 (129·2 to 161·7)	39·0 (32·2 to 46·9)	105·0 (91·1 to 119·5)	0·0 (0·0 to 0·1)	1·0 (0·9 to 1·1)	509·2 (453·7 to 567·9)	137·0 (113·0 to 164·7)	368·8 (320·1 to 419·9)	0·1 (0·0 to 0·2)	3·4 (3·1 to 3·8)
Azerbaijan*	2026	146·9 (130·6 to 163·8)	40·6 (33·5 to 49·2)	105·3 (91·4 to 120·0)	0·0 (0·0 to 0·1)	1·0 (0·9 to 1·2)	516·1 (458·7 to 575·3)	142·5 (117·8 to 172·9)	369·9 (321·1 to 421·4)	0·1 (0·0 to 0·2)	3·6 (3·2 to 4·1)
Azerbaijan*	2027	148·7 (132·1 to 166·0)	42·2 (34·7 to 51·5)	105·4 (91·5 to 120·0)	0·0 (0·0 to 0·1)	1·1 (1·0 to 1·2)	522·2 (464·0 to 583·1)	148·2 (121·9 to 180·9)	370·1 (321·3 to 421·5)	0·1 (0·0 to 0·2)	3·8 (3·4 to 4·3)
Azerbaijan*	2028	150·4 (133·7 to 168·2)	43·7 (35·7 to 53·7)	105·5 (91·6 to 120·1)	0·0 (0·0 to 0·1)	1·1 (1·0 to 1·3)	528·1 (469·7 to 590·7)	153·6 (125·5 to 188·5)	370·4 (321·6 to 422·0)	0·1 (0·0 to 0·3)	3·9 (3·5 to 4·6)
Azerbaijan*	2029	152·2 (134·9 to 170·3)	45·3 (36·4 to 55·9)	105·7 (91·8 to 120·3)	0·0 (0·0 to 0·1)	1·2 (1·0 to 1·4)	534·4 (473·9 to 598·3)	159·0 (128·0 to 196·4)	371·1 (322·4 to 422·7)	0·1 (0·0 to 0·3)	4·1 (3·7 to 4·8)
Azerbaijan*	2030	154·1 (136·8 to 172·6)	46·8 (37·5 to 57·8)	106·0 (92·0 to 120·7)	0·0 (0·0 to 0·1)	1·2 (1·1 to 1·4)	541·2 (480·4 to 606·1)	164·5 (131·7 to 202·9)	372·3 (323·3 to 424·1)	0·2 (0·0 to 0·3)	4·3 (3·8 to 5·1)
Azerbaijan*	2031	156·1 (138·5 to 174·7)	48·4 (38·4 to 59·8)	106·4 (92·3 to 121·2)	0·0 (0·0 to 0·1)	1·3 (1·1 to 1·5)	548·2 (486·5 to 613·7)	170·0 (134·7 to 209·9)	373·6 (324·3 to 425·9)	0·2 (0·0 to 0·3)	4·5 (3·9 to 5·3)
Azerbaijan*	2032	158·0 (140·3 to 177·2)	49·9 (39·5 to 62·4)	106·8 (92·7 to 121·6)	0·0 (0·0 to 0·1)	1·3 (1·1 to 1·6)	555·0 (492·7 to 622·4)	175·2 (138·8 to 219·3)	375·0 (325·7 to 427·3)	0·2 (0·1 to 0·3)	4·6 (4·0 to 5·6)
Azerbaijan*	2033	159·9 (142·1 to 179·3)	51·2 (40·8 to 64·3)	107·3 (93·2 to 122·1)	0·1 (0·0 to 0·1)	1·4 (1·2 to 1·7)	561·7 (499·1 to 629·8)	180·0 (143·5 to 225·8)	376·8 (327·3 to 428·9)	0·2 (0·1 to 0·3)	4·8 (4·1 to 5·9)
Azerbaijan*	2034	162·1 (143·8 to 182·0)	52·6 (41·7 to 65·7)	108·0 (93·9 to 123·0)	0·1 (0·0 to 0·1)	1·4 (1·2 to 1·7)	569·4 (505·2 to 639·2)	184·8 (146·3 to 230·9)	379·4 (329·7 to 432·0)	0·2 (0·1 to 0·4)	4·9 (4·2 to 6·1)

Azerbaijan*	2035	163·9 (145·6 to 184·2)	53·8 (42·7 to 66·8)	108·6 (94·4 to 123·7)	0·1 (0·0 to 0·1)	1·5 (1·2 to 1·9)	575·7 (511·3 to 647·2)	189·0 (149·9 to 234·7)	381·3 (331·4 to 434·4)	0·2 (0·1 to 0·4)	5·1 (4·4 to 6·5)
Azerbaijan*	2036	165·7 (147·4 to 186·5)	55·0 (43·6 to 69·2)	109·1 (94·9 to 124·4)	0·1 (0·0 to 0·1)	1·5 (1·3 to 1·9)	582·0 (517·7 to 655·1)	193·2 (153·3 to 243·1)	383·2 (333·2 to 436·8)	0·2 (0·1 to 0·4)	5·3 (4·5 to 6·8)
Azerbaijan*	2037	167·2 (148·4 to 188·4)	56·1 (44·2 to 70·9)	109·5 (95·3 to 124·9)	0·1 (0·0 to 0·1)	1·6 (1·3 to 2·1)	587·4 (521·2 to 661·6)	196·9 (155·4 to 249·0)	384·7 (334·6 to 438·7)	0·2 (0·1 to 0·4)	5·5 (4·6 to 7·2)
Azerbaijan*	2038	169·0 (150·1 to 190·6)	57·1 (45·3 to 72·3)	110·2 (95·9 to 125·7)	0·1 (0·0 to 0·1)	1·6 (1·4 to 2·2)	593·5 (527·1 to 669·4)	200·5 (159·0 to 253·9)	387·1 (336·8 to 441·5)	0·2 (0·1 to 0·4)	5·7 (4·8 to 7·6)
Azerbaijan*	2039	170·6 (151·1 to 192·6)	58·1 (45·6 to 73·5)	110·8 (96·4 to 126·4)	0·1 (0·0 to 0·1)	1·7 (1·4 to 2·3)	599·4 (530·7 to 676·6)	203·9 (160·0 to 258·1)	389·3 (338·7 to 444·1)	0·2 (0·1 to 0·4)	5·9 (4·9 to 8·0)
Azerbaijan*	2040	172·1 (152·1 to 194·4)	58·9 (46·3 to 75·0)	111·3 (96·8 to 127·0)	0·1 (0·0 to 0·1)	1·8 (1·4 to 2·4)	604·5 (534·2 to 682·7)	207·0 (162·5 to 263·5)	391·0 (340·0 to 446·2)	0·3 (0·1 to 0·5)	6·2 (5·1 to 8·4)
Azerbaijan*	2041	173·7 (153·5 to 196·5)	59·8 (46·7 to 76·5)	112·0 (97·3 to 127·8)	0·1 (0·0 to 0·1)	1·8 (1·5 to 2·5)	610·1 (539·0 to 690·1)	210·2 (164·0 to 268·7)	393·2 (341·7 to 448·8)	0·3 (0·1 to 0·5)	6·5 (5·3 to 8·9)
Azerbaijan*	2042	175·1 (154·5 to 198·3)	60·8 (47·3 to 78·3)	112·4 (97·7 to 128·2)	0·1 (0·0 to 0·1)	1·9 (1·5 to 2·7)	615·2 (542·6 to 696·4)	213·4 (166·0 to 275·0)	394·7 (343·0 to 450·4)	0·3 (0·1 to 0·5)	6·8 (5·4 to 9·6)
Azerbaijan*	2043	176·6 (155·8 to 199·9)	61·7 (48·1 to 78·8)	112·8 (98·0 to 128·7)	0·1 (0·0 to 0·2)	2·0 (1·6 to 2·9)	620·4 (547·2 to 702·2)	216·7 (168·8 to 276·7)	396·3 (344·1 to 452·1)	0·3 (0·1 to 0·5)	7·1 (5·7 to 10·1)
Azerbaijan*	2044	177·8 (156·5 to 201·4)	62·6 (48·5 to 80·2)	113·0 (98·1 to 128·9)	0·1 (0·0 to 0·2)	2·1 (1·7 to 3·1)	624·4 (549·7 to 707·3)	219·8 (170·2 to 281·8)	396·9 (344·6 to 452·6)	0·3 (0·1 to 0·6)	7·5 (6·0 to 10·8)
Azerbaijan*	2045	178·8 (157·1 to 202·9)	63·4 (48·8 to 81·7)	113·0 (98·2 to 128·9)	0·1 (0·0 to 0·2)	2·3 (1·8 to 3·3)	628·2 (551·8 to 712·8)	222·9 (171·3 to 287·0)	397·1 (344·8 to 452·7)	0·3 (0·1 to 0·6)	7·9 (6·2 to 11·7)
Azerbaijan*	2046	180·1 (158·6 to 204·2)	64·5 (49·7 to 83·4)	113·1 (98·2 to 129·0)	0·1 (0·0 to 0·2)	2·4 (1·9 to 3·6)	632·6 (557·1 to 717·2)	226·4 (174·5 to 292·8)	397·4 (344·9 to 453·0)	0·3 (0·1 to 0·6)	8·4 (6·6 to 12·8)
Azerbaijan*	2047	181·3 (160·0 to 206·1)	65·5 (50·5 to 85·5)	113·2 (98·2 to 128·9)	0·1 (0·0 to 0·2)	2·6 (2·0 to 3·9)	636·8 (561·9 to 723·9)	230·0 (177·3 to 300·5)	397·5 (344·9 to 452·9)	0·3 (0·1 to 0·6)	9·0 (7·0 to 13·7)
Azerbaijan*	2048	182·5 (159·7 to 207·8)	66·6 (51·0 to 87·5)	113·1 (98·1 to 128·8)	0·1 (0·0 to 0·2)	2·7 (2·1 to 4·2)	641·1 (561·1 to 729·9)	234·0 (179·0 to 307·3)	397·2 (344·6 to 452·5)	0·4 (0·1 to 0·7)	9·6 (7·4 to 14·8)

Azerbaijan*	2049	183·5 (160·9 to 209·1)	67·7 (51·4 to 89·6)	112·8 (97·9 to 128·5)	0·1 (0·0 to 0·2)	2·9 (2·2 to 4·6)	644·7 (565·0 to 734·4)	237·8 (180·5 to 314·8)	396·2 (344·0 to 451·2)	0·4 (0·1 to 0·7)	10·3 (7·9 to 16·2)
Azerbaijan*	2050	184·6 (161·5 to 211·0)	68·9 (51·9 to 91·1)	112·4 (97·6 to 128·0)	0·1 (0·0 to 0·2)	3·2 (2·4 to 5·1)	648·4 (567·2 to 741·0)	241·9 (182·4 to 320·1)	395·0 (343·0 to 449·7)	0·4 (0·1 to 0·7)	11·1 (8·4 to 17·7)
Burundi*	1995	20·8 (18·2 to 23·8)	6·2 (5·0 to 7·5)	8·2 (6·4 to 10·3)	4·0 (2·7 to 5·6)	2·4 (2·4 to 2·4)	61·8 (54·2 to 70·7)	18·4 (14·8 to 22·2)	24·4 (19·0 to 30·5)	11·9 (8·2 to 16·7)	7·1 (7·1 to 7·1)
Burundi*	1996	19·2 (16·8 to 22·2)	6·1 (4·9 to 7·3)	7·9 (6·2 to 9·9)	4·0 (2·8 to 5·7)	1·1 (1·1 to 1·1)	57·1 (49·9 to 65·9)	18·2 (14·7 to 21·8)	23·6 (18·4 to 29·5)	12·0 (8·2 to 16·9)	3·4 (3·4 to 3·4)
Burundi*	1997	17·9 (15·6 to 20·7)	5·4 (4·3 to 6·4)	7·5 (5·9 to 9·5)	3·8 (2·6 to 5·5)	1·2 (1·2 to 1·2)	53·3 (46·4 to 61·3)	15·9 (12·7 to 19·1)	22·3 (17·5 to 28·1)	11·4 (7·9 to 16·2)	3·6 (3·6 to 3·6)
Burundi*	1998	18·4 (16·0 to 21·2)	5·4 (4·4 to 6·5)	7·7 (6·0 to 9·8)	4·0 (2·8 to 5·7)	1·3 (1·3 to 1·3)	54·6 (47·5 to 63·0)	16·1 (13·0 to 19·4)	22·9 (17·9 to 29·2)	11·8 (8·3 to 16·8)	3·7 (3·7 to 3·7)
Burundi*	1999	18·4 (16·0 to 21·1)	5·1 (4·2 to 6·1)	7·7 (6·0 to 9·7)	4·1 (2·8 to 5·8)	1·5 (1·5 to 1·5)	54·5 (47·6 to 62·6)	15·2 (12·5 to 18·2)	22·9 (17·8 to 28·8)	12·0 (8·3 to 17·3)	4·4 (4·4 to 4·4)
Burundi*	2000	19·0 (16·7 to 21·7)	4·8 (4·0 to 5·8)	7·7 (6·0 to 9·7)	4·4 (2·9 to 6·2)	2·1 (2·1 to 2·1)	56·4 (49·5 to 64·3)	14·4 (11·8 to 17·3)	23·0 (17·8 to 28·9)	12·9 (8·7 to 18·4)	6·1 (6·1 to 6·1)
Burundi*	2001	17·9 (15·6 to 20·4)	4·8 (3·9 to 5·8)	7·8 (6·0 to 9·8)	3·4 (2·3 to 4·9)	1·8 (1·8 to 1·8)	53·0 (46·3 to 60·5)	14·3 (11·7 to 17·2)	23·2 (17·9 to 29·0)	10·2 (6·9 to 14·5)	5·3 (5·3 to 5·3)
Burundi*	2002	17·8 (15·6 to 20·4)	4·5 (3·7 to 5·4)	7·8 (5·9 to 9·7)	3·3 (2·3 to 4·8)	2·2 (2·2 to 2·2)	52·9 (46·2 to 60·5)	13·4 (10·9 to 16·0)	23·1 (17·7 to 28·7)	9·9 (6·8 to 14·2)	6·6 (6·6 to 6·6)
Burundi*	2003	20·4 (17·9 to 23·1)	5·5 (4·5 to 6·6)	8·5 (6·6 to 10·6)	3·4 (2·3 to 4·7)	3·1 (3·1 to 3·1)	60·5 (53·1 to 68·5)	16·3 (13·5 to 19·6)	25·2 (19·5 to 31·4)	9·9 (6·8 to 13·9)	9·1 (9·1 to 9·1)
Burundi*	2004	22·9 (20·3 to 25·7)	6·4 (5·3 to 7·7)	9·1 (7·1 to 11·2)	3·1 (2·1 to 4·4)	4·4 (4·4 to 4·4)	68·0 (60·3 to 76·3)	18·9 (15·6 to 22·7)	26·9 (21·1 to 33·2)	9·1 (6·3 to 12·9)	13·0 (13·0 to 13·0)
Burundi*	2005	24·0 (21·6 to 26·6)	5·7 (4·7 to 6·9)	9·0 (7·2 to 11·1)	3·2 (2·2 to 4·5)	6·0 (6·0 to 6·0)	71·1 (64·0 to 78·8)	17·0 (14·1 to 20·5)	26·8 (21·3 to 32·8)	9·4 (6·6 to 13·3)	17·9 (17·9 to 17·9)
Burundi*	2006	26·8 (24·1 to 29·6)	6·2 (5·1 to 7·5)	9·8 (7·9 to 12·0)	3·9 (2·7 to 5·4)	6·9 (6·9 to 6·9)	79·5 (71·6 to 87·8)	18·5 (15·3 to 22·2)	29·1 (23·3 to 35·6)	11·6 (7·9 to 16·1)	20·4 (20·4 to 20·4)
Burundi*	2007	26·5 (23·8 to 29·4)	6·3 (5·2 to 7·5)	10·1 (8·2 to 12·2)	4·3 (3·0 to 6·1)	5·8 (5·8 to 5·8)	78·8 (70·7 to 87·2)	18·8 (15·5 to 22·2)	29·9 (24·3 to 36·1)	12·8 (8·8 to 18·0)	17·3 (17·3 to 17·3)
Burundi*	2008	28·2 (25·6 to 30·9)	6·2 (5·1 to 7·3)	9·8 (8·1 to 11·5)	4·4 (3·0 to 6·1)	7·9 (7·9 to 7·9)	83·6 (76·0 to 91·7)	18·3 (15·2 to 21·6)	29·0 (24·1 to 34·1)	12·9 (8·8 to 18·1)	23·4 (23·4 to 23·4)
Burundi*	2009	27·9 (25·6 to 30·4)	6·2 (5·2 to 7·2)	9·2 (7·9 to 10·7)	4·0 (2·8 to 5·5)	8·6 (8·6 to 8·6)	82·8 (76·1 to 90·2)	18·3 (15·3 to 21·4)	27·3 (23·5 to 31·6)	11·8 (8·2 to 16·2)	25·4 (25·4 to 25·4)

Burundi*	2010	31·0 (28·8 to 33·4)	7·0 (5·9 to 8·1)	8·8 (7·7 to 10·1)	4·1 (2·9 to 5·7)	11·1 (11·1 to 11·1)	92·1 (85·6 to 99·1)	20·7 (17·6 to 24·1)	26·2 (22·9 to 29·9)	12·2 (8·5 to 17·0)	32·9 (32·9 to 32·9)
Burundi*	2011	32·2 (29·9 to 34·5)	8·0 (6·9 to 9·2)	8·1 (7·1 to 9·1)	4·4 (3·0 to 6·2)	11·6 (11·6 to 11·6)	95·5 (88·9 to 102·3)	23·8 (20·5 to 27·4)	24·0 (21·1 to 27·0)	13·2 (9·1 to 18·4)	34·5 (34·5 to 34·5)
Burundi*	2012	28·7 (26·7 to 30·9)	7·6 (6·6 to 8·7)	7·0 (6·2 to 7·8)	4·5 (3·1 to 6·4)	9·6 (9·6 to 9·6)	85·2 (79·2 to 91·7)	22·6 (19·6 to 25·8)	20·6 (18·3 to 23·2)	13·4 (9·2 to 18·9)	28·6 (28·6 to 28·6)
Burundi*	2013	29·0 (27·1 to 31·2)	7·0 (6·1 to 7·9)	6·3 (5·7 to 7·1)	4·4 (3·1 to 6·2)	11·3 (11·3 to 11·3)	86·2 (80·4 to 92·6)	20·6 (18·0 to 23·6)	18·8 (16·8 to 21·1)	13·2 (9·1 to 18·5)	33·5 (33·5 to 33·5)
Burundi*	2014	28·4 (26·5 to 30·5)	7·3 (6·4 to 8·4)	6·4 (5·7 to 7·2)	4·6 (3·2 to 6·4)	10·2 (10·2 to 10·2)	84·3 (78·6 to 90·5)	21·6 (18·9 to 24·8)	18·9 (16·8 to 21·3)	13·5 (9·4 to 19·1)	30·2 (30·2 to 30·2)
Burundi*	2015	26·4 (24·6 to 28·5)	6·5 (5·7 to 7·6)	5·9 (5·2 to 6·7)	4·3 (3·0 to 6·0)	9·6 (9·6 to 9·6)	78·3 (73·0 to 84·5)	19·4 (16·8 to 22·4)	17·6 (15·5 to 20·0)	12·7 (8·8 to 17·8)	28·5 (28·5 to 28·5)
Burundi*	2016	28·2 (26·4 to 30·2)	5·7 (4·9 to 6·7)	5·6 (4·8 to 6·5)	4·1 (2·8 to 5·7)	12·7 (12·7 to 12·7)	83·6 (78·3 to 89·8)	17·1 (14·6 to 19·8)	16·6 (14·2 to 19·2)	12·3 (8·2 to 17·0)	37·7 (37·7 to 37·7)
Burundi*	2017	28·2 (26·3 to 30·3)	5·7 (4·9 to 6·8)	5·6 (4·7 to 6·6)	4·2 (2·8 to 5·9)	12·6 (12·6 to 12·6)	83·6 (78·0 to 89·8)	17·1 (14·5 to 20·1)	16·6 (14·0 to 19·5)	12·4 (8·4 to 17·4)	37·5 (37·5 to 37·5)
Burundi*	2018	27·3 (25·4 to 29·5)	5·9 (5·0 to 7·1)	5·7 (4·7 to 6·8)	4·4 (3·0 to 6·2)	11·2 (11·2 to 11·2)	80·9 (75·5 to 87·5)	17·6 (14·9 to 21·0)	17·0 (14·1 to 20·2)	12·9 (8·9 to 18·3)	33·4 (33·4 to 33·4)
Burundi*	2019	28·9 (26·9 to 31·3)	6·6 (5·5 to 7·9)	5·7 (4·7 to 6·7)	4·4 (3·0 to 6·2)	12·2 (12·0 to 12·5)	85·7 (79·8 to 92·9)	19·6 (16·2 to 23·4)	16·8 (14·0 to 20·0)	13·1 (9·0 to 18·4)	36·1 (35·6 to 37·0)
Burundi*	2020	41·0 (38·7 to 43·7)	14·6 (13·4 to 15·8)	5·3 (4·4 to 6·3)	4·2 (2·9 to 6·0)	16·9 (15·9 to 18·4)	121·6 (114·9 to 129·8)	43·2 (39·9 to 46·9)	15·8 (13·1 to 18·7)	12·5 (8·6 to 17·7)	50·0 (47·2 to 54·5)
Burundi*	2021	32·7 (30·6 to 35·3)	6·2 (5·0 to 7·4)	5·0 (4·2 to 6·0)	4·1 (2·8 to 5·8)	17·4 (16·8 to 18·8)	97·0 (90·8 to 104·8)	18·3 (15·0 to 22·0)	14·9 (12·4 to 17·7)	12·1 (8·3 to 17·2)	51·7 (49·7 to 55·8)
Burundi*	2022	28·8 (26·6 to 31·5)	6·1 (4·9 to 7·4)	5·1 (4·2 to 6·0)	4·2 (2·9 to 5·9)	13·4 (12·5 to 14·8)	85·5 (79·0 to 93·4)	18·2 (14·6 to 22·1)	15·0 (12·5 to 17·8)	12·5 (8·5 to 17·6)	39·8 (37·1 to 43·9)
Burundi*	2023	29·2 (26·9 to 32·0)	6·2 (5·0 to 7·7)	5·1 (4·2 to 6·0)	4·3 (3·0 to 6·1)	13·6 (12·6 to 15·0)	86·8 (79·9 to 95·0)	18·5 (14·9 to 22·7)	15·1 (12·5 to 17·9)	12·8 (8·8 to 18·2)	40·3 (37·4 to 44·6)
Burundi*	2024	29·7 (27·3 to 32·5)	6·4 (5·0 to 7·8)	5·1 (4·2 to 6·1)	4·4 (3·0 to 6·3)	13·7 (12·7 to 15·1)	88·0 (81·0 to 96·5)	18·9 (15·0 to 23·3)	15·2 (12·6 to 18·0)	13·2 (9·0 to 18·7)	40·8 (37·7 to 44·9)
Burundi*	2025	30·0 (27·6 to 33·0)	6·5 (5·0 to 8·1)	5·2 (4·3 to 6·1)	4·6 (3·1 to 6·5)	13·8 (12·7 to 15·3)	89·1 (82·0 to 98·0)	19·2 (14·9 to 24·0)	15·3 (12·7 to 18·1)	13·6 (9·3 to 19·3)	41·0 (37·8 to 45·5)
Burundi*	2026	30·4 (27·9 to 33·5)	6·6 (5·1 to 8·4)	5·2 (4·3 to 6·1)	4·7 (3·2 to 6·7)	13·9 (12·7 to 15·4)	90·2 (82·8 to 99·4)	19·6 (15·2 to 24·8)	15·4 (12·8 to 18·3)	14·0 (9·5 to 19·9)	41·2 (37·8 to 45·6)

Burundi*	2027	30·7 (28·1 to 33·9)	6·7 (5·1 to 8·5)	5·2 (4·3 to 6·2)	4·9 (3·3 to 6·9)	13·9 (12·7 to 15·5)	91·1 (83·3 to 100·6)	19·9 (15·2 to 25·4)	15·5 (12·9 to 18·4)	14·4 (9·8 to 20·5)	41·3 (37·8 to 45·9)
Burundi*	2028	31·0 (28·3 to 34·4)	6·8 (5·1 to 8·8)	5·3 (4·4 to 6·2)	5·0 (3·4 to 7·1)	14·0 (12·7 to 15·5)	92·1 (84·0 to 102·2)	20·2 (15·3 to 26·1)	15·6 (12·9 to 18·5)	14·9 (10·1 to 21·1)	41·4 (37·6 to 46·0)
Burundi*	2029	31·4 (28·5 to 34·8)	6·9 (5·2 to 9·1)	5·3 (4·4 to 6·3)	5·2 (3·5 to 7·3)	14·0 (12·7 to 15·6)	93·1 (84·5 to 103·2)	20·6 (15·3 to 26·9)	15·7 (13·0 to 18·6)	15·3 (10·4 to 21·7)	41·5 (37·6 to 46·4)
Burundi*	2030	31·6 (28·6 to 35·2)	7·0 (5·2 to 9·2)	5·3 (4·4 to 6·3)	5·3 (3·6 to 7·5)	13·9 (12·5 to 15·6)	93·8 (84·8 to 104·5)	20·9 (15·5 to 27·5)	15·8 (13·1 to 18·7)	15·8 (10·7 to 22·3)	41·3 (37·2 to 46·2)
Burundi*	2031	31·9 (28·7 to 35·6)	7·2 (5·3 to 9·5)	5·4 (4·5 to 6·4)	5·5 (3·7 to 7·8)	13·9 (12·5 to 15·6)	94·8 (85·3 to 105·7)	21·2 (15·8 to 28·1)	15·9 (13·2 to 18·9)	16·3 (11·0 to 23·1)	41·3 (37·2 to 46·3)
Burundi*	2032	32·3 (29·0 to 36·3)	7·3 (5·3 to 9·6)	5·4 (4·5 to 6·4)	5·7 (3·8 to 8·0)	14·0 (12·5 to 15·6)	95·8 (86·0 to 107·8)	21·6 (15·8 to 28·4)	16·0 (13·3 to 19·0)	16·8 (11·3 to 23·9)	41·5 (37·1 to 46·4)
Burundi*	2033	32·7 (29·2 to 36·6)	7·4 (5·3 to 9·8)	5·4 (4·5 to 6·4)	5·8 (3·9 to 8·3)	14·1 (12·6 to 15·8)	96·9 (86·8 to 108·7)	21·9 (15·8 to 29·1)	16·1 (13·4 to 19·1)	17·3 (11·6 to 24·7)	41·7 (37·3 to 46·8)
Burundi*	2034	33·1 (29·6 to 37·2)	7·5 (5·4 to 10·0)	5·4 (4·5 to 6·5)	6·0 (4·0 to 8·6)	14·2 (12·6 to 15·9)	98·1 (87·8 to 110·3)	22·2 (16·0 to 29·7)	16·1 (13·4 to 19·1)	17·8 (11·9 to 25·4)	42·1 (37·4 to 47·2)
Burundi*	2035	33·5 (29·9 to 37·7)	7·6 (5·4 to 10·3)	5·5 (4·5 to 6·5)	6·2 (4·1 to 8·8)	14·3 (12·7 to 16·1)	99·4 (88·6 to 111·9)	22·5 (15·9 to 30·5)	16·2 (13·5 to 19·2)	18·3 (12·2 to 26·2)	42·5 (37·5 to 47·9)
Burundi*	2036	33·9 (30·1 to 38·4)	7·7 (5·4 to 10·5)	5·5 (4·6 to 6·5)	6·3 (4·2 to 9·1)	14·4 (12·7 to 16·3)	100·6 (89·4 to 113·9)	22·8 (16·0 to 31·3)	16·3 (13·5 to 19·3)	18·8 (12·5 to 26·9)	42·8 (37·7 to 48·4)
Burundi*	2037	34·3 (30·5 to 39·0)	7·8 (5·6 to 10·6)	5·5 (4·6 to 6·5)	6·5 (4·3 to 9·4)	14·5 (12·8 to 16·5)	101·9 (90·7 to 115·8)	23·1 (16·5 to 31·4)	16·4 (13·6 to 19·4)	19·3 (12·8 to 27·8)	43·2 (38·0 to 48·8)
Burundi*	2038	34·8 (30·9 to 39·6)	7·9 (5·5 to 11·0)	5·5 (4·6 to 6·6)	6·7 (4·4 to 9·7)	14·7 (12·9 to 16·7)	103·3 (91·6 to 117·5)	23·4 (16·5 to 32·7)	16·4 (13·7 to 19·5)	19·9 (13·1 to 28·7)	43·5 (38·2 to 49·5)
Burundi*	2039	35·2 (31·1 to 40·2)	8·0 (5·5 to 11·2)	5·6 (4·6 to 6·6)	6·9 (4·5 to 10·0)	14·8 (12·9 to 16·9)	104·6 (92·4 to 119·2)	23·7 (16·4 to 33·2)	16·5 (13·7 to 19·6)	20·4 (13·5 to 29·6)	44·0 (38·3 to 50·3)
Burundi*	2040	35·7 (31·4 to 40·7)	8·1 (5·6 to 11·4)	5·6 (4·7 to 6·6)	7·1 (4·7 to 10·3)	14·9 (13·0 to 17·2)	106·0 (93·2 to 120·9)	24·0 (16·7 to 33·7)	16·6 (13·8 to 19·7)	21·0 (13·8 to 30·5)	44·3 (38·7 to 51·1)

Burundi*	2041	36·2 (31·7 to 41·2)	8·2 (5·6 to 11·6)	5·6 (4·7 to 6·7)	7·3 (4·8 to 10·6)	15·0 (13·0 to 17·4)	107·3 (94·1 to 122·2)	24·3 (16·7 to 34·3)	16·7 (13·9 to 19·8)	21·7 (14·2 to 31·5)	44·6 (38·6 to 51·8)
Burundi*	2042	36·6 (32·0 to 41·9)	8·3 (5·7 to 11·9)	5·7 (4·7 to 6·7)	7·5 (4·9 to 11·0)	15·1 (12·9 to 17·4)	108·6 (95·1 to 124·2)	24·7 (17·0 to 35·3)	16·9 (14·0 to 20·0)	22·3 (14·6 to 32·7)	44·8 (38·4 to 51·8)
Burundi*	2043	37·0 (32·4 to 42·5)	8·4 (5·7 to 12·4)	5·7 (4·8 to 6·8)	7·8 (5·1 to 11·4)	15·1 (12·9 to 17·8)	110·0 (96·1 to 126·1)	25·0 (16·8 to 36·8)	17·0 (14·1 to 20·2)	23·0 (15·1 to 33·8)	44·9 (38·3 to 52·8)
Burundi*	2044	37·5 (32·7 to 43·2)	8·6 (5·7 to 12·5)	5·8 (4·8 to 6·9)	8·0 (5·2 to 11·8)	15·2 (13·0 to 17·8)	111·4 (96·9 to 128·1)	25·4 (16·8 to 37·0)	17·2 (14·3 to 20·4)	23·7 (15·5 to 35·0)	45·1 (38·6 to 52·8)
Burundi*	2045	38·0 (33·1 to 44·4)	8·7 (5·7 to 12·8)	5·8 (4·9 to 6·9)	8·3 (5·4 to 12·2)	15·2 (13·0 to 18·0)	112·9 (98·3 to 131·7)	25·9 (17·0 to 37·9)	17·3 (14·4 to 20·6)	24·5 (15·9 to 36·3)	45·2 (38·5 to 53·3)
Burundi*	2046	38·5 (33·4 to 44·7)	8·9 (5·8 to 12·9)	5·9 (4·9 to 7·0)	8·5 (5·5 to 12·7)	15·3 (13·0 to 18·0)	114·3 (99·1 to 132·7)	26·3 (17·3 to 38·2)	17·5 (14·6 to 20·8)	25·3 (16·3 to 37·6)	45·3 (38·5 to 53·4)
Burundi*	2047	39·0 (33·6 to 45·5)	9·0 (6·0 to 12·9)	6·0 (5·0 to 7·1)	8·8 (5·7 to 13·1)	15·2 (12·9 to 18·2)	115·6 (99·7 to 134·9)	26·6 (17·7 to 38·4)	17·7 (14·7 to 21·0)	26·1 (16·8 to 38·9)	45·3 (38·3 to 53·9)
Burundi*	2048	39·4 (34·0 to 46·1)	9·1 (5·9 to 13·1)	6·0 (5·0 to 7·1)	9·1 (5·8 to 13·6)	15·2 (12·9 to 18·0)	117·0 (101·0 to 136·8)	27·0 (17·6 to 38·9)	17·9 (14·9 to 21·2)	26·9 (17·2 to 40·2)	45·2 (38·2 to 53·5)
Burundi*	2049	39·9 (34·2 to 46·6)	9·2 (6·0 to 13·2)	6·1 (5·1 to 7·2)	9·4 (6·0 to 14·0)	15·2 (12·8 to 18·2)	118·4 (101·5 to 138·4)	27·4 (17·7 to 39·3)	18·0 (15·0 to 21·4)	27·8 (17·7 to 41·6)	45·1 (38·0 to 53·9)
Burundi*	2050	40·4 (34·6 to 47·4)	9·4 (6·0 to 13·7)	6·1 (5·1 to 7·3)	9·7 (6·1 to 14·5)	15·2 (12·7 to 18·3)	119·9 (102·8 to 140·7)	27·9 (17·7 to 40·6)	18·2 (15·2 to 21·6)	28·7 (18·2 to 43·1)	45·0 (37·7 to 54·3)
Belgium	1995	2589·9 (2507·6 to 2681·5)	2014·5 (1939·9 to 2095·6)	493·4 (463·8 to 524·9)	82·0 (67·1 to 98·7)	0·0 (0·0 to 0·0)	2961·6 (2867·4 to 3066·3)	2303·6 (2218·2 to 2396·3)	564·2 (530·4 to 600·2)	93·8 (76·7 to 112·8)	0·0 (0·0 to 0·0)
Belgium	1996	2697·1 (2634·7 to 2768·3)	2081·7 (2024·4 to 2148·6)	526·7 (501·9 to 552·4)	88·8 (75·5 to 103·3)	0·0 (0·0 to 0·0)	3084·2 (3012·8 to 3165·6)	2380·4 (2314·9 to 2456·9)	602·3 (573·9 to 631·7)	101·5 (86·3 to 118·1)	0·0 (0·0 to 0·0)
Belgium	1997	2800·8 (2739·6 to 2864·5)	2118·6 (2062·3 to 2178·0)	580·7 (558·3 to 605·3)	101·5 (88·7 to 115·0)	0·0 (0·0 to 0·0)	3202·7 (3132·8 to 3275·5)	2422·6 (2358·2 to 2490·5)	664·0 (638·4 to 692·2)	116·1 (101·4 to 131·5)	0·0 (0·0 to 0·0)
Belgium	1998	2901·7 (2836·6 to 2966·2)	2164·4 (2105·9 to 2220·1)	619·4 (597·7 to 642·3)	117·9 (105·7 to 131·8)	0·0 (0·0 to 0·0)	3318·0 (3243·6 to 3391·9)	2474·9 (2408·1 to 2538·7)	708·3 (683·5 to 734·5)	134·8 (120·8 to 150·8)	0·0 (0·0 to 0·0)

Belgium	1999	3038·3 (2975·0 to 3106·3)	2260·3 (2201·6 to 2321·0)	641·4 (618·4 to 664·0)	136·6 (126·3 to 148·1)	0·0 (0·0 to 0·0)	3474·2 (3401·9 to 3552·0)	2584·6 (2517·5 to 2654·0)	733·5 (707·1 to 759·2)	156·2 (144·4 to 169·4)	0·0 (0·0 to 0·0)
Belgium	2000	3159·4 (3097·3 to 3229·5)	2363·3 (2308·4 to 2424·6)	645·7 (626·7 to 667·0)	150·4 (142·4 to 159·4)	0·0 (0·0 to 0·0)	3612·8 (3541·7 to 3692·8)	2702·4 (2639·6 to 2772·5)	738·4 (716·7 to 762·7)	172·0 (162·8 to 182·2)	0·0 (0·0 to 0·0)
Belgium	2001	3252·5 (3191·7 to 3318·0)	2437·4 (2380·4 to 2500·2)	657·6 (639·7 to 677·1)	157·4 (150·3 to 164·3)	0·0 (0·0 to 0·0)	3719·2 (3649·7 to 3794·1)	2787·2 (2721·9 to 2859·0)	752·0 (731·4 to 774·3)	180·0 (171·9 to 187·8)	0·0 (0·0 to 0·0)
Belgium	2002	3431·3 (3368·0 to 3498·5)	2556·1 (2498·8 to 2619·3)	708·5 (689·1 to 728·8)	166·7 (159·4 to 174·5)	0·0 (0·0 to 0·0)	3923·7 (3851·2 to 4000·5)	2922·9 (2857·3 to 2995·2)	810·2 (788·0 to 833·3)	190·6 (182·3 to 199·6)	0·0 (0·0 to 0·0)
Belgium	2003	3717·4 (3654·2 to 3780·3)	2757·6 (2695·3 to 2818·3)	773·9 (755·6 to 794·4)	185·9 (178·7 to 193·6)	0·0 (0·0 to 0·0)	4250·8 (4178·6 to 4322·7)	3153·2 (3082·1 to 3222·7)	885·0 (864·0 to 908·4)	212·6 (204·3 to 221·4)	0·0 (0·0 to 0·0)
Belgium	2004	3864·5 (3799·6 to 3929·2)	2869·0 (2806·2 to 2929·1)	798·9 (779·6 to 817·3)	196·6 (188·8 to 204·3)	0·0 (0·0 to 0·0)	4419·0 (4344·7 to 4493·0)	3280·7 (3208·9 to 3349·4)	913·6 (891·5 to 934·6)	224·8 (215·9 to 233·6)	0·0 (0·0 to 0·0)
Belgium	2005	4029·6 (3962·2 to 4096·4)	3007·9 (2941·8 to 3072·4)	815·8 (796·5 to 835·7)	205·8 (198·2 to 213·4)	0·0 (0·0 to 0·0)	4607·7 (4530·7 to 4684·1)	3439·5 (3363·9 to 3513·2)	932·9 (910·8 to 955·7)	235·3 (226·6 to 244·0)	0·0 (0·0 to 0·0)
Belgium	2006	3982·0 (3912·0 to 4046·6)	2954·8 (2890·2 to 3016·2)	820·4 (801·0 to 841·5)	206·8 (199·1 to 214·6)	0·0 (0·0 to 0·0)	4553·4 (4473·4 to 4627·2)	3378·8 (3304·9 to 3448·9)	938·1 (915·9 to 962·3)	236·5 (227·6 to 245·4)	0·0 (0·0 to 0·0)
Belgium	2007	4111·9 (4045·1 to 4173·8)	3057·5 (2994·8 to 3115·8)	848·5 (828·4 to 868·2)	205·9 (198·6 to 212·9)	0·0 (0·0 to 0·0)	4701·9 (4625·5 to 4772·7)	3496·2 (3424·5 to 3562·9)	970·3 (947·3 to 992·8)	235·4 (227·1 to 243·4)	0·0 (0·0 to 0·0)
Belgium	2008	4306·9 (4241·8 to 4373·3)	3242·3 (3179·8 to 3307·5)	874·7 (855·0 to 894·9)	190·0 (182·7 to 197·1)	0·0 (0·0 to 0·0)	4924·9 (4850·4 to 5000·8)	3707·5 (3636·0 to 3782·1)	1000·2 (977·7 to 1023·3)	217·2 (208·9 to 225·3)	0·0 (0·0 to 0·0)
Belgium	2009	4492·6 (4427·4 to 4555·8)	3414·7 (3350·4 to 3475·7)	896·3 (877·1 to 916·6)	181·5 (174·4 to 188·8)	0·0 (0·0 to 0·0)	5137·2 (5062·6 to 5209·6)	3904·7 (3831·1 to 3974·4)	1025·0 (1003·0 to 1048·1)	207·5 (199·4 to 215·9)	0·0 (0·0 to 0·0)
Belgium	2010	4557·0 (4489·0 to 4624·7)	3468·1 (3407·4 to 3532·1)	907·0 (886·6 to 926·6)	181·9 (175·2 to 189·1)	0·0 (0·0 to 0·0)	5210·9 (5133·1 to 5288·3)	3965·8 (3896·3 to 4038·9)	1037·2 (1013·8 to 1059·6)	208·0 (200·3 to 216·3)	0·0 (0·0 to 0·0)
Belgium	2011	4649·6 (4584·2 to 4715·1)	3542·4 (3481·7 to 3610·8)	917·9 (897·2 to 938·7)	189·2 (182·0 to 196·9)	0·0 (0·0 to 0·0)	5316·7 (5242·0 to 5391·7)	4050·7 (3981·3 to 4128·9)	1049·7 (1025·9 to 1073·4)	216·4 (208·1 to 225·2)	0·0 (0·0 to 0·0)
Belgium	2012	4723·0 (4657·1 to 4789·7)	3604·2 (3538·7 to 3666·0)	925·8 (903·9 to 946·9)	193·0 (186·0 to 200·2)	0·0 (0·0 to 0·0)	5400·7 (5325·4 to 5477·0)	4121·3 (4046·4 to 4192·0)	1058·7 (1033·6 to 1082·8)	220·7 (212·7 to 228·9)	0·0 (0·0 to 0·0)

Belgium	2013	4716·7 (4651·0 to 4784·6)	3586·8 (3527·9 to 3652·3)	930·8 (909·6 to 951·2)	199·2 (191·5 to 207·0)	0·0 (0·0 to 0·0)	5393·5 (5318·4 to 5471·1)	4101·4 (4034·1 to 4176·3)	1064·4 (1040·1 to 1087·7)	227·8 (219·0 to 236·7)	0·0 (0·0 to 0·0)
Belgium	2014	4740·8 (4675·1 to 4812·3)	3598·4 (3534·7 to 3661·8)	933·5 (911·5 to 954·0)	208·9 (201·1 to 216·6)	0·0 (0·0 to 0·0)	5421·0 (5345·9 to 5502·8)	4114·7 (4041·9 to 4187·3)	1067·4 (1042·3 to 1090·8)	238·8 (229·9 to 247·7)	0·0 (0·0 to 0·0)
Belgium	2015	4761·4 (4691·3 to 4831·5)	3611·4 (3546·9 to 3680·1)	926·5 (907·5 to 947·6)	223·5 (216·3 to 231·2)	0·0 (0·0 to 0·0)	5444·6 (5364·4 to 5524·8)	4129·6 (4055·8 to 4208·2)	1059·4 (1037·7 to 1083·6)	255·6 (247·3 to 264·4)	0·0 (0·0 to 0·0)
Belgium	2016	4800·9 (4728·4 to 4866·1)	3649·2 (3581·7 to 3713·2)	916·4 (896·7 to 935·8)	235·3 (227·6 to 243·4)	0·0 (0·0 to 0·0)	5489·8 (5406·9 to 5564·3)	4172·8 (4095·7 to 4246·0)	1047·9 (1025·3 to 1070·1)	269·1 (260·2 to 278·4)	0·0 (0·0 to 0·0)
Belgium	2017	4853·1 (4784·8 to 4916·2)	3685·3 (3618·3 to 3748·7)	924·3 (904·7 to 943·6)	243·4 (235·7 to 251·6)	0·0 (0·0 to 0·0)	5549·4 (5471·3 to 5621·6)	4214·1 (4137·4 to 4286·6)	1057·0 (1034·5 to 1079·0)	278·4 (269·5 to 287·6)	0·0 (0·0 to 0·0)
Belgium	2018	4920·6 (4831·5 to 5012·0)	3734·2 (3650·6 to 3815·5)	936·3 (910·3 to 962·8)	250·0 (240·5 to 259·6)	0·0 (0·0 to 0·0)	5626·6 (5524·8 to 5731·1)	4270·1 (4174·5 to 4363·0)	1070·6 (1040·9 to 1101·0)	285·9 (275·0 to 296·8)	0·0 (0·0 to 0·0)
Belgium	2019	4982·9 (4887·2 to 5083·5)	3780·7 (3691·3 to 3873·1)	944·0 (917·6 to 970·5)	258·1 (248·4 to 268·4)	0·0 (0·0 to 0·0)	5697·9 (5588·4 to 5812·9)	4323·2 (4221·0 to 4428·9)	1079·5 (1049·3 to 1109·8)	295·2 (284·0 to 307·0)	0·0 (0·0 to 0·0)
Belgium	2020	5282·6 (5178·4 to 5393·0)	4153·0 (4055·8 to 4255·2)	882·6 (858·5 to 907·2)	247·0 (237·0 to 257·2)	0·0 (0·0 to 0·0)	6040·6 (5921·5 to 6166·9)	4748·9 (4637·8 to 4865·7)	1009·2 (981·7 to 1037·4)	282·4 (271·0 to 294·0)	0·0 (0·0 to 0·0)
Belgium	2021	5088·1 (4987·4 to 5194·7)	3997·6 (3904·0 to 4095·9)	847·7 (825·1 to 871·3)	242·8 (232·3 to 253·4)	0·0 (0·0 to 0·0)	5818·2 (5703·1 to 5940·1)	4571·2 (4464·2 to 4683·6)	969·4 (943·5 to 996·3)	277·7 (265·6 to 289·7)	0·0 (0·0 to 0·0)
Belgium	2022	4608·1 (4502·7 to 4721·1)	3510·1 (3408·8 to 3616·7)	849·1 (826·1 to 872·7)	248·9 (238·0 to 260·3)	0·0 (0·0 to 0·0)	5269·3 (5148·8 to 5398·5)	4013·8 (3897·9 to 4135·7)	970·9 (944·6 to 997·9)	284·6 (272·2 to 297·7)	0·0 (0·0 to 0·0)
Belgium	2023	4657·9 (4538·5 to 4786·6)	3552·7 (3435·6 to 3673·3)	850·0 (826·7 to 873·5)	255·2 (243·3 to 267·4)	0·0 (0·0 to 0·0)	5326·2 (5189·8 to 5473·5)	4062·4 (3928·5 to 4200·4)	972·0 (945·3 to 998·9)	291·8 (278·2 to 305·8)	0·0 (0·0 to 0·0)
Belgium	2024	4706·1 (4567·9 to 4849·7)	3593·9 (3459·0 to 3730·3)	850·6 (826·9 to 874·0)	261·5 (248·5 to 275·1)	0·0 (0·0 to 0·0)	5381·3 (5223·3 to 5545·5)	4109·6 (3955·4 to 4265·5)	972·6 (945·6 to 999·4)	299·1 (284·1 to 314·6)	0·0 (0·0 to 0·0)
Belgium	2025	4755·3 (4600·0 to 4916·9)	3636·0 (3478·3 to 3792·7)	851·2 (827·5 to 874·8)	268·1 (252·8 to 283·2)	0·0 (0·0 to 0·0)	5437·6 (5260·1 to 5622·5)	4157·7 (3977·4 to 4336·9)	973·3 (946·3 to 1000·4)	306·6 (289·1 to 323·8)	0·0 (0·0 to 0·0)
Belgium	2026	4805·9 (4631·4 to 4992·9)	3679·1 (3504·9 to 3856·1)	851·9 (828·3 to 875·4)	274·9 (257·3 to 291·9)	0·0 (0·0 to 0·0)	5495·5 (5295·9 to 5709·3)	4207·0 (4007·8 to 4409·4)	974·2 (947·1 to 1001·0)	314·4 (294·3 to 333·8)	0·0 (0·0 to 0·0)

Belgium	2027	4858·0 (4681·5 to 5047·9)	3723·2 (3545·1 to 3899·1)	852·9 (828·9 to 876·5)	281·9 (261·8 to 301·3)	0·0 (0·0 to 0·0)	5555·0 (5353·3 to 5772·2)	4257·4 (4053·8 to 4458·6)	975·3 (947·8 to 1002·2)	322·3 (299·4 to 344·5)	0·0 (0·0 to 0·0)
Belgium	2028	4920·3 (4737·9 to 5117·4)	3776·7 (3602·4 to 3964·9)	854·4 (830·3 to 878·1)	289·2 (266·5 to 310·8)	0·0 (0·0 to 0·0)	5626·3 (5417·7 to 5851·7)	4318·6 (4119·3 to 4533·8)	977·0 (949·5 to 1004·1)	330·7 (304·7 to 355·4)	0·0 (0·0 to 0·0)
Belgium	2029	4982·9 (4794·7 to 5186·1)	3831·4 (3643·8 to 4025·8)	855·0 (830·7 to 878·5)	296·5 (272·9 to 319·6)	0·0 (0·0 to 0·0)	5697·8 (5482·7 to 5930·3)	4381·1 (4166·7 to 4603·4)	977·7 (949·8 to 1004·6)	339·0 (312·1 to 365·4)	0·0 (0·0 to 0·0)
Belgium	2030	5045·8 (4846·6 to 5259·7)	3886·0 (3688·1 to 4087·9)	855·9 (832·1 to 879·6)	303·9 (279·8 to 327·1)	0·0 (0·0 to 0·0)	5769·9 (5542·0 to 6014·4)	4443·6 (4217·3 to 4674·5)	978·7 (951·4 to 1005·9)	347·5 (320·0 to 374·0)	0·0 (0·0 to 0·0)
Belgium	2031	5111·0 (4907·3 to 5327·6)	3941·1 (3741·2 to 4142·8)	858·1 (833·7 to 881·8)	311·9 (286·0 to 337·2)	0·0 (0·0 to 0·0)	5844·4 (5611·4 to 6092·0)	4506·5 (4278·0 to 4737·3)	981·2 (953·3 to 1008·3)	356·6 (327·1 to 385·6)	0·0 (0·0 to 0·0)
Belgium	2032	5177·5 (4967·5 to 5402·0)	3996·7 (3789·5 to 4202·5)	860·6 (836·4 to 884·7)	320·2 (293·3 to 345·6)	0·0 (0·0 to 0·0)	5920·4 (5680·3 to 6177·1)	4570·1 (4333·3 to 4805·5)	984·1 (956·4 to 1011·6)	366·2 (335·3 to 395·2)	0·0 (0·0 to 0·0)
Belgium	2033	5241·5 (5030·1 to 5480·2)	4049·8 (3838·8 to 4264·7)	863·0 (839·0 to 887·2)	328·7 (300·5 to 355·0)	0·0 (0·0 to 0·0)	5993·6 (5751·8 to 6266·6)	4630·9 (4389·7 to 4876·6)	986·8 (959·4 to 1014·4)	375·9 (343·7 to 405·9)	0·0 (0·0 to 0·0)
Belgium	2034	5307·0 (5084·2 to 5553·1)	4103·6 (3879·8 to 4336·9)	865·8 (842·2 to 890·1)	337·6 (307·8 to 366·0)	0·0 (0·0 to 0·0)	6068·5 (5813·7 to 6349·9)	4692·4 (4436·5 to 4959·2)	990·0 (963·0 to 1017·8)	386·1 (351·9 to 418·6)	0·0 (0·0 to 0·0)
Belgium	2035	5370·9 (5139·7 to 5632·3)	4155·6 (3924·6 to 4408·3)	868·6 (844·9 to 893·1)	346·7 (315·5 to 377·7)	0·0 (0·0 to 0·0)	6141·5 (5877·2 to 6440·5)	4751·8 (4487·7 to 5040·8)	993·2 (966·1 to 1021·3)	396·5 (360·8 to 431·9)	0·0 (0·0 to 0·0)
Belgium	2036	5435·9 (5194·8 to 5715·5)	4207·8 (3966·2 to 4470·0)	871·9 (848·1 to 896·7)	356·2 (322·8 to 389·8)	0·0 (0·0 to 0·0)	6215·9 (5940·2 to 6535·6)	4811·5 (4535·3 to 5111·4)	997·0 (969·8 to 1025·3)	407·4 (369·1 to 445·7)	0·0 (0·0 to 0·0)
Belgium	2037	5499·5 (5245·5 to 5784·5)	4258·4 (4006·5 to 4530·6)	875·1 (851·2 to 900·2)	366·0 (329·8 to 402·2)	0·0 (0·0 to 0·0)	6288·6 (5998·1 to 6614·6)	4869·4 (4581·3 to 5180·7)	1000·7 (973·3 to 1029·4)	418·5 (377·1 to 459·9)	0·0 (0·0 to 0·0)
Belgium	2038	5568·3 (5300·5 to 5860·7)	4312·3 (4048·7 to 4597·7)	879·5 (855·2 to 904·8)	376·5 (336·9 to 415·1)	0·0 (0·0 to 0·0)	6367·2 (6061·1 to 6701·6)	4931·1 (4629·6 to 5257·4)	1005·7 (977·9 to 1034·7)	430·5 (385·2 to 474·6)	0·0 (0·0 to 0·0)
Belgium	2039	5640·5 (5359·0 to 5946·3)	4368·3 (4086·6 to 4654·5)	884·7 (860·4 to 910·2)	387·6 (344·3 to 429·9)	0·0 (0·0 to 0·0)	6449·9 (6128·0 to 6799·6)	4995·1 (4673·0 to 5322·4)	1011·6 (983·9 to 1040·8)	443·2 (393·7 to 491·6)	0·0 (0·0 to 0·0)
Belgium	2040	5715·0 (5415·3 to 6032·4)	4425·4 (4122·9 to 4724·0)	890·4 (865·3 to 916·3)	399·2 (352·7 to 444·9)	0·0 (0·0 to 0·0)	6535·0 (6192·4 to 6898·0)	5060·4 (4714·5 to 5401·8)	1018·1 (989·4 to 1047·8)	456·5 (403·3 to 508·7)	0·0 (0·0 to 0·0)

Belgium	2041	5790·6 (5474·8 to 6121·6)	4483·0 (4167·7 to 4799·3)	896·3 (870·7 to 922·6)	411·3 (360·5 to 461·0)	0·0 (0·0 to 0·0)	6621·5 (6260·4 to 7000·0)	5126·2 (4765·7 to 5487·9)	1025·0 (995·6 to 1055·0)	470·3 (412·2 to 527·2)	0·0 (0·0 to 0·0)
Belgium	2042	5866·8 (5533·9 to 6210·8)	4540·7 (4206·3 to 4870·8)	902·4 (876·2 to 929·1)	423·8 (369·3 to 478·3)	0·0 (0·0 to 0·0)	6708·7 (6327·9 to 7102·0)	5192·2 (4809·8 to 5569·7)	1031·9 (1001·9 to 1062·5)	484·6 (422·3 to 547·0)	0·0 (0·0 to 0·0)
Belgium	2043	5944·4 (5585·1 to 6304·1)	4599·0 (4251·8 to 4944·2)	908·7 (881·9 to 935·9)	436·8 (377·6 to 496·5)	0·0 (0·0 to 0·0)	6797·4 (6386·5 to 7208·6)	5258·9 (4861·8 to 5653·6)	1039·1 (1008·5 to 1070·1)	499·4 (431·8 to 567·7)	0·0 (0·0 to 0·0)
Belgium	2044	6021·2 (5652·1 to 6394·5)	4656·1 (4290·0 to 5018·6)	915·0 (888·1 to 942·3)	450·1 (385·0 to 514·9)	0·0 (0·0 to 0·0)	6885·2 (6463·1 to 7312·0)	5324·2 (4905·6 to 5738·8)	1046·3 (1015·5 to 1077·5)	514·7 (440·3 to 588·8)	0·0 (0·0 to 0·0)
Belgium	2045	6097·9 (5713·4 to 6480·9)	4712·8 (4337·7 to 5087·0)	921·3 (894·3 to 948·9)	463·9 (393·1 to 534·4)	0·0 (0·0 to 0·0)	6972·9 (6533·2 to 7410·8)	5389·0 (4960·1 to 5816·9)	1053·5 (1022·6 to 1085·0)	530·4 (449·5 to 611·1)	0·0 (0·0 to 0·0)
Belgium	2046	6173·7 (5774·0 to 6575·9)	4767·8 (4369·5 to 5155·7)	927·8 (900·7 to 955·6)	478·1 (402·0 to 553·9)	0·0 (0·0 to 0·0)	7059·5 (6602·5 to 7519·5)	5451·9 (4996·5 to 5895·5)	1060·9 (1029·9 to 1092·7)	546·7 (459·7 to 633·3)	0·0 (0·0 to 0·0)
Belgium	2047	6251·6 (5834·1 to 6667·4)	4824·1 (4412·6 to 5225·8)	934·6 (906·5 to 962·6)	492·9 (411·2 to 576·7)	0·0 (0·0 to 0·0)	7148·6 (6671·2 to 7624·1)	5516·3 (5045·8 to 5975·6)	1068·7 (1036·6 to 1100·8)	563·7 (470·2 to 659·5)	0·0 (0·0 to 0·0)
Belgium	2048	6330·8 (5886·1 to 6759·1)	4880·7 (4460·5 to 5300·1)	941·7 (912·9 to 970·2)	508·4 (420·7 to 599·8)	0·0 (0·0 to 0·0)	7239·2 (6730·7 to 7728·9)	5581·0 (5100·6 to 6060·6)	1076·9 (1043·9 to 1109·4)	581·3 (481·1 to 685·8)	0·0 (0·0 to 0·0)
Belgium	2049	6410·1 (5950·1 to 6857·8)	4936·7 (4492·7 to 5369·2)	949·0 (920·1 to 978·2)	524·3 (431·3 to 623·0)	0·0 (0·0 to 0·0)	7329·8 (6803·8 to 7841·8)	5645·1 (5137·3 to 6139·7)	1085·2 (1052·2 to 1118·5)	599·5 (493·2 to 712·4)	0·0 (0·0 to 0·0)
Belgium	2050	6489·9 (6018·5 to 6959·6)	4992·5 (4531·0 to 5441·4)	956·5 (926·8 to 986·2)	540·8 (441·0 to 645·5)	0·0 (0·0 to 0·0)	7421·1 (6882·1 to 7958·2)	5708·9 (5181·1 to 6222·2)	1093·8 (1059·8 to 1127·8)	618·4 (504·2 to 738·1)	0·0 (0·0 to 0·0)
Benin*	1995	27·1 (24·0 to 30·9)	7·5 (6·1 to 9·1)	15·5 (12·6 to 19·0)	1·6 (1·1 to 2·3)	2·5 (2·5 to 2·5)	74·2 (65·5 to 84·5)	20·5 (16·8 to 24·8)	42·3 (34·4 to 51·9)	4·5 (3·0 to 6·4)	6·9 (6·9 to 6·9)
Benin*	1996	27·8 (24·9 to 31·5)	7·3 (6·0 to 8·9)	15·3 (12·7 to 18·4)	1·6 (1·1 to 2·3)	3·6 (3·6 to 3·6)	76·1 (68·1 to 86·2)	20·0 (16·4 to 24·3)	41·9 (34·8 to 50·4)	4·4 (3·0 to 6·4)	9·8 (9·8 to 9·8)
Benin*	1997	26·9 (24·1 to 30·2)	7·4 (6·0 to 9·1)	15·4 (13·1 to 18·2)	1·6 (1·1 to 2·3)	2·5 (2·5 to 2·5)	73·7 (66·0 to 82·7)	20·3 (16·5 to 24·8)	42·2 (35·8 to 49·9)	4·4 (3·0 to 6·4)	6·7 (6·7 to 6·7)
Benin*	1998	30·1 (27·7 to 32·8)	7·3 (5·9 to 8·8)	15·3 (13·4 to 17·4)	1·6 (1·1 to 2·3)	5·9 (5·9 to 5·9)	82·3 (75·8 to 89·8)	19·9 (16·2 to 24·1)	41·8 (36·8 to 47·6)	4·3 (2·9 to 6·2)	16·2 (16·2 to 16·2)
Benin*	1999	28·2 (26·3 to 30·4)	6·8 (5·5 to 8·1)	14·9 (13·6 to 16·3)	1·5 (1·0 to 2·1)	5·0 (5·0 to 5·0)	77·1 (72·1 to 83·1)	18·5 (14·9 to 22·2)	40·8 (37·3 to 44·5)	4·1 (2·8 to 5·9)	13·7 (13·7 to 13·7)

Benin*	2000	30·5 (28·7 to 32·5)	7·9 (6·5 to 9·5)	16·0 (15·0 to 17·0)	1·6 (1·1 to 2·3)	5·0 (5·0 to 5·0)	83·4 (78·5 to 89·0)	21·7 (17·7 to 26·0)	43·7 (41·2 to 46·4)	4·3 (2·9 to 6·2)	13·7 (13·7 to 13·7)
Benin*	2001	29·8 (28·0 to 31·6)	8·1 (6·5 to 9·8)	16·2 (15·4 to 17·0)	1·6 (1·0 to 2·2)	4·0 (4·0 to 4·0)	81·4 (76·6 to 86·5)	22·0 (17·8 to 26·7)	44·2 (42·2 to 46·4)	4·2 (2·9 to 6·0)	10·9 (10·9 to 10·9)
Benin*	2002	29·4 (27·5 to 31·2)	7·8 (6·3 to 9·3)	16·2 (15·4 to 17·1)	1·6 (1·1 to 2·2)	3·8 (3·8 to 3·8)	80·3 (75·3 to 85·4)	21·3 (17·2 to 25·6)	44·3 (42·0 to 46·8)	4·3 (2·9 to 6·0)	10·5 (10·5 to 10·5)
Benin*	2003	30·3 (28·5 to 32·1)	7·2 (5·8 to 8·6)	15·8 (14·9 to 16·9)	1·6 (1·1 to 2·2)	5·7 (5·7 to 5·7)	82·9 (77·9 to 87·8)	19·6 (15·9 to 23·5)	43·3 (40·6 to 46·1)	4·3 (2·9 to 6·0)	15·7 (15·7 to 15·7)
Benin*	2004	32·3 (30·5 to 34·1)	7·1 (5·8 to 8·5)	15·7 (14·6 to 16·7)	1·6 (1·1 to 2·2)	8·0 (8·0 to 8·0)	88·4 (83·4 to 93·3)	19·4 (15·9 to 23·3)	42·8 (40·0 to 45·7)	4·3 (2·9 to 6·1)	21·8 (21·8 to 21·8)
Benin*	2005	32·4 (30·7 to 34·1)	7·1 (5·8 to 8·4)	15·4 (14·5 to 16·3)	1·6 (1·1 to 2·3)	8·3 (8·3 to 8·3)	88·6 (84·0 to 93·2)	19·3 (15·9 to 23·1)	42·1 (39·7 to 44·6)	4·4 (3·0 to 6·2)	22·8 (22·8 to 22·8)
Benin*	2006	30·6 (29·1 to 32·3)	6·6 (5·5 to 7·9)	14·6 (13·9 to 15·5)	1·6 (1·1 to 2·2)	7·8 (7·8 to 7·8)	83·8 (79·6 to 88·4)	18·0 (14·9 to 21·7)	40·1 (37·9 to 42·3)	4·3 (2·9 to 6·1)	21·4 (21·4 to 21·4)
Benin*	2007	30·4 (28·7 to 32·2)	7·1 (5·9 to 8·5)	14·6 (13·7 to 15·6)	1·7 (1·1 to 2·4)	7·0 (7·0 to 7·0)	83·1 (78·5 to 88·0)	19·4 (16·2 to 23·3)	40·0 (37·5 to 42·6)	4·5 (3·1 to 6·4)	19·1 (19·1 to 19·1)
Benin*	2008	30·4 (28·7 to 32·2)	6·8 (5·7 to 8·1)	13·7 (12·8 to 14·7)	1·6 (1·1 to 2·4)	8·2 (8·2 to 8·2)	83·1 (78·5 to 88·0)	18·6 (15·6 to 22·3)	37·5 (35·0 to 40·3)	4·5 (3·0 to 6·5)	22·5 (22·5 to 22·5)
Benin*	2009	34·9 (33·1 to 37·0)	7·8 (6·6 to 9·2)	14·0 (12·9 to 15·1)	1·7 (1·1 to 2·4)	11·4 (11·4 to 11·4)	95·6 (90·5 to 101·2)	21·3 (18·0 to 25·3)	38·3 (35·3 to 41·4)	4·7 (3·1 to 6·6)	31·3 (31·3 to 31·3)
Benin*	2010	31·7 (30·0 to 33·6)	7·1 (6·1 to 8·5)	13·3 (12·2 to 14·5)	1·6 (1·1 to 2·3)	9·7 (9·7 to 9·7)	86·7 (82·0 to 91·9)	19·5 (16·7 to 23·1)	36·4 (33·4 to 39·6)	4·4 (2·9 to 6·2)	26·4 (26·4 to 26·4)
Benin*	2011	32·8 (31·2 to 34·6)	7·4 (6·4 to 8·7)	13·3 (12·3 to 14·5)	1·6 (1·1 to 2·3)	10·4 (10·4 to 10·4)	89·6 (85·3 to 94·5)	20·3 (17·6 to 23·9)	36·5 (33·7 to 39·6)	4·4 (2·9 to 6·3)	28·4 (28·4 to 28·4)
Benin*	2012	28·6 (27·2 to 30·1)	7·2 (6·3 to 8·3)	13·0 (12·3 to 13·8)	1·6 (1·1 to 2·3)	6·7 (6·7 to 6·7)	78·1 (74·4 to 82·2)	19·7 (17·2 to 22·8)	35·6 (33·6 to 37·8)	4·4 (2·9 to 6·3)	18·4 (18·4 to 18·4)
Benin*	2013	28·4 (27·1 to 29·8)	7·3 (6·4 to 8·4)	13·1 (12·4 to 13·8)	1·7 (1·2 to 2·4)	6·3 (6·3 to 6·3)	77·6 (74·0 to 81·5)	20·0 (17·5 to 22·9)	35·7 (33·8 to 37·7)	4·7 (3·2 to 6·6)	17·2 (17·2 to 17·2)
Benin*	2014	28·9 (27·6 to 30·2)	6·7 (5·9 to 7·6)	12·7 (12·0 to 13·5)	1·7 (1·1 to 2·4)	7·8 (7·8 to 7·8)	79·1 (75·6 to 82·6)	18·3 (16·1 to 20·9)	34·7 (32·8 to 36·8)	4·6 (3·1 to 6·5)	21·4 (21·4 to 21·4)
Benin*	2015	29·5 (28·0 to 31·1)	7·2 (6·3 to 8·2)	12·9 (12·0 to 13·9)	1·7 (1·1 to 2·4)	7·7 (7·7 to 7·7)	80·8 (76·5 to 85·1)	19·7 (17·1 to 22·5)	35·4 (32·9 to 38·0)	4·7 (3·1 to 6·6)	21·0 (21·0 to 21·0)
Benin*	2016	28·1 (26·3 to 29·7)	6·4 (5·5 to 7·4)	12·3 (11·1 to 13·6)	1·6 (1·1 to 2·2)	7·8 (7·8 to 7·8)	76·8 (72·0 to 81·3)	17·5 (15·0 to 20·2)	33·6 (30·4 to 37·1)	4·4 (2·9 to 6·1)	21·3 (21·3 to 21·3)

Benin*	2017	33·6 (31·4 to 35·9)	7·2 (6·1 to 8·5)	12·8 (11·2 to 14·6)	1·7 (1·1 to 2·4)	11·9 (11·9 to 11·9)	91·8 (86·0 to 98·1)	19·8 (16·8 to 23·2)	35·0 (30·6 to 40·0)	4·7 (3·1 to 6·5)	32·4 (32·4 to 32·4)
Benin*	2018	29·3 (26·9 to 32·1)	6·6 (5·5 to 7·9)	12·5 (10·7 to 14·8)	1·6 (1·1 to 2·3)	8·5 (8·5 to 8·5)	80·2 (73·6 to 87·7)	18·2 (15·1 to 21·7)	34·2 (29·2 to 40·6)	4·5 (3·1 to 6·3)	23·3 (23·3 to 23·3)
Benin*	2019	28·7 (26·3 to 31·5)	6·2 (5·2 to 7·5)	12·9 (11·0 to 15·2)	1·7 (1·2 to 2·4)	7·9 (7·8 to 8·0)	78·5 (71·8 to 86·2)	17·1 (14·2 to 20·6)	35·2 (30·0 to 41·7)	4·7 (3·2 to 6·6)	21·5 (21·3 to 21·8)
Benin*	2020	38·2 (36·0 to 40·9)	13·7 (12·7 to 15·0)	12·1 (10·3 to 14·4)	1·7 (1·1 to 2·3)	10·7 (10·6 to 10·8)	104·6 (98·4 to 111·8)	37·6 (34·8 to 41·0)	33·1 (28·3 to 39·3)	4·6 (3·1 to 6·4)	29·3 (29·0 to 29·6)
Benin*	2021	30·7 (28·3 to 33·2)	6·5 (5·4 to 7·8)	11·4 (9·8 to 13·6)	1·6 (1·1 to 2·3)	11·1 (11·0 to 11·5)	83·9 (77·5 to 90·8)	17·8 (14·8 to 21·4)	31·3 (26·7 to 37·1)	4·4 (3·0 to 6·2)	30·4 (30·1 to 31·5)
Benin*	2022	27·2 (25·0 to 29·8)	5·6 (4·6 to 6·7)	11·6 (9·9 to 13·7)	1·7 (1·1 to 2·3)	8·4 (7·9 to 8·9)	74·3 (68·4 to 81·4)	15·2 (12·5 to 18·3)	31·6 (27·0 to 37·5)	4·6 (3·1 to 6·4)	22·9 (21·7 to 24·3)
Benin*	2023	27·4 (25·1 to 30·0)	5·6 (4·6 to 6·8)	11·7 (10·0 to 13·9)	1·7 (1·2 to 2·4)	8·3 (7·8 to 8·8)	74·9 (68·7 to 82·1)	15·4 (12·7 to 18·7)	32·1 (27·4 to 38·0)	4·7 (3·2 to 6·6)	22·6 (21·3 to 24·2)
Benin*	2024	27·6 (25·3 to 30·2)	5·8 (4·7 to 6·9)	11·9 (10·2 to 14·1)	1·8 (1·2 to 2·5)	8·1 (7·6 to 8·7)	75·4 (69·1 to 82·7)	15·7 (12·8 to 18·9)	32·6 (27·8 to 38·6)	4·9 (3·3 to 6·9)	22·2 (20·8 to 23·9)
Benin*	2025	27·8 (25·4 to 30·6)	5·9 (4·8 to 7·1)	12·1 (10·3 to 14·3)	1·9 (1·3 to 2·6)	8·0 (7·5 to 8·6)	76·0 (69·5 to 83·6)	16·1 (13·1 to 19·5)	33·1 (28·3 to 39·2)	5·1 (3·5 to 7·1)	21·8 (20·4 to 23·5)
Benin*	2026	28·0 (25·6 to 30·8)	6·0 (4·9 to 7·3)	12·3 (10·5 to 14·6)	1·9 (1·3 to 2·7)	7·8 (7·2 to 8·4)	76·6 (69·9 to 84·2)	16·4 (13·3 to 20·1)	33·6 (28·7 to 39·9)	5·3 (3·6 to 7·4)	21·3 (19·8 to 23·0)
Benin*	2027	28·2 (25·8 to 31·1)	6·1 (4·9 to 7·5)	12·5 (10·7 to 14·8)	2·0 (1·4 to 2·8)	7·6 (7·0 to 8·3)	77·2 (70·5 to 85·1)	16·8 (13·5 to 20·6)	34·2 (29·2 to 40·5)	5·5 (3·7 to 7·7)	20·8 (19·3 to 22·6)
Benin*	2028	28·5 (25·9 to 31·5)	6·3 (5·0 to 7·8)	12·7 (10·9 to 15·1)	2·1 (1·4 to 2·9)	7·4 (6·8 to 8·1)	77·9 (70·7 to 86·0)	17·2 (13·7 to 21·2)	34·7 (29·7 to 41·2)	5·7 (3·9 to 8·0)	20·3 (18·7 to 22·2)
Benin*	2029	28·8 (26·1 to 31·8)	6·4 (5·1 to 8·0)	12·9 (11·0 to 15·3)	2·2 (1·5 to 3·0)	7·2 (6·6 to 7·9)	78·6 (71·4 to 87·0)	17·6 (14·0 to 21·9)	35·4 (30·2 to 42·0)	5·9 (4·0 to 8·3)	19·7 (18·0 to 21·6)
Benin*	2030	29·1 (26·2 to 32·2)	6·6 (5·2 to 8·3)	13·2 (11·2 to 15·6)	2·3 (1·5 to 3·2)	7·0 (6·3 to 7·7)	79·5 (71·8 to 88·1)	18·1 (14·4 to 22·6)	36·0 (30·7 to 42·7)	6·2 (4·2 to 8·7)	19·1 (17·4 to 21·0)
Benin*	2031	29·4 (26·5 to 32·6)	6·8 (5·4 to 8·6)	13·4 (11·4 to 15·9)	2·4 (1·6 to 3·3)	6·8 (6·1 to 7·5)	80·4 (72·5 to 89·2)	18·7 (14·6 to 23·4)	36·7 (31·3 to 43·5)	6·5 (4·4 to 9·0)	18·6 (16·8 to 20·5)
Benin*	2032	29·7 (26·9 to 33·1)	7·0 (5·5 to 8·9)	13·7 (11·6 to 16·2)	2·5 (1·7 to 3·4)	6·6 (6·0 to 7·3)	81·4 (73·5 to 90·5)	19·2 (14·9 to 24·3)	37·3 (31·9 to 44·3)	6·7 (4·6 to 9·4)	18·1 (16·3 to 19·9)
Benin*	2033	30·1 (27·2 to 33·6)	7·2 (5·6 to 9·2)	13·9 (11·9 to 16·5)	2·6 (1·7 to 3·6)	6·5 (5·8 to 7·2)	82·4 (74·3 to 91·9)	19·8 (15·4 to 25·2)	38·0 (32·4 to 45·0)	7·0 (4·7 to 9·8)	17·7 (15·9 to 19·6)

Benin*	2034	30·6 (27·5 to 34·1)	7·4 (5·7 to 9·5)	14·1 (12·1 to 16·7)	2·7 (1·8 to 3·8)	6·3 (5·7 to 7·0)	83·6 (75·2 to 93·3)	20·3 (15·6 to 25·9)	38·6 (33·0 to 45·8)	7·3 (4·9 to 10·3)	17·3 (15·5 to 19·2)
Benin*	2035	31·0 (27·8 to 34·7)	7·7 (5·9 to 9·8)	14·4 (12·2 to 17·0)	2·8 (1·9 to 3·9)	6·2 (5·6 to 6·9)	84·9 (76·1 to 95·0)	20·9 (16·0 to 26·7)	39·3 (33·5 to 46·5)	7·6 (5·1 to 10·7)	17·0 (15·2 to 19·0)
Benin*	2036	31·5 (28·3 to 35·3)	7·9 (6·0 to 10·1)	14·6 (12·4 to 17·3)	2·9 (1·9 to 4·1)	6·1 (5·4 to 6·9)	86·2 (77·3 to 96·5)	21·6 (16·5 to 27·6)	40·0 (34·0 to 47·3)	8·0 (5·3 to 11·2)	16·7 (14·9 to 18·7)
Benin*	2037	32·0 (28·7 to 35·9)	8·1 (6·2 to 10·4)	14·9 (12·7 to 17·6)	3·0 (2·0 to 4·3)	6·0 (5·3 to 6·7)	87·6 (78·5 to 98·3)	22·2 (17·0 to 28·4)	40·7 (34·6 to 48·1)	8·3 (5·5 to 11·7)	16·4 (14·6 to 18·4)
Benin*	2038	32·6 (29·2 to 36·5)	8·4 (6·3 to 10·8)	15·1 (12·9 to 17·9)	3·2 (2·1 to 4·5)	5·9 (5·2 to 6·6)	89·2 (79·7 to 99·8)	23·0 (17·3 to 29·5)	41·4 (35·2 to 48·9)	8·7 (5·7 to 12·3)	16·1 (14·4 to 18·1)
Benin*	2039	33·2 (29·5 to 37·3)	8·7 (6·6 to 11·2)	15·4 (13·1 to 18·2)	3·3 (2·2 to 4·7)	5·8 (5·1 to 6·6)	90·8 (80·8 to 102·0)	23·8 (18·0 to 30·6)	42·1 (35·8 to 49·7)	9·1 (5·9 to 12·9)	15·9 (14·0 to 17·9)
Benin*	2040	33·9 (30·1 to 38·1)	9·0 (6·8 to 11·6)	15·7 (13·3 to 18·5)	3·5 (2·3 to 5·0)	5·7 (5·0 to 6·5)	92·6 (82·4 to 104·2)	24·6 (18·5 to 31·8)	42·9 (36·5 to 50·6)	9·5 (6·2 to 13·6)	15·6 (13·8 to 17·9)
Benin*	2041	34·5 (30·6 to 38·9)	9·3 (7·0 to 12·0)	16·0 (13·6 to 18·9)	3·6 (2·3 to 5·2)	5·6 (4·9 to 6·4)	94·5 (83·8 to 106·5)	25·5 (19·2 to 32·7)	43·7 (37·2 to 51·6)	9·9 (6·4 to 14·2)	15·3 (13·5 to 17·5)
Benin*	2042	35·3 (31·2 to 39·8)	9·7 (7·3 to 12·5)	16·3 (13·9 to 19·2)	3·8 (2·4 to 5·4)	5·5 (4·8 to 6·4)	96·5 (85·4 to 109·0)	26·4 (19·9 to 34·2)	44·5 (37·9 to 52·6)	10·4 (6·7 to 14·8)	15·1 (13·1 to 17·4)
Benin*	2043	36·0 (31·8 to 40·8)	10·0 (7·5 to 13·1)	16·6 (14·1 to 19·6)	4·0 (2·5 to 5·7)	5·4 (4·7 to 6·3)	98·5 (86·9 to 111·5)	27·4 (20·5 to 35·9)	45·4 (38·6 to 53·6)	10·9 (6·9 to 15·7)	14·9 (12·9 to 17·3)
Benin*	2044	36·8 (32·4 to 41·7)	10·4 (7·7 to 13·7)	16·9 (14·4 to 20·0)	4·2 (2·6 to 6·0)	5·4 (4·7 to 6·3)	100·7 (88·7 to 114·0)	28·4 (21·0 to 37·4)	46·2 (39·4 to 54·7)	11·4 (7·2 to 16·5)	14·7 (12·8 to 17·2)
Benin*	2045	37·7 (33·2 to 42·9)	10·8 (7·9 to 14·3)	17·2 (14·7 to 20·4)	4·4 (2·7 to 6·4)	5·3 (4·6 to 6·2)	103·0 (90·7 to 117·3)	29·5 (21·6 to 39·1)	47·1 (40·1 to 55·7)	11·9 (7·4 to 17·4)	14·5 (12·5 to 17·0)
Benin*	2046	38·5 (33·7 to 44·0)	11·2 (8·2 to 15·0)	17·6 (14·9 to 20·8)	4·6 (2·8 to 6·7)	5·2 (4·5 to 6·1)	105·4 (92·2 to 120·3)	30·6 (22·4 to 40·9)	48·0 (40·9 to 56·8)	12·5 (7·7 to 18·3)	14·3 (12·2 to 16·8)
Benin*	2047	39·5 (34·5 to 45·1)	11·6 (8·4 to 15·4)	17·9 (15·2 to 21·2)	4·8 (2·9 to 7·1)	5·2 (4·4 to 6·1)	107·9 (94·4 to 123·3)	31·8 (23·0 to 42·2)	48·9 (41·6 to 57·9)	13·1 (8·0 to 19·3)	14·1 (12·1 to 16·7)
Benin*	2048	40·4 (35·3 to 46·2)	12·1 (8·7 to 16·1)	18·2 (15·5 to 21·6)	5·0 (3·0 to 7·4)	5·1 (4·3 to 6·0)	110·5 (96·6 to 126·4)	33·0 (23·9 to 44·2)	49·8 (42·4 to 59·1)	13·7 (8·3 to 20·3)	14·0 (11·9 to 16·5)

Benin*	2049	41·4 (36·2 to 47·5)	12·5 (9·0 to 16·9)	18·5 (15·8 to 22·0)	5·2 (3·1 to 7·8)	5·1 (4·3 to 6·0)	113·2 (98·9 to 129·8)	34·3 (24·6 to 46·2)	50·7 (43·1 to 60·2)	14·3 (8·6 to 21·4)	13·9 (11·8 to 16·4)
Benin*	2050	42·4 (36·9 to 48·6)	13·0 (9·4 to 17·3)	18·9 (16·0 to 22·4)	5·5 (3·2 to 8·2)	5·0 (4·3 to 6·0)	116·0 (100·9 to 133·0)	35·6 (25·6 to 47·3)	51·6 (43·9 to 61·2)	15·0 (8·9 to 22·6)	13·8 (11·7 to 16·5)
Burkina Faso*	1995	16·0 (14·3 to 18·2)	4·2 (3·4 to 5·0)	7·6 (6·0 to 9·5)	0·9 (0·6 to 1·3)	3·3 (3·3 to 3·3)	46·0 (40·9 to 52·2)	12·1 (9·8 to 14·4)	21·7 (17·2 to 27·4)	2·7 (1·9 to 3·9)	9·5 (9·5 to 9·5)
Burkina Faso*	1996	14·2 (12·4 to 16·5)	4·5 (3·7 to 5·4)	7·6 (6·0 to 9·5)	1·0 (0·7 to 1·4)	1·2 (1·2 to 1·2)	40·8 (35·6 to 47·3)	13·0 (10·7 to 15·6)	21·6 (17·3 to 27·2)	2·8 (1·9 to 3·9)	3·3 (3·3 to 3·3)
Burkina Faso*	1997	16·2 (14·4 to 18·5)	4·7 (3·9 to 5·7)	7·4 (5·9 to 9·4)	1·0 (0·7 to 1·4)	3·1 (3·1 to 3·1)	46·5 (41·4 to 53·0)	13·5 (11·1 to 16·2)	21·3 (16·9 to 27·0)	2·7 (1·9 to 3·9)	9·0 (9·0 to 9·0)
Burkina Faso*	1998	16·4 (14·5 to 18·5)	4·9 (4·0 to 5·8)	7·2 (5·7 to 9·1)	0·9 (0·6 to 1·3)	3·4 (3·4 to 3·4)	46·9 (41·7 to 53·0)	13·9 (11·5 to 16·6)	20·7 (16·4 to 26·0)	2·7 (1·8 to 3·8)	9·6 (9·6 to 9·6)
Burkina Faso*	1999	15·2 (13·4 to 17·3)	5·1 (4·2 to 6·1)	6·9 (5·4 to 8·7)	0·8 (0·6 to 1·2)	2·3 (2·3 to 2·3)	43·5 (38·4 to 49·6)	14·5 (11·9 to 17·5)	19·9 (15·5 to 25·0)	2·4 (1·7 to 3·5)	6·7 (6·7 to 6·7)
Burkina Faso*	2000	14·1 (12·4 to 15·9)	4·8 (3·9 to 5·9)	6·2 (4·8 to 7·7)	0·6 (0·4 to 0·9)	2·4 (2·4 to 2·4)	40·3 (35·6 to 45·4)	13·8 (11·3 to 16·8)	17·9 (13·8 to 22·2)	1·8 (1·3 to 2·6)	6·8 (6·8 to 6·8)
Burkina Faso*	2001	15·1 (13·5 to 16·9)	4·9 (4·0 to 6·0)	6·0 (4·6 to 7·5)	0·6 (0·4 to 0·8)	3·6 (3·6 to 3·6)	43·2 (38·6 to 48·4)	14·2 (11·6 to 17·2)	17·1 (13·1 to 21·4)	1·7 (1·2 to 2·3)	10·3 (10·3 to 10·3)
Burkina Faso*	2002	14·8 (13·2 to 16·6)	5·2 (4·3 to 6·3)	5·8 (4·5 to 7·3)	0·6 (0·4 to 0·8)	3·2 (3·2 to 3·2)	42·3 (37·8 to 47·6)	14·9 (12·2 to 17·9)	16·7 (12·9 to 20·9)	1·6 (1·1 to 2·3)	9·1 (9·1 to 9·1)
Burkina Faso*	2003	16·9 (15·3 to 18·7)	5·4 (4·4 to 6·4)	5·8 (4·5 to 7·3)	0·6 (0·4 to 0·8)	5·2 (5·2 to 5·2)	48·4 (43·9 to 53·6)	15·4 (12·7 to 18·3)	16·6 (13·0 to 20·8)	1·6 (1·1 to 2·2)	14·8 (14·8 to 14·8)
Burkina Faso*	2004	19·6 (17·8 to 21·6)	6·5 (5·4 to 7·8)	6·4 (5·1 to 8·0)	0·6 (0·4 to 0·8)	6·0 (6·0 to 6·0)	56·0 (50·9 to 61·9)	18·8 (15·4 to 22·3)	18·4 (14·5 to 22·9)	1·7 (1·2 to 2·3)	17·2 (17·2 to 17·2)
Burkina Faso*	2005	21·2 (19·3 to 23·3)	6·8 (5·6 to 8·1)	6·8 (5·3 to 8·4)	0·6 (0·4 to 0·8)	7·0 (7·0 to 7·0)	60·8 (55·4 to 66·7)	19·6 (16·1 to 23·1)	19·4 (15·2 to 24·2)	1·7 (1·2 to 2·4)	20·1 (20·1 to 20·1)
Burkina Faso*	2006	22·6 (20·5 to 24·7)	7·5 (6·1 to 8·9)	7·4 (5·9 to 9·1)	0·6 (0·4 to 0·9)	7·1 (7·1 to 7·1)	64·6 (58·8 to 70·6)	21·5 (17·4 to 25·4)	21·1 (17·0 to 26·1)	1·8 (1·2 to 2·5)	20·2 (20·2 to 20·2)
Burkina Faso*	2007	23·1 (20·9 to 25·2)	7·9 (6·4 to 9·3)	7·9 (6·4 to 9·6)	0·7 (0·5 to 1·0)	6·6 (6·6 to 6·6)	66·1 (59·9 to 72·3)	22·5 (18·5 to 26·5)	22·5 (18·5 to 27·4)	2·1 (1·4 to 2·8)	18·9 (18·9 to 18·9)
Burkina Faso*	2008	24·3 (22·4 to 26·4)	7·2 (5·9 to 8·5)	7·9 (6·6 to 9·4)	0·9 (0·6 to 1·2)	8·4 (8·4 to 8·4)	69·7 (64·2 to 75·7)	20·6 (17·0 to 24·4)	22·7 (18·8 to 26·9)	2·4 (1·7 to 3·4)	24·0 (24·0 to 24·0)
Burkina Faso*	2009	25·9 (24·0 to 28·0)	8·0 (6·6 to 9·3)	8·6 (7·3 to 10·0)	1·2 (0·8 to 1·6)	8·2 (8·2 to 8·2)	74·3 (68·7 to 80·2)	22·8 (19·0 to 26·7)	24·6 (21·0 to 28·6)	3·4 (2·3 to 4·7)	23·5 (23·5 to 23·5)

Burkina Faso*	2010	27·9 (26·1 to 29·7)	8·0 (6·8 to 9·4)	8·9 (7·9 to 10·1)	1·3 (0·9 to 1·8)	9·6 (9·6 to 9·6)	79·8 (74·8 to 85·0)	23·0 (19·4 to 26·8)	25·6 (22·6 to 29·1)	3·6 (2·5 to 5·1)	27·6 (27·6 to 27·6)
Burkina Faso*	2011	24·0 (22·5 to 25·5)	7·9 (6·7 to 9·1)	9·1 (8·2 to 10·1)	1·5 (1·0 to 2·2)	5·5 (5·5 to 5·5)	68·7 (64·4 to 73·1)	22·5 (19·2 to 26·0)	26·1 (23·6 to 28·9)	4·3 (2·9 to 6·2)	15·7 (15·7 to 15·7)
Burkina Faso*	2012	28·4 (26·9 to 30·0)	8·6 (7·4 to 9·9)	9·9 (9·1 to 10·6)	1·9 (1·3 to 2·7)	8·1 (8·1 to 8·1)	81·4 (77·1 to 85·9)	24·6 (21·3 to 28·4)	28·2 (26·1 to 30·5)	5·4 (3·7 to 7·7)	23·1 (23·1 to 23·1)
Burkina Faso*	2013	31·5 (29·9 to 33·3)	10·4 (9·1 to 11·7)	11·2 (10·4 to 12·0)	2·5 (1·7 to 3·5)	7·5 (7·5 to 7·5)	90·3 (85·6 to 95·4)	29·7 (26·0 to 33·4)	32·0 (29·8 to 34·4)	7·3 (4·9 to 10·0)	21·3 (21·3 to 21·3)
Burkina Faso*	2014	31·7 (30·1 to 33·4)	10·3 (9·2 to 11·5)	11·2 (10·5 to 12·0)	2·8 (1·9 to 3·9)	7·4 (7·4 to 7·4)	90·7 (86·3 to 95·7)	29·5 (26·3 to 32·9)	32·1 (30·2 to 34·2)	7·9 (5·5 to 11·0)	21·1 (21·1 to 21·1)
Burkina Faso*	2015	33·1 (31·5 to 34·8)	11·1 (9·9 to 12·2)	11·6 (10·9 to 12·3)	2·8 (1·9 to 4·0)	7·6 (7·6 to 7·6)	94·9 (90·4 to 99·8)	31·8 (28·5 to 35·1)	33·2 (31·2 to 35·2)	8·1 (5·5 to 11·3)	21·8 (21·8 to 21·8)
Burkina Faso*	2016	35·3 (33·6 to 37·1)	13·6 (12·3 to 14·9)	12·2 (11·5 to 12·9)	2·5 (1·7 to 3·5)	7·0 (7·0 to 7·0)	101·2 (96·3 to 106·3)	38·9 (35·2 to 42·6)	34·9 (32·9 to 37·0)	7·2 (4·9 to 10·0)	20·1 (20·1 to 20·1)
Burkina Faso*	2017	39·6 (37·6 to 41·6)	16·3 (14·9 to 18·0)	12·9 (12·1 to 13·7)	2·7 (1·9 to 3·8)	7·7 (7·7 to 7·7)	113·5 (107·9 to 119·3)	46·8 (42·7 to 51·7)	36·8 (34·6 to 39·2)	7·8 (5·5 to 10·9)	22·0 (22·0 to 22·0)
Burkina Faso*	2018	40·7 (38·8 to 42·8)	15·7 (14·1 to 17·5)	12·5 (11·6 to 13·5)	2·5 (1·7 to 3·5)	10·0 (10·0 to 10·0)	116·6 (111·3 to 122·6)	45·1 (40·5 to 50·1)	35·8 (33·1 to 38·7)	7·1 (5·0 to 9·9)	28·7 (28·7 to 28·7)
Burkina Faso*	2019	41·4 (39·4 to 43·7)	16·4 (14·7 to 18·3)	12·8 (11·9 to 13·9)	2·6 (1·8 to 3·6)	9·6 (9·6 to 9·7)	118·7 (112·8 to 125·2)	47·0 (42·0 to 52·5)	36·7 (34·0 to 39·8)	7·4 (5·2 to 10·4)	27·6 (27·4 to 27·8)
Burkina Faso*	2020	56·3 (54·3 to 58·5)	28·8 (27·1 to 30·6)	12·1 (11·2 to 13·1)	2·5 (1·7 to 3·5)	12·9 (12·8 to 13·1)	161·2 (155·5 to 167·6)	82·4 (77·5 to 87·7)	34·7 (32·1 to 37·5)	7·1 (5·0 to 10·0)	37·0 (36·7 to 37·6)
Burkina Faso*	2021	44·4 (42·2 to 46·9)	17·4 (15·5 to 19·6)	11·5 (10·6 to 12·5)	2·4 (1·7 to 3·4)	13·1 (12·9 to 13·5)	127·2 (121·0 to 134·4)	49·8 (44·4 to 56·1)	33·0 (30·5 to 35·7)	6·9 (4·9 to 9·8)	37·5 (37·1 to 38·8)
Burkina Faso*	2022	39·7 (37·5 to 42·1)	15·7 (14·0 to 17·7)	11·6 (10·8 to 12·6)	2·5 (1·8 to 3·5)	9·8 (9·3 to 10·5)	113·7 (107·5 to 120·5)	45·0 (40·2 to 50·7)	33·4 (30·9 to 36·1)	7·2 (5·0 to 10·2)	28·2 (26·7 to 30·0)
Burkina Faso*	2023	40·3 (38·0 to 42·8)	16·2 (14·5 to 18·3)	11·8 (10·9 to 12·8)	2·6 (1·8 to 3·7)	9·7 (9·2 to 10·4)	115·5 (109·0 to 122·6)	46·4 (41·5 to 52·6)	33·8 (31·3 to 36·6)	7·4 (5·2 to 10·5)	27·9 (26·2 to 29·8)
Burkina Faso*	2024	40·9 (38·5 to 43·5)	16·7 (14·8 to 18·9)	11·9 (11·1 to 12·9)	2·7 (1·9 to 3·8)	9·6 (9·0 to 10·3)	117·2 (110·4 to 124·8)	47·7 (42·5 to 54·3)	34·2 (31·7 to 37·1)	7·7 (5·4 to 10·9)	27·5 (25·8 to 29·6)

Burkina Faso*	2025	41·4 (38·9 to 44·1)	17·1 (15·1 to 19·4)	12·1 (11·2 to 13·1)	2·8 (1·9 to 3·9)	9·4 (8·8 to 10·2)	118·6 (111·5 to 126·4)	48·9 (43·2 to 55·6)	34·7 (32·1 to 37·6)	8·0 (5·6 to 11·2)	27·0 (25·3 to 29·1)
Burkina Faso*	2026	41·9 (39·3 to 44·8)	17·5 (15·5 to 20·0)	12·3 (11·4 to 13·3)	2·9 (2·0 to 4·1)	9·2 (8·6 to 10·0)	120·1 (112·6 to 128·2)	50·1 (44·3 to 57·3)	35·2 (32·6 to 38·1)	8·3 (5·8 to 11·7)	26·5 (24·6 to 28·7)
Burkina Faso*	2027	42·5 (39·7 to 45·5)	18·0 (15·8 to 20·6)	12·5 (11·5 to 13·5)	3·0 (2·1 to 4·2)	9·1 (8·4 to 9·8)	121·7 (113·8 to 130·5)	51·5 (45·2 to 59·0)	35·7 (33·0 to 38·6)	8·6 (6·0 to 12·1)	25·9 (24·0 to 28·2)
Burkina Faso*	2028	43·2 (40·5 to 46·4)	18·6 (16·4 to 21·4)	12·6 (11·7 to 13·7)	3·1 (2·1 to 4·4)	8·9 (8·1 to 9·7)	123·9 (115·9 to 132·9)	53·4 (47·0 to 61·2)	36·2 (33·5 to 39·2)	8·9 (6·1 to 12·6)	25·4 (23·3 to 27·7)
Burkina Faso*	2029	44·0 (41·1 to 47·2)	19·3 (17·0 to 22·2)	12·8 (11·9 to 13·9)	3·2 (2·2 to 4·6)	8·6 (7·9 to 9·5)	126·1 (117·8 to 135·3)	55·3 (48·6 to 63·6)	36·8 (34·0 to 39·8)	9·3 (6·4 to 13·1)	24·7 (22·6 to 27·1)
Burkina Faso*	2030	44·7 (41·8 to 48·1)	20·0 (17·6 to 23·0)	13·0 (12·0 to 14·1)	3·4 (2·3 to 4·8)	8·4 (7·6 to 9·2)	128·2 (119·6 to 137·8)	57·2 (50·3 to 65·9)	37·3 (34·5 to 40·4)	9·6 (6·6 to 13·6)	24·0 (21·8 to 26·4)
Burkina Faso*	2031	45·6 (42·5 to 49·1)	20·7 (18·2 to 23·9)	13·2 (12·2 to 14·3)	3·5 (2·4 to 4·9)	8·2 (7·4 to 9·0)	130·6 (121·7 to 140·6)	59·3 (52·3 to 68·4)	37·9 (35·0 to 41·0)	10·0 (6·8 to 14·2)	23·4 (21·1 to 25·8)
Burkina Faso*	2032	46·5 (43·3 to 50·2)	21·5 (18·9 to 24·7)	13·4 (12·4 to 14·5)	3·6 (2·5 to 5·1)	7·9 (7·2 to 8·8)	133·2 (124·1 to 143·8)	61·6 (54·2 to 70·9)	38·5 (35·6 to 41·6)	10·4 (7·1 to 14·7)	22·7 (20·5 to 25·2)
Burkina Faso*	2033	47·5 (44·1 to 51·3)	22·3 (19·6 to 25·7)	13·6 (12·6 to 14·7)	3·8 (2·6 to 5·3)	7·7 (6·9 to 8·6)	136·0 (126·4 to 146·9)	63·9 (56·2 to 73·7)	39·1 (36·1 to 42·2)	10·8 (7·5 to 15·3)	22·1 (19·8 to 24·5)
Burkina Faso*	2034	48·5 (45·0 to 52·4)	23·1 (20·3 to 26·7)	13·9 (12·8 to 15·0)	3·9 (2·7 to 5·6)	7·5 (6·7 to 8·4)	138·8 (128·8 to 150·2)	66·3 (58·2 to 76·6)	39·7 (36·7 to 42·9)	11·2 (7·8 to 15·9)	21·6 (19·3 to 24·0)
Burkina Faso*	2035	49·5 (45·9 to 53·7)	24·0 (21·0 to 27·8)	14·1 (13·0 to 15·2)	4·1 (2·8 to 5·8)	7·3 (6·6 to 8·2)	141·8 (131·5 to 153·7)	68·8 (60·2 to 79·6)	40·3 (37·3 to 43·6)	11·7 (8·1 to 16·6)	21·0 (18·8 to 23·5)
Burkina Faso*	2036	50·6 (46·8 to 55·0)	24·9 (21·7 to 28·8)	14·3 (13·2 to 15·4)	4·2 (2·9 to 6·0)	7·2 (6·4 to 8·0)	144·9 (134·0 to 157·5)	71·2 (62·1 to 82·6)	40·9 (37·8 to 44·2)	12·2 (8·4 to 17·2)	20·5 (18·3 to 23·0)
Burkina Faso*	2037	51·7 (47·8 to 56·3)	25·7 (22·4 to 30·0)	14·5 (13·4 to 15·7)	4·4 (3·0 to 6·3)	7·0 (6·2 to 7·9)	148·0 (136·8 to 161·3)	73·8 (64·2 to 85·9)	41·6 (38·4 to 44·9)	12·6 (8·7 to 17·9)	20·0 (17·9 to 22·5)
Burkina Faso*	2038	52·8 (48·7 to 57·5)	26·7 (23·2 to 31·2)	14·7 (13·6 to 15·9)	4·6 (3·1 to 6·5)	6·8 (6·1 to 7·7)	151·3 (139·4 to 164·9)	76·4 (66·4 to 89·3)	42·2 (39·0 to 45·6)	13·1 (9·0 to 18·6)	19·5 (17·4 to 22·0)

Burkina Faso*	2039	54·0 (49·7 to 58·9)	27·6 (23·9 to 32·3)	15·0 (13·8 to 16·2)	4·8 (3·3 to 6·8)	6·7 (5·9 to 7·5)	154·7 (142·5 to 168·6)	79·1 (68·6 to 92·5)	42·9 (39·7 to 46·3)	13·7 (9·3 to 19·6)	19·1 (17·0 to 21·6)
Burkina Faso*	2040	55·3 (50·7 to 60·4)	28·6 (24·7 to 33·5)	15·2 (14·1 to 16·4)	5·0 (3·4 to 7·2)	6·5 (5·7 to 7·4)	158·3 (145·3 to 173·0)	81·8 (70·8 to 96·0)	43·6 (40·3 to 47·1)	14·2 (9·7 to 20·5)	18·6 (16·5 to 21·3)
Burkina Faso*	2041	56·5 (51·8 to 61·9)	29·5 (25·5 to 34·7)	15·5 (14·3 to 16·7)	5·2 (3·5 to 7·5)	6·3 (5·6 to 7·3)	162·0 (148·4 to 177·4)	84·6 (73·1 to 99·5)	44·4 (41·0 to 47·9)	14·8 (10·0 to 21·5)	18·2 (16·0 to 20·8)
Burkina Faso*	2042	57·9 (53·0 to 63·5)	30·6 (26·3 to 36·0)	15·7 (14·6 to 17·0)	5·4 (3·6 to 7·8)	6·2 (5·4 to 7·2)	165·8 (151·7 to 182·0)	87·6 (75·4 to 103·2)	45·1 (41·7 to 48·7)	15·4 (10·4 to 22·5)	17·7 (15·4 to 20·5)
Burkina Faso*	2043	59·3 (54·2 to 65·1)	31·6 (27·2 to 37·3)	16·0 (14·8 to 17·3)	5·6 (3·8 to 8·2)	6·0 (5·2 to 7·0)	169·8 (155·2 to 186·5)	90·6 (77·9 to 107·0)	45·9 (42·5 to 49·5)	16·1 (10·7 to 23·5)	17·3 (15·0 to 20·1)
Burkina Faso*	2044	60·7 (55·4 to 66·7)	32·7 (28·0 to 38·7)	16·3 (15·1 to 17·6)	5·8 (3·9 to 8·6)	5·9 (5·1 to 6·9)	174·0 (158·7 to 191·1)	93·6 (80·3 to 110·9)	46·8 (43·3 to 50·4)	16·7 (11·2 to 24·6)	16·9 (14·7 to 19·7)
Burkina Faso*	2045	62·2 (56·6 to 68·5)	33·8 (28·9 to 39·9)	16·6 (15·4 to 17·9)	6·1 (4·0 to 9·0)	5·7 (5·0 to 6·7)	178·2 (162·2 to 196·2)	96·7 (82·9 to 114·4)	47·6 (44·1 to 51·4)	17·4 (11·6 to 25·7)	16·4 (14·2 to 19·3)
Burkina Faso*	2046	63·7 (57·9 to 70·4)	34·9 (29·9 to 41·3)	16·9 (15·7 to 18·3)	6·3 (4·2 to 9·4)	5·6 (4·8 to 6·6)	182·6 (166·0 to 201·7)	99·9 (85·6 to 118·3)	48·5 (44·9 to 52·3)	18·2 (12·0 to 26·9)	16·0 (13·7 to 18·8)
Burkina Faso*	2047	65·3 (59·4 to 72·2)	36·0 (30·8 to 42·6)	17·2 (16·0 to 18·6)	6·6 (4·4 to 9·8)	5·4 (4·6 to 6·4)	187·1 (170·1 to 206·9)	103·2 (88·3 to 122·1)	49·4 (45·7 to 53·3)	19·0 (12·5 to 28·2)	15·6 (13·3 to 18·3)
Burkina Faso*	2048	66·9 (60·8 to 74·2)	37·2 (31·8 to 44·0)	17·6 (16·3 to 19·0)	6·9 (4·5 to 10·3)	5·3 (4·5 to 6·2)	191·8 (174·2 to 212·6)	106·5 (91·1 to 126·0)	50·4 (46·6 to 54·4)	19·8 (13·0 to 29·6)	15·1 (12·9 to 17·9)
Burkina Faso*	2049	68·6 (62·4 to 76·2)	38·4 (32·8 to 45·4)	17·9 (16·6 to 19·3)	7·2 (4·7 to 10·9)	5·1 (4·4 to 6·1)	196·6 (178·7 to 218·3)	109·9 (93·9 to 130·2)	51·3 (47·5 to 55·4)	20·7 (13·5 to 31·1)	14·7 (12·5 to 17·4)
Burkina Faso*	2050	70·3 (63·7 to 78·0)	39·5 (33·8 to 46·9)	18·3 (16·9 to 19·7)	7·5 (4·9 to 11·4)	5·0 (4·3 to 6·0)	201·5 (182·6 to 223·5)	113·3 (96·8 to 134·3)	52·3 (48·4 to 56·5)	21·6 (14·0 to 32·7)	14·4 (12·2 to 17·2)
Bangladesh*	1995	17·1 (14·6 to 19·9)	4·4 (3·6 to 5·2)	11·1 (8·9 to 13·7)	0·6 (0·4 to 0·8)	1·0 (1·0 to 1·0)	45·6 (38·9 to 53·2)	11·7 (9·5 to 14·0)	29·6 (23·7 to 36·5)	1·6 (1·1 to 2·2)	2·8 (2·8 to 2·8)
Bangladesh*	1996	16·0 (13·7 to 18·4)	4·1 (3·4 to 5·0)	10·4 (8·4 to 12·8)	0·6 (0·4 to 0·8)	0·9 (0·9 to 0·9)	42·7 (36·7 to 49·3)	11·0 (9·0 to 13·3)	27·7 (22·5 to 34·1)	1·6 (1·1 to 2·2)	2·4 (2·4 to 2·4)
Bangladesh*	1997	15·2 (13·4 to 17·2)	4·0 (3·3 to 4·9)	9·4 (7·8 to 11·3)	0·6 (0·4 to 0·8)	1·2 (1·2 to 1·2)	40·6 (35·8 to 46·1)	10·7 (8·8 to 13·0)	25·2 (20·9 to 30·1)	1·6 (1·1 to 2·3)	3·1 (3·1 to 3·1)

Bangladesh*	1998	16·1 (14·6 to 17·8)	3·9 (3·2 to 4·7)	9·2 (7·8 to 10·7)	0·6 (0·4 to 0·8)	2·4 (2·4 to 2·4)	43·1 (39·0 to 47·6)	10·4 (8·5 to 12·6)	24·6 (20·9 to 28·5)	1·6 (1·1 to 2·3)	6·5 (6·5 to 6·5)
Bangladesh*	1999	15·9 (14·7 to 17·3)	3·7 (3·0 to 4·5)	9·2 (8·1 to 10·3)	0·6 (0·4 to 0·8)	2·4 (2·4 to 2·4)	42·6 (39·2 to 46·3)	9·9 (8·1 to 11·9)	24·5 (21·7 to 27·6)	1·6 (1·1 to 2·3)	6·5 (6·5 to 6·5)
Bangladesh*	2000	16·1 (15·0 to 17·3)	4·0 (3·3 to 4·8)	9·7 (8·8 to 10·6)	0·5 (0·3 to 0·7)	2·0 (2·0 to 2·0)	43·0 (40·1 to 46·3)	10·7 (8·8 to 12·9)	25·8 (23·6 to 28·3)	1·3 (0·9 to 1·8)	5·2 (5·2 to 5·2)
Bangladesh*	2001	16·8 (15·7 to 18·0)	4·2 (3·5 to 5·1)	10·4 (9·7 to 11·2)	0·5 (0·3 to 0·7)	1·7 (1·7 to 1·7)	45·0 (42·0 to 48·0)	11·3 (9·3 to 13·7)	27·9 (25·9 to 29·9)	1·3 (0·9 to 1·8)	4·5 (4·5 to 4·5)
Bangladesh*	2002	17·3 (16·2 to 18·4)	4·4 (3·6 to 5·4)	11·1 (10·4 to 11·8)	0·5 (0·4 to 0·7)	1·2 (1·2 to 1·2)	46·2 (43·3 to 49·2)	11·8 (9·6 to 14·3)	29·8 (27·9 to 31·7)	1·4 (1·0 to 1·9)	3·3 (3·3 to 3·3)
Bangladesh*	2003	18·4 (17·3 to 19·6)	4·6 (3·8 to 5·6)	12·0 (11·3 to 12·7)	0·6 (0·4 to 0·8)	1·2 (1·2 to 1·2)	49·2 (46·1 to 52·4)	12·4 (10·0 to 15·0)	32·1 (30·2 to 33·9)	1·5 (1·1 to 2·1)	3·2 (3·2 to 3·2)
Bangladesh*	2004	19·6 (18·4 to 20·7)	4·7 (3·9 to 5·7)	12·9 (12·2 to 13·6)	0·6 (0·4 to 0·8)	1·3 (1·3 to 1·3)	52·3 (49·2 to 55·3)	12·7 (10·3 to 15·3)	34·5 (32·6 to 36·5)	1·7 (1·2 to 2·3)	3·4 (3·4 to 3·4)
Bangladesh*	2005	21·7 (20·5 to 22·9)	5·0 (4·1 to 6·1)	14·2 (13·5 to 14·9)	0·7 (0·5 to 1·0)	1·7 (1·7 to 1·7)	57·9 (54·7 to 61·3)	13·4 (11·0 to 16·4)	38·0 (36·1 to 39·9)	1·9 (1·4 to 2·6)	4·5 (4·5 to 4·5)
Bangladesh*	2006	22·9 (21·7 to 24·3)	5·3 (4·4 to 6·4)	15·5 (14·8 to 16·3)	0·8 (0·6 to 1·1)	1·3 (1·3 to 1·3)	61·3 (58·0 to 65·0)	14·1 (11·6 to 17·1)	41·5 (39·5 to 43·5)	2·1 (1·5 to 2·8)	3·6 (3·6 to 3·6)
Bangladesh*	2007	23·7 (22·4 to 25·0)	5·4 (4·4 to 6·5)	16·6 (15·9 to 17·4)	0·8 (0·6 to 1·1)	0·9 (0·9 to 0·9)	63·3 (59·9 to 66·8)	14·4 (11·8 to 17·4)	44·4 (42·5 to 46·4)	2·1 (1·6 to 2·9)	2·4 (2·4 to 2·4)
Bangladesh*	2008	26·8 (25·6 to 28·3)	6·1 (5·0 to 7·3)	18·3 (17·6 to 19·2)	0·8 (0·6 to 1·1)	1·6 (1·6 to 1·6)	71·7 (68·3 to 75·5)	16·2 (13·4 to 19·5)	49·0 (46·9 to 51·2)	2·2 (1·6 to 3·0)	4·3 (4·3 to 4·3)
Bangladesh*	2009	27·6 (26·3 to 29·0)	5·9 (4·9 to 7·2)	19·2 (18·4 to 20·0)	0·8 (0·6 to 1·1)	1·7 (1·7 to 1·7)	73·8 (70·3 to 77·5)	15·9 (13·2 to 19·2)	51·3 (49·2 to 53·5)	2·2 (1·6 to 3·0)	4·5 (4·5 to 4·5)
Bangladesh*	2010	29·6 (28·3 to 31·1)	6·2 (5·2 to 7·5)	20·6 (19·9 to 21·4)	0·8 (0·6 to 1·1)	1·9 (1·9 to 1·9)	79·0 (75·6 to 83·1)	16·6 (13·8 to 20·0)	55·2 (53·1 to 57·3)	2·2 (1·6 to 3·0)	5·0 (5·0 to 5·0)
Bangladesh*	2011	31·9 (30·5 to 33·6)	6·8 (5·7 to 8·3)	22·5 (21·7 to 23·3)	0·9 (0·7 to 1·3)	1·6 (1·6 to 1·6)	85·2 (81·4 to 89·8)	18·3 (15·3 to 22·0)	60·1 (58·0 to 62·3)	2·5 (1·8 to 3·3)	4·3 (4·3 to 4·3)
Bangladesh*	2012	34·1 (32·6 to 35·8)	7·1 (5·8 to 8·5)	23·7 (22·8 to 24·5)	0·9 (0·7 to 1·3)	2·4 (2·4 to 2·4)	91·0 (87·0 to 95·5)	18·8 (15·6 to 22·7)	63·2 (61·0 to 65·5)	2·5 (1·8 to 3·4)	6·5 (6·5 to 6·5)
Bangladesh*	2013	36·0 (34·5 to 37·7)	7·2 (6·0 to 8·8)	24·9 (24·0 to 25·8)	0·9 (0·7 to 1·2)	3·0 (3·0 to 3·0)	96·2 (92·2 to 100·8)	19·3 (16·0 to 23·6)	66·5 (64·2 to 68·8)	2·5 (1·8 to 3·3)	8·0 (8·0 to 8·0)
Bangladesh*	2014	36·5 (35·1 to 38·2)	7·1 (5·9 to 8·6)	26·3 (25·4 to 27·1)	0·9 (0·7 to 1·3)	2·2 (2·2 to 2·2)	97·6 (93·7 to 102·1)	19·0 (15·8 to 23·1)	70·2 (67·9 to 72·5)	2·5 (1·8 to 3·5)	6·0 (6·0 to 6·0)

Bangladesh*	2015	38·2 (36·5 to 40·1)	7·0 (5·8 to 8·5)	27·9 (26·8 to 29·1)	1·0 (0·7 to 1·4)	2·2 (2·2 to 2·2)	102·1 (97·4 to 107·1)	18·8 (15·5 to 22·7)	74·7 (71·5 to 77·8)	2·6 (1·9 to 3·6)	6·0 (6·0 to 6·0)
Bangladesh*	2016	39·6 (37·2 to 42·3)	7·0 (5·8 to 8·3)	29·4 (27·1 to 31·8)	1·0 (0·8 to 1·4)	2·3 (2·3 to 2·3)	105·9 (99·3 to 113·1)	18·6 (15·4 to 22·3)	78·4 (72·4 to 84·9)	2·8 (2·0 to 3·8)	6·0 (6·0 to 6·0)
Bangladesh*	2017	42·3 (38·5 to 46·5)	7·3 (6·0 to 8·7)	31·2 (27·7 to 35·0)	1·1 (0·8 to 1·5)	2·7 (2·7 to 2·7)	112·9 (102·8 to 124·4)	19·5 (16·2 to 23·3)	83·3 (74·0 to 93·6)	3·0 (2·2 to 4·1)	7·1 (7·1 to 7·1)
Bangladesh*	2018	45·4 (40·7 to 51·2)	8·0 (6·6 to 9·5)	33·6 (29·0 to 39·1)	1·2 (0·8 to 1·7)	2·6 (2·6 to 2·6)	121·2 (108·6 to 136·7)	21·3 (17·5 to 25·5)	89·8 (77·4 to 104·5)	3·2 (2·3 to 4·5)	6·8 (6·8 to 6·8)
Bangladesh*	2019	48·3 (43·2 to 54·2)	8·8 (7·3 to 10·7)	35·9 (30·9 to 41·8)	1·3 (0·9 to 1·8)	2·2 (2·1 to 2·3)	129·1 (115·4 to 144·8)	23·6 (19·4 to 28·5)	96·0 (82·7 to 111·6)	3·6 (2·5 to 4·9)	5·9 (5·7 to 6·0)
Bangladesh*	2020	48·7 (43·4 to 54·4)	10·0 (8·5 to 11·8)	34·4 (29·7 to 40·0)	1·3 (0·9 to 1·8)	2·9 (2·8 to 3·0)	130·0 (116·1 to 145·4)	26·8 (22·6 to 31·6)	92·0 (79·3 to 106·9)	3·5 (2·5 to 4·8)	7·7 (7·5 to 7·9)
Bangladesh*	2021	47·0 (41·8 to 52·9)	8·5 (6·9 to 10·3)	34·7 (29·9 to 40·3)	1·4 (0·9 to 1·9)	2·5 (2·4 to 2·6)	125·5 (111·6 to 141·3)	22·7 (18·5 to 27·4)	92·7 (79·8 to 107·7)	3·6 (2·5 to 5·0)	6·6 (6·4 to 7·0)
Bangladesh*	2022	47·3 (42·0 to 53·4)	8·7 (7·1 to 10·5)	35·4 (30·5 to 41·1)	1·4 (1·0 to 2·0)	1·9 (1·7 to 2·0)	126·5 (112·3 to 142·6)	23·1 (19·0 to 28·1)	94·5 (81·6 to 109·9)	3·8 (2·7 to 5·3)	5·0 (4·6 to 5·5)
Bangladesh*	2023	48·3 (43·0 to 54·3)	8·8 (7·3 to 10·7)	36·1 (31·2 to 42·0)	1·5 (1·0 to 2·1)	1·9 (1·7 to 2·1)	129·1 (114·9 to 145·1)	23·6 (19·4 to 28·7)	96·5 (83·3 to 112·3)	4·0 (2·8 to 5·5)	5·0 (4·6 to 5·5)
Bangladesh*	2024	49·3 (43·7 to 55·5)	9·0 (7·4 to 10·9)	36·9 (31·9 to 42·9)	1·6 (1·1 to 2·2)	1·9 (1·7 to 2·0)	131·8 (116·9 to 148·3)	24·0 (19·8 to 29·2)	98·6 (85·2 to 114·7)	4·2 (2·9 to 5·8)	5·0 (4·5 to 5·5)
Bangladesh*	2025	50·4 (45·0 to 56·4)	9·2 (7·5 to 11·1)	37·7 (32·6 to 43·8)	1·6 (1·1 to 2·3)	1·8 (1·6 to 2·0)	134·5 (120·4 to 150·6)	24·5 (20·1 to 29·7)	100·8 (87·1 to 117·1)	4·4 (3·1 to 6·0)	4·9 (4·4 to 5·5)
Bangladesh*	2026	51·4 (45·8 to 57·9)	9·3 (7·6 to 11·4)	38·5 (33·3 to 44·8)	1·7 (1·2 to 2·4)	1·8 (1·6 to 2·0)	137·3 (122·4 to 154·8)	24·9 (20·4 to 30·4)	103·0 (89·0 to 119·7)	4·6 (3·2 to 6·3)	4·8 (4·3 to 5·4)
Bangladesh*	2027	52·4 (46·5 to 59·1)	9·5 (7·8 to 11·6)	39·4 (34·0 to 45·7)	1·8 (1·2 to 2·5)	1·8 (1·6 to 2·0)	140·1 (124·3 to 157·8)	25·4 (20·7 to 31·0)	105·1 (90·8 to 122·2)	4·8 (3·3 to 6·6)	4·8 (4·2 to 5·4)
Bangladesh*	2028	53·5 (47·8 to 60·3)	9·7 (7·9 to 11·8)	40·2 (34·7 to 46·7)	1·9 (1·3 to 2·6)	1·8 (1·5 to 2·1)	142·8 (127·6 to 161·2)	25·8 (21·1 to 31·5)	107·3 (92·6 to 124·7)	5·0 (3·5 to 6·9)	4·7 (4·1 to 5·5)

Bangladesh*	2029	54·5 (48·5 to 61·5)	9·8 (8·0 to 12·0)	41·0 (35·3 to 47·6)	1·9 (1·4 to 2·7)	1·8 (1·5 to 2·1)	145·6 (129·5 to 164·4)	26·3 (21·4 to 32·0)	109·5 (94·4 to 127·2)	5·2 (3·6 to 7·2)	4·7 (4·1 to 5·5)
Bangladesh*	2030	55·6 (49·3 to 62·8)	10·0 (8·1 to 12·2)	41·8 (36·0 to 48·5)	2·0 (1·4 to 2·8)	1·7 (1·5 to 2·1)	148·4 (131·8 to 167·8)	26·7 (21·7 to 32·6)	111·7 (96·3 to 129·7)	5·4 (3·8 to 7·5)	4·6 (4·0 to 5·5)
Bangladesh*	2031	56·6 (50·3 to 64·0)	10·1 (8·2 to 12·4)	42·6 (36·7 to 49·5)	2·1 (1·5 to 2·9)	1·7 (1·5 to 2·1)	151·3 (134·4 to 170·9)	27·1 (22·0 to 33·2)	113·9 (98·1 to 132·2)	5·6 (3·9 to 7·8)	4·6 (3·9 to 5·6)
Bangladesh*	2032	57·7 (51·3 to 65·0)	10·3 (8·4 to 12·6)	43·5 (37·4 to 50·4)	2·2 (1·5 to 3·0)	1·7 (1·4 to 2·1)	154·0 (137·0 to 173·7)	27·5 (22·3 to 33·8)	116·1 (100·0 to 134·7)	5·8 (4·1 to 8·1)	4·6 (3·8 to 5·6)
Bangladesh*	2033	58·7 (52·2 to 66·4)	10·5 (8·5 to 12·9)	44·3 (38·1 to 51·3)	2·3 (1·6 to 3·2)	1·7 (1·4 to 2·1)	156·9 (139·4 to 177·3)	28·0 (22·6 to 34·4)	118·3 (101·9 to 137·1)	6·1 (4·2 to 8·4)	4·6 (3·8 to 5·6)
Bangladesh*	2034	59·8 (53·0 to 67·5)	10·6 (8·6 to 13·1)	45·1 (38·8 to 52·2)	2·4 (1·6 to 3·3)	1·7 (1·4 to 2·1)	159·7 (141·5 to 180·3)	28·4 (22·8 to 35·0)	120·4 (103·6 to 139·6)	6·3 (4·4 to 8·8)	4·6 (3·8 to 5·7)
Bangladesh*	2035	60·8 (53·9 to 68·7)	10·8 (8·7 to 13·4)	45·9 (39·5 to 53·2)	2·4 (1·7 to 3·4)	1·7 (1·4 to 2·2)	162·6 (144·0 to 183·5)	28·9 (23·2 to 35·7)	122·6 (105·5 to 142·1)	6·5 (4·5 to 9·1)	4·6 (3·7 to 5·8)
Bangladesh*	2036	61·9 (54·8 to 70·2)	11·0 (8·8 to 13·6)	46·7 (40·2 to 54·2)	2·5 (1·8 to 3·5)	1·7 (1·4 to 2·2)	165·4 (146·3 to 187·7)	29·3 (23·5 to 36·3)	124·7 (107·3 to 144·7)	6·8 (4·7 to 9·5)	4·6 (3·7 to 5·9)
Bangladesh*	2037	63·0 (55·8 to 71·3)	11·1 (8·9 to 13·8)	47·4 (40·8 to 55·1)	2·6 (1·8 to 3·7)	1·7 (1·4 to 2·2)	168·2 (149·0 to 190·6)	29·8 (23·9 to 37·0)	126·7 (109·0 to 147·3)	7·0 (4·9 to 9·9)	4·6 (3·7 to 6·0)
Bangladesh*	2038	64·0 (56·9 to 72·6)	11·3 (9·1 to 14·1)	48·2 (41·4 to 56·0)	2·7 (1·9 to 3·8)	1·7 (1·4 to 2·3)	170·9 (152·1 to 194·1)	30·2 (24·2 to 37·6)	128·7 (110·7 to 149·5)	7·3 (5·1 to 10·3)	4·7 (3·7 to 6·1)
Bangladesh*	2039	65·0 (57·3 to 73·8)	11·5 (9·2 to 14·3)	48·9 (42·0 to 56·8)	2·8 (2·0 to 4·0)	1·8 (1·4 to 2·3)	173·7 (153·0 to 197·3)	30·7 (24·6 to 38·3)	130·7 (112·3 to 151·8)	7·6 (5·2 to 10·8)	4·7 (3·7 to 6·3)
Bangladesh*	2040	66·0 (58·2 to 74·8)	11·7 (9·3 to 14·6)	49·6 (42·6 to 57·6)	2·9 (2·0 to 4·2)	1·8 (1·4 to 2·4)	176·3 (155·6 to 199·8)	31·2 (24·9 to 38·9)	132·5 (113·9 to 153·8)	7·8 (5·4 to 11·2)	4·8 (3·7 to 6·5)
Bangladesh*	2041	67·0 (59·2 to 75·8)	11·9 (9·5 to 14·8)	50·3 (43·2 to 58·4)	3·0 (2·1 to 4·3)	1·8 (1·4 to 2·5)	179·0 (158·3 to 202·6)	31·7 (25·3 to 39·6)	134·4 (115·4 to 156·0)	8·1 (5·6 to 11·6)	4·9 (3·7 to 6·7)
Bangladesh*	2042	68·0 (60·0 to 77·1)	12·1 (9·6 to 15·1)	50·9 (43·7 to 59·1)	3·1 (2·1 to 4·5)	1·9 (1·4 to 2·6)	181·7 (160·2 to 205·9)	32·2 (25·7 to 40·4)	136·1 (116·8 to 157·9)	8·4 (5·7 to 12·0)	5·0 (3·8 to 7·0)

Bangladesh*	2043	69·0 (60·8 to 77·9)	12·3 (9·8 to 15·4)	51·6 (44·3 to 59·9)	3·2 (2·2 to 4·7)	1·9 (1·4 to 2·7)	184·5 (162·5 to 208·1)	32·8 (26·1 to 41·2)	138·0 (118·4 to 160·2)	8·7 (5·9 to 12·4)	5·1 (3·8 to 7·2)
Bangladesh*	2044	70·1 (62·1 to 79·1)	12·5 (9·9 to 15·7)	52·3 (44·9 to 60·8)	3·3 (2·3 to 4·8)	1·9 (1·4 to 2·8)	187·3 (165·9 to 211·3)	33·4 (26·6 to 42·1)	139·8 (119·9 to 162·4)	8·9 (6·1 to 12·9)	5·2 (3·8 to 7·4)
Bangladesh*	2045	71·1 (63·0 to 80·1)	12·7 (10·1 to 16·1)	53·0 (45·4 to 61·5)	3·5 (2·3 to 5·0)	2·0 (1·5 to 2·8)	190·1 (168·4 to 214·1)	34·0 (27·0 to 42·9)	141·5 (121·3 to 164·4)	9·2 (6·3 to 13·4)	5·3 (3·9 to 7·6)
Bangladesh*	2046	72·2 (64·1 to 81·3)	13·0 (10·3 to 16·4)	53·6 (46·0 to 62·2)	3·6 (2·4 to 5·2)	2·0 (1·5 to 2·9)	192·8 (171·3 to 217·1)	34·7 (27·5 to 43·8)	143·2 (122·8 to 166·3)	9·6 (6·4 to 13·9)	5·4 (3·9 to 7·9)
Bangladesh*	2047	73·2 (65·0 to 82·6)	13·2 (10·5 to 16·7)	54·2 (46·5 to 63·0)	3·7 (2·5 to 5·4)	2·1 (1·5 to 3·1)	195·7 (173·6 to 220·6)	35·3 (28·0 to 44·6)	144·9 (124·2 to 168·4)	9·9 (6·6 to 14·4)	5·5 (4·0 to 8·2)
Bangladesh*	2048	74·3 (66·0 to 83·7)	13·5 (10·7 to 17·1)	54·9 (47·0 to 63·7)	3·8 (2·5 to 5·6)	2·1 (1·5 to 3·1)	198·4 (176·2 to 223·6)	36·0 (28·5 to 45·6)	146·6 (125·6 to 170·3)	10·2 (6·8 to 14·9)	5·7 (4·1 to 8·3)
Bangladesh*	2049	75·3 (66·4 to 85·0)	13·8 (10·9 to 17·4)	55·5 (47·5 to 64·4)	3·9 (2·6 to 5·8)	2·2 (1·5 to 3·2)	201·2 (177·5 to 227·2)	36·7 (29·1 to 46·5)	148·2 (127·0 to 172·2)	10·5 (7·0 to 15·4)	5·8 (4·1 to 8·6)
Bangladesh*	2050	76·3 (67·5 to 86·1)	14·0 (11·1 to 17·8)	56·0 (47·9 to 65·1)	4·0 (2·7 to 5·9)	2·2 (1·6 to 3·4)	203·9 (180·3 to 230·0)	37·5 (29·7 to 47·5)	149·7 (128·1 to 173·9)	10·8 (7·2 to 15·9)	6·0 (4·2 to 9·1)
Bulgaria*	1995	214·5 (194·5 to 236·8)	144·6 (128·0 to 163·2)	69·4 (57·7 to 82·5)	0·5 (0·3 to 0·7)	0·0 (0·0 to 0·0)	518·4 (470·1 to 572·3)	349·5 (309·3 to 394·4)	167·6 (139·4 to 199·4)	1·2 (0·8 to 1·7)	0·0 (0·0 to 0·0)
Bulgaria*	1996	206·5 (188·6 to 226·6)	135·7 (121·0 to 151·1)	70·4 (59·6 to 82·6)	0·4 (0·3 to 0·6)	0·0 (0·0 to 0·0)	499·0 (455·7 to 547·7)	327·9 (292·4 to 365·2)	170·0 (143·9 to 199·5)	1·0 (0·7 to 1·5)	0·0 (0·0 to 0·0)
Bulgaria*	1997	200·6 (184·5 to 218·3)	128·5 (115·9 to 141·5)	71·5 (61·8 to 83·1)	0·4 (0·2 to 0·5)	0·2 (0·2 to 0·2)	484·8 (446·0 to 527·4)	310·5 (280·2 to 341·9)	172·9 (149·4 to 200·9)	0·9 (0·6 to 1·3)	0·5 (0·5 to 0·5)
Bulgaria*	1998	226·9 (209·8 to 246·2)	142·1 (128·6 to 156·0)	81·5 (71·3 to 92·8)	0·4 (0·3 to 0·5)	2·9 (2·9 to 2·9)	548·3 (507·0 to 594·9)	343·5 (310·8 to 377·0)	197·0 (172·3 to 224·3)	0·9 (0·6 to 1·3)	6·9 (6·9 to 6·9)
Bulgaria*	1999	253·4 (236·0 to 272·1)	156·6 (142·4 to 170·8)	96·1 (86·0 to 107·6)	0·4 (0·3 to 0·6)	0·3 (0·3 to 0·3)	612·3 (570·3 to 657·7)	378·3 (344·2 to 412·6)	232·3 (207·9 to 260·1)	0·9 (0·6 to 1·4)	0·7 (0·7 to 0·7)
Bulgaria*	2000	285·7 (267·9 to 304·1)	171·4 (158·4 to 185·6)	112·2 (102·0 to 123·5)	0·4 (0·3 to 0·6)	1·7 (1·7 to 1·7)	690·4 (647·4 to 734·9)	414·1 (382·7 to 448·5)	271·3 (246·4 to 298·4)	1·0 (0·7 to 1·4)	4·0 (4·0 to 4·0)

Bulgaria*	2001	322·5 (304·4 to 340·7)	189·4 (175·7 to 203·6)	130·7 (120·0 to 141·2)	1·0 (0·7 to 1·4)	1·4 (1·4 to 1·4)	779·4 (735·6 to 823·3)	457·7 (424·6 to 492·0)	315·9 (290·1 to 341·2)	2·4 (1·6 to 3·4)	3·4 (3·4 to 3·4)
Bulgaria*	2002	356·4 (338·3 to 374·9)	209·2 (194·0 to 224·3)	143·0 (132·8 to 153·8)	2·3 (1·6 to 3·3)	1·8 (1·8 to 1·8)	861·3 (817·4 to 905·9)	505·6 (468·8 to 542·1)	345·6 (320·9 to 371·6)	5·7 (3·9 to 8·0)	4·4 (4·4 to 4·4)
Bulgaria*	2003	386·1 (367·4 to 406·2)	228·7 (213·2 to 244·6)	152·4 (142·0 to 163·0)	3·9 (2·7 to 5·5)	1·1 (1·1 to 1·1)	933·1 (887·8 to 981·6)	552·7 (515·3 to 591·0)	368·2 (343·2 to 393·9)	9·4 (6·5 to 13·3)	2·7 (2·7 to 2·7)
Bulgaria*	2004	408·0 (388·0 to 427·6)	238·9 (222·1 to 254·0)	160·2 (149·8 to 170·8)	5·1 (3·5 to 7·2)	3·8 (3·8 to 3·8)	985·9 (937·6 to 1033·2)	577·3 (536·8 to 613·7)	387·1 (361·9 to 412·8)	12·3 (8·6 to 17·4)	9·2 (9·2 to 9·2)
Bulgaria*	2005	428·3 (408·8 to 447·4)	247·1 (229·9 to 263·2)	169·9 (159·7 to 180·2)	6·2 (4·4 to 8·7)	5·1 (5·1 to 5·1)	1035·1 (987·8 to 1081·1)	597·1 (555·6 to 635·9)	410·6 (386·0 to 435·5)	15·0 (10·5 to 21·1)	12·4 (12·4 to 12·4)
Bulgaria*	2006	429·5 (411·0 to 447·3)	242·2 (226·1 to 257·1)	178·8 (168·7 to 189·3)	6·9 (4·8 to 9·8)	1·6 (1·6 to 1·6)	1038·0 (993·3 to 1080·8)	585·3 (546·4 to 621·3)	432·1 (407·8 to 457·4)	16·7 (11·7 to 23·6)	3·9 (3·9 to 3·9)
Bulgaria*	2007	469·5 (449·2 to 488·1)	259·0 (242·9 to 274·8)	192·4 (181·3 to 203·6)	7·6 (5·4 to 10·8)	10·5 (10·5 to 10·5)	1134·5 (1085·6 to 1179·6)	625·8 (587·0 to 664·2)	464·9 (438·1 to 492·0)	18·4 (13·0 to 26·0)	25·4 (25·4 to 25·4)
Bulgaria*	2008	491·8 (470·2 to 511·9)	271·6 (255·5 to 289·1)	207·9 (197·1 to 219·4)	8·7 (6·1 to 12·3)	3·6 (3·6 to 3·6)	1188·4 (1136·3 to 1237·1)	656·4 (617·4 to 698·7)	502·4 (476·4 to 530·1)	20·9 (14·7 to 29·7)	8·7 (8·7 to 8·7)
Bulgaria*	2009	529·2 (508·6 to 550·8)	279·5 (262·9 to 296·4)	221·9 (210·8 to 233·7)	8·8 (6·2 to 12·4)	19·0 (19·0 to 19·0)	1278·9 (1229·1 to 1331·0)	675·5 (635·4 to 716·2)	536·2 (509·4 to 564·8)	21·3 (15·0 to 29·9)	45·9 (45·9 to 45·9)
Bulgaria*	2010	540·3 (520·2 to 561·0)	291·5 (276·1 to 307·7)	237·7 (225·8 to 249·9)	8·8 (6·2 to 12·2)	2·3 (2·3 to 2·3)	1305·5 (1257·1 to 1355·6)	704·4 (667·1 to 743·6)	574·4 (545·7 to 603·9)	21·2 (14·9 to 29·5)	5·6 (5·6 to 5·6)
Bulgaria*	2011	559·4 (540·0 to 580·3)	296·6 (280·7 to 313·2)	252·4 (240·4 to 265·2)	8·5 (6·0 to 11·9)	1·9 (1·9 to 1·9)	1351·8 (1304·9 to 1402·2)	716·8 (678·3 to 756·7)	609·9 (580·8 to 640·8)	20·6 (14·6 to 28·7)	4·5 (4·5 to 4·5)
Bulgaria*	2012	580·7 (560·1 to 601·5)	307·6 (291·3 to 323·9)	263·1 (251·5 to 275·3)	8·9 (6·3 to 12·4)	1·0 (1·0 to 1·0)	1403·2 (1353·6 to 1453·6)	743·4 (703·8 to 782·7)	635·9 (607·8 to 665·4)	21·4 (15·3 to 30·0)	2·5 (2·5 to 2·5)
Bulgaria*	2013	601·5 (579·1 to 622·1)	331·4 (313·7 to 348·8)	260·1 (248·3 to 272·2)	9·3 (6·6 to 13·1)	0·7 (0·7 to 0·7)	1453·5 (1399·4 to 1503·3)	800·7 (758·1 to 842·9)	628·6 (600·0 to 657·7)	22·5 (15·9 to 31·6)	1·6 (1·6 to 1·6)
Bulgaria*	2014	641·2 (618·9 to 664·1)	363·7 (346·4 to 383·0)	266·5 (253·7 to 279·2)	10·0 (7·1 to 14·1)	1·0 (1·0 to 1·0)	1549·5 (1495·6 to 1604·9)	878·8 (837·0 to 925·4)	644·0 (613·1 to 674·7)	24·1 (17·2 to 34·0)	2·4 (2·4 to 2·4)

Bulgaria*	2015	660·4 (638·6 to 684·3)	372·9 (355·8 to 392·4)	275·9 (262·9 to 289·4)	10·5 (7·4 to 14·7)	1·0 (1·0 to 1·0)	1595·8 (1543·2 to 1653·7)	901·1 (859·9 to 948·2)	666·8 (635·2 to 699·4)	25·4 (17·8 to 35·5)	2·5 (2·5 to 2·5)
Bulgaria*	2016	661·8 (638·5 to 684·6)	365·0 (347·5 to 382·8)	284·8 (270·6 to 297·6)	11·0 (7·6 to 15·4)	1·0 (1·0 to 1·0)	1599·1 (1542·9 to 1654·5)	882·1 (839·6 to 925·1)	688·2 (653·8 to 719·1)	26·5 (18·3 to 37·2)	2·4 (2·4 to 2·4)
Bulgaria*	2017	692·3 (667·5 to 718·4)	382·4 (365·0 to 402·2)	297·2 (279·8 to 315·1)	11·9 (8·2 to 16·7)	0·7 (0·7 to 0·7)	1673·0 (1613·1 to 1736·1)	924·1 (882·1 to 972·0)	718·2 (676·2 to 761·6)	28·9 (19·7 to 40·3)	1·8 (1·8 to 1·8)
Bulgaria*	2018	733·4 (698·4 to 769·0)	414·9 (390·8 to 440·2)	305·1 (281·7 to 331·0)	13·1 (8·9 to 18·1)	0·4 (0·4 to 0·4)	1772·4 (1687·7 to 1858·3)	1002·5 (944·4 to 1063·8)	737·3 (680·7 to 799·9)	31·6 (21·5 to 43·7)	1·0 (1·0 to 1·0)
Bulgaria*	2019	784·6 (744·6 to 826·4)	453·9 (422·4 to 489·0)	316·4 (292·0 to 342·8)	13·9 (9·4 to 19·3)	0·4 (0·4 to 0·4)	1895·9 (1799·3 to 1997·0)	1096·8 (1020·8 to 1181·7)	764·5 (705·6 to 828·4)	33·6 (22·8 to 46·7)	0·9 (0·9 to 1·0)
Bulgaria*	2020	844·2 (804·9 to 886·0)	530·2 (498·5 to 565·6)	299·9 (276·5 to 325·0)	13·5 (9·1 to 18·7)	0·5 (0·5 to 0·6)	2040·1 (1945·0 to 2141·1)	1281·3 (1204·7 to 1366·9)	724·8 (668·2 to 785·4)	32·7 (22·1 to 45·2)	1·3 (1·3 to 1·4)
Bulgaria*	2021	734·8 (693·5 to 779·7)	434·9 (399·9 to 473·0)	286·0 (263·8 to 309·9)	13·2 (8·9 to 18·3)	0·6 (0·5 to 0·6)	1775·5 (1675·8 to 1884·3)	1051·0 (966·4 to 1143·0)	691·2 (637·5 to 749·0)	31·9 (21·6 to 44·2)	1·4 (1·3 to 1·6)
Bulgaria*	2022	720·2 (673·1 to 771·0)	419·3 (377·7 to 464·3)	286·9 (264·6 to 310·7)	13·6 (9·2 to 18·9)	0·5 (0·4 to 0·5)	1740·4 (1626·6 to 1863·1)	1013·3 (912·8 to 1121·9)	693·2 (639·3 to 750·8)	32·8 (22·2 to 45·6)	1·1 (1·0 to 1·3)
Bulgaria*	2023	727·4 (678·9 to 782·3)	424·6 (382·8 to 473·5)	288·3 (265·8 to 312·3)	14·0 (9·4 to 19·5)	0·5 (0·4 to 0·6)	1757·7 (1640·7 to 1890·5)	1026·1 (925·2 to 1144·1)	696·7 (642·3 to 754·7)	33·8 (22·8 to 47·1)	1·2 (1·1 to 1·4)
Bulgaria*	2024	735·3 (680·9 to 795·3)	430·4 (382·8 to 483·1)	289·9 (267·3 to 314·4)	14·4 (9·7 to 20·1)	0·5 (0·5 to 0·6)	1776·8 (1645·5 to 1921·8)	1040·1 (925·1 to 1167·4)	700·6 (645·8 to 759·8)	34·8 (23·4 to 48·5)	1·2 (1·1 to 1·5)
Bulgaria*	2025	742·8 (688·4 to 805·8)	435·6 (387·2 to 493·0)	291·8 (269·0 to 316·6)	14·8 (10·0 to 20·6)	0·5 (0·5 to 0·6)	1795·0 (1663·6 to 1947·1)	1052·7 (935·6 to 1191·4)	705·2 (650·0 to 765·0)	35·9 (24·2 to 49·8)	1·3 (1·2 to 1·6)
Bulgaria*	2026	749·8 (690·9 to 816·4)	440·0 (386·3 to 498·5)	294·0 (271·0 to 319·1)	15·3 (10·3 to 21·2)	0·6 (0·5 to 0·7)	1811·9 (1669·6 to 1972·8)	1063·2 (933·5 to 1204·6)	710·4 (654·8 to 771·1)	37·0 (24·9 to 51·2)	1·3 (1·2 to 1·6)
Bulgaria*	2027	758·9 (697·6 to 828·0)	446·2 (388·7 to 509·1)	296·4 (273·1 to 321·8)	15·8 (10·6 to 21·9)	0·6 (0·5 to 0·7)	1833·9 (1685·8 to 2000·8)	1078·2 (939·3 to 1230·3)	716·1 (660·0 to 777·5)	38·1 (25·6 to 52·9)	1·4 (1·2 to 1·7)
Bulgaria*	2028	769·9 (704·8 to 840·5)	453·8 (392·2 to 518·3)	299·2 (275·8 to 324·9)	16·3 (10·9 to 22·7)	0·6 (0·5 to 0·7)	1860·5 (1703·2 to 2031·2)	1096·6 (947·8 to 1252·4)	723·0 (666·4 to 785·0)	39·4 (26·3 to 54·8)	1·4 (1·3 to 1·7)

Bulgaria*	2029	781·2 (713·5 to 857·5)	461·5 (399·9 to 530·5)	302·2 (278·6 to 328·1)	16·8 (11·2 to 23·5)	0·6 (0·5 to 0·7)	1887·8 (1724·2 to 2072·2)	1115·3 (966·3 to 1282·0)	730·3 (673·3 to 792·9)	40·7 (27·2 to 56·8)	1·5 (1·3 to 1·8)
Bulgaria*	2030	793·5 (723·3 to 871·0)	470·2 (404·2 to 540·9)	305·4 (281·6 to 331·5)	17·4 (11·6 to 24·3)	0·6 (0·5 to 0·8)	1917·6 (1747·9 to 2104·9)	1136·2 (976·7 to 1307·2)	737·9 (680·4 to 801·1)	42·0 (28·1 to 58·7)	1·5 (1·3 to 1·9)
Bulgaria*	2031	804·2 (728·8 to 883·2)	477·1 (404·2 to 549·2)	308·5 (284·5 to 334·9)	18·0 (12·0 to 25·2)	0·6 (0·6 to 0·8)	1943·3 (1761·2 to 2134·2)	1152·8 (976·7 to 1327·2)	745·5 (687·4 to 809·2)	43·4 (29·0 to 60·9)	1·6 (1·4 to 1·9)
Bulgaria*	2032	814·8 (736·5 to 899·3)	484·2 (409·0 to 563·1)	311·4 (287·1 to 338·1)	18·6 (12·4 to 26·1)	0·7 (0·6 to 0·8)	1968·9 (1779·8 to 2173·2)	1170·0 (988·3 to 1360·8)	752·4 (693·7 to 816·9)	44·9 (29·9 to 63·0)	1·6 (1·4 to 2·0)
Bulgaria*	2033	825·0 (745·4 to 911·1)	491·2 (416·5 to 571·2)	314·0 (289·4 to 341·0)	19·2 (12·8 to 27·0)	0·7 (0·6 to 0·9)	1993·7 (1801·2 to 2201·6)	1187·0 (1006·6 to 1380·4)	758·7 (699·3 to 823·9)	46·3 (30·9 to 65·2)	1·7 (1·4 to 2·1)
Bulgaria*	2034	835·1 (749·5 to 927·7)	498·4 (416·6 to 586·1)	316·1 (291·3 to 343·5)	19·8 (13·1 to 28·0)	0·7 (0·6 to 0·9)	2018·0 (1811·1 to 2241·7)	1204·5 (1006·6 to 1416·2)	764·0 (703·9 to 830·0)	47·8 (31·8 to 67·6)	1·8 (1·5 to 2·2)
Bulgaria*	2035	844·9 (756·1 to 941·7)	505·6 (421·9 to 598·8)	318·2 (293·0 to 345·8)	20·4 (13·5 to 28·9)	0·8 (0·6 to 1·0)	2041·7 (1827·2 to 2275·6)	1221·8 (1019·4 to 1447·0)	768·8 (708·1 to 835·6)	49·3 (32·7 to 69·8)	1·8 (1·6 to 2·3)
Bulgaria*	2036	855·8 (763·2 to 956·3)	513·9 (424·2 to 612·8)	320·0 (294·6 to 347·9)	21·0 (13·9 to 29·8)	0·8 (0·7 to 1·0)	2068·0 (1844·4 to 2311·0)	1241·9 (1025·0 to 1480·9)	773·4 (712·0 to 840·7)	50·8 (33·7 to 72·1)	1·9 (1·6 to 2·4)
Bulgaria*	2037	865·6 (767·5 to 970·0)	521·5 (426·1 to 621·3)	321·6 (295·9 to 349·3)	21·6 (14·4 to 30·8)	0·8 (0·7 to 1·1)	2091·7 (1854·7 to 2344·1)	1260·3 (1029·6 to 1501·5)	777·1 (715·0 to 844·2)	52·3 (34·8 to 74·4)	2·0 (1·7 to 2·6)
Bulgaria*	2038	875·5 (775·0 to 983·9)	529·3 (429·6 to 634·9)	323·0 (297·1 to 350·7)	22·3 (14·8 to 31·8)	0·9 (0·7 to 1·1)	2115·6 (1872·9 to 2377·6)	1279·0 (1038·2 to 1534·3)	780·6 (718·0 to 847·4)	53·9 (35·8 to 76·8)	2·2 (1·8 to 2·8)
Bulgaria*	2039	885·5 (780·2 to 996·3)	537·4 (435·0 to 642·8)	324·2 (298·1 to 352·2)	22·9 (15·3 to 32·7)	0·9 (0·8 to 1·2)	2139·8 (1885·4 to 2407·6)	1298·6 (1051·1 to 1553·5)	783·4 (720·4 to 851·1)	55·5 (36·9 to 79·1)	2·3 (1·9 to 3·0)
Bulgaria*	2040	895·0 (784·6 to 1009·2)	545·2 (437·0 to 654·2)	325·2 (299·2 to 353·6)	23·6 (15·7 to 33·8)	1·0 (0·8 to 1·3)	2162·7 (1896·0 to 2438·8)	1317·5 (1056·1 to 1580·8)	785·7 (723·0 to 854·5)	57·1 (37·9 to 81·7)	2·4 (2·0 to 3·2)
Bulgaria*	2041	904·4 (792·7 to 1024·4)	553·0 (442·1 to 667·5)	326·1 (299·9 to 354·7)	24·3 (16·2 to 35·0)	1·1 (0·9 to 1·4)	2185·6 (1915·6 to 2475·5)	1336·4 (1068·3 to 1613·1)	787·9 (724·7 to 857·1)	58·7 (39·1 to 84·6)	2·6 (2·1 to 3·5)
Bulgaria*	2042	914·5 (797·7 to 1043·6)	561·3 (448·7 to 687·7)	327·0 (300·7 to 355·6)	25·0 (16·6 to 36·3)	1·1 (0·9 to 1·6)	2209·8 (1927·8 to 2521·9)	1356·4 (1084·2 to 1661·8)	790·3 (726·6 to 859·2)	60·4 (40·2 to 87·6)	2·8 (2·2 to 3·8)

Bulgaria*	2043	924·0 (804·7 to 1060·9)	569·0 (454·2 to 703·3)	328·1 (301·5 to 356·8)	25·7 (17·1 to 37·5)	1·2 (1·0 to 1·7)	2232·8 (1944·5 to 2563·6)	1374·9 (1097·5 to 1699·5)	792·9 (728·5 to 862·2)	62·1 (41·4 to 90·6)	2·9 (2·4 to 4·1)
Bulgaria*	2044	933·6 (810·9 to 1068·4)	576·6 (458·0 to 708·9)	329·2 (302·5 to 357·8)	26·4 (17·6 to 39·0)	1·3 (1·0 to 1·8)	2256·0 (1959·5 to 2581·7)	1393·4 (1106·8 to 1713·1)	795·6 (730·9 to 864·6)	63·9 (42·6 to 94·2)	3·1 (2·5 to 4·5)
Bulgaria*	2045	943·8 (818·3 to 1079·7)	584·8 (465·1 to 716·0)	330·5 (303·6 to 359·0)	27·2 (18·1 to 40·3)	1·3 (1·0 to 2·0)	2280·8 (1977·4 to 2609·2)	1413·1 (1124·0 to 1730·1)	798·7 (733·6 to 867·5)	65·7 (43·7 to 97·4)	3·3 (2·5 to 4·8)
Bulgaria*	2046	953·1 (824·0 to 1095·0)	592·1 (470·9 to 730·1)	331·6 (304·5 to 360·0)	28·0 (18·5 to 41·4)	1·4 (0·0 to 2·2)	2303·1 (1991·2 to 2646·1)	1430·8 (1137·9 to 1764·3)	801·2 (735·8 to 870·0)	67·6 (44·8 to 100·0)	3·5 (0·0 to 5·2)
Bulgaria*	2047	962·5 (828·7 to 1116·8)	599·6 (470·8 to 748·4)	332·6 (305·4 to 361·3)	28·7 (18·9 to 42·6)	1·5 (0·0 to 2·4)	2325·8 (2002·5 to 2698·7)	1448·8 (1137·8 to 1808·5)	803·8 (738·0 to 873·0)	69·5 (45·8 to 103·1)	3·6 (0·0 to 5·7)
Bulgaria*	2048	971·3 (834·8 to 1132·6)	606·6 (477·1 to 763·8)	333·5 (306·2 to 362·7)	29·5 (19·4 to 43·9)	1·6 (0·0 to 2·5)	2347·1 (2017·2 to 2737·1)	1465·8 (1152·9 to 1845·8)	806·0 (740·0 to 876·4)	71·4 (47·0 to 106·0)	3·8 (0·0 to 6·1)
Bulgaria*	2049	981·1 (842·2 to 1143·6)	614·7 (480·3 to 772·0)	334·4 (307·1 to 363·7)	30·4 (20·0 to 45·2)	1·7 (0·0 to 2·8)	2371·0 (2035·2 to 2763·5)	1485·3 (1160·6 to 1865·6)	808·2 (742·2 to 879·0)	73·4 (48·2 to 109·3)	4·1 (0·0 to 6·7)
Bulgaria*	2050	991·4 (847·3 to 1161·9)	622·9 (481·3 to 786·0)	335·5 (308·1 to 364·7)	31·2 (20·5 to 46·5)	1·8 (0·0 to 3·0)	2395·7 (2047·5 to 2807·8)	1505·2 (1163·0 to 1899·3)	810·7 (744·5 to 881·4)	75·5 (49·6 to 112·3)	4·3 (0·0 to 7·2)
Bahrain	1995	911·7 (860·2 to 968·0)	601·9 (558·3 to 652·4)	244·5 (217·4 to 274·1)	65·2 (52·2 to 81·6)	0·0 (0·0 to 0·0)	1958·7 (1848·1 to 2079·8)	1293·3 (1199·5 to 1401·7)	525·4 (467·1 to 588·8)	140·0 (112·2 to 175·2)	0·0 (0·0 to 0·0)
Bahrain	1996	894·7 (854·7 to 940·4)	587·3 (554·7 to 623·4)	241·7 (219·2 to 263·8)	65·6 (53·8 to 80·3)	0·0 (0·0 to 0·0)	1922·2 (1836·4 to 2020·4)	1261·9 (1191·7 to 1339·3)	519·3 (470·9 to 566·7)	141·0 (115·7 to 172·5)	0·0 (0·0 to 0·0)
Bahrain	1997	984·2 (944·2 to 1030·6)	661·7 (628·0 to 697·3)	252·1 (230·8 to 274·5)	70·4 (58·1 to 84·0)	0·0 (0·0 to 0·0)	2114·5 (2028·6 to 2214·3)	1421·6 (1349·2 to 1498·2)	541·6 (495·8 to 589·7)	151·3 (124·9 to 180·4)	0·0 (0·0 to 0·0)
Bahrain	1998	928·8 (889·6 to 970·5)	613·9 (583·0 to 647·0)	244·6 (225·1 to 265·7)	70·2 (58·1 to 82·4)	0·1 (0·1 to 0·1)	1995·5 (1911·3 to 2085·1)	1319·0 (1252·5 to 1390·1)	525·6 (483·6 to 570·8)	150·7 (124·9 to 177·0)	0·2 (0·2 to 0·2)
Bahrain	1999	945·4 (905·2 to 986·6)	627·4 (596·6 to 659·7)	244·9 (224·8 to 264·9)	73·2 (63·0 to 84·9)	0·0 (0·0 to 0·0)	2031·2 (1944·8 to 2119·7)	1347·8 (1281·8 to 1417·3)	526·1 (482·9 to 569·2)	157·2 (135·4 to 182·3)	0·1 (0·1 to 0·1)
Bahrain	2000	882·9 (847·8 to 918·9)	576·1 (548·0 to 605·5)	235·0 (216·1 to 253·4)	71·8 (63·1 to 81·1)	0·0 (0·0 to 0·0)	1896·9 (1821·4 to 1974·2)	1237·7 (1177·4 to 1300·9)	504·8 (464·4 to 544·5)	154·3 (135·7 to 174·2)	0·1 (0·1 to 0·1)

Bahrain	2001	930·1 (894·9 to 969·0)	613·5 (583·9 to 645·8)	245·1 (226·8 to 264·0)	71·5 (64·4 to 79·0)	0·0 (0·0 to 0·0)	1998·3 (1922·8 to 2081·8)	1318·1 (1254·5 to 1387·4)	526·5 (487·3 to 567·2)	153·7 (138·4 to 169·7)	0·0 (0·0 to 0·0)
Bahrain	2002	958·4 (923·1 to 996·1)	633·0 (600·9 to 665·7)	259·2 (241·1 to 278·7)	66·2 (60·3 to 72·4)	0·0 (0·0 to 0·0)	2059·2 (1983·3 to 2140·0)	1360·0 (1291·0 to 1430·2)	557·0 (518·1 to 598·7)	142·2 (129·5 to 155·7)	0·0 (0·0 to 0·0)
Bahrain	2003	907·3 (873·0 to 942·2)	589·0 (559·8 to 619·9)	264·7 (247·8 to 284·8)	53·6 (48·4 to 59·5)	0·0 (0·0 to 0·0)	1949·3 (1875·6 to 2024·2)	1265·4 (1202·8 to 1331·8)	568·8 (532·5 to 611·8)	115·2 (103·9 to 127·8)	0·0 (0·0 to 0·0)
Bahrain	2004	819·4 (786·5 to 853·0)	533·5 (505·8 to 561·5)	247·0 (230·4 to 265·7)	38·8 (34·4 to 43·9)	0·0 (0·0 to 0·0)	1760·5 (1689·7 to 1832·6)	1146·3 (1086·6 to 1206·3)	530·7 (495·1 to 570·8)	83·4 (73·8 to 94·4)	0·0 (0·0 to 0·0)
Bahrain	2005	779·8 (747·7 to 812·7)	516·7 (489·8 to 545·3)	229·4 (213·3 to 247·1)	33·7 (29·5 to 38·4)	0·0 (0·0 to 0·0)	1675·3 (1606·4 to 1746·0)	1110·2 (1052·3 to 1171·5)	492·8 (458·2 to 530·8)	72·3 (63·4 to 82·4)	0·0 (0·0 to 0·0)
Bahrain	2006	752·1 (720·7 to 784·8)	501·9 (473·6 to 530·1)	218·1 (202·8 to 235·9)	32·2 (27·9 to 36·6)	0·0 (0·0 to 0·0)	1615·9 (1548·5 to 1686·1)	1078·3 (1017·6 to 1138·8)	468·6 (435·7 to 506·9)	69·1 (59·9 to 78·6)	0·0 (0·0 to 0·0)
Bahrain	2007	763·2 (728·5 to 794·4)	503·6 (474·8 to 531·4)	221·4 (205·7 to 239·2)	38·3 (33·7 to 43·4)	0·0 (0·0 to 0·0)	1639·7 (1565·1 to 1706·7)	1081·9 (1020·0 to 1141·7)	475·6 (441·9 to 513·9)	82·3 (72·3 to 93·2)	0·0 (0·0 to 0·0)
Bahrain	2008	789·5 (754·2 to 824·3)	514·8 (485·9 to 544·9)	224·2 (207·9 to 242·0)	50·6 (45·4 to 56·2)	0·0 (0·0 to 0·0)	1696·3 (1620·4 to 1770·9)	1106·0 (1044·0 to 1170·6)	481·6 (446·7 to 519·9)	108·8 (97·6 to 120·7)	0·0 (0·0 to 0·0)
Bahrain	2009	814·5 (782·1 to 849·6)	519·3 (491·5 to 548·6)	231·7 (214·8 to 249·9)	63·5 (57·9 to 69·6)	0·0 (0·0 to 0·0)	1749·9 (1680·3 to 1825·2)	1115·7 (1055·9 to 1178·6)	497·8 (461·6 to 537·0)	136·5 (124·5 to 149·5)	0·0 (0·0 to 0·0)
Bahrain	2010	843·0 (809·2 to 878·9)	537·8 (508·2 to 568·2)	234·7 (218·3 to 252·3)	70·4 (64·7 to 76·7)	0·0 (0·0 to 0·0)	1811·1 (1738·5 to 1888·4)	1155·5 (1091·8 to 1220·8)	504·3 (468·9 to 542·1)	151·3 (139·1 to 164·8)	0·0 (0·0 to 0·0)
Bahrain	2011	822·4 (790·7 to 856·4)	523·4 (496·8 to 552·7)	229·5 (213·3 to 246·4)	69·5 (63·8 to 75·4)	0·0 (0·0 to 0·0)	1766·9 (1698·8 to 1840·0)	1124·6 (1067·4 to 1187·6)	493·1 (458·4 to 529·4)	149·2 (137·1 to 161·9)	0·0 (0·0 to 0·0)
Bahrain	2012	895·8 (863·8 to 930·9)	582·3 (553·7 to 614·1)	241·2 (224·9 to 257·5)	72·2 (66·5 to 78·3)	0·0 (0·0 to 0·0)	1924·6 (1855·8 to 2000·1)	1251·1 (1189·5 to 1319·4)	518·2 (483·2 to 553·3)	155·2 (142·8 to 168·2)	0·0 (0·0 to 0·0)
Bahrain	2013	987·5 (953·5 to 1023·6)	648·9 (618·9 to 680·0)	258·9 (241·6 to 275·7)	79·7 (73·6 to 85·7)	0·0 (0·0 to 0·0)	2121·6 (2048·5 to 2199·2)	1394·2 (1329·6 to 1460·9)	556·1 (519·1 to 592·3)	171·3 (158·1 to 184·1)	0·0 (0·0 to 0·0)
Bahrain	2014	963·9 (932·1 to 996·4)	614·4 (587·4 to 642·7)	263·3 (246·4 to 280·4)	86·2 (80·2 to 92·3)	0·0 (0·0 to 0·0)	2071·0 (2002·5 to 2140·8)	1319·9 (1262·1 to 1380·7)	565·8 (529·3 to 602·5)	185·2 (172·3 to 198·3)	0·0 (0·0 to 0·0)

Bahrain	2015	1135·7 (1098·8 to 1173·6)	727·3 (695·0 to 759·4)	299·9 (281·2 to 317·8)	108·5 (101·6 to 115·5)	0·0 (0·0 to 0·0)	2440·1 (2360·6 to 2521·4)	1562·7 (1493·2 to 1631·5)	644·3 (604·2 to 682·8)	233·2 (218·2 to 248·1)	0·0 (0·0 to 0·0)
Bahrain	2016	1132·7 (1095·5 to 1169·3)	696·2 (666·6 to 726·1)	315·8 (296·3 to 333·7)	120·7 (113·4 to 127·5)	0·0 (0·0 to 0·0)	2433·6 (2353·6 to 2512·2)	1495·8 (1432·2 to 1560·1)	678·6 (636·7 to 716·9)	259·2 (243·7 to 273·9)	0·0 (0·0 to 0·0)
Bahrain	2017	1096·0 (1053·9 to 1134·0)	646·5 (613·6 to 678·5)	324·1 (303·9 to 344·4)	125·4 (118·7 to 132·7)	0·0 (0·0 to 0·0)	2354·7 (2264·4 to 2436·4)	1388·9 (1318·2 to 1457·7)	696·3 (652·9 to 739·9)	269·5 (255·0 to 285·1)	0·0 (0·0 to 0·0)
Bahrain	2018	1069·3 (1021·7 to 1117·9)	630·3 (588·5 to 674·3)	319·8 (294·8 to 347·0)	119·2 (110·2 to 128·8)	0·0 (0·0 to 0·0)	2297·4 (2195·0 to 2401·7)	1354·1 (1264·3 to 1448·7)	687·2 (633·4 to 745·5)	256·2 (236·7 to 276·7)	0·0 (0·0 to 0·0)
Bahrain	2019	1052·0 (1001·3 to 1102·5)	607·6 (565·8 to 653·1)	319·9 (294·9 to 347·9)	124·5 (114·2 to 134·9)	0·0 (0·0 to 0·0)	2260·3 (2151·2 to 2368·7)	1305·3 (1215·7 to 1403·2)	687·4 (633·5 to 747·5)	267·5 (245·4 to 289·8)	0·0 (0·0 to 0·0)
Bahrain	2020	1356·2 (1306·7 to 1405·9)	933·7 (892·8 to 978·2)	300·1 (276·4 to 326·6)	122·5 (108·9 to 136·2)	0·0 (0·0 to 0·0)	2913·8 (2807·5 to 3020·5)	2005·9 (1918·2 to 2101·7)	644·7 (593·8 to 701·7)	263·1 (234·0 to 292·5)	0·0 (0·0 to 0·0)
Bahrain	2021	919·5 (869·3 to 970·1)	517·3 (475·1 to 559·6)	281·8 (259·0 to 306·8)	120·5 (103·2 to 138·3)	0·0 (0·0 to 0·0)	1975·6 (1867·7 to 2084·1)	1111·4 (1020·8 to 1202·2)	605·4 (556·4 to 659·1)	258·8 (221·7 to 297·0)	0·0 (0·0 to 0·0)
Bahrain	2022	928·0 (870·2 to 985·0)	518·7 (468·3 to 566·9)	283·0 (260·5 to 308·1)	126·3 (104·5 to 150·1)	0·0 (0·0 to 0·0)	1993·7 (1869·6 to 2116·3)	1114·5 (1006·1 to 1217·9)	608·0 (559·6 to 661·9)	271·3 (224·5 to 322·5)	0·0 (0·0 to 0·0)
Bahrain	2023	933·5 (872·8 to 996·7)	518·2 (464·9 to 573·4)	283·6 (260·9 to 308·9)	131·7 (109·2 to 157·0)	0·0 (0·0 to 0·0)	2005·7 (1875·3 to 2141·3)	1113·4 (998·9 to 1231·9)	609·2 (560·6 to 663·7)	283·1 (234·7 to 337·4)	0·0 (0·0 to 0·0)
Bahrain	2024	937·4 (873·7 to 1004·1)	519·3 (463·1 to 579·6)	284·2 (261·2 to 309·6)	134·0 (111·0 to 159·6)	0·0 (0·0 to 0·0)	2014·0 (1877·2 to 2157·2)	1115·6 (995·0 to 1245·3)	610·5 (561·2 to 665·2)	287·8 (238·4 to 343·0)	0·0 (0·0 to 0·0)
Bahrain	2025	940·1 (873·8 to 1009·8)	519·7 (460·9 to 581·1)	284·3 (261·1 to 310·0)	136·1 (112·3 to 162·3)	0·0 (0·0 to 0·0)	2019·7 (1877·4 to 2169·5)	1116·6 (990·2 to 1248·4)	610·8 (561·0 to 666·0)	292·3 (241·2 to 348·7)	0·0 (0·0 to 0·0)
Bahrain	2026	942·9 (874·6 to 1016·0)	520·3 (460·9 to 585·1)	284·4 (261·6 to 310·2)	138·3 (114·2 to 164·6)	0·0 (0·0 to 0·0)	2025·8 (1879·1 to 2182·8)	1117·7 (990·2 to 1257·1)	611·0 (562·1 to 666·4)	297·1 (245·4 to 353·5)	0·0 (0·0 to 0·0)
Bahrain	2027	946·4 (876·9 to 1024·9)	521·7 (461·7 to 588·8)	284·2 (261·6 to 310·3)	140·5 (115·5 to 168·0)	0·0 (0·0 to 0·0)	2033·3 (1883·9 to 2202·0)	1120·9 (991·9 to 1265·0)	610·6 (562·1 to 666·6)	301·8 (248·1 to 360·9)	0·0 (0·0 to 0·0)
Bahrain	2028	949·0 (877·7 to 1027·4)	522·3 (458·6 to 592·4)	283·9 (261·2 to 310·0)	142·8 (117·3 to 171·5)	0·0 (0·0 to 0·0)	2038·9 (1885·8 to 2207·3)	1122·1 (985·4 to 1272·7)	610·0 (561·1 to 666·0)	306·8 (252·0 to 368·5)	0·0 (0·0 to 0·0)

Bahrain	2029	951·7 (877·1 to 1031·8)	523·0 (456·5 to 592·4)	283·5 (260·7 to 309·5)	145·2 (118·7 to 174·7)	0·0 (0·0 to 0·0)	2044·7 (1884·5 to 2216·7)	1123·7 (980·8 to 1272·8)	609·0 (560·0 to 664·9)	312·0 (255·1 to 375·3)	0·0 (0·0 to 0·0)
Bahrain	2030	954·4 (877·9 to 1036·6)	524·4 (455·9 to 596·5)	282·2 (259·5 to 308·1)	147·8 (119·8 to 179·1)	0·0 (0·0 to 0·0)	2050·4 (1886·1 to 2227·2)	1126·7 (979·6 to 1281·5)	606·3 (557·4 to 661·9)	317·5 (257·5 to 384·8)	0·0 (0·0 to 0·0)
Bahrain	2031	958·1 (880·9 to 1042·3)	525·5 (455·7 to 602·0)	280·8 (257·9 to 306·5)	151·8 (122·8 to 185·4)	0·0 (0·0 to 0·0)	2058·4 (1892·5 to 2239·3)	1129·0 (979·0 to 1293·5)	603·3 (554·1 to 658·4)	326·1 (263·8 to 398·3)	0·0 (0·0 to 0·0)
Bahrain	2032	962·4 (881·6 to 1049·7)	526·4 (454·9 to 603·7)	279·4 (256·2 to 305·2)	156·6 (125·9 to 192·7)	0·0 (0·0 to 0·0)	2067·7 (1894·1 to 2255·1)	1131·0 (977·3 to 1297·0)	600·2 (550·5 to 655·8)	336·5 (270·5 to 414·0)	0·0 (0·0 to 0·0)
Bahrain	2033	965·8 (882·9 to 1058·1)	526·5 (453·6 to 609·3)	277·7 (254·9 to 303·4)	161·5 (129·9 to 200·8)	0·0 (0·0 to 0·0)	2074·9 (1896·9 to 2273·3)	1131·2 (974·6 to 1309·1)	596·7 (547·6 to 651·8)	347·0 (279·0 to 431·5)	0·0 (0·0 to 0·0)
Bahrain	2034	969·6 (884·7 to 1064·6)	526·8 (452·1 to 610·9)	276·1 (253·1 to 301·2)	166·7 (131·5 to 208·0)	0·0 (0·0 to 0·0)	2083·2 (1900·7 to 2287·3)	1131·9 (971·4 to 1312·6)	593·2 (543·8 to 647·0)	358·1 (282·6 to 446·9)	0·0 (0·0 to 0·0)
Bahrain	2035	972·6 (885·0 to 1071·8)	526·6 (451·4 to 611·9)	274·2 (251·2 to 298·7)	171·8 (133·6 to 217·0)	0·0 (0·0 to 0·0)	2089·7 (1901·5 to 2302·8)	1131·4 (969·9 to 1314·7)	589·2 (539·6 to 641·8)	369·1 (287·0 to 466·2)	0·0 (0·0 to 0·0)
Bahrain	2036	974·0 (881·3 to 1076·0)	525·2 (446·1 to 615·3)	271·9 (248·8 to 296·1)	176·9 (135·5 to 227·5)	0·0 (0·0 to 0·0)	2092·6 (1893·5 to 2311·8)	1128·4 (958·5 to 1321·9)	584·3 (534·6 to 636·1)	380·0 (291·1 to 488·8)	0·0 (0·0 to 0·0)
Bahrain	2037	976·2 (880·0 to 1082·2)	524·5 (443·7 to 617·6)	269·6 (246·5 to 294·2)	182·0 (136·9 to 239·3)	0·0 (0·0 to 0·0)	2097·3 (1890·7 to 2325·1)	1127·0 (953·3 to 1327·0)	579·3 (529·5 to 632·0)	391·0 (294·1 to 514·1)	0·0 (0·0 to 0·0)
Bahrain	2038	975·2 (874·3 to 1085·2)	521·7 (438·6 to 612·0)	266·6 (243·5 to 291·7)	186·9 (138·1 to 249·7)	0·0 (0·0 to 0·0)	2095·2 (1878·4 to 2331·6)	1120·8 (942·3 to 1314·9)	572·8 (523·1 to 626·6)	401·6 (296·8 to 536·4)	0·0 (0·0 to 0·0)
Bahrain	2039	972·8 (867·9 to 1086·7)	517·8 (434·0 to 612·0)	263·1 (240·1 to 288·2)	191·8 (139·3 to 260·0)	0·0 (0·0 to 0·0)	2089·9 (1864·7 to 2334·8)	1112·5 (932·3 to 1314·9)	565·4 (515·8 to 619·1)	412·1 (299·2 to 558·7)	0·0 (0·0 to 0·0)
Bahrain	2040	970·1 (863·9 to 1086·4)	514·0 (432·8 to 610·8)	259·5 (236·6 to 285·1)	196·6 (139·8 to 270·7)	0·0 (0·0 to 0·0)	2084·3 (1856·1 to 2334·1)	1104·3 (929·8 to 1312·2)	557·5 (508·3 to 612·6)	422·5 (300·5 to 581·6)	0·0 (0·0 to 0·0)
Bahrain	2041	966·7 (858·3 to 1086·1)	509·6 (426·2 to 606·9)	255·7 (232·9 to 281·5)	201·4 (140·3 to 280·4)	0·0 (0·0 to 0·0)	2076·9 (1844·1 to 2333·4)	1094·9 (915·6 to 1303·8)	549·4 (500·4 to 604·8)	432·7 (301·5 to 602·4)	0·0 (0·0 to 0·0)
Bahrain	2042	963·3 (849·8 to 1090·7)	505·5 (417·8 to 607·7)	251·8 (229·0 to 278·3)	206·0 (140·3 to 291·5)	0·0 (0·0 to 0·0)	2069·6 (1825·7 to 2343·3)	1086·0 (897·7 to 1305·6)	541·1 (492·0 to 597·8)	442·5 (301·5 to 626·3)	0·0 (0·0 to 0·0)

Bahrain	2043	958·4 (839·9 to 1090·1)	500·2 (408·9 to 596·3)	247·7 (225·0 to 274·5)	210·5 (139·1 to 305·4)	0·0 (0·0 to 0·0)	2059·1 (1804·6 to 2342·1)	1074·7 (878·4 to 1281·1)	532·3 (483·5 to 589·7)	452·2 (298·9 to 656·2)	0·0 (0·0 to 0·0)
Bahrain	2044	952·1 (828·9 to 1090·3)	494·2 (405·1 to 591·3)	243·2 (220·6 to 270·5)	214·8 (137·6 to 318·3)	0·0 (0·0 to 0·0)	2045·6 (1780·9 to 2342·6)	1061·7 (870·4 to 1270·4)	522·4 (474·0 to 581·1)	461·5 (295·5 to 683·9)	0·0 (0·0 to 0·0)
Bahrain	2045	945·5 (817·3 to 1091·8)	487·9 (400·5 to 583·2)	238·6 (216·3 to 266·4)	219·0 (136·0 to 329·4)	0·0 (0·0 to 0·0)	2031·3 (1755·9 to 2345·6)	1048·2 (860·6 to 1253·0)	512·6 (464·7 to 572·4)	470·4 (292·3 to 707·6)	0·0 (0·0 to 0·0)
Bahrain	2046	939·9 (810·8 to 1093·4)	482·4 (394·8 to 578·5)	234·4 (212·3 to 262·7)	223·0 (135·0 to 341·0)	0·0 (0·0 to 0·0)	2019·3 (1741·9 to 2349·2)	1036·4 (848·3 to 1242·9)	503·7 (456·2 to 564·3)	479·2 (290·1 to 732·5)	0·0 (0·0 to 0·0)
Bahrain	2047	936·3 (802·8 to 1093·4)	478·2 (389·9 to 574·1)	231·0 (209·0 to 259·3)	227·1 (134·8 to 351·1)	0·0 (0·0 to 0·0)	2011·6 (1724·8 to 2349·1)	1027·5 (837·6 to 1233·4)	496·2 (449·0 to 557·0)	487·9 (289·5 to 754·4)	0·0 (0·0 to 0·0)
Bahrain	2048	935·9 (799·5 to 1098·7)	476·0 (387·0 to 577·9)	228·4 (206·5 to 257·0)	231·5 (135·7 to 360·6)	0·0 (0·0 to 0·0)	2010·8 (1717·7 to 2360·5)	1022·7 (831·4 to 1241·5)	490·8 (443·6 to 552·2)	497·4 (291·6 to 774·8)	0·0 (0·0 to 0·0)
Bahrain	2049	936·0 (796·4 to 1100·6)	474·2 (384·7 to 576·9)	226·1 (204·2 to 254·8)	235·8 (136·8 to 368·8)	0·0 (0·0 to 0·0)	2010·9 (1711·0 to 2364·5)	1018·7 (826·6 to 1239·4)	485·7 (438·8 to 547·3)	506·5 (294·0 to 792·4)	0·0 (0·0 to 0·0)
Bahrain	2050	938·0 (793·4 to 1107·9)	473·3 (383·0 to 578·0)	224·3 (202·6 to 253·1)	240·4 (138·5 to 379·0)	0·0 (0·0 to 0·0)	2015·2 (1704·6 to 2380·2)	1016·8 (822·9 to 1241·8)	481·9 (435·3 to 543·7)	516·5 (297·5 to 814·3)	0·0 (0·0 to 0·0)
The Bahamas	1995	1598·1 (1529·2 to 1666·2)	534·9 (484·1 to 583·8)	439·9 (414·6 to 466·0)	623·3 (589·8 to 657·7)	0·0 (0·0 to 0·0)	1799·6 (1722·1 to 1876·4)	602·3 (545·2 to 657·5)	495·3 (466·9 to 524·7)	702·0 (664·1 to 740·6)	0·0 (0·0 to 0·0)
The Bahamas	1996	1577·4 (1523·4 to 1631·9)	542·9 (499·7 to 588·1)	431·5 (412·8 to 451·4)	603·1 (578·8 to 628·5)	0·0 (0·0 to 0·0)	1776·4 (1715·5 to 1837·8)	611·4 (562·7 to 662·3)	485·9 (464·8 to 508·4)	679·1 (651·8 to 707·8)	0·0 (0·0 to 0·0)
The Bahamas	1997	1496·4 (1447·6 to 1550·4)	551·5 (511·5 to 597·3)	416·1 (398·2 to 434·6)	528·8 (504·6 to 552·9)	0·0 (0·0 to 0·0)	1685·1 (1630·2 to 1746·0)	621·0 (576·0 to 672·7)	468·6 (448·4 to 489·4)	595·5 (568·3 to 622·6)	0·0 (0·0 to 0·0)
The Bahamas	1998	1456·2 (1409·3 to 1510·3)	559·3 (519·2 to 601·7)	403·9 (386·0 to 421·0)	493·1 (469·4 to 518·7)	0·0 (0·0 to 0·0)	1639·9 (1587·1 to 1700·8)	629·8 (584·7 to 677·6)	454·8 (434·7 to 474·1)	555·3 (528·6 to 584·1)	0·0 (0·0 to 0·0)
The Bahamas	1999	1376·0 (1329·0 to 1423·3)	531·2 (493·0 to 574·5)	386·9 (370·7 to 402·7)	457·9 (437·5 to 479·2)	0·0 (0·0 to 0·0)	1549·6 (1496·6 to 1602·9)	598·2 (555·2 to 646·9)	435·7 (417·4 to 453·5)	515·7 (492·7 to 539·7)	0·0 (0·0 to 0·0)
The Bahamas	2000	1447·3 (1400·5 to 1499·3)	581·7 (540·4 to 631·0)	398·9 (386·4 to 411·7)	466·6 (450·4 to 484·5)	0·0 (0·0 to 0·0)	1629·9 (1577·2 to 1688·5)	655·1 (608·6 to 710·6)	449·3 (435·1 to 463·6)	525·5 (507·2 to 545·6)	0·0 (0·0 to 0·0)

The Bahamas	2001	1443·2 (1397·4 to 1492·2)	577·7 (535·9 to 623·8)	407·8 (396·5 to 419·7)	457·6 (440·8 to 473·1)	0·0 (0·0 to 0·0)	1625·2 (1573·7 to 1680·4)	650·6 (603·4 to 702·5)	459·3 (446·5 to 472·6)	515·3 (496·4 to 532·8)	0·0 (0·0 to 0·0)
The Bahamas	2002	1460·0 (1410·8 to 1507·7)	586·6 (545·6 to 629·6)	420·2 (409·0 to 431·9)	453·1 (437·6 to 470·3)	0·0 (0·0 to 0·0)	1644·1 (1588·7 to 1697·9)	660·6 (614·4 to 709·0)	473·2 (460·6 to 486·4)	510·3 (492·8 to 529·6)	0·0 (0·0 to 0·0)
The Bahamas	2003	1470·8 (1426·2 to 1517·3)	592·5 (550·8 to 636·8)	429·1 (416·6 to 441·7)	449·2 (433·1 to 465·4)	0·0 (0·0 to 0·0)	1656·3 (1606·1 to 1708·6)	667·2 (620·2 to 717·1)	483·2 (469·1 to 497·4)	505·9 (487·8 to 524·1)	0·0 (0·0 to 0·0)
The Bahamas	2004	1472·9 (1431·5 to 1517·6)	588·9 (548·8 to 630·4)	439·1 (426·9 to 451·2)	445·0 (430·1 to 461·0)	0·0 (0·0 to 0·0)	1658·7 (1612·1 to 1709·0)	663·2 (618·0 to 709·9)	494·4 (480·7 to 508·1)	501·1 (484·3 to 519·1)	0·0 (0·0 to 0·0)
The Bahamas	2005	1530·5 (1490·4 to 1576·5)	610·0 (571·5 to 653·8)	457·9 (446·4 to 470·0)	462·6 (446·5 to 478·4)	0·0 (0·0 to 0·0)	1723·5 (1678·4 to 1775·4)	686·9 (643·6 to 736·3)	515·6 (502·7 to 529·3)	521·0 (502·8 to 538·8)	0·0 (0·0 to 0·0)
The Bahamas	2006	1621·3 (1576·5 to 1671·0)	643·4 (602·4 to 688·4)	475·8 (464·9 to 487·1)	502·1 (485·0 to 519·1)	0·0 (0·0 to 0·0)	1825·8 (1775·3 to 1881·8)	724·6 (678·3 to 775·2)	535·8 (523·6 to 548·5)	565·5 (546·2 to 584·6)	0·0 (0·0 to 0·0)
The Bahamas	2007	1713·2 (1663·8 to 1762·2)	703·6 (660·4 to 748·0)	488·9 (477·3 to 500·5)	520·7 (502·6 to 538·1)	0·0 (0·0 to 0·0)	1929·3 (1873·7 to 1984·5)	792·4 (743·7 to 842·3)	550·5 (537·6 to 563·6)	586·4 (566·0 to 606·0)	0·0 (0·0 to 0·0)
The Bahamas	2008	1737·9 (1689·1 to 1787·3)	746·0 (702·2 to 789·0)	487·3 (475·4 to 499·3)	504·5 (487·2 to 521·7)	0·0 (0·0 to 0·0)	1957·0 (1902·1 to 2012·7)	840·1 (790·8 to 888·5)	548·8 (535·3 to 562·2)	568·1 (548·7 to 587·5)	0·0 (0·0 to 0·0)
The Bahamas	2009	1757·8 (1709·7 to 1810·2)	787·3 (741·6 to 832·1)	486·4 (474·8 to 499·1)	484·2 (468·2 to 501·1)	0·0 (0·0 to 0·0)	1979·5 (1925·3 to 2038·5)	886·6 (835·2 to 937·1)	547·7 (534·6 to 562·0)	545·2 (527·2 to 564·3)	0·0 (0·0 to 0·0)
The Bahamas	2010	1762·3 (1711·0 to 1812·7)	791·7 (744·9 to 838·7)	492·4 (480·3 to 505·0)	478·2 (462·0 to 496·0)	0·0 (0·0 to 0·0)	1984·5 (1926·8 to 2041·3)	891·5 (838·9 to 944·5)	554·5 (540·8 to 568·7)	538·5 (520·2 to 558·6)	0·0 (0·0 to 0·0)
The Bahamas	2011	1831·8 (1777·6 to 1883·5)	827·2 (781·2 to 873·7)	512·7 (501·2 to 525·7)	491·9 (474·9 to 509·6)	0·0 (0·0 to 0·0)	2062·8 (2001·8 to 2121·0)	931·5 (879·8 to 983·9)	577·4 (564·4 to 592·0)	553·9 (534·8 to 573·9)	0·0 (0·0 to 0·0)
The Bahamas	2012	1860·9 (1811·3 to 1910·2)	832·2 (785·6 to 878·7)	527·7 (514·9 to 540·0)	501·0 (485·0 to 518·0)	0·0 (0·0 to 0·0)	2095·6 (2039·7 to 2151·1)	937·2 (884·7 to 989·6)	594·2 (579·9 to 608·1)	564·2 (546·2 to 583·4)	0·0 (0·0 to 0·0)
The Bahamas	2013	1845·8 (1793·9 to 1899·6)	826·6 (780·9 to 874·6)	527·5 (515·6 to 541·0)	491·6 (474·5 to 508·4)	0·0 (0·0 to 0·0)	2078·6 (2020·2 to 2139·2)	930·9 (879·4 to 984·9)	594·1 (580·7 to 609·2)	553·7 (534·3 to 572·6)	0·0 (0·0 to 0·0)
The Bahamas	2014	1842·4 (1789·6 to 1893·8)	835·9 (787·8 to 880·9)	528·6 (516·2 to 540·9)	477·9 (461·5 to 494·2)	0·0 (0·0 to 0·0)	2074·8 (2015·3 to 2132·7)	941·3 (887·2 to 992·1)	595·3 (581·3 to 609·1)	538·2 (519·7 to 556·5)	0·0 (0·0 to 0·0)

The Bahamas	2015	1826·4 (1773·6 to 1875·3)	860·6 (812·3 to 905·6)	525·8 (512·6 to 538·0)	440·0 (424·2 to 457·4)	0·0 (0·0 to 0·0)	2056·8 (1997·3 to 2111·8)	969·1 (914·8 to 1019·8)	592·2 (577·2 to 605·8)	495·5 (477·7 to 515·1)	0·0 (0·0 to 0·0)
The Bahamas	2016	1917·2 (1864·6 to 1968·6)	958·8 (911·2 to 1009·0)	529·7 (516·7 to 541·6)	428·7 (412·9 to 445·8)	0·0 (0·0 to 0·0)	2159·0 (2099·7 to 2216·9)	1079·7 (1026·1 to 1136·2)	596·5 (581·8 to 609·9)	482·8 (465·0 to 502·0)	0·0 (0·0 to 0·0)
The Bahamas	2017	2171·7 (2117·4 to 2230·0)	1157·1 (1105·6 to 1212·4)	559·8 (547·5 to 573·0)	454·9 (438·6 to 471·3)	0·0 (0·0 to 0·0)	2445·6 (2384·4 to 2511·3)	1303·0 (1245·0 to 1365·3)	630·4 (616·5 to 645·3)	512·2 (493·9 to 530·8)	0·0 (0·0 to 0·0)
The Bahamas	2018	2140·2 (2063·7 to 2213·8)	1117·3 (1047·7 to 1180·7)	565·3 (549·8 to 581·9)	457·6 (436·9 to 479·0)	0·0 (0·0 to 0·0)	2410·2 (2324·0 to 2493·0)	1258·3 (1179·9 to 1329·6)	636·6 (619·2 to 655·2)	515·3 (492·0 to 539·4)	0·0 (0·0 to 0·0)
The Bahamas	2019	2191·2 (2110·5 to 2270·4)	1154·9 (1081·9 to 1224·6)	565·8 (550·3 to 582·2)	470·5 (448·4 to 492·9)	0·0 (0·0 to 0·0)	2467·5 (2376·7 to 2556·8)	1300·5 (1218·4 to 1379·0)	637·1 (619·7 to 655·7)	529·9 (504·9 to 555·1)	0·0 (0·0 to 0·0)
The Bahamas	2020	2228·4 (2143·4 to 2315·0)	1250·3 (1172·5 to 1328·7)	527·0 (512·4 to 542·2)	451·1 (428·9 to 474·7)	0·0 (0·0 to 0·0)	2509·4 (2413·7 to 2607·0)	1408·0 (1320·3 to 1496·3)	593·4 (577·0 to 610·6)	508·0 (483·0 to 534·5)	0·0 (0·0 to 0·0)
The Bahamas	2021	2180·4 (2095·6 to 2266·2)	1216·3 (1140·6 to 1292·6)	512·4 (498·0 to 527·4)	451·7 (427·5 to 478·9)	0·0 (0·0 to 0·0)	2455·4 (2360·0 to 2552·0)	1369·7 (1284·5 to 1455·6)	577·0 (560·8 to 593·9)	508·7 (481·4 to 539·3)	0·0 (0·0 to 0·0)
The Bahamas	2022	2102·2 (2014·3 to 2189·9)	1118·8 (1038·7 to 1190·0)	515·4 (500·8 to 531·1)	468·0 (438·9 to 501·3)	0·0 (0·0 to 0·0)	2367·3 (2268·4 to 2466·1)	1259·9 (1169·7 to 1340·1)	580·4 (563·9 to 598·1)	527·0 (494·3 to 564·5)	0·0 (0·0 to 0·0)
The Bahamas	2023	2153·6 (2057·7 to 2248·9)	1149·3 (1065·3 to 1229·0)	519·0 (503·6 to 535·2)	485·2 (450·7 to 525·1)	0·0 (0·0 to 0·0)	2425·2 (2317·3 to 2532·5)	1294·3 (1199·7 to 1384·0)	584·5 (567·1 to 602·7)	546·4 (507·5 to 591·3)	0·0 (0·0 to 0·0)
The Bahamas	2024	2205·7 (2100·0 to 2314·0)	1180·8 (1089·3 to 1265·6)	522·3 (507·1 to 538·3)	502·6 (460·3 to 551·8)	0·0 (0·0 to 0·0)	2483·9 (2364·8 to 2605·8)	1329·7 (1226·7 to 1425·3)	588·2 (571·1 to 606·2)	566·0 (518·4 to 621·4)	0·0 (0·0 to 0·0)
The Bahamas	2025	2256·1 (2144·3 to 2370·6)	1211·0 (1115·9 to 1304·7)	525·1 (509·8 to 541·2)	520·1 (470·0 to 578·0)	0·0 (0·0 to 0·0)	2540·7 (2414·7 to 2669·6)	1363·7 (1256·6 to 1469·2)	591·3 (574·2 to 609·5)	585·7 (529·3 to 650·9)	0·0 (0·0 to 0·0)
The Bahamas	2026	2307·5 (2186·3 to 2437·2)	1241·8 (1143·9 to 1344·1)	527·7 (512·5 to 544·2)	538·0 (478·5 to 606·3)	0·0 (0·0 to 0·0)	2598·6 (2462·1 to 2744·6)	1398·4 (1288·1 to 1513·6)	594·3 (577·2 to 612·8)	605·9 (538·9 to 682·7)	0·0 (0·0 to 0·0)
The Bahamas	2027	2357·9 (2226·1 to 2493·8)	1272·4 (1166·5 to 1377·2)	529·8 (514·1 to 546·1)	555·8 (488·2 to 635·1)	0·0 (0·0 to 0·0)	2655·3 (2506·9 to 2808·4)	1432·8 (1313·7 to 1550·9)	596·6 (579·0 to 615·0)	625·9 (549·8 to 715·2)	0·0 (0·0 to 0·0)
The Bahamas	2028	2404·2 (2264·3 to 2546·1)	1300·1 (1190·3 to 1411·2)	530·9 (515·0 to 547·0)	573·2 (497·2 to 662·6)	0·0 (0·0 to 0·0)	2707·5 (2549·9 to 2867·2)	1464·1 (1340·4 to 1589·1)	597·9 (580·0 to 616·0)	645·5 (559·9 to 746·2)	0·0 (0·0 to 0·0)

The Bahamas	2029	2451·1 (2301·3 to 2603·6)	1328·5 (1206·4 to 1446·0)	531·7 (515·7 to 547·7)	590·9 (506·2 to 692·8)	0·0 (0·0 to 0·0)	2760·2 (2591·5 to 2932·0)	1496·0 (1358·5 to 1628·3)	598·8 (580·7 to 616·8)	665·4 (570·1 to 780·1)	0·0 (0·0 to 0·0)
The Bahamas	2030	2493·1 (2337·0 to 2655·6)	1356·8 (1236·3 to 1479·7)	531·9 (515·7 to 548·2)	604·4 (517·3 to 710·5)	0·0 (0·0 to 0·0)	2807·5 (2631·7 to 2990·6)	1527·9 (1392·2 to 1666·3)	599·0 (580·8 to 617·3)	680·6 (582·5 to 800·1)	0·0 (0·0 to 0·0)
The Bahamas	2031	2533·1 (2373·5 to 2698·9)	1384·8 (1255·8 to 1518·9)	531·8 (515·7 to 548·5)	616·5 (527·6 to 725·0)	0·0 (0·0 to 0·0)	2852·6 (2672·9 to 3039·3)	1559·5 (1414·1 to 1710·5)	598·9 (580·7 to 617·7)	694·2 (594·1 to 816·5)	0·0 (0·0 to 0·0)
The Bahamas	2032	2571·9 (2403·9 to 2751·5)	1412·1 (1279·7 to 1561·4)	531·3 (514·9 to 548·0)	628·5 (536·1 to 739·5)	0·0 (0·0 to 0·0)	2896·2 (2707·1 to 3098·6)	1590·3 (1441·1 to 1758·3)	598·3 (579·9 to 617·1)	707·7 (603·8 to 832·8)	0·0 (0·0 to 0·0)
The Bahamas	2033	2608·2 (2432·2 to 2788·8)	1437·7 (1299·2 to 1584·9)	530·2 (513·6 to 547·0)	640·2 (544·4 to 754·4)	0·0 (0·0 to 0·0)	2937·1 (2739·0 to 3140·6)	1619·1 (1463·1 to 1784·7)	597·1 (578·3 to 616·0)	720·9 (613·0 to 849·6)	0·0 (0·0 to 0·0)
The Bahamas	2034	2641·3 (2462·0 to 2830·0)	1461·0 (1313·7 to 1614·3)	528·7 (512·0 to 545·6)	651·6 (554·0 to 767·2)	0·0 (0·0 to 0·0)	2974·5 (2772·5 to 3187·0)	1645·2 (1479·4 to 1818·0)	595·4 (576·6 to 614·4)	733·8 (623·9 to 864·0)	0·0 (0·0 to 0·0)
The Bahamas	2035	2673·0 (2486·5 to 2866·6)	1483·0 (1336·4 to 1636·1)	527·0 (510·0 to 543·8)	663·0 (562·4 to 779·0)	0·0 (0·0 to 0·0)	3010·1 (2800·2 to 3228·1)	1670·1 (1505·0 to 1842·5)	593·4 (574·4 to 612·4)	746·7 (633·4 to 877·2)	0·0 (0·0 to 0·0)
The Bahamas	2036	2705·4 (2514·2 to 2907·2)	1505·5 (1353·0 to 1667·9)	525·2 (507·9 to 542·0)	674·7 (570·2 to 794·2)	0·0 (0·0 to 0·0)	3046·6 (2831·3 to 3273·9)	1695·4 (1523·6 to 1878·2)	591·4 (572·0 to 610·3)	759·8 (642·1 to 894·4)	0·0 (0·0 to 0·0)
The Bahamas	2037	2738·5 (2541·3 to 2945·8)	1527·8 (1370·9 to 1699·1)	523·7 (506·4 to 540·5)	687·1 (579·0 to 812·6)	0·0 (0·0 to 0·0)	3083·9 (2861·8 to 3317·4)	1720·5 (1543·8 to 1913·4)	589·7 (570·2 to 608·6)	773·7 (652·0 to 915·1)	0·0 (0·0 to 0·0)
The Bahamas	2038	2771·3 (2565·6 to 2982·8)	1549·2 (1383·8 to 1724·8)	522·3 (504·6 to 539·3)	699·9 (588·3 to 830·5)	0·0 (0·0 to 0·0)	3120·9 (2889·2 to 3359·0)	1744·6 (1558·3 to 1942·3)	588·1 (568·2 to 607·3)	788·1 (662·5 to 935·3)	0·0 (0·0 to 0·0)
The Bahamas	2039	2803·5 (2589·9 to 3023·8)	1570·1 (1400·5 to 1749·5)	520·7 (502·7 to 537·8)	712·7 (596·6 to 848·0)	0·0 (0·0 to 0·0)	3157·1 (2916·5 to 3405·2)	1768·1 (1577·1 to 1970·1)	586·4 (566·1 to 605·6)	802·6 (671·9 to 955·0)	0·0 (0·0 to 0·0)
The Bahamas	2040	2840·0 (2619·2 to 3072·0)	1593·4 (1421·6 to 1782·4)	519·8 (501·6 to 537·2)	726·8 (606·4 to 866·0)	0·0 (0·0 to 0·0)	3198·2 (2949·5 to 3459·5)	1794·4 (1600·9 to 2007·2)	585·3 (564·8 to 605·0)	818·5 (682·9 to 975·2)	0·0 (0·0 to 0·0)
The Bahamas	2041	2875·1 (2642·6 to 3111·6)	1615·0 (1435·0 to 1808·7)	518·8 (500·5 to 536·4)	741·2 (616·8 to 886·4)	0·0 (0·0 to 0·0)	3237·7 (2975·9 to 3504·0)	1818·7 (1616·0 to 2036·8)	584·2 (563·6 to 604·1)	834·7 (694·6 to 998·2)	0·0 (0·0 to 0·0)
The Bahamas	2042	2912·3 (2676·8 to 3159·2)	1637·6 (1446·4 to 1837·0)	518·2 (499·8 to 536·4)	756·6 (626·1 to 909·2)	0·0 (0·0 to 0·0)	3279·7 (3014·4 to 3557·6)	1844·1 (1628·8 to 2068·7)	583·5 (562·8 to 604·0)	852·1 (705·0 to 1023·9)	0·0 (0·0 to 0·0)

The Bahamas	2043	2950·9 (2705·7 to 3207·8)	1659·8 (1465·3 to 1863·2)	518·0 (499·4 to 536·7)	773·1 (635·4 to 938·1)	0·0 (0·0 to 0·0)	3323·1 (3046·9 to 3612·4)	1869·1 (1650·2 to 2098·2)	583·3 (562·4 to 604·4)	870·7 (715·5 to 1056·4)	0·0 (0·0 to 0·0)
The Bahamas	2044	2989·8 (2738·3 to 3258·1)	1681·7 (1480·5 to 1888·7)	517·9 (498·8 to 536·8)	790·2 (646·0 to 959·3)	0·0 (0·0 to 0·0)	3366·8 (3083·7 to 3669·0)	1893·8 (1667·2 to 2127·0)	583·2 (561·8 to 604·5)	889·9 (727·5 to 1080·3)	0·0 (0·0 to 0·0)
The Bahamas	2045	3029·0 (2769·8 to 3311·4)	1703·1 (1496·4 to 1917·0)	517·9 (498·4 to 537·0)	808·0 (657·4 to 986·7)	0·0 (0·0 to 0·0)	3411·0 (3119·1 to 3729·1)	1917·9 (1685·2 to 2158·7)	583·2 (561·2 to 604·8)	909·9 (740·3 to 1111·1)	0·0 (0·0 to 0·0)
The Bahamas	2046	3068·8 (2797·7 to 3359·3)	1724·3 (1514·4 to 1938·6)	518·0 (498·0 to 537·5)	826·4 (669·5 to 1015·7)	0·0 (0·0 to 0·0)	3455·8 (3150·6 to 3783·0)	1941·8 (1705·4 to 2183·1)	583·3 (560·8 to 605·3)	930·7 (754·0 to 1143·8)	0·0 (0·0 to 0·0)
The Bahamas	2047	3110·6 (2831·8 to 3412·8)	1746·6 (1532·0 to 1978·3)	518·3 (498·2 to 538·0)	845·6 (681·3 to 1046·0)	0·0 (0·0 to 0·0)	3502·9 (3189·0 to 3843·2)	1966·9 (1725·2 to 2227·8)	583·7 (561·0 to 605·9)	952·3 (767·3 to 1177·9)	0·0 (0·0 to 0·0)
The Bahamas	2048	3151·6 (2867·4 to 3463·2)	1767·7 (1545·9 to 2002·4)	518·6 (498·2 to 538·6)	865·3 (693·8 to 1077·1)	0·0 (0·0 to 0·0)	3549·1 (3229·1 to 3899·9)	1990·6 (1740·9 to 2255·0)	584·0 (561·1 to 606·5)	974·4 (781·3 to 1212·9)	0·0 (0·0 to 0·0)
The Bahamas	2049	3192·6 (2896·3 to 3514·5)	1788·5 (1561·3 to 2030·0)	518·8 (497·8 to 539·0)	885·3 (706·3 to 1108·9)	0·0 (0·0 to 0·0)	3595·3 (3261·6 to 3957·8)	2014·1 (1758·2 to 2286·0)	584·2 (560·6 to 606·9)	996·9 (795·4 to 1248·8)	0·0 (0·0 to 0·0)
The Bahamas	2050	3237·6 (2932·2 to 3571·9)	1811·9 (1580·5 to 2063·1)	519·3 (497·8 to 539·7)	906·3 (719·7 to 1142·4)	0·0 (0·0 to 0·0)	3646·0 (3302·0 to 4022·4)	2040·5 (1779·9 to 2323·3)	584·8 (560·6 to 607·8)	1020·6 (810·4 to 1286·5)	0·0 (0·0 to 0·0)
Bosnia and Herzegovina*	1995	100·5 (88·5 to 112·8)	38·9 (33·1 to 45·6)	61·1 (50·5 to 72·6)	0·0 (0·0 to 0·0)	0·4 (0·4 to 0·4)	259·6 (228·8 to 291·4)	100·7 (85·4 to 117·8)	157·8 (130·4 to 187·6)	0·0 (0·0 to 0·0)	1·1 (1·1 to 1·1)
Bosnia and Herzegovina*	1996	150·0 (135·0 to 166·1)	61·2 (52·7 to 70·3)	83·5 (69·9 to 97·9)	0·0 (0·0 to 0·0)	5·4 (5·4 to 5·4)	387·8 (348·8 to 429·2)	158·1 (136·1 to 181·8)	215·7 (180·7 to 253·1)	0·0 (0·0 to 0·0)	14·0 (14·0 to 14·0)
Bosnia and Herzegovina*	1997	175·0 (158·6 to 191·7)	75·1 (65·3 to 85·5)	98·6 (84·3 to 113·4)	0·0 (0·0 to 0·0)	1·3 (1·3 to 1·3)	452·3 (410·0 to 495·3)	194·1 (168·9 to 221·1)	254·9 (217·9 to 293·2)	0·1 (0·1 to 0·1)	3·3 (3·3 to 3·3)
Bosnia and Herzegovina*	1998	201·9 (185·3 to 219·5)	86·9 (76·7 to 98·1)	107·9 (94·5 to 122·6)	0·0 (0·0 to 0·0)	7·2 (7·2 to 7·2)	521·9 (478·9 to 567·3)	224·5 (198·3 to 253·5)	278·8 (244·1 to 316·8)	0·1 (0·1 to 0·1)	18·5 (18·5 to 18·5)
Bosnia and Herzegovina*	1999	235·4 (217·6 to 254·7)	107·3 (95·8 to 119·7)	108·2 (95·5 to 122·9)	0·0 (0·0 to 0·0)	19·8 (19·8 to 19·8)	608·3 (562·3 to 658·2)	277·4 (247·5 to 309·3)	279·7 (246·7 to 317·6)	0·1 (0·0 to 0·1)	51·1 (51·1 to 51·1)
Bosnia and Herzegovina*	2000	221·6 (204·8 to 239·7)	119·2 (107·0 to 131·8)	97·7 (86·3 to 111·0)	0·0 (0·0 to 0·0)	4·7 (4·7 to 4·7)	572·8 (529·3 to 619·4)	308·1 (276·4 to 340·6)	252·6 (223·0 to 286·9)	0·1 (0·0 to 0·1)	12·0 (12·0 to 12·0)

Bosnia and Herzegovina*	2001	220·3 (204·4 to 238·0)	125·3 (113·6 to 137·1)	91·0 (79·7 to 103·4)	0·0 (0·0 to 0·0)	4·0 (4·0 to 4·0)	569·4 (528·1 to 615·0)	323·8 (293·5 to 354·4)	235·3 (206·0 to 267·3)	0·1 (0·0 to 0·1)	10·3 (10·3 to 10·3)
Bosnia and Herzegovina*	2002	232·3 (216·9 to 249·0)	136·9 (125·4 to 148·7)	91·0 (80·1 to 103·0)	0·0 (0·0 to 0·0)	4·4 (4·4 to 4·4)	600·4 (560·5 to 643·5)	353·7 (324·1 to 384·4)	235·2 (207·0 to 266·1)	0·0 (0·0 to 0·1)	11·4 (11·4 to 11·4)
Bosnia and Herzegovina*	2003	256·7 (240·1 to 274·2)	153·3 (141·1 to 166·4)	99·1 (88·0 to 110·7)	0·0 (0·0 to 0·0)	4·3 (4·3 to 4·3)	663·5 (620·5 to 708·5)	396·1 (364·7 to 429·9)	256·2 (227·4 to 286·2)	0·0 (0·0 to 0·1)	11·2 (11·2 to 11·2)
Bosnia and Herzegovina*	2004	284·6 (266·4 to 302·8)	163·2 (150·8 to 176·8)	118·8 (106·8 to 132·0)	0·0 (0·0 to 0·0)	2·7 (2·7 to 2·7)	735·6 (688·6 to 782·6)	421·7 (389·6 to 456·8)	307·0 (275·9 to 341·2)	0·0 (0·0 to 0·1)	6·9 (6·9 to 6·9)
Bosnia and Herzegovina*	2005	315·8 (297·3 to 333·4)	174·9 (162·5 to 188·4)	137·2 (124·9 to 150·5)	0·0 (0·0 to 0·0)	3·6 (3·6 to 3·6)	816·1 (768·4 to 861·7)	452·0 (419·9 to 487·0)	354·6 (322·9 to 388·8)	0·0 (0·0 to 0·1)	9·4 (9·4 to 9·4)
Bosnia and Herzegovina*	2006	343·9 (325·4 to 362·5)	189·0 (176·6 to 202·6)	150·8 (139·0 to 164·5)	0·0 (0·0 to 0·0)	4·1 (4·1 to 4·1)	888·8 (841·0 to 936·9)	488·5 (456·4 to 523·5)	389·8 (359·2 to 425·0)	0·0 (0·0 to 0·1)	10·5 (10·5 to 10·5)
Bosnia and Herzegovina*	2007	371·1 (351·8 to 391·4)	208·6 (194·5 to 223·1)	157·9 (145·0 to 171·3)	0·0 (0·0 to 0·0)	4·5 (4·5 to 4·5)	959·0 (909·1 to 1011·5)	539·2 (502·7 to 576·6)	408·1 (374·7 to 442·7)	0·0 (0·0 to 0·1)	11·7 (11·7 to 11·7)
Bosnia and Herzegovina*	2008	410·7 (389·8 to 431·4)	237·8 (223·4 to 252·8)	155·2 (142·1 to 169·7)	0·0 (0·0 to 0·0)	17·7 (17·7 to 17·7)	1061·3 (1007·3 to 1114·9)	614·5 (577·4 to 653·2)	401·1 (367·2 to 438·6)	0·0 (0·0 to 0·1)	45·8 (45·8 to 45·8)
Bosnia and Herzegovina*	2009	409·3 (388·7 to 429·8)	253·1 (238·4 to 269·4)	146·8 (133·1 to 161·3)	1·1 (0·8 to 1·6)	8·3 (8·3 to 8·3)	1057·8 (1004·6 to 1110·8)	654·1 (616·1 to 696·2)	379·4 (344·0 to 416·8)	2·8 (2·0 to 4·0)	21·5 (21·5 to 21·5)
Bosnia and Herzegovina*	2010	422·6 (401·7 to 443·7)	271·7 (256·3 to 288·5)	134·6 (121·3 to 148·0)	1·1 (0·8 to 1·6)	15·2 (15·2 to 15·2)	1092·0 (1038·0 to 1146·6)	702·1 (662·5 to 745·5)	347·8 (313·6 to 382·6)	2·9 (2·0 to 4·1)	39·3 (39·3 to 39·3)
Bosnia and Herzegovina*	2011	416·6 (396·1 to 436·9)	278·5 (263·9 to 294·7)	127·4 (115·0 to 140·2)	1·1 (0·7 to 1·5)	9·6 (9·6 to 9·6)	1076·5 (1023·7 to 1129·1)	719·6 (682·1 to 761·5)	329·2 (297·3 to 362·2)	2·8 (1·9 to 3·9)	24·9 (24·9 to 24·9)
Bosnia and Herzegovina*	2012	431·6 (412·2 to 452·8)	295·0 (280·3 to 310·6)	126·8 (114·4 to 139·9)	1·1 (0·7 to 1·5)	8·8 (8·8 to 8·8)	1115·5 (1065·3 to 1170·1)	762·3 (724·3 to 802·7)	327·7 (295·7 to 361·7)	2·8 (1·9 to 3·9)	22·8 (22·8 to 22·8)
Bosnia and Herzegovina*	2013	441·8 (420·8 to 463·2)	305·7 (289·9 to 321·2)	128·0 (116·1 to 141·1)	1·1 (0·8 to 1·6)	7·0 (7·0 to 7·0)	1141·8 (1087·4 to 1197·2)	790·0 (749·2 to 830·0)	330·9 (300·0 to 364·7)	2·9 (2·0 to 4·2)	18·0 (18·0 to 18·0)
Bosnia and Herzegovina*	2014	465·8 (443·5 to 488·0)	323·6 (307·5 to 340·1)	131·9 (119·4 to 145·6)	1·3 (0·9 to 1·8)	9·0 (9·0 to 9·0)	1203·9 (1146·2 to 1261·1)	836·3 (794·6 to 878·9)	341·0 (308·5 to 376·2)	3·3 (2·2 to 4·6)	23·4 (23·4 to 23·4)

Bosnia and Herzegovina*	2015	468·0 (446·5 to 488·0)	329·1 (313·3 to 345·5)	135·0 (122·3 to 147·5)	1·4 (0·9 to 2·0)	2·6 (2·6 to 2·6)	1209·6 (1153·9 to 1261·2)	850·6 (809·7 to 892·9)	348·8 (316·2 to 381·1)	3·6 (2·4 to 5·1)	6·6 (6·6 to 6·6)
Bosnia and Herzegovina*	2016	492·7 (472·0 to 513·5)	340·1 (324·5 to 356·6)	139·4 (126·7 to 152·3)	1·5 (1·1 to 2·1)	11·7 (11·7 to 11·7)	1273·4 (1219·9 to 1327·1)	878·9 (838·7 to 921·6)	360·3 (327·3 to 393·5)	3·9 (2·7 to 5·5)	30·3 (30·3 to 30·3)
Bosnia and Herzegovina*	2017	501·1 (478·3 to 521·9)	346·4 (329·2 to 364·8)	144·1 (130·6 to 158·8)	1·8 (1·2 to 2·5)	8·8 (8·8 to 8·8)	1295·0 (1236·1 to 1348·8)	895·2 (850·8 to 942·8)	372·3 (337·6 to 410·4)	4·6 (3·2 to 6·4)	22·8 (22·8 to 22·8)
Bosnia and Herzegovina*	2018	527·7 (500·3 to 557·6)	364·7 (342·3 to 387·7)	151·3 (134·2 to 169·7)	2·0 (1·4 to 2·8)	9·8 (9·8 to 9·8)	1363·8 (1292·9 to 1441·0)	942·5 (884·6 to 1002·1)	391·0 (346·8 to 438·5)	5·2 (3·6 to 7·3)	25·2 (25·2 to 25·2)
Bosnia and Herzegovina*	2019	548·1 (516·8 to 581·7)	380·0 (352·6 to 408·2)	156·4 (138·8 to 175·4)	2·1 (1·5 to 3·0)	9·6 (9·5 to 9·7)	1416·4 (1335·5 to 1503·3)	982·0 (911·3 to 1055·0)	404·2 (358·7 to 453·3)	5·5 (3·8 to 7·7)	24·8 (24·6 to 25·2)
Bosnia and Herzegovina*	2020	569·3 (536·0 to 606·6)	405·6 (374·4 to 437·7)	148·4 (131·6 to 166·8)	2·1 (1·4 to 2·9)	13·2 (12·9 to 14·0)	1471·3 (1385·3 to 1567·7)	1048·3 (967·6 to 1131·0)	383·6 (340·2 to 431·0)	5·3 (3·7 to 7·4)	34·1 (33·3 to 36·2)
Bosnia and Herzegovina*	2021	551·9 (519·6 to 588·1)	393·1 (362·8 to 424·1)	143·8 (127·6 to 161·9)	2·0 (1·4 to 2·8)	13·0 (12·5 to 13·7)	1426·4 (1342·9 to 1519·8)	1015·9 (937·7 to 1096·1)	371·7 (329·6 to 418·3)	5·2 (3·6 to 7·4)	33·5 (32·3 to 35·5)
Bosnia and Herzegovina*	2022	533·6 (501·7 to 569·4)	375·9 (347·3 to 406·4)	145·3 (129·0 to 163·7)	2·1 (1·4 to 2·9)	10·2 (9·5 to 11·1)	1378·9 (1296·6 to 1471·5)	971·6 (897·5 to 1050·3)	375·6 (333·5 to 423·0)	5·4 (3·7 to 7·6)	26·3 (24·6 to 28·7)
Bosnia and Herzegovina*	2023	547·0 (513·5 to 583·7)	387·4 (357·3 to 419·6)	146·8 (130·5 to 165·4)	2·2 (1·5 to 3·1)	10·6 (9·8 to 11·6)	1413·5 (1327·1 to 1508·5)	1001·2 (923·4 to 1084·4)	379·5 (337·2 to 427·4)	5·6 (3·9 to 7·9)	27·3 (25·2 to 30·0)
Bosnia and Herzegovina*	2024	559·9 (526·1 to 597·9)	398·5 (367·6 to 431·6)	148·1 (131·7 to 166·9)	2·3 (1·5 to 3·2)	11·0 (10·1 to 12·1)	1447·0 (1359·6 to 1545·2)	1029·9 (950·0 to 1115·4)	382·8 (340·3 to 431·3)	5·8 (4·0 to 8·2)	28·4 (26·1 to 31·3)
Bosnia and Herzegovina*	2025	572·8 (538·5 to 611·9)	409·6 (377·8 to 443·9)	149·4 (132·8 to 168·2)	2·3 (1·6 to 3·3)	11·4 (10·4 to 12·6)	1480·3 (1391·7 to 1581·3)	1058·6 (976·5 to 1147·1)	386·1 (343·2 to 434·8)	6·0 (4·1 to 8·5)	29·5 (27·0 to 32·6)
Bosnia and Herzegovina*	2026	585·3 (549·9 to 625·1)	420·7 (389·0 to 457·5)	150·3 (133·6 to 169·2)	2·4 (1·7 to 3·4)	11·9 (10·8 to 13·2)	1512·6 (1421·2 to 1615·6)	1087·2 (1005·3 to 1182·3)	388·4 (345·3 to 437·2)	6·3 (4·3 to 8·8)	30·7 (27·9 to 34·2)
Bosnia and Herzegovina*	2027	599·1 (562·5 to 640·7)	432·8 (398·6 to 470·3)	151·5 (134·8 to 170·6)	2·5 (1·7 to 3·5)	12·2 (11·1 to 13·7)	1548·2 (1453·7 to 1655·7)	1118·5 (1030·1 to 1215·5)	391·6 (348·3 to 440·8)	6·5 (4·5 to 9·2)	31·6 (28·6 to 35·5)
Bosnia and Herzegovina*	2028	613·4 (575·1 to 656·2)	445·4 (409·8 to 483·3)	152·8 (135·9 to 171·9)	2·6 (1·8 to 3·7)	12·6 (11·3 to 14·3)	1585·2 (1486·3 to 1695·7)	1151·0 (1059·2 to 1248·9)	394·9 (351·3 to 444·2)	6·7 (4·6 to 9·5)	32·5 (29·1 to 37·1)

Bosnia and Herzegovina*	2029	628·4 (589·2 to 672·3)	458·5 (422·3 to 498·3)	154·2 (137·2 to 173·5)	2·7 (1·9 to 3·8)	12·9 (11·5 to 14·8)	1623·9 (1522·7 to 1737·4)	1184·9 (1091·4 to 1287·8)	398·6 (354·5 to 448·3)	7·0 (4·8 to 9·9)	33·4 (29·7 to 38·2)
Bosnia and Herzegovina*	2030	643·8 (603·7 to 689·6)	472·1 (434·1 to 512·5)	155·7 (138·5 to 175·1)	2·8 (1·9 to 4·0)	13·2 (11·7 to 15·2)	1663·9 (1560·1 to 1782·2)	1220·1 (1121·8 to 1324·4)	402·4 (357·8 to 452·6)	7·3 (5·0 to 10·3)	34·2 (30·2 to 39·3)
Bosnia and Herzegovina*	2031	659·2 (617·9 to 706·0)	485·6 (445·6 to 529·0)	157·2 (139·7 to 176·8)	2·9 (2·0 to 4·1)	13·6 (11·9 to 15·7)	1703·7 (1596·9 to 1824·5)	1254·9 (1151·6 to 1367·2)	406·1 (361·1 to 456·8)	7·5 (5·2 to 10·7)	35·1 (30·7 to 40·6)
Bosnia and Herzegovina*	2032	674·8 (631·5 to 723·2)	499·1 (457·5 to 543·0)	158·7 (141·1 to 178·6)	3·0 (2·1 to 4·3)	13·9 (12·1 to 16·2)	1743·9 (1631·9 to 1869·0)	1289·9 (1182·4 to 1403·3)	410·2 (364·6 to 461·5)	7·8 (5·3 to 11·1)	35·9 (31·4 to 41·9)
Bosnia and Herzegovina*	2033	690·4 (645·9 to 739·8)	512·7 (469·8 to 557·5)	160·4 (142·5 to 180·5)	3·1 (2·1 to 4·4)	14·2 (12·3 to 16·6)	1784·3 (1669·2 to 1911·8)	1325·0 (1214·2 to 1440·9)	414·4 (368·1 to 466·5)	8·1 (5·5 to 11·5)	36·7 (31·8 to 43·0)
Bosnia and Herzegovina*	2034	706·1 (659·8 to 756·0)	526·2 (480·6 to 572·0)	162·1 (143·9 to 182·4)	3·3 (2·2 to 4·6)	14·5 (12·6 to 17·2)	1824·7 (1705·1 to 1953·8)	1359·8 (1242·1 to 1478·3)	418·9 (371·9 to 471·4)	8·4 (5·7 to 11·9)	37·6 (32·5 to 44·4)
Bosnia and Herzegovina*	2035	721·2 (673·1 to 773·0)	539·2 (493·0 to 585·9)	163·8 (145·4 to 184·3)	3·4 (2·3 to 4·8)	14·9 (12·8 to 17·8)	1863·9 (1739·4 to 1997·6)	1393·4 (1274·0 to 1514·3)	423·4 (375·7 to 476·4)	8·7 (5·9 to 12·4)	38·5 (33·0 to 46·0)
Bosnia and Herzegovina*	2036	736·5 (687·1 to 791·0)	552·2 (503·6 to 601·2)	165·6 (147·0 to 186·3)	3·5 (2·3 to 5·0)	15·2 (13·0 to 18·4)	1903·4 (1775·7 to 2044·3)	1427·1 (1301·4 to 1553·8)	427·9 (379·9 to 481·4)	9·0 (6·0 to 12·8)	39·4 (33·6 to 47·6)
Bosnia and Herzegovina*	2037	750·8 (699·0 to 806·7)	564·4 (513·1 to 616·1)	167·1 (148·4 to 188·0)	3·6 (2·4 to 5·1)	15·7 (13·3 to 19·1)	1940·4 (1806·6 to 2084·8)	1458·7 (1325·9 to 1592·3)	431·8 (383·5 to 485·8)	9·3 (6·2 to 13·2)	40·6 (34·5 to 49·4)
Bosnia and Herzegovina*	2038	765·3 (712·3 to 822·9)	576·8 (527·7 to 630·8)	168·6 (149·7 to 189·7)	3·7 (2·5 to 5·3)	16·2 (13·6 to 19·8)	1977·8 (1840·8 to 2126·8)	1490·7 (1363·9 to 1630·3)	435·6 (386·8 to 490·2)	9·7 (6·4 to 13·7)	41·8 (35·2 to 51·1)
Bosnia and Herzegovina*	2039	780·2 (723·6 to 841·4)	589·5 (536·0 to 647·2)	170·1 (150·9 to 191·4)	3·9 (2·5 to 5·5)	16·7 (14·0 to 20·5)	2016·3 (1870·1 to 2174·5)	1523·4 (1385·1 to 1672·6)	439·6 (390·1 to 494·7)	10·0 (6·6 to 14·2)	43·2 (36·1 to 53·1)
Bosnia and Herzegovina*	2040	795·5 (737·6 to 857·8)	602·5 (546·0 to 661·4)	171·8 (152·3 to 193·3)	4·0 (2·6 to 5·7)	17·3 (14·5 to 21·6)	2055·9 (1906·3 to 2216·9)	1557·1 (1411·0 to 1709·3)	443·9 (393·7 to 499·6)	10·4 (6·8 to 14·7)	44·6 (37·4 to 55·8)
Bosnia and Herzegovina*	2041	810·7 (750·5 to 875·0)	615·3 (557·5 to 676·3)	173·4 (153·9 to 195·2)	4·2 (2·7 to 5·9)	17·8 (14·7 to 22·3)	2095·1 (1939·6 to 2261·3)	1590·2 (1440·7 to 1747·7)	448·2 (397·8 to 504·5)	10·7 (7·0 to 15·3)	46·0 (38·1 to 57·6)
Bosnia and Herzegovina*	2042	826·1 (762·8 to 894·4)	628·2 (567·8 to 690·7)	175·2 (155·5 to 197·3)	4·3 (2·8 to 6·2)	18·4 (15·2 to 23·4)	2134·9 (1971·3 to 2311·3)	1623·5 (1467·4 to 1784·9)	452·8 (401·9 to 509·8)	11·1 (7·2 to 15·9)	47·5 (39·2 to 60·4)

Bosnia and Herzegovina*	2043	841·5 (777·4 to 911·5)	641·0 (578·6 to 705·7)	177·0 (157·1 to 199·3)	4·5 (2·9 to 6·4)	19·0 (15·5 to 24·3)	2174·7 (2009·1 to 2355·7)	1656·7 (1495·4 to 1823·7)	457·5 (406·0 to 515·1)	11·5 (7·5 to 16·5)	49·0 (40·1 to 62·8)
Bosnia and Herzegovina*	2044	857·0 (792·6 to 929·5)	653·8 (590·2 to 719·1)	179·0 (158·8 to 201·6)	4·6 (3·0 to 6·6)	19·5 (15·9 to 25·3)	2214·8 (2048·5 to 2402·2)	1689·7 (1525·2 to 1858·4)	462·7 (410·5 to 521·0)	11·9 (7·7 to 17·1)	50·5 (41·1 to 65·3)
Bosnia and Herzegovina*	2045	872·4 (803·4 to 948·0)	666·5 (599·4 to 735·1)	181·0 (160·6 to 203·8)	4·8 (3·1 to 6·9)	20·1 (16·3 to 26·2)	2254·6 (2076·4 to 2450·0)	1722·5 (1549·0 to 1899·7)	467·8 (414·9 to 526·7)	12·4 (8·0 to 17·8)	52·0 (42·1 to 67·8)
Bosnia and Herzegovina*	2046	888·1 (816·9 to 963·3)	679·4 (609·6 to 748·8)	183·1 (162·4 to 206·2)	5·0 (3·2 to 7·2)	20·7 (16·7 to 27·5)	2295·3 (2111·1 to 2489·5)	1755·7 (1575·4 to 1935·1)	473·2 (419·7 to 532·9)	12·8 (8·2 to 18·6)	53·5 (43·1 to 71·0)
Bosnia and Herzegovina*	2047	904·0 (830·0 to 981·6)	692·3 (619·4 to 765·6)	185·2 (164·2 to 208·5)	5·1 (3·3 to 7·5)	21·4 (17·0 to 28·7)	2336·2 (2144·9 to 2536·8)	1789·1 (1600·7 to 1978·7)	478·6 (424·3 to 538·8)	13·3 (8·5 to 19·3)	55·2 (44·0 to 74·1)
Bosnia and Herzegovina*	2048	919·4 (842·2 to 1000·7)	704·8 (627·4 to 780·3)	187·2 (166·0 to 210·7)	5·3 (3·4 to 7·7)	22·0 (17·6 to 29·9)	2376·1 (2176·5 to 2586·2)	1821·6 (1621·5 to 2016·6)	483·8 (428·9 to 544·6)	13·8 (8·8 to 20·0)	56·9 (45·4 to 77·4)
Bosnia and Herzegovina*	2049	935·1 (858·0 to 1019·5)	717·5 (641·4 to 795·4)	189·3 (167·7 to 213·0)	5·5 (3·5 to 8·1)	22·8 (18·1 to 31·5)	2416·6 (2217·3 to 2634·8)	1854·4 (1657·6 to 2055·5)	489·1 (433·5 to 550·5)	14·3 (9·1 to 20·8)	58·8 (46·8 to 81·3)
Bosnia and Herzegovina*	2050	950·7 (871·8 to 1038·0)	730·1 (653·0 to 808·9)	191·4 (169·5 to 215·4)	5·7 (3·6 to 8·4)	23·5 (18·5 to 32·4)	2457·0 (2253·0 to 2682·4)	1886·8 (1687·6 to 2090·5)	494·6 (438·1 to 556·7)	14·8 (9·4 to 21·7)	60·8 (47·9 to 83·8)
Belarus*	1995	118·0 (103·8 to 133·8)	89·4 (76·7 to 104·2)	20·7 (16·4 to 25·7)	7·9 (5·4 to 11·1)	0·0 (0·0 to 0·0)	380·2 (334·4 to 431·1)	288·2 (247·1 to 335·9)	66·7 (53·0 to 82·7)	25·3 (17·3 to 35·9)	0·0 (0·0 to 0·0)
Belarus*	1996	119·2 (106·4 to 133·5)	92·3 (80·6 to 105·9)	18·8 (15·1 to 23·3)	8·1 (5·6 to 11·4)	0·0 (0·0 to 0·0)	384·0 (342·9 to 430·3)	297·3 (259·9 to 341·2)	60·6 (48·7 to 75·2)	26·0 (17·9 to 36·7)	0·0 (0·0 to 0·0)
Belarus*	1997	126·4 (113·4 to 140·4)	99·9 (88·1 to 113·6)	18·0 (14·6 to 22·2)	8·5 (5·8 to 11·7)	0·0 (0·0 to 0·0)	407·4 (365·4 to 452·5)	321·9 (284·1 to 366·1)	58·2 (46·9 to 71·5)	27·3 (18·6 to 37·9)	0·0 (0·0 to 0·0)
Belarus*	1998	132·6 (120·1 to 147·0)	103·9 (91·8 to 117·0)	20·0 (16·2 to 24·6)	8·7 (6·1 to 12·1)	0·0 (0·0 to 0·0)	427·5 (387·1 to 473·6)	334·7 (295·9 to 377·0)	64·5 (52·2 to 79·3)	28·2 (19·5 to 39·0)	0·1 (0·1 to 0·1)
Belarus*	1999	136·4 (124·0 to 150·1)	105·8 (94·2 to 118·4)	22·0 (17·8 to 26·9)	8·6 (5·9 to 11·9)	0·0 (0·0 to 0·0)	439·7 (399·5 to 483·6)	341·1 (303·5 to 381·5)	70·9 (57·4 to 86·6)	27·6 (19·1 to 38·5)	0·1 (0·1 to 0·1)
Belarus*	2000	141·7 (129·5 to 156·3)	108·7 (97·2 to 121·0)	24·9 (20·2 to 29·9)	8·1 (5·6 to 11·3)	0·0 (0·0 to 0·0)	456·7 (417·2 to 503·7)	350·2 (313·2 to 390·1)	80·1 (65·2 to 96·5)	26·3 (18·0 to 36·3)	0·1 (0·1 to 0·1)

Belarus*	2001	160·5 (147·0 to 175·6)	122·3 (109·5 to 135·6)	30·2 (25·0 to 36·1)	7·9 (5·5 to 11·2)	0·1 (0·1 to 0·1)	517·4 (473·7 to 566·0)	394·2 (352·9 to 437·1)	97·3 (80·7 to 116·2)	25·5 (17·6 to 36·1)	0·4 (0·4 to 0·4)
Belarus*	2002	171·8 (158·0 to 186·2)	129·8 (116·9 to 143·1)	34·6 (29·2 to 40·9)	7·3 (5·1 to 10·4)	0·1 (0·1 to 0·1)	553·6 (509·1 to 600·1)	418·4 (376·8 to 461·0)	111·4 (94·1 to 131·8)	23·7 (16·5 to 33·5)	0·2 (0·2 to 0·2)
Belarus*	2003	185·1 (170·8 to 200·6)	139·3 (127·1 to 153·5)	38·2 (32·6 to 44·4)	7·5 (5·2 to 10·4)	0·1 (0·1 to 0·1)	596·4 (550·3 to 646·5)	449·0 (409·6 to 494·7)	123·1 (105·0 to 143·2)	24·0 (16·6 to 33·6)	0·3 (0·3 to 0·3)
Belarus*	2004	205·5 (190·4 to 222·6)	153·5 (140·4 to 168·9)	43·4 (37·2 to 50·7)	8·3 (5·8 to 11·8)	0·2 (0·2 to 0·2)	662·2 (613·7 to 717·3)	494·7 (452·4 to 544·4)	140·0 (119·8 to 163·4)	26·8 (18·6 to 38·1)	0·7 (0·7 to 0·7)
Belarus*	2005	226·9 (210·5 to 244·8)	165·8 (152·4 to 180·9)	51·1 (43·8 to 59·3)	9·0 (6·3 to 12·6)	1·0 (1·0 to 1·0)	731·3 (678·3 to 788·7)	534·3 (491·1 to 583·1)	164·8 (141·2 to 191·3)	29·1 (20·3 to 40·7)	3·1 (3·1 to 3·1)
Belarus*	2006	246·1 (229·1 to 264·0)	176·0 (162·5 to 190·6)	59·7 (51·4 to 68·8)	9·8 (6·9 to 13·6)	0·6 (0·6 to 0·6)	793·0 (738·3 to 850·8)	567·3 (523·7 to 614·2)	192·2 (165·7 to 221·8)	31·5 (22·1 to 44·0)	2·0 (2·0 to 2·0)
Belarus*	2007	264·5 (247·9 to 283·4)	184·1 (170·3 to 199·1)	69·1 (59·9 to 79·7)	10·4 (7·4 to 14·5)	1·0 (1·0 to 1·0)	852·3 (799·0 to 913·2)	593·1 (548·9 to 641·5)	222·5 (193·0 to 256·9)	33·6 (23·9 to 46·6)	3·1 (3·1 to 3·1)
Belarus*	2008	308·2 (288·8 to 330·2)	212·7 (196·4 to 229·9)	82·4 (72·2 to 94·4)	11·4 (8·2 to 15·8)	1·7 (1·7 to 1·7)	993·0 (930·8 to 1064·1)	685·3 (633·0 to 740·8)	265·5 (232·6 to 304·2)	36·9 (26·5 to 50·9)	5·4 (5·4 to 5·4)
Belarus*	2009	289·1 (270·7 to 307·7)	191·5 (175·9 to 207·0)	85·5 (75·9 to 96·4)	10·4 (7·4 to 14·5)	1·7 (1·7 to 1·7)	931·5 (872·5 to 991·6)	617·0 (566·8 to 667·1)	275·5 (244·5 to 310·8)	33·6 (23·8 to 46·9)	5·5 (5·5 to 5·5)
Belarus*	2010	294·8 (277·0 to 313·5)	192·3 (176·5 to 208·0)	91·5 (82·0 to 101·8)	9·0 (6·3 to 12·6)	2·1 (2·1 to 2·1)	950·2 (892·5 to 1010·2)	619·7 (568·9 to 670·3)	294·8 (264·3 to 328·2)	28·9 (20·4 to 40·6)	6·7 (6·7 to 6·7)
Belarus*	2011	302·7 (284·0 to 320·6)	194·2 (178·6 to 209·3)	98·1 (89·0 to 108·6)	8·8 (6·2 to 12·5)	1·6 (1·6 to 1·6)	975·6 (915·1 to 1033·0)	625·8 (575·6 to 674·5)	316·1 (286·9 to 349·9)	28·5 (20·0 to 40·2)	5·2 (5·2 to 5·2)
Belarus*	2012	317·0 (298·4 to 334·0)	201·1 (185·8 to 215·8)	104·9 (95·8 to 114·0)	9·4 (6·6 to 13·1)	1·6 (1·6 to 1·6)	1021·6 (961·6 to 1076·5)	648·2 (598·7 to 695·4)	337·9 (308·7 to 367·5)	30·2 (21·2 to 42·3)	5·2 (5·2 to 5·2)
Belarus*	2013	338·9 (319·2 to 357·5)	213·8 (198·5 to 228·6)	113·9 (104·0 to 123·2)	9·7 (6·8 to 13·4)	1·5 (1·5 to 1·5)	1092·2 (1028·8 to 1151·9)	688·9 (639·8 to 736·7)	367·0 (335·3 to 397·0)	31·3 (21·9 to 43·3)	5·0 (5·0 to 5·0)
Belarus*	2014	340·9 (323·6 to 359·1)	214·8 (199·4 to 229·7)	116·0 (106·8 to 125·7)	8·7 (6·1 to 12·0)	1·3 (1·3 to 1·3)	1098·5 (1042·7 to 1157·3)	692·3 (642·6 to 740·1)	373·8 (344·3 to 405·2)	28·0 (19·5 to 38·7)	4·3 (4·3 to 4·3)

Belarus*	2015	355·3 (337·4 to 374·3)	229·4 (213·9 to 245·0)	116·1 (106·8 to 125·8)	9·1 (6·4 to 12·5)	0·7 (0·7 to 0·7)	1145·0 (1087·2 to 1206·3)	739·1 (689·2 to 789·6)	374·2 (344·2 to 405·4)	29·3 (20·5 to 40·3)	2·3 (2·3 to 2·3)
Belarus*	2016	347·5 (329·1 to 366·4)	233·4 (217·4 to 249·5)	104·2 (95·0 to 113·1)	8·8 (6·1 to 12·2)	1·1 (1·1 to 1·1)	1119·9 (1060·4 to 1180·8)	752·3 (700·6 to 804·0)	336·0 (306·3 to 364·6)	28·2 (19·7 to 39·2)	3·5 (3·5 to 3·5)
Belarus*	2017	348·0 (327·1 to 369·2)	238·5 (221·2 to 257·0)	97·4 (87·6 to 107·6)	10·9 (7·6 to 15·4)	1·2 (1·2 to 1·2)	1121·6 (1054·1 to 1189·8)	768·5 (712·9 to 828·2)	314·0 (282·4 to 346·8)	35·3 (24·4 to 49·6)	3·9 (3·9 to 3·9)
Belarus*	2018	352·0 (325·6 to 377·6)	244·9 (222·5 to 267·3)	92·9 (81·6 to 104·6)	13·1 (9·0 to 18·4)	1·2 (1·2 to 1·2)	1134·3 (1049·4 to 1216·7)	789·1 (717·1 to 861·4)	299·3 (263·0 to 337·1)	42·2 (29·1 to 59·4)	3·8 (3·8 to 3·8)
Belarus*	2019	360·7 (333·6 to 387·4)	252·8 (230·3 to 276·2)	93·2 (81·9 to 105·0)	13·6 (9·4 to 19·2)	1·2 (1·2 to 1·2)	1162·5 (1074·9 to 1248·4)	814·6 (742·1 to 890·2)	300·2 (263·8 to 338·3)	43·9 (30·4 to 61·9)	3·9 (3·8 to 3·9)
Belarus*	2020	358·9 (331·4 to 385·6)	255·4 (232·6 to 279·5)	88·6 (78·0 to 100·0)	13·3 (9·2 to 18·7)	1·6 (1·6 to 1·7)	1156·6 (1068·0 to 1242·7)	823·0 (749·6 to 900·9)	285·7 (251·4 to 322·1)	42·8 (29·6 to 60·2)	5·2 (5·1 to 5·5)
Belarus*	2021	343·8 (317·4 to 369·5)	244·5 (222·7 to 267·6)	84·7 (74·6 to 95·6)	13·0 (9·0 to 18·2)	1·7 (1·6 to 1·9)	1108·0 (1023·0 to 1190·6)	787·8 (717·6 to 862·4)	273·0 (240·5 to 308·0)	41·8 (29·0 to 58·8)	5·3 (5·1 to 6·0)
Belarus*	2022	337·4 (311·5 to 363·6)	237·9 (215·9 to 261·4)	84·8 (74·6 to 95·6)	13·3 (9·2 to 18·8)	1·3 (1·2 to 1·5)	1087·2 (1003·8 to 1171·7)	766·8 (695·8 to 842·4)	273·1 (240·3 to 308·1)	43·0 (29·6 to 60·7)	4·3 (4·0 to 4·9)
Belarus*	2023	341·2 (315·0 to 367·7)	241·4 (218·4 to 265·3)	84·7 (74·6 to 95·6)	13·7 (9·5 to 19·5)	1·4 (1·3 to 1·6)	1099·7 (1015·0 to 1184·9)	777·8 (703·8 to 854·8)	273·1 (240·3 to 308·1)	44·2 (30·5 to 62·9)	4·6 (4·2 to 5·3)
Belarus*	2024	345·5 (318·4 to 372·2)	245·1 (221·5 to 270·1)	84·7 (74·5 to 95·5)	14·1 (9·8 to 20·1)	1·5 (1·4 to 1·7)	1113·3 (1026·0 to 1199·5)	789·9 (713·8 to 870·4)	272·9 (240·1 to 307·9)	45·5 (31·5 to 64·7)	4·9 (4·5 to 5·6)
Belarus*	2025	350·1 (322·2 to 377·8)	249·3 (225·2 to 274·6)	84·6 (74·5 to 95·4)	14·6 (10·1 to 20·6)	1·6 (1·5 to 1·9)	1128·3 (1038·3 to 1217·5)	803·5 (725·7 to 885·0)	272·7 (240·1 to 307·6)	46·9 (32·5 to 66·5)	5·2 (4·8 to 6·0)
Belarus*	2026	354·5 (325·9 to 383·1)	253·2 (229·0 to 278·7)	84·7 (74·6 to 95·5)	15·0 (10·4 to 21·3)	1·7 (1·6 to 2·0)	1142·6 (1050·3 to 1234·7)	815·9 (738·1 to 898·3)	272·8 (240·3 to 307·8)	48·3 (33·5 to 68·8)	5·5 (5·0 to 6·3)
Belarus*	2027	356·8 (328·0 to 386·4)	254·6 (230·3 to 281·2)	84·9 (74·8 to 95·8)	15·5 (10·7 to 22·1)	1·8 (1·6 to 2·1)	1149·8 (1057·2 to 1245·3)	820·5 (742·0 to 906·2)	273·7 (241·0 to 308·8)	49·8 (34·5 to 71·2)	5·8 (5·2 to 6·7)
Belarus*	2028	361·1 (330·1 to 392·6)	257·9 (231·0 to 286·5)	85·4 (75·2 to 96·4)	16·0 (11·1 to 22·7)	1·9 (1·7 to 2·2)	1163·7 (1063·9 to 1265·2)	831·0 (744·4 to 923·2)	275·2 (242·4 to 310·5)	51·4 (35·7 to 73·2)	6·1 (5·4 to 7·0)

Belarus*	2029	366·7 (334·3 to 401·6)	262·3 (234·3 to 293·7)	86·0 (75·7 to 97·0)	16·5 (11·4 to 23·4)	2·0 (1·7 to 2·3)	1181·7 (1077·2 to 1294·1)	845·2 (755·1 to 946·4)	277·0 (243·8 to 312·5)	53·1 (36·6 to 75·6)	6·3 (5·6 to 7·3)
Belarus*	2030	372·1 (335·7 to 409·8)	266·3 (233·1 to 301·4)	86·7 (76·3 to 97·8)	17·0 (11·7 to 24·2)	2·0 (1·8 to 2·3)	1199·1 (1081·7 to 1320·5)	858·3 (751·1 to 971·4)	279·4 (245·7 to 315·2)	54·9 (37·8 to 78·1)	6·5 (5·7 to 7·5)
Belarus*	2031	376·8 (337·0 to 418·4)	269·7 (232·4 to 308·3)	87·5 (77·0 to 98·8)	17·6 (12·2 to 25·1)	2·1 (1·8 to 2·4)	1214·4 (1086·0 to 1348·4)	869·0 (749·1 to 993·5)	282·0 (248·1 to 318·3)	56·8 (39·2 to 81·0)	6·6 (5·8 to 7·8)
Belarus*	2032	381·6 (339·5 to 427·1)	273·1 (233·7 to 316·2)	88·2 (77·7 to 99·7)	18·2 (12·5 to 25·9)	2·1 (1·8 to 2·5)	1229·8 (1094·2 to 1376·2)	880·0 (753·0 to 1019·0)	284·4 (250·3 to 321·2)	58·6 (40·4 to 83·6)	6·8 (5·9 to 8·1)
Belarus*	2033	387·6 (341·8 to 435·8)	277·5 (235·5 to 323·8)	89·1 (78·5 to 100·8)	18·8 (12·9 to 26·8)	2·2 (1·9 to 2·6)	1249·1 (1101·4 to 1404·5)	894·2 (759·1 to 1043·4)	287·3 (252·9 to 324·7)	60·6 (41·7 to 86·4)	7·0 (6·1 to 8·4)
Belarus*	2034	393·4 (343·6 to 447·4)	281·6 (234·0 to 334·6)	90·2 (79·4 to 102·0)	19·5 (13·4 to 27·8)	2·2 (1·9 to 2·7)	1267·9 (1107·4 to 1441·9)	907·4 (754·1 to 1078·2)	290·6 (255·9 to 328·6)	62·7 (43·1 to 89·4)	7·2 (6·2 to 8·6)
Belarus*	2035	399·5 (345·4 to 457·9)	285·9 (233·0 to 342·2)	91·2 (80·3 to 103·2)	20·1 (13·8 to 28·7)	2·3 (2·0 to 2·8)	1287·5 (1113·1 to 1475·6)	921·3 (751·0 to 1102·7)	294·0 (258·9 to 332·5)	64·9 (44·5 to 92·5)	7·3 (6·3 to 8·9)
Belarus*	2036	405·4 (346·3 to 467·6)	290·1 (234·0 to 351·7)	92·2 (81·1 to 104·3)	20·8 (14·3 to 29·6)	2·3 (2·0 to 2·9)	1306·5 (1116·0 to 1506·8)	934·9 (754·2 to 1133·3)	297·1 (261·5 to 336·1)	67·0 (46·0 to 95·5)	7·5 (6·4 to 9·2)
Belarus*	2037	411·2 (348·8 to 480·4)	294·3 (233·8 to 363·5)	93·1 (81·9 to 105·4)	21·5 (14·7 to 30·6)	2·4 (2·0 to 3·0)	1325·2 (1123·9 to 1548·2)	948·4 (753·4 to 1171·5)	300·0 (264·1 to 339·7)	69·2 (47·4 to 98·7)	7·7 (6·6 to 9·6)
Belarus*	2038	417·2 (350·7 to 488·7)	298·6 (234·5 to 371·0)	93·9 (82·7 to 106·4)	22·2 (15·1 to 31·5)	2·5 (2·1 to 3·1)	1344·3 (1130·2 to 1575·0)	962·3 (755·5 to 1195·7)	302·7 (266·5 to 343·0)	71·4 (48·8 to 101·7)	8·0 (6·7 to 10·0)
Belarus*	2039	422·1 (354·6 to 496·2)	302·0 (237·5 to 376·8)	94·7 (83·3 to 107·4)	22·8 (15·5 to 32·6)	2·6 (2·1 to 3·3)	1360·1 (1142·6 to 1599·0)	973·3 (765·2 to 1214·3)	305·0 (268·4 to 346·0)	73·6 (49·9 to 105·1)	8·2 (6·9 to 10·5)
Belarus*	2040	426·6 (354·9 to 503·7)	304·9 (238·2 to 380·5)	95·4 (84·0 to 108·3)	23·5 (15·9 to 33·8)	2·7 (2·2 to 3·4)	1374·7 (1143·8 to 1623·2)	982·7 (767·6 to 1226·1)	307·6 (270·6 to 349·1)	75·9 (51·1 to 108·8)	8·5 (7·1 to 10·9)
Belarus*	2041	431·4 (359·7 to 514·8)	308·4 (238·7 to 392·6)	96·1 (84·5 to 109·1)	24·2 (16·3 to 34·9)	2·8 (2·3 to 3·6)	1390·4 (1159·2 to 1658·9)	993·7 (769·2 to 1265·1)	309·6 (272·3 to 351·6)	78·1 (52·4 to 112·4)	8·9 (7·4 to 11·5)
Belarus*	2042	435·8 (360·0 to 521·7)	311·3 (237·4 to 396·0)	96·7 (85·0 to 109·9)	25·0 (16·7 to 35·8)	2·9 (2·4 to 3·7)	1404·5 (1160·2 to 1681·3)	1003·1 (765·1 to 1276·1)	311·7 (274·1 to 354·1)	80·5 (53·8 to 115·3)	9·3 (7·6 to 12·1)

Belarus*	2043	439·7 (362·0 to 526·9)	313·8 (240·7 to 401·1)	97·2 (85·5 to 110·5)	25·7 (17·1 to 36·9)	3·0 (2·5 to 3·9)	1417·0 (1166·5 to 1698·0)	1011·2 (775·5 to 1292·7)	313·4 (275·5 to 356·2)	82·8 (55·1 to 118·9)	9·7 (7·9 to 12·7)
Belarus*	2044	443·5 (361·2 to 540·4)	316·3 (236·7 to 412·5)	97·7 (85·8 to 111·1)	26·4 (17·5 to 38·0)	3·2 (2·6 to 4·2)	1429·3 (1163·9 to 1741·5)	1019·2 (762·8 to 1329·2)	314·8 (276·6 to 358·1)	85·1 (56·5 to 122·5)	10·2 (8·3 to 13·6)
Belarus*	2045	447·3 (361·1 to 548·2)	318·6 (235·8 to 419·8)	98·2 (86·3 to 111·8)	27·2 (17·9 to 39·3)	3·3 (2·7 to 4·5)	1441·5 (1163·5 to 1766·6)	1026·8 (760·0 to 1352·7)	316·4 (278·0 to 360·1)	87·6 (57·7 to 126·5)	10·7 (8·7 to 14·5)
Belarus*	2046	450·3 (363·4 to 556·1)	320·3 (236·5 to 422·2)	98·5 (86·5 to 112·1)	27·9 (18·3 to 40·4)	3·5 (2·8 to 4·8)	1451·1 (1171·2 to 1792·2)	1032·3 (762·3 to 1360·6)	317·5 (278·9 to 361·4)	90·0 (59·0 to 130·3)	11·3 (9·2 to 15·4)
Belarus*	2047	453·3 (364·6 to 559·1)	322·1 (235·2 to 427·3)	98·8 (86·8 to 112·4)	28·7 (18·7 to 41·8)	3·7 (3·0 to 5·2)	1460·9 (1175·0 to 1801·8)	1038·1 (758·1 to 1376·9)	318·3 (279·7 to 362·3)	92·5 (60·3 to 134·6)	12·0 (9·6 to 16·8)
Belarus*	2048	455·9 (364·0 to 568·0)	323·6 (235·7 to 433·2)	99·0 (87·0 to 112·7)	29·5 (19·1 to 43·0)	3·9 (3·1 to 5·6)	1469·3 (1173·1 to 1830·5)	1042·7 (759·5 to 1396·1)	318·9 (280·2 to 363·2)	95·0 (61·5 to 138·5)	12·7 (10·1 to 18·0)
Belarus*	2049	457·7 (363·3 to 570·0)	324·2 (233·3 to 436·9)	99·0 (87·0 to 112·8)	30·3 (19·4 to 44·4)	4·2 (3·3 to 6·0)	1474·9 (1170·7 to 1837·0)	1044·9 (751·9 to 1407·9)	318·9 (280·3 to 363·4)	97·6 (62·6 to 143·2)	13·6 (10·7 to 19·4)
Belarus*	2050	459·2 (364·4 to 576·4)	324·7 (232·7 to 438·6)	98·9 (86·9 to 112·7)	31·1 (19·8 to 45·8)	4·5 (3·5 to 6·5)	1479·8 (1174·3 to 1857·6)	1046·4 (749·8 to 1413·6)	318·7 (280·2 to 363·3)	100·1 (63·8 to 147·7)	14·6 (11·4 to 21·0)
Belize*	1995	165·4 (148·5 to 184·4)	93·2 (80·7 to 108·0)	50·3 (42·0 to 60·7)	19·5 (14·2 to 26·5)	2·3 (2·3 to 2·3)	252·3 (226·6 to 281·3)	142·3 (123·1 to 164·8)	76·7 (64·0 to 92·7)	29·8 (21·6 to 40·4)	3·5 (3·5 to 3·5)
Belize*	1996	159·3 (144·5 to 175·2)	87·1 (75·7 to 100·1)	50·5 (42·4 to 60·7)	19·2 (13·9 to 26·0)	2·5 (2·5 to 2·5)	243·1 (220·5 to 267·3)	132·9 (115·6 to 152·7)	77·1 (64·7 to 92·6)	29·3 (21·3 to 39·6)	3·8 (3·8 to 3·8)
Belize*	1997	160·3 (145·7 to 175·9)	85·9 (75·0 to 97·8)	52·0 (44·0 to 62·0)	19·1 (14·0 to 25·4)	3·4 (3·4 to 3·4)	244·6 (222·3 to 268·3)	131·0 (114·4 to 149·2)	79·3 (67·1 to 94·5)	29·1 (21·4 to 38·7)	5·2 (5·2 to 5·2)
Belize*	1998	160·4 (146·5 to 175·6)	85·6 (75·0 to 96·6)	53·7 (45·7 to 63·3)	18·7 (13·8 to 25·6)	2·4 (2·4 to 2·4)	244·8 (223·5 to 267·9)	130·6 (114·4 to 147·3)	81·9 (69·8 to 96·5)	28·5 (21·0 to 39·1)	3·7 (3·7 to 3·7)
Belize*	1999	172·2 (158·0 to 187·5)	89·8 (79·1 to 100·9)	57·8 (50·2 to 67·0)	18·5 (13·7 to 25·1)	6·1 (6·1 to 6·1)	262·7 (241·1 to 286·0)	137·0 (120·7 to 153·9)	88·2 (76·5 to 102·2)	28·2 (20·9 to 38·4)	9·3 (9·3 to 9·3)
Belize*	2000	190·7 (175·7 to 206·8)	95·3 (83·7 to 107·5)	64·3 (55·6 to 73·8)	17·9 (13·1 to 24·0)	13·3 (13·3 to 13·3)	290·9 (268·0 to 315·5)	145·3 (127·8 to 164·0)	98·0 (84·8 to 112·6)	27·3 (20·0 to 36·7)	20·2 (20·2 to 20·2)

Belize*	2001	211·0 (194·5 to 229·0)	109·7 (97·1 to 123·0)	71·1 (61·8 to 82·1)	18·9 (13·7 to 25·1)	11·3 (11·3 to 11·3)	321·8 (296·7 to 349·4)	167·3 (148·2 to 187·7)	108·4 (94·3 to 125·2)	28·8 (20·9 to 38·3)	17·3 (17·3 to 17·3)
Belize*	2002	213·2 (197·5 to 231·8)	112·6 (100·1 to 125·5)	73·2 (64·0 to 84·3)	18·4 (13·6 to 24·6)	9·0 (9·0 to 9·0)	325·2 (301·3 to 353·7)	171·7 (152·7 to 191·5)	111·6 (97·7 to 128·6)	28·1 (20·7 to 37·5)	13·7 (13·7 to 13·7)
Belize*	2003	224·4 (207·6 to 243·0)	121·7 (109·3 to 134·8)	75·5 (66·0 to 86·9)	18·8 (13·8 to 25·2)	8·4 (8·4 to 8·4)	342·4 (316·6 to 370·7)	185·7 (166·7 to 205·6)	115·2 (100·7 to 132·6)	28·7 (21·1 to 38·4)	12·8 (12·8 to 12·8)
Belize*	2004	225·9 (208·9 to 243·7)	124·1 (111·8 to 136·8)	74·2 (64·5 to 84·7)	18·8 (13·7 to 25·0)	8·8 (8·8 to 8·8)	344·6 (318·7 to 371·8)	189·3 (170·5 to 208·7)	113·2 (98·4 to 129·1)	28·7 (20·9 to 38·2)	13·4 (13·4 to 13·4)
Belize*	2005	226·1 (209·3 to 242·8)	128·4 (116·5 to 141·1)	71·5 (62·1 to 82·3)	18·1 (13·1 to 23·9)	8·0 (8·0 to 8·0)	344·9 (319·3 to 370·4)	195·9 (177·7 to 215·2)	109·1 (94·8 to 125·6)	27·6 (20·0 to 36·4)	12·3 (12·3 to 12·3)
Belize*	2006	234·5 (218·9 to 251·3)	138·8 (126·4 to 151·1)	69·3 (60·0 to 79·2)	17·2 (12·3 to 22·7)	9·3 (9·3 to 9·3)	357·8 (333·9 to 383·4)	211·7 (192·8 to 230·6)	105·7 (91·5 to 120·8)	26·2 (18·7 to 34·6)	14·2 (14·2 to 14·2)
Belize*	2007	265·9 (249·2 to 283·4)	170·2 (156·9 to 184·6)	70·8 (61·6 to 80·5)	17·4 (12·6 to 23·3)	7·5 (7·5 to 7·5)	405·7 (380·1 to 432·4)	259·7 (239·4 to 281·6)	107·9 (93·9 to 122·8)	26·6 (19·2 to 35·5)	11·5 (11·5 to 11·5)
Belize*	2008	266·7 (251·0 to 282·7)	174·7 (162·4 to 186·7)	68·2 (60·5 to 77·2)	17·0 (12·2 to 22·9)	6·8 (6·8 to 6·8)	406·9 (382·9 to 431·2)	266·5 (247·8 to 284·8)	104·1 (92·3 to 117·8)	26·0 (18·6 to 34·9)	10·4 (10·4 to 10·4)
Belize*	2009	275·3 (259·9 to 291·5)	183·5 (170·6 to 196·9)	68·3 (61·5 to 76·3)	17·0 (12·3 to 22·8)	6·4 (6·4 to 6·4)	419·9 (396·4 to 444·7)	280·0 (260·2 to 300·3)	104·2 (93·9 to 116·4)	25·9 (18·7 to 34·8)	9·8 (9·8 to 9·8)
Belize*	2010	280·5 (264·7 to 298·1)	185·5 (171·4 to 200·3)	67·8 (61·9 to 74·9)	16·7 (12·0 to 22·7)	10·5 (10·5 to 10·5)	427·8 (403·8 to 454·8)	283·0 (261·4 to 305·6)	103·4 (94·4 to 114·2)	25·4 (18·3 to 34·6)	16·0 (16·0 to 16·0)
Belize*	2011	272·0 (255·3 to 289·4)	182·8 (169·5 to 197·3)	67·4 (60·4 to 74·8)	16·5 (11·9 to 22·4)	5·3 (5·3 to 5·3)	414·9 (389·5 to 441·5)	278·9 (258·5 to 301·1)	102·8 (92·2 to 114·2)	25·2 (18·1 to 34·1)	8·1 (8·1 to 8·1)
Belize*	2012	269·5 (253·3 to 287·5)	177·9 (164·4 to 192·0)	66·8 (58·9 to 75·2)	16·2 (11·5 to 21·9)	8·7 (8·7 to 8·7)	411·2 (386·4 to 438·6)	271·4 (250·7 to 292·9)	101·9 (89·9 to 114·7)	24·6 (17·5 to 33·3)	13·2 (13·2 to 13·2)
Belize*	2013	297·0 (280·2 to 315·9)	179·2 (165·6 to 193·1)	66·6 (57·9 to 75·8)	16·3 (11·8 to 22·4)	34·9 (34·9 to 34·9)	453·1 (427·5 to 481·9)	273·3 (252·6 to 294·6)	101·7 (88·4 to 115·7)	24·8 (17·9 to 34·1)	53·2 (53·2 to 53·2)
Belize*	2014	276·5 (258·8 to 295·7)	186·1 (171·7 to 201·8)	67·0 (58·1 to 76·6)	16·3 (12·0 to 22·3)	7·2 (7·2 to 7·2)	421·8 (394·8 to 451·1)	283·9 (261·9 to 307·8)	102·2 (88·6 to 116·8)	24·9 (18·3 to 34·0)	10·9 (10·9 to 10·9)

Belize*	2015	296·3 (277·5 to 316·3)	197·5 (182·2 to 214·0)	68·1 (58·3 to 77·9)	16·9 (12·3 to 23·2)	13·8 (13·8 to 13·8)	452·0 (423·3 to 482·6)	301·3 (277·9 to 326·5)	103·9 (88·9 to 118·9)	25·8 (18·7 to 35·4)	21·0 (21·0 to 21·0)
Belize*	2016	286·7 (267·8 to 307·3)	193·5 (177·6 to 210·7)	67·2 (57·7 to 77·6)	16·7 (12·2 to 22·7)	9·2 (9·2 to 9·2)	437·4 (408·6 to 468·8)	295·2 (270·9 to 321·4)	102·6 (88·0 to 118·4)	25·5 (18·6 to 34·7)	14·1 (14·1 to 14·1)
Belize*	2017	279·9 (260·7 to 301·2)	191·4 (173·7 to 209·4)	66·2 (56·1 to 77·0)	16·1 (11·5 to 22·1)	6·2 (6·2 to 6·2)	427·0 (397·7 to 459·5)	292·0 (265·0 to 319·5)	101·0 (85·6 to 117·5)	24·5 (17·5 to 33·7)	9·5 (9·5 to 9·5)
Belize*	2018	277·8 (255·1 to 300·3)	189·3 (170·3 to 209·9)	65·4 (54·6 to 76·4)	15·8 (11·3 to 21·4)	7·4 (7·4 to 7·4)	423·8 (389·1 to 458·1)	288·8 (259·8 to 320·2)	99·7 (83·3 to 116·5)	24·0 (17·3 to 32·6)	11·3 (11·3 to 11·3)
Belize*	2019	282·8 (258·3 to 309·0)	195·1 (174·2 to 219·6)	63·4 (53·0 to 74·0)	15·7 (11·3 to 21·3)	8·6 (8·5 to 9·0)	431·4 (394·1 to 471·4)	297·7 (265·7 to 335·0)	96·7 (80·9 to 112·9)	23·9 (17·2 to 32·6)	13·2 (13·0 to 13·7)
Belize*	2020	293·2 (266·9 to 321·5)	207·0 (182·7 to 234·3)	58·6 (49·0 to 68·5)	14·9 (10·7 to 20·2)	12·7 (12·3 to 13·3)	447·3 (407·2 to 490·5)	315·7 (278·7 to 357·4)	89·4 (74·7 to 104·5)	22·7 (16·3 to 30·8)	19·4 (18·8 to 20·2)
Belize*	2021	280·9 (255·7 to 308·0)	197·4 (174·2 to 223·4)	55·9 (46·7 to 65·3)	14·6 (10·4 to 19·7)	13·1 (12·5 to 13·9)	428·5 (390·1 to 469·8)	301·1 (265·7 to 340·8)	85·2 (71·3 to 99·5)	22·3 (15·9 to 30·1)	19·9 (19·1 to 21·2)
Belize*	2022	258·7 (232·2 to 287·5)	176·8 (152·2 to 204·6)	56·8 (47·5 to 66·3)	15·2 (10·9 to 20·6)	9·9 (9·2 to 10·7)	394·7 (354·2 to 438·6)	269·8 (232·2 to 312·1)	86·7 (72·5 to 101·1)	23·2 (16·6 to 31·5)	15·0 (14·0 to 16·3)
Belize*	2023	264·8 (235·8 to 294·0)	181·2 (155·1 to 209·2)	57·8 (48·3 to 67·4)	15·9 (11·4 to 21·5)	9·8 (9·1 to 10·6)	403·9 (359·7 to 448·5)	276·5 (236·6 to 319·1)	88·2 (73·7 to 102·9)	24·3 (17·4 to 32·8)	15·0 (13·9 to 16·2)
Belize*	2024	270·5 (240·6 to 302·5)	185·3 (156·5 to 216·0)	58·8 (49·1 to 68·5)	16·6 (11·9 to 22·6)	9·8 (9·1 to 10·6)	412·6 (367·0 to 461·4)	282·7 (238·7 to 329·5)	89·6 (74·9 to 104·5)	25·3 (18·2 to 34·4)	15·0 (13·9 to 16·2)
Belize*	2025	276·6 (243·9 to 309·8)	189·7 (159·0 to 222·9)	59·7 (49·9 to 69·6)	17·3 (12·5 to 23·6)	9·8 (9·1 to 10·6)	421·9 (372·0 to 472·6)	289·5 (242·6 to 340·0)	91·1 (76·1 to 106·2)	26·4 (19·0 to 35·9)	15·0 (13·9 to 16·2)
Belize*	2026	282·1 (247·2 to 319·3)	193·6 (161·0 to 228·7)	60·6 (50·7 to 70·7)	18·1 (12·9 to 24·6)	9·8 (9·0 to 10·7)	430·3 (377·1 to 487·1)	295·4 (245·6 to 348·8)	92·4 (77·3 to 107·8)	27·6 (19·7 to 37·5)	15·0 (13·7 to 16·3)
Belize*	2027	288·0 (251·0 to 328·3)	197·9 (163·7 to 235·7)	61·4 (51·4 to 71·6)	18·8 (13·5 to 25·7)	9·8 (9·0 to 10·8)	439·3 (382·9 to 500·8)	302·0 (249·7 to 359·6)	93·6 (78·3 to 109·2)	28·7 (20·6 to 39·3)	15·0 (13·7 to 16·5)
Belize*	2028	293·3 (254·7 to 336·1)	201·7 (165·5 to 242·4)	62·1 (52·0 to 72·5)	19·6 (13·9 to 27·0)	9·9 (9·0 to 11·0)	447·4 (388·6 to 512·8)	307·7 (252·5 to 369·8)	94·7 (79·3 to 110·6)	29·9 (21·3 to 41·2)	15·1 (13·8 to 16·8)

Belize*	2029	298·2 (258·9 to 342·0)	205·1 (167·8 to 247·4)	62·7 (52·5 to 73·3)	20·3 (14·5 to 28·2)	10·0 (9·1 to 11·2)	454·9 (395·0 to 521·8)	312·8 (256·0 to 377·4)	95·7 (80·2 to 111·8)	31·0 (22·0 to 43·0)	15·3 (13·9 to 17·1)
Belize*	2030	303·3 (262·0 to 350·5)	208·7 (169·1 to 254·1)	63·3 (53·1 to 73·9)	21·1 (15·0 to 29·0)	10·2 (9·2 to 11·4)	462·7 (399·8 to 534·8)	318·4 (258·0 to 387·6)	96·6 (81·0 to 112·7)	32·2 (22·8 to 44·3)	15·5 (14·0 to 17·3)
Belize*	2031	309·4 (266·2 to 359·0)	213·4 (173·3 to 261·3)	63·8 (53·5 to 74·6)	21·8 (15·5 to 30·0)	10·3 (9·3 to 11·6)	472·0 (406·2 to 547·7)	325·6 (264·4 to 398·7)	97·4 (81·6 to 113·7)	33·3 (23·6 to 45·8)	15·8 (14·2 to 17·8)
Belize*	2032	316·2 (270·6 to 368·3)	218·8 (175·9 to 269·8)	64·4 (53·9 to 75·2)	22·5 (16·0 to 31·0)	10·5 (9·4 to 11·9)	482·4 (412·8 to 561·8)	333·9 (268·4 to 411·6)	98·2 (82·3 to 114·8)	34·4 (24·4 to 47·2)	16·0 (14·4 to 18·2)
Belize*	2033	322·4 (274·9 to 377·6)	223·6 (179·4 to 277·6)	64·8 (54·3 to 75·8)	23·2 (16·4 to 31·9)	10·7 (9·6 to 12·3)	491·8 (419·4 to 576·1)	341·1 (273·7 to 423·4)	98·9 (82·8 to 115·6)	35·4 (25·0 to 48·7)	16·4 (14·6 to 18·7)
Belize*	2034	328·8 (278·7 to 386·9)	228·6 (180·7 to 285·7)	65·3 (54·7 to 76·3)	24·0 (16·8 to 33·0)	11·0 (9·8 to 12·6)	501·6 (425·2 to 590·2)	348·7 (275·7 to 435·8)	99·6 (83·4 to 116·4)	36·6 (25·6 to 50·4)	16·8 (14·9 to 19·2)
Belize*	2035	335·1 (282·6 to 394·1)	233·4 (185·1 to 292·2)	65·7 (55·0 to 76·8)	24·7 (17·2 to 34·1)	11·3 (10·0 to 12·9)	511·2 (431·2 to 601·2)	356·0 (282·5 to 445·7)	100·3 (83·9 to 117·1)	37·7 (26·3 to 51·9)	17·2 (15·2 to 19·7)
Belize*	2036	342·0 (285·8 to 403·8)	238·7 (186·5 to 297·3)	66·2 (55·4 to 77·3)	25·5 (17·8 to 35·3)	11·6 (10·2 to 13·3)	521·7 (436·0 to 616·0)	364·2 (284·5 to 453·6)	101·0 (84·5 to 118·0)	38·9 (27·1 to 53·8)	17·6 (15·5 to 20·4)
Belize*	2037	349·1 (289·7 to 414·1)	244·2 (188·5 to 308·7)	66·7 (55·8 to 77·9)	26·3 (18·2 to 36·3)	11·8 (10·4 to 13·8)	532·5 (441·9 to 631·7)	372·6 (287·6 to 471·0)	101·8 (85·1 to 118·8)	40·1 (27·7 to 55·4)	18·0 (15·9 to 21·0)
Belize*	2038	356·2 (292·6 to 423·8)	249·7 (189·3 to 315·9)	67·3 (56·2 to 78·4)	27·2 (18·6 to 37·6)	12·1 (10·6 to 14·2)	543·5 (446·3 to 646·5)	381·0 (288·8 to 482·0)	102·6 (85·8 to 119·6)	41·4 (28·4 to 57·3)	18·4 (16·1 to 21·7)
Belize*	2039	363·4 (297·3 to 433·5)	255·2 (193·0 to 324·3)	67·7 (56·6 to 79·0)	28·0 (19·2 to 38·9)	12·4 (10·8 to 14·6)	554·4 (453·5 to 661·3)	389·4 (294·5 to 494·7)	103·3 (86·3 to 120·6)	42·7 (29·2 to 59·3)	19·0 (16·5 to 22·3)
Belize*	2040	370·9 (300·3 to 443·7)	261·1 (193·0 to 332·4)	68·1 (56·9 to 79·6)	28·9 (19·6 to 40·0)	12·9 (11·1 to 15·3)	565·9 (458·1 to 676·9)	398·3 (294·4 to 507·1)	104·0 (86·8 to 121·5)	44·0 (29·9 to 61·0)	19·6 (16·9 to 23·4)
Belize*	2041	378·4 (303·8 to 454·6)	266·7 (195·2 to 340·7)	68·6 (57·2 to 80·2)	29·8 (20·1 to 41·2)	13·3 (11·4 to 15·9)	577·2 (463·4 to 693·5)	406·9 (297·8 to 519·8)	104·6 (87·3 to 122·3)	45·4 (30·6 to 62·9)	20·2 (17·4 to 24·3)
Belize*	2042	386·1 (308·2 to 465·5)	272·6 (200·1 to 352·2)	69·1 (57·7 to 80·7)	30·7 (20·7 to 42·9)	13·7 (11·7 to 16·5)	588·9 (470·2 to 710·2)	415·8 (305·3 to 537·3)	105·4 (88·0 to 123·1)	46·8 (31·7 to 65·4)	20·9 (17·8 to 25·2)

Belize*	2043	393·7 (314·0 to 476·4)	278·3 (202·7 to 360·0)	69·6 (58·1 to 81·3)	31·7 (21·3 to 44·6)	14·1 (12·0 to 17·3)	600·6 (479·1 to 726·7)	424·6 (309·2 to 549·3)	106·1 (88·6 to 124·0)	48·3 (32·5 to 68·0)	21·6 (18·3 to 26·4)
Belize*	2044	401·4 (319·0 to 486·6)	284·0 (204·9 to 368·8)	70·0 (58·4 to 81·8)	32·7 (21·8 to 46·0)	14·6 (12·4 to 18·0)	612·3 (486·6 to 742·4)	433·3 (312·7 to 562·7)	106·9 (89·1 to 124·8)	49·8 (33·3 to 70·1)	22·3 (18·8 to 27·4)
Belize*	2045	409·2 (324·2 to 494·5)	289·9 (207·1 to 375·9)	70·5 (58·8 to 82·5)	33·7 (22·4 to 47·9)	15·1 (12·6 to 18·8)	624·3 (494·6 to 754·4)	442·2 (315·9 to 573·4)	107·6 (89·7 to 125·8)	51·4 (34·1 to 73·1)	23·1 (19·3 to 28·7)
Belize*	2046	417·3 (329·4 to 507·1)	295·8 (209·2 to 384·6)	71·0 (59·2 to 83·1)	34·8 (22·9 to 49·5)	15·7 (13·0 to 19·4)	636·6 (502·5 to 773·7)	451·3 (319·2 to 586·7)	108·4 (90·3 to 126·8)	53·0 (34·9 to 75·5)	23·9 (19·9 to 29·6)
Belize*	2047	425·2 (334·0 to 519·5)	301·6 (213·9 to 393·6)	71·6 (59·6 to 83·8)	35·9 (23·5 to 51·4)	16·2 (13·4 to 20·4)	648·7 (509·6 to 792·5)	460·1 (326·4 to 600·5)	109·2 (90·9 to 127·8)	54·7 (35·8 to 78·5)	24·7 (20·4 to 31·1)
Belize*	2048	433·3 (339·2 to 530·7)	307·5 (216·6 to 403·6)	72·0 (59·9 to 84·3)	37·0 (24·1 to 53·6)	16·8 (13·8 to 21·2)	661·0 (517·5 to 809·7)	469·1 (330·4 to 615·7)	109·9 (91·4 to 128·7)	56·4 (36·7 to 81·7)	25·6 (21·1 to 32·4)
Belize*	2049	441·6 (343·5 to 542·3)	313·6 (218·1 to 413·2)	72·5 (60·3 to 85·0)	38·1 (24·6 to 55·7)	17·5 (14·2 to 22·4)	673·7 (524·0 to 827·3)	478·4 (332·8 to 630·4)	110·5 (91·9 to 129·6)	58·1 (37·6 to 84·9)	26·7 (21·6 to 34·2)
Belize*	2050	449·8 (345·7 to 551·0)	319·4 (222·4 to 422·8)	72·9 (60·6 to 85·6)	39·3 (25·2 to 57·9)	18·2 (14·7 to 23·5)	686·2 (527·5 to 840·6)	487·3 (339·2 to 645·0)	111·3 (92·4 to 130·6)	59·9 (38·5 to 88·3)	27·8 (22·4 to 35·8)
Bermuda	1995	4499·9 (3712·3 to 5415·7)	1266·0 (1031·5 to 1554·4)	1080·1 (841·0 to 1370·5)	2153·8 (1441·5 to 3048·4)	0·0 (0·0 to 0·0)	3178·7 (2622·4 to 3825·7)	894·3 (728·7 to 1098·0)	763·0 (594·1 to 968·1)	1521·5 (1018·3 to 2153·4)	0·0 (0·0 to 0·0)
Bermuda	1996	4533·9 (3793·6 to 5457·5)	1286·6 (1050·6 to 1567·1)	1160·9 (902·8 to 1458·3)	2086·4 (1411·7 to 2954·3)	0·0 (0·0 to 0·0)	3202·8 (2679·8 to 3855·2)	908·9 (742·2 to 1107·0)	820·1 (637·8 to 1030·1)	1473·8 (997·2 to 2086·9)	0·0 (0·0 to 0·0)
Bermuda	1997	4300·0 (3612·1 to 5112·5)	1226·9 (1010·5 to 1495·0)	1274·7 (978·2 to 1587·2)	1798·4 (1223·4 to 2536·6)	0·0 (0·0 to 0·0)	3037·6 (2551·6 to 3611·5)	866·7 (713·8 to 1056·1)	900·4 (691·0 to 1121·2)	1270·4 (864·2 to 1791·8)	0·0 (0·0 to 0·0)
Bermuda	1998	4193·1 (3566·4 to 4929·2)	1224·9 (1007·5 to 1486·1)	1467·7 (1136·4 to 1817·9)	1500·5 (1039·6 to 2106·3)	0·0 (0·0 to 0·0)	2962·0 (2519·3 to 3482·0)	865·3 (711·7 to 1049·8)	1036·8 (802·8 to 1284·2)	1060·0 (734·4 to 1487·9)	0·0 (0·0 to 0·0)
Bermuda	1999	4190·3 (3581·0 to 4812·8)	1237·3 (1029·6 to 1497·8)	1714·0 (1343·2 to 2128·3)	1239·0 (875·7 to 1702·8)	0·0 (0·0 to 0·0)	2960·0 (2529·6 to 3399·8)	874·0 (727·3 to 1058·0)	1210·8 (948·8 to 1503·5)	875·2 (618·6 to 1202·8)	0·0 (0·0 to 0·0)
Bermuda	2000	4325·9 (3741·3 to 4915·8)	1285·2 (1085·4 to 1542·3)	1998·1 (1589·4 to 2484·8)	1042·6 (749·4 to 1429·0)	0·0 (0·0 to 0·0)	3055·9 (2642·9 to 3472·5)	907·9 (766·7 to 1089·5)	1411·5 (1122·8 to 1755·3)	736·5 (529·4 to 1009·4)	0·0 (0·0 to 0·0)

Bermuda	2001	4623·1 (4044·7 to 5238·7)	1382·2 (1186·8 to 1616·4)	2320·5 (1864·8 to 2882·4)	920·4 (668·3 to 1231·1)	0·0 (0·0 to 0·0)	3265·8 (2857·2 to 3700·6)	976·4 (838·3 to 1141·9)	1639·2 (1317·3 to 2036·1)	650·2 (472·1 to 869·7)	0·0 (0·0 to 0·0)
Bermuda	2002	4785·0 (4212·6 to 5361·6)	1369·6 (1189·7 to 1576·2)	2534·9 (2059·3 to 3061·5)	880·5 (659·3 to 1165·4)	0·0 (0·0 to 0·0)	3380·2 (2975·8 to 3787·4)	967·5 (840·4 to 1113·5)	1790·7 (1454·7 to 2162·7)	622·0 (465·7 to 823·2)	0·0 (0·0 to 0·0)
Bermuda	2003	5123·3 (4591·4 to 5704·8)	1431·8 (1266·0 to 1613·4)	2777·6 (2322·2 to 3305·6)	913·9 (694·5 to 1178·1)	0·0 (0·0 to 0·0)	3619·1 (3243·4 to 4029·9)	1011·4 (894·3 to 1139·7)	1962·1 (1640·4 to 2335·1)	645·6 (490·6 to 832·2)	0·0 (0·0 to 0·0)
Bermuda	2004	5400·3 (4889·2 to 5983·6)	1470·7 (1333·3 to 1604·2)	2969·3 (2537·6 to 3453·9)	960·3 (754·0 to 1209·3)	0·0 (0·0 to 0·0)	3814·8 (3453·8 to 4226·8)	1038·9 (941·8 to 1133·2)	2097·6 (1792·6 to 2439·9)	678·3 (532·6 to 854·3)	0·0 (0·0 to 0·0)
Bermuda	2005	5355·9 (4881·5 to 5860·0)	1504·6 (1383·4 to 1617·3)	2764·3 (2369·5 to 3172·5)	1087·0 (886·8 to 1315·5)	0·0 (0·0 to 0·0)	3783·4 (3448·4 to 4139·6)	1062·9 (977·2 to 1142·5)	1952·7 (1673·8 to 2241·1)	767·9 (626·5 to 929·3)	0·0 (0·0 to 0·0)
Bermuda	2006	5268·7 (4864·2 to 5710·8)	1554·8 (1435·9 to 1669·1)	2281·3 (1958·1 to 2639·7)	1432·6 (1222·7 to 1667·0)	0·0 (0·0 to 0·0)	3721·8 (3436·1 to 4034·1)	1098·3 (1014·4 to 1179·1)	1611·5 (1383·2 to 1864·7)	1012·0 (863·7 to 1177·6)	0·0 (0·0 to 0·0)
Bermuda	2007	5787·7 (5406·3 to 6237·0)	1651·1 (1535·7 to 1779·4)	1550·3 (1317·0 to 1818·8)	2586·3 (2275·4 to 2940·8)	0·0 (0·0 to 0·0)	4088·5 (3819·1 to 4405·9)	1166·4 (1084·8 to 1257·0)	1095·2 (930·3 to 1284·8)	1826·9 (1607·4 to 2077·4)	0·0 (0·0 to 0·0)
Bermuda	2008	6259·0 (5884·0 to 6655·7)	1722·1 (1609·3 to 1850·5)	1249·6 (1048·4 to 1482·0)	3287·3 (2967·7 to 3621·4)	0·0 (0·0 to 0·0)	4421·4 (4156·5 to 4701·6)	1216·5 (1136·8 to 1307·2)	882·7 (740·6 to 1046·9)	2322·2 (2096·4 to 2558·2)	0·0 (0·0 to 0·0)
Bermuda	2009	6776·2 (6397·2 to 7189·3)	1850·3 (1731·4 to 1972·6)	1112·3 (919·2 to 1339·5)	3813·6 (3512·8 to 4151·8)	0·0 (0·0 to 0·0)	4786·7 (4519·0 to 5078·5)	1307·1 (1223·1 to 1393·5)	785·7 (649·3 to 946·3)	2693·9 (2481·5 to 2932·9)	0·0 (0·0 to 0·0)
Bermuda	2010	7309·9 (6930·1 to 7727·1)	2101·1 (1974·5 to 2232·1)	1069·6 (876·1 to 1290·0)	4139·2 (3837·5 to 4466·1)	0·0 (0·0 to 0·0)	5163·8 (4895·4 to 5458·5)	1484·2 (1394·8 to 1576·8)	755·5 (618·9 to 911·3)	2924·0 (2710·8 to 3154·9)	0·0 (0·0 to 0·0)
Bermuda	2011	7629·8 (7240·2 to 8051·6)	2266·7 (2145·5 to 2398·4)	1035·2 (847·9 to 1261·3)	4327·9 (4024·6 to 4668·5)	0·0 (0·0 to 0·0)	5389·7 (5114·5 to 5687·7)	1601·2 (1515·6 to 1694·2)	731·3 (599·0 to 891·0)	3057·2 (2843·0 to 3297·9)	0·0 (0·0 to 0·0)
Bermuda	2012	7606·0 (7207·5 to 8026·2)	2280·6 (2158·7 to 2418·9)	984·8 (793·1 to 1208·0)	4340·6 (4022·2 to 4666·3)	0·0 (0·0 to 0·0)	5372·9 (5091·4 to 5669·7)	1611·1 (1524·9 to 1708·7)	695·7 (560·3 to 853·3)	3066·2 (2841·3 to 3296·3)	0·0 (0·0 to 0·0)
Bermuda	2013	7610·5 (7221·2 to 8023·9)	2258·3 (2134·9 to 2400·8)	906·2 (730·5 to 1115·3)	4446·0 (4113·0 to 4768·1)	0·0 (0·0 to 0·0)	5376·1 (5101·1 to 5668·2)	1595·3 (1508·1 to 1696·0)	640·2 (516·0 to 787·9)	3140·7 (2905·4 to 3368·2)	0·0 (0·0 to 0·0)
Bermuda	2014	7365·4 (6993·5 to 7776·2)	2139·5 (2012·5 to 2273·8)	804·0 (638·5 to 996·5)	4421·9 (4107·7 to 4737·7)	0·0 (0·0 to 0·0)	5203·0 (4940·3 to 5493·1)	1511·3 (1421·7 to 1606·2)	567·9 (451·1 to 703·9)	3123·7 (2901·7 to 3346·7)	0·0 (0·0 to 0·0)

Bermuda	2015	7267·0 (6867·1 to 7681·4)	2069·4 (1937·2 to 2217·8)	745·1 (587·4 to 931·6)	4452·5 (4140·7 to 4793·0)	0·0 (0·0 to 0·0)	5133·5 (4850·9 to 5426·2)	1461·9 (1368·4 to 1566·6)	526·3 (414·9 to 658·1)	3145·3 (2925·0 to 3385·8)	0·0 (0·0 to 0·0)
Bermuda	2016	7281·9 (6808·9 to 7815·4)	2035·4 (1877·1 to 2221·2)	734·4 (581·0 to 919·5)	4512·0 (4116·2 to 4967·5)	0·0 (0·0 to 0·0)	5144·0 (4809·8 to 5520·9)	1437·8 (1326·0 to 1569·1)	518·8 (410·4 to 649·6)	3187·3 (2907·7 to 3509·1)	0·0 (0·0 to 0·0)
Bermuda	2017	7549·6 (6797·8 to 8321·6)	2111·5 (1904·6 to 2340·8)	749·6 (581·2 to 933·4)	4688·5 (4045·0 to 5418·6)	0·0 (0·0 to 0·0)	5333·1 (4802·0 to 5878·4)	1491·5 (1345·4 to 1653·6)	529·6 (410·6 to 659·3)	3312·0 (2857·4 to 3827·7)	0·0 (0·0 to 0·0)
Bermuda	2018	7688·7 (6701·7 to 8855·3)	2144·7 (1891·0 to 2445·1)	759·2 (586·9 to 937·3)	4784·8 (3829·6 to 5943·5)	0·0 (0·0 to 0·0)	5431·3 (4734·1 to 6255·4)	1515·0 (1335·8 to 1727·2)	536·3 (414·6 to 662·1)	3380·0 (2705·3 to 4198·5)	0·0 (0·0 to 0·0)
Bermuda	2019	8049·1 (7006·8 to 9277·2)	2247·9 (1977·3 to 2560·7)	774·6 (599·0 to 956·2)	5026·6 (4042·4 to 6265·3)	0·0 (0·0 to 0·0)	5686·0 (4949·7 to 6553·5)	1587·9 (1396·8 to 1808·9)	547·2 (423·1 to 675·5)	3550·8 (2855·6 to 4425·8)	0·0 (0·0 to 0·0)
Bermuda	2020	7877·7 (6866·3 to 9085·5)	2309·2 (2020·9 to 2634·0)	724·8 (560·4 to 894·6)	4843·7 (3906·8 to 6066·2)	0·0 (0·0 to 0·0)	5564·9 (4850·4 to 6418·1)	1631·2 (1427·6 to 1860·6)	512·0 (395·9 to 631·9)	3421·6 (2759·8 to 4285·2)	0·0 (0·0 to 0·0)
Bermuda	2021	7680·9 (6684·5 to 8880·6)	2213·5 (1937·1 to 2524·8)	693·5 (536·4 to 856·2)	4773·9 (3859·5 to 5988·7)	0·0 (0·0 to 0·0)	5425·9 (4722·0 to 6273·3)	1563·6 (1368·4 to 1783·5)	489·9 (378·9 to 604·8)	3372·3 (2726·3 to 4230·5)	0·0 (0·0 to 0·0)
Bermuda	2022	7713·3 (6673·9 to 8958·8)	2125·3 (1858·0 to 2420·1)	690·9 (534·6 to 853·4)	4897·1 (3950·5 to 6151·9)	0·0 (0·0 to 0·0)	5448·7 (4714·5 to 6328·5)	1501·4 (1312·5 to 1709·6)	488·0 (377·6 to 602·8)	3459·4 (2790·7 to 4345·8)	0·0 (0·0 to 0·0)
Bermuda	2023	7874·2 (6793·1 to 9187·8)	2163·3 (1885·5 to 2475·1)	688·0 (532·6 to 850·4)	5022·9 (4042·6 to 6343·2)	0·0 (0·0 to 0·0)	5562·3 (4798·7 to 6490·3)	1528·1 (1331·9 to 1748·4)	486·0 (376·2 to 600·7)	3548·2 (2855·7 to 4480·9)	0·0 (0·0 to 0·0)
Bermuda	2024	8044·2 (6918·9 to 9440·9)	2204·1 (1913·9 to 2522·9)	685·2 (530·8 to 847·6)	5154·9 (4118·1 to 6531·4)	0·0 (0·0 to 0·0)	5682·4 (4887·6 to 6669·1)	1557·0 (1352·0 to 1782·2)	484·0 (374·9 to 598·7)	3641·4 (2909·1 to 4613·8)	0·0 (0·0 to 0·0)
Bermuda	2025	8211·2 (7049·1 to 9651·9)	2244·0 (1943·8 to 2563·3)	681·9 (528·4 to 844·0)	5285·4 (4197·2 to 6729·3)	0·0 (0·0 to 0·0)	5800·5 (4979·6 to 6818·2)	1585·2 (1373·1 to 1810·7)	481·7 (373·3 to 596·2)	3733·6 (2964·9 to 4753·6)	0·0 (0·0 to 0·0)
Bermuda	2026	8384·1 (7165·7 to 9891·8)	2286·0 (1974·5 to 2631·0)	678·8 (526·3 to 840·7)	5419·2 (4288·9 to 6937·6)	0·0 (0·0 to 0·0)	5922·5 (5061·9 to 6987·6)	1614·9 (1394·8 to 1858·6)	479·5 (371·8 to 593·9)	3828·2 (3029·7 to 4900·7)	0·0 (0·0 to 0·0)
Bermuda	2027	8549·5 (7280·0 to 10118·4)	2323·9 (2008·5 to 2681·4)	675·4 (524·0 to 837·1)	5550·1 (4353·8 to 7142·2)	0·0 (0·0 to 0·0)	6039·4 (5142·7 to 7147·7)	1641·6 (1418·8 to 1894·2)	477·1 (370·2 to 591·3)	3920·6 (3075·6 to 5045·3)	0·0 (0·0 to 0·0)
Bermuda	2028	8713·7 (7376·7 to 10334·7)	2360·5 (2045·3 to 2726·8)	672·1 (521·5 to 833·4)	5681·0 (4439·2 to 7337·3)	0·0 (0·0 to 0·0)	6155·4 (5210·9 to 7300·5)	1667·5 (1444·8 to 1926·3)	474·8 (368·4 to 588·7)	4013·1 (3135·9 to 5183·1)	0·0 (0·0 to 0·0)

Bermuda	2029	8803·2 (7422·1 to 10491·6)	2375·2 (2056·2 to 2747·4)	663·3 (514·9 to 822·6)	5764·7 (4457·2 to 7475·0)	0·0 (0·0 to 0·0)	6218·6 (5243·0 to 7411·3)	1677·9 (1452·5 to 1940·7)	468·5 (363·7 to 581·1)	4072·2 (3148·6 to 5280·4)	0·0 (0·0 to 0·0)
Bermuda	2030	8886·5 (7439·1 to 10630·0)	2387·1 (2054·5 to 2773·1)	654·3 (508·1 to 811·5)	5845·1 (4478·9 to 7618·4)	0·0 (0·0 to 0·0)	6277·5 (5255·0 to 7509·1)	1686·3 (1451·3 to 1959·0)	462·2 (358·9 to 573·3)	4129·0 (3163·9 to 5381·7)	0·0 (0·0 to 0·0)
Bermuda	2031	8967·9 (7480·7 to 10764·2)	2397·2 (2057·2 to 2783·5)	645·7 (501·5 to 800·9)	5925·0 (4500·7 to 7758·3)	0·0 (0·0 to 0·0)	6335·0 (5284·4 to 7603·9)	1693·4 (1453·2 to 1966·3)	456·1 (354·3 to 565·8)	4185·5 (3179·4 to 5480·5)	0·0 (0·0 to 0·0)
Bermuda	2032	9043·0 (7515·8 to 10882·5)	2405·3 (2061·4 to 2789·3)	637·1 (495·1 to 790·3)	6000·5 (4526·1 to 7851·1)	0·0 (0·0 to 0·0)	6388·0 (5309·2 to 7687·5)	1699·1 (1456·2 to 1970·4)	450·1 (349·7 to 558·3)	4238·8 (3197·2 to 5546·1)	0·0 (0·0 to 0·0)
Bermuda	2033	9110·5 (7535·1 to 11013·3)	2409·1 (2062·8 to 2809·1)	628·6 (488·6 to 779·7)	6072·8 (4539·7 to 7979·3)	0·0 (0·0 to 0·0)	6435·7 (5322·8 to 7779·8)	1701·8 (1457·2 to 1984·4)	444·1 (345·2 to 550·8)	4289·8 (3206·9 to 5636·6)	0·0 (0·0 to 0·0)
Bermuda	2034	9175·0 (7537·2 to 11162·6)	2411·1 (2054·7 to 2819·7)	620·3 (482·3 to 769·3)	6143·6 (4571·9 to 8100·2)	0·0 (0·0 to 0·0)	6481·3 (5324·3 to 7885·3)	1703·2 (1451·5 to 1991·8)	438·2 (340·7 to 543·5)	4339·9 (3229·6 to 5722·0)	0·0 (0·0 to 0·0)
Bermuda	2035	9255·6 (7564·9 to 11282·3)	2415·9 (2054·7 to 2836·4)	613·5 (477·1 to 760·8)	6226·2 (4607·4 to 8255·3)	0·0 (0·0 to 0·0)	6538·2 (5343·9 to 7969·9)	1706·6 (1451·4 to 2003·7)	433·4 (337·0 to 537·5)	4398·2 (3254·7 to 5831·6)	0·0 (0·0 to 0·0)
Bermuda	2036	9328·7 (7586·9 to 11402·9)	2417·0 (2051·1 to 2841·1)	606·6 (471·2 to 752·5)	6305·0 (4623·4 to 8403·1)	0·0 (0·0 to 0·0)	6589·8 (5359·5 to 8055·0)	1707·4 (1448·9 to 2007·0)	428·5 (332·9 to 531·5)	4453·9 (3266·0 to 5936·0)	0·0 (0·0 to 0·0)
Bermuda	2037	9408·4 (7624·9 to 11541·5)	2419·9 (2059·7 to 2816·4)	600·4 (466·4 to 744·8)	6388·1 (4640·5 to 8564·4)	0·0 (0·0 to 0·0)	6646·1 (5386·3 to 8153·0)	1709·5 (1455·0 to 1989·5)	424·1 (329·5 to 526·1)	4512·6 (3278·1 to 6049·9)	0·0 (0·0 to 0·0)
Bermuda	2038	9489·3 (7641·6 to 11678·3)	2422·4 (2058·4 to 2833·5)	594·6 (461·8 to 737·6)	6472·4 (4663·8 to 8704·4)	0·0 (0·0 to 0·0)	6703·3 (5398·0 to 8249·6)	1711·2 (1454·1 to 2001·6)	420·0 (326·2 to 521·0)	4572·2 (3294·5 to 6148·8)	0·0 (0·0 to 0·0)
Bermuda	2039	9578·8 (7679·8 to 11822·6)	2424·5 (2060·8 to 2836·5)	589·7 (458·2 to 731·5)	6564·6 (4702·8 to 8855·4)	0·0 (0·0 to 0·0)	6766·5 (5425·0 to 8351·6)	1712·7 (1455·7 to 2003·7)	416·5 (323·7 to 516·7)	4637·3 (3322·1 to 6255·5)	0·0 (0·0 to 0·0)
Bermuda	2040	9662·4 (7716·9 to 11968·0)	2423·6 (2046·8 to 2846·0)	584·7 (454·4 to 725·4)	6654·2 (4747·1 to 9001·5)	0·0 (0·0 to 0·0)	6825·6 (5451·3 to 8454·2)	1712·0 (1445·9 to 2010·5)	413·0 (321·0 to 512·4)	4700·5 (3353·4 to 6358·7)	0·0 (0·0 to 0·0)
Bermuda	2041	9764·2 (7754·3 to 12132·2)	2426·3 (2045·9 to 2853·6)	581·0 (451·5 to 720·8)	6756·9 (4791·9 to 9190·2)	0·0 (0·0 to 0·0)	6897·5 (5477·7 to 8570·2)	1714·0 (1445·2 to 2015·8)	410·4 (319·0 to 509·2)	4773·1 (3385·0 to 6492·0)	0·0 (0·0 to 0·0)
Bermuda	2042	9859·9 (7795·3 to 12284·7)	2426·9 (2050·0 to 2848·5)	577·0 (448·4 to 716·0)	6856·1 (4835·8 to 9349·1)	0·0 (0·0 to 0·0)	6965·1 (5506·7 to 8678·0)	1714·3 (1448·2 to 2012·2)	407·6 (316·8 to 505·8)	4843·2 (3416·0 to 6604·3)	0·0 (0·0 to 0·0)

Bermuda	2043	9957·2 (7835·6 to 12467·2)	2427·5 (2045·6 to 2845·0)	573·0 (445·6 to 711·8)	6956·7 (4870·1 to 9508·2)	0·0 (0·0 to 0·0)	7033·8 (5535·1 to 8806·9)	1714·8 (1445·0 to 2009·7)	404·8 (314·7 to 502·8)	4914·3 (3440·3 to 6716·7)	0·0 (0·0 to 0·0)
Bermuda	2044	10059·7 (7864·3 to 12628·2)	2428·7 (2048·1 to 2852·7)	569·4 (443·1 to 708·0)	7061·6 (4934·3 to 9672·5)	0·0 (0·0 to 0·0)	7106·2 (5555·4 to 8920·7)	1715·6 (1446·8 to 2015·2)	402·3 (313·0 to 500·2)	4988·3 (3485·6 to 6832·7)	0·0 (0·0 to 0·0)
Bermuda	2045	10159·8 (7910·6 to 12819·3)	2428·1 (2051·8 to 2858·5)	565·8 (440·6 to 703·7)	7165·9 (4984·6 to 9834·2)	0·0 (0·0 to 0·0)	7177·0 (5588·1 to 9055·6)	1715·2 (1449·4 to 2019·3)	399·7 (311·2 to 497·1)	5062·0 (3521·2 to 6946·9)	0·0 (0·0 to 0·0)
Bermuda	2046	10262·5 (7944·4 to 13010·9)	2428·5 (2044·2 to 2855·9)	562·4 (438·2 to 699·8)	7271·6 (5037·6 to 10012·2)	0·0 (0·0 to 0·0)	7249·5 (5612·0 to 9191·0)	1715·5 (1444·1 to 2017·5)	397·3 (309·6 to 494·3)	5136·7 (3558·6 to 7072·7)	0·0 (0·0 to 0·0)
Bermuda	2047	10369·2 (7986·3 to 13178·6)	2427·8 (2036·8 to 2863·7)	559·4 (436·2 to 697·1)	7382·0 (5073·3 to 10221·5)	0·0 (0·0 to 0·0)	7324·8 (5641·6 to 9309·4)	1715·0 (1438·8 to 2022·9)	395·1 (308·1 to 492·4)	5214·7 (3583·8 to 7220·5)	0·0 (0·0 to 0·0)
Bermuda	2048	10485·8 (8044·4 to 13383·7)	2429·3 (2029·3 to 2868·4)	556·9 (434·1 to 694·0)	7499·6 (5121·4 to 10400·6)	0·0 (0·0 to 0·0)	7407·3 (5682·6 to 9454·3)	1716·1 (1433·5 to 2026·2)	393·4 (306·7 to 490·3)	5297·7 (3617·8 to 7347·1)	0·0 (0·0 to 0·0)
Bermuda	2049	10600·5 (8104·1 to 13589·8)	2429·9 (2022·2 to 2861·8)	554·4 (431·8 to 690·8)	7616·3 (5179·5 to 10597·5)	0·0 (0·0 to 0·0)	7488·3 (5724·8 to 9599·9)	1716·5 (1428·5 to 2021·6)	391·6 (305·1 to 488·0)	5380·2 (3658·8 to 7486·1)	0·0 (0·0 to 0·0)
Bermuda	2050	10721·2 (8170·1 to 13823·9)	2430·5 (2027·3 to 2884·8)	552·3 (429·9 to 688·2)	7738·5 (5233·5 to 10826·1)	0·0 (0·0 to 0·0)	7573·5 (5771·4 to 9765·3)	1716·9 (1432·1 to 2037·8)	390·1 (303·7 to 486·1)	5466·5 (3697·0 to 7647·6)	0·0 (0·0 to 0·0)
Bolivia*	1995	84·9 (75·4 to 95·2)	39·1 (31·9 to 45·7)	32·8 (26·2 to 40·0)	6·5 (4·4 to 9·2)	6·5 (6·5 to 6·5)	213·2 (189·3 to 239·0)	98·1 (80·0 to 114·9)	82·4 (65·9 to 100·4)	16·3 (11·1 to 23·1)	16·4 (16·4 to 16·4)
Bolivia*	1996	92·8 (82·8 to 103·5)	44·1 (36·5 to 51·6)	34·1 (27·8 to 41·2)	6·6 (4·5 to 9·5)	7·9 (7·9 to 7·9)	233·0 (207·9 to 259·8)	110·7 (91·7 to 129·6)	85·7 (69·7 to 103·4)	16·7 (11·3 to 23·8)	19·9 (19·9 to 19·9)
Bolivia*	1997	98·1 (87·9 to 109·2)	48·2 (40·7 to 56·5)	35·9 (29·4 to 43·2)	6·7 (4·5 to 9·6)	7·3 (7·3 to 7·3)	246·3 (220·8 to 274·2)	121·1 (102·1 to 141·8)	90·1 (73·9 to 108·6)	16·8 (11·3 to 24·1)	18·2 (18·2 to 18·2)
Bolivia*	1998	109·1 (98·4 to 120·5)	53·7 (46·0 to 62·6)	38·9 (32·3 to 46·7)	6·7 (4·6 to 9·6)	9·7 (9·7 to 9·7)	273·8 (247·2 to 302·6)	135·0 (115·5 to 157·1)	97·7 (81·1 to 117·2)	16·9 (11·5 to 24·0)	24·3 (24·3 to 24·3)
Bolivia*	1999	110·6 (100·4 to 122·5)	55·9 (48·4 to 64·3)	40·3 (33·5 to 48·1)	6·5 (4·4 to 9·1)	7·9 (7·9 to 7·9)	277·8 (252·1 to 307·6)	140·3 (121·5 to 161·5)	101·2 (84·1 to 120·7)	16·3 (11·1 to 22·9)	20·0 (20·0 to 20·0)
Bolivia*	2000	113·0 (103·0 to 124·3)	56·4 (49·2 to 64·9)	38·2 (31·7 to 45·8)	6·0 (4·1 to 8·4)	12·3 (12·3 to 12·3)	283·7 (258·5 to 312·1)	141·7 (123·6 to 162·9)	95·9 (79·6 to 114·9)	15·1 (10·4 to 21·0)	30·9 (30·9 to 30·9)

Bolivia*	2001	114·9 (104·9 to 126·2)	59·9 (52·2 to 68·9)	38·4 (32·0 to 45·7)	6·7 (4·6 to 9·3)	9·8 (9·8 to 9·8)	288·4 (263·3 to 316·9)	150·5 (131·1 to 172·9)	96·4 (80·5 to 114·7)	16·8 (11·7 to 23·3)	24·7 (24·7 to 24·7)
Bolivia*	2002	114·7 (104·0 to 126·1)	61·8 (54·1 to 70·4)	38·6 (32·3 to 46·0)	6·9 (4·7 to 9·7)	7·5 (7·5 to 7·5)	288·0 (261·2 to 316·5)	155·1 (135·9 to 176·8)	96·8 (81·1 to 115·6)	17·3 (11·7 to 24·4)	18·8 (18·8 to 18·8)
Bolivia*	2003	118·1 (107·3 to 129·7)	61·5 (53·9 to 70·3)	38·2 (32·0 to 45·2)	7·0 (4·9 to 10·1)	11·3 (11·3 to 11·3)	296·5 (269·3 to 325·8)	154·5 (135·3 to 176·6)	95·9 (80·5 to 113·6)	17·7 (12·2 to 25·4)	28·4 (28·4 to 28·4)
Bolivia*	2004	118·6 (107·6 to 129·9)	62·9 (55·0 to 71·4)	38·0 (31·8 to 44·9)	7·1 (4·9 to 9·8)	10·6 (10·6 to 10·6)	297·9 (270·2 to 326·3)	157·9 (138·0 to 179·2)	95·5 (79·8 to 112·7)	17·8 (12·2 to 24·7)	26·7 (26·7 to 26·7)
Bolivia*	2005	119·2 (108·5 to 131·2)	65·8 (57·6 to 74·6)	38·3 (32·0 to 45·2)	7·2 (5·1 to 10·0)	7·9 (7·9 to 7·9)	299·4 (272·5 to 329·4)	165·2 (144·7 to 187·3)	96·2 (80·2 to 113·5)	18·2 (12·7 to 25·2)	19·8 (19·8 to 19·8)
Bolivia*	2006	118·4 (108·2 to 129·6)	64·6 (57·0 to 72·9)	37·4 (31·3 to 44·5)	6·8 (4·8 to 9·7)	9·6 (9·6 to 9·6)	297·4 (271·6 to 325·4)	162·2 (143·2 to 183·1)	93·9 (78·5 to 111·7)	17·2 (12·0 to 24·4)	24·1 (24·1 to 24·1)
Bolivia*	2007	124·2 (113·3 to 135·5)	70·4 (62·1 to 79·5)	38·4 (31·9 to 45·9)	6·8 (4·7 to 9·5)	8·7 (8·7 to 8·7)	311·8 (284·5 to 340·3)	176·7 (156·0 to 199·5)	96·4 (80·1 to 115·3)	17·0 (11·7 to 23·9)	21·7 (21·7 to 21·7)
Bolivia*	2008	132·8 (121·1 to 144·7)	77·2 (67·9 to 86·9)	40·5 (33·6 to 48·3)	6·7 (4·6 to 9·3)	8·5 (8·5 to 8·5)	333·4 (304·0 to 363·3)	193·8 (170·4 to 218·3)	101·6 (84·3 to 121·3)	16·8 (11·6 to 23·4)	21·3 (21·3 to 21·3)
Bolivia*	2009	138·8 (126·1 to 151·1)	82·1 (72·4 to 92·1)	43·4 (36·5 to 51·7)	6·8 (4·7 to 9·5)	6·6 (6·6 to 6·6)	348·6 (316·7 to 379·5)	206·2 (181·8 to 231·3)	108·9 (91·7 to 129·8)	17·0 (11·9 to 23·9)	16·5 (16·5 to 16·5)
Bolivia*	2010	139·2 (126·8 to 151·0)	80·2 (70·6 to 89·6)	45·4 (38·5 to 54·0)	6·4 (4·5 to 9·2)	7·1 (7·1 to 7·1)	349·4 (318·4 to 379·2)	201·3 (177·3 to 225·0)	114·1 (96·6 to 135·6)	16·2 (11·3 to 23·0)	17·8 (17·8 to 17·8)
Bolivia*	2011	153·2 (139·4 to 166·4)	89·9 (80·0 to 100·8)	49·5 (41·7 to 58·7)	6·7 (4·6 to 9·5)	7·1 (7·1 to 7·1)	384·7 (350·0 to 417·8)	225·7 (200·9 to 253·0)	124·3 (104·6 to 147·4)	16·9 (11·6 to 23·9)	17·8 (17·8 to 17·8)
Bolivia*	2012	159·5 (145·7 to 172·8)	94·7 (84·6 to 105·1)	52·7 (44·7 to 62·6)	6·8 (4·6 to 9·5)	5·3 (5·3 to 5·3)	400·5 (365·9 to 433·9)	237·8 (212·5 to 264·0)	132·4 (112·2 to 157·2)	17·0 (11·6 to 23·8)	13·3 (13·3 to 13·3)
Bolivia*	2013	176·9 (162·9 to 191·6)	107·3 (96·8 to 119·0)	56·8 (47·9 to 66·9)	7·2 (4·9 to 10·0)	5·7 (5·7 to 5·7)	444·1 (409·0 to 481·0)	269·3 (243·1 to 298·7)	142·5 (120·2 to 167·9)	18·0 (12·3 to 25·1)	14·2 (14·2 to 14·2)
Bolivia*	2014	197·4 (182·3 to 213·1)	126·1 (115·2 to 138·1)	60·1 (51·3 to 70·0)	7·8 (5·3 to 11·0)	3·4 (3·4 to 3·4)	495·6 (457·8 to 535·2)	316·6 (289·1 to 346·8)	150·9 (128·7 to 175·7)	19·7 (13·3 to 27·5)	8·4 (8·4 to 8·4)

Bolivia*	2015	217·9 (202·1 to 234·3)	142·4 (130·4 to 155·2)	61·9 (52·8 to 72·0)	8·7 (5·9 to 12·1)	4·9 (4·9 to 4·9)	547·2 (507·5 to 588·4)	357·6 (327·5 to 389·6)	155·4 (132·6 to 180·7)	21·8 (14·8 to 30·4)	12·4 (12·4 to 12·4)
Bolivia*	2016	221·6 (205·6 to 237·7)	146·9 (135·4 to 159·8)	61·7 (52·6 to 71·9)	9·2 (6·2 to 12·7)	3·8 (3·8 to 3·8)	556·4 (516·3 to 596·8)	369·0 (340·0 to 401·2)	154·9 (132·1 to 180·5)	23·0 (15·6 to 31·8)	9·5 (9·5 to 9·5)
Bolivia*	2017	230·3 (214·7 to 248·8)	155·5 (142·8 to 169·6)	60·0 (50·5 to 71·1)	9·5 (6·5 to 13·4)	5·4 (5·4 to 5·4)	578·3 (539·0 to 624·7)	390·4 (358·6 to 426·0)	150·7 (126·9 to 178·5)	23·8 (16·3 to 33·5)	13·5 (13·5 to 13·5)
Bolivia*	2018	233·3 (214·3 to 254·4)	162·6 (147·3 to 179·7)	58·6 (49·1 to 70·0)	9·4 (6·6 to 13·3)	2·7 (2·7 to 2·7)	585·9 (538·2 to 638·7)	408·3 (369·9 to 451·3)	147·2 (123·2 to 175·8)	23·7 (16·5 to 33·4)	6·8 (6·8 to 6·8)
Bolivia*	2019	232·9 (213·4 to 254·4)	161·8 (146·0 to 179·3)	58·6 (49·0 to 69·9)	9·7 (6·7 to 13·6)	2·8 (2·8 to 2·8)	584·9 (536·0 to 638·8)	406·4 (366·5 to 450·3)	147·1 (123·0 to 175·4)	24·3 (16·9 to 34·1)	7·1 (7·1 to 7·1)
Bolivia*	2020	265·5 (246·9 to 286·0)	199·6 (184·0 to 216·8)	52·5 (43·9 to 62·5)	8·9 (6·2 to 12·5)	4·6 (4·4 to 4·8)	666·8 (620·0 to 718·3)	501·2 (462·1 to 544·3)	131·7 (110·1 to 156·9)	22·3 (15·5 to 31·4)	11·5 (11·2 to 12·2)
Bolivia*	2021	197·0 (180·8 to 215·2)	135·0 (121·8 to 150·2)	48·4 (40·5 to 57·7)	8·4 (5·8 to 11·8)	5·2 (4·9 to 5·7)	494·8 (454·1 to 540·3)	339·0 (306·0 to 377·2)	121·6 (101·7 to 144·8)	21·1 (14·6 to 29·7)	13·0 (12·4 to 14·3)
Bolivia*	2022	197·4 (180·0 to 216·6)	136·1 (121·8 to 151·9)	48·6 (40·7 to 57·9)	8·6 (6·0 to 12·2)	4·1 (3·7 to 4·6)	495·6 (452·1 to 543·8)	341·7 (305·8 to 381·4)	122·2 (102·1 to 145·4)	21·6 (15·0 to 30·6)	10·2 (9·4 to 11·4)
Bolivia*	2023	199·3 (181·7 to 218·9)	137·4 (123·7 to 154·0)	48·8 (40·8 to 58·1)	8·8 (6·1 to 12·5)	4·2 (3·8 to 4·7)	500·4 (456·3 to 549·6)	345·1 (310·5 to 386·6)	122·6 (102·5 to 145·9)	22·2 (15·3 to 31·5)	10·5 (9·6 to 11·9)
Bolivia*	2024	201·1 (183·1 to 221·3)	138·8 (124·6 to 155·3)	49·0 (40·9 to 58·2)	9·1 (6·3 to 12·9)	4·3 (3·9 to 5·0)	505·0 (459·8 to 555·7)	348·4 (312·8 to 390·0)	122·9 (102·8 to 146·2)	22·8 (15·7 to 32·4)	10·9 (9·9 to 12·4)
Bolivia*	2025	202·9 (184·3 to 224·4)	140·0 (124·9 to 157·3)	49·1 (41·0 to 58·4)	9·3 (6·4 to 13·2)	4·5 (4·0 to 5·2)	509·4 (462·9 to 563·5)	351·5 (313·5 to 395·1)	123·3 (103·1 to 146·6)	23·3 (16·0 to 33·2)	11·3 (10·1 to 13·0)
Bolivia*	2026	205·2 (185·8 to 227·3)	141·8 (124·6 to 160·3)	49·3 (41·2 to 58·6)	9·5 (6·5 to 13·5)	4·6 (4·1 to 5·4)	515·2 (466·4 to 570·9)	356·0 (313·0 to 402·5)	123·7 (103·4 to 147·1)	23·9 (16·3 to 34·0)	11·6 (10·4 to 13·5)
Bolivia*	2027	207·5 (187·2 to 230·0)	143·5 (127·0 to 162·4)	49·5 (41·4 to 58·9)	9·8 (6·7 to 13·8)	4·7 (4·2 to 5·5)	521·1 (469·9 to 577·5)	360·3 (318·9 to 407·8)	124·3 (104·0 to 147·9)	24·6 (16·8 to 34·8)	11·9 (10·6 to 13·9)
Bolivia*	2028	210·1 (189·0 to 233·9)	145·3 (127·3 to 165·6)	49·8 (41·6 to 59·2)	10·1 (6·9 to 14·3)	4·8 (4·3 to 5·7)	527·5 (474·6 to 587·2)	364·9 (319·7 to 415·8)	125·1 (104·5 to 148·8)	25·3 (17·2 to 35·8)	12·2 (10·7 to 14·3)

Bolivia*	2029	214·4 (192·9 to 238·8)	148·9 (130·6 to 169·6)	50·2 (41·9 to 59·7)	10·4 (7·1 to 14·8)	4·9 (4·3 to 5·8)	538·3 (484·3 to 599·5)	374·0 (327·8 to 425·9)	126·0 (105·2 to 149·8)	26·0 (17·8 to 37·1)	12·4 (10·8 to 14·7)
Bolivia*	2030	219·0 (197·0 to 244·1)	152·8 (133·7 to 174·2)	50·6 (42·2 to 60·1)	10·7 (7·3 to 15·3)	5·0 (4·3 to 5·9)	550·0 (494·7 to 613·0)	383·7 (335·7 to 437·5)	126·9 (106·0 to 151·0)	26·8 (18·3 to 38·3)	12·5 (10·9 to 14·9)
Bolivia*	2031	223·9 (201·4 to 249·8)	156·8 (137·1 to 178·6)	51·0 (42·5 to 60·6)	11·0 (7·5 to 15·7)	5·0 (4·4 to 6·1)	562·1 (505·7 to 627·1)	393·8 (344·4 to 448·4)	128·0 (106·8 to 152·2)	27·7 (18·8 to 39·4)	12·7 (11·0 to 15·3)
Bolivia*	2032	228·9 (206·1 to 255·5)	161·0 (140·7 to 183·5)	51·4 (42·9 to 61·1)	11·4 (7·7 to 16·1)	5·1 (4·4 to 6·2)	574·8 (517·5 to 641·5)	404·3 (353·3 to 460·8)	129·1 (107·7 to 153·5)	28·5 (19·4 to 40·5)	12·8 (11·1 to 15·5)
Bolivia*	2033	234·3 (211·0 to 261·2)	165·5 (145·0 to 188·5)	51·9 (43·3 to 61·7)	11·7 (8·0 to 16·7)	5·1 (4·4 to 6·2)	588·4 (529·7 to 655·9)	415·6 (364·1 to 473·3)	130·4 (108·8 to 155·0)	29·5 (20·0 to 41·9)	12·9 (11·1 to 15·6)
Bolivia*	2034	240·0 (216·0 to 267·6)	170·2 (149·0 to 193·6)	52·5 (43·8 to 62·4)	12·1 (8·2 to 17·4)	5·2 (4·4 to 6·3)	602·7 (542·4 to 671·8)	427·4 (374·2 to 486·1)	131·8 (110·0 to 156·7)	30·5 (20·6 to 43·7)	12·9 (11·1 to 15·7)
Bolivia*	2035	246·0 (221·2 to 274·4)	175·2 (153·3 to 199·4)	53·1 (44·3 to 63·1)	12·5 (8·5 to 18·2)	5·2 (4·4 to 6·3)	617·7 (555·5 to 689·1)	439·8 (385·0 to 500·6)	133·4 (111·2 to 158·5)	31·5 (21·3 to 45·6)	13·0 (11·1 to 15·8)
Bolivia*	2036	252·0 (226·5 to 281·2)	180·1 (157·6 to 205·2)	53·7 (44·8 to 63·8)	13·0 (8·7 to 18·9)	5·2 (4·4 to 6·4)	632·8 (568·6 to 706·0)	452·3 (395·7 to 515·2)	134·9 (112·5 to 160·3)	32·6 (21·9 to 47·6)	13·0 (11·2 to 16·0)
Bolivia*	2037	258·0 (231·6 to 288·3)	185·1 (161·6 to 211·1)	54·3 (45·2 to 64·5)	13·4 (9·0 to 19·7)	5·2 (4·5 to 6·4)	647·8 (581·6 to 723·8)	464·8 (405·7 to 530·1)	136·3 (113·6 to 162·0)	33·7 (22·6 to 49·6)	13·1 (11·3 to 16·2)
Bolivia*	2038	264·0 (236·9 to 294·8)	190·1 (166·0 to 217·1)	54·8 (45·7 to 65·2)	13·8 (9·3 to 20·4)	5·3 (4·5 to 6·6)	662·9 (594·8 to 740·1)	477·2 (416·8 to 545·3)	137·6 (114·7 to 163·7)	34·8 (23·3 to 51·2)	13·2 (11·3 to 16·5)
Bolivia*	2039	270·2 (242·1 to 301·9)	195·2 (170·3 to 222·9)	55·4 (46·1 to 65·9)	14·3 (9·5 to 21·2)	5·3 (4·5 to 6·6)	678·5 (608·0 to 758·1)	490·2 (427·7 to 559·6)	139·0 (115·9 to 165·5)	35·9 (23·9 to 53·2)	13·4 (11·4 to 16·6)
Bolivia*	2040	276·5 (247·5 to 309·3)	200·4 (174·5 to 229·3)	55·9 (46·6 to 66·5)	14·8 (9·8 to 21·9)	5·4 (4·5 to 6·7)	694·3 (621·4 to 776·5)	503·2 (438·1 to 575·7)	140·4 (116·9 to 167·1)	37·1 (24·5 to 55·0)	13·6 (11·4 to 16·9)
Bolivia*	2041	282·9 (252·7 to 316·6)	205·7 (178·8 to 235·5)	56·5 (47·0 to 67·2)	15·2 (10·0 to 22·7)	5·5 (4·6 to 6·8)	710·3 (634·4 to 795·0)	516·5 (448·9 to 591·3)	141·8 (118·1 to 168·8)	38·3 (25·2 to 57·0)	13·7 (11·6 to 17·2)
Bolivia*	2042	289·1 (258·1 to 323·7)	210·9 (183·1 to 241·6)	57·0 (47·5 to 67·9)	15·7 (10·3 to 23·5)	5·6 (4·6 to 7·0)	726·0 (648·1 to 812·7)	529·4 (459·8 to 606·8)	143·1 (119·2 to 170·4)	39·5 (26·0 to 59·0)	13·9 (11·6 to 17·6)

Bolivia*	2043	295·3 (263·8 to 330·7)	215·9 (187·4 to 247·6)	57·5 (47·9 to 68·5)	16·2 (10·7 to 24·2)	5·6 (4·7 to 7·1)	741·5 (662·4 to 830·4)	542·2 (470·4 to 621·8)	144·4 (120·3 to 172·0)	40·8 (26·8 to 60·8)	14·2 (11·7 to 17·9)
Bolivia*	2044	301·5 (268·9 to 338·1)	221·0 (191·5 to 253·5)	58·0 (48·3 to 69·1)	16·8 (11·0 to 25·0)	5·7 (4·8 to 7·3)	757·2 (675·2 to 848·9)	555·0 (480·8 to 636·5)	145·7 (121·4 to 173·6)	42·1 (27·5 to 62·7)	14·4 (12·0 to 18·4)
Bolivia*	2045	307·7 (274·4 to 345·1)	226·0 (195·8 to 259·4)	58·5 (48·7 to 69·7)	17·3 (11·2 to 25·7)	5·9 (4·8 to 7·5)	772·5 (688·9 to 866·5)	567·6 (491·7 to 651·4)	146·9 (122·4 to 175·0)	43·4 (28·2 to 64·5)	14·7 (12·1 to 18·9)
Bolivia*	2046	313·6 (278·9 to 352·2)	230·9 (199·8 to 265·0)	58·9 (49·1 to 70·3)	17·8 (11·5 to 26·7)	6·0 (4·9 to 7·7)	787·5 (700·4 to 884·3)	579·8 (501·6 to 665·5)	148·0 (123·3 to 176·4)	44·7 (29·0 to 66·9)	15·0 (12·3 to 19·4)
Bolivia*	2047	319·5 (284·5 to 358·9)	235·6 (203·8 to 270·6)	59·4 (49·5 to 70·8)	18·4 (11·8 to 27·6)	6·1 (5·0 to 8·0)	802·3 (714·3 to 901·2)	591·7 (511·7 to 679·4)	149·1 (124·3 to 177·8)	46·1 (29·7 to 69·3)	15·4 (12·5 to 20·0)
Bolivia*	2048	325·3 (289·6 to 365·3)	240·3 (207·8 to 276·1)	59·8 (49·9 to 71·4)	18·9 (12·1 to 28·5)	6·3 (5·1 to 8·1)	816·9 (727·1 to 917·3)	603·3 (521·7 to 693·2)	150·3 (125·2 to 179·3)	47·5 (30·4 to 71·5)	15·7 (12·7 to 20·3)
Bolivia*	2049	330·9 (294·9 to 371·7)	244·7 (211·8 to 281·1)	60·3 (50·2 to 71·9)	19·5 (12·4 to 29·6)	6·4 (5·1 to 8·4)	830·9 (740·4 to 933·3)	614·5 (531·8 to 705·9)	151·4 (126·1 to 180·6)	48·9 (31·2 to 74·4)	16·1 (12·9 to 21·1)
Bolivia*	2050	336·4 (299·3 to 377·8)	249·0 (215·6 to 286·2)	60·7 (50·6 to 72·5)	20·1 (12·7 to 30·5)	6·6 (5·3 to 8·7)	844·7 (751·6 to 948·6)	625·4 (541·4 to 718·6)	152·4 (127·1 to 182·0)	50·4 (32·0 to 76·7)	16·5 (13·2 to 21·8)
Brazil*	1995	421·5 (389·3 to 456·9)	172·8 (150·1 to 197·5)	165·1 (148·8 to 182·2)	82·4 (68·6 to 98·8)	1·1 (1·1 to 1·1)	951·6 (879·0 to 1031·6)	390·2 (338·9 to 445·9)	372·7 (335·9 to 411·4)	186·1 (154·9 to 223·1)	2·6 (2·6 to 2·6)
Brazil*	1996	432·7 (403·3 to 463·4)	179·3 (157·0 to 202·9)	166·6 (152·4 to 181·7)	85·4 (72·1 to 99·7)	1·4 (1·4 to 1·4)	976·9 (910·5 to 1046·4)	404·9 (354·5 to 458·2)	376·2 (344·2 to 410·1)	192·7 (162·7 to 225·2)	3·1 (3·1 to 3·1)
Brazil*	1997	437·3 (407·0 to 467·1)	182·2 (159·6 to 206·7)	166·8 (153·3 to 181·0)	86·7 (74·4 to 99·2)	1·6 (1·6 to 1·6)	987·3 (919·0 to 1054·5)	411·4 (360·3 to 466·7)	376·5 (346·2 to 408·6)	195·8 (168·0 to 223·9)	3·6 (3·6 to 3·6)
Brazil*	1998	443·3 (412·7 to 473·7)	186·2 (165·0 to 209·7)	168·0 (155·1 to 181·5)	88·1 (76·3 to 101·1)	1·0 (1·0 to 1·0)	1000·9 (931·8 to 1069·4)	420·4 (372·5 to 473·5)	379·3 (350·3 to 409·7)	199·0 (172·4 to 228·3)	2·2 (2·2 to 2·2)
Brazil*	1999	444·9 (417·0 to 475·5)	185·5 (163·9 to 210·0)	167·8 (155·0 to 181·7)	90·0 (78·9 to 102·5)	1·6 (1·6 to 1·6)	1004·6 (941·4 to 1073·5)	418·8 (370·0 to 474·2)	378·9 (350·0 to 410·2)	203·3 (178·2 to 231·4)	3·6 (3·6 to 3·6)
Brazil*	2000	443·5 (415·3 to 474·8)	181·9 (160·0 to 204·6)	167·1 (154·3 to 180·9)	93·3 (81·7 to 106·4)	1·2 (1·2 to 1·2)	1001·4 (937·7 to 1072·1)	410·8 (361·2 to 461·9)	377·2 (348·4 to 408·5)	210·7 (184·4 to 240·2)	2·7 (2·7 to 2·7)

Brazil*	2001	456·3 (427·7 to 487·5)	188·4 (166·9 to 210·7)	170·0 (156·4 to 184·0)	96·1 (84·6 to 108·7)	1·7 (1·7 to 1·7)	1030·3 (965·7 to 1100·6)	425·4 (376·9 to 475·7)	383·9 (353·1 to 415·4)	217·1 (190·9 to 245·4)	3·9 (3·9 to 3·9)
Brazil*	2002	469·8 (438·4 to 501·8)	198·2 (176·1 to 222·0)	171·2 (157·5 to 185·4)	99·2 (86·8 to 113·0)	1·2 (1·2 to 1·2)	1060·7 (989·9 to 1133·1)	447·4 (397·7 to 501·2)	386·5 (355·5 to 418·5)	224·0 (196·1 to 255·0)	2·8 (2·8 to 2·8)
Brazil*	2003	471·0 (441·3 to 501·5)	200·9 (178·8 to 225·0)	168·0 (155·3 to 181·7)	99·4 (87·1 to 113·5)	2·7 (2·7 to 2·7)	1063·4 (996·3 to 1132·4)	453·7 (403·7 to 508·0)	379·3 (350·6 to 410·2)	224·4 (196·7 to 256·3)	6·1 (6·1 to 6·1)
Brazil*	2004	465·0 (436·7 to 493·2)	197·8 (175·6 to 221·5)	165·8 (153·6 to 178·8)	100·5 (88·2 to 115·8)	0·9 (0·9 to 0·9)	1049·9 (985·9 to 1113·5)	446·5 (396·4 to 500·1)	374·4 (346·8 to 403·7)	227·0 (199·0 to 261·5)	1·9 (1·9 to 1·9)
Brazil*	2005	480·1 (452·0 to 510·7)	204·9 (182·6 to 229·6)	167·9 (154·5 to 180·6)	106·4 (93·7 to 121·4)	0·9 (0·9 to 0·9)	1083·9 (1020·4 to 1153·1)	462·6 (412·4 to 518·3)	379·0 (348·8 to 407·8)	240·3 (211·6 to 274·1)	2·0 (2·0 to 2·0)
Brazil*	2006	494·8 (465·3 to 526·3)	211·7 (189·3 to 236·0)	168·7 (155·8 to 182·6)	113·7 (100·9 to 127·9)	0·7 (0·7 to 0·7)	1117·2 (1050·6 to 1188·3)	477·9 (427·4 to 532·9)	380·8 (351·7 to 412·4)	256·8 (227·9 to 288·8)	1·7 (1·7 to 1·7)
Brazil*	2007	509·7 (480·5 to 539·5)	218·1 (195·3 to 242·9)	168·1 (155·0 to 182·1)	122·9 (110·4 to 136·9)	0·6 (0·6 to 0·6)	1150·9 (1085·0 to 1218·0)	492·4 (441·0 to 548·4)	379·5 (350·1 to 411·2)	277·6 (249·3 to 309·1)	1·4 (1·4 to 1·4)
Brazil*	2008	526·2 (496·3 to 556·8)	228·2 (205·0 to 252·6)	166·8 (153·9 to 180·0)	130·4 (117·4 to 144·3)	0·7 (0·7 to 0·7)	1188·0 (1120·5 to 1257·1)	515·3 (462·8 to 570·4)	376·5 (347·6 to 406·5)	294·5 (265·1 to 325·9)	1·7 (1·7 to 1·7)
Brazil*	2009	525·7 (497·7 to 556·9)	229·2 (205·4 to 253·9)	163·6 (151·0 to 176·8)	131·9 (118·9 to 145·5)	1·0 (1·0 to 1·0)	1186·8 (1123·8 to 1257·3)	517·4 (463·8 to 573·2)	369·3 (341·0 to 399·2)	297·8 (268·5 to 328·6)	2·3 (2·3 to 2·3)
Brazil*	2010	554·4 (524·1 to 588·4)	248·2 (223·5 to 275·7)	164·7 (151·4 to 178·4)	139·6 (126·4 to 154·4)	1·8 (1·8 to 1·8)	1251·8 (1183·4 to 1328·6)	560·5 (504·7 to 622·5)	371·9 (341·8 to 402·7)	315·3 (285·4 to 348·5)	4·1 (4·1 to 4·1)
Brazil*	2011	555·1 (525·5 to 589·3)	248·0 (223·9 to 275·7)	163·1 (149·8 to 177·3)	142·9 (129·4 to 157·5)	1·0 (1·0 to 1·0)	1253·2 (1186·5 to 1330·6)	559·9 (505·6 to 622·4)	368·3 (338·3 to 400·3)	322·7 (292·1 to 355·6)	2·3 (2·3 to 2·3)
Brazil*	2012	560·9 (530·4 to 594·5)	248·3 (223·8 to 274·4)	163·4 (150·0 to 176·9)	148·3 (134·2 to 163·7)	0·9 (0·9 to 0·9)	1266·4 (1197·6 to 1342·3)	560·6 (505·3 to 619·6)	368·9 (338·6 to 399·5)	334·7 (303·1 to 369·7)	2·1 (2·1 to 2·1)
Brazil*	2013	583·2 (550·3 to 616·0)	257·3 (232·6 to 285·0)	166·5 (153·5 to 180·1)	158·0 (143·8 to 173·2)	1·4 (1·4 to 1·4)	1316·8 (1242·5 to 1390·7)	581·0 (525·1 to 643·5)	375·9 (346·6 to 406·6)	356·7 (324·7 to 391·1)	3·2 (3·2 to 3·2)
Brazil*	2014	604·4 (571·7 to 636·4)	264·9 (239·3 to 291·0)	170·5 (158·8 to 184·8)	168·1 (154·4 to 183·7)	0·9 (0·9 to 0·9)	1364·6 (1290·9 to 1436·9)	598·2 (540·3 to 657·0)	384·9 (358·5 to 417·3)	379·5 (348·6 to 414·7)	2·0 (2·0 to 2·0)

Brazil*	2015	613·5 (578·5 to 645·8)	265·3 (241·7 to 291·5)	172·1 (159·7 to 185·9)	175·8 (160·4 to 191·8)	0·3 (0·3 to 0·3)	1385·2 (1306·3 to 1458·1)	599·0 (545·6 to 658·1)	388·5 (360·5 to 419·8)	396·8 (362·2 to 433·1)	0·8 (0·8 to 0·8)
Brazil*	2016	615·6 (583·3 to 650·2)	262·5 (238·5 to 288·7)	170·7 (157·7 to 184·1)	181·6 (166·9 to 197·7)	0·8 (0·8 to 0·8)	1389·8 (1317·1 to 1468·1)	592·7 (538·6 to 651·9)	385·4 (356·0 to 415·8)	410·0 (376·8 to 446·3)	1·7 (1·7 to 1·7)
Brazil*	2017	622·2 (588·4 to 659·5)	260·9 (234·1 to 290·2)	171·4 (157·3 to 187·2)	189·1 (172·3 to 205·8)	0·8 (0·8 to 0·8)	1404·9 (1328·5 to 1488·9)	589·2 (528·5 to 655·3)	387·0 (355·2 to 422·6)	427·1 (389·0 to 464·6)	1·7 (1·7 to 1·7)
Brazil*	2018	632·5 (591·6 to 674·9)	264·2 (234·0 to 297·0)	173·1 (154·7 to 192·8)	194·9 (174·9 to 216·7)	0·3 (0·3 to 0·3)	1428·1 (1335·8 to 1523·8)	596·5 (528·3 to 670·6)	390·8 (349·3 to 435·4)	440·2 (395·0 to 489·2)	0·7 (0·7 to 0·7)
Brazil*	2019	639·3 (598·0 to 681·9)	265·8 (235·7 to 299·0)	173·4 (154·8 to 193·2)	199·7 (178·9 to 222·2)	0·3 (0·3 to 0·3)	1443·3 (1350·1 to 1539·6)	600·1 (532·1 to 675·1)	391·5 (349·6 to 436·3)	450·9 (404·0 to 501·6)	0·7 (0·7 to 0·7)
Brazil*	2020	681·1 (640·8 to 722·7)	318·7 (288·8 to 351·7)	165·8 (148·0 to 184·8)	196·1 (175·5 to 218·4)	0·4 (0·4 to 0·4)	1537·9 (1446·8 to 1631·8)	719·7 (652·2 to 794·1)	374·4 (334·2 to 417·3)	442·9 (396·1 to 493·2)	1·0 (0·9 to 1·0)
Brazil*	2021	666·0 (623·2 to 710·6)	299·7 (265·3 to 338·4)	165·0 (147·3 to 184·0)	200·9 (180·1 to 223·1)	0·4 (0·4 to 0·4)	1503·7 (1407·2 to 1604·5)	676·6 (598·9 to 764·0)	372·6 (332·6 to 415·5)	453·5 (406·7 to 503·6)	0·8 (0·8 to 0·9)
Brazil*	2022	637·8 (596·5 to 680·6)	264·1 (233·0 to 298·8)	165·8 (148·0 to 185·0)	207·6 (185·5 to 230·6)	0·3 (0·3 to 0·3)	1439·9 (1346·8 to 1536·7)	596·3 (526·1 to 674·6)	374·3 (334·2 to 417·7)	468·7 (418·8 to 520·7)	0·7 (0·6 to 0·7)
Brazil*	2023	649·9 (607·3 to 693·9)	268·5 (236·0 to 303·3)	166·5 (148·7 to 185·7)	214·6 (191·8 to 238·4)	0·3 (0·3 to 0·3)	1467·3 (1371·3 to 1566·7)	606·2 (533·0 to 684·7)	375·9 (335·6 to 419·2)	484·5 (433·1 to 538·3)	0·7 (0·6 to 0·7)
Brazil*	2024	662·7 (618·8 to 708·0)	273·5 (240·8 to 308·8)	167·1 (149·1 to 186·2)	221·8 (197·9 to 246·9)	0·3 (0·3 to 0·3)	1496·2 (1397·1 to 1598·6)	617·5 (543·6 to 697·2)	377·3 (336·7 to 420·4)	500·7 (446·8 to 557·4)	0·7 (0·7 to 0·8)
Brazil*	2025	676·0 (630·5 to 722·9)	278·7 (245·4 to 316·3)	167·7 (149·8 to 187·0)	229·2 (204·8 to 255·0)	0·3 (0·3 to 0·4)	1526·4 (1423·6 to 1632·3)	629·3 (554·1 to 714·2)	378·7 (338·1 to 422·3)	517·6 (462·4 to 575·7)	0·7 (0·7 to 0·8)
Brazil*	2026	689·9 (643·3 to 736·2)	284·3 (250·2 to 323·4)	168·3 (150·3 to 187·7)	236·9 (211·2 to 263·7)	0·3 (0·3 to 0·4)	1557·7 (1452·4 to 1662·2)	642·0 (564·8 to 730·1)	380·1 (339·4 to 423·7)	534·9 (476·8 to 595·4)	0·8 (0·7 to 0·8)
Brazil*	2027	703·4 (655·6 to 752·1)	289·6 (253·6 to 328·5)	168·9 (150·8 to 188·2)	244·6 (217·2 to 274·7)	0·4 (0·3 to 0·4)	1588·2 (1480·2 to 1698·1)	653·8 (572·5 to 741·7)	381·3 (340·5 to 425·0)	552·3 (490·4 to 620·2)	0·8 (0·7 to 0·9)
Brazil*	2028	717·2 (667·6 to 767·8)	295·1 (258·6 to 337·3)	169·3 (151·2 to 188·8)	252·5 (224·2 to 284·9)	0·4 (0·3 to 0·4)	1619·3 (1507·3 to 1733·6)	666·2 (583·9 to 761·5)	382·2 (341·4 to 426·3)	570·0 (506·3 to 643·2)	0·8 (0·8 to 0·9)

Brazil*	2029	731·6 (679·9 to 784·2)	300·9 (264·0 to 344·0)	169·7 (151·6 to 189·2)	260·6 (229·6 to 296·7)	0·4 (0·4 to 0·4)	1651·9 (1535·0 to 1770·6)	679·4 (596·0 to 776·8)	383·2 (342·2 to 427·2)	588·4 (518·5 to 669·8)	0·9 (0·8 to 1·0)
Brazil*	2030	747·1 (693·1 to 802·3)	307·3 (269·4 to 351·0)	170·3 (152·1 to 189·7)	269·2 (235·6 to 308·1)	0·4 (0·4 to 0·4)	1686·9 (1564·9 to 1811·5)	693·8 (608·2 to 792·4)	384·4 (343·4 to 428·3)	607·7 (531·9 to 695·5)	0·9 (0·8 to 1·0)
Brazil*	2031	762·2 (705·4 to 820·4)	313·4 (274·8 to 357·9)	170·7 (152·4 to 190·1)	277·7 (240·8 to 319·7)	0·4 (0·4 to 0·5)	1721·0 (1592·7 to 1852·4)	707·7 (620·4 to 808·1)	385·4 (344·0 to 429·2)	627·0 (543·6 to 721·7)	0·9 (0·9 to 1·0)
Brazil*	2032	777·6 (718·1 to 837·7)	319·6 (279·9 to 366·2)	171·1 (152·8 to 190·5)	286·4 (246·3 to 332·3)	0·4 (0·4 to 0·5)	1755·7 (1621·3 to 1891·3)	721·7 (632·0 to 826·8)	386·3 (345·0 to 430·2)	646·7 (556·1 to 750·3)	1·0 (0·9 to 1·1)
Brazil*	2033	793·8 (731·7 to 856·6)	326·1 (284·9 to 373·1)	171·7 (153·3 to 191·3)	295·5 (252·8 to 344·6)	0·5 (0·4 to 0·5)	1792·2 (1652·0 to 1934·1)	736·4 (643·2 to 842·4)	387·6 (346·1 to 431·9)	667·2 (570·7 to 778·1)	1·0 (0·9 to 1·1)
Brazil*	2034	810·0 (744·3 to 874·0)	332·4 (290·4 to 380·9)	172·3 (153·9 to 192·1)	304·9 (258·7 to 358·3)	0·5 (0·4 to 0·5)	1828·9 (1680·6 to 1973·4)	750·5 (655·7 to 859·9)	389·0 (347·4 to 433·8)	688·4 (584·1 to 809·0)	1·1 (0·9 to 1·2)
Brazil*	2035	826·5 (758·1 to 895·5)	338·7 (295·4 to 386·6)	172·8 (154·4 to 192·9)	314·4 (264·7 to 372·6)	0·5 (0·4 to 0·5)	1866·0 (1711·8 to 2021·9)	764·7 (666·9 to 873·0)	390·2 (348·6 to 435·5)	709·9 (597·6 to 841·3)	1·1 (1·0 to 1·2)
Brazil*	2036	843·4 (771·3 to 915·5)	345·1 (299·7 to 395·3)	173·4 (155·0 to 193·5)	324·3 (270·8 to 387·2)	0·5 (0·5 to 0·6)	1904·1 (1741·4 to 2067·1)	779·3 (676·6 to 892·4)	391·6 (349·9 to 437·0)	732·2 (611·3 to 874·2)	1·1 (1·0 to 1·3)
Brazil*	2037	860·2 (784·9 to 935·9)	351·3 (304·6 to 401·0)	174·0 (155·4 to 194·2)	334·4 (276·6 to 401·3)	0·5 (0·5 to 0·6)	1942·1 (1772·1 to 2113·2)	793·2 (687·7 to 905·4)	392·8 (350·9 to 438·4)	754·9 (624·5 to 906·0)	1·2 (1·1 to 1·4)
Brazil*	2038	877·5 (797·8 to 958·7)	357·7 (309·3 to 409·8)	174·5 (156·0 to 194·8)	344·8 (282·6 to 415·8)	0·6 (0·5 to 0·6)	1981·2 (1801·4 to 2164·6)	807·6 (698·4 to 925·3)	394·0 (352·1 to 439·9)	778·4 (638·0 to 938·8)	1·2 (1·1 to 1·4)
Brazil*	2039	894·7 (812·1 to 980·6)	363·9 (314·2 to 419·5)	174·9 (156·4 to 195·4)	355·3 (289·3 to 430·6)	0·6 (0·5 to 0·7)	2020·1 (1833·5 to 2214·0)	821·7 (709·5 to 947·3)	395·0 (353·1 to 441·2)	802·1 (653·2 to 972·2)	1·3 (1·1 to 1·5)
Brazil*	2040	912·7 (824·7 to 1005·6)	370·5 (318·5 to 427·4)	175·4 (156·9 to 196·0)	366·1 (295·2 to 446·4)	0·6 (0·5 to 0·7)	2060·7 (1862·0 to 2270·4)	836·6 (719·2 to 965·0)	396·1 (354·2 to 442·6)	826·7 (666·4 to 1008·0)	1·4 (1·2 to 1·6)
Brazil*	2041	930·8 (837·7 to 1030·5)	377·1 (322·0 to 437·2)	175·8 (157·2 to 196·4)	377·3 (302·0 to 463·6)	0·6 (0·5 to 0·7)	2101·7 (1891·4 to 2326·8)	851·4 (726·9 to 987·1)	397·0 (355·0 to 443·4)	851·8 (681·9 to 1046·7)	1·4 (1·2 to 1·7)
Brazil*	2042	949·1 (850·1 to 1055·7)	383·6 (327·9 to 444·6)	176·2 (157·5 to 196·7)	388·6 (308·2 to 481·1)	0·7 (0·6 to 0·8)	2142·9 (1919·5 to 2383·6)	866·1 (740·3 to 1003·9)	397·8 (355·7 to 444·1)	877·5 (695·8 to 1086·4)	1·5 (1·3 to 1·8)

Brazil*	2043	967·7 (863·0 to 1079·8)	390·3 (333·6 to 452·6)	176·5 (157·8 to 197·0)	400·3 (313·6 to 499·6)	0·7 (0·6 to 0·8)	2184·9 (1948·4 to 2438·0)	881·1 (753·1 to 1022·0)	398·4 (356·2 to 444·7)	903·7 (708·0 to 1128·0)	1·6 (1·3 to 1·9)
Brazil*	2044	986·1 (874·9 to 1106·1)	396·8 (338·9 to 459·7)	176·6 (157·9 to 197·0)	412·0 (318·9 to 519·0)	0·7 (0·6 to 0·9)	2226·4 (1975·4 to 2497·3)	895·9 (765·2 to 1037·9)	398·7 (356·5 to 444·8)	930·2 (720·0 to 1171·9)	1·7 (1·4 to 2·0)
Brazil*	2045	1004·6 (886·8 to 1130·7)	403·4 (345·6 to 469·4)	176·6 (158·0 to 197·0)	423·9 (324·8 to 539·0)	0·8 (0·7 to 0·9)	2268·3 (2002·2 to 2552·9)	910·7 (780·4 to 1059·8)	398·8 (356·6 to 444·7)	957·1 (733·2 to 1217·0)	1·7 (1·5 to 2·1)
Brazil*	2046	1023·3 (898·9 to 1158·1)	409·9 (350·0 to 477·6)	176·6 (157·9 to 197·1)	436·0 (330·7 to 559·3)	0·8 (0·7 to 1·0)	2310·4 (2029·6 to 2614·9)	925·4 (790·2 to 1078·4)	398·8 (356·5 to 445·0)	984·3 (746·7 to 1262·9)	1·8 (1·6 to 2·2)
Brazil*	2047	1042·2 (912·0 to 1184·8)	416·3 (353·1 to 487·0)	176·7 (158·0 to 197·2)	448·4 (336·8 to 580·3)	0·9 (0·7 to 1·0)	2353·1 (2059·2 to 2675·0)	940·0 (797·1 to 1099·6)	398·9 (356·7 to 445·2)	1012·3 (760·5 to 1310·3)	1·9 (1·6 to 2·4)
Brazil*	2048	1060·5 (925·2 to 1212·2)	422·4 (359·4 to 492·6)	176·6 (158·0 to 197·1)	460·6 (342·6 to 601·7)	0·9 (0·8 to 1·1)	2394·5 (2088·8 to 2737·0)	953·6 (811·4 to 1112·2)	398·7 (356·6 to 445·0)	1040·1 (773·6 to 1358·6)	2·0 (1·7 to 2·5)
Brazil*	2049	1079·6 (936·9 to 1238·3)	428·7 (364·3 to 501·4)	176·6 (157·9 to 197·1)	473·4 (348·6 to 623·9)	1·0 (0·8 to 1·2)	2437·5 (2115·4 to 2795·9)	967·9 (822·6 to 1132·1)	398·7 (356·4 to 445·0)	1068·8 (787·0 to 1408·6)	2·2 (1·8 to 2·7)
Brazil*	2050	1098·6 (949·6 to 1265·7)	434·8 (369·2 to 507·5)	176·6 (157·7 to 197·0)	486·2 (355·3 to 645·1)	1·0 (0·8 to 1·3)	2480·4 (2144·0 to 2857·7)	981·6 (833·7 to 1145·9)	398·6 (356·1 to 444·9)	1097·8 (802·2 to 1456·4)	2·3 (1·9 to 2·8)
Barbados	1995	1046·3 (986·8 to 1111·1)	607·9 (555·6 to 662·5)	353·8 (327·5 to 382·2)	83·6 (68·7 to 100·8)	0·9 (0·9 to 0·9)	947·7 (893·9 to 1006·4)	550·7 (503·3 to 600·1)	320·4 (296·7 to 346·2)	75·7 (62·3 to 91·3)	0·8 (0·8 to 0·8)
Barbados	1996	1088·2 (1037·5 to 1146·4)	637·0 (590·8 to 684·6)	365·1 (342·5 to 388·4)	85·4 (73·3 to 100·4)	0·7 (0·7 to 0·7)	985·7 (939·8 to 1038·4)	577·0 (535·1 to 620·1)	330·7 (310·2 to 351·8)	77·4 (66·4 to 91·0)	0·6 (0·6 to 0·6)
Barbados	1997	1105·7 (1058·0 to 1160·5)	640·1 (596·9 to 685·4)	379·3 (357·8 to 402·0)	85·6 (74·4 to 99·2)	0·7 (0·7 to 0·7)	1001·6 (958·3 to 1051·2)	579·8 (540·7 to 620·8)	343·6 (324·1 to 364·1)	77·5 (67·4 to 89·8)	0·6 (0·6 to 0·6)
Barbados	1998	1072·9 (1023·9 to 1123·7)	605·3 (562·4 to 648·8)	382·2 (361·7 to 404·0)	84·8 (75·3 to 94·9)	0·7 (0·7 to 0·7)	971·8 (927·4 to 1017·9)	548·3 (509·5 to 587·7)	346·2 (327·6 to 366·0)	76·8 (68·2 to 85·9)	0·6 (0·6 to 0·6)
Barbados	1999	1053·8 (1007·0 to 1098·4)	582·6 (539·5 to 625·7)	385·5 (365·0 to 404·1)	85·0 (78·2 to 92·0)	0·7 (0·7 to 0·7)	954·5 (912·1 to 994·9)	527·7 (488·6 to 566·7)	349·2 (330·6 to 366·1)	77·0 (70·8 to 83·3)	0·6 (0·6 to 0·6)
Barbados	2000	1032·9 (988·6 to 1077·8)	562·2 (520·8 to 603·6)	385·9 (366·2 to 404·3)	84·9 (80·9 to 88·8)	0·0 (0·0 to 0·0)	935·6 (895·4 to 976·3)	509·2 (471·7 to 546·8)	349·5 (331·7 to 366·2)	76·9 (73·2 to 80·5)	0·0 (0·0 to 0·0)

Barbados	2001	1000·6 (958·4 to 1045·5)	541·6 (504·7 to 581·9)	377·3 (358·4 to 396·6)	80·3 (76·6 to 84·4)	1·4 (1·4 to 1·4)	906·3 (868·1 to 947·0)	490·5 (457·2 to 527·1)	341·7 (324·7 to 359·2)	72·8 (69·4 to 76·5)	1·2 (1·2 to 1·2)
Barbados	2002	1154·8 (1106·6 to 1204·3)	663·3 (621·5 to 709·5)	408·9 (390·6 to 428·7)	82·6 (78·9 to 86·8)	0·0 (0·0 to 0·0)	1046·0 (1002·4 to 1090·9)	600·8 (562·9 to 642·7)	370·4 (353·8 to 388·3)	74·8 (71·5 to 78·7)	0·0 (0·0 to 0·0)
Barbados	2003	1215·3 (1165·5 to 1264·5)	691·3 (648·0 to 736·5)	426·1 (405·6 to 447·1)	79·5 (74·1 to 85·4)	18·4 (18·4 to 18·4)	1100·8 (1055·7 to 1145·4)	626·1 (586·9 to 667·2)	386·0 (367·4 to 405·0)	72·0 (67·1 to 77·3)	16·6 (16·6 to 16·6)
Barbados	2004	1270·3 (1221·0 to 1317·0)	719·5 (677·0 to 764·5)	442·0 (422·7 to 461·9)	83·0 (76·2 to 90·2)	25·9 (25·9 to 25·9)	1150·6 (1105·9 to 1192·9)	651·7 (613·3 to 692·5)	400·3 (382·9 to 418·4)	75·2 (69·0 to 81·7)	23·4 (23·4 to 23·4)
Barbados	2005	1293·0 (1242·0 to 1341·6)	741·6 (697·0 to 784·9)	454·5 (435·2 to 475·5)	88·1 (82·3 to 94·5)	8·8 (8·8 to 8·8)	1171·2 (1125·0 to 1215·2)	671·7 (631·3 to 710·9)	411·7 (394·2 to 430·8)	79·8 (74·5 to 85·6)	8·0 (8·0 to 8·0)
Barbados	2006	1257·6 (1210·3 to 1305·8)	716·2 (672·2 to 759·1)	453·7 (436·2 to 473·8)	87·8 (84·1 to 91·7)	0·0 (0·0 to 0·0)	1139·2 (1096·2 to 1182·8)	648·7 (608·9 to 687·6)	410·9 (395·1 to 429·2)	79·5 (76·2 to 83·1)	0·0 (0·0 to 0·0)
Barbados	2007	1265·8 (1219·1 to 1313·7)	723·8 (678·4 to 767·8)	458·5 (440·1 to 476·0)	83·5 (80·2 to 86·6)	0·0 (0·0 to 0·0)	1146·6 (1104·2 to 1190·0)	655·6 (614·5 to 695·5)	415·3 (398·7 to 431·1)	75·7 (72·7 to 78·5)	0·0 (0·0 to 0·0)
Barbados	2008	1271·1 (1225·6 to 1317·2)	724·7 (681·5 to 771·1)	466·9 (449·0 to 484·8)	79·5 (76·4 to 82·3)	0·0 (0·0 to 0·0)	1151·4 (1110·1 to 1193·1)	656·5 (617·3 to 698·4)	423·0 (406·7 to 439·1)	72·0 (69·2 to 74·6)	0·0 (0·0 to 0·0)
Barbados	2009	1267·6 (1222·5 to 1316·2)	711·8 (670·0 to 754·2)	479·2 (460·0 to 497·5)	76·6 (73·5 to 79·4)	0·0 (0·0 to 0·0)	1148·2 (1107·4 to 1192·2)	644·8 (606·9 to 683·2)	434·0 (416·7 to 450·7)	69·4 (66·6 to 71·9)	0·0 (0·0 to 0·0)
Barbados	2010	1256·8 (1212·5 to 1304·7)	689·1 (647·9 to 734·9)	491·3 (474·0 to 508·2)	76·4 (73·5 to 79·2)	0·0 (0·0 to 0·0)	1138·4 (1098·3 to 1181·8)	624·2 (586·8 to 665·7)	445·0 (429·3 to 460·3)	69·2 (66·6 to 71·7)	0·0 (0·0 to 0·0)
Barbados	2011	1237·1 (1191·9 to 1282·9)	652·9 (611·2 to 695·3)	508·3 (490·9 to 525·1)	75·8 (73·0 to 78·7)	0·0 (0·0 to 0·0)	1120·5 (1079·6 to 1162·1)	591·4 (553·6 to 629·8)	460·4 (444·7 to 475·6)	68·7 (66·1 to 71·3)	0·0 (0·0 to 0·0)
Barbados	2012	1303·5 (1253·1 to 1351·6)	689·5 (644·1 to 734·3)	534·9 (516·5 to 553·9)	79·1 (76·2 to 82·0)	0·0 (0·0 to 0·0)	1180·7 (1135·0 to 1224·3)	624·6 (583·4 to 665·1)	484·5 (467·9 to 501·7)	71·6 (69·0 to 74·3)	0·0 (0·0 to 0·0)
Barbados	2013	1276·1 (1231·9 to 1321·4)	657·6 (615·9 to 701·6)	536·9 (519·7 to 556·2)	81·7 (78·8 to 84·9)	0·0 (0·0 to 0·0)	1155·9 (1115·9 to 1196·9)	595·6 (557·9 to 635·5)	486·3 (470·8 to 503·8)	74·0 (71·3 to 76·9)	0·0 (0·0 to 0·0)
Barbados	2014	1196·4 (1155·8 to 1237·8)	590·6 (553·1 to 631·3)	524·5 (507·1 to 541·4)	81·4 (78·4 to 84·3)	0·0 (0·0 to 0·0)	1083·7 (1046·9 to 1121·2)	534·9 (501·0 to 571·9)	475·0 (459·3 to 490·4)	73·8 (71·0 to 76·4)	0·0 (0·0 to 0·0)

Barbados	2015	1199·3 (1156·0 to 1245·9)	587·3 (547·4 to 631·4)	528·9 (510·3 to 548·3)	83·1 (80·1 to 86·0)	0·0 (0·0 to 0·0)	1086·3 (1047·1 to 1128·5)	532·0 (495·8 to 571·9)	479·1 (462·2 to 496·6)	75·2 (72·5 to 77·9)	0·0 (0·0 to 0·0)
Barbados	2016	1178·3 (1135·1 to 1225·4)	564·4 (526·4 to 605·3)	530·0 (511·8 to 549·5)	83·9 (81·1 to 87·0)	0·0 (0·0 to 0·0)	1067·3 (1028·1 to 1109·9)	511·2 (476·8 to 548·3)	480·1 (463·6 to 497·8)	76·0 (73·4 to 78·8)	0·0 (0·0 to 0·0)
Barbados	2017	1169·3 (1122·8 to 1216·7)	545·6 (501·2 to 588·0)	537·4 (518·5 to 556·8)	86·2 (83·3 to 89·3)	0·0 (0·0 to 0·0)	1059·1 (1017·0 to 1102·0)	494·2 (454·0 to 532·6)	486·8 (469·6 to 504·4)	78·1 (75·5 to 80·9)	0·0 (0·0 to 0·0)
Barbados	2018	1147·6 (1096·0 to 1202·9)	520·3 (471·5 to 571·1)	540·0 (516·1 to 564·6)	87·3 (83·6 to 91·1)	0·0 (0·0 to 0·0)	1039·5 (992·8 to 1089·6)	471·3 (427·1 to 517·3)	489·1 (467·5 to 511·5)	79·0 (75·7 to 82·6)	0·0 (0·0 to 0·0)
Barbados	2019	1126·2 (1073·8 to 1181·8)	498·6 (449·0 to 549·0)	538·8 (514·9 to 563·5)	88·9 (85·0 to 93·0)	0·0 (0·0 to 0·0)	1020·2 (972·6 to 1070·5)	451·6 (406·7 to 497·3)	488·0 (466·4 to 510·4)	80·5 (77·0 to 84·2)	0·0 (0·0 to 0·0)
Barbados	2020	1136·2 (1078·7 to 1198·4)	549·8 (494·5 to 609·7)	501·7 (479·4 to 524·8)	84·7 (80·8 to 88·5)	0·0 (0·0 to 0·0)	1029·1 (977·1 to 1085·5)	498·1 (447·9 to 552·3)	454·4 (434·2 to 475·4)	76·7 (73·2 to 80·1)	0·0 (0·0 to 0·0)
Barbados	2021	1096·6 (1041·2 to 1156·7)	529·9 (476·5 to 587·6)	482·8 (461·1 to 505·6)	83·8 (79·9 to 87·8)	0·0 (0·0 to 0·0)	993·3 (943·1 to 1047·7)	480·0 (431·6 to 532·2)	437·3 (417·7 to 458·0)	75·9 (72·4 to 79·5)	0·0 (0·0 to 0·0)
Barbados	2022	1032·3 (978·7 to 1091·6)	462·9 (411·8 to 518·6)	483·1 (461·1 to 505·7)	86·3 (81·9 to 90·7)	0·0 (0·0 to 0·0)	935·1 (886·5 to 988·8)	419·3 (373·0 to 469·8)	437·6 (417·7 to 458·1)	78·2 (74·2 to 82·2)	0·0 (0·0 to 0·0)
Barbados	2023	1041·9 (984·6 to 1105·7)	469·5 (411·3 to 531·5)	483·6 (461·9 to 506·6)	88·9 (83·6 to 93·9)	0·0 (0·0 to 0·0)	943·8 (891·8 to 1001·6)	425·3 (372·6 to 481·5)	438·0 (418·4 to 458·8)	80·5 (75·8 to 85·1)	0·0 (0·0 to 0·0)
Barbados	2024	1051·2 (990·5 to 1118·4)	476·1 (417·3 to 540·5)	483·7 (462·1 to 506·5)	91·4 (85·3 to 97·4)	0·0 (0·0 to 0·0)	952·1 (897·2 to 1013·0)	431·2 (378·0 to 489·6)	438·1 (418·6 to 458·7)	82·8 (77·3 to 88·2)	0·0 (0·0 to 0·0)
Barbados	2025	1060·0 (995·7 to 1131·9)	482·4 (419·9 to 552·6)	483·7 (462·1 to 506·3)	93·8 (87·4 to 100·7)	0·0 (0·0 to 0·0)	960·1 (901·9 to 1025·3)	437·0 (380·4 to 500·5)	438·2 (418·5 to 458·6)	85·0 (79·2 to 91·2)	0·0 (0·0 to 0·0)
Barbados	2026	1068·0 (1001·3 to 1142·0)	488·0 (423·5 to 559·7)	483·8 (462·0 to 506·4)	96·2 (89·4 to 103·6)	0·0 (0·0 to 0·0)	967·4 (907·0 to 1034·4)	442·1 (383·6 to 507·0)	438·2 (418·5 to 458·7)	87·1 (81·0 to 93·8)	0·0 (0·0 to 0·0)
Barbados	2027	1073·8 (1004·4 to 1153·0)	492·0 (423·6 to 568·5)	483·4 (461·4 to 505·9)	98·4 (91·3 to 105·8)	0·0 (0·0 to 0·0)	972·6 (909·8 to 1044·4)	445·6 (383·7 to 514·9)	437·8 (417·9 to 458·3)	89·2 (82·7 to 95·8)	0·0 (0·0 to 0·0)
Barbados	2028	1078·5 (1008·2 to 1160·0)	494·8 (424·2 to 575·3)	483·0 (461·6 to 506·0)	100·6 (93·2 to 108·4)	0·0 (0·0 to 0·0)	976·9 (913·2 to 1050·8)	448·2 (384·3 to 521·1)	437·5 (418·1 to 458·3)	91·1 (84·4 to 98·2)	0·0 (0·0 to 0·0)

Barbados	2029	1078·7 (1009·1 to 1160·6)	493·8 (422·8 to 574·1)	482·3 (460·7 to 505·4)	102·7 (94·9 to 110·2)	0·0 (0·0 to 0·0)	977·1 (914·0 to 1051·3)	447·3 (383·0 to 520·0)	436·9 (417·3 to 457·7)	93·0 (86·0 to 99·8)	0·0 (0·0 to 0·0)
Barbados	2030	1078·4 (1007·0 to 1161·7)	492·2 (420·9 to 574·6)	481·5 (459·9 to 504·7)	104·7 (96·7 to 112·6)	0·0 (0·0 to 0·0)	976·8 (912·2 to 1052·3)	445·8 (381·2 to 520·5)	436·2 (416·6 to 457·1)	94·9 (87·6 to 102·0)	0·0 (0·0 to 0·0)
Barbados	2031	1077·4 (1005·9 to 1161·2)	489·8 (418·8 to 573·2)	480·8 (459·1 to 503·7)	106·8 (98·7 to 115·1)	0·0 (0·0 to 0·0)	975·9 (911·1 to 1051·8)	443·7 (379·3 to 519·2)	435·5 (415·9 to 456·3)	96·8 (89·4 to 104·2)	0·0 (0·0 to 0·0)
Barbados	2032	1074·2 (1003·3 to 1156·8)	485·2 (413·8 to 566·9)	480·0 (457·9 to 503·2)	109·0 (100·2 to 117·4)	0·0 (0·0 to 0·0)	973·0 (908·8 to 1047·8)	439·5 (374·8 to 513·5)	434·8 (414·7 to 455·8)	98·7 (90·8 to 106·3)	0·0 (0·0 to 0·0)
Barbados	2033	1070·9 (999·2 to 1153·2)	480·7 (410·5 to 563·3)	479·1 (456·7 to 502·1)	111·1 (101·5 to 120·2)	0·0 (0·0 to 0·0)	970·0 (905·0 to 1044·6)	435·4 (371·8 to 510·3)	434·0 (413·7 to 454·8)	100·6 (91·9 to 108·9)	0·0 (0·0 to 0·0)
Barbados	2034	1067·6 (995·5 to 1152·1)	476·0 (405·9 to 558·1)	478·3 (456·0 to 500·8)	113·3 (103·2 to 123·4)	0·0 (0·0 to 0·0)	967·0 (901·7 to 1043·6)	431·1 (367·7 to 505·5)	433·2 (413·0 to 453·6)	102·7 (93·5 to 111·8)	0·0 (0·0 to 0·0)
Barbados	2035	1064·6 (994·4 to 1150·6)	471·6 (401·1 to 553·7)	477·4 (455·1 to 499·8)	115·6 (104·8 to 126·7)	0·0 (0·0 to 0·0)	964·3 (900·7 to 1042·2)	427·1 (363·4 to 501·5)	432·5 (412·2 to 452·7)	104·7 (94·9 to 114·8)	0·0 (0·0 to 0·0)
Barbados	2036	1062·1 (991·7 to 1148·1)	467·2 (397·8 to 550·0)	476·9 (454·4 to 499·4)	118·0 (106·7 to 130·0)	0·0 (0·0 to 0·0)	962·1 (898·3 to 1039·9)	423·2 (360·3 to 498·2)	431·9 (411·6 to 452·3)	106·9 (96·6 to 117·8)	0·0 (0·0 to 0·0)
Barbados	2037	1060·9 (989·0 to 1148·1)	464·0 (395·4 to 546·7)	476·4 (453·9 to 499·0)	120·5 (108·1 to 133·7)	0·0 (0·0 to 0·0)	960·9 (895·9 to 1040·0)	420·2 (358·2 to 495·2)	431·5 (411·1 to 451·9)	109·2 (98·0 to 121·1)	0·0 (0·0 to 0·0)
Barbados	2038	1059·7 (987·6 to 1148·5)	460·5 (391·6 to 542·7)	476·0 (453·5 to 498·9)	123·1 (109·9 to 137·5)	0·0 (0·0 to 0·0)	959·8 (894·6 to 1040·3)	417·1 (354·7 to 491·6)	431·2 (410·8 to 451·9)	111·5 (99·6 to 124·5)	0·0 (0·0 to 0·0)
Barbados	2039	1059·2 (987·9 to 1148·7)	457·5 (387·8 to 540·5)	475·8 (453·4 to 498·5)	125·9 (111·9 to 141·3)	0·0 (0·0 to 0·0)	959·4 (894·9 to 1040·5)	414·4 (351·2 to 489·6)	431·0 (410·7 to 451·6)	114·0 (101·4 to 128·0)	0·0 (0·0 to 0·0)
Barbados	2040	1060·1 (988·1 to 1150·7)	455·4 (384·6 to 539·1)	476·0 (453·2 to 498·7)	128·7 (113·6 to 145·7)	0·0 (0·0 to 0·0)	960·2 (895·0 to 1042·3)	412·5 (348·4 to 488·3)	431·1 (410·5 to 451·8)	116·6 (102·9 to 132·0)	0·0 (0·0 to 0·0)
Barbados	2041	1061·4 (988·5 to 1152·1)	453·5 (382·7 to 536·6)	476·2 (453·2 to 499·2)	131·7 (115·2 to 149·7)	0·0 (0·0 to 0·0)	961·4 (895·4 to 1043·6)	410·8 (346·6 to 486·1)	431·3 (410·5 to 452·2)	119·3 (104·3 to 135·6)	0·0 (0·0 to 0·0)
Barbados	2042	1063·5 (989·5 to 1153·5)	452·1 (381·3 to 536·0)	476·6 (453·7 to 499·9)	134·8 (117·1 to 154·6)	0·0 (0·0 to 0·0)	963·3 (896·3 to 1044·8)	409·5 (345·4 to 485·5)	431·7 (411·0 to 452·8)	122·1 (106·0 to 140·0)	0·0 (0·0 to 0·0)

Barbados	2043	1066·3 (991·6 to 1157·2)	451·1 (380·5 to 535·1)	477·2 (454·3 to 500·3)	138·0 (119·1 to 159·0)	0·0 (0·0 to 0·0)	965·9 (898·2 to 1048·2)	408·6 (344·7 to 484·7)	432·2 (411·5 to 453·2)	125·0 (107·9 to 144·0)	0·0 (0·0 to 0·0)
Barbados	2044	1070·0 (994·9 to 1162·9)	450·7 (379·4 to 534·2)	477·9 (455·1 to 501·1)	141·4 (121·1 to 163·6)	0·0 (0·0 to 0·0)	969·2 (901·2 to 1053·3)	408·2 (343·7 to 483·8)	432·9 (412·2 to 453·9)	128·1 (109·7 to 148·2)	0·0 (0·0 to 0·0)
Barbados	2045	1073·9 (997·3 to 1169·0)	450·6 (378·9 to 535·5)	478·5 (455·7 to 501·9)	144·8 (123·4 to 168·5)	0·0 (0·0 to 0·0)	972·7 (903·3 to 1058·9)	408·1 (343·2 to 485·1)	433·4 (412·7 to 454·6)	131·2 (111·7 to 152·6)	0·0 (0·0 to 0·0)
Barbados	2046	1078·9 (1002·7 to 1175·3)	451·4 (377·9 to 536·5)	479·2 (455·8 to 502·8)	148·3 (125·4 to 173·6)	0·0 (0·0 to 0·0)	977·2 (908·2 to 1064·6)	408·8 (342·3 to 486·0)	434·0 (412·9 to 455·4)	134·4 (113·6 to 157·3)	0·0 (0·0 to 0·0)
Barbados	2047	1084·5 (1007·0 to 1182·4)	452·4 (378·0 to 537·9)	480·1 (456·8 to 503·9)	152·0 (127·5 to 179·0)	0·0 (0·0 to 0·0)	982·3 (912·2 to 1071·0)	409·8 (342·4 to 487·2)	434·8 (413·8 to 456·5)	137·7 (115·5 to 162·1)	0·0 (0·0 to 0·0)
Barbados	2048	1090·9 (1011·9 to 1190·0)	453·8 (380·5 to 540·6)	481·2 (458·0 to 505·2)	155·9 (129·4 to 184·9)	0·0 (0·0 to 0·0)	988·1 (916·6 to 1077·9)	411·1 (344·7 to 489·7)	435·8 (414·8 to 457·6)	141·2 (117·2 to 167·5)	0·0 (0·0 to 0·0)
Barbados	2049	1096·9 (1016·7 to 1195·7)	454·9 (380·7 to 542·8)	482·1 (458·7 to 506·4)	159·8 (131·5 to 191·5)	0·0 (0·0 to 0·0)	993·5 (921·0 to 1083·1)	412·1 (344·8 to 491·7)	436·7 (415·5 to 458·7)	144·8 (119·2 to 173·4)	0·0 (0·0 to 0·0)
Barbados	2050	1103·6 (1023·1 to 1204·6)	456·8 (381·1 to 545·3)	483·0 (459·4 to 507·6)	163·8 (133·6 to 197·6)	0·0 (0·0 to 0·0)	999·6 (926·7 to 1091·1)	413·8 (345·2 to 494·0)	437·5 (416·2 to 459·8)	148·4 (121·0 to 179·0)	0·0 (0·0 to 0·0)
Brunei	1995	816·8 (766·9 to 871·6)	634·7 (587·3 to 682·2)	181·9 (162·5 to 202·0)	0·1 (0·0 to 0·3)	0·0 (0·0 to 0·0)	2184·7 (2051·3 to 2331·3)	1697·7 (1570·8 to 1824·8)	486·6 (434·5 to 540·2)	0·4 (0·0 to 0·8)	0·0 (0·0 to 0·0)
Brunei	1996	851·1 (810·6 to 893·6)	669·8 (631·3 to 708·5)	181·2 (165·5 to 198·6)	0·1 (0·0 to 0·3)	0·0 (0·0 to 0·0)	2276·7 (2168·2 to 2390·2)	1791·6 (1688·6 to 1895·1)	484·6 (442·8 to 531·1)	0·4 (0·0 to 0·8)	0·0 (0·0 to 0·0)
Brunei	1997	874·8 (838·1 to 912·3)	699·9 (666·1 to 735·5)	174·8 (161·0 to 190·0)	0·1 (0·0 to 0·3)	0·0 (0·0 to 0·0)	2340·1 (2241·8 to 2440·2)	1872·2 (1781·6 to 1967·2)	467·5 (430·5 to 508·2)	0·4 (0·0 to 0·7)	0·0 (0·0 to 0·0)
Brunei	1998	920·1 (880·1 to 956·7)	754·0 (717·4 to 789·8)	165·9 (152·7 to 179·7)	0·1 (0·0 to 0·3)	0·0 (0·0 to 0·0)	2461·0 (2354·1 to 2559·0)	2016·8 (1919·0 to 2112·6)	443·9 (408·5 to 480·7)	0·3 (0·0 to 0·7)	0·0 (0·0 to 0·0)
Brunei	1999	863·6 (822·9 to 902·3)	717·1 (678·3 to 755·6)	146·3 (135·0 to 157·4)	0·1 (0·0 to 0·3)	0·0 (0·0 to 0·0)	2310·0 (2201·2 to 2413·4)	1918·2 (1814·4 to 2021·1)	391·4 (361·1 to 421·1)	0·3 (0·0 to 0·7)	0·0 (0·0 to 0·0)
Brunei	2000	748·2 (713·8 to 784·2)	623·3 (589·9 to 659·2)	124·7 (116·1 to 133·0)	0·1 (0·0 to 0·3)	0·0 (0·0 to 0·0)	2001·3 (1909·3 to 2097·5)	1667·3 (1577·8 to 1763·2)	333·6 (310·6 to 355·8)	0·4 (0·0 to 0·7)	0·0 (0·0 to 0·0)

Brunei	2001	707·6 (674·0 to 740·2)	588·6 (556·4 to 621·0)	118·9 (111·3 to 127·0)	0·2 (0·0 to 0·3)	0·0 (0·0 to 0·0)	1892·6 (1802·9 to 1979·9)	1574·3 (1488·1 to 1661·1)	317·9 (297·6 to 339·6)	0·4 (0·0 to 0·7)	0·0 (0·0 to 0·0)
Brunei	2002	766·5 (730·9 to 803·6)	643·3 (607·6 to 679·4)	123·1 (115·1 to 130·6)	0·1 (0·0 to 0·3)	0·0 (0·0 to 0·0)	2050·2 (1955·0 to 2149·4)	1720·7 (1625·1 to 1817·2)	329·2 (308·0 to 349·2)	0·3 (0·0 to 0·8)	0·0 (0·0 to 0·0)
Brunei	2003	676·5 (644·7 to 708·2)	558·7 (528·4 to 589·3)	117·7 (110·3 to 125·6)	0·2 (0·0 to 0·3)	0·0 (0·0 to 0·0)	1809·6 (1724·5 to 1894·4)	1494·4 (1413·3 to 1576·2)	314·7 (295·0 to 335·8)	0·4 (0·0 to 0·8)	0·0 (0·0 to 0·0)
Brunei	2004	690·2 (657·8 to 723·5)	575·3 (543·2 to 606·2)	114·8 (107·4 to 122·3)	0·1 (0·0 to 0·3)	0·0 (0·0 to 0·0)	1846·2 (1759·4 to 1935·2)	1538·8 (1453·1 to 1621·6)	307·0 (287·3 to 327·1)	0·4 (0·0 to 0·7)	0·0 (0·0 to 0·0)
Brunei	2005	617·2 (586·9 to 650·3)	517·8 (489·3 to 550·1)	99·2 (92·0 to 107·0)	0·2 (0·0 to 0·3)	0·0 (0·0 to 0·0)	1650·8 (1569·9 to 1739·4)	1385·0 (1308·8 to 1471·4)	265·4 (246·0 to 286·2)	0·4 (0·0 to 0·7)	0·0 (0·0 to 0·0)
Brunei	2006	580·4 (551·1 to 611·3)	496·0 (466·8 to 527·3)	84·2 (77·5 to 91·8)	0·2 (0·0 to 0·3)	0·0 (0·0 to 0·0)	1552·5 (1474·1 to 1635·0)	1326·7 (1248·6 to 1410·5)	225·3 (207·3 to 245·5)	0·4 (0·0 to 0·8)	0·0 (0·0 to 0·0)
Brunei	2007	566·6 (537·0 to 597·4)	494·8 (466·0 to 524·0)	71·7 (65·4 to 78·7)	0·1 (0·0 to 0·3)	0·0 (0·0 to 0·0)	1515·7 (1436·3 to 1597·8)	1323·4 (1246·5 to 1401·6)	191·9 (175·0 to 210·5)	0·4 (0·0 to 0·8)	0·0 (0·0 to 0·0)
Brunei	2008	539·1 (512·1 to 569·5)	478·7 (451·9 to 508·1)	60·3 (54·3 to 66·9)	0·1 (0·0 to 0·3)	0·0 (0·0 to 0·0)	1442·1 (1369·7 to 1523·4)	1280·5 (1208·6 to 1359·2)	161·2 (145·3 to 178·8)	0·4 (0·0 to 0·7)	0·0 (0·0 to 0·0)
Brunei	2009	583·2 (552·9 to 613·7)	526·8 (497·9 to 556·1)	56·3 (50·5 to 62·6)	0·1 (0·0 to 0·3)	0·0 (0·0 to 0·0)	1560·0 (1479·0 to 1641·4)	1409·1 (1331·9 to 1487·3)	150·6 (135·1 to 167·5)	0·3 (0·0 to 0·7)	0·0 (0·0 to 0·0)
Brunei	2010	582·2 (551·7 to 615·2)	531·1 (502·2 to 562·0)	50·9 (45·6 to 57·4)	0·1 (0·0 to 0·3)	0·0 (0·0 to 0·0)	1557·2 (1475·7 to 1645·6)	1420·7 (1343·2 to 1503·1)	136·2 (122·1 to 153·4)	0·3 (0·0 to 0·7)	0·0 (0·0 to 0·0)
Brunei	2011	513·4 (485·8 to 542·4)	468·3 (441·4 to 495·7)	45·0 (39·8 to 50·8)	0·2 (0·0 to 0·3)	0·0 (0·0 to 0·0)	1373·4 (1299·4 to 1450·8)	1252·5 (1180·5 to 1326·0)	120·4 (106·6 to 136·0)	0·4 (0·0 to 0·7)	0·0 (0·0 to 0·0)
Brunei	2012	507·7 (478·5 to 538·2)	464·7 (435·5 to 495·0)	42·8 (37·8 to 48·5)	0·1 (0·0 to 0·3)	0·0 (0·0 to 0·0)	1358·0 (1280·0 to 1439·5)	1243·0 (1165·0 to 1324·0)	114·6 (101·1 to 129·7)	0·4 (0·0 to 0·7)	0·0 (0·0 to 0·0)
Brunei	2013	509·6 (480·1 to 538·4)	469·1 (440·0 to 498·0)	40·4 (35·5 to 45·9)	0·1 (0·0 to 0·3)	0·0 (0·0 to 0·0)	1363·1 (1284·1 to 1440·1)	1254·7 (1176·9 to 1332·0)	107·9 (94·9 to 122·8)	0·4 (0·0 to 0·7)	0·0 (0·0 to 0·0)
Brunei	2014	514·0 (484·3 to 545·1)	477·9 (449·9 to 508·2)	35·9 (31·4 to 41·1)	0·1 (0·0 to 0·3)	0·0 (0·0 to 0·0)	1374·8 (1295·3 to 1458·1)	1278·3 (1203·4 to 1359·3)	96·1 (84·0 to 109·8)	0·4 (0·0 to 0·7)	0·0 (0·0 to 0·0)

Brunei	2015	564·3 (531·9 to 598·1)	531·1 (499·2 to 562·3)	33·1 (28·8 to 38·1)	0·1 (0·0 to 0·3)	0·0 (0·0 to 0·0)	1509·5 (1422·7 to 1599·9)	1420·5 (1335·2 to 1504·0)	88·6 (77·0 to 101·8)	0·3 (0·0 to 0·7)	0·0 (0·0 to 0·0)
Brunei	2016	576·8 (542·8 to 607·5)	545·5 (511·3 to 576·4)	31·2 (26·7 to 36·4)	0·1 (0·0 to 0·2)	0·0 (0·0 to 0·0)	1543·0 (1451·8 to 1624·8)	1459·2 (1367·6 to 1541·8)	83·5 (71·5 to 97·3)	0·3 (0·0 to 0·6)	0·0 (0·0 to 0·0)
Brunei	2017	569·3 (535·5 to 601·3)	539·4 (506·8 to 571·1)	29·8 (25·1 to 35·2)	0·1 (0·0 to 0·2)	0·0 (0·0 to 0·0)	1522·9 (1432·4 to 1608·5)	1442·9 (1355·6 to 1527·6)	79·7 (67·3 to 94·1)	0·3 (0·0 to 0·6)	0·0 (0·0 to 0·0)
Brunei	2018	553·4 (515·3 to 595·4)	525·1 (486·4 to 567·0)	28·3 (23·4 to 33·7)	0·1 (0·0 to 0·2)	0·0 (0·0 to 0·0)	1480·4 (1378·4 to 1592·5)	1404·5 (1301·1 to 1516·7)	75·6 (62·6 to 90·0)	0·3 (0·0 to 0·6)	0·0 (0·0 to 0·0)
Brunei	2019	543·7 (500·8 to 591·9)	515·1 (472·3 to 563·1)	28·5 (23·6 to 33·9)	0·1 (0·0 to 0·3)	0·0 (0·0 to 0·0)	1454·2 (1339·5 to 1583·2)	1377·7 (1263·3 to 1506·1)	76·2 (63·0 to 90·7)	0·3 (0·0 to 0·7)	0·0 (0·0 to 0·0)
Brunei	2020	651·6 (591·7 to 719·6)	624·3 (564·0 to 693·4)	27·2 (22·5 to 32·4)	0·1 (0·0 to 0·3)	0·0 (0·0 to 0·0)	1742·8 (1582·7 to 1924·8)	1669·8 (1508·6 to 1854·6)	72·7 (60·2 to 86·6)	0·3 (0·0 to 0·7)	0·0 (0·0 to 0·0)
Brunei	2021	635·0 (576·8 to 701·3)	608·4 (549·7 to 675·8)	26·5 (21·9 to 31·5)	0·1 (0·0 to 0·3)	0·0 (0·0 to 0·0)	1698·5 (1542·7 to 1875·8)	1627·4 (1470·4 to 1807·6)	70·8 (58·6 to 84·2)	0·3 (0·0 to 0·7)	0·0 (0·0 to 0·0)
Brunei	2022	504·0 (448·6 to 570·0)	477·4 (421·9 to 543·4)	26·5 (21·9 to 31·5)	0·1 (0·0 to 0·3)	0·0 (0·0 to 0·0)	1348·1 (1199·8 to 1524·7)	1277·0 (1128·4 to 1453·6)	70·8 (58·5 to 84·2)	0·4 (0·0 to 0·7)	0·0 (0·0 to 0·0)
Brunei	2023	505·4 (445·1 to 573·5)	478·9 (418·0 to 547·0)	26·4 (21·8 to 31·4)	0·1 (0·0 to 0·3)	0·0 (0·0 to 0·0)	1351·8 (1190·7 to 1534·1)	1280·9 (1118·0 to 1463·3)	70·6 (58·3 to 83·9)	0·4 (0·0 to 0·8)	0·0 (0·0 to 0·0)
Brunei	2024	505·8 (442·6 to 579·9)	479·4 (417·0 to 552·5)	26·3 (21·7 to 31·2)	0·1 (0·0 to 0·3)	0·0 (0·0 to 0·0)	1353·0 (1183·8 to 1551·0)	1282·3 (1115·3 to 1477·9)	70·3 (58·1 to 83·6)	0·4 (0·0 to 0·8)	0·0 (0·0 to 0·0)
Brunei	2025	506·3 (440·8 to 581·3)	480·0 (413·8 to 554·3)	26·2 (21·6 to 31·1)	0·2 (0·0 to 0·3)	0·0 (0·0 to 0·0)	1354·3 (1179·1 to 1554·9)	1283·9 (1106·7 to 1482·6)	70·0 (57·8 to 83·2)	0·4 (0·1 to 0·8)	0·0 (0·0 to 0·0)
Brunei	2026	505·5 (436·6 to 586·3)	479·3 (411·2 to 557·2)	26·0 (21·5 to 30·9)	0·2 (0·0 to 0·3)	0·0 (0·0 to 0·0)	1352·2 (1167·8 to 1568·2)	1282·1 (1100·0 to 1490·3)	69·6 (57·6 to 82·8)	0·4 (0·1 to 0·9)	0·0 (0·0 to 0·0)
Brunei	2027	504·1 (432·2 to 584·2)	478·0 (407·6 to 556·1)	25·9 (21·4 to 30·8)	0·2 (0·0 to 0·3)	0·0 (0·0 to 0·0)	1348·4 (1156·1 to 1562·5)	1278·7 (1090·2 to 1487·5)	69·3 (57·3 to 82·3)	0·5 (0·1 to 0·9)	0·0 (0·0 to 0·0)
Brunei	2028	503·7 (428·8 to 588·3)	477·7 (403·3 to 563·9)	25·7 (21·3 to 30·6)	0·2 (0·0 to 0·4)	0·0 (0·0 to 0·0)	1347·2 (1147·0 to 1573·7)	1277·9 (1078·7 to 1508·3)	68·9 (56·9 to 81·8)	0·5 (0·1 to 1·0)	0·0 (0·0 to 0·0)

Brunei	2029	503·0 (424·0 to 589·8)	477·2 (398·4 to 564·1)	25·6 (21·1 to 30·4)	0·2 (0·0 to 0·4)	0·0 (0·0 to 0·0)	1345·4 (1134·1 to 1577·6)	1276·5 (1065·5 to 1509·0)	68·4 (56·5 to 81·3)	0·5 (0·1 to 1·0)	0·0 (0·0 to 0·0)
Brunei	2030	503·0 (418·8 to 595·3)	477·4 (394·8 to 570·5)	25·4 (21·0 to 30·2)	0·2 (0·0 to 0·4)	0·0 (0·0 to 0·0)	1345·4 (1120·2 to 1592·3)	1276·9 (1056·0 to 1526·1)	68·0 (56·2 to 80·8)	0·5 (0·1 to 1·0)	0·0 (0·0 to 0·0)
Brunei	2031	502·4 (416·6 to 598·6)	476·9 (390·9 to 573·4)	25·3 (20·9 to 30·0)	0·2 (0·0 to 0·4)	0·0 (0·0 to 0·0)	1343·9 (1114·3 to 1601·2)	1275·7 (1045·5 to 1533·7)	67·6 (55·9 to 80·3)	0·5 (0·1 to 1·1)	0·0 (0·0 to 0·0)
Brunei	2032	502·5 (410·4 to 598·3)	477·2 (385·0 to 574·3)	25·1 (20·8 to 29·9)	0·2 (0·0 to 0·4)	0·0 (0·0 to 0·0)	1344·1 (1097·8 to 1600·3)	1276·3 (1029·8 to 1536·1)	67·2 (55·5 to 79·8)	0·6 (0·1 to 1·1)	0·0 (0·0 to 0·0)
Brunei	2033	501·9 (407·1 to 607·5)	476·7 (380·5 to 583·1)	25·0 (20·6 to 29·7)	0·2 (0·0 to 0·4)	0·0 (0·0 to 0·0)	1342·4 (1089·0 to 1624·9)	1275·0 (1017·7 to 1559·8)	66·8 (55·2 to 79·4)	0·6 (0·1 to 1·2)	0·0 (0·0 to 0·0)
Brunei	2034	500·9 (403·5 to 605·0)	475·9 (378·3 to 581·7)	24·8 (20·5 to 29·5)	0·2 (0·0 to 0·4)	0·0 (0·0 to 0·0)	1339·8 (1079·3 to 1618·3)	1272·8 (1011·9 to 1555·9)	66·4 (54·8 to 78·8)	0·6 (0·1 to 1·2)	0·0 (0·0 to 0·0)
Brunei	2035	500·4 (399·7 to 610·1)	475·5 (374·8 to 585·0)	24·7 (20·3 to 29·3)	0·2 (0·1 to 0·5)	0·0 (0·0 to 0·0)	1338·5 (1069·0 to 1632·0)	1271·9 (1002·6 to 1564·7)	65·9 (54·4 to 78·3)	0·6 (0·1 to 1·2)	0·0 (0·0 to 0·0)
Brunei	2036	499·7 (398·1 to 611·1)	474·9 (373·5 to 586·4)	24·5 (20·2 to 29·1)	0·2 (0·1 to 0·5)	0·0 (0·0 to 0·0)	1336·5 (1065·0 to 1634·5)	1270·3 (999·0 to 1568·6)	65·5 (54·0 to 77·8)	0·6 (0·1 to 1·3)	0·0 (0·0 to 0·0)
Brunei	2037	499·6 (397·6 to 611·7)	475·0 (374·0 to 587·0)	24·3 (20·0 to 28·9)	0·2 (0·1 to 0·5)	0·0 (0·0 to 0·0)	1336·4 (1063·5 to 1636·2)	1270·6 (1000·4 to 1570·2)	65·1 (53·6 to 77·3)	0·7 (0·2 to 1·3)	0·0 (0·0 to 0·0)
Brunei	2038	499·6 (395·3 to 614·8)	475·1 (370·8 to 591·7)	24·2 (19·9 to 28·8)	0·3 (0·1 to 0·5)	0·0 (0·0 to 0·0)	1336·3 (1057·2 to 1644·4)	1270·8 (991·8 to 1582·6)	64·8 (53·3 to 76·9)	0·7 (0·2 to 1·4)	0·0 (0·0 to 0·0)
Brunei	2039	500·0 (397·2 to 620·9)	475·6 (373·4 to 597·6)	24·1 (19·8 to 28·6)	0·3 (0·1 to 0·5)	0·0 (0·0 to 0·0)	1337·4 (1062·5 to 1660·9)	1272·2 (998·8 to 1598·4)	64·5 (53·1 to 76·5)	0·7 (0·2 to 1·4)	0·0 (0·0 to 0·0)
Brunei	2040	499·8 (396·1 to 616·1)	475·5 (370·9 to 591·1)	24·0 (19·7 to 28·4)	0·3 (0·1 to 0·6)	0·0 (0·0 to 0·0)	1336·8 (1059·4 to 1647·9)	1271·8 (992·1 to 1581·1)	64·2 (52·8 to 76·1)	0·7 (0·2 to 1·5)	0·0 (0·0 to 0·0)
Brunei	2041	499·2 (392·6 to 612·0)	475·0 (368·9 to 588·7)	23·9 (19·6 to 28·3)	0·3 (0·1 to 0·6)	0·0 (0·0 to 0·0)	1335·1 (1050·0 to 1637·1)	1270·6 (986·6 to 1574·5)	63·8 (52·5 to 75·6)	0·8 (0·2 to 1·5)	0·0 (0·0 to 0·0)
Brunei	2042	498·4 (391·8 to 621·1)	474·4 (366·2 to 596·7)	23·7 (19·5 to 28·1)	0·3 (0·1 to 0·6)	0·0 (0·0 to 0·0)	1333·2 (1048·1 to 1661·3)	1269·0 (979·6 to 1596·0)	63·4 (52·2 to 75·2)	0·8 (0·2 to 1·6)	0·0 (0·0 to 0·0)

Brunei	2043	497·8 (391·3 to 618·7)	474·0 (367·7 to 593·6)	23·6 (19·4 to 27·9)	0·3 (0·1 to 0·6)	0·0 (0·0 to 0·0)	1331·6 (1046·7 to 1654·9)	1267·8 (983·6 to 1587·8)	63·0 (51·8 to 74·7)	0·8 (0·2 to 1·6)	0·0 (0·0 to 0·0)
Brunei	2044	497·6 (388·2 to 617·9)	473·9 (365·2 to 595·0)	23·4 (19·3 to 27·8)	0·3 (0·1 to 0·6)	0·0 (0·0 to 0·0)	1330·9 (1038·4 to 1652·8)	1267·5 (976·9 to 1591·6)	62·6 (51·5 to 74·3)	0·8 (0·2 to 1·7)	0·0 (0·0 to 0·0)
Brunei	2045	496·8 (387·5 to 622·3)	473·2 (364·3 to 599·7)	23·2 (19·1 to 27·6)	0·3 (0·1 to 0·6)	0·0 (0·0 to 0·0)	1328·8 (1036·5 to 1664·6)	1265·8 (974·5 to 1604·2)	62·2 (51·1 to 73·8)	0·9 (0·2 to 1·7)	0·0 (0·0 to 0·0)
Brunei	2046	495·3 (385·3 to 630·9)	471·9 (363·2 to 607·2)	23·1 (19·0 to 27·4)	0·3 (0·1 to 0·7)	0·0 (0·0 to 0·0)	1324·7 (1030·7 to 1687·5)	1262·2 (971·6 to 1624·2)	61·7 (50·7 to 73·2)	0·9 (0·2 to 1·8)	0·0 (0·0 to 0·0)
Brunei	2047	494·3 (382·8 to 627·9)	471·1 (360·7 to 605·5)	22·9 (18·8 to 27·1)	0·3 (0·1 to 0·7)	0·0 (0·0 to 0·0)	1322·1 (1023·8 to 1679·4)	1260·1 (964·7 to 1619·6)	61·2 (50·3 to 72·6)	0·9 (0·2 to 1·8)	0·0 (0·0 to 0·0)
Brunei	2048	493·3 (379·2 to 628·8)	470·3 (355·2 to 605·5)	22·7 (18·6 to 26·9)	0·3 (0·1 to 0·7)	0·0 (0·0 to 0·0)	1319·5 (1014·4 to 1682·0)	1257·9 (950·0 to 1619·6)	60·7 (49·9 to 72·0)	0·9 (0·3 to 1·9)	0·0 (0·0 to 0·0)
Brunei	2049	491·4 (371·8 to 623·5)	468·5 (349·1 to 602·0)	22·5 (18·5 to 26·7)	0·4 (0·1 to 0·7)	0·0 (0·0 to 0·0)	1314·3 (994·5 to 1667·6)	1253·2 (933·8 to 1610·2)	60·1 (49·4 to 71·4)	1·0 (0·3 to 1·9)	0·0 (0·0 to 0·0)
Brunei	2050	489·6 (374·4 to 620·2)	467·0 (352·3 to 598·9)	22·3 (18·3 to 26·5)	0·4 (0·1 to 0·7)	0·0 (0·0 to 0·0)	1309·7 (1001·5 to 1659·0)	1249·1 (942·3 to 1602·0)	59·6 (49·0 to 70·8)	1·0 (0·3 to 2·0)	0·0 (0·0 to 0·0)
Bhutan*	1995	48·6 (41·6 to 56·5)	37·8 (31·0 to 45·2)	8·7 (6·8 to 10·9)	0·4 (0·3 to 0·6)	1·6 (1·6 to 1·6)	170·6 (146·1 to 198·4)	132·9 (109·0 to 159·0)	30·6 (23·8 to 38·2)	1·5 (1·0 to 2·2)	5·5 (5·5 to 5·5)
Bhutan*	1996	51·0 (43·9 to 58·7)	40·7 (34·0 to 48·1)	9·4 (7·3 to 11·9)	0·5 (0·3 to 0·7)	0·4 (0·4 to 0·4)	179·3 (154·2 to 206·4)	143·1 (119·3 to 168·8)	33·2 (25·7 to 41·8)	1·6 (1·1 to 2·3)	1·4 (1·4 to 1·4)
Bhutan*	1997	72·8 (65·8 to 80·5)	41·5 (35·0 to 49·1)	10·2 (8·0 to 12·7)	0·5 (0·3 to 0·6)	20·7 (20·7 to 20·7)	255·8 (231·0 to 283·0)	146·0 (122·8 to 172·6)	35·7 (28·0 to 44·7)	1·6 (1·1 to 2·3)	72·6 (72·6 to 72·6)
Bhutan*	1998	60·8 (54·1 to 68·0)	38·6 (32·5 to 45·5)	9·8 (7·8 to 12·2)	0·4 (0·3 to 0·6)	11·9 (11·9 to 11·9)	213·5 (189·9 to 238·8)	135·5 (114·3 to 159·8)	34·6 (27·4 to 43·0)	1·5 (1·0 to 2·1)	41·9 (41·9 to 41·9)
Bhutan*	1999	52·7 (45·6 to 60·1)	41·6 (34·9 to 48·5)	9·4 (7·4 to 11·8)	0·4 (0·3 to 0·6)	1·3 (1·3 to 1·3)	185·2 (160·3 to 211·2)	146·2 (122·7 to 170·2)	32·9 (26·0 to 41·3)	1·5 (1·0 to 2·2)	4·5 (4·5 to 4·5)
Bhutan*	2000	67·2 (60·3 to 74·3)	41·5 (34·8 to 48·7)	7·6 (5·9 to 9·6)	0·4 (0·3 to 0·6)	17·6 (17·6 to 17·6)	236·0 (212·0 to 261·1)	145·8 (122·3 to 171·1)	26·7 (20·8 to 33·7)	1·5 (1·0 to 2·1)	62·0 (62·0 to 62·0)

Bhutan*	2001	57·1 (49·7 to 65·6)	45·1 (38·1 to 53·1)	7·2 (5·6 to 9·0)	0·5 (0·3 to 0·7)	4·3 (4·3 to 4·3)	200·6 (174·7 to 230·6)	158·3 (133·9 to 186·4)	25·3 (19·7 to 31·7)	1·7 (1·2 to 2·4)	15·3 (15·3 to 15·3)
Bhutan*	2002	53·5 (46·8 to 61·4)	41·0 (34·6 to 48·4)	7·5 (5·8 to 9·5)	0·4 (0·2 to 0·5)	4·7 (4·7 to 4·7)	188·0 (164·5 to 215·6)	144·0 (121·7 to 170·0)	26·3 (20·4 to 33·3)	1·3 (0·9 to 1·8)	16·4 (16·4 to 16·4)
Bhutan*	2003	57·1 (50·6 to 64·8)	39·4 (32·8 to 46·3)	8·7 (6·8 to 10·9)	0·3 (0·2 to 0·3)	8·8 (8·8 to 8·8)	200·5 (177·7 to 227·6)	138·3 (115·4 to 162·8)	30·4 (23·8 to 38·3)	0·9 (0·6 to 1·2)	31·0 (31·0 to 31·0)
Bhutan*	2004	54·9 (48·6 to 62·5)	38·3 (32·6 to 45·3)	9·3 (7·4 to 11·7)	0·2 (0·1 to 0·3)	7·0 (7·0 to 7·0)	192·7 (170·8 to 219·5)	134·7 (114·6 to 159·0)	32·8 (26·0 to 41·1)	0·7 (0·5 to 1·0)	24·5 (24·5 to 24·5)
Bhutan*	2005	65·8 (59·1 to 74·0)	42·2 (35·8 to 50·3)	10·1 (8·1 to 12·7)	0·2 (0·1 to 0·3)	13·2 (13·2 to 13·2)	231·0 (207·6 to 260·1)	148·4 (125·7 to 176·7)	35·6 (28·3 to 44·5)	0·7 (0·5 to 0·9)	46·4 (46·4 to 46·4)
Bhutan*	2006	64·3 (57·4 to 72·3)	41·4 (35·0 to 49·0)	10·3 (8·2 to 12·8)	0·2 (0·1 to 0·2)	12·6 (12·6 to 12·6)	226·1 (201·6 to 253·9)	145·3 (122·9 to 172·2)	36·0 (28·8 to 45·0)	0·6 (0·4 to 0·8)	44·1 (44·1 to 44·1)
Bhutan*	2007	65·7 (58·0 to 74·0)	45·0 (38·3 to 53·1)	10·8 (8·6 to 13·4)	0·2 (0·1 to 0·2)	9·8 (9·8 to 9·8)	230·8 (203·7 to 260·0)	158·1 (134·6 to 186·5)	37·9 (30·2 to 47·1)	0·6 (0·4 to 0·8)	34·3 (34·3 to 34·3)
Bhutan*	2008	65·8 (57·8 to 74·3)	47·5 (40·4 to 55·6)	11·3 (8·9 to 13·9)	0·2 (0·2 to 0·3)	6·7 (6·7 to 6·7)	231·2 (203·0 to 261·0)	167·0 (142·1 to 195·3)	39·6 (31·3 to 48·9)	0·8 (0·6 to 1·2)	23·7 (23·7 to 23·7)
Bhutan*	2009	68·9 (60·7 to 77·6)	51·5 (44·1 to 59·9)	12·2 (9·6 to 15·1)	0·3 (0·2 to 0·4)	4·8 (4·8 to 4·8)	241·9 (213·2 to 272·6)	181·0 (155·0 to 210·5)	43·0 (33·8 to 53·1)	1·1 (0·7 to 1·5)	16·8 (16·8 to 16·8)
Bhutan*	2010	76·6 (67·9 to 86·6)	58·9 (50·9 to 68·3)	13·6 (10·8 to 16·8)	0·4 (0·3 to 0·5)	3·8 (3·8 to 3·8)	269·3 (238·4 to 304·4)	206·8 (178·7 to 239·9)	47·9 (38·1 to 59·0)	1·3 (0·9 to 1·8)	13·3 (13·3 to 13·3)
Bhutan*	2011	78·9 (70·4 to 88·0)	60·0 (52·3 to 68·9)	14·7 (11·9 to 18·1)	0·5 (0·3 to 0·7)	3·7 (3·7 to 3·7)	277·2 (247·2 to 309·3)	210·8 (183·8 to 241·9)	51·7 (41·7 to 63·5)	1·7 (1·1 to 2·3)	13·0 (13·0 to 13·0)
Bhutan*	2012	87·6 (79·1 to 96·4)	64·5 (56·8 to 72·9)	16·9 (13·8 to 20·6)	0·8 (0·5 to 1·1)	5·3 (5·3 to 5·3)	307·6 (277·7 to 338·7)	226·7 (199·4 to 256·0)	59·4 (48·6 to 72·3)	2·8 (1·9 to 3·8)	18·7 (18·7 to 18·7)
Bhutan*	2013	91·2 (83·4 to 99·6)	68·8 (61·7 to 76·4)	18·5 (15·6 to 22·0)	1·0 (0·7 to 1·3)	2·9 (2·9 to 2·9)	320·3 (293·1 to 349·9)	241·8 (216·7 to 268·6)	65·0 (54·6 to 77·4)	3·4 (2·3 to 4·6)	10·2 (10·2 to 10·2)
Bhutan*	2014	91·7 (85·1 to 98·5)	67·9 (62·3 to 73·7)	18·9 (16·2 to 21·9)	1·0 (0·7 to 1·4)	3·9 (3·9 to 3·9)	322·0 (299·1 to 346·0)	238·4 (218·9 to 258·9)	66·5 (56·9 to 77·1)	3·5 (2·5 to 4·8)	13·6 (13·6 to 13·6)

Bhutan*	2015	98·1 (93·0 to 103·6)	71·5 (66·8 to 76·4)	19·2 (16·7 to 21·8)	1·0 (0·7 to 1·4)	6·4 (6·4 to 6·4)	344·5 (326·6 to 364·0)	251·2 (234·6 to 268·4)	67·3 (58·8 to 76·6)	3·5 (2·5 to 4·8)	22·5 (22·5 to 22·5)
Bhutan*	2016	105·9 (100·0 to 112·5)	80·6 (75·2 to 86·5)	19·1 (16·9 to 21·6)	1·2 (0·8 to 1·6)	5·0 (5·0 to 5·0)	371·9 (351·4 to 395·2)	283·2 (264·1 to 303·7)	67·2 (59·2 to 75·8)	4·0 (2·8 to 5·6)	17·5 (17·5 to 17·5)
Bhutan*	2017	103·5 (96·0 to 111·6)	82·0 (74·8 to 89·9)	16·9 (14·8 to 19·4)	1·2 (0·8 to 1·6)	3·4 (3·4 to 3·4)	363·8 (337·3 to 392·2)	288·2 (262·9 to 315·8)	59·5 (51·9 to 68·3)	4·1 (2·9 to 5·6)	12·0 (12·0 to 12·0)
Bhutan*	2018	110·5 (100·9 to 121·6)	86·2 (76·5 to 96·5)	16·1 (13·7 to 18·7)	1·3 (0·9 to 1·7)	6·9 (6·9 to 6·9)	388·4 (354·4 to 427·1)	303·0 (268·9 to 339·2)	56·6 (48·3 to 65·6)	4·5 (3·1 to 6·1)	24·3 (24·3 to 24·3)
Bhutan*	2019	89·5 (81·9 to 98·1)	64·9 (57·6 to 72·7)	16·4 (14·0 to 19·0)	1·3 (0·9 to 1·8)	6·9 (6·8 to 6·9)	314·6 (287·7 to 344·7)	228·1 (202·4 to 255·4)	57·5 (49·1 to 66·6)	4·7 (3·2 to 6·4)	24·2 (24·1 to 24·4)
Bhutan*	2020	107·3 (98·1 to 117·8)	81·3 (72·2 to 91·2)	15·7 (13·4 to 18·2)	1·3 (0·9 to 1·8)	9·0 (8·9 to 9·1)	377·1 (344·7 to 413·7)	285·7 (253·5 to 320·6)	55·1 (47·0 to 63·8)	4·6 (3·2 to 6·3)	31·7 (31·4 to 31·9)
Bhutan*	2021	106·4 (97·1 to 116·9)	81·6 (72·4 to 91·5)	15·7 (13·4 to 18·2)	1·3 (0·9 to 1·8)	7·8 (7·7 to 8·0)	374·0 (341·2 to 410·6)	286·6 (254·3 to 321·6)	55·2 (47·0 to 63·9)	4·7 (3·3 to 6·5)	27·4 (26·9 to 28·0)
Bhutan*	2022	86·4 (78·8 to 94·8)	63·1 (56·0 to 71·1)	16·1 (13·7 to 18·6)	1·4 (1·0 to 1·9)	5·8 (5·4 to 6·1)	303·5 (276·8 to 333·2)	221·9 (196·9 to 249·8)	56·5 (48·1 to 65·4)	4·9 (3·4 to 6·8)	20·2 (19·1 to 21·4)
Bhutan*	2023	88·0 (80·2 to 96·7)	64·5 (57·2 to 73·0)	16·5 (14·0 to 19·1)	1·5 (1·0 to 2·0)	5·6 (5·2 to 6·0)	309·3 (281·8 to 339·9)	226·7 (200·8 to 256·4)	57·8 (49·3 to 67·0)	5·2 (3·6 to 7·1)	19·6 (18·4 to 20·9)
Bhutan*	2024	89·8 (81·7 to 98·9)	66·0 (58·3 to 74·5)	16·9 (14·4 to 19·5)	1·5 (1·1 to 2·1)	5·4 (5·1 to 5·8)	315·5 (287·2 to 347·5)	231·8 (205·0 to 261·9)	59·2 (50·4 to 68·6)	5·4 (3·8 to 7·5)	19·1 (17·8 to 20·5)
Bhutan*	2025	91·7 (83·2 to 101·1)	67·5 (59·6 to 76·2)	17·2 (14·7 to 20·0)	1·6 (1·1 to 2·2)	5·3 (4·9 to 5·7)	322·0 (292·4 to 355·1)	237·2 (209·2 to 267·7)	60·6 (51·6 to 70·2)	5·7 (3·9 to 7·8)	18·5 (17·2 to 20·1)
Bhutan*	2026	93·6 (85·1 to 103·1)	69·2 (60·8 to 78·1)	17·7 (15·0 to 20·5)	1·7 (1·2 to 2·4)	5·1 (4·7 to 5·6)	329·0 (299·1 to 362·3)	243·1 (213·7 to 274·5)	62·1 (52·8 to 71·9)	6·0 (4·1 to 8·3)	18·0 (16·5 to 19·6)
Bhutan*	2027	95·8 (87·0 to 105·8)	70·9 (62·6 to 80·2)	18·1 (15·4 to 21·0)	1·8 (1·2 to 2·5)	4·9 (4·5 to 5·4)	336·5 (305·6 to 371·8)	249·2 (220·0 to 281·6)	63·6 (54·2 to 73·7)	6·3 (4·3 to 8·7)	17·3 (15·9 to 19·0)
Bhutan*	2028	98·0 (88·6 to 109·1)	72·8 (63·6 to 83·3)	18·6 (15·8 to 21·6)	1·9 (1·3 to 2·6)	4·8 (4·3 to 5·3)	344·4 (311·1 to 383·1)	255·7 (223·5 to 292·6)	65·4 (55·7 to 75·7)	6·6 (4·6 to 9·1)	16·7 (15·2 to 18·5)

Bhutan*	2029	101·1 (90·7 to 113·3)	75·4 (65·0 to 87·4)	19·1 (16·3 to 22·1)	2·0 (1·4 to 2·7)	4·6 (4·2 to 5·1)	355·2 (318·5 to 398·1)	265·0 (228·4 to 306·9)	67·1 (57·2 to 77·8)	7·0 (4·8 to 9·6)	16·1 (14·6 to 17·9)
Bhutan*	2030	104·2 (92·8 to 117·3)	78·1 (66·9 to 90·5)	19·6 (16·7 to 22·7)	2·1 (1·5 to 2·9)	4·4 (4·0 to 5·0)	366·2 (326·2 to 412·2)	274·4 (235·2 to 317·8)	68·9 (58·8 to 79·9)	7·3 (5·1 to 10·2)	15·5 (13·9 to 17·4)
Bhutan*	2031	107·5 (95·0 to 121·7)	80·9 (68·7 to 94·8)	20·1 (17·1 to 23·3)	2·2 (1·5 to 3·0)	4·3 (3·8 to 4·8)	377·7 (333·7 to 427·6)	284·3 (241·5 to 333·2)	70·7 (60·2 to 81·9)	7·7 (5·4 to 10·6)	15·0 (13·4 to 16·8)
Bhutan*	2032	110·9 (97·6 to 126·4)	83·9 (70·8 to 98·7)	20·6 (17·5 to 23·9)	2·3 (1·6 to 3·2)	4·1 (3·7 to 4·7)	389·7 (343·0 to 444·1)	294·7 (248·8 to 346·8)	72·4 (61·6 to 83·9)	8·2 (5·7 to 11·3)	14·5 (12·8 to 16·4)
Bhutan*	2033	114·3 (100·4 to 131·1)	86·8 (72·3 to 102·9)	21·1 (18·0 to 24·5)	2·4 (1·7 to 3·4)	4·0 (3·5 to 4·6)	401·5 (352·8 to 460·5)	304·8 (254·1 to 361·4)	74·1 (63·1 to 86·0)	8·6 (6·0 to 11·9)	14·0 (12·4 to 16·0)
Bhutan*	2034	117·9 (103·0 to 135·4)	89·9 (74·5 to 106·7)	21·6 (18·4 to 25·0)	2·6 (1·8 to 3·6)	3·9 (3·4 to 4·5)	414·3 (361·7 to 475·8)	315·8 (261·6 to 374·9)	75·8 (64·6 to 87·9)	9·0 (6·3 to 12·6)	13·7 (12·1 to 15·7)
Bhutan*	2035	121·7 (105·1 to 141·1)	93·1 (76·2 to 112·4)	22·0 (18·8 to 25·6)	2·7 (1·9 to 3·8)	3·8 (3·3 to 4·4)	427·4 (369·2 to 495·8)	327·1 (267·7 to 394·7)	77·4 (66·1 to 89·9)	9·5 (6·6 to 13·3)	13·4 (11·8 to 15·4)
Bhutan*	2036	125·5 (108·2 to 145·5)	96·4 (78·8 to 115·9)	22·5 (19·2 to 26·1)	2·9 (2·0 to 4·0)	3·7 (3·3 to 4·3)	440·7 (380·2 to 511·4)	338·5 (276·9 to 407·1)	79·1 (67·5 to 91·8)	10·0 (6·9 to 14·0)	13·1 (11·5 to 15·2)
Bhutan*	2037	129·3 (111·1 to 151·2)	99·7 (81·2 to 121·4)	22·9 (19·6 to 26·6)	3·0 (2·1 to 4·2)	3·7 (3·2 to 4·3)	454·3 (390·2 to 531·2)	350·2 (285·1 to 426·6)	80·6 (68·9 to 93·5)	10·5 (7·2 to 14·7)	13·0 (11·2 to 15·0)
Bhutan*	2038	133·3 (113·6 to 156·0)	103·2 (83·3 to 125·7)	23·4 (19·9 to 27·1)	3·1 (2·2 to 4·4)	3·7 (3·2 to 4·3)	468·5 (399·2 to 548·1)	362·5 (292·6 to 441·5)	82·0 (70·1 to 95·1)	11·1 (7·6 to 15·5)	12·9 (11·1 to 15·0)
Bhutan*	2039	137·6 (116·4 to 162·6)	106·8 (85·0 to 131·7)	23·7 (20·3 to 27·5)	3·3 (2·2 to 4·6)	3·7 (3·2 to 4·3)	483·3 (409·1 to 571·2)	375·4 (298·5 to 462·8)	83·4 (71·2 to 96·7)	11·6 (7·9 to 16·3)	12·9 (11·1 to 15·2)
Bhutan*	2040	142·0 (119·7 to 169·1)	110·8 (88·0 to 137·9)	24·1 (20·6 to 27·9)	3·5 (2·3 to 4·9)	3·7 (3·2 to 4·4)	499·0 (420·5 to 594·1)	389·2 (309·2 to 484·6)	84·7 (72·3 to 98·2)	12·2 (8·2 to 17·3)	12·9 (11·1 to 15·3)
Bhutan*	2041	146·6 (123·1 to 174·7)	114·8 (90·4 to 142·2)	24·5 (20·9 to 28·3)	3·7 (2·5 to 5·2)	3·7 (3·2 to 4·4)	515·1 (432·5 to 613·9)	403·4 (317·6 to 499·6)	85·9 (73·3 to 99·5)	12·8 (8·6 to 18·2)	13·0 (11·1 to 15·4)
Bhutan*	2042	151·5 (126·6 to 180·5)	119·1 (93·9 to 147·7)	24·8 (21·2 to 28·7)	3·8 (2·6 to 5·5)	3·7 (3·2 to 4·5)	532·1 (444·7 to 634·3)	418·4 (329·8 to 519·0)	87·1 (74·3 to 100·9)	13·5 (9·1 to 19·4)	13·1 (11·2 to 15·7)

Bhutan*	2043	156·4 (130·3 to 187·3)	123·5 (96·7 to 153·6)	25·1 (21·4 to 29·1)	4·0 (2·7 to 5·9)	3·8 (3·2 to 4·5)	549·6 (457·7 to 658·1)	433·9 (339·6 to 539·5)	88·2 (75·2 to 102·1)	14·1 (9·4 to 20·7)	13·4 (11·4 to 16·0)
Bhutan*	2044	161·5 (133·5 to 194·8)	128·0 (99·8 to 161·0)	25·4 (21·6 to 29·4)	4·2 (2·8 to 6·3)	3·9 (3·3 to 4·7)	567·5 (469·2 to 684·3)	449·8 (350·7 to 565·8)	89·1 (76·0 to 103·3)	14·8 (9·8 to 22·2)	13·7 (11·7 to 16·5)
Bhutan*	2045	166·9 (136·0 to 201·8)	132·8 (101·6 to 168·1)	25·6 (21·8 to 29·7)	4·4 (2·9 to 6·7)	4·0 (3·4 to 4·8)	586·2 (477·6 to 709·0)	466·6 (356·8 to 590·7)	90·0 (76·7 to 104·3)	15·6 (10·1 to 23·6)	14·1 (11·9 to 16·9)
Bhutan*	2046	172·2 (140·3 to 209·0)	137·6 (105·8 to 173·1)	25·8 (22·0 to 29·9)	4·6 (2·9 to 7·1)	4·2 (3·5 to 5·0)	605·0 (492·9 to 734·3)	483·4 (371·7 to 608·2)	90·7 (77·3 to 105·2)	16·3 (10·3 to 25·0)	14·6 (12·3 to 17·5)
Bhutan*	2047	177·7 (144·1 to 216·5)	142·5 (108·9 to 180·5)	26·0 (22·2 to 30·2)	4·9 (3·0 to 7·5)	4·3 (3·6 to 5·2)	624·3 (506·2 to 760·5)	500·7 (382·7 to 634·3)	91·4 (77·8 to 106·0)	17·1 (10·6 to 26·3)	15·2 (12·8 to 18·2)
Bhutan*	2048	183·1 (147·6 to 223·0)	147·3 (112·3 to 186·6)	26·2 (22·3 to 30·4)	5·1 (3·1 to 7·9)	4·5 (3·8 to 5·4)	643·2 (518·6 to 783·5)	517·6 (394·6 to 655·5)	91·9 (78·3 to 106·8)	17·8 (10·8 to 27·8)	15·8 (13·3 to 18·9)
Bhutan*	2049	188·9 (151·3 to 232·8)	152·6 (114·3 to 195·7)	26·3 (22·3 to 30·5)	5·3 (3·1 to 8·4)	4·7 (4·0 to 5·7)	663·6 (531·5 to 818·0)	536·1 (401·6 to 687·4)	92·3 (78·5 to 107·1)	18·7 (11·1 to 29·4)	16·7 (14·1 to 20·0)
Bhutan*	2050	194·6 (155·0 to 240·9)	157·7 (118·1 to 202·9)	26·3 (22·4 to 30·6)	5·5 (3·2 to 8·9)	5·0 (4·2 to 6·1)	683·7 (544·6 to 846·3)	554·0 (415·1 to 713·0)	92·5 (78·7 to 107·3)	19·5 (11·3 to 31·2)	17·6 (14·9 to 21·4)
Botswana*	1995	203·2 (183·3 to 224·1)	103·0 (89·4 to 118·3)	25·8 (21·2 to 31·2)	59·8 (46·6 to 75·3)	14·6 (14·6 to 14·6)	500·4 (451·6 to 551·9)	253·6 (220·3 to 291·5)	63·4 (52·1 to 76·9)	147·4 (114·7 to 185·4)	36·0 (36·0 to 36·0)
Botswana*	1996	200·2 (180·4 to 220·8)	110·7 (97·8 to 125·1)	26·8 (22·4 to 32·4)	61·9 (49·4 to 76·1)	0·7 (0·7 to 0·7)	493·1 (444·4 to 543·9)	272·8 (240·8 to 308·2)	66·1 (55·2 to 79·9)	152·4 (121·6 to 187·5)	1·8 (1·8 to 1·8)
Botswana*	1997	215·1 (196·7 to 235·3)	122·5 (109·3 to 136·9)	28·1 (23·7 to 33·3)	62·9 (51·1 to 77·0)	1·5 (1·5 to 1·5)	529·9 (484·4 to 579·6)	301·8 (269·3 to 337·2)	69·3 (58·3 to 82·0)	155·0 (125·9 to 189·6)	3·8 (3·8 to 3·8)
Botswana*	1998	214·6 (196·3 to 234·0)	126·0 (112·9 to 140·2)	28·2 (24·7 to 32·2)	59·4 (48·2 to 71·7)	1·1 (1·1 to 1·1)	528·7 (483·6 to 576·3)	310·2 (278·0 to 345·3)	69·5 (60·8 to 79·4)	146·3 (118·8 to 176·6)	2·7 (2·7 to 2·7)
Botswana*	1999	222·7 (204·9 to 241·8)	135·5 (121·9 to 150·3)	28·8 (26·0 to 31·8)	57·7 (46·8 to 70·4)	0·7 (0·7 to 0·7)	548·6 (504·6 to 595·5)	333·8 (300·4 to 370·3)	71·0 (64·2 to 78·4)	142·1 (115·3 to 173·3)	1·8 (1·8 to 1·8)
Botswana*	2000	231·4 (214·3 to 250·3)	146·0 (132·3 to 161·8)	29·4 (27·5 to 31·3)	54·5 (44·1 to 66·0)	1·5 (1·5 to 1·5)	569·9 (527·7 to 616·6)	359·6 (325·8 to 398·6)	72·4 (67·8 to 77·1)	134·2 (108·7 to 162·6)	3·7 (3·7 to 3·7)

Botswana*	2001	248·1 (229·6 to 268·5)	160·5 (146·1 to 176·2)	29·0 (27·5 to 30·4)	56·9 (45·8 to 69·0)	1·8 (1·8 to 1·8)	611·2 (565·4 to 661·4)	395·3 (359·9 to 433·9)	71·3 (67·8 to 75·0)	140·1 (112·9 to 170·0)	4·4 (4·4 to 4·4)
Botswana*	2002	274·8 (254·6 to 296·2)	177·1 (161·1 to 194·1)	28·6 (26·7 to 30·4)	60·9 (49·3 to 73·4)	8·3 (8·3 to 8·3)	676·9 (627·0 to 729·5)	436·1 (396·9 to 478·1)	70·4 (65·8 to 75·0)	150·0 (121·5 to 180·8)	20·4 (20·4 to 20·4)
Botswana*	2003	283·4 (262·3 to 304·3)	176·4 (160·2 to 193·1)	27·2 (24·7 to 30·0)	62·2 (51·1 to 74·3)	17·6 (17·6 to 17·6)	698·0 (646·0 to 749·5)	434·5 (394·5 to 475·7)	67·0 (60·8 to 73·9)	153·1 (125·9 to 183·1)	43·4 (43·4 to 43·4)
Botswana*	2004	282·1 (262·5 to 302·9)	170·2 (154·8 to 186·8)	25·4 (22·3 to 28·9)	62·7 (51·9 to 75·3)	23·8 (23·8 to 23·8)	694·7 (646·5 to 746·0)	419·1 (381·3 to 460·0)	62·6 (54·9 to 71·2)	154·3 (127·9 to 185·5)	58·7 (58·7 to 58·7)
Botswana*	2005	255·4 (237·2 to 274·2)	155·6 (142·3 to 170·4)	23·1 (19·6 to 26·9)	61·5 (51·0 to 73·4)	15·3 (15·3 to 15·3)	629·2 (584·3 to 675·4)	383·2 (350·6 to 419·8)	56·8 (48·3 to 66·2)	151·5 (125·7 to 180·9)	37·7 (37·7 to 37·7)
Botswana*	2006	273·7 (255·9 to 292·9)	158·1 (145·0 to 172·3)	21·8 (17·9 to 26·0)	65·3 (54·2 to 77·5)	28·6 (28·6 to 28·6)	674·2 (630·2 to 721·4)	389·3 (357·2 to 424·4)	53·7 (44·1 to 64·1)	160·9 (133·6 to 190·8)	70·3 (70·3 to 70·3)
Botswana*	2007	304·1 (285·1 to 324·2)	177·5 (162·7 to 193·4)	21·3 (17·4 to 25·9)	73·9 (61·3 to 87·8)	31·3 (31·3 to 31·3)	748·9 (702·1 to 798·4)	437·3 (400·7 to 476·4)	52·5 (42·9 to 63·8)	182·0 (151·0 to 216·2)	77·1 (77·1 to 77·1)
Botswana*	2008	476·7 (455·9 to 500·0)	215·5 (198·2 to 233·5)	21·5 (17·3 to 26·3)	90·1 (76·8 to 104·8)	149·6 (149·6 to 149·6)	1174·2 (1123·0 to 1231·4)	530·8 (488·1 to 575·1)	52·9 (42·7 to 64·8)	221·9 (189·1 to 258·0)	368·6 (368·6 to 368·6)
Botswana*	2009	493·2 (470·3 to 517·7)	219·2 (202·5 to 237·2)	20·1 (16·3 to 24·2)	114·7 (99·4 to 131·4)	139·1 (139·1 to 139·1)	1214·7 (1158·3 to 1275·0)	540·0 (498·8 to 584·3)	49·5 (40·1 to 59·7)	282·6 (244·8 to 323·6)	342·7 (342·7 to 342·7)
Botswana*	2010	406·6 (383·7 to 430·2)	219·8 (203·6 to 236·9)	18·5 (15·2 to 22·2)	118·0 (102·8 to 134·2)	50·2 (50·2 to 50·2)	1001·4 (945·1 to 1059·6)	541·5 (501·4 to 583·4)	45·7 (37·4 to 54·8)	290·6 (253·2 to 330·6)	123·7 (123·7 to 123·7)
Botswana*	2011	405·4 (382·6 to 427·3)	224·8 (208·7 to 241·6)	17·2 (14·4 to 20·4)	111·2 (96·7 to 126·8)	52·3 (52·3 to 52·3)	998·6 (942·4 to 1052·4)	553·6 (514·0 to 595·1)	42·3 (35·6 to 50·3)	274·0 (238·3 to 312·2)	128·8 (128·8 to 128·8)
Botswana*	2012	413·6 (391·2 to 436·5)	246·8 (230·3 to 265·0)	16·6 (14·1 to 19·2)	107·9 (93·3 to 123·0)	42·3 (42·3 to 42·3)	1018·6 (963·5 to 1075·1)	608·0 (567·3 to 652·6)	40·8 (34·8 to 47·4)	265·8 (229·7 to 303·1)	104·1 (104·1 to 104·1)
Botswana*	2013	424·0 (400·7 to 445·9)	260·2 (243·2 to 279·0)	15·8 (13·7 to 18·0)	101·2 (87·2 to 116·3)	46·7 (46·7 to 46·7)	1044·3 (986·8 to 1098·2)	641·0 (599·1 to 687·2)	38·9 (33·8 to 44·2)	249·4 (214·7 to 286·4)	115·0 (115·0 to 115·0)
Botswana*	2014	424·3 (401·1 to 448·9)	281·2 (263·3 to 302·1)	15·2 (13·6 to 16·9)	93·3 (80·8 to 107·7)	34·6 (34·6 to 34·6)	1045·0 (987·9 to 1105·7)	692·6 (648·4 to 744·1)	37·3 (33·4 to 41·6)	229·8 (198·9 to 265·4)	85·3 (85·3 to 85·3)

Botswana*	2015	417·8 (394·9 to 443·1)	288·7 (271·1 to 308·9)	14·3 (13·0 to 15·7)	88·7 (75·8 to 102·1)	26·1 (26·1 to 26·1)	1029·1 (972·7 to 1091·4)	711·2 (667·7 to 760·8)	35·3 (32·1 to 38·8)	218·4 (186·7 to 251·6)	64·2 (64·2 to 64·2)
Botswana*	2016	423·1 (399·4 to 448·0)	291·9 (274·1 to 311·5)	13·7 (12·4 to 15·0)	83·5 (69·8 to 98·0)	34·0 (34·0 to 34·0)	1042·1 (983·8 to 1103·4)	718·8 (675·1 to 767·3)	33·6 (30·6 to 37·0)	205·8 (172·0 to 241·3)	83·8 (83·8 to 83·8)
Botswana*	2017	426·3 (402·6 to 452·5)	315·5 (296·0 to 337·2)	13·6 (12·1 to 15·1)	66·6 (54·5 to 80·2)	30·6 (30·6 to 30·6)	1050·0 (991·5 to 1114·6)	777·0 (729·1 to 830·4)	33·4 (29·9 to 37·3)	164·0 (134·3 to 197·7)	75·5 (75·5 to 75·5)
Botswana*	2018	444·5 (416·9 to 473·3)	339·3 (313·0 to 364·5)	14·0 (12·1 to 15·9)	63·3 (50·8 to 79·0)	28·0 (28·0 to 28·0)	1094·8 (1027·0 to 1165·7)	835·7 (770·9 to 897·7)	34·5 (29·9 to 39·2)	155·8 (125·1 to 194·5)	68·9 (68·9 to 68·9)
Botswana*	2019	464·4 (435·7 to 494·5)	356·1 (328·3 to 381·9)	14·1 (12·2 to 16·1)	65·3 (52·4 to 81·3)	28·9 (28·7 to 29·3)	1143·9 (1073·2 to 1217·9)	877·1 (808·6 to 940·6)	34·8 (30·1 to 39·6)	160·8 (129·1 to 200·3)	71·1 (70·7 to 72·2)
Botswana*	2020	481·7 (452·0 to 512·2)	365·3 (336·7 to 391·6)	13·3 (11·5 to 15·1)	63·2 (50·7 to 78·9)	39·8 (39·0 to 42·7)	1186·3 (1113·4 to 1261·6)	899·7 (829·2 to 964·4)	32·8 (28·4 to 37·3)	155·7 (125·0 to 194·3)	98·1 (96·0 to 105·2)
Botswana*	2021	467·8 (438·8 to 497·4)	353·3 (325·6 to 378·7)	12·9 (11·1 to 14·7)	62·8 (50·1 to 78·6)	38·8 (37·5 to 41·6)	1152·1 (1080·7 to 1225·1)	870·2 (802·0 to 932·8)	31·7 (27·4 to 36·1)	154·6 (123·3 to 193·5)	95·6 (92·4 to 102·6)
Botswana*	2022	454·2 (421·9 to 486·8)	346·7 (318·6 to 376·8)	13·1 (11·3 to 14·9)	65·2 (51·8 to 81·4)	29·2 (27·3 to 31·9)	1118·6 (1039·2 to 1199·1)	853·9 (784·8 to 928·2)	32·2 (27·9 to 36·7)	160·5 (127·6 to 200·5)	72·0 (67·2 to 78·6)
Botswana*	2023	467·9 (432·7 to 503·7)	357·8 (324·8 to 391·4)	13·3 (11·5 to 15·2)	67·8 (53·8 to 84·4)	29·0 (26·8 to 31·8)	1152·4 (1065·6 to 1240·7)	881·2 (799·9 to 963·9)	32·8 (28·4 to 37·4)	167·0 (132·5 to 207·8)	71·3 (66·0 to 78·3)
Botswana*	2024	482·4 (443·8 to 521·6)	369·6 (333·8 to 406·1)	13·6 (11·8 to 15·4)	70·6 (56·0 to 88·1)	28·7 (26·3 to 31·6)	1188·2 (1093·0 to 1284·8)	910·3 (822·3 to 1000·3)	33·5 (29·0 to 38·1)	173·8 (138·0 to 216·9)	70·6 (64·8 to 77·9)
Botswana*	2025	503·5 (463·3 to 544·2)	387·9 (350·3 to 424·8)	13·8 (12·0 to 15·8)	73·5 (58·3 to 91·4)	28·3 (25·8 to 31·5)	1240·1 (1141·0 to 1340·3)	955·3 (862·7 to 1046·3)	34·1 (29·5 to 38·8)	181·0 (143·5 to 225·1)	69·7 (63·6 to 77·5)
Botswana*	2026	521·5 (479·0 to 564·0)	403·0 (362·9 to 441·8)	14·1 (12·2 to 16·1)	76·6 (61·0 to 95·6)	27·8 (25·2 to 31·2)	1284·6 (1179·8 to 1389·1)	992·7 (893·7 to 1088·2)	34·8 (30·1 to 39·6)	188·6 (150·2 to 235·5)	68·5 (62·1 to 76·8)
Botswana*	2027	540·7 (496·1 to 585·0)	419·2 (376·2 to 460·4)	14·4 (12·5 to 16·4)	79·7 (63·6 to 99·5)	27·4 (24·7 to 30·9)	1331·8 (1221·9 to 1440·8)	1032·5 (926·6 to 1134·0)	35·5 (30·7 to 40·3)	196·3 (156·7 to 245·0)	67·5 (60·9 to 76·2)
Botswana*	2028	560·7 (513·0 to 607·6)	436·1 (391·7 to 480·8)	14·7 (12·7 to 16·7)	83·0 (66·5 to 103·7)	26·9 (24·2 to 30·5)	1381·0 (1263·5 to 1496·6)	1074·0 (964·8 to 1184·2)	36·1 (31·3 to 41·1)	204·4 (163·8 to 255·4)	66·4 (59·5 to 75·1)

Botswana*	2029	581·8 (532·0 to 631·4)	453·9 (406·9 to 501·2)	15·0 (13·0 to 17·0)	86·5 (69·0 to 108·1)	26·5 (23·5 to 30·4)	1432·9 (1310·2 to 1555·1)	1117·9 (1002·3 to 1234·6)	36·9 (31·9 to 41·9)	213·0 (169·9 to 266·3)	65·2 (57·9 to 74·8)
Botswana*	2030	604·4 (552·3 to 656·3)	472·8 (423·6 to 522·6)	15·3 (13·2 to 17·4)	90·4 (71·6 to 113·0)	25·9 (22·9 to 29·9)	1488·7 (1360·3 to 1616·4)	1164·6 (1043·3 to 1287·1)	37·6 (32·6 to 42·8)	222·7 (176·5 to 278·3)	63·8 (56·4 to 73·6)
Botswana*	2031	629·1 (574·1 to 682·7)	493·7 (442·5 to 545·0)	15·5 (13·5 to 17·7)	94·4 (74·5 to 117·9)	25·5 (22·3 to 29·5)	1549·4 (1414·0 to 1681·4)	1215·9 (1090·0 to 1342·3)	38·3 (33·2 to 43·5)	232·5 (183·6 to 290·3)	62·8 (55·0 to 72·7)
Botswana*	2032	656·2 (598·0 to 712·9)	516·8 (462·6 to 571·3)	15·8 (13·7 to 18·0)	98·4 (77·9 to 123·8)	25·1 (21·8 to 29·1)	1616·2 (1472·8 to 1755·9)	1273·0 (1139·3 to 1407·2)	39·0 (33·8 to 44·4)	242·5 (192·0 to 305·0)	61·8 (53·7 to 71·8)
Botswana*	2033	684·1 (622·6 to 743·9)	540·5 (482·4 to 597·3)	16·1 (14·0 to 18·3)	102·7 (81·2 to 128·8)	24·7 (21·3 to 29·0)	1684·9 (1533·4 to 1832·1)	1331·4 (1188·2 to 1471·2)	39·7 (34·4 to 45·2)	253·0 (199·9 to 317·2)	60·8 (52·5 to 71·4)
Botswana*	2034	713·0 (649·4 to 775·8)	565·1 (503·0 to 624·3)	16·4 (14·2 to 18·7)	107·2 (84·3 to 134·7)	24·3 (21·1 to 28·7)	1756·3 (1599·5 to 1910·7)	1391·9 (1238·9 to 1537·8)	40·4 (35·1 to 46·0)	264·0 (207·6 to 331·9)	60·0 (51·9 to 70·7)
Botswana*	2035	742·8 (674·9 to 809·6)	590·3 (525·0 to 652·7)	16·7 (14·5 to 19·0)	111·8 (87·7 to 140·7)	24·0 (20·7 to 28·6)	1829·6 (1662·4 to 1994·1)	1453·9 (1293·0 to 1607·6)	41·2 (35·7 to 46·8)	275·4 (216·0 to 346·5)	59·2 (50·9 to 70·4)
Botswana*	2036	772·8 (701·0 to 842·9)	615·4 (546·5 to 682·0)	17·0 (14·8 to 19·4)	116·6 (90·7 to 147·3)	23·8 (20·3 to 28·4)	1903·5 (1726·7 to 2076·2)	1515·8 (1346·0 to 1679·7)	41·9 (36·4 to 47·7)	287·2 (223·3 to 362·7)	58·6 (50·1 to 70·1)
Botswana*	2037	804·3 (728·5 to 878·7)	641·8 (568·9 to 712·2)	17·3 (15·0 to 19·7)	121·5 (94·1 to 154·3)	23·7 (20·0 to 28·3)	1981·0 (1794·4 to 2164·4)	1580·8 (1401·2 to 1754·3)	42·6 (37·0 to 48·5)	299·4 (231·8 to 380·2)	58·3 (49·2 to 69·6)
Botswana*	2038	836·9 (757·3 to 914·8)	669·0 (592·2 to 744·3)	17·6 (15·2 to 20·0)	126·7 (97·6 to 162·8)	23·6 (19·8 to 28·3)	2061·2 (1865·3 to 2253·1)	1647·9 (1458·5 to 1833·2)	43·3 (37·5 to 49·2)	312·1 (240·4 to 400·9)	58·0 (48·8 to 69·6)
Botswana*	2039	869·9 (786·5 to 952·1)	696·4 (616·0 to 774·7)	17·8 (15·5 to 20·3)	132·0 (101·3 to 170·7)	23·6 (19·7 to 28·5)	2142·6 (1937·3 to 2344·9)	1715·4 (1517·3 to 1908·2)	43·9 (38·1 to 50·0)	325·2 (249·5 to 420·5)	58·1 (48·6 to 70·3)
Botswana*	2040	903·7 (815·6 to 989·7)	724·4 (640·4 to 806·5)	18·1 (15·7 to 20·6)	137·6 (104·4 to 179·8)	23·7 (19·7 to 28·9)	2225·9 (2008·9 to 2437·8)	1784·1 (1577·3 to 1986·4)	44·6 (38·6 to 50·9)	338·8 (257·1 to 442·9)	58·3 (48·6 to 71·2)
Botswana*	2041	937·9 (845·4 to 1028·7)	752·5 (663·8 to 838·5)	18·4 (15·9 to 21·0)	143·2 (107·5 to 187·7)	23·8 (19·7 to 29·1)	2310·2 (2082·1 to 2533·7)	1853·4 (1634·9 to 2065·1)	45·2 (39·2 to 51·6)	352·8 (264·9 to 462·3)	58·7 (48·5 to 71·7)
Botswana*	2042	972·8 (876·8 to 1067·4)	781·0 (688·5 to 870·4)	18·6 (16·1 to 21·3)	149·1 (110·7 to 196·5)	24·0 (19·8 to 29·6)	2396·0 (2159·6 to 2629·0)	1923·7 (1695·9 to 2143·8)	45·8 (39·7 to 52·3)	367·3 (272·7 to 484·0)	59·2 (48·7 to 72·8)

Botswana*	2043	1008·2 (907·0 to 1107·8)	809·9 (714·8 to 904·0)	18·8 (16·4 to 21·5)	155·2 (113·8 to 208·6)	24·3 (19·9 to 30·3)	2483·3 (2233·9 to 2728·5)	1994·7 (1760·7 to 2226·6)	46·4 (40·3 to 53·1)	382·2 (280·4 to 513·8)	59·9 (49·0 to 74·8)
Botswana*	2044	1044·1 (938·7 to 1147·9)	838·9 (738·9 to 936·3)	19·1 (16·5 to 21·8)	161·4 (117·4 to 218·5)	24·7 (20·1 to 30·7)	2571·7 (2312·1 to 2827·3)	2066·1 (1820·0 to 2306·2)	47·0 (40·8 to 53·8)	397·6 (289·1 to 538·3)	60·9 (49·6 to 75·7)
Botswana*	2045	1080·6 (971·5 to 1189·1)	868·3 (764·8 to 969·7)	19·3 (16·7 to 22·1)	167·9 (120·8 to 229·1)	25·1 (20·3 to 31·4)	2661·6 (2392·9 to 2928·7)	2138·6 (1883·7 to 2388·5)	47·6 (41·2 to 54·4)	413·6 (297·6 to 564·3)	61·9 (50·0 to 77·4)
Botswana*	2046	1116·8 (1002·6 to 1229·5)	897·1 (790·3 to 1001·7)	19·5 (16·9 to 22·4)	174·6 (124·6 to 241·3)	25·5 (20·3 to 32·4)	2750·7 (2469·4 to 3028·4)	2209·7 (1946·5 to 2467·3)	48·1 (41·6 to 55·1)	430·1 (306·9 to 594·2)	62·8 (50·1 to 79·7)
Botswana*	2047	1152·5 (1032·6 to 1270·8)	925·3 (814·5 to 1032·5)	19·8 (17·1 to 22·7)	181·5 (128·4 to 255·2)	25·8 (20·3 to 33·3)	2838·5 (2543·2 to 3130·0)	2279·2 (2006·0 to 2543·2)	48·7 (42·0 to 55·8)	447·0 (316·3 to 628·5)	63·7 (50·0 to 81·9)
Botswana*	2048	1187·9 (1064·6 to 1311·9)	953·2 (838·8 to 1064·1)	20·0 (17·2 to 22·9)	188·5 (132·1 to 269·1)	26·2 (20·4 to 34·0)	2925·9 (2622·2 to 3231·2)	2347·8 (2066·0 to 2621·0)	49·2 (42·5 to 56·5)	464·4 (325·5 to 662·8)	64·6 (50·3 to 83·7)
Botswana*	2049	1222·7 (1095·3 to 1350·8)	980·1 (861·9 to 1092·6)	20·2 (17·4 to 23·2)	195·7 (136·0 to 281·9)	26·7 (0·0 to 34·8)	3011·6 (2697·7 to 3327·1)	2414·1 (2122·9 to 2691·2)	49·7 (42·9 to 57·1)	482·1 (334·9 to 694·4)	65·8 (0·0 to 85·7)
Botswana*	2050	1257·3 (1124·0 to 1392·1)	1006·6 (886·4 to 1122·2)	20·4 (17·5 to 23·4)	203·1 (139·7 to 295·4)	27·3 (0·0 to 36·2)	3096·9 (2768·4 to 3428·7)	2479·3 (2183·1 to 2764·1)	50·1 (43·2 to 57·6)	500·3 (344·2 to 727·5)	67·1 (0·0 to 89·2)
Central African Republic*	1995	26·6 (23·1 to 30·8)	7·0 (5·7 to 8·5)	15·1 (11·8 to 19·1)	0·3 (0·2 to 0·5)	4·2 (4·2 to 4·2)	53·8 (46·8 to 62·4)	14·2 (11·5 to 17·1)	30·5 (23·9 to 38·7)	0·6 (0·4 to 0·9)	8·4 (8·4 to 8·4)
Central African Republic*	1996	20·0 (16·8 to 23·8)	5·5 (4·5 to 6·6)	13·7 (10·7 to 17·3)	0·3 (0·2 to 0·4)	0·5 (0·5 to 0·5)	40·4 (34·1 to 48·2)	11·1 (9·1 to 13·3)	27·7 (21·6 to 35·0)	0·6 (0·4 to 0·8)	0·9 (0·9 to 0·9)
Central African Republic*	1997	21·7 (18·5 to 25·4)	5·6 (4·6 to 6·7)	13·8 (10·9 to 17·3)	0·3 (0·2 to 0·4)	2·0 (2·0 to 2·0)	43·9 (37·5 to 51·3)	11·4 (9·3 to 13·5)	28·0 (22·0 to 34·9)	0·6 (0·4 to 0·8)	3·9 (3·9 to 3·9)
Central African Republic*	1998	27·3 (24·2 to 30·8)	5·9 (4·9 to 7·1)	13·8 (10·9 to 17·1)	0·3 (0·2 to 0·4)	7·3 (7·3 to 7·3)	55·2 (49·0 to 62·4)	11·9 (9·9 to 14·3)	28·0 (22·0 to 34·6)	0·6 (0·4 to 0·8)	14·7 (14·7 to 14·7)
Central African Republic*	1999	25·6 (22·5 to 29·1)	7·1 (5·9 to 8·5)	13·9 (11·0 to 17·3)	0·3 (0·2 to 0·4)	4·2 (4·2 to 4·2)	51·7 (45·5 to 58·8)	14·4 (12·0 to 17·2)	28·2 (22·2 to 35·0)	0·6 (0·4 to 0·9)	8·5 (8·5 to 8·5)
Central African Republic*	2000	23·8 (20·4 to 27·5)	8·3 (6·9 to 9·7)	13·6 (10·6 to 17·0)	0·3 (0·2 to 0·4)	1·6 (1·6 to 1·6)	48·1 (41·4 to 55·6)	16·7 (14·0 to 19·7)	27·5 (21·5 to 34·4)	0·6 (0·4 to 0·9)	3·3 (3·3 to 3·3)
Central African Republic*	2001	23·8 (20·6 to 27·3)	8·0 (6·6 to 9·5)	13·0 (10·2 to 16·3)	0·3 (0·2 to 0·4)	2·5 (2·5 to 2·5)	48·1 (41·6 to 55·1)	16·2 (13·3 to 19·1)	26·3 (20·7 to 32·9)	0·6 (0·4 to 0·8)	5·0 (5·0 to 5·0)
Central African Republic*	2002	26·2 (23·1 to 29·7)	9·0 (7·5 to 10·7)	13·4 (10·6 to 16·8)	0·3 (0·2 to 0·4)	3·4 (3·4 to 3·4)	52·9 (46·6 to 60·2)	18·3 (15·2 to 21·7)	27·1 (21·5 to 33·9)	0·6 (0·4 to 0·9)	6·9 (6·9 to 6·9)

Central African Republic*	2003	22·4 (19·7 to 25·8)	7·4 (6·2 to 8·8)	12·4 (9·9 to 15·6)	0·3 (0·2 to 0·4)	2·3 (2·3 to 2·3)	45·2 (39·9 to 52·1)	15·0 (12·5 to 17·8)	25·2 (20·1 to 31·6)	0·6 (0·4 to 0·8)	4·6 (4·6 to 4·6)
Central African Republic*	2004	23·6 (21·0 to 26·9)	7·2 (6·1 to 8·5)	12·6 (10·2 to 15·7)	0·3 (0·2 to 0·4)	3·5 (3·5 to 3·5)	47·8 (42·4 to 54·5)	14·5 (12·3 to 17·2)	25·6 (20·6 to 31·8)	0·6 (0·4 to 0·8)	7·1 (7·1 to 7·1)
Central African Republic*	2005	25·0 (22·2 to 28·2)	7·3 (6·1 to 8·6)	13·2 (10·8 to 16·1)	0·3 (0·2 to 0·4)	4·2 (4·2 to 4·2)	50·5 (45·0 to 57·1)	14·7 (12·4 to 17·4)	26·6 (21·8 to 32·7)	0·6 (0·4 to 0·8)	8·6 (8·6 to 8·6)
Central African Republic*	2006	24·5 (21·9 to 27·6)	6·2 (5·2 to 7·3)	12·9 (10·7 to 15·6)	0·3 (0·2 to 0·4)	5·0 (5·0 to 5·0)	49·5 (44·2 to 55·8)	12·6 (10·6 to 14·8)	26·2 (21·6 to 31·7)	0·6 (0·4 to 0·8)	10·2 (10·2 to 10·2)
Central African Republic*	2007	21·6 (18·9 to 24·4)	5·5 (4·6 to 6·6)	13·1 (10·8 to 15·7)	0·3 (0·2 to 0·4)	2·7 (2·7 to 2·7)	43·6 (38·3 to 49·4)	11·2 (9·3 to 13·3)	26·5 (21·9 to 31·7)	0·6 (0·4 to 0·8)	5·4 (5·4 to 5·4)
Central African Republic*	2008	27·2 (24·6 to 29·9)	5·1 (4·2 to 6·0)	13·5 (11·2 to 16·1)	0·3 (0·2 to 0·4)	8·4 (8·4 to 8·4)	55·1 (49·8 to 60·5)	10·3 (8·5 to 12·2)	27·3 (22·7 to 32·5)	0·6 (0·4 to 0·9)	16·9 (16·9 to 16·9)
Central African Republic*	2009	21·1 (18·6 to 23·8)	4·4 (3·6 to 5·2)	13·2 (11·0 to 15·7)	0·3 (0·2 to 0·4)	3·2 (3·2 to 3·2)	42·7 (37·7 to 48·2)	8·8 (7·3 to 10·6)	26·8 (22·3 to 31·8)	0·6 (0·4 to 0·9)	6·5 (6·5 to 6·5)
Central African Republic*	2010	22·5 (20·2 to 25·3)	4·1 (3·4 to 4·9)	13·2 (11·0 to 15·9)	0·3 (0·2 to 0·4)	4·9 (4·9 to 4·9)	45·5 (40·9 to 51·2)	8·2 (6·8 to 9·9)	26·7 (22·3 to 32·2)	0·6 (0·4 to 0·9)	10·0 (10·0 to 10·0)
Central African Republic*	2011	21·4 (19·2 to 24·0)	3·4 (2·8 to 4·1)	12·5 (10·4 to 15·0)	0·3 (0·2 to 0·4)	5·2 (5·2 to 5·2)	43·3 (38·8 to 48·6)	6·9 (5·7 to 8·3)	25·2 (21·0 to 30·4)	0·5 (0·4 to 0·8)	10·6 (10·6 to 10·6)
Central African Republic*	2012	22·0 (19·7 to 24·5)	3·8 (3·1 to 4·6)	12·7 (10·6 to 15·3)	0·3 (0·2 to 0·4)	5·1 (5·1 to 5·1)	44·4 (39·9 to 49·7)	7·7 (6·3 to 9·3)	25·8 (21·4 to 31·0)	0·5 (0·4 to 0·7)	10·4 (10·4 to 10·4)
Central African Republic*	2013	20·1 (18·4 to 22·2)	2·8 (2·3 to 3·4)	10·7 (9·0 to 12·7)	0·2 (0·1 to 0·3)	6·4 (6·4 to 6·4)	40·7 (37·2 to 44·9)	5·7 (4·7 to 6·9)	21·7 (18·2 to 25·7)	0·4 (0·3 to 0·6)	12·9 (12·9 to 12·9)
Central African Republic*	2014	24·4 (22·6 to 26·5)	3·1 (2·5 to 3·8)	11·7 (10·0 to 13·6)	0·2 (0·1 to 0·3)	9·3 (9·3 to 9·3)	49·3 (45·8 to 53·6)	6·3 (5·1 to 7·6)	23·7 (20·3 to 27·6)	0·4 (0·3 to 0·6)	18·9 (18·9 to 18·9)
Central African Republic*	2015	19·6 (18·0 to 21·4)	2·5 (2·1 to 3·1)	11·9 (10·4 to 13·7)	0·2 (0·1 to 0·3)	5·0 (5·0 to 5·0)	39·6 (36·5 to 43·4)	5·1 (4·2 to 6·2)	24·0 (21·0 to 27·6)	0·4 (0·3 to 0·5)	10·0 (10·0 to 10·0)
Central African Republic*	2016	26·8 (25·3 to 28·5)	2·5 (2·0 to 3·0)	13·3 (11·8 to 15·0)	0·2 (0·2 to 0·3)	10·8 (10·8 to 10·8)	54·3 (51·1 to 57·8)	5·0 (4·0 to 6·0)	26·9 (23·9 to 30·3)	0·5 (0·3 to 0·7)	21·9 (21·9 to 21·9)
Central African Republic*	2017	29·8 (28·1 to 31·8)	2·8 (2·3 to 3·4)	16·0 (14·4 to 18·0)	0·3 (0·2 to 0·4)	10·7 (10·7 to 10·7)	60·2 (56·8 to 64·4)	5·7 (4·7 to 6·9)	32·4 (29·1 to 36·3)	0·6 (0·4 to 0·9)	21·6 (21·6 to 21·6)
Central African Republic*	2018	29·5 (27·5 to 32·1)	3·1 (2·6 to 3·7)	18·4 (16·4 to 20·8)	0·3 (0·2 to 0·5)	7·7 (7·7 to 7·7)	59·7 (55·6 to 64·9)	6·3 (5·2 to 7·6)	37·2 (33·2 to 42·1)	0·7 (0·5 to 0·9)	15·6 (15·6 to 15·6)
Central African Republic*	2019	30·0 (27·8 to 32·6)	3·1 (2·4 to 3·8)	18·8 (16·7 to 21·3)	0·3 (0·2 to 0·5)	7·8 (7·7 to 7·9)	60·7 (56·3 to 66·0)	6·2 (4·9 to 7·8)	38·0 (33·9 to 43·1)	0·7 (0·5 to 1·0)	15·8 (15·7 to 15·9)
Central African Republic*	2020	32·8 (30·5 to 35·4)	3·7 (2·9 to 4·8)	17·6 (15·7 to 19·9)	0·3 (0·2 to 0·5)	11·1 (11·0 to 11·8)	66·4 (61·7 to 71·6)	7·6 (5·8 to 9·7)	35·6 (31·7 to 40·3)	0·6 (0·5 to 0·9)	22·5 (22·2 to 24·0)

Central African Republic*	2021	32·2 (30·0 to 34·7)	3·6 (2·7 to 4·6)	16·7 (14·9 to 18·9)	0·3 (0·2 to 0·4)	11·7 (11·4 to 13·1)	65·2 (60·7 to 70·2)	7·2 (5·5 to 9·3)	33·7 (30·0 to 38·3)	0·6 (0·4 to 0·9)	23·6 (23·1 to 26·4)
Central African Republic*	2022	29·1 (26·9 to 31·5)	2·9 (2·0 to 3·9)	16·9 (15·1 to 19·1)	0·3 (0·2 to 0·5)	9·0 (8·4 to 10·1)	58·8 (54·3 to 63·6)	5·8 (4·1 to 8·0)	34·2 (30·5 to 38·7)	0·7 (0·5 to 0·9)	18·2 (17·1 to 20·4)
Central African Republic*	2023	29·5 (27·3 to 31·9)	3·0 (2·1 to 4·1)	17·1 (15·3 to 19·4)	0·3 (0·2 to 0·5)	9·0 (8·4 to 10·2)	59·7 (55·1 to 64·5)	6·0 (4·2 to 8·3)	34·7 (30·9 to 39·3)	0·7 (0·5 to 1·0)	18·3 (17·1 to 20·6)
Central African Republic*	2024	29·9 (27·6 to 32·4)	3·1 (2·1 to 4·3)	17·4 (15·5 to 19·7)	0·4 (0·2 to 0·5)	9·1 (8·5 to 10·3)	60·6 (55·7 to 65·6)	6·2 (4·2 to 8·8)	35·2 (31·4 to 39·9)	0·7 (0·5 to 1·0)	18·4 (17·1 to 20·8)
Central African Republic*	2025	30·4 (27·9 to 33·0)	3·2 (2·1 to 4·5)	17·7 (15·8 to 20·0)	0·4 (0·3 to 0·5)	9·2 (8·5 to 10·4)	61·4 (56·5 to 66·7)	6·4 (4·3 to 9·0)	35·8 (31·9 to 40·6)	0·7 (0·5 to 1·1)	18·5 (17·2 to 21·0)
Central African Republic*	2026	30·8 (28·3 to 33·5)	3·3 (2·1 to 4·8)	18·0 (16·0 to 20·4)	0·4 (0·3 to 0·6)	9·2 (8·4 to 10·4)	62·3 (57·2 to 67·7)	6·6 (4·3 to 9·7)	36·4 (32·4 to 41·2)	0·8 (0·5 to 1·1)	18·6 (17·1 to 21·0)
Central African Republic*	2027	31·2 (28·6 to 34·0)	3·4 (2·1 to 5·0)	18·3 (16·3 to 20·7)	0·4 (0·3 to 0·6)	9·2 (8·4 to 10·4)	63·1 (57·9 to 68·7)	6·8 (4·3 to 10·1)	37·0 (33·0 to 41·9)	0·8 (0·6 to 1·2)	18·6 (17·1 to 21·0)
Central African Republic*	2028	31·6 (28·9 to 34·5)	3·5 (2·1 to 5·3)	18·6 (16·6 to 21·0)	0·4 (0·3 to 0·6)	9·2 (8·4 to 10·4)	64·0 (58·5 to 69·8)	7·0 (4·3 to 10·7)	37·6 (33·5 to 42·6)	0·8 (0·6 to 1·2)	18·5 (16·9 to 21·0)
Central African Republic*	2029	32·0 (29·3 to 35·0)	3·6 (2·1 to 5·6)	18·8 (16·8 to 21·3)	0·4 (0·3 to 0·6)	9·2 (8·3 to 10·4)	64·8 (59·2 to 70·9)	7·3 (4·3 to 11·3)	38·1 (34·0 to 43·2)	0·9 (0·6 to 1·3)	18·6 (16·8 to 21·1)
Central African Republic*	2030	32·5 (29·6 to 35·6)	3·7 (2·1 to 5·9)	19·1 (17·0 to 21·7)	0·5 (0·3 to 0·7)	9·2 (8·3 to 10·5)	65·7 (59·9 to 71·9)	7·5 (4·3 to 11·9)	38·7 (34·5 to 43·8)	0·9 (0·6 to 1·3)	18·6 (16·8 to 21·3)
Central African Republic*	2031	32·9 (29·9 to 36·1)	3·8 (2·2 to 6·1)	19·4 (17·3 to 22·0)	0·5 (0·3 to 0·7)	9·2 (8·3 to 10·6)	66·6 (60·5 to 73·0)	7·8 (4·5 to 12·4)	39·2 (35·0 to 44·4)	1·0 (0·7 to 1·4)	18·7 (16·7 to 21·5)
Central African Republic*	2032	33·4 (30·2 to 36·7)	4·0 (2·2 to 6·5)	19·6 (17·5 to 22·2)	0·5 (0·3 to 0·7)	9·3 (8·3 to 10·7)	67·5 (61·2 to 74·3)	8·0 (4·5 to 13·1)	39·7 (35·4 to 45·0)	1·0 (0·7 to 1·4)	18·8 (16·8 to 21·7)
Central African Republic*	2033	33·9 (30·7 to 37·4)	4·1 (2·2 to 6·6)	19·9 (17·7 to 22·5)	0·5 (0·4 to 0·7)	9·4 (8·3 to 10·9)	68·5 (62·1 to 75·6)	8·3 (4·5 to 13·4)	40·2 (35·8 to 45·5)	1·0 (0·7 to 1·5)	19·0 (16·9 to 22·1)
Central African Republic*	2034	34·4 (31·1 to 38·1)	4·2 (2·3 to 7·0)	20·1 (17·9 to 22·8)	0·5 (0·4 to 0·8)	9·5 (8·5 to 11·2)	69·5 (62·9 to 77·0)	8·5 (4·6 to 14·1)	40·6 (36·2 to 46·0)	1·1 (0·8 to 1·6)	19·3 (17·1 to 22·6)
Central African Republic*	2035	34·9 (31·5 to 38·8)	4·4 (2·4 to 7·3)	20·3 (18·1 to 23·0)	0·5 (0·4 to 0·8)	9·7 (8·6 to 11·5)	70·7 (63·8 to 78·5)	8·8 (4·9 to 14·8)	41·0 (36·6 to 46·5)	1·1 (0·8 to 1·6)	19·7 (17·4 to 23·3)
Central African Republic*	2036	35·5 (32·0 to 39·7)	4·5 (2·4 to 7·6)	20·5 (18·3 to 23·2)	0·6 (0·4 to 0·8)	10·0 (8·8 to 11·9)	71·8 (64·6 to 80·2)	9·1 (4·9 to 15·5)	41·4 (36·9 to 46·9)	1·1 (0·8 to 1·6)	20·2 (17·7 to 24·1)
Central African Republic*	2037	36·1 (32·5 to 40·4)	4·6 (2·5 to 7·9)	20·6 (18·4 to 23·4)	0·6 (0·4 to 0·8)	10·2 (9·0 to 12·3)	73·0 (65·7 to 81·6)	9·3 (5·1 to 16·0)	41·8 (37·3 to 47·3)	1·2 (0·8 to 1·7)	20·7 (18·3 to 24·9)
Central African Republic*	2038	36·7 (32·9 to 41·3)	4·7 (2·5 to 8·4)	20·8 (18·5 to 23·5)	0·6 (0·4 to 0·9)	10·6 (9·3 to 12·7)	74·2 (66·6 to 83·6)	9·6 (5·2 to 17·0)	42·0 (37·5 to 47·6)	1·2 (0·9 to 1·7)	21·4 (18·8 to 25·8)

Central African Republic*	2039	37·3 (33·4 to 42·0)	4·8 (2·5 to 8·5)	20·9 (18·7 to 23·7)	0·6 (0·4 to 0·9)	10·9 (9·5 to 13·4)	75·5 (67·5 to 84·9)	9·8 (5·2 to 17·2)	42·3 (37·8 to 47·9)	1·3 (0·9 to 1·8)	22·1 (19·3 to 27·0)
Central African Republic*	2040	38·0 (33·9 to 42·9)	5·0 (2·6 to 8·6)	21·1 (18·8 to 23·8)	0·6 (0·4 to 0·9)	11·3 (9·8 to 14·0)	76·9 (68·6 to 86·8)	10·0 (5·2 to 17·5)	42·6 (38·0 to 48·2)	1·3 (0·9 to 1·9)	22·9 (19·9 to 28·3)
Central African Republic*	2041	38·7 (34·4 to 43·8)	5·1 (2·6 to 9·2)	21·2 (18·9 to 24·0)	0·7 (0·5 to 1·0)	11·8 (10·2 to 14·7)	78·3 (69·6 to 88·7)	10·3 (5·2 to 18·6)	42·8 (38·3 to 48·5)	1·3 (0·9 to 1·9)	23·8 (20·7 to 29·7)
Central African Republic*	2042	39·5 (35·1 to 44·8)	5·2 (2·5 to 9·8)	21·3 (19·0 to 24·1)	0·7 (0·5 to 1·0)	12·2 (10·5 to 15·5)	79·9 (71·0 to 90·7)	10·6 (5·0 to 19·9)	43·1 (38·5 to 48·8)	1·4 (0·9 to 2·0)	24·7 (21·3 to 31·4)
Central African Republic*	2043	40·3 (35·7 to 45·7)	5·4 (2·5 to 9·9)	21·5 (19·2 to 24·3)	0·7 (0·5 to 1·0)	12·7 (10·9 to 16·3)	81·4 (72·2 to 92·5)	10·9 (5·1 to 20·0)	43·4 (38·8 to 49·1)	1·4 (1·0 to 2·0)	25·7 (22·0 to 32·9)
Central African Republic*	2044	41·1 (36·4 to 46·9)	5·5 (2·5 to 10·4)	21·6 (19·3 to 24·4)	0·7 (0·5 to 1·1)	13·3 (11·3 to 17·2)	83·2 (73·6 to 94·8)	11·2 (5·1 to 21·0)	43·6 (39·0 to 49·4)	1·5 (1·0 to 2·1)	26·9 (22·9 to 34·9)
Central African Republic*	2045	42·0 (36·9 to 48·1)	5·7 (2·6 to 10·6)	21·7 (19·4 to 24·5)	0·7 (0·5 to 1·1)	13·9 (11·7 to 18·1)	84·9 (74·7 to 97·3)	11·5 (5·2 to 21·5)	43·9 (39·2 to 49·7)	1·5 (1·0 to 2·2)	28·0 (23·7 to 36·5)
Central African Republic*	2046	42·9 (37·7 to 49·6)	5·8 (2·6 to 11·3)	21·8 (19·5 to 24·7)	0·8 (0·5 to 1·1)	14·5 (12·2 to 19·2)	86·7 (76·3 to 100·4)	11·8 (5·3 to 22·8)	44·1 (39·4 to 49·9)	1·5 (1·1 to 2·3)	29·3 (24·6 to 38·8)
Central African Republic*	2047	43·8 (38·5 to 51·2)	6·0 (2·7 to 11·8)	21·9 (19·6 to 24·8)	0·8 (0·5 to 1·2)	15·1 (12·6 to 20·3)	88·7 (77·9 to 103·6)	12·1 (5·5 to 23·9)	44·3 (39·6 to 50·2)	1·6 (1·1 to 2·3)	30·6 (25·4 to 41·0)
Central African Republic*	2048	44·8 (39·3 to 52·6)	6·2 (2·7 to 12·6)	22·0 (19·6 to 24·9)	0·8 (0·5 to 1·2)	15·9 (13·2 to 21·6)	90·7 (79·4 to 106·4)	12·5 (5·5 to 25·5)	44·5 (39·7 to 50·4)	1·6 (1·1 to 2·4)	32·1 (26·6 to 43·8)
Central African Republic*	2049	45·9 (40·0 to 53·9)	6·3 (2·8 to 12·7)	22·1 (19·7 to 25·0)	0·8 (0·6 to 1·2)	16·6 (13·7 to 22·8)	92·9 (80·9 to 109·0)	12·8 (5·7 to 25·8)	44·7 (39·9 to 50·7)	1·7 (1·1 to 2·5)	33·6 (27·7 to 46·1)
Central African Republic*	2050	47·0 (40·9 to 55·6)	6·5 (2·8 to 13·2)	22·2 (19·8 to 25·2)	0·9 (0·6 to 1·3)	17·5 (14·3 to 24·2)	95·1 (82·7 to 112·5)	13·1 (5·6 to 26·8)	44·9 (40·1 to 50·9)	1·7 (1·1 to 2·6)	35·4 (28·8 to 48·9)
Canada	1995	2833·6 (2775·8 to 2888·4)	2072·4 (2027·7 to 2120·9)	474·8 (447·1 to 503·6)	286·4 (265·2 to 310·4)	0·0 (0·0 to 0·0)	3203·3 (3137·9 to 3265·2)	2342·8 (2292·2 to 2397·6)	536·8 (505·4 to 569·3)	323·8 (299·8 to 351·0)	0·0 (0·0 to 0·0)
Canada	1996	2818·3 (2772·9 to 2864·2)	2046·1 (2008·7 to 2083·4)	481·2 (460·8 to 503·6)	291·1 (274·1 to 309·0)	0·0 (0·0 to 0·0)	3186·1 (3134·7 to 3238·0)	2313·1 (2270·8 to 2355·2)	544·0 (520·9 to 569·3)	329·0 (309·8 to 349·3)	0·0 (0·0 to 0·0)
Canada	1997	2872·0 (2824·0 to 2920·3)	2072·6 (2033·0 to 2110·3)	498·0 (478·3 to 519·4)	301·4 (284·9 to 319·3)	0·0 (0·0 to 0·0)	3246·7 (3192·5 to 3301·3)	2343·0 (2298·2 to 2385·6)	563·0 (540·7 to 587·2)	340·7 (322·1 to 361·0)	0·0 (0·0 to 0·0)
Canada	1998	3018·1 (2972·1 to 3066·1)	2184·0 (2143·3 to 2221·9)	517·2 (495·5 to 538·2)	317·0 (300·3 to 334·2)	0·0 (0·0 to 0·0)	3411·9 (3359·9 to 3466·2)	2469·0 (2423·0 to 2511·9)	584·6 (560·1 to 608·4)	358·3 (339·5 to 377·8)	0·0 (0·0 to 0·0)

Canada	1999	3121·4 (3079·1 to 3167·4)	2264·4 (2225·9 to 2302·8)	530·4 (510·9 to 552·0)	326·5 (312·4 to 340·2)	0·0 (0·0 to 0·0)	3528·6 (3480·9 to 3580·6)	2559·9 (2516·4 to 2603·2)	599·7 (577·6 to 624·0)	369·1 (353·1 to 384·6)	0·0 (0·0 to 0·0)
Canada	2000	3256·3 (3217·6 to 3297·4)	2370·6 (2336·3 to 2405·9)	540·6 (523·0 to 559·7)	345·1 (337·2 to 353·8)	0·0 (0·0 to 0·0)	3681·2 (3637·4 to 3727·6)	2679·9 (2641·1 to 2719·8)	611·1 (591·2 to 632·7)	390·1 (381·2 to 400·0)	0·0 (0·0 to 0·0)
Canada	2001	3430·0 (3393·9 to 3471·9)	2494·3 (2460·9 to 2530·7)	551·1 (535·9 to 567·1)	384·7 (377·2 to 392·2)	0·0 (0·0 to 0·0)	3877·6 (3836·7 to 3924·9)	2819·7 (2782·0 to 2860·9)	623·0 (605·8 to 641·1)	434·9 (426·4 to 443·4)	0·0 (0·0 to 0·0)
Canada	2002	3573·0 (3535·0 to 3612·6)	2594·8 (2563·2 to 2629·5)	561·9 (546·1 to 577·6)	416·4 (408·8 to 424·0)	0·0 (0·0 to 0·0)	4039·2 (3996·2 to 4084·0)	2933·3 (2897·6 to 2972·6)	635·2 (617·3 to 653·0)	470·7 (462·1 to 479·3)	0·0 (0·0 to 0·0)
Canada	2003	3699·7 (3660·5 to 3739·6)	2700·3 (2664·2 to 2736·0)	568·8 (553·0 to 585·7)	430·5 (423·3 to 438·0)	0·0 (0·0 to 0·0)	4182·4 (4138·1 to 4227·6)	3052·7 (3011·8 to 3093·0)	643·0 (625·1 to 662·1)	486·7 (478·5 to 495·1)	0·0 (0·0 to 0·0)
Canada	2004	3776·3 (3736·4 to 3819·6)	2757·7 (2720·1 to 2794·1)	579·3 (564·6 to 596·7)	439·4 (431·9 to 447·1)	0·0 (0·0 to 0·0)	4269·0 (4223·9 to 4317·9)	3117·5 (3075·1 to 3158·7)	654·8 (638·2 to 674·6)	496·7 (488·3 to 505·5)	0·0 (0·0 to 0·0)
Canada	2005	3873·7 (3833·6 to 3917·4)	2827·1 (2790·9 to 2867·7)	597·7 (582·3 to 614·2)	448·9 (441·3 to 456·4)	0·0 (0·0 to 0·0)	4379·2 (4333·8 to 4428·6)	3196·0 (3155·0 to 3241·9)	675·7 (658·3 to 694·4)	507·5 (498·9 to 516·0)	0·0 (0·0 to 0·0)
Canada	2006	4013·4 (3972·0 to 4055·1)	2923·3 (2887·4 to 2961·4)	621·3 (605·3 to 637·6)	468·8 (460·8 to 476·4)	0·0 (0·0 to 0·0)	4537·1 (4490·2 to 4584·2)	3304·7 (3264·2 to 3347·8)	702·4 (684·3 to 720·8)	530·0 (520·9 to 538·6)	0·0 (0·0 to 0·0)
Canada	2007	4116·8 (4075·1 to 4157·4)	3001·0 (2963·1 to 3039·0)	634·1 (618·0 to 652·6)	481·7 (474·1 to 489·5)	0·0 (0·0 to 0·0)	4653·9 (4606·8 to 4699·8)	3392·5 (3349·8 to 3435·6)	716·8 (698·6 to 737·7)	544·6 (536·0 to 553·4)	0·0 (0·0 to 0·0)
Canada	2008	4207·1 (4168·2 to 4247·2)	3072·6 (3036·4 to 3107·4)	644·9 (627·2 to 661·7)	489·6 (482·2 to 497·7)	0·0 (0·0 to 0·0)	4756·0 (4712·1 to 4801·4)	3473·5 (3432·6 to 3512·9)	729·0 (709·0 to 748·0)	553·5 (545·1 to 562·6)	0·0 (0·0 to 0·0)
Canada	2009	4460·7 (4421·2 to 4498·8)	3273·7 (3238·4 to 3308·7)	669·2 (653·0 to 685·6)	517·8 (509·9 to 525·8)	0·0 (0·0 to 0·0)	5042·7 (4998·0 to 5085·8)	3700·9 (3660·9 to 3740·4)	756·5 (738·2 to 775·1)	585·3 (576·4 to 594·4)	0·0 (0·0 to 0·0)
Canada	2010	4539·0 (4497·0 to 4579·8)	3333·2 (3294·5 to 3370·8)	678·0 (661·3 to 696·6)	527·8 (519·8 to 535·5)	0·0 (0·0 to 0·0)	5131·3 (5083·8 to 5177·4)	3768·1 (3724·4 to 3810·7)	766·5 (747·5 to 787·5)	596·7 (587·6 to 605·4)	0·0 (0·0 to 0·0)
Canada	2011	4514·9 (4472·8 to 4558·1)	3328·8 (3291·3 to 3366·9)	661·1 (643·5 to 677·4)	525·1 (516·9 to 532·7)	0·0 (0·0 to 0·0)	5104·0 (5056·5 to 5152·8)	3763·1 (3720·7 to 3806·2)	747·3 (727·4 to 765·8)	593·6 (584·3 to 602·2)	0·0 (0·0 to 0·0)
Canada	2012	4563·3 (4523·6 to 4604·6)	3380·3 (3344·9 to 3417·6)	651·3 (635·0 to 667·9)	531·7 (524·3 to 539·6)	0·0 (0·0 to 0·0)	5158·7 (5113·8 to 5205·4)	3821·3 (3781·3 to 3863·5)	736·3 (717·9 to 755·0)	601·1 (592·7 to 610·1)	0·0 (0·0 to 0·0)

Canada	2013	4605·2 (4563·1 to 4647·5)	3414·5 (3376·8 to 3451·8)	651·7 (635·5 to 670·2)	539·0 (531·7 to 547·2)	0·0 (0·0 to 0·0)	5206·1 (5158·5 to 5253·9)	3860·0 (3817·4 to 3902·2)	736·7 (718·4 to 757·6)	609·4 (601·0 to 618·6)	0·0 (0·0 to 0·0)
Canada	2014	4637·3 (4597·8 to 4676·3)	3422·7 (3388·9 to 3459·1)	668·9 (652·4 to 686·7)	545·7 (538·3 to 553·3)	0·0 (0·0 to 0·0)	5242·3 (5197·7 to 5286·5)	3869·3 (3831·1 to 3910·4)	756·2 (737·6 to 776·3)	616·9 (608·6 to 625·4)	0·0 (0·0 to 0·0)
Canada	2015	4831·9 (4791·5 to 4870·9)	3563·7 (3527·4 to 3597·3)	702·0 (685·3 to 718·9)	566·3 (558·3 to 574·4)	0·0 (0·0 to 0·0)	5462·3 (5416·7 to 5506·5)	4028·6 (3987·7 to 4066·7)	793·5 (774·7 to 812·7)	640·2 (631·2 to 649·3)	0·0 (0·0 to 0·0)
Canada	2016	4946·0 (4904·3 to 4989·7)	3623·3 (3588·3 to 3659·3)	738·1 (720·6 to 756·2)	584·6 (576·9 to 592·9)	0·0 (0·0 to 0·0)	5591·4 (5544·3 to 5640·8)	4096·1 (4056·5 to 4136·8)	834·4 (814·7 to 854·9)	660·9 (652·2 to 670·2)	0·0 (0·0 to 0·0)
Canada	2017	4994·3 (4950·6 to 5036·6)	3655·4 (3615·7 to 3695·1)	749·1 (732·3 to 767·7)	589·8 (581·9 to 598·2)	0·0 (0·0 to 0·0)	5645·9 (5596·6 to 5693·8)	4132·3 (4087·5 to 4177·2)	846·8 (827·8 to 867·9)	666·8 (657·8 to 676·2)	0·0 (0·0 to 0·0)
Canada	2018	5054·4 (5006·5 to 5103·5)	3711·6 (3669·5 to 3756·9)	747·8 (725·4 to 770·1)	595·0 (585·8 to 604·1)	0·0 (0·0 to 0·0)	5713·9 (5659·7 to 5769·4)	4195·9 (4148·2 to 4247·1)	845·4 (820·0 to 870·6)	672·6 (662·2 to 682·9)	0·0 (0·0 to 0·0)
Canada	2019	5162·9 (5103·8 to 5218·1)	3802·3 (3746·7 to 3853·1)	750·5 (728·3 to 773·1)	610·1 (599·4 to 620·6)	0·0 (0·0 to 0·0)	5836·6 (5769·7 to 5899·0)	4298·5 (4235·6 to 4355·8)	848·4 (823·3 to 873·9)	689·7 (677·6 to 701·5)	0·0 (0·0 to 0·0)
Canada	2020	6165·1 (6076·5 to 6250·7)	4895·5 (4808·3 to 4977·8)	693·8 (673·1 to 714·5)	575·8 (563·2 to 590·0)	0·0 (0·0 to 0·0)	6969·5 (6869·4 to 7066·3)	5534·2 (5435·7 to 5627·3)	784·3 (760·9 to 807·8)	651·0 (636·7 to 667·0)	0·0 (0·0 to 0·0)
Canada	2021	5935·9 (5851·7 to 6018·3)	4705·2 (4621·4 to 4784·3)	666·3 (646·3 to 686·4)	564·4 (549·4 to 580·0)	0·0 (0·0 to 0·0)	6710·4 (6615·2 to 6803·6)	5319·1 (5224·4 to 5408·6)	753·2 (730·6 to 775·9)	638·0 (621·1 to 655·7)	0·0 (0·0 to 0·0)
Canada	2022	4782·0 (4703·8 to 4857·9)	3538·7 (3460·6 to 3610·8)	665·9 (646·0 to 685·8)	577·3 (561·1 to 594·2)	0·0 (0·0 to 0·0)	5405·9 (5317·6 to 5491·8)	4000·4 (3912·1 to 4081·9)	752·8 (730·3 to 775·3)	652·7 (634·3 to 671·8)	0·0 (0·0 to 0·0)
Canada	2023	4831·5 (4749·8 to 4914·6)	3574·5 (3491·8 to 3653·9)	665·2 (645·6 to 685·3)	591·8 (574·2 to 610·1)	0·0 (0·0 to 0·0)	5461·9 (5369·5 to 5555·8)	4040·8 (3947·4 to 4130·7)	752·0 (729·8 to 774·8)	669·0 (649·1 to 689·7)	0·0 (0·0 to 0·0)
Canada	2024	4879·8 (4788·8 to 4973·8)	3609·0 (3519·5 to 3698·2)	664·3 (644·4 to 684·0)	606·5 (585·1 to 628·0)	0·0 (0·0 to 0·0)	5516·5 (5413·6 to 5622·8)	4079·9 (3978·7 to 4180·8)	750·9 (728·5 to 773·3)	685·6 (661·4 to 709·9)	0·0 (0·0 to 0·0)
Canada	2025	4928·8 (4826·5 to 5037·9)	3644·0 (3546·3 to 3748·3)	663·4 (643·5 to 683·4)	621·4 (595·1 to 648·7)	0·0 (0·0 to 0·0)	5571·9 (5456·2 to 5695·2)	4119·5 (4009·0 to 4237·4)	749·9 (727·4 to 772·6)	702·5 (672·7 to 733·4)	0·0 (0·0 to 0·0)
Canada	2026	4980·8 (4863·3 to 5106·7)	3681·2 (3568·8 to 3800·1)	662·8 (642·8 to 682·7)	636·8 (604·9 to 671·2)	0·0 (0·0 to 0·0)	5630·7 (5497·8 to 5773·0)	4161·5 (4034·5 to 4295·9)	749·3 (726·7 to 771·8)	719·9 (683·8 to 758·7)	0·0 (0·0 to 0·0)

Canada	2027	5037·4 (4898·4 to 5181·1)	3722·1 (3589·9 to 3860·2)	662·6 (642·6 to 682·6)	652·6 (614·6 to 693·9)	0·0 (0·0 to 0·0)	5694·6 (5537·5 to 5857·1)	4207·7 (4058·3 to 4363·9)	749·1 (726·5 to 771·6)	737·8 (694·8 to 784·4)	0·0 (0·0 to 0·0)
Canada	2028	5098·7 (4942·5 to 5261·2)	3766·9 (3611·9 to 3920·1)	662·8 (643·0 to 683·3)	668·9 (629·4 to 713·1)	0·0 (0·0 to 0·0)	5764·0 (5587·4 to 5947·6)	4258·4 (4083·2 to 4431·6)	749·3 (727·0 to 772·4)	756·2 (711·5 to 806·1)	0·0 (0·0 to 0·0)
Canada	2029	5162·1 (5002·7 to 5329·3)	3813·3 (3657·3 to 3975·6)	663·3 (643·5 to 683·7)	685·5 (643·7 to 730·1)	0·0 (0·0 to 0·0)	5835·6 (5655·4 to 6024·7)	4310·8 (4134·5 to 4494·3)	749·9 (727·5 to 772·9)	775·0 (727·7 to 825·4)	0·0 (0·0 to 0·0)
Canada	2030	5229·8 (5050·7 to 5417·0)	3862·8 (3685·1 to 4045·2)	664·1 (644·1 to 684·3)	702·8 (659·4 to 747·3)	0·0 (0·0 to 0·0)	5912·2 (5709·7 to 6123·8)	4366·8 (4165·9 to 4573·0)	750·8 (728·1 to 773·5)	794·5 (745·4 to 844·8)	0·0 (0·0 to 0·0)
Canada	2031	5302·8 (5101·2 to 5514·7)	3916·6 (3714·1 to 4119·5)	665·4 (645·1 to 685·6)	720·9 (676·4 to 769·2)	0·0 (0·0 to 0·0)	5994·7 (5766·8 to 6234·3)	4427·6 (4198·7 to 4657·0)	752·2 (729·3 to 775·0)	814·9 (764·7 to 869·6)	0·0 (0·0 to 0·0)
Canada	2032	5377·9 (5155·5 to 5609·1)	3972·0 (3748·5 to 4192·4)	666·9 (646·6 to 686·9)	739·0 (692·3 to 787·9)	0·0 (0·0 to 0·0)	6079·6 (5828·2 to 6341·0)	4490·3 (4237·6 to 4739·4)	753·9 (731·0 to 776·6)	835·4 (782·6 to 890·7)	0·0 (0·0 to 0·0)
Canada	2033	5455·6 (5212·6 to 5707·3)	4029·6 (3782·3 to 4272·4)	668·6 (648·1 to 688·6)	757·4 (707·9 to 807·6)	0·0 (0·0 to 0·0)	6167·5 (5892·8 to 6452·0)	4555·4 (4275·9 to 4829·9)	755·8 (732·7 to 778·5)	856·2 (800·3 to 913·0)	0·0 (0·0 to 0·0)
Canada	2034	5537·0 (5272·0 to 5811·8)	4089·9 (3819·2 to 4354·7)	670·7 (650·1 to 690·7)	776·4 (722·4 to 831·7)	0·0 (0·0 to 0·0)	6259·5 (5959·9 to 6570·1)	4623·6 (4317·5 to 4922·9)	758·2 (735·0 to 780·8)	877·8 (816·7 to 940·2)	0·0 (0·0 to 0·0)
Canada	2035	5621·9 (5333·0 to 5921·9)	4152·7 (3859·1 to 4439·1)	673·1 (652·4 to 693·0)	796·1 (739·1 to 856·4)	0·0 (0·0 to 0·0)	6355·4 (6028·8 to 6694·6)	4694·5 (4362·6 to 5018·4)	760·9 (737·5 to 783·4)	900·0 (835·5 to 968·2)	0·0 (0·0 to 0·0)
Canada	2036	5708·3 (5395·9 to 6028·3)	4216·4 (3899·5 to 4525·6)	675·7 (654·7 to 695·5)	816·2 (755·6 to 882·3)	0·0 (0·0 to 0·0)	6453·1 (6099·9 to 6814·8)	4766·6 (4408·3 to 5116·1)	763·8 (740·1 to 786·2)	922·7 (854·2 to 997·4)	0·0 (0·0 to 0·0)
Canada	2037	5796·7 (5464·5 to 6139·1)	4281·4 (3943·5 to 4610·5)	678·4 (657·2 to 698·7)	836·9 (769·6 to 907·8)	0·0 (0·0 to 0·0)	6553·1 (6177·5 to 6940·1)	4840·0 (4458·1 to 5212·1)	766·9 (743·0 to 789·9)	946·2 (870·0 to 1026·3)	0·0 (0·0 to 0·0)
Canada	2038	5887·3 (5532·0 to 6250·1)	4347·6 (3989·4 to 4697·3)	681·4 (660·0 to 701·9)	858·3 (784·3 to 938·2)	0·0 (0·0 to 0·0)	6655·4 (6253·8 to 7065·6)	4914·8 (4509·9 to 5310·2)	770·3 (746·1 to 793·5)	970·3 (886·7 to 1060·7)	0·0 (0·0 to 0·0)
Canada	2039	5980·7 (5605·3 to 6367·3)	4415·7 (4037·8 to 4789·7)	684·6 (662·9 to 705·5)	880·4 (799·1 to 968·7)	0·0 (0·0 to 0·0)	6761·1 (6336·7 to 7198·1)	4991·9 (4564·6 to 5414·6)	774·0 (749·4 to 797·5)	995·3 (903·4 to 1095·1)	0·0 (0·0 to 0·0)
Canada	2040	6078·0 (5682·6 to 6486·9)	4486·3 (4089·2 to 4876·5)	688·3 (666·1 to 709·5)	903·4 (813·2 to 1001·4)	0·0 (0·0 to 0·0)	6871·1 (6424·0 to 7333·3)	5071·7 (4622·7 to 5512·8)	778·1 (753·0 to 802·0)	1021·3 (919·3 to 1132·1)	0·0 (0·0 to 0·0)

Canada	2041	6176·5 (5761·5 to 6602·3)	4557·4 (4141·8 to 4963·5)	692·0 (669·7 to 713·3)	927·1 (826·5 to 1036·2)	0·0 (0·0 to 0·0)	6982·4 (6513·2 to 7463·8)	5152·1 (4682·2 to 5611·1)	782·3 (757·0 to 806·4)	1048·0 (934·4 to 1171·4)	0·0 (0·0 to 0·0)
Canada	2042	6275·9 (5843·4 to 6723·3)	4628·6 (4197·9 to 5049·0)	695·8 (673·2 to 717·3)	951·5 (841·0 to 1071·5)	0·0 (0·0 to 0·0)	7094·7 (6605·9 to 7600·6)	5232·6 (4745·6 to 5707·8)	786·6 (761·0 to 810·8)	1075·6 (950·7 to 1211·3)	0·0 (0·0 to 0·0)
Canada	2043	6374·9 (5926·7 to 6843·8)	4698·9 (4254·1 to 5136·3)	699·5 (676·6 to 721·2)	976·5 (853·0 to 1110·0)	0·0 (0·0 to 0·0)	7206·7 (6700·0 to 7736·8)	5312·0 (4809·2 to 5806·5)	790·8 (764·9 to 815·3)	1103·9 (964·3 to 1254·8)	0·0 (0·0 to 0·0)
Canada	2044	6473·6 (6006·1 to 6961·1)	4768·4 (4307·7 to 5221·1)	703·2 (680·0 to 725·2)	1002·0 (866·5 to 1150·0)	0·0 (0·0 to 0·0)	7318·3 (6789·8 to 7869·4)	5390·6 (4869·7 to 5902·4)	795·0 (768·7 to 819·8)	1132·8 (979·5 to 1300·1)	0·0 (0·0 to 0·0)
Canada	2045	6573·8 (6084·6 to 7081·8)	4838·3 (4362·4 to 5303·8)	707·0 (683·5 to 729·6)	1028·4 (877·9 to 1190·1)	0·0 (0·0 to 0·0)	7431·5 (6878·5 to 8005·9)	5469·6 (4931·7 to 5995·9)	799·3 (772·7 to 824·8)	1162·6 (992·4 to 1345·4)	0·0 (0·0 to 0·0)
Canada	2046	6674·2 (6176·8 to 7197·3)	4907·8 (4417·7 to 5385·4)	710·9 (687·2 to 734·1)	1055·5 (893·0 to 1232·3)	0·0 (0·0 to 0·0)	7545·0 (6982·8 to 8136·4)	5548·1 (4994·2 to 6088·1)	803·6 (776·9 to 829·9)	1193·2 (1009·5 to 1393·1)	0·0 (0·0 to 0·0)
Canada	2047	6774·1 (6261·5 to 7315·0)	4976·3 (4474·6 to 5465·2)	714·7 (690·8 to 737·9)	1083·1 (908·1 to 1275·0)	0·0 (0·0 to 0·0)	7657·9 (7078·5 to 8269·5)	5625·6 (5058·4 to 6178·3)	807·9 (780·9 to 834·2)	1224·5 (1026·6 to 1441·3)	0·0 (0·0 to 0·0)
Canada	2048	6875·5 (6353·2 to 7434·3)	5045·2 (4533·2 to 5544·7)	718·6 (694·5 to 741·7)	1111·7 (923·5 to 1323·0)	0·0 (0·0 to 0·0)	7772·7 (7182·1 to 8404·3)	5703·5 (5124·7 to 6268·2)	812·4 (785·1 to 838·5)	1256·8 (1044·0 to 1495·6)	0·0 (0·0 to 0·0)
Canada	2049	6976·2 (6438·7 to 7543·0)	5112·8 (4591·9 to 5621·9)	722·5 (697·9 to 745·8)	1141·0 (940·8 to 1370·4)	0·0 (0·0 to 0·0)	7886·4 (7278·9 to 8527·2)	5779·9 (5191·1 to 6355·5)	816·7 (789·0 to 843·1)	1289·8 (1063·5 to 1549·2)	0·0 (0·0 to 0·0)
Canada	2050	7076·9 (6530·8 to 7661·4)	5179·6 (4651·3 to 5697·5)	726·3 (701·3 to 750·1)	1171·0 (958·3 to 1419·9)	0·0 (0·0 to 0·0)	8000·3 (7382·9 to 8661·1)	5855·5 (5258·2 to 6440·9)	821·1 (792·8 to 848·0)	1323·8 (1083·4 to 1605·2)	0·0 (0·0 to 0·0)
Switzerland	1995	6986·9 (6851·9 to 7129·4)	1790·1 (1720·2 to 1861·2)	2091·8 (2004·8 to 2173·0)	3105·1 (3019·5 to 3194·6)	0·0 (0·0 to 0·0)	5834·7 (5721·9 to 5953·7)	1494·9 (1436·6 to 1554·2)	1746·8 (1674·2 to 1814·6)	2593·0 (2521·6 to 2667·8)	0·0 (0·0 to 0·0)
Switzerland	1996	6936·1 (6821·2 to 7046·1)	1839·1 (1786·7 to 1892·1)	2093·8 (2032·1 to 2153·9)	3003·2 (2926·0 to 3080·3)	0·0 (0·0 to 0·0)	5792·3 (5696·3 to 5884·1)	1535·8 (1492·0 to 1580·1)	1748·5 (1697·0 to 1798·7)	2507·9 (2443·5 to 2572·3)	0·0 (0·0 to 0·0)
Switzerland	1997	7084·1 (6975·4 to 7200·6)	1890·6 (1835·0 to 1943·9)	2110·6 (2053·3 to 2170·7)	3082·9 (3010·0 to 3164·3)	0·0 (0·0 to 0·0)	5915·9 (5825·1 to 6013·1)	1578·8 (1532·4 to 1623·3)	1762·5 (1714·7 to 1812·8)	2574·5 (2513·6 to 2642·4)	0·0 (0·0 to 0·0)
Switzerland	1998	7183·6 (7078·9 to 7295·9)	1936·2 (1881·5 to 1993·8)	2131·8 (2071·4 to 2191·3)	3115·6 (3043·7 to 3188·0)	0·0 (0·0 to 0·0)	5999·0 (5911·5 to 6092·7)	1616·9 (1571·2 to 1665·0)	1780·2 (1729·8 to 1830·0)	2601·8 (2541·8 to 2662·3)	0·0 (0·0 to 0·0)

Switzerland	1999	7212·5 (7106·2 to 7318·2)	1979·3 (1926·6 to 2032·7)	2142·1 (2081·8 to 2201·4)	3091·0 (3017·0 to 3167·4)	0·0 (0·0 to 0·0)	6023·1 (5934·3 to 6111·3)	1652·9 (1608·9 to 1697·5)	1788·9 (1738·5 to 1838·4)	2581·3 (2519·4 to 2645·0)	0·0 (0·0 to 0·0)
Switzerland	2000	7005·8 (6903·1 to 7118·8)	2023·4 (1972·4 to 2073·2)	2135·4 (2078·3 to 2195·4)	2846·9 (2774·4 to 2922·1)	0·0 (0·0 to 0·0)	5850·4 (5764·7 to 5944·9)	1689·8 (1647·1 to 1731·3)	1783·3 (1735·6 to 1833·4)	2377·4 (2316·8 to 2440·2)	0·0 (0·0 to 0·0)
Switzerland	2001	7068·0 (6962·8 to 7169·0)	2116·3 (2064·9 to 2166·3)	2135·4 (2078·9 to 2194·6)	2816·3 (2745·2 to 2884·2)	0·0 (0·0 to 0·0)	5902·4 (5814·6 to 5986·7)	1767·3 (1724·4 to 1809·0)	1783·2 (1736·0 to 1832·7)	2351·9 (2292·5 to 2408·6)	0·0 (0·0 to 0·0)
Switzerland	2002	7438·5 (7337·9 to 7547·0)	2322·9 (2270·4 to 2374·3)	2175·4 (2115·7 to 2232·1)	2940·2 (2870·0 to 3007·0)	0·0 (0·0 to 0·0)	6211·8 (6127·8 to 6302·4)	1939·8 (1896·0 to 1982·7)	1816·7 (1766·8 to 1864·0)	2455·3 (2396·7 to 2511·1)	0·0 (0·0 to 0·0)
Switzerland	2003	7548·4 (7442·7 to 7656·0)	2360·3 (2308·8 to 2414·5)	2174·1 (2114·6 to 2234·2)	3013·9 (2943·3 to 3085·2)	0·0 (0·0 to 0·0)	6303·6 (6215·3 to 6393·4)	1971·1 (1928·1 to 2016·3)	1815·6 (1765·9 to 1865·8)	2516·9 (2457·9 to 2576·4)	0·0 (0·0 to 0·0)
Switzerland	2004	7734·9 (7624·2 to 7847·4)	2405·5 (2354·3 to 2461·9)	2186·8 (2130·2 to 2246·9)	3142·5 (3073·6 to 3214·9)	0·0 (0·0 to 0·0)	6459·3 (6366·9 to 6553·3)	2008·8 (1966·0 to 2055·9)	1826·2 (1778·9 to 1876·3)	2624·3 (2566·7 to 2684·7)	0·0 (0·0 to 0·0)
Switzerland	2005	7784·2 (7672·0 to 7896·1)	2402·4 (2350·2 to 2457·6)	2141·7 (2084·4 to 2200·4)	3240·2 (3169·6 to 3313·6)	0·0 (0·0 to 0·0)	6500·5 (6406·8 to 6593·9)	2006·2 (1962·7 to 2052·4)	1788·5 (1740·7 to 1837·5)	2705·8 (2646·9 to 2767·1)	0·0 (0·0 to 0·0)
Switzerland	2006	7683·2 (7577·3 to 7799·4)	2358·7 (2301·8 to 2415·6)	2098·5 (2038·4 to 2157·2)	3225·9 (3154·8 to 3296·8)	0·0 (0·0 to 0·0)	6416·1 (6327·7 to 6513·2)	1969·8 (1922·2 to 2017·2)	1752·4 (1702·3 to 1801·5)	2693·9 (2634·5 to 2753·1)	0·0 (0·0 to 0·0)
Switzerland	2007	7736·8 (7632·6 to 7841·0)	2390·1 (2336·3 to 2443·7)	2098·0 (2040·3 to 2156·0)	3248·7 (3179·5 to 3318·7)	0·0 (0·0 to 0·0)	6460·9 (6373·9 to 6547·9)	1996·0 (1951·0 to 2040·7)	1752·0 (1703·8 to 1800·4)	2712·9 (2655·1 to 2771·4)	0·0 (0·0 to 0·0)
Switzerland	2008	7954·1 (7847·4 to 8070·0)	2504·1 (2452·9 to 2556·0)	2119·0 (2064·0 to 2181·0)	3331·0 (3256·8 to 3400·8)	0·0 (0·0 to 0·0)	6642·4 (6553·3 to 6739·1)	2091·2 (2048·4 to 2134·5)	1769·5 (1723·6 to 1821·3)	2781·7 (2719·7 to 2839·9)	0·0 (0·0 to 0·0)
Switzerland	2009	8143·0 (8038·5 to 8253·7)	2608·7 (2554·5 to 2662·6)	2136·7 (2080·8 to 2196·2)	3397·6 (3326·7 to 3469·2)	0·0 (0·0 to 0·0)	6800·1 (6712·8 to 6892·6)	2178·5 (2133·2 to 2223·5)	1784·3 (1737·7 to 1834·0)	2837·3 (2778·1 to 2897·1)	0·0 (0·0 to 0·0)
Switzerland	2010	8235·4 (8132·0 to 8346·9)	2676·4 (2622·6 to 2732·1)	2143·6 (2086·6 to 2205·0)	3415·4 (3344·2 to 3488·2)	0·0 (0·0 to 0·0)	6877·3 (6790·9 to 6970·4)	2235·0 (2190·1 to 2281·5)	1790·1 (1742·5 to 1841·4)	2852·2 (2792·7 to 2913·0)	0·0 (0·0 to 0·0)
Switzerland	2011	8360·5 (8252·5 to 8475·2)	2764·6 (2709·8 to 2820·9)	2156·6 (2099·1 to 2215·0)	3439·3 (3367·6 to 3512·7)	0·0 (0·0 to 0·0)	6981·8 (6891·6 to 7077·5)	2308·7 (2263·0 to 2355·7)	1800·9 (1752·9 to 1849·7)	2872·2 (2812·2 to 2933·5)	0·0 (0·0 to 0·0)
Switzerland	2012	8516·8 (8412·9 to 8623·8)	2842·0 (2787·8 to 2901·2)	2189·8 (2136·7 to 2250·7)	3485·0 (3417·8 to 3552·2)	0·0 (0·0 to 0·0)	7112·3 (7025·5 to 7201·7)	2373·3 (2328·0 to 2422·8)	1828·6 (1784·3 to 1879·6)	2910·3 (2854·2 to 2966·4)	0·0 (0·0 to 0·0)

Switzerland	2013	8807·6 (8700·5 to 8912·8)	2899·9 (2844·9 to 2958·1)	2242·0 (2182·6 to 2300·8)	3665·7 (3595·4 to 3736·9)	0·0 (0·0 to 0·0)	7355·1 (7265·7 to 7443·0)	2421·7 (2375·8 to 2470·3)	1872·2 (1822·6 to 1921·4)	3061·2 (3002·5 to 3120·6)	0·0 (0·0 to 0·0)
Switzerland	2014	9018·3 (8908·8 to 9120·4)	2903·3 (2849·7 to 2957·4)	2326·5 (2269·3 to 2386·6)	3788·5 (3714·9 to 3858·5)	0·0 (0·0 to 0·0)	7531·1 (7439·6 to 7616·3)	2424·5 (2379·7 to 2469·7)	1942·8 (1895·1 to 1993·0)	3163·7 (3102·3 to 3222·2)	0·0 (0·0 to 0·0)
Switzerland	2015	9361·1 (9252·7 to 9480·5)	2964·8 (2911·2 to 3014·0)	2434·4 (2375·2 to 2495·3)	3961·9 (3889·3 to 4036·5)	0·0 (0·0 to 0·0)	7817·4 (7726·8 to 7917·0)	2475·9 (2431·1 to 2517·0)	2033·0 (1983·5 to 2083·8)	3308·5 (3247·9 to 3370·8)	0·0 (0·0 to 0·0)
Switzerland	2016	9681·7 (9568·6 to 9788·4)	3024·2 (2970·8 to 3079·7)	2549·3 (2486·9 to 2612·9)	4108·2 (4039·5 to 4178·1)	0·0 (0·0 to 0·0)	8085·1 (7990·7 to 8174·2)	2525·5 (2480·9 to 2571·8)	2128·9 (2076·8 to 2182·0)	3430·7 (3373·3 to 3489·1)	0·0 (0·0 to 0·0)
Switzerland	2017	9935·2 (9824·4 to 10049·4)	3107·0 (3048·1 to 3162·6)	2659·9 (2591·2 to 2725·7)	4168·3 (4094·3 to 4246·7)	0·0 (0·0 to 0·0)	8296·8 (8204·3 to 8392·2)	2594·6 (2545·5 to 2641·0)	2221·3 (2163·8 to 2276·2)	3480·9 (3419·1 to 3546·4)	0·0 (0·0 to 0·0)
Switzerland	2018	10071·6 (9932·7 to 10207·0)	3156·2 (3082·5 to 3228·8)	2798·5 (2719·6 to 2878·4)	4117·0 (4027·3 to 4211·1)	0·0 (0·0 to 0·0)	8410·7 (8294·7 to 8523·8)	2635·7 (2574·2 to 2696·3)	2337·0 (2271·1 to 2403·7)	3438·0 (3363·2 to 3516·7)	0·0 (0·0 to 0·0)
Switzerland	2019	10203·5 (10056·9 to 10351·2)	3184·0 (3104·5 to 3268·2)	2805·8 (2722·4 to 2892·7)	4213·7 (4115·8 to 4309·8)	0·0 (0·0 to 0·0)	8520·8 (8398·4 to 8644·1)	2658·9 (2592·5 to 2729·2)	2343·1 (2273·4 to 2415·7)	3518·8 (3437·1 to 3599·0)	0·0 (0·0 to 0·0)
Switzerland	2020	10227·0 (10082·9 to 10375·5)	3474·0 (3395·1 to 3557·4)	2662·5 (2583·3 to 2743·6)	4090·5 (3992·8 to 4194·4)	0·0 (0·0 to 0·0)	8540·5 (8420·1 to 8664·5)	2901·1 (2835·2 to 2970·7)	2223·4 (2157·3 to 2291·1)	3415·9 (3334·4 to 3502·7)	0·0 (0·0 to 0·0)
Switzerland	2021	9801·0 (9651·6 to 9960·9)	3290·4 (3198·1 to 3387·2)	2532·2 (2456·8 to 2610·6)	3978·4 (3876·5 to 4084·0)	0·0 (0·0 to 0·0)	8184·7 (8060·0 to 8318·3)	2747·8 (2670·7 to 2828·6)	2114·6 (2051·6 to 2180·1)	3322·3 (3237·2 to 3410·5)	0·0 (0·0 to 0·0)
Switzerland	2022	9523·5 (9360·2 to 9689·4)	2939·4 (2849·2 to 3030·1)	2525·6 (2450·2 to 2603·7)	4058·6 (3945·7 to 4179·1)	0·0 (0·0 to 0·0)	7953·0 (7816·6 to 8091·5)	2454·6 (2379·3 to 2530·4)	2109·1 (2046·1 to 2174·3)	3389·3 (3295·0 to 3490·0)	0·0 (0·0 to 0·0)
Switzerland	2023	9621·7 (9449·7 to 9804·0)	2964·9 (2876·2 to 3056·2)	2517·8 (2444·1 to 2595·1)	4139·0 (4006·2 to 4275·4)	0·0 (0·0 to 0·0)	8035·0 (7891·4 to 8187·2)	2476·0 (2401·9 to 2552·2)	2102·6 (2041·0 to 2167·1)	3456·4 (3345·5 to 3570·4)	0·0 (0·0 to 0·0)
Switzerland	2024	9714·6 (9527·7 to 9914·4)	2991·5 (2897·9 to 3091·6)	2507·5 (2434·3 to 2583·8)	4215·6 (4070·1 to 4375·8)	0·0 (0·0 to 0·0)	8112·6 (7956·5 to 8279·4)	2498·2 (2420·0 to 2581·8)	2094·0 (2032·9 to 2157·7)	3520·4 (3398·9 to 3654·2)	0·0 (0·0 to 0·0)
Switzerland	2025	9822·0 (9627·1 to 10028·4)	3035·2 (2929·2 to 3135·7)	2496·4 (2423·4 to 2572·1)	4290·4 (4143·5 to 4454·5)	0·0 (0·0 to 0·0)	8202·3 (8039·5 to 8374·6)	2534·7 (2446·1 to 2618·6)	2084·7 (2023·7 to 2148·0)	3582·9 (3460·2 to 3719·9)	0·0 (0·0 to 0·0)
Switzerland	2026	9932·6 (9730·0 to 10155·7)	3081·1 (2978·7 to 3188·9)	2485·1 (2413·1 to 2559·2)	4366·3 (4206·9 to 4540·8)	0·0 (0·0 to 0·0)	8294·6 (8125·4 to 8480·9)	2573·0 (2487·5 to 2663·0)	2075·3 (2015·2 to 2137·1)	3646·3 (3513·1 to 3792·0)	0·0 (0·0 to 0·0)

Switzerland	2027	10061·0 (9848·4 to 10288·1)	3131·1 (3021·1 to 3244·2)	2474·9 (2402·8 to 2550·2)	4455·0 (4280·5 to 4646·2)	0·0 (0·0 to 0·0)	8401·8 (8224·3 to 8591·5)	2614·8 (2522·9 to 2709·2)	2066·7 (2006·6 to 2129·6)	3720·3 (3574·6 to 3880·0)	0·0 (0·0 to 0·0)
Switzerland	2028	10191·1 (9974·3 to 10429·8)	3171·8 (3052·3 to 3287·3)	2464·1 (2392·5 to 2538·9)	4555·2 (4381·0 to 4758·3)	0·0 (0·0 to 0·0)	8510·5 (8329·4 to 8709·8)	2648·7 (2548·9 to 2745·2)	2057·7 (1997·9 to 2120·2)	3804·0 (3658·5 to 3973·6)	0·0 (0·0 to 0·0)
Switzerland	2029	10309·2 (10078·0 to 10560·8)	3199·6 (3072·8 to 3318·6)	2452·2 (2381·0 to 2526·4)	4657·4 (4476·1 to 4872·6)	0·0 (0·0 to 0·0)	8609·1 (8416·1 to 8819·2)	2671·9 (2566·1 to 2771·4)	2047·8 (1988·3 to 2109·7)	3889·4 (3737·9 to 4069·0)	0·0 (0·0 to 0·0)
Switzerland	2030	10430·6 (10179·3 to 10712·2)	3227·9 (3098·8 to 3355·0)	2440·5 (2368·3 to 2514·2)	4762·1 (4543·9 to 5003·0)	0·0 (0·0 to 0·0)	8710·5 (8500·7 to 8945·6)	2695·6 (2587·7 to 2801·7)	2038·1 (1977·8 to 2099·6)	3976·8 (3794·6 to 4177·9)	0·0 (0·0 to 0·0)
Switzerland	2031	10566·2 (10280·1 to 10876·6)	3259·8 (3126·0 to 3393·2)	2434·6 (2363·4 to 2507·3)	4871·8 (4626·3 to 5141·3)	0·0 (0·0 to 0·0)	8823·7 (8584·8 to 9082·9)	2722·2 (2610·5 to 2833·7)	2033·1 (1973·7 to 2093·8)	4068·4 (3863·4 to 4293·4)	0·0 (0·0 to 0·0)
Switzerland	2032	10707·2 (10397·5 to 11049·7)	3293·3 (3151·6 to 3442·6)	2431·4 (2360·4 to 2503·5)	4982·4 (4698·7 to 5291·0)	0·0 (0·0 to 0·0)	8941·4 (8682·9 to 9227·5)	2750·2 (2631·9 to 2874·8)	2030·5 (1971·1 to 2090·6)	4160·7 (3923·8 to 4418·5)	0·0 (0·0 to 0·0)
Switzerland	2033	10859·6 (10514·2 to 11243·6)	3330·4 (3178·3 to 3490·3)	2430·8 (2359·2 to 2502·9)	5098·4 (4775·6 to 5445·8)	0·0 (0·0 to 0·0)	9068·7 (8780·3 to 9389·4)	2781·2 (2654·1 to 2914·7)	2029·9 (1970·1 to 2090·1)	4257·6 (3988·0 to 4547·7)	0·0 (0·0 to 0·0)
Switzerland	2034	11017·1 (10625·4 to 11443·8)	3368·5 (3204·3 to 3539·3)	2431·0 (2358·8 to 2504·7)	5217·6 (4857·9 to 5616·7)	0·0 (0·0 to 0·0)	9200·3 (8873·2 to 9556·6)	2813·0 (2675·9 to 2955·7)	2030·1 (1969·8 to 2091·6)	4357·1 (4056·8 to 4690·4)	0·0 (0·0 to 0·0)
Switzerland	2035	11181·7 (10743·1 to 11655·2)	3408·4 (3230·7 to 3598·3)	2432·5 (2359·7 to 2508·1)	5340·8 (4934·3 to 5778·1)	0·0 (0·0 to 0·0)	9337·7 (8971·5 to 9733·1)	2846·3 (2698·0 to 3004·9)	2031·4 (1970·5 to 2094·5)	4460·0 (4120·5 to 4825·2)	0·0 (0·0 to 0·0)
Switzerland	2036	11352·7 (10863·1 to 11873·7)	3449·9 (3257·2 to 3655·2)	2435·2 (2361·7 to 2511·7)	5467·6 (5024·4 to 5952·3)	0·0 (0·0 to 0·0)	9480·5 (9071·6 to 9915·6)	2880·9 (2720·0 to 3052·4)	2033·6 (1972·3 to 2097·5)	4565·9 (4195·8 to 4970·7)	0·0 (0·0 to 0·0)
Switzerland	2037	11533·5 (10998·2 to 12098·5)	3493·9 (3290·1 to 3716·2)	2439·6 (2366·1 to 2517·5)	5599·9 (5107·4 to 6139·9)	0·0 (0·0 to 0·0)	9631·5 (9184·4 to 10103·3)	2917·8 (2747·5 to 3103·4)	2037·3 (1975·9 to 2102·3)	4676·4 (4265·1 to 5127·3)	0·0 (0·0 to 0·0)
Switzerland	2038	11722·9 (11127·6 to 12343·4)	3540·4 (3327·2 to 3778·1)	2445·6 (2371·4 to 2524·5)	5736·9 (5197·9 to 6328·3)	0·0 (0·0 to 0·0)	9789·7 (9292·6 to 10307·8)	2956·6 (2778·5 to 3155·1)	2042·3 (1980·3 to 2108·2)	4790·8 (4340·8 to 5284·7)	0·0 (0·0 to 0·0)
Switzerland	2039	11923·1 (11283·7 to 12599·0)	3589·9 (3364·1 to 3838·5)	2453·4 (2378·1 to 2533·4)	5879·8 (5290·1 to 6525·5)	0·0 (0·0 to 0·0)	9956·9 (9422·9 to 10521·3)	2997·9 (2809·3 to 3205·4)	2048·8 (1985·9 to 2115·6)	4910·2 (4417·7 to 5449·4)	0·0 (0·0 to 0·0)
Switzerland	2040	12131·5 (11433·6 to 12868·6)	3641·0 (3401·8 to 3902·0)	2462·5 (2385·9 to 2542·2)	6027·9 (5394·7 to 6730·5)	0·0 (0·0 to 0·0)	10130·9 (9548·1 to 10746·5)	3040·6 (2840·8 to 3258·5)	2056·4 (1992·5 to 2122·9)	5033·9 (4505·0 to 5620·6)	0·0 (0·0 to 0·0)

Switzerland	2041	12338·9 (11550·8 to 13128·7)	3690·6 (3438·2 to 3963·0)	2470·9 (2393·0 to 2551·0)	6177·4 (5490·7 to 6942·3)	0·0 (0·0 to 0·0)	10304·1 (9646·0 to 10963·7)	3082·0 (2871·2 to 3309·4)	2063·4 (1998·3 to 2130·3)	5158·7 (4585·2 to 5797·4)	0·0 (0·0 to 0·0)
Switzerland	2042	12547·5 (11703·0 to 13401·2)	3739·2 (3475·5 to 4018·7)	2478·7 (2399·7 to 2559·1)	6329·6 (5587·2 to 7158·9)	0·0 (0·0 to 0·0)	10478·3 (9773·0 to 11191·2)	3122·6 (2902·3 to 3356·0)	2069·9 (2003·9 to 2137·1)	5285·8 (4665·8 to 5978·3)	0·0 (0·0 to 0·0)
Switzerland	2043	12754·6 (11844·9 to 13684·0)	3785·5 (3508·7 to 4080·4)	2485·5 (2405·3 to 2566·0)	6483·6 (5671·8 to 7366·3)	0·0 (0·0 to 0·0)	10651·3 (9891·6 to 11427·4)	3161·2 (2930·1 to 3407·5)	2075·7 (2008·7 to 2142·8)	5414·4 (4736·5 to 6151·5)	0·0 (0·0 to 0·0)
Switzerland	2044	12964·6 (11990·4 to 13963·8)	3831·3 (3543·3 to 4141·0)	2492·3 (2411·1 to 2573·3)	6641·0 (5756·8 to 7582·4)	0·0 (0·0 to 0·0)	10826·6 (10013·1 to 11661·0)	3199·5 (2958·9 to 3458·1)	2081·3 (2013·5 to 2149·0)	5545·8 (4807·5 to 6331·9)	0·0 (0·0 to 0·0)
Switzerland	2045	13170·9 (12130·3 to 14232·4)	3874·5 (3574·2 to 4193·1)	2497·9 (2415·3 to 2580·0)	6798·5 (5839·5 to 7795·8)	0·0 (0·0 to 0·0)	10998·8 (10129·9 to 11885·3)	3235·5 (2984·8 to 3501·6)	2085·9 (2017·0 to 2154·5)	5677·4 (4876·5 to 6510·2)	0·0 (0·0 to 0·0)
Switzerland	2046	13382·8 (12285·8 to 14510·6)	3918·0 (3606·9 to 4245·7)	2504·0 (2420·7 to 2587·4)	6960·7 (5932·7 to 8042·9)	0·0 (0·0 to 0·0)	11175·8 (10259·7 to 12117·6)	3271·9 (3012·1 to 3545·5)	2091·1 (2021·5 to 2160·7)	5812·8 (4954·3 to 6716·5)	0·0 (0·0 to 0·0)
Switzerland	2047	13588·9 (12417·8 to 14799·9)	3958·7 (3640·5 to 4295·0)	2508·6 (2425·2 to 2593·1)	7121·5 (6023·4 to 8290·0)	0·0 (0·0 to 0·0)	11347·9 (10370·0 to 12359·2)	3305·9 (3040·1 to 3586·7)	2094·9 (2025·2 to 2165·4)	5947·1 (5030·1 to 6922·9)	0·0 (0·0 to 0·0)
Switzerland	2048	13801·9 (12559·2 to 15099·1)	4000·2 (3675·7 to 4343·1)	2514·0 (2429·8 to 2599·0)	7287·8 (6116·7 to 8534·1)	0·0 (0·0 to 0·0)	11525·8 (10488·0 to 12609·1)	3340·5 (3069·6 to 3626·9)	2099·4 (2029·1 to 2170·4)	6086·0 (5108·0 to 7126·7)	0·0 (0·0 to 0·0)
Switzerland	2049	14019·0 (12691·6 to 15400·2)	4041·1 (3709·6 to 4390·3)	2519·5 (2434·4 to 2605·9)	7458·4 (6207·3 to 8806·7)	0·0 (0·0 to 0·0)	11707·1 (10598·6 to 12860·6)	3374·7 (3097·8 to 3666·3)	2104·0 (2033·0 to 2176·1)	6228·4 (5183·7 to 7354·4)	0·0 (0·0 to 0·0)
Switzerland	2050	14238·8 (12827·2 to 15690·1)	4081·3 (3745·3 to 4436·4)	2525·1 (2438·6 to 2612·2)	7632·5 (6296·5 to 9087·3)	0·0 (0·0 to 0·0)	11890·7 (10711·9 to 13102·6)	3408·2 (3127·7 to 3704·8)	2108·7 (2036·5 to 2181·4)	6373·8 (5258·1 to 7588·7)	0·0 (0·0 to 0·0)
Chile	1995	469·4 (439·0 to 505·4)	176·2 (159·0 to 194·3)	186·7 (168·9 to 207·5)	104·3 (87·7 to 123·2)	2·1 (2·1 to 2·1)	872·8 (816·3 to 939·9)	327·7 (295·6 to 361·3)	347·2 (314·2 to 386·0)	194·0 (163·0 to 229·1)	3·9 (3·9 to 3·9)
Chile	1996	527·6 (500·6 to 555·9)	196·3 (180·9 to 211·6)	214·5 (197·7 to 232·0)	114·8 (100·6 to 130·9)	2·0 (2·0 to 2·0)	981·2 (931·0 to 1033·8)	365·1 (336·4 to 393·5)	399·0 (367·7 to 431·5)	213·4 (187·2 to 243·4)	3·7 (3·7 to 3·7)
Chile	1997	581·6 (557·7 to 606·9)	212·5 (198·0 to 226·8)	243·7 (228·3 to 260·6)	121·5 (108·5 to 135·8)	4·0 (4·0 to 4·0)	1081·6 (1037·1 to 1128·7)	395·2 (368·2 to 421·8)	453·1 (424·5 to 484·6)	225·9 (201·8 to 252·6)	7·3 (7·3 to 7·3)
Chile	1998	625·2 (599·5 to 649·3)	230·0 (215·2 to 244·0)	265·1 (249·4 to 280·4)	129·8 (117·7 to 142·1)	0·4 (0·4 to 0·4)	1162·7 (1114·8 to 1207·5)	427·7 (400·3 to 453·8)	492·9 (463·8 to 521·4)	241·4 (219·0 to 264·3)	0·7 (0·7 to 0·7)

Chile	1999	638·9 (615·6 to 660·4)	237·5 (223·3 to 252·8)	269·7 (255·9 to 284·1)	131·4 (122·3 to 140·3)	0·3 (0·3 to 0·3)	1188·1 (1144·8 to 1228·1)	441·7 (415·3 to 470·2)	501·5 (475·9 to 528·4)	244·3 (227·4 to 261·0)	0·6 (0·6 to 0·6)
Chile	2000	633·8 (615·5 to 653·3)	234·9 (220·1 to 249·9)	267·2 (255·8 to 278·7)	131·3 (126·0 to 136·7)	0·3 (0·3 to 0·3)	1178·6 (1144·6 to 1215·0)	436·9 (409·4 to 464·7)	496·9 (475·7 to 518·3)	244·2 (234·3 to 254·3)	0·6 (0·6 to 0·6)
Chile	2001	648·8 (631·0 to 666·3)	242·5 (228·8 to 256·9)	272·7 (262·8 to 282·6)	133·1 (128·4 to 137·5)	0·4 (0·4 to 0·4)	1206·6 (1173·4 to 1239·0)	451·1 (425·4 to 477·7)	507·2 (488·7 to 525·6)	247·6 (238·8 to 255·8)	0·8 (0·8 to 0·8)
Chile	2002	667·6 (651·8 to 684·9)	253·0 (241·7 to 265·3)	281·8 (272·7 to 291·5)	132·6 (128·2 to 137·3)	0·2 (0·2 to 0·2)	1241·6 (1212·2 to 1273·6)	470·6 (449·5 to 493·4)	524·0 (507·1 to 542·2)	246·6 (238·5 to 255·4)	0·4 (0·4 to 0·4)
Chile	2003	681·6 (666·1 to 696·9)	261·0 (251·1 to 271·6)	289·2 (278·9 to 299·6)	130·7 (126·3 to 135·5)	0·7 (0·7 to 0·7)	1267·6 (1238·7 to 1296·0)	485·5 (467·0 to 505·0)	537·9 (518·6 to 557·3)	243·0 (234·9 to 252·0)	1·3 (1·3 to 1·3)
Chile	2004	685·0 (671·0 to 699·3)	266·0 (256·8 to 275·2)	289·5 (280·0 to 300·2)	127·5 (123·2 to 132·0)	2·0 (2·0 to 2·0)	1274·0 (1247·9 to 1300·5)	494·7 (477·6 to 511·7)	538·5 (520·8 to 558·2)	237·1 (229·1 to 245·5)	3·7 (3·7 to 3·7)
Chile	2005	682·7 (669·4 to 697·5)	271·9 (262·8 to 281·7)	286·0 (276·3 to 296·1)	123·7 (119·7 to 128·1)	1·1 (1·1 to 1·1)	1269·6 (1244·8 to 1297·1)	505·7 (488·8 to 523·9)	531·8 (513·9 to 550·7)	230·1 (222·5 to 238·2)	2·1 (2·1 to 2·1)
Chile	2006	674·0 (661·0 to 688·0)	275·9 (266·8 to 285·5)	276·6 (266·8 to 286·9)	121·0 (116·7 to 125·6)	0·5 (0·5 to 0·5)	1253·4 (1229·2 to 1279·5)	513·2 (496·1 to 531·0)	514·3 (496·2 to 533·5)	225·1 (217·0 to 233·6)	0·9 (0·9 to 0·9)
Chile	2007	710·6 (696·9 to 725·4)	300·1 (291·1 to 309·0)	278·7 (268·7 to 288·6)	131·3 (127·0 to 135·5)	0·5 (0·5 to 0·5)	1321·5 (1296·1 to 1349·0)	558·2 (541·3 to 574·7)	518·2 (499·7 to 536·6)	244·1 (236·1 to 252·0)	1·0 (1·0 to 1·0)
Chile	2008	777·1 (762·4 to 791·2)	342·2 (332·7 to 352·2)	287·1 (277·5 to 297·3)	147·4 (142·9 to 151·9)	0·3 (0·3 to 0·3)	1445·1 (1417·8 to 1471·4)	636·4 (618·8 to 655·0)	534·0 (516·0 to 552·9)	274·1 (265·8 to 282·6)	0·6 (0·6 to 0·6)
Chile	2009	819·2 (804·1 to 834·5)	378·9 (369·2 to 389·3)	287·1 (277·0 to 297·5)	153·0 (148·5 to 157·9)	0·3 (0·3 to 0·3)	1523·4 (1495·4 to 1551·8)	704·6 (686·5 to 724·0)	533·9 (515·1 to 553·2)	284·5 (276·1 to 293·7)	0·5 (0·5 to 0·5)
Chile	2010	819·1 (805·2 to 834·3)	385·8 (376·7 to 395·8)	282·8 (272·7 to 293·0)	150·2 (145·5 to 155·1)	0·3 (0·3 to 0·3)	1523·2 (1497·4 to 1551·5)	717·4 (700·6 to 736·1)	525·9 (507·2 to 545·0)	279·3 (270·6 to 288·4)	0·5 (0·5 to 0·5)
Chile	2011	853·7 (839·4 to 868·1)	405·7 (395·7 to 416·5)	291·3 (281·2 to 302·0)	156·6 (152·2 to 161·3)	0·2 (0·2 to 0·2)	1587·7 (1561·0 to 1614·5)	754·4 (735·9 to 774·6)	541·7 (522·9 to 561·6)	291·2 (283·1 to 300·0)	0·3 (0·3 to 0·3)
Chile	2012	924·3 (908·4 to 938·4)	441·6 (431·4 to 452·4)	310·7 (300·5 to 321·4)	172·1 (167·1 to 177·3)	0·0 (0·0 to 0·0)	1719·0 (1689·3 to 1745·2)	821·2 (802·3 to 841·3)	577·7 (558·9 to 597·8)	320·0 (310·8 to 329·8)	0·0 (0·0 to 0·0)

Chile	2013	999·8 (983·8 to 1014·9)	475·4 (465·0 to 485·5)	337·4 (327·1 to 347·7)	187·0 (182·0 to 192·3)	0·0 (0·0 to 0·0)	1859·3 (1829·5 to 1887·5)	884·1 (864·8 to 902·9)	627·4 (608·3 to 646·5)	347·8 (338·5 to 357·7)	0·0 (0·0 to 0·0)
Chile	2014	1071·9 (1055·0 to 1087·8)	510·9 (500·1 to 521·6)	366·8 (356·1 to 377·7)	194·1 (189·0 to 199·1)	0·0 (0·0 to 0·0)	1993·3 (1961·9 to 2022·9)	950·2 (930·0 to 970·1)	682·1 (662·2 to 702·4)	361·0 (351·6 to 370·2)	0·0 (0·0 to 0·0)
Chile	2015	1142·7 (1127·3 to 1158·4)	552·4 (541·4 to 563·7)	393·6 (383·0 to 404·9)	196·7 (191·4 to 201·9)	0·0 (0·0 to 0·0)	2125·0 (2096·4 to 2154·3)	1027·3 (1006·8 to 1048·3)	731·9 (712·3 to 753·1)	365·8 (356·0 to 375·4)	0·0 (0·0 to 0·0)
Chile	2016	1193·5 (1177·3 to 1210·3)	591·4 (580·7 to 602·4)	410·7 (399·7 to 421·8)	191·4 (186·5 to 196·6)	0·0 (0·0 to 0·0)	2219·6 (2189·5 to 2250·9)	1099·8 (1079·9 to 1120·3)	763·8 (743·3 to 784·3)	355·9 (346·8 to 365·6)	0·0 (0·0 to 0·0)
Chile	2017	1251·6 (1234·3 to 1268·8)	629·2 (618·2 to 640·8)	421·3 (410·6 to 432·3)	201·1 (196·2 to 206·0)	0·0 (0·0 to 0·0)	2327·7 (2295·5 to 2359·6)	1170·2 (1149·6 to 1191·8)	783·6 (763·6 to 803·9)	373·9 (364·9 to 383·1)	0·0 (0·0 to 0·0)
Chile	2018	1308·1 (1286·7 to 1328·0)	665·6 (652·1 to 679·8)	433·5 (418·9 to 448·2)	209·1 (202·7 to 215·6)	0·0 (0·0 to 0·0)	2432·7 (2392·8 to 2469·6)	1237·8 (1212·6 to 1264·1)	806·1 (779·0 to 833·4)	388·8 (376·9 to 401·0)	0·0 (0·0 to 0·0)
Chile	2019	1329·2 (1304·5 to 1352·8)	686·2 (667·2 to 705·8)	430·6 (416·3 to 445·1)	212·4 (206·2 to 219·0)	0·0 (0·0 to 0·0)	2471·9 (2425·9 to 2515·7)	1276·1 (1240·7 to 1312·6)	800·8 (774·1 to 827·8)	395·0 (383·6 to 407·2)	0·0 (0·0 to 0·0)
Chile	2020	1364·0 (1339·8 to 1386·7)	773·5 (754·6 to 793·0)	392·5 (379·3 to 405·8)	198·0 (191·8 to 204·3)	0·0 (0·0 to 0·0)	2536·6 (2491·7 to 2578·8)	1438·4 (1403·3 to 1474·7)	729·8 (705·4 to 754·6)	368·3 (356·7 to 379·9)	0·0 (0·0 to 0·0)
Chile	2021	1269·5 (1243·7 to 1296·5)	695·3 (675·2 to 718·3)	378·7 (365·9 to 391·6)	195·5 (189·1 to 201·9)	0·0 (0·0 to 0·0)	2361·0 (2312·9 to 2411·1)	1293·1 (1255·7 to 1335·9)	704·2 (680·5 to 728·3)	363·6 (351·6 to 375·4)	0·0 (0·0 to 0·0)
Chile	2022	1226·5 (1197·0 to 1257·7)	646·0 (619·8 to 674·5)	379·8 (366·9 to 392·7)	200·7 (193·7 to 207·9)	0·0 (0·0 to 0·0)	2280·9 (2226·0 to 2338·9)	1201·4 (1152·6 to 1254·3)	706·3 (682·3 to 730·3)	373·2 (360·3 to 386·6)	0·0 (0·0 to 0·0)
Chile	2023	1249·4 (1218·5 to 1281·7)	663·1 (636·1 to 692·6)	380·5 (367·6 to 393·6)	205·8 (198·1 to 213·2)	0·0 (0·0 to 0·0)	2323·5 (2266·0 to 2383·6)	1233·1 (1183·0 to 1288·1)	707·7 (683·7 to 731·9)	382·7 (368·3 to 396·6)	0·0 (0·0 to 0·0)
Chile	2024	1272·7 (1241·2 to 1306·4)	681·1 (653·0 to 710·4)	380·9 (367·9 to 393·9)	210·7 (201·6 to 219·3)	0·0 (0·0 to 0·0)	2366·8 (2308·3 to 2429·5)	1266·6 (1214·4 to 1321·2)	708·3 (684·2 to 732·5)	391·9 (375·0 to 407·9)	0·0 (0·0 to 0·0)
Chile	2025	1296·1 (1262·7 to 1331·5)	699·4 (670·2 to 731·0)	381·0 (368·0 to 394·2)	215·7 (205·7 to 225·5)	0·0 (0·0 to 0·0)	2410·3 (2348·2 to 2476·1)	1300·6 (1246·4 to 1359·4)	708·5 (684·4 to 733·0)	401·2 (382·5 to 419·4)	0·0 (0·0 to 0·0)
Chile	2026	1320·5 (1284·3 to 1358·5)	718·3 (686·4 to 752·3)	381·3 (368·4 to 394·6)	220·9 (209·4 to 232·0)	0·0 (0·0 to 0·0)	2455·8 (2388·4 to 2526·4)	1335·8 (1276·5 to 1399·1)	709·1 (685·0 to 733·8)	410·8 (389·4 to 431·5)	0·0 (0·0 to 0·0)

Chile	2027	1345·5 (1305·9 to 1386·8)	737·5 (702·4 to 774·0)	381·7 (368·6 to 395·0)	226·3 (213·4 to 239·3)	0·0 (0·0 to 0·0)	2502·2 (2428·5 to 2579·0)	1371·5 (1306·2 to 1439·5)	709·9 (685·6 to 734·5)	420·8 (396·8 to 445·0)	0·0 (0·0 to 0·0)
Chile	2028	1372·1 (1330·3 to 1417·6)	757·7 (719·3 to 797·3)	382·4 (369·3 to 395·5)	232·0 (217·2 to 246·5)	0·0 (0·0 to 0·0)	2551·6 (2473·9 to 2636·2)	1409·1 (1337·8 to 1482·7)	711·2 (686·7 to 735·5)	431·4 (404·0 to 458·4)	0·0 (0·0 to 0·0)
Chile	2029	1399·1 (1353·8 to 1448·8)	778·4 (736·4 to 820·7)	383·0 (369·7 to 396·1)	237·7 (221·6 to 254·6)	0·0 (0·0 to 0·0)	2601·9 (2517·6 to 2694·3)	1447·5 (1369·5 to 1526·3)	712·2 (687·6 to 736·7)	442·1 (412·1 to 473·5)	0·0 (0·0 to 0·0)
Chile	2030	1426·5 (1377·4 to 1480·6)	799·3 (754·6 to 844·9)	383·6 (370·2 to 396·7)	243·6 (225·5 to 263·2)	0·0 (0·0 to 0·0)	2652·8 (2561·5 to 2753·4)	1486·4 (1403·3 to 1571·2)	713·3 (688·4 to 737·8)	453·1 (419·4 to 489·5)	0·0 (0·0 to 0·0)
Chile	2031	1456·0 (1403·4 to 1513·8)	821·2 (773·5 to 870·3)	384·7 (371·4 to 397·9)	250·0 (229·6 to 272·0)	0·0 (0·0 to 0·0)	2707·7 (2610·0 to 2815·1)	1527·2 (1438·4 to 1618·6)	715·5 (690·6 to 740·0)	465·0 (427·0 to 505·8)	0·0 (0·0 to 0·0)
Chile	2032	1484·2 (1428·6 to 1546·6)	842·0 (790·3 to 895·5)	385·7 (372·4 to 399·0)	256·5 (233·6 to 281·2)	0·0 (0·0 to 0·0)	2760·2 (2656·7 to 2876·2)	1565·8 (1469·7 to 1665·4)	717·4 (692·5 to 742·0)	477·1 (434·5 to 522·9)	0·0 (0·0 to 0·0)
Chile	2033	1512·7 (1452·5 to 1579·1)	862·3 (806·2 to 920·7)	387·0 (373·6 to 400·0)	263·4 (238·3 to 291·4)	0·0 (0·0 to 0·0)	2813·2 (2701·2 to 2936·6)	1603·6 (1499·3 to 1712·3)	719·8 (694·8 to 744·0)	489·8 (443·1 to 542·0)	0·0 (0·0 to 0·0)
Chile	2034	1541·0 (1475·6 to 1612·3)	882·2 (823·1 to 945·1)	388·4 (374·9 to 401·2)	270·4 (242·7 to 302·1)	0·0 (0·0 to 0·0)	2865·7 (2744·1 to 2998·4)	1640·6 (1530·7 to 1757·6)	722·2 (697·1 to 746·2)	502·9 (451·3 to 561·8)	0·0 (0·0 to 0·0)
Chile	2035	1570·3 (1500·2 to 1647·3)	902·4 (840·3 to 969·1)	390·0 (376·5 to 403·0)	277·9 (246·9 to 313·6)	0·0 (0·0 to 0·0)	2920·2 (2789·9 to 3063·4)	1678·1 (1562·7 to 1802·3)	725·3 (700·1 to 749·4)	516·8 (459·1 to 583·2)	0·0 (0·0 to 0·0)
Chile	2036	1599·4 (1525·8 to 1680·5)	922·2 (857·1 to 992·3)	391·7 (378·1 to 404·8)	285·6 (251·7 to 325·3)	0·0 (0·0 to 0·0)	2974·5 (2837·5 to 3125·3)	1715·1 (1594·0 to 1845·4)	728·4 (703·1 to 752·7)	531·0 (468·0 to 604·9)	0·0 (0·0 to 0·0)
Chile	2037	1629·4 (1550·8 to 1717·3)	942·4 (873·3 to 1017·2)	393·4 (379·8 to 406·9)	293·5 (256·4 to 337·2)	0·0 (0·0 to 0·0)	3030·1 (2884·1 to 3193·7)	1752·5 (1624·0 to 1891·7)	731·7 (706·3 to 756·7)	545·9 (476·9 to 627·1)	0·0 (0·0 to 0·0)
Chile	2038	1660·1 (1577·2 to 1753·3)	962·8 (890·6 to 1042·1)	395·4 (381·7 to 409·1)	301·9 (261·5 to 349·1)	0·0 (0·0 to 0·0)	3087·2 (2933·1 to 3260·5)	1790·5 (1656·2 to 1938·0)	735·3 (709·8 to 760·7)	561·4 (486·3 to 649·3)	0·0 (0·0 to 0·0)
Chile	2039	1691·0 (1603·1 to 1788·7)	983·1 (906·5 to 1066·9)	397·4 (383·7 to 411·3)	310·5 (266·7 to 361·5)	0·0 (0·0 to 0·0)	3144·7 (2981·3 to 3326·4)	1828·3 (1685·9 to 1984·1)	739·1 (713·5 to 764·9)	577·4 (496·0 to 672·3)	0·0 (0·0 to 0·0)
Chile	2040	1721·3 (1627·8 to 1824·6)	1002·8 (921·4 to 1091·6)	399·3 (385·6 to 413·4)	319·2 (271·9 to 374·2)	0·0 (0·0 to 0·0)	3201·0 (3027·2 to 3393·3)	1864·9 (1713·5 to 2030·1)	742·6 (717·0 to 768·7)	593·6 (505·6 to 695·9)	0·0 (0·0 to 0·0)

Chile	2041	1751·2 (1653·2 to 1859·2)	1022·0 (935·9 to 1113·1)	401·1 (387·3 to 415·2)	328·1 (277·2 to 388·0)	0·0 (0·0 to 0·0)	3256·6 (3074·5 to 3457·5)	1900·5 (1740·5 to 2070·1)	745·9 (720·2 to 772·2)	610·2 (515·4 to 721·6)	0·0 (0·0 to 0·0)
Chile	2042	1781·9 (1678·1 to 1896·2)	1041·2 (951·7 to 1135·4)	403·2 (389·3 to 417·4)	337·5 (282·7 to 402·6)	0·0 (0·0 to 0·0)	3313·7 (3120·7 to 3526·3)	1936·4 (1770·0 to 2111·6)	749·7 (723·9 to 776·2)	627·6 (525·7 to 748·7)	0·0 (0·0 to 0·0)
Chile	2043	1813·5 (1705·7 to 1932·2)	1060·7 (968·0 to 1157·9)	405·5 (391·3 to 419·9)	347·3 (288·2 to 418·1)	0·0 (0·0 to 0·0)	3372·5 (3172·0 to 3593·3)	1972·6 (1800·2 to 2153·3)	754·0 (727·7 to 780·9)	645·9 (536·0 to 777·6)	0·0 (0·0 to 0·0)
Chile	2044	1844·4 (1731·4 to 1971·2)	1079·4 (984·5 to 1180·6)	407·7 (393·5 to 422·3)	357·4 (294·3 to 434·1)	0·0 (0·0 to 0·0)	3430·0 (3219·9 to 3665·9)	2007·3 (1830·9 to 2195·5)	758·2 (731·8 to 785·3)	664·6 (547·4 to 807·2)	0·0 (0·0 to 0·0)
Chile	2045	1875·1 (1757·1 to 2008·6)	1097·5 (1000·6 to 1202·4)	409·9 (395·4 to 424·7)	367·7 (300·3 to 449·9)	0·0 (0·0 to 0·0)	3487·1 (3267·6 to 3735·3)	2041·1 (1860·9 to 2236·1)	762·3 (735·4 to 789·8)	683·7 (558·5 to 836·8)	0·0 (0·0 to 0·0)
Chile	2046	1906·0 (1782·6 to 2045·2)	1115·6 (1015·6 to 1224·3)	412·2 (397·5 to 427·1)	378·3 (306·4 to 466·1)	0·0 (0·0 to 0·0)	3544·6 (3315·2 to 3803·5)	2074·6 (1888·7 to 2276·8)	766·5 (739·2 to 794·3)	703·5 (569·7 to 866·8)	0·0 (0·0 to 0·0)
Chile	2047	1935·8 (1807·0 to 2079·6)	1132·8 (1031·1 to 1243·2)	414·1 (399·2 to 429·3)	388·9 (312·3 to 482·4)	0·0 (0·0 to 0·0)	3600·1 (3360·5 to 3867·5)	2106·7 (1917·4 to 2312·0)	770·1 (742·4 to 798·3)	723·2 (580·8 to 897·2)	0·0 (0·0 to 0·0)
Chile	2048	1965·7 (1833·3 to 2115·8)	1149·9 (1045·3 to 1262·7)	416·0 (400·9 to 431·2)	399·8 (318·4 to 499·3)	0·0 (0·0 to 0·0)	3655·6 (3409·3 to 3934·8)	2138·4 (1943·9 to 2348·2)	773·6 (745·5 to 801·9)	743·5 (592·1 to 928·5)	0·0 (0·0 to 0·0)
Chile	2049	1995·0 (1858·5 to 2152·2)	1166·4 (1060·9 to 1280·7)	417·8 (402·4 to 433·1)	410·9 (324·4 to 517·4)	0·0 (0·0 to 0·0)	3710·0 (3456·3 to 4002·4)	2169·1 (1972·9 to 2381·7)	776·9 (748·4 to 805·4)	764·1 (603·3 to 962·2)	0·0 (0·0 to 0·0)
Chile	2050	2024·7 (1884·6 to 2184·4)	1182·9 (1075·4 to 1299·9)	419·6 (404·1 to 435·2)	422·2 (330·6 to 536·7)	0·0 (0·0 to 0·0)	3765·3 (3504·7 to 4062·3)	2199·8 (1999·9 to 2417·4)	780·3 (751·5 to 809·3)	785·2 (614·8 to 998·0)	0·0 (0·0 to 0·0)
China*	1995	62·1 (54·0 to 71·5)	17·0 (14·0 to 20·7)	31·7 (25·3 to 39·2)	13·2 (9·2 to 18·6)	0·1 (0·1 to 0·1)	98·5 (85·8 to 113·5)	26·9 (22·2 to 32·9)	50·4 (40·1 to 62·1)	21·0 (14·6 to 29·5)	0·2 (0·2 to 0·2)
China*	1996	68·3 (59·2 to 78·4)	18·3 (15·0 to 22·2)	36·2 (29·2 to 44·0)	13·7 (9·6 to 19·3)	0·1 (0·1 to 0·1)	108·5 (94·0 to 124·5)	29·0 (23·9 to 35·3)	57·5 (46·3 to 69·8)	21·8 (15·3 to 30·6)	0·2 (0·2 to 0·2)
China*	1997	75·8 (66·2 to 86·5)	19·8 (16·4 to 24·0)	41·6 (33·9 to 50·2)	14·3 (10·1 to 20·1)	0·1 (0·1 to 0·1)	120·3 (105·1 to 137·3)	31·5 (26·0 to 38·1)	66·0 (53·7 to 79·7)	22·7 (16·0 to 31·9)	0·2 (0·2 to 0·2)
China*	1998	83·8 (73·2 to 94·6)	21·4 (17·8 to 25·8)	47·5 (38·9 to 57·2)	14·7 (10·3 to 20·7)	0·1 (0·1 to 0·1)	133·0 (116·1 to 150·2)	34·0 (28·2 to 40·9)	75·4 (61·7 to 90·8)	23·4 (16·4 to 32·8)	0·2 (0·2 to 0·2)

China*	1999	93·6 (81·8 to 105·7)	23·8 (19·9 to 28·7)	54·2 (44·4 to 65·2)	15·4 (11·0 to 21·3)	0·1 (0·1 to 0·1)	148·5 (129·8 to 167·7)	37·7 (31·6 to 45·6)	86·1 (70·6 to 103·5)	24·5 (17·5 to 33·8)	0·2 (0·2 to 0·2)
China*	2000	101·8 (88·7 to 115·4)	25·2 (21·1 to 30·4)	60·5 (50·3 to 72·6)	16·0 (11·4 to 22·3)	0·2 (0·2 to 0·2)	161·6 (140·8 to 183·1)	39·9 (33·4 to 48·3)	96·0 (79·8 to 115·2)	25·4 (18·1 to 35·5)	0·2 (0·2 to 0·2)
China*	2001	109·2 (96·1 to 122·6)	27·8 (23·3 to 33·6)	66·6 (55·8 to 78·8)	14·7 (10·4 to 20·4)	0·1 (0·1 to 0·1)	173·4 (152·6 to 194·6)	44·2 (36·9 to 53·3)	105·7 (88·5 to 125·1)	23·3 (16·6 to 32·4)	0·1 (0·1 to 0·1)
China*	2002	120·2 (106·5 to 134·5)	32·4 (27·1 to 38·7)	73·1 (61·3 to 86·2)	14·6 (10·3 to 20·0)	0·1 (0·1 to 0·1)	190·8 (169·0 to 213·4)	51·4 (43·1 to 61·5)	116·0 (97·4 to 136·8)	23·2 (16·4 to 31·8)	0·2 (0·2 to 0·2)
China*	2003	129·2 (115·3 to 144·1)	36·8 (31·1 to 43·9)	77·5 (65·4 to 91·5)	14·6 (10·5 to 20·0)	0·1 (0·1 to 0·1)	205·0 (183·1 to 228·7)	58·5 (49·4 to 69·7)	123·1 (103·9 to 145·3)	23·2 (16·6 to 31·8)	0·2 (0·2 to 0·2)
China*	2004	137·5 (123·3 to 153·9)	41·8 (35·7 to 49·3)	80·7 (68·8 to 94·5)	14·8 (10·7 to 20·4)	0·2 (0·2 to 0·2)	218·3 (195·7 to 244·2)	66·4 (56·7 to 78·3)	128·1 (109·2 to 150·0)	23·5 (17·0 to 32·4)	0·3 (0·3 to 0·3)
China*	2005	147·9 (132·9 to 165·1)	49·0 (41·7 to 57·6)	84·1 (72·0 to 97·3)	14·6 (10·6 to 20·0)	0·2 (0·2 to 0·2)	234·8 (211·0 to 262·0)	77·9 (66·3 to 91·3)	133·4 (114·3 to 154·5)	23·2 (16·9 to 31·8)	0·3 (0·3 to 0·3)
China*	2006	158·3 (142·9 to 176·0)	57·2 (49·3 to 66·1)	86·3 (74·3 to 100·3)	14·6 (10·4 to 19·8)	0·2 (0·2 to 0·2)	251·3 (226·9 to 279·3)	90·7 (78·2 to 105·0)	137·0 (117·9 to 159·3)	23·2 (16·5 to 31·4)	0·4 (0·4 to 0·4)
China*	2007	170·5 (154·2 to 187·8)	66·9 (58·2 to 77·1)	88·0 (75·4 to 102·5)	15·3 (10·9 to 20·8)	0·3 (0·3 to 0·3)	270·6 (244·8 to 298·2)	106·2 (92·4 to 122·4)	139·6 (119·7 to 162·6)	24·2 (17·3 to 33·1)	0·5 (0·5 to 0·5)
China*	2008	199·8 (181·4 to 219·7)	88·4 (77·9 to 100·3)	95·0 (80·8 to 109·7)	16·2 (11·5 to 22·0)	0·2 (0·2 to 0·2)	317·2 (287·9 to 348·8)	140·4 (123·6 to 159·2)	150·8 (128·3 to 174·1)	25·6 (18·3 to 34·9)	0·4 (0·4 to 0·4)
China*	2009	231·9 (211·0 to 251·7)	112·2 (99·5 to 125·8)	102·3 (87·7 to 117·8)	17·1 (12·1 to 23·0)	0·3 (0·3 to 0·3)	368·1 (334·9 to 399·5)	178·1 (158·0 to 199·7)	162·4 (139·1 to 187·1)	27·1 (19·2 to 36·5)	0·5 (0·5 to 0·5)
China*	2010	254·1 (232·2 to 274·9)	129·5 (116·7 to 143·3)	106·9 (92·1 to 122·3)	17·4 (12·3 to 23·3)	0·2 (0·2 to 0·2)	403·3 (368·6 to 436·3)	205·6 (185·2 to 227·5)	169·7 (146·2 to 194·1)	27·7 (19·6 to 37·0)	0·4 (0·4 to 0·4)
China*	2011	288·0 (265·7 to 310·3)	154·7 (141·1 to 169·2)	115·6 (101·1 to 131·2)	17·4 (12·2 to 23·3)	0·2 (0·2 to 0·2)	457·1 (421·7 to 492·5)	245·6 (224·0 to 268·6)	183·5 (160·4 to 208·3)	27·7 (19·4 to 37·0)	0·3 (0·3 to 0·3)
China*	2012	318·8 (296·8 to 342·3)	177·3 (162·0 to 192·5)	124·0 (108·4 to 140·2)	17·3 (12·3 to 22·8)	0·3 (0·3 to 0·3)	506·1 (471·1 to 543·3)	281·4 (257·1 to 305·6)	196·7 (172·0 to 222·5)	27·5 (19·6 to 36·2)	0·4 (0·4 to 0·4)

China*	2013	348·5 (326·6 to 373·4)	198·5 (183·7 to 214·3)	131·9 (116·8 to 149·4)	18·1 (13·3 to 23·9)	0·1 (0·1 to 0·1)	553·2 (518·5 to 592·6)	315·0 (291·6 to 340·2)	209·3 (185·5 to 237·1)	28·7 (21·0 to 38·0)	0·2 (0·2 to 0·2)
China*	2014	378·7 (356·0 to 402·2)	219·1 (204·7 to 235·0)	139·6 (125·4 to 156·9)	19·9 (14·9 to 25·9)	0·1 (0·1 to 0·1)	601·1 (565·0 to 638·4)	347·7 (324·9 to 373·0)	221·6 (199·0 to 249·0)	31·6 (23·6 to 41·2)	0·1 (0·1 to 0·1)
China*	2015	418·9 (395·3 to 443·7)	245·6 (230·5 to 262·7)	150·6 (135·3 to 167·9)	22·6 (17·1 to 29·3)	0·1 (0·1 to 0·1)	664·9 (627·4 to 704·2)	389·8 (365·9 to 417·0)	239·1 (214·8 to 266·5)	35·9 (27·2 to 46·4)	0·1 (0·1 to 0·1)
China*	2016	450·9 (428·2 to 474·5)	261·2 (245·5 to 277·1)	161·9 (146·3 to 178·4)	27·7 (21·6 to 34·8)	0·2 (0·2 to 0·2)	715·6 (679·7 to 753·1)	414·5 (389·7 to 439·8)	256·9 (232·3 to 283·2)	43·9 (34·3 to 55·2)	0·2 (0·2 to 0·2)
China*	2017	480·1 (456·9 to 503·3)	273·7 (260·0 to 288·7)	172·4 (157·7 to 190·1)	33·8 (27·2 to 41·6)	0·2 (0·2 to 0·2)	762·0 (725·3 to 798·8)	434·4 (412·7 to 458·2)	273·7 (250·3 to 301·8)	53·7 (43·1 to 66·1)	0·2 (0·2 to 0·2)
China*	2018	520·9 (493·4 to 548·5)	295·9 (279·0 to 313·7)	184·9 (166·6 to 204·7)	39·9 (31·7 to 49·3)	0·2 (0·2 to 0·2)	826·7 (783·2 to 870·6)	469·7 (442·8 to 497·9)	293·5 (264·4 to 324·9)	63·3 (50·3 to 78·2)	0·3 (0·3 to 0·3)
China*	2019	562·7 (531·3 to 593·9)	324·6 (304·5 to 345·7)	195·0 (175·6 to 215·9)	43·0 (34·2 to 53·2)	0·2 (0·2 to 0·2)	893·1 (843·3 to 942·7)	515·1 (483·2 to 548·7)	309·5 (278·8 to 342·6)	68·2 (54·3 to 84·4)	0·3 (0·3 to 0·3)
China*	2020	591·6 (558·1 to 625·5)	352·2 (328·7 to 377·6)	195·2 (175·8 to 216·3)	44·0 (35·0 to 54·5)	0·2 (0·2 to 0·2)	939·0 (885·9 to 992·9)	559·0 (521·7 to 599·3)	309·8 (279·0 to 343·3)	69·9 (55·6 to 86·5)	0·3 (0·3 to 0·3)
China*	2021	605·5 (571·3 to 640·4)	360·3 (336·2 to 386·3)	199·1 (179·2 to 220·6)	45·9 (36·4 to 57·0)	0·2 (0·2 to 0·2)	961·1 (906·8 to 1016·4)	571·9 (533·7 to 613·1)	316·1 (284·4 to 350·1)	72·9 (57·9 to 90·5)	0·3 (0·2 to 0·3)
China*	2022	614·6 (576·6 to 652·8)	364·7 (335·2 to 395·0)	202·1 (181·7 to 223·9)	47·6 (37·7 to 59·1)	0·1 (0·1 to 0·1)	975·4 (915·2 to 1036·1)	578·8 (532·0 to 627·0)	320·8 (288·5 to 355·3)	75·6 (59·8 to 93·8)	0·2 (0·2 to 0·2)
China*	2023	635·3 (594·4 to 676·1)	380·0 (348·1 to 412·5)	205·6 (184·9 to 227·9)	49·6 (39·1 to 61·5)	0·1 (0·1 to 0·1)	1008·3 (943·4 to 1073·1)	603·1 (552·4 to 654·7)	326·4 (293·5 to 361·7)	78·7 (62·1 to 97·6)	0·2 (0·2 to 0·2)
China*	2024	656·0 (612·7 to 698·9)	394·9 (359·4 to 430·6)	209·4 (188·3 to 232·0)	51·6 (40·7 to 64·1)	0·1 (0·1 to 0·1)	1041·3 (972·6 to 1109·3)	626·8 (570·4 to 683·4)	332·3 (298·9 to 368·3)	81·9 (64·7 to 101·7)	0·2 (0·2 to 0·2)
China*	2025	677·3 (630·0 to 723·1)	410·1 (371·6 to 448·8)	213·3 (191·8 to 236·4)	53·8 (42·6 to 66·7)	0·1 (0·1 to 0·1)	1075·0 (1000·0 to 1147·8)	651·0 (589·8 to 712·4)	338·5 (304·4 to 375·2)	85·3 (67·7 to 105·8)	0·2 (0·2 to 0·2)
China*	2026	699·0 (649·7 to 747·2)	425·9 (384·4 to 467·7)	217·1 (195·2 to 240·7)	56·0 (44·5 to 69·2)	0·1 (0·1 to 0·1)	1109·5 (1031·2 to 1185·9)	675·9 (610·1 to 742·3)	344·6 (309·8 to 382·0)	88·8 (70·7 to 109·9)	0·2 (0·2 to 0·2)

China*	2027	722·4 (670·5 to 774·6)	443·3 (401·2 to 487·9)	220·8 (198·5 to 244·7)	58·2 (46·3 to 72·1)	0·1 (0·1 to 0·1)	1146·6 (1064·3 to 1229·5)	703·6 (636·8 to 774·4)	350·4 (315·1 to 388·4)	92·4 (73·5 to 114·4)	0·2 (0·2 to 0·2)
China*	2028	746·1 (690·0 to 800·2)	461·7 (414·6 to 510·6)	223·9 (201·1 to 248·1)	60·4 (47·9 to 75·0)	0·1 (0·1 to 0·1)	1184·2 (1095·3 to 1270·2)	732·8 (658·1 to 810·4)	355·4 (319·3 to 393·7)	95·8 (76·1 to 119·0)	0·2 (0·2 to 0·2)
China*	2029	770·9 (711·6 to 828·2)	481·7 (429·7 to 535·3)	226·6 (203·6 to 251·0)	62·5 (49·7 to 77·5)	0·1 (0·1 to 0·1)	1223·6 (1129·4 to 1314·5)	764·6 (682·1 to 849·7)	359·6 (323·1 to 398·4)	99·2 (78·8 to 123·1)	0·2 (0·2 to 0·2)
China*	2030	796·9 (733·7 to 859·8)	503·2 (447·8 to 561·7)	229·0 (205·7 to 253·8)	64·6 (51·2 to 80·1)	0·1 (0·1 to 0·1)	1264·9 (1164·6 to 1364·6)	798·7 (710·7 to 891·6)	363·5 (326·4 to 402·8)	102·6 (81·2 to 127·1)	0·2 (0·2 to 0·2)
China*	2031	824·6 (756·4 to 890·1)	526·3 (465·4 to 587·9)	231·4 (207·8 to 256·4)	66·8 (52·8 to 83·0)	0·1 (0·0 to 0·1)	1308·9 (1200·5 to 1412·8)	835·3 (738·7 to 933·2)	367·3 (329·9 to 407·0)	106·0 (83·8 to 131·8)	0·2 (0·0 to 0·2)
China*	2032	853·1 (778·5 to 921·7)	549·7 (483·8 to 616·7)	234·1 (210·3 to 259·4)	69·1 (54·7 to 86·2)	0·1 (0·0 to 0·1)	1354·0 (1235·7 to 1463·0)	872·5 (767·9 to 978·9)	371·6 (333·8 to 411·8)	109·7 (86·8 to 136·8)	0·2 (0·0 to 0·2)
China*	2033	882·9 (805·0 to 958·4)	574·1 (500·7 to 647·2)	237·1 (213·0 to 262·7)	71·6 (56·5 to 89·6)	0·1 (0·0 to 0·2)	1401·3 (1277·7 to 1521·3)	911·3 (794·8 to 1027·3)	376·3 (338·0 to 417·0)	113·6 (89·6 to 142·1)	0·2 (0·0 to 0·2)
China*	2034	913·5 (828·7 to 995·6)	599·4 (522·4 to 676·9)	240·0 (215·6 to 266·2)	74·1 (58·2 to 92·9)	0·1 (0·0 to 0·2)	1450·0 (1315·3 to 1580·3)	951·4 (829·1 to 1074·5)	380·9 (342·2 to 422·5)	117·6 (92·4 to 147·4)	0·2 (0·0 to 0·2)
China*	2035	944·6 (854·4 to 1030·0)	624·5 (542·5 to 707·6)	243·2 (218·5 to 269·7)	76·8 (60·1 to 96·1)	0·1 (0·0 to 0·2)	1499·2 (1356·1 to 1634·8)	991·3 (861·0 to 1123·1)	385·9 (346·9 to 428·0)	121·9 (95·4 to 152·6)	0·2 (0·0 to 0·2)
China*	2036	977·3 (882·6 to 1066·6)	650·7 (564·8 to 739·5)	246·8 (221·8 to 273·6)	79·7 (61·9 to 100·1)	0·1 (0·0 to 0·2)	1551·2 (1400·8 to 1692·9)	1032·8 (896·4 to 1173·7)	391·7 (352·1 to 434·2)	126·5 (98·2 to 158·8)	0·2 (0·0 to 0·3)
China*	2037	1014·5 (913·8 to 1114·0)	680·0 (583·5 to 776·6)	251·4 (226·0 to 278·5)	83·0 (63·9 to 104·6)	0·1 (0·0 to 0·2)	1610·2 (1450·4 to 1768·1)	1079·3 (926·1 to 1232·7)	399·0 (358·7 to 442·1)	131·8 (101·4 to 166·0)	0·1 (0·0 to 0·2)
China*	2038	1050·2 (944·0 to 1154·8)	708·2 (610·1 to 807·4)	255·6 (229·8 to 283·1)	86·3 (66·0 to 109·4)	0·1 (0·0 to 0·2)	1666·9 (1498·3 to 1833·0)	1124·1 (968·4 to 1281·6)	405·7 (364·8 to 449·4)	137·0 (104·8 to 173·6)	0·1 (0·0 to 0·3)
China*	2039	1084·8 (972·7 to 1196·5)	735·1 (628·7 to 838·0)	259·9 (233·7 to 287·8)	89·8 (68·3 to 114·1)	0·1 (0·0 to 0·2)	1721·8 (1543·8 to 1899·1)	1166·7 (997·8 to 1330·1)	412·5 (370·9 to 456·9)	142·5 (108·5 to 181·1)	0·1 (0·0 to 0·3)
China*	2040	1120·2 (999·4 to 1240·3)	762·4 (653·0 to 875·8)	264·3 (237·7 to 292·7)	93·4 (70·7 to 119·2)	0·1 (0·0 to 0·2)	1777·9 (1586·3 to 1968·6)	1210·2 (1036·5 to 1390·0)	419·5 (377·3 to 464·6)	148·2 (112·2 to 189·2)	0·1 (0·0 to 0·3)

China*	2041	1156·0 (1031·6 to 1282·1)	790·0 (674·9 to 907·1)	268·8 (241·9 to 297·8)	97·2 (73·4 to 124·8)	0·1 (0·0 to 0·2)	1834·9 (1637·4 to 2035·0)	1253·9 (1071·2 to 1439·8)	426·7 (383·9 to 472·6)	154·2 (116·5 to 198·1)	0·1 (0·0 to 0·3)
China*	2042	1190·6 (1060·9 to 1322·4)	816·2 (698·0 to 939·0)	273·4 (246·0 to 302·8)	101·1 (76·4 to 130·6)	0·1 (0·0 to 0·2)	1889·8 (1683·8 to 2098·9)	1295·4 (1107·9 to 1490·3)	433·9 (390·4 to 480·6)	160·4 (121·3 to 207·2)	0·1 (0·0 to 0·3)
China*	2043	1227·4 (1092·8 to 1365·9)	843·7 (721·2 to 975·4)	278·4 (250·5 to 308·3)	105·3 (79·4 to 136·5)	0·0 (0·0 to 0·2)	1948·1 (1734·6 to 2168·0)	1339·1 (1144·7 to 1548·1)	441·8 (397·5 to 489·4)	167·1 (125·9 to 216·7)	0·1 (0·0 to 0·3)
China*	2044	1263·3 (1121·1 to 1408·4)	870·1 (740·1 to 1009·3)	283·5 (255·0 to 314·0)	109·7 (82·4 to 143·0)	0·0 (0·0 to 0·2)	2005·2 (1779·5 to 2235·4)	1381·1 (1174·7 to 1602·0)	450·0 (404·8 to 498·4)	174·0 (130·8 to 227·0)	0·1 (0·0 to 0·3)
China*	2045	1298·9 (1147·7 to 1450·8)	895·8 (760·0 to 1048·6)	288·8 (259·7 to 319·9)	114·3 (85·7 to 150·1)	0·0 (0·0 to 0·2)	2061·7 (1821·6 to 2302·8)	1421·9 (1206·3 to 1664·4)	458·4 (412·2 to 507·7)	181·4 (136·1 to 238·2)	0·1 (0·0 to 0·2)
China*	2046	1333·5 (1176·3 to 1493·7)	920·6 (776·7 to 1076·9)	293·9 (264·1 to 325·5)	119·0 (89·1 to 157·7)	0·0 (0·0 to 0·2)	2116·5 (1867·1 to 2370·9)	1461·1 (1232·8 to 1709·2)	466·5 (419·1 to 516·7)	188·8 (141·5 to 250·3)	0·0 (0·0 to 0·2)
China*	2047	1368·3 (1202·7 to 1533·1)	945·9 (794·4 to 1101·9)	298·7 (268·4 to 330·8)	123·7 (92·4 to 164·3)	0·0 (0·0 to 0·2)	2171·8 (1909·0 to 2433·4)	1501·4 (1260·9 to 1749·0)	474·1 (426·0 to 525·0)	196·3 (146·6 to 260·8)	0·0 (0·0 to 0·2)
China*	2048	1402·1 (1230·7 to 1574·1)	971·2 (814·4 to 1137·2)	302·7 (272·0 to 335·2)	128·2 (95·3 to 171·0)	0·0 (0·0 to 0·2)	2225·4 (1953·4 to 2498·5)	1541·5 (1292·6 to 1805·0)	480·4 (431·8 to 532·1)	203·5 (151·2 to 271·5)	0·0 (0·0 to 0·3)
China*	2049	1435·8 (1262·2 to 1615·6)	997·0 (835·2 to 1168·4)	306·2 (275·2 to 339·2)	132·6 (98·0 to 178·0)	0·0 (0·0 to 0·2)	2279·0 (2003·3 to 2564·4)	1582·4 (1325·6 to 1854·6)	486·0 (436·8 to 538·4)	210·5 (155·5 to 282·5)	0·0 (0·0 to 0·3)
China*	2050	1470·3 (1288·9 to 1657·3)	1024·0 (856·7 to 1203·4)	309·3 (278·0 to 342·8)	137·1 (100·2 to 185·0)	0·0 (0·0 to 0·2)	2333·8 (2045·7 to 2630·5)	1625·3 (1359·7 to 1910·1)	490·9 (441·2 to 544·1)	217·6 (159·1 to 293·6)	0·0 (0·0 to 0·3)
Côte d'Ivoire*	1995	76·1 (67·0 to 87·1)	10·4 (8·4 to 12·6)	48·3 (40·0 to 58·6)	9·8 (6·9 to 13·8)	7·6 (7·6 to 7·6)	178·7 (157·4 to 204·7)	24·4 (19·8 to 29·5)	113·4 (94·1 to 137·6)	23·1 (16·1 to 32·5)	17·9 (17·9 to 17·9)
Côte d'Ivoire*	1996	77·1 (67·9 to 87·6)	11·6 (9·5 to 14·1)	50·9 (43·0 to 60·7)	10·2 (7·1 to 14·1)	4·4 (4·4 to 4·4)	181·1 (159·6 to 205·7)	27·4 (22·3 to 33·2)	119·7 (101·1 to 142·6)	23·9 (16·7 to 33·2)	10·2 (10·2 to 10·2)
Côte d'Ivoire*	1997	78·7 (69·5 to 88·4)	12·8 (10·4 to 15·4)	53·0 (45·3 to 62·5)	10·5 (7·4 to 14·6)	2·3 (2·3 to 2·3)	184·8 (163·2 to 207·6)	30·1 (24·5 to 36·2)	124·6 (106·4 to 146·9)	24·7 (17·3 to 34·3)	5·4 (5·4 to 5·4)
Côte d'Ivoire*	1998	77·8 (69·6 to 86·3)	12·7 (10·3 to 15·4)	51·9 (45·1 to 59·6)	10·6 (7·5 to 14·9)	2·6 (2·6 to 2·6)	182·7 (163·6 to 202·9)	29·8 (24·3 to 36·2)	121·9 (105·9 to 140·1)	24·8 (17·5 to 35·0)	6·1 (6·1 to 6·1)

Côte d'Ivoire*	1999	72·3 (65·2 to 79·5)	12·0 (9·8 to 14·4)	47·0 (41·6 to 52·9)	10·6 (7·5 to 14·8)	2·7 (2·7 to 2·7)	169·9 (153·1 to 186·9)	28·1 (23·1 to 33·9)	110·4 (97·7 to 124·2)	25·0 (17·7 to 34·7)	6·4 (6·4 to 6·4)
Côte d'Ivoire*	2000	62·9 (56·8 to 69·2)	10·7 (8·8 to 12·9)	40·7 (36·3 to 45·2)	10·7 (7·6 to 15·0)	0·9 (0·9 to 0·9)	147·8 (133·4 to 162·6)	25·1 (20·7 to 30·4)	95·6 (85·3 to 106·1)	25·1 (17·8 to 35·3)	2·1 (2·1 to 2·1)
Côte d'Ivoire*	2001	54·8 (49·4 to 60·3)	9·2 (7·6 to 11·2)	34·3 (30·7 to 38·0)	10·2 (7·0 to 14·5)	1·1 (1·1 to 1·1)	128·8 (116·1 to 141·7)	21·7 (17·8 to 26·3)	80·7 (72·2 to 89·2)	23·9 (16·5 to 34·1)	2·6 (2·6 to 2·6)
Côte d'Ivoire*	2002	54·1 (48·9 to 59·7)	9·4 (7·7 to 11·4)	31·9 (28·7 to 35·7)	10·2 (6·9 to 14·4)	2·7 (2·7 to 2·7)	127·2 (114·9 to 140·2)	22·1 (18·1 to 26·9)	74·8 (67·4 to 83·8)	24·0 (16·2 to 33·8)	6·2 (6·2 to 6·2)
Côte d'Ivoire*	2003	52·2 (47·2 to 57·5)	8·8 (7·1 to 10·6)	31·3 (28·3 to 35·3)	9·8 (6·6 to 13·8)	2·2 (2·2 to 2·2)	122·7 (110·9 to 135·1)	20·7 (16·8 to 25·0)	73·6 (66·5 to 83·0)	23·1 (15·6 to 32·3)	5·3 (5·3 to 5·3)
Côte d'Ivoire*	2004	55·0 (49·8 to 60·5)	8·9 (7·3 to 10·6)	33·4 (29·9 to 37·6)	9·9 (6·7 to 13·8)	2·9 (2·9 to 2·9)	129·3 (117·0 to 142·1)	20·8 (17·1 to 25·0)	78·5 (70·2 to 88·2)	23·2 (15·8 to 32·5)	6·7 (6·7 to 6·7)
Côte d'Ivoire*	2005	56·8 (51·6 to 62·1)	8·6 (7·1 to 10·3)	35·7 (32·0 to 39·6)	9·8 (6·8 to 13·6)	2·8 (2·8 to 2·8)	133·5 (121·3 to 145·8)	20·2 (16·7 to 24·3)	83·8 (75·3 to 93·1)	22·9 (16·0 to 31·9)	6·6 (6·6 to 6·6)
Côte d'Ivoire*	2006	61·7 (56·5 to 67·2)	8·9 (7·4 to 10·6)	38·7 (35·0 to 42·6)	9·9 (7·0 to 13·7)	4·2 (4·2 to 4·2)	144·9 (132·7 to 157·8)	20·9 (17·4 to 24·8)	91·0 (82·2 to 100·0)	23·2 (16·4 to 32·2)	9·8 (9·8 to 9·8)
Côte d'Ivoire*	2007	64·1 (58·8 to 69·5)	9·1 (7·7 to 10·7)	41·1 (37·2 to 44·8)	10·0 (7·1 to 13·8)	3·9 (3·9 to 3·9)	150·6 (138·2 to 163·2)	21·4 (18·1 to 25·2)	96·5 (87·4 to 105·3)	23·5 (16·6 to 32·3)	9·2 (9·2 to 9·2)
Côte d'Ivoire*	2008	68·3 (63·3 to 73·4)	9·3 (8·0 to 10·8)	41·6 (38·3 to 45·0)	10·0 (7·1 to 13·9)	7·4 (7·4 to 7·4)	160·4 (148·7 to 172·5)	21·7 (18·7 to 25·3)	97·8 (90·1 to 105·8)	23·4 (16·7 to 32·6)	17·4 (17·4 to 17·4)
Côte d'Ivoire*	2009	66·8 (62·1 to 71·6)	9·5 (8·4 to 10·9)	41·6 (38·8 to 44·6)	10·0 (7·2 to 13·9)	5·6 (5·6 to 5·6)	156·8 (146·0 to 168·2)	22·4 (19·7 to 25·7)	97·7 (91·1 to 104·8)	23·6 (16·9 to 32·6)	13·1 (13·1 to 13·1)
Côte d'Ivoire*	2010	69·3 (65·1 to 73·9)	9·7 (8·6 to 10·8)	40·5 (38·1 to 43·2)	10·1 (7·2 to 14·1)	9·0 (9·0 to 9·0)	162·8 (152·9 to 173·6)	22·7 (20·3 to 25·3)	95·1 (89·5 to 101·4)	23·7 (16·9 to 33·1)	21·2 (21·2 to 21·2)
Côte d'Ivoire*	2011	60·7 (56·9 to 65·1)	9·1 (8·3 to 9·9)	37·1 (34·8 to 39·6)	9·6 (6·8 to 13·4)	5·0 (5·0 to 5·0)	142·6 (133·8 to 153·0)	21·3 (19·5 to 23·2)	87·2 (81·7 to 93·0)	22·4 (16·0 to 31·5)	11·7 (11·7 to 11·7)
Côte d'Ivoire*	2012	65·1 (61·3 to 69·8)	11·4 (10·7 to 12·2)	37·3 (34·9 to 39·8)	10·4 (7·3 to 14·6)	6·0 (6·0 to 6·0)	153·0 (144·0 to 163·9)	26·8 (25·1 to 28·8)	87·6 (82·0 to 93·5)	24·4 (17·2 to 34·4)	14·1 (14·1 to 14·1)

Côte d'Ivoire*	2013	64·9 (61·0 to 69·8)	12·4 (11·7 to 13·2)	34·9 (32·6 to 37·3)	10·3 (7·2 to 14·5)	7·3 (7·3 to 7·3)	152·6 (143·3 to 163·9)	29·2 (27·6 to 30·9)	81·9 (76·7 to 87·6)	24·3 (16·9 to 34·1)	17·2 (17·2 to 17·2)
Côte d'Ivoire*	2014	65·8 (61·7 to 70·7)	13·4 (12·7 to 14·2)	32·0 (29·8 to 34·4)	10·6 (7·4 to 14·7)	9·7 (9·7 to 9·7)	154·5 (145·0 to 166·1)	31·5 (29·8 to 33·5)	75·2 (70·0 to 80·9)	25·0 (17·5 to 34·6)	22·9 (22·9 to 22·9)
Côte d'Ivoire*	2015	63·2 (58·8 to 68·6)	15·3 (14·3 to 16·4)	29·3 (27·0 to 31·6)	12·0 (8·4 to 16·5)	6·6 (6·6 to 6·6)	148·4 (138·0 to 161·3)	35·9 (33·5 to 38·5)	68·8 (63·4 to 74·1)	28·2 (19·7 to 38·7)	15·5 (15·5 to 15·5)
Côte d'Ivoire*	2016	68·1 (63·6 to 73·9)	17·0 (15·5 to 18·6)	27·9 (25·7 to 30·2)	13·0 (9·2 to 17·9)	10·2 (10·2 to 10·2)	160·0 (149·3 to 173·6)	39·9 (36·3 to 43·7)	65·6 (60·4 to 71·0)	30·6 (21·6 to 42·0)	23·9 (23·9 to 23·9)
Côte d'Ivoire*	2017	70·5 (65·4 to 76·2)	18·6 (16·4 to 20·9)	27·4 (25·2 to 29·7)	13·6 (9·6 to 18·7)	10·9 (10·9 to 10·9)	165·5 (153·6 to 179·0)	43·6 (38·4 to 49·1)	64·3 (59·3 to 69·9)	32·0 (22·6 to 44·0)	25·6 (25·6 to 25·6)
Côte d'Ivoire*	2018	70·7 (64·6 to 77·2)	19·3 (16·6 to 22·1)	27·2 (24·4 to 30·1)	13·9 (9·7 to 19·0)	10·4 (10·4 to 10·4)	166·2 (151·7 to 181·4)	45·3 (39·0 to 52·0)	63·9 (57·4 to 70·6)	32·7 (22·8 to 44·7)	24·4 (24·4 to 24·4)
Côte d'Ivoire*	2019	72·2 (65·8 to 79·0)	19·8 (17·0 to 22·8)	27·9 (25·1 to 30·8)	14·7 (10·2 to 20·1)	9·8 (9·7 to 9·9)	169·7 (154·6 to 185·6)	46·6 (40·0 to 53·5)	65·6 (58·9 to 72·5)	34·5 (24·1 to 47·2)	23·0 (22·7 to 23·2)
Côte d'Ivoire*	2020	75·6 (69·2 to 82·4)	21·9 (18·8 to 25·1)	26·7 (24·0 to 29·5)	14·4 (10·1 to 19·8)	12·6 (12·4 to 12·7)	177·6 (162·7 to 193·7)	51·3 (44·1 to 58·9)	62·8 (56·4 to 69·4)	33·9 (23·7 to 46·4)	29·6 (29·2 to 29·9)
Côte d'Ivoire*	2021	73·1 (66·9 to 79·8)	20·9 (18·0 to 24·0)	25·5 (22·9 to 28·2)	14·1 (9·9 to 19·3)	12·6 (12·4 to 12·8)	171·8 (157·2 to 187·5)	49·1 (42·2 to 56·3)	60·0 (53·9 to 66·3)	33·1 (23·2 to 45·4)	29·6 (29·2 to 30·2)
Côte d'Ivoire*	2022	69·2 (63·0 to 75·8)	19·3 (16·6 to 22·3)	25·6 (23·0 to 28·3)	14·5 (10·1 to 19·9)	9·8 (9·3 to 10·3)	162·6 (148·1 to 178·0)	45·4 (39·1 to 52·3)	60·2 (54·2 to 66·5)	34·0 (23·8 to 46·7)	22·9 (21·8 to 24·3)
Côte d'Ivoire*	2023	70·4 (64·1 to 77·2)	19·8 (17·1 to 22·8)	25·7 (23·2 to 28·4)	14·9 (10·5 to 20·3)	10·0 (9·4 to 10·6)	165·4 (150·6 to 181·5)	46·6 (40·1 to 53·6)	60·5 (54·4 to 66·8)	35·0 (24·6 to 47·8)	23·4 (22·1 to 25·0)
Côte d'Ivoire*	2024	71·6 (65·0 to 78·6)	20·2 (17·3 to 23·3)	25·9 (23·3 to 28·6)	15·3 (10·8 to 21·0)	10·1 (9·5 to 10·9)	168·2 (152·6 to 184·8)	47·5 (40·7 to 54·8)	60·8 (54·7 to 67·2)	36·0 (25·3 to 49·4)	23·8 (22·3 to 25·6)
Côte d'Ivoire*	2025	72·6 (65·8 to 79·8)	20·5 (17·5 to 23·7)	26·0 (23·4 to 28·8)	15·8 (11·1 to 21·6)	10·3 (9·6 to 11·1)	170·6 (154·5 to 187·4)	48·1 (41·1 to 55·7)	61·2 (55·0 to 67·6)	37·0 (26·1 to 50·9)	24·2 (22·6 to 26·1)
Côte d'Ivoire*	2026	73·7 (66·8 to 81·1)	20·8 (17·8 to 24·2)	26·2 (23·6 to 29·0)	16·2 (11·4 to 22·2)	10·4 (9·7 to 11·3)	173·1 (157·0 to 190·4)	48·8 (41·8 to 56·8)	61·6 (55·4 to 68·0)	38·2 (26·8 to 52·2)	24·5 (22·7 to 26·6)

Côte d'Ivoire*	2027	74·7 (67·6 to 82·4)	21·1 (18·0 to 24·6)	26·4 (23·7 to 29·1)	16·7 (11·8 to 22·9)	10·5 (9·7 to 11·5)	175·6 (158·9 to 193·6)	49·6 (42·4 to 57·8)	62·0 (55·7 to 68·5)	39·3 (27·8 to 53·8)	24·7 (22·8 to 27·0)
Côte d'Ivoire*	2028	75·9 (68·5 to 83·8)	21·4 (18·3 to 25·1)	26·6 (23·9 to 29·4)	17·3 (12·1 to 23·7)	10·6 (9·7 to 11·7)	178·2 (160·9 to 196·9)	50·4 (43·0 to 58·9)	62·4 (56·2 to 69·0)	40·5 (28·5 to 55·6)	24·9 (22·8 to 27·5)
Côte d'Ivoire*	2029	77·0 (69·5 to 85·2)	21·8 (18·5 to 25·5)	26·8 (24·1 to 29·6)	17·8 (12·5 to 24·6)	10·6 (9·7 to 11·8)	180·9 (163·4 to 200·1)	51·2 (43·6 to 60·0)	63·0 (56·6 to 69·5)	41·8 (29·4 to 57·7)	25·0 (22·8 to 27·7)
Côte d'Ivoire*	2030	78·2 (70·7 to 86·7)	22·2 (18·9 to 26·1)	27·1 (24·3 to 29·9)	18·4 (12·9 to 25·4)	10·6 (9·6 to 11·8)	183·8 (166·1 to 203·7)	52·1 (44·4 to 61·2)	63·6 (57·2 to 70·2)	43·2 (30·2 to 59·8)	24·9 (22·6 to 27·7)
Côte d'Ivoire*	2031	79·5 (71·7 to 88·3)	22·6 (19·2 to 26·6)	27·3 (24·6 to 30·2)	19·0 (13·3 to 26·4)	10·6 (9·5 to 11·9)	186·8 (168·5 to 207·5)	53·0 (45·2 to 62·5)	64·2 (57·7 to 70·9)	44·6 (31·1 to 61·9)	24·9 (22·4 to 27·9)
Côte d'Ivoire*	2032	80·8 (72·7 to 89·8)	23·0 (19·5 to 27·2)	27·6 (24·8 to 30·5)	19·6 (13·7 to 27·3)	10·6 (9·5 to 11·9)	189·9 (170·9 to 211·1)	54·0 (45·8 to 64·0)	64·8 (58·3 to 71·6)	46·1 (32·2 to 64·2)	24·9 (22·4 to 28·0)
Côte d'Ivoire*	2033	82·2 (73·7 to 91·5)	23·4 (19·8 to 27·7)	27·9 (25·1 to 30·8)	20·3 (14·1 to 28·2)	10·6 (9·5 to 12·0)	193·0 (173·1 to 215·0)	55·0 (46·6 to 65·0)	65·5 (58·9 to 72·4)	47·7 (33·1 to 66·3)	24·9 (22·3 to 28·2)
Côte d'Ivoire*	2034	83·6 (74·7 to 93·2)	23·8 (20·1 to 28·2)	28·2 (25·3 to 31·1)	21·0 (14·5 to 29·2)	10·6 (9·5 to 12·0)	196·3 (175·6 to 218·9)	56·0 (47·2 to 66·2)	66·1 (59·5 to 73·1)	49·3 (34·1 to 68·6)	24·9 (22·3 to 28·2)
Côte d'Ivoire*	2035	85·0 (75·9 to 95·1)	24·2 (20·4 to 28·7)	28·4 (25·5 to 31·5)	21·7 (15·0 to 30·1)	10·6 (9·5 to 12·1)	199·7 (178·2 to 223·5)	57·0 (47·9 to 67·4)	66·8 (60·0 to 73·9)	50·9 (35·2 to 70·8)	25·0 (22·3 to 28·5)
Côte d'Ivoire*	2036	86·5 (76·9 to 97·0)	24·7 (20·8 to 29·2)	28·7 (25·8 to 31·8)	22·4 (15·4 to 31·2)	10·6 (9·5 to 12·1)	203·1 (180·7 to 227·9)	58·0 (48·8 to 68·7)	67·5 (60·6 to 74·7)	52·6 (36·2 to 73·2)	25·0 (22·2 to 28·5)
Côte d'Ivoire*	2037	88·0 (78·3 to 98·9)	25·1 (21·0 to 29·8)	29·0 (26·1 to 32·1)	23·1 (15·8 to 32·3)	10·7 (9·5 to 12·2)	206·6 (183·9 to 232·4)	59·1 (49·4 to 70·0)	68·2 (61·3 to 75·5)	54·4 (37·1 to 75·8)	25·0 (22·3 to 28·6)
Côte d'Ivoire*	2038	89·5 (79·5 to 100·9)	25·6 (21·4 to 30·3)	29·3 (26·4 to 32·5)	23·9 (16·3 to 33·4)	10·7 (9·4 to 12·2)	210·4 (186·9 to 237·0)	60·2 (50·2 to 71·1)	68·9 (62·0 to 76·3)	56·2 (38·3 to 78·6)	25·0 (22·2 to 28·7)
Côte d'Ivoire*	2039	91·1 (80·8 to 102·7)	26·1 (21·7 to 30·9)	29·6 (26·7 to 32·8)	24·7 (16·7 to 34·6)	10·7 (9·4 to 12·4)	214·1 (189·9 to 241·4)	61·3 (51·0 to 72·7)	69·6 (62·6 to 77·1)	58·1 (39·3 to 81·3)	25·1 (22·1 to 29·1)
Côte d'Ivoire*	2040	92·8 (81·9 to 104·8)	26·6 (21·9 to 31·6)	29·9 (26·9 to 33·2)	25·6 (17·4 to 35·9)	10·7 (9·4 to 12·5)	218·0 (192·4 to 246·3)	62·5 (51·4 to 74·2)	70·3 (63·2 to 77·9)	60·1 (40·8 to 84·3)	25·2 (22·1 to 29·3)

Côte d'Ivoire*	2041	94·5 (83·1 to 107·0)	27·1 (22·2 to 32·4)	30·2 (27·1 to 33·4)	26·4 (17·9 to 37·2)	10·8 (9·4 to 12·7)	221·9 (195·1 to 251·3)	63·6 (52·2 to 76·0)	70·9 (63·7 to 78·6)	62·0 (42·0 to 87·4)	25·4 (22·2 to 29·7)
Côte d'Ivoire*	2042	96·1 (84·3 to 109·2)	27·5 (22·7 to 32·9)	30·4 (27·3 to 33·7)	27·2 (18·3 to 38·5)	10·9 (9·5 to 12·9)	225·8 (198·1 to 256·6)	64·7 (53·3 to 77·4)	71·5 (64·2 to 79·2)	64·0 (42·9 to 90·4)	25·6 (22·2 to 30·3)
Côte d'Ivoire*	2043	97·8 (85·4 to 111·3)	28·0 (23·0 to 33·5)	30·7 (27·5 to 34·0)	28·1 (18·7 to 39·9)	11·0 (9·5 to 13·1)	229·8 (200·6 to 261·5)	65·7 (54·0 to 78·7)	72·1 (64·7 to 79·9)	66·1 (43·9 to 93·8)	25·9 (22·4 to 30·9)
Côte d'Ivoire*	2044	99·6 (86·7 to 113·8)	28·5 (23·4 to 34·1)	31·0 (27·8 to 34·4)	29·0 (19·1 to 41·4)	11·1 (9·6 to 13·2)	233·9 (203·8 to 267·4)	66·9 (55·1 to 80·1)	72·8 (65·3 to 80·7)	68·2 (44·9 to 97·4)	26·1 (22·5 to 30·9)
Côte d'Ivoire*	2045	101·3 (87·9 to 116·2)	28·9 (23·7 to 34·9)	31·2 (28·0 to 34·6)	30·0 (19·5 to 43·0)	11·2 (9·6 to 13·4)	237·9 (206·5 to 273·0)	67·9 (55·6 to 82·0)	73·4 (65·7 to 81·4)	70·4 (45·9 to 101·0)	26·3 (22·6 to 31·4)
Côte d'Ivoire*	2046	103·0 (88·9 to 118·6)	29·4 (24·0 to 35·8)	31·5 (28·2 to 35·0)	30·9 (20·1 to 44·6)	11·3 (9·7 to 13·5)	242·1 (209·0 to 278·5)	69·0 (56·3 to 84·1)	73·9 (66·2 to 82·1)	72·6 (47·1 to 104·8)	26·5 (22·7 to 31·8)
Côte d'Ivoire*	2047	104·9 (90·4 to 121·1)	29·9 (24·3 to 36·6)	31·7 (28·4 to 35·3)	31·9 (20·8 to 46·2)	11·4 (9·7 to 13·7)	246·4 (212·4 to 284·5)	70·2 (57·0 to 86·0)	74·6 (66·8 to 82·8)	74·9 (48·8 to 108·7)	26·7 (22·7 to 32·2)
Côte d'Ivoire*	2048	106·7 (91·7 to 123·5)	30·4 (24·5 to 37·5)	32·0 (28·6 to 35·5)	32·9 (21·3 to 48·0)	11·4 (9·8 to 13·9)	250·7 (215·4 to 290·2)	71·4 (57·6 to 88·0)	75·2 (67·3 to 83·5)	77·3 (50·0 to 112·7)	26·9 (22·9 to 32·5)
Côte d'Ivoire*	2049	108·5 (92·9 to 126·2)	30·8 (24·8 to 38·0)	32·2 (28·8 to 35·8)	33·9 (21·9 to 49·7)	11·5 (9·8 to 14·0)	254·9 (218·3 to 296·4)	72·4 (58·4 to 89·3)	75·7 (67·8 to 84·1)	79·7 (51·5 to 116·8)	27·1 (23·0 to 33·0)
Côte d'Ivoire*	2050	110·3 (94·1 to 128·6)	31·3 (25·2 to 38·8)	32·5 (29·1 to 36·1)	35·0 (22·4 to 51·9)	11·6 (9·8 to 14·1)	259·2 (221·1 to 302·1)	73·5 (59·1 to 91·2)	76·3 (68·3 to 84·9)	82·1 (52·6 to 122·0)	27·3 (23·0 to 33·1)
Cameroon*	1995	45·6 (38·3 to 54·3)	7·4 (6·1 to 9·0)	35·2 (28·1 to 44·0)	2·7 (1·8 to 3·7)	0·3 (0·3 to 0·3)	113·4 (95·2 to 134·9)	18·5 (15·1 to 22·3)	87·4 (69·9 to 109·3)	6·7 (4·6 to 9·3)	0·9 (0·9 to 0·9)
Cameroon*	1996	50·1 (42·9 to 58·7)	7·5 (6·0 to 9·1)	35·5 (28·5 to 44·0)	2·5 (1·7 to 3·4)	4·7 (4·7 to 4·7)	124·6 (106·5 to 145·8)	18·6 (14·9 to 22·5)	88·1 (70·8 to 109·4)	6·1 (4·2 to 8·6)	11·8 (11·8 to 11·8)
Cameroon*	1997	47·2 (40·1 to 55·1)	7·6 (6·1 to 9·1)	35·5 (28·8 to 43·6)	2·3 (1·6 to 3·2)	1·7 (1·7 to 1·7)	117·2 (99·7 to 136·9)	19·0 (15·2 to 22·7)	88·3 (71·5 to 108·3)	5·6 (3·9 to 7·8)	4·3 (4·3 to 4·3)
Cameroon*	1998	47·6 (40·9 to 55·2)	7·8 (6·3 to 9·4)	35·7 (29·0 to 43·3)	2·1 (1·5 to 3·0)	1·9 (1·9 to 1·9)	118·2 (101·7 to 137·1)	19·3 (15·7 to 23·3)	88·7 (72·2 to 107·6)	5·3 (3·7 to 7·5)	4·8 (4·8 to 4·8)

Cameroon*	1999	48·1 (41·3 to 55·6)	8·2 (6·7 to 9·9)	36·3 (29·8 to 43·5)	2·1 (1·5 to 3·0)	1·4 (1·4 to 1·4)	119·4 (102·6 to 138·2)	20·4 (16·6 to 24·6)	90·3 (73·9 to 108·0)	5·3 (3·7 to 7·5)	3·4 (3·4 to 3·4)
Cameroon*	2000	49·3 (42·4 to 57·3)	8·7 (7·1 to 10·4)	37·4 (30·4 to 45·2)	2·2 (1·5 to 3·0)	1·0 (1·0 to 1·0)	122·4 (105·4 to 142·3)	21·7 (17·5 to 25·9)	92·9 (75·6 to 112·3)	5·4 (3·7 to 7·5)	2·5 (2·5 to 2·5)
Cameroon*	2001	50·8 (43·7 to 59·0)	9·5 (7·7 to 11·5)	37·6 (30·5 to 45·3)	2·2 (1·5 to 3·1)	1·5 (1·5 to 1·5)	126·2 (108·7 to 146·6)	23·6 (19·1 to 28·6)	93·4 (75·7 to 112·7)	5·5 (3·8 to 7·7)	3·8 (3·8 to 3·8)
Cameroon*	2002	50·6 (43·6 to 58·7)	9·8 (8·0 to 11·9)	37·6 (31·1 to 45·3)	2·2 (1·5 to 3·1)	0·9 (0·9 to 0·9)	125·7 (108·3 to 146·0)	24·5 (19·8 to 29·6)	93·5 (77·2 to 112·6)	5·4 (3·7 to 7·6)	2·3 (2·3 to 2·3)
Cameroon*	2003	51·7 (44·6 to 60·0)	9·8 (7·9 to 11·9)	37·8 (31·1 to 46·0)	2·1 (1·5 to 3·0)	1·9 (1·9 to 1·9)	128·5 (110·9 to 149·1)	24·4 (19·6 to 29·5)	94·0 (77·2 to 114·3)	5·3 (3·6 to 7·4)	4·8 (4·8 to 4·8)
Cameroon*	2004	52·5 (45·7 to 60·8)	9·4 (7·6 to 11·4)	38·0 (31·5 to 46·1)	2·0 (1·4 to 2·8)	3·1 (3·1 to 3·1)	130·6 (113·7 to 151·0)	23·4 (18·9 to 28·4)	94·3 (78·2 to 114·5)	5·0 (3·4 to 7·0)	7·8 (7·8 to 7·8)
Cameroon*	2005	50·7 (44·1 to 58·7)	8·5 (6·9 to 10·3)	37·1 (30·8 to 45·0)	1·9 (1·3 to 2·7)	3·2 (3·2 to 3·2)	125·9 (109·5 to 145·8)	21·0 (17·1 to 25·5)	92·2 (76·6 to 111·9)	4·7 (3·2 to 6·6)	8·0 (8·0 to 8·0)
Cameroon*	2006	50·4 (44·1 to 57·8)	7·9 (6·4 to 9·5)	36·5 (30·4 to 44·0)	1·9 (1·3 to 2·6)	4·1 (4·1 to 4·1)	125·2 (109·7 to 143·7)	19·6 (15·9 to 23·7)	90·8 (75·6 to 109·4)	4·8 (3·3 to 6·5)	10·1 (10·1 to 10·1)
Cameroon*	2007	49·5 (43·2 to 56·9)	7·4 (6·0 to 8·9)	36·1 (30·2 to 43·4)	2·1 (1·4 to 2·9)	3·9 (3·9 to 3·9)	123·0 (107·2 to 141·4)	18·4 (15·0 to 22·1)	89·7 (75·0 to 107·8)	5·1 (3·5 to 7·1)	9·7 (9·7 to 9·7)
Cameroon*	2008	49·5 (43·5 to 56·8)	7·3 (5·9 to 8·9)	36·1 (30·1 to 43·2)	2·2 (1·5 to 3·0)	3·9 (3·9 to 3·9)	123·1 (108·1 to 141·2)	18·2 (14·8 to 22·2)	89·7 (74·7 to 107·4)	5·4 (3·7 to 7·5)	9·8 (9·8 to 9·8)
Cameroon*	2009	46·9 (40·6 to 54·1)	6·4 (5·2 to 7·7)	34·4 (28·4 to 41·3)	2·1 (1·4 to 3·0)	4·0 (4·0 to 4·0)	116·4 (100·9 to 134·3)	15·8 (12·9 to 19·2)	85·4 (70·5 to 102·6)	5·3 (3·6 to 7·4)	9·9 (9·9 to 9·9)
Cameroon*	2010	44·9 (38·3 to 52·0)	6·1 (4·9 to 7·4)	33·6 (27·6 to 40·6)	2·1 (1·5 to 3·0)	3·1 (3·1 to 3·1)	111·4 (95·2 to 129·2)	15·1 (12·3 to 18·3)	83·4 (68·6 to 100·9)	5·3 (3·7 to 7·5)	7·6 (7·6 to 7·6)
Cameroon*	2011	50·2 (43·9 to 57·5)	6·8 (5·5 to 8·2)	34·4 (28·2 to 41·7)	2·4 (1·7 to 3·4)	6·6 (6·6 to 6·6)	124·7 (109·0 to 142·8)	16·9 (13·7 to 20·3)	85·6 (70·0 to 103·6)	6·0 (4·3 to 8·5)	16·3 (16·3 to 16·3)
Cameroon*	2012	48·2 (41·9 to 55·1)	6·5 (5·3 to 7·8)	33·6 (27·5 to 40·5)	3·5 (2·5 to 4·9)	4·6 (4·6 to 4·6)	119·8 (104·1 to 137·0)	16·2 (13·2 to 19·4)	83·6 (68·4 to 100·7)	8·7 (6·1 to 12·1)	11·4 (11·4 to 11·4)

Cameroon*	2013	50·6 (44·1 to 57·7)	6·5 (5·2 to 7·8)	34·1 (27·7 to 40·6)	4·2 (3·0 to 6·0)	5·8 (5·8 to 5·8)	125·7 (109·7 to 143·3)	16·1 (13·0 to 19·3)	84·6 (68·8 to 101·0)	10·5 (7·4 to 14·8)	14·5 (14·5 to 14·5)
Cameroon*	2014	49·7 (43·4 to 56·7)	6·2 (5·1 to 7·4)	34·3 (27·9 to 40·8)	4·7 (3·2 to 6·6)	4·5 (4·5 to 4·5)	123·5 (107·8 to 141·0)	15·4 (12·7 to 18·5)	85·2 (69·3 to 101·3)	11·7 (8·1 to 16·5)	11·2 (11·2 to 11·2)
Cameroon*	2015	50·4 (43·9 to 57·1)	5·3 (4·3 to 6·3)	34·1 (27·6 to 40·5)	4·8 (3·3 to 6·7)	6·2 (6·2 to 6·2)	125·3 (109·1 to 141·8)	13·1 (10·8 to 15·6)	84·8 (68·7 to 100·6)	12·0 (8·3 to 16·7)	15·4 (15·4 to 15·4)
Cameroon*	2016	48·0 (41·5 to 55·3)	4·5 (3·7 to 5·4)	34·1 (27·9 to 40·9)	4·9 (3·3 to 6·9)	4·6 (4·6 to 4·6)	119·3 (103·0 to 137·4)	11·1 (9·1 to 13·3)	84·7 (69·3 to 101·7)	12·2 (8·3 to 17·0)	11·3 (11·3 to 11·3)
Cameroon*	2017	49·9 (43·6 to 57·3)	3·4 (2·8 to 4·1)	33·1 (26·9 to 40·0)	4·6 (3·1 to 6·5)	8·9 (8·9 to 8·9)	124·0 (108·3 to 142·4)	8·4 (6·9 to 10·2)	82·1 (66·9 to 99·3)	11·4 (7·7 to 16·2)	22·1 (22·1 to 22·1)
Cameroon*	2018	48·0 (41·2 to 55·1)	3·2 (2·6 to 3·9)	33·3 (26·9 to 40·6)	4·6 (3·1 to 6·5)	7·0 (7·0 to 7·0)	119·3 (102·4 to 136·8)	7·9 (6·5 to 9·6)	82·8 (66·9 to 100·8)	11·3 (7·7 to 16·1)	17·3 (17·3 to 17·3)
Cameroon*	2019	48·6 (41·8 to 55·9)	3·2 (2·6 to 4·0)	33·6 (27·2 to 40·9)	4·7 (3·2 to 6·7)	7·0 (7·0 to 7·1)	120·8 (103·9 to 138·9)	8·0 (6·5 to 9·9)	83·6 (67·5 to 101·7)	11·7 (7·9 to 16·6)	17·5 (17·5 to 17·5)
Cameroon*	2020	52·5 (45·9 to 59·4)	6·5 (5·9 to 7·3)	32·3 (26·1 to 39·4)	4·6 (3·1 to 6·6)	9·0 (8·9 to 9·1)	130·4 (114·0 to 147·7)	16·2 (14·7 to 18·1)	80·4 (64·8 to 97·8)	11·5 (7·7 to 16·3)	22·4 (22·2 to 22·6)
Cameroon*	2021	47·3 (40·9 to 54·0)	2·7 (2·2 to 3·4)	31·2 (25·2 to 38·0)	4·6 (3·1 to 6·5)	8·7 (8·6 to 9·0)	117·4 (101·6 to 134·2)	6·8 (5·4 to 8·6)	77·5 (62·6 to 94·3)	11·4 (7·6 to 16·2)	21·7 (21·4 to 22·2)
Cameroon*	2022	45·8 (39·2 to 52·8)	2·8 (2·2 to 3·7)	31·7 (25·6 to 38·5)	4·8 (3·2 to 6·7)	6·5 (6·2 to 6·9)	113·8 (97·4 to 131·3)	7·1 (5·4 to 9·2)	78·7 (63·5 to 95·8)	11·8 (7·8 to 16·7)	16·3 (15·5 to 17·2)
Cameroon*	2023	46·5 (39·8 to 53·5)	2·9 (2·3 to 3·8)	32·2 (26·0 to 39·2)	4·9 (3·3 to 7·0)	6·4 (6·1 to 6·8)	115·7 (98·9 to 133·0)	7·3 (5·6 to 9·5)	80·1 (64·6 to 97·5)	12·3 (8·2 to 17·5)	16·0 (15·1 to 17·0)
Cameroon*	2024	47·3 (40·5 to 54·4)	3·0 (2·3 to 4·1)	32·9 (26·5 to 40·0)	5·2 (3·4 to 7·4)	6·3 (5·9 to 6·7)	117·6 (100·7 to 135·3)	7·5 (5·6 to 10·1)	81·6 (65·9 to 99·4)	12·8 (8·5 to 18·3)	15·7 (14·7 to 16·7)
Cameroon*	2025	48·2 (41·1 to 55·4)	3·1 (2·3 to 4·2)	33·5 (27·0 to 40·8)	5·4 (3·6 to 7·7)	6·1 (5·8 to 6·6)	119·7 (102·2 to 137·7)	7·8 (5·8 to 10·5)	83·2 (67·2 to 101·4)	13·4 (8·9 to 19·1)	15·3 (14·3 to 16·3)
Cameroon*	2026	49·0 (41·8 to 56·4)	3·2 (2·4 to 4·4)	34·2 (27·6 to 41·7)	5·6 (3·8 to 8·0)	6·0 (5·6 to 6·4)	121·8 (103·9 to 140·1)	8·0 (5·9 to 10·9)	84·9 (68·6 to 103·6)	14·0 (9·3 to 19·9)	14·8 (13·8 to 15·9)

Cameroon*	2027	49·9 (42·4 to 57·4)	3·3 (2·5 to 4·6)	34·9 (28·1 to 42·5)	5·9 (3·9 to 8·4)	5·8 (5·4 to 6·3)	124·0 (105·4 to 142·6)	8·3 (6·1 to 11·5)	86·7 (69·9 to 105·7)	14·6 (9·8 to 20·8)	14·4 (13·4 to 15·6)
Cameroon*	2028	50·8 (43·3 to 58·6)	3·5 (2·5 to 4·8)	35·6 (28·7 to 43·4)	6·1 (4·1 to 8·7)	5·7 (5·2 to 6·2)	126·2 (107·5 to 145·5)	8·6 (6·2 to 12·0)	88·4 (71·3 to 107·8)	15·2 (10·2 to 21·7)	14·0 (13·0 to 15·3)
Cameroon*	2029	51·7 (44·0 to 59·7)	3·6 (2·6 to 4·9)	36·3 (29·3 to 44·3)	6·4 (4·3 to 9·2)	5·5 (5·0 to 6·0)	128·6 (109·3 to 148·3)	8·9 (6·4 to 12·3)	90·1 (72·7 to 110·0)	15·9 (10·6 to 22·7)	13·7 (12·5 to 15·0)
Cameroon*	2030	52·7 (44·9 to 60·8)	3·7 (2·7 to 5·1)	37·0 (29·8 to 45·1)	6·7 (4·4 to 9·5)	5·4 (4·9 to 5·9)	131·0 (111·6 to 151·0)	9·2 (6·6 to 12·8)	91·9 (74·1 to 112·1)	16·5 (11·1 to 23·7)	13·3 (12·1 to 14·7)
Cameroon*	2031	53·7 (45·6 to 62·1)	3·9 (2·7 to 5·4)	37·7 (30·4 to 46·0)	6·9 (4·6 to 9·9)	5·2 (4·7 to 5·8)	133·5 (113·4 to 154·3)	9·6 (6·8 to 13·5)	93·7 (75·5 to 114·4)	17·3 (11·5 to 24·7)	13·0 (11·7 to 14·4)
Cameroon*	2032	54·8 (46·5 to 63·4)	4·0 (2·8 to 5·7)	38·4 (31·0 to 46·9)	7·2 (4·8 to 10·3)	5·1 (4·6 to 5·7)	136·0 (115·5 to 157·5)	9·9 (6·9 to 14·1)	95·5 (77·0 to 116·6)	18·0 (11·9 to 25·7)	12·7 (11·4 to 14·1)
Cameroon*	2033	55·8 (47·4 to 64·6)	4·1 (2·9 to 6·0)	39·1 (31·6 to 47·8)	7·5 (5·0 to 10·8)	5·0 (4·5 to 5·6)	138·7 (117·9 to 160·5)	10·3 (7·1 to 14·8)	97·2 (78·4 to 118·9)	18·7 (12·4 to 26·8)	12·4 (11·1 to 13·8)
Cameroon*	2034	56·9 (48·2 to 65·8)	4·3 (2·9 to 6·2)	39·8 (32·2 to 48·7)	7·9 (5·2 to 11·2)	4·9 (4·4 to 5·5)	141·3 (119·9 to 163·5)	10·7 (7·3 to 15·3)	99·0 (79·9 to 121·1)	19·5 (12·9 to 27·9)	12·2 (10·9 to 13·6)
Cameroon*	2035	58·0 (49·2 to 67·1)	4·4 (3·0 to 6·4)	40·5 (32·8 to 49·6)	8·2 (5·4 to 11·7)	4·8 (4·3 to 5·4)	144·0 (122·1 to 166·8)	11·0 (7·4 to 15·9)	100·7 (81·4 to 123·2)	20·3 (13·5 to 29·0)	12·0 (10·6 to 13·5)
Cameroon*	2036	59·1 (50·1 to 68·5)	4·6 (3·1 to 6·7)	41·2 (33·3 to 50·4)	8·5 (5·6 to 12·2)	4·8 (4·2 to 5·4)	146·7 (124·5 to 170·1)	11·4 (7·6 to 16·7)	102·4 (82·8 to 125·3)	21·1 (14·0 to 30·3)	11·8 (10·5 to 13·4)
Cameroon*	2037	60·2 (50·7 to 69·7)	4·7 (3·2 to 7·0)	41·9 (33·9 to 51·2)	8·8 (5·9 to 12·6)	4·7 (4·1 to 5·4)	149·5 (126·0 to 173·2)	11·8 (7·9 to 17·5)	104·1 (84·1 to 127·3)	21·9 (14·6 to 31·4)	11·7 (10·3 to 13·3)
Cameroon*	2038	61·3 (51·8 to 71·0)	4·9 (3·2 to 7·3)	42·6 (34·4 to 52·1)	9·2 (6·1 to 13·1)	4·7 (4·1 to 5·3)	152·3 (128·8 to 176·5)	12·2 (8·0 to 18·1)	105·8 (85·5 to 129·4)	22·8 (15·2 to 32·6)	11·6 (10·1 to 13·2)
Cameroon*	2039	62·5 (52·8 to 72·6)	5·1 (3·3 to 7·6)	43·3 (35·0 to 53·0)	9·5 (6·3 to 13·6)	4·6 (4·0 to 5·4)	155·3 (131·1 to 180·3)	12·7 (8·2 to 18·8)	107·5 (86·9 to 131·6)	23·7 (15·7 to 33·8)	11·5 (10·0 to 13·3)
Cameroon*	2040	63·8 (53·8 to 74·0)	5·3 (3·4 to 7·9)	44·0 (35·5 to 53·9)	9·9 (6·5 to 14·1)	4·6 (4·0 to 5·4)	158·4 (133·8 to 183·9)	13·1 (8·4 to 19·7)	109·2 (88·2 to 133·8)	24·6 (16·1 to 35·0)	11·5 (9·9 to 13·5)

Cameroon*	2041	65·0 (54·9 to 75·3)	5·5 (3·5 to 8·3)	44·7 (36·0 to 54·7)	10·3 (6·7 to 14·6)	4·6 (3·9 to 5·5)	161·5 (136·4 to 187·2)	13·6 (8·8 to 20·6)	110·9 (89·4 to 136·0)	25·5 (16·6 to 36·2)	11·4 (9·8 to 13·5)
Cameroon*	2042	66·3 (56·0 to 76·6)	5·7 (3·6 to 8·6)	45·3 (36·6 to 55·6)	10·7 (7·0 to 15·1)	4·6 (3·9 to 5·5)	164·7 (139·1 to 190·4)	14·1 (8·9 to 21·5)	112·6 (90·8 to 138·2)	26·5 (17·3 to 37·5)	11·4 (9·7 to 13·7)
Cameroon*	2043	67·6 (57·0 to 78·2)	5·9 (3·7 to 9·0)	46·0 (37·1 to 56·5)	11·1 (7·2 to 15·7)	4·6 (3·9 to 5·6)	167·9 (141·7 to 194·4)	14·6 (9·2 to 22·4)	114·4 (92·2 to 140·3)	27·5 (18·0 to 39·0)	11·5 (9·7 to 13·9)
Cameroon*	2044	68·9 (58·1 to 79·7)	6·1 (3·8 to 9·4)	46·7 (37·6 to 57·3)	11·5 (7·5 to 16·3)	4·6 (3·9 to 5·7)	171·2 (144·5 to 198·0)	15·2 (9·3 to 23·4)	116·0 (93·5 to 142·3)	28·5 (18·7 to 40·5)	11·5 (9·7 to 14·1)
Cameroon*	2045	70·2 (59·2 to 81·2)	6·3 (3·8 to 9·9)	47·4 (38·1 to 58·1)	11·9 (7·8 to 17·0)	4·7 (3·9 to 5·7)	174·5 (147·2 to 201·8)	15·8 (9·6 to 24·6)	117·7 (94·8 to 144·4)	29·5 (19·3 to 42·1)	11·6 (9·7 to 14·1)
Cameroon*	2046	71·6 (60·2 to 82·9)	6·6 (3·9 to 10·4)	48·0 (38·6 to 58·9)	12·3 (8·0 to 17·6)	4·7 (3·9 to 5·9)	177·9 (149·5 to 205·9)	16·3 (9·8 to 25·9)	119·3 (95·9 to 146·4)	30·6 (19·9 to 43·8)	11·7 (9·7 to 14·6)
Cameroon*	2047	73·0 (61·4 to 84·4)	6·8 (4·0 to 10·7)	48·7 (39·1 to 59·7)	12·7 (8·3 to 18·3)	4·7 (3·9 to 5·9)	181·3 (152·4 to 209·7)	17·0 (10·0 to 26·6)	120·9 (97·1 to 148·4)	31·7 (20·5 to 45·5)	11·8 (9·7 to 14·7)
Cameroon*	2048	74·3 (62·4 to 86·2)	7·1 (4·2 to 11·3)	49·3 (39·6 to 60·5)	13·2 (8·5 to 19·0)	4·8 (3·9 to 6·0)	184·7 (155·2 to 214·1)	17·6 (10·4 to 28·0)	122·5 (98·3 to 150·2)	32·7 (21·1 to 47·2)	11·9 (9·7 to 15·0)
Cameroon*	2049	75·7 (63·5 to 87·6)	7·4 (4·3 to 11·9)	49·9 (40·0 to 61·2)	13·6 (8·7 to 19·6)	4·9 (4·0 to 6·1)	188·2 (157·9 to 217·7)	18·3 (10·7 to 29·5)	124·0 (99·5 to 152·1)	33·9 (21·6 to 48·8)	12·1 (9·8 to 15·3)
Cameroon*	2050	77·2 (64·7 to 89·7)	7·6 (4·4 to 12·1)	50·5 (40·5 to 62·0)	14·1 (9·0 to 20·4)	4·9 (4·0 to 6·2)	191·7 (160·7 to 222·9)	19·0 (10·9 to 30·1)	125·5 (100·6 to 154·0)	35·0 (22·3 to 50·8)	12·3 (9·9 to 15·5)
DR Congo*	1995	18·6 (15·0 to 22·8)	0·7 (0·6 to 0·9)	16·6 (13·2 to 20·7)	0·8 (0·5 to 1·1)	0·5 (0·5 to 0·5)	39·8 (32·2 to 48·8)	1·5 (1·3 to 1·8)	35·6 (28·2 to 44·3)	1·7 (1·2 to 2·3)	1·0 (1·0 to 1·0)
DR Congo*	1996	17·8 (14·6 to 21·7)	0·7 (0·6 to 0·8)	15·4 (12·2 to 19·3)	0·8 (0·5 to 1·1)	1·0 (1·0 to 1·0)	38·1 (31·2 to 46·5)	1·4 (1·2 to 1·7)	32·9 (26·1 to 41·2)	1·6 (1·1 to 2·3)	2·1 (2·1 to 2·1)
DR Congo*	1997	16·9 (13·9 to 20·4)	0·7 (0·6 to 0·8)	14·8 (11·8 to 18·5)	0·8 (0·5 to 1·1)	0·6 (0·6 to 0·6)	36·1 (29·8 to 43·7)	1·4 (1·2 to 1·8)	31·6 (25·3 to 39·5)	1·6 (1·1 to 2·3)	1·4 (1·4 to 1·4)
DR Congo*	1998	15·5 (12·8 to 18·7)	0·6 (0·5 to 0·8)	13·4 (10·8 to 16·6)	0·8 (0·5 to 1·1)	0·7 (0·7 to 0·7)	33·2 (27·5 to 40·1)	1·3 (1·1 to 1·7)	28·7 (23·2 to 35·6)	1·6 (1·1 to 2·4)	1·5 (1·5 to 1·5)
DR Congo*	1999	12·0 (10·0 to 14·4)	0·5 (0·4 to 0·6)	10·0 (8·0 to 12·3)	0·7 (0·5 to 1·0)	0·8 (0·8 to 0·8)	25·7 (21·4 to 30·9)	1·0 (0·9 to 1·3)	21·4 (17·1 to 26·4)	1·5 (1·0 to 2·2)	1·7 (1·7 to 1·7)

DR Congo*	2000	8·3 (7·1 to 9·8)	0·4 (0·3 to 0·4)	6·4 (5·1 to 7·9)	0·6 (0·4 to 0·9)	0·9 (0·9 to 0·9)	17·8 (15·1 to 21·0)	0·8 (0·6 to 0·9)	13·7 (11·0 to 17·0)	1·4 (0·9 to 1·9)	1·9 (1·9 to 1·9)
DR Congo*	2001	9·0 (7·5 to 10·6)	0·4 (0·4 to 0·5)	6·9 (5·5 to 8·5)	0·7 (0·5 to 1·0)	0·9 (0·9 to 0·9)	19·2 (16·1 to 22·6)	0·9 (0·8 to 1·1)	14·7 (11·7 to 18·1)	1·5 (1·0 to 2·1)	2·0 (2·0 to 2·0)
DR Congo*	2002	8·9 (7·5 to 10·5)	0·4 (0·3 to 0·5)	6·8 (5·4 to 8·4)	0·6 (0·4 to 0·9)	1·0 (1·0 to 1·0)	18·9 (16·0 to 22·4)	0·9 (0·7 to 1·1)	14·6 (11·5 to 17·9)	1·4 (0·9 to 1·9)	2·1 (2·1 to 2·1)
DR Congo*	2003	11·9 (10·1 to 14·2)	0·7 (0·6 to 0·9)	8·9 (7·0 to 10·9)	0·8 (0·5 to 1·1)	1·5 (1·5 to 1·5)	25·5 (21·5 to 30·3)	1·6 (1·3 to 1·9)	19·0 (15·1 to 23·4)	1·7 (1·1 to 2·4)	3·3 (3·3 to 3·3)
DR Congo*	2004	12·3 (10·5 to 14·6)	0·7 (0·6 to 0·9)	9·2 (7·3 to 11·4)	0·7 (0·5 to 1·0)	1·7 (1·7 to 1·7)	26·4 (22·5 to 31·2)	1·5 (1·2 to 1·8)	19·7 (15·7 to 24·4)	1·6 (1·0 to 2·2)	3·7 (3·7 to 3·7)
DR Congo*	2005	13·8 (12·0 to 16·1)	0·8 (0·6 to 1·0)	9·4 (7·6 to 11·6)	0·8 (0·5 to 1·1)	2·9 (2·9 to 2·9)	29·6 (25·7 to 34·4)	1·7 (1·4 to 2·0)	20·1 (16·3 to 24·8)	1·6 (1·1 to 2·3)	6·2 (6·2 to 6·2)
DR Congo*	2006	13·8 (12·1 to 15·9)	0·9 (0·7 to 1·0)	9·2 (7·5 to 11·3)	0·8 (0·5 to 1·1)	3·0 (3·0 to 3·0)	29·6 (25·8 to 34·1)	1·8 (1·5 to 2·2)	19·8 (16·0 to 24·1)	1·6 (1·1 to 2·3)	6·3 (6·3 to 6·3)
DR Congo*	2007	13·2 (11·6 to 15·1)	1·0 (0·8 to 1·2)	8·8 (7·2 to 10·6)	0·8 (0·5 to 1·1)	2·7 (2·7 to 2·7)	28·3 (24·9 to 32·3)	2·1 (1·7 to 2·5)	18·8 (15·5 to 22·8)	1·7 (1·1 to 2·4)	5·8 (5·8 to 5·8)
DR Congo*	2008	16·5 (15·1 to 18·1)	1·2 (1·0 to 1·4)	8·2 (6·9 to 9·8)	0·8 (0·6 to 1·2)	6·3 (6·3 to 6·3)	35·3 (32·3 to 38·7)	2·5 (2·0 to 3·0)	17·6 (14·7 to 21·0)	1·8 (1·2 to 2·6)	13·4 (13·4 to 13·4)
DR Congo*	2009	14·6 (13·4 to 15·8)	1·3 (1·1 to 1·6)	7·1 (6·0 to 8·4)	0·9 (0·6 to 1·2)	5·3 (5·3 to 5·3)	31·2 (28·7 to 33·9)	2·8 (2·3 to 3·4)	15·3 (12·9 to 17·9)	1·9 (1·3 to 2·6)	11·3 (11·3 to 11·3)
DR Congo*	2010	15·3 (14·2 to 16·4)	1·7 (1·4 to 2·1)	6·6 (5·7 to 7·6)	1·0 (0·7 to 1·5)	6·0 (6·0 to 6·0)	32·7 (30·5 to 35·1)	3·6 (3·0 to 4·4)	14·1 (12·2 to 16·3)	2·2 (1·5 to 3·2)	12·8 (12·8 to 12·8)
DR Congo*	2011	14·8 (13·9 to 15·7)	1·8 (1·5 to 2·1)	6·0 (5·2 to 6·8)	0·9 (0·6 to 1·3)	6·1 (6·1 to 6·1)	31·6 (29·7 to 33·7)	3·8 (3·1 to 4·5)	12·8 (11·2 to 14·5)	1·9 (1·3 to 2·7)	13·1 (13·1 to 13·1)
DR Congo*	2012	16·0 (15·1 to 16·9)	2·1 (1·7 to 2·5)	6·2 (5·5 to 6·9)	1·0 (0·7 to 1·4)	6·7 (6·7 to 6·7)	34·2 (32·3 to 36·1)	4·5 (3·7 to 5·4)	13·2 (11·7 to 14·8)	2·1 (1·4 to 2·9)	14·4 (14·4 to 14·4)
DR Congo*	2013	16·8 (16·0 to 17·7)	2·2 (1·8 to 2·6)	6·5 (5·8 to 7·2)	1·1 (0·7 to 1·5)	7·0 (7·0 to 7·0)	35·9 (34·2 to 37·8)	4·7 (4·0 to 5·6)	13·8 (12·5 to 15·4)	2·3 (1·6 to 3·3)	15·0 (15·0 to 15·0)
DR Congo*	2014	18·6 (17·6 to 19·6)	3·0 (2·5 to 3·6)	7·7 (7·0 to 8·5)	1·4 (1·0 to 2·0)	6·4 (6·4 to 6·4)	39·7 (37·7 to 41·8)	6·5 (5·4 to 7·6)	16·4 (14·9 to 18·1)	3·0 (2·1 to 4·3)	13·7 (13·7 to 13·7)
DR Congo*	2015	20·1 (19·1 to 21·1)	3·1 (2·7 to 3·6)	8·1 (7·4 to 8·9)	1·5 (1·0 to 2·1)	7·4 (7·4 to 7·4)	42·9 (40·9 to 45·1)	6·7 (5·7 to 7·8)	17·3 (15·8 to 19·0)	3·2 (2·2 to 4·5)	15·7 (15·7 to 15·7)
DR Congo*	2016	18·5 (17·6 to 19·6)	2·8 (2·4 to 3·2)	8·2 (7·4 to 9·0)	1·5 (1·0 to 2·2)	6·1 (6·1 to 6·1)	39·7 (37·6 to 41·9)	5·9 (5·0 to 6·8)	17·5 (15·9 to 19·2)	3·2 (2·2 to 4·6)	13·1 (13·1 to 13·1)
DR Congo*	2017	18·9 (17·9 to 19·9)	2·4 (2·1 to 2·8)	8·1 (7·3 to 8·9)	1·5 (1·0 to 2·2)	6·9 (6·9 to 6·9)	40·4 (38·3 to 42·6)	5·2 (4·4 to 6·0)	17·2 (15·5 to 19·1)	3·2 (2·2 to 4·6)	14·8 (14·8 to 14·8)

DR Congo*	2018	19·7 (18·6 to 20·9)	2·6 (2·2 to 3·1)	8·1 (7·2 to 9·1)	1·5 (1·0 to 2·2)	7·5 (7·5 to 7·5)	42·1 (39·7 to 44·8)	5·6 (4·8 to 6·6)	17·3 (15·4 to 19·4)	3·3 (2·2 to 4·7)	16·0 (16·0 to 16·0)
DR Congo*	2019	20·3 (19·1 to 21·6)	3·0 (2·5 to 3·6)	8·2 (7·2 to 9·2)	1·6 (1·1 to 2·3)	7·5 (7·5 to 7·6)	43·3 (40·8 to 46·2)	6·4 (5·3 to 7·6)	17·4 (15·5 to 19·6)	3·4 (2·3 to 4·8)	16·1 (16·0 to 16·3)
DR Congo*	2020	23·4 (22·3 to 24·7)	4·0 (3·5 to 4·6)	7·7 (6·8 to 8·6)	1·5 (1·0 to 2·2)	10·2 (10·0 to 10·8)	50·1 (47·6 to 52·9)	8·6 (7·6 to 9·8)	16·4 (14·6 to 18·4)	3·3 (2·2 to 4·7)	21·8 (21·5 to 23·0)
DR Congo*	2021	21·6 (20·5 to 23·0)	2·6 (2·2 to 3·2)	7·3 (6·5 to 8·2)	1·5 (1·0 to 2·1)	10·2 (9·9 to 10·8)	46·3 (43·8 to 49·2)	5·6 (4·6 to 6·8)	15·7 (13·9 to 17·6)	3·2 (2·1 to 4·6)	21·8 (21·3 to 23·0)
DR Congo*	2022	19·5 (18·3 to 21·0)	2·8 (2·2 to 3·4)	7·4 (6·5 to 8·2)	1·5 (1·0 to 2·2)	7·9 (7·4 to 8·5)	41·8 (39·2 to 44·8)	5·9 (4·8 to 7·3)	15·7 (14·0 to 17·6)	3·3 (2·2 to 4·7)	16·9 (15·8 to 18·3)
DR Congo*	2023	19·8 (18·6 to 21·3)	2·9 (2·3 to 3·6)	7·4 (6·6 to 8·3)	1·6 (1·1 to 2·3)	8·0 (7·5 to 8·7)	42·4 (39·7 to 45·5)	6·1 (4·9 to 7·7)	15·8 (14·0 to 17·7)	3·4 (2·3 to 4·8)	17·1 (16·0 to 18·5)
DR Congo*	2024	20·1 (18·7 to 21·6)	3·0 (2·3 to 3·7)	7·4 (6·6 to 8·3)	1·6 (1·1 to 2·3)	8·1 (7·5 to 8·7)	43·0 (40·1 to 46·2)	6·3 (5·0 to 8·0)	15·9 (14·1 to 17·9)	3·5 (2·3 to 5·0)	17·3 (16·1 to 18·7)
DR Congo*	2025	20·4 (18·9 to 21·9)	3·1 (2·4 to 3·9)	7·5 (6·6 to 8·4)	1·7 (1·1 to 2·4)	8·1 (7·5 to 8·8)	43·6 (40·5 to 46·9)	6·5 (5·1 to 8·3)	16·0 (14·2 to 18·0)	3·6 (2·4 to 5·1)	17·4 (16·1 to 18·9)
DR Congo*	2026	20·6 (19·1 to 22·2)	3·2 (2·4 to 4·1)	7·5 (6·7 to 8·5)	1·7 (1·2 to 2·5)	8·2 (7·5 to 9·0)	44·1 (40·8 to 47·6)	6·7 (5·2 to 8·7)	16·1 (14·3 to 18·1)	3·7 (2·5 to 5·3)	17·5 (16·1 to 19·2)
DR Congo*	2027	20·8 (19·3 to 22·6)	3·2 (2·5 to 4·2)	7·6 (6·7 to 8·5)	1·8 (1·2 to 2·5)	8·2 (7·5 to 9·1)	44·5 (41·2 to 48·3)	7·0 (5·3 to 9·1)	16·3 (14·4 to 18·3)	3·8 (2·6 to 5·4)	17·5 (16·1 to 19·4)
DR Congo*	2028	21·0 (19·3 to 22·9)	3·3 (2·5 to 4·4)	7·7 (6·8 to 8·6)	1·8 (1·2 to 2·6)	8·2 (7·4 to 9·1)	44·9 (41·4 to 48·9)	7·1 (5·3 to 9·4)	16·4 (14·5 to 18·4)	3·9 (2·6 to 5·6)	17·5 (15·9 to 19·5)
DR Congo*	2029	21·2 (19·5 to 23·2)	3·4 (2·5 to 4·6)	7·7 (6·8 to 8·7)	1·9 (1·3 to 2·7)	8·1 (7·4 to 9·2)	45·4 (41·8 to 49·6)	7·4 (5·4 to 9·8)	16·5 (14·6 to 18·6)	4·0 (2·7 to 5·8)	17·4 (15·8 to 19·7)
DR Congo*	2030	21·4 (19·6 to 23·4)	3·5 (2·6 to 4·7)	7·8 (6·9 to 8·7)	1·9 (1·3 to 2·8)	8·1 (7·3 to 9·3)	45·8 (42·0 to 50·1)	7·6 (5·6 to 10·0)	16·7 (14·8 to 18·7)	4·2 (2·8 to 6·0)	17·4 (15·6 to 19·9)
DR Congo*	2031	21·6 (19·8 to 23·7)	3·7 (2·6 to 4·9)	7·9 (7·0 to 8·8)	2·0 (1·3 to 2·9)	8·1 (7·3 to 9·4)	46·2 (42·3 to 50·8)	7·8 (5·6 to 10·4)	16·8 (14·9 to 18·9)	4·3 (2·9 to 6·2)	17·3 (15·5 to 20·0)
DR Congo*	2032	21·8 (19·9 to 24·0)	3·8 (2·7 to 5·1)	7·9 (7·0 to 8·9)	2·1 (1·4 to 3·0)	8·1 (7·2 to 9·4)	46·6 (42·5 to 51·3)	8·0 (5·8 to 10·8)	16·9 (15·0 to 19·0)	4·4 (3·0 to 6·4)	17·2 (15·4 to 20·2)
DR Congo*	2033	22·0 (20·1 to 24·4)	3·9 (2·8 to 5·2)	8·0 (7·1 to 9·0)	2·1 (1·4 to 3·1)	8·0 (7·1 to 9·5)	47·1 (43·0 to 52·1)	8·2 (6·0 to 11·1)	17·1 (15·1 to 19·2)	4·6 (3·1 to 6·5)	17·2 (15·3 to 20·2)
DR Congo*	2034	22·2 (20·2 to 24·6)	4·0 (2·8 to 5·5)	8·1 (7·1 to 9·0)	2·2 (1·5 to 3·2)	8·0 (7·1 to 9·5)	47·5 (43·2 to 52·6)	8·5 (6·1 to 11·7)	17·2 (15·3 to 19·3)	4·7 (3·2 to 6·8)	17·1 (15·1 to 20·3)
DR Congo*	2035	22·4 (20·4 to 24·9)	4·1 (2·9 to 5·7)	8·1 (7·2 to 9·1)	2·3 (1·5 to 3·3)	8·0 (7·0 to 9·6)	47·9 (43·5 to 53·2)	8·7 (6·2 to 12·2)	17·4 (15·4 to 19·5)	4·9 (3·3 to 7·0)	17·0 (15·0 to 20·6)

DR Congo*	2036	22·6 (20·4 to 25·2)	4·2 (2·9 to 5·8)	8·2 (7·2 to 9·2)	2·3 (1·6 to 3·4)	7·9 (6·9 to 9·5)	48·3 (43·6 to 53·9)	8·9 (6·3 to 12·5)	17·5 (15·5 to 19·6)	5·0 (3·4 to 7·2)	16·9 (14·8 to 20·4)
DR Congo*	2037	22·8 (20·5 to 25·5)	4·3 (3·0 to 5·9)	8·2 (7·3 to 9·3)	2·4 (1·6 to 3·4)	7·9 (6·8 to 9·6)	48·7 (44·0 to 54·6)	9·1 (6·4 to 12·6)	17·6 (15·6 to 19·8)	5·2 (3·5 to 7·4)	16·8 (14·6 to 20·6)
DR Congo*	2038	23·0 (20·6 to 25·8)	4·4 (3·0 to 6·1)	8·3 (7·4 to 9·3)	2·5 (1·7 to 3·6)	7·8 (6·8 to 9·6)	49·2 (44·1 to 55·1)	9·3 (6·5 to 13·1)	17·8 (15·7 to 20·0)	5·3 (3·6 to 7·6)	16·7 (14·5 to 20·6)
DR Congo*	2039	23·2 (20·7 to 26·1)	4·5 (3·1 to 6·4)	8·4 (7·4 to 9·4)	2·6 (1·7 to 3·7)	7·8 (6·7 to 9·7)	49·6 (44·3 to 55·8)	9·6 (6·6 to 13·6)	17·9 (15·9 to 20·1)	5·5 (3·7 to 7·8)	16·6 (14·3 to 20·8)
DR Congo*	2040	23·4 (20·9 to 26·4)	4·6 (3·2 to 6·5)	8·4 (7·5 to 9·5)	2·6 (1·8 to 3·8)	7·7 (6·6 to 9·7)	50·1 (44·7 to 56·5)	9·8 (6·8 to 13·9)	18·1 (16·0 to 20·3)	5·7 (3·8 to 8·1)	16·5 (14·1 to 20·7)
DR Congo*	2041	23·6 (21·0 to 26·7)	4·7 (3·2 to 6·6)	8·5 (7·5 to 9·5)	2·7 (1·8 to 3·9)	7·7 (6·5 to 9·7)	50·5 (44·9 to 57·2)	10·1 (6·9 to 14·2)	18·2 (16·1 to 20·4)	5·8 (3·9 to 8·3)	16·4 (13·9 to 20·7)
DR Congo*	2042	23·8 (21·1 to 27·0)	4·8 (3·2 to 6·8)	8·6 (7·6 to 9·6)	2·8 (1·9 to 4·0)	7·6 (6·3 to 9·7)	50·9 (45·2 to 57·8)	10·3 (6·9 to 14·6)	18·4 (16·2 to 20·6)	6·0 (4·0 to 8·6)	16·2 (13·6 to 20·8)
DR Congo*	2043	24·0 (21·2 to 27·2)	4·9 (3·3 to 7·0)	8·7 (7·7 to 9·7)	2·9 (1·9 to 4·1)	7·5 (6·3 to 9·7)	51·3 (45·4 to 58·3)	10·5 (7·1 to 14·9)	18·5 (16·4 to 20·8)	6·2 (4·1 to 8·8)	16·0 (13·4 to 20·7)
DR Congo*	2044	24·2 (21·3 to 27·4)	5·0 (3·4 to 7·1)	8·7 (7·7 to 9·8)	3·0 (2·0 to 4·3)	7·4 (6·2 to 9·5)	51·7 (45·7 to 58·7)	10·8 (7·2 to 15·2)	18·7 (16·5 to 21·0)	6·4 (4·3 to 9·1)	15·8 (13·2 to 20·4)
DR Congo*	2045	24·3 (21·5 to 27·6)	5·1 (3·4 to 7·2)	8·8 (7·8 to 9·9)	3·1 (2·0 to 4·4)	7·3 (6·1 to 9·6)	52·1 (45·9 to 59·0)	11·0 (7·3 to 15·5)	18·8 (16·7 to 21·1)	6·6 (4·4 to 9·4)	15·6 (12·9 to 20·5)
DR Congo*	2046	24·5 (21·6 to 27·9)	5·3 (3·5 to 7·4)	8·9 (7·8 to 9·9)	3·2 (2·1 to 4·5)	7·2 (5·9 to 9·4)	52·5 (46·1 to 59·8)	11·3 (7·4 to 15·8)	19·0 (16·8 to 21·3)	6·8 (4·5 to 9·7)	15·4 (12·6 to 20·2)
DR Congo*	2047	24·7 (21·7 to 28·1)	5·4 (3·6 to 7·7)	8·9 (7·9 to 10·0)	3·3 (2·2 to 4·7)	7·1 (5·8 to 9·2)	52·8 (46·3 to 60·1)	11·5 (7·7 to 16·6)	19·1 (16·9 to 21·5)	7·0 (4·6 to 10·1)	15·1 (12·4 to 19·7)
DR Congo*	2048	24·8 (21·7 to 28·3)	5·5 (3·6 to 7·9)	9·0 (8·0 to 10·1)	3·4 (2·2 to 4·9)	7·0 (5·7 to 9·1)	53·1 (46·5 to 60·6)	11·8 (7·7 to 16·9)	19·3 (17·0 to 21·6)	7·2 (4·7 to 10·4)	14·9 (12·1 to 19·4)
DR Congo*	2049	25·0 (21·8 to 28·7)	5·6 (3·7 to 8·1)	9·1 (8·0 to 10·2)	3·5 (2·3 to 5·0)	6·8 (5·5 to 8·9)	53·5 (46·6 to 61·3)	12·0 (7·8 to 17·4)	19·4 (17·1 to 21·8)	7·4 (4·8 to 10·8)	14·6 (11·9 to 19·0)
DR Congo*	2050	25·1 (21·7 to 28·8)	5·7 (3·7 to 8·3)	9·1 (8·1 to 10·2)	3·6 (2·3 to 5·2)	6·7 (5·4 to 8·7)	53·8 (46·4 to 61·7)	12·2 (7·9 to 17·7)	19·5 (17·3 to 21·9)	7·7 (5·0 to 11·1)	14·3 (11·5 to 18·5)
Congo (Brazzaville)*	1995	49·8 (43·3 to 56·6)	18·9 (15·4 to 22·8)	25·4 (20·3 to 31·3)	0·7 (0·5 to 1·1)	4·8 (4·8 to 4·8)	99·0 (86·2 to 112·5)	37·6 (30·7 to 45·3)	50·4 (40·3 to 62·3)	1·5 (1·0 to 2·1)	9·6 (9·6 to 9·6)
Congo (Brazzaville)*	1996	40·5 (35·1 to 46·9)	15·8 (12·9 to 19·0)	23·5 (18·7 to 29·2)	0·7 (0·5 to 1·0)	0·5 (0·5 to 0·5)	80·5 (69·8 to 93·3)	31·3 (25·7 to 37·7)	46·8 (37·2 to 58·1)	1·4 (0·9 to 2·0)	1·0 (1·0 to 1·0)

Congo (Brazzaville)*	1997	44·7 (38·8 to 51·0)	17·5 (14·3 to 21·0)	24·7 (19·9 to 30·6)	0·7 (0·5 to 1·0)	1·8 (1·8 to 1·8)	88·8 (77·2 to 101·5)	34·8 (28·4 to 41·8)	49·1 (39·6 to 60·8)	1·4 (1·0 to 2·1)	3·5 (3·5 to 3·5)
Congo (Brazzaville)*	1998	46·1 (40·3 to 52·8)	17·8 (14·6 to 21·4)	25·4 (20·6 to 31·3)	0·7 (0·5 to 1·0)	2·2 (2·2 to 2·2)	91·8 (80·1 to 105·1)	35·3 (29·1 to 42·5)	50·5 (41·0 to 62·2)	1·4 (1·0 to 2·1)	4·4 (4·4 to 4·4)
Congo (Brazzaville)*	1999	43·6 (38·2 to 50·3)	16·9 (13·8 to 20·3)	24·7 (20·0 to 30·3)	0·7 (0·5 to 1·0)	1·4 (1·4 to 1·4)	86·8 (75·9 to 100·1)	33·6 (27·5 to 40·4)	49·0 (39·8 to 60·3)	1·4 (0·9 to 2·0)	2·7 (2·7 to 2·7)
Congo (Brazzaville)*	2000	40·3 (35·1 to 46·4)	15·2 (12·5 to 18·5)	23·7 (19·2 to 29·1)	0·7 (0·5 to 0·9)	0·7 (0·7 to 0·7)	80·2 (69·9 to 92·2)	30·3 (24·8 to 36·8)	47·1 (38·1 to 57·9)	1·3 (0·9 to 1·9)	1·5 (1·5 to 1·5)
Congo (Brazzaville)*	2001	43·3 (37·6 to 49·7)	16·7 (13·8 to 20·3)	25·4 (20·7 to 31·3)	0·7 (0·5 to 1·0)	0·5 (0·5 to 0·5)	86·2 (74·8 to 98·9)	33·3 (27·4 to 40·4)	50·4 (41·2 to 62·3)	1·4 (1·0 to 2·0)	1·1 (1·1 to 1·1)
Congo (Brazzaville)*	2002	42·7 (36·9 to 49·5)	15·2 (12·5 to 18·5)	25·8 (20·9 to 31·8)	0·7 (0·5 to 1·0)	1·0 (1·0 to 1·0)	84·9 (73·5 to 98·5)	30·2 (24·9 to 36·7)	51·3 (41·6 to 63·3)	1·4 (0·9 to 1·9)	2·0 (2·0 to 2·0)
Congo (Brazzaville)*	2003	39·3 (33·6 to 45·9)	12·9 (10·7 to 15·5)	24·8 (20·0 to 31·0)	0·6 (0·4 to 0·9)	1·0 (1·0 to 1·0)	78·2 (66·8 to 91·3)	25·6 (21·3 to 30·9)	49·4 (39·7 to 61·7)	1·3 (0·8 to 1·8)	1·9 (1·9 to 1·9)
Congo (Brazzaville)*	2004	38·5 (33·1 to 44·5)	11·9 (9·9 to 14·2)	23·4 (18·8 to 29·2)	0·6 (0·4 to 0·9)	2·6 (2·6 to 2·6)	76·5 (65·9 to 88·5)	23·6 (19·8 to 28·3)	46·5 (37·3 to 58·0)	1·3 (0·8 to 1·8)	5·1 (5·1 to 5·1)
Congo (Brazzaville)*	2005	37·1 (32·2 to 42·7)	12·1 (10·1 to 14·5)	22·1 (17·9 to 27·4)	0·7 (0·4 to 0·9)	2·3 (2·3 to 2·3)	73·9 (64·0 to 85·0)	24·0 (20·1 to 28·8)	44·0 (35·6 to 54·5)	1·3 (0·9 to 1·8)	4·5 (4·5 to 4·5)
Congo (Brazzaville)*	2006	40·7 (35·5 to 46·4)	14·8 (12·4 to 17·7)	22·5 (18·2 to 27·7)	0·7 (0·5 to 1·0)	2·8 (2·8 to 2·8)	81·0 (70·6 to 92·4)	29·4 (24·6 to 35·2)	44·7 (36·3 to 55·1)	1·5 (1·0 to 2·0)	5·5 (5·5 to 5·5)
Congo (Brazzaville)*	2007	41·7 (36·5 to 47·3)	16·3 (13·8 to 19·5)	22·1 (17·7 to 27·0)	0·8 (0·5 to 1·1)	2·5 (2·5 to 2·5)	82·9 (72·7 to 94·1)	32·5 (27·4 to 38·7)	44·0 (35·3 to 53·6)	1·5 (1·1 to 2·1)	4·9 (4·9 to 4·9)
Congo (Brazzaville)*	2008	44·6 (39·5 to 50·2)	17·6 (14·9 to 20·7)	21·6 (17·5 to 26·1)	0·8 (0·6 to 1·1)	4·6 (4·6 to 4·6)	88·6 (78·6 to 99·8)	35·0 (29·6 to 41·2)	42·9 (34·9 to 52·0)	1·6 (1·1 to 2·2)	9·1 (9·1 to 9·1)
Congo (Brazzaville)*	2009	42·8 (38·4 to 48·2)	17·4 (14·8 to 20·3)	21·5 (17·7 to 25·8)	0·8 (0·6 to 1·1)	3·1 (3·1 to 3·1)	85·2 (76·4 to 95·9)	34·6 (29·5 to 40·3)	42·8 (35·2 to 51·3)	1·6 (1·1 to 2·2)	6·1 (6·1 to 6·1)
Congo (Brazzaville)*	2010	47·5 (43·2 to 52·5)	18·8 (16·4 to 21·4)	20·3 (16·9 to 24·3)	0·7 (0·5 to 1·0)	7·7 (7·7 to 7·7)	94·5 (86·0 to 104·3)	37·4 (32·6 to 42·6)	40·4 (33·6 to 48·3)	1·4 (1·0 to 1·9)	15·3 (15·3 to 15·3)
Congo (Brazzaville)*	2011	47·4 (43·6 to 51·7)	20·5 (18·3 to 23·0)	19·3 (16·2 to 22·8)	0·6 (0·4 to 0·8)	7·0 (7·0 to 7·0)	94·4 (86·6 to 102·9)	40·9 (36·4 to 45·7)	38·5 (32·1 to 45·3)	1·2 (0·8 to 1·6)	13·9 (13·9 to 13·9)
Congo (Brazzaville)*	2012	48·5 (45·0 to 52·2)	23·8 (21·6 to 26·2)	19·0 (16·2 to 21·9)	0·7 (0·5 to 1·0)	5·0 (5·0 to 5·0)	96·5 (89·5 to 103·9)	47·4 (42·9 to 52·1)	37·7 (32·2 to 43·6)	1·4 (1·0 to 1·9)	10·0 (10·0 to 10·0)

Congo (Brazzaville)*	2013	53·5 (50·0 to 57·0)	28·0 (25·7 to 30·5)	19·9 (17·1 to 22·7)	1·0 (0·7 to 1·3)	4·7 (4·7 to 4·7)	106·4 (99·5 to 113·4)	55·7 (51·1 to 60·6)	39·5 (34·0 to 45·2)	1·9 (1·4 to 2·6)	9·3 (9·3 to 9·3)
Congo (Brazzaville)*	2014	58·2 (54·6 to 61·7)	31·7 (29·4 to 34·4)	22·0 (19·3 to 24·6)	1·5 (1·1 to 2·0)	3·0 (3·0 to 3·0)	115·8 (108·6 to 122·8)	63·1 (58·5 to 68·4)	43·7 (38·3 to 48·9)	3·0 (2·2 to 4·0)	6·0 (6·0 to 6·0)
Congo (Brazzaville)*	2015	58·9 (55·4 to 62·7)	29·5 (27·1 to 32·1)	23·8 (21·2 to 26·4)	1·8 (1·3 to 2·4)	3·9 (3·9 to 3·9)	117·2 (110·2 to 124·6)	58·7 (54·0 to 63·9)	47·3 (42·1 to 52·4)	3·6 (2·7 to 4·8)	7·7 (7·7 to 7·7)
Congo (Brazzaville)*	2016	60·8 (56·8 to 64·7)	28·2 (25·6 to 31·1)	26·5 (23·7 to 29·5)	1·8 (1·3 to 2·4)	4·3 (4·3 to 4·3)	120·9 (112·9 to 128·7)	56·2 (50·9 to 61·8)	52·7 (47·1 to 58·6)	3·5 (2·6 to 4·7)	8·6 (8·6 to 8·6)
Congo (Brazzaville)*	2017	53·8 (49·8 to 57·9)	21·6 (19·4 to 24·2)	25·0 (22·2 to 28·2)	1·6 (1·2 to 2·2)	5·5 (5·5 to 5·5)	107·1 (99·1 to 115·1)	43·1 (38·6 to 48·1)	49·8 (44·2 to 56·0)	3·2 (2·3 to 4·4)	11·0 (11·0 to 11·0)
Congo (Brazzaville)*	2018	45·7 (42·2 to 49·5)	16·6 (14·6 to 18·9)	22·2 (19·4 to 25·4)	1·3 (0·9 to 1·8)	5·6 (5·6 to 5·6)	90·8 (83·9 to 98·5)	33·0 (28·9 to 37·6)	44·2 (38·7 to 50·5)	2·6 (1·8 to 3·7)	11·1 (11·1 to 11·1)
Congo (Brazzaville)*	2019	47·0 (43·2 to 51·2)	17·7 (15·1 to 21·0)	21·4 (18·7 to 24·5)	1·3 (0·9 to 1·8)	6·5 (6·5 to 6·6)	93·4 (86·0 to 101·9)	35·3 (30·0 to 41·7)	42·6 (37·3 to 48·8)	2·6 (1·8 to 3·6)	13·0 (12·9 to 13·1)
Congo (Brazzaville)*	2020	49·7 (45·4 to 54·5)	19·4 (16·1 to 23·6)	20·5 (17·9 to 23·5)	1·3 (0·9 to 1·8)	8·4 (8·3 to 8·5)	98·8 (90·3 to 108·4)	38·7 (31·9 to 47·0)	40·8 (35·7 to 46·7)	2·5 (1·8 to 3·5)	16·8 (16·6 to 16·9)
Congo (Brazzaville)*	2021	48·2 (43·9 to 52·9)	19·0 (15·7 to 23·0)	20·0 (17·5 to 22·9)	1·3 (0·9 to 1·8)	8·0 (7·8 to 8·2)	95·8 (87·4 to 105·3)	37·7 (31·1 to 45·8)	39·7 (34·7 to 45·5)	2·5 (1·8 to 3·5)	15·9 (15·5 to 16·3)
Congo (Brazzaville)*	2022	45·2 (40·7 to 50·2)	17·5 (13·6 to 22·0)	20·2 (17·7 to 23·1)	1·3 (0·9 to 1·8)	6·1 (5·8 to 6·5)	89·8 (80·9 to 99·9)	34·9 (27·1 to 43·7)	40·2 (35·1 to 46·0)	2·6 (1·8 to 3·6)	12·2 (11·5 to 12·9)
Congo (Brazzaville)*	2023	46·0 (40·9 to 51·5)	18·0 (13·6 to 23·1)	20·5 (17·9 to 23·4)	1·4 (1·0 to 1·9)	6·2 (5·8 to 6·6)	91·4 (81·4 to 102·4)	35·7 (27·0 to 46·0)	40·7 (35·6 to 46·6)	2·7 (1·9 to 3·8)	12·3 (11·5 to 13·1)
Congo (Brazzaville)*	2024	46·7 (41·5 to 52·6)	18·3 (13·8 to 23·9)	20·8 (18·2 to 23·8)	1·4 (1·0 to 2·0)	6·2 (5·8 to 6·6)	92·9 (82·5 to 104·6)	36·4 (27·5 to 47·5)	41·3 (36·2 to 47·3)	2·8 (2·0 to 3·9)	12·3 (11·5 to 13·2)
Congo (Brazzaville)*	2025	47·5 (41·9 to 53·7)	18·7 (13·8 to 24·8)	21·1 (18·5 to 24·2)	1·5 (1·0 to 2·1)	6·2 (5·8 to 6·6)	94·5 (83·4 to 106·9)	37·2 (27·5 to 49·2)	42·0 (36·8 to 48·1)	2·9 (2·1 to 4·1)	12·3 (11·4 to 13·2)
Congo (Brazzaville)*	2026	48·3 (42·3 to 54·9)	19·1 (14·1 to 25·5)	21·5 (18·8 to 24·6)	1·5 (1·1 to 2·2)	6·2 (5·7 to 6·6)	96·1 (84·2 to 109·3)	38·0 (27·9 to 50·7)	42·8 (37·4 to 49·0)	3·1 (2·2 to 4·3)	12·2 (11·3 to 13·2)
Congo (Brazzaville)*	2027	49·2 (43·1 to 56·0)	19·6 (14·2 to 26·1)	21·9 (19·1 to 25·1)	1·6 (1·1 to 2·2)	6·1 (5·6 to 6·6)	97·9 (85·7 to 111·5)	39·0 (28·3 to 51·9)	43·5 (38·1 to 49·9)	3·2 (2·3 to 4·5)	12·1 (11·2 to 13·2)

Congo (Brazzaville)*	2028	50·1 (43·7 to 57·6)	20·1 (14·3 to 27·2)	22·3 (19·5 to 25·5)	1·7 (1·2 to 2·3)	6·1 (5·6 to 6·6)	99·6 (86·8 to 114·5)	39·9 (28·5 to 54·2)	44·3 (38·8 to 50·7)	3·3 (2·4 to 4·7)	12·1 (11·1 to 13·2)
Congo (Brazzaville)*	2029	51·0 (44·0 to 59·0)	20·5 (14·5 to 28·5)	22·7 (19·9 to 26·0)	1·7 (1·2 to 2·5)	6·0 (5·5 to 6·6)	101·4 (87·6 to 117·3)	40·8 (28·8 to 56·7)	45·2 (39·5 to 51·8)	3·5 (2·4 to 4·9)	11·9 (10·9 to 13·1)
Congo (Brazzaville)*	2030	51·9 (44·8 to 60·3)	21·0 (14·7 to 28·9)	23·1 (20·2 to 26·5)	1·8 (1·3 to 2·5)	6·0 (5·4 to 6·6)	103·3 (89·2 to 120·0)	41·8 (29·2 to 57·5)	46·0 (40·2 to 52·7)	3·6 (2·5 to 5·1)	11·8 (10·8 to 13·1)
Congo (Brazzaville)*	2031	52·9 (45·4 to 62·1)	21·6 (14·7 to 30·3)	23·5 (20·6 to 27·0)	1·9 (1·3 to 2·6)	5·9 (5·3 to 6·5)	105·2 (90·2 to 123·6)	42·9 (29·3 to 60·2)	46·8 (40·9 to 53·7)	3·8 (2·6 to 5·3)	11·8 (10·6 to 13·0)
Congo (Brazzaville)*	2032	53·9 (46·2 to 63·4)	22·1 (15·1 to 31·4)	23·9 (20·9 to 27·5)	2·0 (1·4 to 2·8)	5·9 (5·3 to 6·5)	107·1 (91·9 to 126·1)	43·9 (30·1 to 62·5)	47·6 (41·6 to 54·6)	3·9 (2·7 to 5·5)	11·7 (10·6 to 13·0)
Congo (Brazzaville)*	2033	54·7 (47·0 to 64·8)	22·5 (15·3 to 32·6)	24·3 (21·2 to 27·9)	2·0 (1·4 to 2·9)	5·9 (5·3 to 6·6)	108·8 (93·5 to 128·9)	44·7 (30·5 to 64·8)	48·3 (42·2 to 55·4)	4·0 (2·8 to 5·7)	11·8 (10·6 to 13·1)
Congo (Brazzaville)*	2034	55·7 (47·7 to 66·3)	22·9 (15·3 to 33·3)	24·7 (21·6 to 28·3)	2·1 (1·5 to 3·0)	5·9 (5·4 to 6·6)	110·7 (94·8 to 131·8)	45·6 (30·4 to 66·2)	49·1 (42·9 to 56·3)	4·2 (2·9 to 5·9)	11·8 (10·7 to 13·2)
Congo (Brazzaville)*	2035	56·5 (48·2 to 68·0)	23·3 (15·3 to 34·6)	25·1 (21·9 to 28·7)	2·2 (1·5 to 3·1)	6·0 (5·4 to 6·7)	112·4 (95·8 to 135·3)	46·3 (30·4 to 68·8)	49·8 (43·5 to 57·1)	4·3 (3·0 to 6·1)	11·9 (10·7 to 13·4)
Congo (Brazzaville)*	2036	57·5 (49·1 to 69·3)	23·7 (15·4 to 35·3)	25·4 (22·1 to 29·0)	2·3 (1·6 to 3·2)	6·1 (5·4 to 6·9)	114·3 (97·7 to 137·8)	47·2 (30·7 to 70·2)	50·5 (44·0 to 57·8)	4·5 (3·1 to 6·3)	12·1 (10·8 to 13·6)
Congo (Brazzaville)*	2037	58·3 (49·6 to 71·1)	24·1 (15·5 to 36·3)	25·7 (22·4 to 29·4)	2·3 (1·6 to 3·3)	6·2 (5·5 to 6·9)	116·0 (98·6 to 141·4)	48·0 (30·9 to 72·2)	51·1 (44·6 to 58·4)	4·6 (3·2 to 6·6)	12·3 (10·9 to 13·8)
Congo (Brazzaville)*	2038	59·2 (50·0 to 72·1)	24·5 (15·8 to 36·9)	26·0 (22·7 to 29·8)	2·4 (1·7 to 3·4)	6·3 (5·6 to 7·1)	117·8 (99·5 to 143·5)	48·7 (31·4 to 73·4)	51·7 (45·1 to 59·3)	4·8 (3·3 to 6·8)	12·5 (11·1 to 14·1)
Congo (Brazzaville)*	2039	60·1 (50·4 to 73·2)	24·8 (15·8 to 37·7)	26·3 (22·9 to 30·1)	2·5 (1·7 to 3·5)	6·4 (5·7 to 7·3)	119·5 (100·3 to 145·6)	49·4 (31·5 to 75·0)	52·3 (45·6 to 60·0)	5·0 (3·4 to 7·0)	12·8 (11·4 to 14·5)
Congo (Brazzaville)*	2040	60·9 (51·2 to 73·6)	25·2 (15·8 to 38·1)	26·6 (23·2 to 30·5)	2·6 (1·7 to 3·6)	6·6 (5·9 to 7·6)	121·2 (101·7 to 146·3)	50·1 (31·4 to 75·8)	52·8 (46·1 to 60·6)	5·1 (3·5 to 7·2)	13·2 (11·6 to 15·1)
Congo (Brazzaville)*	2041	61·8 (51·6 to 75·2)	25·5 (16·0 to 38·6)	26·8 (23·4 to 30·7)	2·6 (1·8 to 3·8)	6·8 (6·0 to 7·8)	122·9 (102·7 to 149·6)	50·8 (31·9 to 76·8)	53·3 (46·5 to 61·1)	5·3 (3·6 to 7·5)	13·6 (12·0 to 15·6)

Congo (Brazzaville)*	2042	62·6 (52·4 to 75·9)	25·8 (16·2 to 39·2)	27·0 (23·5 to 31·0)	2·7 (1·8 to 3·9)	7·1 (6·2 to 8·2)	124·6 (104·2 to 150·9)	51·4 (32·1 to 77·9)	53·6 (46·8 to 61·6)	5·4 (3·7 to 7·7)	14·2 (12·4 to 16·3)
Congo (Brazzaville)*	2043	63·4 (52·8 to 77·5)	26·1 (16·4 to 40·1)	27·2 (23·7 to 31·2)	2·8 (1·9 to 4·0)	7·4 (6·5 to 8·6)	126·2 (104·9 to 154·2)	51·9 (32·6 to 79·8)	54·0 (47·1 to 62·0)	5·6 (3·7 to 8·0)	14·7 (12·8 to 17·1)
Congo (Brazzaville)*	2044	64·3 (53·3 to 78·5)	26·3 (16·4 to 40·3)	27·3 (23·8 to 31·4)	2·9 (1·9 to 4·1)	7·7 (6·7 to 9·1)	127·8 (106·0 to 156·1)	52·4 (32·7 to 80·2)	54·3 (47·4 to 62·4)	5·7 (3·8 to 8·2)	15·4 (13·4 to 18·1)
Congo (Brazzaville)*	2045	65·1 (53·7 to 80·3)	26·6 (16·3 to 42·2)	27·4 (23·9 to 31·5)	3·0 (2·0 to 4·3)	8·1 (7·0 to 9·6)	129·5 (106·9 to 159·7)	52·9 (32·5 to 83·8)	54·6 (47·6 to 62·7)	5·9 (3·9 to 8·5)	16·1 (13·9 to 19·1)
Congo (Brazzaville)*	2046	66·0 (54·5 to 81·2)	26·9 (16·3 to 41·9)	27·6 (24·1 to 31·7)	3·0 (2·0 to 4·4)	8·5 (7·3 to 10·1)	131·2 (108·5 to 161·6)	53·4 (32·5 to 83·3)	54·9 (47·9 to 63·1)	6·0 (4·0 to 8·7)	16·8 (14·5 to 20·0)
Congo (Brazzaville)*	2047	66·8 (55·3 to 82·0)	27·1 (16·5 to 42·8)	27·7 (24·2 to 31·9)	3·1 (2·1 to 4·5)	8·9 (7·5 to 10·7)	132·9 (109·9 to 163·0)	53·9 (32·7 to 85·1)	55·1 (48·0 to 63·4)	6·2 (4·1 to 9·0)	17·7 (15·0 to 21·3)
Congo (Brazzaville)*	2048	67·7 (55·9 to 83·5)	27·4 (16·6 to 43·2)	27·8 (24·3 to 32·0)	3·2 (2·1 to 4·7)	9·3 (7·9 to 11·3)	134·7 (111·2 to 166·1)	54·4 (33·0 to 85·9)	55·3 (48·3 to 63·7)	6·4 (4·2 to 9·3)	18·6 (15·8 to 22·4)
Congo (Brazzaville)*	2049	68·7 (56·8 to 84·9)	27·7 (16·4 to 44·7)	28·0 (24·4 to 32·2)	3·3 (2·2 to 4·8)	9·8 (8·2 to 11·9)	136·7 (113·0 to 169·0)	55·1 (32·7 to 89·0)	55·7 (48·6 to 64·1)	6·5 (4·3 to 9·6)	19·4 (16·3 to 23·7)
Congo (Brazzaville)*	2050	69·8 (57·7 to 86·4)	28·0 (16·7 to 45·1)	28·1 (24·5 to 32·4)	3·4 (2·2 to 5·0)	10·2 (8·6 to 12·6)	138·7 (114·7 to 171·9)	55·7 (33·3 to 89·7)	55·9 (48·8 to 64·4)	6·7 (4·4 to 9·9)	20·4 (17·0 to 25·0)
Cook Islands	1995	369·8 (317·6 to 433·0)	325·7 (275·9 to 388·3)	40·3 (31·7 to 50·2)	0·1 (0·0 to 0·1)	3·8 (3·8 to 3·8)	516·6 (443·6 to 604·8)	454·8 (385·3 to 542·4)	56·2 (44·3 to 70·1)	0·1 (0·0 to 0·2)	5·3 (5·3 to 5·3)
Cook Islands	1996	383·3 (331·6 to 447·2)	340·5 (289·7 to 402·3)	42·7 (33·9 to 53·3)	0·1 (0·0 to 0·1)	0·0 (0·0 to 0·0)	535·4 (463·1 to 624·7)	475·6 (404·6 to 561·9)	59·7 (47·3 to 74·5)	0·1 (0·0 to 0·2)	0·0 (0·0 to 0·0)
Cook Islands	1997	407·9 (355·4 to 465·7)	360·9 (310·5 to 417·8)	45·8 (36·3 to 56·7)	0·1 (0·0 to 0·1)	1·2 (1·2 to 1·2)	569·8 (496·4 to 650·5)	504·0 (433·7 to 583·6)	64·0 (50·7 to 79·2)	0·1 (0·0 to 0·2)	1·6 (1·6 to 1·6)
Cook Islands	1998	435·2 (385·6 to 491·6)	385·3 (337·5 to 442·0)	49·0 (39·1 to 60·7)	0·1 (0·0 to 0·1)	0·8 (0·8 to 0·8)	607·9 (538·5 to 686·6)	538·2 (471·4 to 617·3)	68·4 (54·7 to 84·7)	0·1 (0·0 to 0·2)	1·2 (1·2 to 1·2)
Cook Islands	1999	589·9 (547·0 to 638·9)	408·1 (365·0 to 456·3)	51·8 (42·2 to 62·9)	0·1 (0·0 to 0·1)	130·0 (130·0 to 130·0)	823·9 (764·0 to 892·4)	569·9 (509·7 to 637·3)	72·3 (58·9 to 87·9)	0·1 (0·0 to 0·2)	181·5 (181·5 to 181·5)

Cook Islands	2000	518·7 (478·9 to 563·9)	450·9 (413·7 to 492·5)	55·6 (45·2 to 67·6)	0·1 (0·0 to 0·1)	12·0 (12·0 to 12·0)	724·4 (668·9 to 787·6)	629·8 (577·8 to 687·8)	77·7 (63·1 to 94·4)	0·1 (0·0 to 0·2)	16·7 (16·7 to 16·7)
Cook Islands	2001	596·4 (557·1 to 637·4)	527·8 (491·7 to 566·3)	62·5 (51·2 to 75·0)	0·1 (0·0 to 0·1)	6·0 (6·0 to 6·0)	832·9 (778·1 to 890·3)	737·2 (686·7 to 790·9)	87·3 (71·5 to 104·8)	0·1 (0·0 to 0·2)	8·3 (8·3 to 8·3)
Cook Islands	2002	629·4 (594·3 to 668·6)	543·3 (509·8 to 581·8)	65·9 (55·1 to 78·5)	0·1 (0·0 to 0·1)	20·1 (20·1 to 20·1)	879·2 (830·0 to 933·9)	758·9 (712·1 to 812·6)	92·1 (77·0 to 109·6)	0·1 (0·0 to 0·2)	28·1 (28·1 to 28·1)
Cook Islands	2003	745·3 (711·2 to 783·4)	544·9 (511·8 to 581·4)	66·3 (56·0 to 78·0)	0·1 (0·0 to 0·1)	134·1 (134·1 to 134·1)	1041·0 (993·4 to 1094·2)	761·1 (714·9 to 812·0)	92·6 (78·2 to 109·0)	0·1 (0·0 to 0·2)	187·2 (187·2 to 187·2)
Cook Islands	2004	689·8 (656·5 to 726·7)	537·3 (504·0 to 572·8)	65·6 (56·0 to 76·7)	0·1 (0·0 to 0·1)	86·8 (86·8 to 86·8)	963·5 (917·0 to 1015·0)	750·5 (704·0 to 800·1)	91·6 (78·2 to 107·1)	0·1 (0·0 to 0·2)	121·3 (121·3 to 121·3)
Cook Islands	2005	633·2 (600·5 to 667·9)	520·2 (488·7 to 553·0)	63·1 (54·3 to 73·1)	0·1 (0·0 to 0·1)	49·8 (49·8 to 49·8)	884·4 (838·7 to 932·9)	726·6 (682·6 to 772·4)	88·2 (75·9 to 102·1)	0·1 (0·0 to 0·2)	69·5 (69·5 to 69·5)
Cook Islands	2006	725·1 (691·7 to 762·3)	561·6 (527·9 to 597·3)	62·9 (54·3 to 72·4)	0·0 (0·0 to 0·1)	100·5 (100·5 to 100·5)	1012·8 (966·2 to 1064·8)	784·4 (737·3 to 834·3)	87·9 (75·8 to 101·1)	0·1 (0·0 to 0·2)	140·4 (140·4 to 140·4)
Cook Islands	2007	648·6 (614·6 to 683·3)	537·0 (506·1 to 569·1)	59·8 (51·5 to 69·0)	0·0 (0·0 to 0·2)	51·8 (51·8 to 51·8)	905·9 (858·5 to 954·4)	750·0 (706·9 to 794·8)	83·5 (71·9 to 96·4)	0·1 (0·0 to 0·2)	72·3 (72·3 to 72·3)
Cook Islands	2008	648·8 (617·7 to 681·2)	547·2 (515·5 to 578·8)	58·1 (49·8 to 67·1)	0·0 (0·0 to 0·1)	43·4 (43·4 to 43·4)	906·2 (862·8 to 951·5)	764·3 (720·0 to 808·4)	81·2 (69·6 to 93·7)	0·0 (0·0 to 0·2)	60·6 (60·6 to 60·6)
Cook Islands	2009	702·6 (664·4 to 741·7)	600·4 (564·6 to 638·6)	58·2 (49·4 to 67·3)	0·0 (0·0 to 0·2)	44·1 (44·1 to 44·1)	981·4 (927·9 to 1036·0)	838·6 (788·6 to 892·0)	81·3 (69·0 to 94·0)	0·0 (0·0 to 0·2)	61·6 (61·6 to 61·6)
Cook Islands	2010	686·7 (650·2 to 725·3)	584·6 (550·0 to 622·1)	56·2 (48·1 to 64·4)	0·0 (0·0 to 0·1)	45·8 (45·8 to 45·8)	959·1 (908·1 to 1013·0)	816·5 (768·2 to 868·8)	78·5 (67·2 to 90·0)	0·0 (0·0 to 0·2)	64·0 (64·0 to 64·0)
Cook Islands	2011	702·2 (665·3 to 741·6)	613·9 (577·5 to 653·3)	56·7 (48·2 to 65·3)	0·0 (0·0 to 0·0)	31·6 (31·6 to 31·6)	980·8 (929·3 to 1035·8)	857·4 (806·7 to 912·4)	79·2 (67·3 to 91·2)	0·0 (0·0 to 0·0)	44·2 (44·2 to 44·2)
Cook Islands	2012	668·0 (632·0 to 704·0)	555·5 (518·9 to 590·0)	54·4 (46·5 to 63·0)	0·0 (0·0 to 0·0)	58·2 (58·2 to 58·2)	933·1 (882·7 to 983·3)	775·8 (724·7 to 824·1)	76·0 (64·9 to 88·0)	0·0 (0·0 to 0·0)	81·2 (81·2 to 81·2)
Cook Islands	2013	647·9 (613·7 to 682·2)	546·8 (512·0 to 581·3)	53·7 (46·0 to 62·4)	0·0 (0·0 to 0·0)	47·4 (47·4 to 47·4)	904·9 (857·2 to 952·8)	763·7 (715·1 to 811·9)	75·1 (64·3 to 87·2)	0·0 (0·0 to 0·0)	66·1 (66·1 to 66·1)

Cook Islands	2014	693·1 (657·5 to 730·5)	601·1 (566·1 to 637·2)	54·9 (46·6 to 63·6)	0·0 (0·0 to 0·0)	37·1 (37·1 to 37·1)	968·0 (918·3 to 1020·3)	839·5 (790·7 to 890·0)	76·7 (65·2 to 88·9)	0·0 (0·0 to 0·0)	51·8 (51·8 to 51·8)
Cook Islands	2015	702·1 (664·0 to 740·1)	630·5 (594·5 to 667·6)	55·3 (47·1 to 64·7)	0·0 (0·0 to 0·0)	16·2 (16·2 to 16·2)	980·6 (927·4 to 1033·7)	880·6 (830·4 to 932·4)	77·3 (65·8 to 90·4)	0·0 (0·0 to 0·0)	22·7 (22·7 to 22·7)
Cook Islands	2016	690·6 (652·6 to 726·9)	624·4 (587·3 to 659·7)	54·6 (46·5 to 64·8)	0·0 (0·0 to 0·0)	11·6 (11·6 to 11·6)	964·6 (911·5 to 1015·3)	872·1 (820·3 to 921·5)	76·2 (65·0 to 90·5)	0·0 (0·0 to 0·0)	16·3 (16·3 to 16·3)
Cook Islands	2017	710·0 (669·8 to 752·2)	645·2 (604·8 to 684·9)	55·3 (46·3 to 65·8)	0·0 (0·0 to 0·0)	9·5 (9·5 to 9·5)	991·6 (935·5 to 1050·6)	901·1 (844·7 to 956·6)	77·2 (64·7 to 91·8)	0·0 (0·0 to 0·0)	13·3 (13·3 to 13·3)
Cook Islands	2018	734·4 (683·9 to 788·2)	660·0 (612·7 to 711·9)	58·0 (47·9 to 69·6)	0·8 (0·6 to 1·0)	15·6 (15·6 to 15·6)	1025·8 (955·2 to 1100·9)	921·9 (855·7 to 994·4)	81·0 (66·9 to 97·2)	1·1 (0·8 to 1·5)	21·8 (21·8 to 21·8)
Cook Islands	2019	756·1 (692·2 to 824·8)	694·8 (634·8 to 761·6)	60·5 (49·7 to 72·5)	0·8 (0·6 to 1·1)	0·0 (0·0 to 0·0)	1056·0 (966·8 to 1152·0)	970·4 (886·7 to 1063·8)	84·4 (69·4 to 101·3)	1·2 (0·8 to 1·6)	0·0 (0·0 to 0·0)
Cook Islands	2020	800·2 (723·9 to 885·2)	739·8 (666·6 to 824·3)	59·6 (48·9 to 71·5)	0·9 (0·6 to 1·2)	0·0 (0·0 to 0·0)	1117·7 (1011·1 to 1236·3)	1033·3 (931·0 to 1151·3)	83·2 (68·3 to 99·8)	1·2 (0·9 to 1·6)	0·0 (0·0 to 0·0)
Cook Islands	2021	789·1 (713·8 to 872·8)	729·7 (657·4 to 813·0)	58·6 (48·1 to 70·3)	0·9 (0·6 to 1·2)	0·0 (0·0 to 0·0)	1102·2 (997·0 to 1219·1)	1019·1 (918·2 to 1135·5)	81·8 (67·2 to 98·1)	1·2 (0·9 to 1·6)	0·0 (0·0 to 0·0)
Cook Islands	2022	755·1 (662·7 to 853·3)	694·9 (603·6 to 795·5)	59·3 (48·7 to 71·0)	0·9 (0·6 to 1·2)	0·0 (0·0 to 0·0)	1054·6 (925·6 to 1191·8)	970·6 (843·1 to 1111·0)	82·8 (68·0 to 99·2)	1·2 (0·9 to 1·7)	0·0 (0·0 to 0·0)
Cook Islands	2023	773·1 (670·2 to 887·2)	712·1 (608·3 to 826·1)	60·1 (49·3 to 72·0)	0·9 (0·7 to 1·3)	0·0 (0·0 to 0·0)	1079·8 (936·0 to 1239·1)	994·6 (849·7 to 1153·8)	83·9 (68·9 to 100·5)	1·3 (0·9 to 1·8)	0·0 (0·0 to 0·0)
Cook Islands	2024	790·3 (680·5 to 922·2)	728·5 (615·9 to 856·5)	60·8 (49·9 to 72·9)	1·0 (0·7 to 1·3)	0·0 (0·0 to 0·0)	1103·9 (950·4 to 1288·0)	1017·6 (860·2 to 1196·3)	85·0 (69·7 to 101·9)	1·4 (1·0 to 1·8)	0·0 (0·0 to 0·0)
Cook Islands	2025	806·8 (694·4 to 943·4)	744·2 (633·7 to 885·2)	61·6 (50·5 to 73·9)	1·0 (0·7 to 1·4)	0·0 (0·0 to 0·0)	1126·9 (969·9 to 1317·6)	1039·4 (885·1 to 1236·4)	86·1 (70·6 to 103·2)	1·4 (1·0 to 1·9)	0·0 (0·0 to 0·0)
Cook Islands	2026	819·3 (699·3 to 968·2)	756·1 (633·6 to 904·4)	62·2 (51·0 to 74·6)	1·0 (0·8 to 1·4)	0·0 (0·0 to 0·0)	1144·3 (976·7 to 1352·4)	1056·0 (885·0 to 1263·2)	86·8 (71·2 to 104·2)	1·5 (1·1 to 2·0)	0·0 (0·0 to 0·0)
Cook Islands	2027	833·5 (705·8 to 999·8)	769·7 (642·1 to 933·8)	62·7 (51·5 to 75·3)	1·1 (0·8 to 1·4)	0·0 (0·0 to 0·0)	1164·1 (985·9 to 1396·4)	1075·1 (896·8 to 1304·2)	87·5 (71·9 to 105·1)	1·5 (1·1 to 2·0)	0·0 (0·0 to 0·0)

Cook Islands	2028	847·7 (705·3 to 1015·6)	783·3 (638·7 to 948·1)	63·2 (51·9 to 75·9)	1·1 (0·8 to 1·5)	0·0 (0·0 to 0·0)	1183·9 (985·1 to 1418·5)	1094·0 (892·1 to 1324·3)	88·3 (72·5 to 106·0)	1·6 (1·1 to 2·1)	0·0 (0·0 to 0·0)
Cook Islands	2029	862·3 (704·8 to 1041·8)	797·3 (640·7 to 975·3)	63·8 (52·4 to 76·6)	1·2 (0·8 to 1·6)	0·0 (0·0 to 0·0)	1204·4 (984·4 to 1455·0)	1113·6 (894·9 to 1362·2)	89·1 (73·1 to 107·0)	1·6 (1·2 to 2·2)	0·0 (0·0 to 0·0)
Cook Islands	2030	876·4 (710·6 to 1069·7)	810·8 (648·7 to 1002·6)	64·4 (52·8 to 77·3)	1·2 (0·9 to 1·6)	0·0 (0·0 to 0·0)	1224·1 (992·5 to 1494·1)	1132·5 (906·1 to 1400·3)	89·9 (73·7 to 108·0)	1·7 (1·2 to 2·3)	0·0 (0·0 to 0·0)
Cook Islands	2031	893·1 (724·4 to 1095·5)	826·8 (656·3 to 1026·3)	65·1 (53·3 to 78·2)	1·2 (0·9 to 1·7)	0·0 (0·0 to 0·0)	1247·5 (1011·7 to 1530·1)	1154·8 (916·6 to 1433·5)	90·9 (74·5 to 109·3)	1·7 (1·3 to 2·3)	0·0 (0·0 to 0·0)
Cook Islands	2032	911·4 (730·5 to 1128·8)	844·2 (664·9 to 1059·1)	65·9 (54·0 to 79·2)	1·3 (0·9 to 1·8)	0·0 (0·0 to 0·0)	1272·9 (1020·3 to 1576·7)	1179·1 (928·7 to 1479·3)	92·1 (75·5 to 110·6)	1·8 (1·3 to 2·4)	0·0 (0·0 to 0·0)
Cook Islands	2033	930·2 (742·9 to 1159·9)	862·1 (674·2 to 1091·0)	66·7 (54·7 to 80·1)	1·4 (1·0 to 1·8)	0·0 (0·0 to 0·0)	1299·2 (1037·6 to 1620·1)	1204·2 (941·7 to 1523·9)	93·1 (76·4 to 111·8)	1·9 (1·4 to 2·6)	0·0 (0·0 to 0·0)
Cook Islands	2034	948·9 (746·5 to 1201·8)	880·0 (676·6 to 1132·8)	67·5 (55·3 to 81·0)	1·4 (1·0 to 1·9)	0·0 (0·0 to 0·0)	1325·3 (1042·7 to 1678·6)	1229·1 (945·0 to 1582·2)	94·2 (77·3 to 113·1)	2·0 (1·4 to 2·7)	0·0 (0·0 to 0·0)
Cook Islands	2035	969·2 (760·4 to 1238·4)	899·4 (689·9 to 1168·4)	68·3 (56·0 to 82·0)	1·5 (1·1 to 2·0)	0·0 (0·0 to 0·0)	1353·6 (1062·0 to 1729·7)	1256·2 (963·6 to 1631·9)	95·4 (78·3 to 114·5)	2·1 (1·5 to 2·8)	0·0 (0·0 to 0·0)
Cook Islands	2036	990·2 (765·4 to 1271·4)	919·5 (698·2 to 1192·2)	69·1 (56·7 to 83·0)	1·5 (1·1 to 2·1)	0·0 (0·0 to 0·0)	1383·0 (1069·0 to 1775·8)	1284·3 (975·1 to 1665·2)	96·6 (79·2 to 115·9)	2·1 (1·5 to 2·9)	0·0 (0·0 to 0·0)
Cook Islands	2037	1010·8 (777·2 to 1307·9)	939·2 (711·5 to 1239·1)	70·0 (57·4 to 83·9)	1·6 (1·2 to 2·2)	0·0 (0·0 to 0·0)	1411·8 (1085·5 to 1826·8)	1311·8 (993·8 to 1730·6)	97·7 (80·2 to 117·2)	2·2 (1·6 to 3·0)	0·0 (0·0 to 0·0)
Cook Islands	2038	1031·1 (784·5 to 1333·7)	958·6 (714·8 to 1259·7)	70·8 (58·1 to 84·9)	1·7 (1·2 to 2·3)	0·0 (0·0 to 0·0)	1440·1 (1095·7 to 1862·9)	1338·8 (998·3 to 1759·5)	98·9 (81·2 to 118·6)	2·3 (1·7 to 3·2)	0·0 (0·0 to 0·0)
Cook Islands	2039	1051·3 (797·5 to 1359·5)	977·9 (723·4 to 1285·2)	71·6 (58·8 to 85·9)	1·7 (1·3 to 2·4)	0·0 (0·0 to 0·0)	1468·4 (1113·9 to 1898·9)	1365·9 (1010·4 to 1795·0)	100·0 (82·1 to 120·0)	2·4 (1·8 to 3·3)	0·0 (0·0 to 0·0)
Cook Islands	2040	1069·0 (804·6 to 1402·1)	994·8 (734·0 to 1335·8)	72·4 (59·4 to 86·8)	1·8 (1·3 to 2·5)	0·0 (0·0 to 0·0)	1493·1 (1123·8 to 1958·4)	1389·5 (1025·2 to 1865·8)	101·1 (83·0 to 121·2)	2·5 (1·8 to 3·4)	0·0 (0·0 to 0·0)
Cook Islands	2041	1090·3 (823·0 to 1444·5)	1015·2 (749·4 to 1364·1)	73·2 (60·1 to 87·8)	1·9 (1·4 to 2·6)	0·0 (0·0 to 0·0)	1522·9 (1149·4 to 2017·6)	1418·0 (1046·7 to 1905·3)	102·3 (84·0 to 122·7)	2·6 (1·9 to 3·6)	0·0 (0·0 to 0·0)

Cook Islands	2042	1111·4 (825·7 to 1489·9)	1035·4 (752·8 to 1413·4)	74·1 (60·9 to 88·8)	2·0 (1·4 to 2·7)	0·0 (0·0 to 0·0)	1552·4 (1153·2 to 2080·9)	1446·1 (1051·4 to 1974·1)	103·5 (85·0 to 124·1)	2·7 (2·0 to 3·7)	0·0 (0·0 to 0·0)
Cook Islands	2043	1129·5 (834·7 to 1519·9)	1052·6 (761·3 to 1433·5)	74·9 (61·5 to 89·8)	2·0 (1·4 to 2·8)	0·0 (0·0 to 0·0)	1577·6 (1165·8 to 2122·8)	1470·2 (1063·3 to 2002·2)	104·6 (85·9 to 125·4)	2·8 (2·0 to 3·9)	0·0 (0·0 to 0·0)
Cook Islands	2044	1153·8 (845·0 to 1547·0)	1075·9 (771·2 to 1464·3)	75·8 (62·3 to 90·9)	2·1 (1·5 to 2·9)	0·0 (0·0 to 0·0)	1611·6 (1180·2 to 2160·7)	1502·7 (1077·2 to 2045·2)	105·9 (87·0 to 126·9)	3·0 (2·1 to 4·1)	0·0 (0·0 to 0·0)
Cook Islands	2045	1175·7 (852·6 to 1567·4)	1096·7 (774·3 to 1487·9)	76·7 (63·0 to 91·9)	2·2 (1·6 to 3·0)	0·0 (0·0 to 0·0)	1642·1 (1190·8 to 2189·3)	1531·8 (1081·5 to 2078·2)	107·1 (88·0 to 128·4)	3·1 (2·2 to 4·3)	0·0 (0·0 to 0·0)
Cook Islands	2046	1197·4 (863·2 to 1608·9)	1117·5 (787·6 to 1530·2)	77·6 (63·8 to 93·0)	2·3 (1·6 to 3·2)	0·0 (0·0 to 0·0)	1672·4 (1205·7 to 2247·2)	1560·8 (1100·0 to 2137·2)	108·4 (89·1 to 129·9)	3·2 (2·2 to 4·4)	0·0 (0·0 to 0·0)
Cook Islands	2047	1221·1 (868·9 to 1643·1)	1140·2 (794·9 to 1558·1)	78·5 (64·5 to 94·0)	2·4 (1·7 to 3·3)	0·0 (0·0 to 0·0)	1705·5 (1213·6 to 2294·9)	1592·5 (1110·3 to 2176·2)	109·6 (90·0 to 131·3)	3·3 (2·3 to 4·6)	0·0 (0·0 to 0·0)
Cook Islands	2048	1242·5 (881·4 to 1682·1)	1160·7 (807·7 to 1592·9)	79·4 (65·2 to 95·1)	2·5 (1·7 to 3·5)	0·0 (0·0 to 0·0)	1735·5 (1231·1 to 2349·5)	1621·1 (1128·1 to 2224·9)	110·9 (91·1 to 132·8)	3·5 (2·4 to 4·8)	0·0 (0·0 to 0·0)
Cook Islands	2049	1264·4 (898·0 to 1731·9)	1181·5 (816·6 to 1644·6)	80·3 (65·9 to 96·2)	2·6 (1·8 to 3·6)	0·0 (0·0 to 0·0)	1765·9 (1254·2 to 2419·0)	1650·2 (1140·5 to 2297·1)	112·1 (92·1 to 134·3)	3·6 (2·5 to 5·0)	0·0 (0·0 to 0·0)
Cook Islands	2050	1286·8 (917·8 to 1772·5)	1203·0 (835·3 to 1687·3)	81·2 (66·6 to 97·2)	2·7 (1·9 to 3·8)	0·0 (0·0 to 0·0)	1797·3 (1281·9 to 2475·7)	1680·2 (1166·7 to 2356·7)	113·3 (93·1 to 135·7)	3·8 (2·6 to 5·3)	0·0 (0·0 to 0·0)
Colombia*	1995	281·8 (257·5 to 305·6)	155·3 (138·5 to 172·6)	105·2 (90·6 to 120·9)	20·8 (15·0 to 28·3)	0·5 (0·5 to 0·5)	765·1 (699·1 to 829·5)	421·6 (376·0 to 468·5)	285·5 (245·9 to 328·2)	56·4 (40·8 to 77·0)	1·4 (1·4 to 1·4)
Colombia*	1996	309·0 (287·6 to 331·9)	181·1 (166·0 to 196·7)	104·3 (91·5 to 117·1)	21·8 (16·0 to 29·6)	1·8 (1·8 to 1·8)	838·8 (780·7 to 900·9)	491·6 (450·7 to 534·0)	283·1 (248·5 to 317·9)	59·2 (43·3 to 80·3)	4·9 (4·9 to 4·9)
Colombia*	1997	320·7 (301·0 to 340·5)	199·6 (185·7 to 214·0)	96·1 (84·4 to 108·0)	23·4 (17·1 to 31·4)	1·7 (1·7 to 1·7)	870·8 (817·2 to 924·3)	541·9 (504·2 to 580·9)	260·9 (229·2 to 293·1)	63·5 (46·5 to 85·2)	4·5 (4·5 to 4·5)
Colombia*	1998	303·0 (285·5 to 320·1)	201·7 (188·9 to 214·8)	75·7 (67·0 to 85·5)	24·7 (18·3 to 32·8)	0·9 (0·9 to 0·9)	822·7 (775·0 to 869·0)	547·5 (512·9 to 583·2)	205·4 (181·9 to 232·1)	67·2 (49·8 to 89·0)	2·6 (2·6 to 2·6)
Colombia*	1999	270·9 (255·4 to 286·0)	189·1 (176·8 to 201·8)	54·6 (47·5 to 62·4)	25·5 (19·0 to 33·8)	1·7 (1·7 to 1·7)	735·3 (693·3 to 776·5)	513·5 (480·0 to 548·0)	148·1 (129·1 to 169·4)	69·2 (51·6 to 91·7)	4·5 (4·5 to 4·5)

Colombia*	2000	222·1 (210·0 to 234·3)	161·3 (151·6 to 170·7)	37·0 (31·9 to 42·2)	23·3 (17·1 to 30·4)	0·5 (0·5 to 0·5)	603·1 (570·0 to 636·1)	438·0 (411·5 to 463·4)	100·4 (86·6 to 114·6)	63·2 (46·5 to 82·4)	1·4 (1·4 to 1·4)
Colombia*	2001	210·4 (199·6 to 222·5)	154·7 (146·6 to 163·5)	30·3 (26·3 to 34·6)	23·2 (17·1 to 30·8)	2·1 (2·1 to 2·1)	571·2 (541·8 to 604·0)	420·1 (398·1 to 443·9)	82·2 (71·4 to 94·0)	63·1 (46·5 to 83·5)	5·8 (5·8 to 5·8)
Colombia*	2002	207·6 (196·4 to 219·3)	153·6 (145·4 to 162·1)	26·9 (23·6 to 30·8)	23·4 (17·3 to 30·6)	3·7 (3·7 to 3·7)	563·7 (533·3 to 595·3)	416·9 (394·6 to 440·1)	73·1 (64·0 to 83·5)	63·7 (47·1 to 83·1)	10·0 (10·0 to 10·0)
Colombia*	2003	213·4 (202·0 to 225·3)	157·6 (149·0 to 166·6)	26·7 (23·3 to 30·0)	24·6 (18·5 to 32·3)	4·4 (4·4 to 4·4)	579·3 (548·3 to 611·8)	427·9 (404·4 to 452·4)	72·4 (63·3 to 81·5)	66·9 (50·2 to 87·7)	12·1 (12·1 to 12·1)
Colombia*	2004	228·0 (217·2 to 240·9)	161·2 (153·2 to 170·0)	31·3 (27·7 to 34·9)	25·0 (18·7 to 32·6)	10·5 (10·5 to 10·5)	619·0 (589·7 to 653·9)	437·5 (415·8 to 461·5)	85·1 (75·3 to 94·9)	67·8 (50·7 to 88·5)	28·6 (28·6 to 28·6)
Colombia*	2005	237·6 (226·5 to 249·7)	168·2 (160·2 to 176·7)	38·0 (34·0 to 41·9)	25·3 (19·1 to 32·7)	6·1 (6·1 to 6·1)	644·9 (615·0 to 678·0)	456·6 (434·8 to 479·8)	103·1 (92·3 to 113·8)	68·7 (51·9 to 88·8)	16·5 (16·5 to 16·5)
Colombia*	2006	268·1 (256·8 to 280·7)	192·0 (182·6 to 201·6)	46·7 (42·3 to 51·2)	26·4 (20·0 to 33·8)	3·0 (3·0 to 3·0)	727·8 (697·1 to 762·1)	521·1 (495·7 to 547·3)	126·8 (114·8 to 139·0)	71·6 (54·3 to 91·7)	8·2 (8·2 to 8·2)
Colombia*	2007	291·5 (279·8 to 304·5)	206·0 (197·2 to 215·9)	54·1 (49·5 to 59·1)	28·2 (21·6 to 36·0)	3·2 (3·2 to 3·2)	791·4 (759·7 to 826·6)	559·4 (535·5 to 586·1)	146·9 (134·3 to 160·4)	76·5 (58·5 to 97·8)	8·6 (8·6 to 8·6)
Colombia*	2008	306·5 (294·0 to 319·0)	215·8 (206·8 to 225·4)	58·9 (54·3 to 63·6)	30·3 (23·4 to 38·0)	1·6 (1·6 to 1·6)	832·1 (798·1 to 866·1)	585·9 (561·5 to 611·8)	159·9 (147·3 to 172·7)	82·2 (63·4 to 103·2)	4·2 (4·2 to 4·2)
Colombia*	2009	334·2 (321·6 to 348·2)	235·5 (226·5 to 245·8)	60·3 (55·6 to 65·3)	33·4 (25·9 to 42·1)	5·0 (5·0 to 5·0)	907·3 (873·2 to 945·4)	639·2 (614·8 to 667·2)	163·7 (151·0 to 177·3)	90·8 (70·3 to 114·4)	13·5 (13·5 to 13·5)
Colombia*	2010	343·2 (330·4 to 356·6)	240·8 (232·1 to 251·3)	59·0 (54·7 to 64·0)	36·0 (29·1 to 44·0)	7·3 (7·3 to 7·3)	931·6 (896·9 to 968·1)	653·8 (630·2 to 682·3)	160·2 (148·4 to 173·6)	97·7 (79·1 to 119·4)	19·9 (19·9 to 19·9)
Colombia*	2011	349·5 (337·2 to 361·5)	249·1 (240·7 to 258·1)	58·9 (54·3 to 63·7)	39·1 (32·5 to 46·7)	2·4 (2·4 to 2·4)	948·8 (915·6 to 981·4)	676·3 (653·6 to 700·8)	159·8 (147·3 to 173·0)	106·2 (88·1 to 126·9)	6·5 (6·5 to 6·5)
Colombia*	2012	355·2 (343·6 to 366·2)	249·7 (240·9 to 259·0)	60·8 (56·2 to 65·7)	42·7 (37·0 to 49·0)	2·0 (2·0 to 2·0)	964·3 (932·9 to 994·3)	677·9 (654·0 to 703·2)	165·1 (152·6 to 178·4)	115·8 (100·5 to 133·1)	5·4 (5·4 to 5·4)
Colombia*	2013	388·1 (376·7 to 399·1)	266·5 (258·1 to 275·4)	63·8 (59·2 to 68·3)	48·8 (44·4 to 54·0)	9·0 (9·0 to 9·0)	1053·5 (1022·7 to 1083·5)	723·4 (700·7 to 747·6)	173·1 (160·8 to 185·5)	132·5 (120·6 to 146·7)	24·5 (24·5 to 24·5)

Colombia*	2014	427·1 (416·0 to 438·5)	288·4 (279·8 to 297·8)	66·2 (61·4 to 71·3)	54·6 (50·5 to 59·1)	17·9 (17·9 to 17·9)	1159·5 (1129·4 to 1190·3)	783·0 (759·7 to 808·5)	179·8 (166·7 to 193·6)	148·2 (137·0 to 160·5)	48·5 (48·5 to 48·5)
Colombia*	2015	435·4 (423·3 to 446·7)	307·2 (297·5 to 315·9)	67·9 (62·6 to 73·0)	59·8 (55·7 to 64·1)	0·4 (0·4 to 0·4)	1182·0 (1149·1 to 1212·6)	834·1 (807·6 to 857·6)	184·3 (170·1 to 198·1)	162·4 (151·1 to 174·1)	1·2 (1·2 to 1·2)
Colombia*	2016	437·1 (425·5 to 448·4)	307·6 (298·5 to 316·8)	67·9 (63·0 to 72·9)	61·3 (57·4 to 65·6)	0·3 (0·3 to 0·3)	1186·6 (1155·1 to 1217·4)	835·1 (810·5 to 860·1)	184·3 (171·1 to 197·8)	166·4 (155·7 to 178·2)	0·8 (0·8 to 0·8)
Colombia*	2017	438·6 (426·5 to 451·2)	309·3 (299·9 to 318·4)	67·8 (62·9 to 73·1)	61·1 (55·9 to 66·3)	0·4 (0·4 to 0·4)	1190·7 (1157·9 to 1225·1)	839·7 (814·3 to 864·4)	184·0 (170·8 to 198·4)	165·9 (151·8 to 180·0)	1·1 (1·1 to 1·1)
Colombia*	2018	463·3 (447·1 to 481·6)	330·8 (318·4 to 344·3)	69·4 (63·0 to 76·9)	62·7 (54·8 to 71·2)	0·3 (0·3 to 0·3)	1257·8 (1213·9 to 1307·5)	898·1 (864·3 to 934·7)	188·5 (171·0 to 208·9)	170·2 (148·7 to 193·4)	0·9 (0·9 to 0·9)
Colombia*	2019	447·3 (429·0 to 467·7)	310·8 (295·9 to 326·3)	70·9 (64·3 to 78·6)	65·2 (57·0 to 74·2)	0·4 (0·4 to 0·4)	1214·3 (1164·6 to 1269·7)	843·9 (803·3 to 885·9)	192·4 (174·5 to 213·3)	177·1 (154·7 to 201·4)	1·0 (1·0 to 1·0)
Colombia*	2020	482·4 (464·8 to 502·1)	357·0 (342·2 to 372·2)	64·4 (58·5 to 71·4)	60·5 (52·9 to 68·8)	0·5 (0·5 to 0·5)	1309·6 (1261·7 to 1363·0)	969·1 (928·9 to 1010·6)	174·8 (158·8 to 193·9)	164·3 (143·7 to 186·7)	1·5 (1·4 to 1·5)
Colombia*	2021	430·9 (411·9 to 452·4)	306·8 (290·5 to 325·5)	63·2 (57·4 to 70·1)	60·5 (53·0 to 68·9)	0·5 (0·5 to 0·5)	1169·9 (1118·1 to 1228·1)	832·8 (788·7 to 883·7)	171·5 (155·8 to 190·2)	164·3 (143·8 to 187·0)	1·4 (1·3 to 1·4)
Colombia*	2022	417·9 (397·1 to 441·7)	291·4 (273·2 to 312·1)	63·8 (57·9 to 70·8)	62·3 (54·4 to 71·0)	0·4 (0·4 to 0·4)	1134·5 (1078·0 to 1199·2)	791·2 (741·8 to 847·4)	173·1 (157·3 to 192·1)	169·1 (147·7 to 192·7)	1·1 (1·0 to 1·1)
Colombia*	2023	429·4 (406·4 to 455·3)	300·3 (279·4 to 324·2)	64·4 (58·5 to 71·4)	64·2 (56·0 to 73·1)	0·4 (0·4 to 0·4)	1165·7 (1103·4 to 1236·1)	815·3 (758·5 to 880·3)	174·9 (158·9 to 194·0)	174·4 (152·0 to 198·6)	1·1 (1·0 to 1·2)
Colombia*	2024	442·0 (416·7 to 470·1)	309·7 (286·9 to 335·5)	65·1 (59·1 to 72·2)	66·7 (58·2 to 75·8)	0·4 (0·4 to 0·4)	1199·9 (1131·3 to 1276·2)	840·8 (778·8 to 910·8)	176·7 (160·6 to 196·0)	181·2 (157·9 to 205·9)	1·1 (1·1 to 1·2)
Colombia*	2025	454·8 (427·3 to 484·9)	319·3 (293·6 to 347·2)	65·7 (59·8 to 72·9)	69·3 (60·0 to 79·0)	0·4 (0·4 to 0·5)	1234·6 (1159·9 to 1316·5)	866·9 (797·0 to 942·7)	178·4 (162·2 to 197·9)	188·1 (162·9 to 214·6)	1·2 (1·1 to 1·3)
Colombia*	2026	468·1 (437·6 to 500·4)	329·3 (301·9 to 359·2)	66·4 (60·3 to 73·6)	71·9 (62·2 to 82·1)	0·4 (0·4 to 0·5)	1270·8 (1187·9 to 1358·5)	894·1 (819·5 to 975·1)	180·2 (163·8 to 199·9)	195·3 (168·8 to 223·0)	1·2 (1·1 to 1·3)
Colombia*	2027	481·2 (449·4 to 516·2)	339·1 (308·9 to 372·4)	67·0 (60·8 to 74·3)	74·6 (64·3 to 85·4)	0·5 (0·4 to 0·5)	1306·3 (1220·0 to 1401·4)	920·6 (838·7 to 1011·0)	181·8 (165·2 to 201·7)	202·6 (174·6 to 231·8)	1·2 (1·1 to 1·3)

Colombia*	2028	494·1 (459·6 to 531·5)	348·8 (316·6 to 384·7)	67·5 (61·4 to 74·9)	77·3 (66·4 to 88·4)	0·5 (0·4 to 0·5)	1341·4 (1247·8 to 1442·8)	946·8 (859·6 to 1044·4)	183·4 (166·6 to 203·2)	210·0 (180·2 to 240·1)	1·3 (1·2 to 1·4)
Colombia*	2029	507·4 (470·5 to 547·0)	358·6 (324·8 to 398·0)	68·1 (61·9 to 75·5)	80·2 (69·0 to 92·2)	0·5 (0·4 to 0·5)	1377·4 (1277·4 to 1485·1)	973·6 (881·7 to 1080·4)	184·9 (168·0 to 204·9)	217·7 (187·2 to 250·3)	1·3 (1·2 to 1·4)
Colombia*	2030	521·3 (482·0 to 563·3)	369·0 (332·5 to 410·0)	68·7 (62·4 to 76·1)	83·1 (71·6 to 95·4)	0·5 (0·4 to 0·5)	1415·1 (1308·4 to 1529·3)	1001·7 (902·6 to 1113·1)	186·5 (169·3 to 206·6)	225·6 (194·5 to 259·1)	1·3 (1·2 to 1·5)
Colombia*	2031	534·5 (492·2 to 579·4)	378·7 (338·9 to 421·7)	69·2 (62·8 to 76·7)	86·1 (73·9 to 98·6)	0·5 (0·5 to 0·6)	1451·1 (1336·2 to 1573·0)	1028·1 (920·0 to 1144·7)	188·0 (170·6 to 208·3)	233·6 (200·5 to 267·8)	1·4 (1·2 to 1·5)
Colombia*	2032	548·1 (503·7 to 596·4)	388·7 (346·0 to 433·2)	69·8 (63·3 to 77·3)	89·1 (76·2 to 102·1)	0·5 (0·5 to 0·6)	1488·0 (1367·6 to 1619·1)	1055·1 (939·4 to 1176·0)	189·5 (171·9 to 209·9)	241·9 (206·9 to 277·2)	1·4 (1·3 to 1·6)
Colombia*	2033	562·0 (514·7 to 613·7)	398·9 (354·2 to 447·1)	70·4 (63·8 to 77·9)	92·2 (78·9 to 106·2)	0·5 (0·5 to 0·6)	1525·9 (1397·4 to 1666·0)	1082·9 (961·6 to 1213·7)	191·1 (173·3 to 211·5)	250·4 (214·3 to 288·3)	1·5 (1·3 to 1·6)
Colombia*	2034	575·3 (524·5 to 627·7)	408·4 (360·8 to 458·3)	71·0 (64·4 to 78·5)	95·4 (81·5 to 110·2)	0·6 (0·5 to 0·6)	1561·8 (1423·9 to 1704·2)	1108·6 (979·5 to 1244·3)	192·6 (174·7 to 213·2)	259·0 (221·3 to 299·3)	1·5 (1·3 to 1·7)
Colombia*	2035	589·1 (536·5 to 645·9)	418·3 (368·4 to 471·9)	71·5 (64·9 to 79·2)	98·7 (84·4 to 114·4)	0·6 (0·5 to 0·6)	1599·3 (1456·6 to 1753·7)	1135·6 (1000·2 to 1281·2)	194·2 (176·1 to 215·0)	267·9 (229·1 to 310·7)	1·5 (1·4 to 1·7)
Colombia*	2036	602·7 (546·6 to 661·5)	428·0 (374·7 to 484·8)	72·1 (65·4 to 79·9)	102·0 (87·2 to 118·7)	0·6 (0·5 to 0·7)	1636·2 (1484·0 to 1796·0)	1161·8 (1017·2 to 1316·3)	195·8 (177·5 to 216·8)	277·0 (236·7 to 322·4)	1·6 (1·4 to 1·8)
Colombia*	2037	616·5 (557·9 to 679·2)	437·7 (383·4 to 496·0)	72·7 (65·9 to 80·5)	105·5 (89·7 to 123·0)	0·6 (0·5 to 0·7)	1673·8 (1514·5 to 1843·9)	1188·3 (1040·9 to 1346·5)	197·4 (179·0 to 218·5)	286·4 (243·4 to 334·1)	1·6 (1·4 to 1·9)
Colombia*	2038	630·4 (569·0 to 695·3)	447·4 (389·7 to 510·2)	73·3 (66·5 to 81·1)	109·1 (92·2 to 127·6)	0·6 (0·5 to 0·7)	1711·5 (1544·8 to 1887·6)	1214·7 (1058·0 to 1385·0)	199·0 (180·5 to 220·3)	296·1 (250·3 to 346·4)	1·7 (1·5 to 2·0)
Colombia*	2039	644·7 (580·0 to 713·8)	457·4 (396·0 to 522·9)	73·9 (67·0 to 81·8)	112·7 (94·8 to 132·7)	0·6 (0·6 to 0·7)	1750·2 (1574·5 to 1937·9)	1241·8 (1075·0 to 1419·6)	200·7 (182·0 to 222·2)	306·0 (257·5 to 360·3)	1·7 (1·5 to 2·0)
Colombia*	2040	658·9 (592·3 to 731·9)	467·2 (403·2 to 536·0)	74·5 (67·6 to 82·6)	116·5 (97·2 to 137·8)	0·7 (0·6 to 0·8)	1788·7 (1608·1 to 1987·1)	1268·4 (1094·6 to 1455·1)	202·3 (183·6 to 224·3)	316·2 (263·9 to 374·2)	1·8 (1·5 to 2·1)
Colombia*	2041	673·1 (603·0 to 747·8)	477·1 (410·8 to 546·8)	75·1 (68·1 to 83·2)	120·3 (99·8 to 143·3)	0·7 (0·6 to 0·8)	1827·4 (1636·9 to 2030·0)	1295·1 (1115·3 to 1484·5)	203·9 (185·0 to 225·9)	326·6 (270·9 to 389·0)	1·8 (1·6 to 2·2)

Colombia*	2042	688·0 (615·6 to 766·8)	487·4 (419·8 to 559·3)	75·7 (68·7 to 83·8)	124·2 (102·1 to 148·8)	0·7 (0·6 to 0·8)	1867·9 (1671·4 to 2081·8)	1323·3 (1139·8 to 1518·4)	205·4 (186·4 to 227·6)	337·3 (277·2 to 403·9)	1·9 (1·6 to 2·3)
Colombia*	2043	702·8 (627·5 to 786·0)	497·5 (428·1 to 575·5)	76·2 (69·2 to 84·4)	128·3 (104·9 to 154·6)	0·7 (0·6 to 0·9)	1907·9 (1703·5 to 2134·0)	1350·8 (1162·1 to 1562·3)	206·9 (187·8 to 229·2)	348·3 (284·8 to 419·8)	2·0 (1·7 to 2·4)
Colombia*	2044	718·1 (638·7 to 803·0)	508·2 (435·3 to 588·0)	76·7 (69·6 to 85·0)	132·5 (107·6 to 160·7)	0·8 (0·6 to 0·9)	1949·6 (1734·0 to 2180·0)	1379·6 (1181·7 to 1596·2)	208·3 (189·0 to 230·9)	359·6 (292·2 to 436·3)	2·1 (1·8 to 2·5)
Colombia*	2045	733·1 (654·0 to 823·1)	518·3 (443·6 to 598·2)	77·2 (70·1 to 85·6)	136·7 (110·3 to 166·8)	0·8 (0·7 to 1·0)	1990·1 (1775·4 to 2234·7)	1407·2 (1204·2 to 1624·0)	209·7 (190·2 to 232·5)	371·1 (299·4 to 452·7)	2·1 (1·8 to 2·6)
Colombia*	2046	748·0 (664·5 to 839·4)	528·4 (452·4 to 611·1)	77·7 (70·5 to 86·3)	141·0 (112·9 to 173·4)	0·8 (0·7 to 1·0)	2030·8 (1804·0 to 2278·9)	1434·6 (1228·2 to 1659·0)	211·0 (191·3 to 234·2)	382·9 (306·5 to 470·8)	2·2 (1·9 to 2·7)
Colombia*	2047	763·2 (676·4 to 861·2)	538·6 (458·5 to 626·0)	78·2 (70·9 to 86·9)	145·5 (115·3 to 179·8)	0·9 (0·7 to 1·1)	2072·1 (1836·2 to 2338·0)	1462·3 (1244·9 to 1699·6)	212·4 (192·5 to 236·0)	395·1 (313·0 to 488·2)	2·3 (1·9 to 2·9)
Colombia*	2048	778·5 (688·7 to 879·1)	548·8 (465·5 to 638·9)	78·7 (71·3 to 87·5)	150·1 (118·1 to 186·2)	0·9 (0·7 to 1·1)	2113·6 (1869·7 to 2386·7)	1490·0 (1263·8 to 1734·5)	213·7 (193·7 to 237·5)	407·5 (320·5 to 505·6)	2·4 (2·0 to 3·0)
Colombia*	2049	794·1 (701·2 to 898·4)	559·2 (478·1 to 650·8)	79·2 (71·8 to 88·0)	154·8 (121·0 to 193·2)	0·9 (0·8 to 1·2)	2155·8 (1903·6 to 2439·1)	1518·1 (1297·9 to 1766·8)	215·0 (194·8 to 239·0)	420·2 (328·5 to 524·6)	2·5 (2·1 to 3·2)
Colombia*	2050	809·5 (714·1 to 917·2)	569·3 (483·9 to 670·2)	79·7 (72·2 to 88·6)	159·6 (123·5 to 200·8)	1·0 (0·8 to 1·2)	2197·8 (1938·6 to 2490·0)	1545·7 (1313·7 to 1819·5)	216·2 (195·9 to 240·4)	433·2 (335·4 to 545·2)	2·7 (2·2 to 3·4)
Comoros*	1995	98·7 (87·2 to 112·4)	10·4 (8·5 to 12·8)	67·5 (56·3 to 80·5)	1·0 (0·7 to 1·5)	19·7 (19·7 to 19·7)	222·3 (196·4 to 253·3)	23·5 (19·2 to 28·8)	152·2 (126·9 to 181·4)	2·3 (1·6 to 3·3)	44·3 (44·3 to 44·3)
Comoros*	1996	84·4 (74·0 to 95·9)	10·0 (8·2 to 12·3)	66·8 (56·4 to 78·5)	1·0 (0·7 to 1·4)	6·6 (6·6 to 6·6)	190·2 (166·8 to 216·1)	22·5 (18·4 to 27·7)	150·6 (127·1 to 176·9)	2·2 (1·6 to 3·2)	14·9 (14·9 to 14·9)
Comoros*	1997	91·5 (81·5 to 102·5)	10·1 (8·3 to 12·4)	67·3 (57·6 to 78·3)	1·0 (0·7 to 1·4)	13·1 (13·1 to 13·1)	206·1 (183·6 to 231·1)	22·9 (18·7 to 28·0)	151·6 (129·7 to 176·5)	2·2 (1·5 to 3·2)	29·5 (29·5 to 29·5)
Comoros*	1998	96·7 (87·0 to 106·9)	9·9 (8·1 to 12·1)	66·8 (57·6 to 76·8)	0·9 (0·6 to 1·3)	19·1 (19·1 to 19·1)	218·0 (196·0 to 241·0)	22·3 (18·3 to 27·4)	150·5 (129·8 to 173·1)	2·1 (1·5 to 3·0)	43·0 (43·0 to 43·0)
Comoros*	1999	80·6 (71·3 to 90·4)	9·8 (8·0 to 12·0)	66·7 (57·7 to 76·6)	0·9 (0·6 to 1·3)	3·2 (3·2 to 3·2)	181·6 (160·7 to 203·8)	22·1 (18·0 to 27·0)	150·3 (130·1 to 172·6)	2·1 (1·4 to 2·9)	7·2 (7·2 to 7·2)

Comoros*	2000	80·8 (72·2 to 90·7)	9·6 (7·9 to 11·6)	66·6 (58·4 to 76·6)	0·9 (0·6 to 1·3)	3·6 (3·6 to 3·6)	182·0 (162·7 to 204·5)	21·7 (17·8 to 26·2)	150·2 (131·6 to 172·5)	2·0 (1·3 to 2·9)	8·1 (8·1 to 8·1)
Comoros*	2001	86·2 (77·4 to 96·7)	11·4 (9·4 to 13·7)	70·1 (61·6 to 80·3)	1·0 (0·6 to 1·4)	3·7 (3·7 to 3·7)	194·2 (174·5 to 217·8)	25·8 (21·3 to 30·9)	158·0 (138·7 to 180·9)	2·2 (1·5 to 3·1)	8·3 (8·3 to 8·3)
Comoros*	2002	92·7 (83·4 to 103·3)	12·7 (10·5 to 15·1)	70·9 (61·5 to 81·2)	1·0 (0·7 to 1·4)	8·1 (8·1 to 8·1)	208·9 (187·9 to 232·8)	28·7 (23·7 to 34·1)	159·7 (138·6 to 183·0)	2·3 (1·5 to 3·3)	18·2 (18·2 to 18·2)
Comoros*	2003	91·9 (82·8 to 102·3)	11·8 (9·7 to 14·1)	67·7 (58·4 to 77·7)	1·0 (0·7 to 1·4)	11·4 (11·4 to 11·4)	207·1 (186·6 to 230·6)	26·6 (21·9 to 31·7)	152·5 (131·6 to 175·1)	2·2 (1·5 to 3·2)	25·8 (25·8 to 25·8)
Comoros*	2004	85·3 (76·1 to 94·5)	10·9 (8·9 to 13·0)	65·4 (56·8 to 74·5)	1·0 (0·7 to 1·4)	7·9 (7·9 to 7·9)	192·1 (171·5 to 212·9)	24·6 (20·2 to 29·3)	147·4 (127·9 to 167·8)	2·3 (1·5 to 3·3)	17·8 (17·8 to 17·8)
Comoros*	2005	81·0 (72·0 to 90·8)	10·5 (8·6 to 12·6)	64·6 (55·4 to 74·6)	1·0 (0·7 to 1·6)	4·9 (4·9 to 4·9)	182·5 (162·2 to 204·6)	23·6 (19·4 to 28·4)	145·5 (124·9 to 168·0)	2·4 (1·6 to 3·5)	11·1 (11·1 to 11·1)
Comoros*	2006	78·1 (69·1 to 88·0)	10·0 (8·2 to 12·1)	63·0 (54·1 to 72·8)	1·1 (0·7 to 1·5)	4·0 (4·0 to 4·0)	175·9 (155·7 to 198·2)	22·5 (18·4 to 27·2)	142·0 (121·9 to 164·1)	2·4 (1·6 to 3·5)	9·0 (9·0 to 9·0)
Comoros*	2007	73·6 (64·9 to 82·9)	8·9 (7·3 to 10·7)	59·9 (51·1 to 69·4)	1·0 (0·7 to 1·5)	3·7 (3·7 to 3·7)	165·8 (146·3 to 186·9)	20·1 (16·5 to 24·2)	135·1 (115·1 to 156·4)	2·3 (1·6 to 3·3)	8·3 (8·3 to 8·3)
Comoros*	2008	73·4 (64·5 to 82·3)	9·0 (7·4 to 10·9)	60·2 (51·3 to 69·1)	1·1 (0·8 to 1·5)	3·1 (3·1 to 3·1)	165·4 (145·2 to 185·5)	20·3 (16·6 to 24·6)	135·5 (115·5 to 155·7)	2·5 (1·7 to 3·5)	7·1 (7·1 to 7·1)
Comoros*	2009	73·5 (65·0 to 82·8)	8·1 (6·6 to 9·9)	57·5 (48·9 to 66·5)	1·1 (0·7 to 1·6)	6·7 (6·7 to 6·7)	165·6 (146·4 to 186·6)	18·3 (14·9 to 22·2)	129·6 (110·1 to 149·8)	2·5 (1·7 to 3·5)	15·2 (15·2 to 15·2)
Comoros*	2010	84·3 (76·0 to 93·9)	8·1 (6·7 to 9·9)	56·1 (48·0 to 65·4)	1·1 (0·8 to 1·6)	18·9 (18·9 to 18·9)	189·9 (171·2 to 211·6)	18·3 (15·0 to 22·2)	126·3 (108·2 to 147·4)	2·6 (1·8 to 3·6)	42·7 (42·7 to 42·7)
Comoros*	2011	78·0 (69·8 to 87·8)	9·1 (7·5 to 11·1)	56·2 (47·9 to 65·8)	1·2 (0·8 to 1·7)	11·5 (11·5 to 11·5)	175·8 (157·3 to 197·9)	20·5 (16·9 to 25·0)	126·6 (107·9 to 148·2)	2·7 (1·9 to 3·8)	26·0 (26·0 to 26·0)
Comoros*	2012	87·8 (79·9 to 97·2)	9·3 (7·7 to 11·3)	56·0 (48·2 to 65·1)	1·3 (0·9 to 1·8)	21·3 (21·3 to 21·3)	197·9 (180·0 to 219·0)	20·8 (17·3 to 25·4)	126·2 (108·7 to 146·8)	2·9 (1·9 to 4·1)	48·0 (48·0 to 48·0)
Comoros*	2013	85·3 (77·2 to 94·3)	9·4 (7·8 to 11·4)	55·9 (48·1 to 64·9)	1·3 (0·9 to 1·9)	18·7 (18·7 to 18·7)	192·1 (173·9 to 212·6)	21·1 (17·5 to 25·7)	126·0 (108·3 to 146·2)	2·9 (2·0 to 4·2)	42·0 (42·0 to 42·0)

Comoros*	2014	77·7 (69·2 to 86·7)	9·3 (7·7 to 11·3)	55·4 (47·3 to 64·0)	1·3 (0·9 to 1·9)	11·7 (11·7 to 11·7)	175·0 (155·9 to 195·5)	20·9 (17·3 to 25·5)	124·7 (106·6 to 144·3)	2·9 (2·0 to 4·2)	26·4 (26·4 to 26·4)
Comoros*	2015	76·6 (67·9 to 86·4)	10·5 (8·6 to 12·7)	57·7 (49·4 to 67·0)	1·4 (0·9 to 1·9)	7·1 (7·1 to 7·1)	172·7 (153·0 to 194·8)	23·7 (19·5 to 28·7)	129·9 (111·2 to 150·9)	3·1 (2·1 to 4·3)	16·0 (16·0 to 16·0)
Comoros*	2016	86·3 (77·6 to 96·5)	10·5 (8·6 to 12·8)	58·1 (49·8 to 67·9)	1·4 (0·9 to 1·9)	16·4 (16·4 to 16·4)	194·5 (174·9 to 217·5)	23·6 (19·5 to 28·7)	130·8 (112·1 to 153·1)	3·1 (2·1 to 4·3)	36·9 (36·9 to 36·9)
Comoros*	2017	76·9 (67·7 to 87·7)	9·3 (7·7 to 11·3)	56·9 (48·0 to 67·7)	1·3 (0·9 to 1·9)	9·4 (9·4 to 9·4)	173·2 (152·5 to 197·7)	20·9 (17·3 to 25·4)	128·2 (108·2 to 152·4)	3·0 (2·0 to 4·2)	21·1 (21·1 to 21·1)
Comoros*	2018	80·2 (71·2 to 92·0)	8·3 (7·0 to 10·2)	56·2 (47·0 to 67·9)	1·3 (0·9 to 1·8)	14·4 (14·4 to 14·4)	180·8 (160·4 to 207·4)	18·8 (15·7 to 23·1)	126·6 (106·0 to 152·9)	3·0 (2·0 to 4·1)	32·5 (32·5 to 32·5)
Comoros*	2019	82·8 (73·7 to 94·5)	10·1 (8·2 to 12·5)	55·8 (46·7 to 67·5)	1·4 (0·9 to 1·9)	15·5 (15·4 to 15·7)	186·6 (166·2 to 212·8)	22·8 (18·5 to 28·2)	125·8 (105·3 to 152·0)	3·0 (2·1 to 4·2)	34·9 (34·6 to 35·3)
Comoros*	2020	86·7 (77·7 to 97·9)	12·1 (9·7 to 15·2)	53·4 (44·6 to 64·6)	1·3 (0·9 to 1·8)	19·9 (19·7 to 20·1)	195·5 (175·1 to 220·5)	27·3 (21·9 to 34·2)	120·3 (100·6 to 145·5)	3·0 (2·0 to 4·2)	44·9 (44·4 to 45·2)
Comoros*	2021	83·7 (74·9 to 94·4)	11·7 (9·4 to 14·6)	51·2 (42·8 to 62·0)	1·3 (0·9 to 1·8)	19·5 (19·2 to 19·8)	188·7 (168·7 to 212·8)	26·3 (21·1 to 33·0)	115·5 (96·5 to 139·6)	2·9 (2·0 to 4·1)	44·0 (43·2 to 44·7)
Comoros*	2022	77·9 (69·4 to 88·7)	9·6 (7·5 to 12·5)	51·7 (43·2 to 62·6)	1·4 (0·9 to 1·9)	15·1 (14·4 to 16·0)	175·5 (156·3 to 200·0)	21·7 (16·9 to 28·1)	116·6 (97·4 to 141·0)	3·0 (2·1 to 4·2)	34·1 (32·5 to 36·0)
Comoros*	2023	79·0 (70·3 to 89·9)	9·8 (7·6 to 12·6)	52·3 (43·7 to 63·3)	1·4 (0·9 to 1·9)	15·5 (14·6 to 16·5)	178·0 (158·4 to 202·5)	22·1 (17·1 to 28·4)	117·9 (98·4 to 142·7)	3·2 (2·1 to 4·4)	34·9 (32·9 to 37·1)
Comoros*	2024	80·1 (71·4 to 91·0)	10·0 (7·5 to 13·1)	52·9 (44·2 to 64·0)	1·5 (1·0 to 2·0)	15·8 (14·8 to 17·0)	180·5 (161·0 to 205·0)	22·5 (17·0 to 29·4)	119·2 (99·5 to 144·3)	3·3 (2·2 to 4·6)	35·6 (33·3 to 38·2)
Comoros*	2025	81·3 (72·3 to 92·6)	10·2 (7·5 to 13·3)	53·5 (44·6 to 64·8)	1·5 (1·0 to 2·1)	16·1 (14·9 to 17·3)	183·1 (162·9 to 208·6)	23·0 (17·0 to 30·0)	120·5 (100·5 to 145·9)	3·4 (2·3 to 4·7)	36·3 (33·6 to 38·9)
Comoros*	2026	82·2 (73·0 to 93·8)	10·4 (7·6 to 13·7)	54·1 (45·1 to 65·5)	1·6 (1·1 to 2·2)	16·2 (14·9 to 17·4)	185·2 (164·4 to 211·2)	23·4 (17·2 to 30·9)	121·8 (101·7 to 147·5)	3·5 (2·4 to 4·9)	36·5 (33·6 to 39·3)
Comoros*	2027	83·2 (73·6 to 95·0)	10·6 (7·8 to 13·9)	54·6 (45·6 to 66·3)	1·6 (1·1 to 2·3)	16·3 (15·0 to 17·7)	187·4 (165·7 to 214·0)	23·9 (17·6 to 31·4)	123·1 (102·7 to 149·3)	3·7 (2·5 to 5·1)	36·7 (33·8 to 40·0)

Comoros*	2028	84·1 (74·4 to 95·8)	10·8 (8·0 to 14·4)	55·2 (46·1 to 66·9)	1·7 (1·1 to 2·4)	16·4 (15·0 to 18·2)	189·5 (167·6 to 215·8)	24·3 (18·0 to 32·4)	124·4 (103·9 to 150·8)	3·8 (2·6 to 5·3)	37·0 (33·8 to 41·0)
Comoros*	2029	85·1 (75·1 to 97·1)	11·0 (8·0 to 14·6)	55·7 (46·6 to 67·5)	1·8 (1·2 to 2·5)	16·6 (15·1 to 18·7)	191·7 (169·2 to 218·9)	24·7 (18·1 to 33·0)	125·6 (104·9 to 152·2)	3·9 (2·7 to 5·5)	37·4 (34·0 to 42·1)
Comoros*	2030	86·0 (76·1 to 98·2)	11·2 (8·2 to 15·0)	56·3 (47·0 to 68·2)	1·8 (1·2 to 2·6)	16·7 (15·0 to 19·1)	193·8 (171·4 to 221·2)	25·2 (18·4 to 33·8)	126·8 (105·9 to 153·6)	4·1 (2·8 to 5·8)	37·7 (33·8 to 43·0)
Comoros*	2031	87·0 (76·9 to 99·0)	11·4 (8·2 to 15·3)	56·8 (47·4 to 68·7)	1·9 (1·3 to 2·7)	16·9 (15·1 to 19·6)	196·0 (173·2 to 223·1)	25·6 (18·5 to 34·4)	128·0 (106·9 to 154·9)	4·3 (2·9 to 6·0)	38·1 (33·9 to 44·2)
Comoros*	2032	88·0 (77·8 to 100·0)	11·6 (8·3 to 15·8)	57·3 (47·8 to 69·3)	2·0 (1·3 to 2·8)	17·1 (15·1 to 20·1)	198·2 (175·3 to 225·3)	26·0 (18·6 to 35·6)	129·1 (107·7 to 156·3)	4·4 (3·0 to 6·2)	38·6 (34·1 to 45·3)
Comoros*	2033	89·0 (78·8 to 100·9)	11·8 (8·3 to 16·3)	57·7 (48·2 to 70·0)	2·0 (1·4 to 2·9)	17·4 (15·3 to 20·8)	200·4 (177·5 to 227·4)	26·5 (18·8 to 36·7)	130·1 (108·6 to 157·7)	4·6 (3·1 to 6·5)	39·3 (34·4 to 46·8)
Comoros*	2034	90·0 (79·5 to 102·1)	11·9 (8·5 to 16·4)	58·2 (48·6 to 70·6)	2·1 (1·4 to 3·0)	17·8 (15·5 to 21·4)	202·7 (179·1 to 230·1)	26·9 (19·2 to 37·0)	131·1 (109·5 to 159·2)	4·8 (3·2 to 6·7)	40·0 (34·9 to 48·3)
Comoros*	2035	91·0 (80·1 to 103·5)	12·1 (8·5 to 16·7)	58·6 (49·1 to 71·1)	2·2 (1·5 to 3·1)	18·1 (15·7 to 22·2)	205·1 (180·5 to 233·3)	27·3 (19·1 to 37·7)	132·0 (110·5 to 160·2)	4·9 (3·3 to 7·0)	40·9 (35·4 to 50·0)
Comoros*	2036	92·1 (81·0 to 105·0)	12·3 (8·6 to 17·1)	59·0 (49·4 to 71·5)	2·3 (1·5 to 3·2)	18·5 (15·9 to 23·0)	207·5 (182·6 to 236·7)	27·7 (19·3 to 38·6)	132·9 (111·4 to 161·0)	5·1 (3·4 to 7·3)	41·8 (35·8 to 51·9)
Comoros*	2037	93·2 (81·7 to 106·0)	12·5 (8·6 to 17·4)	59·4 (49·7 to 71·8)	2·4 (1·6 to 3·4)	19·0 (16·2 to 24·1)	209·9 (184·1 to 238·8)	28·1 (19·4 to 39·2)	133·8 (112·0 to 161·9)	5·3 (3·6 to 7·6)	42·7 (36·5 to 54·2)
Comoros*	2038	94·3 (82·8 to 107·5)	12·6 (8·8 to 17·8)	59·8 (50·0 to 72·2)	2·4 (1·6 to 3·5)	19·4 (16·4 to 24·9)	212·4 (186·7 to 242·2)	28·4 (19·7 to 40·2)	134·7 (112·7 to 162·7)	5·5 (3·7 to 7·9)	43·8 (37·0 to 56·2)
Comoros*	2039	95·4 (83·7 to 108·9)	12·8 (8·8 to 18·0)	60·1 (50·3 to 72·7)	2·5 (1·7 to 3·7)	20·0 (16·8 to 26·3)	215·1 (188·5 to 245·4)	28·8 (19·9 to 40·6)	135·5 (113·2 to 163·8)	5·7 (3·8 to 8·3)	45·0 (37·8 to 59·2)
Comoros*	2040	96·7 (84·7 to 110·7)	13·0 (8·9 to 18·3)	60·4 (50·6 to 73·2)	2·6 (1·7 to 3·8)	20·6 (17·1 to 27·7)	217·8 (190·8 to 249·4)	29·2 (20·0 to 41·2)	136·2 (113·9 to 164·9)	5·9 (3·9 to 8·6)	46·5 (38·5 to 62·5)
Comoros*	2041	97·9 (85·8 to 112·0)	13·1 (8·9 to 18·7)	60·7 (50·8 to 73·6)	2·7 (1·8 to 4·0)	21·4 (17·4 to 28·9)	220·6 (193·4 to 252·3)	29·6 (20·1 to 42·1)	136·8 (114·5 to 165·9)	6·1 (4·0 to 9·0)	48·1 (39·3 to 65·2)

Comoros*	2042	99·2 (86·9 to 113·8)	13·3 (9·0 to 18·9)	60·9 (51·1 to 74·1)	2·8 (1·8 to 4·1)	22·2 (17·9 to 30·7)	223·5 (195·9 to 256·5)	29·9 (20·2 to 42·5)	137·3 (115·1 to 166·9)	6·3 (4·1 to 9·3)	49·9 (40·3 to 69·2)
Comoros*	2043	100·5 (87·8 to 116·2)	13·4 (9·1 to 19·2)	61·2 (51·4 to 74·5)	2·9 (1·9 to 4·3)	23·0 (18·4 to 32·2)	226·5 (197·9 to 261·9)	30·2 (20·5 to 43·3)	137·9 (115·7 to 167·9)	6·6 (4·2 to 9·7)	51·8 (41·4 to 72·5)
Comoros*	2044	101·9 (88·9 to 118·5)	13·6 (9·1 to 19·6)	61·4 (51·6 to 74·8)	3·0 (1·9 to 4·5)	23·9 (19·1 to 34·1)	229·7 (200·4 to 267·0)	30·6 (20·6 to 44·2)	138·4 (116·3 to 168·5)	6·8 (4·3 to 10·1)	53·9 (43·1 to 76·9)
Comoros*	2045	103·4 (90·0 to 120·0)	13·8 (9·2 to 20·0)	61·7 (51·8 to 75·1)	3·1 (2·0 to 4·6)	24·8 (19·6 to 36·2)	232·9 (202·9 to 270·5)	31·0 (20·6 to 45·1)	138·9 (116·8 to 169·2)	7·0 (4·4 to 10·4)	56·0 (44·2 to 81·5)
Comoros*	2046	104·9 (91·5 to 122·5)	14·0 (9·2 to 20·6)	61·9 (52·0 to 75·4)	3·2 (2·0 to 4·8)	25·9 (20·3 to 38·1)	236·4 (206·1 to 276·0)	31·4 (20·8 to 46·4)	139·4 (117·1 to 169·8)	7·2 (4·6 to 10·7)	58·3 (45·8 to 85·8)
Comoros*	2047	106·5 (92·6 to 125·1)	14·1 (9·3 to 20·9)	62·1 (52·1 to 75·6)	3·3 (2·1 to 5·0)	27·0 (21·0 to 40·3)	240·0 (208·6 to 281·8)	31·9 (20·9 to 47·1)	139·9 (117·4 to 170·3)	7·5 (4·7 to 11·2)	60·8 (47·3 to 90·8)
Comoros*	2048	108·2 (93·8 to 127·6)	14·3 (9·4 to 21·1)	62·3 (52·2 to 75·8)	3·4 (2·1 to 5·2)	28·1 (21·7 to 42·6)	243·7 (211·4 to 287·6)	32·2 (21·2 to 47·5)	140·3 (117·6 to 170·9)	7·7 (4·8 to 11·7)	63·4 (49·0 to 96·0)
Comoros*	2049	109·9 (95·1 to 130·6)	14·4 (9·4 to 21·3)	62·5 (52·3 to 76·1)	3·5 (2·2 to 5·4)	29·4 (22·5 to 45·0)	247·6 (214·4 to 294·3)	32·6 (21·1 to 48·0)	140·8 (117·9 to 171·4)	8·0 (4·9 to 12·2)	66·3 (50·8 to 101·5)
Comoros*	2050	111·8 (96·1 to 132·7)	14·6 (9·5 to 21·9)	62·7 (52·4 to 76·3)	3·7 (2·2 to 5·7)	30·8 (23·3 to 47·3)	251·8 (216·5 to 299·1)	33·0 (21·5 to 49·4)	141·2 (118·1 to 171·9)	8·2 (5·1 to 12·7)	69·4 (52·4 to 106·6)
Cape Verde*	1995	81·6 (72·0 to 92·2)	68·1 (58·6 to 78·2)	11·2 (9·0 to 13·9)	1·1 (0·7 to 1·5)	1·1 (1·1 to 1·1)	169·5 (149·7 to 191·6)	141·6 (121·9 to 162·6)	23·4 (18·7 to 29·0)	2·2 (1·5 to 3·1)	2·4 (2·4 to 2·4)
Cape Verde*	1996	83·0 (73·4 to 93·3)	69·1 (59·7 to 78·9)	11·8 (9·5 to 14·4)	1·0 (0·7 to 1·5)	1·1 (1·1 to 1·1)	172·5 (152·6 to 193·8)	143·6 (124·1 to 164·0)	24·5 (19·8 to 30·0)	2·2 (1·5 to 3·1)	2·2 (2·2 to 2·2)
Cape Verde*	1997	96·3 (87·1 to 106·3)	69·6 (60·4 to 78·8)	13·4 (11·0 to 16·4)	1·0 (0·7 to 1·4)	12·2 (12·2 to 12·2)	200·1 (181·0 to 220·9)	144·7 (125·6 to 163·7)	27·9 (22·8 to 34·2)	2·1 (1·5 to 3·0)	25·4 (25·4 to 25·4)
Cape Verde*	1998	91·0 (82·2 to 100·1)	68·3 (60·0 to 76·8)	16·5 (13·6 to 19·7)	1·0 (0·7 to 1·4)	5·2 (5·2 to 5·2)	189·1 (170·9 to 208·0)	142·0 (124·6 to 159·6)	34·3 (28·2 to 41·0)	2·1 (1·4 to 2·9)	10·7 (10·7 to 10·7)
Cape Verde*	1999	102·5 (93·7 to 112·3)	74·0 (65·6 to 83·3)	19·5 (16·3 to 23·1)	1·0 (0·7 to 1·4)	8·0 (8·0 to 8·0)	213·0 (194·7 to 233·3)	153·8 (136·2 to 173·2)	40·5 (33·9 to 48·0)	2·1 (1·4 to 2·9)	16·6 (16·6 to 16·6)

Cape Verde*	2000	106·7 (97·9 to 116·6)	79·4 (71·1 to 88·8)	22·2 (19·1 to 25·7)	0·9 (0·6 to 1·3)	4·1 (4·1 to 4·1)	221·7 (203·5 to 242·4)	165·1 (147·8 to 184·5)	46·2 (39·7 to 53·4)	1·9 (1·3 to 2·6)	8·5 (8·5 to 8·5)
Cape Verde*	2001	113·4 (106·0 to 121·3)	70·0 (63·5 to 77·5)	22·5 (19·6 to 25·5)	0·8 (0·6 to 1·2)	20·0 (20·0 to 20·0)	235·7 (220·4 to 252·2)	145·5 (132·0 to 161·2)	46·8 (40·7 to 53·0)	1·7 (1·2 to 2·4)	41·7 (41·7 to 41·7)
Cape Verde*	2002	112·1 (105·0 to 119·3)	79·7 (73·6 to 86·4)	24·7 (21·7 to 27·8)	0·9 (0·6 to 1·3)	6·8 (6·8 to 6·8)	232·9 (218·1 to 248·0)	165·7 (152·9 to 179·6)	51·3 (45·2 to 57·7)	1·9 (1·3 to 2·6)	14·0 (14·0 to 14·0)
Cape Verde*	2003	118·4 (113·0 to 124·1)	71·9 (67·5 to 76·8)	24·4 (21·7 to 27·5)	0·9 (0·6 to 1·2)	21·2 (21·2 to 21·2)	246·1 (234·8 to 257·8)	149·5 (140·3 to 159·6)	50·7 (45·0 to 57·2)	1·8 (1·3 to 2·6)	44·1 (44·1 to 44·1)
Cape Verde*	2004	126·8 (121·8 to 132·3)	76·9 (72·6 to 81·5)	25·5 (22·8 to 28·5)	0·9 (0·6 to 1·3)	23·5 (23·5 to 23·5)	263·6 (253·1 to 274·9)	159·8 (151·0 to 169·4)	53·1 (47·5 to 59·3)	2·0 (1·3 to 2·8)	48·8 (48·8 to 48·8)
Cape Verde*	2005	136·1 (131·0 to 141·1)	78·3 (74·2 to 82·6)	26·2 (23·5 to 29·1)	1·0 (0·7 to 1·4)	30·6 (30·6 to 30·6)	282·9 (272·3 to 293·2)	162·8 (154·3 to 171·6)	54·5 (48·9 to 60·5)	2·0 (1·4 to 2·9)	63·6 (63·6 to 63·6)
Cape Verde*	2006	139·9 (134·8 to 145·3)	82·2 (78·0 to 86·5)	27·3 (24·6 to 30·1)	1·0 (0·7 to 1·4)	29·5 (29·5 to 29·5)	290·8 (280·1 to 302·0)	170·8 (162·1 to 179·7)	56·7 (51·2 to 62·5)	2·1 (1·5 to 2·9)	61·2 (61·2 to 61·2)
Cape Verde*	2007	136·0 (130·6 to 141·2)	81·9 (77·0 to 86·8)	27·4 (25·0 to 29·9)	1·0 (0·7 to 1·4)	25·6 (25·6 to 25·6)	282·6 (271·4 to 293·5)	170·2 (160·0 to 180·3)	57·0 (52·0 to 62·2)	2·2 (1·5 to 3·0)	53·2 (53·2 to 53·2)
Cape Verde*	2008	141·2 (135·1 to 147·6)	83·7 (78·0 to 89·5)	28·4 (26·3 to 30·5)	1·1 (0·7 to 1·5)	28·1 (28·1 to 28·1)	293·5 (280·8 to 306·7)	173·9 (162·0 to 186·1)	59·0 (54·7 to 63·4)	2·3 (1·5 to 3·2)	58·3 (58·3 to 58·3)
Cape Verde*	2009	131·7 (124·9 to 138·8)	87·1 (80·5 to 93·9)	30·5 (28·5 to 32·6)	1·2 (0·8 to 1·7)	13·0 (13·0 to 13·0)	273·7 (259·7 to 288·4)	181·1 (167·4 to 195·2)	63·3 (59·3 to 67·7)	2·5 (1·7 to 3·5)	26·9 (26·9 to 26·9)
Cape Verde*	2010	159·4 (152·3 to 166·8)	95·4 (88·8 to 102·1)	35·1 (33·2 to 37·1)	3·9 (2·6 to 5·5)	25·1 (25·1 to 25·1)	331·4 (316·5 to 346·7)	198·3 (184·6 to 212·2)	72·9 (69·0 to 77·0)	8·0 (5·4 to 11·4)	52·1 (52·1 to 52·1)
Cape Verde*	2011	173·5 (168·3 to 178·6)	90·2 (85·5 to 95·0)	36·0 (34·0 to 38·0)	3·9 (2·6 to 5·5)	43·5 (43·5 to 43·5)	360·7 (349·9 to 371·1)	187·4 (177·8 to 197·4)	74·8 (70·7 to 79·1)	8·0 (5·5 to 11·4)	90·4 (90·4 to 90·4)
Cape Verde*	2012	165·5 (161·8 to 168·9)	94·8 (92·3 to 97·3)	38·7 (36·7 to 40·8)	4·1 (2·8 to 5·7)	27·8 (27·8 to 27·8)	343·9 (336·3 to 351·1)	197·0 (191·7 to 202·3)	80·5 (76·2 to 84·8)	8·5 (5·9 to 11·9)	57·8 (57·8 to 57·8)
Cape Verde*	2013	183·3 (180·1 to 186·5)	95·8 (93·7 to 97·9)	40·4 (38·4 to 42·5)	4·1 (2·8 to 5·7)	43·0 (43·0 to 43·0)	381·0 (374·3 to 387·5)	199·1 (194·7 to 203·5)	83·9 (79·9 to 88·3)	8·6 (5·9 to 11·9)	89·4 (89·4 to 89·4)

Cape Verde*	2014	169·8 (166·7 to 173·0)	92·9 (91·0 to 94·8)	41·0 (39·0 to 43·2)	3·8 (2·5 to 5·2)	32·1 (32·1 to 32·1)	352·9 (346·5 to 359·5)	193·0 (189·1 to 197·0)	85·3 (81·1 to 89·7)	7·8 (5·3 to 10·9)	66·8 (66·8 to 66·8)
Cape Verde*	2015	167·5 (164·4 to 171·1)	97·1 (95·0 to 99·2)	42·3 (40·1 to 44·5)	3·7 (2·5 to 5·2)	24·4 (24·4 to 24·4)	348·1 (341·6 to 355·5)	201·7 (197·5 to 206·2)	87·9 (83·4 to 92·5)	7·8 (5·2 to 10·8)	50·7 (50·7 to 50·7)
Cape Verde*	2016	156·6 (152·8 to 160·5)	98·2 (95·7 to 100·8)	44·0 (41·3 to 46·7)	4·0 (2·7 to 5·8)	10·4 (10·4 to 10·4)	325·5 (317·5 to 333·5)	204·1 (198·9 to 209·4)	91·4 (85·9 to 97·1)	8·4 (5·6 to 12·0)	21·7 (21·7 to 21·7)
Cape Verde*	2017	166·2 (159·4 to 173·4)	106·6 (101·5 to 111·8)	47·3 (43·1 to 51·7)	4·4 (2·9 to 6·3)	8·0 (8·0 to 8·0)	345·5 (331·3 to 360·3)	221·6 (210·9 to 232·3)	98·2 (89·6 to 107·4)	9·1 (6·1 to 13·1)	16·6 (16·6 to 16·6)
Cape Verde*	2018	175·4 (164·0 to 187·4)	110·0 (101·3 to 120·0)	49·3 (43·3 to 55·3)	4·6 (3·1 to 6·5)	11·6 (11·6 to 11·6)	364·6 (340·9 to 389·4)	228·6 (210·5 to 249·4)	102·4 (89·9 to 115·0)	9·5 (6·3 to 13·5)	24·1 (24·1 to 24·1)
Cape Verde*	2019	182·9 (170·3 to 195·6)	116·0 (106·7 to 126·9)	51·3 (45·0 to 57·6)	4·8 (3·2 to 6·9)	10·8 (10·6 to 11·0)	380·2 (354·0 to 406·6)	241·1 (221·8 to 263·7)	106·6 (93·6 to 119·7)	10·0 (6·7 to 14·4)	22·4 (22·1 to 22·8)
Cape Verde*	2020	210·2 (195·3 to 226·6)	142·2 (129·8 to 156·6)	48·5 (42·5 to 54·4)	4·7 (3·1 to 6·7)	14·9 (14·8 to 15·0)	437·0 (405·9 to 470·9)	295·5 (269·7 to 325·5)	100·8 (88·3 to 113·2)	9·8 (6·5 to 14·0)	31·0 (30·7 to 31·2)
Cape Verde*	2021	204·5 (189·9 to 220·3)	138·3 (126·3 to 152·4)	47·1 (41·3 to 52·9)	4·7 (3·1 to 6·7)	14·4 (14·2 to 14·6)	425·1 (394·7 to 458·0)	287·5 (262·4 to 316·7)	98·0 (85·9 to 110·0)	9·7 (6·5 to 13·9)	29·9 (29·5 to 30·4)
Cape Verde*	2022	176·6 (162·0 to 191·5)	112·8 (100·2 to 126·1)	47·9 (42·0 to 53·8)	4·9 (3·2 to 7·0)	11·0 (10·4 to 11·6)	367·0 (336·8 to 398·0)	234·5 (208·3 to 262·1)	99·5 (87·4 to 111·7)	10·1 (6·7 to 14·5)	22·8 (21·7 to 24·0)
Cape Verde*	2023	180·9 (165·6 to 196·5)	116·2 (103·2 to 129·6)	48·7 (42·7 to 54·6)	5·1 (3·4 to 7·3)	11·0 (10·4 to 11·7)	376·1 (344·1 to 408·3)	241·4 (214·5 to 269·5)	101·2 (88·8 to 113·6)	10·6 (7·0 to 15·2)	22·9 (21·6 to 24·3)
Cape Verde*	2024	185·7 (168·8 to 201·9)	119·9 (104·9 to 134·3)	49·4 (43·3 to 55·5)	5·3 (3·5 to 7·6)	11·1 (10·4 to 11·9)	385·9 (350·8 to 419·5)	249·1 (218·0 to 279·2)	102·7 (90·1 to 115·3)	11·0 (7·2 to 15·7)	23·1 (21·7 to 24·6)
Cape Verde*	2025	190·5 (172·3 to 208·6)	123·7 (107·2 to 140·2)	50·1 (44·0 to 56·2)	5·5 (3·6 to 7·8)	11·2 (10·5 to 12·0)	395·9 (358·2 to 433·5)	257·1 (222·8 to 291·3)	104·2 (91·4 to 116·9)	11·4 (7·5 to 16·3)	23·3 (21·8 to 24·9)
Cape Verde*	2026	195·6 (176·1 to 215·6)	127·8 (110·6 to 145·9)	50·8 (44·6 to 57·0)	5·7 (3·8 to 8·1)	11·2 (10·5 to 12·1)	406·5 (366·0 to 448·0)	265·7 (229·9 to 303·2)	105·6 (92·6 to 118·4)	11·8 (7·8 to 16·9)	23·4 (21·8 to 25·2)
Cape Verde*	2027	200·7 (179·7 to 224·3)	132·1 (112·7 to 153·1)	51·5 (45·1 to 57·8)	5·9 (3·9 to 8·4)	11·3 (10·5 to 12·2)	417·2 (373·5 to 466·1)	274·5 (234·2 to 318·1)	107·0 (93·8 to 120·0)	12·3 (8·1 to 17·4)	23·5 (21·7 to 25·4)

Cape Verde*	2028	206·2 (183·8 to 230·6)	136·6 (116·3 to 158·9)	52·1 (45·7 to 58·5)	6·1 (4·0 to 8·7)	11·3 (10·4 to 12·4)	428·5 (382·0 to 479·3)	283·9 (241·7 to 330·3)	108·4 (95·0 to 121·6)	12·7 (8·4 to 18·1)	23·5 (21·7 to 25·7)
Cape Verde*	2029	211·7 (187·3 to 237·5)	141·2 (117·9 to 164·1)	52·8 (46·2 to 59·2)	6·3 (4·2 to 9·0)	11·4 (10·4 to 12·5)	439·9 (389·3 to 493·6)	293·4 (245·1 to 341·0)	109·7 (96·1 to 123·0)	13·2 (8·7 to 18·8)	23·7 (21·7 to 25·9)
Cape Verde*	2030	216·8 (190·8 to 244·7)	145·4 (121·2 to 169·7)	53·3 (46·7 to 59·7)	6·5 (4·3 to 9·3)	11·6 (10·5 to 12·7)	450·5 (396·6 to 508·7)	302·2 (251·9 to 352·8)	110·7 (97·0 to 124·1)	13·6 (9·0 to 19·4)	24·0 (21·8 to 26·4)
Cape Verde*	2031	221·7 (193·6 to 250·4)	149·4 (124·0 to 175·0)	53·8 (47·1 to 60·3)	6·8 (4·5 to 9·6)	11·8 (10·6 to 13·0)	460·7 (402·5 to 520·4)	310·6 (257·8 to 363·7)	111·7 (97·9 to 125·3)	14·0 (9·3 to 20·0)	24·4 (22·1 to 27·0)
Cape Verde*	2032	226·4 (197·5 to 256·5)	153·2 (126·5 to 180·2)	54·2 (47·5 to 60·8)	7·0 (4·6 to 10·0)	11·9 (10·8 to 13·2)	470·5 (410·5 to 533·1)	318·4 (262·9 to 374·5)	112·7 (98·8 to 126·4)	14·5 (9·6 to 20·7)	24·8 (22·4 to 27·5)
Cape Verde*	2033	231·2 (200·5 to 263·4)	157·0 (129·0 to 186·1)	54·8 (48·0 to 61·4)	7·2 (4·8 to 10·3)	12·1 (10·9 to 13·5)	480·4 (416·8 to 547·4)	326·4 (268·2 to 386·7)	113·8 (99·7 to 127·7)	15·0 (9·9 to 21·4)	25·2 (22·6 to 28·0)
Cape Verde*	2034	236·2 (205·0 to 269·8)	161·1 (132·1 to 191·6)	55·3 (48·4 to 62·1)	7·5 (4·9 to 10·7)	12·3 (11·0 to 13·8)	490·9 (426·1 to 560·8)	334·9 (274·5 to 398·3)	114·9 (100·7 to 129·0)	15·5 (10·3 to 22·1)	25·6 (23·0 to 28·6)
Cape Verde*	2035	241·1 (209·4 to 274·6)	165·0 (134·6 to 196·6)	55·8 (48·9 to 62·7)	7·7 (5·1 to 11·0)	12·6 (11·2 to 14·1)	501·1 (435·3 to 570·7)	343·0 (279·7 to 408·6)	116·0 (101·6 to 130·3)	16·0 (10·6 to 22·9)	26·1 (23·3 to 29·3)
Cape Verde*	2036	245·9 (212·8 to 282·4)	168·8 (137·4 to 202·2)	56·3 (49·3 to 63·3)	8·0 (5·3 to 11·4)	12·8 (11·4 to 14·4)	511·0 (442·3 to 586·9)	350·8 (285·6 to 420·3)	117·1 (102·5 to 131·5)	16·5 (10·9 to 23·7)	26·6 (23·7 to 30·0)
Cape Verde*	2037	250·9 (216·5 to 288·3)	172·8 (140·2 to 208·1)	56·8 (49·8 to 63·9)	8·2 (5·4 to 11·8)	13·1 (11·6 to 14·8)	521·6 (449·9 to 599·1)	359·2 (291·4 to 432·5)	118·2 (103·5 to 132·8)	17·1 (11·3 to 24·6)	27·2 (24·2 to 30·8)
Cape Verde*	2038	256·0 (220·3 to 295·9)	176·9 (143·6 to 214·3)	57·4 (50·3 to 64·5)	8·5 (5·6 to 12·3)	13·3 (11·8 to 15·2)	532·2 (457·8 to 614·9)	367·6 (298·5 to 445·3)	119·2 (104·5 to 134·1)	17·6 (11·7 to 25·5)	27·7 (24·6 to 31·7)
Cape Verde*	2039	261·0 (223·3 to 302·3)	180·6 (144·8 to 220·0)	57·9 (50·7 to 65·1)	8·8 (5·8 to 12·7)	13·7 (12·1 to 15·6)	542·4 (464·2 to 628·3)	375·4 (300·9 to 457·3)	120·3 (105·4 to 135·3)	18·2 (12·1 to 26·4)	28·4 (25·1 to 32·5)
Cape Verde*	2040	266·2 (226·8 to 308·9)	184·7 (147·0 to 225·7)	58·4 (51·2 to 65·8)	9·1 (6·0 to 13·2)	14·0 (12·3 to 16·1)	553·2 (471·4 to 642·1)	383·9 (305·5 to 469·1)	121·4 (106·4 to 136·7)	18·8 (12·4 to 27·4)	29·1 (25·5 to 33·5)
Cape Verde*	2041	271·5 (231·1 to 314·9)	188·8 (147·6 to 231·2)	59·0 (51·6 to 66·4)	9·4 (6·2 to 13·6)	14·3 (12·5 to 16·5)	564·2 (480·3 to 654·4)	392·4 (306·8 to 480·6)	122·5 (107·3 to 138·0)	19·5 (12·9 to 28·4)	29·8 (26·0 to 34·4)

Cape Verde*	2042	276·7 (233·4 to 320·1)	192·9 (149·7 to 235·9)	59·5 (52·1 to 67·0)	9·7 (6·4 to 14·1)	14·7 (12·7 to 17·2)	575·2 (485·1 to 665·3)	400·9 (311·0 to 490·3)	123·6 (108·2 to 139·2)	20·1 (13·3 to 29·4)	30·6 (26·4 to 35·9)
Cape Verde*	2043	282·2 (235·4 to 329·3)	197·1 (150·9 to 242·9)	60·0 (52·5 to 67·6)	10·0 (6·5 to 14·6)	15·2 (13·1 to 17·9)	586·6 (489·2 to 684·5)	409·6 (313·6 to 504·9)	124·6 (109·1 to 140·5)	20·7 (13·6 to 30·4)	31·6 (27·3 to 37·2)
Cape Verde*	2044	287·8 (238·3 to 336·9)	201·4 (152·4 to 249·3)	60·4 (52·9 to 68·1)	10·3 (6·7 to 15·1)	15·7 (13·5 to 18·5)	598·2 (495·4 to 700·3)	418·6 (316·8 to 518·2)	125·6 (110·0 to 141·6)	21·4 (14·0 to 31·3)	32·7 (28·2 to 38·5)
Cape Verde*	2045	293·7 (241·6 to 344·9)	206·0 (155·1 to 255·5)	60·8 (53·3 to 68·6)	10·6 (7·0 to 15·5)	16·3 (14·0 to 19·4)	610·4 (502·1 to 716·8)	428·2 (322·5 to 531·1)	126·4 (110·7 to 142·7)	22·1 (14·5 to 32·3)	33·8 (29·0 to 40·3)
Cape Verde*	2046	299·8 (245·6 to 352·5)	210·7 (158·4 to 262·7)	61·2 (53·6 to 69·2)	10·9 (7·2 to 16·0)	16·9 (14·4 to 20·2)	623·1 (510·6 to 732·6)	437·9 (329·2 to 546·1)	127·3 (111·4 to 143·8)	22·7 (14·9 to 33·3)	35·1 (29·9 to 42·0)
Cape Verde*	2047	305·9 (249·5 to 359·8)	215·4 (159·1 to 266·9)	61·6 (53·9 to 69·6)	11·3 (7·3 to 16·5)	17·6 (14·9 to 21·2)	635·7 (518·5 to 747·8)	447·7 (330·8 to 554·7)	128·1 (112·0 to 144·6)	23·4 (15·3 to 34·3)	36·5 (30·9 to 44·1)
Cape Verde*	2048	312·1 (252·9 to 368·1)	220·3 (162·8 to 275·3)	61·9 (54·2 to 69·9)	11·6 (7·5 to 17·0)	18·4 (15·5 to 22·3)	648·8 (525·6 to 765·1)	457·8 (338·4 to 572·2)	128·7 (112·6 to 145·3)	24·1 (15·6 to 35·4)	38·2 (32·3 to 46·3)
Cape Verde*	2049	318·4 (257·3 to 375·7)	225·1 (165·1 to 279·6)	62·1 (54·3 to 70·1)	11·9 (7·7 to 17·5)	19·4 (16·2 to 23·9)	661·9 (534·7 to 780·9)	467·8 (343·2 to 581·1)	129·0 (112·9 to 145·7)	24·7 (15·9 to 36·5)	40·3 (33·7 to 49·6)
Cape Verde*	2050	325·3 (260·7 to 385·8)	230·4 (167·3 to 288·9)	62·2 (54·4 to 70·2)	12·2 (7·8 to 18·1)	20·5 (16·9 to 25·4)	676·1 (541·8 to 801·7)	478·9 (347·8 to 600·4)	129·4 (113·2 to 146·0)	25·4 (16·2 to 37·6)	42·5 (35·1 to 52·9)
Costa Rica*	1995	421·3 (393·1 to 450·7)	258·5 (238·4 to 281·0)	148·7 (132·2 to 166·1)	11·1 (8·0 to 15·6)	2·9 (2·9 to 2·9)	699·5 (652·7 to 748·4)	429·3 (395·8 to 466·6)	246·9 (219·5 to 275·8)	18·5 (13·2 to 25·9)	4·8 (4·8 to 4·8)
Costa Rica*	1996	431·7 (408·5 to 456·9)	267·1 (249·5 to 285·7)	150·2 (135·6 to 165·8)	11·3 (8·0 to 15·8)	3·1 (3·1 to 3·1)	716·8 (678·3 to 758·6)	443·5 (414·4 to 474·3)	249·4 (225·2 to 275·4)	18·8 (13·3 to 26·2)	5·1 (5·1 to 5·1)
Costa Rica*	1997	441·0 (420·0 to 463·2)	275·8 (260·7 to 291·7)	150·3 (137·3 to 165·2)	11·2 (7·9 to 15·7)	3·7 (3·7 to 3·7)	732·2 (697·3 to 769·1)	457·9 (432·9 to 484·4)	249·5 (228·0 to 274·2)	18·7 (13·1 to 26·1)	6·1 (6·1 to 6·1)
Costa Rica*	1998	470·4 (449·1 to 492·6)	301·4 (285·9 to 317·0)	153·3 (139·4 to 168·9)	11·1 (7·8 to 15·2)	4·6 (4·6 to 4·6)	781·0 (745·7 to 817·9)	500·5 (474·7 to 526·3)	254·6 (231·5 to 280·4)	18·4 (13·0 to 25·3)	7·6 (7·6 to 7·6)
Costa Rica*	1999	490·7 (470·1 to 512·9)	318·1 (302·7 to 334·5)	156·0 (142·0 to 171·8)	11·2 (8·0 to 15·3)	5·5 (5·5 to 5·5)	814·9 (780·6 to 851·6)	528·3 (502·6 to 555·5)	259·0 (235·8 to 285·3)	18·5 (13·3 to 25·4)	9·1 (9·1 to 9·1)

Costa Rica*	2000	510·7 (491·3 to 533·1)	331·8 (316·2 to 348·1)	160·2 (145·7 to 175·0)	11·3 (8·0 to 15·3)	7·4 (7·4 to 7·4)	848·0 (815·8 to 885·2)	551·0 (525·1 to 578·0)	266·0 (242·0 to 290·6)	18·7 (13·3 to 25·5)	12·3 (12·3 to 12·3)
Costa Rica*	2001	522·3 (501·5 to 544·6)	344·7 (329·3 to 360·8)	164·4 (149·6 to 179·5)	10·3 (7·2 to 14·0)	2·8 (2·8 to 2·8)	867·2 (832·7 to 904·3)	572·4 (546·8 to 599·1)	272·9 (248·4 to 298·1)	17·1 (12·0 to 23·2)	4·7 (4·7 to 4·7)
Costa Rica*	2002	560·8 (538·9 to 582·8)	372·5 (356·2 to 388·9)	173·0 (158·1 to 188·0)	11·8 (8·5 to 16·1)	3·5 (3·5 to 3·5)	931·1 (894·9 to 967·8)	618·5 (591·4 to 645·8)	287·3 (262·5 to 312·2)	19·6 (14·0 to 26·7)	5·7 (5·7 to 5·7)
Costa Rica*	2003	570·7 (548·4 to 593·7)	375·6 (360·2 to 392·8)	178·6 (163·6 to 193·8)	13·1 (9·3 to 17·8)	3·4 (3·4 to 3·4)	947·6 (910·7 to 985·8)	623·7 (598·1 to 652·2)	296·6 (271·6 to 321·8)	21·7 (15·5 to 29·5)	5·7 (5·7 to 5·7)
Costa Rica*	2004	581·2 (558·7 to 603·3)	381·2 (364·5 to 397·9)	185·0 (170·5 to 200·3)	12·8 (9·3 to 17·2)	2·1 (2·1 to 2·1)	965·1 (927·7 to 1001·8)	633·0 (605·3 to 660·7)	307·3 (283·1 to 332·6)	21·3 (15·5 to 28·6)	3·5 (3·5 to 3·5)
Costa Rica*	2005	606·9 (584·6 to 630·2)	396·1 (379·4 to 413·8)	193·8 (179·6 to 209·6)	16·0 (11·8 to 21·5)	1·0 (1·0 to 1·0)	1007·7 (970·7 to 1046·4)	657·7 (629·9 to 687·1)	321·8 (298·2 to 348·1)	26·5 (19·5 to 35·7)	1·7 (1·7 to 1·7)
Costa Rica*	2006	647·0 (623·4 to 672·1)	424·5 (407·0 to 441·6)	203·2 (189·1 to 218·0)	17·6 (12·9 to 23·5)	1·8 (1·8 to 1·8)	1074·4 (1035·2 to 1116·1)	704·8 (675·7 to 733·3)	337·4 (313·9 to 362·0)	29·2 (21·4 to 39·0)	3·0 (3·0 to 3·0)
Costa Rica*	2007	699·5 (677·6 to 723·7)	465·2 (447·4 to 483·1)	213·4 (198·9 to 228·8)	19·1 (14·1 to 25·4)	1·8 (1·8 to 1·8)	1161·5 (1125·2 to 1201·6)	772·4 (742·9 to 802·1)	354·3 (330·2 to 379·9)	31·8 (23·5 to 42·2)	3·0 (3·0 to 3·0)
Costa Rica*	2008	768·7 (744·6 to 793·9)	524·0 (505·3 to 543·2)	221·7 (205·9 to 238·2)	20·4 (15·2 to 26·7)	2·6 (2·6 to 2·6)	1276·4 (1236·4 to 1318·3)	870·1 (839·0 to 901·9)	368·1 (341·9 to 395·5)	33·9 (25·2 to 44·4)	4·4 (4·4 to 4·4)
Costa Rica*	2009	814·4 (790·6 to 839·7)	570·0 (549·9 to 588·6)	221·0 (205·4 to 236·6)	21·4 (16·1 to 27·8)	1·9 (1·9 to 1·9)	1352·2 (1312·7 to 1394·2)	946·5 (913·2 to 977·4)	367·0 (341·0 to 392·9)	35·6 (26·8 to 46·1)	3·1 (3·1 to 3·1)
Costa Rica*	2010	874·9 (850·0 to 899·5)	625·0 (604·7 to 643·7)	224·6 (209·1 to 240·8)	24·3 (18·4 to 30·9)	1·0 (1·0 to 1·0)	1452·7 (1411·3 to 1493·6)	1037·8 (1004·1 to 1068·9)	372·9 (347·3 to 399·8)	40·3 (30·6 to 51·3)	1·7 (1·7 to 1·7)
Costa Rica*	2011	878·9 (855·6 to 904·0)	622·5 (603·5 to 640·1)	223·2 (208·2 to 239·3)	32·8 (25·4 to 41·5)	0·4 (0·4 to 0·4)	1459·3 (1420·6 to 1501·1)	1033·7 (1002·1 to 1062·9)	370·6 (345·7 to 397·3)	54·4 (42·2 to 69·0)	0·6 (0·6 to 0·6)
Costa Rica*	2012	895·4 (870·4 to 921·1)	633·8 (615·2 to 653·0)	220·8 (205·8 to 236·7)	40·6 (32·0 to 51·1)	0·2 (0·2 to 0·2)	1486·8 (1445·2 to 1529·5)	1052·5 (1021·5 to 1084·2)	366·6 (341·8 to 393·1)	67·4 (53·1 to 84·9)	0·4 (0·4 to 0·4)
Costa Rica*	2013	920·6 (895·7 to 947·3)	654·9 (635·8 to 675·8)	218·9 (203·4 to 236·2)	46·4 (37·4 to 57·2)	0·4 (0·4 to 0·4)	1528·6 (1487·2 to 1573·0)	1087·4 (1055·7 to 1122·1)	363·5 (337·8 to 392·3)	77·0 (62·1 to 95·0)	0·6 (0·6 to 0·6)

Costa Rica*	2014	926·1 (899·8 to 951·8)	663·0 (643·4 to 683·5)	217·2 (202·1 to 233·5)	45·4 (37·8 to 54·1)	0·4 (0·4 to 0·4)	1537·7 (1494·0 to 1580·3)	1100·9 (1068·3 to 1134·8)	360·7 (335·7 to 387·8)	75·4 (62·7 to 89·9)	0·7 (0·7 to 0·7)
Costa Rica*	2015	937·8 (912·9 to 962·1)	678·4 (659·9 to 696·9)	213·0 (198·6 to 227·7)	45·9 (39·6 to 52·9)	0·4 (0·4 to 0·4)	1557·1 (1515·9 to 1597·5)	1126·4 (1095·8 to 1157·2)	353·8 (329·7 to 378·1)	76·2 (65·8 to 87·8)	0·7 (0·7 to 0·7)
Costa Rica*	2016	963·9 (944·0 to 986·6)	683·9 (667·3 to 701·0)	209·1 (196·6 to 221·9)	45·9 (42·0 to 50·4)	25·0 (25·0 to 25·0)	1600·6 (1567·4 to 1638·2)	1135·7 (1108·0 to 1164·0)	347·2 (326·5 to 368·4)	76·2 (69·7 to 83·6)	41·5 (41·5 to 41·5)
Costa Rica*	2017	959·7 (944·8 to 976·9)	700·7 (688·6 to 712·1)	209·6 (198·7 to 220·3)	49·0 (47·5 to 50·5)	0·4 (0·4 to 0·4)	1593·6 (1568·8 to 1622·1)	1163·5 (1143·4 to 1182·4)	348·1 (329·9 to 365·7)	81·4 (78·9 to 83·9)	0·6 (0·6 to 0·6)
Costa Rica*	2018	1011·9 (992·4 to 1032·3)	715·7 (702·3 to 728·3)	217·3 (204·8 to 230·6)	51·5 (46·4 to 57·0)	27·5 (27·5 to 27·5)	1680·3 (1647·8 to 1714·0)	1188·3 (1166·1 to 1209·3)	360·8 (340·1 to 382·8)	85·5 (77·0 to 94·6)	45·7 (45·7 to 45·7)
Costa Rica*	2019	1082·9 (1061·2 to 1105·0)	780·8 (764·7 to 796·9)	220·0 (207·4 to 233·3)	53·5 (48·2 to 59·2)	28·5 (28·5 to 28·6)	1798·0 (1762·0 to 1834·9)	1296·6 (1269·8 to 1323·1)	365·3 (344·4 to 387·4)	88·8 (80·0 to 98·3)	47·4 (47·3 to 47·5)
Costa Rica*	2020	1110·8 (1089·6 to 1132·0)	815·0 (799·1 to 830·9)	201·3 (189·8 to 213·4)	50·3 (45·1 to 55·7)	44·2 (43·2 to 46·1)	1844·4 (1809·2 to 1879·6)	1353·3 (1326·8 to 1379·6)	334·2 (315·2 to 354·4)	83·5 (75·0 to 92·5)	73·3 (71·7 to 76·5)
Costa Rica*	2021	1032·2 (1010·3 to 1054·5)	743·4 (726·3 to 760·1)	196·2 (184·9 to 208·1)	50·4 (45·1 to 55·8)	42·2 (41·4 to 44·4)	1713·9 (1677·5 to 1751·0)	1234·4 (1206·0 to 1262·1)	325·7 (307·0 to 345·5)	83·7 (75·0 to 92·7)	70·1 (68·7 to 73·8)
Costa Rica*	2022	1034·9 (1008·6 to 1061·0)	752·5 (729·6 to 774·0)	197·1 (185·9 to 209·2)	52·2 (46·6 to 57·8)	33·2 (31·2 to 35·9)	1718·5 (1674·7 to 1761·8)	1249·4 (1211·5 to 1285·3)	327·3 (308·6 to 347·4)	86·6 (77·4 to 96·0)	55·1 (51·8 to 59·6)
Costa Rica*	2023	1061·6 (1031·5 to 1091·2)	774·7 (748·2 to 800·1)	198·4 (187·1 to 210·4)	54·0 (48·2 to 60·0)	34·4 (32·1 to 37·5)	1762·7 (1712·8 to 1811·8)	1286·3 (1242·3 to 1328·5)	329·4 (310·7 to 349·4)	89·7 (80·0 to 99·7)	57·2 (53·3 to 62·3)
Costa Rica*	2024	1084·7 (1050·9 to 1117·2)	793·3 (763·6 to 821·9)	199·6 (188·3 to 211·6)	56·0 (49·7 to 62·4)	35·9 (33·2 to 39·5)	1801·1 (1745·0 to 1855·0)	1317·2 (1267·9 to 1364·8)	331·4 (312·6 to 351·4)	92·9 (82·5 to 103·6)	59·6 (55·2 to 65·6)
Costa Rica*	2025	1107·8 (1068·9 to 1143·5)	812·3 (777·4 to 844·4)	200·2 (188·8 to 212·4)	57·9 (51·5 to 64·5)	37·4 (34·3 to 41·3)	1839·4 (1774·9 to 1898·7)	1348·7 (1290·9 to 1402·2)	332·5 (313·5 to 352·7)	96·1 (85·5 to 107·1)	62·0 (57·0 to 68·5)
Costa Rica*	2026	1130·8 (1087·0 to 1170·9)	831·2 (791·1 to 867·0)	200·8 (189·4 to 213·0)	59·9 (53·1 to 66·9)	38·9 (35·4 to 43·4)	1877·6 (1804·9 to 1944·2)	1380·1 (1313·6 to 1439·7)	333·5 (314·5 to 353·6)	99·4 (88·2 to 111·1)	64·5 (58·9 to 72·1)
Costa Rica*	2027	1154·0 (1106·4 to 1197·9)	850·3 (805·7 to 891·4)	201·6 (190·1 to 213·8)	62·0 (54·6 to 69·6)	40·2 (36·8 to 44·5)	1916·2 (1837·2 to 1989·0)	1411·9 (1337·8 to 1480·2)	334·7 (315·7 to 355·0)	102·9 (90·7 to 115·6)	66·7 (61·2 to 73·9)

Costa Rica*	2028	1177·7 (1126·4 to 1225·8)	869·7 (821·9 to 915·3)	202·4 (190·9 to 214·7)	64·1 (56·2 to 72·5)	41·5 (37·9 to 45·9)	1955·5 (1870·3 to 2035·4)	1444·1 (1364·7 to 1519·8)	336·1 (317·1 to 356·5)	106·5 (93·3 to 120·3)	68·9 (63·0 to 76·3)
Costa Rica*	2029	1201·9 (1150·1 to 1251·3)	889·5 (840·2 to 936·7)	203·2 (191·8 to 215·6)	66·3 (58·1 to 75·1)	42·8 (39·0 to 47·3)	1995·7 (1909·7 to 2077·7)	1476·9 (1395·1 to 1555·3)	337·4 (318·5 to 358·0)	110·1 (96·5 to 124·7)	71·1 (64·8 to 78·6)
Costa Rica*	2030	1226·1 (1172·3 to 1277·9)	909·3 (858·8 to 959·5)	204·1 (192·5 to 216·4)	68·6 (60·2 to 78·1)	44·1 (39·9 to 48·9)	2035·9 (1946·6 to 2121·9)	1509·9 (1426·1 to 1593·3)	338·9 (319·6 to 359·3)	113·9 (100·0 to 129·6)	73·3 (66·3 to 81·1)
Costa Rica*	2031	1249·3 (1190·7 to 1306·1)	928·0 (874·7 to 981·9)	204·9 (193·3 to 217·3)	70·9 (62·2 to 81·0)	45·5 (41·0 to 50·6)	2074·5 (1977·1 to 2168·6)	1540·8 (1452·3 to 1630·3)	340·3 (321·0 to 360·7)	117·8 (103·3 to 134·5)	75·6 (68·1 to 84·0)
Costa Rica*	2032	1272·5 (1209·2 to 1335·8)	946·4 (885·7 to 1005·8)	205·9 (194·2 to 218·3)	73·3 (64·2 to 83·9)	46·9 (41·9 to 52·8)	2112·9 (2007·8 to 2218·1)	1571·4 (1470·7 to 1670·2)	341·8 (322·5 to 362·5)	121·8 (106·6 to 139·4)	77·8 (69·6 to 87·7)
Costa Rica*	2033	1296·1 (1227·6 to 1364·7)	965·2 (899·4 to 1030·7)	207·0 (195·1 to 219·6)	75·8 (66·3 to 87·0)	48·1 (42·8 to 54·9)	2152·1 (2038·4 to 2266·0)	1602·6 (1493·3 to 1711·5)	343·7 (324·0 to 364·6)	125·9 (110·0 to 144·4)	79·9 (71·1 to 91·1)
Costa Rica*	2034	1319·1 (1245·9 to 1392·6)	983·3 (913·0 to 1053·1)	208·0 (196·1 to 220·8)	78·3 (68·3 to 90·0)	49·5 (43·9 to 57·2)	2190·3 (2068·8 to 2312·4)	1632·7 (1516·0 to 1748·6)	345·4 (325·6 to 366·6)	130·0 (113·5 to 149·4)	82·2 (72·8 to 94·9)
Costa Rica*	2035	1342·5 (1265·9 to 1422·5)	1001·5 (926·1 to 1080·0)	209·2 (197·1 to 222·1)	80·9 (70·5 to 93·3)	50·8 (44·7 to 59·8)	2229·1 (2102·0 to 2362·1)	1663·0 (1537·8 to 1793·3)	347·4 (327·4 to 368·7)	134·4 (117·0 to 155·0)	84·4 (74·3 to 99·3)
Costa Rica*	2036	1365·7 (1283·7 to 1453·6)	1019·6 (940·7 to 1107·0)	210·3 (198·2 to 223·2)	83·6 (72·6 to 96·7)	52·2 (45·7 to 62·2)	2267·8 (2131·6 to 2413·7)	1693·1 (1562·0 to 1838·1)	349·3 (329·2 to 370·6)	138·8 (120·5 to 160·6)	86·6 (75·9 to 103·2)
Costa Rica*	2037	1389·1 (1299·1 to 1481·2)	1037·9 (955·4 to 1126·4)	211·6 (199·4 to 224·6)	86·3 (75·0 to 100·3)	53·3 (46·1 to 64·9)	2306·5 (2157·1 to 2459·5)	1723·4 (1586·4 to 1870·3)	351·3 (331·2 to 372·9)	143·3 (124·5 to 166·5)	88·4 (76·5 to 107·7)
Costa Rica*	2038	1412·6 (1316·2 to 1516·1)	1056·4 (965·0 to 1157·0)	212·8 (200·4 to 225·9)	89·1 (77·0 to 104·1)	54·2 (45·5 to 67·0)	2345·5 (2185·4 to 2517·3)	1754·1 (1602·3 to 1921·2)	353·4 (332·8 to 375·1)	148·0 (127·8 to 172·8)	90·0 (75·6 to 111·2)
Costa Rica*	2039	1436·1 (1335·8 to 1545·9)	1075·0 (976·0 to 1181·1)	214·1 (201·6 to 227·3)	92·0 (78·8 to 108·2)	55·0 (0·0 to 70·6)	2384·5 (2218·1 to 2566·9)	1785·0 (1620·6 to 1961·2)	355·5 (334·7 to 377·4)	152·8 (130·9 to 179·6)	91·3 (0·0 to 117·2)
Costa Rica*	2040	1459·6 (1351·9 to 1576·8)	1094·2 (992·1 to 1208·8)	215·4 (202·8 to 228·7)	95·0 (81·1 to 112·1)	55·1 (0·0 to 73·6)	2423·6 (2244·8 to 2618·2)	1816·8 (1647·4 to 2007·1)	357·7 (336·8 to 379·8)	157·7 (134·6 to 186·1)	91·4 (0·0 to 122·2)
Costa Rica*	2041	1483·2 (1372·9 to 1602·1)	1112·8 (1014·2 to 1230·0)	216·6 (204·0 to 230·1)	98·0 (83·2 to 115·9)	55·6 (0·0 to 76·0)	2462·7 (2279·7 to 2660·2)	1847·8 (1684·1 to 2042·4)	359·7 (338·8 to 382·0)	162·8 (138·1 to 192·4)	92·4 (0·0 to 126·2)

Costa Rica*	2042	1506·8 (1393·5 to 1635·2)	1131·5 (1029·3 to 1259·6)	217·8 (205·1 to 231·2)	101·2 (85·5 to 120·6)	56·3 (0·0 to 79·7)	2502·0 (2313·8 to 2715·1)	1878·9 (1709·2 to 2091·5)	361·6 (340·6 to 383·9)	168·0 (142·0 to 200·2)	93·5 (0·0 to 132·3)
Costa Rica*	2043	1529·6 (1413·5 to 1668·3)	1149·6 (1046·4 to 1282·3)	218·8 (206·0 to 232·3)	104·4 (87·7 to 125·2)	56·9 (0·0 to 83·5)	2539·9 (2347·0 to 2770·1)	1908·8 (1737·4 to 2129·3)	363·3 (342·1 to 385·8)	173·4 (145·7 to 207·9)	94·4 (0·0 to 138·6)
Costa Rica*	2044	1551·1 (1430·1 to 1691·1)	1166·5 (1061·3 to 1303·9)	219·5 (206·7 to 233·2)	107·7 (89·8 to 130·1)	57·4 (0·0 to 87·7)	2575·6 (2374·6 to 2807·9)	1936·9 (1762·2 to 2165·0)	364·5 (343·2 to 387·2)	178·8 (149·2 to 216·0)	95·3 (0·0 to 145·5)
Costa Rica*	2045	1572·9 (1444·2 to 1718·0)	1183·6 (1074·6 to 1327·1)	220·1 (207·2 to 233·9)	111·1 (91·6 to 135·1)	58·1 (0·0 to 91·7)	2611·8 (2398·0 to 2852·7)	1965·4 (1784·3 to 2203·6)	365·5 (344·0 to 388·4)	184·4 (152·1 to 224·4)	96·5 (0·0 to 152·2)
Costa Rica*	2046	1596·0 (1464·5 to 1751·2)	1201·5 (1088·0 to 1359·0)	220·7 (207·7 to 234·5)	114·5 (93·4 to 140·9)	59·4 (0·0 to 96·3)	2650·2 (2431·7 to 2907·8)	1995·0 (1806·5 to 2256·6)	366·5 (344·9 to 389·4)	190·1 (155·2 to 234·0)	98·6 (0·0 to 159·9)
Costa Rica*	2047	1617·7 (1482·3 to 1784·5)	1217·8 (1100·4 to 1374·6)	221·2 (208·1 to 235·1)	118·0 (95·7 to 146·6)	60·7 (0·0 to 101·3)	2686·1 (2461·3 to 2963·1)	2022·1 (1827·2 to 2282·5)	367·3 (345·6 to 390·3)	195·9 (158·9 to 243·5)	100·8 (0·0 to 168·1)
Costa Rica*	2048	1639·2 (1498·2 to 1810·4)	1234·1 (1114·4 to 1394·9)	221·7 (208·5 to 235·7)	121·6 (98·1 to 152·0)	61·9 (0·0 to 105·7)	2721·8 (2487·7 to 3006·1)	2049·1 (1850·4 to 2316·2)	368·0 (346·2 to 391·4)	201·8 (162·9 to 252·4)	102·8 (0·0 to 175·5)
Costa Rica*	2049	1660·4 (1514·9 to 1841·8)	1250·3 (1124·5 to 1425·1)	221·9 (208·7 to 236·1)	125·2 (100·1 to 157·7)	62·9 (0·0 to 110·8)	2757·0 (2515·4 to 3058·2)	2076·1 (1867·2 to 2366·3)	368·5 (346·5 to 392·0)	207·8 (166·2 to 261·9)	104·5 (0·0 to 184·0)
Costa Rica*	2050	1681·7 (1530·4 to 1869·5)	1265·8 (1138·8 to 1446·1)	222·2 (208·9 to 236·4)	128·9 (102·3 to 163·4)	64·7 (0·0 to 117·1)	2792·3 (2541·2 to 3104·2)	2101·9 (1890·9 to 2401·2)	369·0 (346·8 to 392·6)	214·0 (169·8 to 271·4)	107·5 (0·0 to 194·5)
Cuba*	1995	228·0 (197·3 to 263·7)	189·5 (160·3 to 223·0)	38·4 (31·3 to 46·7)	0·0 (0·0 to 0·0)	0·0 (0·0 to 0·0)	557·7 (482·7 to 645·2)	463·7 (392·1 to 545·7)	93·9 (76·5 to 114·3)	0·0 (0·0 to 0·1)	0·0 (0·0 to 0·0)
Cuba*	1996	249·6 (217·2 to 284·8)	208·8 (177·7 to 244·9)	40·3 (33·0 to 48·1)	0·0 (0·0 to 0·0)	0·5 (0·5 to 0·5)	610·7 (531·4 to 696·8)	510·8 (434·7 to 599·2)	98·6 (80·7 to 117·7)	0·0 (0·0 to 0·1)	1·3 (1·3 to 1·3)
Cuba*	1997	263·1 (231·5 to 299·4)	220·1 (189·1 to 255·3)	42·4 (35·0 to 50·2)	0·0 (0·0 to 0·0)	0·5 (0·5 to 0·5)	643·6 (566·3 to 732·5)	538·5 (462·7 to 624·7)	103·7 (85·7 to 122·9)	0·1 (0·0 to 0·1)	1·3 (1·3 to 1·3)
Cuba*	1998	272·6 (241·3 to 307·6)	227·3 (196·9 to 261·2)	45·0 (37·7 to 52·6)	0·0 (0·0 to 0·0)	0·3 (0·3 to 0·3)	666·9 (590·4 to 752·4)	556·0 (481·7 to 639·1)	110·0 (92·2 to 128·7)	0·1 (0·0 to 0·1)	0·8 (0·8 to 0·8)
Cuba*	1999	294·7 (262·2 to 330·0)	245·2 (213·7 to 279·2)	48·9 (41·3 to 57·0)	0·0 (0·0 to 0·0)	0·6 (0·6 to 0·6)	721·1 (641·4 to 807·3)	599·9 (522·9 to 682·9)	119·6 (101·0 to 139·5)	0·1 (0·0 to 0·1)	1·5 (1·5 to 1·5)

Cuba*	2000	325·8 (291·7 to 362·2)	270·0 (238·1 to 305·9)	55·4 (47·2 to 64·7)	0·0 (0·0 to 0·0)	0·4 (0·4 to 0·4)	797·0 (713·6 to 886·0)	660·5 (582·4 to 748·2)	135·5 (115·5 to 158·4)	0·1 (0·0 to 0·1)	1·0 (1·0 to 1·0)
Cuba*	2001	346·6 (312·5 to 384·0)	283·6 (251·6 to 319·6)	62·5 (53·5 to 73·2)	0·0 (0·0 to 0·0)	0·5 (0·5 to 0·5)	848·0 (764·4 to 939·3)	693·9 (615·5 to 781·9)	153·0 (130·9 to 179·0)	0·1 (0·0 to 0·1)	1·1 (1·1 to 1·1)
Cuba*	2002	371·8 (336·6 to 410·5)	302·8 (268·3 to 341·1)	68·4 (58·9 to 78·9)	0·0 (0·0 to 0·0)	0·5 (0·5 to 0·5)	909·5 (823·5 to 1004·2)	740·8 (656·4 to 834·4)	167·3 (144·1 to 193·0)	0·1 (0·0 to 0·1)	1·3 (1·3 to 1·3)
Cuba*	2003	398·7 (361·9 to 438·1)	324·8 (289·3 to 364·4)	72·6 (63·0 to 83·0)	0·0 (0·0 to 0·0)	1·3 (1·3 to 1·3)	975·5 (885·4 to 1071·8)	794·6 (707·8 to 891·6)	177·6 (154·2 to 203·1)	0·1 (0·0 to 0·1)	3·2 (3·2 to 3·2)
Cuba*	2004	431·8 (393·5 to 473·6)	356·0 (318·4 to 396·1)	74·4 (64·6 to 85·0)	0·0 (0·0 to 0·1)	1·4 (1·4 to 1·4)	1056·3 (962·6 to 1158·6)	870·9 (778·9 to 969·1)	182·0 (158·1 to 207·9)	0·1 (0·0 to 0·1)	3·3 (3·3 to 3·3)
Cuba*	2005	538·3 (495·6 to 585·9)	458·5 (416·8 to 505·1)	78·9 (68·8 to 91·1)	0·0 (0·0 to 0·1)	0·8 (0·8 to 0·8)	1316·9 (1212·5 to 1433·3)	1121·7 (1019·6 to 1235·7)	193·0 (168·3 to 223·0)	0·1 (0·0 to 0·1)	2·1 (2·1 to 2·1)
Cuba*	2006	636·6 (593·8 to 689·2)	554·9 (510·9 to 607·8)	80·7 (70·5 to 92·0)	0·0 (0·0 to 0·1)	0·9 (0·9 to 0·9)	1557·4 (1452·6 to 1686·1)	1357·5 (1249·8 to 1486·9)	197·5 (172·5 to 225·0)	0·1 (0·0 to 0·2)	2·3 (2·3 to 2·3)
Cuba*	2007	763·6 (715·4 to 821·2)	679·7 (632·4 to 733·5)	82·3 (72·0 to 94·1)	0·0 (0·0 to 0·1)	1·6 (1·6 to 1·6)	1868·1 (1750·3 to 2009·0)	1662·9 (1547·1 to 1794·5)	201·2 (176·1 to 230·2)	0·1 (0·0 to 0·2)	3·9 (3·9 to 3·9)
Cuba*	2008	907·2 (855·9 to 961·8)	820·8 (769·9 to 876·0)	85·3 (75·6 to 96·6)	0·0 (0·0 to 0·1)	1·1 (1·1 to 1·1)	2219·5 (2093·8 to 2353·0)	2008·1 (1883·5 to 2143·0)	208·6 (184·9 to 236·3)	0·1 (0·0 to 0·2)	2·6 (2·6 to 2·6)
Cuba*	2009	939·6 (889·3 to 994·3)	851·5 (801·9 to 904·7)	86·2 (76·7 to 97·6)	0·0 (0·0 to 0·1)	1·9 (1·9 to 1·9)	2298·8 (2175·5 to 2432·6)	2083·1 (1961·7 to 2213·3)	211·0 (187·7 to 238·8)	0·1 (0·0 to 0·2)	4·6 (4·6 to 4·6)
Cuba*	2010	915·8 (866·7 to 970·2)	825·0 (777·3 to 877·9)	89·0 (78·9 to 100·5)	0·0 (0·0 to 0·1)	1·8 (1·8 to 1·8)	2240·4 (2120·3 to 2373·5)	2018·2 (1901·7 to 2147·8)	217·8 (193·0 to 245·9)	0·1 (0·0 to 0·2)	4·3 (4·3 to 4·3)
Cuba*	2011	913·8 (862·6 to 968·4)	815·7 (765·3 to 867·3)	96·6 (85·6 to 108·8)	0·0 (0·0 to 0·1)	1·4 (1·4 to 1·4)	2235·4 (2110·3 to 2369·1)	1995·6 (1872·3 to 2121·8)	236·4 (209·3 to 266·2)	0·1 (0·0 to 0·2)	3·3 (3·3 to 3·3)
Cuba*	2012	942·8 (888·4 to 999·8)	838·2 (786·1 to 894·6)	103·5 (92·1 to 115·8)	0·0 (0·0 to 0·1)	1·1 (1·1 to 1·1)	2306·6 (2173·4 to 2445·9)	2050·5 (1923·2 to 2188·5)	253·2 (225·3 to 283·3)	0·1 (0·0 to 0·2)	2·7 (2·7 to 2·7)
Cuba*	2013	975·9 (922·1 to 1032·5)	866·7 (815·4 to 924·0)	107·9 (96·7 to 119·9)	0·0 (0·0 to 0·1)	1·2 (1·2 to 1·2)	2387·6 (2255·8 to 2525·9)	2120·4 (1994·9 to 2260·5)	264·1 (236·5 to 293·2)	0·1 (0·0 to 0·2)	3·0 (3·0 to 3·0)

Cuba*	2014	1045·6 (990·0 to 1098·8)	934·3 (880·8 to 985·1)	110·7 (98·9 to 122·6)	0·0 (0·0 to 0·1)	0·7 (0·7 to 0·7)	2558·1 (2421·8 to 2688·0)	2285·6 (2154·8 to 2409·9)	270·8 (241·8 to 300·0)	0·1 (0·0 to 0·2)	1·6 (1·6 to 1·6)
Cuba*	2015	1149·1 (1094·5 to 1203·5)	1031·3 (977·0 to 1082·1)	116·7 (104·7 to 128·3)	0·0 (0·0 to 0·1)	1·1 (1·1 to 1·1)	2811·3 (2677·7 to 2944·4)	2523·1 (2390·1 to 2647·4)	285·5 (256·1 to 314·0)	0·1 (0·0 to 0·2)	2·7 (2·7 to 2·7)
Cuba*	2016	1148·5 (1094·5 to 1206·1)	1029·2 (975·8 to 1084·4)	118·3 (106·2 to 130·3)	0·0 (0·0 to 0·1)	0·9 (0·9 to 0·9)	2809·7 (2677·7 to 2950·6)	2517·9 (2387·1 to 2652·9)	289·5 (259·7 to 318·7)	0·1 (0·0 to 0·2)	2·2 (2·2 to 2·2)
Cuba*	2017	1155·4 (1096·0 to 1218·5)	1034·1 (976·9 to 1094·2)	120·3 (107·3 to 134·8)	0·0 (0·0 to 0·1)	1·0 (1·0 to 1·0)	2826·7 (2681·2 to 2980·9)	2529·8 (2390·0 to 2676·8)	294·4 (262·6 to 329·7)	0·1 (0·0 to 0·2)	2·4 (2·4 to 2·4)
Cuba*	2018	1140·7 (1065·6 to 1220·0)	1017·6 (943·2 to 1095·0)	121·7 (106·9 to 138·9)	0·0 (0·0 to 0·1)	1·4 (1·4 to 1·4)	2790·7 (2607·0 to 2984·5)	2489·4 (2307·5 to 2678·9)	297·8 (261·6 to 339·9)	0·1 (0·0 to 0·2)	3·4 (3·4 to 3·4)
Cuba*	2019	1170·1 (1090·3 to 1255·2)	1044·7 (968·3 to 1127·9)	123·9 (108·8 to 141·5)	0·0 (0·0 to 0·1)	1·4 (1·4 to 1·5)	2862·6 (2667·2 to 3070·6)	2555·8 (2368·8 to 2759·2)	303·1 (266·2 to 346·1)	0·1 (0·0 to 0·2)	3·5 (3·5 to 3·6)
Cuba*	2020	1171·6 (1085·5 to 1262·0)	1054·0 (970·0 to 1141·2)	115·5 (101·5 to 131·8)	0·0 (0·0 to 0·1)	2·1 (2·0 to 2·2)	2866·1 (2655·6 to 3087·3)	2578·5 (2373·0 to 2791·7)	282·5 (248·3 to 322·5)	0·1 (0·0 to 0·2)	5·1 (4·9 to 5·3)
Cuba*	2021	1115·5 (1033·5 to 1201·6)	1003·6 (923·6 to 1086·6)	109·7 (96·4 to 125·2)	0·0 (0·0 to 0·1)	2·2 (2·1 to 2·3)	2729·0 (2528·5 to 2939·7)	2455·2 (2259·6 to 2658·3)	268·4 (235·9 to 306·4)	0·1 (0·0 to 0·2)	5·3 (5·2 to 5·5)
Cuba*	2022	1084·6 (1007·4 to 1165·5)	972·7 (897·2 to 1053·3)	110·1 (96·7 to 125·8)	0·0 (0·0 to 0·1)	1·7 (1·6 to 1·8)	2653·3 (2464·5 to 2851·2)	2379·6 (2194·9 to 2576·8)	269·4 (236·6 to 307·7)	0·1 (0·0 to 0·2)	4·2 (4·0 to 4·5)
Cuba*	2023	1107·9 (1027·9 to 1192·9)	995·5 (918·2 to 1079·8)	110·5 (97·0 to 126·1)	0·1 (0·0 to 0·1)	1·8 (1·7 to 2·0)	2710·3 (2514·6 to 2918·2)	2435·4 (2246·3 to 2641·6)	270·3 (237·3 to 308·6)	0·1 (0·0 to 0·3)	4·5 (4·2 to 4·8)
Cuba*	2024	1132·1 (1049·0 to 1218·7)	1019·3 (937·1 to 1107·1)	110·8 (97·3 to 126·5)	0·1 (0·0 to 0·1)	1·9 (1·8 to 2·1)	2769·6 (2566·3 to 2981·5)	2493·7 (2292·6 to 2708·5)	271·1 (237·9 to 309·5)	0·1 (0·0 to 0·3)	4·7 (4·4 to 5·1)
Cuba*	2025	1156·1 (1070·0 to 1245·9)	1043·1 (956·9 to 1132·6)	110·9 (97·4 to 126·7)	0·1 (0·0 to 0·1)	2·0 (1·9 to 2·2)	2828·4 (2617·6 to 3048·0)	2551·9 (2340·9 to 2770·8)	271·4 (238·3 to 309·9)	0·1 (0·0 to 0·3)	5·0 (4·6 to 5·4)
Cuba*	2026	1180·5 (1089·7 to 1273·1)	1067·4 (976·8 to 1158·2)	110·9 (97·4 to 126·7)	0·1 (0·0 to 0·1)	2·2 (2·0 to 2·4)	2888·1 (2665·9 to 3114·6)	2611·3 (2389·8 to 2833·4)	271·3 (238·2 to 309·9)	0·1 (0·0 to 0·3)	5·3 (4·9 to 5·8)
Cuba*	2027	1206·6 (1111·8 to 1300·4)	1093·4 (997·9 to 1188·4)	110·8 (97·3 to 126·6)	0·1 (0·0 to 0·1)	2·3 (2·1 to 2·5)	2951·8 (2719·8 to 3181·2)	2674·9 (2441·4 to 2907·3)	271·1 (238·0 to 309·7)	0·2 (0·0 to 0·3)	5·7 (5·2 to 6·2)

Cuba*	2028	1234·2 (1137·1 to 1334·1)	1121·2 (1020·0 to 1221·0)	110·5 (97·0 to 126·2)	0·1 (0·0 to 0·1)	2·5 (2·3 to 2·8)	3019·4 (2781·7 to 3263·7)	2742·8 (2495·4 to 2987·0)	270·4 (237·3 to 308·9)	0·2 (0·0 to 0·3)	6·1 (5·5 to 6·7)
Cuba*	2029	1263·8 (1159·9 to 1364·9)	1151·0 (1045·1 to 1253·2)	110·0 (96·6 to 125·6)	0·1 (0·0 to 0·1)	2·7 (2·4 to 3·0)	3091·8 (2837·6 to 3339·2)	2815·9 (2556·7 to 3065·7)	269·2 (236·2 to 307·4)	0·2 (0·0 to 0·3)	6·6 (5·9 to 7·3)
Cuba*	2030	1292·9 (1182·2 to 1400·9)	1180·5 (1070·9 to 1285·4)	109·4 (95·9 to 124·9)	0·1 (0·0 to 0·1)	2·9 (2·6 to 3·3)	3162·9 (2892·1 to 3427·2)	2888·1 (2619·9 to 3144·5)	267·6 (234·7 to 305·5)	0·2 (0·0 to 0·3)	7·1 (6·3 to 8·0)
Cuba*	2031	1322·0 (1203·9 to 1434·6)	1210·0 (1094·8 to 1319·1)	108·8 (95·4 to 124·2)	0·1 (0·0 to 0·1)	3·1 (2·8 to 3·6)	3234·1 (2945·3 to 3509·6)	2960·2 (2678·4 to 3227·1)	266·1 (233·3 to 303·8)	0·2 (0·0 to 0·4)	7·7 (6·8 to 8·8)
Cuba*	2032	1350·9 (1230·9 to 1469·3)	1239·0 (1121·2 to 1355·1)	108·4 (95·0 to 123·8)	0·1 (0·0 to 0·2)	3·4 (3·0 to 3·9)	3304·8 (3011·2 to 3594·6)	3031·2 (2742·9 to 3315·1)	265·2 (232·3 to 302·8)	0·2 (0·0 to 0·4)	8·2 (7·3 to 9·5)
Cuba*	2033	1378·8 (1252·8 to 1498·9)	1267·0 (1141·3 to 1390·5)	108·1 (94·6 to 123·5)	0·1 (0·0 to 0·2)	3·6 (3·2 to 4·2)	3373·0 (3064·8 to 3666·9)	3099·6 (2792·1 to 3401·8)	264·4 (231·5 to 302·1)	0·2 (0·0 to 0·4)	8·8 (7·7 to 10·3)
Cuba*	2034	1405·1 (1273·4 to 1530·5)	1293·5 (1158·9 to 1421·3)	107·7 (94·2 to 123·1)	0·1 (0·0 to 0·2)	3·9 (3·4 to 4·6)	3437·4 (3115·2 to 3744·2)	3164·3 (2835·1 to 3477·0)	263·4 (230·5 to 301·1)	0·2 (0·0 to 0·4)	9·5 (8·3 to 11·2)
Cuba*	2035	1431·6 (1293·7 to 1562·8)	1319·9 (1180·7 to 1448·7)	107·4 (93·9 to 122·7)	0·1 (0·0 to 0·2)	4·2 (3·6 to 5·0)	3502·2 (3164·8 to 3823·4)	3229·1 (2888·4 to 3544·2)	262·7 (229·7 to 300·2)	0·2 (0·0 to 0·4)	10·2 (8·8 to 12·1)
Cuba*	2036	1456·1 (1316·2 to 1592·9)	1344·5 (1200·0 to 1479·8)	107·1 (93·6 to 122·4)	0·1 (0·0 to 0·2)	4·5 (3·9 to 5·4)	3562·3 (3220·0 to 3897·0)	3289·1 (2935·7 to 3620·3)	262·0 (229·1 to 299·4)	0·2 (0·1 to 0·4)	11·0 (9·5 to 13·2)
Cuba*	2037	1478·8 (1331·9 to 1618·9)	1367·0 (1218·8 to 1504·3)	106·9 (93·4 to 122·1)	0·1 (0·0 to 0·2)	4·8 (4·1 to 5·8)	3617·8 (3258·3 to 3960·6)	3344·3 (2981·6 to 3680·1)	261·5 (228·6 to 298·8)	0·2 (0·1 to 0·5)	11·7 (10·1 to 14·3)
Cuba*	2038	1501·1 (1348·3 to 1647·2)	1389·1 (1237·0 to 1530·8)	106·8 (93·3 to 122·0)	0·1 (0·0 to 0·2)	5·1 (4·3 to 6·3)	3672·4 (3298·6 to 4029·6)	3398·2 (3026·3 to 3745·0)	261·4 (228·4 to 298·5)	0·2 (0·1 to 0·5)	12·5 (10·6 to 15·4)
Cuba*	2039	1524·0 (1363·4 to 1676·3)	1411·5 (1254·3 to 1560·3)	106·9 (93·4 to 122·1)	0·1 (0·0 to 0·2)	5·4 (4·6 to 6·7)	3728·4 (3335·3 to 4101·0)	3453·2 (3068·6 to 3817·1)	261·6 (228·5 to 298·7)	0·2 (0·1 to 0·5)	13·3 (11·2 to 16·4)
Cuba*	2040	1546·1 (1382·3 to 1704·1)	1433·1 (1269·6 to 1586·1)	107·1 (93·5 to 122·3)	0·1 (0·0 to 0·2)	5·8 (4·9 to 7·2)	3782·4 (3381·7 to 4168·9)	3505·9 (3105·9 to 3880·2)	262·0 (228·8 to 299·1)	0·3 (0·1 to 0·5)	14·2 (11·9 to 17·7)
Cuba*	2041	1566·7 (1396·2 to 1727·4)	1453·1 (1282·0 to 1611·6)	107·4 (93·7 to 122·5)	0·1 (0·0 to 0·2)	6·1 (5·1 to 7·7)	3832·8 (3415·6 to 4225·9)	3554·9 (3136·4 to 3942·7)	262·6 (229·3 to 299·8)	0·3 (0·1 to 0·5)	15·0 (12·5 to 18·9)

Cuba*	2042	1588·1 (1413·9 to 1759·9)	1473·7 (1298·7 to 1638·4)	107·9 (94·2 to 123·1)	0·1 (0·0 to 0·2)	6·5 (5·4 to 8·2)	3885·2 (3458·9 to 4305·4)	3605·2 (3177·1 to 4008·3)	263·9 (230·4 to 301·2)	0·3 (0·1 to 0·6)	15·8 (13·2 to 20·1)
Cuba*	2043	1609·5 (1431·4 to 1777·9)	1494·0 (1317·7 to 1660·6)	108·7 (94·9 to 124·0)	0·1 (0·0 to 0·2)	6·7 (5·6 to 8·6)	3937·5 (3501·7 to 4349·5)	3654·9 (3223·7 to 4062·4)	265·8 (232·1 to 303·3)	0·3 (0·1 to 0·6)	16·5 (13·6 to 21·1)
Cuba*	2044	1630·9 (1450·3 to 1803·6)	1514·1 (1332·4 to 1685·6)	109·7 (95·7 to 125·1)	0·1 (0·0 to 0·3)	7·0 (5·7 to 9·0)	3989·9 (3547·9 to 4412·3)	3704·1 (3259·6 to 4123·6)	268·3 (234·2 to 306·1)	0·3 (0·1 to 0·6)	17·1 (14·1 to 22·0)
Cuba*	2045	1651·9 (1469·6 to 1828·8)	1533·7 (1348·4 to 1708·0)	110·8 (96·7 to 126·3)	0·1 (0·0 to 0·3)	7·3 (5·9 to 9·4)	4041·2 (3595·3 to 4474·1)	3752·1 (3298·8 to 4178·5)	271·0 (236·6 to 309·1)	0·3 (0·1 to 0·7)	17·7 (14·5 to 23·0)
Cuba*	2046	1672·1 (1485·0 to 1853·3)	1552·6 (1366·2 to 1730·5)	111·8 (97·6 to 127·5)	0·1 (0·0 to 0·3)	7·5 (6·1 to 9·8)	4090·6 (3633·0 to 4533·9)	3798·3 (3342·2 to 4233·5)	273·5 (238·8 to 311·9)	0·3 (0·1 to 0·7)	18·5 (14·9 to 23·9)
Cuba*	2047	1691·2 (1495·4 to 1876·9)	1570·5 (1379·4 to 1752·7)	112·6 (98·4 to 128·4)	0·1 (0·0 to 0·3)	7·9 (6·3 to 10·3)	4137·4 (3658·4 to 4591·7)	3842·2 (3374·7 to 4287·7)	275·5 (240·7 to 314·2)	0·4 (0·1 to 0·7)	19·3 (15·5 to 25·2)
Cuba*	2048	1709·7 (1514·8 to 1895·3)	1588·0 (1390·5 to 1775·1)	113·2 (99·0 to 129·1)	0·1 (0·0 to 0·3)	8·3 (6·6 to 10·8)	4182·6 (3705·8 to 4636·6)	3884·9 (3401·7 to 4342·7)	277·0 (242·1 to 315·9)	0·4 (0·1 to 0·8)	20·3 (16·2 to 26·5)
Cuba*	2049	1727·9 (1532·5 to 1919·5)	1605·2 (1409·9 to 1795·6)	113·7 (99·5 to 129·7)	0·2 (0·0 to 0·3)	8·8 (7·0 to 11·6)	4227·1 (3749·0 to 4695·8)	3927·0 (3449·2 to 4392·8)	278·2 (243·3 to 317·4)	0·4 (0·1 to 0·8)	21·5 (17·1 to 28·5)
Cuba*	2050	1744·6 (1549·3 to 1935·7)	1621·0 (1422·7 to 1815·7)	114·1 (99·8 to 130·1)	0·2 (0·0 to 0·3)	9·3 (7·3 to 12·5)	4267·9 (3790·3 to 4735·4)	3965·6 (3480·6 to 4441·9)	279·0 (244·2 to 318·3)	0·4 (0·1 to 0·8)	22·9 (17·9 to 30·6)
Cyprus	1995	718·4 (671·1 to 766·9)	250·7 (227·4 to 276·2)	453·7 (416·6 to 494·5)	14·0 (10·0 to 18·8)	0·0 (0·0 to 0·0)	1070·6 (1000·1 to 1142·8)	373·6 (338·9 to 411·6)	676·1 (620·8 to 736·9)	20·9 (15·0 to 28·0)	0·0 (0·0 to 0·0)
Cyprus	1996	755·7 (717·1 to 794·3)	267·3 (247·6 to 289·4)	472·4 (442·3 to 504·6)	16·1 (11·9 to 22·0)	0·0 (0·0 to 0·0)	1126·2 (1068·7 to 1183·7)	398·3 (368·9 to 431·2)	704·0 (659·2 to 752·0)	24·0 (17·7 to 32·7)	0·0 (0·0 to 0·0)
Cyprus	1997	797·7 (763·3 to 834·9)	294·4 (274·2 to 315·8)	484·7 (457·8 to 513·2)	18·6 (14·1 to 24·9)	0·0 (0·0 to 0·0)	1188·8 (1137·5 to 1244·1)	438·7 (408·6 to 470·7)	722·4 (682·2 to 764·9)	27·8 (21·0 to 37·1)	0·0 (0·0 to 0·0)
Cyprus	1998	842·2 (807·8 to 879·1)	330·3 (310·6 to 350·7)	490·1 (462·2 to 518·7)	21·8 (16·8 to 28·2)	0·0 (0·0 to 0·0)	1255·1 (1203·8 to 1310·0)	492·3 (462·9 to 522·6)	730·4 (688·8 to 773·0)	32·4 (25·0 to 42·0)	0·0 (0·0 to 0·0)
Cyprus	1999	885·0 (851·9 to 921·1)	358·1 (339·1 to 378·3)	497·7 (470·2 to 526·0)	29·3 (23·3 to 37·2)	0·0 (0·0 to 0·0)	1318·9 (1269·5 to 1372·7)	533·7 (505·4 to 563·8)	741·7 (700·6 to 783·9)	43·6 (34·7 to 55·4)	0·0 (0·0 to 0·0)

Cyprus	2000	934·0 (901·7 to 967·8)	388·1 (369·5 to 407·7)	516·6 (490·9 to 542·9)	29·3 (23·8 to 35·9)	0·0 (0·0 to 0·0)	1391·8 (1343·7 to 1442·2)	578·3 (550·7 to 607·5)	769·8 (731·6 to 809·1)	43·7 (35·5 to 53·5)	0·0 (0·0 to 0·0)
Cyprus	2001	973·7 (943·7 to 1007·0)	413·9 (395·3 to 433·0)	527·6 (503·0 to 552·8)	32·2 (26·6 to 39·0)	0·0 (0·0 to 0·0)	1451·0 (1406·3 to 1500·6)	616·8 (589·1 to 645·3)	786·2 (749·6 to 823·8)	48·0 (39·6 to 58·1)	0·0 (0·0 to 0·0)
Cyprus	2002	1010·4 (977·4 to 1044·9)	439·7 (422·0 to 458·2)	530·2 (505·5 to 556·2)	40·6 (34·4 to 47·1)	0·0 (0·0 to 0·0)	1505·8 (1456·6 to 1557·2)	655·2 (628·9 to 682·8)	790·1 (753·4 to 828·9)	60·5 (51·3 to 70·1)	0·0 (0·0 to 0·0)
Cyprus	2003	1048·9 (1015·7 to 1084·9)	455·9 (436·6 to 476·1)	523·6 (497·9 to 551·7)	69·3 (60·9 to 78·4)	0·0 (0·0 to 0·0)	1563·1 (1513·7 to 1616·8)	679·4 (650·7 to 709·4)	780·4 (742·0 to 822·2)	103·3 (90·8 to 116·8)	0·0 (0·0 to 0·0)
Cyprus	2004	1037·7 (1004·7 to 1071·5)	438·7 (420·1 to 457·6)	515·2 (489·7 to 541·1)	83·8 (75·8 to 92·8)	0·0 (0·0 to 0·0)	1546·5 (1497·3 to 1596·9)	653·7 (626·0 to 681·9)	767·8 (729·7 to 806·4)	124·9 (112·9 to 138·3)	0·0 (0·0 to 0·0)
Cyprus	2005	1065·5 (1032·3 to 1099·8)	450·5 (431·5 to 470·0)	525·7 (500·1 to 553·2)	89·4 (81·3 to 98·4)	0·0 (0·0 to 0·0)	1587·9 (1538·4 to 1639·0)	671·3 (643·0 to 700·4)	783·4 (745·3 to 824·4)	133·2 (121·2 to 146·7)	0·0 (0·0 to 0·0)
Cyprus	2006	1095·8 (1062·5 to 1130·4)	464·8 (446·8 to 484·1)	542·0 (515·4 to 568·3)	89·0 (80·1 to 97·6)	0·0 (0·0 to 0·0)	1633·1 (1583·4 to 1684·5)	692·7 (665·9 to 721·4)	807·8 (768·1 to 847·0)	132·6 (119·4 to 145·5)	0·0 (0·0 to 0·0)
Cyprus	2007	1139·4 (1106·1 to 1175·3)	482·6 (464·4 to 500·5)	571·5 (546·0 to 598·2)	85·3 (77·5 to 93·9)	0·0 (0·0 to 0·0)	1698·0 (1648·3 to 1751·5)	719·1 (692·1 to 745·8)	851·7 (813·7 to 891·4)	127·2 (115·6 to 139·9)	0·0 (0·0 to 0·0)
Cyprus	2008	1226·1 (1193·4 to 1261·2)	521·1 (501·4 to 539·1)	616·3 (591·5 to 644·2)	88·7 (80·5 to 97·3)	0·0 (0·0 to 0·0)	1827·2 (1778·5 to 1879·4)	776·6 (747·2 to 803·4)	918·5 (881·5 to 960·0)	132·1 (119·9 to 145·0)	0·0 (0·0 to 0·0)
Cyprus	2009	1289·4 (1255·7 to 1324·5)	565·6 (547·0 to 585·4)	623·0 (595·9 to 651·4)	100·9 (91·9 to 110·1)	0·0 (0·0 to 0·0)	1921·5 (1871·2 to 1973·8)	842·8 (815·2 to 872·4)	928·4 (888·0 to 970·7)	150·3 (137·0 to 164·1)	0·0 (0·0 to 0·0)
Cyprus	2010	1299·8 (1263·8 to 1335·8)	591·4 (572·5 to 610·1)	585·2 (556·7 to 614·3)	123·2 (114·1 to 132·6)	0·0 (0·0 to 0·0)	1937·1 (1883·4 to 1990·7)	881·3 (853·1 to 909·2)	872·1 (829·6 to 915·5)	183·7 (170·0 to 197·6)	0·0 (0·0 to 0·0)
Cyprus	2011	1280·4 (1247·6 to 1316·7)	598·9 (580·2 to 618·4)	549·1 (521·5 to 577·7)	132·4 (122·9 to 142·2)	0·0 (0·0 to 0·0)	1908·0 (1859·2 to 1962·2)	892·5 (864·7 to 921·6)	818·3 (777·2 to 860·9)	197·3 (183·1 to 212·0)	0·0 (0·0 to 0·0)
Cyprus	2012	1225·9 (1191·7 to 1260·0)	569·5 (551·3 to 588·2)	523·4 (497·0 to 549·8)	133·0 (123·7 to 142·2)	0·0 (0·0 to 0·0)	1826·9 (1776·0 to 1877·8)	848·7 (821·6 to 876·6)	780·0 (740·6 to 819·3)	198·1 (184·3 to 212·0)	0·0 (0·0 to 0·0)
Cyprus	2013	1166·3 (1134·9 to 1201·4)	527·0 (508·6 to 545·9)	508·8 (483·9 to 537·2)	130·6 (121·6 to 140·4)	0·0 (0·0 to 0·0)	1738·1 (1691·3 to 1790·4)	785·4 (758·0 to 813·5)	758·2 (721·2 to 800·5)	194·6 (181·2 to 209·2)	0·0 (0·0 to 0·0)

Cyprus	2014	1169·0 (1135·8 to 1205·1)	514·3 (494·9 to 533·4)	518·7 (492·4 to 546·9)	136·0 (126·5 to 145·7)	0·0 (0·0 to 0·0)	1742·0 (1692·7 to 1795·9)	766·4 (737·5 to 794·9)	773·0 (733·8 to 815·0)	202·7 (188·5 to 217·1)	0·0 (0·0 to 0·0)
Cyprus	2015	1156·2 (1123·5 to 1191·2)	486·4 (467·4 to 504·3)	524·9 (498·6 to 553·6)	144·8 (135·8 to 154·3)	0·0 (0·0 to 0·0)	1723·0 (1674·4 to 1775·1)	724·9 (696·6 to 751·6)	782·3 (743·0 to 825·0)	215·8 (202·4 to 229·9)	0·0 (0·0 to 0·0)
Cyprus	2016	1181·5 (1148·8 to 1214·7)	487·0 (470·3 to 505·5)	542·9 (516·2 to 571·2)	151·6 (142·0 to 160·5)	0·0 (0·0 to 0·0)	1760·7 (1712·0 to 1810·2)	725·8 (700·8 to 753·3)	809·0 (769·3 to 851·2)	225·9 (211·7 to 239·2)	0·0 (0·0 to 0·0)
Cyprus	2017	1203·3 (1168·8 to 1240·6)	495·5 (477·0 to 516·3)	553·0 (525·3 to 581·1)	154·8 (144·7 to 164·7)	0·0 (0·0 to 0·0)	1793·3 (1741·8 to 1848·8)	738·5 (710·9 to 769·4)	824·1 (782·9 to 866·0)	230·7 (215·6 to 245·4)	0·0 (0·0 to 0·0)
Cyprus	2018	1276·5 (1227·8 to 1325·0)	544·8 (519·2 to 571·9)	573·4 (536·4 to 612·2)	158·3 (146·2 to 172·6)	0·0 (0·0 to 0·0)	1902·3 (1829·7 to 1974·6)	811·9 (773·7 to 852·3)	854·6 (799·4 to 912·4)	235·8 (217·9 to 257·2)	0·0 (0·0 to 0·0)
Cyprus	2019	1261·9 (1211·7 to 1313·5)	512·0 (483·4 to 542·7)	584·7 (546·7 to 624·5)	165·3 (152·3 to 180·2)	0·0 (0·0 to 0·0)	1880·6 (1805·7 to 1957·4)	763·0 (720·4 to 808·7)	871·3 (814·6 to 930·7)	246·3 (227·0 to 268·5)	0·0 (0·0 to 0·0)
Cyprus	2020	1314·5 (1262·4 to 1368·7)	588·4 (555·7 to 625·7)	562·8 (525·8 to 600·8)	163·3 (150·2 to 178·3)	0·0 (0·0 to 0·0)	1959·0 (1881·4 to 2039·7)	876·9 (828·1 to 932·4)	838·7 (783·5 to 895·3)	243·4 (223·8 to 265·8)	0·0 (0·0 to 0·0)
Cyprus	2021	1275·4 (1224·1 to 1329·1)	569·5 (535·7 to 606·1)	543·9 (508·0 to 580·8)	162·1 (148·8 to 177·1)	0·0 (0·0 to 0·0)	1900·7 (1824·3 to 1980·6)	848·6 (798·3 to 903·3)	810·5 (757·0 to 865·5)	241·6 (221·8 to 263·9)	0·0 (0·0 to 0·0)
Cyprus	2022	1202·4 (1151·4 to 1256·2)	489·0 (456·1 to 525·8)	546·2 (510·1 to 583·5)	167·2 (152·9 to 183·6)	0·0 (0·0 to 0·0)	1791·8 (1715·9 to 1872·0)	728·8 (679·6 to 783·6)	814·0 (760·2 to 869·6)	249·1 (227·9 to 273·6)	0·0 (0·0 to 0·0)
Cyprus	2023	1220·5 (1167·5 to 1275·4)	499·2 (465·1 to 536·3)	549·0 (513·4 to 586·2)	172·4 (157·1 to 189·8)	0·0 (0·0 to 0·0)	1818·9 (1739·8 to 1900·6)	743·9 (693·1 to 799·3)	818·1 (765·0 to 873·6)	256·9 (234·1 to 282·9)	0·0 (0·0 to 0·0)
Cyprus	2024	1237·8 (1183·4 to 1294·5)	508·9 (473·5 to 548·4)	551·3 (515·2 to 589·2)	177·6 (161·6 to 195·4)	0·0 (0·0 to 0·0)	1844·6 (1763·6 to 1929·1)	758·3 (705·6 to 817·3)	821·5 (767·8 to 878·1)	264·7 (240·8 to 291·3)	0·0 (0·0 to 0·0)
Cyprus	2025	1255·6 (1199·3 to 1313·1)	518·9 (480·9 to 560·3)	553·5 (517·2 to 591·9)	183·2 (165·9 to 202·9)	0·0 (0·0 to 0·0)	1871·1 (1787·2 to 1956·8)	773·3 (716·7 to 835·0)	824·9 (770·8 to 882·1)	272·9 (247·2 to 302·4)	0·0 (0·0 to 0·0)
Cyprus	2026	1270·8 (1211·7 to 1329·9)	528·2 (488·4 to 570·9)	554·2 (517·9 to 592·4)	188·4 (170·0 to 210·1)	0·0 (0·0 to 0·0)	1893·7 (1805·7 to 1981·9)	787·1 (727·8 to 850·8)	825·8 (771·8 to 882·8)	280·8 (253·3 to 313·2)	0·0 (0·0 to 0·0)
Cyprus	2027	1286·5 (1226·2 to 1347·4)	537·4 (496·4 to 582·7)	555·1 (519·0 to 593·7)	193·9 (173·9 to 217·5)	0·0 (0·0 to 0·0)	1917·2 (1827·3 to 2008·0)	800·9 (739·7 to 868·4)	827·3 (773·4 to 884·8)	289·0 (259·1 to 324·2)	0·0 (0·0 to 0·0)

Cyprus	2028	1304·5 (1241·8 to 1368·3)	547·1 (503·2 to 594·0)	557·6 (521·3 to 596·1)	199·8 (178·5 to 226·2)	0·0 (0·0 to 0·0)	1944·0 (1850·6 to 2039·1)	815·3 (749·9 to 885·2)	830·9 (776·9 to 888·3)	297·7 (266·0 to 337·1)	0·0 (0·0 to 0·0)
Cyprus	2029	1321·4 (1257·0 to 1387·5)	555·8 (510·5 to 604·5)	559·7 (523·1 to 598·6)	205·8 (183·1 to 233·7)	0·0 (0·0 to 0·0)	1969·2 (1873·3 to 2067·8)	828·3 (760·7 to 900·8)	834·1 (779·5 to 892·0)	306·7 (272·9 to 348·3)	0·0 (0·0 to 0·0)
Cyprus	2030	1337·8 (1272·5 to 1404·9)	563·4 (517·8 to 612·1)	562·2 (525·4 to 601·1)	212·2 (187·1 to 242·5)	0·0 (0·0 to 0·0)	1993·6 (1896·3 to 2093·6)	839·6 (771·6 to 912·2)	837·9 (783·0 to 895·8)	316·2 (278·8 to 361·4)	0·0 (0·0 to 0·0)
Cyprus	2031	1353·6 (1286·2 to 1424·1)	570·6 (524·0 to 619·8)	564·4 (527·5 to 603·5)	218·6 (191·1 to 251·6)	0·0 (0·0 to 0·0)	2017·2 (1916·8 to 2122·3)	850·4 (780·9 to 923·7)	841·0 (786·1 to 899·3)	325·8 (284·8 to 374·9)	0·0 (0·0 to 0·0)
Cyprus	2032	1369·4 (1299·0 to 1441·7)	577·9 (529·6 to 628·6)	566·3 (529·4 to 605·4)	225·2 (195·7 to 260·4)	0·0 (0·0 to 0·0)	2040·8 (1935·9 to 2148·5)	861·2 (789·3 to 936·7)	843·9 (788·9 to 902·2)	335·6 (291·7 to 388·1)	0·0 (0·0 to 0·0)
Cyprus	2033	1386·1 (1313·8 to 1460·5)	585·6 (536·5 to 636·8)	568·4 (531·2 to 607·5)	232·1 (200·4 to 270·3)	0·0 (0·0 to 0·0)	2065·7 (1957·9 to 2176·6)	872·7 (799·5 to 948·9)	847·1 (791·6 to 905·4)	345·9 (298·6 to 402·9)	0·0 (0·0 to 0·0)
Cyprus	2034	1403·0 (1327·8 to 1480·9)	593·4 (542·7 to 644·8)	570·4 (532·9 to 609·7)	239·2 (205·1 to 280·6)	0·0 (0·0 to 0·0)	2090·9 (1978·8 to 2206·9)	884·3 (808·7 to 960·9)	850·1 (794·1 to 908·5)	356·5 (305·7 to 418·2)	0·0 (0·0 to 0·0)
Cyprus	2035	1419·5 (1343·0 to 1502·0)	600·8 (548·1 to 655·1)	572·2 (534·5 to 611·9)	246·4 (209·6 to 290·7)	0·0 (0·0 to 0·0)	2115·4 (2001·4 to 2238·4)	895·4 (816·7 to 976·3)	852·7 (796·6 to 911·8)	367·3 (312·3 to 433·3)	0·0 (0·0 to 0·0)
Cyprus	2036	1435·1 (1354·9 to 1518·8)	607·9 (555·1 to 666·8)	573·5 (535·9 to 613·6)	253·7 (213·7 to 300·6)	0·0 (0·0 to 0·0)	2138·6 (2019·2 to 2263·4)	905·9 (827·2 to 993·7)	854·7 (798·7 to 914·5)	378·1 (318·5 to 448·0)	0·0 (0·0 to 0·0)
Cyprus	2037	1448·1 (1365·5 to 1535·5)	613·7 (560·6 to 672·8)	573·8 (536·0 to 613·6)	260·7 (217·9 to 310·6)	0·0 (0·0 to 0·0)	2158·1 (2034·9 to 2288·3)	914·6 (835·4 to 1002·6)	855·0 (798·8 to 914·5)	388·4 (324·7 to 462·9)	0·0 (0·0 to 0·0)
Cyprus	2038	1461·9 (1375·6 to 1553·6)	619·8 (565·4 to 679·6)	574·2 (536·4 to 613·8)	267·9 (222·1 to 321·9)	0·0 (0·0 to 0·0)	2178·6 (2050·1 to 2315·2)	923·7 (842·5 to 1012·7)	855·7 (799·3 to 914·7)	399·2 (331·0 to 479·6)	0·0 (0·0 to 0·0)
Cyprus	2039	1475·9 (1386·9 to 1572·4)	626·1 (570·9 to 687·0)	574·6 (536·7 to 614·4)	275·3 (226·5 to 333·4)	0·0 (0·0 to 0·0)	2199·5 (2066·8 to 2343·3)	933·0 (850·8 to 1023·8)	856·3 (799·8 to 915·6)	410·2 (337·6 to 496·8)	0·0 (0·0 to 0·0)
Cyprus	2040	1489·9 (1397·7 to 1590·4)	632·2 (573·4 to 694·7)	574·8 (536·8 to 614·4)	282·8 (231·4 to 345·0)	0·0 (0·0 to 0·0)	2220·3 (2082·9 to 2370·1)	942·1 (854·5 to 1035·2)	856·6 (800·0 to 915·7)	421·5 (344·9 to 514·1)	0·0 (0·0 to 0·0)
Cyprus	2041	1504·4 (1408·4 to 1610·6)	638·4 (576·2 to 704·5)	575·2 (537·2 to 614·8)	290·8 (235·3 to 357·8)	0·0 (0·0 to 0·0)	2242·0 (2098·8 to 2400·2)	951·4 (858·7 to 1049·9)	857·2 (800·5 to 916·2)	433·3 (350·7 to 533·2)	0·0 (0·0 to 0·0)

Cyprus	2042	1517·7 (1415·9 to 1627·2)	644·0 (579·6 to 712·9)	574·9 (537·0 to 614·5)	298·8 (239·6 to 370·1)	0·0 (0·0 to 0·0)	2261·7 (2110·1 to 2425·0)	959·7 (863·8 to 1062·4)	856·8 (800·2 to 915·7)	445·2 (357·1 to 551·6)	0·0 (0·0 to 0·0)
Cyprus	2043	1529·1 (1424·1 to 1644·6)	648·8 (587·6 to 719·4)	573·7 (536·0 to 613·3)	306·7 (243·4 to 383·4)	0·0 (0·0 to 0·0)	2278·8 (2122·3 to 2450·8)	966·9 (875·7 to 1072·1)	854·9 (798·7 to 913·9)	457·0 (362·7 to 571·4)	0·0 (0·0 to 0·0)
Cyprus	2044	1537·9 (1427·1 to 1660·5)	652·4 (588·8 to 726·6)	571·1 (533·6 to 610·9)	314·3 (247·0 to 396·4)	0·0 (0·0 to 0·0)	2291·8 (2126·7 to 2474·5)	972·3 (877·5 to 1082·8)	851·2 (795·2 to 910·4)	468·4 (368·1 to 590·7)	0·0 (0·0 to 0·0)
Cyprus	2045	1546·1 (1430·6 to 1673·3)	655·8 (591·5 to 733·2)	568·3 (531·1 to 607·8)	322·0 (249·2 to 411·2)	0·0 (0·0 to 0·0)	2304·1 (2131·9 to 2493·6)	977·4 (881·5 to 1092·7)	846·8 (791·4 to 905·8)	479·9 (371·4 to 612·7)	0·0 (0·0 to 0·0)
Cyprus	2046	1554·6 (1435·9 to 1690·8)	659·2 (593·6 to 738·7)	565·4 (528·2 to 604·8)	330·0 (251·1 to 427·0)	0·0 (0·0 to 0·0)	2316·8 (2139·9 to 2519·8)	982·4 (884·5 to 1100·9)	842·6 (787·1 to 901·2)	491·8 (374·1 to 636·4)	0·0 (0·0 to 0·0)
Cyprus	2047	1562·6 (1438·4 to 1701·6)	662·3 (592·0 to 746·5)	562·3 (525·2 to 601·4)	338·0 (254·6 to 441·7)	0·0 (0·0 to 0·0)	2328·6 (2143·6 to 2535·8)	987·0 (882·2 to 1112·4)	837·9 (782·7 to 896·2)	503·7 (379·5 to 658·2)	0·0 (0·0 to 0·0)
Cyprus	2048	1569·8 (1440·6 to 1716·2)	665·1 (595·4 to 756·7)	558·8 (521·7 to 597·7)	345·9 (258·3 to 458·0)	0·0 (0·0 to 0·0)	2339·4 (2146·9 to 2557·5)	991·1 (887·4 to 1127·7)	832·8 (777·5 to 890·7)	515·5 (385·0 to 682·5)	0·0 (0·0 to 0·0)
Cyprus	2049	1576·1 (1441·5 to 1730·0)	667·1 (595·8 to 764·9)	555·1 (518·1 to 593·7)	353·9 (260·7 to 474·9)	0·0 (0·0 to 0·0)	2348·7 (2148·1 to 2578·1)	994·1 (887·9 to 1139·9)	827·2 (772·2 to 884·8)	527·4 (388·5 to 707·7)	0·0 (0·0 to 0·0)
Cyprus	2050	1582·1 (1442·7 to 1743·4)	668·9 (596·2 to 772·3)	551·2 (514·6 to 589·7)	362·0 (264·0 to 492·2)	0·0 (0·0 to 0·0)	2357·6 (2150·0 to 2598·0)	996·8 (888·5 to 1150·9)	821·4 (766·8 to 878·7)	539·5 (393·4 to 733·4)	0·0 (0·0 to 0·0)
Czech Republic	1995	818·4 (771·8 to 868·0)	723·6 (679·0 to 771·8)	80·7 (68·9 to 93·4)	14·1 (9·6 to 19·3)	0·0 (0·0 to 0·0)	1457·4 (1374·4 to 1545·6)	1288·5 (1209·0 to 1374·3)	143·8 (122·8 to 166·4)	25·1 (17·1 to 34·4)	0·0 (0·0 to 0·0)
Czech Republic	1996	783·6 (747·6 to 822·5)	689·9 (656·7 to 726·6)	79·8 (69·1 to 91·0)	13·8 (9·6 to 19·0)	0·0 (0·0 to 0·0)	1395·3 (1331·2 to 1464·6)	1228·5 (1169·5 to 1293·8)	142·2 (123·1 to 162·0)	24·6 (17·0 to 33·9)	0·0 (0·0 to 0·0)
Czech Republic	1997	791·7 (756·6 to 829·3)	696·8 (663·1 to 732·6)	81·1 (70·9 to 91·7)	13·9 (9·7 to 19·1)	0·0 (0·0 to 0·0)	1409·8 (1347·3 to 1476·8)	1240·7 (1180·7 to 1304·6)	144·3 (126·3 to 163·3)	24·7 (17·3 to 34·0)	0·0 (0·0 to 0·0)
Czech Republic	1998	797·3 (763·0 to 833·9)	701·7 (668·7 to 736·4)	81·7 (71·5 to 92·7)	13·8 (9·8 to 18·8)	0·0 (0·0 to 0·0)	1419·7 (1358·7 to 1484·8)	1249·6 (1190·8 to 1311·2)	145·5 (127·3 to 165·1)	24·5 (17·4 to 33·5)	0·0 (0·0 to 0·0)
Czech Republic	1999	808·2 (773·7 to 842·6)	711·5 (681·1 to 744·9)	83·2 (72·7 to 94·2)	13·5 (9·7 to 18·5)	0·0 (0·0 to 0·0)	1439·1 (1377·8 to 1500·4)	1267·0 (1212·9 to 1326·5)	148·1 (129·4 to 167·8)	24·0 (17·3 to 33·0)	0·0 (0·0 to 0·0)

Czech Republic	2000	840·6 (807·5 to 873·3)	740·9 (709·8 to 773·2)	86·8 (76·2 to 98·2)	12·9 (9·4 to 17·6)	0·0 (0·0 to 0·0)	1496·9 (1437·9 to 1555·0)	1319·4 (1263·9 to 1376·8)	154·5 (135·7 to 174·9)	23·0 (16·8 to 31·3)	0·0 (0·0 to 0·0)
Czech Republic	2001	907·3 (872·6 to 946·6)	798·0 (763·6 to 835·0)	92·9 (81·8 to 104·6)	16·4 (12·1 to 22·0)	0·0 (0·0 to 0·0)	1615·6 (1553·9 to 1685·5)	1420·9 (1359·8 to 1486·8)	165·5 (145·7 to 186·3)	29·2 (21·6 to 39·2)	0·0 (0·0 to 0·0)
Czech Republic	2002	961·9 (926·1 to 1001·5)	844·5 (807·9 to 883·2)	98·3 (87·0 to 110·1)	19·1 (14·2 to 25·3)	0·0 (0·0 to 0·0)	1712·8 (1649·0 to 1783·3)	1503·8 (1438·7 to 1572·8)	175·0 (155·0 to 196·1)	34·0 (25·4 to 45·0)	0·0 (0·0 to 0·0)
Czech Republic	2003	1073·5 (1034·4 to 1118·2)	942·4 (905·1 to 985·2)	108·5 (97·0 to 121·1)	22·7 (17·7 to 29·2)	0·0 (0·0 to 0·0)	1911·6 (1841·9 to 1991·2)	1678·0 (1611·7 to 1754·3)	193·1 (172·7 to 215·7)	40·4 (31·5 to 52·1)	0·0 (0·0 to 0·0)
Czech Republic	2004	1055·1 (1018·3 to 1094·7)	916·6 (881·2 to 954·6)	114·5 (103·9 to 126·7)	24·0 (19·6 to 29·7)	0·0 (0·0 to 0·0)	1878·8 (1813·3 to 1949·4)	1632·2 (1569·2 to 1699·8)	203·8 (185·1 to 225·6)	42·7 (35·0 to 52·9)	0·0 (0·0 to 0·0)
Czech Republic	2005	1124·4 (1086·5 to 1164·6)	964·0 (927·2 to 1000·6)	127·8 (117·7 to 139·0)	32·6 (27·6 to 38·7)	0·0 (0·0 to 0·0)	2002·2 (1934·7 to 2073·8)	1716·5 (1651·1 to 1781·8)	227·6 (209·6 to 247·5)	58·1 (49·1 to 68·9)	0·0 (0·0 to 0·0)
Czech Republic	2006	1176·3 (1138·4 to 1214·3)	997·3 (960·8 to 1031·8)	145·2 (135·7 to 155·1)	33·8 (29·9 to 38·3)	0·0 (0·0 to 0·0)	2094·7 (2027·2 to 2162·3)	1775·9 (1710·9 to 1837·4)	258·5 (241·7 to 276·2)	60·3 (53·2 to 68·1)	0·0 (0·0 to 0·0)
Czech Republic	2007	1230·2 (1193·5 to 1265·3)	1026·9 (992·5 to 1061·4)	169·8 (160·8 to 180·1)	33·5 (30·2 to 37·1)	0·0 (0·0 to 0·0)	2190·5 (2125·2 to 2253·1)	1828·5 (1767·4 to 1890·1)	302·4 (286·3 to 320·8)	59·6 (53·8 to 66·1)	0·0 (0·0 to 0·0)
Czech Republic	2008	1305·8 (1270·5 to 1344·4)	1074·6 (1041·2 to 1110·6)	197·4 (188·7 to 207·4)	33·8 (30·5 to 37·3)	0·0 (0·0 to 0·0)	2325·2 (2262·4 to 2393·9)	1913·5 (1854·1 to 1977·6)	351·5 (335·9 to 369·3)	60·1 (54·4 to 66·3)	0·0 (0·0 to 0·0)
Czech Republic	2009	1379·1 (1342·5 to 1415·2)	1137·6 (1102·6 to 1173·0)	211·9 (202·7 to 221·2)	29·5 (26·4 to 32·9)	0·0 (0·0 to 0·0)	2455·8 (2390·5 to 2520·0)	2025·8 (1963·4 to 2088·8)	377·4 (360·9 to 393·8)	52·6 (47·0 to 58·5)	0·0 (0·0 to 0·0)
Czech Republic	2010	1384·0 (1346·2 to 1421·6)	1146·9 (1110·9 to 1183·7)	212·8 (203·6 to 222·5)	24·3 (21·4 to 27·2)	0·0 (0·0 to 0·0)	2464·4 (2397·2 to 2531·5)	2042·3 (1978·2 to 2107·8)	378·9 (362·5 to 396·2)	43·2 (38·2 to 48·4)	0·0 (0·0 to 0·0)
Czech Republic	2011	1405·6 (1372·2 to 1439·6)	1172·1 (1139·8 to 1206·5)	212·0 (202·6 to 221·8)	21·5 (18·9 to 24·4)	0·0 (0·0 to 0·0)	2503·0 (2443·5 to 2563·5)	2087·1 (2029·6 to 2148·4)	377·5 (360·8 to 395·0)	38·3 (33·7 to 43·4)	0·0 (0·0 to 0·0)
Czech Republic	2012	1454·6 (1418·0 to 1491·5)	1219·9 (1184·3 to 1256·0)	212·6 (202·8 to 222·5)	22·1 (19·6 to 24·8)	0·0 (0·0 to 0·0)	2590·2 (2525·0 to 2655·9)	2172·3 (2108·8 to 2236·5)	378·6 (361·2 to 396·3)	39·3 (34·9 to 44·1)	0·0 (0·0 to 0·0)
Czech Republic	2013	1496·0 (1457·7 to 1534·7)	1239·3 (1201·3 to 1274·2)	212·9 (204·0 to 222·3)	43·8 (40·1 to 48·0)	0·0 (0·0 to 0·0)	2663·8 (2595·7 to 2732·9)	2206·8 (2139·2 to 2268·9)	379·1 (363·3 to 395·8)	77·9 (71·4 to 85·4)	0·0 (0·0 to 0·0)

Czech Republic	2014	1537·9 (1501·3 to 1574·7)	1270·3 (1234·5 to 1306·5)	218·7 (209·7 to 228·2)	49·0 (45·3 to 52·8)	0·0 (0·0 to 0·0)	2738·6 (2673·4 to 2804·0)	2262·0 (2198·2 to 2326·5)	389·4 (373·5 to 406·3)	87·2 (80·7 to 94·1)	0·0 (0·0 to 0·0)
Czech Republic	2015	1567·9 (1529·2 to 1604·6)	1291·4 (1254·0 to 1326·3)	227·6 (217·7 to 237·5)	48·9 (45·3 to 52·8)	0·0 (0·0 to 0·0)	2791·9 (2723·0 to 2857·3)	2299·6 (2233·1 to 2361·7)	405·3 (387·6 to 422·8)	87·0 (80·6 to 94·1)	0·0 (0·0 to 0·0)
Czech Republic	2016	1570·2 (1533·0 to 1609·5)	1286·4 (1250·1 to 1323·4)	233·7 (224·2 to 242·9)	50·1 (46·6 to 53·9)	0·0 (0·0 to 0·0)	2796·1 (2729·8 to 2866·0)	2290·7 (2226·0 to 2356·5)	416·2 (399·2 to 432·5)	89·2 (83·0 to 96·0)	0·0 (0·0 to 0·0)
Czech Republic	2017	1651·3 (1613·8 to 1690·8)	1353·5 (1317·6 to 1392·3)	243·5 (233·5 to 253·0)	54·3 (50·8 to 58·5)	0·0 (0·0 to 0·0)	2940·5 (2873·6 to 3010·9)	2410·2 (2346·2 to 2479·2)	433·6 (415·8 to 450·5)	96·8 (90·4 to 104·1)	0·0 (0·0 to 0·0)
Czech Republic	2018	1780·5 (1727·4 to 1833·3)	1469·2 (1419·4 to 1518·3)	254·6 (241·4 to 268·4)	56·7 (51·9 to 61·8)	0·0 (0·0 to 0·0)	3170·5 (3076·0 to 3264·5)	2616·2 (2527·6 to 2703·6)	453·3 (429·9 to 477·9)	101·0 (92·5 to 110·0)	0·0 (0·0 to 0·0)
Czech Republic	2019	1851·0 (1789·5 to 1910·6)	1532·2 (1475·1 to 1588·6)	259·7 (246·2 to 273·8)	59·1 (54·2 to 64·3)	0·0 (0·0 to 0·0)	3296·1 (3186·5 to 3402·2)	2728·3 (2626·6 to 2828·8)	462·5 (438·5 to 487·6)	105·3 (96·5 to 114·6)	0·0 (0·0 to 0·0)
Czech Republic	2020	2072·2 (2010·9 to 2131·8)	1766·8 (1709·7 to 1823·3)	247·6 (234·5 to 261·3)	57·8 (52·9 to 63·0)	0·0 (0·0 to 0·0)	3689·9 (3580·7 to 3796·0)	3146·1 (3044·4 to 3246·6)	440·9 (417·6 to 465·2)	102·9 (94·1 to 112·1)	0·0 (0·0 to 0·0)
Czech Republic	2021	1971·1 (1902·9 to 2041·3)	1671·7 (1603·9 to 1741·8)	241·6 (229·1 to 254·9)	57·8 (52·8 to 63·3)	0·0 (0·0 to 0·0)	3509·9 (3388·5 to 3635·0)	2976·8 (2856·0 to 3101·7)	430·3 (407·9 to 453·9)	102·8 (94·1 to 112·7)	0·0 (0·0 to 0·0)
Czech Republic	2022	1778·9 (1710·9 to 1851·4)	1477·6 (1413·0 to 1548·4)	242·0 (229·4 to 255·3)	59·2 (54·1 to 65·1)	0·0 (0·0 to 0·0)	3167·7 (3046·6 to 3296·8)	2631·2 (2516·0 to 2757·3)	431·0 (408·5 to 454·6)	105·5 (96·4 to 115·9)	0·0 (0·0 to 0·0)
Czech Republic	2023	1801·2 (1725·4 to 1878·8)	1497·4 (1422·3 to 1574·5)	243·0 (230·4 to 256·3)	60·9 (55·5 to 66·7)	0·0 (0·0 to 0·0)	3207·4 (3072·4 to 3345·5)	2666·3 (2532·7 to 2803·6)	432·7 (410·2 to 456·3)	108·4 (98·9 to 118·8)	0·0 (0·0 to 0·0)
Czech Republic	2024	1824·8 (1739·4 to 1910·1)	1518·0 (1437·6 to 1602·5)	244·3 (231·5 to 257·6)	62·6 (57·1 to 68·7)	0·0 (0·0 to 0·0)	3249·4 (3097·4 to 3401·3)	2703·0 (2559·9 to 2853·6)	435·0 (412·3 to 458·7)	111·4 (101·7 to 122·3)	0·0 (0·0 to 0·0)
Czech Republic	2025	1849·8 (1756·5 to 1943·5)	1539·6 (1450·8 to 1630·2)	245·9 (233·1 to 259·3)	64·4 (58·7 to 70·7)	0·0 (0·0 to 0·0)	3293·9 (3127·8 to 3460·7)	2741·5 (2583·5 to 2902·8)	437·8 (415·2 to 461·7)	114·6 (104·5 to 125·8)	0·0 (0·0 to 0·0)
Czech Republic	2026	1876·7 (1774·9 to 1983·3)	1562·8 (1463·4 to 1669·1)	247·7 (234·9 to 261·4)	66·3 (60·3 to 72·9)	0·0 (0·0 to 0·0)	3341·8 (3160·5 to 3531·7)	2782·8 (2605·9 to 2972·1)	441·0 (418·4 to 465·5)	118·0 (107·3 to 129·9)	0·0 (0·0 to 0·0)
Czech Republic	2027	1905·6 (1796·3 to 2017·9)	1588·0 (1482·0 to 1700·4)	249·4 (236·6 to 263·4)	68·2 (62·0 to 75·0)	0·0 (0·0 to 0·0)	3393·3 (3198·7 to 3593·3)	2827·7 (2639·0 to 3028·0)	444·1 (421·4 to 469·0)	121·5 (110·3 to 133·6)	0·0 (0·0 to 0·0)

Czech Republic	2028	1937·6 (1818·8 to 2059·2)	1615·9 (1496·7 to 1739·3)	251·3 (238·5 to 265·4)	70·3 (63·8 to 77·6)	0·0 (0·0 to 0·0)	3450·2 (3238·8 to 3666·8)	2877·5 (2665·1 to 3097·2)	447·5 (424·7 to 472·6)	125·2 (113·6 to 138·1)	0·0 (0·0 to 0·0)
Czech Republic	2029	1970·1 (1842·8 to 2105·7)	1644·3 (1514·1 to 1779·0)	253·3 (240·0 to 267·4)	72·5 (65·6 to 80·3)	0·0 (0·0 to 0·0)	3508·2 (3281·5 to 3749·6)	2928·0 (2696·1 to 3167·8)	451·0 (427·4 to 476·1)	129·2 (116·9 to 143·0)	0·0 (0·0 to 0·0)
Czech Republic	2030	2005·1 (1868·3 to 2152·5)	1674·9 (1539·2 to 1822·7)	255·3 (242·0 to 269·5)	74·9 (67·5 to 83·0)	0·0 (0·0 to 0·0)	3570·4 (3326·9 to 3832·9)	2982·5 (2740·8 to 3245·7)	454·6 (430·9 to 479·9)	133·3 (120·1 to 147·9)	0·0 (0·0 to 0·0)
Czech Republic	2031	2038·7 (1890·5 to 2192·7)	1704·2 (1554·3 to 1856·9)	257·3 (243·7 to 271·4)	77·2 (69·2 to 86·2)	0·0 (0·0 to 0·0)	3630·3 (3366·3 to 3904·5)	3034·6 (2767·8 to 3306·6)	458·1 (433·9 to 483·3)	137·5 (123·3 to 153·5)	0·0 (0·0 to 0·0)
Czech Republic	2032	2074·2 (1914·1 to 2245·8)	1735·1 (1575·3 to 1902·4)	259·4 (245·8 to 273·7)	79·7 (71·1 to 89·3)	0·0 (0·0 to 0·0)	3693·5 (3408·5 to 3999·0)	3089·7 (2805·0 to 3387·6)	461·9 (437·7 to 487·3)	142·0 (126·6 to 159·1)	0·0 (0·0 to 0·0)
Czech Republic	2033	2106·9 (1939·5 to 2291·3)	1763·5 (1596·5 to 1946·2)	261·2 (247·9 to 275·8)	82·2 (72·7 to 92·7)	0·0 (0·0 to 0·0)	3751·8 (3453·7 to 4080·2)	3140·3 (2842·9 to 3465·5)	465·2 (441·4 to 491·1)	146·3 (129·5 to 165·0)	0·0 (0·0 to 0·0)
Czech Republic	2034	2137·4 (1954·6 to 2329·3)	1789·7 (1606·6 to 1980·9)	263·0 (249·4 to 277·6)	84·7 (74·5 to 96·0)	0·0 (0·0 to 0·0)	3806·0 (3480·5 to 4147·7)	3186·9 (2860·8 to 3527·4)	468·4 (444·1 to 494·3)	150·8 (132·7 to 170·9)	0·0 (0·0 to 0·0)
Czech Republic	2035	2168·2 (1977·1 to 2370·4)	1816·3 (1625·0 to 2020·0)	264·7 (250·9 to 279·4)	87·2 (76·2 to 99·6)	0·0 (0·0 to 0·0)	3860·9 (3520·6 to 4220·9)	3234·3 (2893·6 to 3597·0)	471·3 (446·7 to 497·5)	155·3 (135·6 to 177·4)	0·0 (0·0 to 0·0)
Czech Republic	2036	2200·3 (1996·7 to 2413·9)	1844·1 (1641·6 to 2057·0)	266·3 (252·3 to 281·1)	89·8 (78·0 to 103·3)	0·0 (0·0 to 0·0)	3918·0 (3555·6 to 4298·3)	3283·7 (2923·3 to 3662·8)	474·3 (449·3 to 500·6)	160·0 (138·9 to 183·9)	0·0 (0·0 to 0·0)
Czech Republic	2037	2232·3 (2018·6 to 2462·3)	1872·2 (1663·1 to 2105·1)	267·6 (253·7 to 282·5)	92·5 (79·8 to 107·3)	0·0 (0·0 to 0·0)	3975·0 (3594·5 to 4384·6)	3333·7 (2961·4 to 3748·5)	476·5 (451·7 to 503·1)	164·7 (142·1 to 191·1)	0·0 (0·0 to 0·0)
Czech Republic	2038	2263·6 (2043·2 to 2509·7)	1900·3 (1679·1 to 2151·5)	268·2 (254·1 to 283·4)	95·1 (81·5 to 111·4)	0·0 (0·0 to 0·0)	4030·8 (3638·3 to 4469·0)	3383·8 (2990·0 to 3831·2)	477·6 (452·5 to 504·7)	169·4 (145·1 to 198·3)	0·0 (0·0 to 0·0)
Czech Republic	2039	2293·5 (2065·4 to 2548·1)	1927·8 (1703·7 to 2180·8)	268·0 (253·7 to 283·5)	97·7 (83·2 to 115·0)	0·0 (0·0 to 0·0)	4084·0 (3677·8 to 4537·4)	3432·8 (3033·7 to 3883·3)	477·3 (451·7 to 504·7)	173·9 (148·1 to 204·7)	0·0 (0·0 to 0·0)
Czech Republic	2040	2323·7 (2086·3 to 2579·9)	1955·7 (1725·9 to 2216·0)	267·7 (253·3 to 283·4)	100·3 (84·7 to 118·9)	0·0 (0·0 to 0·0)	4137·7 (3715·1 to 4594·0)	3482·5 (3073·4 to 3946·1)	476·7 (451·1 to 504·7)	178·6 (150·8 to 211·8)	0·0 (0·0 to 0·0)
Czech Republic	2041	2350·8 (2100·9 to 2618·4)	1980·7 (1741·5 to 2245·8)	267·2 (252·9 to 282·9)	102·9 (85·8 to 123·0)	0·0 (0·0 to 0·0)	4186·0 (3741·0 to 4662·5)	3527·0 (3101·1 to 3999·0)	475·8 (450·4 to 503·8)	183·2 (152·7 to 219·1)	0·0 (0·0 to 0·0)

Czech Republic	2042	2376·8 (2121·2 to 2656·2)	2004·2 (1747·3 to 2277·9)	267·1 (252·7 to 282·8)	105·5 (87·1 to 127·0)	0·0 (0·0 to 0·0)	4232·4 (3777·2 to 4729·9)	3568·8 (3111·4 to 4056·2)	475·6 (449·9 to 503·5)	187·9 (155·1 to 226·2)	0·0 (0·0 to 0·0)
Czech Republic	2043	2399·9 (2134·8 to 2687·6)	2024·6 (1766·6 to 2307·4)	267·1 (252·5 to 282·9)	108·2 (88·6 to 131·1)	0·0 (0·0 to 0·0)	4273·4 (3801·4 to 4785·7)	3605·1 (3145·8 to 4108·8)	475·6 (449·7 to 503·7)	192·7 (157·7 to 233·5)	0·0 (0·0 to 0·0)
Czech Republic	2044	2422·9 (2155·1 to 2718·8)	2044·7 (1777·6 to 2338·7)	267·2 (252·7 to 283·0)	111·0 (90·0 to 135·3)	0·0 (0·0 to 0·0)	4314·4 (3837·5 to 4841·4)	3641·0 (3165·3 to 4164·5)	475·7 (450·0 to 504·0)	197·6 (160·3 to 241·0)	0·0 (0·0 to 0·0)
Czech Republic	2045	2444·3 (2170·8 to 2756·9)	2063·2 (1791·0 to 2358·1)	267·3 (252·9 to 283·3)	113·8 (91·5 to 139·6)	0·0 (0·0 to 0·0)	4352·5 (3865·5 to 4909·1)	3673·9 (3189·2 to 4199·0)	476·0 (450·4 to 504·5)	202·6 (162·9 to 248·6)	0·0 (0·0 to 0·0)
Czech Republic	2046	2466·5 (2184·6 to 2779·7)	2082·2 (1800·3 to 2389·9)	267·7 (253·2 to 283·8)	116·7 (93·1 to 144·3)	0·0 (0·0 to 0·0)	4392·0 (3890·2 to 4949·8)	3707·7 (3205·8 to 4255·6)	476·6 (450·8 to 505·4)	207·8 (165·8 to 257·0)	0·0 (0·0 to 0·0)
Czech Republic	2047	2486·9 (2203·5 to 2802·4)	2099·3 (1807·4 to 2409·3)	268·0 (253·5 to 284·4)	119·6 (94·8 to 148·8)	0·0 (0·0 to 0·0)	4428·5 (3923·8 to 4990·1)	3738·2 (3218·3 to 4290·3)	477·2 (451·5 to 506·5)	213·0 (168·7 to 264·9)	0·0 (0·0 to 0·0)
Czech Republic	2048	2508·4 (2210·7 to 2831·7)	2117·1 (1820·0 to 2432·1)	268·6 (254·0 to 285·2)	122·7 (96·4 to 153·7)	0·0 (0·0 to 0·0)	4466·7 (3936·6 to 5042·4)	3770·0 (3240·8 to 4330·8)	478·2 (452·2 to 507·8)	218·5 (171·6 to 273·6)	0·0 (0·0 to 0·0)
Czech Republic	2049	2526·9 (2227·4 to 2849·9)	2131·9 (1829·2 to 2452·3)	269·1 (254·4 to 285·9)	125·8 (97·8 to 158·8)	0·0 (0·0 to 0·0)	4499·6 (3966·3 to 5074·7)	3796·3 (3257·3 to 4366·9)	479·2 (452·9 to 509·0)	224·1 (174·2 to 282·8)	0·0 (0·0 to 0·0)
Czech Republic	2050	2547·1 (2243·8 to 2869·1)	2148·2 (1842·7 to 2466·9)	269·8 (254·9 to 286·6)	129·1 (99·5 to 164·3)	0·0 (0·0 to 0·0)	4535·5 (3995·6 to 5109·0)	3825·3 (3281·2 to 4392·7)	480·4 (453·9 to 510·4)	229·8 (177·3 to 292·5)	0·0 (0·0 to 0·0)
Germany	1995	3299·5 (3233·6 to 3365·2)	2660·8 (2605·3 to 2715·1)	349·7 (324·3 to 376·0)	289·0 (266·9 to 312·7)	0·0 (0·0 to 0·0)	3889·9 (3812·2 to 3967·3)	3136·9 (3071·4 to 3200·9)	412·3 (382·3 to 443·3)	340·7 (314·6 to 368·6)	0·0 (0·0 to 0·0)
Germany	1996	3403·9 (3355·0 to 3457·6)	2741·0 (2696·6 to 2786·8)	364·3 (344·8 to 384·0)	298·7 (281·2 to 316·1)	0·0 (0·0 to 0·0)	4013·0 (3955·4 to 4076·3)	3231·4 (3179·1 to 3285·4)	429·5 (406·4 to 452·7)	352·1 (331·5 to 372·6)	0·0 (0·0 to 0·0)
Germany	1997	3547·7 (3496·0 to 3600·2)	2836·5 (2787·1 to 2885·3)	392·1 (373·2 to 411·4)	319·0 (300·8 to 335·8)	0·0 (0·0 to 0·0)	4182·5 (4121·5 to 4244·4)	3344·1 (3285·8 to 3401·6)	462·2 (440·0 to 485·0)	376·1 (354·6 to 395·9)	0·0 (0·0 to 0·0)
Germany	1998	3622·8 (3575·0 to 3674·8)	2871·3 (2829·7 to 2918·5)	418·5 (399·7 to 437·2)	333·0 (315·1 to 350·0)	0·0 (0·0 to 0·0)	4271·0 (4214·7 to 4332·3)	3385·1 (3336·0 to 3440·8)	493·4 (471·3 to 515·4)	392·5 (371·5 to 412·7)	0·0 (0·0 to 0·0)
Germany	1999	3714·3 (3664·5 to 3762·9)	2922·9 (2878·5 to 2967·0)	442·4 (426·4 to 460·0)	349·0 (335·2 to 361·4)	0·0 (0·0 to 0·0)	4378·9 (4320·2 to 4436·2)	3445·9 (3393·6 to 3497·8)	521·6 (502·7 to 542·3)	411·5 (395·2 to 426·0)	0·0 (0·0 to 0·0)

Germany	2000	3801·6 (3757·4 to 3843·3)	2974·8 (2933·2 to 3013·1)	464·4 (451·1 to 478·6)	362·5 (355·9 to 369·4)	0·0 (0·0 to 0·0)	4481·9 (4429·7 to 4531·0)	3507·1 (3458·1 to 3552·3)	547·4 (531·8 to 564·3)	427·3 (419·6 to 435·5)	0·0 (0·0 to 0·0)
Germany	2001	3908·5 (3864·1 to 3953·1)	3054·7 (3012·5 to 3093·7)	481·8 (469·5 to 495·4)	372·0 (366·0 to 378·5)	0·0 (0·0 to 0·0)	4607·8 (4555·5 to 4660·4)	3601·3 (3551·5 to 3647·2)	568·0 (553·5 to 584·1)	438·6 (431·5 to 446·3)	0·0 (0·0 to 0·0)
Germany	2002	3978·3 (3935·2 to 4024·0)	3096·4 (3057·7 to 3138·2)	500·1 (487·5 to 513·1)	381·8 (375·6 to 388·3)	0·0 (0·0 to 0·0)	4690·1 (4639·4 to 4744·0)	3650·4 (3604·8 to 3699·7)	589·6 (574·8 to 604·9)	450·1 (442·8 to 457·8)	0·0 (0·0 to 0·0)
Germany	2003	4026·1 (3985·4 to 4073·0)	3108·4 (3068·9 to 3150·7)	524·2 (511·1 to 537·0)	393·5 (387·7 to 399·6)	0·0 (0·0 to 0·0)	4746·5 (4698·5 to 4801·8)	3664·6 (3618·0 to 3714·5)	618·0 (602·5 to 633·0)	464·0 (457·1 to 471·2)	0·0 (0·0 to 0·0)
Germany	2004	4000·8 (3958·8 to 4044·9)	3046·1 (3005·5 to 3089·1)	552·9 (539·8 to 565·3)	401·8 (395·5 to 408·0)	0·0 (0·0 to 0·0)	4716·7 (4667·2 to 4768·7)	3591·2 (3543·3 to 3641·9)	651·8 (636·3 to 666·5)	473·7 (466·3 to 481·0)	0·0 (0·0 to 0·0)
Germany	2005	4081·1 (4039·5 to 4124·6)	3084·2 (3045·9 to 3126·6)	580·8 (567·3 to 593·7)	416·2 (410·7 to 422·5)	0·0 (0·0 to 0·0)	4811·3 (4762·3 to 4862·7)	3636·0 (3590·9 to 3686·1)	684·7 (668·8 to 699·9)	490·6 (484·1 to 498·1)	0·0 (0·0 to 0·0)
Germany	2006	4191·1 (4147·4 to 4234·3)	3156·3 (3115·0 to 3197·2)	604·6 (592·0 to 617·6)	430·1 (424·0 to 436·9)	0·0 (0·0 to 0·0)	4941·0 (4889·5 to 4991·9)	3721·1 (3672·4 to 3769·3)	712·8 (697·9 to 728·1)	507·1 (499·9 to 515·1)	0·0 (0·0 to 0·0)
Germany	2007	4264·0 (4221·3 to 4307·0)	3204·7 (3165·5 to 3244·9)	614·3 (601·6 to 628·1)	445·0 (438·7 to 451·5)	0·0 (0·0 to 0·0)	5027·0 (4976·6 to 5077·7)	3778·2 (3731·9 to 3825·5)	724·2 (709·2 to 740·5)	524·6 (517·2 to 532·3)	0·0 (0·0 to 0·0)
Germany	2008	4417·3 (4373·9 to 4461·9)	3326·8 (3284·7 to 3366·4)	624·2 (611·3 to 637·4)	466·3 (459·8 to 472·2)	0·0 (0·0 to 0·0)	5207·6 (5156·6 to 5260·3)	3922·1 (3872·4 to 3968·8)	735·9 (720·7 to 751·4)	549·7 (542·0 to 556·7)	0·0 (0·0 to 0·0)
Germany	2009	4562·4 (4518·5 to 4606·6)	3449·5 (3408·0 to 3491·8)	635·9 (623·0 to 650·7)	477·0 (470·2 to 483·4)	0·0 (0·0 to 0·0)	5378·8 (5327·0 to 5430·9)	4066·7 (4017·8 to 4116·6)	749·7 (734·5 to 767·1)	562·4 (554·3 to 569·9)	0·0 (0·0 to 0·0)
Germany	2010	4711·5 (4660·3 to 4756·6)	3567·0 (3520·2 to 3608·7)	656·1 (642·6 to 669·6)	488·4 (482·0 to 494·8)	0·0 (0·0 to 0·0)	5554·6 (5494·2 to 5607·7)	4205·3 (4150·1 to 4254·4)	773·5 (757·5 to 789·4)	575·8 (568·2 to 583·3)	0·0 (0·0 to 0·0)
Germany	2011	4752·1 (4703·2 to 4794·5)	3582·3 (3539·4 to 3623·2)	668·6 (655·7 to 681·3)	501·2 (495·2 to 507·5)	0·0 (0·0 to 0·0)	5602·4 (5544·8 to 5652·4)	4223·2 (4172·7 to 4271·5)	788·2 (773·0 to 803·2)	590·9 (583·7 to 598·3)	0·0 (0·0 to 0·0)
Germany	2012	4797·0 (4755·6 to 4839·2)	3626·2 (3587·2 to 3665·4)	670·5 (656·7 to 684·2)	500·3 (493·7 to 507·0)	0·0 (0·0 to 0·0)	5655·3 (5606·6 to 5705·1)	4275·0 (4229·0 to 4321·3)	790·5 (774·2 to 806·6)	589·8 (582·1 to 597·7)	0·0 (0·0 to 0·0)
Germany	2013	4875·6 (4829·2 to 4920·0)	3721·6 (3679·5 to 3764·7)	655·4 (642·1 to 668·8)	498·6 (492·4 to 505·0)	0·0 (0·0 to 0·0)	5748·1 (5693·3 to 5800·3)	4387·5 (4337·9 to 4438·3)	772·7 (757·0 to 788·4)	587·8 (580·6 to 595·4)	0·0 (0·0 to 0·0)

Germany	2014	4963·0 (4918·8 to 5007·3)	3816·1 (3775·3 to 3857·5)	650·0 (636·8 to 663·9)	497·0 (491·2 to 503·3)	0·0 (0·0 to 0·0)	5851·1 (5798·9 to 5903·3)	4498·9 (4450·8 to 4547·7)	766·3 (750·8 to 782·7)	585·9 (579·1 to 593·3)	0·0 (0·0 to 0·0)
Germany	2015	5069·5 (5025·3 to 5116·7)	3904·4 (3862·8 to 3947·1)	660·7 (648·5 to 674·6)	504·4 (498·3 to 510·5)	0·0 (0·0 to 0·0)	5976·6 (5924·5 to 6032·2)	4603·0 (4554·0 to 4653·4)	778·9 (764·5 to 795·3)	594·6 (587·4 to 601·9)	0·0 (0·0 to 0·0)
Germany	2016	5184·0 (5138·3 to 5227·3)	4007·8 (3963·3 to 4048·5)	672·1 (658·5 to 685·7)	504·1 (497·8 to 510·3)	0·0 (0·0 to 0·0)	6111·6 (6057·7 to 6162·7)	4724·9 (4672·5 to 4772·9)	792·4 (776·4 to 808·4)	594·3 (586·8 to 601·6)	0·0 (0·0 to 0·0)
Germany	2017	5307·4 (5260·2 to 5353·4)	4122·1 (4077·9 to 4163·7)	679·4 (665·7 to 693·2)	505·9 (499·1 to 512·1)	0·0 (0·0 to 0·0)	6257·0 (6201·4 to 6311·3)	4859·7 (4807·6 to 4908·7)	801·0 (784·9 to 817·2)	596·4 (588·4 to 603·8)	0·0 (0·0 to 0·0)
Germany	2018	5403·9 (5349·9 to 5457·4)	4198·9 (4148·7 to 4247·3)	683·6 (666·4 to 701·3)	521·4 (514·2 to 528·6)	0·0 (0·0 to 0·0)	6370·8 (6307·2 to 6433·9)	4950·3 (4891·0 to 5007·3)	805·9 (785·6 to 826·8)	614·7 (606·2 to 623·2)	0·0 (0·0 to 0·0)
Germany	2019	5498·2 (5427·9 to 5570·1)	4281·6 (4214·2 to 4349·1)	683·7 (666·7 to 701·7)	532·8 (524·2 to 541·4)	0·0 (0·0 to 0·0)	6482·0 (6399·2 to 6566·7)	5047·7 (4968·3 to 5127·3)	806·1 (786·0 to 827·2)	628·2 (618·0 to 638·3)	0·0 (0·0 to 0·0)
Germany	2020	6074·1 (5989·6 to 6161·4)	4898·4 (4817·4 to 4981·1)	654·4 (638·0 to 671·9)	521·2 (509·1 to 532·3)	0·0 (0·0 to 0·0)	7160·9 (7061·3 to 7263·9)	5774·9 (5679·3 to 5872·3)	771·5 (752·1 to 792·1)	614·5 (600·2 to 627·5)	0·0 (0·0 to 0·0)
Germany	2021	5838·1 (5756·9 to 5922·2)	4700·9 (4623·1 to 4780·2)	626·6 (611·1 to 643·2)	510·5 (498·1 to 522·9)	0·0 (0·0 to 0·0)	6882·7 (6787·0 to 6981·9)	5542·1 (5450·3 to 5635·5)	738·8 (720·4 to 758·2)	601·9 (587·3 to 616·4)	0·0 (0·0 to 0·0)
Germany	2022	5137·6 (5050·2 to 5226·4)	3986·2 (3903·3 to 4068·9)	628·1 (612·6 to 644·8)	523·3 (507·7 to 538·1)	0·0 (0·0 to 0·0)	6056·9 (5953·8 to 6161·6)	4699·5 (4601·7 to 4797·0)	740·5 (722·2 to 760·2)	616·9 (598·5 to 634·4)	0·0 (0·0 to 0·0)
Germany	2023	5196·1 (5095·7 to 5298·5)	4030·3 (3934·1 to 4130·3)	629·3 (613·4 to 646·1)	536·6 (519·9 to 553·0)	0·0 (0·0 to 0·0)	6125·9 (6007·5 to 6246·6)	4751·4 (4638·0 to 4869·4)	741·9 (723·2 to 761·8)	632·6 (612·9 to 652·0)	0·0 (0·0 to 0·0)
Germany	2024	5258·5 (5148·8 to 5370·1)	4078·4 (3973·2 to 4186·1)	630·0 (613·9 to 646·9)	550·1 (531·2 to 569·6)	0·0 (0·0 to 0·0)	6199·4 (6070·1 to 6331·0)	4808·2 (4684·1 to 4935·1)	742·7 (723·8 to 762·7)	648·6 (626·2 to 671·5)	0·0 (0·0 to 0·0)
Germany	2025	5325·1 (5200·3 to 5446·6)	4131·0 (4011·1 to 4247·0)	630·3 (614·3 to 647·3)	563·8 (541·4 to 587·0)	0·0 (0·0 to 0·0)	6277·9 (6130·8 to 6421·1)	4870·1 (4728·8 to 5006·9)	743·1 (724·2 to 763·1)	664·7 (638·3 to 692·0)	0·0 (0·0 to 0·0)
Germany	2026	5392·3 (5258·5 to 5524·5)	4184·1 (4054·0 to 4310·1)	630·5 (614·4 to 647·5)	577·7 (551·8 to 606·0)	0·0 (0·0 to 0·0)	6357·1 (6199·4 to 6513·0)	4932·8 (4779·4 to 5081·3)	743·3 (724·4 to 763·4)	681·1 (650·5 to 714·5)	0·0 (0·0 to 0·0)
Germany	2027	5456·2 (5312·4 to 5597·3)	4233·7 (4097·3 to 4368·7)	630·5 (614·7 to 647·2)	591·9 (560·7 to 625·0)	0·0 (0·0 to 0·0)	6432·5 (6263·0 to 6598·8)	4991·3 (4830·4 to 5150·4)	743·3 (724·7 to 763·0)	697·8 (661·1 to 736·8)	0·0 (0·0 to 0·0)

Germany	2028	5517·9 (5360·6 to 5665·9)	4281·2 (4130·7 to 4421·5)	630·3 (614·4 to 647·2)	606·4 (574·1 to 639·1)	0·0 (0·0 to 0·0)	6505·2 (6319·8 to 6679·7)	5047·2 (4869·8 to 5212·6)	743·1 (724·4 to 763·0)	714·9 (676·8 to 753·4)	0·0 (0·0 to 0·0)
Germany	2029	5575·9 (5408·9 to 5732·7)	4325·5 (4166·4 to 4478·1)	629·7 (613·6 to 646·7)	620·7 (587·9 to 656·0)	0·0 (0·0 to 0·0)	6573·6 (6376·8 to 6758·4)	5099·5 (4911·9 to 5279·4)	742·3 (723·4 to 762·4)	731·8 (693·1 to 773·3)	0·0 (0·0 to 0·0)
Germany	2030	5631·1 (5461·2 to 5789·8)	4367·9 (4204·7 to 4518·8)	628·9 (612·9 to 646·0)	634·4 (598·8 to 672·4)	0·0 (0·0 to 0·0)	6638·7 (6438·3 to 6825·7)	5149·4 (4957·0 to 5327·3)	741·4 (722·6 to 761·6)	747·9 (706·0 to 792·8)	0·0 (0·0 to 0·0)
Germany	2031	5686·1 (5512·0 to 5854·9)	4409·8 (4243·1 to 4567·8)	628·4 (612·4 to 645·6)	647·9 (610·1 to 687·8)	0·0 (0·0 to 0·0)	6703·5 (6498·3 to 6902·5)	5198·9 (5002·4 to 5385·1)	740·8 (722·0 to 761·2)	763·8 (719·3 to 810·9)	0·0 (0·0 to 0·0)
Germany	2032	5745·8 (5562·1 to 5918·3)	4455·4 (4279·3 to 4622·2)	628·5 (612·3 to 645·9)	661·9 (623·9 to 704·3)	0·0 (0·0 to 0·0)	6773·8 (6557·4 to 6977·3)	5252·6 (5045·0 to 5449·2)	741·0 (721·9 to 761·5)	780·3 (735·6 to 830·3)	0·0 (0·0 to 0·0)
Germany	2033	5808·6 (5617·6 to 5992·0)	4503·0 (4316·5 to 4680·7)	628·9 (612·5 to 646·6)	676·7 (636·1 to 721·0)	0·0 (0·0 to 0·0)	6848·0 (6622·7 to 7064·2)	5308·8 (5088·9 to 5518·2)	741·4 (722·1 to 762·3)	797·8 (749·9 to 850·0)	0·0 (0·0 to 0·0)
Germany	2034	5874·8 (5676·7 to 6072·6)	4552·8 (4358·3 to 4743·3)	629·6 (613·0 to 647·5)	692·3 (648·6 to 740·1)	0·0 (0·0 to 0·0)	6926·0 (6692·4 to 7159·2)	5367·5 (5138·1 to 5592·0)	742·3 (722·6 to 763·3)	816·2 (764·7 to 872·5)	0·0 (0·0 to 0·0)
Germany	2035	5945·3 (5736·5 to 6156·8)	4605·6 (4404·8 to 4808·6)	630·8 (613·9 to 648·7)	708·9 (661·9 to 760·4)	0·0 (0·0 to 0·0)	7009·1 (6762·9 to 7258·5)	5429·7 (5193·0 to 5669·0)	743·7 (723·8 to 764·8)	835·7 (780·3 to 896·4)	0·0 (0·0 to 0·0)
Germany	2036	6022·1 (5806·1 to 6249·6)	4662·8 (4458·8 to 4881·5)	632·6 (615·5 to 650·5)	726·7 (674·8 to 782·3)	0·0 (0·0 to 0·0)	7099·6 (6845·0 to 7367·8)	5497·1 (5256·7 to 5755·0)	745·8 (725·6 to 766·8)	856·7 (795·5 to 922·3)	0·0 (0·0 to 0·0)
Germany	2037	6106·0 (5877·4 to 6351·6)	4725·0 (4511·1 to 4965·7)	635·0 (617·7 to 653·2)	746·0 (687·6 to 806·0)	0·0 (0·0 to 0·0)	7198·5 (6929·0 to 7488·1)	5570·4 (5318·3 to 5854·2)	748·7 (728·2 to 770·1)	879·4 (810·6 to 950·2)	0·0 (0·0 to 0·0)
Germany	2038	6200·6 (5955·1 to 6464·5)	4795·0 (4567·0 to 5040·3)	638·4 (620·8 to 656·9)	767·2 (703·5 to 832·9)	0·0 (0·0 to 0·0)	7310·0 (7020·7 to 7621·2)	5652·9 (5384·1 to 5942·2)	752·7 (731·8 to 774·4)	904·5 (829·4 to 982·0)	0·0 (0·0 to 0·0)
Germany	2039	6304·7 (6038·3 to 6588·2)	4872·0 (4616·6 to 5142·9)	642·6 (624·6 to 661·2)	790·2 (721·8 to 865·6)	0·0 (0·0 to 0·0)	7432·8 (7118·8 to 7767·0)	5743·7 (5442·7 to 6063·1)	757·6 (736·4 to 779·5)	931·6 (851·0 to 1020·5)	0·0 (0·0 to 0·0)
Germany	2040	6415·5 (6130·5 to 6715·1)	4953·5 (4679·2 to 5244·0)	647·3 (629·0 to 666·3)	814·7 (739·4 to 897·8)	0·0 (0·0 to 0·0)	7563·4 (7227·4 to 7916·6)	5839·8 (5516·4 to 6182·3)	763·1 (741·6 to 785·6)	960·4 (871·7 to 1058·4)	0·0 (0·0 to 0·0)
Germany	2041	6528·0 (6221·5 to 6847·9)	5035·8 (4740·4 to 5350·9)	652·1 (633·7 to 671·4)	840·1 (756·9 to 931·8)	0·0 (0·0 to 0·0)	7696·1 (7334·7 to 8073·2)	5936·9 (5588·6 to 6308·3)	768·8 (747·1 to 791·6)	990·4 (892·4 to 1098·5)	0·0 (0·0 to 0·0)

Germany	2042	6640·0 (6308·4 to 6976·2)	5117·1 (4795·3 to 5447·6)	656·9 (638·1 to 676·2)	866·1 (774·7 to 967·7)	0·0 (0·0 to 0·0)	7828·1 (7437·2 to 8224·5)	6032·7 (5653·3 to 6422·3)	774·4 (752·3 to 797·2)	1021·0 (913·3 to 1140·9)	0·0 (0·0 to 0·0)
Germany	2043	6749·7 (6395·9 to 7107·3)	5196·0 (4862·4 to 5542·6)	661·4 (642·4 to 680·9)	892·4 (792·3 to 1002·4)	0·0 (0·0 to 0·0)	7957·4 (7540·3 to 8379·1)	6125·7 (5732·4 to 6534·3)	779·7 (757·3 to 802·7)	1052·0 (934·1 to 1181·8)	0·0 (0·0 to 0·0)
Germany	2044	6860·9 (6483·1 to 7236·2)	5275·5 (4914·9 to 5640·8)	665·9 (646·9 to 685·8)	919·5 (809·7 to 1038·3)	0·0 (0·0 to 0·0)	8088·6 (7643·1 to 8530·9)	6219·4 (5794·4 to 6650·1)	785·1 (762·7 to 808·5)	1084·1 (954·6 to 1224·1)	0·0 (0·0 to 0·0)
Germany	2045	6973·6 (6565·7 to 7368·2)	5355·4 (4980·3 to 5737·1)	670·6 (651·1 to 690·8)	947·6 (828·1 to 1079·8)	0·0 (0·0 to 0·0)	8221·4 (7740·5 to 8686·6)	6313·6 (5871·5 to 6763·6)	790·6 (767·6 to 814·5)	1117·2 (976·3 to 1273·1)	0·0 (0·0 to 0·0)
Germany	2046	7083·8 (6652·4 to 7494·8)	5432·6 (5030·7 to 5829·0)	675·1 (655·1 to 695·6)	976·2 (845·0 to 1122·6)	0·0 (0·0 to 0·0)	8351·4 (7842·7 to 8835·8)	6404·6 (5930·9 to 6872·0)	795·8 (772·3 to 820·1)	1150·9 (996·2 to 1323·4)	0·0 (0·0 to 0·0)
Germany	2047	7194·0 (6738·6 to 7618·6)	5508·9 (5071·8 to 5919·8)	679·5 (659·1 to 700·5)	1005·6 (861·4 to 1165·9)	0·0 (0·0 to 0·0)	8481·3 (7944·4 to 8981·8)	6494·6 (5979·3 to 6979·0)	801·1 (777·0 to 825·8)	1185·5 (1015·6 to 1374·5)	0·0 (0·0 to 0·0)
Germany	2048	7303·1 (6818·5 to 7741·2)	5583·6 (5109·6 to 6008·1)	683·8 (662·9 to 705·2)	1035·6 (878·9 to 1212·1)	0·0 (0·0 to 0·0)	8609·8 (8038·5 to 9126·4)	6582·7 (6023·8 to 7083·1)	806·2 (781·5 to 831·4)	1220·9 (1036·2 to 1429·0)	0·0 (0·0 to 0·0)
Germany	2049	7410·8 (6898·8 to 7868·3)	5656·5 (5170·6 to 6093·8)	688·1 (666·6 to 709·8)	1066·2 (896·7 to 1256·8)	0·0 (0·0 to 0·0)	8736·8 (8133·2 to 9276·1)	6668·7 (6095·8 to 7184·1)	811·2 (785·8 to 836·8)	1257·0 (1057·1 to 1481·6)	0·0 (0·0 to 0·0)
Germany	2050	7518·1 (6985·7 to 7986·5)	5728·4 (5211·3 to 6171·8)	692·2 (670·5 to 714·3)	1097·6 (915·5 to 1300·6)	0·0 (0·0 to 0·0)	8863·4 (8235·7 to 9415·6)	6753·4 (6143·8 to 7276·1)	816·0 (790·4 to 842·1)	1293·9 (1079·3 to 1533·3)	0·0 (0·0 to 0·0)
Djibouti*	1995	76·9 (68·7 to 86·1)	28·2 (23·3 to 34·1)	33·3 (27·0 to 41·0)	0·4 (0·3 to 0·5)	15·0 (15·0 to 15·0)	127·0 (113·4 to 142·1)	46·6 (38·5 to 56·2)	55·0 (44·6 to 67·6)	0·6 (0·4 to 0·9)	24·8 (24·8 to 24·8)
Djibouti*	1996	63·6 (55·7 to 71·9)	26·5 (22·0 to 31·8)	32·4 (26·3 to 39·3)	0·4 (0·2 to 0·5)	4·3 (4·3 to 4·3)	104·9 (91·9 to 118·6)	43·7 (36·3 to 52·5)	53·4 (43·4 to 64·9)	0·6 (0·4 to 0·9)	7·2 (7·2 to 7·2)
Djibouti*	1997	85·9 (78·0 to 94·4)	28·7 (24·1 to 34·2)	32·7 (26·3 to 39·5)	0·4 (0·3 to 0·5)	24·1 (24·1 to 24·1)	141·8 (128·7 to 155·7)	47·4 (39·8 to 56·4)	53·9 (43·4 to 65·1)	0·6 (0·4 to 0·9)	39·8 (39·8 to 39·8)
Djibouti*	1998	88·1 (80·5 to 96·4)	29·3 (24·5 to 34·8)	32·2 (26·0 to 38·9)	0·4 (0·3 to 0·5)	26·2 (26·2 to 26·2)	145·4 (132·8 to 159·1)	48·3 (40·4 to 57·4)	53·2 (42·9 to 64·1)	0·6 (0·4 to 0·9)	43·3 (43·3 to 43·3)
Djibouti*	1999	72·7 (64·6 to 81·4)	30·4 (25·4 to 36·0)	32·4 (26·5 to 39·3)	0·4 (0·3 to 0·6)	9·5 (9·5 to 9·5)	119·9 (106·7 to 134·3)	50·2 (42·0 to 59·3)	53·4 (43·7 to 64·9)	0·7 (0·4 to 0·9)	15·6 (15·6 to 15·6)

Djibouti*	2000	69·5 (61·6 to 78·0)	29·5 (24·8 to 35·0)	32·0 (26·2 to 38·8)	0·4 (0·3 to 0·6)	7·5 (7·5 to 7·5)	114·7 (101·7 to 128·7)	48·8 (40·9 to 57·7)	52·8 (43·2 to 64·0)	0·7 (0·5 to 1·0)	12·5 (12·5 to 12·5)
Djibouti*	2001	60·0 (52·9 to 68·0)	26·9 (22·5 to 31·8)	30·6 (24·9 to 36·9)	0·4 (0·3 to 0·6)	2·0 (2·0 to 2·0)	98·9 (87·4 to 112·2)	44·4 (37·2 to 52·6)	50·6 (41·1 to 61·0)	0·6 (0·4 to 0·9)	3·3 (3·3 to 3·3)
Djibouti*	2002	63·2 (56·3 to 71·1)	28·0 (23·6 to 33·4)	30·3 (24·5 to 36·3)	0·4 (0·3 to 0·6)	4·6 (4·6 to 4·6)	104·3 (92·8 to 117·4)	46·2 (38·9 to 55·2)	50·0 (40·5 to 59·9)	0·6 (0·4 to 0·9)	7·5 (7·5 to 7·5)
Djibouti*	2003	64·7 (57·2 to 73·1)	30·4 (25·4 to 36·1)	29·8 (24·1 to 35·6)	0·4 (0·3 to 0·6)	4·2 (4·2 to 4·2)	106·8 (94·4 to 120·6)	50·1 (42·0 to 59·6)	49·2 (39·7 to 58·8)	0·6 (0·4 to 0·9)	6·9 (6·9 to 6·9)
Djibouti*	2004	72·3 (64·8 to 80·2)	31·5 (26·4 to 37·4)	28·6 (23·0 to 34·3)	0·4 (0·3 to 0·5)	11·8 (11·8 to 11·8)	119·3 (106·9 to 132·4)	51·9 (43·6 to 61·7)	47·3 (38·0 to 56·6)	0·6 (0·4 to 0·9)	19·5 (19·5 to 19·5)
Djibouti*	2005	78·1 (70·4 to 86·0)	32·0 (27·1 to 37·8)	27·3 (22·1 to 33·0)	0·4 (0·3 to 0·5)	18·4 (18·4 to 18·4)	128·9 (116·2 to 142·0)	52·9 (44·7 to 62·4)	45·0 (36·4 to 54·4)	0·6 (0·4 to 0·8)	30·4 (30·4 to 30·4)
Djibouti*	2006	78·8 (71·1 to 86·9)	33·5 (28·3 to 39·6)	26·0 (21·1 to 31·9)	0·3 (0·2 to 0·5)	18·9 (18·9 to 18·9)	130·0 (117·4 to 143·5)	55·3 (46·7 to 65·3)	42·9 (34·8 to 52·6)	0·5 (0·4 to 0·8)	31·2 (31·2 to 31·2)
Djibouti*	2007	84·6 (76·8 to 93·0)	36·9 (31·1 to 43·6)	24·7 (20·1 to 30·4)	0·3 (0·2 to 0·5)	22·6 (22·6 to 22·6)	139·6 (126·7 to 153·4)	60·8 (51·4 to 71·9)	40·8 (33·1 to 50·2)	0·6 (0·4 to 0·8)	37·3 (37·3 to 37·3)
Djibouti*	2008	84·6 (76·7 to 93·3)	40·6 (34·5 to 47·6)	23·2 (18·8 to 28·6)	0·4 (0·2 to 0·5)	20·5 (20·5 to 20·5)	139·7 (126·6 to 154·0)	67·0 (57·0 to 78·5)	38·3 (31·0 to 47·2)	0·6 (0·4 to 0·8)	33·8 (33·8 to 33·8)
Djibouti*	2009	79·9 (71·8 to 88·0)	41·9 (35·4 to 48·6)	21·4 (17·3 to 26·3)	0·4 (0·2 to 0·5)	16·2 (16·2 to 16·2)	131·9 (118·4 to 145·3)	69·1 (58·4 to 80·3)	35·4 (28·6 to 43·5)	0·6 (0·4 to 0·8)	26·8 (26·8 to 26·8)
Djibouti*	2010	66·5 (59·4 to 74·1)	38·7 (32·8 to 45·1)	19·1 (15·4 to 23·5)	0·4 (0·3 to 0·5)	8·4 (8·4 to 8·4)	109·8 (98·1 to 122·3)	63·8 (54·1 to 74·4)	31·5 (25·4 to 38·8)	0·6 (0·4 to 0·8)	13·8 (13·8 to 13·8)
Djibouti*	2011	68·8 (61·7 to 76·3)	39·5 (33·6 to 46·1)	18·1 (14·5 to 22·4)	0·7 (0·5 to 1·0)	10·5 (10·5 to 10·5)	113·6 (101·9 to 126·0)	65·2 (55·4 to 76·1)	29·9 (24·0 to 37·0)	1·2 (0·9 to 1·7)	17·3 (17·3 to 17·3)
Djibouti*	2012	75·3 (68·3 to 82·7)	39·7 (33·8 to 46·6)	17·2 (13·9 to 21·8)	0·8 (0·6 to 1·1)	17·5 (17·5 to 17·5)	124·3 (112·7 to 136·5)	65·6 (55·8 to 76·8)	28·5 (23·0 to 35·9)	1·3 (1·0 to 1·8)	28·9 (28·9 to 28·9)
Djibouti*	2013	67·3 (60·4 to 74·7)	38·7 (32·9 to 45·1)	16·4 (13·3 to 20·6)	0·8 (0·5 to 1·1)	11·4 (11·4 to 11·4)	111·1 (99·7 to 123·2)	63·9 (54·3 to 74·5)	27·1 (21·9 to 33·9)	1·3 (0·9 to 1·7)	18·8 (18·8 to 18·8)

Djibouti*	2014	65·3 (58·4 to 72·7)	38·8 (32·9 to 45·6)	16·2 (12·9 to 20·1)	0·9 (0·6 to 1·2)	9·5 (9·5 to 9·5)	107·8 (96·4 to 120·0)	64·0 (54·3 to 75·3)	26·7 (21·3 to 33·3)	1·4 (1·0 to 1·9)	15·7 (15·7 to 15·7)
Djibouti*	2015	75·5 (67·2 to 84·3)	44·6 (37·8 to 52·4)	17·1 (13·6 to 21·2)	1·0 (0·7 to 1·4)	13·0 (13·0 to 13·0)	124·7 (110·9 to 139·1)	73·5 (62·4 to 86·5)	28·1 (22·5 to 35·0)	1·6 (1·1 to 2·2)	21·4 (21·4 to 21·4)
Djibouti*	2016	67·7 (60·6 to 75·4)	37·6 (31·9 to 44·3)	16·5 (13·2 to 20·5)	1·0 (0·7 to 1·4)	12·6 (12·6 to 12·6)	111·7 (100·1 to 124·5)	62·0 (52·7 to 73·1)	27·2 (21·7 to 33·9)	1·6 (1·1 to 2·2)	20·8 (20·8 to 20·8)
Djibouti*	2017	58·8 (52·4 to 65·7)	32·5 (27·3 to 38·6)	16·3 (13·0 to 20·2)	1·0 (0·7 to 1·3)	9·1 (9·1 to 9·1)	97·0 (86·5 to 108·4)	53·6 (45·0 to 63·7)	26·8 (21·5 to 33·4)	1·6 (1·1 to 2·2)	15·0 (15·0 to 15·0)
Djibouti*	2018	55·0 (48·5 to 62·5)	32·0 (26·9 to 38·1)	16·8 (13·5 to 21·0)	1·0 (0·7 to 1·4)	5·2 (5·2 to 5·2)	90·8 (80·0 to 103·1)	52·8 (44·4 to 62·9)	27·8 (22·2 to 34·7)	1·7 (1·2 to 2·3)	8·6 (8·6 to 8·6)
Djibouti*	2019	53·6 (47·2 to 60·8)	30·2 (25·4 to 36·2)	17·6 (14·1 to 22·1)	1·1 (0·8 to 1·5)	4·6 (4·5 to 4·7)	88·4 (77·9 to 100·4)	49·9 (41·9 to 59·7)	29·1 (23·3 to 36·4)	1·8 (1·3 to 2·5)	7·6 (7·4 to 7·7)
Djibouti*	2020	53·6 (47·4 to 60·7)	29·6 (24·9 to 35·5)	16·9 (13·5 to 21·1)	1·1 (0·8 to 1·5)	6·1 (6·0 to 6·2)	88·5 (78·3 to 100·2)	48·9 (41·1 to 58·5)	27·8 (22·2 to 34·8)	1·8 (1·2 to 2·5)	10·0 (9·9 to 10·2)
Djibouti*	2021	51·6 (45·6 to 58·2)	28·3 (23·7 to 33·7)	16·3 (13·1 to 20·4)	1·1 (0·7 to 1·5)	5·8 (5·8 to 5·9)	85·1 (75·2 to 96·0)	46·7 (39·1 to 55·6)	26·9 (21·6 to 33·7)	1·8 (1·2 to 2·4)	9·6 (9·5 to 9·8)
Djibouti*	2022	49·7 (43·7 to 56·2)	27·7 (23·1 to 33·1)	16·4 (13·1 to 20·5)	1·1 (0·8 to 1·5)	4·6 (4·4 to 4·9)	82·1 (72·2 to 92·8)	45·7 (38·1 to 54·6)	27·0 (21·6 to 33·8)	1·8 (1·3 to 2·5)	7·6 (7·2 to 8·0)
Djibouti*	2023	50·2 (44·1 to 57·0)	27·9 (23·0 to 33·5)	16·4 (13·2 to 20·6)	1·1 (0·8 to 1·5)	4·8 (4·5 to 5·1)	82·9 (72·8 to 94·1)	46·0 (38·0 to 55·3)	27·1 (21·7 to 34·0)	1·8 (1·3 to 2·6)	7·9 (7·5 to 8·4)
Djibouti*	2024	51·0 (44·6 to 58·2)	28·3 (23·2 to 34·4)	16·5 (13·2 to 20·6)	1·1 (0·8 to 1·6)	5·0 (4·7 to 5·3)	84·1 (73·6 to 96·1)	46·8 (38·3 to 56·7)	27·2 (21·8 to 34·1)	1·9 (1·3 to 2·6)	8·2 (7·7 to 8·8)
Djibouti*	2025	51·4 (44·8 to 58·7)	28·5 (23·2 to 34·6)	16·6 (13·3 to 20·7)	1·2 (0·8 to 1·6)	5·2 (4·8 to 5·6)	84·9 (74·0 to 96·8)	47·0 (38·3 to 57·1)	27·3 (21·9 to 34·2)	1·9 (1·4 to 2·7)	8·5 (8·0 to 9·2)
Djibouti*	2026	52·0 (45·2 to 59·5)	28·8 (23·2 to 35·1)	16·6 (13·3 to 20·8)	1·2 (0·9 to 1·7)	5·4 (5·0 to 5·8)	85·8 (74·5 to 98·1)	47·5 (38·3 to 57·9)	27·4 (22·0 to 34·4)	2·0 (1·4 to 2·8)	8·9 (8·2 to 9·6)
Djibouti*	2027	52·6 (45·5 to 60·6)	29·0 (23·0 to 35·7)	16·7 (13·4 to 20·9)	1·3 (0·9 to 1·8)	5·5 (5·1 to 6·0)	86·7 (75·1 to 100·0)	47·9 (38·0 to 58·9)	27·6 (22·2 to 34·6)	2·1 (1·5 to 2·9)	9·1 (8·4 to 9·9)
Djibouti*	2028	53·1 (45·8 to 61·4)	29·3 (23·2 to 36·7)	16·9 (13·5 to 21·1)	1·3 (0·9 to 1·8)	5·6 (5·2 to 6·2)	87·7 (75·5 to 101·3)	48·4 (38·3 to 60·5)	27·8 (22·3 to 34·8)	2·2 (1·5 to 3·0)	9·3 (8·5 to 10·3)

Djibouti*	2029	53·6 (46·0 to 62·2)	29·5 (23·0 to 37·0)	17·0 (13·7 to 21·3)	1·4 (1·0 to 1·9)	5·8 (5·3 to 6·4)	88·5 (75·9 to 102·6)	48·7 (37·9 to 61·0)	28·0 (22·5 to 35·1)	2·2 (1·6 to 3·1)	9·5 (8·7 to 10·6)
Djibouti*	2030	54·1 (46·3 to 62·8)	29·7 (23·1 to 37·3)	17·1 (13·8 to 21·5)	1·4 (1·0 to 2·0)	5·9 (5·3 to 6·5)	89·3 (76·4 to 103·7)	49·0 (38·1 to 61·5)	28·3 (22·7 to 35·4)	2·3 (1·6 to 3·2)	9·7 (8·8 to 10·8)
Djibouti*	2031	54·7 (46·6 to 63·4)	29·9 (22·9 to 37·9)	17·3 (13·9 to 21·7)	1·5 (1·0 to 2·1)	6·0 (5·4 to 6·7)	90·2 (77·0 to 104·6)	49·3 (37·8 to 62·5)	28·6 (23·0 to 35·8)	2·4 (1·7 to 3·4)	9·9 (8·9 to 11·1)
Djibouti*	2032	55·4 (47·0 to 64·5)	30·3 (23·0 to 38·6)	17·5 (14·1 to 21·9)	1·5 (1·1 to 2·1)	6·0 (5·4 to 6·8)	91·4 (77·6 to 106·4)	50·0 (37·9 to 63·6)	28·9 (23·2 to 36·2)	2·5 (1·8 to 3·5)	10·0 (8·9 to 11·3)
Djibouti*	2033	56·2 (47·5 to 65·6)	30·8 (23·0 to 39·6)	17·7 (14·2 to 22·2)	1·6 (1·1 to 2·3)	6·1 (5·5 to 7·0)	92·8 (78·5 to 108·3)	50·9 (38·0 to 65·4)	29·2 (23·5 to 36·6)	2·7 (1·8 to 3·7)	10·1 (9·0 to 11·5)
Djibouti*	2034	57·1 (48·3 to 66·6)	31·3 (23·3 to 40·3)	17·9 (14·4 to 22·5)	1·7 (1·2 to 2·4)	6·2 (5·5 to 7·0)	94·2 (79·7 to 110·0)	51·7 (38·4 to 66·6)	29·6 (23·7 to 37·1)	2·8 (1·9 to 3·9)	10·2 (9·1 to 11·6)
Djibouti*	2035	58·0 (48·9 to 67·9)	31·9 (23·9 to 40·9)	18·1 (14·6 to 22·8)	1·8 (1·2 to 2·5)	6·2 (5·5 to 7·1)	95·7 (80·8 to 112·1)	52·6 (39·5 to 67·5)	30·0 (24·0 to 37·6)	2·9 (2·0 to 4·1)	10·3 (9·2 to 11·6)
Djibouti*	2036	59·0 (49·6 to 69·2)	32·5 (24·1 to 42·0)	18·4 (14·8 to 23·1)	1·8 (1·3 to 2·6)	6·2 (5·5 to 7·1)	97·4 (81·8 to 114·2)	53·6 (39·8 to 69·4)	30·4 (24·4 to 38·1)	3·0 (2·1 to 4·3)	10·3 (9·1 to 11·8)
Djibouti*	2037	60·1 (50·4 to 70·7)	33·2 (24·8 to 43·0)	18·7 (15·0 to 23·4)	1·9 (1·3 to 2·8)	6·3 (5·6 to 7·2)	99·1 (83·2 to 116·7)	54·7 (41·0 to 71·0)	30·8 (24·8 to 38·7)	3·2 (2·2 to 4·6)	10·4 (9·2 to 11·9)
Djibouti*	2038	61·1 (51·1 to 72·1)	33·8 (25·1 to 44·1)	18·9 (15·2 to 23·8)	2·0 (1·4 to 2·9)	6·3 (5·6 to 7·3)	100·8 (84·3 to 119·0)	55·8 (41·4 to 72·8)	31·2 (25·1 to 39·2)	3·3 (2·3 to 4·8)	10·4 (9·2 to 12·1)
Djibouti*	2039	62·1 (51·5 to 74·0)	34·4 (25·4 to 45·4)	19·2 (15·4 to 24·0)	2·1 (1·4 to 3·1)	6·4 (5·6 to 7·4)	102·4 (85·0 to 122·2)	56·7 (42·0 to 75·0)	31·6 (25·4 to 39·7)	3·5 (2·3 to 5·0)	10·6 (9·3 to 12·2)
Djibouti*	2040	63·0 (52·2 to 75·3)	34·9 (25·6 to 46·4)	19·3 (15·6 to 24·3)	2·2 (1·5 to 3·3)	6·6 (5·7 to 7·7)	103·9 (86·1 to 124·2)	57·5 (42·3 to 76·5)	31·9 (25·7 to 40·1)	3·7 (2·4 to 5·4)	10·9 (9·5 to 12·7)
Djibouti*	2041	63·9 (52·6 to 76·5)	35·3 (25·6 to 47·1)	19·5 (15·7 to 24·5)	2·3 (1·5 to 3·4)	6·8 (5·9 to 7·9)	105·5 (86·8 to 126·2)	58·3 (42·3 to 77·7)	32·2 (25·9 to 40·4)	3·8 (2·5 to 5·6)	11·2 (9·7 to 13·1)
Djibouti*	2042	64·8 (53·6 to 78·3)	35·8 (26·1 to 48·3)	19·6 (15·8 to 24·6)	2·4 (1·6 to 3·6)	7·0 (6·1 to 8·3)	107·0 (88·5 to 129·2)	59·0 (43·0 to 79·6)	32·4 (26·1 to 40·6)	4·0 (2·6 to 5·9)	11·6 (10·0 to 13·7)

Djibouti*	2043	65·7 (53·9 to 79·8)	36·2 (25·8 to 49·1)	19·7 (15·9 to 24·8)	2·5 (1·6 to 3·8)	7·3 (6·3 to 8·7)	108·4 (89·0 to 131·7)	59·7 (42·6 to 81·1)	32·6 (26·2 to 40·9)	4·1 (2·7 to 6·2)	12·1 (10·4 to 14·4)
Djibouti*	2044	66·6 (54·5 to 80·9)	36·5 (25·3 to 50·0)	19·8 (16·0 to 24·9)	2·6 (1·7 to 3·9)	7·7 (6·5 to 9·2)	109·9 (89·9 to 133·6)	60·3 (41·8 to 82·5)	32·7 (26·3 to 41·1)	4·3 (2·8 to 6·5)	12·6 (10·8 to 15·2)
Djibouti*	2045	67·4 (54·9 to 82·7)	36·8 (25·4 to 51·3)	19·9 (16·0 to 25·0)	2·7 (1·7 to 4·1)	8·0 (6·8 to 9·8)	111·3 (90·6 to 136·6)	60·8 (42·0 to 84·6)	32·8 (26·4 to 41·3)	4·5 (2·8 to 6·8)	13·2 (11·2 to 16·1)
Djibouti*	2046	68·3 (55·4 to 82·9)	37·1 (25·9 to 50·8)	19·9 (16·1 to 25·1)	2·8 (1·7 to 4·3)	8·4 (7·1 to 10·3)	112·6 (91·4 to 136·8)	61·2 (42·8 to 83·9)	32·9 (26·5 to 41·5)	4·6 (2·9 to 7·1)	13·9 (11·7 to 17·0)
Djibouti*	2047	69·1 (56·0 to 84·4)	37·3 (25·5 to 51·2)	20·0 (16·1 to 25·1)	2·9 (1·8 to 4·5)	9·0 (7·5 to 11·2)	114·0 (92·4 to 139·3)	61·5 (42·1 to 84·6)	32·9 (26·5 to 41·5)	4·8 (2·9 to 7·4)	14·8 (12·3 to 18·4)
Djibouti*	2048	70·0 (56·4 to 85·6)	37·6 (25·7 to 51·6)	20·0 (16·1 to 25·2)	3·0 (1·8 to 4·7)	9·5 (7·9 to 11·8)	115·6 (93·0 to 141·2)	62·0 (42·4 to 85·1)	33·0 (26·5 to 41·6)	4·9 (3·0 to 7·8)	15·6 (13·0 to 19·4)
Djibouti*	2049	70·9 (57·3 to 86·2)	37·7 (25·5 to 51·6)	20·0 (16·1 to 25·2)	3·1 (1·9 to 4·9)	10·1 (8·3 to 12·8)	117·0 (94·5 to 142·2)	62·3 (42·1 to 85·2)	32·9 (26·5 to 41·6)	5·1 (3·1 to 8·1)	16·7 (13·7 to 21·2)
Djibouti*	2050	71·8 (58·0 to 87·3)	37·9 (25·9 to 51·8)	20·0 (16·1 to 25·2)	3·2 (1·9 to 5·1)	10·8 (8·8 to 13·9)	118·5 (95·8 to 144·1)	62·6 (42·7 to 85·4)	32·9 (26·5 to 41·6)	5·3 (3·1 to 8·4)	17·8 (14·5 to 22·9)
Dominica*	1995	347·7 (323·6 to 373·4)	213·3 (195·4 to 231·1)	133·2 (117·4 to 150·6)	1·2 (0·8 to 1·7)	0·0 (0·0 to 0·0)	545·1 (507·3 to 585·4)	334·4 (306·2 to 362·2)	208·8 (184·1 to 236·0)	1·9 (1·3 to 2·6)	0·0 (0·0 to 0·0)
Dominica*	1996	355·6 (335·5 to 376·8)	219·3 (204·4 to 234·0)	134·9 (122·0 to 149·1)	1·1 (0·8 to 1·6)	0·2 (0·2 to 0·2)	557·4 (525·9 to 590·7)	343·8 (320·4 to 366·9)	211·5 (191·2 to 233·8)	1·8 (1·2 to 2·5)	0·4 (0·4 to 0·4)
Dominica*	1997	369·6 (351·2 to 387·7)	219·1 (204·7 to 232·9)	134·8 (122·7 to 147·4)	1·0 (0·7 to 1·4)	14·6 (14·6 to 14·6)	579·3 (550·5 to 607·8)	343·5 (320·9 to 365·0)	211·4 (192·3 to 231·0)	1·5 (1·0 to 2·2)	22·9 (22·9 to 22·9)
Dominica*	1998	383·6 (364·5 to 401·5)	233·0 (218·7 to 247·6)	138·7 (126·6 to 151·0)	0·8 (0·5 to 1·1)	11·1 (11·1 to 11·1)	601·3 (571·4 to 629·3)	365·2 (342·9 to 388·2)	217·4 (198·4 to 236·7)	1·2 (0·8 to 1·7)	17·5 (17·5 to 17·5)
Dominica*	1999	365·3 (347·8 to 384·4)	227·5 (213·4 to 241·5)	137·2 (125·3 to 149·7)	0·4 (0·3 to 0·5)	0·2 (0·2 to 0·2)	572·7 (545·3 to 602·6)	356·7 (334·5 to 378·6)	215·1 (196·4 to 234·6)	0·6 (0·4 to 0·9)	0·3 (0·3 to 0·3)
Dominica*	2000	353·7 (335·5 to 373·7)	221·0 (206·5 to 236·2)	132·5 (120·5 to 144·9)	0·1 (0·1 to 0·1)	0·1 (0·1 to 0·1)	554·4 (526·0 to 585·8)	346·4 (323·8 to 370·3)	207·8 (188·9 to 227·1)	0·1 (0·1 to 0·2)	0·2 (0·2 to 0·2)

Dominica*	2001	296·8 (280·6 to 313·3)	175·0 (162·7 to 188·2)	121·5 (109·9 to 133·4)	0·0 (0·0 to 0·0)	0·3 (0·3 to 0·3)	465·3 (439·9 to 491·2)	274·4 (255·1 to 294·9)	190·5 (172·3 to 209·1)	0·0 (0·0 to 0·0)	0·4 (0·4 to 0·4)
Dominica*	2002	285·6 (269·5 to 303·4)	165·0 (153·5 to 177·8)	120·4 (108·9 to 131·6)	0·0 (0·0 to 0·0)	0·2 (0·2 to 0·2)	447·7 (422·5 to 475·6)	258·7 (240·6 to 278·6)	188·7 (170·7 to 206·3)	0·0 (0·0 to 0·0)	0·3 (0·3 to 0·3)
Dominica*	2003	307·5 (290·1 to 324·7)	176·2 (163·2 to 189·5)	127·2 (115·5 to 139·6)	0·0 (0·0 to 0·0)	4·1 (4·1 to 4·1)	482·0 (454·7 to 509·0)	276·2 (255·8 to 297·0)	199·5 (181·0 to 218·9)	0·0 (0·0 to 0·0)	6·4 (6·4 to 6·4)
Dominica*	2004	316·5 (299·0 to 333·7)	178·7 (165·9 to 191·5)	132·7 (120·9 to 144·3)	0·0 (0·0 to 0·0)	5·1 (5·1 to 5·1)	496·2 (468·6 to 523·1)	280·1 (260·1 to 300·3)	208·0 (189·5 to 226·2)	0·0 (0·0 to 0·0)	8·1 (8·1 to 8·1)
Dominica*	2005	341·3 (319·6 to 363·5)	186·1 (173·7 to 200·5)	144·0 (128·1 to 162·4)	7·0 (5·0 to 9·5)	4·2 (4·2 to 4·2)	535·0 (501·1 to 569·8)	291·7 (272·3 to 314·3)	225·7 (200·9 to 254·6)	11·0 (7·8 to 14·8)	6·6 (6·6 to 6·6)
Dominica*	2006	350·4 (331·9 to 370·8)	187·6 (176·1 to 201·5)	149·0 (135·2 to 163·8)	6·9 (4·9 to 9·3)	7·0 (7·0 to 7·0)	549·3 (520·3 to 581·2)	294·0 (276·1 to 315·9)	233·5 (211·9 to 256·7)	10·8 (7·7 to 14·6)	11·0 (11·0 to 11·0)
Dominica*	2007	385·1 (366·8 to 404·6)	209·1 (196·3 to 223·6)	164·6 (152·0 to 177·5)	7·0 (4·9 to 9·5)	4·4 (4·4 to 4·4)	603·6 (575·0 to 634·3)	327·8 (307·7 to 350·5)	257·9 (238·3 to 278·3)	10·9 (7·8 to 14·8)	6·9 (6·9 to 6·9)
Dominica*	2008	413·7 (396·4 to 432·3)	223·1 (209·8 to 237·5)	176·7 (164·7 to 189·4)	6·8 (4·9 to 9·2)	7·0 (7·0 to 7·0)	648·5 (621·4 to 677·6)	349·7 (328·8 to 372·3)	277·0 (258·2 to 297·0)	10·7 (7·6 to 14·4)	11·0 (11·0 to 11·0)
Dominica*	2009	420·8 (404·7 to 438·2)	232·2 (220·6 to 245·0)	175·8 (164·9 to 186·7)	6·5 (4·7 to 8·8)	6·2 (6·2 to 6·2)	659·6 (634·5 to 686·8)	364·0 (345·8 to 384·1)	275·6 (258·5 to 292·6)	10·2 (7·4 to 13·9)	9·7 (9·7 to 9·7)
Dominica*	2010	456·3 (444·4 to 469·0)	264·8 (255·9 to 274·1)	176·4 (169·2 to 184·0)	6·6 (4·7 to 9·0)	8·5 (8·5 to 8·5)	715·3 (696·7 to 735·2)	415·0 (401·1 to 429·7)	276·6 (265·3 to 288·4)	10·4 (7·3 to 14·1)	13·3 (13·3 to 13·3)
Dominica*	2011	454·6 (447·8 to 461·3)	268·4 (262·9 to 274·1)	169·3 (166·1 to 172·4)	6·4 (4·6 to 8·8)	10·4 (10·4 to 10·4)	712·6 (702·0 to 723·2)	420·8 (412·1 to 429·6)	265·4 (260·3 to 270·3)	10·1 (7·2 to 13·8)	16·4 (16·4 to 16·4)
Dominica*	2012	470·5 (458·2 to 482·8)	290·6 (280·0 to 300·7)	168·2 (161·4 to 175·4)	6·5 (4·7 to 8·9)	5·1 (5·1 to 5·1)	737·5 (718·3 to 756·8)	455·5 (439·0 to 471·3)	263·7 (252·9 to 275·0)	10·2 (7·3 to 14·0)	8·0 (8·0 to 8·0)
Dominica*	2013	458·0 (442·2 to 473·4)	282·4 (270·8 to 293·7)	159·7 (149·1 to 170·0)	6·3 (4·5 to 8·4)	9·7 (9·7 to 9·7)	717·9 (693·2 to 742·1)	442·6 (424·5 to 460·4)	250·3 (233·7 to 266·5)	9·8 (7·0 to 13·2)	15·1 (15·1 to 15·1)
Dominica*	2014	446·4 (431·2 to 463·0)	281·7 (272·4 to 291·4)	156·1 (143·8 to 168·8)	6·2 (4·4 to 8·2)	2·5 (2·5 to 2·5)	699·8 (675·9 to 725·7)	441·5 (427·1 to 456·7)	244·7 (225·5 to 264·6)	9·6 (6·9 to 12·9)	3·9 (3·9 to 3·9)

Dominica*	2015	435·5 (421·1 to 449·4)	274·0 (268·0 to 280·4)	149·0 (136·1 to 162·5)	5·8 (4·1 to 7·9)	6·6 (6·6 to 6·6)	682·7 (660·0 to 704·5)	429·6 (420·1 to 439·6)	233·6 (213·3 to 254·8)	9·1 (6·5 to 12·4)	10·4 (10·4 to 10·4)
Dominica*	2016	467·2 (449·9 to 483·8)	309·1 (298·8 to 319·1)	151·0 (137·1 to 164·9)	5·9 (4·2 to 8·0)	1·2 (1·2 to 1·2)	732·3 (705·2 to 758·3)	484·5 (468·4 to 500·2)	236·6 (215·0 to 258·5)	9·2 (6·6 to 12·5)	2·0 (2·0 to 2·0)
Dominica*	2017	469·0 (448·5 to 489·1)	313·3 (299·0 to 328·2)	146·9 (133·7 to 161·4)	5·8 (4·2 to 7·7)	3·0 (3·0 to 3·0)	735·2 (703·1 to 766·7)	491·1 (468·7 to 514·5)	230·3 (209·6 to 253·0)	9·0 (6·5 to 12·1)	4·8 (4·8 to 4·8)
Dominica*	2018	512·2 (483·4 to 541·6)	344·3 (321·6 to 366·4)	160·6 (142·2 to 179·9)	5·9 (4·2 to 7·9)	1·4 (1·4 to 1·4)	802·9 (757·7 to 848·9)	539·7 (504·1 to 574·3)	251·7 (222·9 to 282·0)	9·2 (6·7 to 12·4)	2·3 (2·3 to 2·3)
Dominica*	2019	431·2 (402·9 to 460·3)	254·8 (235·4 to 275·1)	168·7 (149·5 to 189·1)	6·3 (4·6 to 8·5)	1·3 (1·3 to 1·3)	675·9 (631·6 to 721·6)	399·5 (369·0 to 431·2)	264·5 (234·3 to 296·4)	9·9 (7·2 to 13·4)	2·0 (2·0 to 2·1)
Dominica*	2020	417·3 (390·2 to 445·5)	254·4 (235·0 to 274·5)	155·0 (137·4 to 173·8)	6·0 (4·3 to 8·1)	2·0 (1·9 to 2·1)	654·1 (611·7 to 698·3)	398·7 (368·3 to 430·4)	242·9 (215·4 to 272·4)	9·3 (6·7 to 12·6)	3·1 (3·0 to 3·2)
Dominica*	2021	334·9 (311·1 to 359·8)	179·6 (162·6 to 196·4)	147·5 (130·8 to 165·4)	5·8 (4·2 to 7·8)	2·1 (2·0 to 2·2)	525·0 (487·7 to 564·1)	281·5 (254·8 to 307·8)	231·2 (205·0 to 259·2)	9·1 (6·6 to 12·3)	3·2 (3·2 to 3·5)
Dominica*	2022	338·9 (313·1 to 365·1)	182·1 (162·8 to 202·0)	149·2 (132·5 to 167·3)	6·0 (4·3 to 8·1)	1·6 (1·5 to 1·8)	531·3 (490·8 to 572·3)	285·5 (255·2 to 316·7)	233·8 (207·7 to 262·2)	9·4 (6·8 to 12·7)	2·5 (2·4 to 2·8)
Dominica*	2023	342·6 (315·1 to 369·9)	183·8 (165·1 to 203·7)	150·9 (134·1 to 169·3)	6·2 (4·5 to 8·4)	1·6 (1·5 to 1·8)	537·1 (494·0 to 579·9)	288·1 (258·8 to 319·3)	236·6 (210·2 to 265·3)	9·8 (7·0 to 13·2)	2·6 (2·4 to 2·8)
Dominica*	2024	349·4 (321·3 to 379·7)	188·6 (167·7 to 210·6)	152·8 (135·7 to 171·3)	6·5 (4·7 to 8·7)	1·7 (1·6 to 1·9)	547·8 (503·7 to 595·2)	295·6 (262·9 to 330·2)	239·4 (212·8 to 268·5)	10·1 (7·3 to 13·7)	2·6 (2·4 to 2·9)
Dominica*	2025	355·2 (325·4 to 388·6)	192·3 (169·4 to 217·8)	154·5 (137·3 to 173·3)	6·7 (4·8 to 9·0)	1·7 (1·6 to 1·9)	556·8 (510·1 to 609·2)	301·4 (265·5 to 341·4)	242·3 (215·3 to 271·7)	10·5 (7·6 to 14·2)	2·7 (2·5 to 3·0)
Dominica*	2026	361·0 (329·0 to 397·0)	196·2 (171·4 to 224·1)	156·2 (138·8 to 175·2)	6·9 (5·0 to 9·3)	1·7 (1·6 to 2·0)	566·0 (515·8 to 622·3)	307·5 (268·7 to 351·3)	244·9 (217·6 to 274·7)	10·8 (7·8 to 14·6)	2·7 (2·5 to 3·1)
Dominica*	2027	366·4 (332·7 to 404·0)	199·8 (173·0 to 229·5)	157·7 (140·0 to 177·0)	7·1 (5·1 to 9·6)	1·8 (1·6 to 2·0)	574·3 (521·5 to 633·4)	313·1 (271·3 to 359·7)	247·2 (219·4 to 277·4)	11·2 (8·1 to 15·1)	2·8 (2·6 to 3·2)
Dominica*	2028	371·2 (334·5 to 409·0)	203·0 (174·0 to 233·2)	158·9 (141·2 to 178·3)	7·4 (5·3 to 10·0)	1·8 (1·7 to 2·1)	581·8 (524·3 to 641·2)	318·3 (272·7 to 365·5)	249·1 (221·4 to 279·5)	11·6 (8·3 to 15·6)	2·9 (2·6 to 3·3)

Dominica*	2029	375·5 (336·9 to 416·9)	205·9 (173·5 to 241·5)	160·1 (142·4 to 179·7)	7·6 (5·4 to 10·2)	1·9 (1·7 to 2·2)	588·6 (528·1 to 653·6)	322·8 (272·0 to 378·6)	250·9 (223·2 to 281·6)	11·9 (8·5 to 16·1)	3·0 (2·7 to 3·4)
Dominica*	2030	379·7 (338·7 to 423·4)	208·7 (174·2 to 247·2)	161·2 (143·2 to 180·7)	7·8 (5·6 to 10·5)	2·0 (1·8 to 2·3)	595·2 (531·0 to 663·7)	327·2 (273·1 to 387·5)	252·6 (224·4 to 283·3)	12·3 (8·8 to 16·5)	3·1 (2·8 to 3·6)
Dominica*	2031	383·4 (340·4 to 428·3)	211·3 (174·3 to 250·2)	162·0 (144·0 to 181·6)	8·0 (5·8 to 10·8)	2·0 (1·8 to 2·4)	600·9 (533·6 to 671·3)	331·2 (273·2 to 392·2)	253·9 (225·7 to 284·7)	12·6 (9·1 to 17·0)	3·2 (2·9 to 3·8)
Dominica*	2032	388·5 (343·5 to 436·1)	215·3 (177·3 to 256·6)	162·8 (144·7 to 182·6)	8·3 (5·9 to 11·2)	2·1 (1·9 to 2·6)	609·0 (538·5 to 683·7)	337·5 (277·9 to 402·2)	255·2 (226·8 to 286·3)	12·9 (9·3 to 17·5)	3·3 (3·0 to 4·0)
Dominica*	2033	392·6 (345·7 to 442·8)	218·7 (177·6 to 265·2)	163·2 (145·0 to 183·1)	8·5 (6·1 to 11·5)	2·2 (2·0 to 2·7)	615·4 (541·9 to 694·2)	342·8 (278·4 to 415·7)	255·9 (227·2 to 287·0)	13·3 (9·6 to 18·0)	3·5 (3·1 to 4·3)
Dominica*	2034	396·5 (349·3 to 449·5)	222·0 (179·8 to 272·4)	163·4 (145·2 to 183·2)	8·7 (6·3 to 11·7)	2·4 (2·1 to 2·9)	621·5 (547·6 to 704·7)	348·0 (281·8 to 426·9)	256·2 (227·6 to 287·2)	13·6 (9·9 to 18·4)	3·7 (3·3 to 4·6)
Dominica*	2035	400·3 (348·9 to 455·4)	225·3 (180·3 to 275·8)	163·6 (145·4 to 183·5)	8·9 (6·4 to 12·0)	2·5 (2·2 to 3·1)	627·5 (546·9 to 713·9)	353·1 (282·7 to 432·3)	256·5 (227·9 to 287·6)	13·9 (10·1 to 18·7)	3·9 (3·5 to 4·9)
Dominica*	2036	403·9 (352·5 to 462·1)	228·2 (181·1 to 281·2)	163·9 (145·6 to 183·8)	9·1 (6·6 to 12·3)	2·6 (2·3 to 3·4)	633·1 (552·5 to 724·4)	357·8 (283·8 to 440·8)	256·9 (228·2 to 288·1)	14·2 (10·3 to 19·3)	4·2 (3·6 to 5·3)
Dominica*	2037	407·6 (354·8 to 467·1)	231·3 (182·2 to 286·0)	164·2 (145·9 to 184·1)	9·3 (6·7 to 12·7)	2·8 (2·4 to 3·6)	639·0 (556·1 to 732·3)	362·6 (285·6 to 448·4)	257·4 (228·7 to 288·6)	14·6 (10·5 to 19·9)	4·4 (3·8 to 5·6)
Dominica*	2038	411·5 (357·4 to 471·0)	234·2 (183·1 to 293·1)	164·8 (146·4 to 184·8)	9·6 (6·9 to 13·1)	2·9 (2·5 to 3·8)	645·0 (560·3 to 738·4)	367·1 (287·0 to 459·4)	258·3 (229·5 to 289·6)	15·0 (10·8 to 20·6)	4·6 (4·0 to 6·0)
Dominica*	2039	415·5 (356·9 to 477·6)	237·1 (184·8 to 296·3)	165·5 (147·0 to 185·6)	9·9 (7·1 to 13·6)	3·1 (2·6 to 4·0)	651·3 (559·4 to 748·7)	371·7 (289·7 to 464·4)	259·4 (230·5 to 291·0)	15·4 (11·1 to 21·3)	4·8 (4·1 to 6·3)
Dominica*	2040	419·4 (360·4 to 479·9)	239·8 (184·3 to 299·6)	166·3 (147·8 to 186·5)	10·1 (7·3 to 14·1)	3·2 (2·7 to 4·2)	657·5 (565·0 to 752·3)	375·8 (288·9 to 469·7)	260·7 (231·7 to 292·4)	15·9 (11·4 to 22·1)	5·0 (4·3 to 6·6)
Dominica*	2041	423·4 (362·5 to 485·6)	242·4 (186·1 to 301·9)	167·2 (148·6 to 187·5)	10·4 (7·5 to 14·5)	3·3 (2·8 to 4·5)	663·7 (568·2 to 761·3)	380·0 (291·7 to 473·2)	262·2 (233·0 to 294·0)	16·4 (11·7 to 22·7)	5·2 (4·4 to 7·0)
Dominica*	2042	427·8 (366·0 to 494·3)	245·3 (188·4 to 307·6)	168·2 (149·5 to 188·5)	10·8 (7·7 to 15·1)	3·5 (2·9 to 4·7)	670·6 (573·8 to 774·8)	384·5 (295·4 to 482·1)	263·7 (234·4 to 295·6)	16·9 (12·0 to 23·6)	5·4 (4·6 to 7·3)

Dominica*	2043	432·3 (370·6 to 498·5)	248·4 (192·0 to 311·2)	169·2 (150·3 to 189·5)	11·1 (7·9 to 15·6)	3·6 (3·0 to 5·0)	677·6 (580·9 to 781·5)	389·4 (301·0 to 487·8)	265·2 (235·6 to 297·1)	17·4 (12·4 to 24·4)	5·7 (4·7 to 7·8)
Dominica*	2044	436·8 (375·0 to 505·3)	251·4 (194·5 to 316·4)	170·2 (151·2 to 190·7)	11·4 (8·1 to 16·2)	3·8 (3·1 to 5·3)	684·8 (587·8 to 792·0)	394·1 (305·0 to 496·0)	266·9 (237·1 to 298·9)	17·9 (12·7 to 25·4)	5·9 (4·9 to 8·3)
Dominica*	2045	441·3 (376·8 to 514·3)	254·4 (194·2 to 324·3)	171·2 (152·0 to 191·8)	11·8 (8·3 to 16·8)	3·9 (3·3 to 5·6)	691·8 (590·6 to 806·2)	398·7 (304·4 to 508·3)	268·4 (238·2 to 300·7)	18·4 (13·0 to 26·3)	6·2 (5·1 to 8·7)
Dominica*	2046	445·9 (380·4 to 521·5)	257·5 (197·0 to 329·9)	172·2 (152·7 to 192·9)	12·1 (8·5 to 17·3)	4·1 (3·4 to 5·9)	699·1 (596·3 to 817·5)	403·6 (308·8 to 517·1)	269·9 (239·4 to 302·4)	19·0 (13·3 to 27·2)	6·5 (5·3 to 9·3)
Dominica*	2047	451·2 (382·6 to 526·7)	261·2 (198·3 to 336·8)	173·2 (153·5 to 194·0)	12·5 (8·7 to 17·9)	4·3 (3·5 to 6·3)	707·3 (599·8 to 825·6)	409·4 (310·9 to 528·0)	271·5 (240·6 to 304·1)	19·6 (13·6 to 28·1)	6·8 (5·5 to 9·9)
Dominica*	2048	456·3 (386·8 to 538·7)	264·7 (200·0 to 344·2)	174·1 (154·1 to 195·0)	12·8 (8·9 to 18·5)	4·6 (3·7 to 6·7)	715·2 (606·4 to 844·5)	415·0 (313·6 to 539·5)	272·9 (241·6 to 305·6)	20·1 (14·0 to 29·0)	7·1 (5·8 to 10·5)
Dominica*	2049	461·4 (390·0 to 543·3)	268·4 (200·8 to 348·0)	174·9 (154·8 to 195·9)	13·2 (9·2 to 19·0)	4·8 (3·8 to 7·2)	723·2 (611·4 to 851·7)	420·8 (314·8 to 545·6)	274·2 (242·6 to 307·1)	20·7 (14·4 to 29·8)	7·5 (6·0 to 11·2)
Dominica*	2050	466·5 (391·4 to 550·4)	272·2 (204·2 to 357·2)	175·7 (155·4 to 196·7)	13·6 (9·4 to 19·5)	5·1 (4·1 to 7·7)	731·2 (613·5 to 862·7)	426·6 (320·2 to 559·9)	275·4 (243·6 to 308·3)	21·3 (14·7 to 30·6)	8·0 (6·4 to 12·1)
Denmark	1995	3537·2 (3424·4 to 3652·1)	2887·3 (2776·0 to 3001·7)	603·0 (575·9 to 630·8)	46·8 (36·3 to 60·0)	0·0 (0·0 to 0·0)	3497·4 (3385·9 to 3611·0)	2854·9 (2744·8 to 2968·0)	596·3 (569·5 to 623·7)	46·3 (35·9 to 59·4)	0·0 (0·0 to 0·0)
Denmark	1996	3620·3 (3536·4 to 3704·4)	2950·9 (2868·8 to 3032·2)	618·2 (595·9 to 639·4)	51·2 (41·3 to 63·1)	0·0 (0·0 to 0·0)	3579·6 (3496·6 to 3662·7)	2917·7 (2836·6 to 2998·1)	611·3 (589·2 to 632·2)	50·6 (40·8 to 62·4)	0·0 (0·0 to 0·0)
Denmark	1997	3716·8 (3633·2 to 3800·7)	3029·5 (2950·4 to 3112·0)	632·5 (611·8 to 653·0)	54·9 (45·8 to 65·9)	0·0 (0·0 to 0·0)	3675·0 (3592·4 to 3757·9)	2995·4 (2917·2 to 3077·0)	625·3 (604·9 to 645·7)	54·3 (45·3 to 65·2)	0·0 (0·0 to 0·0)
Denmark	1998	3886·6 (3805·2 to 3972·0)	3182·6 (3101·8 to 3267·1)	644·2 (622·9 to 665·9)	59·8 (51·5 to 69·5)	0·0 (0·0 to 0·0)	3842·9 (3762·4 to 3927·3)	3146·8 (3066·9 to 3230·4)	637·0 (615·9 to 658·5)	59·1 (50·9 to 68·7)	0·0 (0·0 to 0·0)
Denmark	1999	4129·2 (4048·0 to 4215·1)	3414·5 (3335·8 to 3496·9)	651·6 (632·8 to 671·9)	63·2 (57·2 to 69·4)	0·0 (0·0 to 0·0)	4082·8 (4002·5 to 4167·7)	3376·1 (3298·3 to 3457·5)	644·3 (625·6 to 664·3)	62·5 (56·6 to 68·6)	0·0 (0·0 to 0·0)
Denmark	2000	4299·2 (4214·3 to 4388·9)	3576·6 (3492·3 to 3663·5)	657·7 (642·8 to 673·9)	64·9 (61·4 to 68·7)	0·0 (0·0 to 0·0)	4250·9 (4166·9 to 4339·5)	3536·4 (3453·0 to 3622·3)	650·3 (635·6 to 666·3)	64·2 (60·7 to 67·9)	0·0 (0·0 to 0·0)

Denmark	2001	4442·4 (4356·0 to 4533·8)	3708·7 (3622·6 to 3798·9)	667·7 (653·5 to 682·2)	66·0 (63·2 to 68·9)	0·0 (0·0 to 0·0)	4392·4 (4307·1 to 4482·8)	3666·9 (3581·8 to 3756·2)	660·2 (646·1 to 674·5)	65·2 (62·5 to 68·1)	0·0 (0·0 to 0·0)
Denmark	2002	4580·8 (4492·8 to 4670·6)	3836·4 (3750·8 to 3925·2)	676·3 (662·0 to 690·4)	68·1 (65·2 to 70·9)	0·0 (0·0 to 0·0)	4529·3 (4442·3 to 4618·1)	3793·3 (3708·6 to 3881·0)	668·7 (654·6 to 682·6)	67·3 (64·5 to 70·1)	0·0 (0·0 to 0·0)
Denmark	2003	4703·7 (4616·5 to 4791·4)	3941·8 (3856·3 to 4029·0)	690·1 (676·8 to 705·0)	71·9 (68·9 to 74·7)	0·0 (0·0 to 0·0)	4650·8 (4564·6 to 4737·6)	3897·5 (3813·0 to 3983·7)	682·3 (669·2 to 697·1)	71·1 (68·2 to 73·8)	0·0 (0·0 to 0·0)
Denmark	2004	4886·2 (4797·5 to 4970·9)	4091·4 (4003·3 to 4178·8)	718·9 (705·0 to 733·4)	75·9 (73·1 to 78·9)	0·0 (0·0 to 0·0)	4831·3 (4743·5 to 4915·0)	4045·4 (3958·3 to 4131·8)	710·8 (697·1 to 725·2)	75·1 (72·3 to 78·0)	0·0 (0·0 to 0·0)
Denmark	2005	5013·3 (4922·8 to 5099·4)	4195·6 (4108·6 to 4281·5)	739·4 (725·9 to 753·8)	78·3 (75·5 to 81·3)	0·0 (0·0 to 0·0)	4956·9 (4867·4 to 5042·1)	4148·4 (4062·4 to 4233·3)	731·0 (717·7 to 745·3)	77·5 (74·6 to 80·4)	0·0 (0·0 to 0·0)
Denmark	2006	5217·3 (5123·0 to 5303·4)	4370·8 (4280·3 to 4455·6)	762·0 (747·8 to 776·2)	84·5 (81·5 to 87·5)	0·0 (0·0 to 0·0)	5158·6 (5065·4 to 5243·7)	4321·7 (4232·2 to 4405·5)	753·4 (739·4 to 767·4)	83·5 (80·5 to 86·6)	0·0 (0·0 to 0·0)
Denmark	2007	5345·8 (5260·3 to 5432·0)	4481·5 (4397·7 to 4567·1)	771·5 (757·3 to 785·5)	92·7 (89·7 to 95·7)	0·0 (0·0 to 0·0)	5285·7 (5201·1 to 5370·9)	4431·1 (4348·2 to 4515·8)	762·8 (748·8 to 776·7)	91·7 (88·7 to 94·7)	0·0 (0·0 to 0·0)
Denmark	2008	5460·6 (5373·2 to 5550·3)	4589·3 (4505·0 to 4675·7)	770·6 (756·5 to 785·0)	100·8 (97·7 to 104·0)	0·0 (0·0 to 0·0)	5399·2 (5312·8 to 5487·9)	4537·6 (4454·4 to 4623·1)	761·9 (747·9 to 776·2)	99·6 (96·6 to 102·9)	0·0 (0·0 to 0·0)
Denmark	2009	5674·6 (5583·5 to 5757·4)	4781·2 (4691·7 to 4862·7)	788·7 (775·0 to 803·0)	104·7 (101·5 to 108·2)	0·0 (0·0 to 0·0)	5610·8 (5520·7 to 5692·7)	4727·4 (4638·9 to 4808·0)	779·8 (766·2 to 794·0)	103·6 (100·4 to 107·0)	0·0 (0·0 to 0·0)
Denmark	2010	5665·9 (5579·7 to 5759·5)	4757·2 (4673·3 to 4846·4)	807·9 (792·7 to 821·8)	100·8 (97·7 to 104·1)	0·0 (0·0 to 0·0)	5602·2 (5517·0 to 5694·7)	4703·7 (4620·7 to 4791·9)	798·8 (783·8 to 812·5)	99·7 (96·6 to 102·9)	0·0 (0·0 to 0·0)
Denmark	2011	5594·9 (5506·3 to 5684·1)	4683·8 (4596·7 to 4769·4)	809·8 (795·2 to 824·1)	101·3 (98·2 to 104·7)	0·0 (0·0 to 0·0)	5532·0 (5444·4 to 5620·1)	4631·1 (4545·0 to 4715·8)	800·7 (786·2 to 814·8)	100·2 (97·1 to 103·6)	0·0 (0·0 to 0·0)
Denmark	2012	5640·4 (5546·4 to 5731·8)	4737·9 (4649·0 to 4828·7)	797·8 (782·4 to 811·5)	104·7 (101·5 to 108·0)	0·0 (0·0 to 0·0)	5576·9 (5484·1 to 5667·3)	4684·6 (4596·7 to 4774·4)	788·8 (773·6 to 802·3)	103·5 (100·4 to 106·8)	0·0 (0·0 to 0·0)
Denmark	2013	5594·4 (5513·5 to 5682·8)	4707·7 (4629·2 to 4795·6)	778·2 (764·8 to 792·3)	108·5 (105·5 to 111·5)	0·0 (0·0 to 0·0)	5531·4 (5451·5 to 5618·9)	4654·8 (4577·1 to 4741·7)	769·4 (756·2 to 783·4)	107·3 (104·3 to 110·2)	0·0 (0·0 to 0·0)
Denmark	2014	5675·7 (5586·3 to 5764·4)	4779·8 (4693·3 to 4864·6)	780·1 (765·7 to 795·0)	115·7 (112·6 to 119·1)	0·0 (0·0 to 0·0)	5611·9 (5523·5 to 5699·6)	4726·1 (4640·5 to 4809·9)	771·4 (757·1 to 786·1)	114·4 (111·3 to 117·7)	0·0 (0·0 to 0·0)

Denmark	2015	5816·1 (5721·5 to 5910·4)	4897·9 (4811·1 to 4994·4)	794·7 (780·9 to 808·3)	123·5 (120·5 to 126·8)	0·0 (0·0 to 0·0)	5750·7 (5657·2 to 5844·0)	4842·8 (4757·0 to 4938·2)	785·7 (772·1 to 799·2)	122·1 (119·1 to 125·4)	0·0 (0·0 to 0·0)
Denmark	2016	5891·0 (5793·8 to 5987·2)	4953·5 (4857·7 to 5047·6)	809·2 (794·4 to 823·3)	128·2 (125·2 to 131·3)	0·0 (0·0 to 0·0)	5824·7 (5728·7 to 5919·9)	4897·8 (4803·1 to 4990·9)	800·1 (785·5 to 814·0)	126·8 (123·8 to 129·8)	0·0 (0·0 to 0·0)
Denmark	2017	5973·6 (5882·8 to 6067·1)	5016·7 (4925·2 to 5109·1)	822·6 (807·4 to 837·7)	134·2 (130·9 to 137·6)	0·0 (0·0 to 0·0)	5906·4 (5816·6 to 5998·8)	4960·3 (4869·8 to 5051·6)	813·3 (798·4 to 828·3)	132·7 (129·4 to 136·0)	0·0 (0·0 to 0·0)
Denmark	2018	6086·0 (5972·2 to 6218·6)	5106·4 (4993·8 to 5237·3)	836·9 (818·1 to 856·9)	142·7 (138·4 to 146·7)	0·0 (0·0 to 0·0)	6017·6 (5905·0 to 6148·6)	5049·0 (4937·6 to 5178·4)	827·5 (808·9 to 847·3)	141·1 (136·9 to 145·0)	0·0 (0·0 to 0·0)
Denmark	2019	6181·5 (6034·9 to 6340·2)	5179·0 (5036·3 to 5335·2)	853·4 (834·4 to 874·0)	149·1 (144·5 to 153·5)	0·0 (0·0 to 0·0)	6112·0 (5967·0 to 6268·9)	5120·8 (4979·6 to 5275·2)	843·8 (825·0 to 864·1)	147·4 (142·8 to 151·8)	0·0 (0·0 to 0·0)
Denmark	2020	6612·7 (6429·0 to 6836·7)	5660·8 (5474·7 to 5883·3)	807·4 (789·7 to 826·8)	144·4 (139·7 to 149·0)	0·0 (0·0 to 0·0)	6538·3 (6356·7 to 6759·8)	5597·2 (5413·1 to 5817·1)	798·3 (780·8 to 817·5)	142·8 (138·1 to 147·4)	0·0 (0·0 to 0·0)
Denmark	2021	6300·4 (6125·4 to 6513·7)	5392·5 (5215·1 to 5604·4)	767·4 (750·4 to 785·5)	140·5 (135·5 to 145·6)	0·0 (0·0 to 0·0)	6229·5 (6056·5 to 6440·5)	5331·9 (5156·5 to 5541·3)	758·7 (742·0 to 776·7)	138·9 (134·0 to 143·9)	0·0 (0·0 to 0·0)
Denmark	2022	5719·5 (5502·5 to 5955·2)	4805·2 (4586·6 to 5038·9)	770·0 (752·6 to 788·3)	144·3 (139·0 to 149·7)	0·0 (0·0 to 0·0)	5655·2 (5440·7 to 5888·3)	4751·2 (4535·0 to 4982·2)	761·3 (744·1 to 779·4)	142·7 (137·4 to 148·0)	0·0 (0·0 to 0·0)
Denmark	2023	5789·6 (5545·4 to 6055·5)	4869·1 (4626·6 to 5132·8)	772·3 (754·5 to 790·5)	148·2 (142·4 to 154·0)	0·0 (0·0 to 0·0)	5724·5 (5483·0 to 5987·4)	4814·4 (4574·5 to 5075·1)	763·6 (746·0 to 781·6)	146·5 (140·8 to 152·2)	0·0 (0·0 to 0·0)
Denmark	2024	5870·4 (5611·5 to 6153·6)	4943·5 (4683·2 to 5219·1)	774·7 (757·0 to 793·1)	152·1 (146·3 to 158·6)	0·0 (0·0 to 0·0)	5804·4 (5548·4 to 6084·4)	4888·0 (4630·5 to 5160·4)	766·0 (748·5 to 784·2)	150·4 (144·6 to 156·8)	0·0 (0·0 to 0·0)
Denmark	2025	5952·3 (5682·7 to 6236·3)	5019·4 (4750·1 to 5297·9)	776·8 (758·9 to 795·3)	156·1 (149·7 to 163·4)	0·0 (0·0 to 0·0)	5885·4 (5618·8 to 6166·1)	4963·0 (4696·6 to 5238·4)	768·0 (750·4 to 786·3)	154·4 (148·0 to 161·6)	0·0 (0·0 to 0·0)
Denmark	2026	6031·7 (5759·8 to 6327·1)	5093·2 (4818·2 to 5390·6)	778·4 (760·5 to 797·2)	160·1 (152·8 to 168·4)	0·0 (0·0 to 0·0)	5963·9 (5695·0 to 6256·0)	5035·9 (4764·0 to 5330·0)	769·6 (752·0 to 788·3)	158·3 (151·1 to 166·5)	0·0 (0·0 to 0·0)
Denmark	2027	6103·3 (5818·7 to 6407·0)	5160·4 (4876·3 to 5464·9)	778·9 (761·0 to 797·7)	164·0 (155·2 to 173·5)	0·0 (0·0 to 0·0)	6034·7 (5753·2 to 6334·9)	5102·4 (4821·5 to 5403·5)	770·1 (752·5 to 788·8)	162·2 (153·5 to 171·6)	0·0 (0·0 to 0·0)
Denmark	2028	6168·4 (5883·9 to 6482·7)	5220·6 (4935·9 to 5536·0)	779·7 (761·6 to 798·5)	168·1 (157·9 to 179·0)	0·0 (0·0 to 0·0)	6099·0 (5817·7 to 6409·8)	5161·9 (4880·4 to 5473·8)	770·9 (753·0 to 789·5)	166·2 (156·1 to 177·0)	0·0 (0·0 to 0·0)

Denmark	2029	6237·6 (5944·3 to 6560·9)	5285·4 (4986·4 to 5606·7)	779·9 (761·7 to 798·8)	172·3 (160·7 to 184·7)	0·0 (0·0 to 0·0)	6167·5 (5877·5 to 6487·1)	5226·0 (4930·4 to 5543·7)	771·2 (753·2 to 789·9)	170·4 (158·9 to 182·7)	0·0 (0·0 to 0·0)
Denmark	2030	6306·9 (5992·4 to 6633·9)	5350·2 (5032·6 to 5673·7)	780·1 (761·8 to 798·9)	176·6 (163·5 to 191·3)	0·0 (0·0 to 0·0)	6236·0 (5925·0 to 6559·3)	5290·1 (4976·0 to 5609·9)	771·3 (753·2 to 790·0)	174·6 (161·6 to 189·1)	0·0 (0·0 to 0·0)
Denmark	2031	6375·5 (6046·6 to 6712·7)	5413·3 (5081·7 to 5750·3)	781·1 (762·8 to 800·0)	181·1 (166·5 to 197·3)	0·0 (0·0 to 0·0)	6303·9 (5978·6 to 6637·2)	5352·4 (5024·5 to 5685·6)	772·4 (754·2 to 791·0)	179·1 (164·6 to 195·1)	0·0 (0·0 to 0·0)
Denmark	2032	6441·6 (6101·5 to 6803·3)	5473·8 (5128·0 to 5835·3)	782·1 (763·8 to 801·0)	185·7 (169·6 to 203·6)	0·0 (0·0 to 0·0)	6369·2 (6032·9 to 6726·8)	5412·2 (5070·3 to 5769·7)	773·3 (755·2 to 792·0)	183·6 (167·7 to 201·4)	0·0 (0·0 to 0·0)
Denmark	2033	6509·3 (6158·6 to 6889·4)	5535·8 (5181·0 to 5913·9)	783·2 (764·7 to 802·4)	190·3 (172·4 to 210·1)	0·0 (0·0 to 0·0)	6436·1 (6089·3 to 6812·0)	5473·5 (5122·8 to 5847·4)	774·4 (756·1 to 793·4)	188·1 (170·4 to 207·8)	0·0 (0·0 to 0·0)
Denmark	2034	6575·9 (6208·9 to 6980·9)	5596·7 (5225·8 to 5998·0)	784·3 (765·7 to 803·8)	194·9 (174·9 to 217·2)	0·0 (0·0 to 0·0)	6501·9 (6139·1 to 6902·4)	5533·8 (5167·0 to 5930·6)	775·5 (757·1 to 794·8)	192·7 (172·9 to 214·7)	0·0 (0·0 to 0·0)
Denmark	2035	6642·0 (6253·7 to 7079·2)	5656·9 (5266·2 to 6088·5)	785·6 (767·0 to 805·2)	199·6 (177·5 to 223·6)	0·0 (0·0 to 0·0)	6567·3 (6183·4 to 6999·6)	5593·2 (5207·0 to 6020·1)	776·7 (758·4 to 796·1)	197·3 (175·5 to 221·1)	0·0 (0·0 to 0·0)
Denmark	2036	6712·0 (6302·6 to 7169·2)	5720·3 (5311·5 to 6164·1)	787·2 (768·4 to 806·8)	204·5 (180·4 to 231·0)	0·0 (0·0 to 0·0)	6636·5 (6231·7 to 7088·6)	5655·9 (5251·7 to 6094·8)	778·4 (759·8 to 797·8)	202·2 (178·4 to 228·4)	0·0 (0·0 to 0·0)
Denmark	2037	6786·3 (6359·3 to 7270·8)	5787·3 (5362·4 to 6257·4)	789·4 (770·6 to 809·1)	209·6 (183·5 to 238·8)	0·0 (0·0 to 0·0)	6710·0 (6287·8 to 7189·0)	5722·2 (5302·1 to 6187·1)	780·6 (761·9 to 800·0)	207·3 (181·4 to 236·1)	0·0 (0·0 to 0·0)
Denmark	2038	6861·2 (6413·8 to 7370·1)	5854·2 (5402·0 to 6356·0)	791·9 (772·9 to 811·8)	215·0 (186·5 to 246·6)	0·0 (0·0 to 0·0)	6784·0 (6341·7 to 7287·3)	5788·4 (5341·2 to 6284·5)	783·0 (764·2 to 802·7)	212·6 (184·4 to 243·8)	0·0 (0·0 to 0·0)
Denmark	2039	6936·3 (6467·0 to 7476·5)	5921·0 (5451·5 to 6453·0)	794·7 (775·3 to 814·8)	220·6 (189·6 to 254·3)	0·0 (0·0 to 0·0)	6858·3 (6394·3 to 7392·4)	5854·4 (5390·2 to 6380·4)	785·8 (766·6 to 805·7)	218·1 (187·5 to 251·4)	0·0 (0·0 to 0·0)
Denmark	2040	7017·2 (6527·1 to 7586·8)	5992·5 (5502·5 to 6552·9)	798·1 (778·4 to 818·5)	226·5 (192·9 to 262·4)	0·0 (0·0 to 0·0)	6938·2 (6453·7 to 7501·5)	5925·1 (5440·6 to 6479·2)	789·2 (769·7 to 809·3)	224·0 (190·7 to 259·4)	0·0 (0·0 to 0·0)
Denmark	2041	7103·2 (6598·1 to 7701·6)	6068·4 (5552·7 to 6645·3)	802·2 (782·3 to 823·0)	232·7 (196·2 to 271·6)	0·0 (0·0 to 0·0)	7023·3 (6523·9 to 7615·0)	6000·1 (5490·3 to 6570·6)	793·2 (773·5 to 813·7)	230·1 (194·0 to 268·5)	0·0 (0·0 to 0·0)
Denmark	2042	7196·7 (6674·1 to 7817·6)	6150·6 (5610·8 to 6747·9)	807·0 (786·9 to 827·8)	239·1 (199·8 to 281·4)	0·0 (0·0 to 0·0)	7115·7 (6599·1 to 7729·7)	6081·4 (5547·7 to 6672·0)	797·9 (778·0 to 818·5)	236·4 (197·6 to 278·2)	0·0 (0·0 to 0·0)

Denmark	2043	7297·0 (6746·0 to 7944·6)	6238·6 (5676·4 to 6860·2)	812·5 (792·6 to 833·9)	245·9 (203·3 to 291·8)	0·0 (0·0 to 0·0)	7214·9 (6670·1 to 7855·3)	6168·5 (5612·5 to 6783·1)	803·4 (783·6 to 824·5)	243·1 (201·0 to 288·6)	0·0 (0·0 to 0·0)
Denmark	2044	7404·0 (6837·4 to 8071·6)	6332·1 (5752·8 to 6977·3)	818·9 (798·7 to 840·4)	253·0 (206·8 to 302·9)	0·0 (0·0 to 0·0)	7320·7 (6760·5 to 7980·8)	6260·9 (5688·1 to 6898·8)	809·7 (789·7 to 831·0)	250·1 (204·5 to 299·5)	0·0 (0·0 to 0·0)
Denmark	2045	7515·8 (6925·8 to 8202·8)	6429·6 (5832·9 to 7093·2)	825·8 (804·4 to 847·5)	260·4 (210·6 to 315·2)	0·0 (0·0 to 0·0)	7431·3 (6847·9 to 8110·5)	6357·3 (5767·3 to 7013·4)	816·5 (795·4 to 838·0)	257·4 (208·2 to 311·6)	0·0 (0·0 to 0·0)
Denmark	2046	7630·2 (7028·3 to 8320·1)	6529·3 (5913·6 to 7202·7)	832·9 (810·5 to 855·1)	268·0 (214·8 to 327·6)	0·0 (0·0 to 0·0)	7544·4 (6949·2 to 8226·5)	6455·9 (5847·1 to 7121·7)	823·5 (801·4 to 845·5)	265·0 (212·4 to 323·9)	0·0 (0·0 to 0·0)
Denmark	2047	7747·1 (7127·7 to 8451·6)	6630·8 (6001·1 to 7319·6)	840·4 (817·1 to 863·3)	276·0 (219·3 to 339·7)	0·0 (0·0 to 0·0)	7660·0 (7047·6 to 8356·5)	6556·2 (5933·6 to 7237·3)	830·9 (807·9 to 853·6)	272·9 (216·8 to 335·9)	0·0 (0·0 to 0·0)
Denmark	2048	7860·6 (7224·9 to 8591·0)	6728·9 (6087·0 to 7432·9)	847·6 (823·9 to 871·9)	284·0 (223·9 to 352·0)	0·0 (0·0 to 0·0)	7772·2 (7143·7 to 8494·4)	6653·3 (6018·5 to 7349·4)	838·1 (814·6 to 862·1)	280·8 (221·4 to 348·1)	0·0 (0·0 to 0·0)
Denmark	2049	7978·4 (7323·0 to 8723·7)	6830·7 (6174·6 to 7549·2)	855·4 (830·6 to 880·7)	292·4 (228·8 to 365·7)	0·0 (0·0 to 0·0)	7888·7 (7240·6 to 8625·6)	6753·9 (6105·2 to 7464·3)	845·7 (821·2 to 870·8)	289·1 (226·2 to 361·6)	0·0 (0·0 to 0·0)
Denmark	2050	8095·1 (7433·2 to 8849·3)	6930·9 (6260·9 to 7664·0)	863·1 (837·2 to 889·8)	301·0 (233·6 to 380·0)	0·0 (0·0 to 0·0)	8004·0 (7349·6 to 8749·8)	6853·0 (6190·5 to 7577·8)	853·4 (827·8 to 879·8)	297·7 (231·0 to 375·7)	0·0 (0·0 to 0·0)
Dominican Republic*	1995	152·2 (137·1 to 168·4)	30·7 (25·4 to 36·4)	102·7 (89·1 to 117·5)	17·3 (12·2 to 23·4)	1·5 (1·5 to 1·5)	384·1 (345·9 to 424·8)	77·4 (64·2 to 91·8)	259·2 (224·7 to 296·4)	43·6 (30·8 to 59·0)	3·8 (3·8 to 3·8)
Dominican Republic*	1996	161·2 (146·7 to 176·7)	34·7 (29·1 to 40·5)	102·4 (90·2 to 115·3)	18·3 (13·1 to 24·8)	5·8 (5·8 to 5·8)	406·6 (370·1 to 445·9)	87·5 (73·3 to 102·3)	258·3 (227·6 to 290·9)	46·1 (33·0 to 62·6)	14·7 (14·7 to 14·7)
Dominican Republic*	1997	168·8 (154·6 to 184·2)	43·2 (36·6 to 50·2)	105·0 (93·9 to 116·7)	17·5 (12·7 to 23·9)	3·2 (3·2 to 3·2)	425·8 (390·1 to 464·8)	108·9 (92·2 to 126·7)	264·9 (236·9 to 294·4)	44·1 (32·0 to 60·2)	8·0 (8·0 to 8·0)
Dominican Republic*	1998	178·3 (164·1 to 193·8)	50·2 (43·2 to 58·4)	104·8 (93·6 to 116·3)	16·7 (12·4 to 23·0)	6·5 (6·5 to 6·5)	449·9 (413·9 to 489·0)	126·7 (109·1 to 147·4)	264·5 (236·2 to 293·4)	42·3 (31·2 to 58·0)	16·5 (16·5 to 16·5)
Dominican Republic*	1999	187·2 (172·7 to 201·4)	57·7 (50·5 to 66·1)	105·1 (93·4 to 116·7)	16·3 (11·9 to 21·8)	8·1 (8·1 to 8·1)	472·3 (435·7 to 508·1)	145·5 (127·5 to 166·7)	265·2 (235·6 to 294·5)	41·2 (29·9 to 55·1)	20·4 (20·4 to 20·4)
Dominican Republic*	2000	186·7 (172·0 to 201·3)	61·7 (54·3 to 69·9)	105·1 (92·9 to 116·7)	15·6 (11·3 to 20·9)	4·3 (4·3 to 4·3)	470·9 (433·9 to 507·8)	155·6 (136·9 to 176·4)	265·1 (234·5 to 294·3)	39·3 (28·5 to 52·8)	11·0 (11·0 to 11·0)

Dominican Republic*	2001	187·7 (173·3 to 202·6)	63·3 (56·0 to 71·3)	105·0 (93·1 to 116·6)	15·3 (11·2 to 20·7)	4·1 (4·1 to 4·1)	473·6 (437·3 to 511·2)	159·8 (141·3 to 179·8)	264·8 (234·8 to 294·3)	38·7 (28·4 to 52·2)	10·2 (10·2 to 10·2)
Dominican Republic*	2002	184·4 (169·8 to 199·2)	61·1 (54·4 to 69·1)	103·9 (92·5 to 115·9)	15·3 (11·2 to 20·8)	4·1 (4·1 to 4·1)	465·2 (428·3 to 502·6)	154·1 (137·2 to 174·4)	262·1 (233·3 to 292·5)	38·7 (28·3 to 52·5)	10·3 (10·3 to 10·3)
Dominican Republic*	2003	183·5 (169·2 to 198·1)	60·0 (52·9 to 68·0)	103·5 (92·3 to 115·6)	15·7 (11·3 to 21·5)	4·3 (4·3 to 4·3)	463·0 (426·9 to 499·9)	151·3 (133·5 to 171·5)	261·2 (232·8 to 291·5)	39·7 (28·6 to 54·4)	10·8 (10·8 to 10·8)
Dominican Republic*	2004	178·0 (163·5 to 191·8)	56·2 (49·1 to 63·7)	99·3 (88·5 to 110·8)	16·1 (11·6 to 21·7)	6·4 (6·4 to 6·4)	448·9 (412·5 to 483·9)	141·9 (123·9 to 160·6)	250·4 (223·4 to 279·6)	40·5 (29·3 to 54·8)	16·1 (16·1 to 16·1)
Dominican Republic*	2005	183·6 (170·0 to 198·2)	59·4 (52·0 to 67·3)	97·4 (87·0 to 108·2)	18·5 (13·7 to 24·7)	8·2 (8·2 to 8·2)	463·2 (428·9 to 500·0)	150·0 (131·2 to 169·8)	245·8 (219·6 to 273·0)	46·7 (34·6 to 62·3)	20·7 (20·7 to 20·7)
Dominican Republic*	2006	195·9 (181·7 to 211·8)	71·4 (62·6 to 80·9)	100·3 (89·6 to 111·4)	18·9 (14·0 to 25·4)	5·3 (5·3 to 5·3)	494·2 (458·4 to 534·4)	180·2 (157·8 to 204·1)	253·0 (226·0 to 281·0)	47·8 (35·4 to 64·1)	13·3 (13·3 to 13·3)
Dominican Republic*	2007	213·7 (198·6 to 231·2)	85·0 (75·1 to 95·5)	102·7 (91·6 to 113·8)	21·1 (15·6 to 27·9)	4·9 (4·9 to 4·9)	539·1 (501·1 to 583·4)	214·5 (189·5 to 240·9)	259·1 (231·0 to 287·2)	53·1 (39·3 to 70·3)	12·4 (12·4 to 12·4)
Dominican Republic*	2008	242·8 (226·0 to 261·2)	105·5 (94·0 to 118·2)	109·3 (97·9 to 121·7)	23·0 (17·2 to 30·5)	5·1 (5·1 to 5·1)	612·7 (570·1 to 658·9)	266·1 (237·1 to 298·1)	275·7 (247·0 to 307·1)	58·0 (43·3 to 76·9)	12·9 (12·9 to 12·9)
Dominican Republic*	2009	259·4 (241·9 to 277·8)	111·2 (99·9 to 122·8)	112·1 (100·5 to 124·0)	23·3 (17·3 to 30·9)	12·9 (12·9 to 12·9)	654·5 (610·2 to 700·8)	280·5 (252·0 to 309·9)	282·7 (253·6 to 313·0)	58·7 (43·6 to 77·9)	32·5 (32·5 to 32·5)
Dominican Republic*	2010	282·3 (264·1 to 301·8)	125·2 (113·3 to 137·2)	121·2 (109·7 to 133·0)	24·5 (18·3 to 31·8)	11·4 (11·4 to 11·4)	712·2 (666·4 to 761·4)	315·8 (285·9 to 346·0)	305·8 (276·8 to 335·5)	61·9 (46·2 to 80·3)	28·7 (28·7 to 28·7)
Dominican Republic*	2011	292·4 (274·7 to 313·3)	135·2 (123·5 to 148·1)	127·3 (115·8 to 139·5)	25·2 (19·0 to 32·6)	4·7 (4·7 to 4·7)	737·8 (693·1 to 790·4)	341·2 (311·6 to 373·5)	321·2 (292·2 to 352·0)	63·6 (47·9 to 82·3)	11·8 (11·8 to 11·8)
Dominican Republic*	2012	334·4 (315·0 to 356·2)	159·3 (145·4 to 174·3)	139·7 (127·8 to 152·8)	28·3 (21·4 to 36·7)	7·2 (7·2 to 7·2)	843·6 (794·7 to 898·6)	401·8 (366·9 to 439·8)	352·4 (322·4 to 385·4)	71·3 (53·9 to 92·6)	18·1 (18·1 to 18·1)
Dominican Republic*	2013	329·6 (310·8 to 350·5)	153·0 (139·6 to 168·4)	141·9 (130·4 to 154·1)	28·4 (21·6 to 36·4)	6·3 (6·3 to 6·3)	831·6 (784·1 to 884·3)	386·0 (352·1 to 424·8)	358·1 (329·0 to 388·7)	71·6 (54·5 to 91·7)	15·9 (15·9 to 15·9)
Dominican Republic*	2014	341·6 (321·8 to 362·0)	155·8 (141·9 to 170·0)	150·0 (138·0 to 164·0)	29·6 (23·1 to 37·7)	6·3 (6·3 to 6·3)	861·9 (811·8 to 913·3)	393·0 (358·0 to 428·8)	378·4 (348·2 to 413·7)	74·7 (58·2 to 95·1)	15·8 (15·8 to 15·8)

Dominican Republic*	2015	381·5 (361·0 to 402·7)	162·1 (148·5 to 176·6)	160·2 (146·8 to 174·5)	31·6 (24·5 to 39·9)	27·6 (27·6 to 27·6)	962·5 (910·8 to 1016·0)	408·9 (374·6 to 445·5)	404·0 (370·5 to 440·3)	79·7 (61·8 to 100·8)	69·7 (69·7 to 69·7)
Dominican Republic*	2016	385·6 (363·6 to 408·3)	173·9 (159·2 to 188·3)	171·6 (157·4 to 187·2)	34·1 (26·4 to 43·5)	6·0 (6·0 to 6·0)	972·8 (917·3 to 1030·1)	438·6 (401·6 to 475·0)	433·0 (397·2 to 472·3)	86·1 (66·7 to 109·8)	15·1 (15·1 to 15·1)
Dominican Republic*	2017	407·2 (383·6 to 430·8)	184·5 (169·0 to 200·4)	181·9 (167·0 to 198·1)	36·0 (28·0 to 46·4)	4·7 (4·7 to 4·7)	1027·2 (967·9 to 1087·0)	465·6 (426·4 to 505·6)	459·0 (421·3 to 499·7)	90·8 (70·6 to 117·1)	11·8 (11·8 to 11·8)
Dominican Republic*	2018	423·1 (395·3 to 451·0)	190·6 (172·3 to 209·0)	189·9 (171·9 to 210·2)	40·4 (31·0 to 52·4)	2·3 (2·3 to 2·3)	1067·4 (997·2 to 1137·9)	480·7 (434·8 to 527·4)	479·0 (433·7 to 530·2)	101·9 (78·3 to 132·2)	5·8 (5·8 to 5·8)
Dominican Republic*	2019	445·6 (415·6 to 475·5)	203·6 (182·3 to 224·7)	196·9 (178·3 to 217·8)	42·9 (33·0 to 55·5)	2·2 (2·2 to 2·2)	1124·2 (1048·5 to 1199·7)	513·6 (460·0 to 567·0)	496·8 (449·9 to 549·4)	108·3 (83·2 to 140·0)	5·5 (5·5 to 5·6)
Dominican Republic*	2020	489·0 (459·9 to 517·6)	265·4 (244·3 to 286·3)	180·0 (163·1 to 198·8)	40·4 (30·8 to 51·8)	3·2 (3·2 to 3·3)	1233·6 (1160·2 to 1305·8)	669·5 (616·4 to 722·3)	454·1 (411·6 to 501·6)	101·8 (77·7 to 130·6)	8·2 (8·0 to 8·4)
Dominican Republic*	2021	429·9 (398·7 to 460·5)	216·4 (191·4 to 240·3)	170·8 (154·8 to 188·7)	39·3 (30·0 to 50·6)	3·4 (3·3 to 3·7)	1084·6 (1005·8 to 1161·8)	545·9 (482·9 to 606·3)	430·9 (390·5 to 476·0)	99·2 (75·7 to 127·6)	8·5 (8·2 to 9·3)
Dominican Republic*	2022	403·1 (371·1 to 435·1)	186·9 (161·1 to 211·3)	172·7 (156·5 to 190·8)	40·8 (31·1 to 53·0)	2·6 (2·4 to 2·9)	1016·9 (936·1 to 1097·6)	471·6 (406·4 to 533·1)	435·8 (394·8 to 481·4)	102·9 (78·5 to 133·6)	6·6 (6·1 to 7·2)
Dominican Republic*	2023	411·4 (376·1 to 445·7)	191·7 (163·4 to 219·7)	174·7 (158·3 to 193·1)	42·4 (32·4 to 55·2)	2·6 (2·4 to 2·9)	1038·0 (948·9 to 1124·5)	483·5 (412·2 to 554·2)	440·9 (399·4 to 487·1)	107·0 (81·7 to 139·2)	6·7 (6·2 to 7·3)
Dominican Republic*	2024	419·7 (383·0 to 456·1)	196·3 (166·2 to 227·7)	176·7 (160·1 to 195·2)	44·0 (33·7 to 57·5)	2·7 (2·5 to 3·0)	1058·9 (966·3 to 1150·8)	495·3 (419·3 to 574·4)	445·7 (403·8 to 492·5)	111·1 (85·0 to 145·1)	6·8 (6·3 to 7·5)
Dominican Republic*	2025	427·9 (388·7 to 468·4)	201·0 (168·8 to 236·8)	178·5 (161·6 to 197·1)	45·7 (35·0 to 59·4)	2·8 (2·5 to 3·1)	1079·6 (980·5 to 1181·7)	507·0 (425·8 to 597·5)	450·2 (407·6 to 497·2)	115·4 (88·4 to 149·9)	7·0 (6·3 to 7·8)
Dominican Republic*	2026	436·7 (393·7 to 480·9)	206·2 (171·0 to 243·8)	180·2 (163·2 to 199·0)	47·5 (36·4 to 61·8)	2·8 (2·5 to 3·1)	1101·7 (993·3 to 1213·3)	520·1 (431·5 to 615·1)	454·7 (411·7 to 502·0)	119·8 (91·8 to 155·8)	7·1 (6·4 to 7·9)
Dominican Republic*	2027	444·8 (400·2 to 490·7)	210·7 (173·7 to 251·3)	181·9 (164·6 to 200·7)	49·3 (37·8 to 64·4)	2·9 (2·6 to 3·2)	1122·1 (1009·6 to 1237·9)	531·7 (438·3 to 634·0)	458·9 (415·3 to 506·4)	124·3 (95·4 to 162·4)	7·2 (6·5 to 8·1)
Dominican Republic*	2028	452·6 (407·4 to 502·2)	215·2 (175·1 to 258·8)	183·4 (166·0 to 202·5)	51·1 (39·0 to 67·3)	2·9 (2·6 to 3·3)	1141·8 (1027·8 to 1267·1)	542·8 (441·7 to 653·0)	462·7 (418·9 to 510·9)	128·9 (98·4 to 169·8)	7·3 (6·6 to 8·3)

Dominican Republic*	2029	461·1 (412·9 to 512·9)	220·5 (178·9 to 265·6)	184·7 (167·2 to 203·8)	53·0 (40·5 to 69·7)	3·0 (2·7 to 3·4)	1163·3 (1041·6 to 1294·0)	556·2 (451·5 to 670·2)	466·0 (421·8 to 514·2)	133·6 (102·1 to 175·8)	7·5 (6·8 to 8·5)
Dominican Republic*	2030	469·2 (419·6 to 521·7)	225·3 (180·5 to 274·6)	185·9 (168·3 to 205·2)	54·9 (41·8 to 72·5)	3·1 (2·7 to 3·4)	1183·7 (1058·5 to 1316·3)	568·4 (455·5 to 692·9)	469·1 (424·6 to 517·7)	138·4 (105·5 to 182·9)	7·7 (6·9 to 8·7)
Dominican Republic*	2031	477·9 (426·0 to 533·0)	230·5 (184·2 to 283·3)	187·3 (169·5 to 206·8)	56·9 (43·4 to 75·9)	3·1 (2·8 to 3·5)	1205·6 (1074·8 to 1344·8)	581·6 (464·7 to 714·8)	472·6 (427·7 to 521·6)	143·5 (109·4 to 191·4)	7·9 (7·0 to 8·9)
Dominican Republic*	2032	487·6 (433·2 to 545·5)	236·4 (187·6 to 289·7)	189·0 (170·9 to 208·5)	59·1 (45·0 to 78·8)	3·2 (2·8 to 3·6)	1230·2 (1092·8 to 1376·2)	596·4 (473·3 to 730·9)	476·8 (431·2 to 526·1)	149·0 (113·6 to 198·7)	8·0 (7·2 to 9·1)
Dominican Republic*	2033	498·0 (438·7 to 560·3)	242·5 (188·6 to 300·9)	190·9 (172·7 to 210·7)	61·4 (46·8 to 81·7)	3·2 (2·9 to 3·6)	1256·5 (1106·7 to 1413·6)	611·8 (475·8 to 759·1)	481·7 (435·7 to 531·6)	154·9 (118·1 to 206·2)	8·1 (7·2 to 9·2)
Dominican Republic*	2034	509·5 (448·5 to 574·3)	249·4 (193·1 to 308·8)	193·0 (174·6 to 213·0)	63·8 (48·6 to 84·7)	3·3 (2·9 to 3·7)	1285·4 (1131·6 to 1449·0)	629·3 (487·3 to 779·0)	486·9 (440·5 to 537·4)	161·0 (122·7 to 213·8)	8·3 (7·3 to 9·3)
Dominican Republic*	2035	520·6 (457·0 to 588·8)	255·7 (198·9 to 316·3)	195·1 (176·6 to 215·5)	66·4 (50·6 to 88·0)	3·3 (2·9 to 3·8)	1313·4 (1153·0 to 1485·5)	645·2 (501·8 to 797·9)	492·3 (445·5 to 543·6)	167·5 (127·7 to 222·0)	8·4 (7·4 to 9·5)
Dominican Republic*	2036	532·6 (466·4 to 603·1)	262·7 (203·0 to 326·3)	197·4 (178·6 to 218·1)	69·1 (52·6 to 91·5)	3·4 (3·0 to 3·8)	1343·6 (1176·7 to 1521·5)	662·7 (512·2 to 823·3)	498·1 (450·7 to 550·2)	174·3 (132·6 to 230·9)	8·5 (7·5 to 9·7)
Dominican Republic*	2037	544·8 (475·9 to 619·2)	269·7 (207·3 to 339·4)	199·8 (180·7 to 220·7)	71·9 (54·5 to 95·1)	3·4 (3·0 to 3·9)	1374·5 (1200·6 to 1562·1)	680·3 (523·1 to 856·2)	504·1 (455·9 to 556·8)	181·5 (137·4 to 240·0)	8·6 (7·5 to 9·8)
Dominican Republic*	2038	557·2 (484·2 to 636·3)	276·6 (211·0 to 352·2)	202·2 (182·8 to 223·4)	74·9 (56·5 to 99·1)	3·4 (3·0 to 3·9)	1405·6 (1221·6 to 1605·2)	697·9 (532·3 to 888·7)	510·1 (461·1 to 563·5)	188·9 (142·6 to 250·1)	8·7 (7·6 to 9·9)
Dominican Republic*	2039	569·6 (491·8 to 652·6)	283·8 (214·9 to 362·3)	204·4 (184·6 to 225·6)	77·9 (58·3 to 103·5)	3·5 (3·1 to 4·0)	1436·9 (1240·6 to 1646·6)	716·1 (542·1 to 913·9)	515·6 (465·8 to 569·2)	196·4 (147·1 to 261·2)	8·8 (7·7 to 10·1)
Dominican Republic*	2040	581·7 (501·1 to 667·8)	290·9 (218·6 to 371·7)	206·4 (186·3 to 227·8)	80·9 (60·3 to 108·0)	3·6 (3·1 to 4·1)	1467·5 (1264·2 to 1684·7)	733·8 (551·6 to 937·7)	520·6 (470·1 to 574·8)	204·1 (152·1 to 272·5)	9·0 (7·9 to 10·4)
Dominican Republic*	2041	594·4 (512·9 to 685·9)	298·4 (226·1 to 384·6)	208·3 (188·1 to 230·1)	84·0 (62·2 to 112·7)	3·7 (3·2 to 4·2)	1499·7 (1294·0 to 1730·3)	752·9 (570·5 to 970·4)	525·6 (474·6 to 580·5)	211·9 (157·0 to 284·4)	9·2 (8·0 to 10·6)
Dominican Republic*	2042	607·1 (520·8 to 702·8)	305·9 (230·8 to 392·7)	210·3 (189·9 to 232·3)	87·2 (64·2 to 118·2)	3·7 (3·2 to 4·3)	1531·7 (1313·9 to 1773·1)	771·7 (582·2 to 990·8)	530·5 (479·1 to 586·1)	220·1 (161·9 to 298·2)	9·4 (8·2 to 11·0)

Dominican Republic*	2043	620·3 (530·3 to 719·9)	313·6 (234·1 to 405·9)	212·2 (191·7 to 234·4)	90·6 (66·1 to 122·8)	3·8 (3·3 to 4·5)	1564·8 (1337·9 to 1816·3)	791·2 (590·5 to 1024·1)	535·4 (483·7 to 591·3)	228·5 (166·8 to 309·7)	9·7 (8·3 to 11·3)
Dominican Republic*	2044	634·0 (539·6 to 739·5)	321·8 (237·8 to 418·8)	214·2 (193·5 to 236·4)	94·1 (68·1 to 127·8)	3·9 (3·4 to 4·6)	1599·5 (1361·2 to 1865·6)	811·9 (600·0 to 1056·6)	540·3 (488·3 to 596·4)	237·3 (171·8 to 322·5)	10·0 (8·6 to 11·7)
Dominican Republic*	2045	646·9 (549·1 to 754·4)	329·4 (241·3 to 431·5)	215·9 (195·1 to 238·2)	97·5 (70·3 to 133·0)	4·1 (3·5 to 4·8)	1632·1 (1385·4 to 1903·3)	831·1 (608·8 to 1088·6)	544·6 (492·1 to 600·9)	246·1 (177·4 to 335·6)	10·3 (8·8 to 12·1)
Dominican Republic*	2046	660·4 (560·7 to 770·3)	337·4 (245·3 to 439·2)	217·7 (196·6 to 240·2)	101·2 (72·2 to 138·5)	4·2 (3·6 to 4·9)	1666·1 (1414·6 to 1943·3)	851·1 (618·9 to 1108·0)	549·1 (496·0 to 605·9)	255·3 (182·3 to 349·5)	10·6 (9·1 to 12·4)
Dominican Republic*	2047	673·5 (569·8 to 790·3)	344·8 (250·6 to 453·7)	219·4 (198·2 to 242·1)	104·9 (74·3 to 144·2)	4·3 (3·7 to 5·2)	1699·0 (1437·6 to 1993·8)	870·0 (632·3 to 1144·5)	553·4 (499·9 to 610·8)	264·7 (187·5 to 363·9)	10·9 (9·3 to 13·0)
Dominican Republic*	2048	687·6 (579·7 to 810·0)	353·2 (255·9 to 471·1)	221·1 (199·7 to 244·0)	108·8 (76·5 to 150·3)	4·5 (3·8 to 5·3)	1734·6 (1462·6 to 2043·4)	891·1 (645·6 to 1188·6)	557·8 (503·9 to 615·6)	274·4 (193·0 to 379·3)	11·3 (9·6 to 13·3)
Dominican Republic*	2049	701·3 (589·5 to 831·1)	361·2 (260·3 to 484·1)	222·7 (201·3 to 245·8)	112·7 (78·8 to 157·2)	4·6 (3·9 to 5·5)	1769·3 (1487·4 to 2096·8)	911·3 (656·7 to 1221·3)	561·9 (507·8 to 620·0)	284·4 (198·7 to 396·5)	11·7 (9·9 to 13·9)
Dominican Republic*	2050	715·2 (600·3 to 846·9)	369·2 (266·3 to 496·0)	224·3 (202·7 to 247·4)	116·8 (81·2 to 163·0)	4·8 (4·1 to 5·8)	1804·2 (1514·5 to 2136·5)	931·5 (671·9 to 1251·4)	565·9 (511·3 to 624·2)	294·6 (204·9 to 411·3)	12·2 (10·3 to 14·5)
Algeria*	1995	92·3 (80·9 to 104·3)	65·8 (56·0 to 77·1)	24·5 (19·5 to 29·9)	2·0 (1·4 to 2·8)	0·0 (0·0 to 0·0)	305·9 (268·1 to 345·6)	218·1 (185·5 to 255·4)	81·1 (64·7 to 99·0)	6·7 (4·6 to 9·4)	0·0 (0·0 to 0·0)
Algeria*	1996	90·9 (80·7 to 102·5)	65·1 (56·3 to 75·0)	23·8 (19·1 to 28·7)	2·0 (1·4 to 2·8)	0·0 (0·0 to 0·0)	301·3 (267·5 to 339·6)	215·7 (186·6 to 248·7)	78·9 (63·3 to 95·1)	6·7 (4·7 to 9·4)	0·0 (0·0 to 0·0)
Algeria*	1997	92·9 (83·3 to 104·1)	66·2 (57·6 to 75·4)	24·6 (20·1 to 29·5)	2·1 (1·4 to 2·9)	0·0 (0·0 to 0·0)	307·9 (276·2 to 345·0)	219·4 (191·0 to 249·9)	81·7 (66·5 to 97·8)	6·9 (4·8 to 9·6)	0·1 (0·1 to 0·1)
Algeria*	1998	94·5 (85·1 to 105·2)	67·3 (58·8 to 76·2)	25·0 (20·8 to 29·9)	2·1 (1·4 to 2·9)	0·0 (0·0 to 0·0)	313·2 (281·9 to 348·8)	223·2 (194·8 to 252·4)	83·0 (68·8 to 99·0)	6·9 (4·8 to 9·7)	0·2 (0·2 to 0·2)
Algeria*	1999	95·9 (86·3 to 106·2)	69·0 (60·2 to 78·0)	24·8 (20·8 to 29·1)	2·1 (1·5 to 2·9)	0·1 (0·1 to 0·1)	317·9 (286·0 to 351·9)	228·5 (199·4 to 258·5)	82·2 (68·9 to 96·6)	7·0 (4·8 to 9·6)	0·2 (0·2 to 0·2)
Algeria*	2000	95·8 (86·4 to 106·1)	69·9 (61·0 to 79·8)	23·8 (20·7 to 27·1)	2·1 (1·4 to 2·9)	0·0 (0·0 to 0·0)	317·5 (286·3 to 351·6)	231·6 (202·2 to 264·3)	78·8 (68·5 to 89·7)	7·0 (4·8 to 9·6)	0·1 (0·1 to 0·1)

Algeria*	2001	101·6 (91·3 to 112·6)	75·9 (66·0 to 86·4)	23·5 (21·2 to 25·9)	2·2 (1·5 to 3·1)	0·1 (0·1 to 0·1)	336·9 (302·7 to 373·1)	251·4 (218·9 to 286·4)	77·8 (70·4 to 85·8)	7·4 (5·1 to 10·3)	0·2 (0·2 to 0·2)
Algeria*	2002	108·3 (98·1 to 118·8)	81·9 (71·8 to 92·3)	24·0 (22·3 to 25·7)	2·4 (1·6 to 3·3)	0·1 (0·1 to 0·1)	358·9 (325·1 to 393·8)	271·3 (237·9 to 306·0)	79·6 (74·0 to 85·2)	7·9 (5·4 to 11·0)	0·2 (0·2 to 0·2)
Algeria*	2003	108·8 (98·9 to 119·8)	81·8 (71·9 to 92·7)	24·5 (22·9 to 26·3)	2·4 (1·7 to 3·4)	0·0 (0·0 to 0·0)	360·6 (327·8 to 397·0)	271·1 (238·4 to 307·4)	81·3 (75·9 to 87·2)	8·1 (5·6 to 11·4)	0·1 (0·1 to 0·1)
Algeria*	2004	109·1 (99·1 to 120·1)	80·0 (70·4 to 90·8)	26·4 (24·1 to 28·8)	2·6 (1·8 to 3·7)	0·1 (0·1 to 0·1)	361·5 (328·6 to 398·1)	265·1 (233·4 to 300·9)	87·5 (79·9 to 95·4)	8·7 (6·0 to 12·3)	0·3 (0·3 to 0·3)
Algeria*	2005	107·1 (97·2 to 117·5)	76·4 (68·0 to 85·7)	28·0 (24·9 to 31·5)	2·6 (1·8 to 3·7)	0·1 (0·1 to 0·1)	355·0 (322·2 to 389·4)	253·2 (225·2 to 284·1)	92·9 (82·5 to 104·3)	8·6 (5·8 to 12·3)	0·2 (0·2 to 0·2)
Algeria*	2006	114·9 (104·3 to 125·7)	81·2 (72·4 to 91·1)	31·0 (26·8 to 35·7)	2·6 (1·8 to 3·7)	0·1 (0·1 to 0·1)	380·9 (345·7 to 416·8)	269·2 (239·9 to 301·9)	102·7 (88·8 to 118·2)	8·6 (5·9 to 12·2)	0·4 (0·4 to 0·4)
Algeria*	2007	130·3 (118·8 to 142·7)	92·6 (82·4 to 103·2)	35·0 (29·7 to 40·7)	2·7 (1·8 to 3·8)	0·1 (0·1 to 0·1)	432·0 (393·7 to 473·0)	306·8 (273·2 to 342·0)	116·0 (98·6 to 134·9)	8·8 (6·0 to 12·6)	0·4 (0·4 to 0·4)
Algeria*	2008	149·9 (136·8 to 163·6)	107·4 (96·2 to 118·9)	39·6 (33·3 to 46·0)	2·8 (1·9 to 3·9)	0·2 (0·2 to 0·2)	497·0 (453·5 to 542·2)	355·9 (318·8 to 393·9)	131·3 (110·3 to 152·5)	9·1 (6·2 to 12·9)	0·6 (0·6 to 0·6)
Algeria*	2009	169·5 (155·0 to 184·5)	121·2 (109·6 to 132·6)	45·3 (37·8 to 52·6)	2·9 (2·0 to 4·2)	0·1 (0·1 to 0·1)	561·8 (513·7 to 611·5)	401·8 (363·1 to 439·6)	150·1 (125·2 to 174·5)	9·7 (6·5 to 13·8)	0·2 (0·2 to 0·2)
Algeria*	2010	171·8 (158·1 to 186·8)	121·1 (110·2 to 133·0)	47·8 (40·2 to 55·5)	2·8 (1·9 to 4·0)	0·1 (0·1 to 0·1)	569·5 (524·1 to 619·0)	401·4 (365·3 to 440·7)	158·4 (133·2 to 184·1)	9·3 (6·4 to 13·4)	0·3 (0·3 to 0·3)
Algeria*	2011	190·9 (176·6 to 206·4)	136·0 (123·9 to 148·8)	51·9 (44·2 to 61·0)	2·9 (1·9 to 4·0)	0·2 (0·2 to 0·2)	632·9 (585·4 to 684·2)	450·7 (410·5 to 493·3)	171·9 (146·6 to 202·3)	9·5 (6·4 to 13·4)	0·7 (0·7 to 0·7)
Algeria*	2012	213·7 (197·2 to 230·8)	153·9 (140·7 to 167·6)	56·6 (48·5 to 67·0)	3·0 (2·0 to 4·3)	0·1 (0·1 to 0·1)	708·1 (653·8 to 764·9)	510·2 (466·3 to 555·6)	187·7 (160·6 to 222·1)	9·9 (6·7 to 14·1)	0·3 (0·3 to 0·3)
Algeria*	2013	206·9 (192·3 to 222·3)	145·6 (133·7 to 157·8)	58·2 (49·7 to 68·1)	2·9 (2·0 to 4·2)	0·2 (0·2 to 0·2)	685·7 (637·2 to 736·7)	482·4 (443·1 to 522·9)	192·9 (164·6 to 225·8)	9·8 (6·8 to 13·9)	0·6 (0·6 to 0·6)
Algeria*	2014	230·5 (214·3 to 246·1)	162·9 (150·2 to 177·1)	64·2 (54·9 to 74·0)	3·2 (2·2 to 4·5)	0·1 (0·1 to 0·1)	764·0 (710·3 to 815·6)	540·1 (497·8 to 587·1)	212·9 (181·9 to 245·3)	10·5 (7·4 to 15·0)	0·5 (0·5 to 0·5)

Algeria*	2015	250·7 (233·6 to 267·4)	176·4 (163·7 to 191·0)	70·8 (60·7 to 80·7)	3·4 (2·4 to 4·8)	0·1 (0·1 to 0·1)	830·9 (774·2 to 886·2)	584·8 (542·4 to 633·0)	234·5 (201·3 to 267·4)	11·4 (8·0 to 16·0)	0·2 (0·2 to 0·2)
Algeria*	2016	242·3 (226·4 to 259·2)	165·9 (152·6 to 179·4)	72·9 (63·3 to 83·0)	3·5 (2·5 to 4·9)	0·0 (0·0 to 0·0)	803·1 (750·3 to 859·0)	549·8 (505·9 to 594·6)	241·7 (209·7 to 275·1)	11·5 (8·1 to 16·3)	0·1 (0·1 to 0·1)
Algeria*	2017	234·6 (218·3 to 252·2)	157·2 (144·2 to 171·2)	73·9 (63·0 to 84·6)	3·5 (2·4 to 4·9)	0·0 (0·0 to 0·0)	777·5 (723·5 to 835·7)	521·1 (478·1 to 567·5)	244·8 (208·9 to 280·5)	11·5 (8·1 to 16·3)	0·1 (0·1 to 0·1)
Algeria*	2018	230·4 (210·4 to 251·7)	152·9 (137·9 to 169·9)	74·0 (62·6 to 86·2)	3·5 (2·4 to 4·9)	0·1 (0·1 to 0·1)	763·8 (697·3 to 834·3)	506·8 (457·0 to 563·2)	245·2 (207·6 to 285·8)	11·5 (8·0 to 16·4)	0·3 (0·3 to 0·3)
Algeria*	2019	230·8 (210·1 to 252·9)	154·1 (138·2 to 171·7)	73·1 (61·9 to 85·3)	3·5 (2·4 to 5·0)	0·1 (0·1 to 0·1)	764·9 (696·4 to 838·1)	510·7 (458·0 to 569·1)	242·4 (205·2 to 282·7)	11·6 (8·1 to 16·5)	0·3 (0·3 to 0·3)
Algeria*	2020	227·1 (207·1 to 248·5)	155·9 (140·3 to 173·2)	67·8 (57·5 to 79·1)	3·3 (2·3 to 4·7)	0·1 (0·1 to 0·1)	752·8 (686·4 to 823·7)	516·6 (464·9 to 573·9)	224·8 (190·4 to 262·0)	11·0 (7·7 to 15·6)	0·4 (0·4 to 0·5)
Algeria*	2021	209·6 (189·6 to 230·4)	141·9 (126·5 to 158·6)	64·3 (54·5 to 74·9)	3·2 (2·2 to 4·6)	0·1 (0·1 to 0·1)	694·8 (628·5 to 763·5)	470·5 (419·1 to 525·7)	213·2 (180·6 to 248·2)	10·7 (7·4 to 15·1)	0·5 (0·4 to 0·5)
Algeria*	2022	208·2 (188·4 to 229·6)	140·7 (124·5 to 158·3)	64·1 (54·3 to 74·6)	3·3 (2·3 to 4·6)	0·1 (0·1 to 0·1)	690·0 (624·5 to 760·9)	466·2 (412·5 to 524·6)	212·5 (180·1 to 247·2)	10·9 (7·6 to 15·4)	0·4 (0·3 to 0·4)
Algeria*	2023	209·9 (189·4 to 232·1)	142·4 (125·7 to 160·0)	63·9 (54·2 to 74·4)	3·3 (2·3 to 4·7)	0·1 (0·1 to 0·1)	695·6 (627·7 to 769·1)	472·1 (416·6 to 530·3)	212·0 (179·5 to 246·5)	11·1 (7·7 to 15·7)	0·4 (0·4 to 0·4)
Algeria*	2024	211·5 (190·7 to 234·1)	144·2 (127·2 to 163·0)	63·7 (54·0 to 74·1)	3·4 (2·4 to 4·9)	0·1 (0·1 to 0·1)	701·0 (631·9 to 776·0)	478·1 (421·5 to 540·1)	211·2 (178·9 to 245·6)	11·3 (7·9 to 16·1)	0·4 (0·4 to 0·5)
Algeria*	2025	213·2 (192·0 to 236·3)	146·1 (128·0 to 165·8)	63·5 (53·8 to 73·9)	3·5 (2·4 to 5·0)	0·1 (0·1 to 0·2)	706·7 (636·3 to 783·2)	484·3 (424·3 to 549·7)	210·5 (178·2 to 245·0)	11·5 (8·0 to 16·6)	0·4 (0·4 to 0·5)
Algeria*	2026	215·2 (193·1 to 239·0)	148·2 (129·9 to 168·9)	63·3 (53·6 to 73·8)	3·6 (2·5 to 5·1)	0·1 (0·1 to 0·2)	713·2 (640·1 to 792·0)	491·1 (430·7 to 559·7)	209·9 (177·7 to 244·7)	11·8 (8·2 to 16·9)	0·5 (0·4 to 0·5)
Algeria*	2027	217·3 (194·5 to 241·1)	150·3 (130·7 to 171·5)	63·2 (53·5 to 73·8)	3·6 (2·5 to 5·2)	0·1 (0·1 to 0·2)	720·1 (644·7 to 799·2)	498·1 (433·2 to 568·3)	209·5 (177·3 to 244·6)	12·0 (8·4 to 17·2)	0·5 (0·4 to 0·6)
Algeria*	2028	219·7 (196·8 to 243·9)	152·7 (132·2 to 174·3)	63·1 (53·4 to 73·7)	3·7 (2·6 to 5·3)	0·2 (0·1 to 0·2)	728·3 (652·2 to 808·2)	506·2 (438·1 to 577·7)	209·2 (177·0 to 244·3)	12·3 (8·6 to 17·7)	0·5 (0·4 to 0·6)

Algeria*	2029	222·4 (198·1 to 247·8)	155·4 (134·0 to 178·5)	63·1 (53·3 to 73·8)	3·8 (2·6 to 5·5)	0·2 (0·1 to 0·2)	737·2 (656·5 to 821·2)	515·0 (444·2 to 591·5)	209·0 (176·7 to 244·5)	12·7 (8·8 to 18·2)	0·5 (0·5 to 0·7)
Algeria*	2030	225·4 (200·4 to 251·4)	158·2 (137·0 to 183·1)	63·1 (53·3 to 73·7)	3·9 (2·7 to 5·6)	0·2 (0·1 to 0·2)	746·9 (664·2 to 833·2)	524·3 (454·2 to 606·7)	209·0 (176·7 to 244·3)	13·1 (9·1 to 18·6)	0·6 (0·5 to 0·7)
Algeria*	2031	228·7 (203·3 to 255·7)	161·3 (138·8 to 187·7)	63·1 (53·4 to 73·9)	4·1 (2·8 to 5·8)	0·2 (0·2 to 0·2)	758·0 (673·8 to 847·6)	534·7 (459·9 to 622·2)	209·3 (176·9 to 244·9)	13·5 (9·4 to 19·1)	0·6 (0·5 to 0·7)
Algeria*	2032	232·4 (206·1 to 260·1)	164·8 (141·7 to 191·8)	63·3 (53·5 to 74·1)	4·2 (2·9 to 5·9)	0·2 (0·2 to 0·2)	770·4 (683·2 to 862·0)	546·1 (469·7 to 635·6)	209·8 (177·4 to 245·6)	13·9 (9·7 to 19·7)	0·6 (0·5 to 0·8)
Algeria*	2033	236·7 (209·4 to 265·3)	168·6 (144·4 to 196·3)	63·6 (53·7 to 74·4)	4·3 (3·0 to 6·1)	0·2 (0·2 to 0·2)	784·4 (693·9 to 879·2)	558·7 (478·8 to 650·6)	210·6 (177·9 to 246·5)	14·4 (10·0 to 20·3)	0·6 (0·5 to 0·8)
Algeria*	2034	241·0 (212·7 to 270·5)	172·4 (147·0 to 200·9)	63·9 (53·9 to 74·8)	4·5 (3·1 to 6·4)	0·2 (0·2 to 0·3)	798·8 (704·9 to 896·6)	571·4 (487·3 to 665·9)	211·9 (178·8 to 248·1)	14·9 (10·4 to 21·2)	0·6 (0·5 to 0·8)
Algeria*	2035	245·8 (216·8 to 277·0)	176·6 (149·9 to 206·1)	64·3 (54·2 to 75·4)	4·7 (3·2 to 6·7)	0·2 (0·2 to 0·3)	814·6 (718·5 to 918·1)	585·3 (497·0 to 683·1)	213·2 (179·8 to 250·1)	15·4 (10·7 to 22·2)	0·7 (0·6 to 0·9)
Algeria*	2036	250·7 (220·6 to 282·8)	180·9 (154·4 to 211·6)	64·8 (54·6 to 76·0)	4·8 (3·4 to 7·0)	0·2 (0·2 to 0·3)	830·8 (731·1 to 937·3)	599·5 (511·8 to 701·3)	214·6 (181·0 to 252·0)	16·0 (11·1 to 23·1)	0·7 (0·6 to 0·9)
Algeria*	2037	255·6 (224·6 to 289·4)	185·2 (158·0 to 218·1)	65·2 (55·0 to 76·5)	5·0 (3·5 to 7·2)	0·2 (0·2 to 0·3)	847·3 (744·3 to 959·2)	614·0 (523·7 to 722·9)	216·0 (182·1 to 253·6)	16·6 (11·5 to 24·0)	0·7 (0·6 to 0·9)
Algeria*	2038	260·6 (228·5 to 296·1)	189·6 (161·0 to 223·9)	65·6 (55·3 to 77·0)	5·2 (3·6 to 7·5)	0·2 (0·2 to 0·3)	863·6 (757·5 to 981·3)	628·3 (533·7 to 742·1)	217·4 (183·3 to 255·1)	17·2 (11·9 to 25·0)	0·7 (0·6 to 1·0)
Algeria*	2039	265·4 (232·3 to 303·7)	193·8 (162·5 to 230·1)	66·0 (55·7 to 77·4)	5·4 (3·7 to 7·8)	0·2 (0·2 to 0·3)	879·7 (769·8 to 1006·7)	642·3 (538·6 to 762·6)	218·9 (184·6 to 256·6)	17·8 (12·3 to 26·0)	0·7 (0·6 to 1·0)
Algeria*	2040	270·3 (235·9 to 309·0)	198·1 (166·8 to 234·9)	66·4 (56·0 to 78·0)	5·6 (3·8 to 8·1)	0·2 (0·2 to 0·3)	896·0 (781·9 to 1024·3)	656·6 (552·7 to 778·6)	220·2 (185·7 to 258·4)	18·4 (12·6 to 26·9)	0·7 (0·6 to 1·0)
Algeria*	2041	274·9 (239·3 to 314·0)	202·1 (169·2 to 241·3)	66·8 (56·3 to 78·4)	5·8 (3·9 to 8·4)	0·2 (0·2 to 0·3)	911·1 (793·1 to 1040·6)	669·8 (560·8 to 799·9)	221·4 (186·7 to 259·8)	19·1 (13·0 to 27·9)	0·8 (0·6 to 1·1)
Algeria*	2042	279·7 (242·8 to 320·9)	206·4 (172·2 to 246·2)	67·1 (56·6 to 78·7)	6·0 (4·0 to 8·7)	0·2 (0·2 to 0·3)	927·1 (804·9 to 1063·8)	684·2 (570·9 to 815·9)	222·4 (187·6 to 260·8)	19·7 (13·4 to 28·9)	0·8 (0·7 to 1·1)

Algeria*	2043	284·3 (245·4 to 326·8)	210·5 (173·6 to 252·1)	67·4 (56·8 to 78·9)	6·1 (4·2 to 9·1)	0·3 (0·2 to 0·4)	942·1 (813·5 to 1083·3)	697·7 (575·3 to 835·5)	223·2 (188·3 to 261·5)	20·4 (13·8 to 30·0)	0·8 (0·7 to 1·2)
Algeria*	2044	288·5 (248·4 to 334·1)	214·4 (176·0 to 258·0)	67·5 (56·9 to 79·2)	6·3 (4·3 to 9·4)	0·3 (0·2 to 0·4)	956·1 (823·3 to 1107·5)	710·5 (583·5 to 855·0)	223·7 (188·6 to 262·4)	21·0 (14·1 to 31·1)	0·9 (0·7 to 1·3)
Algeria*	2045	292·4 (251·2 to 340·1)	218·0 (178·5 to 262·6)	67·6 (56·9 to 79·3)	6·5 (4·3 to 9·8)	0·3 (0·2 to 0·4)	969·1 (832·5 to 1127·1)	722·7 (591·7 to 870·5)	223·9 (188·6 to 263·0)	21·7 (14·3 to 32·4)	0·9 (0·7 to 1·4)
Algeria*	2046	296·1 (253·7 to 347·0)	221·5 (181·7 to 270·1)	67·5 (56·8 to 79·5)	6·7 (4·4 to 10·2)	0·3 (0·2 to 0·4)	981·3 (840·9 to 1150·0)	734·1 (602·2 to 895·2)	223·9 (188·3 to 263·4)	22·3 (14·7 to 33·8)	1·0 (0·7 to 1·4)
Algeria*	2047	299·3 (255·9 to 349·8)	224·6 (183·0 to 271·9)	67·5 (56·7 to 79·3)	6·9 (4·6 to 10·5)	0·3 (0·2 to 0·5)	992·1 (848·0 to 1159·4)	744·5 (606·4 to 901·2)	223·6 (187·8 to 262·9)	23·0 (15·1 to 34·9)	1·0 (0·8 to 1·6)
Algeria*	2048	302·6 (257·0 to 355·0)	227·8 (183·6 to 279·8)	67·4 (56·5 to 79·1)	7·1 (4·6 to 10·9)	0·3 (0·2 to 0·5)	1003·1 (851·7 to 1176·7)	755·1 (608·6 to 927·2)	223·3 (187·3 to 262·2)	23·6 (15·4 to 36·0)	1·1 (0·8 to 1·7)
Algeria*	2049	305·6 (259·1 to 359·8)	230·8 (186·0 to 282·2)	67·2 (56·3 to 78·8)	7·3 (4·7 to 11·2)	0·4 (0·3 to 0·5)	1012·9 (858·7 to 1192·4)	764·8 (616·6 to 935·5)	222·6 (186·7 to 261·2)	24·2 (15·6 to 37·1)	1·2 (0·9 to 1·8)
Algeria*	2050	308·2 (260·1 to 362·5)	233·4 (186·2 to 287·6)	66·9 (56·2 to 78·5)	7·5 (4·8 to 11·5)	0·4 (0·3 to 0·6)	1021·4 (862·0 to 1201·6)	773·4 (617·0 to 953·4)	221·8 (186·1 to 260·1)	24·9 (16·0 to 38·3)	1·2 (0·9 to 2·0)
Ecuador*	1995	196·6 (177·1 to 216·2)	108·2 (92·9 to 122·5)	67·5 (56·3 to 80·8)	18·8 (13·1 to 25·3)	2·0 (2·0 to 2·0)	392·5 (353·5 to 431·5)	216·0 (185·4 to 244·5)	134·8 (112·4 to 161·3)	37·6 (26·1 to 50·5)	4·1 (4·1 to 4·1)
Ecuador*	1996	199·8 (181·8 to 218·7)	107·9 (93·8 to 121·3)	70·5 (59·9 to 83·0)	19·0 (13·6 to 25·7)	2·3 (2·3 to 2·3)	398·7 (363·0 to 436·6)	215·3 (187·1 to 242·2)	140·8 (119·6 to 165·7)	38·0 (27·1 to 51·3)	4·7 (4·7 to 4·7)
Ecuador*	1997	197·5 (180·8 to 215·3)	99·0 (87·2 to 111·4)	77·5 (66·8 to 90·0)	18·6 (13·3 to 25·1)	2·3 (2·3 to 2·3)	394·1 (360·8 to 429·8)	197·7 (174·0 to 222·3)	154·7 (133·4 to 179·7)	37·1 (26·6 to 50·1)	4·7 (4·7 to 4·7)
Ecuador*	1998	193·6 (177·1 to 210·7)	85·1 (75·2 to 96·8)	87·3 (76·4 to 100·2)	17·0 (12·0 to 23·3)	4·3 (4·3 to 4·3)	386·5 (353·5 to 420·6)	169·8 (150·0 to 193·2)	174·2 (152·5 to 199·9)	34·0 (23·9 to 46·6)	8·5 (8·5 to 8·5)
Ecuador*	1999	187·7 (172·2 to 205·0)	75·1 (66·0 to 86·1)	96·2 (85·2 to 109·0)	14·0 (9·9 to 19·1)	2·3 (2·3 to 2·3)	374·6 (343·8 to 409·1)	149·9 (131·7 to 171·9)	192·1 (170·0 to 217·5)	28·0 (19·8 to 38·2)	4·6 (4·6 to 4·6)
Ecuador*	2000	178·5 (164·0 to 193·5)	65·1 (56·7 to 74·6)	102·1 (91·4 to 113·8)	8·9 (6·3 to 12·4)	2·3 (2·3 to 2·3)	356·2 (327·3 to 386·2)	130·0 (113·3 to 149·0)	203·9 (182·4 to 227·1)	17·8 (12·5 to 24·8)	4·5 (4·5 to 4·5)

Ecuador*	2001	185·1 (171·6 to 199·3)	62·1 (53·9 to 71·0)	112·7 (102·7 to 123·9)	7·8 (5·4 to 11·0)	2·4 (2·4 to 2·4)	369·4 (342·6 to 397·9)	124·0 (107·5 to 141·7)	225·0 (204·9 to 247·4)	15·6 (10·8 to 21·9)	4·7 (4·7 to 4·7)
Ecuador*	2002	215·1 (201·1 to 230·6)	69·4 (60·4 to 79·0)	134·3 (123·9 to 145·3)	10·2 (7·1 to 14·3)	1·2 (1·2 to 1·2)	429·3 (401·4 to 460·3)	138·5 (120·5 to 157·7)	268·1 (247·2 to 290·0)	20·3 (14·1 to 28·5)	2·4 (2·4 to 2·4)
Ecuador*	2003	241·0 (227·0 to 255·7)	72·6 (63·6 to 82·8)	152·4 (143·1 to 162·0)	13·7 (9·7 to 19·2)	2·3 (2·3 to 2·3)	481·0 (453·1 to 510·4)	144·9 (126·9 to 165·2)	304·2 (285·7 to 323·3)	27·4 (19·3 to 38·3)	4·6 (4·6 to 4·6)
Ecuador*	2004	267·2 (253·6 to 281·4)	80·2 (70·8 to 90·2)	168·1 (159·7 to 177·4)	16·3 (11·5 to 22·2)	2·6 (2·6 to 2·6)	533·3 (506·2 to 561·7)	160·0 (141·3 to 180·0)	335·4 (318·7 to 354·0)	32·6 (23·0 to 44·4)	5·2 (5·2 to 5·2)
Ecuador*	2005	284·7 (271·2 to 299·4)	87·7 (78·2 to 97·9)	176·5 (168·1 to 185·9)	17·7 (12·8 to 23·9)	2·8 (2·8 to 2·8)	568·4 (541·4 to 597·5)	175·1 (156·1 to 195·4)	352·3 (335·4 to 371·0)	35·3 (25·6 to 47·8)	5·7 (5·7 to 5·7)
Ecuador*	2006	289·7 (277·3 to 303·6)	93·7 (84·6 to 103·7)	173·6 (165·7 to 182·0)	18·8 (13·9 to 25·1)	3·6 (3·6 to 3·6)	578·2 (553·6 to 606·1)	187·0 (168·8 to 207·0)	346·5 (330·7 to 363·3)	37·5 (27·8 to 50·2)	7·2 (7·2 to 7·2)
Ecuador*	2007	300·1 (287·1 to 314·4)	107·2 (97·5 to 117·1)	167·2 (159·4 to 176·3)	21·4 (15·8 to 28·5)	4·3 (4·3 to 4·3)	599·0 (573·1 to 627·6)	214·1 (194·6 to 233·8)	333·7 (318·2 to 351·9)	42·7 (31·6 to 56·9)	8·6 (8·6 to 8·6)
Ecuador*	2008	348·0 (332·4 to 364·2)	143·6 (132·7 to 155·4)	175·1 (165·9 to 185·9)	25·2 (18·7 to 33·4)	4·1 (4·1 to 4·1)	694·7 (663·5 to 726·9)	286·6 (264·8 to 310·1)	349·5 (331·2 to 371·1)	50·4 (37·3 to 66·7)	8·2 (8·2 to 8·2)
Ecuador*	2009	349·0 (332·9 to 366·2)	148·3 (138·2 to 159·3)	172·1 (161·4 to 184·7)	25·4 (19·1 to 33·3)	3·1 (3·1 to 3·1)	696·6 (664·4 to 731·0)	295·9 (275·9 to 317·9)	343·6 (322·2 to 368·6)	50·8 (38·1 to 66·4)	6·3 (6·3 to 6·3)
Ecuador*	2010	385·2 (366·8 to 404·9)	172·5 (162·1 to 184·0)	182·3 (169·8 to 195·2)	27·4 (20·7 to 35·6)	3·0 (3·0 to 3·0)	768·8 (732·1 to 808·2)	344·3 (323·6 to 367·3)	363·8 (338·9 to 389·6)	54·7 (41·3 to 71·0)	5·9 (5·9 to 5·9)
Ecuador*	2011	453·2 (433·2 to 474·3)	215·4 (204·8 to 226·7)	203·2 (189·0 to 218·6)	31·2 (24·0 to 40·5)	3·4 (3·4 to 3·4)	904·5 (864·6 to 946·7)	430·0 (408·7 to 452·5)	405·6 (377·3 to 436·4)	62·2 (47·9 to 80·7)	6·7 (6·7 to 6·7)
Ecuador*	2012	495·7 (475·1 to 517·5)	242·6 (231·1 to 254·9)	217·4 (202·2 to 233·6)	33·3 (25·6 to 42·5)	2·4 (2·4 to 2·4)	989·4 (948·3 to 1033·0)	484·2 (461·3 to 508·8)	433·9 (403·7 to 466·2)	66·5 (51·0 to 84·9)	4·8 (4·8 to 4·8)
Ecuador*	2013	539·4 (519·1 to 562·4)	272·6 (260·3 to 286·3)	229·9 (214·7 to 245·9)	34·3 (26·4 to 43·4)	2·5 (2·5 to 2·5)	1076·6 (1036·0 to 1122·6)	544·2 (519·6 to 571·5)	459·0 (428·5 to 490·8)	68·5 (52·8 to 86·6)	4·9 (4·9 to 4·9)
Ecuador*	2014	556·6 (536·2 to 578·9)	283·7 (269·1 to 299·2)	234·3 (221·1 to 248·7)	35·1 (27·6 to 44·1)	3·5 (3·5 to 3·5)	1111·0 (1070·4 to 1155·5)	566·2 (537·2 to 597·2)	467·7 (441·3 to 496·4)	70·1 (55·1 to 88·1)	7·0 (7·0 to 7·0)

Ecuador*	2015	527·4 (507·3 to 547·7)	266·6 (251·1 to 282·8)	224·6 (213·7 to 234·7)	35·3 (27·7 to 44·0)	0·9 (0·9 to 0·9)	1052·8 (1012·6 to 1093·1)	532·2 (501·1 to 564·4)	448·2 (426·5 to 468·5)	70·5 (55·2 to 87·8)	1·9 (1·9 to 1·9)
Ecuador*	2016	507·5 (487·5 to 528·9)	259·6 (243·6 to 277·1)	209·2 (199·4 to 218·7)	37·8 (29·5 to 47·5)	1·0 (1·0 to 1·0)	1012·9 (973·1 to 1055·8)	518·1 (486·1 to 553·0)	417·5 (397·9 to 436·5)	75·4 (58·8 to 94·8)	1·9 (1·9 to 1·9)
Ecuador*	2017	492·1 (470·5 to 514·9)	255·7 (238·8 to 274·2)	198·0 (187·8 to 209·0)	37·4 (28·7 to 48·4)	0·9 (0·9 to 0·9)	982·1 (939·0 to 1027·7)	510·4 (476·6 to 547·3)	395·2 (374·9 to 417·2)	74·7 (57·2 to 96·6)	1·8 (1·8 to 1·8)
Ecuador*	2018	499·3 (470·8 to 528·5)	261·0 (240·2 to 283·0)	194·9 (179·4 to 211·1)	39·2 (29·3 to 51·4)	4·3 (4·3 to 4·3)	996·7 (939·7 to 1054·9)	521·0 (479·4 to 564·9)	389·0 (358·1 to 421·4)	78·2 (58·6 to 102·6)	8·5 (8·5 to 8·5)
Ecuador*	2019	482·7 (454·6 to 512·0)	246·8 (225·6 to 268·6)	191·5 (176·3 to 207·5)	39·6 (29·7 to 52·0)	4·8 (4·7 to 4·8)	963·5 (907·4 to 1021·9)	492·6 (450·4 to 536·1)	382·3 (352·0 to 414·2)	79·1 (59·3 to 103·7)	9·5 (9·4 to 9·7)
Ecuador*	2020	443·6 (416·3 to 471·9)	231·4 (210·1 to 254·4)	168·0 (154·6 to 182·1)	35·7 (26·9 to 46·8)	8·4 (8·0 to 9·1)	885·4 (830·9 to 941·9)	461·9 (419·4 to 507·9)	335·3 (308·7 to 363·5)	71·4 (53·7 to 93·5)	16·8 (16·0 to 18·2)
Ecuador*	2021	416·0 (390·4 to 442·5)	216·1 (196·2 to 237·5)	156·4 (143·9 to 169·5)	34·2 (25·7 to 45·0)	9·3 (8·7 to 10·4)	830·4 (779·1 to 883·2)	431·3 (391·6 to 474·1)	312·3 (287·3 to 338·3)	68·3 (51·3 to 89·8)	18·6 (17·4 to 20·7)
Ecuador*	2022	411·1 (384·0 to 439·3)	210·6 (188·8 to 234·4)	157·9 (145·4 to 171·0)	35·5 (26·5 to 46·6)	7·2 (6·5 to 8·2)	820·6 (766·4 to 876·8)	420·3 (376·9 to 467·9)	315·2 (290·2 to 341·3)	70·8 (53·0 to 92·9)	14·3 (13·0 to 16·3)
Ecuador*	2023	417·6 (389·3 to 448·1)	214·2 (190·4 to 240·3)	159·3 (146·7 to 172·6)	36·8 (27·5 to 48·1)	7·3 (6·6 to 8·4)	833·5 (777·0 to 894·4)	427·5 (380·0 to 479·6)	318·0 (292·9 to 344·5)	73·4 (54·8 to 96·1)	14·6 (13·2 to 16·7)
Ecuador*	2024	425·8 (397·0 to 457·9)	219·6 (195·2 to 246·5)	160·6 (148·0 to 173·8)	38·1 (28·4 to 49·7)	7·5 (6·7 to 8·6)	849·9 (792·5 to 914·0)	438·4 (389·5 to 492·1)	320·6 (295·3 to 346·9)	76·0 (56·7 to 99·3)	15·0 (13·4 to 17·2)
Ecuador*	2025	433·6 (402·5 to 466·9)	224·8 (198·2 to 256·0)	161·8 (148·9 to 175·1)	39·4 (29·4 to 51·7)	7·7 (6·9 to 8·9)	865·5 (803·5 to 932·0)	448·7 (395·6 to 511·0)	322·9 (297·2 to 349·6)	78·7 (58·7 to 103·2)	15·3 (13·7 to 17·7)
Ecuador*	2026	442·0 (409·1 to 476·4)	230·2 (201·6 to 262·3)	163·1 (150·1 to 176·6)	40·8 (30·6 to 53·7)	7·9 (7·0 to 9·1)	882·1 (816·6 to 950·9)	459·4 (402·3 to 523·6)	325·6 (299·5 to 352·4)	81·4 (61·2 to 107·3)	15·7 (13·9 to 18·2)
Ecuador*	2027	450·7 (415·7 to 487·1)	235·9 (205·7 to 270·9)	164·5 (151·4 to 178·1)	42·2 (31·6 to 55·6)	8·0 (7·1 to 9·4)	899·5 (829·8 to 972·2)	470·8 (410·7 to 540·6)	328·3 (302·2 to 355·4)	84·3 (63·0 to 111·1)	16·0 (14·2 to 18·7)
Ecuador*	2028	459·6 (423·5 to 498·2)	241·7 (210·4 to 279·6)	166·0 (152·9 to 179·6)	43·8 (32·5 to 57·8)	8·2 (7·2 to 9·6)	917·4 (845·2 to 994·4)	482·5 (420·0 to 558·2)	331·2 (305·1 to 358·4)	87·4 (64·9 to 115·3)	16·3 (14·4 to 19·2)

Ecuador*	2029	469·0 (431·1 to 509·2)	247·9 (215·3 to 286·4)	167·4 (154·3 to 181·1)	45·4 (33·6 to 60·2)	8·3 (7·3 to 9·9)	936·1 (860·4 to 1016·4)	494·8 (429·7 to 571·6)	334·2 (308·1 to 361·5)	90·5 (67·1 to 120·1)	16·6 (14·5 to 19·7)
Ecuador*	2030	478·8 (439·1 to 520·5)	254·4 (220·7 to 294·9)	168·9 (155·7 to 183·0)	47·0 (34·9 to 63·0)	8·4 (7·4 to 10·1)	955·6 (876·4 to 1039·0)	507·8 (440·6 to 588·6)	337·1 (310·8 to 365·3)	93·8 (69·7 to 125·7)	16·9 (14·7 to 20·1)
Ecuador*	2031	488·3 (446·5 to 533·0)	261·0 (224·2 to 303·9)	170·1 (156·7 to 184·5)	48·7 (36·1 to 65·0)	8·6 (7·5 to 10·4)	974·7 (891·3 to 1063·8)	520·9 (447·5 to 606·6)	339·5 (312·9 to 368·2)	97·1 (72·1 to 129·7)	17·2 (15·0 to 20·7)
Ecuador*	2032	497·7 (453·6 to 543·5)	267·3 (228·4 to 311·4)	171·2 (157·8 to 185·7)	50·4 (37·1 to 67·3)	8·8 (7·6 to 10·6)	993·4 (905·3 to 1084·8)	533·6 (455·8 to 621·6)	341·7 (314·9 to 370·7)	100·5 (74·1 to 134·3)	17·5 (15·2 to 21·2)
Ecuador*	2033	506·7 (460·9 to 553·8)	273·3 (233·1 to 320·1)	172·3 (158·8 to 186·9)	52·1 (38·3 to 69·3)	9·0 (7·8 to 10·9)	1011·3 (920·0 to 1105·4)	545·6 (465·3 to 638·9)	343·9 (316·9 to 373·1)	104·0 (76·5 to 138·4)	17·9 (15·5 to 21·8)
Ecuador*	2034	515·4 (467·9 to 564·1)	279·1 (237·4 to 326·1)	173·3 (159·7 to 188·1)	53·8 (39·3 to 72·3)	9·2 (7·9 to 11·2)	1028·7 (934·0 to 1126·0)	557·0 (473·8 to 650·9)	345·8 (318·7 to 375·5)	107·5 (78·5 to 144·4)	18·4 (15·8 to 22·4)
Ecuador*	2035	524·6 (475·5 to 575·9)	285·3 (242·2 to 337·3)	174·3 (160·6 to 189·2)	55·7 (40·7 to 74·9)	9·4 (8·0 to 11·5)	1047·2 (949·0 to 1149·4)	569·4 (483·4 to 673·2)	347·9 (320·6 to 377·7)	111·1 (81·2 to 149·4)	18·8 (16·1 to 23·0)
Ecuador*	2036	534·0 (483·4 to 588·4)	291·5 (246·2 to 345·1)	175·3 (161·4 to 190·3)	57·5 (42·0 to 77·2)	9·7 (8·3 to 11·9)	1065·8 (964·9 to 1174·4)	581·8 (491·3 to 688·7)	349·9 (322·2 to 379·8)	114·8 (83·9 to 154·2)	19·3 (16·5 to 23·7)
Ecuador*	2037	543·8 (491·0 to 599·6)	298·1 (251·9 to 352·3)	176·3 (162·3 to 191·6)	59·5 (43·3 to 80·1)	9·9 (8·5 to 12·3)	1085·5 (980·0 to 1196·8)	595·1 (502·8 to 703·2)	352·0 (324·0 to 382·3)	118·7 (86·4 to 159·9)	19·7 (16·9 to 24·5)
Ecuador*	2038	553·3 (498·7 to 609·1)	304·3 (255·6 to 360·1)	177·3 (163·2 to 192·6)	61·5 (44·5 to 83·3)	10·1 (8·6 to 12·7)	1104·3 (995·3 to 1215·9)	607·5 (510·2 to 718·7)	353·9 (325·7 to 384·4)	122·7 (88·8 to 166·3)	20·3 (17·2 to 25·3)
Ecuador*	2039	563·5 (508·0 to 621·3)	311·2 (264·1 to 369·1)	178·3 (164·1 to 193·6)	63·5 (45·9 to 85·9)	10·4 (8·8 to 13·0)	1124·8 (1014·0 to 1240·2)	621·2 (527·1 to 736·7)	355·9 (327·5 to 386·5)	126·8 (91·5 to 171·5)	20·8 (17·6 to 26·0)
Ecuador*	2040	573·9 (515·7 to 633·7)	318·1 (267·4 to 376·5)	179·3 (164·9 to 194·8)	65·7 (47·2 to 88·9)	10·7 (9·0 to 13·4)	1145·4 (1029·3 to 1264·8)	635·0 (533·8 to 751·4)	357·9 (329·2 to 388·9)	131·1 (94·2 to 177·5)	21·4 (17·9 to 26·8)
Ecuador*	2041	584·5 (523·7 to 647·0)	325·2 (272·0 to 382·9)	180·3 (165·8 to 196·0)	67·9 (48·5 to 92·2)	11·0 (9·3 to 13·9)	1166·6 (1045·3 to 1291·4)	649·2 (542·8 to 764·2)	359·8 (331·0 to 391·2)	135·6 (96·9 to 184·1)	22·0 (18·5 to 27·8)
Ecuador*	2042	595·0 (531·4 to 659·5)	332·2 (275·2 to 394·4)	181·2 (166·8 to 197·0)	70·2 (49·9 to 96·1)	11·4 (9·5 to 14·4)	1187·6 (1060·7 to 1316·4)	663·1 (549·3 to 787·3)	361·7 (332·9 to 393·2)	140·1 (99·6 to 191·7)	22·7 (18·9 to 28·8)

Ecuador*	2043	605·9 (540·5 to 673·0)	339·5 (280·2 to 403·8)	182·1 (167·8 to 198·0)	72·6 (51·4 to 99·7)	11·7 (9·7 to 14·9)	1209·4 (1078·9 to 1343·4)	677·6 (559·3 to 806·0)	363·5 (334·8 to 395·3)	144·9 (102·6 to 199·0)	23·4 (19·3 to 29·8)
Ecuador*	2044	616·7 (549·0 to 686·9)	346·6 (286·0 to 414·8)	182·9 (168·6 to 198·9)	75·0 (52·7 to 103·1)	12·1 (10·0 to 15·5)	1230·9 (1095·9 to 1371·0)	691·9 (570·8 to 828·0)	365·2 (336·5 to 397·0)	149·7 (105·1 to 205·9)	24·2 (19·9 to 30·9)
Ecuador*	2045	627·8 (558·5 to 702·8)	354·0 (295·9 to 424·1)	183·8 (169·2 to 199·8)	77·5 (54·1 to 107·5)	12·5 (10·1 to 16·1)	1253·1 (1114·8 to 1402·8)	706·6 (590·6 to 846·5)	366·9 (337·8 to 398·9)	154·7 (108·0 to 214·6)	24·9 (20·3 to 32·1)
Ecuador*	2046	639·0 (567·1 to 715·3)	361·5 (299·3 to 433·0)	184·6 (170·0 to 200·6)	80·0 (55·6 to 111·2)	12·9 (10·5 to 16·6)	1275·5 (1132·0 to 1427·8)	721·5 (597·3 to 864·2)	368·5 (339·3 to 400·5)	159·7 (111·0 to 222·0)	25·7 (20·9 to 33·2)
Ecuador*	2047	650·2 (575·3 to 728·8)	368·9 (303·2 to 442·6)	185·3 (170·6 to 201·5)	82·6 (57·1 to 114·9)	13·3 (10·7 to 17·3)	1297·7 (1148·3 to 1454·6)	736·3 (605·2 to 883·4)	369·9 (340·6 to 402·1)	164·9 (114·0 to 229·3)	26·6 (21·4 to 34·5)
Ecuador*	2048	661·4 (583·9 to 744·6)	376·3 (309·5 to 452·7)	186·1 (171·3 to 202·5)	85·3 (58·5 to 118·6)	13·7 (11·1 to 17·7)	1320·2 (1165·5 to 1486·2)	751·1 (617·8 to 903·6)	371·5 (341·8 to 404·1)	170·2 (116·8 to 236·7)	27·4 (22·1 to 35·3)
Ecuador*	2049	672·8 (591·6 to 757·4)	383·8 (315·4 to 464·3)	186·9 (171·9 to 203·3)	88·0 (60·1 to 122·8)	14·2 (11·3 to 18·5)	1343·0 (1180·8 to 1511·8)	766·0 (629·5 to 926·8)	373·0 (343·1 to 405·8)	175·6 (119·9 to 245·1)	28·3 (22·6 to 36·9)
Ecuador*	2050	684·4 (600·2 to 771·4)	391·3 (319·4 to 474·9)	187·7 (172·6 to 204·2)	90·8 (61·8 to 126·9)	14·6 (11·6 to 19·1)	1366·1 (1198·0 to 1539·8)	781·1 (637·5 to 947·8)	374·6 (344·5 to 407·7)	181·3 (123·3 to 253·2)	29·2 (23·2 to 38·1)
Egypt*	1995	89·6 (79·6 to 101·6)	31·8 (26·8 to 37·5)	53·9 (45·3 to 64·3)	1·6 (1·1 to 2·3)	2·2 (2·2 to 2·2)	320·0 (284·2 to 362·8)	113·6 (95·7 to 134·0)	192·7 (161·8 to 229·5)	5·8 (3·9 to 8·2)	7·9 (7·9 to 7·9)
Egypt*	1996	94·4 (84·7 to 106·1)	32·9 (28·0 to 38·4)	57·6 (49·1 to 67·7)	1·8 (1·2 to 2·5)	2·1 (2·1 to 2·1)	337·0 (302·4 to 379·1)	117·4 (100·0 to 137·0)	205·8 (175·5 to 241·7)	6·4 (4·4 to 9·0)	7·5 (7·5 to 7·5)
Egypt*	1997	100·3 (91·1 to 111·4)	34·9 (30·1 to 40·3)	62·3 (54·5 to 71·6)	1·8 (1·2 to 2·5)	1·3 (1·3 to 1·3)	358·4 (325·5 to 397·9)	124·6 (107·4 to 143·8)	222·7 (194·8 to 255·7)	6·3 (4·2 to 8·9)	4·8 (4·8 to 4·8)
Egypt*	1998	107·0 (98·1 to 116·7)	36·6 (32·1 to 41·6)	66·3 (59·0 to 74·0)	2·0 (1·4 to 2·9)	2·0 (2·0 to 2·0)	382·0 (350·4 to 416·7)	130·9 (114·6 to 148·6)	236·7 (210·6 to 264·4)	7·2 (4·8 to 10·3)	7·2 (7·2 to 7·2)
Egypt*	1999	112·2 (104·9 to 120·2)	38·5 (34·3 to 43·0)	70·2 (64·4 to 76·8)	2·2 (1·5 to 3·2)	1·3 (1·3 to 1·3)	400·7 (374·7 to 429·5)	137·5 (122·6 to 153·6)	250·6 (230·1 to 274·2)	7·9 (5·4 to 11·3)	4·7 (4·7 to 4·7)
Egypt*	2000	116·8 (110·3 to 123·5)	39·5 (35·9 to 43·6)	73·4 (68·3 to 78·6)	2·4 (1·7 to 3·5)	1·5 (1·5 to 1·5)	417·3 (394·1 to 441·0)	141·2 (128·2 to 155·9)	262·1 (244·1 to 280·7)	8·7 (6·0 to 12·3)	5·3 (5·3 to 5·3)

Egypt*	2001	123·8 (117·9 to 129·7)	41·6 (38·0 to 45·6)	78·5 (73·9 to 82·8)	2·5 (1·7 to 3·5)	1·3 (1·3 to 1·3)	442·4 (421·2 to 463·3)	148·6 (135·6 to 162·7)	280·2 (264·1 to 295·7)	8·8 (6·0 to 12·5)	4·7 (4·7 to 4·7)
Egypt*	2002	128·5 (122·5 to 134·2)	43·2 (39·8 to 47·0)	81·7 (77·7 to 86·3)	2·5 (1·6 to 3·5)	1·1 (1·1 to 1·1)	458·9 (437·6 to 479·5)	154·5 (142·2 to 167·9)	291·8 (277·4 to 308·2)	8·8 (5·9 to 12·4)	3·8 (3·8 to 3·8)
Egypt*	2003	124·6 (119·3 to 130·1)	41·2 (37·9 to 44·7)	80·3 (76·3 to 84·6)	2·4 (1·6 to 3·4)	0·8 (0·8 to 0·8)	445·1 (426·0 to 464·8)	147·0 (135·4 to 159·5)	286·7 (272·4 to 302·2)	8·5 (5·8 to 12·0)	2·9 (2·9 to 2·9)
Egypt*	2004	121·1 (116·2 to 126·5)	39·8 (36·5 to 43·2)	77·7 (73·7 to 81·7)	2·4 (1·6 to 3·3)	1·3 (1·3 to 1·3)	432·6 (414·9 to 451·9)	142·0 (130·5 to 154·3)	277·5 (263·1 to 291·9)	8·4 (5·8 to 11·9)	4·7 (4·7 to 4·7)
Egypt*	2005	120·0 (114·6 to 125·4)	39·7 (36·6 to 43·2)	76·2 (72·0 to 80·4)	2·5 (1·7 to 3·5)	1·6 (1·6 to 1·6)	428·5 (409·3 to 448·0)	141·9 (130·7 to 154·3)	272·2 (257·2 to 287·1)	8·8 (6·0 to 12·4)	5·6 (5·6 to 5·6)
Egypt*	2006	126·0 (120·2 to 131·7)	44·0 (40·7 to 47·7)	77·1 (72·7 to 81·6)	2·8 (1·9 to 3·9)	2·2 (2·2 to 2·2)	450·2 (429·3 to 470·3)	157·2 (145·5 to 170·2)	275·4 (259·6 to 291·5)	9·8 (6·8 to 13·9)	7·8 (7·8 to 7·8)
Egypt*	2007	121·3 (116·0 to 126·8)	42·1 (39·0 to 45·4)	74·7 (70·3 to 79·1)	3·0 (2·1 to 4·1)	1·4 (1·4 to 1·4)	433·2 (414·5 to 452·9)	150·4 (139·3 to 162·3)	267·0 (251·2 to 282·7)	10·7 (7·5 to 14·7)	5·2 (5·2 to 5·2)
Egypt*	2008	126·1 (120·7 to 131·7)	43·8 (40·5 to 47·3)	76·8 (72·4 to 80·9)	3·5 (2·5 to 4·8)	2·1 (2·1 to 2·1)	450·6 (431·1 to 470·5)	156·4 (144·6 to 168·9)	274·1 (258·6 to 289·1)	12·6 (8·8 to 17·1)	7·5 (7·5 to 7·5)
Egypt*	2009	126·0 (120·8 to 131·5)	43·6 (40·5 to 47·1)	77·4 (73·0 to 81·7)	4·3 (3·0 to 5·7)	0·7 (0·7 to 0·7)	450·2 (431·4 to 469·9)	155·8 (144·6 to 168·2)	276·4 (260·8 to 291·9)	15·4 (10·8 to 20·5)	2·5 (2·5 to 2·5)
Egypt*	2010	127·3 (121·5 to 132·8)	42·7 (39·6 to 46·3)	78·1 (73·9 to 82·4)	5·6 (4·0 to 7·4)	0·8 (0·8 to 0·8)	454·6 (434·0 to 474·5)	152·7 (141·5 to 165·5)	279·1 (263·8 to 294·3)	20·0 (14·2 to 26·5)	2·8 (2·8 to 2·8)
Egypt*	2011	130·7 (124·6 to 136·2)	42·4 (39·3 to 45·9)	79·8 (75·8 to 84·0)	7·9 (5·7 to 10·5)	0·7 (0·7 to 0·7)	466·9 (445·2 to 486·4)	151·3 (140·3 to 163·8)	285·0 (270·6 to 300·0)	28·2 (20·5 to 37·3)	2·3 (2·3 to 2·3)
Egypt*	2012	136·8 (130·4 to 142·8)	42·0 (39·0 to 45·2)	82·3 (78·4 to 86·1)	12·1 (9·0 to 15·9)	0·4 (0·4 to 0·4)	488·5 (465·7 to 510·0)	149·9 (139·2 to 161·3)	293·9 (280·2 to 307·7)	43·3 (32·2 to 56·8)	1·3 (1·3 to 1·3)
Egypt*	2013	149·3 (142·6 to 156·3)	45·9 (43·0 to 48·9)	86·6 (82·7 to 90·8)	16·4 (12·1 to 21·7)	0·4 (0·4 to 0·4)	533·4 (509·5 to 558·4)	164·0 (153·5 to 174·8)	309·5 (295·3 to 324·3)	58·5 (43·1 to 77·4)	1·4 (1·4 to 1·4)
Egypt*	2014	158·1 (150·5 to 165·7)	49·0 (46·4 to 51·9)	90·3 (85·9 to 94·9)	18·3 (13·4 to 24·2)	0·5 (0·5 to 0·5)	564·6 (537·6 to 591·7)	175·0 (165·6 to 185·2)	322·4 (306·9 to 339·1)	65·3 (47·8 to 86·5)	1·9 (1·9 to 1·9)

Egypt*	2015	162·8 (155·3 to 170·0)	50·3 (47·7 to 53·1)	95·4 (91·0 to 100·2)	16·5 (12·2 to 22·0)	0·5 (0·5 to 0·5)	581·5 (554·9 to 607·4)	179·8 (170·4 to 189·8)	340·7 (324·9 to 358·0)	59·1 (43·6 to 78·5)	1·8 (1·8 to 1·8)
Egypt*	2016	170·6 (162·8 to 178·4)	52·4 (49·0 to 56·0)	100·2 (96·1 to 104·8)	17·3 (12·8 to 23·4)	0·6 (0·6 to 0·6)	609·3 (581·5 to 637·2)	187·3 (174·9 to 200·1)	358·0 (343·2 to 374·4)	61·9 (45·8 to 83·5)	2·1 (2·1 to 2·1)
Egypt*	2017	176·0 (168·2 to 184·6)	53·0 (48·7 to 57·6)	103·1 (99·1 to 107·0)	19·0 (13·7 to 26·1)	0·9 (0·9 to 0·9)	628·8 (600·7 to 659·3)	189·3 (173·9 to 205·7)	368·4 (354·0 to 382·3)	67·9 (48·9 to 93·3)	3·2 (3·2 to 3·2)
Egypt*	2018	172·0 (161·9 to 182·4)	50·6 (45·9 to 55·8)	103·1 (95·9 to 110·2)	16·4 (11·7 to 22·4)	1·8 (1·8 to 1·8)	614·2 (578·3 to 651·7)	180·7 (163·9 to 199·4)	368·2 (342·4 to 393·5)	58·7 (41·8 to 80·2)	6·5 (6·5 to 6·5)
Egypt*	2019	172·6 (162·3 to 183·5)	47·9 (43·2 to 53·0)	105·7 (98·2 to 112·9)	17·3 (12·4 to 23·6)	1·8 (1·7 to 1·8)	616·5 (579·8 to 655·3)	171·1 (154·3 to 189·4)	377·5 (350·9 to 403·4)	61·7 (44·2 to 84·3)	6·3 (6·2 to 6·3)
Egypt*	2020	174·6 (164·6 to 185·1)	55·0 (50·3 to 60·0)	100·4 (93·3 to 107·4)	16·9 (12·0 to 23·1)	2·3 (2·3 to 2·4)	623·8 (588·0 to 661·0)	196·3 (179·8 to 214·4)	358·7 (333·4 to 383·6)	60·3 (43·0 to 82·3)	8·4 (8·3 to 8·4)
Egypt*	2021	159·7 (150·0 to 169·9)	43·3 (39·0 to 48·1)	97·3 (90·6 to 104·0)	16·7 (11·9 to 22·9)	2·2 (2·2 to 2·3)	570·3 (535·7 to 606·9)	154·8 (139·2 to 171·8)	347·6 (323·6 to 371·5)	59·8 (42·6 to 81·7)	8·0 (7·9 to 8·2)
Egypt*	2022	161·8 (151·9 to 172·2)	44·5 (40·0 to 49·4)	98·3 (91·5 to 105·0)	17·3 (12·3 to 23·6)	1·7 (1·6 to 1·8)	578·0 (542·5 to 615·2)	158·9 (142·8 to 176·5)	351·2 (326·7 to 375·0)	61·8 (44·1 to 84·4)	6·2 (5·9 to 6·5)
Egypt*	2023	164·3 (154·0 to 175·1)	45·3 (40·7 to 50·4)	99·3 (92·3 to 105·9)	17·9 (12·8 to 24·4)	1·7 (1·6 to 1·8)	586·9 (550·2 to 625·5)	161·9 (145·2 to 180·1)	354·8 (329·8 to 378·4)	63·9 (45·6 to 87·2)	6·2 (5·9 to 6·6)
Egypt*	2024	166·9 (156·4 to 177·9)	46·2 (41·4 to 51·4)	100·4 (93·3 to 107·0)	18·5 (13·2 to 25·3)	1·8 (1·6 to 1·9)	596·3 (558·6 to 635·5)	165·2 (147·8 to 183·8)	358·7 (333·4 to 382·2)	66·2 (47·0 to 90·3)	6·3 (5·9 to 6·7)
Egypt*	2025	169·5 (158·7 to 180·9)	47·1 (42·1 to 52·5)	101·5 (94·3 to 108·1)	19·2 (13·6 to 26·2)	1·8 (1·7 to 1·9)	605·6 (567·0 to 646·0)	168·4 (150·3 to 187·4)	362·5 (336·7 to 386·2)	68·5 (48·6 to 93·6)	6·3 (5·9 to 6·8)
Egypt*	2026	172·4 (161·3 to 184·0)	48·1 (42·9 to 53·6)	102·7 (95·4 to 109·5)	19·8 (14·1 to 27·3)	1·8 (1·6 to 1·9)	615·8 (576·2 to 657·2)	171·9 (153·2 to 191·5)	366·7 (340·7 to 391·0)	70·9 (50·3 to 97·6)	6·3 (5·9 to 6·8)
Egypt*	2027	175·4 (164·0 to 187·2)	49·1 (43·7 to 54·9)	104·0 (96·6 to 111·0)	20·6 (14·5 to 28·4)	1·8 (1·6 to 1·9)	626·5 (585·7 to 668·8)	175·4 (156·2 to 196·1)	371·3 (344·9 to 396·5)	73·5 (52·0 to 101·6)	6·3 (5·8 to 6·8)
Egypt*	2028	178·6 (166·8 to 190·9)	50·1 (44·6 to 56·1)	105·4 (97·8 to 112·5)	21·4 (15·0 to 29·5)	1·7 (1·6 to 1·9)	638·0 (595·9 to 682·0)	179·1 (159·3 to 200·5)	376·4 (349·5 to 402·0)	76·3 (53·7 to 105·3)	6·2 (5·8 to 6·8)

Egypt*	2029	182·0 (169·9 to 194·5)	51·2 (45·3 to 57·5)	106·9 (99·2 to 114·1)	22·2 (15·5 to 30·6)	1·7 (1·6 to 1·9)	650·0 (606·7 to 694·7)	182·9 (161·8 to 205·3)	381·7 (354·3 to 407·6)	79·2 (55·4 to 109·2)	6·2 (5·7 to 6·8)
Egypt*	2030	185·5 (172·9 to 198·7)	52·4 (46·3 to 59·1)	108·4 (100·6 to 115·7)	23·0 (16·1 to 31·8)	1·7 (1·6 to 1·9)	662·7 (617·7 to 709·9)	187·0 (165·2 to 211·1)	387·3 (359·3 to 413·4)	82·3 (57·6 to 113·7)	6·1 (5·5 to 6·7)
Egypt*	2031	189·4 (176·3 to 203·0)	53·6 (47·4 to 61·0)	110·1 (102·2 to 117·5)	24·0 (16·8 to 33·2)	1·7 (1·5 to 1·9)	676·5 (629·9 to 725·2)	191·6 (169·3 to 218·0)	393·2 (365·0 to 419·8)	85·6 (59·9 to 118·6)	6·0 (5·4 to 6·6)
Egypt*	2032	193·3 (179·6 to 207·3)	55·0 (48·3 to 62·8)	111·8 (103·7 to 119·2)	24·9 (17·4 to 34·8)	1·7 (1·5 to 1·8)	690·6 (641·4 to 740·5)	196·4 (172·7 to 224·4)	399·2 (370·6 to 425·9)	89·1 (62·1 to 124·2)	5·9 (5·4 to 6·6)
Egypt*	2033	197·5 (183·4 to 212·2)	56·4 (49·4 to 64·3)	113·5 (105·4 to 121·1)	26·0 (18·1 to 36·3)	1·6 (1·5 to 1·8)	705·5 (655·1 to 758·1)	201·5 (176·5 to 229·8)	405·4 (376·5 to 432·6)	92·7 (64·7 to 129·7)	5·8 (5·3 to 6·5)
Egypt*	2034	202·0 (187·3 to 217·2)	57·9 (50·7 to 66·2)	115·4 (107·2 to 123·1)	27·1 (18·9 to 37·9)	1·6 (1·4 to 1·8)	721·5 (669·0 to 775·8)	206·9 (181·2 to 236·4)	412·2 (382·8 to 439·9)	96·6 (67·5 to 135·2)	5·7 (5·1 to 6·4)
Egypt*	2035	206·4 (191·1 to 222·3)	59·4 (51·5 to 68·4)	117·2 (108·9 to 125·1)	28·2 (19·6 to 39·4)	1·6 (1·4 to 1·8)	737·2 (682·4 to 794·1)	212·2 (183·9 to 244·4)	418·6 (388·8 to 446·9)	100·6 (70·1 to 140·8)	5·7 (5·1 to 6·3)
Egypt*	2036	210·4 (194·7 to 227·0)	60·8 (52·6 to 70·4)	118·8 (110·4 to 126·8)	29·3 (20·3 to 41·1)	1·6 (1·4 to 1·8)	751·6 (695·3 to 810·8)	217·1 (187·7 to 251·6)	424·4 (394·2 to 452·9)	104·5 (72·4 to 146·7)	5·6 (5·0 to 6·3)
Egypt*	2037	214·2 (197·7 to 231·3)	62·1 (53·7 to 72·1)	120·2 (111·7 to 128·3)	30·3 (21·1 to 42·5)	1·6 (1·4 to 1·8)	765·0 (706·1 to 826·3)	221·7 (191·8 to 257·5)	429·3 (398·8 to 458·2)	108·4 (75·2 to 151·8)	5·6 (5·0 to 6·3)
Egypt*	2038	218·0 (201·0 to 235·9)	63·4 (54·8 to 74·2)	121·6 (113·0 to 129·9)	31·5 (21·8 to 44·1)	1·6 (1·4 to 1·8)	778·8 (717·9 to 842·5)	226·5 (195·8 to 265·1)	434·3 (403·5 to 463·9)	112·4 (78·0 to 157·7)	5·6 (5·0 to 6·3)
Egypt*	2039	222·3 (204·5 to 240·9)	64·8 (55·9 to 76·1)	123·2 (114·4 to 131·7)	32·7 (22·6 to 46·0)	1·6 (1·4 to 1·8)	793·9 (730·6 to 860·4)	231·6 (199·5 to 271·9)	440·0 (408·8 to 470·3)	116·7 (80·9 to 164·3)	5·6 (5·0 to 6·4)
Egypt*	2040	226·7 (208·3 to 246·1)	66·3 (56·9 to 78·4)	124·8 (115·9 to 133·4)	33·9 (23·5 to 47·7)	1·6 (1·4 to 1·8)	809·6 (744·0 to 879·2)	237·0 (203·3 to 280·2)	445·8 (413·8 to 476·6)	121·2 (84·0 to 170·5)	5·6 (4·9 to 6·4)
Egypt*	2041	231·3 (212·0 to 251·5)	68·0 (58·0 to 80·6)	126·5 (117·3 to 135·3)	35·3 (24·3 to 49·7)	1·6 (1·4 to 1·8)	826·0 (757·2 to 898·4)	242·8 (207·2 to 287·9)	451·8 (419·1 to 483·2)	125·9 (86·9 to 177·5)	5·6 (4·9 to 6·4)
Egypt*	2042	235·9 (216·1 to 257·1)	69·6 (59·1 to 82·6)	128·1 (118·8 to 137·1)	36·6 (25·2 to 52·0)	1·6 (1·4 to 1·8)	842·6 (771·7 to 918·2)	248·6 (211·0 to 295·0)	457·6 (424·5 to 489·7)	130·8 (90·1 to 185·8)	5·6 (4·9 to 6·5)

Egypt*	2043	240·5 (219·8 to 262·5)	71·2 (60·5 to 85·2)	129·7 (120·3 to 138·9)	38·0 (26·2 to 54·3)	1·6 (1·4 to 1·8)	859·1 (785·1 to 937·7)	254·5 (216·0 to 304·3)	463·3 (429·8 to 496·1)	135·8 (93·5 to 193·9)	5·6 (4·8 to 6·5)
Egypt*	2044	245·3 (223·7 to 268·3)	73·0 (61·4 to 87·6)	131·3 (121·7 to 140·6)	39·4 (27·0 to 56·6)	1·6 (1·4 to 1·8)	876·3 (799·0 to 958·4)	260·9 (219·4 to 313·1)	468·9 (434·8 to 502·3)	140·9 (96·5 to 202·2)	5·6 (4·9 to 6·6)
Egypt*	2045	250·3 (227·5 to 274·6)	75·0 (62·3 to 90·5)	132·8 (123·1 to 142·4)	40·9 (27·7 to 59·1)	1·6 (1·4 to 1·9)	894·0 (812·7 to 980·9)	267·8 (222·7 to 323·3)	474·4 (439·7 to 508·5)	146·2 (99·0 to 211·2)	5·6 (4·9 to 6·6)
Egypt*	2046	255·3 (231·7 to 280·4)	76·9 (63·7 to 92·7)	134·4 (124·5 to 143·9)	42·5 (28·5 to 61·5)	1·6 (1·4 to 1·9)	912·1 (827·6 to 1001·7)	274·7 (227·4 to 331·1)	480·0 (444·6 to 514·2)	151·7 (101·7 to 219·8)	5·7 (4·9 to 6·6)
Egypt*	2047	260·4 (236·2 to 286·6)	78·8 (64·1 to 95·4)	135·9 (125·8 to 145·5)	44·0 (29·5 to 63·9)	1·6 (1·4 to 1·9)	930·1 (843·6 to 1023·8)	281·6 (229·1 to 340·7)	485·4 (449·4 to 519·6)	157·3 (105·2 to 228·3)	5·7 (4·9 to 6·8)
Egypt*	2048	265·4 (240·0 to 292·7)	80·8 (65·1 to 97·8)	137·3 (127·2 to 147·1)	45·6 (30·5 to 66·7)	1·6 (1·4 to 1·9)	948·0 (857·3 to 1045·5)	288·7 (232·6 to 349·4)	490·5 (454·3 to 525·3)	163·0 (108·8 to 238·2)	5·7 (4·9 to 6·8)
Egypt*	2049	270·4 (243·5 to 299·4)	82·8 (66·8 to 101·2)	138·7 (128·6 to 148·7)	47·3 (31·3 to 69·4)	1·6 (1·4 to 1·9)	965·9 (869·7 to 1069·3)	295·7 (238·5 to 361·3)	495·5 (459·2 to 531·1)	168·9 (111·8 to 248·0)	5·8 (4·9 to 6·9)
Egypt*	2050	275·6 (247·6 to 305·9)	84·9 (67·5 to 104·5)	140·1 (129·8 to 150·5)	49·0 (32·1 to 72·5)	1·6 (1·4 to 2·0)	984·5 (884·4 to 1092·5)	303·1 (241·3 to 373·4)	500·6 (463·7 to 537·5)	175·0 (114·7 to 258·8)	5·9 (5·0 to 7·0)
Eritrea*	1995	19·0 (16·7 to 21·9)	5·4 (4·5 to 6·6)	10·3 (8·1 to 12·8)	0·0 (0·0 to 0·0)	3·3 (3·3 to 3·3)	59·3 (52·2 to 68·1)	16·9 (13·9 to 20·5)	32·0 (25·1 to 40·0)	0·0 (0·0 to 0·0)	10·4 (10·4 to 10·4)
Eritrea*	1996	20·2 (17·8 to 23·0)	5·3 (4·3 to 6·4)	10·6 (8·3 to 13·2)	0·0 (0·0 to 0·0)	4·3 (4·3 to 4·3)	63·1 (55·5 to 71·6)	16·5 (13·5 to 20·0)	33·1 (26·0 to 41·3)	0·0 (0·0 to 0·0)	13·5 (13·5 to 13·5)
Eritrea*	1997	18·0 (15·5 to 20·9)	5·2 (4·3 to 6·4)	11·2 (8·9 to 13·9)	0·0 (0·0 to 0·0)	1·6 (1·6 to 1·6)	56·1 (48·2 to 65·1)	16·3 (13·3 to 19·8)	34·8 (27·7 to 43·3)	0·0 (0·0 to 0·0)	4·9 (4·9 to 4·9)
Eritrea*	1998	23·0 (20·2 to 26·2)	6·3 (5·1 to 7·7)	12·5 (9·9 to 15·7)	0·0 (0·0 to 0·0)	4·1 (4·1 to 4·1)	71·6 (63·0 to 81·8)	19·7 (16·1 to 23·9)	39·0 (31·0 to 48·8)	0·0 (0·0 to 0·0)	12·8 (12·8 to 12·8)
Eritrea*	1999	25·6 (22·6 to 29·0)	7·0 (5·7 to 8·5)	13·2 (10·4 to 16·4)	0·0 (0·0 to 0·0)	5·5 (5·5 to 5·5)	79·7 (70·5 to 90·4)	21·7 (17·8 to 26·5)	41·0 (32·6 to 51·0)	0·0 (0·0 to 0·0)	17·0 (17·0 to 17·0)
Eritrea*	2000	25·8 (23·0 to 29·1)	6·3 (5·1 to 7·7)	12·0 (9·5 to 15·0)	0·0 (0·0 to 0·0)	7·4 (7·4 to 7·4)	80·4 (71·9 to 90·7)	19·6 (16·0 to 24·0)	37·5 (29·7 to 46·8)	0·0 (0·0 to 0·0)	23·2 (23·2 to 23·2)
Eritrea*	2001	21·7 (19·0 to 24·9)	6·3 (5·1 to 7·7)	11·4 (9·1 to 14·4)	0·0 (0·0 to 0·0)	4·0 (4·0 to 4·0)	67·5 (59·1 to 77·6)	19·6 (15·9 to 24·0)	35·6 (28·3 to 45·0)	0·0 (0·0 to 0·0)	12·3 (12·3 to 12·3)
Eritrea*	2002	22·4 (19·9 to 25·3)	5·9 (4·8 to 7·2)	10·5 (8·3 to 13·3)	0·0 (0·0 to 0·0)	6·0 (6·0 to 6·0)	69·8 (62·1 to 78·8)	18·4 (15·0 to 22·6)	32·7 (25·8 to 41·5)	0·0 (0·0 to 0·0)	18·7 (18·7 to 18·7)

Eritrea*	2003	24·6 (22·3 to 27·3)	5·4 (4·4 to 6·7)	9·6 (7·6 to 12·1)	0·0 (0·0 to 0·0)	9·6 (9·6 to 9·6)	76·8 (69·7 to 85·0)	17·0 (13·9 to 20·9)	30·0 (23·8 to 37·7)	0·0 (0·0 to 0·0)	29·8 (29·8 to 29·8)
Eritrea*	2004	21·7 (19·8 to 24·0)	4·4 (3·6 to 5·4)	8·5 (6·7 to 10·6)	0·0 (0·0 to 0·0)	8·9 (8·9 to 8·9)	67·7 (61·7 to 74·8)	13·7 (11·1 to 16·7)	26·4 (20·9 to 33·0)	0·0 (0·0 to 0·0)	27·7 (27·7 to 27·7)
Eritrea*	2005	20·3 (18·4 to 22·6)	4·0 (3·2 to 4·8)	8·1 (6·4 to 10·2)	0·0 (0·0 to 0·0)	8·3 (8·3 to 8·3)	63·3 (57·5 to 70·3)	12·3 (10·1 to 14·9)	25·2 (20·1 to 31·7)	0·0 (0·0 to 0·0)	25·8 (25·8 to 25·8)
Eritrea*	2006	14·3 (12·7 to 16·4)	3·2 (2·6 to 3·8)	7·2 (5·8 to 9·1)	0·0 (0·0 to 0·0)	3·9 (3·9 to 3·9)	44·7 (39·6 to 51·2)	9·8 (8·1 to 11·9)	22·5 (18·0 to 28·4)	0·0 (0·0 to 0·0)	12·3 (12·3 to 12·3)
Eritrea*	2007	15·4 (13·8 to 17·5)	3·3 (2·7 to 4·0)	7·1 (5·6 to 8·9)	0·0 (0·0 to 0·0)	5·0 (5·0 to 5·0)	48·0 (43·1 to 54·4)	10·4 (8·5 to 12·5)	22·0 (17·5 to 27·9)	0·0 (0·0 to 0·0)	15·6 (15·6 to 15·6)
Eritrea*	2008	15·4 (14·0 to 17·2)	3·2 (2·6 to 3·9)	6·5 (5·2 to 8·2)	0·0 (0·0 to 0·0)	5·7 (5·7 to 5·7)	48·0 (43·6 to 53·7)	10·0 (8·2 to 12·1)	20·4 (16·2 to 25·6)	0·0 (0·0 to 0·0)	17·7 (17·7 to 17·7)
Eritrea*	2009	13·0 (11·7 to 14·7)	2·7 (2·2 to 3·3)	6·3 (5·0 to 7·9)	0·0 (0·0 to 0·0)	4·0 (4·0 to 4·0)	40·6 (36·4 to 45·8)	8·5 (7·0 to 10·2)	19·6 (15·7 to 24·6)	0·0 (0·0 to 0·0)	12·5 (12·5 to 12·5)
Eritrea*	2010	19·5 (18·0 to 21·2)	2·9 (2·4 to 3·5)	6·6 (5·3 to 8·3)	0·0 (0·0 to 0·0)	9·9 (9·9 to 9·9)	60·7 (56·3 to 66·2)	9·1 (7·4 to 11·0)	20·7 (16·6 to 25·9)	0·0 (0·0 to 0·0)	30·9 (30·9 to 30·9)
Eritrea*	2011	13·8 (12·3 to 15·6)	2·9 (2·4 to 3·6)	6·9 (5·6 to 8·5)	0·0 (0·0 to 0·0)	4·0 (4·0 to 4·0)	43·0 (38·3 to 48·6)	9·2 (7·4 to 11·1)	21·4 (17·4 to 26·6)	0·0 (0·0 to 0·0)	12·4 (12·4 to 12·4)
Eritrea*	2012	15·9 (14·3 to 17·8)	2·8 (2·3 to 3·4)	7·1 (5·7 to 8·9)	0·0 (0·0 to 0·0)	5·9 (5·9 to 5·9)	49·5 (44·7 to 55·6)	8·9 (7·2 to 10·7)	22·1 (17·7 to 27·8)	0·0 (0·0 to 0·0)	18·5 (18·5 to 18·5)
Eritrea*	2013	15·5 (14·0 to 17·4)	2·5 (2·0 to 3·0)	7·0 (5·6 to 8·8)	0·0 (0·0 to 0·0)	6·0 (6·0 to 6·0)	48·4 (43·6 to 54·2)	7·8 (6·3 to 9·3)	21·9 (17·3 to 27·5)	0·0 (0·0 to 0·0)	18·8 (18·8 to 18·8)
Eritrea*	2014	16·2 (14·6 to 18·2)	2·5 (2·0 to 3·0)	7·2 (5·8 to 9·1)	0·0 (0·0 to 0·0)	6·5 (6·5 to 6·5)	50·5 (45·6 to 56·7)	7·6 (6·2 to 9·2)	22·5 (18·0 to 28·4)	0·0 (0·0 to 0·0)	20·3 (20·3 to 20·3)
Eritrea*	2015	11·8 (10·3 to 13·6)	2·3 (1·8 to 2·8)	6·6 (5·3 to 8·4)	0·0 (0·0 to 0·0)	2·9 (2·9 to 2·9)	36·7 (32·2 to 42·4)	7·1 (5·8 to 8·7)	20·6 (16·4 to 26·2)	0·0 (0·0 to 0·0)	8·9 (8·9 to 8·9)
Eritrea*	2016	12·7 (11·2 to 14·5)	2·3 (1·9 to 2·8)	6·6 (5·2 to 8·3)	0·0 (0·0 to 0·0)	3·8 (3·8 to 3·8)	39·5 (34·9 to 45·2)	7·2 (5·8 to 8·7)	20·4 (16·1 to 26·0)	0·0 (0·0 to 0·0)	11·9 (11·9 to 11·9)
Eritrea*	2017	14·8 (13·4 to 16·5)	2·4 (1·9 to 2·9)	6·2 (4·9 to 8·0)	0·0 (0·0 to 0·0)	6·2 (6·2 to 6·2)	46·1 (41·7 to 51·5)	7·4 (6·0 to 9·1)	19·5 (15·3 to 24·9)	0·0 (0·0 to 0·0)	19·2 (19·2 to 19·2)
Eritrea*	2018	14·2 (12·9 to 15·9)	2·0 (1·6 to 2·4)	5·9 (4·6 to 7·5)	0·0 (0·0 to 0·0)	6·3 (6·3 to 6·3)	44·2 (40·1 to 49·6)	6·2 (5·0 to 7·6)	18·5 (14·5 to 23·4)	0·0 (0·0 to 0·0)	19·5 (19·5 to 19·5)
Eritrea*	2019	14·8 (13·4 to 16·6)	2·6 (2·1 to 3·3)	5·9 (4·7 to 7·5)	0·0 (0·0 to 0·0)	6·2 (6·2 to 6·3)	46·0 (41·7 to 51·7)	8·2 (6·6 to 10·2)	18·5 (14·5 to 23·5)	0·0 (0·0 to 0·0)	19·4 (19·2 to 19·6)
Eritrea*	2020	16·9 (15·5 to 18·5)	2·7 (2·1 to 3·3)	5·5 (4·3 to 7·0)	0·0 (0·0 to 0·0)	8·7 (8·5 to 9·0)	52·6 (48·3 to 57·8)	8·3 (6·7 to 10·3)	17·1 (13·5 to 21·7)	0·0 (0·0 to 0·0)	27·2 (26·5 to 28·0)

Eritrea*	2021	16·5 (15·2 to 18·1)	2·5 (2·0 to 3·1)	5·2 (4·1 to 6·6)	0·0 (0·0 to 0·0)	8·8 (8·5 to 9·1)	51·5 (47·3 to 56·5)	7·9 (6·3 to 9·8)	16·3 (12·8 to 20·7)	0·0 (0·0 to 0·0)	27·3 (26·4 to 28·4)
Eritrea*	2022	14·3 (13·0 to 15·9)	2·4 (1·9 to 3·0)	5·3 (4·2 to 6·8)	0·0 (0·0 to 0·0)	6·6 (6·2 to 7·1)	44·7 (40·4 to 49·7)	7·5 (6·0 to 9·4)	16·6 (13·0 to 21·1)	0·0 (0·0 to 0·0)	20·5 (19·2 to 22·1)
Eritrea*	2023	14·4 (13·0 to 16·1)	2·5 (2·0 to 3·1)	5·4 (4·2 to 6·9)	0·0 (0·0 to 0·0)	6·5 (6·0 to 7·1)	45·0 (40·6 to 50·2)	7·7 (6·2 to 9·7)	16·9 (13·2 to 21·4)	0·0 (0·0 to 0·0)	20·4 (18·8 to 22·1)
Eritrea*	2024	14·6 (13·1 to 16·2)	2·6 (2·0 to 3·2)	5·5 (4·3 to 7·0)	0·0 (0·0 to 0·0)	6·5 (6·0 to 7·1)	45·4 (40·9 to 50·7)	8·0 (6·3 to 10·0)	17·1 (13·4 to 21·8)	0·0 (0·0 to 0·0)	20·3 (18·6 to 22·1)
Eritrea*	2025	14·7 (13·2 to 16·4)	2·6 (2·1 to 3·3)	5·6 (4·4 to 7·1)	0·0 (0·0 to 0·0)	6·4 (5·9 to 7·1)	45·7 (41·1 to 51·2)	8·2 (6·4 to 10·4)	17·4 (13·7 to 22·1)	0·0 (0·0 to 0·0)	20·1 (18·4 to 22·0)
Eritrea*	2026	14·8 (13·2 to 16·6)	2·7 (2·1 to 3·4)	5·7 (4·5 to 7·2)	0·0 (0·0 to 0·0)	6·4 (5·8 to 7·0)	46·0 (41·2 to 51·7)	8·5 (6·5 to 10·7)	17·7 (13·9 to 22·5)	0·0 (0·0 to 0·0)	19·8 (18·0 to 21·8)
Eritrea*	2027	14·9 (13·3 to 16·7)	2·8 (2·1 to 3·6)	5·8 (4·5 to 7·3)	0·0 (0·0 to 0·0)	6·3 (5·7 to 7·0)	46·3 (41·5 to 52·0)	8·7 (6·7 to 11·1)	18·0 (14·1 to 22·9)	0·0 (0·0 to 0·0)	19·6 (17·8 to 21·7)
Eritrea*	2028	15·0 (13·4 to 16·8)	2·9 (2·2 to 3·6)	5·9 (4·6 to 7·4)	0·0 (0·0 to 0·0)	6·2 (5·6 to 7·0)	46·7 (41·9 to 52·5)	9·0 (6·9 to 11·4)	18·3 (14·3 to 23·2)	0·0 (0·0 to 0·0)	19·4 (17·6 to 21·7)
Eritrea*	2029	15·1 (13·5 to 17·0)	3·0 (2·3 to 3·8)	6·0 (4·7 to 7·6)	0·0 (0·0 to 0·0)	6·2 (5·6 to 6·9)	47·1 (42·1 to 53·1)	9·3 (7·1 to 11·7)	18·6 (14·6 to 23·6)	0·0 (0·0 to 0·0)	19·2 (17·4 to 21·5)
Eritrea*	2030	15·2 (13·6 to 17·2)	3·0 (2·3 to 3·9)	6·1 (4·7 to 7·7)	0·0 (0·0 to 0·0)	6·1 (5·5 to 6·9)	47·4 (42·3 to 53·5)	9·5 (7·2 to 12·1)	18·9 (14·8 to 23·9)	0·0 (0·0 to 0·0)	19·0 (17·1 to 21·4)
Eritrea*	2031	15·3 (13·6 to 17·4)	3·1 (2·4 to 4·0)	6·1 (4·8 to 7·8)	0·0 (0·0 to 0·0)	6·0 (5·4 to 6·8)	47·8 (42·5 to 54·1)	9·8 (7·4 to 12·6)	19·2 (15·0 to 24·3)	0·0 (0·0 to 0·0)	18·8 (16·9 to 21·2)
Eritrea*	2032	15·4 (13·7 to 17·5)	3·2 (2·4 to 4·2)	6·2 (4·9 to 7·9)	0·0 (0·0 to 0·0)	6·0 (5·3 to 6·8)	48·2 (42·8 to 54·5)	10·0 (7·6 to 13·0)	19·5 (15·3 to 24·7)	0·0 (0·0 to 0·0)	18·6 (16·6 to 21·1)
Eritrea*	2033	15·6 (13·8 to 17·7)	3·3 (2·5 to 4·3)	6·3 (5·0 to 8·1)	0·0 (0·0 to 0·0)	5·9 (5·3 to 6·7)	48·6 (43·1 to 55·0)	10·3 (7·8 to 13·3)	19·8 (15·5 to 25·1)	0·0 (0·0 to 0·0)	18·5 (16·4 to 20·9)
Eritrea*	2034	15·7 (13·9 to 17·9)	3·4 (2·5 to 4·4)	6·4 (5·0 to 8·2)	0·0 (0·0 to 0·0)	5·9 (5·2 to 6·7)	49·0 (43·4 to 55·7)	10·6 (7·9 to 13·8)	20·1 (15·7 to 25·5)	0·0 (0·0 to 0·0)	18·3 (16·3 to 20·8)
Eritrea*	2035	15·9 (14·1 to 18·0)	3·5 (2·6 to 4·6)	6·5 (5·1 to 8·3)	0·0 (0·0 to 0·0)	5·9 (5·2 to 6·7)	49·5 (43·9 to 56·3)	10·9 (8·1 to 14·2)	20·4 (16·0 to 25·9)	0·0 (0·0 to 0·0)	18·3 (16·2 to 20·8)
Eritrea*	2036	16·0 (14·2 to 18·3)	3·6 (2·6 to 4·7)	6·6 (5·2 to 8·5)	0·0 (0·0 to 0·0)	5·8 (5·1 to 6·7)	50·0 (44·2 to 57·0)	11·2 (8·2 to 14·5)	20·7 (16·2 to 26·4)	0·0 (0·0 to 0·0)	18·1 (16·1 to 20·8)
Eritrea*	2037	16·2 (14·3 to 18·6)	3·7 (2·7 to 4·8)	6·7 (5·3 to 8·6)	0·0 (0·0 to 0·0)	5·8 (5·1 to 6·6)	50·6 (44·6 to 57·9)	11·4 (8·3 to 14·9)	21·0 (16·4 to 26·7)	0·0 (0·0 to 0·1)	18·1 (16·0 to 20·7)
Eritrea*	2038	16·4 (14·5 to 18·8)	3·8 (2·7 to 5·0)	6·8 (5·4 to 8·7)	0·0 (0·0 to 0·0)	5·8 (5·1 to 6·7)	51·2 (45·2 to 58·5)	11·8 (8·5 to 15·5)	21·3 (16·7 to 27·1)	0·0 (0·0 to 0·1)	18·1 (15·9 to 20·7)

Eritrea*	2039	16·6 (14·7 to 19·1)	3·9 (2·8 to 5·1)	6·9 (5·4 to 8·8)	0·0 (0·0 to 0·0)	5·8 (5·1 to 6·7)	51·9 (45·8 to 59·5)	12·1 (8·8 to 15·9)	21·6 (16·9 to 27·5)	0·0 (0·0 to 0·1)	18·1 (15·9 to 21·0)
Eritrea*	2040	16·9 (14·8 to 19·3)	4·0 (2·9 to 5·3)	7·0 (5·5 to 8·9)	0·0 (0·0 to 0·0)	5·8 (5·1 to 6·8)	52·6 (46·3 to 60·3)	12·4 (8·9 to 16·4)	21·9 (17·2 to 27·9)	0·0 (0·0 to 0·1)	18·2 (15·9 to 21·2)
Eritrea*	2041	17·1 (15·0 to 19·7)	4·1 (2·9 to 5·4)	7·1 (5·6 to 9·1)	0·0 (0·0 to 0·0)	5·9 (5·1 to 6·9)	53·3 (46·7 to 61·3)	12·7 (9·2 to 16·8)	22·2 (17·4 to 28·3)	0·0 (0·0 to 0·1)	18·3 (15·9 to 21·4)
Eritrea*	2042	17·3 (15·2 to 19·9)	4·2 (3·0 to 5·5)	7·2 (5·6 to 9·2)	0·0 (0·0 to 0·0)	5·9 (5·1 to 7·0)	54·1 (47·3 to 62·1)	13·0 (9·3 to 17·0)	22·5 (17·6 to 28·6)	0·0 (0·0 to 0·1)	18·5 (16·0 to 21·7)
Eritrea*	2043	17·6 (15·4 to 20·2)	4·3 (3·1 to 5·7)	7·3 (5·7 to 9·3)	0·0 (0·0 to 0·0)	6·0 (5·2 to 7·1)	54·9 (48·0 to 63·1)	13·4 (9·5 to 17·6)	22·7 (17·8 to 28·9)	0·0 (0·0 to 0·1)	18·8 (16·2 to 22·2)
Eritrea*	2044	17·9 (15·7 to 20·6)	4·4 (3·2 to 5·9)	7·4 (5·8 to 9·4)	0·0 (0·0 to 0·0)	6·1 (5·3 to 7·2)	55·9 (48·9 to 64·2)	13·8 (9·9 to 18·5)	23·0 (18·0 to 29·3)	0·0 (0·0 to 0·1)	19·0 (16·4 to 22·5)
Eritrea*	2045	18·2 (15·9 to 20·9)	4·5 (3·2 to 6·1)	7·5 (5·8 to 9·5)	0·0 (0·0 to 0·0)	6·2 (5·3 to 7·3)	56·8 (49·6 to 65·2)	14·1 (10·1 to 19·1)	23·3 (18·2 to 29·6)	0·0 (0·0 to 0·1)	19·3 (16·6 to 22·9)
Eritrea*	2046	18·5 (16·2 to 21·3)	4·7 (3·3 to 6·3)	7·6 (5·9 to 9·6)	0·0 (0·0 to 0·0)	6·3 (5·4 to 7·4)	57·8 (50·4 to 66·5)	14·5 (10·2 to 19·6)	23·5 (18·4 to 30·0)	0·0 (0·0 to 0·1)	19·7 (16·9 to 23·2)
Eritrea*	2047	18·9 (16·5 to 21·8)	4·8 (3·4 to 6·4)	7·6 (6·0 to 9·7)	0·0 (0·0 to 0·0)	6·4 (5·5 to 7·6)	58·8 (51·5 to 68·0)	14·9 (10·5 to 20·0)	23·8 (18·7 to 30·4)	0·0 (0·0 to 0·1)	20·0 (17·0 to 23·8)
Eritrea*	2048	19·2 (16·8 to 22·2)	4·9 (3·4 to 6·7)	7·7 (6·0 to 9·8)	0·0 (0·0 to 0·0)	6·6 (5·6 to 7·8)	59·9 (52·4 to 69·3)	15·3 (10·7 to 20·9)	24·1 (18·8 to 30·7)	0·0 (0·0 to 0·1)	20·4 (17·4 to 24·3)
Eritrea*	2049	19·6 (17·0 to 22·6)	5·1 (3·5 to 6·9)	7·8 (6·1 to 9·9)	0·0 (0·0 to 0·0)	6·7 (5·7 to 8·0)	61·0 (53·2 to 70·6)	15·8 (11·0 to 21·5)	24·3 (19·0 to 31·0)	0·1 (0·0 to 0·1)	20·9 (17·7 to 24·8)
Eritrea*	2050	19·9 (17·3 to 23·1)	5·2 (3·6 to 7·1)	7·9 (6·2 to 10·0)	0·0 (0·0 to 0·0)	6·9 (5·9 to 8·2)	62·2 (54·0 to 72·2)	16·1 (11·2 to 22·1)	24·6 (19·2 to 31·3)	0·1 (0·0 to 0·1)	21·4 (18·3 to 25·7)
Spain	1995	1523·2 (1462·1 to 1589·4)	1097·5 (1043·6 to 1152·8)	372·1 (346·2 to 397·5)	53·6 (41·9 to 67·7)	0·0 (0·0 to 0·0)	2166·6 (2079·7 to 2260·7)	1561·1 (1484·4 to 1639·7)	529·2 (492·4 to 565·3)	76·3 (59·6 to 96·3)	0·0 (0·0 to 0·0)
Spain	1996	1551·4 (1501·6 to 1602·3)	1119·1 (1074·5 to 1161·9)	375·5 (356·6 to 395·9)	56·8 (45·6 to 69·8)	0·0 (0·0 to 0·0)	2206·7 (2135·8 to 2279·2)	1591·8 (1528·4 to 1652·7)	534·1 (507·3 to 563·1)	80·7 (64·8 to 99·3)	0·0 (0·0 to 0·0)
Spain	1997	1585·5 (1539·1 to 1632·9)	1144·1 (1103·4 to 1188·1)	382·0 (363·4 to 400·3)	59·4 (48·8 to 71·3)	0·0 (0·0 to 0·0)	2255·2 (2189·3 to 2322·6)	1627·4 (1569·5 to 1690·0)	543·3 (516·9 to 569·4)	84·5 (69·4 to 101·4)	0·0 (0·0 to 0·0)
Spain	1998	1649·1 (1603·2 to 1695·6)	1188·9 (1147·7 to 1229·2)	396·1 (377·8 to 414·2)	64·1 (54·2 to 75·3)	0·0 (0·0 to 0·0)	2345·6 (2280·3 to 2411·8)	1691·1 (1632·5 to 1748·4)	563·4 (537·3 to 589·2)	91·2 (77·1 to 107·2)	0·0 (0·0 to 0·0)

Spain	1999	1705·1 (1659·9 to 1752·4)	1224·9 (1185·6 to 1266·9)	411·1 (396·1 to 427·4)	69·1 (60·2 to 79·5)	0·0 (0·0 to 0·0)	2425·4 (2361·0 to 2492·6)	1742·3 (1686·3 to 1802·0)	584·8 (563·4 to 607·9)	98·3 (85·6 to 113·0)	0·0 (0·0 to 0·0)
Spain	2000	1756·6 (1715·2 to 1801·6)	1256·4 (1215·6 to 1297·1)	426·7 (413·7 to 439·6)	73·6 (65·9 to 82·3)	0·0 (0·0 to 0·0)	2498·6 (2439·7 to 2562·6)	1787·0 (1729·0 to 1845·0)	606·9 (588·4 to 625·3)	104·6 (93·8 to 117·0)	0·0 (0·0 to 0·0)
Spain	2001	1807·7 (1765·5 to 1852·5)	1289·1 (1249·2 to 1329·8)	440·1 (427·9 to 452·1)	78·6 (71·3 to 86·4)	0·0 (0·0 to 0·0)	2571·3 (2511·2 to 2634·9)	1833·5 (1776·9 to 1891·5)	626·0 (608·7 to 643·1)	111·8 (101·4 to 123·0)	0·0 (0·0 to 0·0)
Spain	2002	1890·0 (1846·9 to 1933·7)	1348·3 (1306·8 to 1390·1)	449·8 (438·5 to 462·2)	92·0 (84·5 to 99·8)	0·0 (0·0 to 0·0)	2688·4 (2627·1 to 2750·5)	1917·7 (1858·8 to 1977·2)	639·8 (623·7 to 657·4)	130·9 (120·1 to 141·9)	0·0 (0·0 to 0·0)
Spain	2003	2001·5 (1955·1 to 2044·7)	1422·4 (1380·0 to 1464·3)	458·5 (446·6 to 470·5)	120·7 (111·9 to 129·2)	0·0 (0·0 to 0·0)	2846·9 (2781·0 to 2908·4)	2023·2 (1962·9 to 2082·8)	652·1 (635·3 to 669·2)	171·7 (159·2 to 183·8)	0·0 (0·0 to 0·0)
Spain	2004	2103·6 (2056·8 to 2150·0)	1499·6 (1457·5 to 1541·4)	466·0 (454·0 to 478·4)	138·0 (129·9 to 146·4)	0·0 (0·0 to 0·0)	2992·1 (2925·5 to 3058·1)	2133·0 (2073·1 to 2192·5)	662·9 (645·8 to 680·4)	196·2 (184·8 to 208·2)	0·0 (0·0 to 0·0)
Spain	2005	2159·2 (2110·5 to 2207·1)	1544·0 (1500·2 to 1588·3)	468·2 (455·8 to 481·4)	147·1 (139·2 to 155·5)	0·0 (0·0 to 0·0)	3071·3 (3001·9 to 3139·3)	2196·1 (2133·9 to 2259·2)	666·0 (648·3 to 684·7)	209·2 (198·1 to 221·2)	0·0 (0·0 to 0·0)
Spain	2006	2246·0 (2198·7 to 2293·3)	1616·2 (1572·8 to 1659·5)	474·2 (461·7 to 486·6)	155·7 (147·7 to 163·9)	0·0 (0·0 to 0·0)	3194·7 (3127·5 to 3262·0)	2298·8 (2237·2 to 2360·5)	674·5 (656·7 to 692·1)	221·4 (210·1 to 233·1)	0·0 (0·0 to 0·0)
Spain	2007	2333·3 (2292·3 to 2378·6)	1692·9 (1653·1 to 1735·4)	483·1 (471·6 to 495·0)	157·4 (148·9 to 166·1)	0·0 (0·0 to 0·0)	3318·9 (3260·6 to 3383·3)	2407·9 (2351·4 to 2468·5)	687·1 (670·8 to 704·1)	223·9 (211·8 to 236·3)	0·0 (0·0 to 0·0)
Spain	2008	2431·3 (2384·5 to 2478·2)	1787·9 (1744·9 to 1834·4)	492·4 (480·1 to 504·4)	151·0 (142·9 to 159·7)	0·0 (0·0 to 0·0)	3458·3 (3391·7 to 3525·0)	2543·1 (2481·9 to 2609·2)	700·4 (682·9 to 717·5)	214·8 (203·2 to 227·2)	0·0 (0·0 to 0·0)
Spain	2009	2531·9 (2487·2 to 2581·7)	1889·3 (1847·0 to 1937·5)	495·2 (483·5 to 507·3)	147·4 (139·3 to 156·4)	0·0 (0·0 to 0·0)	3601·4 (3537·7 to 3672·2)	2687·3 (2627·2 to 2755·9)	704·4 (687·8 to 721·6)	209·7 (198·2 to 222·4)	0·0 (0·0 to 0·0)
Spain	2010	2512·5 (2462·4 to 2560·5)	1868·9 (1820·4 to 1915·0)	502·5 (490·5 to 514·5)	141·1 (133·2 to 149·2)	0·0 (0·0 to 0·0)	3573·7 (3502·5 to 3642·0)	2658·3 (2589·4 to 2723·8)	714·7 (697·7 to 731·8)	200·8 (189·4 to 212·2)	0·0 (0·0 to 0·0)
Spain	2011	2474·3 (2430·5 to 2518·3)	1815·4 (1772·3 to 1857·2)	512·2 (499·4 to 524·2)	146·7 (138·7 to 155·1)	0·0 (0·0 to 0·0)	3519·4 (3457·1 to 3582·0)	2582·2 (2520·9 to 2641·6)	728·5 (710·3 to 745·7)	208·7 (197·3 to 220·7)	0·0 (0·0 to 0·0)
Spain	2012	2452·8 (2407·9 to 2499·5)	1774·8 (1733·4 to 1819·9)	529·2 (516·4 to 541·2)	148·7 (141·0 to 157·3)	0·0 (0·0 to 0·0)	3488·8 (3425·0 to 3555·2)	2524·5 (2465·6 to 2588·5)	752·8 (734·5 to 769·9)	211·5 (200·5 to 223·8)	0·0 (0·0 to 0·0)

Spain	2013	2365·8 (2326·2 to 2407·4)	1679·2 (1640·2 to 1717·8)	538·1 (526·3 to 550·1)	148·5 (140·4 to 156·7)	0·0 (0·0 to 0·0)	3365·1 (3308·7 to 3424·3)	2388·4 (2333·0 to 2443·4)	765·4 (748·7 to 782·4)	211·3 (199·8 to 222·9)	0·0 (0·0 to 0·0)
Spain	2014	2419·6 (2374·9 to 2467·5)	1709·4 (1666·2 to 1752·6)	554·4 (541·9 to 566·6)	155·9 (147·8 to 164·6)	0·0 (0·0 to 0·0)	3441·7 (3378·0 to 3509·7)	2431·5 (2370·0 to 2492·9)	788·5 (770·8 to 806·0)	221·7 (210·2 to 234·1)	0·0 (0·0 to 0·0)
Spain	2015	2518·9 (2477·9 to 2567·3)	1791·9 (1752·2 to 1836·8)	564·2 (552·1 to 576·4)	162·9 (154·9 to 171·4)	0·0 (0·0 to 0·0)	3582·9 (3524·6 to 3651·7)	2548·7 (2492·3 to 2612·6)	802·4 (785·3 to 819·9)	231·7 (220·3 to 243·7)	0·0 (0·0 to 0·0)
Spain	2016	2562·9 (2520·9 to 2609·2)	1824·9 (1782·5 to 1867·6)	567·2 (554·6 to 579·9)	170·8 (162·9 to 180·0)	0·0 (0·0 to 0·0)	3645·5 (3585·7 to 3711·3)	2595·7 (2535·5 to 2656·5)	806·8 (788·8 to 824·8)	243·0 (231·8 to 256·0)	0·0 (0·0 to 0·0)
Spain	2017	2636·4 (2592·0 to 2685·2)	1865·9 (1825·7 to 1911·2)	582·7 (569·9 to 595·7)	187·8 (179·2 to 196·8)	0·0 (0·0 to 0·0)	3749·9 (3686·8 to 3819·3)	2654·1 (2596·8 to 2718·4)	828·8 (810·6 to 847·3)	267·1 (254·9 to 279·9)	0·0 (0·0 to 0·0)
Spain	2018	2735·0 (2669·0 to 2797·0)	1927·4 (1868·8 to 1983·3)	604·6 (588·7 to 621·5)	203·1 (191·4 to 216·1)	0·0 (0·0 to 0·0)	3890·3 (3796·4 to 3978·5)	2741·5 (2658·2 to 2821·1)	859·9 (837·4 to 884·0)	288·8 (272·3 to 307·3)	0·0 (0·0 to 0·0)
Spain	2019	2801·4 (2730·5 to 2867·6)	1975·4 (1911·2 to 2038·1)	614·7 (598·5 to 631·5)	211·3 (199·1 to 224·7)	0·0 (0·0 to 0·0)	3984·7 (3883·9 to 4078·8)	2809·8 (2718·4 to 2899·0)	874·3 (851·4 to 898·3)	300·6 (283·2 to 319·6)	0·0 (0·0 to 0·0)
Spain	2020	3092·6 (3011·9 to 3167·6)	2316·8 (2241·6 to 2388·6)	574·0 (558·6 to 589·5)	201·8 (189·6 to 214·6)	0·0 (0·0 to 0·0)	4398·9 (4284·1 to 4505·6)	3295·4 (3188·4 to 3397·5)	816·5 (794·6 to 838·5)	287·1 (269·6 to 305·2)	0·0 (0·0 to 0·0)
Spain	2021	3001·3 (2922·4 to 3074·4)	2246·0 (2173·0 to 2315·5)	555·5 (540·7 to 570·7)	199·8 (187·3 to 212·7)	0·0 (0·0 to 0·0)	4269·0 (4156·9 to 4373·0)	3194·6 (3090·9 to 3293·6)	790·2 (769·0 to 811·7)	284·2 (266·4 to 302·5)	0·0 (0·0 to 0·0)
Spain	2022	2612·5 (2540·8 to 2682·4)	1850·1 (1783·6 to 1915·9)	557·4 (542·3 to 572·5)	205·0 (191·6 to 219·2)	0·0 (0·0 to 0·0)	3716·0 (3614·1 to 3815·5)	2631·6 (2536·9 to 2725·2)	792·9 (771·4 to 814·3)	291·6 (272·6 to 311·7)	0·0 (0·0 to 0·0)
Spain	2023	2647·1 (2569·8 to 2724·6)	1877·9 (1806·3 to 1951·9)	558·8 (544·1 to 574·0)	210·4 (195·7 to 226·0)	0·0 (0·0 to 0·0)	3765·2 (3655·2 to 3875·5)	2671·1 (2569·3 to 2776·3)	794·8 (773·9 to 816·4)	299·2 (278·4 to 321·4)	0·0 (0·0 to 0·0)
Spain	2024	2680·0 (2595·1 to 2768·0)	1904·6 (1824·8 to 1986·9)	559·7 (544·5 to 575·0)	215·7 (199·1 to 232·5)	0·0 (0·0 to 0·0)	3812·0 (3691·2 to 3937·2)	2709·1 (2595·6 to 2826·1)	796·1 (774·5 to 817·9)	306·8 (283·2 to 330·7)	0·0 (0·0 to 0·0)
Spain	2025	2709·8 (2617·1 to 2809·4)	1929·1 (1843·7 to 2023·0)	559·8 (544·7 to 575·3)	220·9 (202·6 to 239·8)	0·0 (0·0 to 0·0)	3854·4 (3722·6 to 3996·1)	2743·9 (2622·5 to 2877·4)	796·3 (774·7 to 818·3)	314·2 (288·1 to 341·1)	0·0 (0·0 to 0·0)
Spain	2026	2736·6 (2640·6 to 2834·6)	1951·3 (1863·9 to 2043·2)	559·3 (543·9 to 574·6)	226·0 (206·7 to 246·3)	0·0 (0·0 to 0·0)	3892·5 (3755·9 to 4032·0)	2775·6 (2651·2 to 2906·2)	795·5 (773·6 to 817·3)	321·5 (294·0 to 350·3)	0·0 (0·0 to 0·0)

Spain	2027	2777·8 (2676·8 to 2884·7)	1987·9 (1890·9 to 2090·0)	558·7 (543·4 to 574·2)	231·2 (210·6 to 252·8)	0·0 (0·0 to 0·0)	3951·1 (3807·4 to 4103·1)	2827·5 (2689·6 to 2972·8)	794·7 (772·9 to 816·7)	328·9 (299·5 to 359·6)	0·0 (0·0 to 0·0)
Spain	2028	2819·1 (2709·5 to 2931·5)	2024·5 (1922·1 to 2131·0)	558·0 (542·5 to 573·6)	236·6 (215·4 to 258·1)	0·0 (0·0 to 0·0)	4009·8 (3853·9 to 4169·8)	2879·6 (2734·0 to 3031·1)	793·7 (771·6 to 815·9)	336·6 (306·4 to 367·2)	0·0 (0·0 to 0·0)
Spain	2029	2863·2 (2750·0 to 2981·7)	2064·0 (1958·3 to 2171·4)	557·2 (542·1 to 572·9)	242·0 (219·9 to 264·6)	0·0 (0·0 to 0·0)	4072·6 (3911·6 to 4241·2)	2935·8 (2785·4 to 3088·6)	792·5 (771·0 to 814·9)	344·3 (312·8 to 376·4)	0·0 (0·0 to 0·0)
Spain	2030	2909·7 (2789·9 to 3031·5)	2105·2 (1994·8 to 2222·3)	556·7 (541·3 to 572·4)	247·7 (224·5 to 271·6)	0·0 (0·0 to 0·0)	4138·7 (3968·3 to 4312·0)	2994·5 (2837·4 to 3161·0)	791·9 (770·0 to 814·2)	352·4 (319·3 to 386·4)	0·0 (0·0 to 0·0)
Spain	2031	2944·3 (2825·6 to 3067·7)	2135·1 (2026·9 to 2251·6)	555·7 (540·2 to 571·3)	253·5 (230·0 to 279·5)	0·0 (0·0 to 0·0)	4187·9 (4019·1 to 4363·5)	3036·9 (2883·0 to 3202·7)	790·5 (768·4 to 812·6)	360·6 (327·1 to 397·6)	0·0 (0·0 to 0·0)
Spain	2032	2972·7 (2846·6 to 3099·0)	2158·8 (2044·8 to 2278·3)	554·5 (538·9 to 569·7)	259·4 (234·9 to 286·3)	0·0 (0·0 to 0·0)	4228·4 (4048·9 to 4408·1)	3070·6 (2908·5 to 3240·6)	788·8 (766·5 to 810·4)	369·0 (334·2 to 407·2)	0·0 (0·0 to 0·0)
Spain	2033	2997·0 (2864·2 to 3132·9)	2179·4 (2057·2 to 2305·7)	552·6 (536·5 to 568·0)	265·0 (239·0 to 293·9)	0·0 (0·0 to 0·0)	4262·9 (4073·9 to 4456·3)	3099·9 (2926·2 to 3279·7)	786·0 (763·2 to 807·9)	377·0 (340·0 to 418·0)	0·0 (0·0 to 0·0)
Spain	2034	3022·1 (2882·5 to 3172·5)	2200·4 (2071·9 to 2343·6)	550·8 (534·5 to 566·2)	270·9 (243·7 to 301·1)	0·0 (0·0 to 0·0)	4298·6 (4100·1 to 4512·5)	3129·8 (2947·1 to 3333·5)	783·4 (760·2 to 805·4)	385·3 (346·7 to 428·3)	0·0 (0·0 to 0·0)
Spain	2035	3048·6 (2903·7 to 3212·8)	2222·2 (2087·2 to 2382·5)	549·3 (533·0 to 565·4)	277·1 (248·2 to 310·2)	0·0 (0·0 to 0·0)	4336·3 (4130·3 to 4569·9)	3160·8 (2968·8 to 3388·9)	781·4 (758·1 to 804·3)	394·1 (353·0 to 441·2)	0·0 (0·0 to 0·0)
Spain	2036	3075·6 (2921·5 to 3257·5)	2244·1 (2101·6 to 2419·9)	548·0 (531·6 to 564·4)	283·5 (252·2 to 319·2)	0·0 (0·0 to 0·0)	4374·7 (4155·5 to 4633·4)	3192·0 (2989·3 to 3442·0)	779·5 (756·1 to 802·8)	403·2 (358·8 to 454·1)	0·0 (0·0 to 0·0)
Spain	2037	3098·6 (2934·6 to 3296·4)	2262·9 (2114·4 to 2453·1)	546·1 (529·5 to 563·3)	289·7 (255·8 to 327·3)	0·0 (0·0 to 0·0)	4407·5 (4174·2 to 4688·7)	3218·7 (3007·5 to 3489·3)	776·7 (753·1 to 801·2)	412·0 (363·8 to 465·6)	0·0 (0·0 to 0·0)
Spain	2038	3120·0 (2947·8 to 3337·8)	2280·4 (2123·1 to 2488·6)	543·8 (527·0 to 561·0)	295·9 (259·3 to 336·9)	0·0 (0·0 to 0·0)	4437·9 (4192·9 to 4747·7)	3243·6 (3019·9 to 3539·7)	773·5 (749·6 to 798·0)	420·8 (368·9 to 479·3)	0·0 (0·0 to 0·0)
Spain	2039	3136·3 (2955·4 to 3377·3)	2293·9 (2126·2 to 2525·0)	540·7 (523·8 to 557·9)	301·7 (262·3 to 347·0)	0·0 (0·0 to 0·0)	4461·0 (4203·7 to 4803·8)	3262·8 (3024·3 to 3591·5)	769·0 (745·1 to 793·6)	429·2 (373·0 to 493·5)	0·0 (0·0 to 0·0)
Spain	2040	3149·8 (2959·7 to 3412·6)	2305·2 (2130·2 to 2561·1)	537·2 (520·4 to 555·2)	307·5 (265·2 to 356·5)	0·0 (0·0 to 0·0)	4480·3 (4209·9 to 4854·0)	3278·8 (3030·0 to 3642·9)	764·1 (740·2 to 789·7)	437·4 (377·2 to 507·1)	0·0 (0·0 to 0·0)

Spain	2041	3163·0 (2963·8 to 3449·5)	2316·0 (2130·8 to 2593·3)	533·7 (516·8 to 552·5)	313·3 (267·8 to 367·7)	0·0 (0·0 to 0·0)	4499·0 (4215·6 to 4906·5)	3294·2 (3030·9 to 3688·7)	759·1 (735·1 to 785·9)	445·7 (381·0 to 523·0)	0·0 (0·0 to 0·0)
Spain	2042	3175·4 (2966·0 to 3483·3)	2325·8 (2132·6 to 2615·3)	530·4 (513·5 to 550·0)	319·3 (270·9 to 378·7)	0·0 (0·0 to 0·0)	4516·7 (4218·8 to 4954·7)	3308·1 (3033·4 to 3719·9)	754·4 (730·3 to 782·3)	454·2 (385·3 to 538·7)	0·0 (0·0 to 0·0)
Spain	2043	3186·5 (2968·5 to 3507·9)	2334·3 (2133·4 to 2649·0)	526·9 (509·8 to 547·3)	325·3 (274·0 to 388·2)	0·0 (0·0 to 0·0)	4532·5 (4222·4 to 4989·6)	3320·3 (3034·5 to 3767·9)	749·5 (725·2 to 778·5)	462·7 (389·7 to 552·2)	0·0 (0·0 to 0·0)
Spain	2044	3196·2 (2969·0 to 3533·4)	2341·7 (2133·3 to 2668·9)	523·3 (505·9 to 544·5)	331·2 (277·2 to 400·3)	0·0 (0·0 to 0·0)	4546·3 (4223·0 to 5025·8)	3330·8 (3034·4 to 3796·2)	744·4 (719·6 to 774·4)	471·1 (394·3 to 569·4)	0·0 (0·0 to 0·0)
Spain	2045	3206·6 (2973·7 to 3557·0)	2349·1 (2134·4 to 2699·1)	520·0 (502·4 to 541·8)	337·4 (280·2 to 411·5)	0·0 (0·0 to 0·0)	4561·0 (4229·7 to 5059·5)	3341·3 (3036·0 to 3839·2)	739·7 (714·6 to 770·7)	480·0 (398·6 to 585·4)	0·0 (0·0 to 0·0)
Spain	2046	3219·4 (2983·2 to 3586·5)	2358·1 (2139·9 to 2723·7)	517·3 (499·4 to 539·8)	344·0 (283·4 to 422·8)	0·0 (0·0 to 0·0)	4579·3 (4243·3 to 5101·4)	3354·2 (3043·8 to 3874·2)	735·8 (710·3 to 767·8)	489·3 (403·1 to 601·5)	0·0 (0·0 to 0·0)
Spain	2047	3235·7 (2994·8 to 3611·4)	2369·2 (2145·9 to 2733·4)	515·3 (497·2 to 538·5)	351·1 (286·9 to 434·5)	0·0 (0·0 to 0·0)	4602·4 (4259·8 to 5136·7)	3369·9 (3052·3 to 3888·0)	733·0 (707·2 to 765·9)	499·5 (408·1 to 618·0)	0·0 (0·0 to 0·0)
Spain	2048	3258·1 (3008·7 to 3641·4)	2384·5 (2159·0 to 2761·3)	514·6 (496·0 to 537·9)	359·1 (291·5 to 447·6)	0·0 (0·0 to 0·0)	4634·3 (4279·6 to 5179·6)	3391·7 (3070·9 to 3927·7)	731·9 (705·5 to 765·1)	510·7 (414·7 to 636·7)	0·0 (0·0 to 0·0)
Spain	2049	3283·5 (3033·9 to 3673·6)	2401·7 (2175·0 to 2781·1)	514·3 (495·6 to 538·1)	367·5 (295·8 to 460·3)	0·0 (0·0 to 0·0)	4670·4 (4315·4 to 5225·3)	3416·1 (3093·8 to 3955·8)	731·6 (704·9 to 765·4)	522·7 (420·8 to 654·7)	0·0 (0·0 to 0·0)
Spain	2050	3312·8 (3063·3 to 3707·8)	2421·4 (2194·1 to 2807·1)	514·9 (495·8 to 539·0)	376·6 (301·0 to 473·9)	0·0 (0·0 to 0·0)	4712·0 (4357·2 to 5274·0)	3444·1 (3120·9 to 3992·8)	732·3 (705·2 to 766·7)	535·6 (428·1 to 674·1)	0·0 (0·0 to 0·0)
Estonia	1995	574·9 (546·6 to 605·4)	488·6 (462·9 to 514·6)	66·2 (54·5 to 79·4)	20·1 (14·7 to 27·3)	0·0 (0·0 to 0·0)	926·1 (880·6 to 975·3)	787·1 (745·7 to 829·0)	106·7 (87·8 to 127·9)	32·3 (23·7 to 44·1)	0·0 (0·0 to 0·0)
Estonia	1996	603·6 (579·9 to 625·6)	510·7 (491·9 to 530·3)	72·4 (61·3 to 85·4)	20·4 (14·8 to 27·3)	0·0 (0·0 to 0·0)	972·3 (934·2 to 1007·8)	822·8 (792·5 to 854·3)	116·7 (98·7 to 137·6)	32·8 (23·9 to 44·0)	0·1 (0·1 to 0·1)
Estonia	1997	619·6 (595·6 to 641·9)	520·4 (500·8 to 539·8)	78·6 (67·6 to 90·3)	20·5 (15·0 to 27·6)	0·1 (0·1 to 0·1)	998·2 (959·5 to 1034·1)	838·3 (806·8 to 869·5)	126·6 (108·9 to 145·5)	33·1 (24·2 to 44·5)	0·2 (0·2 to 0·2)
Estonia	1998	625·7 (601·4 to 648·6)	516·0 (497·5 to 535·8)	88·6 (77·8 to 99·8)	20·7 (15·1 to 27·7)	0·4 (0·4 to 0·4)	1008·0 (968·9 to 1044·9)	831·3 (801·5 to 863·2)	142·7 (125·3 to 160·7)	33·3 (24·4 to 44·6)	0·7 (0·7 to 0·7)

Estonia	1999	604·1 (582·8 to 626·4)	483·9 (467·0 to 501·0)	98·5 (88·4 to 109·3)	20·2 (14·6 to 26·7)	1·5 (1·5 to 1·5)	973·2 (938·9 to 1009·1)	779·5 (752·2 to 807·1)	158·7 (142·5 to 176·1)	32·6 (23·6 to 43·0)	2·4 (2·4 to 2·4)
Estonia	2000	593·1 (574·1 to 611·1)	461·9 (447·9 to 476·2)	111·1 (100·9 to 121·8)	19·9 (14·7 to 26·2)	0·2 (0·2 to 0·2)	955·5 (924·8 to 984·4)	744·1 (721·5 to 767·2)	179·0 (162·6 to 196·2)	32·1 (23·7 to 42·3)	0·3 (0·3 to 0·3)
Estonia	2001	596·1 (578·8 to 612·1)	458·5 (445·1 to 471·0)	119·5 (110·6 to 129·3)	18·1 (13·5 to 23·9)	0·0 (0·0 to 0·0)	960·3 (932·4 to 986·1)	738·6 (717·0 to 758·8)	192·5 (178·1 to 208·4)	29·1 (21·7 to 38·6)	0·0 (0·0 to 0·0)
Estonia	2002	644·1 (626·9 to 660·4)	491·0 (478·0 to 504·3)	133·8 (124·8 to 144·0)	19·4 (14·5 to 25·3)	0·0 (0·0 to 0·0)	1037·6 (1009·9 to 1063·9)	791·0 (770·0 to 812·4)	215·5 (201·1 to 232·0)	31·2 (23·4 to 40·7)	0·0 (0·0 to 0·0)
Estonia	2003	708·8 (692·2 to 726·2)	535·6 (522·0 to 549·7)	151·7 (142·7 to 161·7)	20·5 (15·7 to 26·2)	1·0 (1·0 to 1·0)	1141·9 (1115·2 to 1169·8)	862·8 (840·9 to 885·6)	244·4 (229·9 to 260·4)	33·1 (25·4 to 42·2)	1·6 (1·6 to 1·6)
Estonia	2004	778·4 (762·0 to 795·6)	581·6 (568·2 to 596·0)	173·5 (163·5 to 183·8)	21·6 (17·2 to 26·9)	1·8 (1·8 to 1·8)	1254·0 (1227·6 to 1281·8)	936·9 (915·3 to 960·1)	279·5 (263·4 to 296·0)	34·7 (27·8 to 43·3)	2·8 (2·8 to 2·8)
Estonia	2005	850·8 (833·4 to 867·8)	634·1 (621·3 to 647·9)	193·6 (183·5 to 204·0)	20·7 (17·1 to 24·9)	2·4 (2·4 to 2·4)	1370·6 (1342·6 to 1398·0)	1021·5 (1000·9 to 1043·8)	311·8 (295·5 to 328·6)	33·4 (27·6 to 40·2)	3·9 (3·9 to 3·9)
Estonia	2006	912·4 (894·2 to 929·6)	685·4 (671·8 to 699·8)	209·6 (199·7 to 220·3)	17·4 (15·0 to 20·4)	0·0 (0·0 to 0·0)	1469·9 (1440·5 to 1497·5)	1104·2 (1082·3 to 1127·3)	337·6 (321·7 to 354·9)	28·0 (24·1 to 32·8)	0·0 (0·0 to 0·0)
Estonia	2007	1006·4 (989·3 to 1025·0)	761·0 (746·8 to 775·3)	224·8 (214·9 to 236·1)	20·5 (18·1 to 23·2)	0·0 (0·0 to 0·0)	1621·3 (1593·7 to 1651·3)	1226·0 (1203·1 to 1249·0)	362·2 (346·1 to 380·4)	33·1 (29·2 to 37·4)	0·0 (0·0 to 0·0)
Estonia	2008	1099·7 (1080·7 to 1118·6)	841·6 (826·9 to 857·5)	232·7 (222·2 to 243·8)	25·4 (23·0 to 28·2)	0·0 (0·0 to 0·0)	1771·6 (1741·0 to 1802·0)	1355·7 (1332·1 to 1381·4)	374·9 (358·0 to 392·7)	41·0 (37·1 to 45·4)	0·0 (0·0 to 0·0)
Estonia	2009	1099·5 (1080·9 to 1119·2)	844·9 (830·1 to 860·1)	229·9 (220·2 to 240·5)	24·7 (22·3 to 27·1)	0·0 (0·0 to 0·0)	1771·2 (1741·3 to 1802·9)	1361·1 (1337·2 to 1385·7)	370·4 (354·7 to 387·5)	39·7 (36·0 to 43·7)	0·0 (0·0 to 0·0)
Estonia	2010	1045·3 (1027·9 to 1063·2)	795·0 (781·6 to 809·5)	227·6 (217·4 to 237·7)	22·7 (20·5 to 25·1)	0·0 (0·0 to 0·0)	1683·9 (1655·9 to 1712·8)	1280·7 (1259·1 to 1304·1)	366·6 (350·2 to 383·0)	36·6 (33·0 to 40·4)	0·0 (0·0 to 0·0)
Estonia	2011	1049·7 (1032·6 to 1067·5)	796·7 (783·1 to 811·4)	231·2 (221·4 to 241·3)	21·8 (19·6 to 24·1)	0·0 (0·0 to 0·0)	1691·1 (1663·4 to 1719·8)	1283·5 (1261·6 to 1307·1)	372·4 (356·7 to 388·7)	35·2 (31·6 to 38·8)	0·0 (0·0 to 0·0)
Estonia	2012	1117·5 (1100·2 to 1135·1)	851·5 (837·1 to 866·0)	244·4 (234·3 to 255·7)	21·6 (19·5 to 23·8)	0·0 (0·0 to 0·0)	1800·2 (1772·3 to 1828·5)	1371·7 (1348·6 to 1395·1)	393·7 (377·4 to 412·0)	34·9 (31·4 to 38·3)	0·0 (0·0 to 0·0)

Estonia	2013	1156·6 (1138·7 to 1174·5)	876·8 (862·5 to 891·7)	258·3 (247·6 to 271·1)	21·4 (19·3 to 23·7)	0·0 (0·0 to 0·0)	1863·2 (1834·4 to 1892·1)	1412·5 (1389·5 to 1436·6)	416·2 (398·9 to 436·7)	34·5 (31·2 to 38·1)	0·0 (0·0 to 0·0)
Estonia	2014	1213·2 (1194·9 to 1232·5)	916·7 (902·1 to 931·3)	274·9 (263·4 to 287·5)	21·6 (19·5 to 23·8)	0·0 (0·0 to 0·0)	1954·4 (1924·9 to 1985·5)	1476·7 (1453·3 to 1500·3)	442·9 (424·4 to 463·1)	34·7 (31·4 to 38·3)	0·0 (0·0 to 0·0)
Estonia	2015	1302·5 (1283·0 to 1322·5)	983·2 (967·5 to 998·7)	297·1 (285·3 to 309·0)	22·1 (20·0 to 24·4)	0·0 (0·0 to 0·0)	2098·3 (2066·8 to 2130·5)	1584·0 (1558·6 to 1608·9)	478·7 (459·6 to 497·8)	35·7 (32·3 to 39·3)	0·0 (0·0 to 0·0)
Estonia	2016	1366·4 (1348·1 to 1386·2)	1022·9 (1008·5 to 1039·0)	320·6 (309·1 to 331·9)	22·9 (20·9 to 25·4)	0·0 (0·0 to 0·0)	2201·3 (2171·7 to 2233·2)	1647·9 (1624·6 to 1673·8)	516·4 (497·9 to 534·7)	36·9 (33·7 to 40·9)	0·0 (0·0 to 0·0)
Estonia	2017	1463·8 (1444·3 to 1483·2)	1083·3 (1067·5 to 1099·6)	356·4 (344·8 to 368·4)	24·1 (22·0 to 26·6)	0·0 (0·0 to 0·0)	2358·2 (2326·8 to 2389·4)	1745·1 (1719·8 to 1771·5)	574·2 (555·5 to 593·4)	38·8 (35·4 to 42·8)	0·0 (0·0 to 0·0)
Estonia	2018	1558·0 (1532·8 to 1584·4)	1145·7 (1125·7 to 1166·2)	385·9 (369·9 to 402·8)	26·4 (23·5 to 29·7)	0·0 (0·0 to 0·0)	2509·8 (2469·3 to 2552·4)	1845·7 (1813·5 to 1878·7)	621·6 (595·9 to 648·9)	42·5 (37·9 to 47·9)	0·0 (0·0 to 0·0)
Estonia	2019	1628·2 (1586·2 to 1669·0)	1198·2 (1160·6 to 1235·0)	402·0 (385·4 to 419·6)	27·9 (24·9 to 31·5)	0·0 (0·0 to 0·0)	2622·9 (2555·3 to 2688·8)	1930·3 (1869·6 to 1989·6)	647·6 (620·8 to 675·9)	45·0 (40·2 to 50·8)	0·0 (0·0 to 0·0)
Estonia	2020	1845·3 (1803·2 to 1885·9)	1430·0 (1392·3 to 1466·9)	387·8 (371·8 to 404·6)	27·5 (24·6 to 31·0)	0·0 (0·0 to 0·0)	2972·8 (2904·9 to 3038·1)	2303·7 (2242·9 to 2363·2)	624·7 (599·0 to 651·7)	44·4 (39·6 to 50·0)	0·0 (0·0 to 0·0)
Estonia	2021	1575·8 (1526·8 to 1627·2)	1174·3 (1128·5 to 1222·4)	374·3 (358·9 to 390·5)	27·2 (24·3 to 30·5)	0·0 (0·0 to 0·0)	2538·5 (2459·6 to 2621·3)	1891·8 (1818·0 to 1969·2)	602·9 (578·2 to 629·1)	43·8 (39·1 to 49·2)	0·0 (0·0 to 0·0)
Estonia	2022	1553·6 (1493·0 to 1620·9)	1150·7 (1094·7 to 1214·7)	375·0 (359·6 to 391·3)	27·8 (24·8 to 31·3)	0·0 (0·0 to 0·0)	2502·7 (2405·2 to 2611·3)	1853·7 (1763·5 to 1956·8)	604·2 (579·3 to 630·4)	44·8 (40·0 to 50·5)	0·0 (0·0 to 0·0)
Estonia	2023	1571·3 (1500·7 to 1647·3)	1166·8 (1097·4 to 1239·0)	376·0 (360·5 to 392·5)	28·5 (25·4 to 32·1)	0·0 (0·0 to 0·0)	2531·4 (2417·6 to 2653·7)	1879·7 (1767·9 to 1996·0)	605·8 (580·8 to 632·4)	45·9 (40·9 to 51·8)	0·0 (0·0 to 0·0)
Estonia	2024	1589·6 (1513·9 to 1668·4)	1183·4 (1110·6 to 1258·1)	377·0 (361·4 to 393·5)	29·2 (26·0 to 32·9)	0·0 (0·0 to 0·0)	2560·8 (2438·8 to 2687·7)	1906·4 (1789·1 to 2026·8)	607·4 (582·2 to 634·0)	47·1 (41·8 to 53·0)	0·0 (0·0 to 0·0)
Estonia	2025	1609·1 (1525·4 to 1694·9)	1201·0 (1121·1 to 1282·9)	378·2 (362·6 to 394·7)	30·0 (26·5 to 33·7)	0·0 (0·0 to 0·0)	2592·3 (2457·3 to 2730·4)	1934·7 (1806·1 to 2066·7)	609·3 (584·2 to 635·9)	48·3 (42·8 to 54·4)	0·0 (0·0 to 0·0)
Estonia	2026	1630·8 (1541·5 to 1725·1)	1220·1 (1132·3 to 1312·0)	380·0 (364·0 to 396·5)	30·8 (27·2 to 34·7)	0·0 (0·0 to 0·0)	2627·2 (2483·3 to 2779·1)	1965·5 (1824·2 to 2113·5)	612·1 (586·3 to 638·7)	49·6 (43·9 to 56·0)	0·0 (0·0 to 0·0)

Estonia	2027	1652·7 (1555·5 to 1756·2)	1239·0 (1146·1 to 1339·2)	382·1 (366·2 to 398·6)	31·6 (27·9 to 35·7)	0·0 (0·0 to 0·0)	2662·5 (2505·9 to 2829·2)	1995·9 (1846·4 to 2157·4)	615·6 (589·9 to 642·1)	51·0 (44·9 to 57·5)	0·0 (0·0 to 0·0)
Estonia	2028	1677·0 (1573·5 to 1784·4)	1259·6 (1161·1 to 1364·4)	384·9 (368·6 to 401·4)	32·6 (28·6 to 37·0)	0·0 (0·0 to 0·0)	2701·6 (2534·9 to 2874·6)	2029·1 (1870·5 to 2197·9)	620·0 (593·8 to 646·7)	52·5 (46·1 to 59·6)	0·0 (0·0 to 0·0)
Estonia	2029	1705·0 (1595·9 to 1818·8)	1282·8 (1177·1 to 1393·2)	388·6 (372·1 to 405·4)	33·6 (29·5 to 38·2)	0·0 (0·0 to 0·0)	2746·7 (2571·0 to 2930·1)	2066·5 (1896·3 to 2244·4)	626·0 (599·4 to 653·1)	54·2 (47·4 to 61·5)	0·0 (0·0 to 0·0)
Estonia	2030	1732·4 (1617·6 to 1847·0)	1305·4 (1193·4 to 1421·2)	392·3 (375·6 to 409·4)	34·7 (30·3 to 39·5)	0·0 (0·0 to 0·0)	2790·8 (2605·9 to 2975·4)	2102·9 (1922·5 to 2289·4)	632·0 (605·1 to 659·5)	55·9 (48·8 to 63·6)	0·0 (0·0 to 0·0)
Estonia	2031	1760·4 (1639·5 to 1883·7)	1328·3 (1210·5 to 1448·7)	396·2 (379·3 to 413·5)	35·8 (31·2 to 40·9)	0·0 (0·0 to 0·0)	2835·9 (2641·2 to 3034·6)	2139·9 (1950·0 to 2333·7)	638·2 (611·1 to 666·1)	57·7 (50·3 to 65·8)	0·0 (0·0 to 0·0)
Estonia	2032	1787·0 (1659·0 to 1921·2)	1350·5 (1224·1 to 1480·0)	399·5 (382·6 to 417·3)	37·0 (32·2 to 42·3)	0·0 (0·0 to 0·0)	2878·8 (2672·6 to 3095·0)	2175·6 (1972·0 to 2384·3)	643·7 (616·3 to 672·2)	59·5 (51·8 to 68·1)	0·0 (0·0 to 0·0)
Estonia	2033	1813·5 (1677·0 to 1950·1)	1372·6 (1238·2 to 1508·8)	402·8 (385·7 to 420·8)	38·1 (33·1 to 43·8)	0·0 (0·0 to 0·0)	2921·5 (2701·7 to 3141·5)	2211·2 (1994·7 to 2430·6)	648·9 (621·4 to 677·9)	61·4 (53·3 to 70·5)	0·0 (0·0 to 0·0)
Estonia	2034	1839·4 (1701·1 to 1985·6)	1394·2 (1259·0 to 1536·9)	405·9 (388·7 to 424·1)	39·3 (34·0 to 45·3)	0·0 (0·0 to 0·0)	2963·3 (2740·4 to 3198·7)	2246·1 (2028·1 to 2475·9)	653·9 (626·2 to 683·3)	63·3 (54·8 to 72·9)	0·0 (0·0 to 0·0)
Estonia	2035	1865·5 (1719·3 to 2018·3)	1416·0 (1274·8 to 1569·9)	409·0 (391·7 to 427·7)	40·5 (34·9 to 46·8)	0·0 (0·0 to 0·0)	3005·2 (2769·8 to 3251·5)	2281·1 (2053·7 to 2529·1)	659·0 (631·0 to 689·0)	65·2 (56·2 to 75·4)	0·0 (0·0 to 0·0)
Estonia	2036	1891·2 (1739·7 to 2047·4)	1437·6 (1289·8 to 1592·2)	411·9 (394·4 to 430·9)	41·7 (35·8 to 48·7)	0·0 (0·0 to 0·0)	3046·7 (2802·7 to 3298·2)	2316·0 (2077·9 to 2564·9)	663·6 (635·4 to 694·1)	67·1 (57·7 to 78·5)	0·0 (0·0 to 0·0)
Estonia	2037	1915·5 (1758·6 to 2081·5)	1458·1 (1300·0 to 1619·5)	414·5 (396·7 to 433·5)	42·9 (36·5 to 50·4)	0·0 (0·0 to 0·0)	3085·8 (2833·0 to 3353·2)	2349·0 (2094·3 to 2608·9)	667·7 (639·0 to 698·3)	69·1 (58·9 to 81·2)	0·0 (0·0 to 0·0)
Estonia	2038	1941·0 (1776·8 to 2107·4)	1479·7 (1314·3 to 1643·3)	417·2 (399·2 to 436·4)	44·1 (37·4 to 52·3)	0·0 (0·0 to 0·0)	3126·8 (2862·3 to 3394·9)	2383·7 (2117·3 to 2647·2)	672·1 (643·0 to 703·0)	71·1 (60·3 to 84·2)	0·0 (0·0 to 0·0)
Estonia	2039	1965·8 (1799·5 to 2140·7)	1500·8 (1336·5 to 1678·7)	419·6 (401·5 to 438·8)	45·4 (38·2 to 53·9)	0·0 (0·0 to 0·0)	3166·8 (2899·0 to 3448·6)	2417·8 (2153·0 to 2704·4)	675·9 (646·8 to 707·0)	73·1 (61·6 to 86·8)	0·0 (0·0 to 0·0)
Estonia	2040	1990·4 (1810·8 to 2173·2)	1521·7 (1345·5 to 1706·4)	422·0 (404·0 to 441·5)	46·7 (39·0 to 55·7)	0·0 (0·0 to 0·0)	3206·4 (2917·1 to 3501·0)	2451·4 (2167·6 to 2748·9)	679·9 (650·8 to 711·3)	75·2 (62·9 to 89·8)	0·0 (0·0 to 0·0)

Estonia	2041	2014·5 (1825·4 to 2200·7)	1542·1 (1352·2 to 1726·7)	424·4 (406·3 to 444·1)	48·0 (39·8 to 57·6)	0·0 (0·0 to 0·0)	3245·3 (2940·6 to 3545·3)	2484·3 (2178·4 to 2781·6)	683·7 (654·5 to 715·5)	77·3 (64·2 to 92·8)	0·0 (0·0 to 0·0)
Estonia	2042	2038·8 (1848·3 to 2230·6)	1562·7 (1368·5 to 1755·9)	426·8 (408·5 to 446·5)	49·4 (40·7 to 59·7)	0·0 (0·0 to 0·0)	3284·5 (2977·5 to 3593·4)	2517·4 (2204·5 to 2828·7)	687·6 (658·1 to 719·3)	79·5 (65·5 to 96·2)	0·0 (0·0 to 0·0)
Estonia	2043	2062·5 (1866·9 to 2263·2)	1582·9 (1385·1 to 1788·9)	428·9 (410·4 to 448·5)	50·7 (41·5 to 61·8)	0·0 (0·0 to 0·0)	3322·6 (3007·4 to 3645·9)	2550·0 (2231·3 to 2881·8)	690·9 (661·1 to 722·5)	81·7 (66·9 to 99·6)	0·0 (0·0 to 0·0)
Estonia	2044	2085·4 (1880·7 to 2295·7)	1602·3 (1406·0 to 1818·8)	431·0 (412·4 to 450·5)	52·1 (42·4 to 63·8)	0·0 (0·0 to 0·0)	3359·5 (3029·8 to 3698·3)	2581·2 (2265·0 to 2930·0)	694·3 (664·4 to 725·7)	84·0 (68·2 to 102·8)	0·0 (0·0 to 0·0)
Estonia	2045	2108·1 (1898·8 to 2324·5)	1621·6 (1416·3 to 1840·2)	433·0 (414·1 to 452·6)	53·6 (43·1 to 65·9)	0·0 (0·0 to 0·0)	3396·1 (3058·9 to 3744·6)	2612·3 (2281·7 to 2964·5)	697·5 (667·2 to 729·2)	86·3 (69·4 to 106·1)	0·0 (0·0 to 0·0)
Estonia	2046	2129·3 (1921·5 to 2345·2)	1639·7 (1429·4 to 1857·3)	434·6 (415·5 to 454·4)	55·0 (43·9 to 68·4)	0·0 (0·0 to 0·0)	3430·2 (3095·5 to 3778·0)	2641·6 (2302·7 to 2992·0)	700·1 (669·3 to 732·0)	88·6 (70·8 to 110·2)	0·0 (0·0 to 0·0)
Estonia	2047	2150·6 (1935·9 to 2368·2)	1658·1 (1446·4 to 1876·8)	436·0 (416·9 to 456·3)	56·4 (44·7 to 70·6)	0·0 (0·0 to 0·0)	3464·5 (3118·7 to 3815·1)	2671·2 (2330·2 to 3023·5)	702·4 (671·6 to 735·0)	90·9 (72·0 to 113·7)	0·0 (0·0 to 0·0)
Estonia	2048	2171·1 (1943·0 to 2402·1)	1676·0 (1451·5 to 1905·8)	437·2 (417·9 to 457·9)	57·9 (45·5 to 72·6)	0·0 (0·0 to 0·0)	3497·6 (3130·1 to 3869·8)	2700·0 (2338·3 to 3070·1)	704·4 (673·2 to 737·7)	93·2 (73·2 to 117·0)	0·0 (0·0 to 0·0)
Estonia	2049	2191·6 (1960·2 to 2431·6)	1694·1 (1466·5 to 1942·8)	438·2 (418·6 to 458·8)	59·3 (46·2 to 75·0)	0·0 (0·0 to 0·0)	3530·7 (3157·8 to 3917·3)	2729·2 (2362·5 to 3129·7)	705·9 (674·3 to 739·1)	95·6 (74·5 to 120·8)	0·0 (0·0 to 0·0)
Estonia	2050	2210·7 (1973·3 to 2456·0)	1711·0 (1483·8 to 1955·6)	438·9 (419·0 to 460·0)	60·8 (47·1 to 77·8)	0·0 (0·0 to 0·0)	3561·4 (3178·9 to 3956·5)	2756·4 (2390·4 to 3150·3)	707·1 (675·0 to 741·1)	97·9 (75·9 to 125·3)	0·0 (0·0 to 0·0)
Ethiopia*	1995	10·4 (9·1 to 11·9)	4·5 (3·7 to 5·6)	4·1 (3·2 to 5·0)	0·7 (0·5 to 1·0)	1·2 (1·2 to 1·2)	29·6 (26·0 to 33·8)	12·7 (10·4 to 15·8)	11·5 (9·2 to 14·4)	2·0 (1·3 to 2·7)	3·4 (3·4 to 3·4)
Ethiopia*	1996	10·9 (9·7 to 12·4)	4·6 (3·8 to 5·6)	4·1 (3·3 to 5·2)	0·7 (0·5 to 1·0)	1·5 (1·5 to 1·5)	31·1 (27·5 to 35·2)	13·1 (10·7 to 16·1)	11·7 (9·5 to 14·7)	1·9 (1·3 to 2·8)	4·3 (4·3 to 4·3)
Ethiopia*	1997	10·4 (9·2 to 11·8)	4·5 (3·7 to 5·5)	4·1 (3·3 to 5·1)	0·7 (0·4 to 1·0)	1·1 (1·1 to 1·1)	29·6 (26·2 to 33·5)	12·7 (10·4 to 15·7)	11·7 (9·5 to 14·5)	1·9 (1·3 to 2·8)	3·2 (3·2 to 3·2)
Ethiopia*	1998	10·4 (9·2 to 11·7)	4·5 (3·7 to 5·5)	4·2 (3·4 to 5·0)	0·7 (0·5 to 1·0)	1·0 (1·0 to 1·0)	29·5 (26·1 to 33·3)	12·9 (10·6 to 15·7)	11·8 (9·7 to 14·3)	2·1 (1·3 to 3·0)	2·8 (2·8 to 2·8)
Ethiopia*	1999	11·9 (10·6 to 13·3)	5·0 (4·2 to 6·2)	4·3 (3·6 to 5·2)	0·8 (0·6 to 1·2)	1·7 (1·7 to 1·7)	33·8 (30·2 to 37·7)	14·3 (11·8 to 17·6)	12·4 (10·2 to 14·8)	2·4 (1·6 to 3·5)	4·7 (4·7 to 4·7)

Ethiopia*	2000	11·8 (10·6 to 13·2)	5·1 (4·2 to 6·3)	4·3 (3·6 to 5·2)	0·8 (0·6 to 1·2)	1·5 (1·5 to 1·5)	33·6 (30·1 to 37·7)	14·6 (12·1 to 17·9)	12·3 (10·2 to 14·7)	2·3 (1·6 to 3·3)	4·3 (4·3 to 4·3)
Ethiopia*	2001	12·0 (10·7 to 13·2)	5·1 (4·2 to 6·3)	4·4 (3·7 to 5·2)	0·8 (0·5 to 1·1)	1·6 (1·6 to 1·6)	34·0 (30·5 to 37·7)	14·6 (12·0 to 17·9)	12·5 (10·4 to 14·9)	2·2 (1·5 to 3·2)	4·6 (4·6 to 4·6)
Ethiopia*	2002	12·5 (11·2 to 13·9)	5·3 (4·4 to 6·5)	4·6 (3·8 to 5·4)	0·8 (0·5 to 1·1)	1·8 (1·8 to 1·8)	35·5 (31·9 to 39·5)	15·2 (12·4 to 18·5)	13·0 (10·9 to 15·4)	2·2 (1·5 to 3·1)	5·2 (5·2 to 5·2)
Ethiopia*	2003	13·6 (12·3 to 15·1)	5·2 (4·3 to 6·2)	4·6 (3·8 to 5·4)	0·7 (0·5 to 1·0)	3·2 (3·2 to 3·2)	38·8 (35·1 to 42·8)	14·8 (12·1 to 17·8)	13·0 (10·8 to 15·4)	2·0 (1·4 to 2·9)	9·0 (9·0 to 9·0)
Ethiopia*	2004	12·8 (11·7 to 14·1)	4·6 (3·8 to 5·5)	4·5 (3·8 to 5·3)	0·6 (0·4 to 0·9)	3·1 (3·1 to 3·1)	36·5 (33·3 to 40·1)	13·0 (10·7 to 15·5)	12·8 (10·9 to 15·1)	1·8 (1·3 to 2·7)	8·8 (8·8 to 8·8)
Ethiopia*	2005	13·9 (12·7 to 15·2)	4·6 (3·8 to 5·5)	4·8 (4·1 to 5·7)	0·6 (0·4 to 0·9)	3·8 (3·8 to 3·8)	39·4 (36·3 to 43·2)	13·1 (10·7 to 15·7)	13·8 (11·6 to 16·1)	1·8 (1·3 to 2·7)	10·7 (10·7 to 10·7)
Ethiopia*	2006	16·2 (15·1 to 17·5)	4·2 (3·5 to 5·0)	5·5 (4·7 to 6·3)	0·7 (0·5 to 0·9)	5·9 (5·9 to 5·9)	46·2 (43·1 to 49·8)	12·0 (9·9 to 14·4)	15·6 (13·4 to 18·0)	1·9 (1·3 to 2·6)	16·8 (16·8 to 16·8)
Ethiopia*	2007	18·5 (17·4 to 19·7)	4·0 (3·3 to 4·8)	6·3 (5·4 to 7·2)	0·7 (0·5 to 1·0)	7·5 (7·5 to 7·5)	52·7 (49·4 to 56·1)	11·4 (9·4 to 13·8)	17·9 (15·5 to 20·5)	2·0 (1·4 to 2·8)	21·4 (21·4 to 21·4)
Ethiopia*	2008	18·5 (17·4 to 19·7)	3·4 (2·8 to 4·1)	6·9 (5·9 to 7·9)	0·8 (0·5 to 1·0)	7·5 (7·5 to 7·5)	52·7 (49·6 to 56·2)	9·7 (8·0 to 11·7)	19·6 (16·9 to 22·5)	2·2 (1·5 to 3·0)	21·2 (21·2 to 21·2)
Ethiopia*	2009	20·3 (19·1 to 21·7)	3·5 (2·9 to 4·2)	8·0 (6·9 to 9·1)	1·1 (0·8 to 1·6)	7·7 (7·7 to 7·7)	57·9 (54·3 to 61·8)	9·9 (8·2 to 11·9)	22·7 (19·7 to 26·0)	3·3 (2·3 to 4·5)	22·0 (22·0 to 22·0)
Ethiopia*	2010	23·6 (22·2 to 25·2)	3·8 (3·2 to 4·5)	9·3 (8·1 to 10·7)	1·6 (1·2 to 2·2)	8·8 (8·8 to 8·8)	67·2 (63·0 to 71·8)	10·8 (9·0 to 12·9)	26·6 (23·2 to 30·4)	4·7 (3·4 to 6·3)	25·1 (25·1 to 25·1)
Ethiopia*	2011	25·8 (24·3 to 27·4)	3·7 (3·1 to 4·4)	9·8 (8·6 to 11·1)	2·0 (1·4 to 2·6)	10·3 (10·3 to 10·3)	73·4 (69·1 to 78·1)	10·5 (8·9 to 12·5)	27·9 (24·4 to 31·7)	5·6 (4·1 to 7·3)	29·3 (29·3 to 29·3)
Ethiopia*	2012	25·5 (23·9 to 27·2)	4·3 (3·6 to 5·1)	10·1 (8·9 to 11·4)	2·3 (1·8 to 2·9)	8·8 (8·8 to 8·8)	72·5 (67·9 to 77·3)	12·2 (10·3 to 14·4)	28·7 (25·4 to 32·5)	6·6 (5·0 to 8·3)	24·9 (24·9 to 24·9)
Ethiopia*	2013	29·8 (28·2 to 31·4)	5·0 (4·2 to 5·9)	10·3 (9·2 to 11·5)	3·1 (2·4 to 3·8)	11·4 (11·4 to 11·4)	84·8 (80·4 to 89·4)	14·4 (12·1 to 16·8)	29·4 (26·3 to 32·9)	8·7 (6·9 to 10·8)	32·4 (32·4 to 32·4)
Ethiopia*	2014	28·4 (26·8 to 30·1)	5·2 (4·4 to 6·1)	10·1 (9·1 to 11·3)	4·7 (3·8 to 5·7)	8·4 (8·4 to 8·4)	80·9 (76·3 to 85·8)	14·8 (12·5 to 17·3)	28·7 (25·9 to 32·0)	13·4 (10·9 to 16·1)	24·0 (24·0 to 24·0)
Ethiopia*	2015	28·6 (26·9 to 30·5)	5·7 (4·8 to 6·7)	9·9 (9·0 to 11·0)	5·2 (4·2 to 6·4)	7·8 (7·8 to 7·8)	81·3 (76·7 to 86·8)	16·3 (13·6 to 19·0)	28·2 (25·6 to 31·3)	14·7 (11·9 to 18·3)	22·1 (22·1 to 22·1)
Ethiopia*	2016	27·7 (26·0 to 29·8)	6·3 (5·3 to 7·4)	9·8 (8·9 to 10·9)	4·6 (3·5 to 5·8)	7·0 (7·0 to 7·0)	78·9 (74·0 to 84·8)	18·0 (15·1 to 21·1)	27·9 (25·3 to 31·0)	12·9 (10·0 to 16·6)	20·0 (20·0 to 20·0)
Ethiopia*	2017	27·0 (25·4 to 28·9)	6·6 (5·5 to 7·8)	9·7 (8·7 to 10·8)	2·6 (2·0 to 3·5)	8·1 (8·1 to 8·1)	76·9 (72·2 to 82·3)	18·8 (15·7 to 22·1)	27·5 (24·7 to 30·8)	7·5 (5·6 to 9·8)	23·1 (23·1 to 23·1)

Ethiopia*	2018	26·4 (24·6 to 28·3)	6·5 (5·5 to 7·7)	9·6 (8·4 to 10·9)	2·1 (1·6 to 2·8)	8·1 (8·1 to 8·1)	75·0 (70·0 to 80·5)	18·6 (15·6 to 22·0)	27·4 (24·0 to 31·0)	6·0 (4·4 to 8·1)	23·0 (23·0 to 23·0)
Ethiopia*	2019	26·3 (24·4 to 28·2)	6·5 (5·4 to 7·8)	10·2 (8·9 to 11·5)	2·3 (1·7 to 3·0)	7·3 (7·2 to 7·4)	74·8 (69·4 to 80·2)	18·6 (15·5 to 22·1)	28·9 (25·3 to 32·8)	6·5 (4·7 to 8·6)	20·8 (20·6 to 21·0)
Ethiopia*	2020	32·4 (30·6 to 34·2)	10·8 (9·8 to 12·0)	9·5 (8·3 to 10·8)	2·2 (1·6 to 2·9)	9·8 (9·7 to 9·9)	92·1 (87·0 to 97·3)	30·8 (27·9 to 34·3)	27·1 (23·7 to 30·7)	6·2 (4·5 to 8·2)	27·9 (27·6 to 28·1)
Ethiopia*	2021	26·5 (24·9 to 28·3)	5·6 (4·7 to 6·7)	9·0 (7·9 to 10·3)	2·1 (1·5 to 2·8)	9·7 (9·6 to 9·9)	75·5 (70·8 to 80·4)	16·0 (13·4 to 19·1)	25·7 (22·5 to 29·2)	6·0 (4·4 to 7·9)	27·7 (27·2 to 28·2)
Ethiopia*	2022	24·2 (22·6 to 26·0)	5·6 (4·7 to 6·6)	9·2 (8·1 to 10·5)	2·2 (1·6 to 2·9)	7·2 (6·9 to 7·6)	69·0 (64·2 to 73·9)	15·9 (13·4 to 18·8)	26·2 (23·0 to 29·8)	6·2 (4·6 to 8·3)	20·6 (19·6 to 21·7)
Ethiopia*	2023	24·4 (22·6 to 26·2)	5·6 (4·7 to 6·7)	9·4 (8·2 to 10·7)	2·3 (1·7 to 3·0)	7·1 (6·7 to 7·5)	69·4 (64·4 to 74·6)	16·0 (13·4 to 19·0)	26·8 (23·4 to 30·3)	6·5 (4·7 to 8·6)	20·1 (19·0 to 21·4)
Ethiopia*	2024	24·6 (22·8 to 26·4)	5·7 (4·7 to 6·7)	9·6 (8·4 to 10·9)	2·4 (1·7 to 3·2)	6·9 (6·5 to 7·4)	70·0 (64·9 to 75·2)	16·1 (13·5 to 19·1)	27·3 (23·9 to 31·0)	6·8 (4·9 to 9·0)	19·7 (18·5 to 21·2)
Ethiopia*	2025	24·8 (22·9 to 26·6)	5·7 (4·8 to 6·8)	9·8 (8·6 to 11·1)	2·5 (1·8 to 3·3)	6·8 (6·3 to 7·3)	70·5 (65·3 to 75·7)	16·3 (13·6 to 19·2)	27·9 (24·4 to 31·7)	7·1 (5·2 to 9·4)	19·2 (17·9 to 20·7)
Ethiopia*	2026	25·0 (23·1 to 26·9)	5·8 (4·8 to 6·9)	10·0 (8·8 to 11·4)	2·6 (1·9 to 3·5)	6·6 (6·1 to 7·1)	71·0 (65·7 to 76·6)	16·4 (13·7 to 19·5)	28·5 (25·0 to 32·4)	7·4 (5·4 to 9·9)	18·7 (17·2 to 20·3)
Ethiopia*	2027	25·2 (23·2 to 27·2)	5·8 (4·8 to 6·9)	10·2 (9·0 to 11·6)	2·7 (2·0 to 3·6)	6·4 (5·8 to 7·0)	71·6 (66·1 to 77·4)	16·6 (13·8 to 19·7)	29·1 (25·5 to 33·1)	7·8 (5·7 to 10·3)	18·2 (16·6 to 19·9)
Ethiopia*	2028	25·4 (23·3 to 27·5)	5·9 (4·9 to 7·0)	10·4 (9·2 to 11·9)	2·8 (2·1 to 3·8)	6·2 (5·6 to 6·9)	72·2 (66·4 to 78·2)	16·7 (13·9 to 20·0)	29·7 (26·0 to 33·8)	8·1 (5·9 to 10·7)	17·7 (15·9 to 19·6)
Ethiopia*	2029	25·6 (23·6 to 27·7)	5·9 (4·9 to 7·1)	10·7 (9·3 to 12·1)	3·0 (2·2 to 3·9)	6·1 (5·4 to 6·8)	72·9 (67·1 to 79·0)	16·9 (14·1 to 20·2)	30·3 (26·6 to 34·5)	8·5 (6·1 to 11·2)	17·2 (15·4 to 19·3)
Ethiopia*	2030	25·9 (23·8 to 28·1)	6·0 (5·0 to 7·2)	10·9 (9·5 to 12·4)	3·1 (2·3 to 4·1)	5·9 (5·2 to 6·7)	73·6 (67·7 to 80·0)	17·1 (14·2 to 20·4)	31·0 (27·1 to 35·2)	8·8 (6·4 to 11·7)	16·7 (14·8 to 18·9)
Ethiopia*	2031	26·2 (24·1 to 28·5)	6·1 (5·1 to 7·3)	11·1 (9·7 to 12·6)	3·2 (2·3 to 4·3)	5·7 (5·0 to 6·6)	74·4 (68·6 to 81·0)	17·3 (14·4 to 20·6)	31·6 (27·7 to 35·9)	9·2 (6·7 to 12·3)	16·3 (14·2 to 18·7)
Ethiopia*	2032	26·5 (24·3 to 28·8)	6·1 (5·1 to 7·3)	11·3 (9·9 to 12·9)	3·4 (2·4 to 4·5)	5·6 (4·8 to 6·5)	75·3 (69·1 to 82·1)	17·5 (14·5 to 20·9)	32·3 (28·3 to 36·7)	9·6 (7·0 to 12·8)	15·9 (13·7 to 18·4)
Ethiopia*	2033	26·8 (24·5 to 29·2)	6·2 (5·1 to 7·4)	11·6 (10·1 to 13·2)	3·5 (2·6 to 4·7)	5·4 (4·6 to 6·5)	76·2 (69·7 to 83·2)	17·7 (14·6 to 21·2)	33·0 (28·9 to 37·5)	10·1 (7·3 to 13·4)	15·5 (13·2 to 18·4)
Ethiopia*	2034	27·1 (24·9 to 29·6)	6·3 (5·2 to 7·6)	11·8 (10·4 to 13·5)	3·7 (2·7 to 4·9)	5·3 (4·5 to 6·4)	77·2 (70·8 to 84·4)	17·9 (14·7 to 21·5)	33·7 (29·5 to 38·3)	10·5 (7·6 to 14·1)	15·1 (12·7 to 18·2)
Ethiopia*	2035	27·5 (25·3 to 30·1)	6·4 (5·3 to 7·7)	12·1 (10·6 to 13·7)	3·9 (2·8 to 5·2)	5·2 (4·3 to 6·3)	78·3 (71·9 to 85·8)	18·2 (15·0 to 21·9)	34·4 (30·1 to 39·1)	11·0 (7·9 to 14·8)	14·7 (12·3 to 18·1)

Ethiopia*	2036	27·9 (25·5 to 30·7)	6·5 (5·3 to 7·9)	12·4 (10·8 to 14·0)	4·0 (2·9 to 5·4)	5·0 (4·2 to 6·3)	79·5 (72·4 to 87·4)	18·5 (15·1 to 22·4)	35·2 (30·8 to 39·9)	11·5 (8·2 to 15·5)	14·3 (11·9 to 17·8)
Ethiopia*	2037	28·4 (25·8 to 31·2)	6·6 (5·4 to 8·1)	12·6 (11·1 to 14·4)	4·2 (3·0 to 5·7)	4·9 (4·1 to 6·2)	80·8 (73·5 to 88·9)	18·9 (15·2 to 23·0)	36·0 (31·5 to 40·9)	12·0 (8·5 to 16·3)	14·0 (11·5 to 17·6)
Ethiopia*	2038	28·9 (26·3 to 31·9)	6·8 (5·4 to 8·3)	12·9 (11·3 to 14·7)	4·4 (3·1 to 6·0)	4·8 (3·9 to 6·1)	82·2 (74·8 to 90·8)	19·3 (15·4 to 23·6)	36·8 (32·2 to 41·9)	12·5 (8·9 to 17·1)	13·6 (11·2 to 17·5)
Ethiopia*	2039	29·4 (26·7 to 32·5)	6·9 (5·5 to 8·6)	13·2 (11·6 to 15·1)	4·6 (3·3 to 6·3)	4·7 (3·8 to 6·1)	83·7 (75·9 to 92·6)	19·7 (15·6 to 24·4)	37·6 (33·0 to 42·8)	13·1 (9·3 to 17·8)	13·3 (10·8 to 17·3)
Ethiopia*	2040	30·0 (27·0 to 33·1)	7·1 (5·6 to 8·8)	13·5 (11·8 to 15·4)	4·8 (3·4 to 6·6)	4·6 (3·6 to 6·0)	85·3 (76·9 to 94·1)	20·1 (15·9 to 25·2)	38·5 (33·7 to 43·8)	13·7 (9·6 to 18·7)	13·0 (10·4 to 17·1)
Ethiopia*	2041	30·6 (27·6 to 33·8)	7·3 (5·7 to 9·2)	13·8 (12·1 to 15·7)	5·0 (3·5 to 6·9)	4·5 (3·6 to 6·0)	87·0 (78·6 to 96·2)	20·7 (16·1 to 26·0)	39·3 (34·4 to 44·8)	14·3 (10·0 to 19·6)	12·7 (10·1 to 16·9)
Ethiopia*	2042	31·2 (28·1 to 34·8)	7·5 (5·8 to 9·5)	14·1 (12·4 to 16·1)	5·2 (3·7 to 7·2)	4·4 (3·5 to 5·9)	88·8 (80·0 to 99·1)	21·3 (16·4 to 26·9)	40·2 (35·2 to 45·8)	14·9 (10·4 to 20·5)	12·4 (9·9 to 16·9)
Ethiopia*	2043	31·9 (28·6 to 35·6)	7·7 (5·9 to 9·8)	14·4 (12·6 to 16·5)	5·5 (3·8 to 7·5)	4·3 (3·3 to 6·0)	90·8 (81·4 to 101·2)	21·9 (16·8 to 28·0)	41·1 (35·9 to 46·9)	15·6 (10·9 to 21·5)	12·2 (9·5 to 17·0)
Ethiopia*	2044	32·6 (29·1 to 36·5)	7·9 (6·0 to 10·2)	14·8 (12·9 to 16·8)	5·7 (4·0 to 7·9)	4·2 (3·2 to 5·9)	92·8 (82·9 to 103·8)	22·6 (17·1 to 29·1)	42·0 (36·6 to 47·9)	16·2 (11·3 to 22·4)	12·0 (9·2 to 16·7)
Ethiopia*	2045	33·3 (29·7 to 37·5)	8·2 (6·2 to 10·7)	15·1 (13·1 to 17·2)	6·0 (4·1 to 8·3)	4·1 (3·2 to 5·8)	94·9 (84·4 to 106·7)	23·3 (17·6 to 30·3)	42·9 (37·4 to 49·0)	17·0 (11·8 to 23·5)	11·7 (9·0 to 16·6)
Ethiopia*	2046	34·1 (30·3 to 38·4)	8·5 (6·3 to 11·1)	15·4 (13·4 to 17·6)	6·2 (4·3 to 8·6)	4·0 (3·1 to 5·9)	97·1 (86·1 to 109·3)	24·1 (18·0 to 31·6)	43·8 (38·2 to 50·1)	17·7 (12·2 to 24·6)	11·5 (8·7 to 16·7)
Ethiopia*	2047	35·0 (31·0 to 39·7)	8·8 (6·5 to 11·6)	15·7 (13·7 to 18·0)	6·5 (4·5 to 9·0)	3·9 (3·0 to 5·7)	99·6 (88·1 to 113·0)	25·0 (18·5 to 33·1)	44·8 (39·1 to 51·3)	18·5 (12·7 to 25·7)	11·2 (8·5 to 16·2)
Ethiopia*	2048	35·8 (31·7 to 40·6)	9·1 (6·7 to 12·2)	16·1 (14·0 to 18·4)	6·8 (4·6 to 9·4)	3·9 (2·9 to 5·6)	102·0 (90·2 to 115·6)	26·0 (19·0 to 34·7)	45·8 (39·9 to 52·4)	19·3 (13·1 to 26·8)	11·0 (8·2 to 15·9)
Ethiopia*	2049	36·7 (32·4 to 41·7)	9·5 (6·9 to 12·7)	16·4 (14·3 to 18·8)	7·1 (4·8 to 9·8)	3·8 (2·8 to 5·5)	104·6 (92·2 to 118·6)	26·9 (19·6 to 36·2)	46·7 (40·7 to 53·6)	20·1 (13·6 to 28·0)	10·8 (8·0 to 15·6)
Ethiopia*	2050	37·7 (33·0 to 42·9)	9·8 (7·1 to 13·3)	16·8 (14·6 to 19·2)	7·4 (5·0 to 10·3)	3·7 (2·8 to 5·4)	107·2 (93·9 to 122·2)	28·0 (20·2 to 37·9)	47·7 (41·5 to 54·7)	21·0 (14·1 to 29·3)	10·6 (7·8 to 15·4)
Finland	1995	2480·8 (2405·1 to 2552·7)	1883·7 (1818·5 to 1953·2)	539·3 (511·6 to 568·6)	57·8 (46·1 to 71·8)	0·0 (0·0 to 0·0)	2526·4 (2449·2 to 2599·5)	1918·3 (1851·9 to 1989·0)	549·2 (521·0 to 579·1)	58·9 (46·9 to 73·2)	0·0 (0·0 to 0·0)

Finland	1996	2562·9 (2499·8 to 2625·7)	1948·7 (1891·9 to 2005·9)	552·4 (530·4 to 574·8)	61·9 (51·3 to 75·3)	0·0 (0·0 to 0·0)	2610·0 (2545·7 to 2673·8)	1984·4 (1926·6 to 2042·7)	562·5 (540·1 to 585·4)	63·0 (52·2 to 76·7)	0·0 (0·0 to 0·0)
Finland	1997	2634·6 (2574·4 to 2699·2)	2010·3 (1957·8 to 2066·2)	559·6 (539·2 to 582·0)	64·8 (55·5 to 76·5)	0·0 (0·0 to 0·0)	2683·0 (2621·7 to 2748·7)	2047·2 (1993·7 to 2104·1)	569·9 (549·1 to 592·7)	65·9 (56·5 to 77·9)	0·0 (0·0 to 0·0)
Finland	1998	2680·8 (2623·8 to 2739·0)	2045·6 (1993·3 to 2098·1)	568·3 (548·6 to 590·8)	66·9 (58·2 to 76·3)	0·0 (0·0 to 0·0)	2730·0 (2672·0 to 2789·2)	2083·1 (2029·9 to 2136·6)	578·7 (558·6 to 601·6)	68·2 (59·3 to 77·7)	0·0 (0·0 to 0·0)
Finland	1999	2787·4 (2732·8 to 2846·3)	2117·6 (2066·2 to 2170·3)	597·9 (578·9 to 618·0)	72·0 (65·3 to 78·7)	0·0 (0·0 to 0·0)	2838·6 (2782·9 to 2898·5)	2156·5 (2104·1 to 2210·2)	608·8 (589·6 to 629·3)	73·3 (66·5 to 80·1)	0·0 (0·0 to 0·0)
Finland	2000	2923·0 (2868·6 to 2973·8)	2217·4 (2164·3 to 2268·6)	628·9 (612·5 to 645·0)	76·7 (72·9 to 80·6)	0·0 (0·0 to 0·0)	2976·6 (2921·3 to 3028·3)	2258·1 (2204·0 to 2310·3)	640·5 (623·7 to 656·8)	78·1 (74·3 to 82·0)	0·0 (0·0 to 0·0)
Finland	2001	3064·3 (3004·1 to 3118·3)	2333·8 (2276·9 to 2385·3)	652·3 (636·4 to 669·0)	78·2 (75·2 to 81·3)	0·0 (0·0 to 0·0)	3120·5 (3059·2 to 3175·5)	2376·6 (2318·7 to 2429·0)	664·3 (648·1 to 681·2)	79·6 (76·6 to 82·8)	0·0 (0·0 to 0·0)
Finland	2002	3263·3 (3212·2 to 3317·2)	2505·5 (2453·8 to 2555·3)	678·5 (662·8 to 694·3)	79·3 (76·2 to 82·4)	0·0 (0·0 to 0·0)	3323·2 (3271·1 to 3378·1)	2551·5 (2498·9 to 2602·2)	691·0 (675·0 to 707·0)	80·7 (77·6 to 83·9)	0·0 (0·0 to 0·0)
Finland	2003	3455·3 (3402·1 to 3509·7)	2671·2 (2619·0 to 2727·2)	702·7 (687·7 to 718·2)	81·5 (78·3 to 84·6)	0·0 (0·0 to 0·0)	3518·7 (3464·6 to 3574·1)	2720·2 (2667·1 to 2777·3)	715·6 (700·4 to 731·4)	82·9 (79·8 to 86·1)	0·0 (0·0 to 0·0)
Finland	2004	3653·0 (3596·6 to 3714·0)	2839·4 (2786·2 to 2895·5)	726·6 (710·8 to 744·0)	87·0 (83·9 to 90·4)	0·0 (0·0 to 0·0)	3720·0 (3662·7 to 3782·1)	2891·5 (2837·3 to 2948·6)	739·9 (723·8 to 757·6)	88·6 (85·5 to 92·1)	0·0 (0·0 to 0·0)
Finland	2005	3825·0 (3766·1 to 3884·6)	2984·9 (2928·5 to 3042·4)	744·2 (728·6 to 760·6)	95·9 (92·8 to 99·1)	0·0 (0·0 to 0·0)	3895·1 (3835·2 to 3955·9)	3039·7 (2982·3 to 3098·2)	757·8 (742·0 to 774·5)	97·6 (94·5 to 100·9)	0·0 (0·0 to 0·0)
Finland	2006	3972·7 (3920·3 to 4034·7)	3119·6 (3068·6 to 3176·9)	755·0 (738·9 to 771·4)	98·1 (95·0 to 101·4)	0·0 (0·0 to 0·0)	4045·6 (3992·2 to 4108·8)	3176·8 (3124·9 to 3235·2)	768·9 (752·4 to 785·5)	99·9 (96·8 to 103·3)	0·0 (0·0 to 0·0)
Finland	2007	4067·6 (4011·2 to 4124·9)	3201·3 (3150·4 to 3255·2)	767·1 (750·9 to 784·1)	99·2 (96·0 to 102·3)	0·0 (0·0 to 0·0)	4142·2 (4084·8 to 4200·6)	3260·0 (3208·3 to 3314·9)	781·2 (764·7 to 798·5)	101·0 (97·8 to 104·2)	0·0 (0·0 to 0·0)
Finland	2008	4189·9 (4134·5 to 4247·2)	3302·4 (3248·5 to 3359·0)	780·0 (763·7 to 797·3)	107·4 (104·3 to 110·7)	0·0 (0·0 to 0·0)	4266·7 (4210·4 to 4325·2)	3363·0 (3308·1 to 3420·6)	794·3 (777·7 to 812·0)	109·4 (106·2 to 112·7)	0·0 (0·0 to 0·0)
Finland	2009	4248·0 (4189·4 to 4307·4)	3350·4 (3294·8 to 3404·7)	785·7 (769·2 to 802·3)	111·9 (108·4 to 115·3)	0·0 (0·0 to 0·0)	4325·9 (4266·2 to 4386·5)	3411·8 (3355·2 to 3467·2)	800·2 (783·4 to 817·0)	113·9 (110·4 to 117·4)	0·0 (0·0 to 0·0)

Finland	2010	4330·0 (4272·8 to 4384·8)	3411·6 (3356·3 to 3464·0)	803·5 (786·9 to 821·0)	115·0 (111·6 to 118·4)	0·0 (0·0 to 0·0)	4409·5 (4351·2 to 4465·3)	3474·2 (3417·9 to 3527·5)	818·2 (801·3 to 836·1)	117·1 (113·7 to 120·6)	0·0 (0·0 to 0·0)
Finland	2011	4454·3 (4391·0 to 4517·4)	3526·0 (3467·2 to 3583·2)	808·7 (793·0 to 825·3)	119·6 (116·0 to 123·3)	0·0 (0·0 to 0·0)	4536·1 (4471·6 to 4600·3)	3590·8 (3530·9 to 3649·0)	823·5 (807·5 to 840·4)	121·8 (118·2 to 125·6)	0·0 (0·0 to 0·0)
Finland	2012	4549·1 (4490·8 to 4605·2)	3621·4 (3566·3 to 3674·5)	803·6 (787·6 to 819·6)	124·1 (120·7 to 127·7)	0·0 (0·0 to 0·0)	4632·6 (4573·2 to 4689·7)	3687·9 (3631·8 to 3742·0)	818·3 (802·0 to 834·6)	126·4 (122·9 to 130·0)	0·0 (0·0 to 0·0)
Finland	2013	4587·7 (4525·8 to 4642·6)	3653·8 (3597·0 to 3706·9)	807·6 (790·8 to 824·1)	126·3 (123·1 to 129·6)	0·0 (0·0 to 0·0)	4671·9 (4608·9 to 4727·8)	3720·9 (3663·0 to 3774·9)	822·4 (805·4 to 839·2)	128·6 (125·3 to 132·0)	0·0 (0·0 to 0·0)
Finland	2014	4553·3 (4490·9 to 4615·1)	3613·1 (3554·4 to 3668·5)	813·1 (797·6 to 829·8)	127·1 (123·8 to 130·7)	0·0 (0·0 to 0·0)	4636·8 (4573·3 to 4699·8)	3679·4 (3619·6 to 3735·9)	828·0 (812·2 to 845·1)	129·4 (126·0 to 133·1)	0·0 (0·0 to 0·0)
Finland	2015	4493·0 (4430·3 to 4552·6)	3530·7 (3472·0 to 3589·6)	835·5 (818·6 to 853·0)	126·7 (123·4 to 130·5)	0·0 (0·0 to 0·0)	4575·4 (4511·6 to 4636·2)	3595·5 (3535·7 to 3655·5)	850·8 (833·6 to 868·6)	129·1 (125·7 to 132·9)	0·0 (0·0 to 0·0)
Finland	2016	4482·2 (4425·3 to 4540·3)	3495·6 (3439·7 to 3551·4)	857·6 (841·5 to 875·1)	129·0 (125·6 to 132·3)	0·0 (0·0 to 0·0)	4564·5 (4506·5 to 4623·7)	3559·7 (3502·8 to 3616·6)	873·4 (856·9 to 891·1)	131·4 (127·9 to 134·8)	0·0 (0·0 to 0·0)
Finland	2017	4483·7 (4427·6 to 4542·1)	3497·6 (3443·5 to 3551·8)	854·2 (838·0 to 870·8)	132·0 (128·4 to 135·4)	0·0 (0·0 to 0·0)	4566·0 (4508·8 to 4625·5)	3561·8 (3506·7 to 3617·0)	869·9 (853·4 to 886·8)	134·4 (130·8 to 137·9)	0·0 (0·0 to 0·0)
Finland	2018	4514·3 (4438·4 to 4590·4)	3543·5 (3471·0 to 3613·9)	835·2 (813·4 to 856·0)	135·7 (131·4 to 139·8)	0·0 (0·0 to 0·0)	4597·2 (4519·8 to 4674·7)	3608·5 (3534·7 to 3680·2)	850·5 (828·4 to 871·7)	138·2 (133·8 to 142·4)	0·0 (0·0 to 0·0)
Finland	2019	4595·2 (4504·1 to 4690·1)	3612·0 (3524·2 to 3703·4)	842·8 (820·8 to 864·0)	140·4 (135·9 to 144·8)	0·0 (0·0 to 0·0)	4679·5 (4586·7 to 4776·2)	3678·3 (3588·9 to 3771·4)	858·3 (835·9 to 879·8)	143·0 (138·4 to 147·5)	0·0 (0·0 to 0·0)
Finland	2020	4802·3 (4711·2 to 4897·6)	3871·7 (3784·0 to 3962·6)	794·9 (774·7 to 814·7)	135·8 (131·1 to 140·5)	0·0 (0·0 to 0·0)	4890·4 (4797·6 to 4987·5)	3942·7 (3853·4 to 4035·3)	809·5 (788·9 to 829·7)	138·3 (133·5 to 143·1)	0·0 (0·0 to 0·0)
Finland	2021	4553·0 (4442·7 to 4661·7)	3666·0 (3558·4 to 3769·7)	754·9 (735·9 to 773·9)	132·2 (127·0 to 137·4)	0·0 (0·0 to 0·0)	4636·6 (4524·2 to 4747·3)	3733·3 (3623·7 to 3838·9)	768·7 (749·4 to 788·1)	134·6 (129·4 to 139·9)	0·0 (0·0 to 0·0)
Finland	2022	4225·1 (4095·5 to 4344·6)	3333·4 (3206·6 to 3450·1)	756·0 (737·2 to 775·2)	135·6 (129·5 to 141·9)	0·0 (0·0 to 0·0)	4302·6 (4170·7 to 4424·3)	3394·6 (3265·4 to 3513·4)	769·9 (750·7 to 789·4)	138·1 (131·9 to 144·5)	0·0 (0·0 to 0·0)
Finland	2023	4269·4 (4125·8 to 4400·8)	3372·5 (3236·8 to 3499·6)	757·6 (738·6 to 776·7)	139·2 (132·1 to 146·7)	0·0 (0·0 to 0·0)	4347·7 (4201·5 to 4481·6)	3434·4 (3296·2 to 3563·9)	771·5 (752·2 to 790·9)	141·8 (134·5 to 149·4)	0·0 (0·0 to 0·0)

Finland	2024	4321·0 (4173·8 to 4453·9)	3418·1 (3276·7 to 3547·8)	759·9 (741·1 to 778·5)	143·0 (134·6 to 151·9)	0·0 (0·0 to 0·0)	4400·3 (4250·4 to 4535·6)	3480·8 (3336·8 to 3612·9)	773·8 (754·7 to 792·8)	145·6 (137·1 to 154·6)	0·0 (0·0 to 0·0)
Finland	2025	4371·5 (4220·1 to 4510·5)	3462·6 (3313·6 to 3600·5)	762·1 (743·1 to 780·6)	146·8 (137·2 to 157·4)	0·0 (0·0 to 0·0)	4451·7 (4297·6 to 4593·3)	3526·1 (3374·4 to 3666·6)	776·1 (756·8 to 795·0)	149·5 (139·7 to 160·2)	0·0 (0·0 to 0·0)
Finland	2026	4416·5 (4261·2 to 4563·3)	3500·7 (3349·7 to 3643·6)	765·0 (746·1 to 784·2)	150·8 (140·1 to 162·9)	0·0 (0·0 to 0·0)	4497·6 (4339·4 to 4647·0)	3565·0 (3411·2 to 3710·4)	779·1 (759·8 to 798·6)	153·6 (142·6 to 165·9)	0·0 (0·0 to 0·0)
Finland	2027	4459·6 (4299·8 to 4613·2)	3536·5 (3382·8 to 3687·5)	768·1 (748·5 to 787·5)	154·9 (142·9 to 168·9)	0·0 (0·0 to 0·0)	4541·4 (4378·7 to 4697·9)	3601·4 (3444·9 to 3755·1)	782·2 (762·2 to 802·0)	157·8 (145·6 to 172·0)	0·0 (0·0 to 0·0)
Finland	2028	4506·7 (4335·1 to 4671·7)	3575·6 (3408·0 to 3738·6)	771·7 (751·7 to 791·3)	159·3 (145·7 to 175·0)	0·0 (0·0 to 0·0)	4589·4 (4414·6 to 4757·5)	3641·3 (3470·6 to 3807·2)	785·9 (765·5 to 805·8)	162·2 (148·4 to 178·2)	0·0 (0·0 to 0·0)
Finland	2029	4554·7 (4369·2 to 4728·1)	3615·4 (3439·7 to 3786·2)	775·5 (755·5 to 794·8)	163·8 (148·5 to 181·2)	0·0 (0·0 to 0·0)	4638·3 (4449·4 to 4814·9)	3681·7 (3502·8 to 3855·6)	789·7 (769·3 to 809·4)	166·8 (151·3 to 184·6)	0·0 (0·0 to 0·0)
Finland	2030	4601·6 (4408·3 to 4791·5)	3654·0 (3468·9 to 3840·7)	779·1 (758·5 to 798·4)	168·4 (151·5 to 187·5)	0·0 (0·0 to 0·0)	4686·0 (4489·2 to 4879·4)	3721·1 (3532·5 to 3911·2)	793·4 (772·4 to 813·1)	171·5 (154·2 to 191·0)	0·0 (0·0 to 0·0)
Finland	2031	4641·7 (4445·2 to 4836·2)	3687·1 (3494·5 to 3876·2)	781·7 (760·9 to 800·9)	172·8 (154·2 to 193·8)	0·0 (0·0 to 0·0)	4726·9 (4526·8 to 4925·0)	3754·8 (3558·6 to 3947·3)	796·1 (774·8 to 815·6)	176·0 (157·0 to 197·4)	0·0 (0·0 to 0·0)
Finland	2032	4682·4 (4478·6 to 4883·8)	3720·0 (3521·3 to 3914·6)	785·0 (764·2 to 804·3)	177·4 (156·4 to 200·3)	0·0 (0·0 to 0·0)	4768·4 (4560·8 to 4973·4)	3788·3 (3585·9 to 3986·5)	799·4 (778·2 to 819·1)	180·6 (159·3 to 203·9)	0·0 (0·0 to 0·0)
Finland	2033	4730·0 (4514·9 to 4936·1)	3757·9 (3547·8 to 3969·4)	789·8 (768·7 to 809·7)	182·3 (159·1 to 207·2)	0·0 (0·0 to 0·0)	4816·8 (4597·8 to 5026·7)	3826·8 (3613·0 to 4042·2)	804·3 (782·8 to 824·5)	185·7 (162·0 to 211·0)	0·0 (0·0 to 0·0)
Finland	2034	4775·8 (4560·8 to 4995·0)	3793·9 (3589·5 to 4010·2)	794·6 (773·0 to 814·9)	187·3 (162·1 to 214·2)	0·0 (0·0 to 0·0)	4863·4 (4644·5 to 5086·6)	3863·5 (3655·3 to 4083·8)	809·1 (787·2 to 829·9)	190·8 (165·1 to 218·2)	0·0 (0·0 to 0·0)
Finland	2035	4822·6 (4595·5 to 5039·8)	3830·3 (3609·3 to 4052·8)	799·8 (778·0 to 820·3)	192·5 (165·1 to 221·8)	0·0 (0·0 to 0·0)	4911·1 (4679·9 to 5132·2)	3900·6 (3675·5 to 4127·2)	814·5 (792·2 to 835·3)	196·1 (168·2 to 225·8)	0·0 (0·0 to 0·0)
Finland	2036	4873·8 (4630·0 to 5103·8)	3870·5 (3640·0 to 4103·4)	805·4 (782·7 to 826·2)	197·9 (168·3 to 229·8)	0·0 (0·0 to 0·0)	4963·3 (4714·9 to 5197·4)	3941·5 (3706·8 to 4178·7)	820·2 (797·0 to 841·4)	201·5 (171·4 to 234·0)	0·0 (0·0 to 0·0)
Finland	2037	4920·0 (4661·1 to 5167·3)	3906·1 (3663·4 to 4154·1)	810·6 (787·5 to 831·8)	203·4 (171·9 to 238·4)	0·0 (0·0 to 0·0)	5010·3 (4746·7 to 5262·1)	3977·7 (3730·7 to 4230·3)	825·5 (801·9 to 847·0)	207·1 (175·1 to 242·7)	0·0 (0·0 to 0·0)

Finland	2038	4969·3 (4706·6 to 5228·6)	3944·2 (3691·6 to 4197·8)	816·1 (792·8 to 837·7)	209·0 (174·9 to 247·4)	0·0 (0·0 to 0·0)	5060·5 (4792·9 to 5324·5)	4016·6 (3759·4 to 4274·9)	831·0 (807·3 to 853·1)	212·9 (178·1 to 252·0)	0·0 (0·0 to 0·0)
Finland	2039	5022·4 (4750·9 to 5287·4)	3985·3 (3724·9 to 4247·1)	822·1 (798·6 to 844·1)	215·0 (178·1 to 257·2)	0·0 (0·0 to 0·0)	5114·6 (4838·1 to 5384·4)	4058·5 (3793·3 to 4325·0)	837·2 (813·2 to 859·6)	218·9 (181·3 to 262·0)	0·0 (0·0 to 0·0)
Finland	2040	5076·9 (4795·7 to 5355·0)	4027·4 (3756·3 to 4300·5)	828·3 (804·2 to 850·7)	221·2 (181·6 to 266·9)	0·0 (0·0 to 0·0)	5170·0 (4883·7 to 5453·3)	4101·3 (3825·3 to 4379·4)	843·5 (819·0 to 866·3)	225·2 (184·9 to 271·8)	0·0 (0·0 to 0·0)
Finland	2041	5130·8 (4831·9 to 5417·2)	4069·1 (3779·7 to 4356·6)	834·2 (809·7 to 857·1)	227·5 (185·1 to 276·6)	0·0 (0·0 to 0·0)	5225·0 (4920·5 to 5516·6)	4143·8 (3849·0 to 4436·6)	849·6 (824·5 to 872·8)	231·6 (188·5 to 281·7)	0·0 (0·0 to 0·0)
Finland	2042	5186·0 (4888·0 to 5483·2)	4111·6 (3825·4 to 4408·0)	840·4 (815·5 to 863·6)	234·0 (188·4 to 287·2)	0·0 (0·0 to 0·0)	5281·2 (4977·7 to 5583·8)	4187·1 (3895·6 to 4488·9)	855·8 (830·5 to 879·5)	238·3 (191·9 to 292·4)	0·0 (0·0 to 0·0)
Finland	2043	5242·8 (4928·4 to 5542·7)	4155·5 (3852·2 to 4447·7)	846·5 (821·4 to 870·0)	240·8 (192·3 to 298·2)	0·0 (0·0 to 0·0)	5339·0 (5018·8 to 5644·4)	4231·7 (3922·9 to 4529·3)	862·0 (836·5 to 886·0)	245·2 (195·8 to 303·7)	0·0 (0·0 to 0·0)
Finland	2044	5298·8 (4972·0 to 5619·7)	4198·8 (3882·5 to 4517·6)	852·3 (827·0 to 876·6)	247·7 (196·0 to 309·8)	0·0 (0·0 to 0·0)	5396·0 (5063·2 to 5722·8)	4275·8 (3953·8 to 4600·5)	867·9 (842·1 to 892·6)	252·2 (199·6 to 315·5)	0·0 (0·0 to 0·0)
Finland	2045	5353·5 (5011·8 to 5684·6)	4240·7 (3911·5 to 4565·3)	858·1 (832·5 to 882·8)	254·8 (199·9 to 321·9)	0·0 (0·0 to 0·0)	5451·8 (5103·8 to 5789·0)	4318·5 (3983·2 to 4649·0)	873·8 (847·7 to 899·0)	259·5 (203·6 to 327·8)	0·0 (0·0 to 0·0)
Finland	2046	5407·9 (5060·4 to 5751·6)	4282·3 (3948·2 to 4624·1)	863·5 (837·0 to 888·8)	262·0 (203·7 to 334·3)	0·0 (0·0 to 0·0)	5507·1 (5153·2 to 5857·2)	4360·9 (4020·7 to 4709·0)	879·4 (852·3 to 905·1)	266·8 (207·4 to 340·4)	0·0 (0·0 to 0·0)
Finland	2047	5465·8 (5103·7 to 5812·5)	4327·0 (3985·7 to 4673·9)	869·2 (841·9 to 895·0)	269·5 (207·5 to 347·2)	0·0 (0·0 to 0·0)	5566·1 (5197·3 to 5919·2)	4406·5 (4058·9 to 4759·6)	885·2 (857·4 to 911·4)	274·5 (211·3 to 353·6)	0·0 (0·0 to 0·0)
Finland	2048	5522·2 (5143·2 to 5871·1)	4370·0 (4020·9 to 4712·6)	874·9 (847·4 to 901·2)	277·3 (211·3 to 360·6)	0·0 (0·0 to 0·0)	5623·6 (5237·6 to 5978·8)	4450·2 (4094·7 to 4799·0)	891·0 (862·9 to 917·8)	282·3 (215·1 to 367·2)	0·0 (0·0 to 0·0)
Finland	2049	5579·8 (5200·2 to 5947·0)	4414·0 (4060·0 to 4768·0)	880·5 (852·1 to 907·7)	285·2 (215·1 to 374·5)	0·0 (0·0 to 0·0)	5682·2 (5295·6 to 6056·2)	4495·1 (4134·5 to 4855·5)	896·7 (867·7 to 924·3)	290·4 (219·1 to 381·4)	0·0 (0·0 to 0·0)
Finland	2050	5641·8 (5261·3 to 6023·0)	4461·9 (4090·4 to 4826·4)	886·4 (857·5 to 914·3)	293·4 (219·1 to 388·8)	0·0 (0·0 to 0·0)	5745·3 (5357·8 to 6133·6)	4543·8 (4165·4 to 4915·0)	902·7 (873·3 to 931·0)	298·8 (223·1 to 396·0)	0·0 (0·0 to 0·0)
Fiji*	1995	105·7 (94·1 to 118·5)	81·1 (70·3 to 92·8)	14·3 (11·1 to 17·8)	9·5 (6·4 to 13·4)	0·9 (0·9 to 0·9)	270·1 (240·6 to 302·8)	207·2 (179·8 to 237·3)	36·5 (28·3 to 45·4)	24·2 (16·4 to 34·1)	2·3 (2·3 to 2·3)

Fiji*	1996	114·1 (102·2 to 127·1)	89·5 (79·0 to 101·6)	14·8 (11·6 to 18·5)	9·6 (6·5 to 13·8)	0·1 (0·1 to 0·1)	291·6 (261·2 to 324·8)	228·9 (201·9 to 259·6)	37·9 (29·8 to 47·4)	24·6 (16·6 to 35·2)	0·2 (0·2 to 0·2)
Fiji*	1997	118·4 (107·5 to 130·5)	93·2 (82·8 to 104·7)	14·9 (11·9 to 18·5)	9·1 (6·2 to 13·0)	1·1 (1·1 to 1·1)	302·7 (274·7 to 333·7)	238·4 (211·6 to 267·6)	38·2 (30·5 to 47·4)	23·4 (15·9 to 33·3)	2·8 (2·8 to 2·8)
Fiji*	1998	126·7 (116·8 to 136·6)	90·8 (81·6 to 100·2)	14·6 (11·7 to 18·1)	7·6 (5·3 to 10·8)	13·6 (13·6 to 13·6)	323·8 (298·4 to 349·2)	232·1 (208·5 to 256·2)	37·4 (29·9 to 46·3)	19·5 (13·5 to 27·5)	34·8 (34·8 to 34·8)
Fiji*	1999	128·5 (119·9 to 137·1)	91·1 (83·5 to 99·0)	14·9 (11·8 to 18·6)	5·2 (3·6 to 7·4)	17·3 (17·3 to 17·3)	328·4 (306·5 to 350·4)	232·8 (213·3 to 253·0)	38·0 (30·2 to 47·6)	13·3 (9·2 to 18·9)	44·2 (44·2 to 44·2)
Fiji*	2000	130·6 (123·2 to 138·1)	93·8 (87·3 to 100·8)	15·1 (12·1 to 19·0)	5·8 (4·0 to 8·4)	15·9 (15·9 to 15·9)	333·8 (315·0 to 352·9)	239·9 (223·3 to 257·6)	38·5 (31·0 to 48·5)	14·9 (10·2 to 21·4)	40·5 (40·5 to 40·5)
Fiji*	2001	120·9 (114·2 to 127·8)	93·2 (87·8 to 99·1)	15·5 (12·6 to 19·2)	6·0 (4·1 to 8·6)	6·2 (6·2 to 6·2)	309·1 (292·0 to 326·6)	238·3 (224·3 to 253·4)	39·5 (32·1 to 49·0)	15·4 (10·5 to 21·9)	15·9 (15·9 to 15·9)
Fiji*	2002	126·0 (119·3 to 133·0)	95·8 (90·4 to 101·9)	15·9 (13·1 to 19·6)	6·5 (4·4 to 9·1)	7·8 (7·8 to 7·8)	322·1 (305·0 to 339·9)	244·9 (231·0 to 260·5)	40·8 (33·5 to 50·1)	16·6 (11·3 to 23·3)	19·9 (19·9 to 19·9)
Fiji*	2003	137·5 (131·3 to 144·2)	94·7 (89·5 to 100·1)	16·5 (13·6 to 19·9)	6·6 (4·6 to 9·3)	19·7 (19·7 to 19·7)	351·6 (335·5 to 368·7)	242·0 (228·7 to 255·9)	42·1 (34·8 to 50·7)	17·0 (11·6 to 23·6)	50·4 (50·4 to 50·4)
Fiji*	2004	133·7 (127·3 to 140·9)	97·3 (92·2 to 103·0)	17·8 (15·0 to 21·2)	7·4 (5·1 to 10·1)	11·2 (11·2 to 11·2)	341·8 (325·3 to 360·2)	248·8 (235·6 to 263·3)	45·4 (38·2 to 54·3)	18·9 (13·1 to 25·9)	28·6 (28·6 to 28·6)
Fiji*	2005	133·1 (126·5 to 140·4)	99·8 (94·3 to 105·7)	19·0 (16·3 to 22·4)	8·0 (5·5 to 11·0)	6·4 (6·4 to 6·4)	340·3 (323·4 to 359·0)	255·1 (241·1 to 270·2)	48·5 (41·6 to 57·1)	20·4 (14·1 to 28·1)	16·4 (16·4 to 16·4)
Fiji*	2006	148·5 (141·4 to 156·1)	107·1 (101·1 to 113·4)	21·7 (18·9 to 24·8)	9·9 (6·8 to 13·7)	9·8 (9·8 to 9·8)	379·6 (361·4 to 398·9)	273·8 (258·5 to 289·8)	55·5 (48·4 to 63·4)	25·3 (17·5 to 35·1)	25·1 (25·1 to 25·1)
Fiji*	2007	148·9 (141·6 to 156·2)	101·3 (95·6 to 107·1)	24·0 (21·2 to 27·0)	10·9 (7·6 to 15·0)	12·7 (12·7 to 12·7)	380·6 (362·0 to 399·3)	259·0 (244·2 to 273·8)	61·3 (54·2 to 69·1)	27·8 (19·3 to 38·3)	32·5 (32·5 to 32·5)
Fiji*	2008	141·6 (134·6 to 148·6)	94·4 (88·9 to 99·7)	26·6 (24·0 to 29·4)	10·7 (7·5 to 15·0)	9·9 (9·9 to 9·9)	361·9 (344·2 to 379·8)	241·2 (227·3 to 254·9)	68·0 (61·4 to 75·1)	27·5 (19·2 to 38·2)	25·2 (25·2 to 25·2)
Fiji*	2009	148·0 (140·7 to 155·4)	95·3 (90·0 to 101·1)	31·7 (29·1 to 34·5)	11·1 (7·8 to 15·5)	9·8 (9·8 to 9·8)	378·2 (359·6 to 397·1)	243·7 (230·0 to 258·3)	81·1 (74·3 to 88·1)	28·3 (20·0 to 39·5)	25·2 (25·2 to 25·2)

Fiji*	2010	151·1 (144·3 to 158·4)	89·2 (84·3 to 94·3)	33·4 (30·6 to 36·1)	12·0 (8·5 to 16·4)	16·5 (16·5 to 16·5)	386·1 (368·9 to 404·9)	228·1 (215·4 to 241·1)	85·4 (78·3 to 92·4)	30·6 (21·8 to 42·0)	42·1 (42·1 to 42·1)
Fiji*	2011	151·6 (144·3 to 159·7)	87·5 (82·1 to 92·6)	34·7 (32·0 to 37·7)	13·7 (9·9 to 18·6)	15·6 (15·6 to 15·6)	387·5 (369·0 to 408·3)	223·6 (209·9 to 236·7)	88·8 (81·8 to 96·3)	35·1 (25·4 to 47·5)	40·0 (40·0 to 40·0)
Fiji*	2012	144·1 (136·7 to 152·1)	85·2 (80·0 to 90·4)	34·6 (31·8 to 37·3)	15·7 (11·7 to 21·0)	8·7 (8·7 to 8·7)	368·4 (349·3 to 388·8)	217·7 (204·4 to 231·1)	88·4 (81·2 to 95·4)	40·1 (29·8 to 53·6)	22·2 (22·2 to 22·2)
Fiji*	2013	149·2 (142·0 to 157·1)	85·9 (81·0 to 91·3)	33·5 (30·8 to 36·3)	19·0 (14·4 to 24·6)	10·8 (10·8 to 10·8)	381·5 (363·0 to 401·6)	219·7 (207·1 to 233·4)	85·6 (78·8 to 92·9)	48·5 (36·7 to 62·9)	27·7 (27·7 to 27·7)
Fiji*	2014	166·4 (158·6 to 174·6)	99·0 (93·6 to 104·7)	33·8 (30·9 to 36·7)	22·5 (17·6 to 28·2)	11·0 (11·0 to 11·0)	425·3 (405·5 to 446·4)	253·1 (239·3 to 267·7)	86·5 (79·0 to 93·9)	57·5 (44·9 to 72·2)	28·2 (28·2 to 28·2)
Fiji*	2015	170·3 (162·9 to 178·4)	105·7 (100·3 to 111·6)	33·4 (30·2 to 36·5)	23·5 (19·3 to 28·3)	7·6 (7·6 to 7·6)	435·3 (416·5 to 456·0)	270·3 (256·3 to 285·3)	85·5 (77·2 to 93·4)	60·0 (49·3 to 72·4)	19·5 (19·5 to 19·5)
Fiji*	2016	167·3 (160·2 to 174·8)	104·4 (98·7 to 109·8)	31·2 (27·9 to 34·7)	23·5 (20·5 to 27·0)	8·2 (8·2 to 8·2)	427·7 (409·5 to 446·9)	266·9 (252·4 to 280·8)	79·8 (71·4 to 88·7)	60·1 (52·3 to 68·9)	20·9 (20·9 to 20·9)
Fiji*	2017	177·2 (168·5 to 186·2)	115·1 (107·5 to 123·0)	29·4 (25·8 to 33·0)	27·0 (24·1 to 30·2)	5·7 (5·7 to 5·7)	453·0 (430·7 to 475·8)	294·3 (274·8 to 314·3)	75·1 (65·8 to 84·3)	69·1 (61·7 to 77·2)	14·6 (14·6 to 14·6)
Fiji*	2018	200·7 (188·5 to 213·5)	128·3 (117·3 to 140·2)	29·0 (24·7 to 33·2)	29·9 (25·9 to 34·1)	13·5 (13·5 to 13·5)	513·0 (481·7 to 545·7)	327·9 (299·7 to 358·3)	74·2 (63·1 to 84·9)	76·3 (66·1 to 87·3)	34·6 (34·6 to 34·6)
Fiji*	2019	194·9 (182·1 to 207·6)	120·9 (109·5 to 133·1)	28·6 (24·3 to 32·7)	30·3 (26·2 to 34·7)	15·0 (14·9 to 15·4)	498·1 (465·6 to 530·7)	309·1 (279·9 to 340·3)	73·1 (62·2 to 83·6)	77·5 (67·0 to 88·7)	38·4 (38·0 to 39·4)
Fiji*	2020	207·3 (193·1 to 221·0)	130·4 (117·5 to 143·9)	28·1 (23·9 to 32·1)	30·6 (26·4 to 35·1)	18·2 (17·6 to 19·1)	530·0 (493·6 to 564·8)	333·4 (300·3 to 367·9)	71·8 (61·1 to 82·2)	78·2 (67·5 to 89·7)	46·5 (44·9 to 48·8)
Fiji*	2021	204·8 (190·5 to 218·4)	129·3 (116·5 to 142·7)	27·9 (23·7 to 31·9)	31·1 (26·8 to 35·7)	16·5 (15·9 to 17·5)	523·5 (487·0 to 558·2)	330·6 (297·8 to 364·7)	71·2 (60·5 to 81·4)	79·5 (68·4 to 91·2)	42·2 (40·7 to 44·7)
Fiji*	2022	196·5 (182·6 to 211·3)	123·3 (110·6 to 137·6)	28·0 (23·8 to 32·0)	32·1 (27·6 to 36·8)	13·1 (12·3 to 14·1)	502·3 (466·6 to 540·2)	315·3 (282·7 to 351·6)	71·6 (60·8 to 81·8)	82·0 (70·6 to 94·0)	33·6 (31·4 to 36·1)
Fiji*	2023	200·8 (185·7 to 217·4)	125·8 (112·4 to 141·4)	28·1 (23·9 to 32·1)	33·1 (28·4 to 38·1)	13·8 (12·8 to 14·8)	513·3 (474·7 to 555·6)	321·6 (287·4 to 361·4)	71·9 (61·1 to 82·2)	84·6 (72·6 to 97·3)	35·2 (32·8 to 37·9)

Fiji*	2024	204·2 (188·7 to 221·2)	127·3 (113·2 to 143·4)	28·3 (24·0 to 32·3)	34·2 (29·2 to 39·4)	14·4 (13·5 to 15·6)	521·9 (482·3 to 565·5)	325·3 (289·4 to 366·5)	72·3 (61·4 to 82·6)	87·4 (74·8 to 100·8)	36·8 (34·4 to 39·8)
Fiji*	2025	206·3 (190·1 to 223·6)	127·5 (113·3 to 143·5)	28·5 (24·2 to 32·5)	35·3 (30·3 to 40·8)	15·1 (14·0 to 16·2)	527·3 (485·8 to 571·5)	325·8 (289·6 to 366·7)	72·7 (61·8 to 83·1)	90·3 (77·4 to 104·2)	38·5 (35·8 to 41·5)
Fiji*	2026	208·6 (192·0 to 226·0)	127·8 (113·5 to 144·0)	28·6 (24·4 to 32·7)	36·5 (31·3 to 42·1)	15·7 (14·5 to 16·9)	533·1 (490·8 to 577·7)	326·6 (290·0 to 368·1)	73·2 (62·3 to 83·5)	93·3 (79·9 to 107·6)	40·1 (37·1 to 43·2)
Fiji*	2027	210·9 (194·1 to 228·6)	128·2 (113·6 to 144·4)	28·8 (24·5 to 32·9)	37·7 (32·3 to 43·6)	16·2 (15·0 to 17·6)	539·1 (496·1 to 584·5)	327·6 (290·3 to 369·1)	73·7 (62·7 to 84·1)	96·3 (82·5 to 111·5)	41·5 (38·4 to 44·9)
Fiji*	2028	213·4 (196·5 to 231·2)	128·7 (113·9 to 144·9)	29·0 (24·7 to 33·2)	38·9 (33·2 to 45·3)	16·8 (15·4 to 18·3)	545·6 (502·2 to 591·1)	328·9 (291·0 to 370·3)	74·2 (63·2 to 84·7)	99·6 (84·8 to 115·7)	42·9 (39·4 to 46·7)
Fiji*	2029	216·0 (198·5 to 234·5)	129·2 (114·2 to 146·1)	29·3 (24·9 to 33·4)	40·2 (34·2 to 47·0)	17·4 (15·9 to 19·0)	552·2 (507·4 to 599·4)	330·2 (291·8 to 373·4)	74·8 (63·7 to 85·4)	102·9 (87·4 to 120·1)	44·3 (40·6 to 48·5)
Fiji*	2030	219·0 (200·8 to 237·2)	130·0 (114·5 to 147·1)	29·5 (25·2 to 33·8)	41·7 (35·3 to 48·9)	17·7 (16·1 to 19·5)	559·7 (513·4 to 606·3)	332·3 (292·6 to 376·1)	75·5 (64·3 to 86·4)	106·5 (90·1 to 124·9)	45·3 (41·2 to 49·9)
Fiji*	2031	222·0 (203·7 to 240·7)	130·9 (115·0 to 148·3)	29·8 (25·4 to 34·1)	43·1 (36·3 to 50·8)	18·1 (16·4 to 20·1)	567·4 (520·8 to 615·3)	334·6 (293·8 to 379·0)	76·3 (65·0 to 87·3)	110·2 (92·7 to 129·9)	46·4 (42·0 to 51·4)
Fiji*	2032	225·1 (205·6 to 245·0)	131·8 (115·5 to 149·6)	30·1 (25·7 to 34·5)	44·6 (37·4 to 52·9)	18·5 (16·7 to 20·5)	575·4 (525·6 to 626·3)	337·0 (295·1 to 382·4)	77·0 (65·6 to 88·1)	113·9 (95·7 to 135·1)	47·4 (42·7 to 52·5)
Fiji*	2033	228·3 (208·7 to 248·5)	132·9 (115·7 to 151·5)	30·4 (25·9 to 34·8)	46·0 (38·6 to 54·8)	19·0 (17·0 to 21·0)	583·6 (533·5 to 635·3)	339·7 (295·7 to 387·4)	77·8 (66·3 to 88·9)	117·7 (98·6 to 140·2)	48·5 (43·5 to 53·8)
Fiji*	2034	231·8 (211·4 to 252·6)	134·1 (116·4 to 153·8)	30·8 (26·2 to 35·2)	47·6 (39·6 to 56·7)	19·3 (17·4 to 21·6)	592·6 (540·2 to 645·7)	342·9 (297·5 to 393·0)	78·6 (67·0 to 90·0)	121·6 (101·3 to 144·9)	49·5 (44·5 to 55·2)
Fiji*	2035	235·3 (214·2 to 257·7)	135·3 (116·9 to 155·7)	31·1 (26·5 to 35·5)	49·1 (40·7 to 58·7)	19·8 (17·7 to 22·3)	601·5 (547·4 to 658·8)	346·0 (298·8 to 398·0)	79·4 (67·6 to 90·7)	125·5 (104·1 to 150·2)	50·6 (45·3 to 57·0)
Fiji*	2036	238·9 (216·9 to 261·8)	136·6 (117·6 to 157·7)	31·4 (26·7 to 35·8)	50·6 (41·9 to 60·9)	20·3 (18·0 to 23·0)	610·7 (554·5 to 669·1)	349·2 (300·7 to 403·2)	80·2 (68·3 to 91·6)	129·4 (107·1 to 155·8)	51·8 (46·1 to 58·7)
Fiji*	2037	242·5 (219·5 to 266·0)	137·8 (118·6 to 159·8)	31·6 (26·9 to 36·2)	52·2 (42·9 to 62·8)	20·8 (18·5 to 23·7)	619·8 (561·2 to 680·0)	352·3 (303·1 to 408·6)	80·9 (68·9 to 92·4)	133·4 (109·7 to 160·5)	53·3 (47·2 to 60·5)

Fiji*	2038	246·1 (222·8 to 270·2)	139·0 (119·4 to 161·7)	31·9 (27·2 to 36·5)	53·7 (44·2 to 64·8)	21·5 (19·0 to 24·5)	629·2 (569·4 to 690·7)	355·4 (305·3 to 413·4)	81·5 (69·4 to 93·2)	137·4 (113·0 to 165·6)	54·8 (48·6 to 62·7)
Fiji*	2039	250·0 (225·9 to 274·9)	140·4 (120·2 to 163·4)	32·2 (27·4 to 36·8)	55·3 (45·6 to 67·2)	22·1 (19·6 to 25·5)	639·0 (577·3 to 702·8)	358·8 (307·3 to 417·8)	82·2 (70·1 to 94·0)	141·5 (116·5 to 171·7)	56·6 (50·0 to 65·1)
Fiji*	2040	254·0 (228·3 to 280·3)	141·7 (121·0 to 165·5)	32·4 (27·7 to 37·1)	57·0 (46·8 to 69·6)	22·9 (20·1 to 26·4)	649·3 (583·6 to 716·4)	362·3 (309·4 to 423·1)	82·9 (70·7 to 94·8)	145·7 (119·7 to 177·8)	58·4 (51·3 to 67·4)
Fiji*	2041	258·2 (231·8 to 285·7)	143·2 (122·1 to 167·9)	32·7 (27·9 to 37·4)	58·7 (47·9 to 72·0)	23·6 (20·7 to 27·3)	660·0 (592·4 to 730·2)	366·0 (312·2 to 429·3)	83·6 (71·3 to 95·6)	150·1 (122·4 to 184·1)	60·3 (52·8 to 69·7)
Fiji*	2042	262·6 (236·2 to 290·8)	144·7 (123·2 to 170·0)	33·0 (28·1 to 37·7)	60·5 (49·0 to 74·5)	24·4 (21·1 to 28·5)	671·2 (603·9 to 743·4)	369·9 (314·8 to 434·5)	84·2 (71·7 to 96·5)	154·7 (125·3 to 190·4)	62·4 (53·9 to 72·9)
Fiji*	2043	267·0 (240·1 to 296·2)	146·1 (124·1 to 171·8)	33·2 (28·2 to 38·0)	62·4 (50·1 to 77·0)	25·4 (21·9 to 29·9)	682·5 (613·7 to 757·2)	373·4 (317·1 to 439·3)	84·8 (72·2 to 97·2)	159·4 (128·1 to 196·9)	64·9 (56·1 to 76·4)
Fiji*	2044	271·7 (243·7 to 301·9)	147·5 (124·9 to 173·7)	33·4 (28·4 to 38·3)	64·3 (51·1 to 79·6)	26·5 (22·8 to 31·5)	694·4 (622·9 to 771·8)	377·0 (319·3 to 443·9)	85·4 (72·6 to 97·9)	164·3 (130·7 to 203·3)	67·7 (58·2 to 80·6)
Fiji*	2045	276·4 (247·0 to 307·6)	148·9 (125·8 to 175·4)	33·6 (28·6 to 38·5)	66·3 (52·3 to 82·4)	27·6 (23·7 to 33·1)	706·5 (631·3 to 786·2)	380·6 (321·7 to 448·5)	85·9 (73·1 to 98·5)	169·4 (133·7 to 210·6)	70·6 (60·5 to 84·6)
Fiji*	2046	281·4 (251·5 to 313·1)	150·4 (127·1 to 177·4)	33·8 (28·8 to 38·8)	68·3 (53·4 to 85·3)	28·8 (24·5 to 34·7)	719·4 (643·0 to 800·3)	384·5 (325·0 to 453·4)	86·5 (73·5 to 99·1)	174·7 (136·4 to 218·1)	73·7 (62·5 to 88·6)
Fiji*	2047	286·6 (256·1 to 319·2)	152·1 (128·5 to 179·4)	34·1 (28·9 to 39·1)	70·4 (54·6 to 88·4)	30·1 (25·3 to 36·7)	732·7 (654·6 to 816·0)	388·7 (328·5 to 458·4)	87·1 (74·0 to 99·8)	180·0 (139·6 to 226·0)	76·9 (64·6 to 93·8)
Fiji*	2048	292·1 (260·4 to 325·0)	153·8 (129·8 to 181·5)	34·3 (29·1 to 39·4)	72·6 (55·6 to 91·7)	31·4 (26·3 to 38·4)	746·6 (665·5 to 830·7)	393·2 (331·9 to 463·9)	87·7 (74·5 to 100·6)	185·6 (142·2 to 234·4)	80·1 (67·3 to 98·2)
Fiji*	2049	297·7 (264·5 to 331·9)	155·6 (131·2 to 183·6)	34·5 (29·3 to 39·6)	74·8 (56·8 to 94·8)	32·8 (27·3 to 40·6)	761·0 (676·2 to 848·5)	397·8 (335·3 to 469·2)	88·2 (74·9 to 101·2)	191·1 (145·3 to 242·2)	83·8 (69·8 to 103·9)
Fiji*	2050	303·8 (269·5 to 340·4)	157·7 (132·8 to 186·0)	34·8 (29·5 to 39·9)	77·1 (58·2 to 98·4)	34·2 (28·0 to 42·7)	776·5 (688·9 to 870·0)	403·1 (339·5 to 475·4)	88·9 (75·5 to 101·9)	197·1 (148·7 to 251·5)	87·3 (71·5 to 109·2)
France	1995	3226·3 (3167·7 to 3281·3)	2360·9 (2321·7 to 2401·9)	236·7 (214·5 to 262·6)	628·7 (597·6 to 661·3)	0·0 (0·0 to 0·0)	3733·1 (3665·3 to 3796·8)	2731·8 (2686·5 to 2779·2)	273·9 (248·2 to 303·8)	727·5 (691·5 to 765·2)	0·0 (0·0 to 0·0)

France	1996	3251·2 (3204·8 to 3299·0)	2375·2 (2341·5 to 2411·1)	230·7 (212·4 to 251·8)	645·4 (621·1 to 669·8)	0·0 (0·0 to 0·0)	3762·0 (3708·3 to 3817·3)	2748·3 (2709·4 to 2789·9)	266·9 (245·8 to 291·3)	746·7 (718·7 to 775·0)	0·0 (0·0 to 0·0)
France	1997	3291·2 (3246·8 to 3337·0)	2404·0 (2371·5 to 2435·4)	229·0 (209·9 to 248·4)	658·2 (633·6 to 683·8)	0·0 (0·0 to 0·0)	3808·3 (3756·9 to 3861·2)	2781·7 (2744·1 to 2818·0)	265·0 (242·9 to 287·4)	761·6 (733·1 to 791·3)	0·0 (0·0 to 0·0)
France	1998	3353·7 (3307·9 to 3398·7)	2444·9 (2412·1 to 2477·1)	233·7 (215·8 to 252·7)	675·1 (650·6 to 700·1)	0·0 (0·0 to 0·0)	3880·6 (3827·6 to 3932·6)	2829·0 (2791·1 to 2866·2)	270·5 (249·7 to 292·4)	781·2 (752·8 to 810·1)	0·0 (0·0 to 0·0)
France	1999	3453·8 (3411·4 to 3500·2)	2515·2 (2484·1 to 2546·3)	244·5 (228·0 to 261·8)	694·1 (669·7 to 718·5)	0·0 (0·0 to 0·0)	3996·4 (3947·3 to 4050·1)	2910·4 (2874·4 to 2946·4)	282·9 (263·9 to 302·9)	803·1 (774·9 to 831·3)	0·0 (0·0 to 0·0)
France	2000	3548·0 (3512·9 to 3583·9)	2582·9 (2557·9 to 2609·3)	255·0 (239·8 to 270·8)	710·1 (692·1 to 730·0)	0·0 (0·0 to 0·0)	4105·4 (4064·8 to 4147·0)	2988·7 (2959·7 to 3019·2)	295·1 (277·4 to 313·3)	821·7 (800·9 to 844·7)	0·0 (0·0 to 0·0)
France	2001	3645·6 (3608·8 to 3680·9)	2656·0 (2630·8 to 2680·7)	263·5 (249·6 to 278·5)	726·1 (707·5 to 744·6)	0·0 (0·0 to 0·0)	4218·3 (4175·7 to 4259·2)	3073·3 (3044·1 to 3101·9)	304·9 (288·8 to 322·3)	840·2 (818·7 to 861·6)	0·0 (0·0 to 0·0)
France	2002	3770·5 (3733·2 to 3805·9)	2750·4 (2723·5 to 2778·1)	270·9 (256·3 to 285·1)	749·3 (731·2 to 767·8)	0·0 (0·0 to 0·0)	4362·9 (4319·7 to 4403·8)	3182·4 (3151·4 to 3214·5)	313·4 (296·6 to 329·9)	867·1 (846·0 to 888·5)	0·0 (0·0 to 0·0)
France	2003	3820·9 (3784·6 to 3857·5)	2778·2 (2750·2 to 2805·5)	278·2 (263·4 to 293·6)	764·5 (746·5 to 783·8)	0·0 (0·0 to 0·0)	4421·1 (4379·2 to 4463·5)	3214·6 (3182·3 to 3246·2)	321·9 (304·8 to 339·8)	884·6 (863·8 to 907·0)	0·0 (0·0 to 0·0)
France	2004	3920·3 (3885·6 to 3954·9)	2841·2 (2813·8 to 2867·0)	292·0 (277·4 to 307·6)	787·1 (768·4 to 805·4)	0·0 (0·0 to 0·0)	4536·1 (4496·1 to 4576·2)	3287·5 (3255·8 to 3317·4)	337·9 (321·0 to 356·0)	910·7 (889·2 to 931·9)	0·0 (0·0 to 0·0)
France	2005	4012·2 (3977·7 to 4048·5)	2895·6 (2869·7 to 2921·6)	318·5 (302·8 to 333·8)	798·2 (778·1 to 818·1)	0·0 (0·0 to 0·0)	4642·6 (4602·6 to 4684·6)	3350·5 (3320·6 to 3380·6)	368·5 (350·4 to 386·2)	923·6 (900·3 to 946·6)	0·0 (0·0 to 0·0)
France	2006	4095·5 (4059·9 to 4130·5)	2933·3 (2906·8 to 2959·5)	362·7 (346·7 to 379·3)	799·5 (780·1 to 818·9)	0·0 (0·0 to 0·0)	4738·9 (4697·7 to 4779·4)	3394·1 (3363·5 to 3424·4)	419·7 (401·1 to 438·9)	925·1 (902·7 to 947·6)	0·0 (0·0 to 0·0)
France	2007	4169·0 (4133·0 to 4205·6)	2964·6 (2938·0 to 2990·3)	397·8 (381·8 to 414·7)	806·7 (786·2 to 826·6)	0·0 (0·0 to 0·0)	4823·9 (4782·3 to 4866·3)	3430·3 (3399·6 to 3460·1)	460·3 (441·8 to 479·9)	933·4 (909·7 to 956·4)	0·0 (0·0 to 0·0)
France	2008	4237·6 (4201·3 to 4275·7)	2995·4 (2969·0 to 3022·3)	424·6 (408·1 to 442·5)	817·7 (798·2 to 837·5)	0·0 (0·0 to 0·0)	4903·4 (4861·3 to 4947·5)	3466·0 (3435·4 to 3497·1)	491·3 (472·2 to 512·0)	946·2 (923·6 to 969·1)	0·0 (0·0 to 0·0)
France	2009	4380·4 (4343·3 to 4418·7)	3095·4 (3068·4 to 3121·2)	445·0 (428·2 to 462·1)	840·0 (820·7 to 857·8)	0·0 (0·0 to 0·0)	5068·5 (5025·6 to 5112·9)	3581·7 (3550·5 to 3611·5)	514·9 (495·5 to 534·7)	972·0 (949·7 to 992·6)	0·0 (0·0 to 0·0)

France	2010	4437·2 (4402·3 to 4473·9)	3126·8 (3100·5 to 3152·0)	454·5 (437·0 to 472·4)	855·8 (837·9 to 874·8)	0·0 (0·0 to 0·0)	5134·2 (5093·9 to 5176·8)	3618·1 (3587·5 to 3647·2)	525·9 (505·7 to 546·6)	990·3 (969·6 to 1012·3)	0·0 (0·0 to 0·0)
France	2011	4484·5 (4447·4 to 4523·1)	3151·4 (3125·1 to 3179·4)	458·5 (442·1 to 475·5)	874·7 (856·0 to 892·7)	0·0 (0·0 to 0·0)	5189·1 (5146·0 to 5233·7)	3646·4 (3616·0 to 3678·9)	530·5 (511·6 to 550·2)	1012·1 (990·5 to 1032·9)	0·0 (0·0 to 0·0)
France	2012	4530·4 (4492·2 to 4567·0)	3183·6 (3156·4 to 3212·2)	458·2 (442·2 to 475·3)	888·6 (870·0 to 909·1)	0·0 (0·0 to 0·0)	5242·2 (5197·9 to 5284·5)	3683·8 (3652·3 to 3716·9)	530·2 (511·6 to 550·0)	1028·3 (1006·6 to 1051·9)	0·0 (0·0 to 0·0)
France	2013	4588·7 (4550·0 to 4626·2)	3228·4 (3202·2 to 3256·5)	456·8 (440·4 to 473·7)	903·4 (884·0 to 922·6)	0·0 (0·0 to 0·0)	5309·6 (5264·8 to 5353·0)	3735·6 (3705·2 to 3768·1)	528·6 (509·6 to 548·1)	1045·3 (1022·9 to 1067·6)	0·0 (0·0 to 0·0)
France	2014	4643·8 (4607·2 to 4679·5)	3288·9 (3264·7 to 3314·2)	455·1 (438·6 to 471·5)	899·8 (881·9 to 919·1)	0·0 (0·0 to 0·0)	5373·4 (5331·0 to 5414·7)	3805·6 (3777·6 to 3834·9)	526·6 (507·5 to 545·5)	1041·2 (1020·4 to 1063·5)	0·0 (0·0 to 0·0)
France	2015	4661·6 (4623·4 to 4696·4)	3361·6 (3335·4 to 3387·6)	451·9 (435·2 to 467·6)	848·1 (828·2 to 866·8)	0·0 (0·0 to 0·0)	5393·9 (5349·8 to 5434·3)	3889·7 (3859·4 to 3919·8)	522·9 (503·6 to 541·1)	981·3 (958·3 to 1003·0)	0·0 (0·0 to 0·0)
France	2016	4688·3 (4651·7 to 4726·3)	3411·7 (3384·5 to 3438·1)	449·2 (432·1 to 465·4)	827·4 (808·1 to 846·5)	0·0 (0·0 to 0·0)	5424·8 (5382·5 to 5468·8)	3947·7 (3916·2 to 3978·3)	519·8 (500·0 to 538·5)	957·3 (935·0 to 979·5)	0·0 (0·0 to 0·0)
France	2017	4737·5 (4698·4 to 4778·6)	3471·8 (3443·1 to 3499·4)	447·8 (431·0 to 464·9)	818·0 (799·0 to 838·3)	0·0 (0·0 to 0·0)	5481·8 (5436·5 to 5529·3)	4017·2 (3984·0 to 4049·2)	518·1 (498·7 to 537·9)	946·5 (924·5 to 970·0)	0·0 (0·0 to 0·0)
France	2018	4756·1 (4710·9 to 4799·0)	3487·9 (3458·0 to 3518·9)	443·5 (421·9 to 466·6)	824·6 (800·8 to 847·9)	0·0 (0·0 to 0·0)	5503·3 (5451·1 to 5552·9)	4035·9 (4001·3 to 4071·8)	513·2 (488·2 to 539·9)	954·2 (926·6 to 981·1)	0·0 (0·0 to 0·0)
France	2019	4843·6 (4791·9 to 4891·0)	3543·9 (3504·2 to 3579·6)	447·2 (425·4 to 470·4)	852·5 (826·4 to 878·1)	0·0 (0·0 to 0·0)	5604·5 (5544·7 to 5659·4)	4100·6 (4054·7 to 4141·9)	517·4 (492·2 to 544·3)	986·4 (956·3 to 1016·0)	0·0 (0·0 to 0·0)
France	2020	5003·2 (4951·6 to 5051·7)	3770·9 (3731·3 to 3807·1)	417·3 (396·8 to 439·2)	815·0 (790·3 to 839·1)	0·0 (0·0 to 0·0)	5789·2 (5729·5 to 5845·4)	4363·4 (4317·5 to 4405·1)	482·8 (459·1 to 508·2)	943·0 (914·5 to 971·0)	0·0 (0·0 to 0·0)
France	2021	4837·7 (4772·3 to 4894·5)	3629·4 (3576·0 to 3679·8)	402·9 (382·9 to 423·9)	805·4 (779·4 to 830·6)	0·0 (0·0 to 0·0)	5597·7 (5522·1 to 5663·4)	4199·6 (4137·8 to 4257·9)	466·2 (443·0 to 490·5)	931·9 (901·8 to 961·0)	0·0 (0·0 to 0·0)
France	2022	4497·8 (4406·8 to 4584·7)	3266·7 (3183·3 to 3346·7)	404·2 (384·3 to 425·1)	826·9 (797·6 to 855·8)	0·0 (0·0 to 0·0)	5204·4 (5099·1 to 5305·0)	3779·9 (3683·5 to 3872·5)	467·7 (444·7 to 491·9)	956·8 (922·9 to 990·2)	0·0 (0·0 to 0·0)
France	2023	4553·6 (4447·1 to 4659·1)	3299·8 (3196·5 to 3398·1)	405·4 (385·2 to 426·7)	848·4 (816·4 to 878·0)	0·0 (0·0 to 0·0)	5269·0 (5145·7 to 5391·0)	3818·2 (3698·7 to 3931·9)	469·1 (445·7 to 493·7)	981·7 (944·6 to 1015·9)	0·0 (0·0 to 0·0)

France	2024	4610·7 (4478·9 to 4735·7)	3333·7 (3208·7 to 3450·0)	406·7 (386·4 to 428·1)	870·2 (834·4 to 904·0)	0·0 (0·0 to 0·0)	5335·0 (5182·6 to 5479·7)	3857·5 (3712·7 to 3992·0)	470·6 (447·1 to 495·4)	1007·0 (965·5 to 1046·0)	0·0 (0·0 to 0·0)
France	2025	4660·8 (4509·8 to 4806·7)	3362·2 (3215·3 to 3499·2)	407·3 (387·1 to 428·9)	891·3 (852·0 to 929·4)	0·0 (0·0 to 0·0)	5393·1 (5218·3 to 5561·8)	3890·4 (3720·4 to 4048·9)	471·3 (447·9 to 496·3)	1031·3 (985·8 to 1075·4)	0·0 (0·0 to 0·0)
France	2026	4700·8 (4531·4 to 4873·9)	3382·8 (3216·7 to 3539·1)	407·0 (386·8 to 428·6)	911·0 (867·9 to 952·9)	0·0 (0·0 to 0·0)	5439·2 (5243·3 to 5639·6)	3914·2 (3722·1 to 4095·1)	470·9 (447·6 to 495·9)	1054·1 (1004·3 to 1102·6)	0·0 (0·0 to 0·0)
France	2027	4741·5 (4556·6 to 4928·6)	3403·8 (3217·0 to 3577·5)	406·6 (386·4 to 428·3)	931·0 (884·8 to 976·7)	0·0 (0·0 to 0·0)	5486·4 (5272·5 to 5702·9)	3938·6 (3722·3 to 4139·5)	470·5 (447·1 to 495·5)	1077·3 (1023·8 to 1130·2)	0·0 (0·0 to 0·0)
France	2028	4794·0 (4606·9 to 4989·1)	3434·0 (3246·1 to 3622·0)	406·8 (386·4 to 428·5)	953·1 (903·8 to 1004·0)	0·0 (0·0 to 0·0)	5547·1 (5330·6 to 5772·8)	3973·5 (3756·0 to 4191·0)	470·7 (447·1 to 495·9)	1102·9 (1045·8 to 1161·8)	0·0 (0·0 to 0·0)
France	2029	4844·1 (4645·3 to 5054·9)	3462·7 (3271·0 to 3655·7)	406·9 (386·5 to 428·5)	974·5 (919·4 to 1030·8)	0·0 (0·0 to 0·0)	5605·1 (5375·1 to 5849·0)	4006·7 (3784·9 to 4230·0)	470·8 (447·3 to 495·8)	1127·6 (1063·8 to 1192·7)	0·0 (0·0 to 0·0)
France	2030	4900·4 (4691·6 to 5126·2)	3496·2 (3294·6 to 3706·3)	407·5 (387·3 to 429·1)	996·7 (940·3 to 1053·9)	0·0 (0·0 to 0·0)	5670·2 (5428·6 to 5931·5)	4045·4 (3812·2 to 4288·5)	471·5 (448·2 to 496·5)	1153·2 (1088·1 to 1219·4)	0·0 (0·0 to 0·0)
France	2031	4955·6 (4732·2 to 5195·3)	3528·3 (3317·8 to 3750·2)	408·1 (388·1 to 429·7)	1019·2 (959·0 to 1078·0)	0·0 (0·0 to 0·0)	5734·2 (5475·7 to 6011·5)	4082·6 (3839·1 to 4339·4)	472·2 (449·1 to 497·3)	1179·3 (1109·6 to 1247·4)	0·0 (0·0 to 0·0)
France	2032	5016·1 (4781·7 to 5270·5)	3563·4 (3341·8 to 3794·9)	409·2 (389·3 to 430·9)	1043·4 (977·4 to 1107·0)	0·0 (0·0 to 0·0)	5804·1 (5532·9 to 6098·5)	4123·3 (3866·8 to 4391·1)	473·5 (450·4 to 498·6)	1207·4 (1130·9 to 1280·9)	0·0 (0·0 to 0·0)
France	2033	5077·2 (4824·6 to 5344·2)	3598·5 (3356·3 to 3845·8)	410·3 (390·1 to 432·1)	1068·4 (997·9 to 1137·8)	0·0 (0·0 to 0·0)	5874·9 (5582·6 to 6183·7)	4163·9 (3883·5 to 4449·9)	474·7 (451·3 to 499·9)	1236·2 (1154·7 to 1316·5)	0·0 (0·0 to 0·0)
France	2034	5136·2 (4872·1 to 5414·6)	3631·3 (3378·1 to 3890·3)	411·3 (390·7 to 433·0)	1093·7 (1018·4 to 1169·9)	0·0 (0·0 to 0·0)	5943·1 (5637·5 to 6265·2)	4201·8 (3908·8 to 4501·5)	475·9 (452·1 to 501·0)	1265·5 (1178·4 to 1353·7)	0·0 (0·0 to 0·0)
France	2035	5194·5 (4906·3 to 5489·2)	3662·7 (3388·0 to 3935·6)	412·3 (391·8 to 434·0)	1119·6 (1036·5 to 1203·4)	0·0 (0·0 to 0·0)	6010·6 (5677·1 to 6351·5)	4238·1 (3920·2 to 4553·9)	477·0 (453·4 to 502·2)	1295·4 (1199·3 to 1392·4)	0·0 (0·0 to 0·0)
France	2036	5251·6 (4946·8 to 5560·0)	3692·4 (3404·1 to 3979·0)	413·2 (392·9 to 435·0)	1145·9 (1055·0 to 1239·3)	0·0 (0·0 to 0·0)	6076·6 (5723·9 to 6433·5)	4272·5 (3938·8 to 4604·1)	478·1 (454·6 to 503·3)	1326·0 (1220·8 to 1434·0)	0·0 (0·0 to 0·0)
France	2037	5308·8 (4978·7 to 5640·3)	3721·1 (3411·9 to 4026·4)	414·3 (393·9 to 436·2)	1173·4 (1074·3 to 1279·3)	0·0 (0·0 to 0·0)	6142·8 (5760·9 to 6526·4)	4305·7 (3947·9 to 4659·0)	479·4 (455·8 to 504·8)	1357·7 (1243·1 to 1480·3)	0·0 (0·0 to 0·0)

France	2038	5367·2 (5028·0 to 5714·2)	3749·1 (3421·6 to 4063·0)	415·7 (395·3 to 437·8)	1202·4 (1094·8 to 1319·1)	0·0 (0·0 to 0·0)	6210·4 (5817·9 to 6611·9)	4338·1 (3959·2 to 4701·3)	481·0 (457·4 to 506·5)	1391·3 (1266·9 to 1526·3)	0·0 (0·0 to 0·0)
France	2039	5426·8 (5075·3 to 5808·0)	3776·8 (3447·0 to 4103·8)	417·2 (396·8 to 439·5)	1232·7 (1117·6 to 1361·2)	0·0 (0·0 to 0·0)	6279·4 (5872·7 to 6720·5)	4370·2 (3988·5 to 4748·6)	482·8 (459·1 to 508·5)	1426·4 (1293·2 to 1575·1)	0·0 (0·0 to 0·0)
France	2040	5491·3 (5127·6 to 5882·1)	3807·1 (3451·5 to 4141·3)	419·2 (398·7 to 441·5)	1265·0 (1138·2 to 1406·6)	0·0 (0·0 to 0·0)	6354·0 (5933·2 to 6806·2)	4405·3 (3993·8 to 4791·9)	485·0 (461·3 to 510·8)	1463·7 (1317·1 to 1627·6)	0·0 (0·0 to 0·0)
France	2041	5556·4 (5179·5 to 5950·9)	3836·5 (3477·0 to 4190·2)	421·3 (401·0 to 443·6)	1298·7 (1159·7 to 1453·7)	0·0 (0·0 to 0·0)	6429·4 (5993·2 to 6885·9)	4439·2 (4023·2 to 4848·5)	487·5 (464·0 to 513·3)	1502·7 (1341·9 to 1682·1)	0·0 (0·0 to 0·0)
France	2042	5624·3 (5242·9 to 6028·9)	3866·6 (3489·2 to 4230·5)	423·7 (403·1 to 446·2)	1334·0 (1183·1 to 1503·4)	0·0 (0·0 to 0·0)	6507·9 (6066·6 to 6976·1)	4474·1 (4037·4 to 4895·1)	490·2 (466·4 to 516·3)	1543·6 (1368·9 to 1739·6)	0·0 (0·0 to 0·0)
France	2043	5693·3 (5298·1 to 6128·6)	3896·5 (3518·3 to 4277·3)	426·2 (405·1 to 448·7)	1370·7 (1203·9 to 1555·6)	0·0 (0·0 to 0·0)	6587·8 (6130·4 to 7091·4)	4508·6 (4071·0 to 4949·2)	493·1 (468·8 to 519·2)	1586·0 (1393·1 to 1800·0)	0·0 (0·0 to 0·0)
France	2044	5764·2 (5346·1 to 6226·4)	3926·6 (3542·6 to 4319·9)	428·8 (407·4 to 451·5)	1408·8 (1229·2 to 1610·6)	0·0 (0·0 to 0·0)	6669·7 (6186·0 to 7204·6)	4543·4 (4099·2 to 4998·5)	496·2 (471·4 to 522·5)	1630·1 (1422·3 to 1863·6)	0·0 (0·0 to 0·0)
France	2045	5838·9 (5416·3 to 6324·2)	3958·6 (3565·1 to 4364·3)	431·6 (409·9 to 454·7)	1448·7 (1256·0 to 1668·0)	0·0 (0·0 to 0·0)	6756·2 (6267·3 to 7317·8)	4580·5 (4125·2 to 5049·9)	499·5 (474·3 to 526·2)	1676·3 (1453·3 to 1930·0)	0·0 (0·0 to 0·0)
France	2046	5915·0 (5457·0 to 6410·0)	3990·4 (3585·8 to 4400·8)	434·6 (412·6 to 458·2)	1490·1 (1284·0 to 1729·2)	0·0 (0·0 to 0·0)	6844·3 (6314·3 to 7417·0)	4617·3 (4149·1 to 5092·2)	502·9 (477·4 to 530·1)	1724·1 (1485·7 to 2000·9)	0·0 (0·0 to 0·0)
France	2047	5993·9 (5522·8 to 6507·1)	4022·7 (3608·1 to 4446·9)	437·8 (415·3 to 461·7)	1533·4 (1311·8 to 1793·1)	0·0 (0·0 to 0·0)	6935·5 (6390·5 to 7529·4)	4654·7 (4175·0 to 5145·5)	506·6 (480·6 to 534·2)	1774·2 (1517·9 to 2074·8)	0·0 (0·0 to 0·0)
France	2048	6075·1 (5565·7 to 6605·6)	4055·9 (3627·3 to 4493·0)	441·1 (418·6 to 465·3)	1578·2 (1340·4 to 1856·5)	0·0 (0·0 to 0·0)	7029·5 (6440·1 to 7643·3)	4693·1 (4197·2 to 5198·8)	510·4 (484·4 to 538·4)	1826·1 (1551·0 to 2148·2)	0·0 (0·0 to 0·0)
France	2049	6158·6 (5664·6 to 6712·3)	4089·8 (3660·9 to 4528·3)	444·4 (421·2 to 468·9)	1624·4 (1369·8 to 1925·3)	0·0 (0·0 to 0·0)	7126·2 (6554·6 to 7766·8)	4732·3 (4236·0 to 5239·7)	514·2 (487·4 to 542·6)	1879·6 (1585·0 to 2227·8)	0·0 (0·0 to 0·0)
France	2050	6244·8 (5714·0 to 6811·4)	4124·0 (3685·9 to 4579·5)	448·0 (424·1 to 472·6)	1672·8 (1400·3 to 1999·8)	0·0 (0·0 to 0·0)	7225·9 (6611·7 to 7881·5)	4771·8 (4264·9 to 5298·9)	518·4 (490·7 to 546·8)	1935·6 (1620·3 to 2314·0)	0·0 (0·0 to 0·0)
Federated States of Micronesia*	1995	108·8 (95·1 to 124·0)	90·2 (76·8 to 105·5)	16·8 (13·4 to 21·0)	0·0 (0·0 to 0·0)	1·8 (1·8 to 1·8)	97·3 (85·1 to 110·9)	80·7 (68·7 to 94·4)	15·0 (12·0 to 18·8)	0·0 (0·0 to 0·0)	1·6 (1·6 to 1·6)

Federated States of Micronesia*	1996	103·0 (90·9 to 117·2)	86·4 (74·7 to 100·4)	16·6 (13·1 to 20·9)	0·0 (0·0 to 0·0)	0·0 (0·0 to 0·0)	92·1 (81·3 to 104·8)	77·3 (66·8 to 89·8)	14·8 (11·7 to 18·7)	0·0 (0·0 to 0·0)	0·0 (0·0 to 0·0)
Federated States of Micronesia*	1997	95·6 (85·2 to 108·3)	78·7 (67·8 to 90·7)	16·0 (12·7 to 20·2)	0·0 (0·0 to 0·0)	0·9 (0·9 to 0·9)	85·5 (76·2 to 96·9)	70·4 (60·7 to 81·1)	14·3 (11·4 to 18·1)	0·0 (0·0 to 0·0)	0·8 (0·8 to 0·8)
Federated States of Micronesia*	1998	100·4 (89·4 to 113·4)	83·4 (72·6 to 95·6)	16·3 (12·9 to 20·7)	0·0 (0·0 to 0·0)	0·7 (0·7 to 0·7)	89·8 (79·9 to 101·4)	74·6 (64·9 to 85·5)	14·6 (11·6 to 18·5)	0·0 (0·0 to 0·0)	0·6 (0·6 to 0·6)
Federated States of Micronesia*	1999	192·0 (181·9 to 204·8)	80·9 (71·0 to 93·0)	16·0 (12·5 to 19·9)	0·0 (0·0 to 0·0)	95·1 (95·1 to 95·1)	171·8 (162·7 to 183·2)	72·3 (63·5 to 83·2)	14·3 (11·2 to 17·8)	0·0 (0·0 to 0·0)	85·1 (85·1 to 85·1)
Federated States of Micronesia*	2000	92·3 (82·6 to 103·7)	74·2 (64·8 to 85·0)	15·4 (12·0 to 19·1)	0·0 (0·0 to 0·0)	2·7 (2·7 to 2·7)	82·5 (73·9 to 92·8)	66·3 (58·0 to 76·0)	13·7 (10·7 to 17·1)	0·0 (0·0 to 0·0)	2·4 (2·4 to 2·4)
Federated States of Micronesia*	2001	91·7 (81·4 to 102·8)	73·6 (64·4 to 84·6)	15·1 (11·9 to 19·0)	0·0 (0·0 to 0·0)	2·9 (2·9 to 2·9)	82·0 (72·8 to 92·0)	65·8 (57·6 to 75·6)	13·5 (10·6 to 17·0)	0·0 (0·0 to 0·0)	2·6 (2·6 to 2·6)
Federated States of Micronesia*	2002	87·7 (78·0 to 98·1)	70·8 (62·1 to 81·1)	14·7 (11·4 to 18·4)	0·0 (0·0 to 0·0)	2·2 (2·2 to 2·2)	78·4 (69·8 to 87·7)	63·3 (55·6 to 72·5)	13·2 (10·2 to 16·5)	0·0 (0·0 to 0·0)	2·0 (2·0 to 2·0)
Federated States of Micronesia*	2003	151·5 (141·3 to 162·6)	82·7 (73·5 to 93·9)	15·3 (11·9 to 19·1)	0·0 (0·0 to 0·0)	53·5 (53·5 to 53·5)	135·5 (126·4 to 145·5)	74·0 (65·7 to 84·0)	13·7 (10·6 to 17·1)	0·0 (0·0 to 0·0)	47·8 (47·8 to 47·8)
Federated States of Micronesia*	2004	322·0 (312·1 to 333·0)	82·9 (73·6 to 93·1)	14·8 (11·4 to 18·6)	0·0 (0·0 to 0·0)	224·3 (224·3 to 224·3)	288·0 (279·1 to 297·9)	74·1 (65·8 to 83·2)	13·2 (10·2 to 16·6)	0·0 (0·0 to 0·0)	200·7 (200·7 to 200·7)
Federated States of Micronesia*	2005	348·0 (338·1 to 358·7)	89·8 (80·8 to 100·4)	14·8 (11·6 to 18·5)	0·0 (0·0 to 0·0)	243·4 (243·4 to 243·4)	311·3 (302·4 to 320·8)	80·4 (72·2 to 89·8)	13·2 (10·4 to 16·6)	0·0 (0·0 to 0·0)	217·7 (217·7 to 217·7)
Federated States of Micronesia*	2006	316·4 (306·7 to 326·4)	95·3 (86·2 to 105·0)	14·6 (11·5 to 18·3)	0·0 (0·0 to 0·0)	206·6 (206·6 to 206·6)	283·0 (274·4 to 291·9)	85·2 (77·1 to 94·0)	13·0 (10·2 to 16·3)	0·0 (0·0 to 0·0)	184·8 (184·8 to 184·8)
Federated States of Micronesia*	2007	318·9 (309·7 to 327·9)	93·6 (84·7 to 102·1)	14·0 (11·1 to 17·4)	0·0 (0·0 to 0·0)	211·3 (211·3 to 211·3)	285·2 (277·0 to 293·3)	83·7 (75·8 to 91·3)	12·5 (9·9 to 15·5)	0·0 (0·0 to 0·0)	189·0 (189·0 to 189·0)
Federated States of Micronesia*	2008	333·2 (323·9 to 342·1)	94·2 (85·0 to 102·9)	13·5 (10·7 to 16·6)	0·0 (0·0 to 0·0)	225·5 (225·5 to 225·5)	298·0 (289·7 to 306·0)	84·3 (76·0 to 92·0)	12·0 (9·5 to 14·9)	0·0 (0·0 to 0·0)	201·7 (201·7 to 201·7)
Federated States of Micronesia*	2009	390·2 (381·1 to 399·2)	94·4 (85·5 to 103·0)	13·0 (10·3 to 15·9)	0·0 (0·0 to 0·0)	282·8 (282·8 to 282·8)	349·0 (340·9 to 357·1)	84·5 (76·5 to 92·1)	11·6 (9·3 to 14·2)	0·0 (0·0 to 0·0)	252·9 (252·9 to 252·9)

Federated States of Micronesia*	2010	132·7 (123·4 to 142·2)	101·1 (92·3 to 110·8)	12·8 (10·4 to 15·7)	0·0 (0·0 to 0·0)	18·7 (18·7 to 18·7)	118·7 (110·4 to 127·2)	90·4 (82·5 to 99·1)	11·5 (9·3 to 14·0)	0·0 (0·0 to 0·0)	16·7 (16·7 to 16·7)
Federated States of Micronesia*	2011	142·3 (132·7 to 152·9)	108·6 (98·6 to 119·1)	12·6 (10·3 to 15·3)	0·0 (0·0 to 0·0)	21·0 (21·0 to 21·0)	127·2 (118·6 to 136·8)	97·2 (88·2 to 106·6)	11·3 (9·2 to 13·7)	0·0 (0·0 to 0·0)	18·8 (18·8 to 18·8)
Federated States of Micronesia*	2012	373·2 (362·9 to 384·0)	109·2 (98·7 to 119·7)	12·2 (10·0 to 14·7)	0·0 (0·0 to 0·0)	251·9 (251·9 to 251·9)	333·8 (324·6 to 343·5)	97·6 (88·3 to 107·0)	10·9 (9·0 to 13·2)	0·0 (0·0 to 0·0)	225·3 (225·3 to 225·3)
Federated States of Micronesia*	2013	132·3 (121·5 to 143·9)	108·1 (97·5 to 119·1)	11·7 (9·7 to 14·0)	0·0 (0·0 to 0·0)	12·5 (12·5 to 12·5)	118·4 (108·7 to 128·7)	96·7 (87·3 to 106·6)	10·5 (8·7 to 12·5)	0·0 (0·0 to 0·0)	11·2 (11·2 to 11·2)
Federated States of Micronesia*	2014	420·3 (409·5 to 431·4)	108·0 (97·7 to 119·1)	11·3 (9·7 to 13·1)	0·0 (0·0 to 0·0)	301·0 (301·0 to 301·0)	376·0 (366·3 to 385·8)	96·6 (87·4 to 106·5)	10·1 (8·7 to 11·7)	0·0 (0·0 to 0·0)	269·2 (269·2 to 269·2)
Federated States of Micronesia*	2015	222·0 (210·7 to 233·6)	115·8 (104·9 to 127·5)	11·3 (10·1 to 12·5)	0·0 (0·0 to 0·0)	94·9 (94·9 to 94·9)	198·6 (188·4 to 209·0)	103·6 (93·9 to 114·0)	10·1 (9·0 to 11·2)	0·0 (0·0 to 0·0)	84·8 (84·8 to 84·8)
Federated States of Micronesia*	2016	144·7 (132·2 to 157·9)	128·3 (115·9 to 141·5)	11·3 (10·5 to 12·2)	0·0 (0·0 to 0·0)	5·1 (5·1 to 5·1)	129·4 (118·3 to 141·2)	114·7 (103·7 to 126·5)	10·1 (9·4 to 11·0)	0·0 (0·0 to 0·0)	4·6 (4·6 to 4·6)
Federated States of Micronesia*	2017	147·6 (134·4 to 162·6)	129·5 (116·0 to 144·4)	10·9 (10·1 to 11·8)	0·0 (0·0 to 0·0)	7·2 (7·2 to 7·2)	132·1 (120·3 to 145·4)	115·9 (103·7 to 129·2)	9·8 (9·0 to 10·6)	0·0 (0·0 to 0·0)	6·4 (6·4 to 6·4)
Federated States of Micronesia*	2018	146·9 (131·9 to 163·3)	127·8 (113·1 to 144·4)	10·5 (9·4 to 11·6)	0·0 (0·0 to 0·0)	8·6 (8·6 to 8·6)	131·4 (118·0 to 146·1)	114·3 (101·1 to 129·2)	9·4 (8·4 to 10·4)	0·0 (0·0 to 0·0)	7·7 (7·7 to 7·7)
Federated States of Micronesia*	2019	147·0 (131·1 to 164·1)	127·3 (111·4 to 144·4)	10·6 (9·5 to 11·8)	0·0 (0·0 to 0·0)	9·0 (9·0 to 9·1)	131·4 (117·3 to 146·8)	113·9 (99·7 to 129·2)	9·5 (8·5 to 10·5)	0·0 (0·0 to 0·0)	8·1 (8·0 to 8·1)
Federated States of Micronesia*	2020	162·5 (144·5 to 182·6)	141·5 (123·3 to 161·8)	10·6 (9·5 to 11·8)	0·0 (0·0 to 0·0)	10·3 (10·0 to 10·4)	145·3 (129·3 to 163·3)	126·6 (110·3 to 144·7)	9·5 (8·5 to 10·5)	0·0 (0·0 to 0·0)	9·2 (8·9 to 9·3)
Federated States of Micronesia*	2021	160·1 (142·3 to 180·1)	140·3 (122·2 to 160·3)	10·6 (9·5 to 11·7)	0·0 (0·0 to 0·0)	9·3 (8·9 to 9·5)	143·2 (127·2 to 161·1)	125·5 (109·3 to 143·4)	9·4 (8·5 to 10·5)	0·0 (0·0 to 0·0)	8·3 (8·0 to 8·5)
Federated States of Micronesia*	2022	148·7 (129·6 to 169·2)	130·8 (111·7 to 151·4)	10·7 (9·6 to 11·9)	0·0 (0·0 to 0·1)	7·2 (6·7 to 7·6)	133·0 (116·0 to 151·4)	117·0 (99·9 to 135·4)	9·6 (8·6 to 10·6)	0·0 (0·0 to 0·0)	6·4 (6·0 to 6·8)
Federated States of Micronesia*	2023	151·6 (131·8 to 172·9)	133·5 (113·5 to 154·8)	10·8 (9·7 to 12·0)	0·0 (0·0 to 0·1)	7·3 (6·9 to 7·8)	135·6 (117·9 to 154·7)	119·4 (101·6 to 138·4)	9·7 (8·7 to 10·7)	0·0 (0·0 to 0·1)	6·6 (6·2 to 7·0)

Federated States of Micronesia*	2024	154·8 (134·9 to 176·8)	136·3 (115·7 to 158·1)	10·9 (9·8 to 12·1)	0·0 (0·0 to 0·1)	7·5 (7·1 to 8·1)	138·5 (120·6 to 158·2)	121·9 (103·5 to 141·5)	9·8 (8·8 to 10·8)	0·0 (0·0 to 0·1)	6·7 (6·3 to 7·2)
Federated States of Micronesia*	2025	157·9 (136·5 to 180·6)	139·1 (118·0 to 162·0)	11·0 (9·9 to 12·2)	0·0 (0·0 to 0·1)	7·7 (7·2 to 8·3)	141·2 (122·1 to 161·5)	124·4 (105·5 to 144·9)	9·9 (8·9 to 11·0)	0·0 (0·0 to 0·1)	6·9 (6·4 to 7·4)
Federated States of Micronesia*	2026	160·9 (138·9 to 184·1)	141·8 (119·7 to 165·4)	11·1 (10·0 to 12·4)	0·0 (0·0 to 0·1)	7·9 (7·4 to 8·6)	143·9 (124·2 to 164·7)	126·9 (107·1 to 148·0)	10·0 (8·9 to 11·1)	0·0 (0·0 to 0·1)	7·1 (6·6 to 7·7)
Federated States of Micronesia*	2027	164·1 (140·7 to 188·7)	144·8 (121·7 to 169·4)	11·2 (10·1 to 12·5)	0·0 (0·0 to 0·1)	8·1 (7·5 to 8·8)	146·8 (125·9 to 168·8)	129·5 (108·9 to 151·5)	10·0 (9·0 to 11·1)	0·0 (0·0 to 0·1)	7·2 (6·7 to 7·9)
Federated States of Micronesia*	2028	168·8 (145·5 to 193·8)	149·2 (125·6 to 174·5)	11·3 (10·1 to 12·5)	0·0 (0·0 to 0·1)	8·3 (7·7 to 9·1)	151·0 (130·1 to 173·3)	133·4 (112·4 to 156·1)	10·1 (9·1 to 11·2)	0·0 (0·0 to 0·1)	7·4 (6·9 to 8·1)
Federated States of Micronesia*	2029	173·1 (148·9 to 198·5)	153·1 (128·8 to 178·9)	11·3 (10·2 to 12·6)	0·0 (0·0 to 0·1)	8·5 (7·8 to 9·4)	154·8 (133·2 to 177·5)	137·0 (115·2 to 160·0)	10·1 (9·1 to 11·3)	0·0 (0·0 to 0·1)	7·6 (7·0 to 8·4)
Federated States of Micronesia*	2030	177·2 (152·2 to 203·6)	157·0 (131·7 to 183·7)	11·4 (10·2 to 12·7)	0·0 (0·0 to 0·1)	8·8 (7·9 to 9·7)	158·5 (136·2 to 182·1)	140·4 (117·8 to 164·3)	10·2 (9·2 to 11·3)	0·0 (0·0 to 0·1)	7·8 (7·1 to 8·6)
Federated States of Micronesia*	2031	181·3 (155·3 to 209·6)	160·8 (135·2 to 188·9)	11·4 (10·3 to 12·7)	0·0 (0·0 to 0·1)	9·0 (8·1 to 10·0)	162·2 (138·9 to 187·5)	143·8 (120·9 to 168·9)	10·2 (9·2 to 11·4)	0·0 (0·0 to 0·1)	8·0 (7·3 to 8·9)
Federated States of Micronesia*	2032	185·3 (159·3 to 214·1)	164·4 (138·6 to 193·4)	11·5 (10·3 to 12·8)	0·0 (0·0 to 0·1)	9·3 (8·4 to 10·3)	165·7 (142·4 to 191·5)	147·1 (124·0 to 173·0)	10·3 (9·2 to 11·4)	0·0 (0·0 to 0·1)	8·3 (7·5 to 9·2)
Federated States of Micronesia*	2033	189·2 (162·4 to 219·0)	168·1 (141·3 to 197·7)	11·5 (10·3 to 12·8)	0·0 (0·0 to 0·1)	9·6 (8·6 to 10·8)	169·3 (145·3 to 195·9)	150·3 (126·4 to 176·8)	10·3 (9·2 to 11·5)	0·0 (0·0 to 0·1)	8·6 (7·7 to 9·7)
Federated States of Micronesia*	2034	193·2 (165·5 to 223·8)	171·6 (144·7 to 201·8)	11·5 (10·4 to 12·9)	0·0 (0·0 to 0·1)	10·0 (8·9 to 11·2)	172·8 (148·1 to 200·2)	153·5 (129·5 to 180·5)	10·3 (9·3 to 11·5)	0·0 (0·0 to 0·1)	8·9 (8·0 to 10·1)
Federated States of Micronesia*	2035	196·9 (168·6 to 228·7)	175·0 (147·5 to 206·5)	11·6 (10·4 to 12·9)	0·1 (0·0 to 0·1)	10·4 (9·2 to 11·8)	176·2 (150·8 to 204·6)	156·5 (131·9 to 184·7)	10·3 (9·3 to 11·5)	0·0 (0·0 to 0·1)	9·3 (8·3 to 10·6)
Federated States of Micronesia*	2036	200·5 (171·7 to 232·8)	178·1 (149·5 to 211·0)	11·6 (10·4 to 12·9)	0·1 (0·0 to 0·1)	10·8 (9·5 to 12·4)	179·3 (153·6 to 208·3)	159·3 (133·7 to 188·7)	10·4 (9·3 to 11·5)	0·0 (0·0 to 0·1)	9·6 (8·5 to 11·1)
Federated States of Micronesia*	2037	203·9 (174·0 to 236·2)	181·1 (152·1 to 213·6)	11·6 (10·4 to 12·9)	0·1 (0·0 to 0·1)	11·2 (9·9 to 12·9)	182·4 (155·6 to 211·3)	161·9 (136·0 to 191·0)	10·4 (9·3 to 11·6)	0·0 (0·0 to 0·1)	10·0 (8·9 to 11·6)

Federated States of Micronesia*	2038	207·3 (176·7 to 240·5)	184·0 (154·4 to 217·6)	11·6 (10·4 to 12·9)	0·1 (0·0 to 0·1)	11·7 (10·3 to 13·6)	185·4 (158·0 to 215·2)	164·6 (138·1 to 194·6)	10·4 (9·3 to 11·6)	0·1 (0·0 to 0·1)	10·4 (9·2 to 12·2)
Federated States of Micronesia*	2039	210·7 (179·7 to 244·7)	186·8 (156·7 to 221·1)	11·7 (10·5 to 13·0)	0·1 (0·0 to 0·1)	12·1 (10·6 to 14·3)	188·5 (160·7 to 218·8)	167·1 (140·1 to 197·7)	10·4 (9·3 to 11·6)	0·1 (0·0 to 0·1)	10·9 (9·5 to 12·8)
Federated States of Micronesia*	2040	214·1 (182·8 to 250·0)	189·8 (159·5 to 224·9)	11·7 (10·5 to 13·0)	0·1 (0·0 to 0·1)	12·6 (10·9 to 15·0)	191·5 (163·5 to 223·6)	169·7 (142·7 to 201·1)	10·5 (9·4 to 11·7)	0·1 (0·0 to 0·1)	11·3 (9·8 to 13·4)
Federated States of Micronesia*	2041	217·4 (185·2 to 253·7)	192·6 (161·2 to 228·8)	11·7 (10·5 to 13·1)	0·1 (0·0 to 0·1)	13·1 (11·3 to 15·7)	194·5 (165·6 to 227·0)	172·2 (144·2 to 204·6)	10·5 (9·4 to 11·7)	0·1 (0·0 to 0·1)	11·7 (10·1 to 14·1)
Federated States of Micronesia*	2042	220·9 (187·8 to 255·7)	195·5 (163·3 to 231·8)	11·8 (10·6 to 13·1)	0·1 (0·0 to 0·1)	13·5 (11·6 to 16·4)	197·6 (168·0 to 228·7)	174·8 (146·1 to 207·3)	10·6 (9·5 to 11·8)	0·1 (0·0 to 0·1)	12·1 (10·4 to 14·6)
Federated States of Micronesia*	2043	224·2 (190·4 to 261·2)	198·3 (166·0 to 235·6)	11·9 (10·6 to 13·2)	0·1 (0·0 to 0·1)	14·0 (11·9 to 17·2)	200·5 (170·3 to 233·7)	177·3 (148·5 to 210·7)	10·6 (9·5 to 11·8)	0·1 (0·0 to 0·1)	12·5 (10·7 to 15·3)
Federated States of Micronesia*	2044	227·5 (193·4 to 265·0)	201·1 (167·6 to 238·9)	11·9 (10·7 to 13·3)	0·1 (0·0 to 0·1)	14·4 (12·3 to 17·8)	203·5 (173·0 to 237·0)	179·8 (149·9 to 213·7)	10·7 (9·6 to 11·9)	0·1 (0·0 to 0·1)	12·9 (11·0 to 15·9)
Federated States of Micronesia*	2045	230·8 (195·6 to 268·9)	203·8 (170·2 to 242·2)	12·0 (10·8 to 13·4)	0·1 (0·0 to 0·1)	14·9 (12·5 to 18·3)	206·4 (175·0 to 240·5)	182·3 (152·2 to 216·6)	10·7 (9·6 to 12·0)	0·1 (0·0 to 0·1)	13·3 (11·2 to 16·4)
Federated States of Micronesia*	2046	234·1 (199·1 to 273·0)	206·5 (172·1 to 245·3)	12·1 (10·8 to 13·5)	0·1 (0·0 to 0·2)	15·3 (12·8 to 19·1)	209·4 (178·1 to 244·2)	184·7 (154·0 to 219·4)	10·8 (9·7 to 12·1)	0·1 (0·0 to 0·1)	13·7 (11·4 to 17·1)
Federated States of Micronesia*	2047	237·6 (202·1 to 277·3)	209·5 (173·9 to 248·8)	12·2 (10·9 to 13·6)	0·1 (0·0 to 0·2)	15·8 (13·1 to 19·8)	212·5 (180·8 to 248·0)	187·3 (155·5 to 222·6)	10·9 (9·8 to 12·2)	0·1 (0·0 to 0·1)	14·2 (11·7 to 17·7)
Federated States of Micronesia*	2048	241·1 (205·0 to 280·7)	212·4 (176·9 to 252·3)	12·3 (11·0 to 13·7)	0·1 (0·0 to 0·2)	16·3 (13·6 to 20·7)	215·7 (183·3 to 251·1)	190·0 (158·3 to 225·6)	11·0 (9·8 to 12·2)	0·1 (0·0 to 0·1)	14·6 (12·2 to 18·5)
Federated States of Micronesia*	2049	244·9 (208·0 to 285·4)	215·5 (179·8 to 257·0)	12·3 (11·1 to 13·8)	0·1 (0·0 to 0·2)	16·9 (13·8 to 21·7)	219·0 (186·1 to 255·3)	192·8 (160·8 to 229·9)	11·0 (9·9 to 12·3)	0·1 (0·0 to 0·2)	15·1 (12·4 to 19·4)
Federated States of Micronesia*	2050	248·7 (211·2 to 290·2)	218·6 (182·4 to 260·1)	12·4 (11·1 to 13·9)	0·1 (0·0 to 0·2)	17·5 (14·2 to 22·6)	222·4 (188·9 to 259·5)	195·5 (163·1 to 232·6)	11·1 (10·0 to 12·4)	0·1 (0·0 to 0·2)	15·7 (12·7 to 20·2)
Gabon*	1995	308·6 (286·0 to 334·5)	102·6 (90·5 to 115·4)	182·1 (164·0 to 203·3)	23·6 (16·9 to 32·1)	0·2 (0·2 to 0·2)	680·8 (631·1 to 738·0)	226·5 (199·7 to 254·7)	401·8 (361·8 to 448·5)	52·2 (37·4 to 70·9)	0·4 (0·4 to 0·4)

Gabon*	1996	302·8 (284·2 to 323·6)	99·1 (88·9 to 109·8)	178·1 (162·9 to 194·4)	22·9 (16·8 to 30·7)	2·6 (2·6 to 2·6)	668·0 (627·1 to 714·1)	218·6 (196·0 to 242·2)	393·0 (359·5 to 428·8)	50·6 (37·0 to 67·7)	5·8 (5·8 to 5·8)
Gabon*	1997	321·4 (303·3 to 341·7)	109·2 (98·6 to 121·0)	182·5 (169·1 to 198·4)	23·5 (17·5 to 31·7)	6·1 (6·1 to 6·1)	709·2 (669·2 to 753·9)	241·0 (217·5 to 266·9)	402·7 (373·0 to 437·8)	51·9 (38·5 to 69·9)	13·6 (13·6 to 13·6)
Gabon*	1998	368·4 (348·0 to 390·8)	132·7 (120·4 to 145·9)	200·7 (185·1 to 218·6)	26·4 (19·5 to 35·6)	8·6 (8·6 to 8·6)	812·7 (767·9 to 862·3)	292·8 (265·7 to 321·9)	442·8 (408·5 to 482·3)	58·2 (43·1 to 78·4)	18·9 (18·9 to 18·9)
Gabon*	1999	287·5 (270·4 to 306·6)	96·1 (86·9 to 106·5)	165·2 (151·7 to 179·8)	22·0 (16·3 to 29·1)	4·2 (4·2 to 4·2)	634·3 (596·6 to 676·5)	212·1 (191·7 to 235·0)	364·4 (334·7 to 396·6)	48·4 (36·1 to 64·1)	9·3 (9·3 to 9·3)
Gabon*	2000	250·2 (234·6 to 266·6)	86·3 (78·3 to 96·6)	141·3 (129·2 to 153·9)	19·4 (14·4 to 25·8)	3·1 (3·1 to 3·1)	552·0 (517·6 to 588·2)	190·5 (172·7 to 213·2)	311·7 (285·0 to 339·6)	42·9 (31·9 to 56·8)	6·9 (6·9 to 6·9)
Gabon*	2001	272·7 (254·3 to 293·3)	105·2 (95·2 to 117·1)	143·7 (129·9 to 157·2)	21·1 (15·5 to 28·2)	2·7 (2·7 to 2·7)	601·7 (561·2 to 647·1)	232·0 (210·0 to 258·4)	317·2 (286·7 to 346·7)	46·6 (34·2 to 62·3)	6·0 (6·0 to 6·0)
Gabon*	2002	265·8 (247·3 to 285·4)	101·3 (91·6 to 112·4)	138·5 (124·9 to 152·5)	21·1 (15·6 to 27·6)	5·0 (5·0 to 5·0)	586·5 (545·6 to 629·7)	223·5 (202·1 to 248·1)	305·6 (275·7 to 336·4)	46·4 (34·5 to 61·0)	11·0 (11·0 to 11·0)
Gabon*	2003	251·8 (234·0 to 271·0)	95·9 (86·9 to 106·6)	131·8 (119·2 to 145·4)	20·2 (14·8 to 26·8)	3·9 (3·9 to 3·9)	555·7 (516·2 to 598·0)	211·6 (191·6 to 235·2)	290·8 (262·9 to 320·9)	44·7 (32·7 to 59·2)	8·6 (8·6 to 8·6)
Gabon*	2004	248·9 (232·0 to 267·7)	95·3 (86·2 to 106·4)	127·6 (114·5 to 141·7)	19·5 (14·2 to 26·3)	6·5 (6·5 to 6·5)	549·2 (511·8 to 590·6)	210·4 (190·2 to 234·9)	281·5 (252·6 to 312·6)	42·9 (31·3 to 57·9)	14·4 (14·4 to 14·4)
Gabon*	2005	244·0 (227·0 to 262·8)	94·8 (84·9 to 105·0)	124·1 (111·6 to 137·5)	18·7 (13·5 to 25·3)	6·4 (6·4 to 6·4)	538·3 (500·9 to 579·7)	209·1 (187·4 to 231·7)	273·8 (246·3 to 303·4)	41·2 (29·8 to 55·8)	14·2 (14·2 to 14·2)
Gabon*	2006	242·3 (225·4 to 260·6)	93·0 (83·4 to 103·3)	120·3 (108·2 to 133·4)	18·2 (13·3 to 24·7)	10·7 (10·7 to 10·7)	534·6 (497·3 to 574·9)	205·2 (184·1 to 228·0)	265·5 (238·7 to 294·3)	40·2 (29·3 to 54·4)	23·6 (23·6 to 23·6)
Gabon*	2007	233·2 (216·7 to 250·3)	91·9 (81·9 to 102·1)	115·7 (103·8 to 129·4)	18·1 (13·1 to 24·6)	7·5 (7·5 to 7·5)	514·5 (478·0 to 552·3)	202·7 (180·8 to 225·3)	255·2 (229·0 to 285·4)	40·0 (28·9 to 54·3)	16·6 (16·6 to 16·6)
Gabon*	2008	219·9 (204·4 to 237·5)	90·9 (81·2 to 100·6)	105·8 (94·7 to 118·7)	17·7 (13·0 to 23·8)	5·5 (5·5 to 5·5)	485·1 (451·0 to 524·0)	200·5 (179·1 to 222·0)	233·4 (209·0 to 261·8)	39·1 (28·7 to 52·6)	12·1 (12·1 to 12·1)
Gabon*	2009	226·9 (212·1 to 244·3)	103·2 (92·8 to 113·5)	96·2 (86·5 to 107·5)	19·5 (14·5 to 25·9)	8·0 (8·0 to 8·0)	500·7 (468·0 to 539·0)	227·8 (204·8 to 250·5)	212·4 (190·9 to 237·2)	43·0 (32·1 to 57·1)	17·6 (17·6 to 17·6)

Gabon*	2010	209·7 (195·0 to 225·4)	113·5 (102·9 to 124·7)	73·4 (65·7 to 81·1)	19·3 (14·3 to 25·9)	3·4 (3·4 to 3·4)	462·6 (430·3 to 497·4)	250·5 (227·1 to 275·0)	162·0 (144·9 to 179·0)	42·5 (31·6 to 57·1)	7·6 (7·6 to 7·6)
Gabon*	2011	204·8 (190·4 to 219·4)	117·6 (106·8 to 128·6)	64·2 (57·7 to 71·2)	19·1 (14·2 to 25·8)	3·8 (3·8 to 3·8)	451·8 (420·2 to 484·1)	259·5 (235·7 to 283·8)	141·7 (127·3 to 157·0)	42·2 (31·3 to 56·8)	8·4 (8·4 to 8·4)
Gabon*	2012	211·7 (196·4 to 226·6)	126·6 (115·5 to 138·5)	61·2 (55·1 to 67·9)	19·9 (14·7 to 26·7)	4·0 (4·0 to 4·0)	467·0 (433·4 to 499·9)	279·3 (254·9 to 305·5)	135·1 (121·6 to 149·8)	43·8 (32·4 to 58·8)	8·7 (8·7 to 8·7)
Gabon*	2013	260·4 (243·3 to 277·1)	165·4 (151·0 to 179·7)	66·9 (60·1 to 73·9)	22·4 (16·5 to 29·6)	5·8 (5·8 to 5·8)	574·6 (536·8 to 611·3)	364·8 (333·3 to 396·5)	147·6 (132·5 to 163·1)	49·4 (36·4 to 65·3)	12·8 (12·8 to 12·8)
Gabon*	2014	227·8 (213·1 to 242·3)	137·4 (126·0 to 149·0)	60·5 (54·6 to 67·2)	24·2 (18·3 to 31·8)	5·8 (5·8 to 5·8)	502·6 (470·2 to 534·6)	303·1 (278·1 to 328·7)	133·5 (120·5 to 148·3)	53·4 (40·3 to 70·2)	12·7 (12·7 to 12·7)
Gabon*	2015	234·2 (219·4 to 249·3)	141·9 (130·9 to 152·3)	59·9 (53·9 to 66·3)	29·1 (21·8 to 38·8)	3·2 (3·2 to 3·2)	516·7 (484·1 to 550·0)	313·1 (288·9 to 336·0)	132·2 (119·0 to 146·3)	64·2 (48·2 to 85·5)	7·0 (7·0 to 7·0)
Gabon*	2016	255·0 (240·6 to 270·6)	157·5 (147·1 to 167·8)	61·6 (55·3 to 68·7)	32·7 (24·3 to 42·7)	3·2 (3·2 to 3·2)	562·6 (530·8 to 597·1)	347·5 (324·5 to 370·3)	135·9 (122·0 to 151·6)	72·1 (53·7 to 94·2)	7·1 (7·1 to 7·1)
Gabon*	2017	268·8 (256·6 to 282·4)	144·9 (138·7 to 151·5)	59·0 (52·4 to 66·5)	31·4 (23·6 to 40·8)	33·4 (33·4 to 33·4)	593·0 (566·2 to 623·2)	319·8 (306·1 to 334·2)	130·2 (115·7 to 146·7)	69·4 (52·1 to 90·0)	73·7 (73·7 to 73·7)
Gabon*	2018	237·2 (224·1 to 250·8)	139·7 (135·7 to 143·4)	57·1 (49·6 to 65·4)	36·3 (27·2 to 47·4)	4·2 (4·2 to 4·2)	523·4 (494·5 to 553·3)	308·2 (299·4 to 316·3)	125·9 (109·3 to 144·2)	80·0 (60·1 to 104·5)	9·2 (9·2 to 9·2)
Gabon*	2019	245·1 (228·3 to 261·1)	145·2 (135·3 to 156·5)	57·8 (50·3 to 66·3)	37·9 (28·4 to 49·5)	4·2 (4·2 to 4·2)	540·9 (503·6 to 576·1)	320·4 (298·4 to 345·3)	127·6 (110·9 to 146·2)	83·6 (62·8 to 109·2)	9·2 (9·2 to 9·3)
Gabon*	2020	296·6 (280·8 to 312·2)	199·1 (189·3 to 210·2)	54·9 (47·7 to 62·9)	36·9 (27·7 to 48·5)	5·7 (5·6 to 5·8)	654·5 (619·5 to 688·9)	439·3 (417·7 to 463·8)	121·2 (105·3 to 138·9)	81·5 (61·2 to 107·0)	12·5 (12·4 to 12·7)
Gabon*	2021	252·6 (234·6 to 270·5)	158·0 (143·1 to 172·3)	52·6 (45·7 to 60·3)	36·3 (27·2 to 47·5)	5·7 (5·7 to 6·0)	557·2 (517·6 to 596·9)	348·5 (315·8 to 380·2)	116·0 (100·8 to 133·0)	80·0 (60·0 to 104·8)	12·7 (12·5 to 13·1)
Gabon*	2022	231·9 (212·7 to 251·1)	136·8 (121·2 to 153·0)	53·2 (46·2 to 61·0)	37·6 (28·1 to 49·3)	4·4 (4·2 to 4·7)	511·7 (469·2 to 553·9)	301·7 (267·3 to 337·6)	117·3 (101·9 to 134·5)	82·9 (62·1 to 108·7)	9·8 (9·3 to 10·4)
Gabon*	2023	237·5 (217·0 to 257·4)	140·5 (123·6 to 157·7)	53·8 (46·7 to 61·6)	38·8 (29·0 to 50·9)	4·5 (4·2 to 4·8)	524·0 (478·8 to 568·0)	309·9 (272·7 to 348·0)	118·6 (103·0 to 136·0)	85·5 (63·9 to 112·4)	9·9 (9·3 to 10·7)

Gabon*	2024	244·2 (222·7 to 264·9)	145·2 (127·7 to 163·2)	54·4 (47·2 to 62·3)	40·0 (29·9 to 52·5)	4·6 (4·3 to 5·0)	538·8 (491·4 to 584·5)	320·4 (281·7 to 360·1)	120·0 (104·1 to 137·5)	88·3 (66·0 to 115·9)	10·1 (9·5 to 10·9)
Gabon*	2025	251·3 (228·7 to 272·8)	150·3 (131·8 to 169·2)	55·0 (47·7 to 63·0)	41·4 (30·9 to 54·5)	4·7 (4·3 to 5·0)	554·5 (504·5 to 601·9)	331·5 (290·8 to 373·2)	121·4 (105·3 to 139·1)	91·3 (68·1 to 120·3)	10·3 (9·5 to 11·1)
Gabon*	2026	258·5 (234·6 to 281·0)	155·4 (136·1 to 175·2)	55·6 (48·3 to 63·8)	42·7 (31·9 to 56·3)	4·7 (4·4 to 5·2)	570·3 (517·6 to 619·9)	342·9 (300·3 to 386·6)	122·8 (106·6 to 140·7)	94·2 (70·4 to 124·1)	10·4 (9·6 to 11·4)
Gabon*	2027	266·0 (241·5 to 289·1)	160·8 (140·4 to 181·5)	56·3 (48·9 to 64·5)	44·1 (33·0 to 58·3)	4·8 (4·4 to 5·2)	586·8 (532·7 to 637·8)	354·7 (309·9 to 400·6)	124·2 (107·8 to 142·4)	97·4 (72·9 to 128·5)	10·5 (9·7 to 11·6)
Gabon*	2028	273·8 (248·9 to 297·8)	166·4 (144·7 to 188·7)	57·0 (49·5 to 65·3)	45·6 (34·2 to 60·1)	4·8 (4·4 to 5·4)	604·1 (549·2 to 657·0)	367·1 (319·2 to 416·3)	125·7 (109·2 to 144·1)	100·7 (75·5 to 132·6)	10·6 (9·7 to 11·8)
Gabon*	2029	281·8 (256·1 to 307·5)	172·1 (149·5 to 195·2)	57·7 (50·1 to 66·1)	47·1 (35·2 to 62·0)	4·9 (4·4 to 5·4)	621·7 (565·0 to 678·4)	379·7 (330·0 to 430·7)	127·2 (110·5 to 145·8)	104·0 (77·7 to 136·7)	10·7 (9·8 to 12·0)
Gabon*	2030	290·0 (262·8 to 317·4)	178·0 (154·4 to 202·4)	58·4 (50·7 to 66·9)	48·7 (36·3 to 64·1)	4·9 (4·4 to 5·5)	639·9 (579·8 to 700·4)	392·8 (340·6 to 446·6)	128·8 (111·8 to 147·6)	107·5 (80·0 to 141·4)	10·8 (9·8 to 12·1)
Gabon*	2031	298·4 (270·6 to 327·2)	184·0 (159·2 to 209·6)	59·1 (51·3 to 67·7)	50·4 (37·4 to 66·1)	5·0 (4·5 to 5·6)	658·5 (597·1 to 721·9)	406·1 (351·3 to 462·4)	130·3 (113·2 to 149·3)	111·1 (82·6 to 145·9)	11·0 (9·8 to 12·4)
Gabon*	2032	307·1 (277·9 to 337·6)	190·3 (164·4 to 217·1)	59·8 (52·0 to 68·5)	52·0 (38·6 to 68·5)	5·0 (4·5 to 5·7)	677·6 (613·1 to 744·9)	419·9 (362·7 to 479·1)	131·9 (114·6 to 151·1)	114·8 (85·3 to 151·1)	11·1 (9·9 to 12·6)
Gabon*	2033	316·1 (285·1 to 348·4)	196·8 (169·7 to 225·6)	60·5 (52·6 to 69·3)	53·8 (39·9 to 70·8)	5·1 (4·5 to 5·8)	697·5 (628·9 to 768·7)	434·2 (374·4 to 497·8)	133·4 (116·1 to 153·0)	118·7 (88·0 to 156·2)	11·2 (10·0 to 12·8)
Gabon*	2034	324·9 (293·0 to 359·0)	203·1 (174·3 to 233·1)	61·2 (53·2 to 70·1)	55·6 (41·2 to 73·1)	5·2 (4·6 to 5·9)	716·9 (646·5 to 792·0)	448·0 (384·5 to 514·4)	134·9 (117·4 to 154·7)	122·6 (90·9 to 161·3)	11·4 (10·2 to 13·0)
Gabon*	2035	333·8 (299·5 to 369·5)	209·3 (179·0 to 240·7)	61·8 (53·8 to 70·9)	57·4 (42·5 to 75·9)	5·2 (4·7 to 6·0)	736·4 (660·8 to 815·2)	461·8 (394·9 to 531·1)	136·4 (118·6 to 156·4)	126·7 (93·7 to 167·5)	11·6 (10·3 to 13·2)
Gabon*	2036	342·6 (306·8 to 381·2)	215·5 (184·2 to 248·6)	62·5 (54·3 to 71·7)	59·3 (43·6 to 78·7)	5·3 (4·7 to 6·1)	756·0 (676·9 to 841·0)	475·6 (406·5 to 548·4)	137·9 (119·8 to 158·2)	130·8 (96·2 to 173·7)	11·7 (10·4 to 13·5)
Gabon*	2037	351·4 (314·3 to 391·4)	221·6 (188·6 to 256·3)	63·2 (54·9 to 72·5)	61·2 (44·8 to 81·6)	5·4 (4·8 to 6·3)	775·3 (693·4 to 863·6)	488·9 (416·1 to 565·5)	139·4 (121·0 to 160·0)	135·0 (98·9 to 180·1)	12·0 (10·6 to 13·8)

Gabon*	2038	359·8 (321·5 to 402·0)	227·4 (192·8 to 263·1)	63·8 (55·4 to 73·2)	63·1 (46·1 to 84·6)	5·5 (4·9 to 6·5)	793·8 (709·5 to 887·0)	501·7 (425·3 to 580·6)	140·7 (122·2 to 161·4)	139·2 (101·7 to 186·7)	12·2 (10·8 to 14·3)
Gabon*	2039	368·5 (328·9 to 412·9)	233·3 (197·3 to 270·6)	64·4 (55·9 to 73·9)	65·1 (47·4 to 87·5)	5·7 (5·0 to 6·6)	813·0 (725·8 to 911·0)	514·8 (435·2 to 597·0)	142·0 (123·3 to 163·0)	143·6 (104·7 to 193·0)	12·5 (10·9 to 14·6)
Gabon*	2040	376·8 (334·9 to 422·9)	239·0 (201·4 to 277·7)	64·9 (56·3 to 74·5)	67·1 (48·7 to 89·8)	5·9 (5·1 to 6·9)	831·4 (738·9 to 933·0)	527·2 (444·4 to 612·8)	143·2 (124·2 to 164·4)	148·0 (107·4 to 198·0)	12·9 (11·2 to 15·3)
Gabon*	2041	384·9 (341·1 to 433·0)	244·4 (205·5 to 285·1)	65·4 (56·7 to 75·1)	69·1 (50·1 to 92·8)	6·0 (5·2 to 7·2)	849·3 (752·7 to 955·4)	539·2 (453·4 to 629·0)	144·4 (125·1 to 165·7)	152·4 (110·5 to 204·8)	13·3 (11·5 to 15·8)
Gabon*	2042	393·0 (347·8 to 442·4)	249·7 (209·5 to 293·0)	65·9 (57·1 to 75·7)	71·1 (51·4 to 95·7)	6·3 (5·4 to 7·5)	867·1 (767·3 to 976·2)	551·0 (462·3 to 646·6)	145·4 (125·9 to 166·9)	156·8 (113·5 to 211·0)	13·8 (11·9 to 16·5)
Gabon*	2043	400·7 (354·3 to 452·5)	254·8 (213·3 to 301·0)	66·4 (57·4 to 76·2)	73·1 (52·8 to 98·2)	6·5 (5·6 to 7·9)	884·2 (781·8 to 998·3)	562·2 (470·6 to 664·0)	146·4 (126·7 to 168·1)	161·3 (116·4 to 216·7)	14·3 (12·3 to 17·4)
Gabon*	2044	408·5 (360·1 to 462·4)	259·7 (217·0 to 307·0)	66·8 (57·8 to 76·7)	75·2 (54·2 to 101·4)	6·8 (5·8 to 8·3)	901·3 (794·5 to 1020·2)	573·1 (478·7 to 677·3)	147·4 (127·5 to 169·3)	165·9 (119·5 to 223·6)	14·9 (12·7 to 18·2)
Gabon*	2045	416·5 (366·7 to 473·2)	264·8 (220·7 to 316·5)	67·3 (58·2 to 77·3)	77·4 (55·6 to 104·6)	7·0 (5·9 to 8·7)	918·9 (809·1 to 1044·0)	584·3 (487·0 to 698·4)	148·5 (128·4 to 170·6)	170·7 (122·7 to 230·8)	15·5 (13·1 to 19·1)
Gabon*	2046	424·1 (373·0 to 483·7)	269·5 (224·2 to 322·8)	67·8 (58·6 to 77·8)	79·5 (57·0 to 108·0)	7·3 (6·1 to 9·0)	935·8 (823·0 to 1067·3)	594·7 (494·7 to 712·1)	149·5 (129·4 to 171·7)	175·5 (125·8 to 238·2)	16·1 (13·5 to 19·9)
Gabon*	2047	431·3 (377·6 to 493·0)	273·9 (227·4 to 329·8)	68·1 (59·0 to 78·3)	81·7 (58·4 to 111·6)	7·6 (6·3 to 9·6)	951·7 (833·1 to 1087·7)	604·3 (501·7 to 727·6)	150·3 (130·2 to 172·7)	180·2 (128·8 to 246·2)	16·9 (14·0 to 21·2)
Gabon*	2048	438·3 (383·8 to 503·4)	277·9 (230·3 to 336·0)	68·5 (59·3 to 78·7)	83·9 (59·6 to 114·8)	8·0 (6·7 to 10·1)	967·1 (846·7 to 1110·8)	613·2 (508·1 to 741·4)	151·2 (130·8 to 173·6)	185·0 (131·4 to 253·3)	17·7 (14·7 to 22·2)
Gabon*	2049	445·2 (389·6 to 512·2)	281·9 (233·1 to 342·6)	68·9 (59·6 to 79·1)	86·1 (60·6 to 118·8)	8·4 (6·9 to 10·8)	982·4 (859·5 to 1130·1)	621·9 (514·4 to 755·9)	151·9 (131·6 to 174·5)	189·9 (133·8 to 262·1)	18·6 (15·2 to 23·8)
Gabon*	2050	452·0 (395·2 to 521·5)	285·6 (235·8 to 350·2)	69·2 (60·0 to 79·5)	88·3 (62·1 to 122·3)	8·9 (7·2 to 11·5)	997·3 (871·9 to 1150·6)	630·1 (520·3 to 772·7)	152·7 (132·3 to 175·4)	194·9 (136·9 to 269·9)	19·6 (15·9 to 25·3)
UK	1995	2212·0 (2149·7 to 2270·4)	1757·0 (1707·3 to 1809·1)	370·1 (337·8 to 402·6)	84·9 (70·8 to 101·8)	0·0 (0·0 to 0·0)	2497·9 (2427·5 to 2563·8)	1984·1 (1927·9 to 2042·9)	417·9 (381·5 to 454·6)	95·9 (79·9 to 115·0)	0·0 (0·0 to 0·0)

UK	1996	2228·3 (2181·5 to 2280·1)	1746·0 (1708·5 to 1786·0)	379·9 (351·5 to 406·5)	102·5 (89·3 to 118·1)	0·0 (0·0 to 0·0)	2516·4 (2463·5 to 2574·8)	1971·6 (1929·4 to 2016·8)	429·0 (396·9 to 459·1)	115·8 (100·8 to 133·4)	0·0 (0·0 to 0·0)
UK	1997	2279·7 (2235·0 to 2327·4)	1751·6 (1712·4 to 1791·5)	395·0 (368·3 to 420·1)	133·0 (118·6 to 148·0)	0·0 (0·0 to 0·0)	2574·4 (2523·9 to 2628·3)	1978·0 (1933·7 to 2023·0)	446·1 (415·9 to 474·4)	150·2 (133·9 to 167·1)	0·0 (0·0 to 0·0)
UK	1998	2368·7 (2320·8 to 2417·6)	1809·2 (1769·5 to 1847·1)	411·6 (384·5 to 437·3)	147·9 (134·5 to 161·2)	0·0 (0·0 to 0·0)	2674·9 (2620·7 to 2730·1)	2043·1 (1998·2 to 2085·8)	464·8 (434·2 to 493·9)	167·0 (151·9 to 182·0)	0·0 (0·0 to 0·0)
UK	1999	2487·0 (2440·6 to 2537·2)	1902·6 (1863·2 to 1942·0)	430·2 (404·6 to 455·6)	154·3 (144·3 to 164·0)	0·0 (0·0 to 0·0)	2808·5 (2756·1 to 2865·2)	2148·5 (2104·1 to 2193·0)	485·8 (456·9 to 514·5)	174·2 (162·9 to 185·3)	0·0 (0·0 to 0·0)
UK	2000	2616·9 (2575·0 to 2662·3)	2007·1 (1968·5 to 2044·7)	452·3 (429·8 to 475·0)	157·5 (151·6 to 163·3)	0·0 (0·0 to 0·0)	2955·1 (2907·8 to 3006·4)	2266·5 (2223·0 to 2308·9)	510·8 (485·4 to 536·4)	177·8 (171·2 to 184·4)	0·0 (0·0 to 0·0)
UK	2001	2791·3 (2751·8 to 2836·9)	2147·5 (2111·8 to 2182·5)	477·4 (454·3 to 501·0)	166·4 (161·5 to 171·5)	0·0 (0·0 to 0·0)	3152·1 (3107·5 to 3203·6)	2425·1 (2384·7 to 2464·5)	539·1 (513·0 to 565·8)	188·0 (182·4 to 193·7)	0·0 (0·0 to 0·0)
UK	2002	2966·0 (2921·4 to 3007·9)	2304·7 (2265·8 to 2339·7)	491·3 (470·8 to 515·8)	170·0 (165·0 to 174·9)	0·0 (0·0 to 0·0)	3349·3 (3299·0 to 3396·7)	2602·6 (2558·6 to 2642·1)	554·8 (531·6 to 582·5)	191·9 (186·3 to 197·5)	0·0 (0·0 to 0·0)
UK	2003	3141·1 (3099·6 to 3183·7)	2479·8 (2443·1 to 2517·6)	491·9 (468·9 to 515·3)	169·4 (165·0 to 174·2)	0·0 (0·0 to 0·0)	3547·1 (3500·2 to 3595·2)	2800·3 (2758·9 to 2843·0)	555·5 (529·5 to 581·9)	191·3 (186·3 to 196·8)	0·0 (0·0 to 0·0)
UK	2004	3292·3 (3250·6 to 3335·2)	2642·3 (2605·7 to 2679·5)	480·7 (457·7 to 504·1)	169·3 (165·0 to 174·2)	0·0 (0·0 to 0·0)	3717·9 (3670·8 to 3766·3)	2983·8 (2942·5 to 3025·8)	542·8 (516·9 to 569·2)	191·2 (186·3 to 196·7)	0·0 (0·0 to 0·0)
UK	2005	3435·9 (3391·6 to 3483·1)	2789·4 (2752·8 to 2830·1)	470·2 (447·3 to 492·8)	176·4 (171·5 to 181·0)	0·0 (0·0 to 0·0)	3880·0 (3829·9 to 3933·3)	3149·9 (3108·6 to 3195·9)	530·9 (505·1 to 556·5)	199·2 (193·7 to 204·4)	0·0 (0·0 to 0·0)
UK	2006	3545·6 (3500·9 to 3590·6)	2891·6 (2855·9 to 2928·6)	472·2 (449·4 to 496·1)	181·8 (177·0 to 186·9)	0·0 (0·0 to 0·0)	4003·9 (3953·4 to 4054·7)	3265·4 (3225·0 to 3307·2)	533·3 (507·4 to 560·3)	205·3 (199·8 to 211·0)	0·0 (0·0 to 0·0)
UK	2007	3632·6 (3588·8 to 3679·6)	2957·3 (2917·9 to 2997·3)	488·6 (465·3 to 512·1)	186·7 (182·0 to 191·8)	0·0 (0·0 to 0·0)	4102·1 (4052·7 to 4155·2)	3339·6 (3295·1 to 3384·7)	551·7 (525·4 to 578·3)	210·8 (205·5 to 216·6)	0·0 (0·0 to 0·0)
UK	2008	3784·4 (3737·2 to 3833·3)	3099·5 (3059·8 to 3143·8)	493·1 (471·2 to 516·4)	191·8 (187·1 to 196·8)	0·0 (0·0 to 0·0)	4273·6 (4220·3 to 4328·8)	3500·1 (3455·3 to 3550·2)	556·8 (532·1 to 583·2)	216·6 (211·3 to 222·3)	0·0 (0·0 to 0·0)
UK	2009	3878·2 (3834·3 to 3924·1)	3198·2 (3159·4 to 3235·3)	493·0 (470·1 to 515·6)	187·0 (182·1 to 192·1)	0·0 (0·0 to 0·0)	4379·5 (4329·9 to 4431·3)	3611·6 (3567·7 to 3653·4)	556·7 (530·9 to 582·3)	211·2 (205·7 to 216·9)	0·0 (0·0 to 0·0)

UK	2010	3952·4 (3907·8 to 3999·7)	3259·6 (3220·1 to 3298·8)	506·5 (483·6 to 529·9)	186·3 (181·4 to 191·4)	0·0 (0·0 to 0·0)	4463·3 (4412·8 to 4516·7)	3681·0 (3636·3 to 3725·2)	572·0 (546·1 to 598·4)	210·3 (204·9 to 216·2)	0·0 (0·0 to 0·0)
UK	2011	3944·5 (3898·3 to 3991·1)	3230·6 (3190·1 to 3269·5)	526·1 (502·3 to 550·0)	187·8 (183·0 to 192·5)	0·0 (0·0 to 0·0)	4454·3 (4402·2 to 4507·0)	3648·2 (3602·4 to 3692·1)	594·1 (567·3 to 621·1)	212·1 (206·7 to 217·4)	0·0 (0·0 to 0·0)
UK	2012	4017·9 (3974·8 to 4064·9)	3263·1 (3223·3 to 3301·1)	562·8 (539·5 to 586·8)	191·9 (186·9 to 197·0)	0·0 (0·0 to 0·0)	4537·2 (4488·6 to 4590·3)	3684·9 (3639·9 to 3727·8)	635·6 (609·3 to 662·7)	216·8 (211·1 to 222·5)	0·0 (0·0 to 0·0)
UK	2013	4024·5 (3977·3 to 4068·8)	3232·4 (3191·6 to 3268·2)	598·4 (575·0 to 622·4)	193·6 (188·5 to 198·7)	0·0 (0·0 to 0·0)	4544·6 (4491·3 to 4594·7)	3650·2 (3604·1 to 3690·6)	675·8 (649·3 to 702·8)	218·7 (212·9 to 224·4)	0·0 (0·0 to 0·0)
UK	2014	4097·2 (4049·6 to 4143·8)	3282·1 (3243·4 to 3319·5)	620·7 (598·2 to 644·4)	194·5 (189·2 to 199·4)	0·0 (0·0 to 0·0)	4626·8 (4573·0 to 4679·4)	3706·3 (3662·6 to 3748·5)	700·9 (675·5 to 727·6)	219·6 (213·7 to 225·1)	0·0 (0·0 to 0·0)
UK	2015	4167·2 (4121·2 to 4214·7)	3342·0 (3302·0 to 3384·8)	633·8 (609·1 to 657·0)	191·4 (186·3 to 196·3)	0·0 (0·0 to 0·0)	4705·8 (4653·9 to 4759·5)	3773·9 (3728·8 to 3822·3)	715·7 (687·8 to 741·9)	216·1 (210·4 to 221·7)	0·0 (0·0 to 0·0)
UK	2016	4192·2 (4147·4 to 4240·2)	3360·1 (3317·7 to 3399·0)	648·3 (624·5 to 671·9)	183·8 (179·3 to 188·9)	0·0 (0·0 to 0·0)	4734·1 (4683·4 to 4788·3)	3794·4 (3746·5 to 3838·4)	732·1 (705·2 to 758·8)	207·5 (202·4 to 213·3)	0·0 (0·0 to 0·0)
UK	2017	4244·4 (4197·1 to 4294·6)	3376·1 (3332·8 to 3418·8)	677·6 (653·8 to 703·0)	190·6 (185·5 to 195·4)	0·0 (0·0 to 0·0)	4792·9 (4739·5 to 4849·7)	3812·5 (3763·6 to 3860·7)	765·2 (738·3 to 793·8)	215·2 (209·5 to 220·7)	0·0 (0·0 to 0·0)
UK	2018	4326·0 (4268·5 to 4385·7)	3411·4 (3361·5 to 3459·3)	714·8 (682·2 to 748·1)	199·9 (193·8 to 206·7)	0·0 (0·0 to 0·0)	4885·2 (4820·2 to 4952·5)	3852·3 (3796·0 to 3906·5)	807·1 (770·4 to 844·8)	225·8 (218·8 to 233·4)	0·0 (0·0 to 0·0)
UK	2019	4392·5 (4328·5 to 4457·1)	3463·1 (3404·9 to 3518·6)	723·8 (690·7 to 757·6)	205·5 (199·1 to 213·0)	0·0 (0·0 to 0·0)	4960·2 (4888·0 to 5033·2)	3910·7 (3845·0 to 3973·3)	817·4 (780·0 to 855·5)	232·1 (224·8 to 240·5)	0·0 (0·0 to 0·0)
UK	2020	5157·5 (5065·5 to 5243·3)	4307·5 (4217·6 to 4385·0)	658·9 (628·7 to 689·8)	191·0 (184·2 to 198·8)	0·0 (0·0 to 0·0)	5824·1 (5720·2 to 5921·1)	4864·3 (4762·7 to 4951·8)	744·1 (710·0 to 779·0)	215·7 (208·0 to 224·5)	0·0 (0·0 to 0·0)
UK	2021	4902·9 (4815·7 to 4984·3)	4093·0 (4007·6 to 4166·7)	624·7 (595·7 to 654·0)	185·2 (178·7 to 192·7)	0·0 (0·0 to 0·0)	5536·6 (5438·1 to 5628·5)	4622·0 (4525·5 to 4705·2)	705·4 (672·7 to 738·5)	209·2 (201·7 to 217·7)	0·0 (0·0 to 0·0)
UK	2022	3903·9 (3812·8 to 3993·8)	3086·8 (2997·7 to 3172·5)	626·9 (597·9 to 656·3)	190·2 (183·4 to 198·0)	0·0 (0·0 to 0·0)	4408·5 (4305·6 to 4510·0)	3485·8 (3385·2 to 3582·5)	707·9 (675·2 to 741·1)	214·8 (207·1 to 223·6)	0·0 (0·0 to 0·0)
UK	2023	3949·4 (3842·0 to 4059·1)	3125·5 (3019·6 to 3227·5)	628·7 (599·7 to 658·4)	195·2 (187·7 to 203·7)	0·0 (0·0 to 0·0)	4459·8 (4338·6 to 4583·8)	3529·4 (3409·8 to 3644·7)	709·9 (677·2 to 743·5)	220·4 (211·9 to 230·0)	0·0 (0·0 to 0·0)

UK	2024	3996·9 (3882·0 to 4118·1)	3166·1 (3052·3 to 3278·1)	630·5 (601·5 to 660·2)	200·3 (191·3 to 209·4)	0·0 (0·0 to 0·0)	4513·5 (4383·8 to 4650·3)	3575·3 (3446·8 to 3701·7)	712·0 (679·3 to 745·5)	226·2 (216·1 to 236·5)	0·0 (0·0 to 0·0)
UK	2025	4031·5 (3910·8 to 4154·8)	3197·7 (3085·3 to 3320·3)	629·2 (600·0 to 658·8)	204·6 (193·9 to 214·9)	0·0 (0·0 to 0·0)	4552·5 (4416·2 to 4691·8)	3611·0 (3484·0 to 3749·4)	710·6 (677·6 to 743·9)	231·0 (219·0 to 242·7)	0·0 (0·0 to 0·0)
UK	2026	4067·7 (3942·0 to 4197·1)	3231·1 (3113·5 to 3355·7)	627·7 (599·0 to 657·2)	208·9 (197·4 to 220·2)	0·0 (0·0 to 0·0)	4593·5 (4451·5 to 4739·5)	3648·8 (3515·9 to 3789·5)	708·8 (676·5 to 742·1)	235·9 (222·9 to 248·6)	0·0 (0·0 to 0·0)
UK	2027	4106·3 (3974·7 to 4243·9)	3266·4 (3145·7 to 3399·9)	626·4 (598·2 to 655·7)	213·5 (201·2 to 225·6)	0·0 (0·0 to 0·0)	4637·1 (4488·4 to 4792·4)	3688·6 (3552·3 to 3839·3)	707·3 (675·5 to 740·4)	241·1 (227·2 to 254·8)	0·0 (0·0 to 0·0)
UK	2028	4148·8 (4006·8 to 4294·4)	3305·2 (3173·7 to 3450·9)	625·3 (597·3 to 655·0)	218·3 (204·5 to 232·1)	0·0 (0·0 to 0·0)	4685·1 (4524·7 to 4849·4)	3732·4 (3583·9 to 3897·0)	706·1 (674·5 to 739·6)	246·6 (230·9 to 262·1)	0·0 (0·0 to 0·0)
UK	2029	4193·2 (4042·3 to 4348·9)	3346·0 (3201·9 to 3502·1)	624·3 (596·4 to 654·2)	222·9 (208·7 to 237·2)	0·0 (0·0 to 0·0)	4735·2 (4564·7 to 4911·0)	3778·4 (3615·7 to 3954·7)	705·0 (673·5 to 738·8)	251·7 (235·7 to 267·8)	0·0 (0·0 to 0·0)
UK	2030	4240·1 (4079·7 to 4409·4)	3388·8 (3233·4 to 3560·6)	623·6 (595·7 to 653·1)	227·7 (212·9 to 242·5)	0·0 (0·0 to 0·0)	4788·1 (4607·0 to 4979·3)	3826·8 (3651·4 to 4020·8)	704·2 (672·7 to 737·5)	257·1 (240·4 to 273·8)	0·0 (0·0 to 0·0)
UK	2031	4293·1 (4121·3 to 4484·6)	3436·4 (3272·3 to 3624·6)	623·8 (596·0 to 653·3)	232·9 (217·5 to 248·6)	0·0 (0·0 to 0·0)	4848·0 (4654·0 to 5064·3)	3880·5 (3695·3 to 4093·1)	704·4 (673·0 to 737·7)	263·0 (245·6 to 280·8)	0·0 (0·0 to 0·0)
UK	2032	4349·5 (4165·1 to 4558·0)	3486·2 (3305·6 to 3689·6)	624·8 (597·1 to 654·4)	238·6 (222·5 to 255·0)	0·0 (0·0 to 0·0)	4911·7 (4703·5 to 5147·2)	3936·8 (3732·9 to 4166·5)	705·5 (674·3 to 739·0)	269·4 (251·2 to 287·9)	0·0 (0·0 to 0·0)
UK	2033	4403·5 (4200·9 to 4626·5)	3533·9 (3335·7 to 3754·0)	625·4 (597·7 to 655·2)	244·2 (227·1 to 261·9)	0·0 (0·0 to 0·0)	4972·6 (4743·9 to 5224·5)	3990·6 (3766·9 to 4239·2)	706·2 (675·0 to 739·9)	275·8 (256·5 to 295·8)	0·0 (0·0 to 0·0)
UK	2034	4459·9 (4238·8 to 4695·1)	3583·3 (3372·3 to 3819·5)	626·4 (598·5 to 656·6)	250·1 (231·8 to 269·6)	0·0 (0·0 to 0·0)	5036·3 (4786·7 to 5302·0)	4046·4 (3808·1 to 4313·2)	707·4 (675·8 to 741·5)	282·5 (261·7 to 304·5)	0·0 (0·0 to 0·0)
UK	2035	4514·4 (4277·4 to 4767·9)	3630·6 (3402·6 to 3876·9)	627·6 (599·7 to 658·1)	256·2 (235·8 to 277·7)	0·0 (0·0 to 0·0)	5097·9 (4830·2 to 5384·2)	4099·8 (3842·4 to 4377·9)	708·7 (677·2 to 743·1)	289·4 (266·3 to 313·6)	0·0 (0·0 to 0·0)
UK	2036	4569·4 (4319·2 to 4834·1)	3677·5 (3430·5 to 3944·5)	629·2 (601·3 to 660·0)	262·7 (240·3 to 287·0)	0·0 (0·0 to 0·0)	5160·0 (4877·5 to 5458·9)	4152·9 (3873·9 to 4454·3)	710·5 (679·0 to 745·3)	296·6 (271·4 to 324·1)	0·0 (0·0 to 0·0)
UK	2037	4622·9 (4355·1 to 4898·3)	3722·5 (3460·1 to 4002·9)	631·1 (603·2 to 661·8)	269·3 (244·9 to 296·0)	0·0 (0·0 to 0·0)	5220·5 (4918·0 to 5531·4)	4203·6 (3907·4 to 4520·3)	712·7 (681·1 to 747·3)	304·1 (276·6 to 334·3)	0·0 (0·0 to 0·0)

UK	2038	4676·6 (4396·5 to 4968·9)	3766·4 (3488·2 to 4046·3)	633·8 (605·7 to 664·6)	276·4 (249·8 to 305·8)	0·0 (0·0 to 0·0)	5281·1 (4964·7 to 5611·2)	4253·2 (3939·0 to 4569·3)	715·7 (684·0 to 750·6)	312·2 (282·1 to 345·3)	0·0 (0·0 to 0·0)
UK	2039	4730·9 (4430·8 to 5032·7)	3809·9 (3523·3 to 4112·9)	637·0 (608·8 to 668·0)	283·9 (254·8 to 315·5)	0·0 (0·0 to 0·0)	5342·3 (5003·5 to 5683·1)	4302·4 (3978·7 to 4644·5)	719·3 (687·5 to 754·4)	320·6 (287·7 to 356·3)	0·0 (0·0 to 0·0)
UK	2040	4784·5 (4474·9 to 5107·4)	3852·3 (3551·2 to 4181·3)	640·5 (612·0 to 671·7)	291·7 (260·2 to 327·2)	0·0 (0·0 to 0·0)	5402·9 (5053·2 to 5767·5)	4350·2 (4010·2 to 4721·7)	723·2 (691·1 to 758·6)	329·4 (293·8 to 369·5)	0·0 (0·0 to 0·0)
UK	2041	4837·8 (4515·2 to 5175·6)	3894·1 (3585·0 to 4238·4)	644·0 (615·2 to 675·6)	299·7 (265·4 to 339·6)	0·0 (0·0 to 0·0)	5463·1 (5098·8 to 5844·5)	4397·4 (4048·3 to 4786·3)	727·2 (694·7 to 763·0)	338·5 (299·7 to 383·5)	0·0 (0·0 to 0·0)
UK	2042	4890·2 (4554·5 to 5241·2)	3935·2 (3606·5 to 4289·6)	647·1 (618·2 to 678·9)	307·9 (270·6 to 350·8)	0·0 (0·0 to 0·0)	5522·3 (5143·2 to 5918·7)	4443·9 (4072·6 to 4844·0)	730·8 (698·1 to 766·6)	347·6 (305·6 to 396·1)	0·0 (0·0 to 0·0)
UK	2043	4942·7 (4595·0 to 5303·4)	3976·5 (3640·1 to 4347·7)	650·1 (621·1 to 682·1)	316·1 (275·1 to 363·3)	0·0 (0·0 to 0·0)	5581·5 (5188·9 to 5988·9)	4490·5 (4110·5 to 4909·7)	734·1 (701·4 to 770·3)	356·9 (310·7 to 410·2)	0·0 (0·0 to 0·0)
UK	2044	4994·0 (4635·9 to 5366·3)	4016·5 (3667·6 to 4378·9)	653·0 (623·8 to 684·9)	324·5 (280·6 to 375·6)	0·0 (0·0 to 0·0)	5639·5 (5235·1 to 6059·9)	4535·6 (4141·7 to 4944·9)	737·4 (704·4 to 773·4)	366·5 (316·9 to 424·2)	0·0 (0·0 to 0·0)
UK	2045	5044·4 (4667·3 to 5429·3)	4055·6 (3687·2 to 4432·7)	655·7 (626·2 to 688·3)	333·1 (285·6 to 387·0)	0·0 (0·0 to 0·0)	5696·4 (5270·5 to 6131·1)	4579·8 (4163·8 to 5005·6)	740·5 (707·1 to 777·3)	376·1 (322·5 to 437·0)	0·0 (0·0 to 0·0)
UK	2046	5093·3 (4706·2 to 5483·5)	4093·2 (3722·0 to 4479·8)	658·3 (628·6 to 691·1)	341·8 (291·3 to 401·1)	0·0 (0·0 to 0·0)	5751·6 (5314·5 to 6192·2)	4622·3 (4203·1 to 5058·8)	743·4 (709·8 to 780·5)	386·0 (329·0 to 453·0)	0·0 (0·0 to 0·0)
UK	2047	5140·6 (4747·1 to 5545·8)	4129·3 (3757·7 to 4521·3)	660·7 (630·6 to 693·8)	350·7 (296·6 to 414·1)	0·0 (0·0 to 0·0)	5805·0 (5360·7 to 6262·7)	4663·0 (4243·4 to 5105·6)	746·1 (712·1 to 783·5)	396·0 (335·0 to 467·6)	0·0 (0·0 to 0·0)
UK	2048	5186·5 (4784·9 to 5592·8)	4163·9 (3782·1 to 4563·8)	662·9 (632·3 to 696·2)	359·7 (301·9 to 427·5)	0·0 (0·0 to 0·0)	5856·9 (5403·4 to 6315·6)	4702·1 (4270·9 to 5153·7)	748·6 (714·0 to 786·1)	406·2 (340·9 to 482·8)	0·0 (0·0 to 0·0)
UK	2049	5233·3 (4822·2 to 5654·3)	4199·2 (3800·8 to 4608·7)	665·2 (634·1 to 698·6)	368·9 (307·0 to 441·8)	0·0 (0·0 to 0·0)	5909·7 (5445·5 to 6385·1)	4741·9 (4292·0 to 5204·4)	751·1 (716·1 to 788·9)	416·6 (346·7 to 499·0)	0·0 (0·0 to 0·0)
UK	2050	5279·5 (4863·1 to 5701·7)	4233·7 (3833·3 to 4646·7)	667·4 (636·2 to 701·0)	378·4 (311·9 to 457·5)	0·0 (0·0 to 0·0)	5961·8 (5491·7 to 6438·7)	4780·9 (4328·7 to 5247·3)	753·6 (718·5 to 791·7)	427·3 (352·3 to 516·6)	0·0 (0·0 to 0·0)
Georgia*	1995	53·8 (45·3 to 64·1)	3·0 (2·5 to 3·6)	50·7 (41·8 to 60·9)	0·0 (0·0 to 0·0)	0·2 (0·2 to 0·2)	185·0 (155·5 to 220·3)	10·4 (8·5 to 12·5)	174·0 (143·5 to 209·3)	0·0 (0·0 to 0·0)	0·5 (0·5 to 0·5)

Georgia*	1996	68·8 (58·6 to 80·4)	6·0 (4·9 to 7·2)	62·1 (52·1 to 73·9)	0·0 (0·0 to 0·0)	0·7 (0·7 to 0·7)	236·2 (201·2 to 276·4)	20·6 (16·9 to 24·7)	213·4 (178·9 to 253·9)	0·0 (0·0 to 0·0)	2·2 (2·2 to 2·2)
Georgia*	1997	80·9 (70·3 to 93·1)	9·7 (8·0 to 11·6)	70·3 (59·5 to 82·7)	0·0 (0·0 to 0·0)	0·9 (0·9 to 0·9)	278·0 (241·4 to 320·0)	33·2 (27·6 to 39·8)	241·7 (204·4 to 284·2)	0·0 (0·0 to 0·0)	3·1 (3·1 to 3·1)
Georgia*	1998	79·3 (70·6 to 90·3)	10·5 (8·7 to 12·5)	67·1 (58·1 to 77·9)	0·0 (0·0 to 0·0)	1·8 (1·8 to 1·8)	272·4 (242·4 to 310·3)	35·9 (29·7 to 42·8)	230·4 (199·6 to 267·8)	0·0 (0·0 to 0·0)	6·1 (6·1 to 6·1)
Georgia*	1999	84·4 (76·0 to 93·8)	11·3 (9·3 to 13·5)	68·7 (60·4 to 78·4)	0·0 (0·0 to 0·0)	4·3 (4·3 to 4·3)	289·8 (261·2 to 322·4)	38·9 (32·1 to 46·5)	236·0 (207·6 to 269·2)	0·0 (0·0 to 0·0)	14·9 (14·9 to 14·9)
Georgia*	2000	89·8 (81·6 to 99·1)	11·8 (9·7 to 14·0)	73·9 (66·1 to 82·9)	0·1 (0·0 to 0·1)	4·1 (4·1 to 4·1)	308·6 (280·3 to 340·4)	40·5 (33·2 to 48·2)	253·8 (227·2 to 284·8)	0·2 (0·1 to 0·3)	14·1 (14·1 to 14·1)
Georgia*	2001	100·7 (92·5 to 110·2)	14·0 (11·5 to 16·5)	82·1 (74·5 to 91·3)	0·3 (0·2 to 0·4)	4·3 (4·3 to 4·3)	346·0 (317·8 to 378·7)	48·1 (39·5 to 56·5)	282·1 (256·0 to 313·6)	1·0 (0·7 to 1·4)	14·9 (14·9 to 14·9)
Georgia*	2002	112·3 (103·6 to 121·3)	15·5 (13·0 to 18·2)	91·4 (83·0 to 100·2)	0·6 (0·4 to 0·8)	4·7 (4·7 to 4·7)	385·7 (355·9 to 416·8)	53·3 (44·6 to 62·6)	314·1 (285·2 to 344·2)	2·0 (1·4 to 2·9)	16·3 (16·3 to 16·3)
Georgia*	2003	125·2 (115·8 to 134·3)	17·8 (15·0 to 20·8)	103·8 (94·9 to 112·5)	0·9 (0·7 to 1·3)	2·6 (2·6 to 2·6)	430·2 (398·0 to 461·5)	61·3 (51·4 to 71·4)	356·7 (326·0 to 386·3)	3·3 (2·2 to 4·6)	9·0 (9·0 to 9·0)
Georgia*	2004	142·4 (132·9 to 152·3)	21·1 (18·0 to 24·3)	116·4 (107·8 to 125·8)	1·5 (1·0 to 2·2)	3·4 (3·4 to 3·4)	489·2 (456·4 to 523·1)	72·5 (61·9 to 83·4)	399·8 (370·4 to 432·1)	5·1 (3·6 to 7·4)	11·8 (11·8 to 11·8)
Georgia*	2005	163·3 (153·7 to 173·5)	24·9 (21·6 to 28·5)	128·0 (118·3 to 137·7)	2·2 (1·5 to 3·1)	8·3 (8·3 to 8·3)	561·1 (527·9 to 596·1)	85·5 (74·3 to 98·0)	439·6 (406·4 to 473·2)	7·4 (5·1 to 10·6)	28·6 (28·6 to 28·6)
Georgia*	2006	176·0 (166·3 to 186·5)	28·0 (24·6 to 31·8)	134·9 (125·6 to 144·6)	3·1 (2·1 to 4·4)	10·0 (10·0 to 10·0)	604·5 (571·4 to 640·8)	96·0 (84·4 to 109·3)	463·5 (431·6 to 496·7)	10·6 (7·3 to 15·0)	34·4 (34·4 to 34·4)
Georgia*	2007	200·7 (189·7 to 212·4)	35·3 (31·1 to 40·0)	149·3 (139·5 to 159·8)	5·4 (3·7 to 7·6)	10·7 (10·7 to 10·7)	689·5 (651·8 to 729·7)	121·4 (107·0 to 137·3)	513·1 (479·1 to 549·0)	18·4 (12·8 to 25·9)	36·6 (36·6 to 36·6)
Georgia*	2008	221·1 (209·0 to 232·7)	43·2 (38·7 to 48·0)	162·4 (152·1 to 172·6)	8·4 (5·8 to 11·7)	7·1 (7·1 to 7·1)	759·8 (717·9 to 799·6)	148·4 (133·0 to 165·0)	558·1 (522·6 to 593·0)	28·7 (20·0 to 40·2)	24·6 (24·6 to 24·6)
Georgia*	2009	245·2 (232·6 to 257·4)	49·3 (44·6 to 54·5)	175·7 (165·0 to 187·1)	9·0 (6·3 to 12·6)	11·1 (11·1 to 11·1)	842·3 (799·2 to 884·3)	169·5 (153·4 to 187·4)	603·7 (566·7 to 642·8)	31·0 (21·8 to 43·2)	38·2 (38·2 to 38·2)

Georgia*	2010	252·7 (240·6 to 265·4)	50·8 (46·3 to 55·6)	183·8 (173·7 to 194·6)	7·9 (5·6 to 11·1)	10·2 (10·2 to 10·2)	868·1 (826·7 to 911·6)	174·5 (158·9 to 191·1)	631·5 (596·7 to 668·6)	27·2 (19·3 to 38·0)	34·9 (34·9 to 34·9)
Georgia*	2011	256·9 (245·7 to 268·7)	50·5 (46·3 to 55·2)	186·6 (176·9 to 197·5)	8·6 (6·1 to 11·8)	11·2 (11·2 to 11·2)	882·8 (844·2 to 923·0)	173·4 (159·0 to 189·6)	641·1 (607·8 to 678·4)	29·7 (21·1 to 40·7)	38·6 (38·6 to 38·6)
Georgia*	2012	273·3 (261·1 to 285·5)	58·2 (53·5 to 63·2)	193·4 (183·3 to 203·5)	10·0 (7·2 to 13·8)	11·7 (11·7 to 11·7)	939·0 (896·9 to 980·7)	199·9 (183·9 to 217·0)	664·3 (629·8 to 699·3)	34·4 (24·7 to 47·5)	40·4 (40·4 to 40·4)
Georgia*	2013	275·8 (263·9 to 288·2)	67·3 (62·2 to 72·6)	190·6 (180·7 to 200·6)	11·4 (8·2 to 15·8)	6·5 (6·5 to 6·5)	947·5 (906·8 to 990·1)	231·1 (213·8 to 249·4)	654·8 (620·6 to 689·1)	39·2 (28·1 to 54·3)	22·4 (22·4 to 22·4)
Georgia*	2014	287·6 (275·2 to 299·9)	82·3 (76·8 to 88·3)	186·6 (176·0 to 196·7)	12·7 (9·0 to 17·4)	6·0 (6·0 to 6·0)	988·0 (945·4 to 1030·3)	282·6 (263·8 to 303·4)	641·2 (604·6 to 675·9)	43·5 (30·9 to 59·9)	20·6 (20·6 to 20·6)
Georgia*	2015	295·1 (282·4 to 308·5)	96·4 (90·8 to 102·6)	175·6 (166·1 to 185·5)	14·5 (10·4 to 19·9)	8·6 (8·6 to 8·6)	1013·8 (970·1 to 1059·8)	331·2 (311·9 to 352·4)	603·2 (570·5 to 637·3)	50·0 (35·8 to 68·3)	29·5 (29·5 to 29·5)
Georgia*	2016	301·5 (288·8 to 315·3)	109·7 (103·9 to 115·9)	169·5 (159·6 to 179·6)	17·7 (12·8 to 24·1)	4·5 (4·5 to 4·5)	1035·7 (992·0 to 1083·1)	376·9 (356·9 to 398·0)	582·5 (548·5 to 617·1)	61·0 (43·9 to 82·9)	15·4 (15·4 to 15·4)
Georgia*	2017	305·2 (291·9 to 320·7)	116·4 (109·9 to 123·0)	161·4 (150·8 to 172·5)	20·5 (15·1 to 28·3)	6·8 (6·8 to 6·8)	1048·4 (1002·8 to 1101·8)	399·8 (377·7 to 422·5)	554·6 (517·9 to 592·7)	70·5 (51·8 to 97·1)	23·4 (23·4 to 23·4)
Georgia*	2018	313·7 (296·2 to 331·6)	123·3 (114·6 to 131·8)	156·8 (143·4 to 170·9)	29·7 (21·9 to 40·9)	3·9 (3·9 to 3·9)	1077·7 (1017·5 to 1139·4)	423·6 (393·9 to 452·8)	538·8 (492·8 to 587·1)	102·0 (75·2 to 140·4)	13·2 (13·2 to 13·2)
Georgia*	2019	329·7 (310·5 to 349·6)	132·8 (120·8 to 144·4)	161·5 (147·8 to 176·2)	31·7 (23·3 to 43·6)	3·7 (3·7 to 3·8)	1132·7 (1066·9 to 1201·2)	456·1 (415·2 to 496·2)	554·7 (507·8 to 605·3)	109·0 (80·2 to 149·7)	12·9 (12·7 to 13·1)
Georgia*	2020	382·9 (363·6 to 402·7)	187·8 (175·9 to 199·5)	158·5 (144·9 to 172·8)	32·0 (23·6 to 44·0)	4·5 (4·3 to 4·7)	1315·4 (1249·3 to 1383·3)	645·4 (604·3 to 685·5)	544·7 (497·9 to 593·6)	109·9 (81·1 to 151·1)	15·5 (14·9 to 16·1)
Georgia*	2021	339·4 (318·2 to 361·9)	146·8 (132·4 to 162·3)	156·1 (142·7 to 170·1)	32·3 (23·8 to 44·5)	4·2 (3·9 to 4·5)	1165·9 (1093·1 to 1243·3)	504·4 (454·8 to 557·6)	536·3 (490·2 to 584·3)	110·8 (81·8 to 152·8)	14·3 (13·3 to 15·3)
Georgia*	2022	324·4 (301·7 to 348·5)	132·3 (115·1 to 150·7)	155·8 (142·3 to 169·7)	33·0 (24·3 to 45·3)	3·4 (3·1 to 3·7)	1114·6 (1036·5 to 1197·2)	454·5 (395·5 to 517·9)	535·1 (488·8 to 583·1)	113·2 (83·4 to 155·5)	11·7 (10·7 to 12·7)
Georgia*	2023	326·0 (302·8 to 351·3)	133·3 (115·2 to 152·9)	155·3 (141·9 to 169·3)	33·7 (24·8 to 46·2)	3·7 (3·4 to 4·0)	1120·0 (1040·3 to 1207·0)	458·0 (395·7 to 525·1)	533·6 (487·4 to 581·5)	115·8 (85·2 to 158·8)	12·6 (11·6 to 13·7)

Georgia*	2024	327·2 (302·7 to 354·3)	134·0 (114·6 to 154·9)	154·8 (141·4 to 168·6)	34·4 (25·3 to 47·1)	4·0 (3·7 to 4·3)	1124·0 (1040·0 to 1217·1)	460·4 (393·7 to 532·1)	531·8 (485·7 to 579·2)	118·2 (86·8 to 161·9)	13·6 (12·6 to 14·8)
Georgia*	2025	329·5 (303·7 to 359·3)	135·7 (114·8 to 160·0)	154·4 (141·1 to 168·2)	35·2 (25·8 to 48·2)	4·3 (4·0 to 4·7)	1132·2 (1043·4 to 1234·2)	466·2 (394·5 to 549·6)	530·6 (484·6 to 577·8)	120·8 (88·8 to 165·4)	14·7 (13·6 to 16·0)
Georgia*	2026	331·9 (304·3 to 364·8)	137·1 (113·4 to 163·9)	154·3 (141·0 to 168·1)	36·0 (26·3 to 49·1)	4·6 (4·2 to 5·0)	1140·1 (1045·5 to 1253·2)	470·9 (389·5 to 563·1)	530·0 (484·4 to 577·5)	123·5 (90·4 to 168·5)	15·7 (14·5 to 17·1)
Georgia*	2027	334·3 (304·7 to 369·0)	138·3 (112·7 to 167·3)	154·3 (141·1 to 168·1)	36·8 (26·9 to 50·1)	4·8 (4·5 to 5·3)	1148·5 (1046·7 to 1267·9)	475·1 (387·3 to 574·7)	530·3 (484·7 to 577·4)	126·5 (92·4 to 172·0)	16·6 (15·4 to 18·1)
Georgia*	2028	337·5 (306·7 to 373·4)	140·1 (113·8 to 170·6)	154·5 (141·2 to 168·3)	37·8 (27·6 to 51·6)	5·1 (4·7 to 5·6)	1159·5 (1053·6 to 1282·7)	481·2 (390·9 to 586·1)	530·9 (485·0 to 578·4)	129·9 (94·9 to 177·2)	17·5 (16·1 to 19·1)
Georgia*	2029	343·2 (311·1 to 381·5)	144·1 (116·4 to 177·7)	154·9 (141·5 to 168·7)	38·8 (28·4 to 53·2)	5·3 (4·9 to 5·8)	1179·1 (1068·8 to 1310·8)	495·0 (399·8 to 610·6)	532·3 (486·2 to 579·7)	133·4 (97·5 to 182·7)	18·3 (16·8 to 20·1)
Georgia*	2030	352·0 (318·9 to 391·6)	151·0 (121·9 to 187·0)	155·5 (142·0 to 169·4)	40·0 (29·2 to 55·0)	5·6 (5·1 to 6·1)	1209·3 (1095·6 to 1345·3)	518·8 (418·9 to 642·3)	534·1 (487·9 to 582·0)	137·3 (100·4 to 189·0)	19·1 (17·4 to 21·0)
Georgia*	2031	361·5 (327·2 to 403·0)	158·4 (128·0 to 196·2)	156·2 (142·7 to 170·2)	41·1 (30·1 to 56·9)	5·8 (5·2 to 6·4)	1241·9 (1124·0 to 1384·5)	544·0 (439·7 to 674·2)	536·7 (490·2 to 584·8)	141·4 (103·5 to 195·5)	19·8 (18·0 to 21·9)
Georgia*	2032	370·7 (334·7 to 414·0)	165·5 (133·0 to 205·7)	156·8 (143·2 to 171·0)	42·3 (31·0 to 58·7)	6·0 (5·4 to 6·6)	1273·7 (1150·0 to 1422·4)	568·7 (457·0 to 706·7)	538·8 (492·0 to 587·3)	145·5 (106·5 to 201·6)	20·6 (18·7 to 22·8)
Georgia*	2033	380·2 (342·5 to 424·6)	172·7 (138·3 to 214·4)	157·6 (143·9 to 171·9)	43·6 (31·9 to 60·6)	6·2 (5·6 to 6·9)	1306·0 (1176·7 to 1458·7)	593·3 (475·2 to 736·5)	541·5 (494·2 to 590·4)	149·9 (109·5 to 208·0)	21·4 (19·2 to 23·7)
Georgia*	2034	390·1 (351·2 to 436·1)	180·0 (144·4 to 221·6)	158·6 (144·7 to 173·0)	45·0 (32·8 to 62·6)	6·4 (5·8 to 7·2)	1340·1 (1206·4 to 1498·3)	618·5 (496·2 to 761·3)	544·9 (497·2 to 594·2)	154·6 (112·7 to 215·1)	22·1 (19·8 to 24·6)
Georgia*	2035	400·1 (359·8 to 448·0)	187·4 (150·8 to 232·2)	159·7 (145·7 to 174·2)	46·4 (33·8 to 64·7)	6·7 (5·9 to 7·5)	1374·6 (1236·1 to 1539·0)	643·7 (518·0 to 797·9)	548·6 (500·5 to 598·4)	159·5 (116·0 to 222·1)	22·9 (20·4 to 25·6)
Georgia*	2036	409·7 (368·2 to 459·7)	194·4 (156·2 to 240·9)	160·6 (146·5 to 175·3)	47·9 (34·6 to 66·8)	6·9 (6·1 to 7·7)	1407·7 (1264·9 to 1579·3)	667·9 (536·6 to 827·8)	551·7 (503·3 to 602·2)	164·4 (119·0 to 229·4)	23·6 (21·0 to 26·6)
Georgia*	2037	418·9 (376·6 to 470·7)	201·1 (160·6 to 248·9)	161·3 (147·1 to 176·1)	49·3 (35·6 to 68·9)	7·2 (6·4 to 8·1)	1439·1 (1293·7 to 1617·2)	691·0 (551·7 to 855·0)	554·2 (505·4 to 605·0)	169·3 (122·2 to 236·5)	24·6 (21·9 to 27·8)

Georgia*	2038	428·3 (384·6 to 480·8)	208·0 (166·2 to 256·5)	162·2 (147·8 to 177·0)	50·8 (36·5 to 71·2)	7·4 (6·6 to 8·4)	1471·6 (1321·4 to 1651·7)	714·5 (571·1 to 881·2)	557·1 (507·9 to 608·0)	174·4 (125·3 to 244·6)	25·5 (22·7 to 29·0)
Georgia*	2039	437·6 (392·7 to 491·8)	214·6 (171·4 to 264·7)	163·0 (148·5 to 177·9)	52·3 (37·3 to 73·7)	7·7 (6·8 to 8·8)	1503·3 (1349·1 to 1689·5)	737·3 (588·7 to 909·4)	559·9 (510·3 to 611·3)	179·6 (128·3 to 253·0)	26·6 (23·4 to 30·4)
Georgia*	2040	446·5 (399·2 to 501·5)	220·9 (175·6 to 270·7)	163·6 (149·1 to 178·8)	53·8 (38·2 to 75·9)	8·1 (7·1 to 9·3)	1533·8 (1371·5 to 1723·0)	759·1 (603·1 to 930·1)	562·1 (512·3 to 614·1)	184·9 (131·3 to 260·8)	27·8 (24·5 to 31·9)
Georgia*	2041	454·9 (406·1 to 511·3)	226·9 (180·0 to 279·9)	164·2 (149·6 to 179·5)	55·3 (39·1 to 78·2)	8·5 (7·4 to 9·8)	1562·7 (1395·3 to 1756·5)	779·4 (618·3 to 961·7)	564·1 (514·1 to 616·7)	190·1 (134·2 to 268·6)	29·0 (25·4 to 33·8)
Georgia*	2042	463·1 (413·7 to 521·3)	232·6 (185·7 to 285·8)	164·7 (150·1 to 180·1)	56·9 (39·9 to 80·5)	8·9 (7·7 to 10·4)	1590·8 (1421·4 to 1791·1)	799·1 (638·0 to 981·7)	565·8 (515·6 to 618·8)	195·5 (137·2 to 276·4)	30·5 (26·4 to 35·6)
Georgia*	2043	470·6 (419·5 to 530·9)	237·9 (188·0 to 293·3)	165·0 (150·3 to 180·5)	58·4 (40·8 to 82·7)	9·4 (8·0 to 11·0)	1616·8 (1441·3 to 1824·0)	817·2 (645·9 to 1007·6)	566·7 (516·3 to 620·2)	200·7 (140·2 to 284·1)	32·1 (27·7 to 37·9)
Georgia*	2044	478·1 (425·7 to 539·3)	243·0 (193·1 to 299·5)	165·2 (150·5 to 180·9)	60·0 (41·7 to 85·0)	9·9 (8·5 to 11·7)	1642·4 (1462·6 to 1852·8)	834·7 (663·4 to 1029·0)	567·6 (517·0 to 621·6)	206·1 (143·3 to 292·0)	33·9 (29·2 to 40·2)
Georgia*	2045	485·5 (432·0 to 548·6)	247·9 (195·7 to 303·0)	165·6 (150·8 to 181·4)	61·6 (42·7 to 87·4)	10·4 (8·9 to 12·5)	1667·9 (1484·1 to 1884·7)	851·6 (672·4 to 1041·0)	568·9 (518·2 to 623·3)	211·7 (146·8 to 300·2)	35·7 (30·5 to 42·9)
Georgia*	2046	492·3 (436·8 to 557·0)	252·3 (199·1 to 309·5)	165·8 (151·0 to 181·6)	63·2 (43·7 to 89·8)	11·0 (9·3 to 13·3)	1691·3 (1500·7 to 1913·6)	866·7 (684·1 to 1063·2)	569·5 (518·8 to 623·9)	217·3 (150·2 to 308·4)	37·8 (32·0 to 45·7)
Georgia*	2047	498·5 (440·9 to 563·7)	256·2 (202·8 to 316·3)	165·8 (151·0 to 181·6)	64·9 (44·6 to 92·1)	11·7 (9·8 to 14·2)	1712·7 (1514·9 to 1936·7)	880·2 (696·7 to 1086·8)	569·5 (518·9 to 624·0)	222·8 (153·2 to 316·4)	40·1 (33·6 to 48·8)
Georgia*	2048	504·5 (446·6 to 571·4)	259·9 (205·2 to 321·5)	165·7 (151·0 to 181·6)	66·5 (45·4 to 94·6)	12·4 (10·4 to 15·2)	1733·3 (1534·1 to 1963·2)	893·0 (705·1 to 1104·6)	569·3 (518·7 to 623·7)	228·3 (155·8 to 325·1)	42·6 (35·8 to 52·2)
Georgia*	2049	510·6 (450·9 to 578·6)	263·5 (208·0 to 325·8)	165·7 (150·9 to 181·5)	68·1 (46·2 to 97·7)	13·2 (10·9 to 16·5)	1754·0 (1549·1 to 1987·9)	905·4 (714·6 to 1119·4)	569·2 (518·6 to 623·6)	234·0 (158·8 to 335·8)	45·4 (37·6 to 56·6)
Georgia*	2050	516·1 (456·4 to 584·7)	266·7 (209·7 to 328·9)	165·6 (150·8 to 181·3)	69·8 (47·1 to 100·4)	14·1 (11·6 to 17·7)	1773·2 (1568·0 to 2008·7)	916·2 (720·4 to 1130·0)	568·8 (518·1 to 622·8)	239·8 (161·9 to 345·1)	48·4 (39·7 to 60·8)
Ghana*	1995	31·7 (27·4 to 36·4)	9·4 (7·7 to 11·5)	17·7 (14·0 to 21·9)	2·9 (2·0 to 4·1)	1·6 (1·6 to 1·6)	82·6 (71·6 to 94·9)	24·6 (20·0 to 30·0)	46·2 (36·6 to 57·2)	7·7 (5·2 to 10·6)	4·1 (4·1 to 4·1)
Ghana*	1996	32·7 (28·5 to 37·3)	9·9 (8·1 to 11·9)	18·1 (14·5 to 22·2)	3·0 (2·0 to 4·1)	1·8 (1·8 to 1·8)	85·2 (74·3 to 97·3)	25·7 (21·1 to 31·1)	47·1 (37·9 to 57·9)	7·8 (5·3 to 10·8)	4·6 (4·6 to 4·6)

Ghana*	1997	36·5 (32·4 to 41·1)	9·5 (7·8 to 11·4)	18·3 (14·8 to 22·6)	2·9 (2·0 to 4·1)	5·7 (5·7 to 5·7)	95·2 (84·4 to 107·2)	24·8 (20·4 to 29·8)	47·8 (38·5 to 58·9)	7·6 (5·2 to 10·7)	14·9 (14·9 to 14·9)
Ghana*	1998	33·4 (29·2 to 38·1)	9·8 (8·1 to 11·9)	18·5 (14·8 to 23·0)	2·9 (2·0 to 4·1)	2·2 (2·2 to 2·2)	87·2 (76·1 to 99·4)	25·6 (21·2 to 31·0)	48·3 (38·5 to 59·9)	7·6 (5·3 to 10·6)	5·6 (5·6 to 5·6)
Ghana*	1999	34·8 (30·5 to 39·6)	9·6 (8·0 to 11·6)	18·6 (14·8 to 22·8)	2·8 (1·9 to 4·0)	3·8 (3·8 to 3·8)	90·7 (79·5 to 103·3)	25·1 (20·8 to 30·4)	48·4 (38·5 to 59·6)	7·3 (5·0 to 10·4)	9·9 (9·9 to 9·9)
Ghana*	2000	35·1 (30·5 to 40·0)	9·9 (8·1 to 11·9)	18·9 (15·0 to 23·4)	2·6 (1·8 to 3·6)	3·7 (3·7 to 3·7)	91·5 (79·6 to 104·3)	25·8 (21·2 to 31·0)	49·3 (39·2 to 61·0)	6·7 (4·6 to 9·4)	9·7 (9·7 to 9·7)
Ghana*	2001	38·5 (33·8 to 44·2)	11·0 (9·0 to 13·3)	19·6 (15·7 to 24·4)	2·6 (1·8 to 3·7)	5·3 (5·3 to 5·3)	100·5 (88·3 to 115·2)	28·6 (23·4 to 34·6)	51·2 (40·9 to 63·6)	6·8 (4·7 to 9·5)	13·9 (13·9 to 13·9)
Ghana*	2002	34·6 (30·5 to 39·8)	9·3 (7·6 to 11·2)	18·5 (14·8 to 23·1)	2·4 (1·6 to 3·3)	4·4 (4·4 to 4·4)	90·3 (79·5 to 103·9)	24·3 (19·9 to 29·2)	48·3 (38·5 to 60·2)	6·1 (4·2 to 8·6)	11·6 (11·6 to 11·6)
Ghana*	2003	40·0 (35·6 to 45·4)	10·7 (8·8 to 12·8)	19·4 (15·5 to 24·1)	2·5 (1·7 to 3·5)	7·5 (7·5 to 7·5)	104·4 (92·7 to 118·3)	27·9 (22·9 to 33·4)	50·5 (40·3 to 62·8)	6·5 (4·4 to 9·1)	19·5 (19·5 to 19·5)
Ghana*	2004	46·6 (41·8 to 52·0)	12·6 (10·4 to 15·1)	20·4 (16·3 to 25·3)	2·7 (1·9 to 3·8)	10·9 (10·9 to 10·9)	121·5 (109·1 to 135·6)	32·9 (27·0 to 39·5)	53·1 (42·6 to 66·0)	7·1 (4·9 to 9·9)	28·5 (28·5 to 28·5)
Ghana*	2005	49·9 (44·7 to 55·4)	14·9 (12·3 to 17·8)	21·1 (16·9 to 26·1)	3·0 (2·1 to 4·3)	10·8 (10·8 to 10·8)	130·2 (116·6 to 144·3)	38·9 (32·0 to 46·5)	55·1 (44·0 to 68·1)	8·0 (5·4 to 11·2)	28·2 (28·2 to 28·2)
Ghana*	2006	53·2 (47·6 to 59·1)	18·1 (14·9 to 21·5)	22·4 (18·0 to 27·5)	3·2 (2·2 to 4·4)	9·5 (9·5 to 9·5)	138·7 (124·2 to 154·0)	47·2 (38·8 to 56·0)	58·5 (47·0 to 71·7)	8·3 (5·6 to 11·5)	24·7 (24·7 to 24·7)
Ghana*	2007	59·1 (53·0 to 65·7)	22·3 (18·6 to 26·4)	23·9 (19·4 to 29·2)	3·4 (2·3 to 4·8)	9·4 (9·4 to 9·4)	154·2 (138·1 to 171·3)	58·2 (48·6 to 68·8)	62·4 (50·7 to 76·2)	9·0 (6·1 to 12·6)	24·6 (24·6 to 24·6)
Ghana*	2008	64·8 (58·7 to 71·8)	25·1 (21·0 to 29·5)	24·8 (20·3 to 30·3)	3·8 (2·6 to 5·3)	11·2 (11·2 to 11·2)	169·1 (153·2 to 187·4)	65·4 (54·7 to 77·0)	64·8 (52·9 to 79·0)	9·8 (6·7 to 13·9)	29·1 (29·1 to 29·1)
Ghana*	2009	68·6 (62·4 to 75·5)	27·4 (22·9 to 32·1)	25·4 (21·1 to 30·8)	4·1 (2·8 to 5·8)	11·7 (11·7 to 11·7)	179·0 (162·7 to 197·0)	71·4 (59·8 to 83·6)	66·2 (54·9 to 80·4)	10·7 (7·4 to 15·0)	30·6 (30·6 to 30·6)
Ghana*	2010	72·8 (65·9 to 80·4)	31·7 (26·6 to 37·0)	27·2 (22·9 to 32·3)	4·0 (2·8 to 5·8)	10·0 (10·0 to 10·0)	189·9 (171·9 to 209·7)	82·6 (69·3 to 96·5)	70·8 (59·8 to 84·3)	10·5 (7·3 to 15·1)	25·9 (25·9 to 25·9)

Ghana*	2011	76·0 (69·1 to 83·7)	33·5 (28·0 to 39·2)	29·3 (25·3 to 33·8)	3·6 (2·5 to 5·1)	9·6 (9·6 to 9·6)	198·1 (180·2 to 218·3)	87·5 (73·0 to 102·2)	76·4 (66·0 to 88·3)	9·3 (6·4 to 13·3)	24·9 (24·9 to 24·9)
Ghana*	2012	82·8 (76·1 to 90·0)	35·2 (29·7 to 41·2)	31·6 (28·1 to 35·6)	3·2 (2·2 to 4·6)	12·8 (12·8 to 12·8)	216·0 (198·5 to 234·8)	91·9 (77·4 to 107·5)	82·3 (73·4 to 92·8)	8·4 (5·8 to 12·0)	33·4 (33·4 to 33·4)
Ghana*	2013	81·8 (75·5 to 88·8)	33·7 (28·2 to 39·8)	32·6 (29·9 to 35·6)	3·0 (2·1 to 4·3)	12·4 (12·4 to 12·4)	213·2 (197·0 to 231·5)	87·9 (73·6 to 103·8)	85·1 (77·9 to 92·9)	7·8 (5·4 to 11·2)	32·3 (32·3 to 32·3)
Ghana*	2014	74·8 (69·0 to 81·5)	31·1 (25·9 to 36·9)	32·3 (30·0 to 34·8)	3·4 (2·3 to 4·8)	8·0 (8·0 to 8·0)	195·1 (179·9 to 212·5)	81·2 (67·5 to 96·2)	84·1 (78·3 to 90·6)	8·8 (6·0 to 12·6)	20·9 (20·9 to 20·9)
Ghana*	2015	71·6 (66·2 to 78·0)	27·7 (22·8 to 33·2)	29·5 (27·2 to 32·1)	4·0 (2·8 to 5·6)	10·3 (10·3 to 10·3)	186·7 (172·6 to 203·5)	72·3 (59·5 to 86·5)	77·0 (70·9 to 83·8)	10·5 (7·2 to 14·7)	26·9 (26·9 to 26·9)
Ghana*	2016	69·1 (63·2 to 75·5)	27·0 (22·6 to 32·6)	27·5 (24·8 to 30·7)	5·7 (4·0 to 8·2)	8·8 (8·8 to 8·8)	180·2 (164·9 to 197·0)	70·5 (58·9 to 85·0)	71·8 (64·6 to 80·1)	15·0 (10·5 to 21·4)	22·9 (22·9 to 22·9)
Ghana*	2017	66·3 (60·6 to 72·7)	24·7 (20·7 to 29·8)	26·3 (23·0 to 30·4)	6·7 (4·7 to 9·6)	8·5 (8·5 to 8·5)	172·8 (158·1 to 189·6)	64·5 (53·9 to 77·8)	68·7 (60·1 to 79·2)	17·4 (12·2 to 24·9)	22·3 (22·3 to 22·3)
Ghana*	2018	70·8 (64·1 to 78·5)	28·0 (23·3 to 33·9)	27·3 (23·3 to 32·1)	7·6 (5·3 to 10·8)	7·9 (7·9 to 7·9)	184·6 (167·2 to 204·7)	72·9 (60·8 to 88·3)	71·2 (60·8 to 83·6)	19·8 (13·8 to 28·2)	20·6 (20·6 to 20·6)
Ghana*	2019	72·5 (65·5 to 80·6)	28·7 (24·0 to 34·9)	28·4 (24·2 to 33·3)	8·1 (5·6 to 11·6)	7·3 (7·2 to 7·4)	189·0 (170·9 to 210·3)	74·9 (62·5 to 91·0)	74·0 (63·2 to 86·9)	21·1 (14·7 to 30·2)	19·1 (18·8 to 19·3)
Ghana*	2020	80·9 (73·2 to 90·2)	36·3 (30·4 to 44·2)	27·1 (23·2 to 31·9)	7·9 (5·5 to 11·3)	9·5 (9·4 to 9·7)	211·1 (190·8 to 235·3)	94·8 (79·2 to 115·3)	70·8 (60·4 to 83·1)	20·6 (14·4 to 29·5)	24·9 (24·6 to 25·2)
Ghana*	2021	78·4 (70·9 to 87·3)	35·1 (29·3 to 42·7)	26·2 (22·3 to 30·7)	7·8 (5·4 to 11·2)	9·3 (9·2 to 9·4)	204·4 (184·9 to 227·8)	91·5 (76·5 to 111·4)	68·2 (58·2 to 80·1)	20·3 (14·1 to 29·1)	24·3 (24·0 to 24·6)
Ghana*	2022	69·4 (62·7 to 77·1)	27·7 (23·2 to 33·8)	26·4 (22·6 to 31·0)	8·1 (5·6 to 11·5)	7·1 (6·8 to 7·5)	180·9 (163·5 to 201·1)	72·3 (60·4 to 88·1)	68·9 (58·8 to 80·9)	21·0 (14·6 to 30·1)	18·5 (17·6 to 19·5)
Ghana*	2023	70·6 (63·8 to 78·5)	28·4 (23·6 to 34·7)	26·7 (22·8 to 31·4)	8·4 (5·8 to 11·9)	7·1 (6·7 to 7·6)	184·2 (166·4 to 204·8)	74·1 (61·7 to 90·4)	69·7 (59·5 to 81·8)	21·8 (15·1 to 31·2)	18·6 (17·6 to 19·7)
Ghana*	2024	72·0 (65·0 to 80·1)	29·1 (24·1 to 35·5)	27·0 (23·1 to 31·7)	8·6 (6·0 to 12·4)	7·2 (6·7 to 7·6)	187·7 (169·6 to 208·9)	75·9 (62·9 to 92·6)	70·5 (60·2 to 82·7)	22·6 (15·6 to 32·2)	18·7 (17·6 to 19·9)

Ghana*	2025	73·3 (66·1 to 81·6)	29·8 (24·6 to 36·4)	27·4 (23·4 to 32·1)	9·0 (6·2 to 12·8)	7·2 (6·7 to 7·7)	191·1 (172·5 to 212·8)	77·7 (64·1 to 94·9)	71·4 (60·9 to 83·7)	23·4 (16·1 to 33·4)	18·7 (17·5 to 20·0)
Ghana*	2026	74·7 (67·2 to 83·3)	30·5 (25·1 to 37·3)	27·7 (23·6 to 32·5)	9·3 (6·4 to 13·3)	7·2 (6·7 to 7·7)	194·7 (175·3 to 217·1)	79·5 (65·3 to 97·2)	72·3 (61·7 to 84·7)	24·2 (16·7 to 34·6)	18·7 (17·5 to 20·1)
Ghana*	2027	76·0 (68·6 to 84·8)	31·2 (25·6 to 38·1)	28·0 (23·9 to 32·8)	9·6 (6·6 to 13·8)	7·2 (6·6 to 7·7)	198·2 (179·0 to 221·2)	81·3 (66·8 to 99·5)	73·1 (62·4 to 85·6)	25·1 (17·2 to 35·9)	18·7 (17·3 to 20·2)
Ghana*	2028	77·4 (69·6 to 86·4)	31·9 (26·2 to 39·1)	28·4 (24·2 to 33·2)	10·0 (6·8 to 14·3)	7·2 (6·6 to 7·8)	201·8 (181·4 to 225·4)	83·2 (68·4 to 101·9)	73·9 (63·1 to 86·6)	26·0 (17·8 to 37·3)	18·7 (17·3 to 20·3)
Ghana*	2029	78·8 (70·8 to 88·1)	32·7 (26·8 to 40·3)	28·7 (24·5 to 33·6)	10·3 (7·1 to 14·8)	7·2 (6·6 to 7·8)	205·5 (184·7 to 229·9)	85·2 (69·9 to 105·1)	74·8 (63·8 to 87·6)	26·9 (18·5 to 38·7)	18·7 (17·2 to 20·4)
Ghana*	2030	80·3 (71·9 to 89·8)	33·4 (27·3 to 41·4)	29·0 (24·8 to 34·0)	10·7 (7·3 to 15·4)	7·1 (6·5 to 7·9)	209·3 (187·5 to 234·3)	87·1 (71·2 to 108·0)	75·7 (64·6 to 88·7)	27·9 (19·1 to 40·1)	18·6 (17·0 to 20·5)
Ghana*	2031	81·7 (73·2 to 91·8)	34·2 (27·8 to 42·5)	29·4 (25·1 to 34·5)	11·1 (7·5 to 15·9)	7·1 (6·5 to 7·9)	213·2 (190·9 to 239·4)	89·1 (72·5 to 110·9)	76·6 (65·4 to 89·9)	28·9 (19·7 to 41·5)	18·6 (16·8 to 20·6)
Ghana*	2032	83·4 (74·7 to 93·7)	35·1 (28·4 to 43·3)	29·8 (25·4 to 34·9)	11·5 (7·8 to 16·6)	7·1 (6·4 to 7·8)	217·5 (194·7 to 244·4)	91·4 (74·1 to 113·0)	77·6 (66·3 to 91·1)	29·9 (20·3 to 43·2)	18·5 (16·7 to 20·4)
Ghana*	2033	85·2 (76·0 to 96·4)	36·1 (29·1 to 45·2)	30·2 (25·8 to 35·5)	11·9 (8·0 to 17·3)	7·1 (6·4 to 7·8)	222·1 (198·2 to 251·3)	94·0 (75·8 to 117·8)	78·7 (67·2 to 92·5)	31·0 (21·0 to 45·0)	18·4 (16·6 to 20·4)
Ghana*	2034	87·0 (77·4 to 98·8)	37·1 (29·5 to 46·8)	30·6 (26·1 to 36·0)	12·3 (8·3 to 18·0)	7·0 (6·3 to 7·8)	226·9 (202·0 to 257·7)	96·6 (76·9 to 122·0)	79·8 (68·1 to 93·8)	32·1 (21·7 to 46·8)	18·4 (16·5 to 20·3)
Ghana*	2035	88·9 (79·0 to 100·7)	38·1 (30·2 to 47·6)	31·0 (26·5 to 36·5)	12·8 (8·7 to 18·6)	7·0 (6·3 to 7·8)	231·8 (206·1 to 262·6)	99·3 (78·8 to 124·3)	81·0 (69·0 to 95·2)	33·3 (22·6 to 48·4)	18·3 (16·4 to 20·4)
Ghana*	2036	90·8 (80·4 to 103·3)	39·1 (30·8 to 49·4)	31·5 (26·9 to 37·0)	13·2 (9·0 to 19·2)	7·0 (6·2 to 7·8)	236·7 (209·7 to 269·4)	101·9 (80·2 to 128·8)	82·1 (70·0 to 96·6)	34·5 (23·4 to 50·1)	18·2 (16·3 to 20·3)
Ghana*	2037	92·8 (81·9 to 106·0)	40·2 (31·5 to 51·1)	32·0 (27·2 to 37·6)	13·7 (9·3 to 20·0)	6·9 (6·2 to 7·8)	242·0 (213·6 to 276·4)	104·8 (82·1 to 133·3)	83·4 (71·1 to 98·1)	35·8 (24·2 to 52·1)	18·1 (16·1 to 20·3)
Ghana*	2038	94·8 (83·4 to 108·3)	41·3 (32·0 to 52·7)	32·4 (27·7 to 38·2)	14·2 (9·6 to 20·7)	6·9 (6·2 to 7·8)	247·3 (217·5 to 282·5)	107·7 (83·5 to 137·5)	84·6 (72·1 to 99·6)	37·1 (25·0 to 54·0)	18·0 (16·1 to 20·2)

Ghana*	2039	96·9 (85·3 to 111·4)	42·4 (32·9 to 54·1)	32·9 (28·0 to 38·7)	14·7 (9·9 to 21·5)	6·9 (6·1 to 7·8)	252·7 (222·5 to 290·4)	110·5 (85·7 to 141·2)	85·8 (73·1 to 100·9)	38·4 (25·9 to 56·1)	18·0 (16·0 to 20·3)
Ghana*	2040	99·0 (86·9 to 114·1)	43·5 (33·6 to 56·1)	33·3 (28·4 to 39·2)	15·3 (10·3 to 22·3)	6·9 (6·1 to 7·9)	258·3 (226·7 to 297·4)	113·5 (87·7 to 146·2)	86·9 (74·1 to 102·3)	39·8 (26·9 to 58·1)	18·1 (16·0 to 20·6)
Ghana*	2041	101·2 (88·5 to 117·2)	44·6 (34·5 to 57·5)	33·8 (28·8 to 39·7)	15·8 (10·7 to 23·0)	6·9 (6·1 to 8·0)	263·8 (230·8 to 305·6)	116·4 (90·1 to 150·1)	88·0 (75·1 to 103·5)	41·2 (27·8 to 60·1)	18·1 (16·0 to 20·8)
Ghana*	2042	103·3 (90·2 to 119·3)	45·8 (35·4 to 58·8)	34·2 (29·1 to 40·2)	16·4 (11·0 to 23·8)	7·0 (6·1 to 8·0)	269·4 (235·1 to 311·1)	119·4 (92·4 to 153·3)	89·2 (76·0 to 104·8)	42·6 (28·7 to 62·0)	18·2 (15·9 to 21·0)
Ghana*	2043	105·4 (91·9 to 121·8)	46·9 (36·2 to 60·7)	34·6 (29·5 to 40·7)	16·9 (11·3 to 24·6)	7·0 (6·1 to 8·1)	275·0 (239·7 to 317·6)	122·2 (94·4 to 158·4)	90·3 (76·9 to 106·1)	44·1 (29·6 to 64·1)	18·3 (15·9 to 21·2)
Ghana*	2044	107·7 (93·7 to 124·9)	48·0 (36·6 to 62·2)	35·1 (29·9 to 41·2)	17·5 (11·7 to 25·4)	7·1 (6·2 to 8·3)	280·8 (244·2 to 325·7)	125·2 (95·4 to 162·3)	91·4 (77·9 to 107·4)	45·6 (30·5 to 66·2)	18·5 (16·1 to 21·6)
Ghana*	2045	109·8 (95·3 to 127·5)	49·1 (37·2 to 63·3)	35·5 (30·2 to 41·6)	18·1 (12·1 to 26·2)	7·1 (6·2 to 8·4)	286·4 (248·6 to 332·6)	128·1 (96·9 to 165·2)	92·5 (78·8 to 108·5)	47·2 (31·5 to 68·4)	18·6 (16·1 to 21·9)
Ghana*	2046	112·1 (97·1 to 130·0)	50·3 (38·0 to 65·4)	35·9 (30·5 to 42·1)	18·7 (12·5 to 27·2)	7·2 (6·2 to 8·5)	292·3 (253·1 to 339·1)	131·1 (99·2 to 170·5)	93·6 (79·7 to 109·8)	48·8 (32·6 to 70·9)	18·8 (16·2 to 22·0)
Ghana*	2047	114·4 (98·8 to 133·1)	51·5 (38·4 to 67·4)	36·3 (30·8 to 42·6)	19·3 (12·9 to 28·2)	7·3 (6·2 to 8·6)	298·3 (257·6 to 347·2)	134·3 (100·0 to 175·7)	94·6 (80·4 to 111·1)	50·4 (33·6 to 73·6)	19·0 (16·2 to 22·4)
Ghana*	2048	116·7 (100·4 to 136·5)	52·6 (39·5 to 69·0)	36·7 (31·1 to 43·1)	20·0 (13·2 to 29·1)	7·3 (6·3 to 8·7)	304·2 (262·0 to 356·1)	137·3 (102·9 to 180·0)	95·7 (81·2 to 112·3)	52·1 (34·5 to 76·0)	19·1 (16·4 to 22·6)
Ghana*	2049	118·9 (102·4 to 139·9)	53·8 (39·5 to 72·3)	37·1 (31·4 to 43·5)	20·6 (13·6 to 30·4)	7·4 (6·3 to 8·8)	310·1 (266·9 to 364·8)	140·2 (102·9 to 188·6)	96·7 (82·0 to 113·5)	53·8 (35·5 to 79·4)	19·4 (16·5 to 22·9)
Ghana*	2050	121·1 (103·8 to 142·3)	54·8 (40·5 to 73·1)	37·5 (31·8 to 44·0)	21·3 (14·0 to 31·5)	7·5 (6·4 to 9·0)	315·9 (270·6 to 371·1)	143·0 (105·6 to 190·6)	97·7 (82·8 to 114·6)	55·5 (36·5 to 82·0)	19·6 (16·6 to 23·4)
Guinea*	1995	32·2 (27·6 to 37·9)	2·0 (1·6 to 2·5)	23·3 (18·6 to 29·0)	0·2 (0·1 to 0·2)	6·7 (6·7 to 6·7)	79·5 (68·0 to 93·6)	5·0 (4·0 to 6·1)	57·6 (46·0 to 71·6)	0·4 (0·3 to 0·5)	16·5 (16·5 to 16·5)
Guinea*	1996	28·7 (24·0 to 34·7)	2·3 (1·9 to 2·8)	23·8 (19·1 to 29·8)	0·2 (0·1 to 0·2)	2·4 (2·4 to 2·4)	70·7 (59·3 to 85·6)	5·8 (4·7 to 7·0)	58·7 (47·1 to 73·6)	0·4 (0·3 to 0·6)	5·9 (5·9 to 5·9)
Guinea*	1997	31·2 (26·6 to 37·0)	2·5 (2·0 to 3·0)	23·7 (19·1 to 29·6)	0·2 (0·1 to 0·2)	4·9 (4·9 to 4·9)	77·1 (65·7 to 91·4)	6·1 (5·0 to 7·5)	58·5 (47·3 to 73·0)	0·4 (0·3 to 0·6)	12·1 (12·1 to 12·1)

Guinea*	1998	29·8 (25·2 to 35·4)	2·5 (2·0 to 3·0)	23·1 (18·5 to 28·5)	0·2 (0·1 to 0·2)	4·1 (4·1 to 4·1)	73·5 (62·1 to 87·4)	6·2 (5·1 to 7·5)	56·9 (45·6 to 70·4)	0·4 (0·3 to 0·5)	10·1 (10·1 to 10·1)
Guinea*	1999	28·9 (24·2 to 34·0)	2·7 (2·2 to 3·3)	23·3 (18·7 to 28·5)	0·2 (0·1 to 0·2)	2·8 (2·8 to 2·8)	71·3 (59·7 to 83·8)	6·6 (5·4 to 8·1)	57·5 (46·1 to 70·3)	0·4 (0·3 to 0·6)	6·8 (6·8 to 6·8)
Guinea*	2000	29·9 (25·4 to 34·8)	2·7 (2·2 to 3·3)	23·4 (19·0 to 28·5)	0·2 (0·1 to 0·2)	3·6 (3·6 to 3·6)	73·7 (62·6 to 86·0)	6·7 (5·5 to 8·2)	57·7 (46·9 to 70·4)	0·4 (0·3 to 0·6)	8·9 (8·9 to 8·9)
Guinea*	2001	32·9 (28·3 to 38·2)	3·2 (2·6 to 3·8)	24·5 (19·9 to 30·0)	0·2 (0·1 to 0·3)	5·0 (5·0 to 5·0)	81·1 (69·8 to 94·3)	7·8 (6·3 to 9·5)	60·5 (49·2 to 74·1)	0·4 (0·3 to 0·6)	12·5 (12·5 to 12·5)
Guinea*	2002	32·2 (27·5 to 37·8)	3·3 (2·7 to 4·1)	24·9 (20·3 to 30·5)	0·2 (0·1 to 0·3)	3·7 (3·7 to 3·7)	79·4 (67·9 to 93·3)	8·2 (6·7 to 10·0)	61·5 (50·1 to 75·3)	0·5 (0·3 to 0·7)	9·2 (9·2 to 9·2)
Guinea*	2003	30·8 (26·4 to 36·0)	3·0 (2·5 to 3·7)	24·3 (19·7 to 29·7)	0·2 (0·1 to 0·3)	3·3 (3·3 to 3·3)	76·0 (65·0 to 88·8)	7·5 (6·1 to 9·0)	59·9 (48·6 to 73·3)	0·4 (0·3 to 0·6)	8·2 (8·2 to 8·2)
Guinea*	2004	29·6 (25·2 to 34·8)	2·5 (2·0 to 3·0)	23·1 (18·8 to 28·2)	0·2 (0·1 to 0·2)	3·8 (3·8 to 3·8)	73·0 (62·3 to 85·8)	6·1 (5·0 to 7·4)	57·0 (46·5 to 69·5)	0·4 (0·3 to 0·6)	9·5 (9·5 to 9·5)
Guinea*	2005	27·6 (23·8 to 32·3)	2·1 (1·8 to 2·6)	21·3 (17·5 to 26·1)	0·2 (0·1 to 0·3)	4·0 (4·0 to 4·0)	68·1 (58·6 to 79·6)	5·3 (4·4 to 6·4)	52·6 (43·2 to 64·4)	0·4 (0·3 to 0·6)	9·8 (9·8 to 9·8)
Guinea*	2006	28·3 (24·5 to 32·5)	2·3 (2·0 to 2·8)	21·2 (17·4 to 25·7)	0·2 (0·1 to 0·3)	4·6 (4·6 to 4·6)	69·9 (60·5 to 80·3)	5·8 (4·8 to 6·9)	52·3 (42·9 to 63·4)	0·5 (0·3 to 0·7)	11·3 (11·3 to 11·3)
Guinea*	2007	24·1 (20·9 to 27·8)	1·9 (1·6 to 2·3)	18·8 (15·6 to 22·5)	0·2 (0·1 to 0·3)	3·2 (3·2 to 3·2)	59·5 (51·6 to 68·7)	4·7 (3·9 to 5·6)	46·4 (38·5 to 55·5)	0·4 (0·3 to 0·6)	7·9 (7·9 to 7·9)
Guinea*	2008	25·2 (22·3 to 28·5)	2·1 (1·8 to 2·6)	18·7 (15·9 to 22·0)	0·2 (0·1 to 0·3)	4·2 (4·2 to 4·2)	62·3 (55·0 to 70·4)	5·3 (4·4 to 6·3)	46·2 (39·2 to 54·2)	0·5 (0·3 to 0·7)	10·3 (10·3 to 10·3)
Guinea*	2009	25·8 (23·0 to 28·8)	2·7 (2·3 to 3·3)	19·6 (16·9 to 22·6)	0·2 (0·1 to 0·3)	3·2 (3·2 to 3·2)	63·6 (56·7 to 71·1)	6·8 (5·6 to 8·1)	48·4 (41·7 to 55·7)	0·5 (0·3 to 0·7)	7·9 (7·9 to 7·9)
Guinea*	2010	28·2 (25·8 to 30·8)	3·5 (2·9 to 4·3)	20·0 (17·7 to 22·5)	0·2 (0·1 to 0·3)	4·5 (4·5 to 4·5)	69·7 (63·6 to 75·9)	8·7 (7·1 to 10·5)	49·3 (43·7 to 55·6)	0·5 (0·4 to 0·8)	11·1 (11·1 to 11·1)
Guinea*	2011	25·9 (24·0 to 27·8)	3·5 (2·9 to 4·2)	18·8 (17·0 to 20·6)	0·2 (0·2 to 0·3)	3·4 (3·4 to 3·4)	63·9 (59·2 to 68·7)	8·7 (7·1 to 10·4)	46·4 (42·0 to 50·8)	0·5 (0·4 to 0·8)	8·3 (8·3 to 8·3)
Guinea*	2012	30·3 (28·6 to 32·1)	4·4 (3·6 to 5·2)	20·1 (18·6 to 21·7)	0·3 (0·2 to 0·4)	5·5 (5·5 to 5·5)	74·7 (70·6 to 79·2)	10·9 (9·0 to 12·9)	49·6 (45·9 to 53·6)	0·7 (0·5 to 1·0)	13·6 (13·6 to 13·6)
Guinea*	2013	29·1 (27·4 to 30·9)	4·5 (3·7 to 5·4)	20·7 (19·2 to 22·3)	0·3 (0·2 to 0·4)	3·6 (3·6 to 3·6)	71·8 (67·7 to 76·2)	11·1 (9·2 to 13·3)	51·1 (47·4 to 55·0)	0·7 (0·5 to 1·0)	8·9 (8·9 to 8·9)
Guinea*	2014	51·4 (48·8 to 54·7)	5·1 (4·2 to 6·0)	23·9 (22·2 to 25·5)	6·7 (4·6 to 9·3)	15·8 (15·8 to 15·8)	126·9 (120·3 to 135·0)	12·5 (10·5 to 14·7)	59·0 (54·9 to 62·9)	16·4 (11·3 to 23·0)	39·1 (39·1 to 39·1)

Guinea*	2015	54·3 (51·1 to 57·8)	5·4 (4·5 to 6·5)	25·8 (24·0 to 27·6)	7·6 (5·2 to 10·4)	15·5 (15·5 to 15·5)	134·0 (126·1 to 142·7)	13·4 (11·2 to 16·0)	63·7 (59·3 to 68·2)	18·8 (12·9 to 25·8)	38·2 (38·2 to 38·2)
Guinea*	2016	49·5 (46·3 to 52·9)	5·4 (4·5 to 6·4)	25·7 (23·5 to 27·7)	6·2 (4·2 to 8·7)	12·3 (12·3 to 12·3)	122·1 (114·2 to 130·5)	13·2 (11·1 to 15·8)	63·3 (58·0 to 68·5)	15·3 (10·3 to 21·4)	30·3 (30·3 to 30·3)
Guinea*	2017	49·6 (45·6 to 53·5)	6·5 (5·4 to 7·8)	27·2 (24·0 to 30·2)	6·1 (4·1 to 8·6)	9·8 (9·8 to 9·8)	122·4 (112·5 to 132·1)	16·0 (13·4 to 19·1)	67·2 (59·3 to 74·6)	15·0 (10·1 to 21·1)	24·2 (24·2 to 24·2)
Guinea*	2018	49·7 (45·0 to 54·5)	6·5 (5·4 to 7·7)	27·1 (23·1 to 31·1)	5·9 (4·1 to 8·4)	10·2 (10·2 to 10·2)	122·7 (111·0 to 134·5)	16·0 (13·2 to 19·1)	67·0 (57·1 to 76·8)	14·5 (10·0 to 20·7)	25·2 (25·2 to 25·2)
Guinea*	2019	50·1 (45·2 to 55·0)	6·5 (5·3 to 7·7)	27·7 (23·6 to 31·8)	6·1 (4·2 to 8·8)	9·8 (9·8 to 9·9)	123·5 (111·6 to 135·7)	15·9 (13·1 to 19·1)	68·3 (58·2 to 78·4)	15·1 (10·4 to 21·6)	24·3 (24·1 to 24·5)
Guinea*	2020	59·3 (54·7 to 64·0)	14·0 (12·9 to 15·3)	26·1 (22·2 to 30·0)	5·9 (4·1 to 8·5)	13·2 (13·1 to 13·4)	146·3 (134·9 to 157·9)	34·6 (31·8 to 37·6)	64·5 (54·9 to 74·0)	14·6 (10·1 to 20·9)	32·6 (32·3 to 33·1)
Guinea*	2021	50·9 (46·4 to 55·4)	6·8 (5·6 to 8·3)	24·7 (21·1 to 28·4)	5·7 (4·0 to 8·2)	13·5 (13·4 to 14·0)	125·5 (114·5 to 136·8)	16·9 (13·9 to 20·5)	61·1 (52·0 to 70·1)	14·2 (9·8 to 20·3)	33·4 (33·0 to 34·6)
Guinea*	2022	47·0 (42·4 to 51·6)	5·9 (4·7 to 7·2)	25·0 (21·3 to 28·7)	5·9 (4·1 to 8·5)	10·2 (9·7 to 10·8)	116·0 (104·7 to 127·4)	14·5 (11·5 to 17·9)	61·7 (52·6 to 70·8)	14·7 (10·1 to 20·9)	25·2 (23·9 to 26·8)
Guinea*	2023	47·5 (42·8 to 52·2)	6·0 (4·7 to 7·3)	25·4 (21·6 to 29·1)	6·2 (4·2 to 8·8)	10·0 (9·5 to 10·7)	117·3 (105·7 to 128·8)	14·7 (11·7 to 18·1)	62·6 (53·3 to 71·8)	15·2 (10·4 to 21·7)	24·8 (23·4 to 26·3)
Guinea*	2024	48·1 (43·3 to 52·9)	6·1 (4·8 to 7·5)	25·8 (22·0 to 29·6)	6·4 (4·4 to 9·2)	9·8 (9·3 to 10·5)	118·7 (106·9 to 130·6)	15·0 (11·8 to 18·6)	63·6 (54·2 to 73·0)	15·9 (10·8 to 22·8)	24·3 (22·8 to 25·9)
Guinea*	2025	48·9 (44·0 to 53·8)	6·2 (4·9 to 7·7)	26·2 (22·3 to 30·0)	6·7 (4·6 to 9·6)	9·8 (9·1 to 10·5)	120·6 (108·6 to 132·7)	15·4 (12·0 to 19·1)	64·5 (55·0 to 74·1)	16·5 (11·3 to 23·8)	24·1 (22·5 to 25·9)
Guinea*	2026	49·6 (44·6 to 54·7)	6·4 (4·9 to 8·0)	26·6 (22·6 to 30·5)	7·0 (4·7 to 10·1)	9·6 (8·9 to 10·4)	122·3 (110·0 to 134·9)	15·8 (12·2 to 19·7)	65·6 (55·9 to 75·2)	17·2 (11·7 to 24·8)	23·8 (22·0 to 25·7)
Guinea*	2027	50·3 (45·1 to 55·6)	6·6 (5·1 to 8·3)	27·0 (23·0 to 31·0)	7·3 (5·0 to 10·5)	9·4 (8·7 to 10·2)	124·0 (111·3 to 137·1)	16·3 (12·5 to 20·5)	66·6 (56·8 to 76·5)	17·9 (12·2 to 26·0)	23·3 (21·6 to 25·2)
Guinea*	2028	51·1 (45·8 to 56·5)	6·9 (5·3 to 8·7)	27·5 (23·5 to 31·6)	7·6 (5·2 to 11·0)	9·1 (8·4 to 10·0)	126·0 (113·0 to 139·4)	16·9 (13·1 to 21·4)	67·8 (57·9 to 77·9)	18·7 (12·7 to 27·1)	22·6 (20·8 to 24·6)

Guinea*	2029	51·9 (46·4 to 57·6)	7·1 (5·5 to 9·0)	28·0 (23·9 to 32·2)	7·9 (5·4 to 11·4)	8·9 (8·1 to 9·8)	128·2 (114·5 to 142·2)	17·6 (13·6 to 22·3)	69·2 (59·1 to 79·4)	19·5 (13·3 to 28·2)	21·9 (20·0 to 24·1)
Guinea*	2030	52·9 (47·2 to 58·6)	7·5 (5·7 to 9·4)	28·6 (24·4 to 32·8)	8·3 (5·6 to 12·0)	8·6 (7·7 to 9·6)	130·5 (116·4 to 144·7)	18·4 (14·2 to 23·3)	70·6 (60·3 to 81·1)	20·4 (13·9 to 29·5)	21·1 (19·1 to 23·6)
Guinea*	2031	53·9 (47·9 to 59·8)	7·8 (6·0 to 9·8)	29·2 (24·9 to 33·5)	8·6 (5·9 to 12·6)	8·3 (7·4 to 9·3)	132·9 (118·3 to 147·6)	19·2 (14·8 to 24·3)	72·0 (61·5 to 82·7)	21·3 (14·5 to 31·0)	20·4 (18·3 to 23·0)
Guinea*	2032	54·9 (48·8 to 61·3)	8·1 (6·2 to 10·3)	29·7 (25·4 to 34·2)	9·0 (6·1 to 13·2)	8·0 (7·1 to 9·2)	135·4 (120·4 to 151·2)	20·0 (15·4 to 25·3)	73·4 (62·6 to 84·4)	22·3 (15·2 to 32·5)	19·8 (17·6 to 22·6)
Guinea*	2033	56·0 (49·7 to 62·5)	8·4 (6·5 to 10·7)	30·3 (25·9 to 34·9)	9·4 (6·4 to 13·7)	7·8 (6·9 to 9·1)	138·1 (122·7 to 154·3)	20·8 (15·9 to 26·4)	74·8 (63·8 to 86·0)	23·2 (15·8 to 33·9)	19·3 (17·0 to 22·4)
Guinea*	2034	57·1 (50·7 to 63·8)	8·7 (6·7 to 11·1)	30·9 (26·4 to 35·5)	9·8 (6·7 to 14·3)	7·6 (6·7 to 8·9)	140·8 (125·2 to 157·4)	21·6 (16·5 to 27·4)	76·2 (65·1 to 87·7)	24·2 (16·6 to 35·3)	18·8 (16·4 to 22·1)
Guinea*	2035	58·2 (51·6 to 65·2)	9·1 (7·0 to 11·6)	31·5 (26·9 to 36·2)	10·2 (7·0 to 14·9)	7·4 (6·5 to 8·8)	143·7 (127·2 to 160·8)	22·4 (17·2 to 28·5)	77·7 (66·3 to 89·4)	25·3 (17·2 to 36·9)	18·4 (15·9 to 21·8)
Guinea*	2036	59·4 (52·5 to 66·6)	9·4 (7·2 to 12·0)	32·0 (27·4 to 36·9)	10·7 (7·3 to 15·6)	7·3 (6·2 to 8·8)	146·6 (129·7 to 164·2)	23·2 (17·8 to 29·6)	79·1 (67·6 to 91·1)	26·4 (17·9 to 38·4)	18·0 (15·4 to 21·7)
Guinea*	2037	60·6 (53·6 to 67·9)	9·7 (7·5 to 12·4)	32·6 (27·9 to 37·6)	11·1 (7·5 to 16·3)	7·1 (6·1 to 8·7)	149·7 (132·3 to 167·6)	24·0 (18·5 to 30·7)	80·6 (68·8 to 92·8)	27·5 (18·6 to 40·2)	17·6 (15·0 to 21·6)
Guinea*	2038	61·9 (54·6 to 69·5)	10·1 (7·8 to 12·9)	33·2 (28·4 to 38·3)	11·6 (7·8 to 16·9)	7·0 (5·9 to 8·7)	152·8 (134·7 to 171·5)	24·9 (19·2 to 31·8)	82·0 (70·1 to 94·5)	28·6 (19·3 to 41·8)	17·3 (14·6 to 21·5)
Guinea*	2039	63·3 (55·7 to 71·0)	10·4 (8·1 to 13·3)	33·9 (28·9 to 39·0)	12·1 (8·2 to 17·7)	6·9 (5·7 to 8·7)	156·1 (137·5 to 175·3)	25·8 (19·9 to 32·9)	83·5 (71·4 to 96·2)	29·8 (20·1 to 43·7)	17·0 (14·2 to 21·5)
Guinea*	2040	64·6 (56·8 to 72·7)	10·8 (8·3 to 13·8)	34·5 (29·5 to 39·7)	12·6 (8·5 to 18·5)	6·8 (5·6 to 8·8)	159·5 (140·2 to 179·4)	26·6 (20·6 to 34·0)	85·1 (72·8 to 97·9)	31·1 (21·0 to 45·6)	16·7 (13·8 to 21·7)
Guinea*	2041	66·1 (58·0 to 74·6)	11·2 (8·6 to 14·3)	35·1 (30·0 to 40·5)	13·1 (8·8 to 19·3)	6·7 (5·4 to 8·8)	163·0 (143·0 to 184·0)	27·5 (21·2 to 35·2)	86·7 (74·1 to 99·8)	32·4 (21·8 to 47·7)	16·5 (13·4 to 21·7)
Guinea*	2042	67·6 (59·1 to 76·4)	11·5 (8·9 to 14·8)	35·8 (30·6 to 41·2)	13·7 (9·2 to 20·2)	6·6 (5·3 to 8·8)	166·8 (146·0 to 188·7)	28·5 (21·9 to 36·4)	88·3 (75·4 to 101·7)	33·7 (22·7 to 49·8)	16·3 (13·1 to 21·8)

Guinea*	2043	69·1 (60·4 to 78·2)	11·9 (9·2 to 15·2)	36·4 (31·1 to 42·0)	14·2 (9·5 to 21·0)	6·5 (5·2 to 8·9)	170·5 (149·1 to 193·1)	29·4 (22·6 to 37·6)	89·9 (76·8 to 103·7)	35·1 (23·4 to 51·9)	16·1 (12·8 to 21·8)
Guinea*	2044	70·7 (61·7 to 80·2)	12·3 (9·5 to 15·8)	37·1 (31·7 to 42·8)	14·8 (9·9 to 21·9)	6·5 (5·1 to 8·9)	174·5 (152·4 to 197·9)	30·3 (23·4 to 38·9)	91·6 (78·2 to 105·7)	36·5 (24·3 to 54·1)	16·0 (12·6 to 22·1)
Guinea*	2045	72·4 (63·1 to 81·9)	12·7 (9·8 to 16·3)	37·8 (32·3 to 43·6)	15·4 (10·2 to 22·9)	6·4 (5·0 to 8·9)	178·6 (155·7 to 202·2)	31·3 (24·1 to 40·2)	93·3 (79·7 to 107·7)	38·1 (25·2 to 56·4)	15·8 (12·4 to 22·0)
Guinea*	2046	74·1 (64·5 to 84·0)	13·1 (10·1 to 16·8)	38·5 (32·9 to 44·4)	16·1 (10·6 to 23·9)	6·4 (4·9 to 9·0)	182·8 (159·1 to 207·4)	32·3 (24·9 to 41·5)	95·1 (81·1 to 109·7)	39·7 (26·2 to 58·9)	15·7 (12·1 to 22·1)
Guinea*	2047	75·8 (65·9 to 86·3)	13·5 (10·4 to 17·4)	39·3 (33·5 to 45·3)	16·7 (10·9 to 24·9)	6·3 (4·8 to 9·0)	187·2 (162·7 to 212·9)	33·4 (25·6 to 42·8)	96·9 (82·6 to 111·8)	41·3 (27·0 to 61·5)	15·5 (12·0 to 22·3)
Guinea*	2048	77·6 (67·5 to 88·6)	13·9 (10·7 to 17·9)	40·0 (34·1 to 46·2)	17·4 (11·3 to 26·1)	6·3 (4·8 to 9·1)	191·6 (166·5 to 218·7)	34·4 (26·4 to 44·2)	98·8 (84·1 to 114·0)	43·0 (27·8 to 64·3)	15·4 (11·7 to 22·4)
Guinea*	2049	79·5 (69·0 to 90·9)	14·4 (11·0 to 18·5)	40·8 (34·7 to 47·1)	18·1 (11·6 to 27·2)	6·2 (4·7 to 9·1)	196·2 (170·3 to 224·3)	35·4 (27·2 to 45·5)	100·7 (85·7 to 116·3)	44·8 (28·7 to 67·2)	15·4 (11·6 to 22·5)
Guinea*	2050	81·4 (70·6 to 93·2)	14·8 (11·3 to 19·0)	41·6 (35·4 to 48·0)	18·9 (12·0 to 28·4)	6·2 (4·6 to 9·3)	200·9 (174·2 to 230·0)	36·5 (28·0 to 46·9)	102·5 (87·4 to 118·4)	46·6 (29·7 to 70·1)	15·3 (11·5 to 23·0)
The Gambia*	1995	14·3 (12·5 to 16·3)	5·9 (4·8 to 7·1)	5·7 (4·5 to 7·2)	1·9 (1·3 to 2·7)	0·8 (0·8 to 0·8)	42·8 (37·4 to 48·8)	17·6 (14·3 to 21·3)	17·2 (13·4 to 21·5)	5·7 (3·9 to 8·0)	2·3 (2·3 to 2·3)
The Gambia*	1996	14·5 (12·7 to 16·6)	5·9 (4·7 to 7·1)	5·9 (4·7 to 7·4)	1·9 (1·3 to 2·7)	0·8 (0·8 to 0·8)	43·5 (38·2 to 49·7)	17·6 (14·2 to 21·4)	17·7 (14·0 to 22·3)	5·8 (3·9 to 8·1)	2·4 (2·4 to 2·4)
The Gambia*	1997	15·2 (13·4 to 17·2)	5·8 (4·6 to 6·9)	5·9 (4·7 to 7·3)	1·9 (1·3 to 2·7)	1·6 (1·6 to 1·6)	45·5 (40·3 to 51·7)	17·2 (13·9 to 20·8)	17·6 (14·0 to 21·8)	5·8 (3·8 to 8·1)	4·9 (4·9 to 4·9)
The Gambia*	1998	17·1 (15·5 to 19·2)	5·6 (4·6 to 6·8)	6·0 (4·7 to 7·4)	1·9 (1·3 to 2·6)	3·7 (3·7 to 3·7)	51·4 (46·4 to 57·6)	16·7 (13·7 to 20·3)	17·9 (14·2 to 22·2)	5·7 (3·8 to 7·9)	11·1 (11·1 to 11·1)
The Gambia*	1999	16·9 (15·2 to 18·8)	5·4 (4·4 to 6·6)	6·1 (4·9 to 7·5)	1·9 (1·3 to 2·7)	3·5 (3·5 to 3·5)	50·6 (45·7 to 56·5)	16·3 (13·3 to 19·8)	18·3 (14·7 to 22·4)	5·7 (3·9 to 8·0)	10·4 (10·4 to 10·4)
The Gambia*	2000	17·0 (15·5 to 18·8)	5·1 (4·2 to 6·3)	6·2 (5·2 to 7·4)	1·8 (1·2 to 2·6)	3·9 (3·9 to 3·9)	51·1 (46·4 to 56·5)	15·4 (12·6 to 18·8)	18·5 (15·5 to 22·3)	5·5 (3·7 to 7·9)	11·6 (11·6 to 11·6)
The Gambia*	2001	16·6 (15·1 to 18·2)	5·0 (4·2 to 6·2)	6·0 (5·1 to 7·1)	1·8 (1·2 to 2·6)	3·8 (3·8 to 3·8)	49·7 (45·4 to 54·7)	15·1 (12·6 to 18·5)	18·0 (15·4 to 21·3)	5·4 (3·7 to 7·7)	11·3 (11·3 to 11·3)
The Gambia*	2002	18·8 (17·6 to 20·2)	4·4 (3·6 to 5·3)	5·5 (4·7 to 6·3)	1·7 (1·2 to 2·4)	7·3 (7·3 to 7·3)	56·5 (52·8 to 60·6)	13·2 (10·9 to 15·9)	16·4 (14·1 to 19·0)	5·1 (3·6 to 7·2)	21·8 (21·8 to 21·8)

The Gambia*	2003	19·9 (18·6 to 21·3)	4·7 (3·9 to 5·7)	5·1 (4·4 to 5·9)	1·8 (1·3 to 2·6)	8·2 (8·2 to 8·2)	59·7 (55·7 to 63·9)	14·2 (11·7 to 17·1)	15·4 (13·3 to 17·7)	5·5 (3·8 to 7·8)	24·6 (24·6 to 24·6)
The Gambia*	2004	26·8 (25·3 to 28·5)	6·1 (5·0 to 7·3)	5·3 (4·5 to 6·1)	2·2 (1·5 to 3·0)	13·4 (13·4 to 13·4)	80·5 (75·9 to 85·5)	18·2 (14·9 to 21·8)	15·8 (13·4 to 18·3)	6·5 (4·6 to 9·1)	40·1 (40·1 to 40·1)
The Gambia*	2005	30·6 (29·0 to 32·3)	6·1 (5·0 to 7·3)	5·2 (4·4 to 6·2)	2·1 (1·5 to 3·1)	17·1 (17·1 to 17·1)	91·8 (87·0 to 96·9)	18·4 (15·1 to 22·0)	15·7 (13·2 to 18·5)	6·4 (4·5 to 9·4)	51·3 (51·3 to 51·3)
The Gambia*	2006	24·5 (22·7 to 26·3)	6·4 (5·2 to 7·7)	5·5 (4·5 to 6·6)	2·1 (1·5 to 3·1)	10·5 (10·5 to 10·5)	73·5 (68·2 to 79·0)	19·2 (15·7 to 23·1)	16·5 (13·5 to 19·7)	6·4 (4·4 to 9·2)	31·5 (31·5 to 31·5)
The Gambia*	2007	24·4 (22·8 to 26·0)	5·6 (4·6 to 6·7)	5·4 (4·3 to 6·5)	1·9 (1·3 to 2·8)	11·5 (11·5 to 11·5)	73·1 (68·3 to 78·1)	16·6 (13·7 to 20·1)	16·1 (13·0 to 19·6)	5·8 (4·0 to 8·3)	34·5 (34·5 to 34·5)
The Gambia*	2008	23·5 (21·8 to 25·3)	5·8 (4·7 to 7·0)	5·6 (4·5 to 6·8)	1·9 (1·3 to 2·7)	10·2 (10·2 to 10·2)	70·5 (65·5 to 76·0)	17·3 (14·2 to 21·1)	16·8 (13·4 to 20·4)	5·8 (4·0 to 8·2)	30·7 (30·7 to 30·7)
The Gambia*	2009	26·8 (24·9 to 28·8)	6·9 (5·7 to 8·4)	6·0 (4·9 to 7·3)	2·0 (1·4 to 2·9)	11·9 (11·9 to 11·9)	80·4 (74·8 to 86·3)	20·8 (17·0 to 25·3)	18·1 (14·6 to 22·0)	6·0 (4·1 to 8·7)	35·6 (35·6 to 35·6)
The Gambia*	2010	31·8 (30·0 to 33·7)	7·2 (6·0 to 8·6)	6·1 (5·0 to 7·4)	1·9 (1·3 to 2·8)	16·6 (16·6 to 16·6)	95·4 (90·1 to 101·2)	21·5 (17·9 to 25·9)	18·3 (15·0 to 22·3)	5·7 (4·0 to 8·3)	49·8 (49·8 to 49·8)
The Gambia*	2011	33·8 (32·1 to 35·7)	7·4 (6·2 to 8·7)	6·2 (5·1 to 7·4)	1·8 (1·2 to 2·6)	18·4 (18·4 to 18·4)	101·4 (96·4 to 107·0)	22·3 (18·6 to 26·2)	18·5 (15·3 to 22·1)	5·3 (3·7 to 7·7)	55·3 (55·3 to 55·3)
The Gambia*	2012	35·9 (34·3 to 37·7)	8·2 (7·0 to 9·6)	6·4 (5·4 to 7·5)	1·8 (1·2 to 2·6)	19·5 (19·5 to 19·5)	107·6 (102·7 to 113·0)	24·6 (20·9 to 28·7)	19·1 (16·1 to 22·5)	5·3 (3·7 to 7·7)	58·5 (58·5 to 58·5)
The Gambia*	2013	39·5 (38·1 to 41·1)	7·8 (6·7 to 8·9)	6·2 (5·3 to 7·1)	1·7 (1·2 to 2·5)	23·8 (23·8 to 23·8)	118·4 (114·2 to 123·2)	23·3 (20·1 to 26·7)	18·6 (16·0 to 21·4)	5·2 (3·6 to 7·5)	71·3 (71·3 to 71·3)
The Gambia*	2014	32·5 (31·2 to 34·0)	7·6 (6·6 to 8·7)	6·1 (5·4 to 6·9)	1·9 (1·3 to 2·6)	16·9 (16·9 to 16·9)	97·5 (93·6 to 102·0)	22·8 (19·9 to 26·0)	18·4 (16·1 to 20·8)	5·6 (3·8 to 7·9)	50·7 (50·7 to 50·7)
The Gambia*	2015	30·7 (29·4 to 32·2)	7·6 (6·7 to 8·6)	6·2 (5·4 to 7·0)	1·8 (1·2 to 2·6)	15·1 (15·1 to 15·1)	92·1 (88·2 to 96·5)	22·9 (20·1 to 25·9)	18·5 (16·3 to 20·9)	5·4 (3·7 to 7·7)	45·4 (45·4 to 45·4)
The Gambia*	2016	31·5 (30·1 to 32·8)	7·6 (6·6 to 8·6)	6·5 (5·7 to 7·3)	1·3 (0·9 to 1·8)	16·2 (16·2 to 16·2)	94·4 (90·4 to 98·5)	22·7 (19·9 to 25·8)	19·3 (17·2 to 21·8)	3·8 (2·6 to 5·5)	48·5 (48·5 to 48·5)
The Gambia*	2017	46·2 (44·7 to 47·8)	8·2 (7·1 to 9·5)	6·8 (5·9 to 7·8)	1·3 (0·9 to 1·8)	29·9 (29·9 to 29·9)	138·6 (134·0 to 143·3)	24·7 (21·4 to 28·3)	20·4 (17·8 to 23·3)	3·8 (2·6 to 5·4)	89·7 (89·7 to 89·7)
The Gambia*	2018	38·8 (37·3 to 40·5)	7·7 (6·5 to 8·9)	6·8 (5·8 to 7·9)	1·2 (0·8 to 1·8)	23·2 (23·2 to 23·2)	116·5 (111·9 to 121·4)	23·0 (19·6 to 26·7)	20·3 (17·3 to 23·7)	3·6 (2·5 to 5·3)	69·5 (69·5 to 69·5)

The Gambia*	2019	39·4 (37·7 to 41·2)	8·5 (7·2 to 9·9)	6·9 (5·9 to 8·1)	1·3 (0·9 to 1·8)	22·7 (22·4 to 22·9)	118·1 (113·1 to 123·7)	25·5 (21·6 to 29·7)	20·8 (17·7 to 24·2)	3·8 (2·6 to 5·5)	68·0 (67·3 to 68·7)
The Gambia*	2020	48·8 (46·7 to 51·2)	8·9 (7·6 to 10·4)	6·4 (5·5 to 7·5)	1·2 (0·8 to 1·7)	32·2 (31·4 to 33·9)	146·3 (140·1 to 153·4)	26·8 (22·8 to 31·3)	19·2 (16·4 to 22·4)	3·6 (2·5 to 5·2)	96·7 (94·2 to 101·6)
The Gambia*	2021	47·7 (45·0 to 51·1)	8·6 (7·3 to 10·0)	6·1 (5·2 to 7·1)	1·2 (0·8 to 1·7)	31·8 (30·1 to 34·8)	142·9 (134·9 to 153·2)	25·7 (21·9 to 30·0)	18·4 (15·7 to 21·4)	3·5 (2·4 to 5·1)	95·4 (90·4 to 104·4)
The Gambia*	2022	39·0 (36·3 to 42·1)	7·7 (6·5 to 9·0)	6·2 (5·3 to 7·2)	1·2 (0·8 to 1·7)	23·9 (21·8 to 26·6)	117·1 (109·0 to 126·2)	23·2 (19·6 to 27·1)	18·7 (15·9 to 21·7)	3·6 (2·5 to 5·2)	71·6 (65·4 to 79·8)
The Gambia*	2023	39·1 (36·3 to 42·3)	7·9 (6·7 to 9·4)	6·3 (5·4 to 7·4)	1·2 (0·9 to 1·8)	23·6 (21·5 to 26·4)	117·4 (109·0 to 126·7)	23·8 (20·1 to 28·2)	19·0 (16·1 to 22·1)	3·7 (2·6 to 5·4)	70·9 (64·4 to 79·1)
The Gambia*	2024	39·3 (36·4 to 42·6)	8·2 (6·9 to 9·8)	6·4 (5·5 to 7·5)	1·3 (0·9 to 1·8)	23·4 (21·1 to 26·1)	117·8 (109·3 to 127·6)	24·5 (20·6 to 29·3)	19·3 (16·4 to 22·4)	3·8 (2·7 to 5·5)	70·1 (63·3 to 78·4)
The Gambia*	2025	39·3 (36·4 to 42·6)	8·4 (7·0 to 10·0)	6·5 (5·6 to 7·6)	1·3 (0·9 to 1·9)	23·0 (20·8 to 25·9)	117·9 (109·2 to 127·9)	25·2 (21·1 to 30·0)	19·6 (16·7 to 22·8)	4·0 (2·8 to 5·7)	69·1 (62·3 to 77·8)
The Gambia*	2026	39·3 (36·3 to 42·7)	8·6 (7·2 to 10·3)	6·6 (5·7 to 7·7)	1·4 (1·0 to 2·0)	22·7 (20·4 to 25·6)	117·9 (108·9 to 127·9)	25·8 (21·6 to 30·9)	19·9 (17·0 to 23·2)	4·1 (2·9 to 5·9)	68·0 (61·1 to 76·9)
The Gambia*	2027	39·3 (36·3 to 42·7)	8·8 (7·3 to 10·5)	6·8 (5·7 to 7·9)	1·4 (1·0 to 2·1)	22·3 (20·0 to 25·3)	117·8 (108·9 to 128·1)	26·3 (22·0 to 31·6)	20·2 (17·2 to 23·6)	4·3 (3·0 to 6·2)	67·0 (60·1 to 75·9)
The Gambia*	2028	39·3 (36·2 to 42·9)	8·9 (7·5 to 10·7)	6·9 (5·8 to 8·0)	1·5 (1·0 to 2·1)	22·0 (19·7 to 25·0)	117·8 (108·7 to 128·7)	26·8 (22·4 to 32·1)	20·6 (17·5 to 23·9)	4·4 (3·1 to 6·4)	66·0 (59·0 to 75·0)
The Gambia*	2029	39·3 (36·1 to 42·9)	9·1 (7·6 to 10·9)	7·0 (5·9 to 8·1)	1·5 (1·1 to 2·2)	21·7 (19·3 to 24·8)	117·9 (108·3 to 128·7)	27·2 (22·8 to 32·6)	20·9 (17·8 to 24·3)	4·6 (3·2 to 6·7)	65·1 (57·9 to 74·3)
The Gambia*	2030	39·3 (36·0 to 42·8)	9·2 (7·7 to 11·0)	7·1 (6·0 to 8·2)	1·6 (1·1 to 2·3)	21·4 (18·9 to 24·5)	117·8 (108·0 to 128·4)	27·6 (23·1 to 33·1)	21·2 (18·1 to 24·7)	4·8 (3·3 to 6·9)	64·1 (56·7 to 73·5)
The Gambia*	2031	39·3 (35·9 to 42·9)	9·4 (7·8 to 11·2)	7·2 (6·1 to 8·4)	1·7 (1·2 to 2·4)	21·1 (18·6 to 24·2)	117·9 (107·7 to 128·8)	28·1 (23·4 to 33·6)	21·6 (18·4 to 25·1)	5·0 (3·5 to 7·2)	63·3 (55·9 to 72·4)
The Gambia*	2032	39·4 (36·0 to 43·1)	9·5 (7·9 to 11·4)	7·3 (6·2 to 8·5)	1·7 (1·2 to 2·5)	20·9 (18·3 to 23·9)	118·1 (107·8 to 129·2)	28·5 (23·8 to 34·1)	21·9 (18·7 to 25·5)	5·2 (3·6 to 7·5)	62·6 (54·8 to 71·7)

The Gambia*	2033	39·5 (36·1 to 43·2)	9·6 (8·0 to 11·6)	7·4 (6·3 to 8·6)	1·8 (1·2 to 2·6)	20·7 (18·2 to 23·8)	118·5 (108·2 to 129·7)	28·9 (24·1 to 34·7)	22·2 (18·9 to 25·8)	5·4 (3·7 to 7·8)	62·0 (54·5 to 71·3)
The Gambia*	2034	39·7 (36·3 to 43·5)	9·8 (8·2 to 11·8)	7·5 (6·4 to 8·7)	1·9 (1·3 to 2·7)	20·5 (18·0 to 23·7)	119·0 (108·8 to 130·3)	29·3 (24·4 to 35·3)	22·5 (19·2 to 26·2)	5·6 (3·9 to 8·1)	61·6 (53·9 to 71·1)
The Gambia*	2035	39·9 (36·4 to 43·9)	9·9 (8·3 to 12·0)	7·6 (6·5 to 8·9)	1·9 (1·4 to 2·8)	20·4 (17·8 to 23·7)	119·7 (109·3 to 131·7)	29·7 (24·8 to 35·9)	22·9 (19·5 to 26·6)	5·8 (4·1 to 8·4)	61·3 (53·5 to 71·0)
The Gambia*	2036	40·1 (36·6 to 44·2)	10·1 (8·4 to 12·2)	7·7 (6·6 to 9·0)	2·0 (1·4 to 2·9)	20·3 (17·6 to 23·7)	120·4 (109·8 to 132·6)	30·2 (25·1 to 36·4)	23·2 (19·8 to 27·0)	6·0 (4·2 to 8·8)	61·0 (52·9 to 71·0)
The Gambia*	2037	40·4 (36·8 to 44·5)	10·2 (8·5 to 12·4)	7·8 (6·7 to 9·1)	2·1 (1·5 to 3·0)	20·3 (17·6 to 23·6)	121·2 (110·5 to 133·4)	30·6 (25·5 to 37·1)	23·5 (20·0 to 27·4)	6·3 (4·4 to 9·1)	60·7 (52·9 to 70·7)
The Gambia*	2038	40·7 (37·1 to 44·9)	10·4 (8·6 to 12·6)	8·0 (6·8 to 9·3)	2·2 (1·5 to 3·2)	20·2 (17·5 to 23·6)	122·1 (111·3 to 134·6)	31·1 (25·8 to 37·7)	23·9 (20·3 to 27·8)	6·6 (4·6 to 9·5)	60·6 (52·4 to 70·7)
The Gambia*	2039	41·2 (37·5 to 45·4)	10·5 (8·7 to 12·8)	8·1 (6·9 to 9·4)	2·3 (1·6 to 3·3)	20·3 (17·5 to 23·8)	123·4 (112·4 to 136·3)	31·6 (26·2 to 38·3)	24·2 (20·6 to 28·1)	6·8 (4·8 to 9·9)	60·8 (52·6 to 71·5)
The Gambia*	2040	41·6 (37·9 to 46·3)	10·7 (8·9 to 13·0)	8·2 (6·9 to 9·5)	2·4 (1·6 to 3·4)	20·4 (17·6 to 24·1)	124·9 (113·6 to 138·8)	32·1 (26·6 to 38·9)	24·5 (20·8 to 28·5)	7·1 (4·9 to 10·3)	61·2 (52·9 to 72·2)
The Gambia*	2041	42·2 (38·3 to 46·9)	10·9 (9·0 to 13·2)	8·3 (7·0 to 9·6)	2·5 (1·7 to 3·6)	20·6 (17·6 to 24·4)	126·5 (114·8 to 140·6)	32·5 (26·9 to 39·5)	24·8 (21·1 to 28·8)	7·4 (5·1 to 10·8)	61·7 (52·7 to 73·1)
The Gambia*	2042	42·7 (38·8 to 47·6)	11·0 (9·1 to 13·4)	8·4 (7·1 to 9·7)	2·6 (1·8 to 3·7)	20·8 (17·8 to 24·8)	128·2 (116·3 to 142·7)	33·0 (27·2 to 40·1)	25·1 (21·4 to 29·1)	7·7 (5·3 to 11·2)	62·4 (53·4 to 74·3)
The Gambia*	2043	43·4 (39·3 to 48·5)	11·2 (9·2 to 13·6)	8·4 (7·2 to 9·8)	2·7 (1·8 to 3·9)	21·1 (17·9 to 25·2)	130·1 (117·8 to 145·4)	33·6 (27·6 to 40·8)	25·3 (21·6 to 29·4)	8·1 (5·5 to 11·7)	63·1 (53·8 to 75·6)
The Gambia*	2044	44·1 (39·9 to 49·2)	11·4 (9·3 to 13·8)	8·5 (7·3 to 9·9)	2·8 (1·9 to 4·1)	21·3 (18·2 to 25·5)	132·1 (119·6 to 147·6)	34·1 (28·0 to 41·5)	25·6 (21·9 to 29·8)	8·4 (5·6 to 12·3)	64·0 (54·7 to 76·5)
The Gambia*	2045	44·7 (40·4 to 50·0)	11·6 (9·5 to 14·1)	8·6 (7·4 to 10·0)	2·9 (1·9 to 4·3)	21·6 (18·5 to 26·0)	134·2 (121·3 to 149·9)	34·7 (28·5 to 42·2)	25·9 (22·1 to 30·1)	8·8 (5·8 to 12·9)	64·8 (55·4 to 78·1)
The Gambia*	2046	45·5 (41·1 to 50·8)	11·7 (9·7 to 14·3)	8·7 (7·4 to 10·2)	3·0 (2·0 to 4·5)	21·9 (18·6 to 26·3)	136·4 (123·3 to 152·4)	35·2 (29·0 to 42·9)	26·2 (22·3 to 30·4)	9·1 (6·0 to 13·5)	65·8 (55·7 to 79·0)

The Gambia*	2047	46·3 (41·7 to 51·9)	12·0 (9·8 to 14·6)	8·8 (7·5 to 10·3)	3·2 (2·1 to 4·7)	22·3 (18·7 to 27·0)	138·8 (125·0 to 155·8)	35·9 (29·5 to 43·7)	26·5 (22·5 to 30·8)	9·5 (6·2 to 14·1)	66·9 (56·2 to 81·1)
The Gambia*	2048	47·1 (42·4 to 52·8)	12·2 (10·0 to 14·8)	8·9 (7·6 to 10·4)	3·3 (2·1 to 4·9)	22·7 (19·2 to 27·5)	141·3 (127·3 to 158·4)	36·5 (30·0 to 44·4)	26·8 (22·8 to 31·1)	9·9 (6·4 to 14·8)	68·2 (57·6 to 82·4)
The Gambia*	2049	48·0 (43·1 to 53·9)	12·4 (10·2 to 15·1)	9·0 (7·7 to 10·5)	3·4 (2·2 to 5·2)	23·2 (19·5 to 28·2)	144·1 (129·2 to 161·8)	37·1 (30·5 to 45·2)	27·1 (23·0 to 31·4)	10·3 (6·6 to 15·5)	69·6 (58·4 to 84·6)
The Gambia*	2050	49·0 (44·1 to 55·2)	12·6 (10·3 to 15·3)	9·1 (7·7 to 10·6)	3·6 (2·2 to 5·4)	23·7 (19·9 to 28·9)	147·0 (132·3 to 165·7)	37·8 (31·0 to 46·0)	27·3 (23·2 to 31·7)	10·7 (6·7 to 16·2)	71·2 (59·8 to 86·8)
Guinea-Bissau*	1995	55·8 (51·4 to 60·8)	7·5 (6·2 to 9·2)	20·6 (16·4 to 25·3)	1·6 (1·1 to 2·3)	26·1 (26·1 to 26·1)	170·3 (156·7 to 185·7)	23·0 (18·9 to 28·1)	62·9 (50·0 to 77·3)	4·9 (3·3 to 6·9)	79·5 (79·5 to 79·5)
Guinea-Bissau*	1996	33·7 (29·4 to 38·6)	7·8 (6·4 to 9·6)	20·1 (16·0 to 24·7)	1·6 (1·1 to 2·3)	4·3 (4·3 to 4·3)	103·0 (89·8 to 117·8)	23·9 (19·5 to 29·2)	61·2 (48·9 to 75·4)	4·9 (3·3 to 6·9)	13·0 (13·0 to 13·0)
Guinea-Bissau*	1997	35·8 (31·7 to 40·8)	9·4 (7·7 to 11·5)	19·2 (15·3 to 23·6)	1·7 (1·2 to 2·5)	5·5 (5·5 to 5·5)	109·3 (96·8 to 124·6)	28·6 (23·4 to 34·9)	58·5 (46·7 to 72·0)	5·2 (3·6 to 7·5)	16·9 (16·9 to 16·9)
Guinea-Bissau*	1998	30·9 (27·6 to 34·8)	7·8 (6·4 to 9·4)	15·1 (12·0 to 18·5)	1·5 (1·0 to 2·1)	6·5 (6·5 to 6·5)	94·3 (84·3 to 106·1)	23·8 (19·5 to 28·8)	46·0 (36·6 to 56·5)	4·6 (3·2 to 6·5)	19·9 (19·9 to 19·9)
Guinea-Bissau*	1999	26·7 (23·5 to 30·5)	10·1 (8·3 to 12·2)	14·2 (11·4 to 17·4)	1·6 (1·1 to 2·2)	0·8 (0·8 to 0·8)	81·5 (71·7 to 93·2)	30·8 (25·2 to 37·2)	43·4 (34·9 to 53·2)	4·8 (3·3 to 6·8)	2·6 (2·6 to 2·6)
Guinea-Bissau*	2000	36·4 (32·5 to 40·8)	15·3 (12·5 to 18·6)	15·2 (12·1 to 18·4)	1·7 (1·2 to 2·4)	4·2 (4·2 to 4·2)	111·1 (99·0 to 124·6)	46·7 (38·2 to 56·7)	46·4 (37·0 to 56·3)	5·2 (3·6 to 7·2)	12·9 (12·9 to 12·9)
Guinea-Bissau*	2001	37·9 (33·9 to 42·0)	14·5 (11·8 to 17·6)	14·6 (11·8 to 17·7)	1·6 (1·1 to 2·3)	7·2 (7·2 to 7·2)	115·6 (103·6 to 128·3)	44·2 (36·1 to 53·7)	44·6 (36·0 to 54·1)	5·0 (3·4 to 6·9)	21·8 (21·8 to 21·8)
Guinea-Bissau*	2002	32·8 (29·5 to 36·7)	11·9 (9·8 to 14·5)	13·5 (10·9 to 16·5)	1·5 (1·0 to 2·1)	5·9 (5·9 to 5·9)	100·2 (90·1 to 112·0)	36·4 (29·9 to 44·3)	41·2 (33·3 to 50·2)	4·6 (3·1 to 6·4)	18·0 (18·0 to 18·0)
Guinea-Bissau*	2003	35·4 (31·7 to 39·7)	13·7 (11·3 to 16·6)	13·9 (11·2 to 17·1)	1·5 (1·0 to 2·1)	6·2 (6·2 to 6·2)	108·1 (96·7 to 121·1)	41·9 (34·4 to 50·8)	42·5 (34·3 to 52·1)	4·7 (3·2 to 6·5)	18·9 (18·9 to 18·9)
Guinea-Bissau*	2004	42·0 (37·8 to 47·1)	16·8 (13·8 to 20·4)	15·0 (12·1 to 18·3)	1·7 (1·1 to 2·3)	8·6 (8·6 to 8·6)	128·3 (115·3 to 143·8)	51·2 (42·0 to 62·3)	45·7 (36·9 to 56·0)	5·1 (3·5 to 7·1)	26·2 (26·2 to 26·2)

Guinea-Bissau*	2005	41·3 (37·2 to 46·2)	15·3 (12·5 to 18·6)	14·8 (11·9 to 18·3)	1·6 (1·1 to 2·3)	9·6 (9·6 to 9·6)	126·1 (113·7 to 141·0)	46·6 (38·2 to 56·8)	45·2 (36·3 to 55·7)	5·0 (3·4 to 7·0)	29·3 (29·3 to 29·3)
Guinea-Bissau*	2006	41·9 (37·9 to 46·2)	14·1 (11·5 to 17·2)	15·1 (12·2 to 18·5)	1·7 (1·1 to 2·4)	11·0 (11·0 to 11·0)	127·8 (115·6 to 141·0)	43·0 (35·1 to 52·4)	46·0 (37·1 to 56·6)	5·1 (3·5 to 7·2)	33·7 (33·7 to 33·7)
Guinea-Bissau*	2007	47·6 (43·4 to 52·2)	14·5 (12·0 to 17·6)	16·1 (13·1 to 19·9)	1·8 (1·2 to 2·5)	15·2 (15·2 to 15·2)	145·4 (132·6 to 159·4)	44·3 (36·5 to 53·6)	49·2 (39·9 to 60·8)	5·4 (3·7 to 7·7)	46·4 (46·4 to 46·4)
Guinea-Bissau*	2008	41·1 (37·1 to 45·6)	12·5 (10·2 to 15·0)	16·6 (13·4 to 20·5)	1·8 (1·2 to 2·5)	10·3 (10·3 to 10·3)	125·4 (113·3 to 139·0)	38·0 (31·3 to 45·9)	50·7 (41·0 to 62·7)	5·4 (3·6 to 7·6)	31·3 (31·3 to 31·3)
Guinea-Bissau*	2009	41·1 (37·2 to 45·7)	10·3 (8·4 to 12·4)	17·5 (14·2 to 21·6)	1·8 (1·2 to 2·6)	11·5 (11·5 to 11·5)	125·6 (113·5 to 139·3)	31·3 (25·6 to 38·0)	53·5 (43·3 to 65·8)	5·6 (3·8 to 7·8)	35·1 (35·1 to 35·1)
Guinea-Bissau*	2010	45·7 (42·0 to 49·8)	7·2 (5·9 to 8·7)	18·2 (14·8 to 22·1)	1·8 (1·2 to 2·5)	18·5 (18·5 to 18·5)	139·5 (128·0 to 151·9)	21·9 (18·0 to 26·5)	55·6 (45·2 to 67·5)	5·4 (3·7 to 7·6)	56·5 (56·5 to 56·5)
Guinea-Bissau*	2011	38·3 (34·6 to 42·2)	5·3 (4·4 to 6·4)	20·1 (16·5 to 23·8)	1·8 (1·2 to 2·5)	11·2 (11·2 to 11·2)	116·8 (105·6 to 128·6)	16·1 (13·3 to 19·4)	61·2 (50·4 to 72·7)	5·4 (3·7 to 7·5)	34·1 (34·1 to 34·1)
Guinea-Bissau*	2012	32·7 (29·2 to 36·9)	3·8 (3·1 to 4·6)	22·5 (19·2 to 26·4)	1·7 (1·1 to 2·3)	4·7 (4·7 to 4·7)	99·9 (89·1 to 112·5)	11·6 (9·6 to 13·9)	68·7 (58·5 to 80·6)	5·1 (3·4 to 7·0)	14·5 (14·5 to 14·5)
Guinea-Bissau*	2013	51·7 (48·2 to 55·7)	3·5 (2·8 to 4·2)	26·7 (23·4 to 30·7)	1·6 (1·1 to 2·3)	19·9 (19·9 to 19·9)	157·7 (147·1 to 169·9)	10·6 (8·7 to 12·7)	81·5 (71·4 to 93·6)	5·0 (3·3 to 6·9)	60·7 (60·7 to 60·7)
Guinea-Bissau*	2014	52·3 (48·8 to 56·1)	4·6 (3·7 to 5·5)	34·4 (31·1 to 37·9)	1·8 (1·2 to 2·5)	11·5 (11·5 to 11·5)	159·6 (148·8 to 171·1)	14·0 (11·3 to 16·8)	104·9 (94·9 to 115·6)	5·5 (3·7 to 7·6)	35·2 (35·2 to 35·2)
Guinea-Bissau*	2015	56·8 (54·2 to 59·3)	4·3 (3·4 to 5·1)	36·0 (33·5 to 38·4)	1·7 (1·2 to 2·4)	14·9 (14·9 to 14·9)	173·4 (165·4 to 180·9)	13·0 (10·5 to 15·6)	109·8 (102·3 to 117·2)	5·2 (3·6 to 7·2)	45·4 (45·4 to 45·4)
Guinea-Bissau*	2016	58·6 (56·5 to 60·7)	4·1 (3·3 to 4·9)	36·2 (34·3 to 38·1)	1·8 (1·2 to 2·4)	16·6 (16·6 to 16·6)	178·9 (172·4 to 185·3)	12·5 (10·1 to 15·0)	110·5 (104·5 to 116·3)	5·3 (3·7 to 7·4)	50·6 (50·6 to 50·6)
Guinea-Bissau*	2017	57·1 (54·6 to 59·7)	3·9 (3·2 to 4·7)	34·8 (32·5 to 37·2)	1·8 (1·2 to 2·4)	16·6 (16·6 to 16·6)	174·2 (166·7 to 182·1)	12·0 (9·8 to 14·5)	106·3 (99·2 to 113·5)	5·3 (3·6 to 7·3)	50·6 (50·6 to 50·6)
Guinea-Bissau*	2018	57·5 (53·8 to 61·3)	4·2 (3·5 to 5·1)	35·1 (31·8 to 38·9)	1·8 (1·2 to 2·5)	16·4 (16·4 to 16·4)	175·6 (164·3 to 187·1)	12·9 (10·5 to 15·5)	107·2 (97·0 to 118·6)	5·6 (3·8 to 7·7)	49·9 (49·9 to 49·9)

Guinea-Bissau*	2019	58·4 (54·6 to 62·2)	4·3 (3·5 to 5·2)	35·7 (32·3 to 39·6)	1·9 (1·3 to 2·6)	16·4 (16·3 to 16·6)	178·2 (166·7 to 189·9)	13·1 (10·7 to 15·9)	109·1 (98·6 to 120·9)	5·9 (3·9 to 8·1)	50·1 (49·7 to 50·6)
Guinea-Bissau*	2020	62·8 (59·0 to 66·6)	5·0 (4·0 to 6·1)	33·6 (30·3 to 37·2)	1·8 (1·2 to 2·5)	22·4 (21·8 to 23·8)	191·7 (180·0 to 203·3)	15·3 (12·3 to 18·7)	102·4 (92·5 to 113·4)	5·6 (3·8 to 7·8)	68·3 (66·4 to 72·6)
Guinea-Bissau*	2021	61·4 (57·7 to 65·0)	4·8 (3·8 to 5·8)	31·8 (28·7 to 35·2)	1·8 (1·2 to 2·5)	23·1 (22·4 to 24·3)	187·5 (176·2 to 198·3)	14·5 (11·6 to 17·8)	97·0 (87·7 to 107·5)	5·5 (3·7 to 7·5)	70·4 (68·4 to 74·1)
Guinea-Bissau*	2022	55·5 (51·9 to 59·3)	3·9 (3·1 to 4·8)	32·1 (29·0 to 35·5)	1·9 (1·2 to 2·5)	17·6 (16·6 to 19·0)	169·3 (158·2 to 181·0)	11·9 (9·5 to 14·7)	97·9 (88·4 to 108·5)	5·6 (3·8 to 7·8)	53·8 (50·6 to 57·9)
Guinea-Bissau*	2023	56·0 (52·3 to 59·9)	4·0 (3·1 to 5·0)	32·4 (29·3 to 35·9)	1·9 (1·3 to 2·6)	17·7 (16·5 to 19·1)	170·9 (159·6 to 182·9)	12·1 (9·6 to 15·1)	98·9 (89·3 to 109·5)	5·8 (3·9 to 8·0)	54·1 (50·5 to 58·3)
Guinea-Bissau*	2024	56·6 (52·8 to 60·6)	4·0 (3·2 to 5·0)	32·8 (29·6 to 36·3)	2·0 (1·3 to 2·7)	17·8 (16·5 to 19·3)	172·6 (161·1 to 184·9)	12·3 (9·6 to 15·3)	100·0 (90·3 to 110·7)	6·0 (4·1 to 8·3)	54·3 (50·3 to 58·8)
Guinea-Bissau*	2025	57·1 (53·2 to 61·2)	4·1 (3·2 to 5·2)	33·1 (29·9 to 36·7)	2·0 (1·4 to 2·8)	17·8 (16·5 to 19·2)	174·1 (162·5 to 186·7)	12·5 (9·8 to 15·9)	101·1 (91·2 to 111·9)	6·3 (4·2 to 8·5)	54·2 (50·2 to 58·7)
Guinea-Bissau*	2026	57·5 (53·6 to 61·7)	4·2 (3·2 to 5·3)	33·5 (30·2 to 37·1)	2·1 (1·4 to 2·9)	17·7 (16·3 to 19·2)	175·5 (163·6 to 188·3)	12·8 (9·8 to 16·2)	102·4 (92·3 to 113·3)	6·5 (4·4 to 8·8)	53·9 (49·7 to 58·5)
Guinea-Bissau*	2027	58·0 (54·1 to 62·2)	4·3 (3·3 to 5·4)	34·0 (30·6 to 37·6)	2·2 (1·5 to 3·0)	17·5 (16·1 to 19·0)	176·9 (165·0 to 189·9)	13·0 (10·0 to 16·6)	103·7 (93·5 to 114·8)	6·7 (4·6 to 9·2)	53·4 (49·1 to 58·1)
Guinea-Bissau*	2028	58·4 (54·4 to 62·8)	4·4 (3·3 to 5·6)	34·5 (31·1 to 38·2)	2·3 (1·5 to 3·1)	17·3 (15·8 to 19·0)	178·2 (166·0 to 191·6)	13·3 (10·2 to 17·2)	105·2 (94·8 to 116·5)	7·0 (4·7 to 9·5)	52·7 (48·2 to 57·9)
Guinea-Bissau*	2029	58·9 (54·7 to 63·3)	4·5 (3·4 to 5·7)	35·0 (31·5 to 38·8)	2·4 (1·6 to 3·3)	17·0 (15·5 to 18·7)	179·6 (166·9 to 193·1)	13·7 (10·4 to 17·5)	106·8 (96·2 to 118·3)	7·2 (4·9 to 10·0)	51·9 (47·4 to 57·1)
Guinea-Bissau*	2030	59·3 (55·0 to 63·9)	4·6 (3·4 to 5·9)	35·5 (32·0 to 39·3)	2·5 (1·7 to 3·4)	16·7 (15·2 to 18·6)	181·0 (167·9 to 195·2)	14·0 (10·5 to 18·1)	108·4 (97·7 to 120·0)	7·5 (5·1 to 10·3)	51·1 (46·4 to 56·6)
Guinea-Bissau*	2031	59·8 (55·3 to 64·4)	4·7 (3·6 to 6·2)	36·1 (32·5 to 39·9)	2·6 (1·7 to 3·5)	16·5 (14·9 to 18·3)	182·5 (168·8 to 196·7)	14·4 (10·8 to 18·8)	110·0 (99·3 to 121·8)	7·8 (5·3 to 10·8)	50·3 (45·4 to 55·8)
Guinea-Bissau*	2032	60·3 (55·9 to 65·1)	4·8 (3·6 to 6·4)	36·6 (33·1 to 40·5)	2·7 (1·8 to 3·7)	16·2 (14·6 to 17·9)	184·1 (170·6 to 198·7)	14·8 (11·1 to 19·4)	111·8 (100·9 to 123·7)	8·1 (5·5 to 11·2)	49·4 (44·7 to 54·7)

Guinea-Bissau*	2033	60·9 (56·3 to 65·8)	5·0 (3·7 to 6·6)	37·2 (33·6 to 41·2)	2·8 (1·9 to 3·9)	15·9 (14·3 to 17·6)	185·8 (171·9 to 200·7)	15·2 (11·4 to 20·2)	113·7 (102·7 to 125·8)	8·5 (5·6 to 11·8)	48·5 (43·8 to 53·7)
Guinea-Bissau*	2034	61·5 (56·9 to 66·4)	5·1 (3·8 to 6·9)	37·9 (34·2 to 41·9)	2·9 (1·9 to 4·0)	15·6 (14·1 to 17·4)	187·7 (173·6 to 202·5)	15·7 (11·5 to 21·1)	115·6 (104·4 to 127·9)	8·8 (5·9 to 12·2)	47·6 (42·9 to 53·1)
Guinea-Bissau*	2035	62·2 (57·4 to 67·1)	5·3 (3·9 to 7·1)	38·5 (34·8 to 42·6)	3·0 (2·0 to 4·2)	15·3 (13·8 to 17·2)	189·7 (175·1 to 204·8)	16·2 (12·0 to 21·6)	117·5 (106·2 to 130·0)	9·2 (6·1 to 12·8)	46·7 (42·1 to 52·4)
Guinea-Bissau*	2036	62·8 (57·9 to 67·7)	5·5 (4·0 to 7·4)	39·2 (35·4 to 43·3)	3·2 (2·1 to 4·4)	15·0 (13·5 to 17·0)	191·8 (176·7 to 206·6)	16·7 (12·4 to 22·7)	119·6 (107·9 to 132·3)	9·6 (6·4 to 13·4)	45·9 (41·1 to 51·7)
Guinea-Bissau*	2037	63·6 (58·6 to 68·6)	5·7 (4·1 to 7·7)	39·9 (36·0 to 44·1)	3·3 (2·2 to 4·6)	14·8 (13·2 to 16·7)	194·0 (178·9 to 209·5)	17·3 (12·6 to 23·4)	121·6 (109·9 to 134·6)	10·1 (6·7 to 14·0)	45·1 (40·3 to 51·0)
Guinea-Bissau*	2038	64·4 (59·3 to 69·6)	5·8 (4·2 to 7·9)	40·6 (36·7 to 44·9)	3·4 (2·3 to 4·8)	14·5 (12·9 to 16·5)	196·4 (181·0 to 212·4)	17·8 (12·9 to 24·1)	123·7 (111·8 to 137·0)	10·5 (7·0 to 14·8)	44·3 (39·4 to 50·2)
Guinea-Bissau*	2039	65·2 (60·0 to 70·5)	6·0 (4·3 to 8·3)	41·2 (37·3 to 45·6)	3·6 (2·4 to 5·1)	14·3 (12·8 to 16·4)	199·0 (183·2 to 215·0)	18·5 (13·1 to 25·2)	125·8 (113·7 to 139·3)	11·0 (7·3 to 15·4)	43·8 (38·9 to 50·1)
Guinea-Bissau*	2040	66·1 (60·7 to 71·5)	6·3 (4·4 to 8·5)	41·9 (37·8 to 46·4)	3·8 (2·5 to 5·3)	14·2 (12·5 to 16·4)	201·8 (185·4 to 218·3)	19·1 (13·5 to 25·8)	127·8 (115·5 to 141·6)	11·5 (7·6 to 16·2)	43·4 (38·3 to 50·0)
Guinea-Bissau*	2041	67·1 (61·7 to 72·7)	6·5 (4·6 to 8·8)	42·5 (38·4 to 47·1)	3·9 (2·6 to 5·6)	14·1 (12·4 to 16·3)	204·6 (188·1 to 221·8)	19·8 (13·9 to 26·8)	129·8 (117·2 to 143·7)	12·0 (7·9 to 17·0)	43·0 (37·7 to 49·8)
Guinea-Bissau*	2042	68·1 (62·4 to 73·8)	6·7 (4·7 to 9·3)	43·2 (39·0 to 47·8)	4·1 (2·7 to 5·8)	14·1 (12·3 to 16·5)	207·7 (190·5 to 225·3)	20·6 (14·4 to 28·3)	131·7 (119·0 to 145·9)	12·5 (8·2 to 17·8)	42·9 (37·5 to 50·2)
Guinea-Bissau*	2043	69·1 (63·2 to 75·1)	7·0 (4·9 to 9·7)	43·8 (39·6 to 48·5)	4·3 (2·8 to 6·2)	14·0 (12·2 to 16·4)	210·9 (192·8 to 229·0)	21·4 (14·9 to 29·6)	133·6 (120·8 to 148·0)	13·1 (8·6 to 18·9)	42·8 (37·3 to 50·0)
Guinea-Bissau*	2044	70·2 (64·2 to 76·3)	7·3 (5·0 to 10·3)	44·4 (40·1 to 49·2)	4·5 (2·9 to 6·5)	14·0 (12·2 to 16·6)	214·3 (195·8 to 232·8)	22·2 (15·3 to 31·4)	135·5 (122·5 to 150·1)	13·7 (8·9 to 19·8)	42·8 (37·1 to 50·5)
Guinea-Bissau*	2045	71·3 (65·0 to 77·7)	7·6 (5·2 to 10·6)	45·0 (40·7 to 49·8)	4·7 (3·0 to 6·8)	14·1 (12·2 to 16·7)	217·7 (198·5 to 237·0)	23·1 (15·8 to 32·3)	137·3 (124·2 to 152·1)	14·3 (9·3 to 20·8)	42·9 (37·1 to 50·9)
Guinea-Bissau*	2046	72·6 (66·2 to 79·0)	7·9 (5·4 to 11·1)	45·6 (41·2 to 50·5)	4·9 (3·2 to 7·1)	14·2 (12·2 to 16·8)	221·4 (202·0 to 241·0)	24·1 (16·4 to 34·0)	139·2 (125·8 to 154·1)	15·0 (9·7 to 21·8)	43·2 (37·1 to 51·3)

Guinea-Bissau*	2047	73·8 (67·3 to 80·4)	8·2 (5·6 to 11·5)	46·2 (41·8 to 51·1)	5·1 (3·3 to 7·5)	14·3 (12·1 to 17·1)	225·2 (205·2 to 245·4)	25·0 (17·0 to 35·0)	140·9 (127·4 to 156·0)	15·7 (10·1 to 22·8)	43·5 (36·8 to 52·2)
Guinea-Bissau*	2048	75·1 (68·2 to 81·9)	8·5 (5·7 to 12·0)	46·8 (42·2 to 51·8)	5·4 (3·4 to 7·9)	14·4 (12·3 to 17·4)	229·1 (208·3 to 250·0)	26·1 (17·5 to 36·7)	142·7 (128·9 to 158·0)	16·4 (10·5 to 24·0)	43·9 (37·5 to 53·0)
Guinea-Bissau*	2049	76·4 (69·5 to 83·5)	8·9 (6·0 to 12·6)	47·3 (42·7 to 52·4)	5·6 (3·6 to 8·3)	14·6 (12·3 to 17·7)	233·2 (212·1 to 254·7)	27·2 (18·3 to 38·4)	144·3 (130·3 to 159·9)	17·2 (10·9 to 25·2)	44·5 (37·5 to 54·1)
Guinea-Bissau*	2050	77·8 (70·6 to 85·0)	9·3 (6·2 to 13·1)	47·9 (43·2 to 53·0)	5·9 (3·7 to 8·8)	14·8 (12·5 to 18·0)	237·5 (215·5 to 259·3)	28·4 (19·0 to 40·0)	146·0 (131·8 to 161·7)	18·0 (11·2 to 26·7)	45·1 (38·0 to 54·8)
Equatorial Guinea*	1995	113·1 (94·3 to 134·7)	7·4 (6·1 to 8·9)	101·5 (82·4 to 122·5)	2·4 (1·6 to 3·4)	1·8 (1·8 to 1·8)	282·1 (235·1 to 335·9)	18·5 (15·2 to 22·3)	253·2 (205·4 to 305·4)	6·0 (4·1 to 8·5)	4·4 (4·4 to 4·4)
Equatorial Guinea*	1996	103·2 (88·1 to 120·0)	4·7 (3·9 to 5·7)	84·6 (69·9 to 101·3)	1·8 (1·2 to 2·6)	12·1 (12·1 to 12·1)	257·2 (219·7 to 299·3)	11·7 (9·6 to 14·2)	210·9 (174·2 to 252·6)	4·5 (3·0 to 6·4)	30·1 (30·1 to 30·1)
Equatorial Guinea*	1997	115·9 (98·9 to 134·4)	7·7 (6·3 to 9·3)	102·9 (86·1 to 121·1)	2·6 (1·8 to 3·7)	2·7 (2·7 to 2·7)	289·0 (246·6 to 335·1)	19·1 (15·7 to 23·3)	256·7 (214·8 to 302·1)	6·5 (4·4 to 9·3)	6·7 (6·7 to 6·7)
Equatorial Guinea*	1998	142·3 (122·2 to 163·2)	13·0 (10·7 to 15·8)	123·0 (103·4 to 143·7)	3·8 (2·6 to 5·5)	2·5 (2·5 to 2·5)	354·9 (304·6 to 406·9)	32·4 (26·8 to 39·5)	306·6 (257·8 to 358·5)	9·5 (6·4 to 13·6)	6·3 (6·3 to 6·3)
Equatorial Guinea*	1999	131·7 (113·8 to 150·7)	11·4 (9·5 to 13·8)	112·3 (95·0 to 131·7)	3·8 (2·6 to 5·3)	4·2 (4·2 to 4·2)	328·5 (283·8 to 375·9)	28·5 (23·6 to 34·5)	280·1 (237·0 to 328·4)	9·4 (6·5 to 13·3)	10·5 (10·5 to 10·5)
Equatorial Guinea*	2000	151·0 (132·1 to 172·8)	15·7 (12·9 to 18·9)	122·9 (104·6 to 144·5)	4·9 (3·4 to 6·8)	7·5 (7·5 to 7·5)	376·6 (329·4 to 430·9)	39·2 (32·2 to 47·1)	306·5 (260·9 to 360·3)	12·2 (8·6 to 16·9)	18·8 (18·8 to 18·8)
Equatorial Guinea*	2001	165·2 (145·7 to 188·2)	15·7 (12·9 to 18·8)	137·4 (118·1 to 159·9)	6·1 (4·4 to 8·3)	6·1 (6·1 to 6·1)	412·0 (363·2 to 469·3)	39·1 (32·2 to 46·8)	342·5 (294·6 to 398·8)	15·2 (10·9 to 20·8)	15·2 (15·2 to 15·2)
Equatorial Guinea*	2002	173·4 (153·7 to 195·2)	15·5 (12·9 to 18·4)	146·9 (127·7 to 168·6)	6·8 (4·9 to 9·2)	4·2 (4·2 to 4·2)	432·5 (383·3 to 486·8)	38·7 (32·1 to 45·9)	366·4 (318·5 to 420·4)	17·1 (12·3 to 23·0)	10·4 (10·4 to 10·4)
Equatorial Guinea*	2003	185·2 (164·0 to 207·5)	18·7 (15·7 to 21·9)	153·4 (133·0 to 175·5)	7·9 (5·8 to 10·4)	5·2 (5·2 to 5·2)	461·8 (408·9 to 517·4)	46·6 (39·2 to 54·7)	382·6 (331·7 to 437·7)	19·7 (14·5 to 26·0)	12·9 (12·9 to 12·9)
Equatorial Guinea*	2004	202·5 (180·6 to 225·5)	24·5 (21·0 to 28·6)	161·7 (139·8 to 183·6)	9·5 (7·2 to 12·1)	6·8 (6·8 to 6·8)	505·0 (450·4 to 562·4)	61·2 (52·3 to 71·2)	403·2 (348·5 to 457·9)	23·7 (17·8 to 30·2)	16·9 (16·9 to 16·9)

Equatorial Guinea*	2005	212·6 (191·2 to 235·6)	29·0 (25·0 to 33·2)	160·3 (139·0 to 182·8)	10·2 (8·0 to 12·6)	13·2 (13·2 to 13·2)	530·2 (476·7 to 587·6)	72·2 (62·5 to 82·8)	399·6 (346·6 to 455·9)	25·4 (20·0 to 31·4)	33·0 (33·0 to 33·0)
Equatorial Guinea*	2006	225·8 (203·2 to 250·5)	36·0 (31·7 to 40·8)	162·8 (141·1 to 186·6)	10·9 (9·1 to 12·8)	16·2 (16·2 to 16·2)	563·1 (506·7 to 624·6)	89·7 (79·0 to 101·8)	405·9 (351·8 to 465·3)	27·1 (22·7 to 32·0)	40·4 (40·4 to 40·4)
Equatorial Guinea*	2007	224·3 (201·6 to 248·8)	43·2 (38·8 to 48·2)	169·6 (146·9 to 193·8)	11·5 (10·2 to 13·0)	0·0 (0·0 to 0·0)	559·3 (502·8 to 620·5)	107·7 (96·8 to 120·2)	422·8 (366·4 to 483·4)	28·7 (25·4 to 32·3)	0·0 (0·0 to 0·0)
Equatorial Guinea*	2008	243·5 (220·0 to 268·1)	51·6 (47·2 to 56·5)	179·9 (158·1 to 203·8)	12·0 (11·0 to 13·2)	0·0 (0·0 to 0·0)	607·1 (548·6 to 668·5)	128·6 (117·8 to 140·8)	448·6 (394·2 to 508·2)	30·0 (27·4 to 33·0)	0·0 (0·0 to 0·0)
Equatorial Guinea*	2009	285·2 (261·5 to 313·3)	80·4 (74·6 to 86·6)	191·2 (168·7 to 218·7)	13·6 (12·4 to 14·8)	0·0 (0·0 to 0·0)	711·3 (652·0 to 781·3)	200·6 (186·0 to 215·9)	476·9 (420·8 to 545·3)	33·8 (30·9 to 36·8)	0·0 (0·0 to 0·0)
Equatorial Guinea*	2010	236·2 (215·8 to 259·7)	63·5 (58·9 to 68·2)	161·5 (142·3 to 184·6)	11·2 (10·2 to 12·3)	0·0 (0·0 to 0·0)	589·1 (538·2 to 647·7)	158·5 (146·8 to 170·1)	402·7 (354·7 to 460·3)	27·9 (25·4 to 30·6)	0·0 (0·0 to 0·0)
Equatorial Guinea*	2011	220·7 (201·5 to 241·6)	60·5 (55·9 to 65·8)	149·3 (131·1 to 170·5)	10·9 (9·8 to 12·0)	0·0 (0·0 to 0·0)	550·5 (502·4 to 602·6)	151·0 (139·5 to 164·1)	372·4 (326·8 to 425·1)	27·1 (24·4 to 29·8)	0·0 (0·0 to 0·0)
Equatorial Guinea*	2012	241·0 (219·9 to 263·5)	72·6 (66·8 to 79·0)	156·5 (136·2 to 178·1)	11·9 (10·8 to 13·1)	0·0 (0·0 to 0·0)	601·0 (548·3 to 657·2)	181·0 (166·7 to 197·1)	390·3 (339·7 to 444·0)	29·7 (26·9 to 32·8)	0·0 (0·0 to 0·0)
Equatorial Guinea*	2013	244·3 (221·8 to 266·5)	60·4 (55·2 to 66·0)	173·3 (151·3 to 194·5)	10·6 (9·5 to 11·8)	0·0 (0·0 to 0·0)	609·3 (553·1 to 664·5)	150·7 (137·8 to 164·7)	432·2 (377·3 to 484·9)	26·4 (23·7 to 29·4)	0·0 (0·0 to 0·0)
Equatorial Guinea*	2014	276·9 (252·9 to 300·5)	62·9 (56·7 to 69·3)	203·9 (180·6 to 227·8)	10·2 (8·9 to 11·5)	0·0 (0·0 to 0·0)	690·6 (630·7 to 749·5)	156·8 (141·4 to 172·9)	508·4 (450·4 to 568·2)	25·4 (22·2 to 28·7)	0·0 (0·0 to 0·0)
Equatorial Guinea*	2015	317·1 (290·9 to 344·5)	69·9 (62·2 to 77·7)	234·9 (209·2 to 261·1)	9·5 (8·0 to 11·1)	2·7 (2·7 to 2·7)	790·8 (725·4 to 859·1)	174·4 (155·0 to 193·7)	585·9 (521·6 to 651·1)	23·7 (19·9 to 27·7)	6·8 (6·8 to 6·8)
Equatorial Guinea*	2016	290·8 (265·8 to 318·8)	56·6 (49·4 to 64·0)	221·5 (197·1 to 248·9)	7·9 (6·3 to 9·7)	4·7 (4·7 to 4·7)	725·2 (662·7 to 795·1)	141·2 (123·3 to 159·5)	552·4 (491·4 to 620·6)	19·8 (15·8 to 24·2)	11·7 (11·7 to 11·7)
Equatorial Guinea*	2017	265·8 (238·3 to 295·0)	47·4 (40·8 to 54·5)	204·7 (178·9 to 233·3)	7·0 (5·3 to 9·0)	6·7 (6·7 to 6·7)	662·9 (594·2 to 735·6)	118·2 (101·8 to 136·0)	510·5 (446·1 to 581·7)	17·4 (13·3 to 22·4)	16·7 (16·7 to 16·7)
Equatorial Guinea*	2018	244·2 (215·6 to 276·6)	44·4 (37·5 to 52·2)	192·0 (163·8 to 223·5)	6·5 (4·7 to 8·6)	1·2 (1·2 to 1·2)	608·9 (537·6 to 689·7)	110·8 (93·5 to 130·3)	478·8 (408·6 to 557·5)	16·2 (11·8 to 21·4)	3·1 (3·1 to 3·1)

Equatorial Guinea*	2019	222·1 (195·9 to 251·5)	38·4 (32·6 to 45·0)	176·0 (150·0 to 204·5)	6·0 (4·4 to 8·0)	1·7 (1·7 to 1·8)	554·0 (488·4 to 627·2)	95·8 (81·2 to 112·1)	438·8 (374·1 to 510·0)	15·1 (11·0 to 20·0)	4·4 (4·2 to 4·6)
Equatorial Guinea*	2020	280·9 (255·9 to 308·7)	105·8 (100·1 to 112·2)	166·9 (142·4 to 194·0)	5·9 (4·3 to 7·8)	2·3 (2·3 to 2·5)	700·5 (638·3 to 769·9)	263·8 (249·6 to 279·7)	416·3 (355·2 to 483·8)	14·7 (10·7 to 19·4)	5·8 (5·6 to 6·2)
Equatorial Guinea*	2021	212·4 (188·1 to 239·3)	43·4 (36·8 to 51·4)	160·9 (137·3 to 186·9)	5·8 (4·3 to 7·7)	2·2 (2·2 to 2·4)	529·6 (469·2 to 596·7)	108·3 (91·8 to 128·3)	401·2 (342·3 to 466·0)	14·5 (10·6 to 19·2)	5·6 (5·4 to 6·0)
Equatorial Guinea*	2022	213·9 (188·8 to 242·3)	38·6 (31·7 to 46·4)	167·6 (142·9 to 194·5)	6·2 (4·5 to 8·2)	1·5 (1·5 to 1·7)	533·5 (470·8 to 604·3)	96·3 (79·1 to 115·6)	417·9 (356·3 to 485·1)	15·4 (11·3 to 20·5)	3·9 (3·6 to 4·2)
Equatorial Guinea*	2023	222·9 (196·7 to 252·6)	40·8 (33·1 to 49·7)	174·0 (148·4 to 202·1)	6·6 (4·8 to 8·7)	1·4 (1·3 to 1·5)	555·7 (490·5 to 630·0)	101·8 (82·5 to 124·1)	434·0 (370·0 to 504·0)	16·4 (12·1 to 21·8)	3·5 (3·3 to 3·8)
Equatorial Guinea*	2024	232·6 (205·1 to 263·7)	43·3 (34·8 to 52·8)	181·0 (154·3 to 210·1)	7·0 (5·2 to 9·3)	1·3 (1·2 to 1·4)	580·1 (511·6 to 657·5)	108·0 (86·7 to 131·8)	451·3 (384·7 to 523·9)	17·5 (12·9 to 23·2)	3·3 (3·1 to 3·6)
Equatorial Guinea*	2025	242·5 (213·8 to 275·3)	45·8 (35·9 to 56·6)	188·0 (160·2 to 218·2)	7·5 (5·5 to 9·9)	1·2 (1·2 to 1·4)	604·7 (533·3 to 686·5)	114·3 (89·6 to 141·2)	468·7 (399·6 to 544·2)	18·6 (13·6 to 24·7)	3·1 (2·9 to 3·4)
Equatorial Guinea*	2026	252·3 (222·2 to 287·3)	48·2 (37·6 to 61·0)	195·0 (166·0 to 226·2)	7·9 (5·8 to 10·5)	1·2 (1·1 to 1·3)	629·1 (554·2 to 716·6)	120·2 (93·8 to 152·0)	486·2 (414·0 to 564·2)	19·8 (14·4 to 26·2)	2·9 (2·7 to 3·3)
Equatorial Guinea*	2027	260·1 (228·8 to 296·7)	50·3 (38·5 to 63·8)	200·3 (170·6 to 232·4)	8·4 (6·1 to 11·1)	1·1 (1·0 to 1·3)	648·5 (570·6 to 739·9)	125·5 (96·1 to 159·2)	499·4 (425·5 to 579·4)	20·8 (15·2 to 27·7)	2·8 (2·6 to 3·2)
Equatorial Guinea*	2028	268·4 (235·9 to 306·4)	52·5 (40·0 to 68·2)	206·0 (175·3 to 238·8)	8·8 (6·4 to 11·7)	1·1 (1·0 to 1·2)	669·2 (588·3 to 764·2)	130·9 (99·7 to 170·2)	513·6 (437·2 to 595·6)	22·0 (16·1 to 29·3)	2·7 (2·4 to 3·1)
Equatorial Guinea*	2029	276·9 (243·2 to 315·6)	54·9 (41·0 to 70·5)	211·7 (180·3 to 245·4)	9·3 (6·8 to 12·4)	1·0 (0·9 to 1·2)	690·6 (606·4 to 787·0)	136·8 (102·3 to 175·8)	528·0 (449·5 to 612·1)	23·2 (17·0 to 30·9)	2·6 (2·3 to 3·0)
Equatorial Guinea*	2030	284·9 (250·0 to 325·5)	56·9 (41·7 to 75·4)	217·3 (185·2 to 251·9)	9·8 (7·1 to 13·0)	1·0 (0·9 to 1·2)	710·5 (623·4 to 811·6)	141·8 (103·9 to 188·1)	541·8 (461·7 to 628·1)	24·4 (17·8 to 32·5)	2·5 (2·2 to 3·0)
Equatorial Guinea*	2031	295·3 (259·1 to 336·8)	61·6 (45·1 to 81·9)	222·4 (189·7 to 257·9)	10·3 (7·5 to 13·7)	1·0 (0·9 to 1·2)	736·3 (646·0 to 839·9)	153·6 (112·5 to 204·2)	554·6 (473·1 to 643·2)	25·6 (18·6 to 34·2)	2·5 (2·1 to 2·9)
Equatorial Guinea*	2032	305·3 (267·6 to 348·0)	66·1 (48·4 to 87·7)	227·5 (193·9 to 263·9)	10·8 (7·9 to 14·4)	1·0 (0·8 to 1·2)	761·3 (667·3 to 867·9)	164·7 (120·8 to 218·6)	567·2 (483·6 to 658·1)	27·0 (19·7 to 36·0)	2·4 (2·0 to 2·9)

Equatorial Guinea*	2033	315·8 (276·5 to 359·1)	70·6 (51·8 to 93·2)	232·9 (198·3 to 270·3)	11·4 (8·3 to 15·2)	0·9 (0·8 to 1·1)	787·5 (689·6 to 895·5)	176·0 (129·1 to 232·5)	580·7 (494·6 to 674·0)	28·5 (20·7 to 38·0)	2·3 (2·0 to 2·8)
Equatorial Guinea*	2034	326·5 (286·1 to 371·2)	75·3 (55·4 to 99·1)	238·3 (202·9 to 276·6)	12·0 (8·8 to 16·1)	0·9 (0·8 to 1·1)	814·2 (713·3 to 925·6)	187·8 (138·1 to 247·1)	594·2 (505·9 to 689·9)	30·0 (21·8 to 40·2)	2·3 (1·9 to 2·8)
Equatorial Guinea*	2035	337·3 (295·8 to 384·1)	80·1 (58·9 to 106·4)	243·6 (207·5 to 282·8)	12·7 (9·2 to 17·1)	0·9 (0·7 to 1·1)	841·1 (737·6 to 957·9)	199·8 (146·8 to 265·3)	607·4 (517·4 to 705·3)	31·7 (22·8 to 42·5)	2·2 (1·8 to 2·8)
Equatorial Guinea*	2036	348·3 (304·8 to 397·2)	85·2 (62·5 to 113·5)	248·9 (212·1 to 288·9)	13·4 (9·6 to 18·2)	0·8 (0·0 to 1·1)	868·7 (760·1 to 990·4)	212·5 (155·8 to 282·9)	620·7 (528·9 to 720·4)	33·4 (24·0 to 45·3)	2·1 (0·0 to 2·8)
Equatorial Guinea*	2037	359·1 (314·3 to 411·0)	90·4 (66·1 to 120·5)	253·8 (216·2 to 294·5)	14·1 (10·1 to 19·2)	0·8 (0·0 to 1·1)	895·5 (783·8 to 1024·8)	225·5 (165·0 to 300·5)	632·8 (539·2 to 734·3)	35·1 (25·1 to 47·8)	2·1 (0·0 to 2·8)
Equatorial Guinea*	2038	370·9 (323·9 to 425·2)	96·2 (70·9 to 129·5)	259·1 (220·9 to 300·5)	14·8 (10·6 to 20·1)	0·8 (0·0 to 1·1)	924·8 (807·7 to 1060·3)	239·9 (176·7 to 322·9)	646·1 (550·7 to 749·5)	36·9 (26·4 to 50·2)	1·9 (0·0 to 2·7)
Equatorial Guinea*	2039	381·9 (333·1 to 439·2)	101·6 (75·0 to 137·1)	264·0 (225·1 to 306·1)	15·6 (11·1 to 21·2)	0·8 (0·0 to 1·1)	952·2 (830·7 to 1095·3)	253·3 (187·1 to 341·9)	658·2 (561·3 to 763·4)	38·8 (27·6 to 52·8)	1·9 (0·0 to 2·7)
Equatorial Guinea*	2040	393·4 (342·5 to 453·3)	107·3 (78·8 to 144·9)	269·0 (229·5 to 311·9)	16·3 (11·5 to 22·3)	0·7 (0·0 to 1·1)	981·0 (854·0 to 1130·3)	267·7 (196·4 to 361·4)	670·8 (572·3 to 777·9)	40·8 (28·8 to 55·6)	1·8 (0·0 to 2·8)
Equatorial Guinea*	2041	405·4 (352·4 to 468·5)	113·5 (82·5 to 153·4)	274·0 (233·7 to 317·8)	17·2 (12·0 to 23·5)	0·7 (0·0 to 1·1)	1010·9 (878·7 to 1168·3)	283·0 (205·6 to 382·6)	683·4 (582·9 to 792·5)	42·8 (30·0 to 58·6)	1·7 (0·0 to 2·8)
Equatorial Guinea*	2042	415·4 (361·0 to 480·4)	118·8 (85·5 to 161·6)	278·0 (237·2 to 322·4)	18·0 (12·5 to 24·6)	0·7 (0·0 to 1·1)	1035·8 (900·2 to 1197·9)	296·1 (213·1 to 403·0)	693·2 (591·4 to 803·9)	44·8 (31·2 to 61·4)	1·6 (0·0 to 2·8)
Equatorial Guinea*	2043	427·2 (370·8 to 494·0)	125·1 (89·1 to 170·9)	282·7 (241·2 to 328·0)	18·9 (13·0 to 25·9)	0·6 (0·0 to 1·2)	1065·4 (924·6 to 1231·9)	311·9 (222·2 to 426·2)	704·9 (601·6 to 817·8)	47·1 (32·4 to 64·6)	1·6 (0·0 to 2·9)
Equatorial Guinea*	2044	438·8 (379·9 to 509·2)	131·1 (92·4 to 180·7)	287·3 (245·3 to 333·6)	19·8 (13·5 to 27·3)	0·6 (0·0 to 1·2)	1094·3 (947·4 to 1269·7)	326·8 (230·4 to 450·6)	716·4 (611·7 to 831·8)	49·5 (33·7 to 68·0)	1·5 (0·0 to 2·9)
Equatorial Guinea*	2045	450·7 (389·4 to 523·9)	137·4 (95·8 to 191·2)	291·9 (249·2 to 339·1)	20·8 (14·0 to 28·9)	0·6 (0·0 to 1·2)	1123·8 (971·0 to 1306·4)	342·5 (239·0 to 476·9)	727·9 (621·4 to 845·6)	51·9 (35·0 to 72·1)	1·5 (0·0 to 3·0)
Equatorial Guinea*	2046	462·9 (398·6 to 540·5)	143·8 (99·3 to 202·6)	296·6 (253·3 to 344·8)	21·9 (14·6 to 30·7)	0·6 (0·0 to 1·2)	1154·3 (993·9 to 1347·8)	358·7 (247·6 to 505·4)	739·6 (631·7 to 859·8)	54·6 (36·3 to 76·5)	1·4 (0·0 to 3·0)

Equatorial Guinea*	2047	474·2 (407·4 to 556·2)	150·3 (102·8 to 214·1)	300·5 (256·7 to 349·6)	22·9 (15·2 to 32·3)	0·6 (0·0 to 1·2)	1182·6 (1016·0 to 1387·1)	374·7 (256·5 to 533·8)	749·4 (640·1 to 871·7)	57·2 (37·8 to 80·6)	1·4 (0·0 to 3·1)
Equatorial Guinea*	2048	485·6 (415·5 to 572·1)	156·4 (106·0 to 225·6)	304·6 (260·0 to 354·6)	24·0 (15·8 to 34·3)	0·5 (0·0 to 1·3)	1210·8 (1036·2 to 1426·7)	390·0 (264·2 to 562·5)	759·5 (648·3 to 884·3)	59·9 (39·3 to 85·5)	1·3 (0·0 to 3·2)
Equatorial Guinea*	2049	496·7 (423·1 to 587·9)	162·5 (108·8 to 235·9)	308·5 (263·1 to 359·6)	25·2 (16·4 to 36·5)	0·5 (0·0 to 1·3)	1238·6 (1055·1 to 1466·0)	405·2 (271·4 to 588·2)	769·3 (656·1 to 896·6)	62·8 (40·8 to 91·0)	1·3 (0·0 to 3·3)
Equatorial Guinea*	2050	507·3 (430·9 to 603·0)	168·3 (111·8 to 246·2)	312·1 (265·9 to 363·9)	26·4 (16·9 to 38·9)	0·5 (0·0 to 1·4)	1265·1 (1074·4 to 1503·7)	419·8 (278·8 to 613·9)	778·3 (663·2 to 907·4)	65·8 (42·2 to 97·0)	1·3 (0·0 to 3·4)
Greece	1995	1275·6 (1203·4 to 1356·8)	658·1 (598·8 to 722·6)	596·1 (553·6 to 642·7)	21·4 (15·3 to 29·4)	0·0 (0·0 to 0·0)	2040·0 (1924·5 to 2169·8)	1052·5 (957·7 to 1155·6)	953·3 (885·3 to 1027·7)	34·2 (24·4 to 47·1)	0·0 (0·0 to 0·0)
Greece	1996	1289·8 (1227·0 to 1351·6)	670·7 (622·7 to 727·5)	597·4 (562·5 to 631·6)	21·8 (15·8 to 29·3)	0·0 (0·0 to 0·0)	2062·7 (1962·3 to 2161·5)	1072·5 (995·8 to 1163·4)	955·3 (899·6 to 1010·1)	34·9 (25·2 to 46·8)	0·0 (0·0 to 0·0)
Greece	1997	1310·0 (1255·9 to 1372·2)	689·6 (642·5 to 739·6)	598·1 (565·6 to 630·4)	22·3 (16·1 to 29·0)	0·0 (0·0 to 0·0)	2095·0 (2008·5 to 2194·5)	1102·9 (1027·4 to 1182·7)	956·5 (904·6 to 1008·1)	35·6 (25·8 to 46·4)	0·0 (0·0 to 0·0)
Greece	1998	1347·1 (1292·8 to 1404·7)	728·8 (682·9 to 776·6)	595·2 (563·9 to 626·4)	23·2 (17·4 to 30·0)	0·0 (0·0 to 0·0)	2154·3 (2067·4 to 2246·4)	1165·4 (1092·1 to 1241·9)	951·8 (901·8 to 1001·8)	37·1 (27·8 to 48·0)	0·0 (0·0 to 0·0)
Greece	1999	1386·5 (1326·6 to 1441·7)	782·9 (733·9 to 830·3)	578·7 (548·1 to 609·8)	24·9 (19·1 to 31·7)	0·0 (0·0 to 0·0)	2217·3 (2121·4 to 2305·5)	1252·1 (1173·7 to 1327·9)	925·4 (876·5 to 975·2)	39·8 (30·6 to 50·6)	0·0 (0·0 to 0·0)
Greece	2000	1442·8 (1381·1 to 1501·7)	859·3 (810·7 to 909·2)	555·0 (524·5 to 587·1)	28·5 (22·3 to 35·6)	0·0 (0·0 to 0·0)	2307·4 (2208·7 to 2401·5)	1374·2 (1296·5 to 1454·0)	887·6 (838·8 to 939·0)	45·6 (35·7 to 56·9)	0·0 (0·0 to 0·0)
Greece	2001	1548·8 (1488·8 to 1610·9)	943·9 (891·7 to 997·7)	572·8 (543·9 to 603·7)	32·1 (25·7 to 39·5)	0·0 (0·0 to 0·0)	2476·9 (2380·9 to 2576·1)	1509·4 (1426·0 to 1595·6)	916·1 (869·8 to 965·4)	51·4 (41·0 to 63·2)	0·0 (0·0 to 0·0)
Greece	2002	1661·4 (1601·9 to 1720·2)	1006·3 (955·3 to 1058·3)	619·0 (590·6 to 649·7)	36·0 (29·2 to 43·6)	0·0 (0·0 to 0·0)	2656·9 (2561·8 to 2751·0)	1609·4 (1527·8 to 1692·5)	989·9 (944·5 to 1039·0)	57·6 (46·6 to 69·7)	0·0 (0·0 to 0·0)
Greece	2003	1783·7 (1723·8 to 1843·3)	1070·8 (1019·6 to 1125·4)	672·9 (641·7 to 704·8)	40·0 (33·0 to 47·5)	0·0 (0·0 to 0·0)	2852·5 (2756·7 to 2947·9)	1712·5 (1630·5 to 1799·8)	1076·1 (1026·2 to 1127·1)	64·0 (52·8 to 75·9)	0·0 (0·0 to 0·0)
Greece	2004	1891·9 (1830·1 to 1951·8)	1135·7 (1082·2 to 1191·4)	712·8 (681·6 to 744·2)	43·4 (36·0 to 51·4)	0·0 (0·0 to 0·0)	3025·5 (2926·7 to 3121·4)	1816·2 (1730·6 to 1905·2)	1139·9 (1090·0 to 1190·1)	69·4 (57·6 to 82·2)	0·0 (0·0 to 0·0)

Greece	2005	1910·7 (1847·6 to 1974·8)	1150·2 (1097·8 to 1203·6)	715·9 (682·8 to 748·5)	44·5 (37·6 to 52·4)	0·0 (0·0 to 0·0)	3055·6 (2954·6 to 3158·1)	1839·4 (1755·6 to 1924·8)	1144·9 (1092·0 to 1197·0)	71·2 (60·1 to 83·8)	0·0 (0·0 to 0·0)
Greece	2006	1998·1 (1936·3 to 2065·7)	1231·6 (1172·9 to 1289·2)	719·4 (687·8 to 754·4)	47·1 (39·3 to 55·1)	0·0 (0·0 to 0·0)	3195·3 (3096·6 to 3303·5)	1969·6 (1875·8 to 2061·7)	1150·5 (1100·0 to 1206·4)	75·3 (62·9 to 88·1)	0·0 (0·0 to 0·0)
Greece	2007	2101·4 (2036·3 to 2164·5)	1337·3 (1276·3 to 1394·7)	713·6 (681·7 to 745·7)	50·5 (42·9 to 58·8)	0·0 (0·0 to 0·0)	3360·6 (3256·5 to 3461·6)	2138·6 (2041·1 to 2230·4)	1141·2 (1090·1 to 1192·6)	80·8 (68·7 to 94·0)	0·0 (0·0 to 0·0)
Greece	2008	2210·4 (2143·7 to 2279·8)	1457·5 (1393·0 to 1518·5)	698·9 (666·3 to 733·9)	54·0 (46·3 to 62·3)	0·0 (0·0 to 0·0)	3534·9 (3428·2 to 3645·9)	2330·8 (2227·7 to 2428·3)	1117·6 (1065·6 to 1173·7)	86·4 (74·1 to 99·6)	0·0 (0·0 to 0·0)
Greece	2009	2238·0 (2171·6 to 2305·7)	1516·2 (1451·1 to 1576·3)	665·2 (634·6 to 696·8)	56·7 (49·1 to 65·3)	0·0 (0·0 to 0·0)	3579·1 (3472·8 to 3687·3)	2424·7 (2320·6 to 2520·8)	1063·8 (1014·8 to 1114·3)	90·6 (78·5 to 104·4)	0·0 (0·0 to 0·0)
Greece	2010	2082·8 (2013·6 to 2148·5)	1406·7 (1351·0 to 1464·0)	613·1 (584·1 to 642·5)	62·9 (54·9 to 71·7)	0·0 (0·0 to 0·0)	3330·8 (3220·1 to 3435·8)	2249·6 (2160·5 to 2341·2)	980·5 (934·0 to 1027·4)	100·7 (87·7 to 114·6)	0·0 (0·0 to 0·0)
Greece	2011	1885·9 (1823·2 to 1947·3)	1246·7 (1194·5 to 1303·5)	571·0 (541·4 to 601·2)	68·2 (60·1 to 76·8)	0·0 (0·0 to 0·0)	3015·9 (2915·7 to 3114·2)	1993·7 (1910·3 to 2084·6)	913·1 (865·8 to 961·5)	109·0 (96·2 to 122·9)	0·0 (0·0 to 0·0)
Greece	2012	1705·9 (1647·1 to 1770·3)	1085·7 (1033·7 to 1140·2)	539·6 (510·3 to 569·1)	80·6 (71·7 to 90·7)	0·0 (0·0 to 0·0)	2728·1 (2634·1 to 2831·1)	1736·3 (1653·2 to 1823·3)	863·0 (816·1 to 910·1)	128·9 (114·7 to 145·1)	0·0 (0·0 to 0·0)
Greece	2013	1619·8 (1562·1 to 1681·4)	1002·3 (949·0 to 1057·8)	543·4 (514·0 to 574·3)	74·1 (66·1 to 82·4)	0·0 (0·0 to 0·0)	2590·4 (2498·2 to 2689·0)	1602·9 (1517·6 to 1691·6)	869·1 (821·9 to 918·5)	118·4 (105·8 to 131·8)	0·0 (0·0 to 0·0)
Greece	2014	1486·8 (1427·6 to 1547·8)	876·4 (828·1 to 927·4)	542·7 (513·9 to 569·3)	67·7 (61·0 to 75·0)	0·0 (0·0 to 0·0)	2377·6 (2283·0 to 2475·3)	1401·5 (1324·3 to 1483·2)	867·9 (821·8 to 910·5)	108·3 (97·6 to 119·9)	0·0 (0·0 to 0·0)
Greece	2015	1492·8 (1435·6 to 1550·8)	871·1 (820·8 to 923·1)	559·3 (530·3 to 586·7)	62·3 (57·4 to 68·1)	0·0 (0·0 to 0·0)	2387·2 (2295·8 to 2480·0)	1393·1 (1312·7 to 1476·3)	894·5 (848·1 to 938·2)	99·7 (91·8 to 108·9)	0·0 (0·0 to 0·0)
Greece	2016	1602·9 (1542·0 to 1662·9)	826·5 (778·6 to 877·2)	561·0 (532·4 to 590·4)	215·4 (201·4 to 229·5)	0·0 (0·0 to 0·0)	2563·3 (2466·1 to 2659·3)	1321·7 (1245·2 to 1402·8)	897·2 (851·4 to 944·2)	344·4 (322·1 to 367·1)	0·0 (0·0 to 0·0)
Greece	2017	1568·4 (1507·4 to 1636·1)	814·0 (760·6 to 870·7)	560·7 (531·3 to 591·3)	193·8 (182·3 to 205·7)	0·0 (0·0 to 0·0)	2508·3 (2410·7 to 2616·4)	1301·7 (1216·4 to 1392·4)	896·6 (849·6 to 945·6)	309·9 (291·5 to 328·9)	0·0 (0·0 to 0·0)
Greece	2018	1568·6 (1490·1 to 1648·1)	819·1 (757·1 to 888·2)	567·4 (528·7 to 605·9)	182·1 (167·7 to 196·2)	0·0 (0·0 to 0·0)	2508·5 (2382·9 to 2635·7)	1309·9 (1210·8 to 1420·5)	907·4 (845·6 to 969·0)	291·2 (268·3 to 313·8)	0·0 (0·0 to 0·0)

Greece	2019	1595·5 (1514·7 to 1677·3)	828·4 (764·9 to 898·8)	577·7 (538·5 to 616·8)	189·3 (174·7 to 204·2)	0·0 (0·0 to 0·0)	2551·5 (2422·2 to 2682·4)	1324·9 (1223·2 to 1437·3)	923·8 (861·2 to 986·4)	302·8 (279·5 to 326·6)	0·0 (0·0 to 0·0)
Greece	2020	1677·7 (1592·7 to 1768·4)	955·0 (879·2 to 1038·9)	541·1 (504·4 to 577·8)	181·6 (167·0 to 196·1)	0·0 (0·0 to 0·0)	2683·0 (2547·0 to 2828·1)	1527·3 (1406·1 to 1661·4)	865·4 (806·7 to 924·0)	290·4 (267·0 to 313·7)	0·0 (0·0 to 0·0)
Greece	2021	1599·3 (1518·3 to 1685·6)	909·2 (837·0 to 989·0)	513·6 (478·7 to 548·1)	176·5 (162·2 to 191·0)	0·0 (0·0 to 0·0)	2557·6 (2428·1 to 2695·7)	1454·0 (1338·6 to 1581·7)	821·4 (765·6 to 876·6)	282·3 (259·4 to 305·5)	0·0 (0·0 to 0·0)
Greece	2022	1434·0 (1361·7 to 1510·0)	739·2 (677·9 to 804·0)	513·9 (478·7 to 548·4)	180·9 (165·9 to 196·4)	0·0 (0·0 to 0·0)	2293·3 (2177·6 to 2414·9)	1182·2 (1084·1 to 1285·7)	821·8 (765·6 to 877·1)	289·3 (265·4 to 314·0)	0·0 (0·0 to 0·0)
Greece	2023	1443·7 (1370·7 to 1520·7)	744·1 (681·6 to 809·5)	514·1 (478·9 to 548·6)	185·5 (170·0 to 202·6)	0·0 (0·0 to 0·0)	2308·8 (2192·1 to 2431·9)	1190·1 (1090·1 to 1294·6)	822·2 (765·8 to 877·3)	296·6 (271·9 to 324·0)	0·0 (0·0 to 0·0)
Greece	2024	1453·6 (1378·8 to 1532·8)	749·2 (684·4 to 817·0)	514·2 (478·9 to 548·5)	190·2 (173·6 to 208·2)	0·0 (0·0 to 0·0)	2324·7 (2204·9 to 2451·2)	1198·2 (1094·5 to 1306·6)	822·3 (765·9 to 877·2)	304·1 (277·6 to 333·0)	0·0 (0·0 to 0·0)
Greece	2025	1463·3 (1388·3 to 1543·0)	754·0 (690·0 to 823·5)	514·3 (479·0 to 548·6)	195·0 (176·8 to 213·9)	0·0 (0·0 to 0·0)	2340·2 (2220·2 to 2467·6)	1205·8 (1103·5 to 1317·0)	822·5 (766·0 to 877·3)	311·9 (282·8 to 342·1)	0·0 (0·0 to 0·0)
Greece	2026	1473·8 (1398·4 to 1555·0)	759·2 (693·1 to 826·8)	514·5 (479·4 to 548·8)	200·1 (180·3 to 220·4)	0·0 (0·0 to 0·0)	2357·0 (2236·3 to 2486·8)	1214·2 (1108·4 to 1322·2)	822·9 (766·7 to 877·6)	320·0 (288·4 to 352·5)	0·0 (0·0 to 0·0)
Greece	2027	1484·3 (1405·2 to 1568·5)	765·1 (698·3 to 837·2)	514·2 (479·2 to 548·4)	205·0 (183·9 to 227·0)	0·0 (0·0 to 0·0)	2373·7 (2247·2 to 2508·4)	1223·5 (1116·7 to 1338·9)	822·3 (766·4 to 877·1)	327·9 (294·0 to 362·9)	0·0 (0·0 to 0·0)
Greece	2028	1494·5 (1413·7 to 1582·5)	770·6 (702·2 to 847·4)	513·8 (478·5 to 547·9)	210·1 (187·1 to 233·9)	0·0 (0·0 to 0·0)	2390·0 (2260·8 to 2530·8)	1232·4 (1122·9 to 1355·2)	821·7 (765·2 to 876·3)	335·9 (299·2 to 374·1)	0·0 (0·0 to 0·0)
Greece	2029	1505·5 (1422·7 to 1596·8)	776·5 (703·3 to 854·3)	513·6 (478·3 to 547·8)	215·3 (190·2 to 241·4)	0·0 (0·0 to 0·0)	2407·6 (2275·1 to 2553·7)	1241·9 (1124·8 to 1366·2)	821·4 (764·9 to 876·0)	344·4 (304·1 to 386·0)	0·0 (0·0 to 0·0)
Greece	2030	1515·6 (1430·2 to 1611·2)	781·4 (708·2 to 864·6)	513·4 (478·2 to 547·5)	220·8 (193·5 to 249·0)	0·0 (0·0 to 0·0)	2423·8 (2287·2 to 2576·7)	1249·6 (1132·6 to 1382·7)	821·1 (764·7 to 875·6)	353·1 (309·4 to 398·3)	0·0 (0·0 to 0·0)
Greece	2031	1524·6 (1436·9 to 1621·9)	785·6 (710·2 to 870·8)	512·8 (477·5 to 547·0)	226·2 (196·7 to 257·2)	0·0 (0·0 to 0·0)	2438·1 (2297·9 to 2593·8)	1256·3 (1135·7 to 1392·6)	820·0 (763·6 to 874·8)	361·8 (314·5 to 411·3)	0·0 (0·0 to 0·0)
Greece	2032	1533·2 (1440·8 to 1632·5)	789·7 (712·6 to 879·1)	511·8 (476·6 to 546·0)	231·7 (200·3 to 266·3)	0·0 (0·0 to 0·0)	2451·9 (2304·1 to 2610·7)	1262·9 (1139·5 to 1405·9)	818·5 (762·1 to 873·1)	370·5 (320·3 to 425·9)	0·0 (0·0 to 0·0)

Greece	2033	1538·1 (1443·9 to 1639·3)	791·6 (713·8 to 882·9)	509·7 (474·6 to 543·8)	236·9 (203·5 to 274·1)	0·0 (0·0 to 0·0)	2459·8 (2309·1 to 2621·6)	1265·9 (1141·5 to 1412·0)	815·1 (758·9 to 869·6)	378·8 (325·4 to 438·3)	0·0 (0·0 to 0·0)
Greece	2034	1542·8 (1445·9 to 1645·8)	793·4 (710·9 to 886·0)	507·2 (472·3 to 541·4)	242·1 (206·4 to 281·9)	0·0 (0·0 to 0·0)	2467·2 (2312·3 to 2632·0)	1268·8 (1136·9 to 1416·8)	811·2 (755·3 to 865·9)	387·2 (330·1 to 450·8)	0·0 (0·0 to 0·0)
Greece	2035	1546·0 (1447·2 to 1649·0)	794·4 (708·5 to 884·9)	504·3 (469·6 to 538·3)	247·3 (209·1 to 290·6)	0·0 (0·0 to 0·0)	2472·4 (2314·4 to 2637·0)	1270·4 (1133·1 to 1415·2)	806·5 (750·9 to 860·8)	395·5 (334·4 to 464·7)	0·0 (0·0 to 0·0)
Greece	2036	1548·9 (1449·4 to 1656·1)	795·0 (708·7 to 885·4)	501·3 (466·8 to 535·2)	252·6 (211·8 to 299·7)	0·0 (0·0 to 0·0)	2477·1 (2317·9 to 2648·5)	1271·4 (1133·4 to 1416·0)	801·7 (746·5 to 855·9)	403·9 (338·7 to 479·2)	0·0 (0·0 to 0·0)
Greece	2037	1553·8 (1449·1 to 1663·3)	797·0 (709·0 to 891·8)	498·8 (464·4 to 532·7)	258·1 (214·7 to 308·1)	0·0 (0·0 to 0·0)	2484·9 (2317·4 to 2659·9)	1274·5 (1133·8 to 1426·2)	797·7 (742·7 to 851·9)	412·7 (343·3 to 492·7)	0·0 (0·0 to 0·0)
Greece	2038	1557·1 (1449·7 to 1670·2)	797·8 (705·7 to 890·1)	495·9 (461·7 to 529·9)	263·5 (217·3 to 317·1)	0·0 (0·0 to 0·0)	2490·2 (2318·3 to 2670·9)	1275·8 (1128·5 to 1423·5)	793·1 (738·4 to 847·3)	421·3 (347·6 to 507·0)	0·0 (0·0 to 0·0)
Greece	2039	1561·7 (1448·4 to 1678·5)	799·0 (703·5 to 894·0)	493·5 (459·4 to 527·5)	269·2 (220·3 to 327·3)	0·0 (0·0 to 0·0)	2497·5 (2316·3 to 2684·3)	1277·8 (1125·1 to 1429·8)	789·2 (734·7 to 843·6)	430·5 (352·3 to 523·3)	0·0 (0·0 to 0·0)
Greece	2040	1565·1 (1451·4 to 1686·5)	799·7 (706·7 to 894·5)	490·6 (456·6 to 524·6)	274·7 (223·0 to 337·7)	0·0 (0·0 to 0·0)	2502·8 (2321·2 to 2697·0)	1278·9 (1130·1 to 1430·5)	784·5 (730·2 to 838·9)	439·4 (356·6 to 540·0)	0·0 (0·0 to 0·0)
Greece	2041	1568·5 (1452·7 to 1693·6)	800·5 (705·0 to 895·2)	487·6 (454·0 to 521·6)	280·3 (225·1 to 347·4)	0·0 (0·0 to 0·0)	2508·3 (2323·2 to 2708·5)	1280·1 (1127·4 to 1431·5)	779·8 (726·0 to 834·2)	448·3 (360·1 to 555·6)	0·0 (0·0 to 0·0)
Greece	2042	1571·3 (1450·7 to 1699·6)	800·8 (706·9 to 897·9)	484·5 (451·2 to 518·5)	285·9 (227·7 to 357·8)	0·0 (0·0 to 0·0)	2512·8 (2320·0 to 2718·1)	1280·6 (1130·6 to 1435·9)	774·9 (721·6 to 829·3)	457·3 (364·1 to 572·2)	0·0 (0·0 to 0·0)
Greece	2043	1573·8 (1450·5 to 1704·2)	800·8 (705·9 to 903·2)	481·4 (448·5 to 515·3)	291·6 (230·2 to 367·3)	0·0 (0·0 to 0·0)	2516·9 (2319·7 to 2725·3)	1280·7 (1129·0 to 1444·4)	769·8 (717·3 to 824·1)	466·4 (368·1 to 587·3)	0·0 (0·0 to 0·0)
Greece	2044	1576·6 (1450·8 to 1712·6)	801·0 (701·0 to 899·5)	478·1 (445·3 to 511·8)	297·5 (232·5 to 377·5)	0·0 (0·0 to 0·0)	2521·3 (2320·1 to 2738·9)	1281·0 (1121·0 to 1438·4)	764·6 (712·1 to 818·5)	475·7 (371·9 to 603·6)	0·0 (0·0 to 0·0)
Greece	2045	1579·3 (1449·8 to 1719·2)	801·4 (703·8 to 904·4)	474·7 (441·8 to 508·3)	303·2 (234·7 to 388·6)	0·0 (0·0 to 0·0)	2525·6 (2318·5 to 2749·3)	1281·6 (1125·6 to 1446·3)	759·1 (706·6 to 812·9)	484·9 (375·4 to 621·5)	0·0 (0·0 to 0·0)
Greece	2046	1583·3 (1450·6 to 1728·6)	802·4 (702·3 to 909·2)	471·7 (439·0 to 505·1)	309·2 (237·0 to 399·5)	0·0 (0·0 to 0·0)	2532·0 (2319·7 to 2764·4)	1283·2 (1123·1 to 1454·1)	754·4 (702·1 to 807·8)	494·4 (379·0 to 638·8)	0·0 (0·0 to 0·0)

Greece	2047	1589·8 (1452·5 to 1740·4)	804·6 (703·8 to 915·6)	469·7 (436·7 to 502·8)	315·5 (239·4 to 410·9)	0·0 (0·0 to 0·0)	2542·4 (2322·8 to 2783·3)	1286·8 (1125·5 to 1464·2)	751·1 (698·4 to 804·1)	504·6 (382·9 to 657·2)	0·0 (0·0 to 0·0)
Greece	2048	1598·3 (1457·4 to 1750·4)	807·7 (702·8 to 917·2)	468·3 (435·6 to 501·3)	322·2 (242·4 to 423·1)	0·0 (0·0 to 0·0)	2556·0 (2330·7 to 2799·2)	1291·8 (1123·9 to 1466·8)	749·0 (696·5 to 801·7)	515·3 (387·6 to 676·6)	0·0 (0·0 to 0·0)
Greece	2049	1608·2 (1464·5 to 1765·6)	811·7 (706·4 to 923·6)	467·4 (434·8 to 500·4)	329·1 (245·3 to 435·7)	0·0 (0·0 to 0·0)	2571·9 (2342·0 to 2823·5)	1298·0 (1129·6 to 1477·1)	747·5 (695·4 to 800·3)	526·4 (392·3 to 696·7)	0·0 (0·0 to 0·0)
Greece	2050	1620·4 (1469·9 to 1784·5)	816·9 (710·4 to 935·0)	467·0 (434·6 to 500·2)	336·4 (248·5 to 448·1)	0·0 (0·0 to 0·0)	2591·3 (2350·6 to 2853·9)	1306·4 (1136·0 to 1495·3)	746·9 (695·0 to 799·9)	538·0 (397·4 to 716·6)	0·0 (0·0 to 0·0)
Grenada*	1995	457·0 (426·3 to 488·8)	224·2 (205·2 to 246·0)	211·1 (188·7 to 233·9)	21·6 (14·8 to 30·3)	0·0 (0·0 to 0·0)	765·2 (713·8 to 818·5)	375·5 (343·6 to 411·8)	353·5 (316·0 to 391·7)	36·1 (24·8 to 50·8)	0·0 (0·0 to 0·0)
Grenada*	1996	472·7 (447·2 to 499·9)	227·4 (211·5 to 245·4)	224·5 (204·8 to 244·1)	20·4 (14·1 to 28·5)	0·3 (0·3 to 0·3)	791·6 (748·9 to 837·1)	380·9 (354·1 to 410·9)	376·0 (342·9 to 408·8)	34·2 (23·5 to 47·7)	0·5 (0·5 to 0·5)
Grenada*	1997	472·3 (446·4 to 497·3)	212·3 (197·2 to 227·7)	236·6 (218·4 to 255·2)	18·5 (12·6 to 26·2)	4·9 (4·9 to 4·9)	790·8 (747·6 to 832·7)	355·6 (330·2 to 381·3)	396·2 (365·7 to 427·3)	30·9 (21·2 to 43·8)	8·1 (8·1 to 8·1)
Grenada*	1998	467·8 (443·7 to 493·8)	196·9 (182·1 to 212·4)	249·9 (232·0 to 269·1)	17·2 (11·9 to 24·6)	3·8 (3·8 to 3·8)	783·3 (743·0 to 826·9)	329·7 (304·9 to 355·6)	418·5 (388·5 to 450·6)	28·8 (19·9 to 41·2)	6·3 (6·3 to 6·3)
Grenada*	1999	442·3 (420·0 to 468·2)	176·3 (161·7 to 190·6)	249·5 (231·7 to 267·1)	16·1 (11·1 to 23·1)	0·4 (0·4 to 0·4)	740·6 (703·4 to 784·0)	295·2 (270·7 to 319·1)	417·7 (387·9 to 447·2)	26·9 (18·6 to 38·7)	0·7 (0·7 to 0·7)
Grenada*	2000	412·1 (391·2 to 436·2)	160·9 (146·9 to 174·6)	235·7 (218·4 to 253·6)	15·3 (10·7 to 21·8)	0·2 (0·2 to 0·2)	690·0 (655·0 to 730·4)	269·4 (245·9 to 292·3)	394·7 (365·7 to 424·6)	25·6 (17·9 to 36·6)	0·3 (0·3 to 0·3)
Grenada*	2001	406·4 (382·9 to 430·2)	165·2 (151·2 to 178·7)	225·9 (209·6 to 243·4)	14·9 (10·5 to 20·8)	0·3 (0·3 to 0·3)	680·5 (641·1 to 720·3)	276·7 (253·3 to 299·3)	378·3 (351·1 to 407·6)	24·9 (17·6 to 34·8)	0·5 (0·5 to 0·5)
Grenada*	2002	434·6 (410·7 to 459·7)	191·5 (176·7 to 207·3)	227·2 (209·4 to 245·5)	15·5 (10·9 to 21·6)	0·3 (0·3 to 0·3)	727·7 (687·8 to 769·7)	320·7 (295·9 to 347·0)	380·5 (350·6 to 411·1)	25·9 (18·3 to 36·2)	0·5 (0·5 to 0·5)
Grenada*	2003	412·5 (391·4 to 436·5)	180·1 (166·4 to 194·8)	213·5 (197·2 to 230·2)	15·0 (10·6 to 20·7)	3·9 (3·9 to 3·9)	690·7 (655·5 to 730·9)	301·6 (278·6 to 326·1)	357·5 (330·3 to 385·5)	25·1 (17·8 to 34·6)	6·5 (6·5 to 6·5)
Grenada*	2004	392·4 (371·4 to 414·3)	170·2 (157·6 to 183·8)	202·4 (187·2 to 219·7)	14·9 (10·5 to 20·2)	5·0 (5·0 to 5·0)	657·1 (622·0 to 693·7)	284·9 (263·9 to 307·7)	338·9 (313·4 to 367·9)	25·0 (17·6 to 33·8)	8·3 (8·3 to 8·3)

Grenada*	2005	426·9 (404·0 to 450·0)	188·4 (174·6 to 202·2)	218·3 (202·4 to 235·8)	17·4 (12·4 to 23·4)	2·8 (2·8 to 2·8)	714·8 (676·5 to 753·5)	315·5 (292·3 to 338·6)	365·6 (338·9 to 394·8)	29·1 (20·8 to 39·1)	4·7 (4·7 to 4·7)
Grenada*	2006	453·2 (428·6 to 476·2)	194·6 (180·3 to 209·4)	234·1 (217·9 to 252·9)	18·4 (13·3 to 24·8)	6·1 (6·1 to 6·1)	758·9 (717·7 to 797·4)	325·9 (301·9 to 350·6)	392·1 (364·8 to 423·5)	30·7 (22·3 to 41·5)	10·1 (10·1 to 10·1)
Grenada*	2007	452·1 (430·3 to 476·0)	181·7 (167·8 to 196·2)	243·9 (228·0 to 262·1)	18·4 (13·5 to 25·3)	8·1 (8·1 to 8·1)	757·0 (720·5 to 797·0)	304·2 (281·0 to 328·6)	408·4 (381·8 to 439·0)	30·7 (22·5 to 42·4)	13·6 (13·6 to 13·6)
Grenada*	2008	467·8 (444·7 to 492·9)	184·8 (170·5 to 199·8)	256·2 (239·6 to 275·6)	19·9 (14·6 to 26·7)	6·9 (6·9 to 6·9)	783·3 (744·6 to 825·4)	309·5 (285·6 to 334·6)	429·0 (401·1 to 461·6)	33·2 (24·5 to 44·7)	11·6 (11·6 to 11·6)
Grenada*	2009	461·2 (438·1 to 484·9)	180·3 (166·6 to 195·1)	255·4 (238·5 to 276·4)	20·1 (14·8 to 27·0)	5·4 (5·4 to 5·4)	772·3 (733·5 to 811·9)	301·9 (279·0 to 326·7)	427·7 (399·4 to 462·9)	33·6 (24·8 to 45·2)	9·1 (9·1 to 9·1)
Grenada*	2010	469·4 (445·0 to 492·3)	185·4 (173·2 to 199·0)	260·2 (241·6 to 279·5)	20·3 (14·8 to 27·4)	3·6 (3·6 to 3·6)	786·0 (745·2 to 824·3)	310·4 (290·0 to 333·2)	435·7 (404·6 to 468·1)	33·9 (24·8 to 45·8)	5·9 (5·9 to 5·9)
Grenada*	2011	490·8 (466·7 to 514·5)	197·6 (184·9 to 211·2)	267·5 (249·7 to 286·6)	20·6 (15·1 to 27·4)	5·1 (5·1 to 5·1)	821·8 (781·5 to 861·6)	330·9 (309·7 to 353·7)	447·9 (418·1 to 479·8)	34·5 (25·3 to 45·9)	8·5 (8·5 to 8·5)
Grenada*	2012	478·4 (455·7 to 501·9)	190·6 (179·7 to 201·6)	262·3 (245·2 to 280·4)	19·8 (14·5 to 26·1)	5·8 (5·8 to 5·8)	801·1 (763·0 to 840·4)	319·2 (300·9 to 337·6)	439·2 (410·5 to 469·5)	33·1 (24·3 to 43·7)	9·7 (9·7 to 9·7)
Grenada*	2013	483·6 (461·9 to 507·0)	196·3 (185·1 to 206·8)	260·8 (243·9 to 279·4)	19·7 (14·5 to 26·2)	6·9 (6·9 to 6·9)	809·9 (773·4 to 849·0)	328·7 (309·9 to 346·3)	436·7 (408·4 to 467·9)	32·9 (24·2 to 43·9)	11·6 (11·6 to 11·6)
Grenada*	2014	483·1 (460·7 to 505·4)	203·6 (193·3 to 214·9)	257·1 (239·0 to 276·4)	19·8 (14·6 to 26·7)	2·6 (2·6 to 2·6)	808·9 (771·5 to 846·2)	340·8 (323·7 to 359·8)	430·5 (400·3 to 462·8)	33·2 (24·4 to 44·7)	4·3 (4·3 to 4·3)
Grenada*	2015	475·0 (454·2 to 495·9)	193·8 (184·0 to 204·3)	255·2 (237·7 to 273·5)	19·3 (14·0 to 26·1)	6·7 (6·7 to 6·7)	795·3 (760·5 to 830·3)	324·4 (308·0 to 342·0)	427·3 (398·0 to 458·0)	32·3 (23·5 to 43·6)	11·3 (11·3 to 11·3)
Grenada*	2016	472·3 (451·9 to 494·3)	191·4 (181·4 to 201·6)	260·3 (243·8 to 278·4)	19·5 (14·1 to 26·5)	1·1 (1·1 to 1·1)	790·9 (756·6 to 827·8)	320·5 (303·8 to 337·5)	435·9 (408·2 to 466·1)	32·7 (23·6 to 44·4)	1·8 (1·8 to 1·8)
Grenada*	2017	506·8 (483·1 to 531·4)	197·1 (185·3 to 209·6)	271·5 (253·4 to 292·6)	21·1 (15·3 to 29·5)	17·0 (17·0 to 17·0)	848·6 (808·9 to 889·9)	330·1 (310·2 to 351·0)	454·6 (424·2 to 490·0)	35·4 (25·6 to 49·4)	28·5 (28·5 to 28·5)
Grenada*	2018	510·6 (481·6 to 541·9)	204·0 (187·6 to 222·3)	283·3 (260·1 to 309·0)	22·2 (15·9 to 30·9)	1·1 (1·1 to 1·1)	855·0 (806·4 to 907·4)	341·7 (314·2 to 372·2)	474·3 (435·5 to 517·4)	37·2 (26·6 to 51·7)	1·8 (1·8 to 1·8)

Grenada*	2019	515·1 (482·5 to 550·9)	202·4 (179·1 to 228·0)	288·4 (264·8 to 314·8)	23·2 (16·6 to 32·3)	1·1 (1·1 to 1·1)	862·5 (807·9 to 922·5)	338·9 (299·8 to 381·8)	483·0 (443·4 to 527·1)	38·8 (27·8 to 54·1)	1·9 (1·9 to 1·9)
Grenada*	2020	517·7 (481·1 to 558·7)	228·4 (199·4 to 260·6)	265·7 (244·0 to 290·0)	21·8 (15·7 to 30·4)	1·7 (1·7 to 1·7)	866·8 (805·6 to 935·5)	382·5 (333·9 to 436·3)	444·9 (408·6 to 485·5)	36·6 (26·2 to 50·9)	2·8 (2·8 to 2·9)
Grenada*	2021	491·6 (456·9 to 530·7)	217·0 (189·4 to 247·5)	251·7 (231·1 to 274·5)	21·1 (15·2 to 29·4)	1·8 (1·7 to 1·9)	823·2 (765·0 to 888·6)	363·4 (317·2 to 414·5)	421·4 (387·0 to 459·6)	35·4 (25·4 to 49·2)	3·0 (2·9 to 3·2)
Grenada*	2022	453·7 (422·8 to 488·4)	176·1 (152·9 to 201·2)	254·4 (233·6 to 277·5)	21·8 (15·7 to 30·3)	1·4 (1·3 to 1·5)	759·8 (708·0 to 817·8)	294·8 (256·1 to 336·9)	426·1 (391·2 to 464·6)	36·6 (26·2 to 50·8)	2·3 (2·2 to 2·5)
Grenada*	2023	460·1 (427·1 to 496·9)	178·9 (153·4 to 206·9)	257·2 (236·1 to 280·3)	22·6 (16·2 to 31·2)	1·4 (1·3 to 1·5)	770·4 (715·1 to 832·1)	299·5 (256·8 to 346·5)	430·6 (395·4 to 469·4)	37·8 (27·2 to 52·3)	2·4 (2·2 to 2·6)
Grenada*	2024	466·1 (429·8 to 505·4)	181·3 (153·2 to 211·8)	259·9 (238·6 to 283·4)	23·4 (16·8 to 32·5)	1·5 (1·4 to 1·6)	780·4 (719·6 to 846·3)	303·7 (256·5 to 354·6)	435·1 (399·5 to 474·5)	39·2 (28·2 to 54·4)	2·5 (2·3 to 2·7)
Grenada*	2025	472·6 (434·1 to 514·1)	184·3 (152·8 to 218·5)	262·5 (240·9 to 286·5)	24·2 (17·4 to 33·8)	1·5 (1·4 to 1·7)	791·3 (726·9 to 860·8)	308·7 (255·8 to 365·8)	439·5 (403·4 to 479·8)	40·6 (29·1 to 56·5)	2·5 (2·3 to 2·8)
Grenada*	2026	479·9 (438·5 to 524·2)	188·0 (153·9 to 223·8)	265·2 (243·4 to 289·7)	25·1 (18·0 to 34·9)	1·6 (1·4 to 1·7)	803·5 (734·3 to 877·8)	314·9 (257·6 to 374·7)	444·0 (407·5 to 485·1)	42·1 (30·1 to 58·4)	2·6 (2·4 to 2·9)
Grenada*	2027	486·3 (442·8 to 535·9)	191·7 (155·9 to 233·6)	267·0 (245·0 to 291·8)	26·0 (18·6 to 36·0)	1·6 (1·5 to 1·8)	814·3 (741·4 to 897·3)	321·0 (261·1 to 391·2)	447·1 (410·2 to 488·6)	43·5 (31·1 to 60·3)	2·7 (2·5 to 3·0)
Grenada*	2028	492·4 (447·2 to 543·9)	195·0 (156·7 to 240·9)	268·9 (246·8 to 293·9)	26·9 (19·2 to 37·1)	1·7 (1·5 to 1·9)	824·5 (748·8 to 910·8)	326·5 (262·3 to 403·4)	450·3 (413·2 to 492·1)	45·0 (32·2 to 62·2)	2·8 (2·5 to 3·1)
Grenada*	2029	497·3 (450·2 to 550·3)	197·2 (155·3 to 244·3)	270·7 (248·3 to 295·8)	27·7 (19·8 to 38·3)	1·7 (1·6 to 2·0)	832·8 (753·8 to 921·4)	330·3 (260·1 to 409·1)	453·2 (415·9 to 495·4)	46·4 (33·2 to 64·1)	2·9 (2·6 to 3·3)
Grenada*	2030	501·5 (452·3 to 558·3)	199·4 (156·1 to 251·0)	271·8 (249·4 to 297·1)	28·5 (20·4 to 39·4)	1·8 (1·6 to 2·1)	839·8 (757·3 to 934·9)	333·9 (261·4 to 420·3)	455·1 (417·6 to 497·5)	47·7 (34·2 to 65·9)	3·0 (2·7 to 3·4)
Grenada*	2031	507·2 (456·1 to 566·4)	202·7 (156·6 to 256·8)	273·3 (250·8 to 298·8)	29·3 (21·0 to 40·4)	1·9 (1·7 to 2·2)	849·3 (763·7 to 948·4)	339·4 (262·2 to 430·0)	457·7 (419·9 to 500·3)	49·1 (35·1 to 67·6)	3·1 (2·8 to 3·6)
Grenada*	2032	513·3 (460·3 to 574·0)	206·4 (158·4 to 262·1)	274·8 (252·2 to 300·5)	30·2 (21·6 to 41·4)	1·9 (1·7 to 2·2)	859·6 (770·7 to 961·1)	345·6 (265·3 to 438·9)	460·2 (422·3 to 503·1)	50·5 (36·1 to 69·3)	3·2 (2·9 to 3·7)

Grenada*	2033	519·8 (464·2 to 584·8)	210·1 (158·9 to 270·2)	276·6 (254·0 to 302·3)	31·1 (22·2 to 42·8)	2·0 (1·8 to 2·3)	870·3 (777·2 to 979·3)	351·9 (266·1 to 452·4)	463·1 (425·3 to 506·1)	52·1 (37·2 to 71·6)	3·3 (2·9 to 3·9)
Grenada*	2034	525·9 (468·0 to 594·2)	214·0 (161·3 to 276·8)	277·9 (255·3 to 303·6)	32·0 (22·8 to 44·1)	2·1 (1·8 to 2·4)	880·7 (783·6 to 994·9)	358·3 (270·0 to 463·5)	465·4 (427·5 to 508·4)	53·6 (38·1 to 73·9)	3·4 (3·0 to 4·0)
Grenada*	2035	532·2 (472·6 to 601·5)	217·8 (162·1 to 284·8)	279·4 (256·8 to 305·1)	32·9 (23·4 to 45·6)	2·1 (1·9 to 2·5)	891·2 (791·4 to 1007·2)	364·6 (271·4 to 476·9)	467·8 (429·9 to 510·9)	55·2 (39·3 to 76·4)	3·6 (3·1 to 4·2)
Grenada*	2036	538·2 (476·9 to 610·7)	221·2 (163·5 to 290·9)	280·9 (258·2 to 306·9)	33·9 (24·1 to 47·1)	2·2 (1·9 to 2·6)	901·2 (798·5 to 1022·5)	370·4 (273·8 to 487·1)	470·4 (432·3 to 514·0)	56·8 (40·4 to 78·9)	3·7 (3·2 to 4·4)
Grenada*	2037	544·2 (479·2 to 619·9)	224·5 (166·9 to 299·7)	282·5 (259·6 to 308·6)	34·9 (24·7 to 48·4)	2·3 (2·0 to 2·7)	911·2 (802·4 to 1038·1)	375·9 (279·5 to 501·8)	473·0 (434·8 to 516·8)	58·5 (41·3 to 81·0)	3·8 (3·3 to 4·6)
Grenada*	2038	550·3 (484·4 to 630·6)	227·7 (166·0 to 309·0)	284·3 (261·3 to 310·5)	36·0 (25·3 to 49·8)	2·4 (2·0 to 2·8)	921·5 (811·1 to 1056·0)	381·3 (277·9 to 517·4)	476·0 (437·5 to 519·9)	60·2 (42·4 to 83·4)	4·0 (3·4 to 4·8)
Grenada*	2039	557·3 (489·7 to 639·1)	231·3 (167·7 to 309·7)	286·4 (263·1 to 312·8)	37·1 (26·1 to 51·0)	2·4 (2·1 to 2·9)	933·1 (820·0 to 1070·1)	387·3 (280·9 to 518·6)	479·6 (440·5 to 523·7)	62·1 (43·7 to 85·4)	4·1 (3·5 to 4·9)
Grenada*	2040	564·4 (494·2 to 650·1)	234·8 (172·0 to 318·0)	288·8 (265·2 to 315·5)	38·3 (26·9 to 52·8)	2·5 (2·1 to 3·1)	945·1 (827·5 to 1088·6)	393·1 (288·0 to 532·5)	483·6 (444·1 to 528·3)	64·2 (45·1 to 88·3)	4·2 (3·6 to 5·1)
Grenada*	2041	571·7 (498·5 to 659·0)	238·2 (171·5 to 330·3)	291·4 (267·6 to 318·4)	39·6 (27·8 to 54·7)	2·6 (2·2 to 3·2)	957·3 (834·8 to 1103·5)	398·9 (287·2 to 553·1)	487·9 (448·0 to 533·1)	66·3 (46·5 to 91·7)	4·3 (3·6 to 5·3)
Grenada*	2042	580·0 (508·1 to 671·0)	242·3 (175·2 to 333·1)	294·2 (270·2 to 321·4)	40·9 (28·7 to 56·6)	2·6 (2·2 to 3·3)	971·2 (850·7 to 1123·6)	405·7 (293·3 to 557·7)	492·6 (452·4 to 538·2)	68·5 (48·1 to 94·8)	4·4 (3·7 to 5·4)
Grenada*	2043	588·6 (513·5 to 682·2)	246·5 (175·7 to 338·3)	297·2 (273·0 to 324·6)	42·3 (29·7 to 58·6)	2·6 (0·0 to 3·4)	985·6 (859·9 to 1142·3)	412·7 (294·3 to 566·4)	497·6 (457·1 to 543·6)	70·9 (49·8 to 98·1)	4·4 (0·0 to 5·6)
Grenada*	2044	597·2 (518·5 to 695·3)	250·2 (176·4 to 345·4)	300·5 (276·0 to 328·2)	43·8 (30·6 to 60·8)	2·7 (0·0 to 3·5)	1000·1 (868·3 to 1164·3)	418·9 (295·4 to 578·4)	503·3 (462·1 to 549·6)	73·4 (51·3 to 101·8)	4·5 (0·0 to 5·8)
Grenada*	2045	606·4 (525·4 to 706·0)	254·3 (179·1 to 349·9)	304·0 (278·9 to 332·0)	45·4 (31·6 to 63·0)	2·7 (0·0 to 3·6)	1015·3 (879·8 to 1182·1)	425·9 (299·9 to 585·9)	509·0 (467·0 to 555·9)	76·0 (53·0 to 105·5)	4·5 (0·0 to 6·0)
Grenada*	2046	614·8 (533·2 to 717·5)	257·9 (181·2 to 354·0)	307·2 (281·7 to 335·6)	47·0 (32·6 to 65·4)	2·7 (0·0 to 3·6)	1029·5 (892·8 to 1201·4)	431·9 (303·4 to 592·8)	514·4 (471·7 to 561·9)	78·7 (54·6 to 109·5)	4·5 (0·0 to 6·1)

Grenada*	2047	623·3 (539·4 to 729·3)	261·7 (183·9 to 363·7)	310·3 (284·4 to 339·1)	48·6 (33·6 to 67·9)	2·7 (0·0 to 3·8)	1043·7 (903·2 to 1221·3)	438·3 (308·0 to 609·0)	519·5 (476·1 to 567·8)	81·3 (56·3 to 113·6)	4·5 (0·0 to 6·3)
Grenada*	2048	632·6 (547·6 to 740·5)	266·4 (185·6 to 373·0)	313·3 (287·2 to 342·6)	50·2 (34·7 to 70·4)	2·7 (0·0 to 3·9)	1059·3 (917·0 to 1240·0)	446·1 (310·8 to 624·5)	524·6 (481·0 to 573·7)	84·1 (58·1 to 117·9)	4·5 (0·0 to 6·5)
Grenada*	2049	640·6 (553·0 to 754·8)	270·0 (184·6 to 378·5)	315·9 (289·7 to 345·5)	51·9 (35·7 to 73·3)	2·8 (0·0 to 4·1)	1072·6 (926·0 to 1263·9)	452·1 (309·1 to 633·7)	529·0 (485·1 to 578·6)	86·8 (59·8 to 122·8)	4·6 (0·0 to 6·8)
Grenada*	2050	649·7 (559·6 to 766·0)	274·7 (188·2 to 383·9)	318·7 (292·3 to 348·6)	53·6 (36·8 to 76·1)	2·7 (0·0 to 4·3)	1088·0 (937·1 to 1282·6)	459·9 (315·1 to 642·9)	533·7 (489·4 to 583·7)	89·7 (61·6 to 127·4)	4·6 (0·0 to 7·1)
Greenland	1995	3521·8 (2897·0 to 4240·7)	3521·4 (2896·4 to 4240·4)	0·2 (0·0 to 0·3)	0·2 (0·0 to 0·3)	0·0 (0·0 to 0·0)	2880·3 (2369·3 to 3468·2)	2880·0 (2368·8 to 3468·0)	0·1 (0·0 to 0·3)	0·1 (0·0 to 0·3)	0·0 (0·0 to 0·0)
Greenland	1996	3577·7 (2965·4 to 4272·6)	3577·4 (2965·3 to 4272·3)	0·2 (0·0 to 0·3)	0·2 (0·0 to 0·3)	0·0 (0·0 to 0·0)	2926·1 (2425·3 to 3494·4)	2925·8 (2425·2 to 3494·1)	0·1 (0·0 to 0·3)	0·1 (0·0 to 0·3)	0·0 (0·0 to 0·0)
Greenland	1997	3697·0 (3073·3 to 4408·0)	3696·6 (3073·3 to 4407·4)	0·2 (0·0 to 0·3)	0·2 (0·0 to 0·3)	0·0 (0·0 to 0·0)	3023·6 (2513·5 to 3605·1)	3023·3 (2513·5 to 3604·6)	0·2 (0·0 to 0·3)	0·1 (0·0 to 0·3)	0·0 (0·0 to 0·0)
Greenland	1998	4001·1 (3361·9 to 4688·2)	4000·8 (3361·9 to 4687·9)	0·2 (0·0 to 0·3)	0·2 (0·0 to 0·3)	0·0 (0·0 to 0·0)	3272·3 (2749·6 to 3834·3)	3272·0 (2749·6 to 3834·0)	0·2 (0·0 to 0·3)	0·1 (0·0 to 0·3)	0·0 (0·0 to 0·0)
Greenland	1999	4124·4 (3520·1 to 4775·5)	4124·1 (3519·4 to 4775·5)	0·2 (0·0 to 0·4)	0·2 (0·0 to 0·4)	0·0 (0·0 to 0·0)	3373·2 (2878·9 to 3905·7)	3372·9 (2878·3 to 3905·7)	0·2 (0·0 to 0·3)	0·1 (0·0 to 0·3)	0·0 (0·0 to 0·0)
Greenland	2000	4395·3 (3813·6 to 5012·8)	4395·0 (3813·2 to 5012·4)	0·2 (0·0 to 0·4)	0·2 (0·0 to 0·4)	0·0 (0·0 to 0·0)	3594·7 (3119·0 to 4099·7)	3594·4 (3118·7 to 4099·4)	0·1 (0·0 to 0·3)	0·1 (0·0 to 0·3)	0·0 (0·0 to 0·0)
Greenland	2001	4601·2 (4135·9 to 5107·0)	4600·8 (4135·1 to 5107·0)	0·2 (0·0 to 0·4)	0·2 (0·0 to 0·4)	0·0 (0·0 to 0·0)	3763·1 (3382·5 to 4176·8)	3762·8 (3381·9 to 4176·8)	0·1 (0·0 to 0·3)	0·1 (0·0 to 0·3)	0·0 (0·0 to 0·0)
Greenland	2002	4638·6 (4326·9 to 4976·7)	4638·2 (4326·9 to 4976·4)	0·2 (0·0 to 0·4)	0·2 (0·0 to 0·4)	0·0 (0·0 to 0·0)	3793·7 (3538·8 to 4070·2)	3793·4 (3538·8 to 4069·9)	0·2 (0·0 to 0·3)	0·2 (0·0 to 0·3)	0·0 (0·0 to 0·0)
Greenland	2003	4748·9 (4561·1 to 4942·5)	4748·5 (4560·7 to 4942·1)	0·2 (0·0 to 0·4)	0·2 (0·0 to 0·4)	0·0 (0·0 to 0·0)	3883·9 (3730·3 to 4042·2)	3883·5 (3730·0 to 4041·9)	0·2 (0·0 to 0·3)	0·2 (0·0 to 0·3)	0·0 (0·0 to 0·0)
Greenland	2004	4910·5 (4755·3 to 5061·3)	4910·1 (4754·9 to 5060·9)	0·2 (0·0 to 0·4)	0·2 (0·0 to 0·4)	0·0 (0·0 to 0·0)	4016·1 (3889·1 to 4139·4)	4015·7 (3888·8 to 4139·1)	0·2 (0·0 to 0·3)	0·2 (0·0 to 0·3)	0·0 (0·0 to 0·0)

Greenland	2005	5054·8 (4910·8 to 5199·1)	5054·3 (4910·4 to 5198·5)	0·2 (0·0 to 0·4)	0·2 (0·0 to 0·4)	0·0 (0·0 to 0·0)	4134·1 (4016·3 to 4252·1)	4133·7 (4016·0 to 4251·6)	0·2 (0·0 to 0·3)	0·2 (0·0 to 0·3)	0·0 (0·0 to 0·0)
Greenland	2006	5313·6 (5169·7 to 5468·5)	5313·1 (5169·6 to 5467·9)	0·2 (0·0 to 0·4)	0·2 (0·0 to 0·4)	0·0 (0·0 to 0·0)	4345·7 (4228·0 to 4472·4)	4345·3 (4228·0 to 4471·9)	0·2 (0·0 to 0·4)	0·2 (0·0 to 0·4)	0·0 (0·0 to 0·0)
Greenland	2007	5609·7 (5462·4 to 5765·1)	5609·2 (5462·4 to 5764·6)	0·2 (0·0 to 0·5)	0·2 (0·0 to 0·5)	0·0 (0·0 to 0·0)	4587·9 (4467·4 to 4715·0)	4587·5 (4467·4 to 4714·6)	0·2 (0·0 to 0·4)	0·2 (0·0 to 0·4)	0·0 (0·0 to 0·0)
Greenland	2008	6187·2 (6034·0 to 6332·2)	6186·8 (6033·5 to 6331·8)	0·2 (0·0 to 0·5)	0·2 (0·0 to 0·5)	0·0 (0·0 to 0·0)	5060·2 (4934·9 to 5178·8)	5059·9 (4934·5 to 5178·5)	0·2 (0·0 to 0·4)	0·2 (0·0 to 0·4)	0·0 (0·0 to 0·0)
Greenland	2009	6383·0 (6240·3 to 6533·5)	6382·6 (6239·5 to 6533·1)	0·2 (0·0 to 0·5)	0·2 (0·0 to 0·5)	0·0 (0·0 to 0·0)	5220·3 (5103·7 to 5343·4)	5220·0 (5103·0 to 5343·1)	0·2 (0·0 to 0·4)	0·2 (0·0 to 0·4)	0·0 (0·0 to 0·0)
Greenland	2010	6422·1 (6271·2 to 6588·3)	6421·6 (6270·3 to 6587·3)	0·2 (0·0 to 0·5)	0·2 (0·0 to 0·5)	0·0 (0·0 to 0·0)	5252·3 (5129·0 to 5388·3)	5251·9 (5128·1 to 5387·5)	0·2 (0·0 to 0·4)	0·2 (0·0 to 0·4)	0·0 (0·0 to 0·0)
Greenland	2011	6404·8 (6246·0 to 6564·7)	6404·4 (6245·5 to 6564·1)	0·2 (0·0 to 0·5)	0·2 (0·0 to 0·5)	0·0 (0·0 to 0·0)	5238·2 (5108·3 to 5369·0)	5237·8 (5107·9 to 5368·5)	0·2 (0·0 to 0·4)	0·2 (0·0 to 0·4)	0·0 (0·0 to 0·0)
Greenland	2012	6476·0 (6316·9 to 6627·1)	6475·5 (6316·4 to 6626·6)	0·2 (0·0 to 0·5)	0·2 (0·0 to 0·5)	0·0 (0·0 to 0·0)	5296·4 (5166·3 to 5420·0)	5296·0 (5165·9 to 5419·6)	0·2 (0·0 to 0·4)	0·2 (0·0 to 0·4)	0·0 (0·0 to 0·0)
Greenland	2013	6421·3 (6272·2 to 6578·8)	6420·8 (6271·2 to 6577·8)	0·2 (0·0 to 0·5)	0·3 (0·0 to 0·5)	0·0 (0·0 to 0·0)	5251·6 (5129·7 to 5380·5)	5251·2 (5128·9 to 5379·7)	0·2 (0·0 to 0·4)	0·2 (0·0 to 0·4)	0·0 (0·0 to 0·0)
Greenland	2014	6376·4 (6213·0 to 6538·4)	6375·9 (6212·5 to 6538·4)	0·3 (0·0 to 0·5)	0·3 (0·0 to 0·5)	0·0 (0·0 to 0·0)	5215·0 (5081·3 to 5347·5)	5214·5 (5080·9 to 5347·5)	0·2 (0·0 to 0·4)	0·2 (0·0 to 0·4)	0·0 (0·0 to 0·0)
Greenland	2015	5943·0 (5804·5 to 6102·5)	5942·4 (5803·9 to 6102·0)	0·3 (0·0 to 0·5)	0·3 (0·0 to 0·5)	0·0 (0·0 to 0·0)	4860·5 (4747·2 to 4990·9)	4860·0 (4746·8 to 4990·5)	0·2 (0·0 to 0·4)	0·2 (0·0 to 0·4)	0·0 (0·0 to 0·0)
Greenland	2016	5970·2 (5777·0 to 6178·9)	5969·7 (5777·0 to 6177·8)	0·3 (0·0 to 0·5)	0·3 (0·0 to 0·5)	0·0 (0·0 to 0·0)	4882·8 (4724·8 to 5053·4)	4882·3 (4724·8 to 5052·5)	0·2 (0·0 to 0·4)	0·2 (0·0 to 0·4)	0·0 (0·0 to 0·0)
Greenland	2017	6066·8 (5715·5 to 6444·7)	6066·2 (5714·5 to 6443·7)	0·3 (0·0 to 0·5)	0·3 (0·0 to 0·5)	0·0 (0·0 to 0·0)	4961·7 (4674·4 to 5270·8)	4961·3 (4673·6 to 5270·0)	0·2 (0·0 to 0·4)	0·2 (0·0 to 0·4)	0·0 (0·0 to 0·0)
Greenland	2018	6367·7 (5789·6 to 6952·8)	6367·2 (5789·5 to 6952·8)	0·2 (0·0 to 0·6)	0·2 (0·0 to 0·6)	0·0 (0·0 to 0·0)	5207·8 (4735·0 to 5686·4)	5207·4 (4735·0 to 5686·4)	0·2 (0·0 to 0·5)	0·2 (0·0 to 0·5)	0·0 (0·0 to 0·0)

Greenland	2019	6579·0 (5965·9 to 7194·4)	6578·5 (5964·8 to 7193·8)	0·3 (0·0 to 0·6)	0·3 (0·0 to 0·6)	0·0 (0·0 to 0·0)	5380·6 (4879·2 to 5884·0)	5380·2 (4878·3 to 5883·5)	0·2 (0·0 to 0·5)	0·2 (0·0 to 0·5)	0·0 (0·0 to 0·0)
Greenland	2020	6570·8 (5950·7 to 7161·0)	6570·3 (5950·6 to 7161·0)	0·2 (0·0 to 0·5)	0·3 (0·0 to 0·6)	0·0 (0·0 to 0·0)	5374·0 (4866·8 to 5856·6)	5373·6 (4866·7 to 5856·6)	0·2 (0·0 to 0·4)	0·2 (0·0 to 0·5)	0·0 (0·0 to 0·0)
Greenland	2021	6340·8 (5742·4 to 6910·3)	6340·3 (5742·2 to 6910·2)	0·2 (0·0 to 0·5)	0·3 (0·0 to 0·6)	0·0 (0·0 to 0·0)	5185·8 (4696·4 to 5651·6)	5185·4 (4696·3 to 5651·5)	0·2 (0·0 to 0·4)	0·2 (0·0 to 0·5)	0·0 (0·0 to 0·0)
Greenland	2022	5943·6 (5344·9 to 6525·7)	5943·1 (5344·2 to 6525·7)	0·2 (0·0 to 0·5)	0·3 (0·0 to 0·6)	0·0 (0·0 to 0·0)	4861·0 (4371·4 to 5337·1)	4860·5 (4370·8 to 5337·0)	0·2 (0·0 to 0·4)	0·2 (0·0 to 0·5)	0·0 (0·0 to 0·0)
Greenland	2023	5963·0 (5350·8 to 6558·8)	5962·4 (5350·8 to 6558·2)	0·2 (0·0 to 0·5)	0·3 (0·0 to 0·7)	0·0 (0·0 to 0·0)	4876·8 (4376·2 to 5364·1)	4876·4 (4376·2 to 5363·6)	0·2 (0·0 to 0·4)	0·2 (0·0 to 0·5)	0·0 (0·0 to 0·0)
Greenland	2024	5981·1 (5361·9 to 6585·2)	5980·5 (5361·2 to 6585·1)	0·2 (0·0 to 0·5)	0·3 (0·0 to 0·7)	0·0 (0·0 to 0·0)	4891·6 (4385·3 to 5385·7)	4891·2 (4384·6 to 5385·7)	0·2 (0·0 to 0·4)	0·3 (0·0 to 0·6)	0·0 (0·0 to 0·0)
Greenland	2025	5991·5 (5386·5 to 6602·3)	5990·9 (5385·3 to 6602·2)	0·2 (0·0 to 0·5)	0·3 (0·0 to 0·7)	0·0 (0·0 to 0·0)	4900·1 (4405·3 to 5399·7)	4899·7 (4404·4 to 5399·6)	0·2 (0·0 to 0·4)	0·3 (0·0 to 0·6)	0·0 (0·0 to 0·0)
Greenland	2026	6000·7 (5397·1 to 6608·7)	6000·2 (5395·9 to 6608·6)	0·2 (0·0 to 0·5)	0·4 (0·1 to 0·8)	0·0 (0·0 to 0·0)	4907·7 (4414·0 to 5404·9)	4907·2 (4413·1 to 5404·9)	0·2 (0·0 to 0·4)	0·3 (0·0 to 0·6)	0·0 (0·0 to 0·0)
Greenland	2027	6011·4 (5395·4 to 6616·6)	6010·8 (5395·3 to 6615·4)	0·2 (0·0 to 0·5)	0·4 (0·1 to 0·8)	0·0 (0·0 to 0·0)	4916·4 (4412·6 to 5411·4)	4915·9 (4412·5 to 5410·4)	0·2 (0·0 to 0·4)	0·3 (0·1 to 0·6)	0·0 (0·0 to 0·0)
Greenland	2028	6013·8 (5368·4 to 6639·0)	6013·2 (5368·3 to 6638·9)	0·2 (0·0 to 0·5)	0·4 (0·1 to 0·8)	0·0 (0·0 to 0·0)	4918·4 (4390·5 to 5429·7)	4917·9 (4390·4 to 5429·6)	0·2 (0·0 to 0·4)	0·3 (0·1 to 0·7)	0·0 (0·0 to 0·0)
Greenland	2029	6015·6 (5365·8 to 6656·3)	6014·9 (5365·6 to 6655·6)	0·2 (0·0 to 0·5)	0·4 (0·1 to 0·9)	0·0 (0·0 to 0·0)	4919·8 (4388·4 to 5443·9)	4919·3 (4388·3 to 5443·3)	0·2 (0·0 to 0·4)	0·3 (0·1 to 0·7)	0·0 (0·0 to 0·0)
Greenland	2030	6012·7 (5350·1 to 6673·8)	6012·0 (5348·9 to 6672·6)	0·2 (0·0 to 0·5)	0·4 (0·1 to 0·9)	0·0 (0·0 to 0·0)	4917·5 (4375·6 to 5458·2)	4917·0 (4374·6 to 5457·2)	0·2 (0·0 to 0·4)	0·3 (0·1 to 0·7)	0·0 (0·0 to 0·0)
Greenland	2031	6017·9 (5345·1 to 6668·6)	6017·2 (5344·5 to 6667·9)	0·2 (0·0 to 0·5)	0·4 (0·1 to 0·9)	0·0 (0·0 to 0·0)	4921·7 (4371·5 to 5453·9)	4921·2 (4371·0 to 5453·3)	0·2 (0·0 to 0·4)	0·4 (0·1 to 0·8)	0·0 (0·0 to 0·0)
Greenland	2032	6039·5 (5367·9 to 6695·9)	6038·8 (5367·1 to 6694·6)	0·2 (0·0 to 0·5)	0·5 (0·1 to 1·0)	0·0 (0·0 to 0·0)	4939·4 (4390·1 to 5476·2)	4938·9 (4389·5 to 5475·2)	0·2 (0·0 to 0·4)	0·4 (0·1 to 0·8)	0·0 (0·0 to 0·0)

Greenland	2033	6074·5 (5392·8 to 6752·4)	6073·8 (5392·7 to 6751·7)	0·2 (0·0 to 0·5)	0·5 (0·1 to 1·0)	0·0 (0·0 to 0·0)	4968·1 (4410·5 to 5522·4)	4967·5 (4410·4 to 5521·9)	0·2 (0·0 to 0·4)	0·4 (0·1 to 0·8)	0·0 (0·0 to 0·0)
Greenland	2034	6118·1 (5447·1 to 6798·7)	6117·4 (5445·8 to 6797·4)	0·2 (0·0 to 0·5)	0·5 (0·1 to 1·1)	0·0 (0·0 to 0·0)	5003·7 (4454·9 to 5560·3)	5003·1 (4453·9 to 5559·2)	0·2 (0·0 to 0·4)	0·4 (0·1 to 0·9)	0·0 (0·0 to 0·0)
Greenland	2035	6170·7 (5481·6 to 6855·4)	6169·9 (5481·3 to 6854·6)	0·2 (0·0 to 0·5)	0·5 (0·1 to 1·1)	0·0 (0·0 to 0·0)	5046·7 (4483·1 to 5606·7)	5046·1 (4482·9 to 5606·0)	0·2 (0·0 to 0·4)	0·4 (0·1 to 0·9)	0·0 (0·0 to 0·0)
Greenland	2036	6237·7 (5550·8 to 6932·8)	6236·9 (5550·2 to 6932·5)	0·2 (0·0 to 0·5)	0·6 (0·1 to 1·2)	0·0 (0·0 to 0·0)	5101·5 (4539·7 to 5670·0)	5100·9 (4539·2 to 5669·8)	0·2 (0·0 to 0·4)	0·5 (0·1 to 0·9)	0·0 (0·0 to 0·0)
Greenland	2037	6321·8 (5626·3 to 7045·9)	6321·0 (5624·9 to 7045·0)	0·2 (0·0 to 0·5)	0·6 (0·1 to 1·2)	0·0 (0·0 to 0·0)	5170·3 (4601·5 to 5762·5)	5169·7 (4600·3 to 5761·8)	0·2 (0·0 to 0·4)	0·5 (0·1 to 1·0)	0·0 (0·0 to 0·0)
Greenland	2038	6416·7 (5708·5 to 7151·1)	6415·8 (5707·0 to 7150·9)	0·2 (0·0 to 0·5)	0·6 (0·2 to 1·3)	0·0 (0·0 to 0·0)	5247·9 (4668·7 to 5848·6)	5247·2 (4667·5 to 5848·3)	0·2 (0·0 to 0·4)	0·5 (0·1 to 1·0)	0·0 (0·0 to 0·0)
Greenland	2039	6525·9 (5789·9 to 7268·1)	6525·0 (5788·5 to 7267·9)	0·2 (0·0 to 0·5)	0·6 (0·2 to 1·3)	0·0 (0·0 to 0·0)	5337·2 (4735·3 to 5944·2)	5336·5 (4734·2 to 5944·0)	0·2 (0·0 to 0·4)	0·5 (0·1 to 1·1)	0·0 (0·0 to 0·0)
Greenland	2040	6641·2 (5893·3 to 7391·7)	6640·3 (5892·5 to 7390·7)	0·2 (0·0 to 0·5)	0·7 (0·2 to 1·4)	0·0 (0·0 to 0·0)	5431·5 (4819·8 to 6045·3)	5430·8 (4819·2 to 6044·5)	0·2 (0·0 to 0·4)	0·6 (0·1 to 1·2)	0·0 (0·0 to 0·0)
Greenland	2041	6755·3 (5968·2 to 7521·5)	6754·3 (5967·8 to 7520·4)	0·2 (0·0 to 0·5)	0·7 (0·2 to 1·5)	0·0 (0·0 to 0·0)	5524·8 (4881·1 to 6151·5)	5524·0 (4880·7 to 6150·6)	0·2 (0·0 to 0·4)	0·6 (0·2 to 1·2)	0·0 (0·0 to 0·0)
Greenland	2042	6870·2 (6092·1 to 7667·9)	6869·2 (6090·4 to 7666·8)	0·2 (0·0 to 0·5)	0·7 (0·2 to 1·6)	0·0 (0·0 to 0·0)	5618·8 (4982·5 to 6271·2)	5618·0 (4981·1 to 6270·3)	0·2 (0·0 to 0·4)	0·6 (0·2 to 1·3)	0·0 (0·0 to 0·0)
Greenland	2043	6977·8 (6168·1 to 7811·6)	6976·8 (6166·4 to 7810·5)	0·2 (0·0 to 0·6)	0·8 (0·2 to 1·7)	0·0 (0·0 to 0·0)	5706·8 (5044·6 to 6388·8)	5706·0 (5043·2 to 6387·9)	0·2 (0·0 to 0·5)	0·6 (0·2 to 1·4)	0·0 (0·0 to 0·0)
Greenland	2044	7080·7 (6246·4 to 7950·4)	7079·6 (6244·7 to 7949·2)	0·2 (0·0 to 0·6)	0·8 (0·2 to 1·7)	0·0 (0·0 to 0·0)	5790·9 (5108·7 to 6502·2)	5790·0 (5107·2 to 6501·2)	0·2 (0·0 to 0·5)	0·7 (0·2 to 1·4)	0·0 (0·0 to 0·0)
Greenland	2045	7184·7 (6334·0 to 8077·0)	7183·6 (6332·4 to 8075·8)	0·2 (0·0 to 0·6)	0·9 (0·2 to 1·8)	0·0 (0·0 to 0·0)	5876·0 (5180·3 to 6605·8)	5875·1 (5178·9 to 6604·8)	0·2 (0·0 to 0·5)	0·7 (0·2 to 1·5)	0·0 (0·0 to 0·0)
Greenland	2046	7289·7 (6465·4 to 8195·2)	7288·5 (6463·7 to 8193·9)	0·3 (0·0 to 0·6)	0·9 (0·2 to 1·9)	0·0 (0·0 to 0·0)	5961·9 (5287·7 to 6702·4)	5960·9 (5286·3 to 6701·4)	0·2 (0·0 to 0·5)	0·7 (0·2 to 1·6)	0·0 (0·0 to 0·0)

Greenland	2047	7384·1 (6521·7 to 8311·2)	7382·9 (6521·3 to 8309·9)	0·3 (0·0 to 0·6)	0·9 (0·3 to 2·0)	0·0 (0·0 to 0·0)	6039·1 (5333·8 to 6797·3)	6038·1 (5333·4 to 6796·2)	0·2 (0·0 to 0·5)	0·8 (0·2 to 1·6)	0·0 (0·0 to 0·0)
Greenland	2048	7474·8 (6582·9 to 8434·0)	7473·6 (6581·1 to 8432·6)	0·3 (0·0 to 0·6)	1·0 (0·3 to 2·1)	0·0 (0·0 to 0·0)	6113·3 (5383·8 to 6897·8)	6112·3 (5382·3 to 6896·7)	0·2 (0·0 to 0·5)	0·8 (0·2 to 1·7)	0·0 (0·0 to 0·0)
Greenland	2049	7565·9 (6668·3 to 8517·2)	7564·6 (6666·5 to 8515·1)	0·3 (0·0 to 0·6)	1·0 (0·3 to 2·2)	0·0 (0·0 to 0·0)	6187·7 (5453·7 to 6965·8)	6186·7 (5452·2 to 6964·1)	0·2 (0·0 to 0·5)	0·8 (0·2 to 1·8)	0·0 (0·0 to 0·0)
Greenland	2050	7651·4 (6719·1 to 8605·8)	7650·0 (6717·5 to 8605·3)	0·3 (0·0 to 0·6)	1·1 (0·3 to 2·3)	0·0 (0·0 to 0·0)	6257·7 (5495·3 to 7038·3)	6256·6 (5493·9 to 7037·8)	0·2 (0·0 to 0·5)	0·9 (0·2 to 1·9)	0·0 (0·0 to 0·0)
Guatemala*	1995	149·1 (133·8 to 165·5)	50·9 (43·4 to 59·2)	92·2 (79·1 to 106·1)	3·2 (2·1 to 4·5)	2·9 (2·9 to 2·9)	290·8 (260·9 to 322·7)	99·2 (84·7 to 115·4)	179·8 (154·2 to 206·8)	6·2 (4·2 to 8·8)	5·6 (5·6 to 5·6)
Guatemala*	1996	146·8 (133·0 to 161·6)	50·2 (42·6 to 57·9)	90·9 (79·1 to 103·4)	3·2 (2·2 to 4·5)	2·5 (2·5 to 2·5)	286·2 (259·4 to 315·1)	97·9 (83·1 to 112·9)	177·2 (154·2 to 201·6)	6·2 (4·2 to 8·9)	4·9 (4·9 to 4·9)
Guatemala*	1997	164·0 (150·8 to 177·5)	54·1 (46·3 to 62·3)	92·7 (81·7 to 104·5)	3·4 (2·3 to 4·8)	13·8 (13·8 to 13·8)	319·9 (294·0 to 346·1)	105·4 (90·2 to 121·4)	180·8 (159·3 to 203·9)	6·6 (4·5 to 9·3)	27·0 (27·0 to 27·0)
Guatemala*	1998	163·9 (150·4 to 178·1)	59·9 (51·9 to 68·1)	96·9 (85·5 to 109·4)	3·8 (2·6 to 5·3)	3·3 (3·3 to 3·3)	319·5 (293·2 to 347·3)	116·8 (101·3 to 132·8)	189·0 (166·8 to 213·3)	7·3 (5·0 to 10·3)	6·4 (6·4 to 6·4)
Guatemala*	1999	181·7 (166·7 to 196·7)	64·9 (56·3 to 73·9)	104·9 (92·9 to 117·8)	5·1 (3·6 to 7·1)	6·7 (6·7 to 6·7)	354·2 (325·0 to 383·5)	126·6 (109·7 to 144·2)	204·6 (181·2 to 229·6)	10·0 (6·9 to 13·9)	13·1 (13·1 to 13·1)
Guatemala*	2000	194·3 (178·6 to 209·8)	67·5 (59·2 to 76·7)	116·8 (105·1 to 129·8)	5·9 (4·0 to 8·2)	4·2 (4·2 to 4·2)	379·0 (348·2 to 409·1)	131·5 (115·3 to 149·5)	227·7 (205·0 to 253·1)	11·4 (7·8 to 16·0)	8·3 (8·3 to 8·3)
Guatemala*	2001	212·6 (197·1 to 229·5)	70·7 (62·3 to 79·6)	129·4 (117·0 to 142·1)	6·8 (4·7 to 9·5)	5·8 (5·8 to 5·8)	414·5 (384·3 to 447·4)	137·8 (121·5 to 155·3)	252·3 (228·2 to 277·1)	13·2 (9·2 to 18·6)	11·3 (11·3 to 11·3)
Guatemala*	2002	217·4 (202·6 to 233·9)	71·1 (62·3 to 80·1)	135·5 (123·2 to 149·5)	7·3 (5·0 to 10·3)	3·6 (3·6 to 3·6)	423·9 (395·1 to 456·1)	138·6 (121·5 to 156·2)	264·2 (240·2 to 291·6)	14·2 (9·7 to 20·0)	7·0 (7·0 to 7·0)
Guatemala*	2003	231·3 (215·8 to 248·7)	76·4 (67·4 to 86·4)	142·5 (129·9 to 156·2)	7·6 (5·1 to 10·7)	4·8 (4·8 to 4·8)	451·0 (420·8 to 484·9)	149·0 (131·5 to 168·5)	277·9 (253·4 to 304·5)	14·9 (10·0 to 20·9)	9·4 (9·4 to 9·4)
Guatemala*	2004	227·4 (212·1 to 245·0)	72·9 (64·2 to 82·3)	142·3 (129·6 to 155·8)	9·0 (6·0 to 12·9)	3·1 (3·1 to 3·1)	443·4 (413·6 to 477·7)	142·2 (125·1 to 160·5)	277·5 (252·6 to 303·8)	17·5 (11·7 to 25·1)	6·1 (6·1 to 6·1)

Guatemala*	2005	232·6 (216·8 to 249·6)	74·3 (65·7 to 83·6)	145·6 (132·4 to 159·0)	9·3 (6·2 to 13·1)	3·4 (3·4 to 3·4)	453·6 (422·8 to 486·6)	145·0 (128·2 to 162·9)	283·9 (258·1 to 310·1)	18·0 (12·0 to 25·5)	6·6 (6·6 to 6·6)
Guatemala*	2006	243·9 (227·7 to 260·3)	80·3 (71·0 to 90·0)	151·0 (137·8 to 164·0)	8·6 (5·8 to 12·1)	4·0 (4·0 to 4·0)	475·6 (444·0 to 507·6)	156·6 (138·5 to 175·5)	294·5 (268·7 to 319·8)	16·7 (11·2 to 23·6)	7·8 (7·8 to 7·8)
Guatemala*	2007	248·8 (232·5 to 265·5)	82·5 (73·2 to 92·4)	152·1 (139·1 to 165·7)	9·4 (6·3 to 13·1)	4·8 (4·8 to 4·8)	485·2 (453·3 to 517·7)	160·9 (142·7 to 180·2)	296·6 (271·2 to 323·1)	18·3 (12·3 to 25·6)	9·4 (9·4 to 9·4)
Guatemala*	2008	249·9 (233·8 to 266·5)	82·9 (73·5 to 92·5)	149·2 (135·9 to 162·6)	10·7 (7·2 to 14·6)	7·2 (7·2 to 7·2)	487·2 (456·0 to 519·7)	161·6 (143·3 to 180·4)	290·9 (265·0 to 317·1)	20·8 (14·1 to 28·6)	14·0 (14·0 to 14·0)
Guatemala*	2009	250·7 (234·5 to 268·1)	86·3 (76·8 to 96·3)	148·1 (135·1 to 161·4)	10·7 (7·1 to 14·8)	5·6 (5·6 to 5·6)	488·8 (457·2 to 522·8)	168·4 (149·7 to 187·7)	288·7 (263·5 to 314·7)	20·8 (13·9 to 28·8)	10·9 (10·9 to 10·9)
Guatemala*	2010	250·5 (233·9 to 268·1)	87·3 (77·9 to 97·5)	146·1 (134·0 to 158·8)	11·3 (7·6 to 15·5)	5·8 (5·8 to 5·8)	488·5 (456·0 to 522·8)	170·2 (151·8 to 190·2)	285·0 (261·3 to 309·7)	22·1 (14·9 to 30·3)	11·2 (11·2 to 11·2)
Guatemala*	2011	252·6 (235·9 to 269·3)	89·2 (79·7 to 100·1)	144·7 (132·6 to 156·9)	11·4 (7·7 to 15·6)	7·3 (7·3 to 7·3)	492·5 (459·9 to 525·1)	173·9 (155·4 to 195·1)	282·1 (258·7 to 305·9)	22·3 (15·1 to 30·5)	14·2 (14·2 to 14·2)
Guatemala*	2012	250·4 (235·0 to 266·3)	91·0 (81·5 to 101·4)	143·0 (132·6 to 154·8)	11·4 (7·9 to 15·5)	4·9 (4·9 to 4·9)	488·2 (458·2 to 519·3)	177·5 (158·9 to 197·7)	278·8 (258·5 to 301·9)	22·3 (15·4 to 30·2)	9·6 (9·6 to 9·6)
Guatemala*	2013	257·9 (243·6 to 273·0)	95·9 (85·9 to 106·2)	142·1 (133·3 to 152·0)	11·5 (8·1 to 15·5)	8·4 (8·4 to 8·4)	502·8 (475·0 to 532·4)	187·0 (167·5 to 207·0)	277·0 (259·9 to 296·4)	22·4 (15·8 to 30·3)	16·3 (16·3 to 16·3)
Guatemala*	2014	261·3 (246·5 to 276·5)	100·3 (89·5 to 110·3)	145·7 (135·9 to 156·3)	11·6 (8·2 to 15·8)	3·6 (3·6 to 3·6)	509·5 (480·6 to 539·1)	195·6 (174·6 to 215·1)	284·1 (265·0 to 304·8)	22·7 (16·1 to 30·8)	7·1 (7·1 to 7·1)
Guatemala*	2015	283·9 (268·3 to 299·4)	100·9 (90·4 to 111·3)	149·3 (138·9 to 160·3)	13·1 (9·4 to 18·1)	20·6 (20·6 to 20·6)	553·6 (523·2 to 583·9)	196·7 (176·3 to 217·1)	291·2 (270·8 to 312·5)	25·5 (18·3 to 35·4)	40·2 (40·2 to 40·2)
Guatemala*	2016	276·6 (261·2 to 292·0)	103·8 (93·4 to 114·5)	155·1 (145·4 to 165·7)	14·9 (10·8 to 20·5)	2·8 (2·8 to 2·8)	539·4 (509·3 to 569·3)	202·5 (182·2 to 223·3)	302·4 (283·6 to 323·0)	29·1 (21·1 to 40·0)	5·4 (5·4 to 5·4)
Guatemala*	2017	289·0 (273·1 to 304·4)	106·0 (94·5 to 118·1)	160·6 (152·7 to 168·8)	18·9 (13·6 to 25·9)	3·4 (3·4 to 3·4)	563·4 (532·6 to 593·5)	206·7 (184·2 to 230·3)	313·2 (297·7 to 329·2)	36·9 (26·6 to 50·6)	6·7 (6·7 to 6·7)
Guatemala*	2018	300·2 (283·6 to 317·4)	109·5 (96·7 to 123·1)	168·2 (159·0 to 178·5)	17·0 (11·8 to 23·1)	5·6 (5·6 to 5·6)	585·4 (553·0 to 619·0)	213·4 (188·5 to 240·0)	328·0 (310·0 to 348·1)	33·2 (23·1 to 45·0)	10·9 (10·9 to 10·9)

Guatemala*	2019	308·3 (290·9 to 326·1)	114·4 (101·2 to 129·2)	170·6 (161·3 to 181·1)	17·6 (12·2 to 23·9)	5·6 (5·6 to 5·6)	601·1 (567·2 to 635·9)	223·1 (197·3 to 251·9)	332·8 (314·5 to 353·1)	34·4 (23·9 to 46·5)	10·9 (10·8 to 10·9)
Guatemala*	2020	307·1 (289·4 to 325·6)	124·7 (110·6 to 141·0)	157·5 (148·9 to 167·2)	16·7 (11·7 to 22·6)	8·2 (8·1 to 8·5)	598·9 (564·4 to 634·9)	243·1 (215·7 to 274·9)	307·2 (290·3 to 326·0)	32·6 (22·8 to 44·1)	16·1 (15·8 to 16·6)
Guatemala*	2021	298·6 (281·1 to 316·7)	121·3 (107·3 to 137·2)	152·8 (144·4 to 162·2)	16·6 (11·6 to 22·6)	7·9 (7·8 to 8·2)	582·2 (548·2 to 617·6)	236·5 (209·3 to 267·5)	297·9 (281·6 to 316·2)	32·4 (22·6 to 44·0)	15·4 (15·2 to 16·0)
Guatemala*	2022	287·2 (270·7 to 304·3)	109·4 (96·4 to 123·4)	154·6 (146·1 to 164·2)	17·2 (12·0 to 23·3)	6·0 (5·7 to 6·4)	560·1 (527·9 to 593·4)	213·2 (188·0 to 240·6)	301·5 (284·8 to 320·3)	33·6 (23·5 to 45·5)	11·7 (11·1 to 12·5)
Guatemala*	2023	292·8 (275·8 to 310·4)	112·4 (98·9 to 126·8)	156·4 (147·8 to 166·3)	17·9 (12·5 to 24·3)	6·0 (5·7 to 6·5)	570·8 (537·9 to 605·4)	219·1 (192·8 to 247·3)	305·1 (288·2 to 324·2)	34·9 (24·4 to 47·3)	11·7 (11·0 to 12·6)
Guatemala*	2024	298·3 (280·9 to 316·4)	115·4 (101·6 to 130·2)	158·2 (149·4 to 168·1)	18·6 (13·0 to 25·2)	6·1 (5·7 to 6·5)	581·6 (547·8 to 616·9)	225·0 (198·2 to 253·9)	308·5 (291·4 to 327·8)	36·2 (25·4 to 49·1)	11·8 (11·1 to 12·8)
Guatemala*	2025	303·7 (285·7 to 322·3)	118·4 (104·1 to 133·7)	159·9 (151·0 to 169·7)	19·3 (13·5 to 26·1)	6·1 (5·7 to 6·6)	592·2 (557·0 to 628·5)	231·0 (203·0 to 260·8)	311·8 (294·4 to 331·0)	37·6 (26·3 to 51·0)	11·9 (11·1 to 12·9)
Guatemala*	2026	309·2 (290·8 to 328·4)	121·5 (106·5 to 137·3)	161·5 (152·6 to 171·6)	20·0 (14·0 to 27·2)	6·1 (5·7 to 6·7)	602·9 (567·0 to 640·4)	237·0 (207·7 to 267·8)	315·0 (297·5 to 334·6)	39·0 (27·2 to 53·1)	12·0 (11·1 to 13·0)
Guatemala*	2027	314·5 (295·5 to 334·4)	124·6 (108·8 to 140·8)	163·0 (153·9 to 173·2)	20·7 (14·4 to 28·3)	6·2 (5·7 to 6·8)	613·2 (576·2 to 652·0)	242·9 (212·1 to 274·6)	317·9 (300·0 to 337·7)	40·4 (28·2 to 55·3)	12·1 (11·1 to 13·2)
Guatemala*	2028	319·9 (300·2 to 340·5)	127·7 (111·4 to 144·4)	164·5 (155·2 to 174·8)	21·4 (14·9 to 29·3)	6·2 (5·7 to 6·9)	623·7 (585·4 to 663·9)	248·9 (217·3 to 281·5)	320·8 (302·7 to 340·8)	41·8 (29·1 to 57·2)	12·2 (11·1 to 13·5)
Guatemala*	2029	325·4 (305·0 to 346·6)	131·0 (114·2 to 148·4)	165·9 (156·6 to 176·3)	22·2 (15·4 to 30·4)	6·3 (5·7 to 7·0)	634·6 (594·8 to 675·8)	255·5 (222·7 to 289·3)	323·5 (305·3 to 343·7)	43·3 (30·1 to 59·2)	12·3 (11·2 to 13·7)
Guatemala*	2030	331·2 (310·3 to 353·0)	134·5 (117·3 to 152·7)	167·3 (158·0 to 177·8)	23·0 (16·0 to 31·4)	6·4 (5·7 to 7·1)	645·8 (605·1 to 688·4)	262·3 (228·6 to 297·7)	326·3 (308·1 to 346·7)	44·8 (31·2 to 61·3)	12·4 (11·2 to 13·8)
Guatemala*	2031	337·0 (315·4 to 359·6)	138·0 (120·0 to 157·1)	168·8 (159·3 to 179·4)	23·8 (16·5 to 32·6)	6·4 (5·8 to 7·2)	657·2 (615·0 to 701·2)	269·2 (234·0 to 306·4)	329·1 (310·6 to 349·8)	46·4 (32·2 to 63·6)	12·5 (11·2 to 14·1)
Guatemala*	2032	343·0 (320·8 to 366·3)	141·6 (123·2 to 161·2)	170·3 (160·8 to 181·0)	24·6 (17·0 to 33·7)	6·5 (5·8 to 7·3)	668·8 (625·6 to 714·2)	276·2 (240·2 to 314·4)	332·0 (313·5 to 352·9)	48·0 (33·2 to 65·8)	12·6 (11·3 to 14·2)

Guatemala*	2033	349·4 (326·4 to 373·4)	145·5 (126·3 to 166·3)	171·9 (162·4 to 182·6)	25·5 (17·6 to 34·9)	6·5 (5·8 to 7·4)	681·3 (636·5 to 728·2)	283·6 (246·2 to 324·2)	335·2 (316·6 to 356·1)	49·8 (34·3 to 68·1)	12·7 (11·3 to 14·4)
Guatemala*	2034	356·0 (331·8 to 380·9)	149·4 (129·4 to 171·0)	173·5 (163·9 to 184·3)	26·5 (18·2 to 36·2)	6·6 (5·9 to 7·4)	694·1 (647·0 to 742·8)	291·3 (252·3 to 333·5)	338·4 (319·7 to 359·3)	51·6 (35·5 to 70·5)	12·8 (11·5 to 14·5)
Guatemala*	2035	362·8 (337·9 to 388·6)	153·4 (132·8 to 175·8)	175·3 (165·7 to 186·1)	27·5 (18·8 to 37·7)	6·6 (5·9 to 7·5)	707·4 (658·9 to 757·7)	299·1 (259·0 to 342·8)	341·8 (323·0 to 362·8)	53·5 (36·7 to 73·6)	12·9 (11·5 to 14·6)
Guatemala*	2036	369·7 (343·6 to 396·3)	157·5 (136·2 to 181·0)	177·1 (167·4 to 187·9)	28·5 (19·3 to 39·3)	6·7 (5·9 to 7·6)	721·0 (670·1 to 772·8)	307·1 (265·7 to 352·8)	345·3 (326·4 to 366·3)	55·6 (37·7 to 76·6)	13·0 (11·5 to 14·8)
Guatemala*	2037	377·0 (350·2 to 404·9)	161·7 (139·6 to 186·0)	179·0 (169·1 to 189·9)	29·6 (20·0 to 40·8)	6·7 (5·9 to 7·7)	735·2 (682·8 to 789·5)	315·4 (272·2 to 362·6)	349·0 (329·8 to 370·2)	57·7 (39·1 to 79·5)	13·1 (11·6 to 15·0)
Guatemala*	2038	384·1 (356·6 to 413·0)	165·8 (142·3 to 190·8)	180·8 (170·7 to 191·7)	30·7 (20·8 to 42·5)	6·8 (6·0 to 7·8)	749·0 (695·4 to 805·3)	323·4 (277·6 to 372·1)	352·5 (332·9 to 373·9)	59·9 (40·6 to 82·9)	13·2 (11·7 to 15·2)
Guatemala*	2039	390·9 (362·3 to 420·4)	169·8 (145·4 to 195·5)	182·3 (172·0 to 193·4)	31·9 (21·6 to 44·7)	6·9 (6·0 to 7·9)	762·2 (706·5 to 819·7)	331·1 (283·6 to 381·3)	355·5 (335·5 to 377·1)	62·1 (42·0 to 87·1)	13·4 (11·8 to 15·4)
Guatemala*	2040	397·4 (367·8 to 427·4)	173·7 (148·7 to 199·9)	183·6 (173·3 to 194·9)	33·0 (22·2 to 46·6)	7·0 (6·1 to 8·2)	774·9 (717·3 to 833·3)	338·8 (290·0 to 389·9)	358·0 (338·0 to 380·0)	64·4 (43·3 to 90·9)	13·7 (12·0 to 15·9)
Guatemala*	2041	403·7 (373·1 to 434·6)	177·5 (151·7 to 204·3)	184·8 (174·4 to 196·2)	34·2 (22·9 to 48·7)	7·2 (6·2 to 8·4)	787·2 (727·6 to 847·4)	346·2 (295·9 to 398·3)	360·3 (340·1 to 382·6)	66·7 (44·6 to 95·0)	14·0 (12·2 to 16·3)
Guatemala*	2042	410·0 (378·7 to 442·3)	181·4 (154·7 to 208·9)	185·9 (175·2 to 197·6)	35·4 (23·5 to 50·6)	7·4 (6·4 to 8·7)	799·5 (738·5 to 862·4)	353·6 (301·6 to 407·3)	362·4 (341·7 to 385·3)	69·0 (45·8 to 98·6)	14·4 (12·4 to 16·9)
Guatemala*	2043	416·4 (384·1 to 449·9)	185·2 (157·7 to 213·6)	186·9 (176·1 to 198·9)	36·7 (24·1 to 52·9)	7·6 (6·5 to 9·0)	811·9 (748·9 to 877·3)	361·2 (307·4 to 416·5)	364·5 (343·4 to 387·9)	71·5 (47·0 to 103·1)	14·8 (12·7 to 17·6)
Guatemala*	2044	422·6 (389·2 to 457·0)	189·0 (160·7 to 218·0)	187·8 (176·9 to 199·9)	38·0 (24·8 to 55·0)	7·8 (6·7 to 9·3)	824·1 (758·9 to 891·1)	368·6 (313·4 to 425·1)	366·2 (344·9 to 389·8)	74·0 (48·4 to 107·2)	15·2 (13·0 to 18·1)
Guatemala*	2045	428·8 (394·0 to 464·1)	192·8 (163·8 to 222·5)	188·6 (177·5 to 200·9)	39·3 (25·4 to 57·2)	8·1 (6·9 to 9·7)	836·1 (768·3 to 904·9)	376·0 (319·4 to 433·9)	367·8 (346·0 to 391·7)	76·6 (49·5 to 111·5)	15·7 (13·4 to 18·9)
Guatemala*	2046	435·0 (398·7 to 471·2)	196·6 (166·9 to 227·1)	189·4 (178·0 to 201·8)	40·6 (26·0 to 59·7)	8·3 (7·1 to 10·1)	848·2 (777·5 to 918·8)	383·4 (325·4 to 442·9)	369·2 (347·1 to 393·4)	79·3 (50·7 to 116·5)	16·3 (13·8 to 19·6)

Guatemala*	2047	441·4 (403·7 to 478·7)	200·6 (170·0 to 232·2)	190·2 (178·6 to 202·8)	42·1 (26·7 to 62·3)	8·6 (7·3 to 10·4)	860·7 (787·2 to 933·5)	391·1 (331·5 to 452·7)	370·8 (348·3 to 395·5)	82·0 (52·0 to 121·4)	16·8 (14·2 to 20·4)
Guatemala*	2048	447·8 (409·6 to 486·3)	204·5 (173·4 to 236·8)	190·9 (179·2 to 203·6)	43·5 (27·3 to 64·8)	8·9 (7·5 to 10·8)	873·2 (798·7 to 948·2)	398·8 (338·0 to 461·7)	372·3 (349·4 to 397·1)	84·8 (53·2 to 126·4)	17·4 (14·6 to 21·0)
Guatemala*	2049	454·4 (414·8 to 494·0)	208·5 (177·1 to 242·8)	191·7 (179·8 to 204·4)	45·0 (27·9 to 67·9)	9·2 (7·7 to 11·3)	886·1 (808·9 to 963·4)	406·7 (345·2 to 473·4)	373·9 (350·5 to 398·6)	87·7 (54·5 to 132·4)	17·9 (15·0 to 22·1)
Guatemala*	2050	460·9 (420·3 to 501·0)	212·5 (180·2 to 247·3)	192·4 (180·3 to 205·3)	46·5 (28·6 to 70·7)	9·5 (7·9 to 11·8)	898·8 (819·6 to 977·0)	414·4 (351·5 to 482·3)	375·2 (351·6 to 400·3)	90·6 (55·8 to 138·0)	18·6 (15·3 to 23·0)
Guam	1995	622·4 (537·9 to 723·3)	423·6 (348·7 to 517·0)	189·2 (148·0 to 236·7)	9·6 (6·6 to 13·4)	0·0 (0·0 to 0·0)	622·4 (537·9 to 723·3)	423·6 (348·7 to 517·0)	189·2 (148·0 to 236·7)	9·6 (6·6 to 13·4)	0·0 (0·0 to 0·0)
Guam	1996	630·1 (544·0 to 733·3)	431·0 (355·7 to 518·3)	189·0 (146·9 to 236·1)	10·1 (6·8 to 14·1)	0·0 (0·0 to 0·0)	630·1 (544·0 to 733·3)	431·0 (355·7 to 518·3)	189·0 (146·9 to 236·1)	10·1 (6·8 to 14·1)	0·0 (0·0 to 0·0)
Guam	1997	637·0 (545·3 to 740·5)	438·3 (359·2 to 529·9)	188·6 (145·3 to 238·6)	10·1 (6·8 to 14·3)	0·0 (0·0 to 0·0)	637·0 (545·3 to 740·5)	438·3 (359·2 to 529·9)	188·6 (145·3 to 238·6)	10·1 (6·8 to 14·3)	0·0 (0·0 to 0·0)
Guam	1998	637·8 (548·3 to 741·5)	441·4 (364·0 to 527·8)	187·0 (145·9 to 236·0)	9·3 (6·2 to 13·4)	0·0 (0·0 to 0·0)	637·8 (548·3 to 741·5)	441·4 (364·0 to 527·8)	187·0 (145·9 to 236·0)	9·3 (6·2 to 13·4)	0·0 (0·0 to 0·0)
Guam	1999	639·1 (543·5 to 743·7)	445·8 (368·0 to 535·3)	185·7 (145·4 to 238·2)	7·7 (5·1 to 11·0)	0·0 (0·0 to 0·0)	639·1 (543·5 to 743·7)	445·8 (368·0 to 535·3)	185·7 (145·4 to 238·2)	7·7 (5·1 to 11·0)	0·0 (0·0 to 0·0)
Guam	2000	645·2 (549·6 to 752·1)	454·3 (373·8 to 545·8)	185·3 (145·0 to 238·1)	5·6 (3·7 to 8·0)	0·0 (0·0 to 0·0)	645·2 (549·6 to 752·1)	454·3 (373·8 to 545·8)	185·3 (145·0 to 238·1)	5·6 (3·7 to 8·0)	0·0 (0·0 to 0·0)
Guam	2001	650·8 (553·6 to 761·9)	461·8 (377·0 to 556·4)	186·0 (144·5 to 234·8)	3·1 (2·1 to 4·3)	0·0 (0·0 to 0·0)	650·8 (553·6 to 761·9)	461·8 (377·0 to 556·4)	186·0 (144·5 to 234·8)	3·1 (2·1 to 4·3)	0·0 (0·0 to 0·0)
Guam	2002	663·8 (568·5 to 772·1)	470·5 (386·0 to 569·7)	190·0 (146·5 to 239·6)	3·4 (2·3 to 4·8)	0·0 (0·0 to 0·0)	663·8 (568·5 to 772·1)	470·5 (386·0 to 569·7)	190·0 (146·5 to 239·6)	3·4 (2·3 to 4·8)	0·0 (0·0 to 0·0)
Guam	2003	681·0 (584·8 to 795·2)	480·9 (393·8 to 583·1)	195·7 (151·6 to 247·4)	4·5 (3·0 to 6·2)	0·0 (0·0 to 0·0)	681·0 (584·8 to 795·2)	480·9 (393·8 to 583·1)	195·7 (151·6 to 247·4)	4·5 (3·0 to 6·2)	0·0 (0·0 to 0·0)
Guam	2004	721·7 (621·0 to 846·2)	510·9 (420·9 to 621·9)	205·4 (160·6 to 259·6)	5·5 (3·7 to 7·6)	0·0 (0·0 to 0·0)	721·7 (621·0 to 846·2)	510·9 (420·9 to 621·9)	205·4 (160·6 to 259·6)	5·5 (3·7 to 7·6)	0·0 (0·0 to 0·0)

Guam	2005	756·6 (647·8 to 883·6)	535·2 (439·8 to 651·8)	214·9 (170·3 to 270·1)	6·5 (4·4 to 9·1)	0·0 (0·0 to 0·0)	756·6 (647·8 to 883·6)	535·2 (439·8 to 651·8)	214·9 (170·3 to 270·1)	6·5 (4·4 to 9·1)	0·0 (0·0 to 0·0)
Guam	2006	757·0 (648·6 to 874·6)	530·9 (433·7 to 637·8)	218·3 (173·9 to 274·4)	7·8 (5·3 to 11·1)	0·0 (0·0 to 0·0)	757·0 (648·6 to 874·6)	530·9 (433·7 to 637·8)	218·3 (173·9 to 274·4)	7·8 (5·3 to 11·1)	0·0 (0·0 to 0·0)
Guam	2007	770·4 (660·1 to 894·0)	537·2 (440·1 to 650·4)	222·7 (175·7 to 282·4)	10·6 (7·2 to 15·1)	0·0 (0·0 to 0·0)	770·4 (660·1 to 894·0)	537·2 (440·1 to 650·4)	222·7 (175·7 to 282·4)	10·6 (7·2 to 15·1)	0·0 (0·0 to 0·0)
Guam	2008	788·0 (677·9 to 920·3)	543·4 (442·4 to 662·6)	226·5 (179·6 to 286·5)	18·1 (12·4 to 25·9)	0·0 (0·0 to 0·0)	788·0 (677·9 to 920·3)	543·4 (442·4 to 662·6)	226·5 (179·6 to 286·5)	18·1 (12·4 to 25·9)	0·0 (0·0 to 0·0)
Guam	2009	795·6 (687·3 to 922·3)	543·5 (443·0 to 665·8)	228·2 (179·0 to 288·1)	23·9 (16·6 to 33·6)	0·0 (0·0 to 0·0)	795·6 (687·3 to 922·3)	543·5 (443·0 to 665·8)	228·2 (179·0 to 288·1)	23·9 (16·6 to 33·6)	0·0 (0·0 to 0·0)
Guam	2010	810·5 (697·5 to 946·2)	551·8 (450·4 to 677·9)	230·7 (180·9 to 291·3)	28·0 (19·2 to 38·8)	0·0 (0·0 to 0·0)	810·5 (697·5 to 946·2)	551·8 (450·4 to 677·9)	230·7 (180·9 to 291·3)	28·0 (19·2 to 38·8)	0·0 (0·0 to 0·0)
Guam	2011	822·0 (706·6 to 961·4)	558·7 (458·2 to 684·0)	232·7 (184·4 to 294·9)	30·6 (20·9 to 42·6)	0·0 (0·0 to 0·0)	822·0 (706·6 to 961·4)	558·7 (458·2 to 684·0)	232·7 (184·4 to 294·9)	30·6 (20·9 to 42·6)	0·0 (0·0 to 0·0)
Guam	2012	844·5 (726·1 to 987·1)	574·9 (470·4 to 705·5)	236·5 (187·0 to 301·1)	33·1 (22·6 to 45·8)	0·0 (0·0 to 0·0)	844·5 (726·1 to 987·1)	574·9 (470·4 to 705·5)	236·5 (187·0 to 301·1)	33·1 (22·6 to 45·8)	0·0 (0·0 to 0·0)
Guam	2013	869·7 (746·9 to 1008·5)	593·5 (485·9 to 724·9)	240·8 (191·1 to 308·5)	35·4 (24·1 to 49·0)	0·0 (0·0 to 0·0)	869·7 (746·9 to 1008·5)	593·5 (485·9 to 724·9)	240·8 (191·1 to 308·5)	35·4 (24·1 to 49·0)	0·0 (0·0 to 0·0)
Guam	2014	891·6 (761·3 to 1032·5)	610·0 (495·8 to 741·7)	244·7 (192·8 to 311·3)	36·9 (25·1 to 52·0)	0·0 (0·0 to 0·0)	891·6 (761·3 to 1032·5)	610·0 (495·8 to 741·7)	244·7 (192·8 to 311·3)	36·9 (25·1 to 52·0)	0·0 (0·0 to 0·0)
Guam	2015	905·4 (776·7 to 1047·8)	620·6 (504·5 to 750·2)	247·1 (192·6 to 314·7)	37·7 (25·3 to 53·8)	0·0 (0·0 to 0·0)	905·4 (776·7 to 1047·8)	620·6 (504·5 to 750·2)	247·1 (192·6 to 314·7)	37·7 (25·3 to 53·8)	0·0 (0·0 to 0·0)
Guam	2016	915·9 (787·2 to 1065·9)	629·2 (512·3 to 772·2)	247·9 (192·8 to 314·1)	38·8 (26·5 to 54·2)	0·0 (0·0 to 0·0)	915·9 (787·2 to 1065·9)	629·2 (512·3 to 772·2)	247·9 (192·8 to 314·1)	38·8 (26·5 to 54·2)	0·0 (0·0 to 0·0)
Guam	2017	926·4 (795·5 to 1077·7)	636·6 (521·7 to 778·7)	247·9 (194·0 to 312·7)	41·9 (28·5 to 58·1)	0·0 (0·0 to 0·0)	926·4 (795·5 to 1077·7)	636·6 (521·7 to 778·7)	247·9 (194·0 to 312·7)	41·9 (28·5 to 58·1)	0·0 (0·0 to 0·0)
Guam	2018	945·7 (819·4 to 1093·0)	643·9 (530·4 to 785·0)	249·1 (195·5 to 314·0)	52·7 (36·1 to 74·4)	0·0 (0·0 to 0·0)	945·7 (819·4 to 1093·0)	643·9 (530·4 to 785·0)	249·1 (195·5 to 314·0)	52·7 (36·1 to 74·4)	0·0 (0·0 to 0·0)

Guam	2019	953·1 (825·9 to 1098·9)	650·2 (537·5 to 791·4)	248·5 (194·9 to 313·1)	54·4 (37·3 to 76·8)	0·0 (0·0 to 0·0)	953·1 (825·9 to 1098·9)	650·2 (537·5 to 791·4)	248·5 (194·9 to 313·1)	54·4 (37·3 to 76·8)	0·0 (0·0 to 0·0)
Guam	2020	932·7 (805·9 to 1077·4)	638·8 (527·8 to 777·4)	240·0 (188·4 to 302·5)	54·0 (36·9 to 76·1)	0·0 (0·0 to 0·0)	932·7 (805·9 to 1077·4)	638·8 (527·8 to 777·4)	240·0 (188·4 to 302·5)	54·0 (36·9 to 76·1)	0·0 (0·0 to 0·0)
Guam	2021	913·1 (790·8 to 1053·9)	624·4 (516·0 to 760·0)	234·5 (184·0 to 295·6)	54·1 (37·0 to 76·1)	0·0 (0·0 to 0·0)	913·1 (790·8 to 1053·9)	624·4 (516·0 to 760·0)	234·5 (184·0 to 295·6)	54·1 (37·0 to 76·1)	0·0 (0·0 to 0·0)
Guam	2022	925·6 (800·7 to 1070·3)	636·5 (525·2 to 775·8)	233·6 (183·2 to 294·2)	55·4 (38·1 to 77·9)	0·0 (0·0 to 0·0)	925·6 (800·7 to 1070·3)	636·5 (525·2 to 775·8)	233·6 (183·2 to 294·2)	55·4 (38·1 to 77·9)	0·0 (0·0 to 0·0)
Guam	2023	929·5 (804·3 to 1075·8)	640·2 (529·9 to 783·4)	232·6 (182·5 to 293·0)	56·7 (39·0 to 79·5)	0·0 (0·0 to 0·0)	929·5 (804·3 to 1075·8)	640·2 (529·9 to 783·4)	232·6 (182·5 to 293·0)	56·7 (39·0 to 79·5)	0·0 (0·0 to 0·0)
Guam	2024	931·1 (805·9 to 1078·6)	641·9 (532·4 to 785·3)	231·4 (181·4 to 291·6)	57·9 (39·7 to 80·5)	0·0 (0·0 to 0·0)	931·1 (805·9 to 1078·6)	641·9 (532·4 to 785·3)	231·4 (181·4 to 291·6)	57·9 (39·7 to 80·5)	0·0 (0·0 to 0·0)
Guam	2025	932·3 (806·3 to 1078·0)	643·0 (533·9 to 785·8)	230·1 (180·4 to 290·1)	59·2 (40·4 to 82·4)	0·0 (0·0 to 0·0)	932·3 (806·3 to 1078·0)	643·0 (533·9 to 785·8)	230·1 (180·4 to 290·1)	59·2 (40·4 to 82·4)	0·0 (0·0 to 0·0)
Guam	2026	936·0 (809·2 to 1083·8)	646·0 (536·4 to 788·1)	229·3 (179·7 to 289·1)	60·7 (41·3 to 85·4)	0·0 (0·0 to 0·0)	936·0 (809·2 to 1083·8)	646·0 (536·4 to 788·1)	229·3 (179·7 to 289·1)	60·7 (41·3 to 85·4)	0·0 (0·0 to 0·0)
Guam	2027	940·3 (812·0 to 1088·1)	649·6 (540·2 to 791·4)	228·6 (179·3 to 288·1)	62·2 (42·2 to 87·5)	0·0 (0·0 to 0·0)	940·3 (812·0 to 1088·1)	649·6 (540·2 to 791·4)	228·6 (179·3 to 288·1)	62·2 (42·2 to 87·5)	0·0 (0·0 to 0·0)
Guam	2028	944·6 (816·3 to 1093·9)	653·0 (542·1 to 797·5)	228·0 (178·8 to 287·1)	63·7 (43·2 to 90·0)	0·0 (0·0 to 0·0)	944·6 (816·3 to 1093·9)	653·0 (542·1 to 797·5)	228·0 (178·8 to 287·1)	63·7 (43·2 to 90·0)	0·0 (0·0 to 0·0)
Guam	2029	949·4 (819·8 to 1099·7)	656·5 (542·0 to 802·2)	227·6 (178·4 to 287·1)	65·3 (44·2 to 92·3)	0·0 (0·0 to 0·0)	949·4 (819·8 to 1099·7)	656·5 (542·0 to 802·2)	227·6 (178·4 to 287·1)	65·3 (44·2 to 92·3)	0·0 (0·0 to 0·0)
Guam	2030	954·7 (823·7 to 1106·8)	660·4 (545·2 to 803·4)	227·3 (178·2 to 286·7)	67·0 (45·2 to 94·9)	0·0 (0·0 to 0·0)	954·7 (823·7 to 1106·8)	660·4 (545·2 to 803·4)	227·3 (178·2 to 286·7)	67·0 (45·2 to 94·9)	0·0 (0·0 to 0·0)
Guam	2031	960·7 (829·6 to 1114·8)	664·8 (549·4 to 810·2)	227·2 (178·2 to 286·5)	68·7 (46·3 to 98·0)	0·0 (0·0 to 0·0)	960·7 (829·6 to 1114·8)	664·8 (549·4 to 810·2)	227·2 (178·2 to 286·5)	68·7 (46·3 to 98·0)	0·0 (0·0 to 0·0)
Guam	2032	967·5 (835·7 to 1123·4)	669·8 (554·0 to 815·3)	227·2 (178·2 to 286·3)	70·5 (47·2 to 101·3)	0·0 (0·0 to 0·0)	967·5 (835·7 to 1123·4)	669·8 (554·0 to 815·3)	227·2 (178·2 to 286·3)	70·5 (47·2 to 101·3)	0·0 (0·0 to 0·0)

Guam	2033	974·6 (840·8 to 1130·5)	675·0 (558·8 to 824·7)	227·4 (178·5 to 286·4)	72·2 (48·1 to 104·5)	0·0 (0·0 to 0·0)	974·6 (840·8 to 1130·5)	675·0 (558·8 to 824·7)	227·4 (178·5 to 286·4)	72·2 (48·1 to 104·5)	0·0 (0·0 to 0·0)
Guam	2034	982·1 (846·4 to 1140·1)	680·5 (561·4 to 829·1)	227·5 (178·8 to 286·6)	74·1 (49·2 to 108·2)	0·0 (0·0 to 0·0)	982·1 (846·4 to 1140·1)	680·5 (561·4 to 829·1)	227·5 (178·8 to 286·6)	74·1 (49·2 to 108·2)	0·0 (0·0 to 0·0)
Guam	2035	990·1 (852·5 to 1149·3)	686·4 (566·3 to 838·5)	227·8 (179·2 to 286·9)	75·9 (50·4 to 111·7)	0·0 (0·0 to 0·0)	990·1 (852·5 to 1149·3)	686·4 (566·3 to 838·5)	227·8 (179·2 to 286·9)	75·9 (50·4 to 111·7)	0·0 (0·0 to 0·0)
Guam	2036	998·5 (860·7 to 1158·1)	692·5 (572·1 to 842·8)	228·2 (179·6 to 287·3)	77·8 (51·3 to 114·8)	0·0 (0·0 to 0·0)	998·5 (860·7 to 1158·1)	692·5 (572·1 to 842·8)	228·2 (179·6 to 287·3)	77·8 (51·3 to 114·8)	0·0 (0·0 to 0·0)
Guam	2037	1008·6 (868·2 to 1170·4)	699·9 (576·9 to 852·5)	228·9 (180·1 to 288·3)	79·9 (52·4 to 117·9)	0·0 (0·0 to 0·0)	1008·6 (868·2 to 1170·4)	699·9 (576·9 to 852·5)	228·9 (180·1 to 288·3)	79·9 (52·4 to 117·9)	0·0 (0·0 to 0·0)
Guam	2038	1019·0 (879·2 to 1182·2)	707·5 (583·0 to 865·5)	229·6 (180·8 to 289·2)	81·9 (53·6 to 121·2)	0·0 (0·0 to 0·0)	1019·0 (879·2 to 1182·2)	707·5 (583·0 to 865·5)	229·6 (180·8 to 289·2)	81·9 (53·6 to 121·2)	0·0 (0·0 to 0·0)
Guam	2039	1029·3 (887·2 to 1192·7)	715·1 (586·9 to 875·3)	230·3 (181·4 to 290·1)	83·9 (54·7 to 124·8)	0·0 (0·0 to 0·0)	1029·3 (887·2 to 1192·7)	715·1 (586·9 to 875·3)	230·3 (181·4 to 290·1)	83·9 (54·7 to 124·8)	0·0 (0·0 to 0·0)
Guam	2040	1039·6 (894·4 to 1205·6)	722·7 (594·7 to 884·4)	231·0 (182·1 to 291·3)	85·9 (55·9 to 128·3)	0·0 (0·0 to 0·0)	1039·6 (894·4 to 1205·6)	722·7 (594·7 to 884·4)	231·0 (182·1 to 291·3)	85·9 (55·9 to 128·3)	0·0 (0·0 to 0·0)
Guam	2041	1050·8 (903·6 to 1218·4)	730·9 (601·2 to 896·0)	231·9 (182·7 to 292·6)	88·0 (56·8 to 131·2)	0·0 (0·0 to 0·0)	1050·8 (903·6 to 1218·4)	730·9 (601·2 to 896·0)	231·9 (182·7 to 292·6)	88·0 (56·8 to 131·2)	0·0 (0·0 to 0·0)
Guam	2042	1062·7 (914·3 to 1230·5)	739·7 (607·4 to 901·3)	232·8 (183·5 to 294·0)	90·1 (58·2 to 135·3)	0·0 (0·0 to 0·0)	1062·7 (914·3 to 1230·5)	739·7 (607·4 to 901·3)	232·8 (183·5 to 294·0)	90·1 (58·2 to 135·3)	0·0 (0·0 to 0·0)
Guam	2043	1074·6 (923·7 to 1245·0)	748·5 (613·8 to 913·3)	233·8 (184·2 to 295·3)	92·3 (59·2 to 139·7)	0·0 (0·0 to 0·0)	1074·6 (923·7 to 1245·0)	748·5 (613·8 to 913·3)	233·8 (184·2 to 295·3)	92·3 (59·2 to 139·7)	0·0 (0·0 to 0·0)
Guam	2044	1086·9 (935·1 to 1258·7)	757·5 (621·0 to 922·8)	234·9 (185·0 to 296·4)	94·5 (60·5 to 143·4)	0·0 (0·0 to 0·0)	1086·9 (935·1 to 1258·7)	757·5 (621·0 to 922·8)	234·9 (185·0 to 296·4)	94·5 (60·5 to 143·4)	0·0 (0·0 to 0·0)
Guam	2045	1099·6 (947·2 to 1273·3)	766·8 (630·2 to 935·1)	236·0 (185·7 to 297·9)	96·7 (62·0 to 147·2)	0·0 (0·0 to 0·0)	1099·6 (947·2 to 1273·3)	766·8 (630·2 to 935·1)	236·0 (185·7 to 297·9)	96·7 (62·0 to 147·2)	0·0 (0·0 to 0·0)
Guam	2046	1112·3 (957·4 to 1290·5)	776·2 (635·5 to 947·2)	237·1 (186·5 to 299·4)	99·0 (63·5 to 150·1)	0·0 (0·0 to 0·0)	1112·3 (957·4 to 1290·5)	776·2 (635·5 to 947·2)	237·1 (186·5 to 299·4)	99·0 (63·5 to 150·1)	0·0 (0·0 to 0·0)

Guam	2047	1124·7 (966·1 to 1305·0)	785·2 (642·8 to 960·4)	238·1 (187·2 to 300·8)	101·3 (65·0 to 154·7)	0·0 (0·0 to 0·0)	1124·7 (966·1 to 1305·0)	785·2 (642·8 to 960·4)	238·1 (187·2 to 300·8)	101·3 (65·0 to 154·7)	0·0 (0·0 to 0·0)
Guam	2048	1137·9 (977·4 to 1321·7)	794·8 (651·5 to 974·5)	239·3 (188·0 to 302·3)	103·8 (66·1 to 159·3)	0·0 (0·0 to 0·0)	1137·9 (977·4 to 1321·7)	794·8 (651·5 to 974·5)	239·3 (188·0 to 302·3)	103·8 (66·1 to 159·3)	0·0 (0·0 to 0·0)
Guam	2049	1151·0 (988·5 to 1336·2)	804·4 (657·7 to 988·4)	240·4 (188·8 to 303·4)	106·2 (67·7 to 163·4)	0·0 (0·0 to 0·0)	1151·0 (988·5 to 1336·2)	804·4 (657·7 to 988·4)	240·4 (188·8 to 303·4)	106·2 (67·7 to 163·4)	0·0 (0·0 to 0·0)
Guam	2050	1164·3 (1000·4 to 1352·0)	814·0 (666·8 to 997·8)	241·5 (189·5 to 304·4)	108·7 (69·2 to 168·8)	0·0 (0·0 to 0·0)	1164·3 (1000·4 to 1352·0)	814·0 (666·8 to 997·8)	241·5 (189·5 to 304·4)	108·7 (69·2 to 168·8)	0·0 (0·0 to 0·0)
Guyana*	1995	131·6 (117·6 to 145·7)	74·1 (63·9 to 84·8)	44·9 (36·8 to 54·4)	5·4 (3·6 to 7·6)	7·1 (7·1 to 7·1)	264·1 (236·1 to 292·4)	148·7 (128·4 to 170·3)	90·2 (73·8 to 109·2)	10·9 (7·3 to 15·3)	14·3 (14·3 to 14·3)
Guyana*	1996	137·1 (123·9 to 150·9)	78·2 (68·2 to 89·5)	46·4 (38·3 to 55·1)	5·5 (3·7 to 7·9)	7·0 (7·0 to 7·0)	275·1 (248·8 to 302·9)	156·9 (136·9 to 179·6)	93·1 (76·8 to 110·6)	11·1 (7·5 to 15·8)	14·0 (14·0 to 14·0)
Guyana*	1997	144·0 (130·8 to 157·1)	81·5 (71·7 to 92·2)	47·8 (39·5 to 56·5)	5·6 (3·9 to 7·9)	9·1 (9·1 to 9·1)	289·1 (262·5 to 315·4)	163·6 (143·9 to 185·1)	95·9 (79·3 to 113·5)	11·3 (7·7 to 15·8)	18·3 (18·3 to 18·3)
Guyana*	1998	137·0 (124·7 to 149·6)	77·4 (68·3 to 87·8)	47·5 (39·7 to 55·9)	5·4 (3·7 to 7·6)	6·7 (6·7 to 6·7)	274·9 (250·3 to 300·3)	155·3 (137·1 to 176·2)	95·3 (79·7 to 112·2)	10·9 (7·5 to 15·3)	13·4 (13·4 to 13·4)
Guyana*	1999	130·2 (118·0 to 142·6)	72·3 (63·6 to 81·6)	47·0 (39·5 to 55·3)	5·1 (3·5 to 7·1)	5·8 (5·8 to 5·8)	261·3 (236·9 to 286·3)	145·1 (127·7 to 163·9)	94·3 (79·2 to 111·0)	10·2 (7·1 to 14·3)	11·7 (11·7 to 11·7)
Guyana*	2000	131·2 (118·8 to 144·1)	74·9 (65·9 to 84·9)	49·0 (41·0 to 57·9)	4·7 (3·3 to 6·7)	2·6 (2·6 to 2·6)	263·4 (238·4 to 289·3)	150·3 (132·3 to 170·5)	98·4 (82·2 to 116·3)	9·5 (6·6 to 13·4)	5·2 (5·2 to 5·2)
Guyana*	2001	133·8 (121·8 to 146·5)	73·4 (64·3 to 83·5)	50·0 (42·0 to 59·0)	4·6 (3·2 to 6·4)	5·8 (5·8 to 5·8)	268·5 (244·5 to 294·0)	147·4 (129·1 to 167·6)	100·4 (84·3 to 118·5)	9·2 (6·5 to 12·8)	11·6 (11·6 to 11·6)
Guyana*	2002	129·9 (118·0 to 142·6)	70·6 (62·1 to 80·4)	50·3 (42·2 to 59·1)	4·5 (3·1 to 6·3)	4·5 (4·5 to 4·5)	260·6 (236·8 to 286·3)	141·8 (124·7 to 161·5)	100·9 (84·7 to 118·6)	8·9 (6·2 to 12·7)	9·0 (9·0 to 9·0)
Guyana*	2003	145·3 (133·4 to 157·9)	69·6 (61·5 to 78·3)	51·0 (43·3 to 59·3)	4·4 (3·1 to 6·2)	20·3 (20·3 to 20·3)	291·6 (267·7 to 317·0)	139·6 (123·4 to 157·2)	102·4 (86·9 to 119·1)	8·9 (6·1 to 12·5)	40·7 (40·7 to 40·7)
Guyana*	2004	168·5 (156·8 to 180·4)	69·3 (61·6 to 77·6)	53·1 (45·2 to 61·6)	4·6 (3·2 to 6·3)	41·5 (41·5 to 41·5)	338·2 (314·8 to 362·1)	139·1 (123·6 to 155·8)	106·5 (90·7 to 123·7)	9·1 (6·4 to 12·7)	83·4 (83·4 to 83·4)

Guyana*	2005	172·7 (161·3 to 185·8)	74·8 (67·4 to 83·5)	57·8 (49·8 to 67·3)	5·0 (3·4 to 6·9)	35·1 (35·1 to 35·1)	346·7 (323·7 to 372·9)	150·2 (135·4 to 167·6)	116·0 (99·9 to 135·2)	10·0 (6·9 to 13·8)	70·5 (70·5 to 70·5)
Guyana*	2006	194·9 (183·9 to 208·5)	78·3 (71·4 to 86·4)	63·2 (54·9 to 73·3)	5·5 (3·8 to 7·6)	47·9 (47·9 to 47·9)	391·2 (369·0 to 418·6)	157·2 (143·3 to 173·4)	126·8 (110·2 to 147·2)	11·0 (7·6 to 15·3)	96·2 (96·2 to 96·2)
Guyana*	2007	191·0 (180·2 to 203·3)	78·8 (72·8 to 85·3)	67·6 (59·1 to 77·9)	5·9 (4·1 to 8·3)	38·7 (38·7 to 38·7)	383·4 (361·6 to 408·0)	158·1 (146·1 to 171·1)	135·7 (118·6 to 156·4)	11·9 (8·2 to 16·7)	77·8 (77·8 to 77·8)
Guyana*	2008	204·1 (193·6 to 214·9)	81·7 (76·6 to 86·9)	71·5 (62·9 to 82·1)	6·3 (4·3 to 8·7)	44·6 (44·6 to 44·6)	409·6 (388·6 to 431·3)	164·0 (153·7 to 174·4)	143·6 (126·2 to 164·7)	12·6 (8·7 to 17·6)	89·4 (89·4 to 89·4)
Guyana*	2009	219·0 (207·8 to 229·2)	91·7 (87·1 to 96·3)	76·1 (66·8 to 86·1)	6·7 (4·7 to 9·4)	44·4 (44·4 to 44·4)	439·5 (417·2 to 460·0)	184·0 (174·8 to 193·2)	152·8 (134·2 to 172·9)	13·4 (9·4 to 18·8)	89·2 (89·2 to 89·2)
Guyana*	2010	222·8 (212·6 to 233·8)	95·9 (91·7 to 100·2)	78·6 (69·1 to 88·9)	6·9 (4·9 to 9·6)	41·5 (41·5 to 41·5)	447·2 (426·7 to 469·2)	192·4 (184·0 to 201·1)	157·7 (138·6 to 178·4)	13·9 (9·8 to 19·3)	83·2 (83·2 to 83·2)
Guyana*	2011	226·2 (215·6 to 238·2)	104·2 (99·9 to 108·4)	81·2 (71·0 to 92·1)	7·2 (5·1 to 10·1)	33·6 (33·6 to 33·6)	454·0 (432·7 to 478·1)	209·1 (200·6 to 217·5)	163·0 (142·6 to 184·8)	14·4 (10·2 to 20·3)	67·5 (67·5 to 67·5)
Guyana*	2012	221·9 (210·8 to 233·8)	108·7 (104·2 to 112·8)	81·4 (71·5 to 92·3)	7·1 (5·0 to 9·8)	24·7 (24·7 to 24·7)	445·4 (423·2 to 469·2)	218·2 (209·2 to 226·4)	163·4 (143·6 to 185·3)	14·2 (10·0 to 19·7)	49·6 (49·6 to 49·6)
Guyana*	2013	225·3 (214·2 to 236·8)	116·4 (111·9 to 120·8)	82·8 (72·5 to 93·8)	7·2 (5·1 to 10·1)	18·9 (18·9 to 18·9)	452·2 (429·8 to 475·2)	233·6 (224·5 to 242·5)	166·2 (145·5 to 188·2)	14·5 (10·1 to 20·4)	37·9 (37·9 to 37·9)
Guyana*	2014	241·2 (229·1 to 253·9)	133·1 (128·2 to 137·6)	85·1 (74·6 to 96·4)	7·5 (5·3 to 10·5)	15·5 (15·5 to 15·5)	484·1 (459·8 to 509·7)	267·1 (257·3 to 276·2)	170·8 (149·7 to 193·5)	15·1 (10·6 to 21·1)	31·1 (31·1 to 31·1)
Guyana*	2015	242·1 (230·6 to 254·1)	140·3 (135·7 to 144·7)	83·0 (73·0 to 94·4)	7·2 (5·1 to 10·0)	11·5 (11·5 to 11·5)	485·9 (462·9 to 510·0)	281·6 (272·5 to 290·5)	166·6 (146·6 to 189·6)	14·5 (10·3 to 20·2)	23·2 (23·2 to 23·2)
Guyana*	2016	264·4 (252·7 to 277·6)	162·5 (156·8 to 167·9)	84·1 (74·0 to 95·8)	7·3 (5·2 to 10·1)	10·6 (10·6 to 10·6)	530·8 (507·2 to 557·2)	326·1 (314·7 to 337·0)	168·8 (148·6 to 192·4)	14·6 (10·4 to 20·2)	21·3 (21·3 to 21·3)
Guyana*	2017	284·0 (268·8 to 300·6)	179·0 (169·5 to 188·2)	86·8 (75·5 to 99·5)	7·6 (5·3 to 10·5)	10·6 (10·6 to 10·6)	570·0 (539·5 to 603·3)	359·3 (340·2 to 377·8)	174·2 (151·5 to 199·7)	15·2 (10·7 to 21·1)	21·4 (21·4 to 21·4)
Guyana*	2018	304·5 (283·9 to 326·7)	195·0 (180·9 to 210·2)	94·7 (81·5 to 110·4)	8·6 (6·0 to 12·2)	6·2 (6·2 to 6·2)	611·1 (569·9 to 655·8)	391·5 (363·0 to 422·0)	190·0 (163·6 to 221·6)	17·3 (12·1 to 24·4)	12·4 (12·4 to 12·4)

Guyana*	2019	322·6 (300·9 to 345·6)	207·9 (192·5 to 223·7)	99·8 (85·9 to 116·5)	9·3 (6·5 to 13·2)	5·7 (5·6 to 5·8)	647·5 (603·9 to 693·7)	417·2 (386·4 to 449·1)	200·2 (172·3 to 233·8)	18·6 (13·1 to 26·5)	11·5 (11·3 to 11·7)
Guyana*	2020	351·2 (326·7 to 378·0)	213·5 (197·7 to 230·2)	122·8 (105·7 to 143·3)	11·7 (8·2 to 16·7)	3·2 (2·8 to 3·6)	705·0 (655·7 to 758·8)	428·5 (396·9 to 462·0)	246·4 (212·1 to 287·5)	23·5 (16·5 to 33·4)	6·5 (5·6 to 7·3)
Guyana*	2021	340·4 (316·6 to 366·4)	206·8 (191·5 to 222·9)	118·8 (102·3 to 138·7)	11·7 (8·2 to 16·7)	3·2 (2·8 to 3·6)	683·3 (635·6 to 735·5)	415·0 (384·3 to 447·4)	238·5 (205·3 to 278·4)	23·4 (16·4 to 33·5)	6·4 (5·6 to 7·2)
Guyana*	2022	347·9 (323·7 to 375·0)	213·8 (197·6 to 231·1)	119·6 (103·0 to 139·4)	12·1 (8·4 to 17·2)	2·5 (2·2 to 2·9)	698·4 (649·7 to 752·6)	429·1 (396·7 to 463·8)	240·0 (206·7 to 279·8)	24·2 (16·9 to 34·5)	5·1 (4·4 to 5·8)
Guyana*	2023	352·3 (324·2 to 381·7)	216·9 (197·2 to 237·3)	120·3 (103·6 to 140·3)	12·5 (8·6 to 17·7)	2·6 (2·3 to 3·0)	707·1 (650·7 to 766·1)	435·4 (395·8 to 476·3)	241·4 (208·0 to 281·5)	25·0 (17·4 to 35·5)	5·3 (4·6 to 6·0)
Guyana*	2024	359·1 (328·1 to 391·5)	222·5 (197·9 to 248·9)	120·9 (104·3 to 141·1)	12·9 (8·9 to 18·3)	2·8 (2·4 to 3·2)	720·7 (658·6 to 785·8)	446·5 (397·2 to 499·6)	242·7 (209·3 to 283·3)	25·9 (17·9 to 36·7)	5·5 (4·8 to 6·3)
Guyana*	2025	364·9 (329·9 to 403·0)	227·1 (198·2 to 260·7)	121·5 (104·8 to 141·8)	13·3 (9·2 to 18·9)	2·9 (2·5 to 3·3)	732·4 (662·1 to 808·9)	455·9 (397·8 to 523·3)	244·0 (210·3 to 284·5)	26·8 (18·5 to 37·9)	5·8 (5·1 to 6·6)
Guyana*	2026	370·8 (332·4 to 412·7)	231·8 (200·1 to 270·3)	122·2 (105·3 to 142·4)	13·8 (9·5 to 19·3)	3·0 (2·7 to 3·4)	744·3 (667·1 to 828·3)	465·3 (401·6 to 542·4)	245·2 (211·3 to 285·7)	27·7 (19·0 to 38·8)	6·1 (5·3 to 6·9)
Guyana*	2027	375·5 (333·2 to 422·5)	235·3 (199·0 to 281·0)	122·8 (105·8 to 143·1)	14·2 (9·8 to 20·0)	3·2 (2·8 to 3·6)	753·7 (668·7 to 848·1)	472·3 (399·5 to 563·9)	246·4 (212·3 to 287·2)	28·6 (19·6 to 40·2)	6·3 (5·6 to 7·2)
Guyana*	2028	380·2 (335·8 to 429·7)	238·9 (201·1 to 285·6)	123·4 (106·3 to 143·8)	14·7 (10·1 to 20·8)	3·3 (2·9 to 3·8)	763·2 (674·0 to 862·4)	479·5 (403·6 to 573·2)	247·6 (213·4 to 288·5)	29·5 (20·3 to 41·7)	6·6 (5·8 to 7·6)
Guyana*	2029	384·9 (337·8 to 440·2)	242·5 (200·5 to 293·8)	124·0 (106·8 to 144·5)	15·1 (10·4 to 21·3)	3·4 (3·0 to 3·9)	772·6 (678·1 to 883·6)	486·7 (402·4 to 589·8)	248·8 (214·3 to 290·1)	30·2 (20·8 to 42·8)	6·9 (6·1 to 7·9)
Guyana*	2030	390·5 (340·5 to 448·7)	246·9 (203·3 to 300·7)	124·5 (107·3 to 145·2)	15·5 (10·6 to 21·9)	3·6 (3·2 to 4·1)	783·7 (683·3 to 900·5)	495·5 (408·1 to 603·6)	250·0 (215·3 to 291·5)	31·0 (21·3 to 43·9)	7·2 (6·3 to 8·2)
Guyana*	2031	396·1 (342·2 to 456·6)	251·3 (204·0 to 311·0)	125·2 (107·9 to 145·8)	15·9 (10·9 to 22·5)	3·7 (3·3 to 4·2)	795·0 (686·8 to 916·4)	504·4 (409·4 to 624·3)	251·3 (216·6 to 292·7)	31·8 (21·9 to 45·2)	7·5 (6·6 to 8·5)
Guyana*	2032	403·2 (345·4 to 467·3)	257·1 (204·6 to 321·1)	125·9 (108·5 to 146·7)	16·3 (11·2 to 23·1)	3·9 (3·4 to 4·4)	809·2 (693·2 to 937·9)	516·0 (410·7 to 644·5)	252·7 (217·8 to 294·4)	32·7 (22·5 to 46·3)	7·7 (6·9 to 8·8)

Guyana*	2033	410·3 (349·6 to 480·2)	262·9 (206·2 to 332·2)	126·6 (109·1 to 147·5)	16·7 (11·5 to 23·6)	4·0 (3·5 to 4·6)	823·6 (701·6 to 963·9)	527·8 (413·8 to 666·7)	254·2 (219·0 to 296·1)	33·6 (23·0 to 47·3)	8·0 (7·1 to 9·1)
Guyana*	2034	417·5 (353·8 to 489·4)	268·7 (209·0 to 339·1)	127·4 (109·8 to 148·4)	17·2 (11·8 to 24·2)	4·2 (3·7 to 4·7)	838·0 (710·1 to 982·3)	539·4 (419·5 to 680·5)	255·8 (220·3 to 297·9)	34·5 (23·8 to 48·6)	8·3 (7·4 to 9·5)
Guyana*	2035	425·0 (357·4 to 500·5)	274·7 (211·5 to 345·9)	128·2 (110·4 to 149·3)	17·7 (12·1 to 24·9)	4·3 (3·8 to 4·9)	852·9 (717·3 to 1004·5)	551·5 (424·4 to 694·2)	257·3 (221·6 to 299·7)	35·5 (24·3 to 49·9)	8·7 (7·7 to 9·9)
Guyana*	2036	432·1 (361·0 to 512·5)	280·4 (213·5 to 357·5)	129·0 (111·1 to 150·3)	18·2 (12·5 to 25·8)	4·5 (3·9 to 5·1)	867·3 (724·6 to 1028·7)	562·8 (428·6 to 717·5)	259·0 (222·9 to 301·7)	36·5 (25·0 to 51·8)	9·0 (7·9 to 10·3)
Guyana*	2037	439·8 (366·1 to 524·1)	286·5 (216·4 to 368·0)	129·9 (111·8 to 151·4)	18·7 (12·9 to 26·3)	4·6 (4·1 to 5·3)	882·8 (734·8 to 1052·0)	575·1 (434·4 to 738·6)	260·8 (224·4 to 303·9)	37·6 (25·8 to 52·8)	9·3 (8·2 to 10·6)
Guyana*	2038	447·8 (369·7 to 537·2)	292·8 (217·9 to 381·2)	130·9 (112·6 to 152·5)	19·3 (13·2 to 27·0)	4·8 (4·2 to 5·5)	898·7 (742·0 to 1078·3)	587·7 (437·3 to 765·0)	262·7 (225·9 to 306·1)	38·7 (26·6 to 54·3)	9·6 (8·5 to 11·0)
Guyana*	2039	456·1 (372·7 to 548·9)	299·5 (219·8 to 391·7)	131·8 (113·3 to 153·6)	19·8 (13·7 to 27·9)	5·0 (4·4 to 5·7)	915·4 (748·1 to 1101·7)	601·2 (441·1 to 786·3)	264·5 (227·4 to 308·3)	39·8 (27·5 to 56·0)	10·0 (8·8 to 11·5)
Guyana*	2040	464·2 (379·6 to 565·9)	305·9 (222·8 to 406·3)	132·7 (114·0 to 154·7)	20·4 (14·1 to 28·8)	5·2 (4·6 to 6·0)	931·7 (761·9 to 1135·8)	614·1 (447·3 to 815·5)	266·3 (228·9 to 310·5)	40·9 (28·3 to 57·8)	10·4 (9·2 to 12·0)
Guyana*	2041	471·1 (384·7 to 576·3)	311·2 (228·3 to 414·9)	133·5 (114·7 to 155·7)	21·0 (14·5 to 29·6)	5·4 (4·7 to 6·2)	945·5 (772·1 to 1156·7)	624·6 (458·2 to 832·7)	268·0 (230·2 to 312·5)	42·1 (29·2 to 59·5)	10·8 (9·5 to 12·5)
Guyana*	2042	478·4 (387·4 to 586·9)	316·9 (232·4 to 425·4)	134·3 (115·3 to 156·6)	21·6 (15·0 to 30·5)	5·6 (4·9 to 6·6)	960·2 (777·6 to 1178·0)	636·1 (466·4 to 853·7)	269·5 (231·5 to 314·4)	43·3 (30·1 to 61·2)	11·3 (9·8 to 13·2)
Guyana*	2043	486·6 (392·0 to 598·5)	323·3 (236·1 to 430·2)	135·2 (116·1 to 157·8)	22·2 (15·5 to 31·6)	5·9 (5·1 to 6·8)	976·7 (786·9 to 1201·3)	649·0 (474·0 to 863·6)	271·4 (233·0 to 316·6)	44·6 (31·0 to 63·5)	11·8 (10·2 to 13·6)
Guyana*	2044	495·4 (396·9 to 610·2)	330·3 (235·4 to 441·5)	136·1 (116·8 to 158·8)	22·9 (15·9 to 32·7)	6·1 (5·3 to 7·2)	994·3 (796·7 to 1224·8)	663·0 (472·5 to 886·1)	273·2 (234·5 to 318·8)	45·9 (31·9 to 65·6)	12·3 (10·6 to 14·5)
Guyana*	2045	502·9 (402·1 to 624·2)	336·0 (237·3 to 456·5)	137·0 (117·5 to 159·8)	23·5 (16·3 to 33·8)	6·4 (5·5 to 7·5)	1009·3 (807·1 to 1252·8)	674·4 (476·2 to 916·3)	274·9 (235·9 to 320·8)	47·2 (32·7 to 67·8)	12·8 (11·0 to 15·1)
Guyana*	2046	510·9 (404·1 to 642·2)	342·2 (242·1 to 473·4)	137·8 (118·2 to 160·9)	24·2 (16·8 to 34·7)	6·7 (5·7 to 7·8)	1025·5 (811·0 to 1289·0)	686·9 (485·9 to 950·1)	276·6 (237·3 to 322·9)	48·6 (33·6 to 69·7)	13·4 (11·4 to 15·7)

Guyana*	2047	519·2 (407·0 to 655·5)	348·6 (244·3 to 483·2)	138·7 (118·9 to 161·9)	24·9 (17·2 to 35·8)	6·9 (5·9 to 8·3)	1042·1 (816·9 to 1315·7)	699·8 (490·4 to 969·9)	278·4 (238·7 to 324·9)	50·0 (34·6 to 71·9)	13·9 (11·8 to 16·6)
Guyana*	2048	526·5 (411·0 to 667·0)	354·1 (245·9 to 492·1)	139·5 (119·6 to 162·8)	25·6 (17·6 to 36·8)	7·2 (6·1 to 8·6)	1056·7 (824·9 to 1338·8)	710·6 (493·6 to 987·8)	280·1 (240·0 to 326·8)	51·5 (35·3 to 73·8)	14·5 (12·3 to 17·2)
Guyana*	2049	533·9 (416·4 to 681·6)	359·5 (251·8 to 506·3)	140·4 (120·3 to 163·9)	26·4 (18·1 to 37·7)	7·5 (6·4 to 9·0)	1071·6 (835·8 to 1368·1)	721·6 (505·4 to 1016·1)	281·8 (241·4 to 328·9)	53·0 (36·3 to 75·8)	15·1 (12·9 to 18·1)
Guyana*	2050	541·7 (421·8 to 692·5)	365·3 (251·9 to 512·4)	141·3 (121·0 to 165·0)	27·2 (18·5 to 38·9)	7·9 (6·6 to 9·4)	1087·2 (846·7 to 1389·9)	733·1 (505·7 to 1028·6)	283·7 (242·9 to 331·1)	54·6 (37·2 to 78·1)	15·8 (13·3 to 18·9)
Honduras*	1995	98·9 (88·1 to 111·1)	40·3 (33·9 to 47·7)	47·7 (39·5 to 57·5)	6·3 (4·4 to 8·9)	4·5 (4·5 to 4·5)	226·9 (202·1 to 255·0)	92·6 (77·8 to 109·6)	109·5 (90·6 to 132·0)	14·6 (10·1 to 20·4)	10·3 (10·3 to 10·3)
Honduras*	1996	103·9 (93·6 to 115·5)	40·0 (33·6 to 47·7)	48·3 (40·8 to 57·4)	6·3 (4·4 to 8·9)	9·3 (9·3 to 9·3)	238·5 (214·9 to 265·0)	91·7 (77·2 to 109·5)	110·9 (93·8 to 131·8)	14·6 (10·1 to 20·5)	21·2 (21·2 to 21·2)
Honduras*	1997	108·3 (98·1 to 118·9)	41·9 (35·2 to 49·5)	49·9 (42·8 to 58·1)	6·5 (4·4 to 9·1)	9·9 (9·9 to 9·9)	248·6 (225·2 to 273·1)	96·3 (80·9 to 113·5)	114·7 (98·3 to 133·3)	14·8 (10·2 to 20·9)	22·8 (22·8 to 22·8)
Honduras*	1998	107·9 (97·9 to 117·5)	44·2 (37·6 to 51·7)	52·0 (45·7 to 59·0)	6·6 (4·6 to 9·3)	5·1 (5·1 to 5·1)	247·6 (224·8 to 269·8)	101·5 (86·4 to 118·7)	119·3 (105·0 to 135·3)	15·1 (10·5 to 21·5)	11·7 (11·7 to 11·7)
Honduras*	1999	121·2 (111·2 to 131·1)	47·7 (40·2 to 55·9)	55·7 (50·6 to 61·9)	6·2 (4·3 to 8·8)	11·6 (11·6 to 11·6)	278·2 (255·3 to 301·0)	109·4 (92·4 to 128·3)	127·9 (116·1 to 142·1)	14·2 (9·9 to 20·2)	26·7 (26·7 to 26·7)
Honduras*	2000	121·2 (112·1 to 130·5)	50·3 (42·8 to 58·5)	58·1 (54·0 to 62·7)	4·9 (3·4 to 7·0)	7·9 (7·9 to 7·9)	278·3 (257·3 to 299·5)	115·5 (98·2 to 134·2)	133·4 (124·1 to 144·0)	11·2 (7·8 to 16·0)	18·2 (18·2 to 18·2)
Honduras*	2001	125·7 (116·9 to 134·5)	53·1 (45·5 to 61·2)	61·9 (58·4 to 65·8)	4·7 (3·4 to 6·8)	5·9 (5·9 to 5·9)	288·5 (268·4 to 308·8)	122·0 (104·3 to 140·6)	142·2 (134·0 to 151·0)	10·9 (7·7 to 15·6)	13·5 (13·5 to 13·5)
Honduras*	2002	130·5 (121·4 to 139·7)	55·5 (47·6 to 64·0)	65·7 (62·4 to 69·1)	4·9 (3·4 to 6·8)	4·5 (4·5 to 4·5)	299·6 (278·7 to 320·7)	127·5 (109·2 to 146·9)	150·7 (143·2 to 158·6)	11·1 (7·8 to 15·5)	10·3 (10·3 to 10·3)
Honduras*	2003	138·7 (129·6 to 148·0)	57·0 (48·8 to 65·4)	69·3 (66·0 to 72·9)	5·6 (3·9 to 7·8)	6·8 (6·8 to 6·8)	318·5 (297·5 to 339·7)	130·8 (111·9 to 150·2)	159·2 (151·6 to 167·4)	12·8 (9·0 to 17·9)	15·7 (15·7 to 15·7)
Honduras*	2004	150·1 (140·9 to 159·5)	59·3 (51·1 to 68·0)	74·5 (71·1 to 78·3)	6·0 (4·1 to 8·3)	10·2 (10·2 to 10·2)	344·6 (323·5 to 366·1)	136·2 (117·4 to 156·0)	171·1 (163·2 to 179·8)	13·7 (9·5 to 19·1)	23·5 (23·5 to 23·5)

Honduras*	2005	161·5 (152·6 to 170·9)	63·2 (55·2 to 72·3)	81·2 (77·7 to 85·1)	5·9 (4·1 to 8·3)	11·3 (11·3 to 11·3)	370·8 (350·2 to 392·2)	145·0 (126·8 to 166·0)	186·4 (178·3 to 195·4)	13·6 (9·5 to 19·0)	25·9 (25·9 to 25·9)
Honduras*	2006	175·1 (165·5 to 185·4)	71·6 (63·0 to 81·2)	90·3 (86·3 to 94·2)	6·1 (4·3 to 8·5)	7·2 (7·2 to 7·2)	402·1 (379·9 to 425·7)	164·3 (144·5 to 186·3)	207·3 (198·1 to 216·3)	14·0 (9·8 to 19·6)	16·5 (16·5 to 16·5)
Honduras*	2007	187·9 (177·5 to 198·4)	77·3 (68·2 to 87·4)	96·8 (92·8 to 100·9)	6·2 (4·3 to 8·5)	7·7 (7·7 to 7·7)	431·4 (407·4 to 455·5)	177·4 (156·6 to 200·6)	222·2 (213·0 to 231·5)	14·2 (9·8 to 19·4)	17·7 (17·7 to 17·7)
Honduras*	2008	199·5 (187·6 to 211·4)	84·2 (73·9 to 94·8)	101·3 (97·3 to 105·3)	6·3 (4·3 to 8·7)	7·7 (7·7 to 7·7)	457·9 (430·7 to 485·4)	193·3 (169·7 to 217·7)	232·5 (223·3 to 241·7)	14·5 (9·9 to 20·0)	17·6 (17·6 to 17·6)
Honduras*	2009	199·9 (187·5 to 211·8)	88·4 (77·5 to 99·0)	98·8 (94·5 to 103·1)	6·5 (4·5 to 9·2)	6·2 (6·2 to 6·2)	458·8 (430·3 to 486·3)	202·8 (177·9 to 227·3)	226·9 (216·9 to 236·8)	15·0 (10·4 to 21·2)	14·1 (14·1 to 14·1)
Honduras*	2010	194·5 (182·9 to 206·1)	85·8 (75·9 to 96·8)	96·3 (91·5 to 101·4)	7·4 (5·1 to 10·4)	5·0 (5·0 to 5·0)	446·6 (419·9 to 473·2)	197·0 (174·3 to 222·2)	221·0 (210·1 to 232·7)	17·0 (11·7 to 23·8)	11·6 (11·6 to 11·6)
Honduras*	2011	197·2 (186·7 to 208·9)	85·3 (75·9 to 96·2)	97·3 (93·1 to 101·6)	7·9 (5·5 to 11·2)	6·6 (6·6 to 6·6)	452·8 (428·6 to 479·5)	195·8 (174·3 to 220·9)	223·5 (213·7 to 233·3)	18·2 (12·6 to 25·7)	15·2 (15·2 to 15·2)
Honduras*	2012	200·5 (190·1 to 211·2)	83·3 (73·9 to 93·2)	99·6 (95·7 to 103·5)	8·4 (5·8 to 11·9)	9·1 (9·1 to 9·1)	460·2 (436·4 to 484·9)	191·3 (169·7 to 213·9)	228·7 (219·8 to 237·5)	19·3 (13·4 to 27·4)	20·8 (20·8 to 20·8)
Honduras*	2013	202·9 (191·9 to 214·1)	85·8 (75·9 to 96·9)	100·8 (96·9 to 105·0)	9·0 (6·3 to 12·7)	7·3 (7·3 to 7·3)	465·8 (440·6 to 491·6)	196·9 (174·2 to 222·4)	231·3 (222·4 to 241·1)	20·7 (14·4 to 29·1)	16·8 (16·8 to 16·8)
Honduras*	2014	191·7 (181·2 to 202·2)	79·8 (70·4 to 90·7)	96·6 (92·8 to 100·6)	9·2 (6·4 to 12·8)	6·0 (6·0 to 6·0)	440·1 (415·9 to 464·3)	183·3 (161·5 to 208·2)	221·7 (213·0 to 231·0)	21·2 (14·7 to 29·5)	13·8 (13·8 to 13·8)
Honduras*	2015	183·9 (173·8 to 194·4)	75·1 (66·1 to 84·9)	93·7 (89·8 to 97·4)	9·3 (6·6 to 13·1)	5·7 (5·7 to 5·7)	422·1 (399·1 to 446·3)	172·5 (151·8 to 195·0)	215·2 (206·2 to 223·6)	21·4 (15·1 to 30·0)	13·0 (13·0 to 13·0)
Honduras*	2016	186·4 (175·0 to 197·5)	77·1 (67·3 to 87·3)	94·3 (90·2 to 98·3)	10·0 (6·9 to 14·1)	4·9 (4·9 to 4·9)	427·9 (401·7 to 453·3)	177·1 (154·5 to 200·4)	216·5 (207·2 to 225·7)	23·0 (15·9 to 32·4)	11·3 (11·3 to 11·3)
Honduras*	2017	181·6 (169·9 to 194·5)	76·3 (66·4 to 87·8)	92·4 (87·0 to 97·5)	10·4 (7·2 to 14·5)	2·5 (2·5 to 2·5)	417·0 (390·0 to 446·4)	175·2 (152·4 to 201·6)	212·1 (199·7 to 223·8)	23·9 (16·6 to 33·3)	5·7 (5·7 to 5·7)
Honduras*	2018	186·3 (172·0 to 201·3)	76·5 (65·8 to 88·6)	92·6 (84·8 to 100·7)	10·4 (7·2 to 14·5)	6·7 (6·7 to 6·7)	427·6 (394·8 to 462·2)	175·6 (151·1 to 203·5)	212·7 (194·6 to 231·1)	24·0 (16·6 to 33·3)	15·4 (15·4 to 15·4)

Honduras*	2019	187·0 (172·5 to 202·7)	76·2 (65·2 to 88·7)	93·1 (85·2 to 101·3)	10·8 (7·5 to 15·0)	6·9 (6·9 to 6·9)	429·3 (396·1 to 465·4)	175·0 (149·7 to 203·6)	213·8 (195·6 to 232·4)	24·7 (17·1 to 34·5)	15·8 (15·8 to 15·9)
Honduras*	2020	201·4 (187·6 to 216·5)	94·8 (84·0 to 107·0)	86·4 (79·1 to 94·0)	10·2 (7·1 to 14·2)	10·0 (9·8 to 10·3)	462·4 (430·6 to 496·9)	217·6 (192·8 to 245·7)	198·4 (181·5 to 215·7)	23·5 (16·4 to 32·7)	22·9 (22·6 to 23·7)
Honduras*	2021	181·8 (167·5 to 197·6)	77·5 (66·1 to 90·4)	84·9 (77·7 to 92·3)	10·3 (7·2 to 14·3)	9·2 (9·1 to 9·5)	417·4 (384·5 to 453·6)	177·8 (151·9 to 207·5)	195·0 (178·4 to 211·9)	23·6 (16·4 to 32·9)	21·1 (20·8 to 21·9)
Honduras*	2022	175·3 (160·9 to 191·0)	71·7 (61·0 to 84·4)	86·0 (78·7 to 93·5)	10·6 (7·4 to 14·9)	7·0 (6·6 to 7·4)	402·4 (369·4 to 438·4)	164·5 (139·9 to 193·8)	197·5 (180·7 to 214·6)	24·4 (17·0 to 34·1)	16·0 (15·1 to 17·0)
Honduras*	2023	178·3 (163·5 to 194·3)	73·1 (61·9 to 86·6)	87·2 (79·7 to 94·8)	11·0 (7·7 to 15·4)	7·0 (6·6 to 7·5)	409·3 (375·3 to 446·0)	167·9 (142·0 to 198·8)	200·1 (183·0 to 217·5)	25·2 (17·6 to 35·4)	16·0 (15·1 to 17·2)
Honduras*	2024	181·4 (166·3 to 198·0)	74·7 (63·0 to 88·2)	88·2 (80·7 to 95·9)	11·4 (7·9 to 16·0)	7·0 (6·6 to 7·6)	416·4 (381·8 to 454·4)	171·5 (144·6 to 202·6)	202·6 (185·3 to 220·2)	26·2 (18·2 to 36·7)	16·1 (15·1 to 17·3)
Honduras*	2025	184·5 (169·0 to 201·7)	76·3 (64·1 to 90·8)	89·3 (81·7 to 97·1)	11·8 (8·2 to 16·6)	7·0 (6·6 to 7·6)	423·6 (388·0 to 463·0)	175·3 (147·2 to 208·4)	205·0 (187·5 to 222·9)	27·1 (18·9 to 38·1)	16·2 (15·1 to 17·5)
Honduras*	2026	187·7 (171·0 to 206·0)	78·0 (64·6 to 93·2)	90·3 (82·6 to 98·3)	12·2 (8·5 to 17·1)	7·1 (6·6 to 7·7)	430·8 (392·6 to 473·0)	179·1 (148·2 to 214·0)	207·4 (189·7 to 225·6)	28·1 (19·6 to 39·2)	16·2 (15·0 to 17·6)
Honduras*	2027	190·9 (173·8 to 210·4)	79·7 (66·2 to 95·5)	91·4 (83·6 to 99·5)	12·7 (8·8 to 17·6)	7·1 (6·5 to 7·7)	438·3 (399·0 to 482·9)	183·1 (151·9 to 219·3)	209·8 (191·9 to 228·3)	29·1 (20·3 to 40·5)	16·3 (15·0 to 17·7)
Honduras*	2028	194·2 (176·5 to 214·7)	81·4 (67·5 to 98·9)	92·5 (84·6 to 100·6)	13·2 (9·1 to 18·2)	7·1 (6·5 to 7·8)	445·7 (405·3 to 492·8)	187·0 (154·9 to 227·1)	212·3 (194·1 to 230·9)	30·2 (21·0 to 41·8)	16·3 (14·9 to 18·0)
Honduras*	2029	197·4 (178·9 to 218·5)	83·2 (68·0 to 101·6)	93·5 (85·4 to 101·7)	13·6 (9·5 to 19·0)	7·1 (6·5 to 7·9)	453·2 (410·7 to 501·6)	190·9 (156·1 to 233·1)	214·6 (196·1 to 233·5)	31·3 (21·8 to 43·6)	16·4 (14·9 to 18·1)
Honduras*	2030	200·7 (181·6 to 222·3)	84·9 (69·5 to 103·4)	94·5 (86·3 to 102·8)	14·1 (9·9 to 19·7)	7·1 (6·5 to 7·9)	460·7 (416·9 to 510·3)	194·8 (159·5 to 237·4)	217·0 (198·2 to 236·0)	32·4 (22·7 to 45·2)	16·4 (14·8 to 18·2)
Honduras*	2031	204·1 (184·4 to 226·7)	86·7 (71·2 to 106·5)	95·6 (87·4 to 104·0)	14·6 (10·2 to 20·5)	7·2 (6·4 to 8·0)	468·4 (423·3 to 520·3)	198·9 (163·3 to 244·5)	219·5 (200·6 to 238·7)	33·6 (23·5 to 46·9)	16·5 (14·8 to 18·4)
Honduras*	2032	207·4 (186·8 to 230·5)	88·5 (72·1 to 109·4)	96·6 (88·3 to 105·1)	15·2 (10·6 to 21·3)	7·2 (6·5 to 8·1)	476·2 (428·8 to 529·2)	203·1 (165·4 to 251·1)	221·8 (202·7 to 241·2)	34·8 (24·4 to 48·9)	16·5 (14·8 to 18·5)

Honduras*	2033	211·0 (189·9 to 235·5)	90·3 (73·4 to 112·9)	97·7 (89·3 to 106·2)	15·7 (11·0 to 22·1)	7·2 (6·4 to 8·1)	484·3 (435·9 to 540·6)	207·4 (168·5 to 259·2)	224·2 (204·9 to 243·8)	36·1 (25·2 to 50·8)	16·5 (14·8 to 18·7)
Honduras*	2034	214·7 (192·8 to 239·8)	92·4 (74·2 to 114·9)	98·8 (90·3 to 107·4)	16·3 (11·3 to 23·1)	7·2 (6·5 to 8·2)	492·8 (442·6 to 550·4)	212·0 (170·4 to 263·9)	226·7 (207·3 to 246·5)	37·5 (26·0 to 52·9)	16·6 (14·9 to 18·7)
Honduras*	2035	218·3 (195·8 to 244·8)	94·3 (75·6 to 118·1)	99·9 (91·3 to 108·6)	17·0 (11·7 to 24·0)	7·3 (6·5 to 8·2)	501·3 (449·4 to 562·0)	216·4 (173·5 to 271·1)	229·2 (209·6 to 249·2)	39·0 (26·9 to 55·0)	16·7 (14·9 to 18·8)
Honduras*	2036	222·2 (198·8 to 249·6)	96·3 (76·4 to 120·9)	101·0 (92·3 to 109·8)	17·6 (12·2 to 24·9)	7·3 (6·5 to 8·3)	510·1 (456·5 to 572·9)	221·1 (175·4 to 277·5)	231·8 (211·9 to 252·0)	40·5 (27·9 to 57·2)	16·7 (14·9 to 19·0)
Honduras*	2037	226·2 (201·8 to 254·6)	98·5 (77·2 to 123·8)	102·1 (93·4 to 111·1)	18·3 (12·5 to 25·9)	7·3 (6·5 to 8·4)	519·4 (463·3 to 584·5)	226·0 (177·2 to 284·3)	234·5 (214·5 to 255·0)	42·1 (28·7 to 59·6)	16·8 (14·9 to 19·2)
Honduras*	2038	230·2 (205·0 to 259·7)	100·5 (79·5 to 126·9)	103·3 (94·6 to 112·3)	19·0 (12·9 to 26·9)	7·4 (6·5 to 8·5)	528·5 (470·5 to 596·2)	230·8 (182·4 to 291·2)	237·1 (217·1 to 257·7)	43·7 (29·6 to 61·8)	16·9 (15·0 to 19·4)
Honduras*	2039	234·3 (207·8 to 265·0)	102·7 (80·6 to 130·4)	104·3 (95·5 to 113·5)	19·8 (13·3 to 28·1)	7·4 (6·6 to 8·6)	537·9 (476·9 to 608·3)	235·9 (185·0 to 299·3)	239·5 (219·3 to 260·5)	45·4 (30·6 to 64·4)	17·1 (15·0 to 19·7)
Honduras*	2040	238·4 (211·6 to 271·2)	105·0 (82·2 to 134·6)	105·3 (96·5 to 114·6)	20·5 (13·8 to 29·3)	7·5 (6·6 to 8·7)	547·4 (485·7 to 622·7)	241·1 (188·7 to 309·0)	241·8 (221·6 to 263·1)	47·1 (31·7 to 67·2)	17·3 (15·2 to 20·0)
Honduras*	2041	242·6 (214·7 to 276·1)	107·3 (83·8 to 138·4)	106·3 (97·4 to 115·8)	21·3 (14·3 to 30·6)	7·6 (6·7 to 8·9)	556·9 (492·8 to 633·9)	246·3 (192·3 to 317·7)	244·1 (223·6 to 265·8)	49·0 (32·8 to 70·3)	17·6 (15·3 to 20·4)
Honduras*	2042	246·9 (218·1 to 281·8)	109·6 (85·1 to 142·0)	107·3 (98·3 to 116·9)	22·1 (14·8 to 32·1)	7·8 (6·7 to 9·1)	566·8 (500·7 to 646·9)	251·6 (195·4 to 325·9)	246·4 (225·8 to 268·5)	50·8 (33·9 to 73·7)	17·8 (15·4 to 20·9)
Honduras*	2043	251·3 (221·1 to 287·6)	112·0 (86·2 to 143·5)	108·4 (99·1 to 118·0)	23·0 (15·3 to 33·5)	7·9 (6·8 to 9·3)	576·9 (507·7 to 660·3)	257·2 (197·8 to 329·4)	248·7 (227·5 to 270·9)	52·8 (35·1 to 76·9)	18·1 (15·7 to 21·4)
Honduras*	2044	255·8 (224·5 to 292·4)	114·5 (87·2 to 148·3)	109·4 (100·0 to 119·2)	23·9 (15·8 to 34·9)	8·1 (6·9 to 9·5)	587·1 (515·5 to 671·1)	262·8 (200·2 to 340·3)	251·0 (229·5 to 273·6)	54·8 (36·2 to 80·2)	18·5 (15·9 to 21·8)
Honduras*	2045	260·0 (227·6 to 298·2)	116·7 (89·4 to 152·6)	110·3 (100·7 to 120·3)	24·8 (16·2 to 36·4)	8·2 (7·0 to 9·8)	596·9 (522·5 to 684·6)	268·0 (205·2 to 350·2)	253·2 (231·2 to 276·1)	56·9 (37·2 to 83·6)	18·8 (16·2 to 22·5)
Honduras*	2046	264·6 (230·3 to 304·8)	119·2 (89·5 to 155·1)	111·3 (101·8 to 121·4)	25·7 (16·7 to 37·9)	8·4 (7·1 to 10·0)	607·5 (528·8 to 699·6)	273·7 (205·5 to 356·0)	255·6 (233·6 to 278·6)	59·1 (38·3 to 87·0)	19·2 (16·4 to 23·0)

Honduras*	2047	269·1 (234·5 to 311·2)	121·6 (91·0 to 158·4)	112·3 (102·7 to 122·3)	26·7 (17·2 to 39·5)	8·5 (7·2 to 10·3)	617·8 (538·3 to 714·4)	279·2 (208·8 to 363·6)	257·7 (235·7 to 280·9)	61·3 (39·4 to 90·6)	19·6 (16·6 to 23·7)
Honduras*	2048	273·9 (237·5 to 317·9)	124·2 (92·5 to 165·1)	113·2 (103·5 to 123·5)	27·7 (17·7 to 41·1)	8·7 (7·4 to 10·6)	628·7 (545·3 to 729·9)	285·1 (212·4 to 378·9)	259·9 (237·6 to 283·4)	63·6 (40·6 to 94·3)	20·0 (17·0 to 24·3)
Honduras*	2049	278·6 (240·8 to 324·6)	126·8 (94·1 to 169·4)	114·1 (104·3 to 124·4)	28·7 (18·3 to 42·7)	8·9 (7·5 to 10·9)	639·7 (552·9 to 745·2)	291·1 (216·0 to 389·0)	262·0 (239·4 to 285·7)	66·0 (42·0 to 98·0)	20·5 (17·3 to 25·0)
Honduras*	2050	283·3 (244·7 to 330·7)	129·3 (94·8 to 172·6)	115·1 (105·0 to 125·4)	29·8 (18·8 to 44·5)	9·2 (7·6 to 11·2)	650·4 (561·6 to 759·3)	296·8 (217·6 to 396·3)	264·2 (241·1 to 287·8)	68·4 (43·1 to 102·1)	21·0 (17·5 to 25·8)
Croatia	1995	523·7 (486·6 to 563·8)	449·3 (414·8 to 487·9)	60·4 (50·2 to 72·2)	9·4 (6·4 to 13·4)	4·5 (4·5 to 4·5)	1032·9 (959·7 to 1112·1)	886·2 (818·2 to 962·3)	119·2 (98·9 to 142·4)	18·6 (12·6 to 26·5)	8·9 (8·9 to 8·9)
Croatia	1996	552·4 (518·7 to 585·9)	474·1 (443·9 to 505·1)	63·1 (52·9 to 75·1)	9·8 (6·4 to 14·2)	5·3 (5·3 to 5·3)	1089·5 (1023·1 to 1155·5)	935·2 (875·6 to 996·2)	124·5 (104·3 to 148·1)	19·4 (12·7 to 28·0)	10·4 (10·4 to 10·4)
Croatia	1997	562·9 (530·4 to 592·2)	481·8 (454·1 to 508·6)	67·0 (56·7 to 79·1)	10·1 (6·7 to 14·3)	4·0 (4·0 to 4·0)	1110·3 (1046·1 to 1168·0)	950·3 (895·7 to 1003·2)	132·1 (111·9 to 156·0)	19·9 (13·2 to 28·2)	7·9 (7·9 to 7·9)
Croatia	1998	619·1 (586·0 to 649·5)	533·2 (501·5 to 561·1)	73·0 (62·5 to 84·4)	10·9 (7·5 to 15·4)	2·0 (2·0 to 2·0)	1221·1 (1155·9 to 1281·2)	1051·6 (989·2 to 1106·7)	144·0 (123·3 to 166·4)	21·6 (14·8 to 30·5)	4·0 (4·0 to 4·0)
Croatia	1999	657·2 (623·3 to 689·0)	563·9 (532·5 to 592·9)	81·2 (70·6 to 93·1)	11·6 (8·0 to 16·3)	0·5 (0·5 to 0·5)	1296·3 (1229·3 to 1359·0)	1112·2 (1050·3 to 1169·5)	160·2 (139·3 to 183·7)	22·8 (15·7 to 32·1)	1·0 (1·0 to 1·0)
Croatia	2000	677·9 (646·8 to 708·8)	571·4 (542·7 to 600·7)	93·4 (81·9 to 106·5)	12·1 (8·3 to 16·9)	1·0 (1·0 to 1·0)	1337·0 (1275·7 to 1398·0)	1127·1 (1070·3 to 1184·8)	184·2 (161·5 to 210·0)	23·8 (16·4 to 33·3)	1·9 (1·9 to 1·9)
Croatia	2001	683·5 (654·0 to 716·8)	561·6 (534·4 to 589·4)	103·1 (91·4 to 116·9)	17·4 (12·3 to 24·2)	1·4 (1·4 to 1·4)	1348·1 (1290·0 to 1413·9)	1107·7 (1054·1 to 1162·5)	203·4 (180·3 to 230·5)	34·2 (24·3 to 47·7)	2·8 (2·8 to 2·8)
Croatia	2002	709·6 (676·9 to 742·6)	576·8 (548·5 to 607·9)	114·4 (101·7 to 128·4)	16·6 (11·7 to 22·9)	1·8 (1·8 to 1·8)	1399·6 (1335·2 to 1464·7)	1137·8 (1081·9 to 1199·0)	225·6 (200·6 to 253·3)	32·7 (23·1 to 45·1)	3·6 (3·6 to 3·6)
Croatia	2003	734·7 (703·1 to 767·9)	595·6 (566·6 to 625·6)	120·6 (107·4 to 135·6)	16·0 (11·4 to 21·9)	2·4 (2·4 to 2·4)	1449·1 (1386·7 to 1514·7)	1174·8 (1117·5 to 1234·0)	237·8 (211·8 to 267·5)	31·6 (22·5 to 43·3)	4·8 (4·8 to 4·8)
Croatia	2004	775·4 (740·2 to 808·5)	631·7 (603·4 to 663·1)	125·9 (112·8 to 141·0)	16·3 (11·6 to 21·9)	1·5 (1·5 to 1·5)	1529·3 (1460·0 to 1594·7)	1245·9 (1190·2 to 1308·0)	248·3 (222·5 to 278·2)	32·1 (23·0 to 43·2)	3·0 (3·0 to 3·0)

Croatia	2005	836·6 (803·2 to 870·5)	691·0 (660·7 to 723·7)	125·5 (112·8 to 139·6)	16·2 (11·8 to 21·3)	4·0 (4·0 to 4·0)	1650·1 (1584·3 to 1717·1)	1363·0 (1303·2 to 1427·4)	247·5 (222·5 to 275·3)	31·9 (23·3 to 42·1)	7·8 (7·8 to 7·8)
Croatia	2006	928·2 (892·9 to 962·6)	773·8 (740·5 to 806·3)	130·7 (118·8 to 144·6)	17·8 (13·5 to 22·9)	5·9 (5·9 to 5·9)	1830·7 (1761·1 to 1898·7)	1526·2 (1460·6 to 1590·3)	257·8 (234·2 to 285·3)	35·1 (26·7 to 45·2)	11·6 (11·6 to 11·6)
Croatia	2007	1009·8 (976·3 to 1044·9)	852·7 (821·1 to 884·7)	139·0 (128·5 to 151·5)	17·9 (14·0 to 22·6)	0·2 (0·2 to 0·2)	1991·8 (1925·6 to 2061·1)	1681·8 (1619·6 to 1745·1)	274·2 (253·5 to 298·8)	35·3 (27·7 to 44·5)	0·4 (0·4 to 0·4)
Croatia	2008	1065·4 (1035·1 to 1097·5)	894·0 (865·1 to 923·7)	148·3 (137·6 to 159·7)	23·1 (18·7 to 27·9)	0·0 (0·0 to 0·0)	2101·3 (2041·7 to 2164·6)	1763·2 (1706·3 to 1821·8)	292·4 (271·4 to 315·0)	45·6 (37·0 to 55·1)	0·0 (0·0 to 0·0)
Croatia	2009	1063·3 (1031·8 to 1091·5)	884·3 (856·6 to 912·2)	146·0 (136·0 to 156·5)	33·0 (28·0 to 38·6)	0·0 (0·0 to 0·0)	2097·2 (2035·1 to 2153·0)	1744·1 (1689·6 to 1799·2)	287·9 (268·2 to 308·7)	65·1 (55·3 to 76·2)	0·0 (0·0 to 0·0)
Croatia	2010	1023·7 (994·1 to 1053·7)	848·8 (820·1 to 876·5)	136·7 (127·5 to 146·8)	38·2 (33·2 to 43·6)	0·0 (0·0 to 0·0)	2019·2 (1960·8 to 2078·3)	1674·2 (1617·6 to 1728·8)	269·6 (251·6 to 289·6)	75·4 (65·5 to 85·9)	0·0 (0·0 to 0·0)
Croatia	2011	996·7 (965·0 to 1029·2)	821·7 (791·8 to 853·7)	122·8 (113·5 to 132·4)	52·2 (46·6 to 58·7)	0·0 (0·0 to 0·0)	1965·9 (1903·3 to 2029·9)	1620·6 (1561·7 to 1683·8)	242·2 (224·0 to 261·1)	103·1 (91·8 to 115·7)	0·0 (0·0 to 0·0)
Croatia	2012	935·0 (903·2 to 964·2)	771·4 (741·9 to 798·7)	108·0 (99·5 to 116·8)	55·5 (50·1 to 61·6)	0·0 (0·0 to 0·0)	1844·1 (1781·6 to 1901·8)	1521·5 (1463·4 to 1575·4)	213·1 (196·3 to 230·3)	109·5 (98·9 to 121·5)	0·0 (0·0 to 0·0)
Croatia	2013	866·6 (836·0 to 896·3)	721·3 (692·5 to 749·7)	93·9 (86·2 to 102·0)	51·4 (46·4 to 56·9)	0·0 (0·0 to 0·0)	1709·2 (1649·0 to 1767·9)	1422·7 (1365·9 to 1478·8)	185·2 (170·1 to 201·2)	101·3 (91·5 to 112·1)	0·0 (0·0 to 0·0)
Croatia	2014	844·6 (816·1 to 874·3)	704·6 (675·9 to 733·1)	92·8 (84·7 to 100·2)	47·1 (42·5 to 52·6)	0·0 (0·0 to 0·0)	1665·8 (1609·7 to 1724·5)	1389·8 (1333·2 to 1446·1)	183·1 (167·1 to 197·7)	92·9 (83·8 to 103·7)	0·0 (0·0 to 0·0)
Croatia	2015	868·8 (840·2 to 898·5)	722·8 (695·4 to 750·9)	94·7 (86·6 to 102·3)	51·3 (45·9 to 57·1)	0·0 (0·0 to 0·0)	1713·7 (1657·2 to 1772·2)	1425·7 (1371·6 to 1481·0)	186·8 (170·9 to 201·8)	101·2 (90·6 to 112·6)	0·0 (0·0 to 0·0)
Croatia	2016	908·9 (879·5 to 937·4)	746·3 (719·0 to 773·5)	97·6 (89·9 to 105·8)	55·4 (50·1 to 60·8)	9·6 (9·6 to 9·6)	1792·8 (1734·7 to 1848·9)	1472·1 (1418·1 to 1525·6)	192·6 (177·3 to 208·7)	109·2 (98·8 to 119·9)	18·9 (18·9 to 18·9)
Croatia	2017	922·1 (890·1 to 955·8)	764·3 (733·9 to 795·1)	100·0 (91·7 to 110·0)	57·8 (52·0 to 63·8)	0·0 (0·0 to 0·0)	1818·6 (1755·7 to 1885·3)	1507·5 (1447·4 to 1568·2)	197·2 (180·8 to 216·9)	113·9 (102·5 to 125·9)	0·0 (0·0 to 0·0)
Croatia	2018	973·6 (930·3 to 1014·6)	809·1 (767·7 to 848·7)	103·1 (92·6 to 115·4)	61·4 (54·4 to 69·0)	0·0 (0·0 to 0·0)	1920·3 (1834·9 to 2001·3)	1595·9 (1514·2 to 1674·1)	203·3 (182·6 to 227·5)	121·0 (107·2 to 136·1)	0·0 (0·0 to 0·0)

Croatia	2019	1024·0 (963·6 to 1081·9)	852·8 (795·5 to 912·2)	106·4 (95·5 to 119·2)	64·7 (57·3 to 72·9)	0·0 (0·0 to 0·0)	2019·8 (1900·7 to 2133·8)	1682·2 (1569·1 to 1799·2)	209·9 (188·4 to 235·1)	127·7 (113·0 to 143·8)	0·0 (0·0 to 0·0)
Croatia	2020	1047·4 (985·0 to 1114·7)	883·0 (823·4 to 953·1)	101·4 (91·0 to 113·5)	63·0 (55·8 to 71·2)	0·0 (0·0 to 0·0)	2065·9 (1942·9 to 2198·7)	1741·7 (1624·1 to 1879·8)	199·9 (179·6 to 223·8)	124·3 (110·0 to 140·4)	0·0 (0·0 to 0·0)
Croatia	2021	1011·7 (940·1 to 1082·0)	851·3 (780·9 to 924·2)	98·1 (88·1 to 109·9)	62·4 (55·3 to 70·3)	0·0 (0·0 to 0·0)	1995·6 (1854·2 to 2134·0)	1679·1 (1540·3 to 1822·9)	193·5 (173·7 to 216·7)	123·0 (109·2 to 138·7)	0·0 (0·0 to 0·0)
Croatia	2022	966·6 (879·7 to 1052·0)	804·6 (723·2 to 891·3)	98·2 (88·1 to 110·0)	63·8 (56·6 to 72·1)	0·0 (0·0 to 0·0)	1906·4 (1735·1 to 2074·9)	1587·0 (1426·5 to 1758·1)	193·6 (173·8 to 217·1)	125·9 (111·5 to 142·3)	0·0 (0·0 to 0·0)
Croatia	2023	974·7 (881·6 to 1068·2)	810·9 (716·1 to 901·6)	98·3 (88·2 to 110·2)	65·5 (58·1 to 74·0)	0·0 (0·0 to 0·0)	1922·6 (1738·9 to 2106·9)	1599·3 (1412·4 to 1778·3)	194·0 (173·9 to 217·3)	129·3 (114·5 to 145·9)	0·0 (0·0 to 0·0)
Croatia	2024	984·4 (883·9 to 1087·5)	818·7 (718·6 to 923·9)	98·4 (88·2 to 110·3)	67·2 (59·4 to 76·1)	0·0 (0·0 to 0·0)	1941·6 (1743·5 to 2145·0)	1614·8 (1417·3 to 1822·3)	194·1 (174·0 to 217·7)	132·6 (117·2 to 150·0)	0·0 (0·0 to 0·0)
Croatia	2025	995·9 (886·6 to 1109·7)	828·3 (718·1 to 938·8)	98·6 (88·4 to 110·6)	69·0 (60·9 to 78·1)	0·0 (0·0 to 0·0)	1964·2 (1748·7 to 2188·7)	1633·7 (1416·4 to 1851·7)	194·5 (174·4 to 218·2)	136·1 (120·2 to 154·0)	0·0 (0·0 to 0·0)
Croatia	2026	1006·3 (889·2 to 1125·5)	836·7 (722·0 to 953·4)	98·8 (88·6 to 110·8)	70·8 (62·4 to 80·2)	0·0 (0·0 to 0·0)	1984·8 (1753·9 to 2219·9)	1650·2 (1424·0 to 1880·5)	194·8 (174·7 to 218·6)	139·7 (123·1 to 158·1)	0·0 (0·0 to 0·0)
Croatia	2027	1017·5 (898·0 to 1140·8)	845·9 (724·4 to 967·2)	99·0 (88·7 to 111·0)	72·7 (63·9 to 82·4)	0·0 (0·0 to 0·0)	2007·0 (1771·2 to 2250·2)	1668·5 (1428·7 to 1907·6)	195·2 (174·9 to 218·9)	143·3 (126·1 to 162·5)	0·0 (0·0 to 0·0)
Croatia	2028	1031·2 (904·8 to 1169·1)	857·4 (733·0 to 989·9)	99·2 (89·0 to 111·3)	74·7 (65·9 to 84·9)	0·0 (0·0 to 0·0)	2034·0 (1784·6 to 2306·0)	1691·0 (1445·8 to 1952·4)	195·7 (175·4 to 219·5)	147·3 (130·0 to 167·4)	0·0 (0·0 to 0·0)
Croatia	2029	1043·3 (909·5 to 1189·3)	867·1 (733·6 to 1007·5)	99·4 (89·1 to 111·6)	76·7 (67·6 to 87·0)	0·0 (0·0 to 0·0)	2057·7 (1794·0 to 2345·8)	1710·3 (1447·0 to 1987·3)	196·1 (175·7 to 220·0)	151·3 (133·3 to 171·6)	0·0 (0·0 to 0·0)
Croatia	2030	1054·7 (915·8 to 1206·8)	876·6 (739·5 to 1027·5)	99·5 (89·1 to 111·6)	78·7 (69·1 to 89·7)	0·0 (0·0 to 0·0)	2080·4 (1806·3 to 2380·3)	1729·0 (1458·6 to 2026·7)	196·2 (175·7 to 220·1)	155·2 (136·3 to 176·9)	0·0 (0·0 to 0·0)
Croatia	2031	1068·1 (919·3 to 1232·7)	887·5 (740·1 to 1050·5)	99·7 (89·2 to 111·8)	80·9 (71·0 to 92·0)	0·0 (0·0 to 0·0)	2106·6 (1813·2 to 2431·3)	1750·4 (1459·7 to 2072·0)	196·7 (176·0 to 220·5)	159·6 (140·0 to 181·5)	0·0 (0·0 to 0·0)
Croatia	2032	1082·3 (924·2 to 1257·8)	899·0 (740·6 to 1070·9)	100·1 (89·5 to 112·2)	83·2 (73·1 to 95·1)	0·0 (0·0 to 0·0)	2134·7 (1822·9 to 2480·9)	1773·2 (1460·8 to 2112·2)	197·4 (176·4 to 221·3)	164·1 (144·2 to 187·5)	0·0 (0·0 to 0·0)

Croatia	2033	1094·9 (933·1 to 1275·6)	909·0 (748·6 to 1086·9)	100·4 (89·7 to 112·5)	85·6 (75·2 to 97·9)	0·0 (0·0 to 0·0)	2159·7 (1840·5 to 2516·0)	1792·8 (1476·4 to 2143·7)	198·1 (176·9 to 222·0)	168·7 (148·3 to 193·2)	0·0 (0·0 to 0·0)
Croatia	2034	1108·5 (947·1 to 1289·5)	919·7 (759·5 to 1102·2)	100·8 (90·0 to 112·9)	88·0 (77·2 to 100·9)	0·0 (0·0 to 0·0)	2186·4 (1868·1 to 2543·4)	1814·0 (1498·0 to 2173·9)	198·9 (177·5 to 222·8)	173·5 (152·2 to 199·0)	0·0 (0·0 to 0·0)
Croatia	2035	1120·8 (952·0 to 1313·6)	929·1 (763·1 to 1118·9)	101·3 (90·3 to 113·4)	90·5 (79·0 to 104·0)	0·0 (0·0 to 0·0)	2210·7 (1877·7 to 2591·0)	1832·5 (1505·1 to 2206·9)	199·8 (178·1 to 223·7)	178·4 (155·9 to 205·1)	0·0 (0·0 to 0·0)
Croatia	2036	1132·9 (951·4 to 1333·4)	938·3 (758·3 to 1140·9)	101·7 (90·6 to 113·8)	92·9 (80·9 to 106·9)	0·0 (0·0 to 0·0)	2234·6 (1876·6 to 2630·0)	1850·7 (1495·6 to 2250·3)	200·6 (178·7 to 224·4)	183·3 (159·5 to 210·9)	0·0 (0·0 to 0·0)
Croatia	2037	1144·6 (957·7 to 1350·7)	947·2 (766·0 to 1151·6)	102·0 (90·9 to 114·1)	95·5 (82·8 to 110·0)	0·0 (0·0 to 0·0)	2257·7 (1888·9 to 2664·2)	1868·2 (1510·8 to 2271·3)	201·2 (179·2 to 225·0)	188·3 (163·3 to 217·0)	0·0 (0·0 to 0·0)
Croatia	2038	1157·7 (964·2 to 1374·4)	957·4 (767·6 to 1175·0)	102·3 (91·1 to 114·4)	98·0 (84·5 to 113·3)	0·0 (0·0 to 0·0)	2283·5 (1901·7 to 2710·8)	1888·3 (1514·0 to 2317·7)	201·9 (179·7 to 225·7)	193·4 (166·7 to 223·5)	0·0 (0·0 to 0·0)
Croatia	2039	1171·5 (964·4 to 1389·5)	968·0 (763·0 to 1186·3)	102·7 (91·4 to 114·8)	100·7 (86·5 to 117·2)	0·0 (0·0 to 0·0)	2310·7 (1902·3 to 2740·6)	1909·3 (1505·0 to 2339·8)	202·6 (180·4 to 226·4)	198·7 (170·5 to 231·2)	0·0 (0·0 to 0·0)
Croatia	2040	1184·2 (976·6 to 1416·3)	977·6 (771·4 to 1206·4)	103·1 (91·7 to 115·1)	103·5 (88·3 to 120·9)	0·0 (0·0 to 0·0)	2335·7 (1926·2 to 2793·4)	1928·3 (1521·5 to 2379·6)	203·3 (180·9 to 227·1)	204·1 (174·2 to 238·4)	0·0 (0·0 to 0·0)
Croatia	2041	1195·5 (975·3 to 1437·6)	985·9 (770·5 to 1221·4)	103·4 (91·9 to 115·5)	106·3 (90·1 to 124·4)	0·0 (0·0 to 0·0)	2358·0 (1923·8 to 2835·5)	1944·5 (1519·7 to 2409·1)	203·9 (181·2 to 227·7)	209·6 (177·6 to 245·3)	0·0 (0·0 to 0·0)
Croatia	2042	1207·6 (987·7 to 1453·3)	994·7 (780·7 to 1237·7)	103·8 (92·1 to 115·9)	109·2 (92·0 to 129·1)	0·0 (0·0 to 0·0)	2381·9 (1948·2 to 2866·5)	1961·9 (1539·9 to 2441·2)	204·6 (181·7 to 228·5)	215·4 (181·5 to 254·6)	0·0 (0·0 to 0·0)
Croatia	2043	1219·7 (994·0 to 1472·4)	1003·6 (778·2 to 1255·9)	104·0 (92·3 to 116·1)	112·1 (93·9 to 133·4)	0·0 (0·0 to 0·0)	2405·8 (1960·5 to 2904·1)	1979·6 (1534·8 to 2477·1)	205·1 (182·0 to 229·0)	221·1 (185·2 to 263·2)	0·0 (0·0 to 0·0)
Croatia	2044	1232·2 (996·8 to 1493·7)	1012·9 (780·9 to 1269·6)	104·2 (92·4 to 116·4)	115·1 (95·8 to 137·5)	0·0 (0·0 to 0·0)	2430·3 (1966·0 to 2946·3)	1997·8 (1540·2 to 2504·2)	205·5 (182·2 to 229·5)	227·0 (188·9 to 271·3)	0·0 (0·0 to 0·0)
Croatia	2045	1244·1 (1008·5 to 1518·1)	1021·5 (791·5 to 1290·8)	104·4 (92·4 to 116·5)	118·2 (97·5 to 141·8)	0·0 (0·0 to 0·0)	2453·8 (1989·2 to 2994·2)	2014·9 (1561·2 to 2546·0)	205·9 (182·3 to 229·9)	233·0 (192·2 to 279·7)	0·0 (0·0 to 0·0)
Croatia	2046	1256·0 (1016·4 to 1540·3)	1030·3 (795·7 to 1315·1)	104·5 (92·5 to 116·7)	121·2 (99·3 to 146·5)	0·0 (0·0 to 0·0)	2477·4 (2004·8 to 3038·1)	2032·1 (1569·4 to 2593·9)	206·1 (182·5 to 230·2)	239·1 (195·8 to 289·0)	0·0 (0·0 to 0·0)

Croatia	2047	1266·7 (1020·7 to 1558·7)	1037·8 (798·0 to 1327·1)	104·6 (92·7 to 116·8)	124·3 (101·0 to 151·0)	0·0 (0·0 to 0·0)	2498·5 (2013·3 to 3074·3)	2046·9 (1573·9 to 2617·7)	206·3 (182·8 to 230·4)	245·2 (199·2 to 297·8)	0·0 (0·0 to 0·0)
Croatia	2048	1278·5 (1029·0 to 1578·9)	1046·3 (802·3 to 1339·5)	104·7 (92·7 to 117·0)	127·5 (102·7 to 156·0)	0·0 (0·0 to 0·0)	2521·7 (2029·5 to 3114·3)	2063·7 (1582·5 to 2641·9)	206·5 (182·9 to 230·7)	251·5 (202·5 to 307·7)	0·0 (0·0 to 0·0)
Croatia	2049	1287·0 (1032·9 to 1585·9)	1051·4 (801·9 to 1351·4)	104·8 (92·8 to 117·2)	130·8 (104·5 to 161·1)	0·0 (0·0 to 0·0)	2538·6 (2037·2 to 3128·1)	2073·8 (1581·7 to 2665·4)	206·8 (183·0 to 231·3)	257·9 (206·1 to 317·7)	0·0 (0·0 to 0·0)
Croatia	2050	1299·5 (1040·3 to 1618·7)	1060·3 (807·5 to 1370·0)	105·0 (92·9 to 117·6)	134·2 (106·2 to 166·1)	0·0 (0·0 to 0·0)	2563·1 (2051·8 to 3192·7)	2091·3 (1592·8 to 2702·1)	207·1 (183·2 to 231·9)	264·7 (209·5 to 327·5)	0·0 (0·0 to 0·0)
Haiti*	1995	59·7 (53·7 to 65·8)	9·6 (8·0 to 11·8)	27·1 (21·5 to 32·8)	2·1 (1·5 to 3·0)	20·9 (20·9 to 20·9)	141·0 (126·7 to 155·4)	22·8 (18·8 to 27·8)	63·9 (50·7 to 77·4)	5·0 (3·4 to 7·1)	49·3 (49·3 to 49·3)
Haiti*	1996	42·6 (36·8 to 48·7)	9·4 (7·8 to 11·6)	26·9 (21·3 to 32·6)	1·8 (1·2 to 2·6)	4·6 (4·6 to 4·6)	100·7 (86·8 to 114·9)	22·1 (18·3 to 27·3)	63·4 (50·3 to 77·1)	4·2 (2·9 to 6·1)	10·9 (10·9 to 10·9)
Haiti*	1997	41·6 (35·9 to 47·5)	9·1 (7·5 to 11·3)	26·1 (20·7 to 31·8)	1·6 (1·1 to 2·3)	4·8 (4·8 to 4·8)	98·1 (84·7 to 112·1)	21·5 (17·7 to 26·6)	61·6 (48·9 to 75·0)	3·7 (2·5 to 5·3)	11·3 (11·3 to 11·3)
Haiti*	1998	40·8 (35·5 to 46·4)	8·6 (7·1 to 10·8)	24·8 (19·7 to 30·4)	1·4 (1·0 to 2·0)	5·9 (5·9 to 5·9)	96·2 (83·8 to 109·6)	20·4 (16·7 to 25·4)	58·5 (46·6 to 71·8)	3·3 (2·3 to 4·7)	14·0 (14·0 to 14·0)
Haiti*	1999	44·5 (39·0 to 50·4)	9·7 (7·9 to 12·0)	25·5 (20·4 to 31·2)	1·5 (1·0 to 2·1)	7·9 (7·9 to 7·9)	105·1 (92·0 to 119·1)	22·8 (18·6 to 28·2)	60·1 (48·1 to 73·6)	3·4 (2·3 to 4·9)	18·7 (18·7 to 18·7)
Haiti*	2000	42·3 (36·9 to 48·4)	9·6 (7·9 to 11·8)	25·2 (20·3 to 30·6)	1·4 (1·0 to 2·0)	6·0 (6·0 to 6·0)	99·9 (87·0 to 114·2)	22·8 (18·6 to 27·9)	59·6 (48·0 to 72·2)	3·4 (2·3 to 4·8)	14·2 (14·2 to 14·2)
Haiti*	2001	39·9 (35·0 to 45·4)	8·8 (7·3 to 10·7)	24·1 (19·5 to 29·4)	1·4 (1·0 to 2·0)	5·7 (5·7 to 5·7)	94·3 (82·5 to 107·2)	20·7 (17·1 to 25·3)	56·9 (46·0 to 69·4)	3·3 (2·3 to 4·6)	13·4 (13·4 to 13·4)
Haiti*	2002	36·0 (31·3 to 41·5)	7·9 (6·5 to 9·6)	22·9 (18·5 to 28·0)	1·4 (1·0 to 1·9)	3·9 (3·9 to 3·9)	85·0 (73·9 to 98·0)	18·6 (15·4 to 22·7)	54·0 (43·6 to 66·2)	3·2 (2·3 to 4·5)	9·2 (9·2 to 9·2)
Haiti*	2003	38·8 (34·3 to 44·2)	7·5 (6·3 to 9·2)	21·9 (17·7 to 26·7)	1·4 (1·0 to 1·9)	8·0 (8·0 to 8·0)	91·6 (80·9 to 104·3)	17·8 (14·8 to 21·7)	51·8 (41·7 to 63·1)	3·3 (2·3 to 4·6)	18·8 (18·8 to 18·8)
Haiti*	2004	37·1 (32·9 to 41·8)	6·9 (5·7 to 8·4)	20·3 (16·4 to 24·8)	1·4 (0·9 to 1·9)	8·6 (8·6 to 8·6)	87·5 (77·7 to 98·7)	16·2 (13·4 to 19·8)	47·8 (38·8 to 58·5)	3·2 (2·2 to 4·4)	20·3 (20·3 to 20·3)
Haiti*	2005	37·5 (33·3 to 42·0)	6·9 (5·7 to 8·4)	19·5 (15·8 to 23·6)	1·4 (1·0 to 2·0)	9·7 (9·7 to 9·7)	88·5 (78·7 to 99·2)	16·2 (13·4 to 19·9)	46·0 (37·3 to 55·8)	3·3 (2·3 to 4·6)	23·0 (23·0 to 23·0)

Haiti*	2006	41·6 (37·8 to 45·9)	6·3 (5·2 to 7·7)	18·2 (14·7 to 22·1)	1·4 (0·9 to 2·0)	15·7 (15·7 to 15·7)	98·2 (89·1 to 108·4)	14·9 (12·2 to 18·2)	42·9 (34·7 to 52·2)	3·2 (2·2 to 4·6)	37·2 (37·2 to 37·2)
Haiti*	2007	45·2 (41·0 to 49·9)	8·0 (6·5 to 9·7)	18·9 (15·2 to 23·1)	1·5 (1·0 to 2·2)	16·9 (16·9 to 16·9)	106·8 (96·9 to 117·8)	18·8 (15·3 to 22·9)	44·5 (35·9 to 54·6)	3·6 (2·4 to 5·2)	39·8 (39·8 to 39·8)
Haiti*	2008	46·8 (42·5 to 51·4)	8·2 (6·7 to 9·9)	18·5 (14·8 to 22·8)	1·6 (1·1 to 2·2)	18·6 (18·6 to 18·6)	110·5 (100·4 to 121·4)	19·3 (15·8 to 23·4)	43·6 (35·0 to 53·7)	3·7 (2·5 to 5·2)	43·8 (43·8 to 43·8)
Haiti*	2009	46·0 (41·5 to 50·7)	8·8 (7·2 to 10·6)	19·2 (15·3 to 23·5)	1·6 (1·1 to 2·3)	16·5 (16·5 to 16·5)	108·7 (98·1 to 119·6)	20·7 (16·9 to 25·0)	45·2 (36·0 to 55·4)	3·9 (2·7 to 5·4)	38·9 (38·9 to 38·9)
Haiti*	2010	46·3 (41·8 to 51·2)	8·0 (6·6 to 9·7)	19·0 (15·2 to 23·6)	1·6 (1·1 to 2·3)	17·6 (17·6 to 17·6)	109·4 (98·8 to 120·8)	18·9 (15·6 to 22·9)	45·0 (35·8 to 55·7)	3·8 (2·6 to 5·4)	41·7 (41·7 to 41·7)
Haiti*	2011	49·0 (44·6 to 54·0)	7·1 (5·8 to 8·5)	19·0 (15·0 to 23·4)	1·6 (1·1 to 2·3)	21·3 (21·3 to 21·3)	115·8 (105·4 to 127·4)	16·7 (13·7 to 20·2)	44·9 (35·5 to 55·2)	3·9 (2·7 to 5·4)	50·3 (50·3 to 50·3)
Haiti*	2012	49·9 (45·3 to 54·8)	7·1 (5·8 to 8·6)	19·8 (15·8 to 24·5)	1·9 (1·3 to 2·6)	21·0 (21·0 to 21·0)	117·7 (106·9 to 129·4)	16·9 (13·8 to 20·4)	46·8 (37·2 to 57·8)	4·4 (3·0 to 6·2)	49·7 (49·7 to 49·7)
Haiti*	2013	50·6 (46·0 to 55·5)	7·1 (5·7 to 8·5)	20·0 (15·9 to 24·7)	2·1 (1·4 to 2·9)	21·4 (21·4 to 21·4)	119·3 (108·7 to 131·0)	16·7 (13·6 to 20·1)	47·3 (37·6 to 58·3)	4·8 (3·3 to 6·8)	50·5 (50·5 to 50·5)
Haiti*	2014	48·1 (43·5 to 53·2)	6·9 (5·6 to 8·2)	20·1 (16·0 to 24·8)	2·2 (1·5 to 3·1)	18·9 (18·9 to 18·9)	113·5 (102·8 to 125·5)	16·2 (13·2 to 19·4)	47·5 (37·7 to 58·6)	5·2 (3·6 to 7·4)	44·5 (44·5 to 44·5)
Haiti*	2015	49·2 (44·6 to 54·5)	6·7 (5·5 to 8·1)	20·9 (16·7 to 25·7)	2·4 (1·7 to 3·4)	19·1 (19·1 to 19·1)	116·0 (105·2 to 128·6)	15·9 (12·9 to 19·0)	49·3 (39·4 to 60·8)	5·8 (4·0 to 8·0)	45·1 (45·1 to 45·1)
Haiti*	2016	47·8 (43·2 to 53·3)	6·5 (5·2 to 7·8)	21·4 (17·1 to 26·5)	2·6 (1·7 to 3·6)	17·3 (17·3 to 17·3)	112·8 (102·1 to 125·7)	15·3 (12·4 to 18·3)	50·5 (40·3 to 62·6)	6·1 (4·1 to 8·6)	40·9 (40·9 to 40·9)
Haiti*	2017	45·9 (41·2 to 51·5)	6·5 (5·2 to 7·8)	22·2 (17·9 to 27·6)	2·7 (1·9 to 3·9)	14·4 (14·4 to 14·4)	108·3 (97·2 to 121·6)	15·4 (12·4 to 18·5)	52·4 (42·2 to 65·2)	6·4 (4·4 to 9·1)	34·0 (34·0 to 34·0)
Haiti*	2018	46·9 (42·1 to 52·7)	6·7 (5·4 to 8·1)	23·0 (18·5 to 28·2)	2·9 (2·0 to 4·1)	14·3 (14·3 to 14·3)	110·8 (99·5 to 124·5)	15·9 (12·8 to 19·2)	54·3 (43·7 to 66·6)	6·8 (4·6 to 9·7)	33·8 (33·8 to 33·8)
Haiti*	2019	47·1 (42·5 to 52·7)	5·3 (4·3 to 6·5)	22·5 (18·1 to 27·7)	2·9 (1·9 to 4·1)	16·4 (16·2 to 16·7)	111·3 (100·4 to 124·4)	12·6 (10·1 to 15·4)	53·2 (42·8 to 65·4)	6·8 (4·6 to 9·7)	38·7 (38·2 to 39·5)

Haiti*	2020	53·8 (49·4 to 59·3)	6·6 (5·3 to 8·1)	21·0 (17·0 to 25·8)	2·7 (1·9 to 3·9)	23·5 (22·8 to 24·6)	127·1 (116·6 to 140·0)	15·6 (12·5 to 19·1)	49·7 (40·0 to 61·0)	6·5 (4·4 to 9·3)	55·4 (53·9 to 58·1)
Haiti*	2021	53·1 (48·8 to 58·5)	6·3 (5·0 to 7·7)	20·0 (16·1 to 24·6)	2·7 (1·8 to 3·8)	24·2 (23·4 to 25·9)	125·4 (115·2 to 138·2)	14·9 (11·9 to 18·2)	47·2 (38·1 to 58·0)	6·3 (4·3 to 9·0)	57·1 (55·2 to 61·2)
Haiti*	2022	46·4 (41·9 to 52·1)	4·5 (3·6 to 5·6)	20·1 (16·2 to 24·6)	2·7 (1·9 to 3·9)	19·1 (17·7 to 21·0)	109·5 (99·0 to 123·0)	10·7 (8·6 to 13·1)	47·3 (38·2 to 58·2)	6·5 (4·4 to 9·2)	45·0 (41·9 to 49·5)
Haiti*	2023	47·1 (42·7 to 53·1)	4·5 (3·6 to 5·5)	20·2 (16·3 to 24·8)	2·8 (1·9 to 4·0)	19·7 (18·2 to 21·8)	111·3 (100·9 to 125·3)	10·6 (8·5 to 13·0)	47·6 (38·4 to 58·5)	6·6 (4·5 to 9·5)	46·5 (43·0 to 51·6)
Haiti*	2024	47·9 (43·4 to 54·0)	4·4 (3·5 to 5·4)	20·3 (16·3 to 25·0)	2·9 (2·0 to 4·1)	20·3 (18·7 to 22·8)	113·1 (102·4 to 127·5)	10·4 (8·3 to 12·8)	47·8 (38·6 to 58·9)	6·8 (4·6 to 9·8)	48·0 (44·0 to 53·8)
Haiti*	2025	48·7 (43·9 to 55·1)	4·4 (3·5 to 5·4)	20·4 (16·4 to 25·1)	3·0 (2·0 to 4·3)	20·9 (19·1 to 23·5)	114·9 (103·7 to 130·0)	10·3 (8·3 to 12·7)	48·1 (38·8 to 59·3)	7·0 (4·8 to 10·1)	49·4 (45·0 to 55·4)
Haiti*	2026	49·4 (44·5 to 55·9)	4·3 (3·4 to 5·3)	20·5 (16·5 to 25·3)	3·1 (2·1 to 4·4)	21·5 (19·5 to 24·4)	116·6 (105·0 to 132·0)	10·2 (8·1 to 12·5)	48·4 (39·0 to 59·6)	7·2 (4·9 to 10·4)	50·8 (46·0 to 57·6)
Haiti*	2027	50·1 (45·1 to 56·7)	4·3 (3·4 to 5·3)	20·6 (16·6 to 25·4)	3·1 (2·1 to 4·5)	22·1 (19·9 to 25·2)	118·3 (106·4 to 133·8)	10·1 (8·0 to 12·5)	48·7 (39·2 to 60·0)	7·4 (5·1 to 10·7)	52·1 (47·0 to 59·4)
Haiti*	2028	50·8 (45·7 to 57·6)	4·2 (3·4 to 5·3)	20·7 (16·7 to 25·6)	3·2 (2·2 to 4·7)	22·6 (20·3 to 26·1)	120·0 (107·8 to 135·9)	10·0 (7·9 to 12·4)	49·0 (39·5 to 60·3)	7·7 (5·2 to 11·0)	53·4 (47·8 to 61·6)
Haiti*	2029	51·6 (46·4 to 58·6)	4·2 (3·3 to 5·2)	20·9 (16·8 to 25·7)	3·3 (2·3 to 4·8)	23·2 (20·7 to 26·8)	121·8 (109·5 to 138·4)	9·9 (7·9 to 12·3)	49·3 (39·7 to 60·7)	7·9 (5·3 to 11·3)	54·7 (48·8 to 63·4)
Haiti*	2030	52·3 (46·8 to 59·5)	4·2 (3·3 to 5·2)	21·0 (16·9 to 25·9)	3·4 (2·3 to 4·9)	23·7 (21·0 to 27·7)	123·5 (110·6 to 140·4)	9·8 (7·8 to 12·2)	49·6 (39·9 to 61·1)	8·1 (5·5 to 11·6)	56·0 (49·7 to 65·3)
Haiti*	2031	53·1 (47·5 to 60·3)	4·1 (3·3 to 5·1)	21·1 (17·0 to 26·1)	3·5 (2·4 to 5·1)	24·2 (21·5 to 28·6)	125·2 (112·2 to 142·4)	9·8 (7·7 to 12·1)	49·9 (40·1 to 61·5)	8·3 (5·6 to 12·0)	57·2 (50·7 to 67·5)
Haiti*	2032	53·8 (48·2 to 61·4)	4·1 (3·3 to 5·1)	21·3 (17·1 to 26·2)	3·6 (2·5 to 5·2)	24·8 (21·7 to 29·2)	127·0 (113·8 to 145·0)	9·7 (7·7 to 12·0)	50·2 (40·3 to 61·9)	8·6 (5·8 to 12·3)	58·5 (51·3 to 69·0)
Haiti*	2033	54·5 (48·9 to 62·1)	4·1 (3·2 to 5·1)	21·4 (17·2 to 26·4)	3·7 (2·5 to 5·4)	25·3 (22·1 to 29·9)	128·7 (115·4 to 146·7)	9·7 (7·6 to 12·0)	50·6 (40·6 to 62·3)	8·8 (6·0 to 12·7)	59·6 (52·1 to 70·6)

Haiti*	2034	55·3 (49·5 to 62·9)	4·1 (3·2 to 5·1)	21·6 (17·3 to 26·6)	3·9 (2·6 to 5·5)	25·7 (22·4 to 30·5)	130·5 (116·8 to 148·6)	9·7 (7·6 to 12·0)	51·0 (40·9 to 62·8)	9·1 (6·2 to 13·0)	60·6 (52·9 to 72·0)
Haiti*	2035	56·0 (50·0 to 63·9)	4·1 (3·2 to 5·1)	21·8 (17·5 to 26·8)	4·0 (2·7 to 5·7)	26·2 (22·7 to 31·1)	132·3 (118·1 to 150·8)	9·7 (7·6 to 12·0)	51·4 (41·3 to 63·3)	9·4 (6·3 to 13·5)	61·8 (53·7 to 73·5)
Haiti*	2036	56·8 (50·7 to 64·6)	4·1 (3·2 to 5·1)	22·0 (17·6 to 27·0)	4·1 (2·8 to 5·9)	26·6 (23·1 to 31·9)	134·1 (119·8 to 152·6)	9·7 (7·6 to 12·0)	51·9 (41·6 to 63·8)	9·7 (6·6 to 14·0)	62·8 (54·4 to 75·3)
Haiti*	2037	57·6 (51·5 to 65·7)	4·1 (3·2 to 5·2)	22·2 (17·8 to 27·3)	4·2 (2·8 to 6·1)	27·1 (23·5 to 32·6)	136·0 (121·5 to 155·1)	9·8 (7·6 to 12·2)	52·3 (41·9 to 64·3)	10·0 (6·7 to 14·5)	63·9 (55·5 to 77·0)
Haiti*	2038	58·5 (52·1 to 66·8)	4·2 (3·3 to 5·2)	22·4 (17·9 to 27·5)	4·4 (2·9 to 6·4)	27·6 (23·8 to 33·3)	138·0 (122·9 to 157·6)	9·8 (7·7 to 12·3)	52·8 (42·3 to 64·9)	10·3 (6·9 to 15·0)	65·1 (56·2 to 78·7)
Haiti*	2039	59·4 (53·1 to 67·8)	4·2 (3·3 to 5·2)	22·5 (18·0 to 27·7)	4·5 (3·0 to 6·6)	28·2 (24·3 to 34·0)	140·3 (125·3 to 160·1)	9·9 (7·8 to 12·4)	53·2 (42·6 to 65·4)	10·6 (7·1 to 15·5)	66·5 (57·3 to 80·2)
Haiti*	2040	60·5 (53·9 to 69·3)	4·2 (3·3 to 5·3)	22·7 (18·2 to 27·9)	4·6 (3·1 to 6·7)	28·9 (24·6 to 35·1)	142·7 (127·3 to 163·5)	10·0 (7·8 to 12·5)	53·6 (42·9 to 65·9)	10·9 (7·2 to 15·9)	68·2 (58·2 to 82·9)
Haiti*	2041	61·5 (54·7 to 70·6)	4·3 (3·3 to 5·4)	22·9 (18·3 to 28·1)	4·8 (3·1 to 6·9)	29·6 (25·3 to 36·0)	145·2 (129·0 to 166·7)	10·1 (7·9 to 12·7)	54·0 (43·2 to 66·3)	11·3 (7·4 to 16·3)	69·8 (59·7 to 85·0)
Haiti*	2042	62·6 (55·4 to 71·9)	4·4 (3·4 to 5·4)	23·0 (18·5 to 28·3)	4·9 (3·2 to 7·1)	30·3 (25·7 to 37·1)	147·9 (130·8 to 169·7)	10·3 (8·0 to 12·8)	54·4 (43·6 to 66·8)	11·6 (7·6 to 16·8)	71·6 (60·7 to 87·7)
Haiti*	2043	63·8 (56·6 to 73·5)	4·4 (3·5 to 5·5)	23·2 (18·6 to 28·4)	5·1 (3·3 to 7·3)	31·1 (26·3 to 38·2)	150·6 (133·6 to 173·6)	10·4 (8·2 to 13·0)	54·7 (43·9 to 67·1)	11·9 (7·8 to 17·3)	73·5 (62·0 to 90·2)
Haiti*	2044	65·0 (57·6 to 75·0)	4·5 (3·5 to 5·6)	23·3 (18·7 to 28·6)	5·2 (3·4 to 7·6)	32·0 (27·0 to 39·4)	153·5 (136·1 to 177·0)	10·6 (8·3 to 13·3)	55·1 (44·1 to 67·6)	12·3 (8·0 to 17·8)	75·6 (63·8 to 93·1)
Haiti*	2045	66·2 (58·5 to 77·1)	4·6 (3·6 to 5·7)	23·5 (18·8 to 28·8)	5·3 (3·5 to 7·8)	32·8 (27·5 to 40·5)	156·3 (138·2 to 182·0)	10·8 (8·4 to 13·5)	55·4 (44·4 to 68·1)	12·6 (8·2 to 18·4)	77·4 (64·9 to 95·7)
Haiti*	2046	67·5 (59·6 to 78·1)	4·7 (3·6 to 5·8)	23·6 (18·9 to 29·0)	5·5 (3·6 to 8·1)	33·7 (28·1 to 41·4)	159·2 (140·7 to 184·4)	11·0 (8·6 to 13·7)	55·8 (44·7 to 68·5)	13·0 (8·4 to 19·0)	79·5 (66·3 to 97·8)
Haiti*	2047	68·7 (60·6 to 79·4)	4·7 (3·7 to 5·9)	23·7 (19·0 to 29·2)	5·6 (3·6 to 8·3)	34·6 (28·8 to 42·7)	162·2 (143·0 to 187·5)	11·2 (8·7 to 14·0)	56·1 (44·9 to 68·9)	13·3 (8·6 to 19·6)	81·7 (67·9 to 100·7)

Haiti*	2048	70·0 (61·6 to 81·0)	4·8 (3·8 to 6·0)	23·9 (19·1 to 29·3)	5·8 (3·7 to 8·5)	35·5 (29·7 to 43·7)	165·2 (145·4 to 191·2)	11·4 (8·9 to 14·3)	56·3 (45·1 to 69·3)	13·7 (8·8 to 20·1)	83·9 (70·0 to 103·1)
Haiti*	2049	71·3 (62·7 to 83·0)	4·9 (3·8 to 6·2)	23·9 (19·2 to 29·5)	5·9 (3·8 to 8·7)	36·5 (30·1 to 45·1)	168·4 (148·0 to 196·0)	11·6 (9·1 to 14·6)	56·5 (45·3 to 69·6)	14·0 (9·0 to 20·6)	86·2 (71·1 to 106·5)
Haiti*	2050	72·6 (63·9 to 84·1)	5·0 (3·9 to 6·3)	24·1 (19·2 to 29·6)	6·1 (3·9 to 9·0)	37·4 (30·8 to 46·3)	171·4 (150·8 to 198·6)	11·9 (9·3 to 14·9)	56·8 (45·4 to 69·9)	14·4 (9·1 to 21·3)	88·3 (72·8 to 109·4)
Hungary	1995	608·7 (576·1 to 646·4)	475·9 (448·0 to 508·4)	106·0 (91·8 to 121·4)	26·6 (18·5 to 37·2)	0·2 (0·2 to 0·2)	1284·2 (1215·4 to 1363·7)	1004·0 (945·1 to 1072·5)	223·7 (193·6 to 256·0)	56·1 (39·1 to 78·4)	0·4 (0·4 to 0·4)
Hungary	1996	590·7 (564·8 to 618·9)	451·9 (430·0 to 474·0)	111·2 (98·3 to 125·7)	26·0 (18·4 to 35·9)	1·5 (1·5 to 1·5)	1246·2 (1191·7 to 1305·8)	953·5 (907·2 to 1000·0)	234·7 (207·5 to 265·2)	54·8 (38·9 to 75·8)	3·2 (3·2 to 3·2)
Hungary	1997	594·5 (569·0 to 622·2)	445·3 (423·9 to 467·8)	121·8 (109·7 to 135·9)	26·0 (19·0 to 35·3)	1·4 (1·4 to 1·4)	1254·3 (1200·5 to 1312·8)	939·4 (894·3 to 987·0)	257·0 (231·3 to 286·8)	54·8 (40·1 to 74·6)	3·0 (3·0 to 3·0)
Hungary	1998	622·1 (597·1 to 648·3)	452·6 (431·9 to 474·7)	142·1 (129·6 to 155·0)	26·6 (19·9 to 35·6)	0·8 (0·8 to 0·8)	1312·4 (1259·7 to 1367·8)	955·0 (911·2 to 1001·6)	299·8 (273·5 to 327·1)	56·1 (42·0 to 75·1)	1·6 (1·6 to 1·6)
Hungary	1999	640·0 (615·0 to 664·7)	450·9 (429·4 to 472·8)	162·2 (150·6 to 174·2)	26·4 (19·8 to 35·1)	0·4 (0·4 to 0·4)	1350·2 (1297·6 to 1402·5)	951·3 (906·0 to 997·4)	342·3 (317·8 to 367·4)	55·7 (41·9 to 74·0)	0·8 (0·8 to 0·8)
Hungary	2000	665·7 (641·6 to 691·9)	458·4 (436·8 to 480·1)	180·7 (170·9 to 190·2)	26·3 (19·8 to 34·9)	0·4 (0·4 to 0·4)	1404·5 (1353·6 to 1459·7)	967·2 (921·5 to 1012·8)	381·2 (360·6 to 401·3)	55·4 (41·7 to 73·7)	0·7 (0·7 to 0·7)
Hungary	2001	710·0 (686·3 to 735·0)	483·8 (462·8 to 506·9)	198·4 (189·8 to 206·8)	27·8 (21·2 to 36·6)	0·0 (0·0 to 0·0)	1497·9 (1448·0 to 1550·6)	1020·7 (976·4 to 1069·5)	418·6 (400·5 to 436·2)	58·5 (44·7 to 77·2)	0·0 (0·0 to 0·0)
Hungary	2002	803·0 (779·3 to 828·8)	555·7 (532·9 to 579·2)	218·3 (209·6 to 227·1)	28·9 (22·6 to 37·1)	0·0 (0·0 to 0·0)	1694·1 (1644·3 to 1748·5)	1172·5 (1124·3 to 1222·0)	460·5 (442·3 to 479·2)	61·0 (47·7 to 78·4)	0·1 (0·1 to 0·1)
Hungary	2003	871·7 (846·2 to 897·1)	605·0 (582·7 to 628·5)	233·3 (224·7 to 242·1)	33·4 (26·7 to 41·5)	0·0 (0·0 to 0·0)	1839·1 (1785·3 to 1892·7)	1276·3 (1229·4 to 1326·1)	492·1 (474·0 to 510·8)	70·6 (56·3 to 87·5)	0·1 (0·1 to 0·1)
Hungary	2004	926·1 (900·8 to 952·3)	645·4 (622·7 to 668·8)	242·0 (233·3 to 250·5)	38·7 (31·7 to 46·6)	0·0 (0·0 to 0·0)	1954·0 (1900·5 to 2009·3)	1361·7 (1313·7 to 1411·0)	510·5 (492·3 to 528·4)	81·7 (67·0 to 98·3)	0·1 (0·1 to 0·1)
Hungary	2005	969·3 (944·3 to 994·1)	677·6 (654·7 to 700·3)	249·8 (241·0 to 258·3)	41·9 (36·0 to 48·7)	0·0 (0·0 to 0·0)	2045·0 (1992·3 to 2097·4)	1429·6 (1381·3 to 1477·5)	526·9 (508·4 to 545·0)	88·4 (75·9 to 102·8)	0·0 (0·0 to 0·0)

Hungary	2006	991·7 (969·3 to 1014·7)	692·1 (671·8 to 713·0)	253·2 (244·3 to 262·1)	46·3 (41·8 to 51·1)	0·0 (0·0 to 0·0)	2092·2 (2045·0 to 2140·9)	1460·2 (1417·3 to 1504·2)	534·3 (515·4 to 553·1)	97·6 (88·1 to 107·9)	0·0 (0·0 to 0·0)
Hungary	2007	943·2 (923·2 to 965·0)	646·7 (627·9 to 666·4)	248·6 (240·1 to 256·9)	47·9 (45·3 to 50·8)	0·0 (0·0 to 0·0)	1989·9 (1947·7 to 2036·0)	1364·3 (1324·7 to 1406·1)	524·5 (506·6 to 542·0)	101·1 (95·5 to 107·2)	0·0 (0·0 to 0·0)
Hungary	2008	920·7 (901·5 to 941·5)	625·3 (607·2 to 645·0)	245·8 (237·4 to 254·1)	49·6 (47·5 to 51·8)	0·0 (0·0 to 0·0)	1942·4 (1902·0 to 1986·3)	1319·3 (1281·1 to 1360·9)	518·5 (500·8 to 536·1)	104·6 (100·2 to 109·2)	0·0 (0·0 to 0·0)
Hungary	2009	902·8 (883·1 to 923·1)	608·8 (590·7 to 627·1)	242·6 (234·0 to 251·2)	51·3 (49·1 to 53·6)	0·0 (0·0 to 0·0)	1904·6 (1863·2 to 1947·6)	1284·5 (1246·3 to 1323·0)	511·9 (493·8 to 530·0)	108·2 (103·5 to 113·1)	0·0 (0·0 to 0·0)
Hungary	2010	915·5 (893·8 to 935·2)	609·9 (589·9 to 628·5)	252·1 (243·8 to 260·9)	53·5 (51·4 to 55·8)	0·0 (0·0 to 0·0)	1931·6 (1885·7 to 1973·1)	1286·7 (1244·6 to 1326·0)	531·9 (514·4 to 550·5)	112·9 (108·5 to 117·8)	0·0 (0·0 to 0·0)
Hungary	2011	938·4 (917·0 to 957·9)	620·2 (601·5 to 639·4)	265·0 (255·9 to 274·6)	53·1 (50·4 to 55·7)	0·0 (0·0 to 0·0)	1979·8 (1934·7 to 2021·0)	1308·6 (1269·1 to 1349·1)	559·2 (539·9 to 579·3)	112·0 (106·4 to 117·6)	0·0 (0·0 to 0·0)
Hungary	2012	921·4 (901·8 to 941·3)	603·0 (585·8 to 620·8)	267·2 (258·8 to 276·1)	51·0 (47·2 to 54·9)	0·2 (0·2 to 0·2)	1944·0 (1902·6 to 1985·9)	1272·3 (1236·0 to 1309·9)	563·7 (546·0 to 582·4)	107·6 (99·5 to 115·8)	0·5 (0·5 to 0·5)
Hungary	2013	925·0 (905·5 to 946·4)	611·2 (593·6 to 629·6)	265·3 (256·2 to 274·3)	48·6 (45·1 to 52·2)	0·0 (0·0 to 0·0)	1951·7 (1910·4 to 1996·7)	1289·4 (1252·5 to 1328·4)	559·7 (540·5 to 578·8)	102·5 (95·2 to 110·2)	0·1 (0·1 to 0·1)
Hungary	2014	938·3 (918·0 to 959·2)	627·2 (609·0 to 645·5)	264·8 (256·2 to 274·0)	46·3 (43·8 to 48·9)	0·0 (0·0 to 0·0)	1979·6 (1936·8 to 2023·7)	1323·2 (1284·9 to 1361·9)	558·6 (540·5 to 578·0)	97·8 (92·3 to 103·1)	0·0 (0·0 to 0·0)
Hungary	2015	973·1 (952·7 to 994·6)	659·3 (640·8 to 679·6)	268·0 (259·0 to 277·0)	45·8 (43·5 to 48·1)	0·0 (0·0 to 0·0)	2053·0 (2010·1 to 2098·4)	1391·0 (1351·9 to 1433·7)	565·3 (546·3 to 584·5)	96·6 (91·7 to 101·4)	0·0 (0·0 to 0·0)
Hungary	2016	976·2 (954·4 to 997·9)	661·4 (642·8 to 681·7)	270·0 (261·6 to 278·4)	44·8 (42·8 to 46·8)	0·0 (0·0 to 0·0)	2059·6 (2013·7 to 2105·4)	1395·5 (1356·3 to 1438·2)	569·6 (552·0 to 587·4)	94·5 (90·3 to 98·8)	0·0 (0·0 to 0·0)
Hungary	2017	1012·9 (991·6 to 1034·1)	694·3 (675·1 to 712·6)	275·0 (266·3 to 284·2)	43·5 (41·5 to 45·6)	0·0 (0·0 to 0·0)	2136·9 (2092·2 to 2181·8)	1464·9 (1424·3 to 1503·4)	580·3 (561·8 to 599·7)	91·8 (87·6 to 96·3)	0·0 (0·0 to 0·0)
Hungary	2018	1052·5 (1025·4 to 1080·3)	727·9 (701·9 to 753·4)	282·1 (271·0 to 293·8)	42·4 (39·6 to 45·4)	0·0 (0·0 to 0·0)	2220·5 (2163·4 to 2279·1)	1535·8 (1480·8 to 1589·5)	595·2 (571·8 to 619·9)	89·5 (83·6 to 95·7)	0·0 (0·0 to 0·0)
Hungary	2019	1099·9 (1067·8 to 1132·6)	759·3 (728·0 to 789·7)	295·2 (283·6 to 307·4)	45·4 (42·4 to 48·6)	0·0 (0·0 to 0·0)	2320·6 (2252·8 to 2389·7)	1602·1 (1536·0 to 1666·1)	622·7 (598·2 to 648·5)	95·8 (89·5 to 102·6)	0·0 (0·0 to 0·0)

Hungary	2020	1277·4 (1245·3 to 1310·0)	948·4 (917·0 to 978·9)	284·0 (272·8 to 295·7)	44·9 (41·8 to 48·4)	0·0 (0·0 to 0·0)	2695·0 (2627·4 to 2763·8)	2001·0 (1934·7 to 2065·2)	599·2 (575·6 to 623·8)	94·8 (88·1 to 102·0)	0·0 (0·0 to 0·0)
Hungary	2021	1110·3 (1075·2 to 1148·0)	793·4 (759·0 to 829·6)	272·6 (261·9 to 284·0)	44·3 (41·2 to 47·8)	0·0 (0·0 to 0·0)	2342·6 (2268·5 to 2422·0)	1673·8 (1601·4 to 1750·3)	575·2 (552·6 to 599·2)	93·5 (86·9 to 100·9)	0·0 (0·0 to 0·0)
Hungary	2022	1031·0 (995·7 to 1068·0)	712·3 (677·7 to 748·5)	273·1 (262·3 to 284·5)	45·6 (42·2 to 49·4)	0·0 (0·0 to 0·0)	2175·3 (2100·7 to 2253·2)	1502·9 (1429·9 to 1579·2)	576·3 (553·4 to 600·3)	96·1 (89·1 to 104·1)	0·0 (0·0 to 0·0)
Hungary	2023	1039·4 (1001·9 to 1078·5)	718·2 (681·2 to 756·0)	274·3 (263·3 to 285·7)	46·9 (43·4 to 50·9)	0·0 (0·0 to 0·0)	2192·8 (2113·7 to 2275·4)	1515·3 (1437·2 to 1594·9)	578·7 (555·6 to 602·8)	98·9 (91·5 to 107·4)	0·0 (0·0 to 0·0)
Hungary	2024	1050·1 (1009·8 to 1092·6)	725·9 (687·4 to 767·6)	275·9 (264·9 to 287·5)	48·2 (44·6 to 52·7)	0·0 (0·0 to 0·0)	2215·4 (2130·6 to 2305·1)	1531·5 (1450·3 to 1619·4)	582·1 (558·9 to 606·5)	101·8 (94·1 to 111·1)	0·0 (0·0 to 0·0)
Hungary	2025	1061·5 (1020·4 to 1106·5)	734·1 (694·5 to 777·7)	277·8 (266·7 to 289·6)	49·7 (45·7 to 54·3)	0·0 (0·0 to 0·0)	2239·6 (2152·9 to 2334·5)	1548·8 (1465·3 to 1640·8)	586·0 (562·6 to 611·0)	104·8 (96·5 to 114·7)	0·0 (0·0 to 0·0)
Hungary	2026	1073·4 (1029·7 to 1121·6)	742·5 (700·7 to 789·7)	279·7 (268·6 to 291·6)	51·1 (47·0 to 56·1)	0·0 (0·0 to 0·0)	2264·6 (2172·5 to 2366·3)	1566·5 (1478·3 to 1666·2)	590·2 (566·8 to 615·3)	107·9 (99·1 to 118·3)	0·0 (0·0 to 0·0)
Hungary	2027	1085·0 (1038·5 to 1134·6)	750·8 (704·9 to 800·2)	281·6 (270·3 to 293·6)	52·6 (48·2 to 57·8)	0·0 (0·0 to 0·0)	2289·2 (2190·9 to 2393·8)	1584·0 (1487·2 to 1688·3)	594·2 (570·3 to 619·5)	111·0 (101·6 to 122·0)	0·0 (0·0 to 0·0)
Hungary	2028	1097·7 (1048·6 to 1150·0)	759·9 (711·3 to 811·3)	283·7 (272·2 to 295·7)	54·2 (49·5 to 59·9)	0·0 (0·0 to 0·0)	2316·0 (2212·3 to 2426·4)	1603·3 (1500·6 to 1711·7)	598·5 (574·3 to 623·8)	114·3 (104·4 to 126·3)	0·0 (0·0 to 0·0)
Hungary	2029	1111·6 (1057·5 to 1167·8)	769·9 (717·5 to 826·1)	285·9 (274·4 to 298·1)	55·8 (50·7 to 61·8)	0·0 (0·0 to 0·0)	2345·3 (2231·2 to 2463·7)	1624·4 (1513·8 to 1742·8)	603·2 (578·9 to 628·9)	117·7 (106·9 to 130·4)	0·0 (0·0 to 0·0)
Hungary	2030	1125·2 (1070·6 to 1184·2)	779·7 (726·8 to 838·3)	288·2 (276·5 to 300·5)	57·4 (52·0 to 63·8)	0·0 (0·0 to 0·0)	2374·0 (2258·7 to 2498·4)	1645·0 (1533·4 to 1768·6)	608·0 (583·4 to 634·0)	121·1 (109·8 to 134·6)	0·0 (0·0 to 0·0)
Hungary	2031	1138·6 (1080·7 to 1200·5)	789·0 (732·1 to 851·5)	290·4 (278·8 to 302·8)	59·1 (53·7 to 65·7)	0·0 (0·0 to 0·0)	2402·2 (2280·1 to 2532·7)	1664·6 (1544·6 to 1796·5)	612·8 (588·1 to 638·9)	124·8 (113·4 to 138·7)	0·0 (0·0 to 0·0)
Hungary	2032	1151·2 (1090·8 to 1218·0)	797·8 (739·4 to 865·2)	292·5 (280·7 to 304·9)	60·9 (55·3 to 67·7)	0·0 (0·0 to 0·0)	2428·7 (2301·4 to 2569·7)	1683·2 (1560·0 to 1825·5)	617·0 (592·1 to 643·3)	128·5 (116·7 to 142·8)	0·0 (0·0 to 0·0)
Hungary	2033	1162·4 (1100·4 to 1229·9)	805·6 (743·0 to 874·3)	294·2 (282·4 to 306·7)	62·6 (56·8 to 69·7)	0·0 (0·0 to 0·0)	2452·3 (2321·5 to 2594·9)	1699·6 (1567·7 to 1844·7)	620·6 (595·7 to 647·0)	132·1 (119·8 to 147·1)	0·0 (0·0 to 0·0)

Hungary	2034	1172·8 (1109·1 to 1241·3)	812·8 (750·5 to 881·7)	295·6 (283·7 to 308·5)	64·4 (58·4 to 71·8)	0·0 (0·0 to 0·0)	2474·3 (2340·0 to 2618·8)	1714·8 (1583·4 to 1860·3)	623·7 (598·6 to 650·8)	135·8 (123·3 to 151·5)	0·0 (0·0 to 0·0)
Hungary	2035	1182·4 (1115·1 to 1253·5)	819·4 (752·8 to 890·9)	296·9 (285·0 to 309·8)	66·1 (59·8 to 74·0)	0·0 (0·0 to 0·0)	2494·6 (2352·7 to 2644·5)	1728·8 (1588·2 to 1879·5)	626·3 (601·2 to 653·7)	139·5 (126·1 to 156·0)	0·0 (0·0 to 0·0)
Hungary	2036	1191·3 (1121·8 to 1266·7)	825·5 (757·6 to 899·6)	297·9 (286·0 to 310·9)	67·8 (61·1 to 76·3)	0·0 (0·0 to 0·0)	2513·4 (2366·8 to 2672·6)	1741·7 (1598·4 to 1897·9)	628·5 (603·4 to 656·0)	143·1 (128·9 to 160·9)	0·0 (0·0 to 0·0)
Hungary	2037	1199·4 (1127·1 to 1275·4)	831·1 (758·3 to 906·0)	298·7 (286·8 to 311·8)	69·6 (62·4 to 78·3)	0·0 (0·0 to 0·0)	2530·4 (2377·9 to 2690·8)	1753·4 (1599·8 to 1911·6)	630·3 (605·1 to 657·8)	146·8 (131·6 to 165·2)	0·0 (0·0 to 0·0)
Hungary	2038	1205·1 (1130·7 to 1280·6)	835·0 (761·9 to 910·5)	298·9 (287·0 to 311·9)	71·2 (63·5 to 80·3)	0·0 (0·0 to 0·0)	2542·6 (2385·5 to 2701·9)	1761·8 (1607·5 to 1920·9)	630·7 (605·4 to 658·1)	150·2 (134·1 to 169·4)	0·0 (0·0 to 0·0)
Hungary	2039	1208·4 (1132·9 to 1283·4)	837·0 (763·4 to 912·7)	298·6 (286·7 to 311·7)	72·7 (64·6 to 82·4)	0·0 (0·0 to 0·0)	2549·4 (2390·2 to 2707·7)	1765·9 (1610·7 to 1925·7)	630·1 (604·8 to 657·6)	153·4 (136·2 to 173·9)	0·0 (0·0 to 0·0)
Hungary	2040	1211·3 (1132·4 to 1289·5)	838·9 (760·6 to 916·9)	298·1 (286·2 to 311·2)	74·3 (65·6 to 84·4)	0·0 (0·0 to 0·0)	2555·7 (2389·2 to 2720·5)	1770·0 (1604·7 to 1934·4)	629·0 (603·8 to 656·6)	156·7 (138·4 to 178·1)	0·0 (0·0 to 0·0)
Hungary	2041	1214·4 (1132·8 to 1294·5)	840·9 (760·8 to 921·0)	297·7 (285·8 to 310·5)	75·8 (66·6 to 86·7)	0·0 (0·0 to 0·0)	2562·1 (2390·0 to 2731·2)	1774·1 (1605·1 to 1943·2)	628·1 (602·9 to 655·1)	160·0 (140·4 to 182·9)	0·0 (0·0 to 0·0)
Hungary	2042	1219·7 (1138·8 to 1302·0)	844·5 (765·4 to 929·6)	297·6 (285·6 to 310·5)	77·5 (67·6 to 89·1)	0·0 (0·0 to 0·0)	2573·3 (2402·6 to 2746·9)	1781·8 (1614·8 to 1961·3)	628·0 (602·6 to 655·0)	163·6 (142·7 to 188·1)	0·0 (0·0 to 0·0)
Hungary	2043	1225·2 (1141·4 to 1310·5)	848·1 (766·8 to 933·7)	297·7 (285·5 to 310·5)	79·3 (68·7 to 91·5)	0·0 (0·0 to 0·0)	2584·9 (2408·0 to 2764·8)	1789·4 (1617·8 to 1970·0)	628·2 (602·4 to 655·1)	167·3 (144·9 to 193·1)	0·0 (0·0 to 0·0)
Hungary	2044	1231·5 (1146·3 to 1320·4)	852·2 (770·7 to 940·2)	298·1 (285·8 to 311·0)	81·2 (69·8 to 94·3)	0·0 (0·0 to 0·0)	2598·2 (2418·6 to 2785·9)	1798·0 (1626·0 to 1983·5)	628·8 (603·0 to 656·1)	171·3 (147·2 to 198·9)	0·0 (0·0 to 0·0)
Hungary	2045	1238·8 (1150·2 to 1332·0)	857·1 (769·4 to 947·2)	298·5 (286·0 to 311·6)	83·2 (70·9 to 97·3)	0·0 (0·0 to 0·0)	2613·6 (2426·8 to 2810·3)	1808·4 (1623·3 to 1998·4)	629·7 (603·4 to 657·5)	175·5 (149·7 to 205·2)	0·0 (0·0 to 0·0)
Hungary	2046	1246·8 (1155·1 to 1340·9)	862·5 (773·6 to 955·6)	299·1 (286·4 to 312·3)	85·2 (72·2 to 100·8)	0·0 (0·0 to 0·0)	2630·6 (2437·0 to 2828·9)	1819·6 (1632·1 to 2016·0)	631·1 (604·2 to 658·9)	179·9 (152·4 to 212·6)	0·0 (0·0 to 0·0)
Hungary	2047	1256·8 (1166·1 to 1353·4)	869·3 (779·8 to 963·5)	300·1 (287·2 to 313·4)	87·5 (73·6 to 104·5)	0·0 (0·0 to 0·0)	2651·7 (2460·2 to 2855·4)	1834·0 (1645·1 to 2032·8)	633·1 (605·9 to 661·2)	184·5 (155·2 to 220·4)	0·0 (0·0 to 0·0)

Hungary	2048	1268·4 (1174·6 to 1367·3)	877·1 (784·9 to 977·1)	301·5 (288·5 to 315·0)	89·9 (75·1 to 108·1)	0·0 (0·0 to 0·0)	2676·1 (2478·2 to 2884·8)	1850·4 (1655·9 to 2061·4)	636·0 (608·7 to 664·6)	189·6 (158·4 to 228·0)	0·0 (0·0 to 0·0)
Hungary	2049	1281·7 (1186·9 to 1386·5)	886·0 (791·1 to 985·2)	303·2 (290·2 to 317·0)	92·5 (76·7 to 112·3)	0·0 (0·0 to 0·0)	2704·1 (2504·1 to 2925·3)	1869·3 (1669·1 to 2078·7)	639·7 (612·2 to 668·9)	195·1 (161·7 to 236·9)	0·0 (0·0 to 0·0)
Hungary	2050	1295·7 (1197·0 to 1404·7)	895·4 (798·0 to 1000·0)	305·1 (292·0 to 319·0)	95·2 (78·3 to 116·7)	0·0 (0·0 to 0·0)	2733·7 (2525·5 to 2963·6)	1889·2 (1683·7 to 2109·8)	643·8 (616·1 to 673·1)	200·8 (165·3 to 246·2)	0·0 (0·0 to 0·0)
Indonesia*	1995	40·1 (35·0 to 46·3)	10·9 (8·9 to 13·2)	16·3 (12·8 to 19·9)	11·7 (8·1 to 16·4)	1·2 (1·2 to 1·2)	122·6 (106·9 to 141·5)	33·3 (27·3 to 40·2)	49·7 (39·2 to 60·7)	35·7 (24·9 to 50·1)	3·8 (3·8 to 3·8)
Indonesia*	1996	41·4 (36·1 to 47·7)	11·4 (9·4 to 13·8)	17·1 (13·5 to 21·0)	12·2 (8·4 to 17·0)	0·7 (0·7 to 0·7)	126·6 (110·3 to 146·0)	35·0 (28·6 to 42·3)	52·4 (41·3 to 64·2)	37·3 (25·8 to 52·0)	2·0 (2·0 to 2·0)
Indonesia*	1997	44·5 (38·5 to 51·2)	12·7 (10·5 to 15·3)	18·2 (14·6 to 22·1)	12·9 (8·9 to 17·7)	0·7 (0·7 to 0·7)	135·9 (117·8 to 156·6)	38·8 (31·9 to 46·9)	55·6 (44·6 to 67·7)	39·4 (27·4 to 54·0)	2·1 (2·1 to 2·1)
Indonesia*	1998	41·4 (36·0 to 47·6)	11·4 (9·4 to 13·7)	17·3 (14·1 to 21·1)	11·9 (8·4 to 16·4)	0·7 (0·7 to 0·7)	126·4 (110·1 to 145·7)	34·8 (28·9 to 42·0)	53·0 (43·2 to 64·6)	36·5 (25·6 to 50·0)	2·1 (2·1 to 2·1)
Indonesia*	1999	41·1 (35·9 to 46·8)	11·5 (9·5 to 13·7)	17·4 (14·3 to 20·9)	11·4 (8·0 to 16·2)	0·8 (0·8 to 0·8)	125·8 (109·7 to 143·2)	35·3 (29·1 to 42·0)	53·2 (43·7 to 64·0)	34·9 (24·6 to 49·4)	2·3 (2·3 to 2·3)
Indonesia*	2000	40·2 (35·6 to 45·5)	11·9 (9·9 to 14·2)	17·2 (14·5 to 20·6)	9·7 (6·9 to 13·7)	1·3 (1·3 to 1·3)	122·8 (108·9 to 139·1)	36·3 (30·2 to 43·5)	52·6 (44·3 to 63·0)	29·8 (20·9 to 41·8)	4·0 (4·0 to 4·0)
Indonesia*	2001	44·4 (39·4 to 49·8)	14·8 (12·3 to 17·8)	18·9 (16·1 to 22·2)	9·8 (6·9 to 13·5)	0·9 (0·9 to 0·9)	135·6 (120·5 to 152·3)	45·2 (37·5 to 54·4)	57·7 (49·3 to 67·8)	29·8 (21·0 to 41·1)	2·8 (2·8 to 2·8)
Indonesia*	2002	43·4 (38·7 to 48·4)	14·3 (12·0 to 17·2)	19·6 (17·1 to 22·5)	8·9 (6·2 to 12·3)	0·6 (0·6 to 0·6)	132·6 (118·2 to 148·0)	43·7 (36·6 to 52·6)	59·8 (52·3 to 68·7)	27·2 (18·9 to 37·6)	1·9 (1·9 to 1·9)
Indonesia*	2003	48·1 (43·4 to 53·3)	16·0 (13·6 to 19·2)	22·0 (19·3 to 24·8)	9·2 (6·4 to 12·6)	0·9 (0·9 to 0·9)	147·0 (132·7 to 162·9)	49·0 (41·5 to 58·7)	67·3 (59·0 to 75·8)	28·0 (19·5 to 38·4)	2·7 (2·7 to 2·7)
Indonesia*	2004	51·2 (46·5 to 56·6)	16·7 (14·1 to 20·2)	24·3 (21·6 to 27·4)	9·2 (6·4 to 12·6)	0·9 (0·9 to 0·9)	156·4 (142·2 to 172·9)	51·1 (43·2 to 61·9)	74·3 (65·9 to 83·7)	28·1 (19·6 to 38·6)	2·8 (2·8 to 2·8)
Indonesia*	2005	56·2 (51·3 to 62·0)	17·6 (14·8 to 21·4)	28·2 (25·1 to 31·5)	9·4 (6·7 to 12·8)	1·0 (1·0 to 1·0)	171·8 (156·7 to 189·6)	53·9 (45·3 to 65·4)	86·2 (76·8 to 96·2)	28·8 (20·5 to 39·0)	2·9 (2·9 to 2·9)

Indonesia*	2006	63·3 (57·4 to 69·9)	20·0 (16·6 to 24·2)	31·9 (28·6 to 35·5)	10·0 (7·1 to 13·6)	1·4 (1·4 to 1·4)	193·5 (175·5 to 213·7)	61·3 (50·8 to 74·1)	97·4 (87·5 to 108·4)	30·7 (21·6 to 41·6)	4·1 (4·1 to 4·1)
Indonesia*	2007	69·0 (62·7 to 76·0)	22·3 (18·5 to 26·8)	34·8 (31·5 to 38·4)	10·7 (7·5 to 14·8)	1·1 (1·1 to 1·1)	210·8 (191·7 to 232·5)	68·2 (56·5 to 82·0)	106·3 (96·2 to 117·4)	32·8 (23·1 to 45·4)	3·4 (3·4 to 3·4)
Indonesia*	2008	75·0 (68·4 to 82·0)	24·2 (20·1 to 28·9)	37·6 (33·9 to 41·3)	11·7 (8·3 to 16·4)	1·5 (1·5 to 1·5)	229·2 (209·1 to 250·6)	74·0 (61·5 to 88·4)	114·8 (103·7 to 126·1)	35·8 (25·5 to 50·1)	4·6 (4·6 to 4·6)
Indonesia*	2009	76·7 (70·1 to 83·5)	23·5 (19·6 to 28·0)	39·9 (36·3 to 43·4)	12·1 (8·5 to 16·7)	1·3 (1·3 to 1·3)	234·5 (214·4 to 255·3)	71·7 (60·0 to 85·7)	122·0 (111·1 to 132·8)	36·9 (26·0 to 51·1)	3·9 (3·9 to 3·9)
Indonesia*	2010	83·2 (76·4 to 90·1)	24·2 (20·5 to 28·6)	44·8 (41·3 to 48·1)	13·1 (9·2 to 17·7)	1·2 (1·2 to 1·2)	254·4 (233·7 to 275·3)	74·0 (62·6 to 87·5)	136·9 (126·3 to 147·0)	39·9 (28·1 to 54·2)	3·6 (3·6 to 3·6)
Indonesia*	2011	90·6 (83·8 to 97·8)	27·0 (22·8 to 31·4)	48·4 (45·5 to 51·4)	14·0 (9·9 to 19·0)	1·2 (1·2 to 1·2)	276·9 (256·0 to 299·0)	82·4 (69·7 to 96·1)	147·9 (139·0 to 157·1)	42·9 (30·2 to 58·2)	3·7 (3·7 to 3·7)
Indonesia*	2012	96·5 (89·7 to 103·7)	30·6 (26·3 to 35·3)	49·2 (46·4 to 51·9)	15·5 (11·0 to 21·0)	1·2 (1·2 to 1·2)	294·9 (274·1 to 317·0)	93·6 (80·3 to 107·8)	150·3 (142·0 to 158·6)	47·3 (33·6 to 64·2)	3·7 (3·7 to 3·7)
Indonesia*	2013	102·1 (94·7 to 110·2)	34·7 (30·4 to 39·2)	48·3 (45·7 to 50·9)	17·7 (12·6 to 23·9)	1·4 (1·4 to 1·4)	312·2 (289·5 to 336·7)	106·1 (93·0 to 120·0)	147·6 (139·6 to 155·7)	54·1 (38·7 to 73·2)	4·4 (4·4 to 4·4)
Indonesia*	2014	106·3 (98·8 to 114·3)	39·5 (35·4 to 44·1)	46·4 (43·9 to 49·0)	19·6 (13·9 to 26·3)	0·8 (0·8 to 0·8)	324·9 (302·2 to 349·4)	120·7 (108·3 to 134·7)	141·8 (134·1 to 149·7)	59·9 (42·6 to 80·5)	2·5 (2·5 to 2·5)
Indonesia*	2015	107·9 (101·1 to 115·7)	44·1 (40·4 to 48·1)	44·2 (41·7 to 46·9)	18·7 (13·3 to 24·9)	0·8 (0·8 to 0·8)	329·7 (309·1 to 353·7)	134·9 (123·5 to 147·2)	135·3 (127·6 to 143·3)	57·2 (40·6 to 76·0)	2·4 (2·4 to 2·4)
Indonesia*	2016	111·0 (104·2 to 118·7)	49·2 (45·9 to 52·5)	42·6 (40·0 to 45·4)	18·5 (13·0 to 24·8)	0·8 (0·8 to 0·8)	339·3 (318·6 to 362·9)	150·4 (140·4 to 160·3)	130·1 (122·2 to 138·8)	56·5 (39·8 to 75·7)	2·4 (2·4 to 2·4)
Indonesia*	2017	113·5 (106·6 to 120·9)	53·0 (50·6 to 55·3)	41·0 (38·2 to 43·9)	18·6 (13·0 to 25·1)	0·9 (0·9 to 0·9)	346·9 (326·0 to 369·5)	161·9 (154·7 to 169·1)	125·2 (116·8 to 134·2)	56·9 (39·6 to 76·7)	2·8 (2·8 to 2·8)
Indonesia*	2018	117·0 (110·3 to 124·8)	57·0 (55·2 to 58·9)	40·8 (37·2 to 44·4)	18·6 (13·1 to 25·5)	0·6 (0·6 to 0·6)	357·8 (337·0 to 381·6)	174·2 (168·7 to 180·1)	124·8 (113·8 to 135·6)	57·0 (40·1 to 77·9)	1·8 (1·8 to 1·8)
Indonesia*	2019	122·0 (114·7 to 130·6)	59·6 (57·2 to 62·2)	42·1 (38·4 to 45·8)	19·7 (13·8 to 27·1)	0·5 (0·5 to 0·6)	372·9 (350·7 to 399·3)	182·2 (174·7 to 190·2)	128·7 (117·4 to 139·9)	60·3 (42·1 to 82·7)	1·7 (1·7 to 1·7)

Indonesia*	2020	139·3 (132·2 to 147·8)	78·2 (75·7 to 80·8)	40·9 (37·3 to 44·4)	19·6 (13·7 to 26·8)	0·7 (0·7 to 0·7)	426·0 (404·1 to 452·0)	239·0 (231·5 to 246·9)	125·0 (114·2 to 135·8)	59·9 (41·8 to 82·1)	2·1 (2·1 to 2·1)
Indonesia*	2021	126·5 (119·0 to 135·6)	65·0 (61·6 to 68·6)	40·9 (37·4 to 44·4)	20·0 (14·0 to 27·5)	0·6 (0·6 to 0·6)	386·7 (363·6 to 414·4)	198·7 (188·4 to 209·8)	125·0 (114·2 to 135·9)	61·1 (42·7 to 84·0)	1·8 (1·8 to 1·9)
Indonesia*	2022	125·3 (117·1 to 134·9)	62·5 (58·2 to 66·5)	41·6 (38·0 to 45·3)	20·8 (14·5 to 28·5)	0·5 (0·4 to 0·5)	383·0 (358·0 to 412·4)	191·0 (178·1 to 203·2)	127·2 (116·1 to 138·4)	63·5 (44·3 to 87·0)	1·4 (1·3 to 1·5)
Indonesia*	2023	129·3 (120·7 to 139·4)	65·0 (60·2 to 69·2)	42·3 (38·6 to 46·1)	21·6 (15·0 to 29·8)	0·5 (0·4 to 0·5)	395·3 (368·8 to 426·1)	198·6 (183·9 to 211·6)	129·4 (118·0 to 140·8)	66·0 (45·9 to 91·0)	1·4 (1·3 to 1·5)
Indonesia*	2024	133·7 (124·5 to 144·3)	67·7 (62·6 to 72·2)	43·1 (39·2 to 46·8)	22·5 (15·6 to 30·9)	0·5 (0·4 to 0·5)	408·7 (380·6 to 441·2)	206·9 (191·4 to 220·7)	131·6 (120·0 to 143·1)	68·8 (47·7 to 94·5)	1·4 (1·3 to 1·5)
Indonesia*	2025	138·1 (128·3 to 149·1)	70·3 (64·8 to 75·4)	43·8 (39·9 to 47·7)	23·5 (16·3 to 32·3)	0·5 (0·4 to 0·5)	422·0 (392·2 to 455·9)	214·9 (198·2 to 230·5)	133·9 (121·9 to 145·7)	71·8 (49·9 to 98·6)	1·4 (1·3 to 1·5)
Indonesia*	2026	142·8 (132·3 to 154·7)	73·3 (67·3 to 78·9)	44·5 (40·5 to 48·5)	24·5 (17·0 to 33·9)	0·5 (0·4 to 0·5)	436·5 (404·5 to 473·0)	223·9 (205·8 to 241·1)	136·1 (124·0 to 148·2)	75·0 (52·0 to 103·5)	1·4 (1·3 to 1·5)
Indonesia*	2027	147·3 (136·3 to 160·1)	76·0 (69·4 to 82·3)	45·3 (41·2 to 49·2)	25·6 (17·8 to 35·5)	0·5 (0·4 to 0·5)	450·4 (416·5 to 489·3)	232·4 (212·0 to 251·5)	138·3 (126·0 to 150·4)	78·3 (54·3 to 108·5)	1·4 (1·3 to 1·5)
Indonesia*	2028	152·0 (140·3 to 165·2)	78·8 (71·5 to 85·9)	46·0 (41·9 to 49·9)	26·7 (18·5 to 37·1)	0·5 (0·4 to 0·5)	464·6 (428·8 to 505·1)	241·0 (218·7 to 262·5)	140·5 (127·9 to 152·5)	81·7 (56·5 to 113·4)	1·4 (1·3 to 1·5)
Indonesia*	2029	157·0 (144·4 to 171·2)	82·0 (73·9 to 89·9)	46·7 (42·5 to 50·7)	27·9 (19·3 to 38·7)	0·5 (0·4 to 0·5)	480·0 (441·5 to 523·5)	250·8 (226·0 to 274·9)	142·6 (129·9 to 154·9)	85·2 (59·1 to 118·2)	1·4 (1·3 to 1·6)
Indonesia*	2030	162·5 (149·3 to 177·2)	85·6 (76·9 to 94·4)	47·4 (43·1 to 51·5)	29·1 (20·2 to 40·4)	0·5 (0·4 to 0·5)	496·6 (456·4 to 541·7)	261·5 (235·1 to 288·6)	144·8 (131·8 to 157·3)	88·9 (61·6 to 123·5)	1·4 (1·3 to 1·6)
Indonesia*	2031	167·7 (153·9 to 183·5)	88·8 (79·4 to 98·3)	48·0 (43·7 to 52·2)	30·4 (21·0 to 42·1)	0·5 (0·4 to 0·5)	512·6 (470·4 to 561·0)	271·6 (242·8 to 300·5)	146·8 (133·6 to 159·6)	92·8 (64·3 to 128·7)	1·4 (1·3 to 1·6)
Indonesia*	2032	172·8 (158·0 to 189·6)	92·1 (81·5 to 102·9)	48·6 (44·2 to 52·8)	31·7 (21·9 to 44·4)	0·5 (0·4 to 0·5)	528·3 (483·1 to 579·6)	281·4 (249·3 to 314·5)	148·7 (135·2 to 161·5)	96·8 (66·8 to 135·8)	1·4 (1·3 to 1·6)
Indonesia*	2033	177·9 (161·8 to 196·3)	95·2 (83·5 to 107·5)	49·2 (44·8 to 53·4)	33·0 (22·7 to 46·6)	0·5 (0·4 to 0·5)	543·7 (494·5 to 600·0)	290·9 (255·1 to 328·6)	150·5 (136·9 to 163·3)	100·8 (69·4 to 142·6)	1·4 (1·3 to 1·6)

Indonesia*	2034	182·9 (165·3 to 202·4)	98·3 (85·1 to 112·2)	49·8 (45·3 to 54·0)	34·3 (23·6 to 48·4)	0·5 (0·4 to 0·5)	559·1 (505·3 to 618·8)	300·4 (260·1 to 342·8)	152·2 (138·4 to 165·0)	105·0 (72·2 to 147·9)	1·5 (1·3 to 1·6)
Indonesia*	2035	188·0 (169·0 to 209·2)	101·4 (86·3 to 116·8)	50·3 (45·8 to 54·6)	35·7 (24·5 to 50·9)	0·5 (0·4 to 0·5)	574·6 (516·5 to 639·4)	310·0 (263·9 to 357·2)	153·8 (139·9 to 166·9)	109·3 (74·9 to 155·6)	1·5 (1·3 to 1·7)
Indonesia*	2036	193·1 (172·6 to 216·3)	104·6 (89·2 to 121·6)	50·9 (46·2 to 55·2)	37·2 (25·4 to 53·2)	0·5 (0·4 to 0·6)	590·3 (527·5 to 661·3)	319·7 (272·6 to 371·8)	155·5 (141·4 to 168·8)	113·7 (77·7 to 162·6)	1·5 (1·4 to 1·7)
Indonesia*	2037	198·1 (175·9 to 223·1)	107·6 (90·4 to 126·6)	51·4 (46·7 to 55·7)	38·6 (26·3 to 55·5)	0·5 (0·5 to 0·6)	605·7 (537·7 to 681·9)	329·0 (276·3 to 387·2)	157·0 (142·7 to 170·4)	118·1 (80·4 to 169·5)	1·6 (1·4 to 1·7)
Indonesia*	2038	203·3 (179·5 to 230·1)	110·7 (91·0 to 131·3)	51·9 (47·2 to 56·3)	40·2 (27·3 to 58·2)	0·5 (0·5 to 0·6)	621·4 (548·8 to 703·3)	338·5 (278·1 to 401·4)	158·6 (144·2 to 172·0)	122·8 (83·4 to 177·9)	1·6 (1·4 to 1·8)
Indonesia*	2039	208·8 (183·0 to 237·1)	114·1 (93·4 to 136·3)	52·4 (47·7 to 56·9)	41·8 (28·3 to 60·6)	0·5 (0·5 to 0·6)	638·2 (559·4 to 724·7)	348·7 (285·5 to 416·5)	160·2 (145·7 to 173·9)	127·6 (86·4 to 185·3)	1·6 (1·4 to 1·8)
Indonesia*	2040	214·4 (187·2 to 244·6)	117·5 (94·9 to 141·3)	53·0 (48·2 to 57·6)	43·4 (29·2 to 63·4)	0·5 (0·5 to 0·6)	655·4 (572·2 to 747·9)	359·1 (290·1 to 431·9)	161·9 (147·4 to 176·0)	132·7 (89·3 to 193·8)	1·7 (1·5 to 1·9)
Indonesia*	2041	220·3 (191·1 to 253·2)	121·0 (97·0 to 146·8)	53·6 (48·7 to 58·2)	45·2 (30·3 to 66·0)	0·6 (0·5 to 0·6)	673·4 (584·2 to 774·0)	369·9 (296·4 to 448·8)	163·7 (149·0 to 178·0)	138·0 (92·6 to 201·7)	1·7 (1·5 to 1·9)
Indonesia*	2042	226·4 (195·5 to 261·5)	124·7 (100·2 to 152·4)	54·2 (49·3 to 58·9)	47·0 (31·4 to 68·7)	0·6 (0·5 to 0·7)	692·0 (597·6 to 799·3)	381·2 (306·3 to 466·0)	165·6 (150·7 to 180·2)	143·6 (95·9 to 210·1)	1·7 (1·5 to 2·0)
Indonesia*	2043	232·7 (200·1 to 270·4)	128·6 (102·9 to 158·1)	54·8 (49·9 to 59·6)	48·8 (32·4 to 71·9)	0·6 (0·5 to 0·7)	711·5 (611·7 to 826·7)	393·0 (314·7 to 483·4)	167·4 (152·5 to 182·3)	149·3 (99·2 to 219·8)	1·8 (1·5 to 2·0)
Indonesia*	2044	239·4 (205·0 to 278·9)	132·6 (104·7 to 163·7)	55·4 (50·4 to 60·2)	50·8 (33·5 to 74·9)	0·6 (0·5 to 0·7)	731·7 (626·6 to 852·5)	405·3 (320·2 to 500·5)	169·3 (154·2 to 184·2)	155·2 (102·3 to 228·9)	1·8 (1·6 to 2·1)
Indonesia*	2045	246·0 (209·7 to 287·9)	136·6 (106·8 to 169·5)	56·0 (51·0 to 60·9)	52·8 (34·6 to 78·0)	0·6 (0·5 to 0·7)	752·2 (641·0 to 880·2)	417·6 (326·4 to 518·1)	171·3 (155·9 to 186·2)	161·4 (105·8 to 238·6)	1·8 (1·6 to 2·2)
Indonesia*	2046	253·0 (214·3 to 297·2)	140·8 (108·9 to 175·6)	56·7 (51·6 to 61·6)	54·9 (35·9 to 82·1)	0·6 (0·5 to 0·7)	773·5 (655·0 to 908·4)	430·5 (333·0 to 536·9)	173·3 (157·7 to 188·3)	167·8 (109·9 to 250·8)	1·9 (1·6 to 2·2)
Indonesia*	2047	260·2 (219·5 to 306·8)	145·1 (112·7 to 181·2)	57·4 (52·2 to 62·4)	57·0 (37·2 to 85·5)	0·6 (0·5 to 0·7)	795·4 (671·0 to 937·8)	443·6 (344·4 to 553·8)	175·4 (159·6 to 190·7)	174·4 (113·7 to 261·5)	1·9 (1·6 to 2·3)

Indonesia*	2048	267·6 (225·2 to 316·3)	149·6 (115·7 to 187·6)	58·1 (52·8 to 63·2)	59·3 (38·2 to 88·9)	0·6 (0·5 to 0·8)	817·9 (688·5 to 966·9)	457·2 (353·7 to 573·6)	177·6 (161·5 to 193·1)	181·2 (116·8 to 271·8)	1·9 (1·7 to 2·3)
Indonesia*	2049	275·0 (230·7 to 326·3)	154·0 (118·0 to 193·3)	58·8 (53·5 to 64·0)	61·6 (39·4 to 92·6)	0·6 (0·6 to 0·8)	840·7 (705·3 to 997·6)	470·8 (360·6 to 590·8)	179·8 (163·4 to 195·6)	188·2 (120·5 to 283·0)	2·0 (1·7 to 2·4)
Indonesia*	2050	282·7 (236·8 to 336·3)	158·5 (121·5 to 199·4)	59·6 (54·1 to 64·8)	64·0 (40·8 to 96·7)	0·7 (0·6 to 0·8)	864·2 (723·9 to 1028·2)	484·6 (371·4 to 609·5)	182·0 (165·4 to 198·2)	195·5 (124·7 to 295·5)	2·0 (1·7 to 2·4)
India*	1995	26·6 (22·4 to 31·2)	4·9 (4·0 to 5·9)	19·8 (15·6 to 24·4)	1·4 (1·0 to 1·9)	0·5 (0·5 to 0·5)	88·9 (74·9 to 104·6)	16·5 (13·4 to 19·9)	66·1 (52·3 to 81·6)	4·7 (3·2 to 6·4)	1·7 (1·7 to 1·7)
India*	1996	27·6 (23·3 to 32·5)	5·1 (4·1 to 6·1)	20·4 (16·2 to 25·1)	1·4 (1·0 to 2·0)	0·7 (0·7 to 0·7)	92·6 (78·0 to 108·8)	17·1 (13·9 to 20·6)	68·2 (54·2 to 84·1)	4·8 (3·3 to 6·7)	2·4 (2·4 to 2·4)
India*	1997	28·6 (24·2 to 33·5)	5·4 (4·4 to 6·5)	21·2 (17·1 to 26·2)	1·5 (1·0 to 2·1)	0·5 (0·5 to 0·5)	95·7 (81·1 to 112·3)	18·1 (14·7 to 21·8)	71·0 (57·2 to 87·8)	5·0 (3·4 to 7·1)	1·5 (1·5 to 1·5)
India*	1998	29·7 (25·3 to 34·5)	5·8 (4·7 to 6·9)	21·9 (17·6 to 27·0)	1·6 (1·0 to 2·3)	0·4 (0·4 to 0·4)	99·4 (84·8 to 115·4)	19·3 (15·8 to 23·1)	73·4 (58·9 to 90·5)	5·3 (3·5 to 7·6)	1·5 (1·5 to 1·5)
India*	1999	30·6 (26·2 to 35·4)	6·0 (4·9 to 7·3)	22·4 (18·2 to 27·2)	1·6 (1·1 to 2·4)	0·5 (0·5 to 0·5)	102·3 (87·7 to 118·4)	20·2 (16·4 to 24·4)	75·0 (61·0 to 91·0)	5·5 (3·7 to 7·9)	1·7 (1·7 to 1·7)
India*	2000	31·5 (27·1 to 36·3)	6·1 (5·0 to 7·4)	23·0 (18·9 to 27·6)	1·6 (1·1 to 2·4)	0·7 (0·7 to 0·7)	105·3 (90·8 to 121·5)	20·5 (16·8 to 24·9)	77·1 (63·2 to 92·4)	5·5 (3·7 to 7·9)	2·3 (2·3 to 2·3)
India*	2001	32·7 (28·3 to 38·1)	6·3 (5·1 to 7·6)	24·1 (19·8 to 29·4)	1·8 (1·2 to 2·5)	0·6 (0·6 to 0·6)	109·7 (94·7 to 127·5)	21·1 (17·2 to 25·5)	80·6 (66·5 to 98·4)	5·9 (4·0 to 8·4)	2·1 (2·1 to 2·1)
India*	2002	33·8 (29·3 to 39·0)	6·4 (5·2 to 7·7)	24·7 (20·5 to 29·7)	2·0 (1·3 to 2·8)	0·7 (0·7 to 0·7)	113·3 (98·1 to 130·7)	21·5 (17·5 to 25·9)	82·8 (68·7 to 99·5)	6·7 (4·5 to 9·4)	2·4 (2·4 to 2·4)
India*	2003	34·9 (30·2 to 40·1)	6·7 (5·5 to 8·1)	25·5 (21·2 to 30·6)	2·2 (1·5 to 3·1)	0·5 (0·5 to 0·5)	116·7 (101·2 to 134·2)	22·5 (18·3 to 27·2)	85·3 (71·0 to 102·5)	7·3 (4·9 to 10·3)	1·7 (1·7 to 1·7)
India*	2004	35·6 (31·2 to 41·0)	6·8 (5·6 to 8·2)	25·8 (21·5 to 31·2)	2·3 (1·6 to 3·2)	0·7 (0·7 to 0·7)	119·2 (104·3 to 137·2)	22·8 (18·6 to 27·5)	86·5 (72·1 to 104·4)	7·7 (5·2 to 10·8)	2·3 (2·3 to 2·3)
India*	2005	36·7 (31·9 to 42·5)	7·2 (6·0 to 8·8)	26·5 (22·0 to 32·0)	2·2 (1·5 to 3·1)	0·7 (0·7 to 0·7)	122·8 (106·7 to 142·5)	24·2 (20·0 to 29·4)	88·9 (73·6 to 107·0)	7·3 (4·9 to 10·3)	2·4 (2·4 to 2·4)

India*	2006	38·0 (32·9 to 43·9)	7·8 (6·4 to 9·4)	27·3 (22·4 to 33·1)	2·3 (1·6 to 3·3)	0·5 (0·5 to 0·5)	127·3 (110·0 to 146·9)	26·3 (21·5 to 31·6)	91·6 (74·9 to 110·7)	7·8 (5·3 to 10·9)	1·7 (1·7 to 1·7)
India*	2007	39·7 (34·3 to 45·8)	8·5 (6·9 to 10·1)	28·0 (22·9 to 33·9)	2·6 (1·7 to 3·7)	0·7 (0·7 to 0·7)	132·9 (114·8 to 153·5)	28·3 (23·0 to 33·8)	93·7 (76·8 to 113·7)	8·6 (5·8 to 12·3)	2·2 (2·2 to 2·2)
India*	2008	41·5 (35·8 to 47·5)	9·4 (7·7 to 11·2)	28·7 (23·5 to 34·4)	2·7 (1·8 to 3·9)	0·7 (0·7 to 0·7)	138·9 (120·0 to 159·1)	31·5 (25·9 to 37·4)	96·0 (78·6 to 115·2)	9·1 (6·1 to 13·1)	2·3 (2·3 to 2·3)
India*	2009	43·6 (38·0 to 49·9)	10·4 (8·7 to 12·3)	29·4 (24·1 to 35·5)	3·0 (2·0 to 4·2)	0·8 (0·8 to 0·8)	146·0 (127·3 to 167·0)	35·0 (29·1 to 41·2)	98·6 (80·8 to 118·9)	10·0 (6·7 to 14·0)	2·5 (2·5 to 2·5)
India*	2010	45·8 (39·8 to 52·4)	11·4 (9·5 to 13·4)	30·3 (25·1 to 36·4)	3·3 (2·3 to 4·6)	0·8 (0·8 to 0·8)	153·2 (133·4 to 175·4)	38·1 (31·9 to 44·9)	101·4 (84·0 to 121·9)	11·2 (7·6 to 15·6)	2·5 (2·5 to 2·5)
India*	2011	48·5 (42·7 to 55·1)	12·4 (10·4 to 14·5)	31·6 (26·6 to 37·8)	3·7 (2·5 to 5·2)	0·8 (0·8 to 0·8)	162·6 (142·9 to 184·6)	41·4 (35·0 to 48·5)	106·0 (89·2 to 126·6)	12·4 (8·5 to 17·5)	2·7 (2·7 to 2·7)
India*	2012	51·3 (45·7 to 57·6)	12·9 (11·2 to 15·1)	33·7 (28·8 to 39·3)	4·0 (2·7 to 5·7)	0·6 (0·6 to 0·6)	171·8 (153·1 to 193·0)	43·4 (37·4 to 50·4)	112·8 (96·4 to 131·6)	13·6 (9·2 to 19·1)	2·2 (2·2 to 2·2)
India*	2013	55·3 (49·8 to 61·0)	13·4 (11·7 to 15·4)	36·7 (31·7 to 42·0)	4·5 (3·0 to 6·2)	0·8 (0·8 to 0·8)	185·1 (166·8 to 204·4)	44·9 (39·1 to 51·5)	122·7 (106·3 to 140·6)	14·9 (10·0 to 20·8)	2·6 (2·6 to 2·6)
India*	2014	58·0 (52·5 to 63·9)	14·1 (12·5 to 15·8)	38·4 (33·8 to 43·7)	5·0 (3·3 to 6·9)	0·5 (0·5 to 0·5)	194·1 (175·7 to 214·1)	47·2 (41·8 to 53·0)	128·7 (113·0 to 146·4)	16·6 (11·1 to 23·2)	1·5 (1·5 to 1·5)
India*	2015	61·8 (56·2 to 68·1)	15·5 (13·7 to 17·4)	40·1 (35·1 to 45·3)	5·5 (3·7 to 7·6)	0·6 (0·6 to 0·6)	207·0 (188·3 to 227·9)	52·0 (45·9 to 58·3)	134·4 (117·6 to 151·8)	18·5 (12·4 to 25·6)	2·1 (2·1 to 2·1)
India*	2016	65·3 (59·2 to 72·0)	17·0 (15·0 to 19·2)	41·7 (36·3 to 47·8)	6·1 (4·1 to 8·5)	0·5 (0·5 to 0·5)	218·8 (198·1 to 241·1)	56·9 (50·1 to 64·2)	139·8 (121·7 to 160·1)	20·4 (13·7 to 28·6)	1·8 (1·8 to 1·8)
India*	2017	68·9 (61·5 to 77·2)	18·2 (15·8 to 21·0)	43·5 (36·9 to 50·7)	6·6 (4·5 to 9·3)	0·6 (0·6 to 0·6)	230·8 (205·9 to 258·4)	61·0 (53·1 to 70·2)	145·5 (123·4 to 169·7)	22·2 (15·2 to 31·3)	2·0 (2·0 to 2·0)
India*	2018	72·5 (64·1 to 82·2)	19·5 (16·7 to 22·7)	45·4 (37·6 to 53·7)	7·1 (4·9 to 9·9)	0·5 (0·5 to 0·5)	242·7 (214·5 to 275·1)	65·3 (55·9 to 75·9)	152·0 (126·0 to 179·8)	23·8 (16·3 to 33·2)	1·5 (1·5 to 1·5)
India*	2019	75·5 (66·7 to 85·5)	20·9 (17·9 to 24·4)	46·6 (38·6 to 55·1)	7·5 (5·1 to 10·4)	0·4 (0·4 to 0·5)	252·7 (223·5 to 286·2)	70·1 (59·9 to 81·7)	156·2 (129·4 to 184·7)	25·0 (17·1 to 34·9)	1·5 (1·5 to 1·5)

India*	2020	77·4 (69·0 to 87·2)	24·3 (21·3 to 27·7)	45·2 (37·5 to 53·4)	7·4 (5·0 to 10·4)	0·6 (0·5 to 0·6)	259·3 (230·9 to 292·1)	81·3 (71·3 to 92·9)	151·4 (125·5 to 179·0)	24·8 (16·8 to 34·7)	1·8 (1·8 to 1·9)
India*	2021	76·3 (67·6 to 86·2)	23·1 (19·7 to 27·0)	45·2 (37·5 to 53·4)	7·6 (5·1 to 10·6)	0·5 (0·5 to 0·5)	255·5 (226·4 to 288·7)	77·3 (66·1 to 90·5)	151·3 (125·6 to 178·9)	25·4 (17·2 to 35·4)	1·6 (1·5 to 1·7)
India*	2022	75·8 (67·0 to 85·8)	21·4 (18·3 to 25·1)	46·1 (38·3 to 54·5)	7·9 (5·3 to 10·9)	0·4 (0·3 to 0·4)	253·7 (224·4 to 287·3)	71·7 (61·2 to 84·1)	154·3 (128·2 to 182·5)	26·5 (17·9 to 36·6)	1·2 (1·1 to 1·3)
India*	2023	77·7 (68·7 to 87·9)	22·1 (18·8 to 25·9)	47·0 (39·0 to 55·6)	8·2 (5·6 to 11·4)	0·4 (0·3 to 0·4)	260·1 (230·2 to 294·4)	73·9 (62·8 to 86·7)	157·4 (130·8 to 186·2)	27·6 (18·6 to 38·3)	1·2 (1·1 to 1·3)
India*	2024	79·6 (70·4 to 90·1)	22·8 (19·4 to 26·8)	47·9 (39·8 to 56·7)	8·6 (5·8 to 11·9)	0·3 (0·3 to 0·4)	266·6 (235·9 to 301·7)	76·2 (64·9 to 89·7)	160·5 (133·2 to 189·8)	28·8 (19·4 to 40·0)	1·2 (1·1 to 1·3)
India*	2025	81·7 (72·3 to 92·4)	23·5 (20·0 to 27·7)	48·9 (40·6 to 57·8)	9·0 (6·0 to 12·4)	0·3 (0·3 to 0·4)	273·5 (242·0 to 309·3)	78·6 (67·0 to 92·8)	163·7 (135·9 to 193·5)	30·0 (20·2 to 41·6)	1·1 (1·0 to 1·3)
India*	2026	83·8 (74·2 to 94·8)	24·2 (20·7 to 28·5)	49·9 (41·4 to 58·9)	9·4 (6·3 to 13·0)	0·3 (0·3 to 0·4)	280·5 (248·4 to 317·5)	81·1 (69·2 to 95·4)	167·0 (138·7 to 197·3)	31·4 (21·1 to 43·6)	1·1 (1·0 to 1·2)
India*	2027	85·9 (76·1 to 97·2)	24·9 (21·2 to 29·3)	50·8 (42·2 to 60·1)	9·8 (6·6 to 13·7)	0·3 (0·3 to 0·4)	287·7 (254·7 to 325·5)	83·5 (71·0 to 98·2)	170·2 (141·4 to 201·3)	32·8 (22·1 to 45·9)	1·1 (1·0 to 1·2)
India*	2028	88·1 (78·0 to 99·7)	25·7 (21·9 to 30·3)	51·8 (43·0 to 61·3)	10·3 (6·9 to 14·3)	0·3 (0·3 to 0·4)	295·2 (261·3 to 333·8)	86·2 (73·3 to 101·3)	173·6 (144·1 to 205·3)	34·4 (23·1 to 48·0)	1·1 (1·0 to 1·2)
India*	2029	90·5 (80·1 to 102·3)	26·6 (22·6 to 31·3)	52·9 (43·9 to 62·5)	10·7 (7·2 to 15·0)	0·3 (0·3 to 0·4)	303·0 (268·2 to 342·5)	89·0 (75·6 to 104·9)	177·0 (147·0 to 209·4)	36·0 (24·2 to 50·2)	1·1 (0·9 to 1·2)
India*	2030	92·9 (82·2 to 104·9)	27·4 (23·1 to 32·3)	53·9 (44·8 to 63·8)	11·2 (7·6 to 15·7)	0·3 (0·3 to 0·4)	311·0 (275·4 to 351·4)	91·9 (77·4 to 108·1)	180·5 (149·9 to 213·5)	37·7 (25·4 to 52·7)	1·0 (0·9 to 1·2)
India*	2031	95·3 (84·4 to 107·5)	28·3 (23·9 to 33·3)	54·9 (45·6 to 65·0)	11·8 (7·9 to 16·4)	0·3 (0·3 to 0·3)	319·3 (282·6 to 360·0)	94·9 (80·0 to 111·7)	184·0 (152·6 to 217·7)	39·4 (26·6 to 55·0)	1·0 (0·9 to 1·2)
India*	2032	97·9 (86·7 to 110·4)	29·3 (24·6 to 34·6)	56·0 (46·4 to 66·2)	12·3 (8·3 to 17·3)	0·3 (0·3 to 0·3)	327·7 (290·2 to 369·8)	98·0 (82·5 to 115·9)	187·5 (155·4 to 221·7)	41·3 (27·9 to 57·9)	1·0 (0·9 to 1·1)
India*	2033	100·5 (88·9 to 113·4)	30·2 (25·5 to 35·8)	57·0 (47·3 to 67·4)	12·9 (8·7 to 18·1)	0·3 (0·3 to 0·3)	336·4 (297·6 to 379·8)	101·3 (85·5 to 120·0)	190·9 (158·2 to 225·8)	43·2 (29·2 to 60·8)	1·0 (0·8 to 1·1)

India*	2034	103·1 (91·2 to 116·5)	31·3 (26·5 to 37·2)	58·1 (48·1 to 68·7)	13·5 (9·1 to 19·1)	0·3 (0·2 to 0·3)	345·4 (305·5 to 390·0)	104·7 (88·9 to 124·6)	194·5 (161·0 to 229·9)	45·2 (30·5 to 64·1)	1·0 (0·8 to 1·1)
India*	2035	105·9 (93·7 to 119·6)	32·3 (27·4 to 38·4)	59·1 (48·9 to 69·9)	14·1 (9·6 to 20·1)	0·3 (0·2 to 0·3)	354·5 (313·7 to 400·5)	108·3 (91·7 to 128·6)	198·0 (163·8 to 234·0)	47·4 (32·0 to 67·4)	0·9 (0·8 to 1·1)
India*	2036	108·6 (96·1 to 122·7)	33·4 (28·1 to 39·6)	60·1 (49·7 to 71·1)	14·8 (10·0 to 21·1)	0·3 (0·2 to 0·3)	363·8 (321·7 to 411·0)	111·9 (94·2 to 132·6)	201·4 (166·6 to 237·9)	49·6 (33·4 to 70·7)	0·9 (0·8 to 1·1)
India*	2037	111·3 (98·4 to 125·8)	34·5 (29·0 to 40·8)	61·1 (50·5 to 72·1)	15·5 (10·4 to 22·1)	0·3 (0·2 to 0·3)	372·6 (329·3 to 421·2)	115·4 (97·0 to 136·6)	204·5 (169·0 to 241·5)	51·8 (34·8 to 74·0)	0·9 (0·8 to 1·1)
India*	2038	113·9 (100·6 to 128·9)	35·6 (29·8 to 42·2)	62·0 (51·2 to 73·2)	16·1 (10·8 to 23·1)	0·3 (0·2 to 0·3)	381·6 (336·9 to 431·6)	119·1 (99·7 to 141·3)	207·5 (171·4 to 245·2)	54·1 (36·2 to 77·3)	0·9 (0·8 to 1·1)
India*	2039	116·7 (103·1 to 132·2)	36·7 (30·7 to 43·6)	62·9 (51·9 to 74·3)	16·9 (11·2 to 24·1)	0·3 (0·2 to 0·3)	390·8 (345·1 to 442·6)	122·8 (103·0 to 145·9)	210·6 (173·9 to 248·9)	56·4 (37·5 to 80·8)	0·9 (0·8 to 1·1)
India*	2040	119·5 (105·5 to 135·6)	37·8 (31·7 to 44·9)	63·8 (52·7 to 75·5)	17·6 (11·5 to 25·2)	0·3 (0·2 to 0·3)	400·2 (353·2 to 454·0)	126·7 (106·2 to 150·4)	213·7 (176·4 to 252·7)	58·9 (38·7 to 84·3)	0·9 (0·8 to 1·1)
India*	2041	122·4 (107·9 to 138·7)	39·0 (32·6 to 46·4)	64·7 (53·4 to 76·5)	18·4 (12·1 to 26·3)	0·3 (0·2 to 0·3)	409·8 (361·3 to 464·3)	130·6 (109·1 to 155·5)	216·7 (178·9 to 256·3)	61·5 (40·4 to 88·0)	0·9 (0·8 to 1·1)
India*	2042	125·3 (110·4 to 142·1)	40·2 (33·5 to 48·0)	65·6 (54·2 to 77·6)	19·2 (12·5 to 27·4)	0·3 (0·2 to 0·3)	419·5 (369·7 to 475·9)	134·7 (112·3 to 160·9)	219·7 (181·5 to 260·0)	64·2 (41·8 to 91·9)	0·9 (0·8 to 1·1)
India*	2043	128·2 (112·9 to 145·6)	41·4 (34·6 to 49·6)	66·5 (55·0 to 78·7)	20·0 (12·9 to 28·7)	0·3 (0·2 to 0·3)	429·4 (378·0 to 487·6)	138·8 (115·9 to 166·1)	222·7 (184·0 to 263·6)	67·0 (43·2 to 96·1)	0·9 (0·8 to 1·1)
India*	2044	131·3 (115·5 to 149·2)	42·7 (35·8 to 51·0)	67·4 (55·7 to 79·7)	20·9 (13·4 to 30·0)	0·3 (0·2 to 0·3)	439·7 (386·9 to 499·5)	143·1 (119·9 to 170·7)	225·7 (186·6 to 267·0)	69·9 (44·8 to 100·4)	0·9 (0·8 to 1·1)
India*	2045	134·4 (118·2 to 152·8)	44·0 (36·7 to 52·6)	68·3 (56·5 to 80·7)	21·8 (13·9 to 31·5)	0·3 (0·2 to 0·3)	450·0 (395·7 to 511·7)	147·5 (123·0 to 176·3)	228·7 (189·1 to 270·3)	72·9 (46·6 to 105·6)	0·9 (0·8 to 1·2)
India*	2046	137·5 (120·8 to 156·6)	45·4 (37·8 to 54·5)	69·1 (57·2 to 81·7)	22·7 (14·5 to 33·0)	0·3 (0·2 to 0·3)	460·4 (404·5 to 524·2)	151·9 (126·5 to 182·4)	231·5 (191·5 to 273·5)	76·1 (48·5 to 110·5)	1·0 (0·8 to 1·2)
India*	2047	140·7 (123·5 to 160·4)	46·7 (38·8 to 56·3)	70·0 (57·9 to 82·6)	23·7 (15·0 to 34·7)	0·3 (0·2 to 0·4)	471·1 (413·6 to 537·2)	156·4 (130·0 to 188·7)	234·4 (194·0 to 276·7)	79·3 (50·4 to 116·2)	1·0 (0·8 to 1·2)

India*	2048	143·9 (126·2 to 164·2)	48·1 (39·9 to 58·2)	70·8 (58·6 to 83·5)	24·7 (15·6 to 36·5)	0·3 (0·2 to 0·3)	481·8 (422·6 to 549·9)	161·0 (133·5 to 194·9)	237·1 (196·3 to 279·7)	82·7 (52·2 to 122·2)	1·0 (0·8 to 1·2)
India*	2049	147·1 (128·9 to 168·3)	49·5 (40·8 to 59·9)	71·6 (59·3 to 84·4)	25·7 (16·0 to 38·1)	0·3 (0·2 to 0·4)	492·5 (431·6 to 563·6)	165·7 (136·7 to 200·6)	239·7 (198·5 to 282·6)	86·1 (53·7 to 127·6)	1·0 (0·8 to 1·2)
India*	2050	150·4 (131·6 to 172·6)	50·9 (42·0 to 61·8)	72·4 (59·9 to 85·4)	26·8 (16·6 to 39·8)	0·3 (0·2 to 0·4)	503·5 (440·6 to 577·9)	170·5 (140·6 to 206·8)	242·3 (200·7 to 285·9)	89·7 (55·6 to 133·4)	1·0 (0·8 to 1·2)
Ireland	1995	1772·2 (1628·1 to 1938·7)	1335·0 (1198·8 to 1493·0)	186·6 (160·6 to 217·1)	250·7 (211·8 to 295·9)	0·0 (0·0 to 0·0)	1987·6 (1826·0 to 2174·2)	1497·2 (1344·4 to 1674·4)	209·2 (180·1 to 243·5)	281·1 (237·5 to 331·9)	0·0 (0·0 to 0·0)
Ireland	1996	1882·5 (1739·3 to 2034·7)	1424·7 (1297·4 to 1574·9)	198·3 (171·4 to 226·9)	259·5 (225·8 to 298·0)	0·0 (0·0 to 0·0)	2111·2 (1950·6 to 2281·9)	1597·8 (1455·0 to 1766·2)	222·4 (192·2 to 254·5)	291·0 (253·2 to 334·2)	0·0 (0·0 to 0·0)
Ireland	1997	2042·0 (1903·6 to 2194·9)	1561·8 (1432·5 to 1706·9)	213·0 (187·1 to 241·6)	267·2 (234·0 to 302·6)	0·0 (0·0 to 0·0)	2290·1 (2134·9 to 2461·5)	1751·5 (1606·5 to 1914·3)	238·8 (209·9 to 271·0)	299·7 (262·5 to 339·4)	0·0 (0·0 to 0·0)
Ireland	1998	2201·6 (2068·9 to 2342·3)	1697·3 (1572·2 to 1841·8)	229·2 (203·6 to 257·9)	275·1 (244·3 to 309·0)	0·0 (0·0 to 0·0)	2469·1 (2320·2 to 2626·9)	1903·5 (1763·2 to 2065·5)	257·0 (228·3 to 289·2)	308·5 (274·0 to 346·5)	0·0 (0·0 to 0·0)
Ireland	1999	2408·3 (2268·3 to 2559·6)	1869·8 (1737·6 to 2013·6)	257·9 (232·3 to 286·6)	280·6 (249·7 to 312·8)	0·0 (0·0 to 0·0)	2700·9 (2543·8 to 2870·6)	2096·9 (1948·8 to 2258·2)	289·3 (260·5 to 321·5)	314·7 (280·0 to 350·8)	0·0 (0·0 to 0·0)
Ireland	2000	2639·8 (2499·9 to 2781·1)	2054·8 (1923·5 to 2193·3)	295·6 (267·7 to 326·7)	289·4 (259·3 to 322·4)	0·0 (0·0 to 0·0)	2960·6 (2803·7 to 3119·0)	2304·5 (2157·2 to 2459·8)	331·6 (300·2 to 366·4)	324·6 (290·8 to 361·6)	0·0 (0·0 to 0·0)
Ireland	2001	2981·1 (2831·8 to 3131·4)	2332·9 (2196·0 to 2478·8)	332·9 (305·8 to 364·4)	315·3 (285·5 to 348·5)	0·0 (0·0 to 0·0)	3343·3 (3175·9 to 3511·8)	2616·3 (2462·8 to 2780·0)	373·4 (342·9 to 408·7)	353·6 (320·1 to 390·9)	0·0 (0·0 to 0·0)
Ireland	2002	3281·4 (3132·5 to 3456·1)	2580·5 (2432·1 to 2742·2)	376·7 (348·7 to 407·9)	324·2 (294·0 to 359·0)	0·0 (0·0 to 0·0)	3680·1 (3513·1 to 3876·0)	2894·0 (2727·6 to 3075·3)	422·5 (391·0 to 457·5)	363·6 (329·7 to 402·6)	0·0 (0·0 to 0·0)
Ireland	2003	3480·7 (3327·7 to 3645·0)	2742·2 (2591·7 to 2905·0)	431·3 (402·9 to 462·5)	307·2 (276·6 to 340·4)	0·0 (0·0 to 0·0)	3903·6 (3732·0 to 4087·9)	3075·4 (2906·6 to 3258·0)	483·7 (451·9 to 518·7)	344·6 (310·2 to 381·8)	0·0 (0·0 to 0·0)
Ireland	2004	3756·2 (3581·3 to 3928·7)	2959·2 (2794·1 to 3127·6)	478·5 (449·7 to 511·4)	318·5 (285·7 to 351·1)	0·0 (0·0 to 0·0)	4212·6 (4016·4 to 4406·0)	3318·7 (3133·5 to 3507·6)	536·7 (504·3 to 573·6)	357·2 (320·4 to 393·8)	0·0 (0·0 to 0·0)
Ireland	2005	4002·8 (3832·5 to 4165·6)	3154·5 (2991·1 to 3314·7)	524·7 (493·9 to 560·9)	323·6 (291·9 to 355·1)	0·0 (0·0 to 0·0)	4489·1 (4298·1 to 4671·7)	3537·7 (3354·6 to 3717·4)	588·5 (553·9 to 629·0)	362·9 (327·3 to 398·3)	0·0 (0·0 to 0·0)

Ireland	2006	4210·8 (4039·5 to 4373·1)	3313·8 (3151·3 to 3472·3)	548·9 (517·4 to 582·5)	348·1 (317·1 to 382·4)	0·0 (0·0 to 0·0)	4722·4 (4530·3 to 4904·4)	3716·4 (3534·2 to 3894·2)	615·6 (580·3 to 653·2)	390·4 (355·6 to 428·9)	0·0 (0·0 to 0·0)
Ireland	2007	4528·9 (4356·2 to 4700·4)	3566·9 (3393·7 to 3736·9)	564·9 (533·0 to 599·2)	397·1 (363·6 to 432·7)	0·0 (0·0 to 0·0)	5079·2 (4885·5 to 5271·5)	4000·3 (3806·0 to 4190·9)	633·5 (597·8 to 672·0)	445·4 (407·8 to 485·2)	0·0 (0·0 to 0·0)
Ireland	2008	4822·0 (4660·7 to 4997·7)	3775·6 (3620·1 to 3946·6)	601·8 (570·3 to 636·6)	444·7 (410·8 to 481·2)	0·0 (0·0 to 0·0)	5407·9 (5226·9 to 5604·9)	4234·3 (4059·9 to 4426·1)	674·9 (639·6 to 714·0)	498·7 (460·7 to 539·7)	0·0 (0·0 to 0·0)
Ireland	2009	4972·3 (4814·6 to 5137·5)	3816·7 (3656·8 to 3972·1)	647·9 (614·2 to 683·2)	507·7 (473·1 to 542·4)	0·0 (0·0 to 0·0)	5576·4 (5399·5 to 5761·6)	4280·4 (4101·0 to 4454·7)	726·6 (688·8 to 766·2)	569·3 (530·6 to 608·3)	0·0 (0·0 to 0·0)
Ireland	2010	5877·3 (5682·2 to 6077·2)	4518·3 (4323·7 to 4705·8)	742·6 (707·0 to 781·0)	616·4 (579·9 to 656·4)	0·0 (0·0 to 0·0)	6591·3 (6372·5 to 6815·5)	5067·2 (4849·0 to 5277·6)	832·8 (792·9 to 875·9)	691·3 (650·3 to 736·1)	0·0 (0·0 to 0·0)
Ireland	2011	5180·0 (5009·4 to 5356·2)	3770·6 (3608·9 to 3940·4)	726·0 (691·1 to 761·6)	683·4 (643·0 to 727·8)	0·0 (0·0 to 0·0)	5809·4 (5618·0 to 6007·0)	4228·8 (4047·3 to 4419·2)	814·2 (775·1 to 854·2)	766·5 (721·1 to 816·2)	0·0 (0·0 to 0·0)
Ireland	2012	5116·8 (4947·1 to 5286·3)	3649·9 (3493·2 to 3818·0)	730·3 (698·4 to 764·9)	736·5 (694·0 to 777·7)	0·0 (0·0 to 0·0)	5738·5 (5548·2 to 5928·6)	4093·4 (3917·6 to 4281·9)	819·0 (783·2 to 857·8)	826·0 (778·3 to 872·2)	0·0 (0·0 to 0·0)
Ireland	2013	5095·6 (4920·1 to 5278·3)	3618·6 (3453·0 to 3783·5)	722·6 (689·3 to 757·6)	754·5 (714·1 to 797·8)	0·0 (0·0 to 0·0)	5714·7 (5517·9 to 5919·6)	4058·2 (3872·6 to 4243·1)	810·3 (773·1 to 849·6)	846·1 (800·8 to 894·8)	0·0 (0·0 to 0·0)
Ireland	2014	5098·7 (4919·3 to 5287·3)	3638·5 (3474·0 to 3816·7)	701·2 (667·0 to 736·3)	759·0 (717·8 to 798·1)	0·0 (0·0 to 0·0)	5718·2 (5517·0 to 5929·7)	4080·6 (3896·1 to 4280·4)	786·4 (748·1 to 825·7)	851·2 (805·0 to 895·0)	0·0 (0·0 to 0·0)
Ireland	2015	4965·3 (4786·1 to 5140·8)	3565·9 (3405·6 to 3737·8)	664·4 (631·7 to 700·6)	735·1 (696·9 to 774·1)	0·0 (0·0 to 0·0)	5568·6 (5367·6 to 5765·4)	3999·1 (3819·3 to 4191·9)	745·1 (708·5 to 785·7)	824·4 (781·6 to 868·1)	0·0 (0·0 to 0·0)
Ireland	2016	5036·1 (4869·9 to 5215·3)	3646·2 (3494·7 to 3815·3)	647·8 (615·2 to 680·8)	742·2 (701·5 to 780·4)	0·0 (0·0 to 0·0)	5648·0 (5461·5 to 5848·9)	4089·2 (3919·2 to 4278·9)	726·5 (690·0 to 763·5)	832·4 (786·7 to 875·2)	0·0 (0·0 to 0·0)
Ireland	2017	5168·4 (4978·1 to 5352·6)	3767·7 (3587·9 to 3940·9)	648·1 (615·0 to 684·9)	752·6 (712·0 to 792·4)	0·0 (0·0 to 0·0)	5796·3 (5582·9 to 6002·9)	4225·4 (4023·8 to 4419·7)	726·8 (689·7 to 768·1)	844·0 (798·6 to 888·6)	0·0 (0·0 to 0·0)
Ireland	2018	5471·0 (5226·2 to 5712·8)	4029·3 (3797·6 to 4270·4)	666·2 (621·5 to 713·9)	775·5 (723·5 to 834·5)	0·0 (0·0 to 0·0)	6135·7 (5861·2 to 6406·9)	4518·8 (4259·0 to 4789·3)	747·2 (697·1 to 800·6)	869·7 (811·4 to 935·9)	0·0 (0·0 to 0·0)
Ireland	2019	5673·1 (5413·3 to 5932·4)	4156·5 (3917·2 to 4406·1)	689·5 (643·4 to 739·1)	827·0 (770·6 to 889·4)	0·0 (0·0 to 0·0)	6362·4 (6071·0 to 6653·2)	4661·5 (4393·1 to 4941·4)	773·3 (721·6 to 828·8)	927·5 (864·2 to 997·4)	0·0 (0·0 to 0·0)

Ireland	2020	6240·2 (5944·1 to 6535·4)	4787·9 (4504·8 to 5081·2)	651·1 (607·2 to 698·0)	801·3 (746·0 to 865·3)	0·0 (0·0 to 0·0)	6998·3 (6666·3 to 7329·5)	5369·6 (5052·1 to 5698·5)	730·2 (680·9 to 782·9)	898·6 (836·7 to 970·4)	0·0 (0·0 to 0·0)
Ireland	2021	6128·1 (5838·3 to 6417·0)	4690·0 (4412·7 to 4977·3)	636·8 (593·6 to 682·8)	801·3 (742·9 to 865·2)	0·0 (0·0 to 0·0)	6872·6 (6547·6 to 7196·6)	5259·8 (4948·8 to 5582·0)	714·2 (665·8 to 765·8)	898·7 (833·1 to 970·4)	0·0 (0·0 to 0·0)
Ireland	2022	5466·8 (5201·2 to 5729·1)	3997·0 (3744·8 to 4255·4)	642·7 (599·1 to 688·9)	827·1 (766·4 to 893·2)	0·0 (0·0 to 0·0)	6131·0 (5833·1 to 6425·2)	4482·6 (4199·7 to 4772·4)	720·7 (671·9 to 772·6)	927·6 (859·5 to 1001·7)	0·0 (0·0 to 0·0)
Ireland	2023	5538·9 (5259·0 to 5820·1)	4036·7 (3763·3 to 4308·8)	648·5 (604·6 to 695·0)	853·7 (791·3 to 925·4)	0·0 (0·0 to 0·0)	6211·8 (5898·0 to 6527·3)	4527·1 (4220·6 to 4832·3)	727·3 (678·0 to 779·4)	957·4 (887·5 to 1037·8)	0·0 (0·0 to 0·0)
Ireland	2024	5635·5 (5341·9 to 5931·2)	4098·8 (3810·2 to 4389·2)	654·9 (610·5 to 701·7)	881·8 (815·8 to 958·6)	0·0 (0·0 to 0·0)	6320·2 (5991·0 to 6651·8)	4596·8 (4273·2 to 4922·5)	734·5 (684·7 to 786·9)	988·9 (915·0 to 1075·0)	0·0 (0·0 to 0·0)
Ireland	2025	5677·1 (5375·9 to 5976·9)	4121·0 (3823·5 to 4411·3)	654·6 (610·2 to 701·5)	901·6 (830·1 to 984·5)	0·0 (0·0 to 0·0)	6366·9 (6029·1 to 6703·1)	4621·7 (4288·0 to 4947·2)	734·1 (684·3 to 786·7)	1011·1 (930·9 to 1104·1)	0·0 (0·0 to 0·0)
Ireland	2026	5722·3 (5411·1 to 6026·8)	4144·2 (3843·2 to 4435·7)	654·8 (610·1 to 701·6)	923·3 (849·5 to 1012·3)	0·0 (0·0 to 0·0)	6417·6 (6068·5 to 6759·0)	4647·7 (4310·1 to 4974·6)	734·4 (684·3 to 786·8)	1035·5 (952·7 to 1135·3)	0·0 (0·0 to 0·0)
Ireland	2027	5776·6 (5456·8 to 6092·0)	4174·4 (3867·1 to 4480·9)	655·2 (610·2 to 702·1)	947·0 (869·1 to 1036·4)	0·0 (0·0 to 0·0)	6478·5 (6119·7 to 6832·2)	4681·6 (4337·0 to 5025·3)	734·8 (684·3 to 787·4)	1062·0 (974·7 to 1162·4)	0·0 (0·0 to 0·0)
Ireland	2028	5837·0 (5501·6 to 6160·5)	4207·8 (3886·7 to 4524·1)	656·3 (611·0 to 703·4)	972·8 (893·2 to 1065·1)	0·0 (0·0 to 0·0)	6546·1 (6170·1 to 6908·9)	4719·1 (4359·0 to 5073·8)	736·1 (685·2 to 788·9)	1091·0 (1001·7 to 1194·5)	0·0 (0·0 to 0·0)
Ireland	2029	5904·9 (5565·5 to 6234·2)	4245·9 (3922·9 to 4564·5)	658·2 (612·5 to 706·0)	1000·8 (916·6 to 1098·6)	0·0 (0·0 to 0·0)	6622·4 (6241·6 to 6991·7)	4761·8 (4399·5 to 5119·1)	738·2 (686·9 to 791·7)	1122·4 (1028·0 to 1232·0)	0·0 (0·0 to 0·0)
Ireland	2030	5957·2 (5609·2 to 6294·6)	4267·3 (3941·4 to 4590·0)	660·2 (614·1 to 708·5)	1029·7 (941·1 to 1132·1)	0·0 (0·0 to 0·0)	6681·0 (6290·6 to 7059·3)	4785·7 (4420·3 to 5147·7)	740·4 (688·7 to 794·6)	1154·8 (1055·4 to 1269·6)	0·0 (0·0 to 0·0)
Ireland	2031	6103·7 (5743·4 to 6465·5)	4367·7 (4030·9 to 4720·2)	667·7 (621·0 to 716·4)	1068·3 (972·5 to 1177·6)	0·0 (0·0 to 0·0)	6845·3 (6441·2 to 7251·1)	4898·4 (4520·7 to 5293·7)	748·8 (696·5 to 803·4)	1198·1 (1090·6 to 1320·6)	0·0 (0·0 to 0·0)
Ireland	2032	6244·0 (5865·7 to 6626·1)	4462·4 (4115·5 to 4816·9)	674·2 (627·1 to 723·4)	1107·3 (1002·4 to 1228·3)	0·0 (0·0 to 0·0)	7002·6 (6578·3 to 7431·2)	5004·6 (4615·5 to 5402·2)	756·1 (703·3 to 811·3)	1241·9 (1124·2 to 1377·5)	0·0 (0·0 to 0·0)
Ireland	2033	6368·0 (5980·8 to 6764·3)	4541·8 (4184·7 to 4928·2)	679·9 (632·4 to 729·7)	1146·2 (1031·9 to 1279·2)	0·0 (0·0 to 0·0)	7141·6 (6707·5 to 7586·1)	5093·6 (4693·2 to 5527·0)	762·6 (709·3 to 818·4)	1285·5 (1157·3 to 1434·6)	0·0 (0·0 to 0·0)

Ireland	2034	6492·4 (6091·9 to 6889·1)	4621·7 (4247·6 to 5008·3)	685·1 (637·3 to 735·5)	1185·5 (1059·9 to 1333·9)	0·0 (0·0 to 0·0)	7281·2 (6832·0 to 7726·1)	5183·2 (4763·7 to 5616·8)	768·4 (714·7 to 824·9)	1329·6 (1188·7 to 1496·0)	0·0 (0·0 to 0·0)
Ireland	2035	6616·6 (6187·9 to 7028·0)	4700·7 (4311·5 to 5100·7)	690·2 (641·8 to 741·1)	1225·7 (1086·8 to 1385·7)	0·0 (0·0 to 0·0)	7420·5 (6939·7 to 7881·8)	5271·8 (4835·3 to 5720·4)	774·1 (719·8 to 831·2)	1374·6 (1218·8 to 1554·0)	0·0 (0·0 to 0·0)
Ireland	2036	6735·7 (6301·3 to 7164·6)	4775·0 (4386·6 to 5193·5)	694·7 (646·1 to 746·1)	1266·0 (1115·5 to 1438·3)	0·0 (0·0 to 0·0)	7554·0 (7066·8 to 8035·1)	5355·2 (4919·6 to 5824·5)	779·1 (724·6 to 836·7)	1419·8 (1251·0 to 1613·1)	0·0 (0·0 to 0·0)
Ireland	2037	6853·9 (6394·6 to 7302·4)	4847·7 (4448·9 to 5276·2)	699·1 (650·4 to 750·7)	1307·1 (1143·0 to 1491·3)	0·0 (0·0 to 0·0)	7686·6 (7171·6 to 8189·6)	5436·7 (4989·4 to 5917·2)	784·0 (729·5 to 841·9)	1465·9 (1281·9 to 1672·5)	0·0 (0·0 to 0·0)
Ireland	2038	6974·8 (6497·9 to 7456·8)	4921·5 (4503·3 to 5365·7)	703·6 (655·0 to 755·4)	1349·6 (1169·9 to 1551·1)	0·0 (0·0 to 0·0)	7822·2 (7287·3 to 8362·8)	5519·5 (5050·4 to 6017·7)	789·1 (734·5 to 847·2)	1513·6 (1312·1 to 1739·5)	0·0 (0·0 to 0·0)
Ireland	2039	7092·4 (6609·4 to 7587·0)	4992·3 (4563·1 to 5435·5)	707·6 (658·7 to 759·6)	1392·5 (1197·1 to 1608·9)	0·0 (0·0 to 0·0)	7954·1 (7412·4 to 8508·8)	5598·8 (5117·5 to 6095·9)	793·6 (738·7 to 851·9)	1561·7 (1342·5 to 1804·4)	0·0 (0·0 to 0·0)
Ireland	2040	7210·0 (6707·0 to 7732·5)	5062·8 (4625·5 to 5541·0)	711·3 (661·9 to 763·5)	1435·9 (1224·1 to 1673·2)	0·0 (0·0 to 0·0)	8086·0 (7521·9 to 8672·0)	5677·9 (5187·5 to 6214·2)	797·7 (742·4 to 856·2)	1610·3 (1372·8 to 1876·5)	0·0 (0·0 to 0·0)
Ireland	2041	7322·2 (6807·4 to 7864·3)	5129·0 (4685·6 to 5610·7)	714·2 (664·7 to 766·5)	1478·9 (1251·1 to 1738·0)	0·0 (0·0 to 0·0)	8211·8 (7634·5 to 8819·8)	5752·2 (5254·9 to 6292·4)	801·0 (745·5 to 859·6)	1658·6 (1403·1 to 1949·1)	0·0 (0·0 to 0·0)
Ireland	2042	7434·9 (6903·1 to 8002·1)	5194·6 (4745·4 to 5702·3)	717·1 (667·5 to 769·4)	1523·2 (1278·4 to 1802·6)	0·0 (0·0 to 0·0)	8338·2 (7741·8 to 8974·3)	5825·8 (5321·9 to 6395·1)	804·2 (748·6 to 862·9)	1708·3 (1433·7 to 2021·6)	0·0 (0·0 to 0·0)
Ireland	2043	7554·8 (7005·9 to 8141·7)	5266·7 (4817·3 to 5771·3)	719·6 (669·9 to 771·9)	1568·5 (1306·0 to 1870·9)	0·0 (0·0 to 0·0)	8472·7 (7857·1 to 9130·9)	5906·6 (5402·6 to 6472·5)	807·0 (751·3 to 865·7)	1759·1 (1464·7 to 2098·2)	0·0 (0·0 to 0·0)
Ireland	2044	7671·1 (7095·6 to 8305·5)	5335·0 (4870·7 to 5833·9)	721·5 (671·8 to 773·7)	1614·6 (1332·3 to 1943·9)	0·0 (0·0 to 0·0)	8603·1 (7957·7 to 9314·6)	5983·2 (5462·4 to 6542·7)	809·2 (753·5 to 867·7)	1810·8 (1494·2 to 2180·1)	0·0 (0·0 to 0·0)
Ireland	2045	7791·6 (7204·2 to 8432·6)	5407·6 (4945·5 to 5927·0)	723·1 (673·4 to 775·5)	1661·0 (1358·7 to 2020·6)	0·0 (0·0 to 0·0)	8738·3 (8079·5 to 9457·2)	6064·6 (5546·3 to 6647·1)	810·9 (755·2 to 869·7)	1862·8 (1523·8 to 2266·1)	0·0 (0·0 to 0·0)
Ireland	2046	7912·6 (7301·6 to 8570·7)	5479·4 (4991·2 to 6003·0)	724·9 (675·2 to 777·4)	1708·4 (1384·9 to 2092·2)	0·0 (0·0 to 0·0)	8874·0 (8188·8 to 9612·0)	6145·1 (5597·6 to 6732·3)	813·0 (757·2 to 871·8)	1915·9 (1553·2 to 2346·4)	0·0 (0·0 to 0·0)
Ireland	2047	8043·2 (7405·8 to 8735·3)	5556·7 (5065·0 to 6101·4)	728·0 (677·8 to 780·6)	1758·5 (1413·1 to 2165·1)	0·0 (0·0 to 0·0)	9020·5 (8305·6 to 9796·6)	6231·9 (5680·4 to 6842·7)	816·5 (760·1 to 875·5)	1972·1 (1584·8 to 2428·2)	0·0 (0·0 to 0·0)

Ireland	2048	8180·0 (7530·9 to 8896·0)	5636·5 (5136·2 to 6191·9)	732·3 (681·3 to 785·3)	1811·2 (1443·2 to 2242·7)	0·0 (0·0 to 0·0)	9173·8 (8445·9 to 9976·8)	6321·3 (5760·2 to 6944·2)	821·2 (764·0 to 880·8)	2031·3 (1618·6 to 2515·2)	0·0 (0·0 to 0·0)
Ireland	2049	8314·4 (7645·1 to 9069·6)	5712·6 (5183·5 to 6271·6)	736·7 (685·0 to 790·0)	1865·1 (1472·3 to 2322·9)	0·0 (0·0 to 0·0)	9324·5 (8574·0 to 10171·5)	6406·7 (5813·3 to 7033·5)	826·2 (768·2 to 886·0)	2091·7 (1651·2 to 2605·1)	0·0 (0·0 to 0·0)
Ireland	2050	8456·4 (7757·9 to 9224·5)	5791·8 (5268·8 to 6373·2)	742·2 (690·2 to 795·8)	1922·4 (1505·9 to 2414·1)	0·0 (0·0 to 0·0)	9483·9 (8700·5 to 10345·3)	6495·5 (5909·0 to 7147·5)	832·4 (774·0 to 892·5)	2155·9 (1688·8 to 2707·4)	0·0 (0·0 to 0·0)
Iran*	1995	229·0 (208·1 to 253·2)	92·8 (80·2 to 106·3)	129·7 (112·6 to 149·0)	6·4 (4·1 to 9·1)	0·1 (0·1 to 0·1)	377·5 (343·0 to 417·4)	152·9 (132·2 to 175·3)	213·9 (185·6 to 245·7)	10·5 (6·8 to 15·0)	0·1 (0·1 to 0·1)
Iran*	1996	235·3 (215·6 to 256·1)	95·5 (83·7 to 107·5)	132·6 (116·6 to 149·4)	6·5 (4·4 to 9·4)	0·7 (0·7 to 0·7)	387·8 (355·3 to 422·2)	157·4 (137·9 to 177·2)	218·5 (192·3 to 246·3)	10·8 (7·2 to 15·5)	1·1 (1·1 to 1·1)
Iran*	1997	250·1 (230·2 to 270·7)	101·8 (90·2 to 113·8)	140·1 (124·4 to 155·9)	7·5 (5·1 to 10·6)	0·6 (0·6 to 0·6)	412·3 (379·4 to 446·3)	167·9 (148·7 to 187·5)	231·0 (205·1 to 257·0)	12·4 (8·4 to 17·5)	1·1 (1·1 to 1·1)
Iran*	1998	264·9 (246·1 to 285·2)	106·5 (95·2 to 118·9)	149·8 (135·2 to 165·2)	8·2 (5·6 to 11·4)	0·4 (0·4 to 0·4)	436·7 (405·6 to 470·1)	175·6 (157·0 to 196·0)	247·0 (222·8 to 272·3)	13·5 (9·3 to 18·9)	0·6 (0·6 to 0·6)
Iran*	1999	274·7 (257·0 to 294·3)	106·3 (95·6 to 118·1)	159·0 (144·6 to 174·3)	9·2 (6·3 to 12·6)	0·2 (0·2 to 0·2)	452·8 (423·6 to 485·1)	175·2 (157·6 to 194·7)	262·1 (238·5 to 287·4)	15·1 (10·4 to 20·8)	0·3 (0·3 to 0·3)
Iran*	2000	284·6 (267·4 to 303·1)	107·3 (96·9 to 118·8)	168·1 (154·2 to 182·5)	9·0 (6·2 to 12·3)	0·1 (0·1 to 0·1)	469·1 (440·7 to 499·6)	176·9 (159·8 to 195·9)	277·1 (254·2 to 300·8)	14·8 (10·2 to 20·4)	0·2 (0·2 to 0·2)
Iran*	2001	298·5 (279·5 to 316·8)	112·3 (101·7 to 124·1)	175·9 (161·5 to 190·6)	10·1 (7·1 to 13·9)	0·2 (0·2 to 0·2)	492·2 (460·7 to 522·3)	185·2 (167·6 to 204·6)	290·0 (266·3 to 314·3)	16·6 (11·7 to 22·9)	0·4 (0·4 to 0·4)
Iran*	2002	335·8 (316·0 to 356·4)	131·8 (119·8 to 146·0)	189·0 (174·0 to 203·4)	15·0 (10·6 to 20·3)	0·1 (0·1 to 0·1)	553·7 (521·0 to 587·6)	217·3 (197·5 to 240·7)	311·5 (286·8 to 335·3)	24·7 (17·4 to 33·5)	0·1 (0·1 to 0·1)
Iran*	2003	358·0 (337·8 to 377·7)	139·6 (126·9 to 153·7)	199·3 (185·1 to 213·8)	19·1 (13·7 to 25·9)	0·0 (0·0 to 0·0)	590·2 (556·8 to 622·7)	230·1 (209·1 to 253·4)	328·6 (305·1 to 352·4)	31·5 (22·5 to 42·7)	0·0 (0·0 to 0·0)
Iran*	2004	362·8 (343·2 to 383·0)	137·1 (125·1 to 150·0)	204·4 (190·9 to 218·8)	21·3 (15·4 to 28·9)	0·1 (0·1 to 0·1)	598·1 (565·7 to 631·4)	226·0 (206·3 to 247·3)	337·0 (314·8 to 360·7)	35·0 (25·3 to 47·6)	0·2 (0·2 to 0·2)
Iran*	2005	397·9 (376·5 to 419·9)	155·2 (141·4 to 169·0)	218·1 (203·8 to 233·8)	23·6 (17·2 to 31·7)	1·0 (1·0 to 1·0)	655·9 (620·7 to 692·3)	255·8 (233·2 to 278·6)	359·5 (335·9 to 385·5)	39·0 (28·4 to 52·3)	1·7 (1·7 to 1·7)

Iran*	2006	424·6 (402·5 to 446·9)	171·5 (157·3 to 187·6)	227·6 (212·4 to 244·8)	25·2 (18·2 to 34·0)	0·2 (0·2 to 0·2)	699·9 (663·6 to 736·7)	282·8 (259·3 to 309·2)	375·2 (350·2 to 403·6)	41·6 (30·0 to 56·0)	0·3 (0·3 to 0·3)
Iran*	2007	423·9 (402·0 to 447·3)	163·8 (150·0 to 178·5)	233·7 (218·6 to 249·8)	26·2 (19·3 to 34·5)	0·1 (0·1 to 0·1)	698·7 (662·6 to 737·5)	270·1 (247·3 to 294·3)	385·3 (360·4 to 411·8)	43·2 (31·9 to 56·9)	0·2 (0·2 to 0·2)
Iran*	2008	470·6 (448·1 to 494·2)	177·9 (163·1 to 192·7)	262·0 (247·3 to 279·1)	30·5 (22·9 to 39·6)	0·2 (0·2 to 0·2)	775·7 (738·8 to 814·7)	293·2 (268·9 to 317·7)	431·9 (407·7 to 460·1)	50·2 (37·7 to 65·3)	0·4 (0·4 to 0·4)
Iran*	2009	513·6 (491·1 to 537·5)	181·8 (168·1 to 195·9)	294·0 (278·2 to 310·7)	37·5 (28·7 to 47·5)	0·2 (0·2 to 0·2)	846·6 (809·6 to 886·1)	299·7 (277·1 to 323·0)	484·7 (458·6 to 512·2)	61·8 (47·3 to 78·4)	0·4 (0·4 to 0·4)
Iran*	2010	554·0 (529·9 to 578·2)	188·8 (175·0 to 203·3)	320·4 (304·4 to 337·8)	44·7 (34·8 to 55·9)	0·1 (0·1 to 0·1)	913·4 (873·6 to 953·2)	311·2 (288·5 to 335·1)	528·3 (501·7 to 557·0)	73·6 (57·4 to 92·2)	0·2 (0·2 to 0·2)
Iran*	2011	581·9 (556·7 to 608·2)	205·7 (190·4 to 221·4)	324·7 (306·7 to 341·6)	51·3 (40·1 to 63·4)	0·2 (0·2 to 0·2)	959·2 (917·7 to 1002·6)	339·1 (313·9 to 365·0)	535·3 (505·5 to 563·2)	84·5 (66·1 to 104·5)	0·3 (0·3 to 0·3)
Iran*	2012	514·8 (491·1 to 539·4)	184·5 (171·0 to 197·6)	280·3 (264·2 to 295·9)	49·8 (39·0 to 61·8)	0·3 (0·3 to 0·3)	848·7 (809·6 to 889·3)	304·1 (281·9 to 325·7)	462·1 (435·6 to 487·8)	82·1 (64·3 to 101·9)	0·4 (0·4 to 0·4)
Iran*	2013	513·4 (489·3 to 536·1)	208·3 (193·7 to 222·4)	256·4 (239·9 to 272·1)	48·5 (38·0 to 60·0)	0·1 (0·1 to 0·1)	846·3 (806·6 to 883·7)	343·4 (319·3 to 366·6)	422·7 (395·6 to 448·6)	80·0 (62·6 to 98·8)	0·2 (0·2 to 0·2)
Iran*	2014	563·4 (538·6 to 588·2)	260·5 (244·9 to 276·5)	254·3 (237·8 to 270·7)	48·4 (38·4 to 59·7)	0·2 (0·2 to 0·2)	928·7 (887·9 to 969·7)	429·5 (403·8 to 455·9)	419·2 (392·1 to 446·2)	79·7 (63·4 to 98·5)	0·3 (0·3 to 0·3)
Iran*	2015	635·5 (611·8 to 661·3)	314·9 (299·6 to 329·8)	268·1 (251·6 to 285·8)	52·3 (41·9 to 64·2)	0·1 (0·1 to 0·1)	1047·6 (1008·5 to 1090·1)	519·1 (493·9 to 543·7)	442·0 (414·7 to 471·1)	86·3 (69·1 to 105·8)	0·2 (0·2 to 0·2)
Iran*	2016	737·9 (711·3 to 766·1)	375·2 (360·5 to 390·6)	298·5 (282·2 to 317·3)	64·1 (52·0 to 77·5)	0·1 (0·1 to 0·1)	1216·4 (1172·5 to 1263·0)	618·6 (594·4 to 644·0)	492·1 (465·2 to 523·1)	105·6 (85·7 to 127·7)	0·1 (0·1 to 0·1)
Iran*	2017	764·5 (735·9 to 793·9)	385·4 (371·2 to 401·0)	302·6 (284·1 to 322·0)	76·4 (62·9 to 91·9)	0·1 (0·1 to 0·1)	1260·2 (1213·2 to 1308·8)	635·3 (612·0 to 661·1)	498·8 (468·4 to 530·9)	126·0 (103·6 to 151·5)	0·1 (0·1 to 0·1)
Iran*	2018	734·3 (699·8 to 768·8)	345·6 (328·5 to 362·6)	279·0 (257·2 to 302·4)	109·7 (90·1 to 132·5)	0·1 (0·1 to 0·1)	1210·5 (1153·6 to 1267·4)	569·7 (541·5 to 597·8)	459·9 (424·1 to 498·5)	180·8 (148·6 to 218·4)	0·1 (0·1 to 0·1)
Iran*	2019	674·8 (642·1 to 706·3)	311·1 (296·0 to 326·7)	258·1 (237·9 to 279·7)	105·5 (86·2 to 127·9)	0·1 (0·1 to 0·1)	1112·5 (1058·6 to 1164·4)	512·9 (488·0 to 538·6)	425·5 (392·2 to 461·1)	173·9 (142·0 to 210·8)	0·1 (0·1 to 0·1)

Iran*	2020	780·7 (749·7 to 811·2)	443·3 (428·3 to 458·7)	237·2 (218·9 to 257·3)	100·0 (81·5 to 121·1)	0·1 (0·1 to 0·1)	1287·0 (1235·9 to 1337·2)	730·8 (706·1 to 756·3)	391·1 (360·9 to 424·1)	164·9 (134·4 to 199·7)	0·2 (0·2 to 0·2)
Iran*	2021	620·0 (588·9 to 650·5)	298·9 (281·4 to 316·9)	223·7 (206·4 to 242·9)	97·2 (79·1 to 117·7)	0·1 (0·1 to 0·2)	1022·1 (970·8 to 1072·4)	492·8 (464·0 to 522·5)	368·8 (340·3 to 400·4)	160·2 (130·4 to 194·1)	0·2 (0·2 to 0·3)
Iran*	2022	600·5 (567·7 to 632·4)	277·2 (257·8 to 298·5)	223·4 (206·0 to 242·7)	99·8 (80·8 to 121·1)	0·1 (0·1 to 0·1)	990·0 (935·9 to 1042·5)	457·0 (425·1 to 492·0)	368·2 (339·6 to 400·1)	164·6 (133·1 to 199·6)	0·2 (0·2 to 0·2)
Iran*	2023	612·3 (578·9 to 644·9)	286·1 (266·6 to 308·3)	223·3 (205·8 to 242·6)	102·8 (82·5 to 125·2)	0·1 (0·1 to 0·1)	1009·5 (954·3 to 1063·2)	471·6 (439·6 to 508·2)	368·2 (339·3 to 399·9)	169·4 (136·0 to 206·4)	0·2 (0·2 to 0·2)
Iran*	2024	623·9 (589·4 to 657·9)	294·8 (274·4 to 316·9)	223·2 (205·7 to 242·6)	105·8 (85·3 to 129·6)	0·1 (0·1 to 0·1)	1028·6 (971·6 to 1084·6)	485·9 (452·4 to 522·4)	368·0 (339·0 to 399·9)	174·4 (140·6 to 213·6)	0·2 (0·2 to 0·2)
Iran*	2025	636·0 (600·3 to 670·6)	303·6 (281·7 to 327·4)	223·2 (205·8 to 242·5)	109·0 (87·4 to 134·0)	0·1 (0·1 to 0·2)	1048·5 (989·7 to 1105·6)	500·6 (464·3 to 539·8)	368·0 (339·3 to 399·8)	179·7 (144·1 to 220·9)	0·2 (0·2 to 0·3)
Iran*	2026	649·1 (611·6 to 685·8)	313·2 (290·5 to 337·6)	223·3 (206·0 to 242·6)	112·4 (90·3 to 138·6)	0·1 (0·1 to 0·2)	1070·0 (1008·2 to 1130·5)	516·3 (478·9 to 556·6)	368·2 (339·7 to 399·9)	185·3 (148·9 to 228·5)	0·2 (0·2 to 0·3)
Iran*	2027	662·2 (623·2 to 700·8)	322·4 (298·2 to 348·4)	223·7 (206·4 to 243·0)	116·0 (93·5 to 143·7)	0·2 (0·1 to 0·2)	1091·7 (1027·3 to 1155·3)	531·5 (491·7 to 574·3)	368·8 (340·3 to 400·6)	191·2 (154·1 to 236·9)	0·2 (0·2 to 0·3)
Iran*	2028	674·9 (634·6 to 714·3)	331·3 (306·3 to 358·8)	223·9 (206·4 to 243·0)	119·6 (95·7 to 149·1)	0·2 (0·1 to 0·2)	1112·6 (1046·2 to 1177·5)	546·2 (504·9 to 591·5)	369·0 (340·3 to 400·6)	197·1 (157·7 to 245·7)	0·3 (0·2 to 0·3)
Iran*	2029	688·9 (647·4 to 730·0)	341·2 (315·7 to 368·8)	224·2 (206·5 to 243·4)	123·4 (98·2 to 154·4)	0·2 (0·1 to 0·2)	1135·6 (1067·2 to 1203·4)	562·5 (520·4 to 607·9)	369·5 (340·4 to 401·3)	203·4 (161·8 to 254·5)	0·3 (0·2 to 0·3)
Iran*	2030	703·2 (660·2 to 747·4)	351·3 (325·6 to 380·4)	224·5 (206·5 to 243·9)	127·2 (100·8 to 158·5)	0·2 (0·1 to 0·2)	1159·2 (1088·4 to 1232·1)	579·2 (536·8 to 627·2)	370·0 (340·4 to 402·1)	209·7 (166·2 to 261·3)	0·3 (0·2 to 0·4)
Iran*	2031	717·7 (673·2 to 764·5)	361·9 (335·1 to 392·4)	224·5 (206·4 to 244·4)	131·1 (103·5 to 163·9)	0·2 (0·2 to 0·2)	1183·1 (1109·7 to 1260·3)	596·6 (552·4 to 646·8)	370·1 (340·2 to 402·9)	216·1 (170·7 to 270·2)	0·3 (0·3 to 0·4)
Iran*	2032	732·3 (686·3 to 780·5)	372·4 (346·0 to 402·7)	224·6 (206·3 to 244·6)	135·1 (106·5 to 171·0)	0·2 (0·2 to 0·3)	1207·1 (1131·5 to 1286·6)	613·9 (570·4 to 663·9)	370·2 (340·1 to 403·2)	222·7 (175·5 to 281·9)	0·3 (0·3 to 0·4)
Iran*	2033	747·3 (699·5 to 796·4)	383·0 (355·4 to 414·1)	224·7 (206·3 to 244·6)	139·3 (109·9 to 176·8)	0·2 (0·2 to 0·3)	1231·9 (1153·2 to 1312·9)	631·4 (585·8 to 682·7)	370·5 (340·1 to 403·3)	229·6 (181·2 to 291·5)	0·3 (0·3 to 0·4)

Iran*	2034	763·1 (712·8 to 814·4)	394·0 (364·5 to 426·0)	225·1 (206·4 to 245·2)	143·8 (112·9 to 183·2)	0·2 (0·2 to 0·3)	1258·0 (1175·0 to 1342·5)	649·4 (600·9 to 702·3)	371·2 (340·2 to 404·3)	237·1 (186·1 to 302·1)	0·4 (0·3 to 0·5)
Iran*	2035	778·3 (725·4 to 832·1)	404·4 (373·3 to 437·7)	225·3 (206·3 to 245·7)	148·4 (115·3 to 191·1)	0·2 (0·2 to 0·3)	1283·1 (1195·9 to 1371·8)	666·6 (615·5 to 721·6)	371·5 (340·1 to 405·0)	244·6 (190·1 to 315·0)	0·4 (0·3 to 0·5)
Iran*	2036	791·1 (735·7 to 848·5)	413·4 (380·6 to 448·2)	224·9 (205·9 to 245·3)	152·6 (117·9 to 198·8)	0·2 (0·2 to 0·3)	1304·2 (1212·8 to 1398·7)	681·5 (627·4 to 738·9)	370·7 (339·4 to 404·3)	251·5 (194·4 to 327·7)	0·4 (0·3 to 0·5)
Iran*	2037	803·7 (745·0 to 864·5)	422·1 (386·2 to 459·9)	224·4 (205·4 to 245·0)	156·9 (120·4 to 206·2)	0·3 (0·2 to 0·4)	1324·9 (1228·1 to 1425·2)	695·8 (636·6 to 758·2)	370·0 (338·6 to 403·8)	258·6 (198·5 to 340·0)	0·4 (0·3 to 0·6)
Iran*	2038	815·4 (754·2 to 878·4)	430·1 (391·1 to 469·9)	223·8 (204·8 to 244·6)	161·1 (122·8 to 213·8)	0·3 (0·2 to 0·4)	1344·1 (1243·3 to 1448·1)	709·0 (644·8 to 774·7)	369·0 (337·7 to 403·3)	265·7 (202·5 to 352·4)	0·5 (0·4 to 0·6)
Iran*	2039	827·1 (762·4 to 892·4)	437·9 (396·3 to 479·6)	223·3 (204·3 to 244·4)	165·5 (124·7 to 221·4)	0·3 (0·2 to 0·4)	1363·5 (1256·9 to 1471·1)	721·9 (653·4 to 790·6)	368·2 (336·8 to 402·8)	272·9 (205·5 to 365·1)	0·5 (0·4 to 0·7)
Iran*	2040	838·4 (769·2 to 907·5)	445·3 (401·0 to 488·9)	222·8 (203·6 to 244·0)	170·0 (126·6 to 229·5)	0·3 (0·3 to 0·4)	1382·1 (1268·1 to 1496·0)	734·1 (661·0 to 805·9)	367·2 (335·6 to 402·2)	280·3 (208·8 to 378·3)	0·5 (0·4 to 0·7)
Iran*	2041	848·8 (775·8 to 921·1)	452·0 (404·7 to 498·1)	222·0 (202·5 to 243·4)	174·5 (128·2 to 237·6)	0·3 (0·3 to 0·5)	1399·3 (1278·9 to 1518·4)	745·2 (667·1 to 821·1)	365·9 (333·9 to 401·3)	287·6 (211·4 to 391·7)	0·6 (0·4 to 0·8)
Iran*	2042	857·2 (780·9 to 932·8)	457·5 (406·9 to 505·5)	220·6 (201·0 to 242·2)	178·7 (129·7 to 247·3)	0·4 (0·3 to 0·5)	1413·2 (1287·3 to 1537·7)	754·2 (670·7 to 833·4)	363·7 (331·4 to 399·3)	294·6 (213·9 to 407·8)	0·6 (0·5 to 0·9)
Iran*	2043	865·0 (784·0 to 946·7)	462·5 (408·1 to 512·7)	219·0 (199·3 to 240·9)	183·0 (131·9 to 255·9)	0·4 (0·3 to 0·6)	1425·9 (1292·5 to 1560·6)	762·5 (672·8 to 845·1)	361·0 (328·5 to 397·2)	301·7 (217·4 to 421·9)	0·7 (0·5 to 1·0)
Iran*	2044	871·3 (785·2 to 957·1)	466·6 (408·3 to 518·9)	217·0 (197·1 to 238·9)	187·3 (132·7 to 265·1)	0·5 (0·3 to 0·7)	1436·4 (1294·5 to 1577·8)	769·1 (673·0 to 855·4)	357·8 (325·0 to 393·9)	308·7 (218·8 to 437·0)	0·7 (0·6 to 1·1)
Iran*	2045	877·4 (785·3 to 968·9)	470·3 (407·8 to 524·5)	215·0 (194·9 to 237·3)	191·6 (133·5 to 274·0)	0·5 (0·4 to 0·7)	1446·5 (1294·5 to 1597·2)	775·4 (672·3 to 864·6)	354·4 (321·4 to 391·1)	315·9 (220·0 to 451·7)	0·8 (0·6 to 1·2)
Iran*	2046	882·3 (784·3 to 979·3)	473·2 (406·9 to 529·5)	212·8 (192·6 to 235·3)	195·8 (134·1 to 281·9)	0·6 (0·4 to 0·8)	1454·6 (1293·0 to 1614·4)	780·1 (670·8 to 872·9)	350·7 (317·5 to 387·9)	322·9 (221·1 to 464·8)	0·9 (0·7 to 1·4)
Iran*	2047	885·4 (782·3 to 989·5)	474·7 (404·8 to 533·1)	210·2 (190·0 to 232·9)	199·8 (134·6 to 291·9)	0·6 (0·4 to 0·9)	1459·5 (1289·7 to 1631·2)	782·6 (667·3 to 878·9)	346·5 (313·2 to 383·9)	329·4 (221·9 to 481·3)	1·0 (0·7 to 1·6)

Iran*	2048	888·3 (780·2 to 998·9)	476·0 (402·4 to 535·9)	207·7 (187·4 to 230·5)	203·9 (135·3 to 303·5)	0·7 (0·5 to 1·1)	1464·3 (1286·2 to 1646·7)	784·7 (663·4 to 883·4)	342·4 (309·0 to 379·9)	336·1 (223·1 to 500·4)	1·1 (0·8 to 1·8)
Iran*	2049	890·1 (775·7 to 1005·6)	476·4 (399·2 to 537·7)	205·0 (184·6 to 227·8)	207·9 (135·4 to 313·3)	0·8 (0·5 to 1·2)	1467·3 (1278·7 to 1657·7)	785·4 (658·1 to 886·5)	337·9 (304·3 to 375·6)	342·7 (223·2 to 516·5)	1·3 (0·9 to 2·0)
Iran*	2050	890·2 (770·4 to 1013·8)	475·7 (395·1 to 539·2)	202·0 (181·6 to 224·9)	211·7 (135·3 to 322·5)	0·9 (0·6 to 1·4)	1467·5 (1270·0 to 1671·4)	784·1 (651·4 to 888·9)	333·0 (299·3 to 370·8)	348·9 (223·0 to 531·6)	1·4 (1·0 to 2·3)
Iraq*	1995	56·2 (48·4 to 65·4)	27·4 (22·3 to 33·3)	28·0 (22·0 to 35·4)	0·0 (0·0 to 0·0)	0·7 (0·7 to 0·7)	126·0 (108·6 to 146·7)	61·4 (50·0 to 74·6)	62·8 (49·4 to 79·4)	0·0 (0·0 to 0·0)	1·7 (1·7 to 1·7)
Iraq*	1996	58·2 (50·3 to 67·4)	29·8 (24·4 to 36·1)	28·2 (22·3 to 35·4)	0·0 (0·0 to 0·0)	0·2 (0·2 to 0·2)	130·5 (112·8 to 151·2)	66·8 (54·7 to 80·9)	63·3 (50·1 to 79·4)	0·0 (0·0 to 0·0)	0·4 (0·4 to 0·4)
Iraq*	1997	67·3 (58·2 to 78·0)	37·0 (30·6 to 45·0)	30·2 (23·9 to 37·5)	0·0 (0·0 to 0·0)	0·1 (0·1 to 0·1)	151·0 (130·5 to 174·8)	83·0 (68·7 to 101·0)	67·8 (53·7 to 84·1)	0·0 (0·0 to 0·0)	0·2 (0·2 to 0·2)
Iraq*	1998	83·0 (71·7 to 95·8)	49·0 (40·8 to 59·9)	33·9 (26·8 to 42·0)	0·0 (0·0 to 0·0)	0·0 (0·0 to 0·0)	186·1 (160·8 to 214·8)	109·9 (91·6 to 134·3)	76·1 (60·2 to 94·1)	0·0 (0·0 to 0·1)	0·1 (0·1 to 0·1)
Iraq*	1999	84·2 (72·8 to 96·4)	49·8 (41·7 to 60·2)	34·2 (27·4 to 41·7)	0·0 (0·0 to 0·0)	0·1 (0·1 to 0·1)	188·8 (163·2 to 216·2)	111·7 (93·4 to 135·0)	76·7 (61·4 to 93·6)	0·0 (0·0 to 0·1)	0·3 (0·3 to 0·3)
Iraq*	2000	86·5 (75·2 to 98·7)	51·5 (43·1 to 62·0)	34·8 (28·6 to 41·8)	0·0 (0·0 to 0·0)	0·1 (0·1 to 0·1)	193·9 (168·5 to 221·2)	115·6 (96·6 to 139·0)	78·1 (64·1 to 93·8)	0·0 (0·0 to 0·1)	0·2 (0·2 to 0·2)
Iraq*	2001	92·3 (81·0 to 104·7)	55·9 (46·2 to 66·7)	36·3 (30·7 to 43·2)	0·0 (0·0 to 0·0)	0·1 (0·1 to 0·1)	207·0 (181·7 to 234·8)	125·3 (103·7 to 149·7)	81·4 (68·9 to 96·8)	0·0 (0·0 to 0·1)	0·3 (0·3 to 0·3)
Iraq*	2002	94·0 (83·2 to 105·9)	57·2 (47·6 to 68·5)	36·7 (31·6 to 42·2)	0·0 (0·0 to 0·0)	0·0 (0·0 to 0·0)	210·8 (186·7 to 237·4)	128·3 (106·7 to 153·7)	82·4 (70·9 to 94·6)	0·0 (0·0 to 0·1)	0·1 (0·1 to 0·1)
Iraq*	2003	75·8 (67·8 to 85·1)	43·0 (36·0 to 51·2)	31·7 (27·8 to 36·0)	0·0 (0·0 to 0·0)	1·1 (1·1 to 1·1)	170·1 (152·1 to 190·7)	96·5 (80·8 to 114·8)	71·0 (62·3 to 80·7)	0·0 (0·0 to 0·1)	2·5 (2·5 to 2·5)
Iraq*	2004	115·9 (103·3 to 130·6)	76·7 (64·7 to 90·4)	36·3 (32·2 to 40·8)	0·0 (0·0 to 0·0)	2·8 (2·8 to 2·8)	259·8 (231·7 to 292·9)	172·1 (145·2 to 202·7)	81·3 (72·3 to 91·4)	0·1 (0·0 to 0·1)	6·3 (6·3 to 6·3)
Iraq*	2005	115·2 (104·2 to 127·7)	66·7 (56·2 to 78·8)	31·4 (27·9 to 35·3)	0·0 (0·0 to 0·0)	17·0 (17·0 to 17·0)	258·2 (233·8 to 286·4)	149·6 (126·1 to 176·6)	70·5 (62·6 to 79·1)	0·1 (0·0 to 0·1)	38·0 (38·0 to 38·0)

Iraq*	2006	107·0 (96·7 to 119·2)	64·1 (54·4 to 75·7)	29·6 (26·3 to 33·5)	0·0 (0·0 to 0·0)	13·2 (13·2 to 13·2)	239·9 (216·8 to 267·3)	143·8 (122·1 to 169·7)	66·4 (58·9 to 75·0)	0·1 (0·0 to 0·1)	29·6 (29·6 to 29·6)
Iraq*	2007	103·5 (93·4 to 116·1)	66·4 (56·5 to 77·6)	29·2 (25·7 to 32·9)	0·0 (0·0 to 0·0)	7·9 (7·9 to 7·9)	232·2 (209·4 to 260·2)	148·9 (126·8 to 174·0)	65·4 (57·6 to 73·7)	0·1 (0·0 to 0·1)	17·8 (17·8 to 17·8)
Iraq*	2008	116·4 (103·9 to 131·4)	83·2 (70·6 to 97·8)	30·4 (26·9 to 34·4)	0·0 (0·0 to 0·0)	2·8 (2·8 to 2·8)	261·1 (233·0 to 294·6)	186·5 (158·3 to 219·4)	68·2 (60·3 to 77·1)	0·1 (0·1 to 0·1)	6·2 (6·2 to 6·2)
Iraq*	2009	124·5 (110·5 to 139·9)	88·6 (75·2 to 104·0)	33·4 (29·6 to 37·5)	0·0 (0·0 to 0·0)	2·5 (2·5 to 2·5)	279·1 (247·8 to 313·7)	198·6 (168·7 to 233·3)	74·8 (66·5 to 84·1)	0·1 (0·0 to 0·1)	5·6 (5·6 to 5·6)
Iraq*	2010	116·2 (103·8 to 129·5)	77·8 (65·9 to 91·2)	36·1 (32·2 to 40·4)	0·0 (0·0 to 0·0)	2·3 (2·3 to 2·3)	260·5 (232·7 to 290·4)	174·4 (147·7 to 204·6)	80·8 (72·2 to 90·6)	0·1 (0·0 to 0·1)	5·3 (5·3 to 5·3)
Iraq*	2011	109·7 (98·6 to 121·5)	67·1 (56·8 to 78·5)	41·7 (37·7 to 46·0)	0·0 (0·0 to 0·0)	0·9 (0·9 to 0·9)	245·9 (221·1 to 272·5)	150·4 (127·3 to 176·0)	93·5 (84·6 to 103·1)	0·1 (0·0 to 0·1)	2·0 (2·0 to 2·0)
Iraq*	2012	115·0 (104·7 to 125·9)	57·7 (48·6 to 67·4)	56·8 (52·1 to 61·7)	0·0 (0·0 to 0·0)	0·6 (0·6 to 0·6)	258·0 (234·7 to 282·2)	129·3 (109·0 to 151·1)	127·3 (116·7 to 138·4)	0·1 (0·0 to 0·1)	1·3 (1·3 to 1·3)
Iraq*	2013	132·3 (121·3 to 143·2)	58·7 (49·4 to 68·5)	72·9 (67·7 to 78·3)	0·0 (0·0 to 0·0)	0·8 (0·8 to 0·8)	296·8 (272·1 to 321·2)	131·6 (110·9 to 153·6)	163·4 (151·9 to 175·7)	0·1 (0·0 to 0·1)	1·8 (1·8 to 1·8)
Iraq*	2014	133·8 (124·2 to 144·2)	50·5 (42·0 to 59·0)	82·3 (77·1 to 88·2)	0·0 (0·0 to 0·0)	1·0 (1·0 to 1·0)	300·0 (278·5 to 323·4)	113·2 (94·2 to 132·3)	184·5 (172·9 to 197·7)	0·1 (0·0 to 0·1)	2·3 (2·3 to 2·3)
Iraq*	2015	142·7 (133·1 to 153·5)	47·3 (39·6 to 55·7)	94·9 (88·3 to 101·6)	0·0 (0·0 to 0·0)	0·5 (0·5 to 0·5)	320·1 (298·5 to 344·1)	106·2 (88·8 to 124·8)	212·9 (198·1 to 227·8)	0·1 (0·0 to 0·1)	1·0 (1·0 to 1·0)
Iraq*	2016	160·7 (148·8 to 173·8)	53·4 (45·1 to 62·7)	106·9 (98·7 to 114·7)	0·0 (0·0 to 0·0)	0·4 (0·4 to 0·4)	360·3 (333·7 to 389·8)	119·8 (101·1 to 140·5)	239·6 (221·2 to 257·1)	0·1 (0·0 to 0·1)	0·9 (0·9 to 0·9)
Iraq*	2017	167·1 (154·4 to 181·7)	61·3 (52·0 to 71·4)	105·4 (96·9 to 114·2)	0·0 (0·0 to 0·0)	0·5 (0·5 to 0·5)	374·8 (346·2 to 407·4)	137·4 (116·7 to 160·2)	236·2 (217·2 to 256·2)	0·1 (0·0 to 0·1)	1·1 (1·1 to 1·1)
Iraq*	2018	169·0 (155·4 to 183·6)	68·5 (57·7 to 80·3)	99·9 (91·7 to 109·2)	0·0 (0·0 to 0·0)	0·6 (0·6 to 0·6)	378·9 (348·5 to 411·8)	153·7 (129·5 to 180·1)	223·9 (205·6 to 244·9)	0·1 (0·0 to 0·1)	1·3 (1·3 to 1·3)
Iraq*	2019	184·9 (168·4 to 203·0)	82·7 (68·9 to 97·9)	101·6 (93·3 to 111·1)	0·0 (0·0 to 0·0)	0·6 (0·6 to 0·6)	414·7 (377·6 to 455·3)	185·5 (154·6 to 219·5)	227·9 (209·3 to 249·2)	0·1 (0·0 to 0·1)	1·3 (1·3 to 1·3)

Iraq*	2020	204·4 (182·4 to 228·8)	108·3 (88·4 to 131·6)	95·3 (87·6 to 104·3)	0·0 (0·0 to 0·0)	0·8 (0·8 to 0·8)	458·3 (409·1 to 513·1)	242·7 (198·1 to 295·2)	213·7 (196·4 to 233·9)	0·1 (0·0 to 0·1)	1·8 (1·7 to 1·8)
Iraq*	2021	197·5 (176·9 to 221·4)	104·8 (85·5 to 127·4)	92·0 (84·5 to 100·7)	0·0 (0·0 to 0·0)	0·8 (0·7 to 0·8)	443·0 (396·7 to 496·5)	234·9 (191·7 to 285·7)	206·3 (189·6 to 225·8)	0·1 (0·0 to 0·1)	1·7 (1·7 to 1·8)
Iraq*	2022	176·9 (157·9 to 198·7)	83·4 (65·8 to 103·1)	93·0 (85·5 to 101·8)	0·0 (0·0 to 0·0)	0·6 (0·5 to 0·6)	396·8 (354·1 to 445·5)	186·9 (147·6 to 231·2)	208·5 (191·7 to 228·2)	0·1 (0·0 to 0·1)	1·3 (1·2 to 1·4)
Iraq*	2023	180·6 (160·5 to 204·2)	86·2 (67·4 to 108·6)	93·8 (86·3 to 102·7)	0·0 (0·0 to 0·0)	0·6 (0·5 to 0·6)	405·0 (359·9 to 457·8)	193·2 (151·1 to 243·4)	210·4 (193·5 to 230·4)	0·1 (0·1 to 0·1)	1·3 (1·2 to 1·4)
Iraq*	2024	184·5 (162·7 to 209·7)	89·2 (68·6 to 113·3)	94·7 (87·1 to 103·6)	0·0 (0·0 to 0·0)	0·6 (0·5 to 0·6)	413·7 (364·9 to 470·3)	200·0 (153·8 to 254·1)	212·3 (195·3 to 232·4)	0·1 (0·1 to 0·1)	1·3 (1·2 to 1·4)
Iraq*	2025	188·3 (165·5 to 215·6)	92·1 (70·5 to 117·8)	95·5 (87·9 to 104·5)	0·0 (0·0 to 0·1)	0·6 (0·5 to 0·6)	422·2 (371·0 to 483·4)	206·6 (158·0 to 264·2)	214·2 (197·1 to 234·4)	0·1 (0·1 to 0·1)	1·3 (1·2 to 1·4)
Iraq*	2026	192·1 (167·3 to 221·0)	95·0 (72·0 to 122·4)	96·4 (88·7 to 105·6)	0·0 (0·0 to 0·1)	0·6 (0·5 to 0·6)	430·7 (375·1 to 495·6)	213·1 (161·5 to 274·4)	216·2 (198·8 to 236·8)	0·1 (0·1 to 0·1)	1·3 (1·2 to 1·4)
Iraq*	2027	196·5 (170·5 to 227·2)	98·4 (74·6 to 128·6)	97·5 (89·6 to 106·7)	0·0 (0·0 to 0·1)	0·6 (0·5 to 0·6)	440·6 (382·3 to 509·5)	220·6 (167·2 to 288·4)	218·6 (201·0 to 239·3)	0·1 (0·1 to 0·1)	1·3 (1·2 to 1·4)
Iraq*	2028	199·7 (172·2 to 232·1)	100·5 (75·0 to 133·2)	98·6 (90·4 to 107·8)	0·0 (0·0 to 0·1)	0·6 (0·5 to 0·7)	447·7 (386·2 to 520·5)	225·3 (168·2 to 298·8)	221·0 (202·7 to 241·8)	0·1 (0·1 to 0·1)	1·3 (1·2 to 1·5)
Iraq*	2029	203·9 (175·6 to 237·1)	103·5 (76·0 to 137·2)	99·8 (91·5 to 109·2)	0·0 (0·0 to 0·1)	0·6 (0·5 to 0·7)	457·3 (393·7 to 531·6)	232·0 (170·5 to 307·6)	223·9 (205·2 to 244·9)	0·1 (0·1 to 0·1)	1·3 (1·2 to 1·5)
Iraq*	2030	208·4 (178·5 to 243·2)	106·6 (78·0 to 142·0)	101·1 (92·7 to 110·6)	0·1 (0·0 to 0·1)	0·6 (0·5 to 0·6)	467·3 (400·2 to 545·5)	239·1 (175·0 to 318·4)	226·8 (207·9 to 248·0)	0·1 (0·1 to 0·1)	1·3 (1·2 to 1·5)
Iraq*	2031	214·0 (183·7 to 251·2)	111·0 (81·6 to 148·3)	102·4 (93·9 to 111·9)	0·1 (0·0 to 0·1)	0·6 (0·5 to 0·7)	479·9 (412·0 to 563·3)	248·9 (183·0 to 332·5)	229·6 (210·5 to 251·0)	0·1 (0·1 to 0·2)	1·3 (1·2 to 1·5)
Iraq*	2032	219·3 (187·4 to 257·4)	114·9 (85·6 to 153·6)	103·7 (95·1 to 113·3)	0·1 (0·0 to 0·1)	0·6 (0·5 to 0·7)	491·7 (420·3 to 577·1)	257·7 (191·9 to 344·4)	232·6 (213·3 to 254·2)	0·1 (0·1 to 0·2)	1·3 (1·2 to 1·5)
Iraq*	2033	224·3 (190·7 to 264·4)	118·8 (87·6 to 158·6)	104·9 (96·3 to 114·6)	0·1 (0·0 to 0·1)	0·6 (0·5 to 0·7)	503·0 (427·5 to 592·9)	266·3 (196·5 to 355·6)	235·2 (215·8 to 257·1)	0·1 (0·1 to 0·2)	1·3 (1·2 to 1·5)

Iraq*	2034	230·3 (194·7 to 273·7)	123·4 (89·3 to 167·5)	106·2 (97·4 to 116·1)	0·1 (0·0 to 0·1)	0·6 (0·5 to 0·7)	516·4 (436·5 to 613·8)	276·7 (200·2 to 375·7)	238·2 (218·5 to 260·4)	0·1 (0·1 to 0·2)	1·3 (1·2 to 1·5)
Iraq*	2035	236·6 (199·6 to 282·9)	128·3 (91·3 to 174·3)	107·6 (98·5 to 117·7)	0·1 (0·0 to 0·1)	0·6 (0·5 to 0·7)	530·6 (447·7 to 634·3)	287·7 (204·7 to 390·9)	241·4 (220·9 to 264·0)	0·1 (0·1 to 0·2)	1·3 (1·2 to 1·5)
Iraq*	2036	242·4 (201·1 to 289·9)	132·9 (93·0 to 180·8)	108·8 (99·7 to 119·1)	0·1 (0·0 to 0·1)	0·6 (0·5 to 0·7)	543·5 (450·8 to 650·2)	298·0 (208·6 to 405·4)	244·0 (223·5 to 267·2)	0·2 (0·1 to 0·2)	1·3 (1·2 to 1·5)
Iraq*	2037	248·9 (205·6 to 300·4)	138·0 (95·7 to 189·3)	110·3 (100·8 to 120·8)	0·1 (0·1 to 0·1)	0·6 (0·5 to 0·7)	558·1 (461·0 to 673·6)	309·4 (214·6 to 424·4)	247·2 (226·0 to 270·8)	0·2 (0·1 to 0·2)	1·3 (1·2 to 1·5)
Iraq*	2038	255·1 (208·6 to 308·6)	142·8 (98·3 to 196·1)	111·7 (102·0 to 122·5)	0·1 (0·1 to 0·1)	0·6 (0·5 to 0·7)	572·1 (467·9 to 692·1)	320·2 (220·5 to 439·7)	250·5 (228·7 to 274·7)	0·2 (0·1 to 0·2)	1·3 (1·2 to 1·5)
Iraq*	2039	260·9 (213·0 to 318·0)	147·2 (99·7 to 204·2)	113·0 (103·0 to 124·0)	0·1 (0·1 to 0·1)	0·6 (0·5 to 0·7)	585·1 (477·6 to 713·0)	330·1 (223·5 to 457·9)	253·4 (231·0 to 278·0)	0·2 (0·1 to 0·2)	1·3 (1·2 to 1·5)
Iraq*	2040	266·8 (215·7 to 328·0)	151·9 (103·0 to 213·1)	114·3 (104·1 to 125·4)	0·1 (0·1 to 0·1)	0·6 (0·5 to 0·7)	598·4 (483·7 to 735·5)	340·6 (231·1 to 477·8)	256·2 (233·3 to 281·2)	0·2 (0·1 to 0·2)	1·3 (1·2 to 1·6)
Iraq*	2041	273·0 (219·2 to 336·5)	156·6 (104·8 to 220·1)	115·7 (105·3 to 127·1)	0·1 (0·1 to 0·1)	0·6 (0·5 to 0·7)	612·2 (491·6 to 754·5)	351·2 (235·1 to 493·5)	259·4 (236·1 to 284·9)	0·2 (0·1 to 0·3)	1·3 (1·2 to 1·6)
Iraq*	2042	278·5 (221·8 to 343·4)	160·9 (106·9 to 226·4)	116·9 (106·2 to 128·6)	0·1 (0·1 to 0·1)	0·6 (0·5 to 0·7)	624·6 (497·3 to 770·0)	360·9 (239·7 to 507·6)	262·1 (238·2 to 288·3)	0·2 (0·1 to 0·3)	1·4 (1·2 to 1·6)
Iraq*	2043	283·9 (225·1 to 353·4)	164·9 (107·6 to 234·7)	118·3 (107·4 to 130·3)	0·1 (0·1 to 0·1)	0·6 (0·5 to 0·7)	636·7 (504·8 to 792·5)	369·8 (241·3 to 526·4)	265·3 (240·8 to 292·1)	0·2 (0·1 to 0·3)	1·4 (1·2 to 1·6)
Iraq*	2044	289·3 (228·7 to 361·7)	169·0 (111·1 to 240·8)	119·6 (108·4 to 131·8)	0·1 (0·1 to 0·1)	0·6 (0·5 to 0·7)	648·8 (512·8 to 811·2)	379·1 (249·2 to 540·0)	268·2 (243·1 to 295·6)	0·2 (0·2 to 0·3)	1·4 (1·2 to 1·7)
Iraq*	2045	294·6 (232·5 to 368·1)	172·9 (114·1 to 247·9)	120·9 (109·5 to 133·3)	0·1 (0·1 to 0·1)	0·6 (0·5 to 0·8)	660·6 (521·3 to 825·5)	387·8 (255·8 to 555·9)	271·2 (245·5 to 299·0)	0·2 (0·2 to 0·3)	1·4 (1·2 to 1·7)
Iraq*	2046	299·9 (235·2 to 374·8)	176·8 (115·0 to 253·4)	122·3 (110·6 to 134·9)	0·1 (0·1 to 0·1)	0·6 (0·5 to 0·8)	672·4 (527·4 to 840·5)	396·4 (257·9 to 568·3)	274·3 (247·9 to 302·6)	0·2 (0·2 to 0·3)	1·4 (1·2 to 1·7)
Iraq*	2047	305·3 (237·2 to 383·1)	180·9 (116·1 to 259·0)	123·6 (111·6 to 136·4)	0·1 (0·1 to 0·2)	0·6 (0·5 to 0·8)	684·6 (531·9 to 859·0)	405·7 (260·2 to 580·7)	277·1 (250·3 to 305·8)	0·3 (0·2 to 0·4)	1·4 (1·2 to 1·8)

Iraq*	2048	310·4 (239·1 to 389·5)	184·7 (116·1 to 265·7)	124·9 (112·6 to 137·9)	0·1 (0·1 to 0·2)	0·7 (0·5 to 0·8)	696·0 (536·0 to 873·4)	414·3 (260·3 to 595·8)	280·1 (252·5 to 309·1)	0·3 (0·2 to 0·4)	1·5 (1·2 to 1·8)
Iraq*	2049	316·1 (242·5 to 397·6)	189·0 (119·6 to 272·2)	126·4 (113·7 to 139·5)	0·1 (0·1 to 0·2)	0·7 (0·6 to 0·8)	708·9 (543·8 to 891·5)	423·7 (268·2 to 610·4)	283·4 (255·0 to 312·8)	0·3 (0·2 to 0·4)	1·5 (1·2 to 1·8)
Iraq*	2050	322·4 (246·2 to 409·1)	193·5 (121·1 to 280·7)	128·1 (115·2 to 141·3)	0·1 (0·1 to 0·2)	0·7 (0·5 to 0·8)	722·9 (552·0 to 917·3)	433·9 (271·6 to 629·4)	287·2 (258·3 to 316·9)	0·3 (0·2 to 0·4)	1·5 (1·2 to 1·9)
Iceland	1995	3031·3 (2842·7 to 3220·7)	2301·4 (2123·5 to 2484·0)	729·7 (688·8 to 774·9)	0·2 (0·0 to 0·4)	0·0 (0·0 to 0·0)	2887·7 (2708·1 to 3068·2)	2192·4 (2022·9 to 2366·4)	695·1 (656·2 to 738·3)	0·2 (0·0 to 0·4)	0·0 (0·0 to 0·0)
Iceland	1996	3123·1 (2978·8 to 3277·2)	2385·9 (2239·7 to 2528·6)	737·1 (704·1 to 770·3)	0·2 (0·0 to 0·4)	0·0 (0·0 to 0·0)	2975·3 (2837·8 to 3122·0)	2272·9 (2133·6 to 2408·9)	702·2 (670·8 to 733·9)	0·2 (0·0 to 0·4)	0·0 (0·0 to 0·0)
Iceland	1997	3285·3 (3141·6 to 3430·1)	2534·2 (2399·2 to 2669·6)	750·9 (719·9 to 783·5)	0·2 (0·0 to 0·4)	0·0 (0·0 to 0·0)	3129·7 (2992·8 to 3267·7)	2414·2 (2285·6 to 2543·2)	715·4 (685·8 to 746·4)	0·2 (0·0 to 0·4)	0·0 (0·0 to 0·0)
Iceland	1998	3584·1 (3439·7 to 3724·3)	2810·2 (2665·5 to 2947·0)	773·8 (743·1 to 807·3)	0·1 (0·0 to 0·4)	0·0 (0·0 to 0·0)	3414·4 (3276·8 to 3547·9)	2677·1 (2539·3 to 2807·4)	737·1 (707·9 to 769·1)	0·1 (0·0 to 0·4)	0·0 (0·0 to 0·0)
Iceland	1999	3892·5 (3740·9 to 4041·5)	3101·6 (2951·2 to 3243·2)	790·8 (759·0 to 823·9)	0·1 (0·0 to 0·4)	0·0 (0·0 to 0·0)	3708·2 (3563·8 to 3850·1)	2954·7 (2811·5 to 3089·7)	753·4 (723·0 to 784·8)	0·1 (0·0 to 0·4)	0·0 (0·0 to 0·0)
Iceland	2000	4067·3 (3922·7 to 4225·3)	3268·3 (3116·7 to 3422·8)	798·9 (767·9 to 830·5)	0·1 (0·0 to 0·5)	0·0 (0·0 to 0·0)	3874·7 (3737·0 to 4025·3)	3113·6 (2969·1 to 3260·8)	761·0 (731·6 to 791·2)	0·1 (0·0 to 0·4)	0·0 (0·0 to 0·0)
Iceland	2001	4231·0 (4079·6 to 4395·1)	3430·0 (3280·4 to 3587·1)	800·9 (771·4 to 832·2)	0·0 (0·0 to 0·5)	0·0 (0·0 to 0·0)	4030·6 (3886·5 to 4187·0)	3267·6 (3125·0 to 3417·3)	763·0 (734·8 to 792·8)	0·0 (0·0 to 0·4)	0·0 (0·0 to 0·0)
Iceland	2002	4369·6 (4213·4 to 4530·0)	3589·6 (3437·9 to 3745·0)	780·0 (751·4 to 808·9)	0·0 (0·0 to 0·0)	0·0 (0·0 to 0·0)	4162·7 (4013·9 to 4315·5)	3419·7 (3275·1 to 3567·6)	743·0 (715·8 to 770·6)	0·0 (0·0 to 0·0)	0·0 (0·0 to 0·0)
Iceland	2003	4699·1 (4539·7 to 4852·9)	3787·1 (3631·2 to 3934·0)	849·2 (819·7 to 881·3)	62·8 (59·5 to 66·1)	0·0 (0·0 to 0·0)	4476·6 (4324·8 to 4623·1)	3607·8 (3459·3 to 3747·7)	809·0 (780·9 to 839·6)	59·8 (56·7 to 63·0)	0·0 (0·0 to 0·0)
Iceland	2004	4799·2 (4645·1 to 4959·2)	3903·5 (3757·0 to 4062·7)	830·2 (802·1 to 860·5)	65·4 (62·9 to 68·1)	0·0 (0·0 to 0·0)	4571·9 (4425·1 to 4724·4)	3718·7 (3579·1 to 3870·3)	790·9 (764·1 to 819·8)	62·3 (59·9 to 64·8)	0·0 (0·0 to 0·0)
Iceland	2005	4855·6 (4705·1 to 5014·2)	3966·6 (3822·7 to 4125·2)	821·3 (793·2 to 847·8)	67·7 (64·9 to 70·4)	0·0 (0·0 to 0·0)	4625·7 (4482·3 to 4776·8)	3778·8 (3641·6 to 3929·9)	782·4 (755·7 to 807·7)	64·5 (61·8 to 67·1)	0·0 (0·0 to 0·0)

Iceland	2006	4814·9 (4668·4 to 4970·0)	3942·1 (3799·2 to 4097·2)	803·0 (774·9 to 829·0)	69·8 (67·3 to 72·4)	0·0 (0·0 to 0·0)	4586·9 (4447·3 to 4734·6)	3755·5 (3619·3 to 3903·2)	765·0 (738·2 to 789·7)	66·4 (64·1 to 68·9)	0·0 (0·0 to 0·0)
Iceland	2007	4786·7 (4635·3 to 4937·4)	3924·2 (3781·6 to 4073·8)	791·6 (763·5 to 818·7)	70·9 (68·4 to 73·5)	0·0 (0·0 to 0·0)	4560·1 (4415·8 to 4703·6)	3738·4 (3602·5 to 3880·9)	754·1 (727·3 to 779·9)	67·6 (65·2 to 70·1)	0·0 (0·0 to 0·0)
Iceland	2008	5415·7 (5239·6 to 5587·0)	4505·7 (4332·6 to 4678·6)	835·5 (803·9 to 866·6)	74·5 (71·7 to 77·0)	0·0 (0·0 to 0·0)	5159·2 (4991·5 to 5322·5)	4292·3 (4127·5 to 4457·1)	796·0 (765·8 to 825·6)	70·9 (68·3 to 73·4)	0·0 (0·0 to 0·0)
Iceland	2009	4638·2 (4478·0 to 4803·1)	3781·5 (3624·0 to 3942·4)	791·1 (760·9 to 820·4)	65·7 (63·2 to 68·0)	0·0 (0·0 to 0·0)	4418·6 (4266·0 to 4575·6)	3602·4 (3452·4 to 3755·7)	753·6 (724·9 to 781·6)	62·6 (60·2 to 64·8)	0·0 (0·0 to 0·0)
Iceland	2010	4471·2 (4314·3 to 4641·2)	3611·0 (3456·8 to 3777·4)	797·4 (767·7 to 826·7)	62·8 (60·4 to 65·1)	0·0 (0·0 to 0·0)	4259·5 (4110·0 to 4421·4)	3440·0 (3293·2 to 3598·6)	759·7 (731·4 to 787·6)	59·9 (57·6 to 62·0)	0·0 (0·0 to 0·0)
Iceland	2011	4341·3 (4192·5 to 4502·4)	3475·8 (3334·3 to 3633·9)	802·2 (772·4 to 832·8)	63·3 (60·9 to 65·8)	0·0 (0·0 to 0·0)	4135·7 (3994·0 to 4289·2)	3311·2 (3176·4 to 3461·8)	764·2 (735·9 to 793·4)	60·3 (58·0 to 62·7)	0·0 (0·0 to 0·0)
Iceland	2012	4389·5 (4243·2 to 4542·6)	3510·3 (3365·4 to 3663·2)	813·9 (781·7 to 844·5)	65·3 (62·8 to 67·7)	0·0 (0·0 to 0·0)	4181·6 (4042·3 to 4327·5)	3344·1 (3206·0 to 3489·8)	775·4 (744·7 to 804·6)	62·2 (59·8 to 64·5)	0·0 (0·0 to 0·0)
Iceland	2013	4456·6 (4316·7 to 4608·3)	3567·1 (3430·7 to 3713·6)	822·0 (791·2 to 852·1)	67·4 (65·0 to 70·0)	0·0 (0·0 to 0·0)	4245·6 (4112·3 to 4390·1)	3398·2 (3268·2 to 3537·7)	783·1 (753·7 to 811·8)	64·3 (62·0 to 66·7)	0·0 (0·0 to 0·0)
Iceland	2014	4610·9 (4454·5 to 4768·4)	3712·0 (3561·8 to 3866·0)	828·9 (800·4 to 858·9)	70·0 (67·5 to 72·5)	0·0 (0·0 to 0·0)	4392·6 (4243·6 to 4542·7)	3536·3 (3393·1 to 3683·0)	789·6 (762·5 to 818·2)	66·7 (64·3 to 69·1)	0·0 (0·0 to 0·0)
Iceland	2015	4635·6 (4485·0 to 4802·5)	3742·1 (3593·8 to 3901·0)	821·1 (792·7 to 851·9)	72·4 (70·0 to 74·8)	0·0 (0·0 to 0·0)	4416·1 (4272·6 to 4575·1)	3564·9 (3423·6 to 3716·3)	782·3 (755·1 to 811·5)	68·9 (66·7 to 71·3)	0·0 (0·0 to 0·0)
Iceland	2016	5053·5 (4889·9 to 5225·6)	4127·5 (3967·2 to 4300·3)	845·3 (815·3 to 875·7)	80·7 (78·0 to 83·2)	0·0 (0·0 to 0·0)	4814·2 (4658·4 to 4978·2)	3932·1 (3779·3 to 4096·7)	805·2 (776·7 to 834·2)	76·9 (74·3 to 79·3)	0·0 (0·0 to 0·0)
Iceland	2017	5231·0 (5069·5 to 5399·2)	4285·8 (4127·7 to 4448·7)	857·6 (825·1 to 890·7)	87·5 (84·7 to 90·3)	0·0 (0·0 to 0·0)	4983·3 (4829·5 to 5143·6)	4082·9 (3932·2 to 4238·1)	817·0 (786·0 to 848·5)	83·4 (80·7 to 86·0)	0·0 (0·0 to 0·0)
Iceland	2018	5452·0 (5243·3 to 5667·9)	4482·5 (4272·9 to 4696·6)	876·7 (835·6 to 920·5)	92·8 (89·6 to 96·4)	0·0 (0·0 to 0·0)	5193·9 (4995·0 to 5399·5)	4270·2 (4070·5 to 4474·2)	835·2 (796·0 to 876·9)	88·4 (85·4 to 91·8)	0·0 (0·0 to 0·0)
Iceland	2019	5486·1 (5255·8 to 5722·4)	4513·4 (4281·8 to 4748·3)	877·6 (836·1 to 921·9)	95·1 (91·7 to 98·9)	0·0 (0·0 to 0·0)	5226·3 (5006·9 to 5451·4)	4299·7 (4079·1 to 4523·5)	836·1 (796·5 to 878·3)	90·6 (87·3 to 94·2)	0·0 (0·0 to 0·0)

Iceland	2020	5835·6 (5529·7 to 6130·2)	4919·9 (4611·0 to 5208·7)	824·4 (785·7 to 865·9)	91·3 (87·8 to 95·1)	0·0 (0·0 to 0·0)	5559·3 (5267·9 to 5840·0)	4687·0 (4392·7 to 4962·1)	785·4 (748·5 to 824·9)	86·9 (83·6 to 90·6)	0·0 (0·0 to 0·0)
Iceland	2021	5670·1 (5373·0 to 5956·4)	4779·5 (4479·4 to 5060·1)	800·1 (762·8 to 840·1)	90·5 (86·9 to 94·7)	0·0 (0·0 to 0·0)	5401·6 (5118·6 to 5674·4)	4553·2 (4267·3 to 4820·5)	762·2 (726·7 to 800·3)	86·3 (82·8 to 90·2)	0·0 (0·0 to 0·0)
Iceland	2022	5201·4 (4895·1 to 5506·8)	4306·5 (3998·1 to 4617·2)	802·1 (765·0 to 842·3)	92·8 (88·7 to 97·3)	0·0 (0·0 to 0·0)	4955·1 (4663·3 to 5246·1)	4102·6 (3808·8 to 4398·6)	764·1 (728·8 to 802·4)	88·4 (84·5 to 92·7)	0·0 (0·0 to 0·0)
Iceland	2023	5271·9 (4954·3 to 5604·6)	4373·1 (4058·1 to 4709·3)	803·7 (766·7 to 843·9)	95·1 (90·4 to 100·5)	0·0 (0·0 to 0·0)	5022·3 (4719·7 to 5339·2)	4166·0 (3866·0 to 4486·3)	765·7 (730·4 to 803·9)	90·6 (86·2 to 95·8)	0·0 (0·0 to 0·0)
Iceland	2024	5347·1 (4983·8 to 5715·4)	4444·2 (4078·2 to 4816·1)	805·4 (768·2 to 845·8)	97·5 (92·3 to 103·7)	0·0 (0·0 to 0·0)	5093·9 (4747·8 to 5444·8)	4233·8 (3885·1 to 4588·0)	767·3 (731·8 to 805·8)	92·8 (87·9 to 98·8)	0·0 (0·0 to 0·0)
Iceland	2025	5414·0 (5028·4 to 5802·5)	4508·2 (4115·3 to 4898·3)	806·0 (769·2 to 846·9)	99·8 (94·2 to 107·2)	0·0 (0·0 to 0·0)	5157·6 (4790·3 to 5527·7)	4294·8 (3920·4 to 4666·4)	767·8 (732·8 to 806·8)	95·1 (89·7 to 102·1)	0·0 (0·0 to 0·0)
Iceland	2026	5482·0 (5074·5 to 5909·5)	4572·7 (4157·6 to 4997·9)	807·0 (769·9 to 848·6)	102·3 (96·2 to 110·8)	0·0 (0·0 to 0·0)	5222·5 (4834·2 to 5629·7)	4356·2 (3960·8 to 4761·3)	768·8 (733·4 to 808·4)	97·4 (91·7 to 105·6)	0·0 (0·0 to 0·0)
Iceland	2027	5505·2 (5084·9 to 5936·8)	4596·9 (4172·1 to 5026·2)	803·9 (766·0 to 845·4)	104·3 (97·8 to 114·1)	0·0 (0·0 to 0·0)	5244·5 (4844·1 to 5655·7)	4379·3 (3974·5 to 4788·2)	765·8 (729·7 to 805·3)	99·4 (93·1 to 108·7)	0·0 (0·0 to 0·0)
Iceland	2028	5525·3 (5096·4 to 5974·0)	4617·1 (4169·1 to 5069·7)	801·4 (764·0 to 842·5)	106·7 (99·6 to 117·5)	0·0 (0·0 to 0·0)	5263·6 (4855·0 to 5691·2)	4398·5 (3971·7 to 4829·6)	763·5 (727·8 to 802·6)	101·7 (94·9 to 111·9)	0·0 (0·0 to 0·0)
Iceland	2029	5552·3 (5112·8 to 6027·0)	4642·1 (4197·5 to 5110·5)	800·7 (763·8 to 841·6)	109·4 (101·7 to 121·4)	0·0 (0·0 to 0·0)	5289·4 (4870·7 to 5741·6)	4422·3 (3998·8 to 4868·5)	762·8 (727·6 to 801·7)	104·3 (96·9 to 115·7)	0·0 (0·0 to 0·0)
Iceland	2030	5593·4 (5128·8 to 6086·0)	4679·2 (4212·2 to 5173·2)	801·8 (764·7 to 842·5)	112·4 (104·0 to 125·7)	0·0 (0·0 to 0·0)	5328·6 (4886·0 to 5797·9)	4457·6 (4012·8 to 4928·2)	763·9 (728·5 to 802·6)	107·1 (99·1 to 119·8)	0·0 (0·0 to 0·0)
Iceland	2031	5647·0 (5179·3 to 6159·0)	4726·1 (4256·7 to 5232·8)	805·1 (767·8 to 845·8)	115·8 (106·6 to 130·4)	0·0 (0·0 to 0·0)	5379·6 (4934·1 to 5867·4)	4502·3 (4055·1 to 4985·1)	767·0 (731·5 to 805·7)	110·3 (101·5 to 124·3)	0·0 (0·0 to 0·0)
Iceland	2032	5702·5 (5220·0 to 6214·0)	4774·8 (4288·5 to 5268·7)	808·5 (771·0 to 849·7)	119·2 (109·2 to 135·3)	0·0 (0·0 to 0·0)	5432·5 (4972·8 to 5919·8)	4548·8 (4085·4 to 5019·2)	770·2 (734·5 to 809·4)	113·5 (104·0 to 128·9)	0·0 (0·0 to 0·0)
Iceland	2033	5759·1 (5261·9 to 6272·9)	4824·1 (4328·0 to 5342·2)	812·3 (774·2 to 853·9)	122·7 (111·6 to 139·9)	0·0 (0·0 to 0·0)	5486·4 (5012·7 to 5975·9)	4595·7 (4123·0 to 5089·3)	773·8 (737·5 to 813·5)	116·9 (106·3 to 133·3)	0·0 (0·0 to 0·0)

Iceland	2034	5814·5 (5303·4 to 6345·8)	4872·7 (4358·6 to 5399·9)	815·8 (777·1 to 857·9)	126·1 (113·9 to 144·7)	0·0 (0·0 to 0·0)	5539·2 (5052·3 to 6045·3)	4641·9 (4152·2 to 5144·2)	777·1 (740·3 to 817·3)	120·2 (108·5 to 137·9)	0·0 (0·0 to 0·0)
Iceland	2035	5870·5 (5332·1 to 6417·7)	4921·4 (4386·4 to 5470·8)	819·5 (781·0 to 862·1)	129·7 (116·2 to 149·9)	0·0 (0·0 to 0·0)	5592·5 (5079·6 to 6113·8)	4688·3 (4178·7 to 5211·8)	780·7 (744·0 to 821·3)	123·5 (110·7 to 142·8)	0·0 (0·0 to 0·0)
Iceland	2036	5927·8 (5385·1 to 6480·8)	4971·3 (4424·2 to 5530·4)	823·2 (784·2 to 866·3)	133·3 (118·6 to 154·9)	0·0 (0·0 to 0·0)	5647·1 (5130·1 to 6173·9)	4735·9 (4214·7 to 5268·5)	784·2 (747·0 to 825·3)	127·0 (112·9 to 147·5)	0·0 (0·0 to 0·0)
Iceland	2037	5986·7 (5432·2 to 6551·8)	5023·1 (4472·2 to 5574·3)	826·6 (787·1 to 870·2)	137·0 (121·0 to 160·0)	0·0 (0·0 to 0·0)	5703·3 (5175·0 to 6241·6)	4785·3 (4260·5 to 5310·4)	787·5 (749·8 to 829·0)	130·5 (115·3 to 152·5)	0·0 (0·0 to 0·0)
Iceland	2038	6046·8 (5478·7 to 6610·2)	5075·4 (4520·6 to 5635·1)	830·4 (790·4 to 874·5)	140·9 (123·4 to 165·5)	0·0 (0·0 to 0·0)	5760·5 (5219·2 to 6297·2)	4835·1 (4306·5 to 5368·3)	791·1 (752·9 to 833·1)	134·3 (117·6 to 157·6)	0·0 (0·0 to 0·0)
Iceland	2039	6106·0 (5530·2 to 6703·7)	5126·9 (4551·6 to 5701·3)	834·1 (793·7 to 878·6)	144·9 (126·1 to 171·1)	0·0 (0·0 to 0·0)	5816·8 (5268·4 to 6386·2)	4884·1 (4336·1 to 5431·3)	794·6 (756·1 to 837·0)	138·1 (120·1 to 163·0)	0·0 (0·0 to 0·0)
Iceland	2040	6170·2 (5579·5 to 6746·7)	5182·6 (4597·6 to 5767·8)	838·4 (797·9 to 883·0)	149·1 (128·6 to 177·4)	0·0 (0·0 to 0·0)	5878·0 (5315·3 to 6427·3)	4937·2 (4379·9 to 5494·7)	798·7 (760·1 to 841·2)	142·1 (122·5 to 169·0)	0·0 (0·0 to 0·0)
Iceland	2041	6237·2 (5641·8 to 6853·9)	5240·9 (4635·6 to 5865·7)	842·9 (802·3 to 888·2)	153·5 (131·3 to 184·2)	0·0 (0·0 to 0·0)	5941·9 (5374·7 to 6529·4)	4992·7 (4416·1 to 5587·9)	802·9 (764·3 to 846·1)	146·2 (125·1 to 175·4)	0·0 (0·0 to 0·0)
Iceland	2042	6310·4 (5692·4 to 6935·3)	5304·7 (4691·5 to 5921·9)	847·6 (807·0 to 893·4)	158·0 (134·1 to 191·3)	0·0 (0·0 to 0·0)	6011·6 (5422·8 to 6606·9)	5053·5 (4469·4 to 5641·5)	807·5 (768·8 to 851·1)	150·5 (127·8 to 182·2)	0·0 (0·0 to 0·0)
Iceland	2043	6383·7 (5754·1 to 7030·0)	5368·5 (4722·6 to 6016·0)	852·6 (811·4 to 898·8)	162·7 (137·0 to 198·6)	0·0 (0·0 to 0·0)	6081·5 (5481·6 to 6697·1)	5114·3 (4499·0 to 5731·2)	812·2 (773·0 to 856·3)	155·0 (130·5 to 189·2)	0·0 (0·0 to 0·0)
Iceland	2044	6456·0 (5818·6 to 7109·8)	5430·8 (4791·9 to 6093·3)	857·7 (816·0 to 904·2)	167·5 (140·0 to 206·2)	0·0 (0·0 to 0·0)	6150·3 (5543·1 to 6773·1)	5173·6 (4565·0 to 5804·8)	817·1 (777·3 to 861·3)	159·6 (133·3 to 196·4)	0·0 (0·0 to 0·0)
Iceland	2045	6531·6 (5860·1 to 7212·1)	5496·4 (4826·5 to 6190·2)	862·8 (820·5 to 909·2)	172·4 (142·8 to 213·4)	0·0 (0·0 to 0·0)	6222·3 (5582·6 to 6870·6)	5236·1 (4597·9 to 5897·0)	822·0 (781·7 to 866·2)	164·3 (136·0 to 203·3)	0·0 (0·0 to 0·0)
Iceland	2046	6604·8 (5921·1 to 7281·0)	5559·7 (4869·2 to 6237·3)	867·7 (824·8 to 914·0)	177·5 (145·5 to 221·3)	0·0 (0·0 to 0·0)	6292·1 (5640·7 to 6936·3)	5296·4 (4638·6 to 5942·0)	826·6 (785·8 to 870·7)	169·1 (138·6 to 210·8)	0·0 (0·0 to 0·0)
Iceland	2047	6679·4 (5975·3 to 7374·7)	5624·1 (4914·5 to 6317·2)	872·6 (829·5 to 918·9)	182·6 (147·8 to 229·6)	0·0 (0·0 to 0·0)	6363·1 (5692·4 to 7025·5)	5357·8 (4681·8 to 6018·1)	831·3 (790·2 to 875·4)	174·0 (140·8 to 218·7)	0·0 (0·0 to 0·0)

Iceland	2048	6764·3 (6033·5 to 7491·2)	5697·5 (4966·6 to 6422·4)	878·7 (835·4 to 925·2)	188·1 (150·5 to 238·6)	0·0 (0·0 to 0·0)	6444·0 (5747·8 to 7136·5)	5427·7 (4731·5 to 6118·3)	837·1 (795·8 to 881·4)	179·2 (143·4 to 227·3)	0·0 (0·0 to 0·0)
Iceland	2049	6849·3 (6103·7 to 7585·5)	5771·0 (5032·8 to 6480·5)	884·6 (840·8 to 931·6)	193·7 (153·6 to 247·8)	0·0 (0·0 to 0·0)	6525·0 (5814·7 to 7226·3)	5497·7 (4794·5 to 6173·6)	842·7 (801·0 to 887·5)	184·6 (146·3 to 236·0)	0·0 (0·0 to 0·0)
Iceland	2050	6930·7 (6188·6 to 7676·4)	5841·5 (5112·9 to 6562·8)	889·8 (845·4 to 937·3)	199·3 (156·6 to 255·8)	0·0 (0·0 to 0·0)	6602·5 (5895·6 to 7312·9)	5564·9 (4870·8 to 6252·1)	847·7 (805·3 to 892·9)	189·9 (149·2 to 243·7)	0·0 (0·0 to 0·0)
Israel	1995	2131·6 (2068·0 to 2201·6)	1437·5 (1388·9 to 1490·7)	641·6 (603·8 to 682·2)	52·4 (40·4 to 66·3)	0·0 (0·0 to 0·0)	2006·8 (1947·0 to 2072·7)	1353·4 (1307·7 to 1403·4)	604·1 (568·5 to 642·2)	49·3 (38·1 to 62·4)	0·0 (0·0 to 0·0)
Israel	1996	2196·2 (2147·9 to 2249·6)	1490·3 (1450·0 to 1528·3)	646·6 (616·2 to 677·5)	59·3 (47·4 to 72·6)	0·0 (0·0 to 0·0)	2067·7 (2022·2 to 2117·9)	1403·1 (1365·2 to 1438·9)	608·8 (580·1 to 637·9)	55·8 (44·6 to 68·3)	0·0 (0·0 to 0·0)
Israel	1997	2225·4 (2178·0 to 2273·2)	1502·1 (1466·3 to 1542·6)	651·6 (621·2 to 681·3)	71·6 (59·7 to 84·8)	0·0 (0·0 to 0·0)	2095·1 (2050·5 to 2140·1)	1414·2 (1380·5 to 1452·4)	613·5 (584·9 to 641·4)	67·4 (56·2 to 79·8)	0·0 (0·0 to 0·0)
Israel	1998	2205·6 (2158·5 to 2255·4)	1466·1 (1427·6 to 1503·1)	655·8 (628·9 to 683·8)	83·8 (72·1 to 96·4)	0·0 (0·0 to 0·0)	2076·5 (2032·2 to 2123·5)	1380·3 (1344·0 to 1415·1)	617·4 (592·1 to 643·8)	78·9 (67·9 to 90·8)	0·0 (0·0 to 0·0)
Israel	1999	2197·1 (2150·2 to 2247·1)	1435·4 (1399·4 to 1473·5)	663·5 (636·9 to 692·8)	98·2 (87·7 to 109·9)	0·0 (0·0 to 0·0)	2068·6 (2024·4 to 2115·6)	1351·4 (1317·5 to 1387·2)	624·7 (599·6 to 652·3)	92·5 (82·6 to 103·5)	0·0 (0·0 to 0·0)
Israel	2000	2265·3 (2221·9 to 2309·7)	1464·9 (1430·7 to 1501·4)	686·8 (660·2 to 713·9)	113·7 (104·8 to 124·5)	0·0 (0·0 to 0·0)	2132·7 (2091·9 to 2174·6)	1379·1 (1347·0 to 1413·5)	646·6 (621·6 to 672·1)	107·0 (98·6 to 117·2)	0·0 (0·0 to 0·0)
Israel	2001	2311·0 (2265·4 to 2358·2)	1490·1 (1455·0 to 1524·1)	696·7 (669·7 to 722·6)	124·2 (116·2 to 133·4)	0·0 (0·0 to 0·0)	2175·8 (2132·8 to 2220·2)	1402·9 (1369·9 to 1434·9)	656·0 (630·5 to 680·3)	116·9 (109·4 to 125·6)	0·0 (0·0 to 0·0)
Israel	2002	2325·4 (2282·2 to 2371·8)	1504·7 (1469·6 to 1537·5)	681·7 (655·7 to 708·0)	139·0 (130·7 to 148·5)	0·0 (0·0 to 0·0)	2189·3 (2148·7 to 2233·0)	1416·6 (1383·6 to 1447·5)	641·8 (617·3 to 666·6)	130·8 (123·0 to 139·8)	0·0 (0·0 to 0·0)
Israel	2003	2261·6 (2216·8 to 2305·7)	1441·1 (1405·7 to 1474·7)	668·1 (641·0 to 695·2)	152·5 (143·8 to 161·5)	0·0 (0·0 to 0·0)	2129·3 (2087·0 to 2170·8)	1356·8 (1323·5 to 1388·4)	629·0 (603·5 to 654·5)	143·5 (135·4 to 152·1)	0·0 (0·0 to 0·0)
Israel	2004	2272·8 (2228·7 to 2317·2)	1428·3 (1397·0 to 1462·3)	675·2 (649·6 to 702·9)	169·3 (160·6 to 178·8)	0·0 (0·0 to 0·0)	2139·8 (2098·3 to 2181·6)	1344·7 (1315·3 to 1376·8)	635·6 (611·6 to 661·8)	159·4 (151·2 to 168·4)	0·0 (0·0 to 0·0)
Israel	2005	2336·8 (2295·2 to 2380·3)	1454·1 (1422·2 to 1487·5)	684·5 (658·0 to 712·1)	198·1 (189·1 to 208·1)	0·0 (0·0 to 0·0)	2200·0 (2160·8 to 2241·0)	1369·0 (1339·0 to 1400·4)	644·5 (619·5 to 670·5)	186·5 (178·0 to 195·9)	0·0 (0·0 to 0·0)

Israel	2006	2417·1 (2372·8 to 2460·8)	1501·7 (1470·7 to 1533·6)	666·9 (640·1 to 693·9)	248·5 (238·5 to 258·9)	0·0 (0·0 to 0·0)	2275·7 (2234·0 to 2316·8)	1413·9 (1384·7 to 1443·8)	627·9 (602·6 to 653·3)	233·9 (224·6 to 243·7)	0·0 (0·0 to 0·0)
Israel	2007	2477·6 (2435·1 to 2522·3)	1529·2 (1494·7 to 1561·7)	661·4 (635·7 to 687·2)	287·0 (276·5 to 297·4)	0·0 (0·0 to 0·0)	2332·6 (2292·6 to 2374·7)	1439·7 (1407·2 to 1470·3)	622·7 (598·5 to 647·0)	270·2 (260·3 to 280·0)	0·0 (0·0 to 0·0)
Israel	2008	2537·9 (2493·4 to 2582·2)	1572·9 (1537·3 to 1607·5)	656·6 (631·2 to 682·1)	308·4 (298·3 to 319·3)	0·0 (0·0 to 0·0)	2389·4 (2347·5 to 2431·1)	1480·9 (1447·3 to 1513·4)	618·1 (594·3 to 642·1)	290·3 (280·9 to 300·6)	0·0 (0·0 to 0·0)
Israel	2009	2567·1 (2522·2 to 2611·1)	1603·8 (1568·9 to 1637·7)	649·1 (622·3 to 675·5)	314·2 (304·1 to 324·9)	0·0 (0·0 to 0·0)	2416·9 (2374·6 to 2458·3)	1510·0 (1477·1 to 1541·9)	611·1 (585·8 to 636·0)	295·8 (286·3 to 305·9)	0·0 (0·0 to 0·0)
Israel	2010	2618·4 (2574·3 to 2664·7)	1653·8 (1620·2 to 1687·4)	642·4 (616·5 to 669·1)	322·2 (311·4 to 333·1)	0·0 (0·0 to 0·0)	2465·2 (2423·6 to 2508·8)	1557·0 (1525·4 to 1588·7)	604·8 (580·5 to 629·9)	303·3 (293·2 to 313·6)	0·0 (0·0 to 0·0)
Israel	2011	2676·3 (2629·6 to 2721·5)	1698·4 (1664·1 to 1733·1)	643·3 (618·0 to 669·9)	334·6 (324·5 to 345·3)	0·0 (0·0 to 0·0)	2519·7 (2475·7 to 2562·2)	1599·0 (1566·7 to 1631·7)	605·6 (581·8 to 630·7)	315·0 (305·5 to 325·1)	0·0 (0·0 to 0·0)
Israel	2012	2747·8 (2704·3 to 2791·0)	1751·8 (1717·9 to 1786·3)	647·6 (623·0 to 673·5)	348·4 (337·9 to 360·1)	0·0 (0·0 to 0·0)	2587·0 (2546·1 to 2627·7)	1649·3 (1617·3 to 1681·7)	609·7 (586·5 to 634·1)	328·0 (318·1 to 339·0)	0·0 (0·0 to 0·0)
Israel	2013	2813·8 (2769·1 to 2860·3)	1804·1 (1769·5 to 1839·9)	652·4 (627·8 to 678·9)	357·3 (346·3 to 368·5)	0·0 (0·0 to 0·0)	2649·1 (2607·0 to 2692·9)	1698·6 (1665·9 to 1732·3)	614·2 (591·1 to 639·1)	336·4 (326·0 to 347·0)	0·0 (0·0 to 0·0)
Israel	2014	2852·4 (2808·3 to 2899·1)	1828·4 (1793·6 to 1865·3)	660·2 (635·4 to 686·6)	363·9 (353·4 to 374·7)	0·0 (0·0 to 0·0)	2685·5 (2644·0 to 2729·4)	1721·4 (1688·6 to 1756·1)	621·6 (598·3 to 646·4)	342·6 (332·7 to 352·8)	0·0 (0·0 to 0·0)
Israel	2015	2872·5 (2828·2 to 2916·4)	1841·7 (1807·4 to 1875·9)	663·1 (637·0 to 689·5)	367·8 (356·8 to 378·2)	0·0 (0·0 to 0·0)	2704·4 (2662·7 to 2745·7)	1733·9 (1701·6 to 1766·2)	624·3 (599·7 to 649·1)	346·3 (336·0 to 356·0)	0·0 (0·0 to 0·0)
Israel	2016	2969·0 (2923·5 to 3012·5)	1914·5 (1879·3 to 1950·5)	677·2 (651·9 to 702·5)	377·3 (366·8 to 388·3)	0·0 (0·0 to 0·0)	2795·2 (2752·4 to 2836·2)	1802·5 (1769·3 to 1836·3)	637·5 (613·7 to 661·3)	355·3 (345·4 to 365·6)	0·0 (0·0 to 0·0)
Israel	2017	3104·9 (3062·2 to 3149·6)	2019·2 (1985·0 to 2051·8)	690·9 (664·5 to 717·3)	394·8 (384·1 to 406·5)	0·0 (0·0 to 0·0)	2923·2 (2883·0 to 2965·3)	1901·0 (1868·9 to 1931·7)	650·5 (625·7 to 675·3)	371·7 (361·6 to 382·7)	0·0 (0·0 to 0·0)
Israel	2018	3201·9 (3146·0 to 3259·8)	2102·8 (2056·8 to 2149·4)	694·8 (660·3 to 731·5)	404·3 (390·2 to 419·2)	0·0 (0·0 to 0·0)	3014·5 (2961·9 to 3069·0)	1979·7 (1936·5 to 2023·7)	654·1 (621·7 to 688·7)	380·7 (367·3 to 394·7)	0·0 (0·0 to 0·0)
Israel	2019	3246·3 (3183·8 to 3307·4)	2122·4 (2069·3 to 2176·9)	704·4 (669·7 to 741·3)	419·5 (404·6 to 435·4)	0·0 (0·0 to 0·0)	3056·4 (2997·5 to 3113·9)	1998·2 (1948·2 to 2049·5)	663·2 (630·5 to 698·0)	394·9 (381·0 to 410·0)	0·0 (0·0 to 0·0)

Israel	2020	3569·4 (3508·0 to 3629·2)	2512·5 (2460·2 to 2566·1)	656·8 (624·5 to 691·6)	400·1 (385·0 to 417·1)	0·0 (0·0 to 0·0)	3360·6 (3302·7 to 3416·9)	2365·5 (2316·2 to 2415·9)	618·4 (587·9 to 651·1)	376·7 (362·5 to 392·7)	0·0 (0·0 to 0·0)
Israel	2021	3259·2 (3189·9 to 3327·2)	2241·5 (2179·6 to 2302·3)	627·1 (596·2 to 660·2)	390·7 (374·6 to 408·9)	0·0 (0·0 to 0·0)	3068·5 (3003·2 to 3132·5)	2110·3 (2052·0 to 2167·6)	590·4 (561·3 to 621·6)	367·8 (352·7 to 385·0)	0·0 (0·0 to 0·0)
Israel	2022	2954·8 (2884·3 to 3026·9)	1922·2 (1859·3 to 1984·9)	630·6 (599·5 to 663·9)	402·0 (383·0 to 422·5)	0·0 (0·0 to 0·0)	2781·9 (2715·5 to 2849·7)	1809·7 (1750·5 to 1868·8)	593·7 (564·4 to 625·1)	378·5 (360·6 to 397·8)	0·0 (0·0 to 0·0)
Israel	2023	2988·1 (2913·2 to 3063·1)	1940·1 (1872·6 to 2008·1)	634·2 (602·9 to 667·9)	413·7 (391·6 to 437·6)	0·0 (0·0 to 0·0)	2813·2 (2742·8 to 2883·8)	1826·6 (1763·0 to 1890·6)	597·1 (567·6 to 628·8)	389·5 (368·7 to 411·9)	0·0 (0·0 to 0·0)
Israel	2024	3022·4 (2939·4 to 3103·4)	1958·7 (1886·6 to 2035·0)	637·9 (606·3 to 671·4)	425·8 (400·2 to 453·1)	0·0 (0·0 to 0·0)	2845·6 (2767·4 to 2921·8)	1844·1 (1776·2 to 1915·9)	600·6 (570·8 to 632·1)	400·9 (376·8 to 426·6)	0·0 (0·0 to 0·0)
Israel	2025	3058·9 (2971·0 to 3148·2)	1978·7 (1902·1 to 2058·5)	641·7 (609·9 to 675·6)	438·5 (411·2 to 468·8)	0·0 (0·0 to 0·0)	2879·9 (2797·1 to 2964·0)	1862·9 (1790·8 to 1938·1)	604·2 (574·2 to 636·0)	412·8 (387·1 to 441·4)	0·0 (0·0 to 0·0)
Israel	2026	3097·8 (3007·9 to 3190·5)	2000·1 (1923·2 to 2085·1)	645·9 (613·9 to 679·8)	451·8 (421·3 to 485·0)	0·0 (0·0 to 0·0)	2916·5 (2831·9 to 3003·8)	1883·1 (1810·7 to 1963·1)	608·1 (578·0 to 640·0)	425·3 (396·7 to 456·6)	0·0 (0·0 to 0·0)
Israel	2027	3138·1 (3039·0 to 3237·9)	2022·1 (1934·9 to 2110·6)	650·4 (617·9 to 684·8)	465·6 (432·7 to 500·6)	0·0 (0·0 to 0·0)	2954·4 (2861·1 to 3048·4)	1903·8 (1821·7 to 1987·1)	612·3 (581·7 to 644·7)	438·4 (407·4 to 471·3)	0·0 (0·0 to 0·0)
Israel	2028	3179·8 (3074·3 to 3287·2)	2044·7 (1952·1 to 2144·6)	655·3 (622·5 to 689·8)	479·9 (446·5 to 516·2)	0·0 (0·0 to 0·0)	2993·7 (2894·4 to 3094·8)	1925·0 (1837·9 to 2019·1)	616·9 (586·1 to 649·5)	451·8 (420·4 to 486·0)	0·0 (0·0 to 0·0)
Israel	2029	3223·1 (3105·1 to 3344·1)	2067·6 (1963·8 to 2176·9)	660·7 (627·8 to 695·7)	494·8 (460·5 to 533·5)	0·0 (0·0 to 0·0)	3034·4 (2923·4 to 3148·4)	1946·6 (1848·9 to 2049·5)	622·0 (591·1 to 655·0)	465·8 (433·5 to 502·3)	0·0 (0·0 to 0·0)
Israel	2030	3263·5 (3133·5 to 3392·6)	2087·5 (1970·6 to 2207·0)	666·1 (632·8 to 700·8)	509·8 (473·7 to 549·3)	0·0 (0·0 to 0·0)	3072·5 (2950·1 to 3194·1)	1965·4 (1855·3 to 2077·8)	627·1 (595·7 to 659·8)	480·0 (446·0 to 517·1)	0·0 (0·0 to 0·0)
Israel	2031	3305·9 (3164·2 to 3446·6)	2109·0 (1980·4 to 2242·9)	671·6 (638·2 to 706·9)	525·3 (488·3 to 566·3)	0·0 (0·0 to 0·0)	3112·4 (2979·0 to 3244·9)	1985·6 (1864·5 to 2111·7)	632·3 (600·8 to 665·5)	494·6 (459·7 to 533·2)	0·0 (0·0 to 0·0)
Israel	2032	3347·6 (3197·3 to 3501·4)	2129·5 (1997·6 to 2278·6)	676·9 (643·3 to 712·7)	541·2 (502·6 to 584·7)	0·0 (0·0 to 0·0)	3151·7 (3010·2 to 3296·5)	2004·9 (1880·7 to 2145·2)	637·3 (605·6 to 671·0)	509·5 (473·2 to 550·5)	0·0 (0·0 to 0·0)
Israel	2033	3390·8 (3232·4 to 3553·5)	2151·3 (2005·3 to 2310·3)	682·0 (648·5 to 718·4)	557·5 (514·5 to 603·7)	0·0 (0·0 to 0·0)	3192·4 (3043·2 to 3345·5)	2025·4 (1887·9 to 2175·1)	642·1 (610·6 to 676·3)	524·8 (484·4 to 568·4)	0·0 (0·0 to 0·0)

Israel	2034	3435·7 (3265·9 to 3604·6)	2173·8 (2013·9 to 2342·7)	687·4 (653·6 to 724·1)	574·5 (527·3 to 623·8)	0·0 (0·0 to 0·0)	3234·6 (3074·8 to 3393·7)	2046·6 (1896·0 to 2205·6)	647·1 (615·3 to 681·7)	540·9 (496·5 to 587·3)	0·0 (0·0 to 0·0)
Israel	2035	3480·2 (3304·7 to 3657·0)	2196·0 (2027·5 to 2368·1)	692·4 (658·0 to 729·5)	591·9 (540·1 to 644·2)	0·0 (0·0 to 0·0)	3276·6 (3111·3 to 3443·0)	2067·5 (1908·9 to 2229·5)	651·9 (619·5 to 686·8)	557·2 (508·5 to 606·5)	0·0 (0·0 to 0·0)
Israel	2036	3524·7 (3334·9 to 3724·0)	2218·3 (2043·9 to 2400·9)	697·0 (662·0 to 734·6)	609·4 (553·3 to 668·0)	0·0 (0·0 to 0·0)	3318·4 (3139·8 to 3506·0)	2088·5 (1924·3 to 2260·4)	656·2 (623·3 to 691·6)	573·7 (520·9 to 628·9)	0·0 (0·0 to 0·0)
Israel	2037	3567·6 (3367·8 to 3774·7)	2238·9 (2057·5 to 2444·1)	701·4 (665·8 to 739·0)	627·3 (567·3 to 691·1)	0·0 (0·0 to 0·0)	3358·9 (3170·8 to 3553·8)	2107·9 (1937·1 to 2301·1)	660·4 (626·8 to 695·7)	590·6 (534·1 to 650·6)	0·0 (0·0 to 0·0)
Israel	2038	3609·1 (3402·1 to 3829·6)	2258·8 (2072·8 to 2464·8)	705·1 (669·3 to 742·6)	645·2 (580·7 to 714·1)	0·0 (0·0 to 0·0)	3397·9 (3203·0 to 3605·5)	2126·6 (1951·5 to 2320·6)	663·8 (630·1 to 699·2)	607·5 (546·8 to 672·3)	0·0 (0·0 to 0·0)
Israel	2039	3653·4 (3437·0 to 3879·3)	2281·6 (2084·2 to 2494·8)	708·4 (672·4 to 746·3)	663·5 (594·0 to 738·2)	0·0 (0·0 to 0·0)	3439·6 (3235·9 to 3652·3)	2148·1 (1962·2 to 2348·8)	666·9 (633·1 to 702·6)	624·6 (559·3 to 695·0)	0·0 (0·0 to 0·0)
Israel	2040	3696·0 (3475·2 to 3927·1)	2302·4 (2099·4 to 2521·0)	711·5 (675·3 to 749·9)	682·1 (604·7 to 763·5)	0·0 (0·0 to 0·0)	3479·7 (3271·8 to 3697·3)	2167·7 (1976·5 to 2373·4)	669·9 (635·8 to 706·0)	642·2 (569·3 to 718·8)	0·0 (0·0 to 0·0)
Israel	2041	3739·1 (3513·9 to 3990·0)	2323·1 (2120·8 to 2559·8)	714·8 (678·4 to 753·5)	701·2 (617·4 to 789·9)	0·0 (0·0 to 0·0)	3520·3 (3308·3 to 3756·5)	2187·1 (1996·7 to 2410·0)	673·0 (638·7 to 709·4)	660·2 (581·3 to 743·7)	0·0 (0·0 to 0·0)
Israel	2042	3783·0 (3550·2 to 4045·3)	2343·6 (2132·9 to 2586·9)	718·4 (681·7 to 757·3)	721·0 (631·2 to 819·4)	0·0 (0·0 to 0·0)	3561·6 (3342·5 to 3808·6)	2206·5 (2008·1 to 2435·5)	676·4 (641·8 to 713·0)	678·8 (594·3 to 771·5)	0·0 (0·0 to 0·0)
Israel	2043	3828·9 (3582·6 to 4105·2)	2365·5 (2135·5 to 2625·6)	722·2 (685·3 to 761·5)	741·2 (643·5 to 850·2)	0·0 (0·0 to 0·0)	3604·8 (3372·9 to 3864·9)	2227·0 (2010·5 to 2471·9)	679·9 (645·2 to 717·0)	697·8 (605·9 to 800·5)	0·0 (0·0 to 0·0)
Israel	2044	3873·4 (3618·9 to 4161·0)	2385·2 (2151·3 to 2647·7)	726·2 (689·0 to 766·0)	762·0 (655·1 to 879·9)	0·0 (0·0 to 0·0)	3646·7 (3407·1 to 3917·5)	2245·6 (2025·4 to 2492·7)	683·7 (648·7 to 721·1)	717·5 (616·8 to 828·4)	0·0 (0·0 to 0·0)
Israel	2045	3921·8 (3658·7 to 4210·0)	2407·6 (2158·9 to 2674·7)	730·4 (693·1 to 770·8)	783·7 (668·7 to 912·4)	0·0 (0·0 to 0·0)	3692·3 (3444·6 to 3963·7)	2266·8 (2032·5 to 2518·2)	687·7 (652·5 to 725·6)	737·8 (629·6 to 859·0)	0·0 (0·0 to 0·0)
Israel	2046	3970·9 (3698·0 to 4264·8)	2429·9 (2177·0 to 2706·7)	735·0 (697·7 to 775·8)	806·1 (681·8 to 945·0)	0·0 (0·0 to 0·0)	3738·6 (3481·6 to 4015·2)	2287·7 (2049·6 to 2548·3)	692·0 (656·9 to 730·4)	758·9 (641·9 to 889·7)	0·0 (0·0 to 0·0)
Israel	2047	4020·0 (3729·3 to 4335·1)	2451·2 (2186·4 to 2727·3)	739·7 (702·0 to 781·0)	829·2 (694·8 to 979·1)	0·0 (0·0 to 0·0)	3784·8 (3511·1 to 4081·4)	2307·8 (2058·5 to 2567·7)	696·4 (660·9 to 735·3)	780·6 (654·2 to 921·8)	0·0 (0·0 to 0·0)

Israel	2048	4069·2 (3772·5 to 4396·3)	2471·5 (2208·1 to 2766·5)	744·6 (706·7 to 786·4)	853·1 (709·2 to 1014·7)	0·0 (0·0 to 0·0)	3831·1 (3551·7 to 4139·0)	2326·9 (2078·9 to 2604·6)	701·0 (665·3 to 740·4)	803·1 (667·7 to 955·4)	0·0 (0·0 to 0·0)
Israel	2049	4120·3 (3810·7 to 4448·4)	2492·9 (2210·8 to 2792·7)	749·7 (711·6 to 791·9)	877·8 (724·1 to 1056·9)	0·0 (0·0 to 0·0)	3879·2 (3587·7 to 4188·1)	2347·0 (2081·4 to 2629·3)	705·8 (670·0 to 745·5)	826·4 (681·7 to 995·1)	0·0 (0·0 to 0·0)
Israel	2050	4174·1 (3854·2 to 4521·9)	2515·5 (2239·0 to 2813·6)	755·1 (716·8 to 797·8)	903·5 (737·7 to 1096·3)	0·0 (0·0 to 0·0)	3929·9 (3628·7 to 4257·2)	2368·3 (2108·0 to 2648·9)	710·9 (674·9 to 751·1)	850·6 (694·5 to 1032·2)	0·0 (0·0 to 0·0)
Italy	1995	2202·1 (2136·8 to 2263·3)	1579·0 (1519·9 to 1638·0)	599·5 (570·2 to 629·1)	23·7 (17·6 to 30·6)	0·0 (0·0 to 0·0)	2879·4 (2794·0 to 2959·4)	2064·6 (1987·4 to 2141·8)	783·9 (745·6 to 822·5)	30·9 (23·0 to 40·0)	0·0 (0·0 to 0·0)
Italy	1996	2273·9 (2223·7 to 2321·9)	1628·9 (1581·6 to 1672·1)	620·4 (597·4 to 642·5)	24·5 (18·6 to 31·1)	0·0 (0·0 to 0·0)	2973·3 (2907·6 to 3036·0)	2130·0 (2068·0 to 2186·4)	811·3 (781·1 to 840·2)	32·0 (24·3 to 40·6)	0·0 (0·0 to 0·0)
Italy	1997	2356·5 (2309·3 to 2404·2)	1680·5 (1637·3 to 1721·3)	651·0 (628·8 to 673·1)	24·9 (19·6 to 30·5)	0·0 (0·0 to 0·0)	3081·2 (3019·6 to 3143·7)	2197·4 (2140·8 to 2250·7)	851·3 (822·2 to 880·1)	32·6 (25·6 to 39·9)	0·0 (0·0 to 0·0)
Italy	1998	2440·4 (2391·0 to 2491·8)	1735·9 (1691·6 to 1785·8)	680·0 (658·2 to 701·2)	24·5 (20·4 to 29·1)	0·0 (0·0 to 0·0)	3191·0 (3126·4 to 3258·1)	2269·9 (2211·9 to 2335·1)	889·1 (860·6 to 916·8)	32·0 (26·7 to 38·1)	0·0 (0·0 to 0·0)
Italy	1999	2522·9 (2468·6 to 2571·7)	1804·5 (1756·9 to 1848·7)	694·0 (673·8 to 713·4)	24·5 (21·4 to 27·7)	0·0 (0·0 to 0·0)	3298·9 (3227·9 to 3362·6)	2359·5 (2297·2 to 2417·3)	907·4 (881·1 to 932·9)	32·0 (28·0 to 36·2)	0·0 (0·0 to 0·0)
Italy	2000	2649·4 (2601·8 to 2694·6)	1928·0 (1885·8 to 1969·4)	697·2 (680·4 to 714·5)	24·2 (22·2 to 26·1)	0·0 (0·0 to 0·0)	3464·3 (3402·0 to 3523·4)	2521·0 (2465·8 to 2575·2)	911·7 (889·7 to 934·2)	31·6 (29·0 to 34·2)	0·0 (0·0 to 0·0)
Italy	2001	2765·2 (2721·3 to 2809·8)	2053·5 (2011·8 to 2095·2)	686·7 (670·8 to 704·2)	24·9 (23·4 to 26·6)	0·0 (0·0 to 0·0)	3615·7 (3558·3 to 3674·0)	2685·2 (2630·6 to 2739·6)	898·0 (877·1 to 920·8)	32·6 (30·6 to 34·8)	0·0 (0·0 to 0·0)
Italy	2002	2784·2 (2736·4 to 2833·6)	2086·6 (2042·6 to 2132·4)	672·1 (656·6 to 689·2)	25·5 (24·0 to 27·2)	0·0 (0·0 to 0·0)	3640·5 (3578·1 to 3705·2)	2728·4 (2670·9 to 2788·3)	878·8 (858·6 to 901·2)	33·4 (31·4 to 35·6)	0·0 (0·0 to 0·0)
Italy	2003	2827·4 (2781·1 to 2875·3)	2137·4 (2093·7 to 2181·5)	663·8 (648·5 to 680·0)	26·2 (24·6 to 27·9)	0·0 (0·0 to 0·0)	3697·0 (3636·5 to 3759·6)	2794·8 (2737·7 to 2852·5)	868·0 (847·9 to 889·1)	34·2 (32·2 to 36·5)	0·0 (0·0 to 0·0)
Italy	2004	2902·5 (2855·2 to 2950·2)	2216·5 (2171·6 to 2259·1)	659·2 (643·2 to 674·8)	26·8 (25·2 to 28·4)	0·0 (0·0 to 0·0)	3795·2 (3733·4 to 3857·6)	2898·2 (2839·5 to 2953·9)	861·9 (841·0 to 882·4)	35·1 (33·0 to 37·1)	0·0 (0·0 to 0·0)
Italy	2005	2972·7 (2927·7 to 3017·0)	2295·6 (2252·4 to 2338·7)	649·7 (633·9 to 665·5)	27·4 (25·8 to 28·9)	0·0 (0·0 to 0·0)	3887·0 (3828·2 to 3944·9)	3001·7 (2945·2 to 3058·0)	849·6 (828·9 to 870·2)	35·8 (33·7 to 37·8)	0·0 (0·0 to 0·0)

Italy	2006	3030·2 (2983·6 to 3077·0)	2354·0 (2309·8 to 2399·5)	647·7 (631·7 to 663·6)	28·5 (26·9 to 30·2)	0·0 (0·0 to 0·0)	3962·2 (3901·3 to 4023·4)	3078·1 (3020·3 to 3137·5)	846·9 (826·0 to 867·7)	37·3 (35·2 to 39·5)	0·0 (0·0 to 0·0)
Italy	2007	2997·5 (2947·5 to 3044·8)	2326·8 (2278·7 to 2372·6)	641·4 (624·4 to 656·7)	29·3 (27·7 to 31·0)	0·0 (0·0 to 0·0)	3919·5 (3854·1 to 3981·3)	3042·5 (2979·6 to 3102·3)	838·6 (816·4 to 858·7)	38·3 (36·2 to 40·5)	0·0 (0·0 to 0·0)
Italy	2008	3026·3 (2977·7 to 3077·4)	2355·1 (2309·4 to 2401·4)	641·0 (624·9 to 656·5)	30·2 (28·7 to 31·7)	0·0 (0·0 to 0·0)	3957·1 (3893·5 to 4023·9)	3079·4 (3019·7 to 3140·0)	838·2 (817·1 to 858·4)	39·5 (37·6 to 41·5)	0·0 (0·0 to 0·0)
Italy	2009	3042·7 (2996·5 to 3095·4)	2380·2 (2336·1 to 2428·1)	631·6 (616·2 to 647·5)	30·8 (29·2 to 32·6)	0·0 (0·0 to 0·0)	3978·5 (3918·1 to 4047·4)	3112·3 (3054·7 to 3174·9)	825·9 (805·7 to 846·7)	40·3 (38·2 to 42·7)	0·0 (0·0 to 0·0)
Italy	2010	3025·2 (2980·1 to 3070·9)	2361·5 (2318·3 to 2402·4)	633·4 (618·9 to 649·6)	30·3 (28·8 to 32·0)	0·0 (0·0 to 0·0)	3955·6 (3896·7 to 4015·5)	3087·8 (3031·4 to 3141·3)	828·2 (809·2 to 849·5)	39·6 (37·6 to 41·8)	0·0 (0·0 to 0·0)
Italy	2011	2972·8 (2929·4 to 3019·3)	2299·2 (2257·3 to 2342·6)	644·4 (628·8 to 659·8)	29·2 (28·0 to 30·6)	0·0 (0·0 to 0·0)	3887·2 (3830·4 to 3947·9)	3006·3 (2951·6 to 3063·1)	842·6 (822·2 to 862·8)	38·2 (36·6 to 40·0)	0·0 (0·0 to 0·0)
Italy	2012	2930·4 (2883·4 to 2976·8)	2227·0 (2180·8 to 2271·3)	644·5 (628·9 to 661·3)	58·9 (56·8 to 60·9)	0·0 (0·0 to 0·0)	3831·7 (3770·3 to 3892·3)	2912·0 (2851·6 to 2969·9)	842·8 (822·3 to 864·7)	77·0 (74·3 to 79·6)	0·0 (0·0 to 0·0)
Italy	2013	2861·7 (2815·6 to 2908·5)	2165·3 (2124·1 to 2207·7)	638·8 (623·6 to 654·0)	57·6 (55·8 to 59·4)	0·0 (0·0 to 0·0)	3741·9 (3681·5 to 3803·1)	2831·2 (2777·4 to 2886·7)	835·3 (815·4 to 855·1)	75·3 (72·9 to 77·7)	0·0 (0·0 to 0·0)
Italy	2014	2862·1 (2817·3 to 2907·7)	2152·9 (2109·0 to 2195·6)	650·8 (634·0 to 666·3)	58·3 (56·4 to 60·1)	0·0 (0·0 to 0·0)	3742·4 (3683·9 to 3802·1)	2815·1 (2757·7 to 2870·9)	851·0 (829·0 to 871·3)	76·3 (73·7 to 78·6)	0·0 (0·0 to 0·0)
Italy	2015	2889·1 (2841·1 to 2936·5)	2156·9 (2110·7 to 2200·7)	671·1 (655·2 to 686·8)	61·0 (59·2 to 62·9)	0·0 (0·0 to 0·0)	3777·7 (3715·0 to 3839·7)	2820·3 (2759·8 to 2877·5)	877·5 (856·8 to 898·1)	79·8 (77·4 to 82·3)	0·0 (0·0 to 0·0)
Italy	2016	2898·5 (2848·7 to 2944·6)	2151·0 (2106·2 to 2196·0)	681·7 (664·2 to 698·5)	65·8 (64·0 to 67·8)	0·0 (0·0 to 0·0)	3790·0 (3724·8 to 3850·2)	2812·6 (2754·0 to 2871·5)	891·4 (868·5 to 913·4)	86·1 (83·7 to 88·6)	0·0 (0·0 to 0·0)
Italy	2017	2929·0 (2879·0 to 2978·0)	2164·0 (2119·0 to 2210·0)	693·4 (675·9 to 709·8)	71·7 (69·8 to 73·8)	0·0 (0·0 to 0·0)	3829·9 (3764·5 to 3894·0)	2829·5 (2770·8 to 2889·8)	906·6 (883·7 to 928·1)	93·7 (91·2 to 96·5)	0·0 (0·0 to 0·0)
Italy	2018	2958·2 (2897·8 to 3022·3)	2184·3 (2123·0 to 2242·3)	697·9 (678·0 to 717·9)	76·0 (73·6 to 78·6)	0·0 (0·0 to 0·0)	3868·1 (3789·1 to 3951·9)	2856·2 (2775·9 to 2932·0)	912·6 (886·6 to 938·8)	99·3 (96·2 to 102·7)	0·0 (0·0 to 0·0)
Italy	2019	2994·9 (2931·4 to 3061·0)	2217·0 (2155·1 to 2278·9)	699·9 (680·1 to 720·2)	78·1 (75·4 to 80·9)	0·0 (0·0 to 0·0)	3916·0 (3833·1 to 4002·5)	2898·8 (2817·9 to 2979·8)	915·1 (889·3 to 941·7)	102·1 (98·6 to 105·8)	0·0 (0·0 to 0·0)

Italy	2020	3286·5 (3207·2 to 3363·5)	2553·9 (2477·1 to 2626·0)	657·5 (638·8 to 676·4)	75·2 (72·4 to 78·2)	0·0 (0·0 to 0·0)	4297·3 (4193·6 to 4398·1)	3339·4 (3239·0 to 3433·7)	859·7 (835·2 to 884·5)	98·3 (94·6 to 102·3)	0·0 (0·0 to 0·0)
Italy	2021	3205·0 (3127·8 to 3280·0)	2490·5 (2415·7 to 2560·9)	639·5 (621·0 to 657·9)	74·9 (71·7 to 78·4)	0·0 (0·0 to 0·0)	4190·8 (4089·9 to 4288·8)	3256·5 (3158·7 to 3348·5)	836·2 (812·0 to 860·3)	98·0 (93·8 to 102·5)	0·0 (0·0 to 0·0)
Italy	2022	2821·3 (2742·5 to 2900·5)	2103·4 (2025·3 to 2176·9)	641·0 (622·3 to 659·5)	77·0 (73·3 to 81·0)	0·0 (0·0 to 0·0)	3689·1 (3586·0 to 3792·7)	2750·3 (2648·2 to 2846·4)	838·2 (813·7 to 862·4)	100·7 (95·9 to 105·9)	0·0 (0·0 to 0·0)
Italy	2023	2855·6 (2774·7 to 2937·6)	2134·5 (2052·8 to 2211·5)	642·1 (623·5 to 660·8)	79·1 (74·8 to 83·7)	0·0 (0·0 to 0·0)	3733·9 (3628·1 to 3841·1)	2791·0 (2684·2 to 2891·7)	839·6 (815·2 to 864·0)	103·4 (97·8 to 109·4)	0·0 (0·0 to 0·0)
Italy	2024	2890·6 (2804·5 to 2976·1)	2166·4 (2081·3 to 2249·0)	643·0 (624·4 to 661·8)	81·2 (76·4 to 86·3)	0·0 (0·0 to 0·0)	3779·7 (3667·1 to 3891·4)	2832·7 (2721·5 to 2940·8)	840·8 (816·4 to 865·3)	106·2 (99·9 to 112·8)	0·0 (0·0 to 0·0)
Italy	2025	2916·3 (2826·4 to 3006·3)	2191·6 (2100·8 to 2280·4)	641·6 (623·0 to 660·3)	83·1 (77·8 to 88·7)	0·0 (0·0 to 0·0)	3813·2 (3695·7 to 3931·0)	2865·7 (2746·9 to 2981·8)	839·0 (814·6 to 863·3)	108·6 (101·7 to 116·0)	0·0 (0·0 to 0·0)
Italy	2026	2936·2 (2841·8 to 3033·0)	2211·0 (2115·3 to 2304·6)	640·3 (621·7 to 658·7)	85·0 (79·1 to 91·5)	0·0 (0·0 to 0·0)	3839·3 (3715·9 to 3965·8)	2891·0 (2765·8 to 3013·4)	837·2 (812·9 to 861·4)	111·1 (103·5 to 119·6)	0·0 (0·0 to 0·0)
Italy	2027	2952·3 (2847·3 to 3054·9)	2226·8 (2125·7 to 2323·6)	638·5 (620·0 to 657·0)	87·0 (80·3 to 94·5)	0·0 (0·0 to 0·0)	3860·3 (3723·1 to 3994·5)	2911·7 (2779·5 to 3038·2)	834·9 (810·7 to 859·1)	113·8 (105·0 to 123·6)	0·0 (0·0 to 0·0)
Italy	2028	2966·9 (2857·8 to 3074·8)	2241·8 (2132·3 to 2346·9)	636·1 (617·7 to 654·7)	89·0 (81·4 to 97·7)	0·0 (0·0 to 0·0)	3879·5 (3736·8 to 4020·5)	2931·4 (2788·1 to 3068·8)	831·7 (807·7 to 856·0)	116·4 (106·4 to 127·8)	0·0 (0·0 to 0·0)
Italy	2029	2981·7 (2867·9 to 3098·7)	2257·2 (2141·9 to 2373·9)	633·4 (615·1 to 651·9)	91·1 (82·4 to 101·1)	0·0 (0·0 to 0·0)	3898·8 (3750·0 to 4051·7)	2951·4 (2800·7 to 3104·0)	828·2 (804·2 to 852·4)	119·1 (107·8 to 132·2)	0·0 (0·0 to 0·0)
Italy	2030	2995·0 (2875·1 to 3116·1)	2271·5 (2147·5 to 2394·0)	630·3 (612·1 to 648·9)	93·2 (83·3 to 104·6)	0·0 (0·0 to 0·0)	3916·2 (3759·4 to 4074·6)	2970·2 (2808·1 to 3130·3)	824·1 (800·3 to 848·4)	121·9 (108·9 to 136·8)	0·0 (0·0 to 0·0)
Italy	2031	3008·2 (2882·9 to 3136·8)	2286·0 (2164·1 to 2417·8)	626·9 (608·4 to 645·9)	95·3 (84·2 to 108·2)	0·0 (0·0 to 0·0)	3933·4 (3769·5 to 4101·6)	2989·1 (2829·7 to 3161·5)	819·7 (795·5 to 844·6)	124·6 (110·1 to 141·5)	0·0 (0·0 to 0·0)
Italy	2032	3020·2 (2888·8 to 3156·8)	2299·4 (2165·2 to 2432·2)	623·3 (604·9 to 642·3)	97·4 (85·2 to 111·6)	0·0 (0·0 to 0·0)	3949·1 (3777·3 to 4127·7)	3006·6 (2831·1 to 3180·3)	815·1 (790·9 to 839·8)	127·4 (111·4 to 146·0)	0·0 (0·0 to 0·0)
Italy	2033	3030·5 (2893·8 to 3171·8)	2311·2 (2173·1 to 2455·8)	619·7 (601·0 to 638·8)	99·5 (86·2 to 115·3)	0·0 (0·0 to 0·0)	3962·6 (3783·9 to 4147·3)	3022·1 (2841·5 to 3211·2)	810·3 (785·9 to 835·3)	130·1 (112·7 to 150·7)	0·0 (0·0 to 0·0)

Italy	2034	3040·7 (2898·0 to 3188·1)	2323·1 (2176·7 to 2467·8)	615·9 (597·3 to 635·1)	101·7 (87·1 to 119·0)	0·0 (0·0 to 0·0)	3975·9 (3789·3 to 4168·7)	3037·6 (2846·3 to 3226·8)	805·3 (781·0 to 830·5)	132·9 (113·9 to 155·6)	0·0 (0·0 to 0·0)
Italy	2035	3050·0 (2893·3 to 3205·7)	2334·3 (2177·0 to 2485·3)	611·9 (592·9 to 631·5)	103·8 (87·9 to 122·8)	0·0 (0·0 to 0·0)	3988·1 (3783·2 to 4191·6)	3052·2 (2846·6 to 3249·8)	800·1 (775·2 to 825·7)	135·7 (115·0 to 160·6)	0·0 (0·0 to 0·0)
Italy	2036	3058·1 (2893·9 to 3222·5)	2344·2 (2178·3 to 2504·0)	608·0 (589·1 to 627·7)	105·9 (88·8 to 126·6)	0·0 (0·0 to 0·0)	3998·7 (3783·9 to 4213·6)	3065·2 (2848·2 to 3274·2)	795·0 (770·3 to 820·7)	138·5 (116·1 to 165·6)	0·0 (0·0 to 0·0)
Italy	2037	3062·9 (2890·5 to 3233·1)	2351·2 (2175·8 to 2519·2)	603·7 (585·0 to 623·7)	108·0 (89·6 to 130·4)	0·0 (0·0 to 0·0)	4005·0 (3779·5 to 4227·4)	3074·4 (2845·0 to 3294·0)	789·4 (764·9 to 815·5)	141·2 (117·2 to 170·5)	0·0 (0·0 to 0·0)
Italy	2038	3067·1 (2892·0 to 3249·2)	2357·9 (2182·5 to 2534·7)	599·2 (580·3 to 619·8)	110·1 (90·5 to 134·1)	0·0 (0·0 to 0·0)	4010·5 (3781·5 to 4248·5)	3083·1 (2853·7 to 3314·3)	783·4 (758·8 to 810·4)	144·0 (118·3 to 175·4)	0·0 (0·0 to 0·0)
Italy	2039	3070·1 (2890·3 to 3259·7)	2363·3 (2183·7 to 2552·1)	594·6 (575·5 to 615·7)	112·2 (91·3 to 138·0)	0·0 (0·0 to 0·0)	4014·4 (3779·3 to 4262·3)	3090·2 (2855·4 to 3337·1)	777·5 (752·5 to 805·1)	146·7 (119·4 to 180·4)	0·0 (0·0 to 0·0)
Italy	2040	3073·9 (2882·7 to 3270·6)	2369·2 (2178·2 to 2569·1)	590·3 (570·9 to 611·9)	114·3 (92·0 to 141·9)	0·0 (0·0 to 0·0)	4019·4 (3769·4 to 4276·5)	3098·0 (2848·1 to 3359·3)	771·9 (746·5 to 800·0)	149·5 (120·4 to 185·5)	0·0 (0·0 to 0·0)
Italy	2041	3076·4 (2882·1 to 3284·5)	2373·7 (2181·9 to 2578·7)	586·2 (566·4 to 608·6)	116·5 (92·8 to 145·8)	0·0 (0·0 to 0·0)	4022·7 (3768·5 to 4294·7)	3103·8 (2853·0 to 3371·9)	766·5 (740·6 to 795·8)	152·3 (121·3 to 190·6)	0·0 (0·0 to 0·0)
Italy	2042	3081·8 (2881·4 to 3287·1)	2380·1 (2176·1 to 2594·3)	583·0 (562·8 to 605·8)	118·8 (93·8 to 149·8)	0·0 (0·0 to 0·0)	4029·7 (3767·6 to 4298·1)	3112·1 (2845·4 to 3392·3)	762·3 (735·9 to 792·2)	155·3 (122·6 to 195·9)	0·0 (0·0 to 0·0)
Italy	2043	3088·3 (2882·9 to 3309·3)	2386·6 (2178·7 to 2607·6)	580·5 (560·0 to 603·6)	121·1 (94·7 to 153·9)	0·0 (0·0 to 0·0)	4038·1 (3769·6 to 4327·1)	3120·7 (2848·8 to 3409·6)	759·0 (732·2 to 789·3)	158·4 (123·9 to 201·2)	0·0 (0·0 to 0·0)
Italy	2044	3095·3 (2881·1 to 3323·5)	2393·1 (2174·9 to 2618·2)	578·5 (558·1 to 601·9)	123·6 (95·8 to 158·0)	0·0 (0·0 to 0·0)	4047·3 (3767·2 to 4345·7)	3129·2 (2843·8 to 3423·5)	756·5 (729·8 to 787·1)	161·6 (125·3 to 206·6)	0·0 (0·0 to 0·0)
Italy	2045	3103·5 (2885·0 to 3338·7)	2400·2 (2182·4 to 2625·5)	577·1 (556·4 to 600·7)	126·2 (97·0 to 162·3)	0·0 (0·0 to 0·0)	4058·0 (3772·4 to 4365·6)	3138·4 (2853·7 to 3433·0)	754·6 (727·6 to 785·5)	165·0 (126·8 to 212·2)	0·0 (0·0 to 0·0)
Italy	2046	3112·0 (2887·2 to 3350·0)	2407·3 (2185·8 to 2651·5)	575·9 (555·0 to 599·6)	128·8 (98·5 to 166·6)	0·0 (0·0 to 0·0)	4069·2 (3775·2 to 4380·3)	3147·7 (2858·1 to 3467·1)	753·1 (725·7 to 784·0)	168·4 (128·8 to 217·8)	0·0 (0·0 to 0·0)
Italy	2047	3121·8 (2898·8 to 3360·0)	2415·0 (2188·8 to 2653·3)	575·3 (554·1 to 598·9)	131·5 (100·1 to 171·1)	0·0 (0·0 to 0·0)	4082·0 (3790·3 to 4393·5)	3157·8 (2862·0 to 3469·3)	752·2 (724·5 to 783·1)	172·0 (130·8 to 223·7)	0·0 (0·0 to 0·0)

Italy	2048	3133·0 (2897·6 to 3381·5)	2423·9 (2186·2 to 2661·1)	574·8 (553·3 to 598·7)	134·4 (101·3 to 175·6)	0·0 (0·0 to 0·0)	4096·7 (3788·8 to 4421·5)	3169·4 (2858·6 to 3479·6)	751·6 (723·5 to 782·8)	175·7 (132·5 to 229·6)	0·0 (0·0 to 0·0)
Italy	2049	3145·5 (2908·9 to 3398·7)	2433·2 (2201·2 to 2676·4)	575·0 (553·2 to 598·9)	137·3 (103·0 to 180·4)	0·0 (0·0 to 0·0)	4113·0 (3803·6 to 4444·0)	3181·6 (2878·2 to 3499·6)	751·8 (723·4 to 783·1)	179·6 (134·7 to 235·9)	0·0 (0·0 to 0·0)
Italy	2050	3159·9 (2923·7 to 3424·5)	2443·8 (2209·8 to 2701·7)	575·6 (553·6 to 599·7)	140·4 (104·9 to 185·3)	0·0 (0·0 to 0·0)	4131·8 (3823·0 to 4477·8)	3195·5 (2889·4 to 3532·7)	752·6 (723·9 to 784·2)	183·6 (137·2 to 242·3)	0·0 (0·0 to 0·0)
Jamaica*	1995	250·8 (228·8 to 274·6)	129·4 (114·8 to 146·2)	61·6 (50·8 to 74·3)	44·1 (34·1 to 57·6)	15·6 (15·6 to 15·6)	491·0 (448·0 to 537·7)	253·4 (224·8 to 286·2)	120·7 (99·4 to 145·4)	86·4 (66·8 to 112·8)	30·6 (30·6 to 30·6)
Jamaica*	1996	276·6 (256·2 to 301·0)	152·4 (137·1 to 168·3)	67·2 (56·7 to 78·9)	46·4 (36·3 to 59·1)	10·6 (10·6 to 10·6)	541·4 (501·6 to 589·3)	298·3 (268·5 to 329·4)	131·6 (111·0 to 154·5)	90·9 (71·1 to 115·7)	20·7 (20·7 to 20·7)
Jamaica*	1997	290·3 (270·3 to 311·9)	160·0 (146·1 to 174·4)	74·9 (63·6 to 86·9)	46·5 (36·9 to 58·9)	9·0 (9·0 to 9·0)	568·3 (529·2 to 610·7)	313·2 (285·9 to 341·5)	146·6 (124·5 to 170·1)	91·0 (72·3 to 115·3)	17·5 (17·5 to 17·5)
Jamaica*	1998	291·4 (272·0 to 312·9)	159·3 (145·6 to 174·2)	76·4 (65·7 to 88·1)	45·9 (36·7 to 57·4)	9·7 (9·7 to 9·7)	570·4 (532·5 to 612·6)	311·8 (285·1 to 341·0)	149·6 (128·6 to 172·6)	89·9 (71·8 to 112·3)	19·0 (19·0 to 19·0)
Jamaica*	1999	286·3 (267·3 to 306·4)	152·5 (139·3 to 166·6)	80·7 (70·5 to 92·3)	44·5 (35·5 to 54·9)	8·5 (8·5 to 8·5)	560·4 (523·3 to 600·0)	298·6 (272·7 to 326·2)	158·1 (138·1 to 180·8)	87·0 (69·5 to 107·4)	16·7 (16·7 to 16·7)
Jamaica*	2000	278·9 (261·0 to 298·8)	143·5 (131·5 to 156·2)	84·7 (75·0 to 95·9)	41·0 (32·6 to 50·5)	9·7 (9·7 to 9·7)	546·1 (511·1 to 585·0)	280·9 (257·5 to 305·8)	165·9 (146·8 to 187·8)	80·3 (63·9 to 98·9)	19·0 (19·0 to 19·0)
Jamaica*	2001	284·6 (267·0 to 304·1)	137·5 (126·2 to 150·4)	86·7 (77·3 to 96·9)	39·3 (31·0 to 48·9)	21·1 (21·1 to 21·1)	557·3 (522·7 to 595·4)	269·2 (247·1 to 294·5)	169·7 (151·3 to 189·6)	77·0 (60·8 to 95·7)	41·3 (41·3 to 41·3)
Jamaica*	2002	259·2 (242·4 to 277·4)	135·6 (124·5 to 148·0)	80·9 (72·1 to 90·0)	38·5 (30·5 to 47·6)	4·1 (4·1 to 4·1)	507·4 (474·6 to 543·2)	265·6 (243·8 to 289·8)	158·5 (141·1 to 176·3)	75·4 (59·7 to 93·1)	8·0 (8·0 to 8·0)
Jamaica*	2003	255·0 (238·9 to 272·7)	130·5 (120·5 to 141·3)	79·1 (70·3 to 88·4)	40·5 (32·4 to 50·4)	5·0 (5·0 to 5·0)	499·3 (467·8 to 534·0)	255·5 (236·0 to 276·5)	154·8 (137·7 to 173·1)	79·2 (63·4 to 98·7)	9·7 (9·7 to 9·7)
Jamaica*	2004	256·8 (240·3 to 274·1)	128·1 (118·6 to 138·1)	76·6 (68·3 to 85·6)	46·0 (36·5 to 57·2)	6·1 (6·1 to 6·1)	502·7 (470·4 to 536·7)	250·8 (232·2 to 270·4)	149·9 (133·7 to 167·5)	90·2 (71·4 to 112·1)	11·9 (11·9 to 11·9)
Jamaica*	2005	249·4 (233·1 to 266·5)	121·4 (113·3 to 129·8)	74·6 (66·8 to 83·5)	47·8 (38·4 to 58·9)	5·6 (5·6 to 5·6)	488·3 (456·4 to 521·7)	237·6 (221·9 to 254·0)	146·1 (130·7 to 163·5)	93·6 (75·2 to 115·2)	11·0 (11·0 to 11·0)

Jamaica*	2006	262·7 (247·1 to 280·1)	128·6 (120·5 to 137·3)	76·7 (68·8 to 85·9)	50·0 (40·3 to 61·0)	7·3 (7·3 to 7·3)	514·3 (483·7 to 548·3)	251·8 (235·9 to 268·8)	150·2 (134·6 to 168·2)	97·8 (78·8 to 119·4)	14·4 (14·4 to 14·4)
Jamaica*	2007	280·1 (263·5 to 297·7)	140·8 (132·7 to 149·3)	81·7 (72·7 to 91·0)	50·5 (40·6 to 62·0)	7·1 (7·1 to 7·1)	548·3 (515·8 to 582·9)	275·6 (259·7 to 292·3)	159·9 (142·4 to 178·2)	98·9 (79·6 to 121·3)	13·9 (13·9 to 13·9)
Jamaica*	2008	294·3 (277·1 to 311·3)	155·2 (146·7 to 164·3)	81·5 (72·1 to 92·1)	50·3 (40·5 to 62·0)	7·3 (7·3 to 7·3)	576·1 (542·5 to 609·4)	303·9 (287·2 to 321·7)	159·5 (141·1 to 180·4)	98·4 (79·2 to 121·5)	14·3 (14·3 to 14·3)
Jamaica*	2009	296·6 (278·7 to 314·7)	166·2 (157·3 to 175·7)	74·5 (64·9 to 85·1)	49·9 (40·8 to 61·5)	6·0 (6·0 to 6·0)	580·7 (545·7 to 616·1)	325·3 (307·9 to 344·1)	145·9 (127·1 to 166·5)	97·7 (79·9 to 120·4)	11·8 (11·8 to 11·8)
Jamaica*	2010	285·0 (268·7 to 301·9)	155·6 (147·3 to 164·8)	67·0 (58·3 to 76·5)	47·2 (38·0 to 57·6)	15·2 (15·2 to 15·2)	558·0 (526·0 to 591·0)	304·7 (288·4 to 322·7)	131·2 (114·2 to 149·8)	92·4 (74·3 to 112·8)	29·8 (29·8 to 29·8)
Jamaica*	2011	277·7 (261·8 to 293·6)	158·9 (151·0 to 168·1)	63·0 (54·5 to 72·3)	47·5 (37·8 to 57·4)	8·3 (8·3 to 8·3)	543·7 (512·6 to 574·8)	311·2 (295·5 to 329·1)	123·4 (106·7 to 141·6)	93·0 (74·1 to 112·3)	16·2 (16·2 to 16·2)
Jamaica*	2012	266·5 (251·4 to 281·4)	155·8 (147·6 to 164·5)	58·9 (51·4 to 67·6)	47·6 (38·2 to 58·0)	4·2 (4·2 to 4·2)	521·8 (492·2 to 551·0)	305·1 (289·1 to 322·1)	115·3 (100·5 to 132·3)	93·2 (74·7 to 113·5)	8·2 (8·2 to 8·2)
Jamaica*	2013	268·1 (252·7 to 283·8)	156·4 (147·9 to 164·8)	56·1 (48·8 to 64·5)	48·7 (39·3 to 58·6)	6·9 (6·9 to 6·9)	524·9 (494·7 to 555·6)	306·1 (289·6 to 322·6)	109·9 (95·5 to 126·3)	95·4 (77·0 to 114·7)	13·5 (13·5 to 13·5)
Jamaica*	2014	275·9 (260·7 to 291·1)	166·3 (157·9 to 174·7)	56·6 (49·1 to 64·8)	49·0 (39·3 to 58·8)	4·1 (4·1 to 4·1)	540·1 (510·3 to 570·0)	325·5 (309·1 to 342·1)	110·7 (96·1 to 127·0)	95·9 (76·8 to 115·2)	7·9 (7·9 to 7·9)
Jamaica*	2015	293·7 (278·4 to 309·9)	182·9 (174·1 to 192·8)	56·9 (49·3 to 65·0)	50·0 (40·0 to 60·0)	3·9 (3·9 to 3·9)	575·0 (545·1 to 606·6)	358·2 (340·9 to 377·4)	111·3 (96·6 to 127·3)	97·9 (78·4 to 117·5)	7·6 (7·6 to 7·6)
Jamaica*	2016	313·2 (296·0 to 330·3)	199·3 (188·0 to 210·2)	57·1 (48·9 to 66·5)	52·1 (41·9 to 63·7)	4·7 (4·7 to 4·7)	613·1 (579·5 to 646·6)	390·1 (368·1 to 411·6)	111·8 (95·7 to 130·1)	102·1 (82·0 to 124·8)	9·1 (9·1 to 9·1)
Jamaica*	2017	330·6 (311·0 to 350·9)	212·6 (198·0 to 227·1)	57·4 (48·7 to 67·8)	53·5 (42·4 to 66·4)	7·1 (7·1 to 7·1)	647·2 (608·9 to 687·0)	416·2 (387·7 to 444·6)	112·4 (95·3 to 132·8)	104·8 (83·0 to 130·0)	13·8 (13·8 to 13·8)
Jamaica*	2018	358·7 (333·3 to 383·7)	221·3 (201·6 to 240·8)	57·9 (48·3 to 69·8)	55·3 (43·2 to 68·5)	24·1 (24·1 to 24·1)	702·2 (652·5 to 751·3)	433·3 (394·7 to 471·5)	113·4 (94·6 to 136·6)	108·3 (84·6 to 134·2)	47·2 (47·2 to 47·2)
Jamaica*	2019	365·0 (337·3 to 392·8)	224·4 (200·6 to 247·2)	58·2 (48·5 to 70·1)	56·9 (44·5 to 70·5)	25·5 (25·3 to 26·0)	714·6 (660·4 to 769·0)	439·3 (392·8 to 483·9)	114·0 (95·0 to 137·3)	111·4 (87·1 to 138·0)	49·9 (49·6 to 50·9)

Jamaica*	2020	405·6 (378·4 to 433·1)	259·9 (236·1 to 282·6)	54·4 (45·4 to 65·5)	54·5 (42·4 to 67·6)	36·8 (36·3 to 37·7)	794·2 (740·8 to 848·0)	508·8 (462·3 to 553·3)	106·5 (88·8 to 128·2)	106·8 (83·0 to 132·4)	72·1 (71·0 to 73·8)
Jamaica*	2021	354·4 (327·2 to 381·7)	211·0 (188·1 to 233·8)	51·8 (43·1 to 62·3)	53·1 (41·3 to 66·0)	38·5 (37·7 to 40·4)	693·8 (640·5 to 747·3)	413·1 (368·2 to 457·6)	101·3 (84·5 to 122·0)	104·0 (80·8 to 129·1)	75·5 (73·8 to 79·0)
Jamaica*	2022	343·2 (315·1 to 374·1)	205·8 (181·8 to 231·7)	52·1 (43·4 to 62·8)	54·7 (42·4 to 68·0)	30·5 (28·7 to 32·8)	671·8 (616·9 to 732·3)	403·0 (355·9 to 453·7)	102·0 (85·0 to 123·0)	107·1 (83·0 to 133·1)	59·7 (56·2 to 64·2)
Jamaica*	2023	349·4 (319·4 to 381·2)	208·7 (181·9 to 237·7)	52·5 (43·7 to 63·1)	56·3 (43·8 to 69·9)	31·9 (29·8 to 34·6)	684·1 (625·3 to 746·3)	408·6 (356·2 to 465·3)	102·7 (85·6 to 123·6)	110·3 (85·7 to 136·9)	62·5 (58·4 to 67·7)
Jamaica*	2024	355·4 (324·2 to 390·0)	211·2 (184·3 to 242·5)	52·7 (43·9 to 63·5)	57·9 (44·9 to 72·0)	33·7 (31·3 to 37·0)	695·8 (634·8 to 763·5)	413·4 (360·7 to 474·7)	103·1 (86·0 to 124·2)	113·3 (87·9 to 141·0)	66·0 (61·3 to 72·3)
Jamaica*	2025	361·2 (327·6 to 399·3)	213·3 (185·5 to 247·4)	52·9 (44·1 to 63·7)	59·4 (46·3 to 74·2)	35·6 (32·8 to 39·3)	707·1 (641·4 to 781·7)	417·5 (363·1 to 484·4)	103·5 (86·3 to 124·8)	116·3 (90·7 to 145·3)	69·8 (64·2 to 76·9)
Jamaica*	2026	367·2 (331·5 to 407·1)	215·6 (184·1 to 249·9)	53·0 (44·2 to 63·8)	60·9 (47·2 to 76·1)	37·7 (34·5 to 42·1)	718·9 (649·0 to 797·1)	422·1 (360·3 to 489·3)	103·7 (86·5 to 125·0)	119·3 (92·4 to 149·0)	73·8 (67·5 to 82·3)
Jamaica*	2027	373·4 (335·7 to 413·7)	218·0 (183·9 to 253·0)	53·1 (44·3 to 64·0)	62·6 (48·5 to 78·3)	39·8 (36·1 to 44·7)	731·0 (657·2 to 810·0)	426·7 (360·1 to 495·4)	103·9 (86·7 to 125·3)	122·6 (95·0 to 153·3)	77·9 (70·7 to 87·6)
Jamaica*	2028	379·3 (339·9 to 422·2)	219·8 (184·3 to 257·9)	53·1 (44·3 to 64·0)	64·3 (49·7 to 80·3)	42·0 (37·9 to 47·9)	742·5 (665·4 to 826·6)	430·4 (360·7 to 504·9)	104·0 (86·7 to 125·3)	125·8 (97·2 to 157·3)	82·3 (74·2 to 93·8)
Jamaica*	2029	386·1 (344·7 to 431·7)	222·4 (186·8 to 264·0)	53·2 (44·4 to 64·0)	66·0 (51·2 to 82·9)	44·5 (39·8 to 51·1)	755·8 (674·7 to 845·1)	435·4 (365·7 to 516·8)	104·1 (86·8 to 125·4)	129·3 (100·3 to 162·2)	87·0 (78·0 to 100·0)
Jamaica*	2030	393·0 (349·5 to 440·8)	225·0 (187·5 to 268·1)	53·2 (44·4 to 64·0)	67·8 (52·5 to 85·5)	47·0 (41·8 to 54·5)	769·4 (684·2 to 863·0)	440·4 (367·1 to 524·9)	104·2 (86·9 to 125·4)	132·8 (102·9 to 167·3)	92·0 (81·7 to 106·7)
Jamaica*	2031	399·8 (355·5 to 449·5)	227·2 (188·7 to 272·0)	53·2 (44·4 to 64·1)	69·6 (54·1 to 88·0)	49·8 (44·0 to 58·6)	782·7 (695·9 to 880·1)	444·8 (369·5 to 532·5)	104·2 (86·9 to 125·4)	136·3 (105·9 to 172·2)	97·4 (86·1 to 114·7)
Jamaica*	2032	407·0 (360·5 to 457·2)	229·5 (189·1 to 276·0)	53·2 (44·4 to 64·0)	71·5 (55·6 to 90·1)	52·7 (46·3 to 62·5)	796·8 (705·8 to 895·2)	449·3 (370·2 to 540·4)	104·2 (86·9 to 125·4)	140·0 (108·8 to 176·4)	103·2 (90·7 to 122·3)
Jamaica*	2033	414·3 (366·6 to 466·6)	231·7 (188·3 to 280·6)	53·2 (44·4 to 64·0)	73·4 (57·0 to 92·1)	56·1 (48·9 to 66·9)	811·2 (717·7 to 913·5)	453·6 (368·7 to 549·4)	104·1 (86·9 to 125·3)	143·7 (111·7 to 180·3)	109·7 (95·7 to 131·0)

Jamaica*	2034	422·1 (371·2 to 479·4)	233·9 (188·5 to 286·2)	53·1 (44·3 to 63·9)	75·3 (58·4 to 94·3)	59·8 (51·8 to 71·9)	826·3 (726·7 to 938·5)	457·9 (369·0 to 560·3)	103·9 (86·7 to 125·1)	147·4 (114·4 to 184·6)	117·1 (101·4 to 140·8)
Jamaica*	2035	430·0 (377·4 to 487·2)	235·9 (188·3 to 289·1)	53·0 (44·2 to 63·7)	77·2 (59·9 to 97·1)	63·9 (55·0 to 77·7)	841·8 (738·8 to 953·7)	461·7 (368·6 to 566·1)	103·7 (86·5 to 124·8)	151·2 (117·2 to 190·1)	125·2 (107·6 to 152·0)
Jamaica*	2036	438·8 (384·7 to 501·0)	238·6 (191·4 to 295·2)	52·9 (44·1 to 63·6)	79·2 (61·4 to 100·1)	68·2 (58·4 to 83·7)	859·1 (753·2 to 980·9)	467·0 (374·7 to 577·9)	103·5 (86·4 to 124·6)	155·0 (120·2 to 195·9)	133·5 (114·3 to 163·9)
Jamaica*	2037	447·1 (391·8 to 511·7)	240·3 (194·3 to 299·2)	52·8 (44·0 to 63·6)	81·2 (62·8 to 103·2)	72·7 (62·0 to 90·2)	875·3 (767·0 to 1001·7)	470·5 (380·5 to 585·7)	103·4 (86·2 to 124·5)	159·0 (122·9 to 202·0)	142·4 (121·4 to 176·6)
Jamaica*	2038	456·0 (397·9 to 522·5)	242·5 (193·2 to 302·9)	52·8 (44·0 to 63·5)	83·3 (64·2 to 106·5)	77·4 (65·5 to 97·0)	892·7 (779·0 to 1023·0)	474·7 (378·2 to 593·1)	103·3 (86·1 to 124·4)	163·1 (125·6 to 208·6)	151·5 (128·2 to 189·9)
Jamaica*	2039	465·4 (406·5 to 535·6)	244·9 (194·6 to 307·9)	52·8 (44·0 to 63·5)	85·6 (65·9 to 109·8)	82·2 (69·1 to 103·4)	911·2 (795·9 to 1048·7)	479·4 (381·0 to 602·8)	103·3 (86·1 to 124·4)	167·5 (129·0 to 214·9)	161·0 (135·3 to 202·3)
Jamaica*	2040	475·4 (413·8 to 547·5)	247·3 (195·7 to 310·6)	52·8 (44·0 to 63·6)	87·8 (67·5 to 113·4)	87·4 (72·7 to 110·4)	930·7 (810·1 to 1072·0)	484·2 (383·2 to 608·2)	103·3 (86·1 to 124·5)	172·0 (132·1 to 222·1)	171·2 (142·4 to 216·1)
Jamaica*	2041	485·4 (422·3 to 559·4)	249·6 (197·5 to 317·2)	52·8 (44·0 to 63·6)	90·2 (69·4 to 117·7)	92·8 (77·2 to 118·2)	950·3 (826·7 to 1095·2)	488·6 (386·7 to 621·0)	103·4 (86·2 to 124·5)	176·6 (135·9 to 230·5)	181·7 (151·1 to 231·4)
Jamaica*	2042	495·9 (428·5 to 571·5)	251·8 (197·0 to 321·1)	52·8 (44·0 to 63·6)	92·6 (71·1 to 121·2)	98·7 (81·6 to 126·8)	970·9 (839·0 to 1118·9)	492·9 (385·8 to 628·7)	103·4 (86·1 to 124·5)	181·3 (139·2 to 237·4)	193·3 (159·7 to 248·2)
Jamaica*	2043	506·6 (437·8 to 585·8)	253·9 (197·6 to 323·5)	52·8 (44·0 to 63·7)	95·1 (72·5 to 124·6)	104·8 (85·7 to 135·2)	991·8 (857·2 to 1146·9)	497·0 (386·8 to 633·4)	103·4 (86·1 to 124·6)	186·2 (142·0 to 244·0)	205·1 (167·8 to 264·7)
Jamaica*	2044	518·4 (446·7 to 600·5)	256·5 (199·0 to 329·0)	52·9 (44·0 to 63·7)	97·7 (74·2 to 128·6)	111·4 (90·4 to 144·8)	1014·8 (874·5 to 1175·6)	502·2 (389·6 to 644·2)	103·5 (86·1 to 124·7)	191·2 (145·2 to 251·8)	218·0 (176·9 to 283·5)
Jamaica*	2045	530·0 (457·9 to 613·7)	258·8 (202·7 to 330·2)	52·9 (44·0 to 63·7)	100·2 (75·5 to 132·6)	118·2 (95·0 to 154·8)	1037·7 (896·4 to 1201·5)	506·6 (396·9 to 646·5)	103·5 (86·1 to 124·7)	196·2 (147·7 to 259·7)	231·4 (185·9 to 303·0)
Jamaica*	2046	542·5 (468·4 to 626·7)	261·0 (203·4 to 335·7)	52·8 (43·9 to 63·7)	102·8 (76·8 to 136·6)	125·8 (99·8 to 165·3)	1062·0 (917·0 to 1226·9)	511·0 (398·2 to 657·1)	103·4 (86·0 to 124·6)	201·3 (150·3 to 267·5)	246·3 (195·4 to 323·6)
Jamaica*	2047	555·5 (477·0 to 644·6)	263·3 (203·4 to 339·4)	52·8 (43·9 to 63·6)	105·5 (78·0 to 141·2)	133·9 (105·8 to 176·9)	1087·5 (933·9 to 1262·0)	515·5 (398·1 to 664·4)	103·3 (85·9 to 124·5)	206·5 (152·7 to 276·5)	262·2 (207·2 to 346·3)

Jamaica*	2048	569·0 (486·5 to 662·3)	265·5 (204·6 to 341·7)	52·6 (43·8 to 63·5)	108·1 (79·1 to 146·3)	142·6 (112·8 to 186·8)	1113·9 (952·5 to 1296·5)	519·8 (400·6 to 668·9)	103·1 (85·7 to 124·2)	211·7 (154·9 to 286·5)	279·2 (220·9 to 365·8)
Jamaica*	2049	583·0 (499·4 to 677·7)	267·5 (207·5 to 344·6)	52·5 (43·6 to 63·3)	110·8 (80·1 to 150·6)	152·2 (118·8 to 201·8)	1141·4 (977·6 to 1326·8)	523·7 (406·3 to 674·7)	102·8 (85·4 to 123·9)	217·0 (156·9 to 294·8)	297·9 (232·6 to 395·1)
Jamaica*	2050	597·8 (509·0 to 697·9)	269·6 (206·9 to 348·2)	52·3 (43·4 to 63·1)	113·6 (81·3 to 155·2)	162·4 (126·5 to 215·4)	1170·4 (996·5 to 1366·4)	527·8 (405·0 to 681·7)	102·5 (85·0 to 123·5)	222·3 (159·1 to 303·9)	317·9 (247·7 to 421·6)
Jordan*	1995	286·0 (263·4 to 313·9)	180·3 (161·6 to 200·9)	73·9 (62·7 to 88·0)	25·8 (18·6 to 35·0)	6·0 (6·0 to 6·0)	685·2 (631·0 to 751·8)	431·8 (387·2 to 481·2)	177·1 (150·1 to 210·9)	61·8 (44·6 to 83·9)	14·4 (14·4 to 14·4)
Jordan*	1996	290·8 (270·0 to 315·1)	180·8 (163·7 to 199·3)	80·5 (69·2 to 93·5)	26·7 (19·9 to 35·5)	2·9 (2·9 to 2·9)	696·6 (646·7 to 754·7)	433·0 (392·1 to 477·3)	192·8 (165·7 to 224·1)	63·9 (47·7 to 85·0)	6·9 (6·9 to 6·9)
Jordan*	1997	299·3 (280·0 to 319·8)	177·5 (162·7 to 192·6)	89·7 (78·6 to 101·6)	27·5 (20·6 to 35·8)	4·6 (4·6 to 4·6)	717·0 (670·6 to 766·0)	425·1 (389·7 to 461·4)	214·9 (188·3 to 243·4)	65·9 (49·3 to 85·8)	11·0 (11·0 to 11·0)
Jordan*	1998	319·9 (301·8 to 339·5)	181·3 (166·9 to 195·9)	102·5 (91·7 to 115·3)	29·6 (22·9 to 37·6)	6·5 (6·5 to 6·5)	766·1 (722·8 to 813·2)	434·3 (399·8 to 469·2)	245·4 (219·6 to 276·2)	70·9 (54·9 to 90·0)	15·5 (15·5 to 15·5)
Jordan*	1999	341·4 (323·9 to 360·2)	177·4 (165·1 to 190·4)	118·5 (107·8 to 131·4)	32·9 (25·8 to 41·2)	12·5 (12·5 to 12·5)	817·7 (775·7 to 862·8)	424·8 (395·4 to 456·0)	283·9 (258·2 to 314·8)	78·9 (61·9 to 98·6)	30·1 (30·1 to 30·1)
Jordan*	2000	373·7 (355·7 to 391·8)	179·7 (168·1 to 191·3)	139·6 (127·2 to 152·5)	43·9 (34·2 to 54·6)	10·4 (10·4 to 10·4)	895·0 (852·0 to 938·5)	430·4 (402·6 to 458·2)	334·4 (304·6 to 365·4)	105·1 (82·0 to 130·8)	25·0 (25·0 to 25·0)
Jordan*	2001	391·1 (372·4 to 410·2)	180·7 (170·5 to 191·3)	154·0 (140·9 to 167·0)	47·3 (36·9 to 59·3)	9·2 (9·2 to 9·2)	936·9 (891·9 to 982·6)	432·8 (408·5 to 458·2)	369·0 (337·6 to 399·9)	113·3 (88·5 to 142·0)	21·9 (21·9 to 21·9)
Jordan*	2002	402·4 (383·4 to 422·4)	185·2 (175·2 to 195·9)	164·3 (151·3 to 177·5)	42·8 (33·3 to 54·1)	10·0 (10·0 to 10·0)	963·8 (918·5 to 1011·8)	443·7 (419·6 to 469·2)	393·6 (362·4 to 425·1)	102·6 (79·8 to 129·6)	23·9 (23·9 to 23·9)
Jordan*	2003	399·3 (381·0 to 419·6)	186·9 (176·1 to 197·9)	168·2 (155·7 to 182·1)	34·0 (26·3 to 42·9)	10·2 (10·2 to 10·2)	956·5 (912·7 to 1005·0)	447·6 (421·9 to 474·1)	403·0 (373·0 to 436·3)	81·3 (62·9 to 102·8)	24·5 (24·5 to 24·5)
Jordan*	2004	383·2 (366·0 to 401·4)	183·9 (173·3 to 194·2)	164·5 (152·4 to 178·3)	26·6 (20·3 to 34·1)	8·1 (8·1 to 8·1)	917·9 (876·6 to 961·6)	440·5 (415·1 to 465·2)	394·1 (365·0 to 427·0)	63·8 (48·6 to 81·6)	19·5 (19·5 to 19·5)
Jordan*	2005	382·7 (365·4 to 401·5)	196·5 (185·3 to 206·6)	158·8 (146·6 to 172·7)	22·8 (17·1 to 29·5)	4·6 (4·6 to 4·6)	916·7 (875·2 to 961·8)	470·6 (444·0 to 494·9)	380·4 (351·2 to 413·7)	54·7 (40·9 to 70·7)	11·0 (11·0 to 11·0)

Jordan*	2006	372·4 (355·1 to 390·1)	202·3 (192·2 to 213·0)	147·0 (135·3 to 160·6)	19·8 (14·5 to 25·8)	3·4 (3·4 to 3·4)	892·0 (850·5 to 934·5)	484·6 (460·4 to 510·2)	352·0 (324·1 to 384·8)	47·4 (34·8 to 61·8)	8·0 (8·0 to 8·0)
Jordan*	2007	397·7 (379·7 to 414·4)	234·6 (223·5 to 245·8)	139·8 (128·3 to 153·0)	20·5 (14·9 to 26·8)	2·7 (2·7 to 2·7)	952·6 (909·4 to 992·6)	562·0 (535·2 to 588·8)	334·9 (307·3 to 366·6)	49·2 (35·8 to 64·2)	6·5 (6·5 to 6·5)
Jordan*	2008	420·2 (402·5 to 438·5)	261·4 (250·0 to 273·6)	127·7 (116·5 to 139·8)	25·2 (18·8 to 32·9)	5·9 (5·9 to 5·9)	1006·6 (964·1 to 1050·3)	626·1 (598·8 to 655·2)	305·9 (279·1 to 334·9)	60·4 (45·1 to 78·7)	14·2 (14·2 to 14·2)
Jordan*	2009	440·2 (421·7 to 459·1)	291·3 (279·2 to 303·7)	113·6 (102·3 to 124·7)	29·1 (21·6 to 37·9)	6·2 (6·2 to 6·2)	1054·4 (1010·1 to 1099·6)	697·9 (668·8 to 727·6)	272·0 (244·9 to 298·7)	69·6 (51·8 to 90·7)	14·9 (14·9 to 14·9)
Jordan*	2010	403·2 (386·7 to 420·5)	270·5 (259·5 to 281·8)	97·6 (87·3 to 108·0)	29·3 (22·2 to 37·9)	5·9 (5·9 to 5·9)	965·8 (926·3 to 1007·2)	647·9 (621·5 to 674·9)	233·7 (209·0 to 258·8)	70·2 (53·1 to 90·8)	14·0 (14·0 to 14·0)
Jordan*	2011	395·3 (377·5 to 413·6)	267·0 (255·6 to 279·0)	90·9 (81·3 to 101·5)	31·4 (24·2 to 40·5)	6·0 (6·0 to 6·0)	946·9 (904·3 to 990·7)	639·5 (612·3 to 668·2)	217·7 (194·7 to 243·1)	75·2 (58·0 to 97·0)	14·4 (14·4 to 14·4)
Jordan*	2012	369·1 (352·9 to 385·4)	240·1 (229·1 to 251·5)	85·6 (77·2 to 95·3)	31·3 (24·2 to 40·2)	12·1 (12·1 to 12·1)	884·1 (845·3 to 923·1)	575·1 (548·8 to 602·4)	205·0 (184·8 to 228·2)	75·1 (57·9 to 96·4)	28·9 (28·9 to 28·9)
Jordan*	2013	330·8 (316·7 to 345·7)	203·4 (193·2 to 213·9)	81·7 (74·8 to 89·4)	32·3 (25·1 to 41·0)	13·5 (13·5 to 13·5)	792·5 (758·6 to 827·9)	487·1 (462·7 to 512·5)	195·6 (179·2 to 214·0)	77·3 (60·1 to 98·1)	32·4 (32·4 to 32·4)
Jordan*	2014	327·3 (313·7 to 341·4)	196·2 (185·9 to 207·4)	85·7 (80·7 to 91·0)	35·6 (27·7 to 44·3)	9·8 (9·8 to 9·8)	783·9 (751·4 to 817·7)	469·9 (445·4 to 496·9)	205·3 (193·2 to 218·0)	85·4 (66·3 to 106·0)	23·4 (23·4 to 23·4)
Jordan*	2015	311·0 (297·0 to 326·8)	172·4 (161·6 to 183·3)	92·8 (89·5 to 96·1)	44·3 (35·2 to 55·7)	1·6 (1·6 to 1·6)	745·0 (711·4 to 782·7)	412·9 (387·1 to 439·0)	222·3 (214·3 to 230·2)	106·1 (84·3 to 133·3)	3·8 (3·8 to 3·8)
Jordan*	2016	293·3 (278·6 to 308·9)	148·9 (138·7 to 159·4)	94·8 (91·4 to 98·1)	47·0 (37·0 to 57·9)	2·7 (2·7 to 2·7)	702·6 (667·3 to 739·9)	356·6 (332·1 to 381·7)	227·1 (219·0 to 234·9)	112·7 (88·6 to 138·7)	6·4 (6·4 to 6·4)
Jordan*	2017	300·2 (283·5 to 317·6)	146·4 (133·8 to 159·7)	98·5 (95·3 to 101·8)	49·5 (38·6 to 61·5)	5·8 (5·8 to 5·8)	719·2 (679·0 to 760·7)	350·7 (320·6 to 382·6)	236·0 (228·4 to 244·0)	118·5 (92·4 to 147·3)	14·0 (14·0 to 14·0)
Jordan*	2018	304·9 (283·4 to 328·0)	149·8 (133·5 to 167·4)	99·3 (93·1 to 105·6)	50·2 (38·5 to 62·8)	5·6 (5·6 to 5·6)	730·4 (678·7 to 785·7)	358·8 (319·8 to 400·9)	237·7 (223·0 to 253·0)	120·4 (92·2 to 150·5)	13·5 (13·5 to 13·5)
Jordan*	2019	306·4 (283·5 to 330·8)	149·5 (132·7 to 168·2)	99·5 (93·4 to 105·9)	51·6 (39·7 to 64·8)	5·8 (5·7 to 5·8)	733·9 (679·1 to 792·4)	358·0 (318·0 to 403·0)	238·4 (223·7 to 253·7)	123·7 (95·0 to 155·3)	13·8 (13·8 to 13·9)

Jordan*	2020	305·4 (283·1 to 329·0)	155·1 (138·7 to 173·5)	92·5 (86·8 to 98·4)	49·5 (37·9 to 62·2)	8·3 (8·2 to 8·6)	731·5 (678·0 to 788·1)	371·4 (332·2 to 415·6)	221·6 (208·0 to 235·8)	118·5 (90·8 to 148·9)	19·9 (19·5 to 20·6)
Jordan*	2021	284·2 (262·1 to 307·5)	141·1 (124·6 to 159·5)	86·4 (81·1 to 91·9)	47·6 (36·4 to 59·5)	9·0 (8·8 to 9·7)	680·7 (627·8 to 736·5)	338·1 (298·5 to 382·0)	207·0 (194·2 to 220·2)	114·0 (87·1 to 142·5)	21·7 (21·0 to 23·2)
Jordan*	2022	278·6 (255·2 to 304·1)	135·0 (116·1 to 156·2)	87·3 (82·0 to 92·8)	49·4 (37·8 to 61·9)	6·9 (6·5 to 7·6)	667·3 (611·2 to 728·4)	323·3 (278·1 to 374·0)	209·2 (196·4 to 222·4)	118·2 (90·5 to 148·3)	16·6 (15·5 to 18·1)
Jordan*	2023	284·7 (259·4 to 312·8)	138·4 (118·1 to 163·0)	88·2 (82·8 to 93·7)	51·1 (39·0 to 64·0)	7·0 (6·5 to 7·7)	681·8 (621·4 to 749·2)	331·5 (283·0 to 390·4)	211·2 (198·2 to 224·5)	122·4 (93·4 to 153·3)	16·7 (15·5 to 18·4)
Jordan*	2024	290·9 (263·8 to 321·4)	142·0 (119·9 to 169·6)	89·1 (83·6 to 94·7)	52·9 (40·3 to 66·6)	7·0 (6·5 to 7·8)	696·9 (631·9 to 769·8)	340·0 (287·1 to 406·2)	213·4 (200·2 to 226·9)	126·6 (96·4 to 159·4)	16·9 (15·6 to 18·7)
Jordan*	2025	297·3 (268·2 to 329·3)	145·7 (121·6 to 174·9)	89·9 (84·4 to 95·7)	54·6 (41·5 to 68·9)	7·1 (6·5 to 7·9)	712·1 (642·5 to 788·7)	348·9 (291·4 to 419·0)	215·4 (202·0 to 229·3)	130·8 (99·5 to 165·0)	17·0 (15·6 to 18·8)
Jordan*	2026	303·4 (272·8 to 337·6)	149·1 (122·3 to 180·3)	90·8 (85·0 to 96·6)	56·4 (42·9 to 71·3)	7·2 (6·5 to 8·0)	726·8 (653·5 to 808·6)	357·1 (292·9 to 431·9)	217·4 (203·7 to 231·5)	135·1 (102·8 to 170·8)	17·2 (15·7 to 19·2)
Jordan*	2027	309·6 (276·9 to 346·3)	152·5 (124·8 to 186·2)	91·5 (85·7 to 97·5)	58·3 (44·4 to 73·4)	7·2 (6·6 to 8·1)	741·6 (663·3 to 829·5)	365·4 (298·9 to 445·9)	219·3 (205·3 to 233·5)	139·6 (106·3 to 175·9)	17·3 (15·8 to 19·5)
Jordan*	2028	315·2 (280·6 to 353·0)	155·6 (126·6 to 189·1)	92·2 (86·3 to 98·2)	60·1 (45·8 to 75·9)	7·3 (6·6 to 8·3)	755·1 (672·2 to 845·4)	372·7 (303·2 to 452·9)	220·7 (206·7 to 235·2)	144·0 (109·8 to 181·9)	17·6 (15·9 to 19·9)
Jordan*	2029	320·8 (284·8 to 360·0)	158·8 (128·0 to 194·6)	92·6 (86·7 to 98·7)	61·9 (47·1 to 78·2)	7·5 (6·7 to 8·6)	768·4 (682·2 to 862·3)	380·4 (306·7 to 466·2)	221·8 (207·7 to 236·5)	148·2 (112·8 to 187·3)	17·9 (16·1 to 20·5)
Jordan*	2030	325·3 (287·6 to 366·8)	160·6 (128·2 to 196·5)	93·1 (87·1 to 99·2)	64·0 (48·7 to 80·8)	7·6 (6·8 to 8·8)	779·3 (688·9 to 878·6)	384·7 (307·0 to 470·8)	222·9 (208·6 to 237·6)	153·4 (116·6 to 193·5)	18·3 (16·4 to 21·0)
Jordan*	2031	331·8 (292·4 to 375·3)	164·0 (129·6 to 202·1)	93·6 (87·5 to 99·8)	66·4 (50·5 to 84·3)	7·8 (6·9 to 9·1)	794·8 (700·4 to 899·0)	392·9 (310·4 to 484·1)	224·1 (209·6 to 239·1)	159·1 (121·0 to 201·9)	18·7 (16·6 to 21·7)
Jordan*	2032	339·1 (297·7 to 383·9)	168·3 (132·8 to 208·7)	94·0 (87·9 to 100·4)	68·8 (52·5 to 87·6)	8·0 (7·1 to 9·3)	812·3 (713·0 to 919·6)	403·2 (318·1 to 500·0)	225·1 (210·6 to 240·4)	164·9 (125·8 to 209·9)	19·1 (16·9 to 22·3)
Jordan*	2033	346·5 (303·8 to 393·4)	172·7 (135·5 to 215·6)	94·3 (88·4 to 100·7)	71·3 (53·9 to 91·6)	8·2 (7·2 to 9·6)	830·1 (727·6 to 942·3)	413·7 (324·7 to 516·3)	225·9 (211·7 to 241·3)	170·8 (129·1 to 219·4)	19·6 (17·3 to 23·1)

Jordan*	2034	353·8 (309·9 to 402·6)	177·0 (138·8 to 219·6)	94·6 (88·6 to 100·9)	73·8 (55·5 to 94·8)	8·5 (7·4 to 10·0)	847·5 (742·3 to 964·3)	424·0 (332·6 to 526·1)	226·5 (212·2 to 241·7)	176·8 (133·1 to 227·1)	20·3 (17·8 to 24·0)
Jordan*	2035	361·2 (315·8 to 412·6)	181·4 (142·3 to 225·4)	94·7 (88·8 to 101·2)	76·3 (57·4 to 98·7)	8·8 (7·6 to 10·4)	865·2 (756·5 to 988·3)	434·5 (340·9 to 539·9)	226·9 (212·6 to 242·5)	182·8 (137·4 to 236·3)	21·0 (18·2 to 24·9)
Jordan*	2036	368·3 (322·1 to 421·5)	185·5 (145·6 to 232·4)	94·9 (88·9 to 101·4)	78·9 (59·2 to 102·8)	9·1 (7·8 to 10·9)	882·3 (771·5 to 1009·6)	444·3 (348·7 to 556·7)	227·3 (212·9 to 242·9)	188·9 (141·9 to 246·2)	21·7 (18·8 to 26·0)
Jordan*	2037	376·2 (327·8 to 430·8)	190·0 (149·2 to 239·6)	95·2 (89·2 to 101·8)	81·6 (60·9 to 107·4)	9·3 (8·1 to 11·3)	901·0 (785·2 to 1031·8)	455·2 (357·3 to 573·8)	228·1 (213·6 to 243·8)	195·4 (145·8 to 257·2)	22·3 (19·4 to 27·1)
Jordan*	2038	383·6 (332·4 to 441·8)	194·1 (150·6 to 247·9)	95·5 (89·4 to 102·2)	84·3 (62·5 to 112·0)	9·6 (8·3 to 11·7)	918·8 (796·1 to 1058·2)	465·0 (360·8 to 593·7)	228·7 (214·2 to 244·8)	202·0 (149·7 to 268·2)	23·0 (19·8 to 28·1)
Jordan*	2039	390·5 (339·0 to 451·1)	197·9 (153·5 to 252·3)	95·7 (89·6 to 102·6)	87·0 (64·1 to 116·5)	10·0 (8·5 to 12·2)	935·5 (811·9 to 1080·5)	474·0 (367·7 to 604·3)	229·2 (214·5 to 245·7)	208·4 (153·5 to 279·0)	23·9 (20·4 to 29·2)
Jordan*	2040	397·9 (343·9 to 461·4)	201·9 (156·0 to 257·3)	95·9 (89·7 to 103·0)	89·8 (65·7 to 120·8)	10·3 (8·7 to 12·8)	953·2 (823·7 to 1105·2)	483·7 (373·8 to 616·2)	229·6 (214·9 to 246·6)	215·1 (157·4 to 289·3)	24·8 (20·9 to 30·6)
Jordan*	2041	405·2 (349·6 to 471·1)	205·8 (159·0 to 262·5)	96·0 (89·9 to 103·3)	92·6 (67·5 to 124·7)	10·7 (9·1 to 13·3)	970·7 (837·4 to 1128·4)	493·0 (380·9 to 628·7)	230·1 (215·4 to 247·3)	221·9 (161·7 to 298·8)	25·7 (21·7 to 32·0)
Jordan*	2042	412·7 (355·7 to 479·6)	209·8 (163·0 to 268·7)	96·2 (90·1 to 103·6)	95·6 (69·2 to 129·7)	11·1 (9·3 to 13·9)	988·5 (851·9 to 1148·9)	502·5 (390·5 to 643·5)	230·5 (215·7 to 248·0)	228·9 (165·8 to 310·6)	26·6 (22·3 to 33·4)
Jordan*	2043	419·8 (361·9 to 487·8)	213·4 (164·7 to 271·5)	96·3 (90·1 to 103·8)	98·5 (71·2 to 134·3)	11·6 (9·6 to 14·6)	1005·6 (866·9 to 1168·5)	511·2 (394·6 to 650·3)	230·8 (215·8 to 248·6)	235·9 (170·5 to 321·7)	27·7 (23·0 to 35·0)
Jordan*	2044	427·2 (367·7 to 496·6)	217·3 (166·0 to 277·3)	96·4 (90·0 to 103·9)	101·5 (72·9 to 139·9)	12·0 (10·0 to 15·3)	1023·2 (880·7 to 1189·4)	520·4 (397·5 to 664·3)	230·9 (215·7 to 248·8)	243·1 (174·5 to 335·0)	28·9 (23·9 to 36·8)
Jordan*	2045	434·5 (372·4 to 506·3)	220·9 (169·3 to 281·1)	96·5 (90·1 to 104·1)	104·6 (74·2 to 144·7)	12·5 (10·2 to 16·1)	1040·8 (892·1 to 1212·7)	529·2 (405·5 to 673·4)	231·2 (215·8 to 249·3)	250·5 (177·8 to 346·6)	30·0 (24·5 to 38·6)
Jordan*	2046	442·6 (378·5 to 517·1)	225·0 (170·7 to 287·4)	96·8 (90·3 to 104·4)	107·9 (76·0 to 151·0)	13·0 (10·6 to 16·7)	1060·1 (906·7 to 1238·5)	538·9 (408·9 to 688·4)	231·7 (216·2 to 250·1)	258·4 (182·0 to 361·7)	31·0 (25·3 to 40·0)
Jordan*	2047	450·4 (384·0 to 526·3)	228·8 (174·3 to 295·2)	97·0 (90·4 to 104·7)	111·2 (77·4 to 157·4)	13·4 (10·8 to 17·4)	1078·9 (919·8 to 1260·6)	548·1 (417·5 to 707·0)	232·2 (216·6 to 250·8)	266·3 (185·5 to 377·1)	32·2 (25·9 to 41·7)

Jordan*	2048	458·5 (389·3 to 536·9)	232·8 (178·3 to 301·2)	97·2 (90·7 to 105·0)	114·6 (79·7 to 163·9)	13·9 (11·2 to 18·0)	1098·1 (932·5 to 1285·9)	557·6 (427·1 to 721·5)	232·8 (217·2 to 251·5)	274·4 (190·9 to 392·5)	33·2 (26·8 to 43·0)
Jordan*	2049	466·4 (396·9 to 548·6)	236·5 (179·9 to 307·4)	97·5 (90·9 to 105·3)	118·1 (81·5 to 170·1)	14·3 (11·4 to 18·8)	1117·3 (950·7 to 1314·0)	566·6 (430·9 to 736·3)	233·5 (217·8 to 252·3)	282·8 (195·1 to 407·5)	34·3 (27·3 to 45·0)
Jordan*	2050	474·8 (403·4 to 559·9)	240·5 (182·7 to 309·4)	97·8 (91·2 to 105·8)	121·7 (83·4 to 176·5)	14·8 (11·7 to 19·4)	1137·3 (966·3 to 1341·0)	576·0 (437·5 to 741·2)	234·4 (218·4 to 253·4)	291·6 (199·7 to 422·7)	35·3 (28·0 to 46·4)
Japan	1995	1925·0 (1828·1 to 2033·6)	1560·2 (1467·4 to 1661·0)	292·6 (271·1 to 315·7)	72·2 (58·3 to 87·4)	0·0 (0·0 to 0·0)	2052·6 (1949·3 to 2168·4)	1663·6 (1564·7 to 1771·2)	312·0 (289·1 to 336·6)	77·0 (62·2 to 93·2)	0·0 (0·0 to 0·0)
Japan	1996	2001·7 (1921·7 to 2085·9)	1619·3 (1543·0 to 1698·1)	307·4 (290·8 to 324·5)	75·0 (63·0 to 88·6)	0·0 (0·0 to 0·0)	2134·4 (2049·1 to 2224·1)	1726·7 (1645·3 to 1810·6)	327·7 (310·1 to 346·1)	80·0 (67·2 to 94·4)	0·0 (0·0 to 0·0)
Japan	1997	2053·5 (1978·1 to 2130·2)	1647·0 (1578·5 to 1717·8)	329·3 (313·0 to 346·1)	77·2 (66·2 to 89·2)	0·0 (0·0 to 0·0)	2189·7 (2109·3 to 2271·5)	1756·2 (1683·1 to 1831·7)	351·1 (333·7 to 369·1)	82·3 (70·6 to 95·1)	0·0 (0·0 to 0·0)
Japan	1998	2221·5 (2146·1 to 2303·9)	1781·7 (1707·1 to 1860·3)	357·6 (341·0 to 374·6)	82·2 (72·9 to 92·4)	0·0 (0·0 to 0·0)	2368·7 (2288·4 to 2456·7)	1899·8 (1820·3 to 1983·7)	381·3 (363·6 to 399·5)	87·7 (77·7 to 98·6)	0·0 (0·0 to 0·0)
Japan	1999	2268·4 (2191·4 to 2350·7)	1811·1 (1735·6 to 1890·2)	372·8 (358·9 to 387·6)	84·5 (77·8 to 91·5)	0·0 (0·0 to 0·0)	2418·8 (2336·6 to 2506·6)	1931·2 (1850·6 to 2015·5)	397·5 (382·7 to 413·3)	90·1 (83·0 to 97·5)	0·0 (0·0 to 0·0)
Japan	2000	2444·3 (2365·0 to 2525·5)	1963·1 (1884·7 to 2041·2)	392·4 (382·6 to 403·1)	88·8 (85·2 to 92·5)	0·0 (0·0 to 0·0)	2606·4 (2521·8 to 2692·9)	2093·2 (2009·6 to 2176·5)	418·4 (408·0 to 429·8)	94·7 (90·9 to 98·6)	0·0 (0·0 to 0·0)
Japan	2001	2528·0 (2454·1 to 2606·8)	2038·9 (1963·7 to 2116·2)	403·9 (394·8 to 413·2)	85·1 (82·2 to 88·1)	0·0 (0·0 to 0·0)	2695·6 (2616·7 to 2779·7)	2174·1 (2093·9 to 2256·5)	430·7 (420·9 to 440·6)	90·8 (87·7 to 94·0)	0·0 (0·0 to 0·0)
Japan	2002	2615·2 (2535·7 to 2693·5)	2109·7 (2030·2 to 2186·9)	421·3 (412·2 to 431·1)	84·2 (81·1 to 87·1)	0·0 (0·0 to 0·0)	2788·6 (2703·8 to 2872·1)	2249·6 (2164·7 to 2331·9)	449·3 (439·5 to 459·7)	89·7 (86·5 to 92·9)	0·0 (0·0 to 0·0)
Japan	2003	2700·5 (2615·9 to 2780·0)	2170·6 (2085·9 to 2250·9)	445·3 (436·0 to 455·2)	84·6 (81·6 to 87·5)	0·0 (0·0 to 0·0)	2879·5 (2789·3 to 2964·3)	2314·5 (2224·2 to 2400·1)	474·8 (465·0 to 485·4)	90·2 (87·0 to 93·3)	0·0 (0·0 to 0·0)
Japan	2004	2745·7 (2667·4 to 2821·4)	2206·0 (2126·8 to 2281·2)	453·5 (443·9 to 462·7)	86·1 (83·2 to 89·0)	0·0 (0·0 to 0·0)	2927·7 (2844·3 to 3008·5)	2352·3 (2267·7 to 2432·4)	483·6 (473·3 to 493·4)	91·8 (88·8 to 94·9)	0·0 (0·0 to 0·0)
Japan	2005	2825·0 (2748·3 to 2905·3)	2278·9 (2203·4 to 2357·5)	456·5 (447·5 to 466·0)	89·6 (86·9 to 92·5)	0·0 (0·0 to 0·0)	3012·3 (2930·5 to 3097·9)	2430·0 (2349·5 to 2513·8)	486·7 (477·1 to 496·9)	95·6 (92·6 to 98·6)	0·0 (0·0 to 0·0)

Japan	2006	2877·4 (2799·8 to 2961·7)	2320·1 (2243·1 to 2400·8)	464·0 (455·0 to 473·4)	93·3 (90·3 to 96·2)	0·0 (0·0 to 0·0)	3068·1 (2985·4 to 3158·1)	2473·9 (2391·8 to 2560·0)	494·7 (485·2 to 504·8)	99·4 (96·3 to 102·6)	0·0 (0·0 to 0·0)
Japan	2007	2945·6 (2862·5 to 3025·8)	2383·5 (2301·2 to 2462·6)	463·9 (454·5 to 473·5)	98·3 (95·3 to 101·3)	0·0 (0·0 to 0·0)	3140·9 (3052·3 to 3226·4)	2541·5 (2453·8 to 2625·8)	494·6 (484·7 to 504·8)	104·8 (101·6 to 108·0)	0·0 (0·0 to 0·0)
Japan	2008	3032·7 (2950·8 to 3115·4)	2459·2 (2376·1 to 2538·5)	466·2 (457·1 to 475·9)	107·2 (104·3 to 110·3)	0·0 (0·0 to 0·0)	3233·7 (3146·4 to 3322·0)	2622·2 (2533·7 to 2706·8)	497·1 (487·4 to 507·5)	114·4 (111·2 to 117·6)	0·0 (0·0 to 0·0)
Japan	2009	3232·1 (3147·7 to 3317·5)	2639·2 (2556·9 to 2725·3)	479·2 (469·7 to 489·1)	113·7 (110·4 to 116·8)	0·0 (0·0 to 0·0)	3446·4 (3356·3 to 3537·5)	2814·2 (2726·4 to 2905·9)	511·0 (500·9 to 521·6)	121·2 (117·7 to 124·6)	0·0 (0·0 to 0·0)
Japan	2010	3450·3 (3364·8 to 3536·8)	2841·5 (2757·6 to 2927·1)	491·3 (481·7 to 501·3)	117·5 (114·5 to 120·9)	0·0 (0·0 to 0·0)	3679·0 (3587·9 to 3771·2)	3029·8 (2940·5 to 3121·2)	523·9 (513·6 to 534·5)	125·3 (122·1 to 128·9)	0·0 (0·0 to 0·0)
Japan	2011	3768·2 (3682·4 to 3858·0)	3138·9 (3051·8 to 3228·2)	507·7 (498·8 to 517·6)	121·6 (118·4 to 124·8)	0·0 (0·0 to 0·0)	4018·0 (3926·5 to 4113·8)	3347·0 (3254·1 to 3442·2)	541·4 (531·9 to 551·9)	129·7 (126·3 to 133·1)	0·0 (0·0 to 0·0)
Japan	2012	3990·1 (3906·3 to 4080·0)	3347·8 (3261·7 to 3435·4)	518·1 (508·9 to 527·6)	124·1 (120·9 to 127·2)	0·0 (0·0 to 0·0)	4254·6 (4165·3 to 4350·4)	3569·8 (3477·9 to 3663·2)	552·5 (542·6 to 562·6)	132·3 (128·9 to 135·6)	0·0 (0·0 to 0·0)
Japan	2013	4125·4 (4038·2 to 4221·0)	3476·9 (3390·9 to 3571·9)	523·9 (513·9 to 533·8)	124·5 (121·5 to 127·9)	0·0 (0·0 to 0·0)	4398·9 (4305·9 to 4500·8)	3707·4 (3615·6 to 3808·7)	558·7 (548·0 to 569·1)	132·8 (129·6 to 136·4)	0·0 (0·0 to 0·0)
Japan	2014	4153·7 (4057·3 to 4248·2)	3497·6 (3402·2 to 3590·2)	531·8 (521·7 to 541·7)	124·2 (120·8 to 127·3)	0·0 (0·0 to 0·0)	4429·1 (4326·3 to 4529·9)	3729·5 (3627·8 to 3828·2)	567·1 (556·2 to 577·6)	132·5 (128·8 to 135·8)	0·0 (0·0 to 0·0)
Japan	2015	4190·3 (4099·4 to 4278·0)	3523·3 (3434·7 to 3608·7)	541·2 (531·3 to 551·4)	125·8 (122·6 to 129·1)	0·0 (0·0 to 0·0)	4468·1 (4371·1 to 4561·6)	3756·8 (3662·4 to 3848·0)	577·1 (566·6 to 588·0)	134·2 (130·7 to 137·7)	0·0 (0·0 to 0·0)
Japan	2016	4239·5 (4153·9 to 4333·6)	3565·0 (3477·9 to 3658·4)	543·8 (534·2 to 553·9)	130·7 (127·5 to 134·2)	0·0 (0·0 to 0·0)	4520·5 (4429·3 to 4620·9)	3801·4 (3708·4 to 3900·9)	579·8 (569·6 to 590·6)	139·3 (135·9 to 143·1)	0·0 (0·0 to 0·0)
Japan	2017	4307·5 (4211·5 to 4399·6)	3624·5 (3525·9 to 3718·9)	547·9 (537·6 to 557·6)	135·0 (131·7 to 138·5)	0·0 (0·0 to 0·0)	4593·0 (4490·7 to 4691·2)	3864·8 (3759·6 to 3965·4)	584·3 (573·3 to 594·5)	144·0 (140·5 to 147·7)	0·0 (0·0 to 0·0)
Japan	2018	4396·9 (4279·2 to 4522·3)	3698·0 (3583·8 to 3822·5)	559·8 (547·8 to 572·3)	139·1 (135·2 to 143·0)	0·0 (0·0 to 0·0)	4688·4 (4562·9 to 4822·2)	3943·1 (3821·4 to 4075·9)	597·0 (584·1 to 610·2)	148·3 (144·2 to 152·5)	0·0 (0·0 to 0·0)
Japan	2019	4489·3 (4371·7 to 4622·5)	3782·4 (3663·7 to 3914·1)	563·5 (551·3 to 575·9)	143·4 (139·2 to 147·6)	0·0 (0·0 to 0·0)	4787·0 (4661·6 to 4928·9)	4033·2 (3906·6 to 4173·6)	600·9 (587·8 to 614·1)	152·9 (148·4 to 157·4)	0·0 (0·0 to 0·0)

Japan	2020	5240·4 (5090·9 to 5402·8)	4567·8 (4414·9 to 4726·8)	533·7 (522·0 to 545·4)	138·9 (134·6 to 143·4)	0·0 (0·0 to 0·0)	5587·8 (5428·4 to 5760·9)	4870·7 (4707·5 to 5040·2)	569·1 (556·6 to 581·6)	148·1 (143·5 to 152·9)	0·0 (0·0 to 0·0)
Japan	2021	5105·3 (4959·5 to 5263·5)	4448·7 (4299·7 to 4603·5)	518·6 (507·2 to 530·2)	137·9 (133·1 to 142·6)	0·0 (0·0 to 0·0)	5443·7 (5288·3 to 5612·5)	4743·6 (4584·7 to 4908·7)	553·0 (540·8 to 565·3)	147·1 (141·9 to 152·0)	0·0 (0·0 to 0·0)
Japan	2022	4282·8 (4150·5 to 4431·7)	3619·3 (3486·5 to 3763·6)	521·6 (510·0 to 533·2)	141·9 (137·0 to 146·9)	0·0 (0·0 to 0·0)	4566·8 (4425·7 to 4725·5)	3859·3 (3717·7 to 4013·1)	556·2 (543·8 to 568·5)	151·3 (146·1 to 156·6)	0·0 (0·0 to 0·0)
Japan	2023	4354·2 (4216·2 to 4509·4)	3683·9 (3544·7 to 3837·6)	524·4 (512·7 to 536·1)	145·9 (140·6 to 151·8)	0·0 (0·0 to 0·0)	4642·9 (4495·7 to 4808·3)	3928·1 (3779·7 to 4092·0)	559·2 (546·7 to 571·7)	155·6 (149·9 to 161·9)	0·0 (0·0 to 0·0)
Japan	2024	4426·6 (4273·2 to 4594·1)	3749·0 (3592·7 to 3914·5)	527·5 (515·8 to 539·4)	150·1 (143·8 to 156·8)	0·0 (0·0 to 0·0)	4720·1 (4556·5 to 4898·7)	3997·5 (3830·9 to 4174·0)	562·5 (550·0 to 575·2)	160·1 (153·4 to 167·2)	0·0 (0·0 to 0·0)
Japan	2025	4498·4 (4326·2 to 4684·2)	3813·5 (3642·2 to 3996·3)	530·5 (518·6 to 542·5)	154·4 (146·9 to 162·3)	0·0 (0·0 to 0·0)	4796·6 (4613·0 to 4994·7)	4066·3 (3883·6 to 4261·2)	565·7 (553·0 to 578·5)	164·6 (156·7 to 173·0)	0·0 (0·0 to 0·0)
Japan	2026	4570·6 (4384·9 to 4770·3)	3878·4 (3687·5 to 4074·2)	533·4 (521·3 to 545·4)	158·8 (149·9 to 168·0)	0·0 (0·0 to 0·0)	4873·6 (4675·6 to 5086·5)	4135·5 (3932·0 to 4344·3)	568·8 (555·9 to 581·6)	169·3 (159·9 to 179·2)	0·0 (0·0 to 0·0)
Japan	2027	4632·4 (4430·3 to 4845·3)	3934·7 (3728·8 to 4147·3)	534·9 (522·8 to 546·9)	162·8 (152·3 to 173·5)	0·0 (0·0 to 0·0)	4939·5 (4724·0 to 5166·5)	4195·6 (3976·0 to 4422·3)	570·3 (557·5 to 583·1)	173·6 (162·4 to 185·0)	0·0 (0·0 to 0·0)
Japan	2028	4708·0 (4492·9 to 4943·6)	4002·9 (3783·5 to 4231·8)	537·7 (525·5 to 550·0)	167·4 (155·4 to 179·7)	0·0 (0·0 to 0·0)	5020·1 (4790·8 to 5271·4)	4268·3 (4034·3 to 4512·3)	573·3 (560·3 to 586·4)	178·5 (165·7 to 191·6)	0·0 (0·0 to 0·0)
Japan	2029	4784·6 (4546·7 to 5036·0)	4071·8 (3830·6 to 4320·0)	540·6 (528·3 to 552·8)	172·2 (158·4 to 186·1)	0·0 (0·0 to 0·0)	5101·7 (4848·1 to 5369·9)	4341·8 (4084·6 to 4606·4)	576·4 (563·3 to 589·4)	183·6 (168·9 to 198·4)	0·0 (0·0 to 0·0)
Japan	2030	4861·7 (4595·6 to 5134·0)	4141·4 (3880·4 to 4403·6)	543·3 (531·0 to 555·5)	177·0 (161·3 to 192·7)	0·0 (0·0 to 0·0)	5184·0 (4900·3 to 5474·3)	4415·9 (4137·7 to 4695·5)	579·3 (566·2 to 592·3)	188·8 (172·0 to 205·5)	0·0 (0·0 to 0·0)
Japan	2031	4938·0 (4648·2 to 5230·8)	4210·6 (3924·1 to 4495·6)	545·5 (533·4 to 557·9)	181·9 (164·5 to 199·5)	0·0 (0·0 to 0·0)	5265·4 (4956·3 to 5577·6)	4489·7 (4184·3 to 4793·6)	581·7 (568·8 to 594·8)	194·0 (175·4 to 212·8)	0·0 (0·0 to 0·0)
Japan	2032	5013·1 (4707·2 to 5321·0)	4278·8 (3970·0 to 4588·0)	547·4 (535·2 to 559·8)	186·9 (167·3 to 206·5)	0·0 (0·0 to 0·0)	5345·4 (5019·3 to 5673·7)	4562·5 (4233·2 to 4892·1)	583·7 (570·7 to 596·9)	199·2 (178·4 to 220·1)	0·0 (0·0 to 0·0)
Japan	2033	5086·0 (4757·5 to 5413·7)	4345·6 (4021·1 to 4678·6)	548·6 (536·3 to 561·1)	191·8 (170·1 to 213·4)	0·0 (0·0 to 0·0)	5423·2 (5072·9 to 5772·6)	4633·7 (4287·6 to 4988·8)	585·0 (571·8 to 598·3)	204·5 (181·4 to 227·6)	0·0 (0·0 to 0·0)

Japan	2034	5157·2 (4803·9 to 5517·5)	4411·2 (4060·5 to 4771·3)	549·2 (536·7 to 561·7)	196·7 (173·3 to 220·7)	0·0 (0·0 to 0·0)	5499·1 (5122·3 to 5883·3)	4703·7 (4329·7 to 5087·6)	585·6 (572·3 to 598·9)	209·8 (184·8 to 235·3)	0·0 (0·0 to 0·0)
Japan	2035	5227·2 (4862·8 to 5606·9)	4475·9 (4109·9 to 4856·3)	549·5 (536·9 to 562·0)	201·8 (176·8 to 227·9)	0·0 (0·0 to 0·0)	5573·7 (5185·2 to 5978·6)	4772·7 (4382·3 to 5178·2)	585·9 (572·5 to 599·3)	215·1 (188·6 to 243·0)	0·0 (0·0 to 0·0)
Japan	2036	5294·7 (4908·8 to 5687·6)	4538·3 (4152·0 to 4938·6)	549·5 (536·9 to 562·3)	206·8 (179·6 to 235·7)	0·0 (0·0 to 0·0)	5645·7 (5234·2 to 6064·7)	4839·2 (4427·2 to 5266·0)	586·0 (572·5 to 599·6)	220·5 (191·5 to 251·3)	0·0 (0·0 to 0·0)
Japan	2037	5358·1 (4958·7 to 5765·0)	4596·8 (4196·3 to 5006·6)	549·4 (536·7 to 562·3)	211·9 (182·5 to 243·4)	0·0 (0·0 to 0·0)	5713·3 (5287·5 to 6147·1)	4901·5 (4474·5 to 5338·5)	585·8 (572·3 to 599·6)	226·0 (194·6 to 259·6)	0·0 (0·0 to 0·0)
Japan	2038	5419·3 (5000·6 to 5851·0)	4653·3 (4230·8 to 5082·0)	549·0 (536·2 to 562·2)	217·0 (185·2 to 251·2)	0·0 (0·0 to 0·0)	5778·6 (5332·1 to 6238·8)	4961·8 (4511·3 to 5418·9)	585·4 (571·7 to 599·5)	231·4 (197·5 to 267·9)	0·0 (0·0 to 0·0)
Japan	2039	5479·2 (5050·9 to 5920·3)	4708·7 (4269·3 to 5149·1)	548·3 (535·3 to 561·8)	222·2 (188·2 to 259·2)	0·0 (0·0 to 0·0)	5842·4 (5385·7 to 6312·7)	5020·8 (4552·3 to 5490·5)	584·7 (570·8 to 599·1)	237·0 (200·6 to 276·4)	0·0 (0·0 to 0·0)
Japan	2040	5535·9 (5088·9 to 5993·7)	4760·9 (4309·4 to 5220·5)	547·5 (534·3 to 561·2)	227·5 (190·9 to 267·3)	0·0 (0·0 to 0·0)	5902·9 (5426·3 to 6391·1)	5076·6 (4595·1 to 5566·6)	583·8 (569·7 to 598·4)	242·6 (203·6 to 285·0)	0·0 (0·0 to 0·0)
Japan	2041	5594·6 (5127·1 to 6072·5)	4814·5 (4339·5 to 5290·7)	547·2 (533·9 to 561·1)	233·0 (193·8 to 276·7)	0·0 (0·0 to 0·0)	5965·5 (5467·0 to 6475·0)	5133·6 (4627·1 to 5641·5)	583·5 (569·3 to 598·3)	248·4 (206·6 to 295·0)	0·0 (0·0 to 0·0)
Japan	2042	5652·8 (5170·6 to 6145·2)	4866·7 (4374·0 to 5358·2)	547·4 (534·0 to 561·6)	238·7 (196·8 to 286·0)	0·0 (0·0 to 0·0)	6027·6 (5513·4 to 6552·6)	5189·3 (4664·0 to 5713·4)	583·7 (569·4 to 598·8)	254·5 (209·9 to 304·9)	0·0 (0·0 to 0·0)
Japan	2043	5711·6 (5217·7 to 6218·2)	4918·7 (4418·5 to 5419·7)	548·2 (534·7 to 562·6)	244·7 (199·9 to 295·1)	0·0 (0·0 to 0·0)	6090·3 (5563·6 to 6630·4)	5244·8 (4711·4 to 5779·0)	584·6 (570·2 to 599·9)	260·9 (213·1 to 314·6)	0·0 (0·0 to 0·0)
Japan	2044	5772·4 (5268·8 to 6292·6)	4971·8 (4456·1 to 5490·2)	549·6 (535·9 to 564·2)	251·0 (203·4 to 304·2)	0·0 (0·0 to 0·0)	6155·1 (5618·1 to 6709·8)	5301·4 (4751·5 to 5854·2)	586·0 (571·5 to 601·6)	267·7 (216·9 to 324·4)	0·0 (0·0 to 0·0)
Japan	2045	5833·2 (5321·6 to 6373·7)	5024·3 (4497·6 to 5549·0)	551·3 (537·4 to 566·1)	257·6 (207·2 to 314·0)	0·0 (0·0 to 0·0)	6219·9 (5674·3 to 6796·3)	5357·4 (4795·8 to 5916·9)	587·9 (573·0 to 603·6)	274·7 (221·0 to 334·8)	0·0 (0·0 to 0·0)
Japan	2046	5897·4 (5371·1 to 6440·2)	5079·4 (4537·6 to 5614·3)	553·5 (539·5 to 568·5)	264·5 (211·0 to 324·5)	0·0 (0·0 to 0·0)	6288·4 (5727·2 to 6867·2)	5416·1 (4838·4 to 5986·5)	590·2 (575·2 to 606·2)	282·1 (225·0 to 346·0)	0·0 (0·0 to 0·0)
Japan	2047	5960·3 (5422·0 to 6511·7)	5132·9 (4580·7 to 5680·4)	555·7 (541·4 to 570·9)	271·6 (214·3 to 336·1)	0·0 (0·0 to 0·0)	6355·4 (5781·4 to 6943·4)	5473·2 (4884·3 to 6056·9)	592·6 (577·3 to 608·7)	289·6 (228·5 to 358·4)	0·0 (0·0 to 0·0)

Japan	2048	6021·9 (5472·3 to 6590·3)	5185·1 (4631·1 to 5748·3)	557·9 (543·2 to 573·4)	278·9 (218·0 to 347·8)	0·0 (0·0 to 0·0)	6421·2 (5835·0 to 7027·2)	5528·8 (4938·1 to 6129·4)	594·9 (579·2 to 611·4)	297·4 (232·5 to 370·9)	0·0 (0·0 to 0·0)
Japan	2049	6084·6 (5529·7 to 6662·9)	5237·8 (4676·1 to 5807·5)	560·3 (545·3 to 576·0)	286·5 (221·4 to 359·5)	0·0 (0·0 to 0·0)	6488·0 (5896·3 to 7104·6)	5585·1 (4986·1 to 6192·5)	597·5 (581·4 to 614·2)	305·5 (236·1 to 383·4)	0·0 (0·0 to 0·0)
Japan	2050	6150·1 (5583·0 to 6733·9)	5292·4 (4716·5 to 5873·3)	563·2 (548·0 to 579·1)	294·5 (225·4 to 371·8)	0·0 (0·0 to 0·0)	6557·8 (5953·1 to 7180·3)	5643·2 (5029·2 to 6262·6)	600·6 (584·4 to 617·5)	314·0 (240·4 to 396·5)	0·0 (0·0 to 0·0)
Kazakhstan*	1995	170·3 (152·4 to 191·6)	90·9 (78·3 to 105·5)	76·9 (65·1 to 90·4)	2·2 (1·5 to 3·1)	0·4 (0·4 to 0·4)	515·7 (461·4 to 580·1)	275·1 (237·0 to 319·5)	232·7 (197·2 to 273·6)	6·7 (4·6 to 9·4)	1·2 (1·2 to 1·2)
Kazakhstan*	1996	170·1 (154·1 to 188·0)	90·7 (78·6 to 104·7)	76·6 (65·9 to 88·1)	2·2 (1·5 to 3·1)	0·6 (0·6 to 0·6)	515·0 (466·7 to 569·3)	274·7 (238·1 to 317·1)	232·1 (199·6 to 266·6)	6·6 (4·6 to 9·3)	1·7 (1·7 to 1·7)
Kazakhstan*	1997	169·4 (154·3 to 185·1)	91·0 (79·7 to 104·7)	75·7 (66·0 to 86·0)	2·1 (1·4 to 3·0)	0·5 (0·5 to 0·5)	513·0 (467·3 to 560·6)	275·7 (241·2 to 317·0)	229·3 (200·0 to 260·4)	6·5 (4·4 to 9·1)	1·5 (1·5 to 1·5)
Kazakhstan*	1998	161·5 (148·3 to 175·8)	85·7 (74·9 to 97·2)	72·9 (63·7 to 82·2)	2·0 (1·3 to 2·7)	1·0 (1·0 to 1·0)	488·8 (448·9 to 532·2)	259·4 (226·8 to 294·4)	220·6 (193·0 to 249·0)	5·9 (4·1 to 8·2)	2·9 (2·9 to 2·9)
Kazakhstan*	1999	154·5 (142·6 to 167·7)	81·1 (71·4 to 91·4)	70·2 (62·2 to 79·1)	1·6 (1·1 to 2·3)	1·6 (1·6 to 1·6)	467·7 (431·6 to 507·8)	245·6 (216·2 to 276·8)	212·4 (188·3 to 239·4)	5·0 (3·4 to 7·0)	4·7 (4·7 to 4·7)
Kazakhstan*	2000	153·7 (141·3 to 167·4)	81·9 (71·6 to 93·0)	69·2 (61·6 to 77·4)	1·1 (0·8 to 1·6)	1·5 (1·5 to 1·5)	465·4 (427·7 to 506·8)	248·0 (216·9 to 281·7)	209·5 (186·5 to 234·4)	3·4 (2·3 to 4·7)	4·7 (4·7 to 4·7)
Kazakhstan*	2001	159·9 (147·2 to 174·3)	88·1 (77·0 to 99·4)	69·2 (62·2 to 77·5)	1·0 (0·7 to 1·4)	1·6 (1·6 to 1·6)	484·2 (445·8 to 527·8)	266·6 (233·1 to 301·0)	209·6 (188·3 to 234·6)	3·1 (2·1 to 4·4)	4·9 (4·9 to 4·9)
Kazakhstan*	2002	164·0 (150·9 to 177·6)	90·7 (80·4 to 101·3)	71·6 (63·9 to 80·0)	1·0 (0·7 to 1·4)	0·8 (0·8 to 0·8)	496·6 (457·0 to 537·8)	274·5 (243·4 to 306·7)	216·7 (193·4 to 242·3)	3·1 (2·2 to 4·4)	2·3 (2·3 to 2·3)
Kazakhstan*	2003	182·6 (168·3 to 197·6)	104·2 (92·7 to 116·3)	75·9 (67·5 to 85·1)	1·1 (0·8 to 1·6)	1·4 (1·4 to 1·4)	552·9 (509·7 to 598·2)	315·5 (280·6 to 352·1)	229·7 (204·5 to 257·7)	3·4 (2·4 to 4·8)	4·3 (4·3 to 4·3)
Kazakhstan*	2004	197·2 (182·1 to 214·5)	118·0 (106·2 to 130·9)	77·0 (68·2 to 87·7)	1·2 (0·8 to 1·7)	1·1 (1·1 to 1·1)	597·2 (551·3 to 649·5)	357·2 (321·6 to 396·3)	233·0 (206·5 to 265·4)	3·7 (2·6 to 5·2)	3·2 (3·2 to 3·2)
Kazakhstan*	2005	209·5 (193·3 to 227·6)	133·5 (121·0 to 147·3)	73·9 (64·6 to 84·7)	1·3 (0·9 to 1·9)	0·8 (0·8 to 0·8)	634·4 (585·2 to 689·1)	404·1 (366·5 to 445·9)	223·8 (195·7 to 256·6)	4·1 (2·8 to 5·8)	2·5 (2·5 to 2·5)

Kazakhstan*	2006	202·3 (186·5 to 218·1)	133·2 (120·5 to 146·5)	66·3 (57·7 to 76·1)	1·4 (1·0 to 2·1)	1·3 (1·3 to 1·3)	612·4 (564·7 to 660·5)	403·3 (364·7 to 443·6)	200·9 (174·8 to 230·5)	4·4 (3·0 to 6·2)	3·8 (3·8 to 3·8)
Kazakhstan*	2007	215·3 (198·8 to 231·9)	149·7 (136·1 to 164·7)	63·0 (54·6 to 72·4)	1·7 (1·2 to 2·4)	0·8 (0·8 to 0·8)	651·8 (602·0 to 702·1)	453·4 (412·2 to 498·7)	190·7 (165·3 to 219·1)	5·2 (3·6 to 7·3)	2·4 (2·4 to 2·4)
Kazakhstan*	2008	233·2 (215·9 to 252·2)	169·1 (154·3 to 186·0)	60·4 (52·6 to 69·0)	2·3 (1·5 to 3·2)	1·5 (1·5 to 1·5)	706·2 (653·6 to 763·5)	512·0 (467·2 to 563·1)	182·7 (159·2 to 209·0)	6·8 (4·6 to 9·5)	4·6 (4·6 to 4·6)
Kazakhstan*	2009	218·4 (202·9 to 234·7)	156·0 (142·4 to 170·6)	56·2 (49·1 to 64·1)	3·0 (2·0 to 4·2)	3·1 (3·1 to 3·1)	661·2 (614·5 to 710·7)	472·3 (431·2 to 516·4)	170·3 (148·5 to 194·1)	9·2 (6·2 to 12·6)	9·5 (9·5 to 9·5)
Kazakhstan*	2010	216·5 (200·9 to 232·8)	151·8 (137·4 to 167·4)	55·6 (48·9 to 62·8)	5·6 (3·8 to 7·8)	3·4 (3·4 to 3·4)	655·6 (608·2 to 704·8)	459·6 (416·1 to 506·7)	168·5 (148·2 to 190·1)	17·1 (11·6 to 23·5)	10·4 (10·4 to 10·4)
Kazakhstan*	2011	219·8 (204·0 to 235·6)	153·5 (138·7 to 168·7)	56·5 (50·4 to 63·3)	7·4 (5·1 to 10·2)	2·4 (2·4 to 2·4)	665·5 (617·7 to 713·4)	464·7 (420·1 to 510·8)	171·2 (152·5 to 191·5)	22·4 (15·4 to 30·9)	7·2 (7·2 to 7·2)
Kazakhstan*	2012	235·2 (219·0 to 251·9)	161·9 (146·8 to 177·7)	61·7 (55·5 to 68·3)	9·0 (6·2 to 12·5)	2·6 (2·6 to 2·6)	712·2 (663·2 to 762·7)	490·2 (444·5 to 537·9)	186·8 (168·1 to 206·8)	27·3 (18·8 to 37·7)	8·0 (8·0 to 8·0)
Kazakhstan*	2013	233·5 (217·4 to 249·9)	158·3 (143·7 to 173·3)	63·7 (57·3 to 70·4)	9·9 (6·9 to 13·5)	1·7 (1·7 to 1·7)	707·0 (658·2 to 756·6)	479·2 (435·0 to 524·7)	192·7 (173·6 to 213·3)	30·0 (20·9 to 40·9)	5·1 (5·1 to 5·1)
Kazakhstan*	2014	254·1 (237·1 to 272·0)	170·5 (155·6 to 187·4)	71·3 (64·7 to 78·0)	11·3 (8·0 to 15·5)	1·1 (1·1 to 1·1)	769·3 (717·9 to 823·5)	516·1 (471·0 to 567·5)	215·7 (195·8 to 236·1)	34·1 (24·1 to 46·8)	3·3 (3·3 to 3·3)
Kazakhstan*	2015	268·4 (249·9 to 286·1)	172·6 (157·0 to 189·0)	82·4 (75·3 to 89·9)	12·3 (8·7 to 16·8)	1·1 (1·1 to 1·1)	812·6 (756·7 to 866·1)	522·7 (475·3 to 572·3)	249·6 (228·1 to 272·2)	37·1 (26·5 to 50·9)	3·2 (3·2 to 3·2)
Kazakhstan*	2016	269·5 (251·2 to 287·5)	164·7 (150·0 to 180·2)	89·8 (82·7 to 97·9)	12·7 (8·8 to 17·4)	2·3 (2·3 to 2·3)	815·9 (760·7 to 870·5)	498·7 (454·1 to 545·6)	272·0 (250·4 to 296·3)	38·4 (26·8 to 52·6)	6·9 (6·9 to 6·9)
Kazakhstan*	2017	284·6 (264·5 to 304·8)	175·9 (160·5 to 193·1)	93·6 (85·1 to 102·4)	13·4 (9·4 to 18·2)	1·7 (1·7 to 1·7)	861·7 (800·9 to 922·9)	532·6 (485·9 to 584·7)	283·3 (257·7 to 310·1)	40·5 (28·5 to 55·1)	5·3 (5·3 to 5·3)
Kazakhstan*	2018	260·9 (240·9 to 283·5)	157·5 (139·9 to 175·5)	89·1 (79·7 to 99·6)	13·5 (9·4 to 18·4)	0·8 (0·8 to 0·8)	789·8 (729·4 to 858·3)	476·7 (423·4 to 531·2)	269·8 (241·4 to 301·6)	40·7 (28·5 to 55·8)	2·6 (2·6 to 2·6)
Kazakhstan*	2019	282·2 (258·2 to 308·4)	176·9 (155·5 to 199·1)	90·4 (80·9 to 101·1)	14·1 (9·9 to 19·3)	0·8 (0·8 to 0·9)	854·5 (781·7 to 933·7)	535·6 (470·7 to 602·9)	273·8 (245·0 to 306·2)	42·7 (29·9 to 58·3)	2·6 (2·5 to 2·6)

Kazakhstan*	2020	298·5 (271·4 to 328·2)	196·7 (172·4 to 223·7)	86·9 (77·7 to 97·2)	13·9 (9·7 to 19·0)	1·1 (1·1 to 1·1)	903·9 (821·6 to 993·8)	595·6 (521·9 to 677·3)	263·1 (235·1 to 294·2)	42·0 (29·3 to 57·6)	3·3 (3·2 to 3·3)
Kazakhstan*	2021	292·8 (266·2 to 321·9)	192·9 (169·0 to 219·4)	85·0 (76·0 to 95·1)	13·9 (9·7 to 19·1)	1·0 (1·0 to 1·0)	886·6 (805·9 to 974·7)	584·0 (511·8 to 664·1)	257·4 (230·1 to 288·0)	42·1 (29·4 to 57·8)	3·0 (3·0 to 3·1)
Kazakhstan*	2022	274·0 (247·9 to 302·9)	174·1 (150·4 to 199·5)	85·0 (76·1 to 95·0)	14·2 (9·9 to 19·6)	0·8 (0·8 to 0·9)	829·7 (750·5 to 917·0)	527·0 (455·3 to 603·9)	257·2 (230·5 to 287·8)	43·0 (30·0 to 59·2)	2·4 (2·3 to 2·6)
Kazakhstan*	2023	273·8 (247·8 to 302·4)	173·4 (149·7 to 199·0)	85·0 (76·1 to 95·2)	14·5 (10·1 to 19·9)	0·9 (0·8 to 0·9)	828·9 (750·3 to 915·5)	524·9 (453·2 to 602·5)	257·4 (230·5 to 288·2)	44·0 (30·7 to 60·4)	2·6 (2·4 to 2·8)
Kazakhstan*	2024	274·7 (248·5 to 304·9)	173·7 (149·7 to 200·5)	85·2 (76·2 to 95·3)	14·9 (10·4 to 20·5)	0·9 (0·8 to 1·0)	831·6 (752·3 to 923·0)	526·0 (453·2 to 606·9)	257·9 (230·9 to 288·4)	45·0 (31·4 to 62·1)	2·7 (2·6 to 2·9)
Kazakhstan*	2025	277·6 (249·3 to 308·9)	176·1 (150·6 to 203·4)	85·4 (76·5 to 95·5)	15·2 (10·6 to 21·0)	1·0 (0·9 to 1·0)	840·6 (754·9 to 935·2)	533·1 (455·9 to 615·8)	258·6 (231·5 to 289·2)	46·1 (32·0 to 63·6)	2·9 (2·7 to 3·1)
Kazakhstan*	2026	281·1 (251·3 to 313·1)	178·8 (151·3 to 207·1)	85·8 (76·8 to 95·9)	15·6 (10·8 to 21·5)	1·0 (0·9 to 1·1)	851·2 (760·9 to 947·9)	541·3 (458·1 to 626·9)	259·7 (232·6 to 290·4)	47·2 (32·7 to 65·2)	3·0 (2·8 to 3·3)
Kazakhstan*	2027	284·4 (253·1 to 318·6)	181·2 (152·9 to 211·5)	86·1 (77·1 to 96·4)	16·0 (11·0 to 22·1)	1·0 (1·0 to 1·1)	861·1 (766·3 to 964·7)	548·7 (462·8 to 640·4)	260·8 (233·5 to 291·9)	48·4 (33·4 to 67·1)	3·1 (2·9 to 3·5)
Kazakhstan*	2028	288·4 (255·7 to 324·1)	184·1 (155·0 to 217·1)	86·7 (77·6 to 97·0)	16·5 (11·4 to 22·7)	1·1 (1·0 to 1·2)	873·1 (774·3 to 981·1)	557·4 (469·2 to 657·2)	262·6 (235·0 to 293·7)	49·8 (34·5 to 68·7)	3·2 (3·0 to 3·6)
Kazakhstan*	2029	292·9 (258·9 to 329·5)	187·4 (157·0 to 220·7)	87·4 (78·3 to 97·8)	17·0 (11·8 to 23·4)	1·1 (1·0 to 1·2)	886·9 (783·9 to 997·6)	567·5 (475·5 to 668·4)	264·8 (237·0 to 296·1)	51·3 (35·6 to 70·8)	3·3 (3·0 to 3·7)
Kazakhstan*	2030	298·1 (262·9 to 337·4)	191·2 (158·4 to 227·6)	88·3 (79·0 to 98·7)	17·5 (12·2 to 24·1)	1·1 (1·0 to 1·3)	902·5 (796·1 to 1021·4)	578·9 (479·6 to 689·0)	267·3 (239·3 to 298·9)	53·0 (36·8 to 72·9)	3·4 (3·1 to 3·9)
Kazakhstan*	2031	303·8 (267·0 to 346·2)	195·5 (160·8 to 233·4)	89·1 (79·8 to 99·6)	18·0 (12·6 to 24·8)	1·1 (1·0 to 1·3)	919·8 (808·4 to 1048·2)	591·8 (486·8 to 706·6)	269·9 (241·6 to 301·6)	54·6 (38·0 to 75·2)	3·4 (3·1 to 4·0)
Kazakhstan*	2032	309·9 (271·1 to 353·8)	200·0 (163·1 to 240·5)	90·0 (80·6 to 100·7)	18·6 (13·0 to 25·7)	1·2 (1·0 to 1·4)	938·2 (820·7 to 1071·4)	605·6 (494·0 to 728·1)	272·6 (244·0 to 304·9)	56·4 (39·3 to 77·8)	3·5 (3·1 to 4·1)
Kazakhstan*	2033	316·1 (276·3 to 362·9)	204·5 (166·0 to 244·7)	91·1 (81·5 to 102·0)	19·3 (13·4 to 26·7)	1·2 (1·0 to 1·4)	956·9 (836·6 to 1098·8)	619·2 (502·5 to 740·8)	275·8 (246·9 to 308·8)	58·4 (40·7 to 80·8)	3·5 (3·1 to 4·2)

Kazakhstan*	2034	322·9 (281·2 to 373·9)	209·4 (167·8 to 254·9)	92·3 (82·6 to 103·3)	20·0 (14·0 to 27·7)	1·2 (1·0 to 1·4)	977·5 (851·5 to 1132·0)	634·0 (508·2 to 771·6)	279·5 (250·1 to 312·6)	60·5 (42·3 to 83·8)	3·6 (3·2 to 4·3)
Kazakhstan*	2035	329·5 (285·3 to 378·8)	214·1 (172·4 to 261·7)	93·4 (83·6 to 104·4)	20·7 (14·4 to 28·7)	1·2 (1·0 to 1·4)	997·5 (863·7 to 1146·9)	648·4 (522·0 to 792·3)	282·9 (253·1 to 316·2)	62·6 (43·5 to 86·9)	3·6 (3·2 to 4·4)
Kazakhstan*	2036	335·2 (289·4 to 386·0)	218·2 (173·7 to 267·7)	94·5 (84·5 to 105·6)	21·4 (14·9 to 29·6)	1·2 (1·0 to 1·5)	1015·0 (876·3 to 1168·6)	660·6 (525·9 to 810·6)	286·0 (255·9 to 319·7)	64·8 (45·1 to 89·7)	3·6 (3·2 to 4·5)
Kazakhstan*	2037	340·6 (290·8 to 396·0)	222·0 (174·5 to 275·1)	95·3 (85·3 to 106·5)	22·1 (15·4 to 30·6)	1·2 (1·1 to 1·5)	1031·2 (880·3 to 1198·9)	672·1 (528·4 to 832·9)	288·5 (258·1 to 322·3)	66·9 (46·5 to 92·7)	3·7 (3·2 to 4·6)
Kazakhstan*	2038	345·5 (293·4 to 401·4)	225·4 (175·8 to 280·0)	96·0 (85·9 to 107·3)	22·8 (15·8 to 31·8)	1·3 (1·1 to 1·6)	1046·0 (888·3 to 1215·2)	682·5 (532·1 to 847·7)	290·8 (260·1 to 324·8)	68·9 (47·8 to 96·2)	3·8 (3·3 to 4·8)
Kazakhstan*	2039	350·6 (296·9 to 408·0)	229·0 (177·7 to 283·1)	96·8 (86·6 to 108·1)	23·5 (16·2 to 33·1)	1·3 (1·1 to 1·6)	1061·5 (898·9 to 1235·2)	693·4 (538·2 to 857·1)	293·1 (262·2 to 327·4)	71·1 (49·2 to 100·1)	3·9 (3·4 to 5·0)
Kazakhstan*	2040	356·1 (299·8 to 415·5)	232·9 (180·2 to 288·7)	97·6 (87·3 to 109·0)	24·2 (16·7 to 34·3)	1·3 (1·1 to 1·7)	1078·1 (907·6 to 1258·1)	705·3 (545·7 to 874·0)	295·5 (264·3 to 330·0)	73·3 (50·6 to 103·8)	4·0 (3·4 to 5·1)
Kazakhstan*	2041	361·0 (302·1 to 422·1)	236·5 (181·5 to 296·8)	98·2 (87·8 to 109·7)	24·9 (17·2 to 35·5)	1·4 (1·2 to 1·8)	1093·1 (914·7 to 1277·9)	716·1 (549·6 to 898·8)	297·4 (265·9 to 332·0)	75·5 (52·1 to 107·4)	4·1 (3·5 to 5·4)
Kazakhstan*	2042	366·3 (306·9 to 429·8)	240·3 (184·3 to 302·6)	98·9 (88·4 to 110·4)	25·7 (17·6 to 36·6)	1·4 (1·2 to 1·9)	1109·0 (929·1 to 1301·2)	727·5 (558·0 to 916·3)	299·4 (267·5 to 334·2)	77·8 (53·4 to 110·9)	4·2 (3·6 to 5·6)
Kazakhstan*	2043	371·1 (310·1 to 437·2)	243·7 (188·1 to 307·6)	99·5 (88·8 to 111·0)	26·4 (18·1 to 37·7)	1·4 (1·2 to 1·9)	1123·6 (938·8 to 1323·8)	737·9 (569·6 to 931·3)	301·2 (268·9 to 336·1)	80·1 (54·7 to 114·3)	4·4 (3·7 to 5·9)
Kazakhstan*	2044	375·6 (312·8 to 443·0)	246·9 (189·0 to 311·4)	100·0 (89·2 to 111·6)	27·2 (18·6 to 39·0)	1·5 (1·3 to 2·0)	1137·2 (947·1 to 1341·1)	747·5 (572·1 to 942·9)	302·8 (270·2 to 337·8)	82·4 (56·2 to 118·1)	4·5 (3·8 to 6·2)
Kazakhstan*	2045	379·7 (318·0 to 450·4)	249·7 (188·7 to 315·9)	100·5 (89·6 to 112·1)	28·0 (19·1 to 40·3)	1·6 (1·3 to 2·1)	1149·7 (962·9 to 1363·6)	756·1 (571·3 to 956·5)	304·2 (271·3 to 339·4)	84·7 (57·7 to 122·0)	4·7 (3·9 to 6·5)
Kazakhstan*	2046	384·3 (318·2 to 456·4)	253·0 (190·5 to 323·4)	101·0 (90·0 to 112·6)	28·8 (19·6 to 41·6)	1·6 (1·3 to 2·3)	1163·6 (963·3 to 1382·0)	765·9 (576·7 to 979·2)	305·7 (272·5 to 341·0)	87·1 (59·3 to 126·1)	4·9 (4·0 to 6·8)
Kazakhstan*	2047	388·6 (318·7 to 464·5)	256·0 (190·3 to 329·6)	101·4 (90·3 to 113·1)	29·6 (20·1 to 43·0)	1·7 (1·4 to 2·4)	1176·5 (965·0 to 1406·4)	775·0 (576·1 to 998·0)	307·0 (273·5 to 342·4)	89·5 (60·8 to 130·1)	5·1 (4·1 to 7·2)

Kazakhstan*	2048	392·6 (325·2 to 469·4)	258·8 (193·3 to 331·1)	101·7 (90·6 to 113·5)	30·3 (20·5 to 44·3)	1·8 (1·4 to 2·6)	1188·6 (984·5 to 1421·2)	783·5 (585·2 to 1002·5)	308·0 (274·3 to 343·5)	91·8 (62·1 to 134·2)	5·3 (4·3 to 7·8)
Kazakhstan*	2049	397·6 (327·3 to 475·4)	262·5 (195·2 to 340·9)	102·1 (90·9 to 113·9)	31·2 (20·9 to 45·8)	1·8 (1·4 to 2·7)	1203·9 (990·8 to 1439·5)	794·8 (591·0 to 1032·2)	309·3 (275·2 to 344·8)	94·3 (63·3 to 138·6)	5·5 (4·3 to 8·1)
Kazakhstan*	2050	402·6 (331·1 to 483·8)	266·2 (199·8 to 348·1)	102·5 (91·2 to 114·3)	32·0 (21·3 to 47·2)	1·9 (0·0 to 2·9)	1219·0 (1002·4 to 1464·9)	806·0 (604·9 to 1054·0)	310·4 (276·2 to 346·1)	96·8 (64·6 to 143·0)	5·7 (0·0 to 8·7)
Kenya*	1995	64·0 (56·0 to 72·4)	22·9 (19·0 to 27·4)	29·9 (23·8 to 36·8)	7·5 (5·2 to 10·4)	3·7 (3·7 to 3·7)	154·0 (134·8 to 174·3)	55·2 (45·7 to 66·0)	71·9 (57·4 to 88·5)	18·1 (12·5 to 25·0)	8·8 (8·8 to 8·8)
Kenya*	1996	64·0 (56·6 to 72·2)	21·9 (18·2 to 26·3)	29·6 (23·9 to 36·6)	7·4 (5·1 to 10·5)	5·1 (5·1 to 5·1)	154·1 (136·2 to 173·8)	52·8 (43·9 to 63·3)	71·3 (57·4 to 88·1)	17·8 (12·2 to 25·2)	12·2 (12·2 to 12·2)
Kenya*	1997	62·5 (55·0 to 70·3)	21·9 (18·2 to 26·2)	29·8 (24·2 to 36·2)	7·5 (5·2 to 10·4)	3·3 (3·3 to 3·3)	150·3 (132·4 to 169·1)	52·7 (43·8 to 62·9)	71·6 (58·3 to 87·0)	18·0 (12·4 to 25·1)	8·0 (8·0 to 8·0)
Kenya*	1998	63·7 (56·7 to 71·5)	21·7 (18·1 to 25·8)	29·7 (24·6 to 35·9)	7·6 (5·2 to 10·4)	4·7 (4·7 to 4·7)	153·2 (136·5 to 171·9)	52·3 (43·4 to 62·0)	71·4 (59·1 to 86·3)	18·2 (12·5 to 25·1)	11·3 (11·3 to 11·3)
Kenya*	1999	60·3 (53·7 to 67·4)	20·3 (16·8 to 24·1)	29·1 (24·2 to 34·6)	7·4 (5·2 to 10·4)	3·5 (3·5 to 3·5)	145·1 (129·3 to 162·2)	48·9 (40·5 to 57·9)	69·9 (58·3 to 83·3)	17·9 (12·5 to 25·0)	8·4 (8·4 to 8·4)
Kenya*	2000	59·7 (53·5 to 66·2)	19·0 (15·7 to 22·8)	28·5 (24·1 to 33·5)	7·3 (5·1 to 10·4)	4·8 (4·8 to 4·8)	143·6 (128·6 to 159·4)	45·8 (37·8 to 54·8)	68·6 (57·9 to 80·5)	17·6 (12·4 to 25·0)	11·6 (11·6 to 11·6)
Kenya*	2001	60·3 (54·7 to 66·7)	19·3 (15·9 to 23·1)	28·8 (24·8 to 33·2)	7·4 (5·3 to 10·4)	4·8 (4·8 to 4·8)	145·2 (131·5 to 160·6)	46·4 (38·3 to 55·6)	69·3 (59·6 to 80·0)	17·8 (12·7 to 25·1)	11·6 (11·6 to 11·6)
Kenya*	2002	60·3 (54·6 to 67·0)	19·7 (16·4 to 23·7)	29·0 (25·1 to 33·4)	7·4 (5·2 to 10·4)	4·3 (4·3 to 4·3)	145·2 (131·4 to 161·1)	47·4 (39·4 to 57·0)	69·8 (60·3 to 80·5)	17·7 (12·5 to 24·9)	10·3 (10·3 to 10·3)
Kenya*	2003	62·3 (56·5 to 68·5)	19·5 (16·3 to 23·3)	29·3 (25·0 to 33·9)	7·3 (5·1 to 10·0)	6·2 (6·2 to 6·2)	149·8 (135·9 to 164·9)	47·0 (39·2 to 56·0)	70·5 (60·1 to 81·6)	17·5 (12·3 to 24·1)	14·9 (14·9 to 14·9)
Kenya*	2004	63·7 (57·9 to 70·3)	19·2 (16·0 to 22·5)	29·5 (25·3 to 34·4)	7·1 (5·0 to 10·0)	7·9 (7·9 to 7·9)	153·3 (139·4 to 169·2)	46·1 (38·5 to 54·2)	71·0 (60·9 to 82·8)	17·1 (12·1 to 24·0)	19·0 (19·0 to 19·0)
Kenya*	2005	66·1 (60·2 to 73·0)	20·5 (17·3 to 24·0)	30·6 (26·0 to 35·9)	7·4 (5·1 to 10·4)	7·7 (7·7 to 7·7)	159·0 (144·9 to 175·7)	49·4 (41·7 to 57·8)	73·5 (62·6 to 86·4)	17·7 (12·4 to 25·0)	18·4 (18·4 to 18·4)

Kenya*	2006	71·1 (65·0 to 78·0)	21·4 (18·0 to 24·7)	30·6 (25·6 to 36·4)	7·6 (5·3 to 10·7)	11·5 (11·5 to 11·5)	171·1 (156·5 to 187·7)	51·4 (43·3 to 59·5)	73·7 (61·7 to 87·6)	18·3 (12·8 to 25·8)	27·7 (27·7 to 27·7)
Kenya*	2007	72·8 (66·9 to 79·5)	22·9 (19·9 to 26·1)	30·6 (25·7 to 36·1)	8·6 (6·0 to 12·3)	10·8 (10·8 to 10·8)	175·2 (160·9 to 191·4)	55·2 (48·0 to 62·8)	73·6 (61·9 to 87·0)	20·6 (14·5 to 29·5)	25·9 (25·9 to 25·9)
Kenya*	2008	76·4 (70·6 to 82·7)	23·6 (20·9 to 26·2)	29·4 (25·0 to 34·2)	9·3 (6·6 to 13·2)	14·1 (14·1 to 14·1)	183·9 (169·9 to 198·9)	56·8 (50·4 to 63·1)	70·8 (60·3 to 82·4)	22·3 (15·9 to 31·8)	34·0 (34·0 to 34·0)
Kenya*	2009	78·0 (72·6 to 83·7)	24·5 (22·3 to 26·6)	28·5 (24·8 to 32·7)	9·9 (7·0 to 13·9)	15·0 (15·0 to 15·0)	187·6 (174·7 to 201·4)	58·9 (53·6 to 64·1)	68·7 (59·6 to 78·6)	23·9 (16·8 to 33·6)	36·2 (36·2 to 36·2)
Kenya*	2010	83·0 (77·8 to 88·3)	26·7 (24·9 to 28·4)	28·7 (25·0 to 32·4)	10·8 (7·5 to 14·9)	16·8 (16·8 to 16·8)	199·7 (187·1 to 212·6)	64·2 (60·0 to 68·4)	69·1 (60·3 to 77·9)	25·9 (17·9 to 35·9)	40·5 (40·5 to 40·5)
Kenya*	2011	84·2 (79·0 to 89·4)	27·5 (25·8 to 29·2)	28·4 (25·1 to 32·0)	10·8 (7·6 to 14·7)	17·5 (17·5 to 17·5)	202·6 (190·2 to 215·1)	66·1 (62·2 to 70·2)	68·4 (60·4 to 76·9)	26·0 (18·2 to 35·4)	42·1 (42·1 to 42·1)
Kenya*	2012	87·4 (82·3 to 92·6)	28·9 (27·3 to 30·7)	28·3 (25·1 to 31·7)	10·9 (7·7 to 14·9)	19·3 (19·3 to 19·3)	210·3 (198·1 to 222·8)	69·5 (65·6 to 73·8)	68·1 (60·3 to 76·3)	26·2 (18·6 to 35·8)	46·4 (46·4 to 46·4)
Kenya*	2013	92·4 (87·1 to 97·8)	31·3 (29·7 to 33·1)	28·1 (25·0 to 31·5)	11·4 (8·0 to 15·6)	21·5 (21·5 to 21·5)	222·2 (209·6 to 235·4)	75·4 (71·4 to 79·8)	67·7 (60·1 to 75·8)	27·4 (19·3 to 37·6)	51·7 (51·7 to 51·7)
Kenya*	2014	93·4 (87·8 to 99·2)	34·8 (33·0 to 36·7)	27·2 (24·2 to 30·4)	12·7 (8·9 to 17·3)	18·7 (18·7 to 18·7)	224·8 (211·3 to 238·8)	83·7 (79·4 to 88·3)	65·5 (58·1 to 73·3)	30·6 (21·4 to 41·7)	45·1 (45·1 to 45·1)
Kenya*	2015	93·3 (87·8 to 99·4)	37·5 (35·7 to 39·3)	25·7 (23·0 to 28·8)	13·9 (9·8 to 19·0)	16·2 (16·2 to 16·2)	224·4 (211·2 to 239·1)	90·2 (86·0 to 94·5)	61·8 (55·2 to 69·2)	33·5 (23·6 to 45·6)	39·0 (39·0 to 39·0)
Kenya*	2016	97·4 (92·0 to 103·4)	40·4 (38·6 to 42·3)	24·5 (21·9 to 27·3)	15·4 (10·8 to 21·0)	17·2 (17·2 to 17·2)	234·4 (221·3 to 248·9)	97·1 (92·8 to 101·7)	59·0 (52·7 to 65·7)	37·0 (26·1 to 50·6)	41·3 (41·3 to 41·3)
Kenya*	2017	99·9 (93·8 to 106·7)	40·8 (38·2 to 43·7)	23·4 (21·0 to 26·0)	16·4 (11·7 to 22·7)	19·4 (19·4 to 19·4)	240·4 (225·8 to 256·7)	98·1 (91·9 to 105·2)	56·2 (50·4 to 62·5)	39·5 (28·2 to 54·6)	46·6 (46·6 to 46·6)
Kenya*	2018	100·8 (93·1 to 109·5)	43·0 (38·9 to 47·8)	23·7 (21·0 to 26·9)	18·4 (13·1 to 25·3)	15·8 (15·8 to 15·8)	242·6 (224·0 to 263·6)	103·5 (93·6 to 115·0)	57·0 (50·6 to 64·7)	44·2 (31·5 to 60·9)	37·9 (37·9 to 37·9)
Kenya*	2019	103·1 (95·2 to 112·3)	44·3 (40·0 to 49·2)	24·4 (21·6 to 27·6)	19·3 (13·9 to 26·7)	15·2 (15·0 to 15·3)	248·2 (229·0 to 270·1)	106·6 (96·3 to 118·4)	58·6 (52·0 to 66·4)	46·5 (33·3 to 64·2)	36·5 (36·2 to 36·8)

Kenya*	2020	107·5 (99·7 to 116·4)	45·3 (41·2 to 50·2)	23·1 (20·5 to 26·2)	18·8 (13·5 to 26·0)	20·2 (19·8 to 20·7)	258·6 (239·8 to 280·0)	109·1 (99·0 to 120·7)	55·7 (49·4 to 63·2)	45·3 (32·5 to 62·6)	48·5 (47·8 to 49·9)
Kenya*	2021	101·0 (93·6 to 109·9)	40·6 (36·7 to 45·3)	22·4 (19·9 to 25·4)	18·7 (13·4 to 25·9)	19·3 (18·6 to 20·2)	243·1 (225·1 to 264·4)	97·8 (88·4 to 109·1)	53·9 (47·9 to 61·1)	44·9 (32·2 to 62·2)	46·5 (44·8 to 48·7)
Kenya*	2022	98·6 (90·7 to 107·8)	42·0 (37·8 to 46·7)	22·8 (20·3 to 25·8)	19·4 (13·9 to 26·8)	14·4 (13·4 to 15·4)	237·3 (218·3 to 259·5)	101·0 (90·9 to 112·4)	54·9 (48·8 to 62·2)	46·8 (33·5 to 64·4)	34·6 (32·3 to 37·0)
Kenya*	2023	100·7 (92·6 to 110·4)	43·1 (38·8 to 48·1)	23·3 (20·7 to 26·4)	20·2 (14·5 to 27·8)	14·1 (13·1 to 15·3)	242·3 (222·7 to 265·6)	103·7 (93·3 to 115·8)	56·0 (49·7 to 63·4)	48·7 (34·9 to 67·0)	33·9 (31·5 to 36·8)
Kenya*	2024	102·9 (94·4 to 113·0)	44·3 (39·7 to 49·4)	23·7 (21·1 to 26·9)	21·1 (15·1 to 29·1)	13·8 (12·8 to 15·0)	247·6 (227·2 to 271·8)	106·5 (95·6 to 118·9)	57·1 (50·7 to 64·6)	50·8 (36·3 to 69·9)	33·2 (30·7 to 36·2)
Kenya*	2025	105·1 (96·5 to 115·5)	45·4 (40·8 to 50·6)	24·2 (21·5 to 27·3)	22·0 (15·7 to 30·3)	13·5 (12·4 to 14·7)	252·9 (232·1 to 278·0)	109·3 (98·1 to 121·7)	58·2 (51·6 to 65·8)	52·9 (37·7 to 72·9)	32·5 (29·9 to 35·4)
Kenya*	2026	107·3 (98·2 to 118·1)	46·6 (41·7 to 51·9)	24·6 (21·9 to 27·9)	22·9 (16·3 to 31·5)	13·2 (12·1 to 14·5)	258·2 (236·4 to 284·2)	112·1 (100·4 to 124·8)	59·2 (52·6 to 67·0)	55·0 (39·3 to 75·8)	31·8 (29·0 to 34·8)
Kenya*	2027	109·5 (100·2 to 120·8)	47·7 (42·6 to 53·2)	25·0 (22·2 to 28·4)	23·8 (16·9 to 32·8)	13·0 (11·8 to 14·2)	263·5 (241·0 to 290·6)	114·8 (102·4 to 128·0)	60·3 (53·5 to 68·2)	57·3 (40·7 to 78·9)	31·2 (28·4 to 34·2)
Kenya*	2028	111·8 (102·1 to 123·4)	48·8 (43·7 to 54·6)	25·5 (22·6 to 28·8)	24·7 (17·6 to 34·1)	12·7 (11·5 to 14·0)	268·9 (245·6 to 297·0)	117·5 (105·1 to 131·4)	61·3 (54·4 to 69·4)	59·5 (42·3 to 82·1)	30·6 (27·8 to 33·8)
Kenya*	2029	114·1 (104·1 to 126·3)	50·0 (44·8 to 56·1)	25·9 (23·0 to 29·3)	25·7 (18·3 to 35·5)	12·5 (11·3 to 13·9)	274·4 (250·4 to 303·8)	120·3 (107·8 to 134·9)	62·2 (55·3 to 70·5)	61·8 (44·0 to 85·3)	30·2 (27·2 to 33·5)
Kenya*	2030	116·3 (105·9 to 128·9)	51·1 (45·6 to 57·3)	26·3 (23·3 to 29·7)	26·7 (19·0 to 37·0)	12·3 (11·0 to 13·8)	279·9 (254·7 to 310·1)	123·0 (109·7 to 137·9)	63·2 (56·1 to 71·6)	64·1 (45·6 to 89·0)	29·7 (26·6 to 33·1)
Kenya*	2031	118·7 (107·9 to 131·6)	52·3 (46·5 to 58·8)	26·7 (23·7 to 30·2)	27·7 (19·7 to 38·2)	12·1 (10·8 to 13·6)	285·7 (259·6 to 316·8)	125·8 (112·0 to 141·6)	64·2 (57·0 to 72·7)	66·6 (47·4 to 92·0)	29·2 (26·1 to 32·6)
Kenya*	2032	121·2 (109·9 to 134·7)	53·4 (47·5 to 60·1)	27·0 (24·0 to 30·6)	28·7 (20·4 to 39·9)	12·0 (10·7 to 13·4)	291·6 (264·6 to 324·1)	128·6 (114·3 to 144·7)	65·1 (57·8 to 73·7)	69·1 (49·1 to 96·1)	28·8 (25·8 to 32·2)
Kenya*	2033	123·7 (112·0 to 137·8)	54·6 (48·5 to 61·3)	27·4 (24·4 to 31·1)	29·8 (21·1 to 41·6)	11·8 (10·5 to 13·3)	297·7 (269·6 to 331·5)	131·5 (116·6 to 147·6)	66·0 (58·7 to 74·8)	71·7 (50·8 to 100·0)	28·5 (25·3 to 32·0)

Kenya*	2034	126·3 (114·2 to 140·7)	55·9 (49·5 to 62·6)	27·8 (24·7 to 31·5)	30·9 (22·0 to 43·2)	11·7 (10·4 to 13·2)	304·0 (274·9 to 338·4)	134·4 (119·1 to 150·6)	67·0 (59·5 to 75·9)	74·4 (52·8 to 104·0)	28·1 (25·0 to 31·7)
Kenya*	2035	129·0 (116·2 to 143·9)	57·1 (50·5 to 64·0)	28·2 (25·0 to 31·9)	32·1 (22·7 to 44·9)	11·6 (10·3 to 13·1)	310·3 (279·6 to 346·2)	137·3 (121·4 to 154·1)	67·9 (60·2 to 76·9)	77·2 (54·7 to 108·0)	27·9 (24·8 to 31·5)
Kenya*	2036	131·7 (118·4 to 147·2)	58·3 (51·4 to 65·9)	28·6 (25·4 to 32·4)	33·3 (23·7 to 46·7)	11·5 (10·2 to 13·1)	316·8 (285·0 to 354·3)	140·3 (123·7 to 158·5)	68·8 (61·0 to 77·9)	80·0 (56·9 to 112·3)	27·7 (24·6 to 31·5)
Kenya*	2037	134·4 (120·8 to 150·6)	59·6 (52·5 to 67·3)	29·0 (25·7 to 32·8)	34·5 (24·4 to 48·6)	11·4 (10·1 to 13·0)	323·5 (290·6 to 362·3)	143·3 (126·4 to 161·9)	69·7 (61·8 to 79·0)	83·0 (58·8 to 116·9)	27·5 (24·3 to 31·2)
Kenya*	2038	137·3 (123·2 to 153·9)	60·8 (53·7 to 69·0)	29·3 (26·0 to 33·3)	35·7 (25·2 to 50·2)	11·4 (10·0 to 12·9)	330·3 (296·5 to 370·4)	146·4 (129·1 to 166·0)	70·6 (62·5 to 80·0)	86·0 (60·6 to 120·8)	27·3 (24·1 to 31·1)
Kenya*	2039	140·2 (125·3 to 157·3)	62·1 (54·6 to 70·6)	29·7 (26·3 to 33·7)	37·0 (26·0 to 52·0)	11·3 (10·0 to 13·0)	337·3 (301·5 to 378·5)	149·5 (131·3 to 169·9)	71·5 (63·3 to 81·1)	89·1 (62·5 to 125·0)	27·2 (24·0 to 31·2)
Kenya*	2040	143·1 (127·8 to 161·4)	63·4 (55·4 to 72·3)	30·1 (26·6 to 34·1)	38·4 (26·7 to 54·0)	11·3 (9·9 to 13·2)	344·2 (307·5 to 388·4)	152·4 (133·2 to 174·0)	72·3 (63·9 to 82·0)	92·3 (64·1 to 129·8)	27·2 (23·8 to 31·7)
Kenya*	2041	146·1 (130·0 to 165·3)	64·7 (56·3 to 74·3)	30·4 (26·9 to 34·5)	39·7 (27·5 to 56·1)	11·2 (9·8 to 13·1)	351·6 (312·9 to 397·8)	155·6 (135·5 to 178·7)	73·2 (64·7 to 83·1)	95·6 (66·2 to 135·0)	27·1 (23·6 to 31·6)
Kenya*	2042	149·2 (132·5 to 169·4)	66·0 (57·4 to 75·8)	30·8 (27·2 to 34·9)	41·2 (28·4 to 58·2)	11·2 (9·8 to 13·2)	358·9 (318·7 to 407·6)	158·7 (138·2 to 182·4)	74·1 (65·5 to 84·1)	99·0 (68·3 to 140·0)	27·0 (23·5 to 31·8)
Kenya*	2043	152·2 (134·7 to 173·3)	67·3 (58·5 to 77·4)	31·2 (27·5 to 35·3)	42·6 (29·3 to 60·7)	11·2 (9·7 to 13·2)	366·3 (324·2 to 417·1)	161·9 (140·7 to 186·3)	75·0 (66·2 to 85·0)	102·5 (70·4 to 146·0)	27·0 (23·2 to 31·7)
Kenya*	2044	155·5 (137·2 to 177·5)	68·6 (59·3 to 79·4)	31·5 (27·8 to 35·8)	44·1 (30·3 to 63·3)	11·2 (9·7 to 13·3)	374·0 (330·1 to 427·1)	165·1 (142·7 to 191·1)	75·8 (67·0 to 86·1)	106·2 (72·9 to 152·3)	26·9 (23·3 to 32·0)
Kenya*	2045	158·7 (139·6 to 181·7)	70·0 (60·6 to 81·5)	31·9 (28·1 to 36·2)	45·7 (31·2 to 65·4)	11·2 (9·6 to 13·3)	381·8 (335·9 to 437·2)	168·3 (145·7 to 196·1)	76·7 (67·7 to 87·1)	109·9 (75·2 to 157·4)	26·9 (23·1 to 32·1)
Kenya*	2046	162·0 (142·1 to 185·9)	71·3 (61·5 to 83·8)	32·2 (28·4 to 36·6)	47·3 (32·2 to 68·2)	11·1 (9·6 to 13·2)	389·8 (342·0 to 447·3)	171·7 (148·0 to 201·6)	77·6 (68·4 to 88·1)	113·8 (77·4 to 164·1)	26·8 (23·0 to 31·9)
Kenya*	2047	165·4 (144·7 to 190·4)	72·7 (62·5 to 85·5)	32·6 (28·7 to 37·0)	49·0 (33·1 to 70·7)	11·1 (9·4 to 13·4)	397·9 (348·2 to 458·2)	175·0 (150·4 to 205·8)	78·5 (69·1 to 89·0)	117·8 (79·6 to 170·1)	26·6 (22·6 to 32·1)

Kenya*	2048	168·8 (147·4 to 195·4)	74·2 (63·2 to 88·0)	33·0 (29·1 to 37·4)	50·7 (34·1 to 73·4)	11·0 (9·4 to 13·2)	406·2 (354·7 to 470·2)	178·5 (152·0 to 211·8)	79·3 (70·0 to 90·0)	121·9 (82·0 to 176·6)	26·5 (22·5 to 31·8)
Kenya*	2049	172·3 (149·9 to 200·1)	75·6 (64·3 to 89·7)	33·3 (29·4 to 37·8)	52·4 (35·0 to 76·5)	11·0 (9·3 to 13·1)	414·6 (360·7 to 481·6)	181·9 (154·6 to 215·9)	80·2 (70·8 to 91·0)	126·1 (84·3 to 184·1)	26·4 (22·3 to 31·6)
Kenya*	2050	175·8 (152·4 to 204·4)	77·0 (65·3 to 92·4)	33·7 (29·7 to 38·2)	54·2 (36·0 to 79·8)	10·9 (9·1 to 13·1)	423·1 (366·7 to 491·9)	185·3 (157·1 to 222·3)	81·1 (71·5 to 92·0)	130·5 (86·6 to 192·1)	26·2 (22·0 to 31·6)
Kyrgyzstan*	1995	44·5 (38·9 to 50·8)	22·2 (18·1 to 26·7)	18·7 (14·7 to 23·0)	0·0 (0·0 to 0·0)	3·7 (3·7 to 3·7)	187·2 (163·4 to 213·7)	93·3 (76·2 to 112·3)	78·5 (61·7 to 96·5)	0·0 (0·0 to 0·0)	15·4 (15·4 to 15·4)
Kyrgyzstan*	1996	41·6 (36·2 to 47·3)	20·1 (16·6 to 24·1)	18·6 (14·9 to 22·9)	0·0 (0·0 to 0·0)	2·9 (2·9 to 2·9)	174·7 (152·1 to 198·7)	84·3 (69·6 to 101·1)	78·3 (62·5 to 96·4)	0·0 (0·0 to 0·0)	12·0 (12·0 to 12·0)
Kyrgyzstan*	1997	42·2 (36·9 to 47·9)	20·5 (17·0 to 24·7)	19·0 (15·5 to 23·6)	0·0 (0·0 to 0·0)	2·7 (2·7 to 2·7)	177·4 (155·0 to 201·5)	86·2 (71·3 to 103·9)	79·8 (65·1 to 99·1)	0·0 (0·0 to 0·0)	11·3 (11·3 to 11·3)
Kyrgyzstan*	1998	45·4 (40·2 to 51·3)	20·6 (17·0 to 24·7)	20·1 (16·6 to 25·1)	0·0 (0·0 to 0·0)	4·7 (4·7 to 4·7)	190·9 (169·0 to 215·6)	86·5 (71·6 to 103·6)	84·6 (70·0 to 105·5)	0·0 (0·0 to 0·0)	19·8 (19·8 to 19·8)
Kyrgyzstan*	1999	41·3 (36·4 to 46·5)	19·9 (16·4 to 23·9)	19·6 (16·4 to 24·1)	0·0 (0·0 to 0·0)	1·8 (1·8 to 1·8)	173·6 (152·8 to 195·4)	83·5 (69·0 to 100·4)	82·4 (69·0 to 101·1)	0·0 (0·0 to 0·0)	7·7 (7·7 to 7·7)
Kyrgyzstan*	2000	40·4 (36·0 to 45·2)	18·1 (14·9 to 21·7)	18·7 (15·8 to 22·3)	0·0 (0·0 to 0·0)	3·6 (3·6 to 3·6)	169·8 (151·1 to 189·9)	76·0 (62·6 to 91·1)	78·7 (66·3 to 93·5)	0·0 (0·0 to 0·0)	15·1 (15·1 to 15·1)
Kyrgyzstan*	2001	38·4 (34·3 to 43·0)	17·7 (14·7 to 21·2)	19·1 (16·3 to 22·3)	0·0 (0·0 to 0·0)	1·6 (1·6 to 1·6)	161·3 (144·0 to 180·7)	74·5 (61·9 to 89·3)	80·2 (68·5 to 93·7)	0·0 (0·0 to 0·0)	6·5 (6·5 to 6·5)
Kyrgyzstan*	2002	42·4 (38·3 to 47·1)	18·9 (15·8 to 22·4)	20·5 (17·8 to 23·5)	0·0 (0·0 to 0·0)	3·0 (3·0 to 3·0)	178·3 (160·8 to 198·1)	79·4 (66·3 to 94·0)	86·3 (74·7 to 98·9)	0·0 (0·0 to 0·0)	12·6 (12·6 to 12·6)
Kyrgyzstan*	2003	49·3 (44·7 to 54·3)	20·6 (17·2 to 24·3)	23·0 (20·1 to 26·2)	0·0 (0·0 to 0·0)	5·7 (5·7 to 5·7)	207·2 (188·0 to 228·3)	86·4 (72·1 to 102·1)	96·7 (84·5 to 110·1)	0·0 (0·0 to 0·0)	24·0 (24·0 to 24·0)
Kyrgyzstan*	2004	52·6 (47·4 to 58·3)	23·7 (19·9 to 27·8)	25·4 (22·3 to 28·8)	0·0 (0·0 to 0·0)	3·5 (3·5 to 3·5)	221·3 (199·4 to 245·0)	99·7 (83·6 to 116·9)	106·6 (93·7 to 120·8)	0·0 (0·0 to 0·0)	14·9 (14·9 to 14·9)
Kyrgyzstan*	2005	60·7 (55·1 to 66·6)	27·3 (23·0 to 32·2)	27·2 (24·1 to 30·7)	0·0 (0·0 to 0·0)	6·1 (6·1 to 6·1)	255·0 (231·5 to 280·1)	114·9 (96·5 to 135·5)	114·3 (101·3 to 129·2)	0·0 (0·0 to 0·0)	25·8 (25·8 to 25·8)

Kyrgyzstan*	2006	64·2 (58·3 to 70·9)	29·6 (24·9 to 35·1)	28·3 (25·1 to 31·8)	0·0 (0·0 to 0·0)	6·3 (6·3 to 6·3)	270·0 (245·2 to 298·0)	124·5 (104·5 to 147·4)	118·9 (105·4 to 133·7)	0·0 (0·0 to 0·0)	26·5 (26·5 to 26·5)
Kyrgyzstan*	2007	66·3 (60·2 to 72·8)	30·6 (25·6 to 36·1)	29·0 (25·8 to 32·4)	0·0 (0·0 to 0·0)	6·7 (6·7 to 6·7)	278·8 (253·2 to 306·0)	128·5 (107·8 to 151·7)	122·0 (108·6 to 136·2)	0·0 (0·0 to 0·0)	28·3 (28·3 to 28·3)
Kyrgyzstan*	2008	68·5 (62·5 to 74·6)	30·2 (25·4 to 35·2)	29·5 (26·4 to 33·1)	0·0 (0·0 to 0·0)	8·8 (8·8 to 8·8)	288·1 (262·5 to 313·4)	127·1 (106·8 to 148·0)	124·0 (111·0 to 138·9)	0·0 (0·0 to 0·0)	36·9 (36·9 to 36·9)
Kyrgyzstan*	2009	70·2 (63·5 to 76·5)	33·3 (28·1 to 38·4)	30·4 (27·0 to 33·9)	0·0 (0·0 to 0·0)	6·5 (6·5 to 6·5)	295·1 (266·9 to 321·6)	139·9 (118·2 to 161·6)	127·7 (113·3 to 142·6)	0·0 (0·0 to 0·0)	27·5 (27·5 to 27·5)
Kyrgyzstan*	2010	74·3 (68·1 to 80·1)	34·7 (29·6 to 39·4)	32·1 (28·8 to 35·5)	0·0 (0·0 to 0·0)	7·6 (7·6 to 7·6)	312·5 (286·1 to 336·6)	145·7 (124·4 to 165·8)	134·9 (120·9 to 149·4)	0·0 (0·0 to 0·0)	31·9 (31·9 to 31·9)
Kyrgyzstan*	2011	79·9 (74·2 to 85·3)	35·9 (31·3 to 40·2)	34·9 (31·3 to 38·5)	0·0 (0·0 to 0·0)	9·0 (9·0 to 9·0)	335·7 (311·8 to 358·6)	151·1 (131·4 to 168·9)	146·7 (131·8 to 161·7)	0·0 (0·0 to 0·0)	37·9 (37·9 to 37·9)
Kyrgyzstan*	2012	82·6 (77·0 to 87·9)	36·5 (32·6 to 40·1)	38·7 (35·3 to 42·3)	0·0 (0·0 to 0·0)	7·4 (7·4 to 7·4)	347·2 (323·8 to 369·4)	153·4 (136·9 to 168·7)	162·7 (148·4 to 177·7)	0·0 (0·0 to 0·0)	31·0 (31·0 to 31·0)
Kyrgyzstan*	2013	85·0 (80·4 to 88·9)	35·1 (32·3 to 37·6)	42·3 (39·0 to 45·5)	0·0 (0·0 to 0·0)	7·6 (7·6 to 7·6)	357·2 (337·9 to 373·8)	147·5 (135·8 to 158·0)	177·7 (163·8 to 191·0)	0·0 (0·0 to 0·0)	31·9 (31·9 to 31·9)
Kyrgyzstan*	2014	84·5 (81·2 to 87·7)	32·3 (30·6 to 33·9)	44·2 (41·3 to 47·2)	0·0 (0·0 to 0·0)	8·0 (8·0 to 8·0)	355·3 (341·1 to 368·8)	135·7 (128·8 to 142·4)	185·8 (173·4 to 198·5)	0·0 (0·0 to 0·1)	33·7 (33·7 to 33·7)
Kyrgyzstan*	2015	83·3 (80·2 to 86·3)	30·5 (29·3 to 31·7)	46·0 (43·2 to 48·6)	0·0 (0·0 to 0·0)	6·8 (6·8 to 6·8)	350·1 (337·3 to 362·8)	128·2 (123·1 to 133·3)	193·2 (181·6 to 204·3)	0·1 (0·0 to 0·1)	28·7 (28·7 to 28·7)
Kyrgyzstan*	2016	80·6 (77·8 to 83·6)	30·1 (28·8 to 31·3)	44·8 (42·2 to 47·3)	0·0 (0·0 to 0·0)	5·7 (5·7 to 5·7)	338·9 (326·9 to 351·4)	126·5 (121·2 to 131·5)	188·4 (177·3 to 198·7)	0·1 (0·0 to 0·1)	24·0 (24·0 to 24·0)
Kyrgyzstan*	2017	80·0 (76·9 to 83·2)	29·5 (27·9 to 31·0)	43·6 (41·1 to 46·4)	0·0 (0·0 to 0·0)	6·9 (6·9 to 6·9)	336·3 (323·2 to 349·5)	123·9 (117·4 to 130·4)	183·3 (172·9 to 194·9)	0·1 (0·1 to 0·1)	29·1 (29·1 to 29·1)
Kyrgyzstan*	2018	80·4 (76·8 to 84·6)	29·9 (27·6 to 32·3)	43·2 (40·1 to 46·7)	0·0 (0·0 to 0·0)	7·3 (7·3 to 7·3)	338·0 (322·7 to 355·8)	125·8 (116·1 to 135·9)	181·4 (168·5 to 196·2)	0·1 (0·1 to 0·1)	30·8 (30·8 to 30·8)
Kyrgyzstan*	2019	83·2 (79·2 to 87·8)	32·2 (29·3 to 35·3)	43·6 (40·5 to 47·2)	0·0 (0·0 to 0·0)	7·4 (7·4 to 7·4)	349·8 (332·9 to 368·9)	135·3 (123·1 to 148·2)	183·4 (170·3 to 198·4)	0·1 (0·1 to 0·1)	31·1 (30·9 to 31·3)

Kyrgyzstan*	2020	86·8 (82·4 to 91·6)	35·3 (31·9 to 38·9)	41·5 (38·6 to 44·9)	0·0 (0·0 to 0·0)	9·9 (9·7 to 10·2)	364·6 (346·5 to 384·9)	148·6 (134·1 to 163·4)	174·5 (162·1 to 188·7)	0·1 (0·1 to 0·1)	41·5 (41·0 to 42·7)
Kyrgyzstan*	2021	84·7 (80·5 to 89·4)	34·8 (31·4 to 38·3)	40·8 (37·9 to 44·1)	0·0 (0·0 to 0·0)	9·1 (8·9 to 9·4)	356·1 (338·3 to 375·9)	146·3 (132·1 to 161·0)	171·4 (159·2 to 185·4)	0·1 (0·1 to 0·1)	38·2 (37·3 to 39·3)
Kyrgyzstan*	2022	79·2 (74·8 to 84·0)	31·2 (27·6 to 35·0)	40·7 (37·7 to 44·0)	0·0 (0·0 to 0·0)	7·3 (6·9 to 7·8)	333·0 (314·5 to 353·2)	131·3 (115·9 to 147·3)	170·9 (158·6 to 184·9)	0·1 (0·1 to 0·1)	30·7 (29·1 to 32·9)
Kyrgyzstan*	2023	79·7 (75·1 to 84·9)	31·4 (27·4 to 35·8)	40·6 (37·6 to 43·9)	0·0 (0·0 to 0·0)	7·7 (7·3 to 8·4)	335·1 (315·5 to 356·9)	131·9 (115·0 to 150·3)	170·5 (158·2 to 184·5)	0·1 (0·1 to 0·1)	32·5 (30·5 to 35·4)
Kyrgyzstan*	2024	80·2 (75·3 to 85·3)	31·5 (27·2 to 36·0)	40·5 (37·5 to 43·8)	0·0 (0·0 to 0·0)	8·2 (7·6 to 9·1)	337·0 (316·6 to 358·7)	132·4 (114·5 to 151·2)	170·0 (157·7 to 184·0)	0·1 (0·1 to 0·1)	34·5 (32·1 to 38·2)
Kyrgyzstan*	2025	80·6 (75·4 to 86·0)	31·6 (27·0 to 36·4)	40·3 (37·4 to 43·7)	0·0 (0·0 to 0·0)	8·7 (8·0 to 9·8)	338·9 (317·0 to 361·5)	132·7 (113·7 to 152·8)	169·6 (157·3 to 183·6)	0·1 (0·1 to 0·1)	36·5 (33·7 to 41·1)
Kyrgyzstan*	2026	81·2 (75·8 to 87·0)	31·7 (27·0 to 36·6)	40·3 (37·3 to 43·6)	0·0 (0·0 to 0·0)	9·2 (8·4 to 10·4)	341·2 (318·4 to 365·9)	133·3 (113·4 to 154·0)	169·2 (156·9 to 183·2)	0·1 (0·1 to 0·2)	38·6 (35·3 to 43·9)
Kyrgyzstan*	2027	81·7 (76·2 to 87·9)	31·8 (26·8 to 37·1)	40·3 (37·3 to 43·6)	0·0 (0·0 to 0·0)	9·6 (8·7 to 11·1)	343·3 (320·3 to 369·4)	133·7 (112·8 to 156·0)	169·3 (156·9 to 183·4)	0·1 (0·1 to 0·2)	40·3 (36·7 to 46·6)
Kyrgyzstan*	2028	82·4 (76·7 to 88·7)	32·1 (26·9 to 37·9)	40·4 (37·4 to 43·7)	0·0 (0·0 to 0·0)	9·9 (9·0 to 11·7)	346·2 (322·3 to 372·7)	134·7 (113·3 to 159·1)	169·7 (157·3 to 183·9)	0·1 (0·1 to 0·2)	41·7 (37·7 to 49·1)
Kyrgyzstan*	2029	83·6 (77·5 to 90·3)	32·8 (27·6 to 38·9)	40·5 (37·6 to 43·9)	0·0 (0·0 to 0·0)	10·2 (9·2 to 12·2)	351·6 (325·6 to 379·7)	138·1 (116·0 to 163·5)	170·3 (157·9 to 184·6)	0·1 (0·1 to 0·2)	43·0 (38·8 to 51·2)
Kyrgyzstan*	2030	84·8 (78·4 to 91·6)	33·5 (28·1 to 39·8)	40·7 (37·8 to 44·2)	0·0 (0·0 to 0·0)	10·5 (9·4 to 12·6)	356·4 (329·7 to 385·2)	141·0 (118·0 to 167·4)	171·2 (158·7 to 185·6)	0·1 (0·1 to 0·2)	44·1 (39·5 to 53·0)
Kyrgyzstan*	2031	86·0 (79·6 to 92·9)	34·2 (28·5 to 40·7)	41·0 (38·0 to 44·5)	0·0 (0·0 to 0·0)	10·7 (9·5 to 13·0)	361·3 (334·5 to 390·6)	143·9 (119·7 to 171·2)	172·3 (159·7 to 186·9)	0·1 (0·1 to 0·2)	45·0 (40·1 to 54·7)
Kyrgyzstan*	2032	87·1 (80·6 to 94·4)	34·9 (29·1 to 41·2)	41·2 (38·2 to 44·7)	0·0 (0·0 to 0·0)	11·0 (9·7 to 13·4)	366·1 (338·9 to 396·8)	146·5 (122·1 to 173·2)	173·4 (160·6 to 188·1)	0·1 (0·1 to 0·2)	46·0 (40·8 to 56·4)
Kyrgyzstan*	2033	88·3 (81·7 to 96·0)	35·5 (29·5 to 42·2)	41·5 (38·5 to 45·0)	0·0 (0·0 to 0·0)	11·2 (9·9 to 13·8)	371·0 (343·2 to 403·7)	149·3 (124·2 to 177·2)	174·6 (161·7 to 189·3)	0·1 (0·1 to 0·2)	47·0 (41·5 to 58·0)

Kyrgyzstan*	2034	89·5 (82·8 to 97·1)	36·2 (30·1 to 43·1)	41·9 (38·8 to 45·4)	0·0 (0·0 to 0·0)	11·4 (10·0 to 14·2)	376·0 (348·0 to 408·2)	152·1 (126·6 to 181·3)	175·9 (162·9 to 190·7)	0·1 (0·1 to 0·2)	47·8 (42·1 to 59·6)
Kyrgyzstan*	2035	90·7 (83·7 to 98·7)	36·9 (30·6 to 43·9)	42·2 (39·1 to 45·7)	0·0 (0·0 to 0·1)	11·6 (10·1 to 14·7)	381·1 (352·0 to 414·8)	154·9 (128·5 to 184·3)	177·3 (164·2 to 192·1)	0·2 (0·1 to 0·2)	48·7 (42·6 to 61·7)
Kyrgyzstan*	2036	91·8 (84·7 to 100·1)	37·4 (30·9 to 44·7)	42·5 (39·3 to 46·0)	0·0 (0·0 to 0·1)	11·8 (10·3 to 15·2)	385·8 (355·8 to 420·6)	157·4 (130·0 to 188·0)	178·5 (165·3 to 193·4)	0·2 (0·1 to 0·2)	49·7 (43·2 to 63·9)
Kyrgyzstan*	2037	92·8 (85·6 to 101·3)	38·0 (31·3 to 45·3)	42·7 (39·5 to 46·2)	0·0 (0·0 to 0·1)	12·2 (10·6 to 15·8)	390·2 (359·9 to 425·6)	159·5 (131·5 to 190·3)	179·4 (166·2 to 194·3)	0·2 (0·1 to 0·2)	51·1 (44·5 to 66·2)
Kyrgyzstan*	2038	93·9 (86·5 to 102·5)	38·5 (31·7 to 46·3)	42·9 (39·7 to 46·4)	0·0 (0·0 to 0·1)	12·5 (10·8 to 16·3)	394·6 (363·7 to 430·9)	161·6 (133·0 to 194·5)	180·3 (167·0 to 195·1)	0·2 (0·1 to 0·2)	52·6 (45·5 to 68·7)
Kyrgyzstan*	2039	95·0 (87·4 to 103·9)	39·0 (32·0 to 47·0)	43·1 (40·0 to 46·6)	0·0 (0·0 to 0·1)	12·9 (11·1 to 17·1)	399·3 (367·2 to 436·6)	163·8 (134·4 to 197·5)	181·2 (167·9 to 196·0)	0·2 (0·1 to 0·2)	54·1 (46·5 to 71·7)
Kyrgyzstan*	2040	96·1 (88·2 to 105·1)	39·5 (32·2 to 47·3)	43·3 (40·2 to 46·9)	0·0 (0·0 to 0·1)	13·3 (11·3 to 17·8)	403·9 (370·7 to 441·7)	165·9 (135·3 to 199·0)	182·1 (168·8 to 196·9)	0·2 (0·1 to 0·3)	55·8 (47·5 to 74·7)
Kyrgyzstan*	2041	97·2 (89·1 to 106·4)	40·0 (32·7 to 48·4)	43·5 (40·3 to 47·1)	0·0 (0·0 to 0·1)	13·7 (11·6 to 18·5)	408·6 (374·5 to 447·3)	168·0 (137·6 to 203·3)	183·0 (169·6 to 197·8)	0·2 (0·1 to 0·3)	57·5 (48·7 to 77·6)
Kyrgyzstan*	2042	98·3 (90·1 to 108·0)	40·4 (33·1 to 49·3)	43·7 (40·5 to 47·3)	0·0 (0·0 to 0·1)	14·1 (11·9 to 19·4)	413·3 (378·8 to 453·9)	170·0 (138·9 to 207·1)	183·8 (170·3 to 198·8)	0·2 (0·1 to 0·3)	59·4 (49·9 to 81·4)
Kyrgyzstan*	2043	99·5 (90·9 to 109·5)	40·9 (33·5 to 49·9)	43·9 (40·7 to 47·5)	0·0 (0·0 to 0·1)	14·6 (12·2 to 20·2)	418·1 (382·1 to 460·3)	171·9 (140·7 to 209·6)	184·6 (171·1 to 199·6)	0·2 (0·1 to 0·3)	61·3 (51·4 to 84·9)
Kyrgyzstan*	2044	100·6 (91·8 to 111·0)	41·4 (33·8 to 50·4)	44·1 (40·9 to 47·7)	0·1 (0·0 to 0·1)	15·1 (12·6 to 21·2)	422·9 (385·9 to 466·6)	173·8 (142·2 to 211·8)	185·3 (171·7 to 200·4)	0·2 (0·1 to 0·3)	63·5 (52·9 to 89·1)
Kyrgyzstan*	2045	101·7 (92·8 to 112·6)	41·8 (34·2 to 50·8)	44·2 (41·0 to 47·8)	0·1 (0·0 to 0·1)	15·7 (12·9 to 22·1)	427·6 (390·2 to 473·3)	175·6 (143·8 to 213·6)	185·9 (172·2 to 201·1)	0·2 (0·2 to 0·3)	65·8 (54·3 to 92·7)
Kyrgyzstan*	2046	102·9 (93·8 to 114·3)	42·2 (34·2 to 51·4)	44·3 (41·0 to 48·0)	0·1 (0·0 to 0·1)	16·3 (13·4 to 23·4)	432·5 (394·2 to 480·5)	177·4 (143·7 to 215·8)	186·3 (172·5 to 201·6)	0·2 (0·2 to 0·3)	68·6 (56·3 to 98·5)
Kyrgyzstan*	2047	104·1 (94·9 to 115·8)	42·7 (34·8 to 51·7)	44·4 (41·1 to 48·1)	0·1 (0·0 to 0·1)	17·0 (13·8 to 24·6)	437·6 (398·7 to 486·9)	179·3 (146·1 to 217·3)	186·6 (172·7 to 202·0)	0·2 (0·2 to 0·3)	71·5 (58·1 to 103·4)

Kyrgyzstan*	2048	105·4 (95·8 to 117·6)	43·2 (35·1 to 52·4)	44·4 (41·1 to 48·1)	0·1 (0·0 to 0·1)	17·8 (14·4 to 26·2)	443·2 (402·7 to 494·1)	181·5 (147·6 to 220·2)	186·8 (172·8 to 202·3)	0·2 (0·2 to 0·3)	74·7 (60·7 to 110·1)
Kyrgyzstan*	2049	106·8 (97·1 to 119·5)	43·7 (35·5 to 53·2)	44·4 (41·1 to 48·1)	0·1 (0·0 to 0·1)	18·6 (14·8 to 27·5)	449·1 (408·1 to 502·1)	183·8 (149·2 to 223·6)	186·8 (172·8 to 202·4)	0·2 (0·2 to 0·3)	78·3 (62·4 to 115·5)
Kyrgyzstan*	2050	108·3 (98·1 to 121·4)	44·3 (36·0 to 53·9)	44·4 (41·1 to 48·1)	0·1 (0·0 to 0·1)	19·5 (15·5 to 29·2)	455·3 (412·4 to 510·4)	186·2 (151·4 to 226·6)	186·8 (172·7 to 202·4)	0·3 (0·2 to 0·4)	82·1 (65·3 to 122·5)
Cambodia*	1995	42·7 (38·0 to 48·0)	9·0 (7·3 to 10·7)	20·4 (16·2 to 25·3)	0·4 (0·3 to 0·5)	12·9 (12·9 to 12·9)	120·6 (107·4 to 135·7)	25·3 (20·6 to 30·3)	57·6 (45·8 to 71·6)	1·1 (0·8 to 1·5)	36·6 (36·6 to 36·6)
Cambodia*	1996	38·4 (33·6 to 44·1)	9·8 (8·1 to 11·7)	21·4 (17·0 to 26·8)	0·4 (0·3 to 0·5)	6·8 (6·8 to 6·8)	108·5 (94·9 to 124·7)	27·7 (22·8 to 33·2)	60·6 (47·9 to 75·8)	1·1 (0·8 to 1·5)	19·1 (19·1 to 19·1)
Cambodia*	1997	36·2 (31·7 to 41·7)	9·1 (7·5 to 10·9)	21·1 (16·8 to 26·2)	0·4 (0·3 to 0·5)	5·6 (5·6 to 5·6)	102·2 (89·5 to 117·8)	25·6 (21·2 to 30·8)	59·6 (47·3 to 74·1)	1·0 (0·7 to 1·5)	15·9 (15·9 to 15·9)
Cambodia*	1998	35·0 (30·4 to 40·5)	8·9 (7·4 to 10·7)	21·9 (17·7 to 26·9)	0·4 (0·3 to 0·5)	3·9 (3·9 to 3·9)	98·9 (86·0 to 114·4)	25·2 (20·9 to 30·2)	61·7 (50·0 to 75·9)	1·0 (0·7 to 1·5)	10·9 (10·9 to 10·9)
Cambodia*	1999	35·4 (30·7 to 40·9)	8·8 (7·2 to 10·6)	22·9 (18·7 to 27·9)	0·4 (0·3 to 0·5)	3·4 (3·4 to 3·4)	100·1 (86·8 to 115·6)	24·7 (20·4 to 29·9)	64·7 (52·9 to 78·9)	1·1 (0·7 to 1·5)	9·6 (9·6 to 9·6)
Cambodia*	2000	36·9 (32·1 to 42·3)	8·3 (6·7 to 9·9)	23·9 (19·5 to 29·1)	0·4 (0·3 to 0·5)	4·3 (4·3 to 4·3)	104·2 (90·8 to 119·4)	23·4 (19·1 to 28·0)	67·7 (55·1 to 82·3)	1·1 (0·7 to 1·5)	12·1 (12·1 to 12·1)
Cambodia*	2001	38·5 (33·8 to 44·0)	8·1 (6·6 to 9·8)	24·9 (20·6 to 30·1)	0·4 (0·3 to 0·5)	5·1 (5·1 to 5·1)	108·7 (95·4 to 124·2)	22·8 (18·6 to 27·6)	70·4 (58·2 to 85·1)	1·1 (0·7 to 1·5)	14·4 (14·4 to 14·4)
Cambodia*	2002	39·6 (34·6 to 45·5)	8·9 (7·3 to 10·8)	26·8 (22·1 to 32·4)	0·4 (0·3 to 0·6)	3·4 (3·4 to 3·4)	111·8 (97·7 to 128·4)	25·3 (20·5 to 30·4)	75·8 (62·3 to 91·6)	1·2 (0·8 to 1·6)	9·6 (9·6 to 9·6)
Cambodia*	2003	43·3 (38·7 to 49·0)	9·1 (7·4 to 10·9)	27·8 (23·3 to 33·0)	0·4 (0·3 to 0·6)	6·0 (6·0 to 6·0)	122·3 (109·3 to 138·4)	25·7 (21·0 to 30·8)	78·5 (66·0 to 93·2)	1·2 (0·8 to 1·7)	16·9 (16·9 to 16·9)
Cambodia*	2004	44·4 (40·0 to 48·9)	8·9 (7·3 to 10·6)	28·2 (24·2 to 32·6)	0·5 (0·3 to 0·7)	6·8 (6·8 to 6·8)	125·5 (113·1 to 138·2)	25·2 (20·6 to 30·0)	79·8 (68·4 to 92·0)	1·3 (0·9 to 1·9)	19·2 (19·2 to 19·2)
Cambodia*	2005	47·1 (43·4 to 51·4)	8·7 (7·1 to 10·4)	28·6 (25·1 to 32·3)	0·5 (0·3 to 0·7)	9·3 (9·3 to 9·3)	133·2 (122·5 to 145·3)	24·5 (20·0 to 29·3)	80·9 (71·0 to 91·2)	1·4 (1·0 to 1·9)	26·4 (26·4 to 26·4)

Cambodia*	2006	46·8 (43·1 to 50·7)	8·9 (7·3 to 10·7)	28·2 (24·9 to 31·4)	0·5 (0·4 to 0·7)	9·2 (9·2 to 9·2)	132·2 (121·9 to 143·3)	25·2 (20·8 to 30·1)	79·6 (70·4 to 88·6)	1·5 (1·0 to 2·0)	25·9 (25·9 to 25·9)
Cambodia*	2007	48·4 (45·0 to 52·1)	9·8 (8·1 to 11·7)	28·7 (25·7 to 31·6)	0·5 (0·4 to 0·8)	9·4 (9·4 to 9·4)	136·8 (127·2 to 147·2)	27·6 (22·8 to 33·0)	81·0 (72·7 to 89·4)	1·5 (1·1 to 2·2)	26·6 (26·6 to 26·6)
Cambodia*	2008	53·3 (49·9 to 56·8)	10·6 (9·0 to 12·5)	31·9 (29·2 to 34·6)	0·6 (0·4 to 0·8)	10·2 (10·2 to 10·2)	150·5 (141·1 to 160·4)	29·9 (25·3 to 35·3)	90·0 (82·4 to 97·8)	1·6 (1·1 to 2·3)	28·9 (28·9 to 28·9)
Cambodia*	2009	60·4 (57·1 to 63·7)	12·7 (11·0 to 14·9)	36·5 (34·0 to 39·0)	0·6 (0·4 to 0·8)	10·6 (10·6 to 10·6)	170·7 (161·4 to 179·9)	35·9 (30·9 to 42·0)	103·2 (96·2 to 110·2)	1·7 (1·1 to 2·4)	30·0 (30·0 to 30·0)
Cambodia*	2010	66·3 (63·5 to 69·3)	13·6 (11·9 to 15·4)	39·8 (37·3 to 42·2)	0·6 (0·4 to 0·8)	12·4 (12·4 to 12·4)	187·4 (179·4 to 195·7)	38·3 (33·7 to 43·5)	112·4 (105·4 to 119·2)	1·6 (1·1 to 2·3)	35·1 (35·1 to 35·1)
Cambodia*	2011	72·0 (69·2 to 74·8)	14·1 (12·8 to 15·6)	44·5 (41·8 to 46·9)	0·5 (0·3 to 0·7)	12·9 (12·9 to 12·9)	203·5 (195·6 to 211·5)	39·9 (36·1 to 44·0)	125·7 (118·2 to 132·5)	1·4 (1·0 to 2·0)	36·5 (36·5 to 36·5)
Cambodia*	2012	73·1 (70·2 to 75·9)	15·5 (14·4 to 16·7)	48·4 (45·9 to 51·0)	0·4 (0·3 to 0·6)	8·8 (8·8 to 8·8)	206·6 (198·4 to 214·5)	43·8 (40·6 to 47·1)	136·8 (129·7 to 144·1)	1·1 (0·7 to 1·6)	24·9 (24·9 to 24·9)
Cambodia*	2013	75·6 (72·8 to 78·3)	16·2 (15·3 to 17·1)	49·4 (46·9 to 52·1)	0·2 (0·1 to 0·3)	9·8 (9·8 to 9·8)	213·5 (205·8 to 221·1)	45·7 (43·2 to 48·4)	139·6 (132·6 to 147·1)	0·5 (0·3 to 0·7)	27·7 (27·7 to 27·7)
Cambodia*	2014	76·0 (73·1 to 79·0)	16·7 (15·8 to 17·6)	48·9 (46·2 to 51·8)	0·2 (0·1 to 0·3)	10·2 (10·2 to 10·2)	214·7 (206·6 to 223·2)	47·1 (44·6 to 49·7)	138·2 (130·4 to 146·3)	0·6 (0·4 to 0·9)	28·9 (28·9 to 28·9)
Cambodia*	2015	74·7 (71·7 to 77·7)	17·0 (16·0 to 18·0)	47·8 (45·1 to 50·6)	0·3 (0·2 to 0·5)	9·6 (9·6 to 9·6)	211·1 (202·5 to 219·6)	48·0 (45·3 to 50·8)	135·1 (127·5 to 143·0)	1·0 (0·7 to 1·4)	27·0 (27·0 to 27·0)
Cambodia*	2016	75·8 (72·4 to 79·4)	18·1 (16·8 to 19·3)	48·9 (45·7 to 52·5)	0·5 (0·3 to 0·7)	8·4 (8·4 to 8·4)	214·2 (204·5 to 224·3)	51·0 (47·6 to 54·5)	138·1 (129·1 to 148·3)	1·4 (0·9 to 1·9)	23·6 (23·6 to 23·6)
Cambodia*	2017	77·8 (72·9 to 83·4)	18·6 (17·0 to 20·4)	50·3 (45·7 to 55·8)	0·6 (0·4 to 0·8)	8·3 (8·3 to 8·3)	219·8 (205·9 to 235·6)	52·6 (47·9 to 57·5)	142·0 (129·1 to 157·7)	1·6 (1·1 to 2·2)	23·6 (23·6 to 23·6)
Cambodia*	2018	79·9 (72·8 to 87·9)	20·1 (17·9 to 22·6)	52·4 (46·1 to 59·7)	0·6 (0·4 to 0·8)	6·7 (6·7 to 6·7)	225·6 (205·8 to 248·4)	56·8 (50·6 to 63·7)	148·2 (130·2 to 168·8)	1·7 (1·2 to 2·4)	18·9 (18·9 to 18·9)
Cambodia*	2019	82·6 (75·2 to 91·0)	21·0 (18·4 to 23·8)	54·9 (48·2 to 62·5)	0·7 (0·5 to 0·9)	6·0 (5·9 to 6·1)	233·3 (212·4 to 257·2)	59·4 (51·9 to 67·2)	155·1 (136·3 to 176·6)	1·9 (1·3 to 2·6)	17·0 (16·7 to 17·3)

Cambodia*	2020	88·1 (81·0 to 96·4)	26·7 (24·1 to 29·4)	53·3 (46·9 to 60·7)	0·7 (0·5 to 0·9)	7·5 (7·3 to 7·7)	249·0 (228·8 to 272·3)	75·4 (68·0 to 83·1)	150·7 (132·5 to 171·5)	1·9 (1·3 to 2·6)	21·1 (20·5 to 21·6)
Cambodia*	2021	82·3 (75·0 to 90·7)	21·4 (18·7 to 24·2)	54·0 (47·5 to 61·5)	0·7 (0·5 to 0·9)	6·2 (5·9 to 6·5)	232·5 (211·9 to 256·3)	60·5 (52·9 to 68·5)	152·5 (134·2 to 173·7)	1·9 (1·3 to 2·7)	17·5 (16·8 to 18·3)
Cambodia*	2022	80·5 (73·2 to 88·8)	20·6 (18·0 to 23·2)	54·5 (47·9 to 61·9)	0·7 (0·5 to 1·0)	4·8 (4·5 to 5·1)	227·4 (206·9 to 250·9)	58·1 (51·0 to 65·7)	153·8 (135·3 to 175·0)	2·0 (1·4 to 2·7)	13·5 (12·6 to 14·5)
Cambodia*	2023	81·0 (73·7 to 89·6)	20·5 (18·0 to 23·2)	54·9 (48·3 to 62·5)	0·7 (0·5 to 1·0)	4·9 (4·5 to 5·2)	228·9 (208·2 to 253·0)	58·0 (50·9 to 65·7)	155·1 (136·4 to 176·5)	2·0 (1·4 to 2·8)	13·8 (12·8 to 14·8)
Cambodia*	2024	81·6 (74·2 to 90·2)	20·5 (17·9 to 23·2)	55·4 (48·7 to 63·0)	0·7 (0·5 to 1·0)	5·0 (4·6 to 5·4)	230·6 (209·5 to 254·8)	58·0 (50·6 to 65·5)	156·5 (137·7 to 178·1)	2·1 (1·5 to 2·9)	14·0 (12·9 to 15·1)
Cambodia*	2025	82·2 (74·7 to 90·8)	20·5 (17·9 to 23·2)	55·9 (49·2 to 63·7)	0·8 (0·5 to 1·1)	5·0 (4·6 to 5·4)	232·3 (211·2 to 256·6)	58·0 (50·6 to 65·6)	158·0 (139·0 to 179·8)	2·2 (1·5 to 3·0)	14·2 (13·0 to 15·4)
Cambodia*	2026	82·8 (75·2 to 91·5)	20·5 (17·9 to 23·3)	56·4 (49·6 to 64·2)	0·8 (0·6 to 1·1)	5·1 (4·6 to 5·6)	234·0 (212·6 to 258·5)	57·9 (50·6 to 65·8)	159·4 (140·2 to 181·5)	2·3 (1·6 to 3·1)	14·4 (13·1 to 15·7)
Cambodia*	2027	83·5 (75·9 to 92·2)	20·5 (17·9 to 23·4)	57·1 (50·2 to 65·0)	0·8 (0·6 to 1·1)	5·1 (4·7 to 5·6)	235·9 (214·3 to 260·4)	57·9 (50·5 to 66·1)	161·2 (141·7 to 183·7)	2·3 (1·6 to 3·2)	14·5 (13·2 to 15·9)
Cambodia*	2028	84·2 (76·4 to 93·1)	20·5 (17·8 to 23·5)	57·7 (50·7 to 65·7)	0·9 (0·6 to 1·2)	5·2 (4·7 to 5·7)	237·8 (215·9 to 262·9)	57·9 (50·3 to 66·4)	162·9 (143·2 to 185·5)	2·4 (1·7 to 3·3)	14·6 (13·3 to 16·1)
Cambodia*	2029	84·9 (77·0 to 93·9)	20·5 (17·8 to 23·6)	58·3 (51·3 to 66·4)	0·9 (0·6 to 1·2)	5·2 (4·7 to 5·8)	239·8 (217·6 to 265·3)	58·0 (50·2 to 66·7)	164·7 (144·8 to 187·5)	2·5 (1·7 to 3·5)	14·7 (13·3 to 16·3)
Cambodia*	2030	85·6 (77·6 to 94·5)	20·5 (17·7 to 23·7)	58·9 (51·8 to 67·0)	0·9 (0·6 to 1·3)	5·2 (4·7 to 5·8)	241·7 (219·1 to 266·9)	58·0 (50·1 to 66·8)	166·4 (146·3 to 189·3)	2·6 (1·8 to 3·6)	14·8 (13·3 to 16·4)
Cambodia*	2031	86·3 (78·2 to 95·3)	20·5 (17·7 to 23·8)	59·6 (52·4 to 67·8)	0·9 (0·7 to 1·3)	5·3 (4·7 to 5·9)	243·9 (221·0 to 269·3)	58·1 (49·9 to 67·1)	168·3 (148·1 to 191·5)	2·7 (1·9 to 3·8)	14·9 (13·2 to 16·6)
Cambodia*	2032	87·2 (78·9 to 96·4)	20·6 (17·7 to 23·9)	60·3 (53·1 to 68·7)	1·0 (0·7 to 1·4)	5·3 (4·7 to 5·9)	246·3 (223·0 to 272·2)	58·3 (50·1 to 67·5)	170·4 (150·0 to 194·0)	2·8 (1·9 to 3·9)	14·9 (13·2 to 16·6)
Cambodia*	2033	88·1 (79·7 to 97·5)	20·7 (17·7 to 24·1)	61·1 (53·8 to 69·6)	1·0 (0·7 to 1·4)	5·3 (4·7 to 5·9)	248·8 (225·1 to 275·4)	58·5 (50·1 to 68·0)	172·5 (151·9 to 196·5)	2·9 (2·0 to 4·1)	14·9 (13·2 to 16·7)

Cambodia*	2034	89·1 (80·7 to 98·7)	20·9 (17·8 to 24·3)	61·9 (54·5 to 70·5)	1·1 (0·7 to 1·5)	5·3 (4·6 to 5·9)	251·6 (228·1 to 278·7)	58·9 (50·2 to 68·7)	174·9 (154·1 to 199·2)	3·0 (2·1 to 4·2)	14·9 (13·1 to 16·7)
Cambodia*	2035	90·2 (81·7 to 99·9)	21·0 (17·9 to 24·6)	62·8 (55·4 to 71·6)	1·1 (0·8 to 1·5)	5·2 (4·6 to 5·9)	254·8 (230·7 to 282·2)	59·4 (50·5 to 69·4)	177·5 (156·4 to 202·2)	3·1 (2·2 to 4·4)	14·8 (13·0 to 16·8)
Cambodia*	2036	91·3 (82·7 to 101·1)	21·2 (18·0 to 24·8)	63·7 (56·2 to 72·6)	1·1 (0·8 to 1·6)	5·2 (4·6 to 6·0)	258·0 (233·6 to 285·7)	60·0 (50·8 to 70·1)	180·0 (158·7 to 205·2)	3·2 (2·2 to 4·5)	14·7 (12·9 to 16·9)
Cambodia*	2037	92·6 (83·7 to 102·6)	21·5 (18·1 to 25·2)	64·7 (57·1 to 73·8)	1·2 (0·8 to 1·7)	5·2 (4·5 to 5·9)	261·5 (236·4 to 289·8)	60·7 (51·2 to 71·2)	182·8 (161·3 to 208·4)	3·4 (2·3 to 4·7)	14·6 (12·7 to 16·7)
Cambodia*	2038	93·8 (84·7 to 104·0)	21·7 (18·2 to 25·5)	65·7 (57·9 to 74·9)	1·2 (0·9 to 1·7)	5·2 (4·5 to 5·9)	265·0 (239·4 to 293·8)	61·4 (51·6 to 72·1)	185·5 (163·6 to 211·5)	3·5 (2·4 to 4·9)	14·6 (12·6 to 16·6)
Cambodia*	2039	95·1 (85·9 to 105·5)	22·0 (18·5 to 26·0)	66·6 (58·8 to 76·0)	1·3 (0·9 to 1·8)	5·2 (4·4 to 6·0)	268·6 (242·6 to 298·0)	62·2 (52·2 to 73·4)	188·1 (166·0 to 214·6)	3·6 (2·5 to 5·2)	14·6 (12·6 to 16·9)
Cambodia*	2040	96·3 (87·0 to 107·0)	22·3 (18·7 to 26·3)	67·5 (59·6 to 77·0)	1·3 (0·9 to 1·9)	5·2 (4·4 to 6·1)	272·2 (245·7 to 302·3)	63·0 (52·8 to 74·4)	190·7 (168·3 to 217·6)	3·8 (2·6 to 5·4)	14·6 (12·5 to 17·2)
Cambodia*	2041	97·6 (88·1 to 108·4)	22·6 (18·9 to 26·7)	68·4 (60·3 to 78·0)	1·4 (0·9 to 2·0)	5·2 (4·4 to 6·1)	275·7 (248·8 to 306·3)	63·9 (53·4 to 75·5)	193·2 (170·4 to 220·5)	3·9 (2·7 to 5·6)	14·7 (12·5 to 17·2)
Cambodia*	2042	98·8 (89·0 to 109·8)	22·9 (19·1 to 27·1)	69·2 (61·0 to 79·0)	1·4 (1·0 to 2·1)	5·3 (4·5 to 6·2)	279·1 (251·5 to 310·3)	64·7 (54·0 to 76·6)	195·5 (172·4 to 223·2)	4·0 (2·8 to 5·8)	14·9 (12·6 to 17·5)
Cambodia*	2043	100·0 (90·1 to 111·1)	23·2 (19·4 to 27·6)	70·0 (61·7 to 79·9)	1·5 (1·0 to 2·1)	5·3 (4·5 to 6·3)	282·6 (254·6 to 314·0)	65·6 (54·7 to 77·9)	197·8 (174·4 to 225·8)	4·2 (2·8 to 6·1)	15·0 (12·6 to 17·9)
Cambodia*	2044	101·2 (91·2 to 112·5)	23·6 (19·6 to 28·0)	70·8 (62·4 to 80·8)	1·5 (1·0 to 2·2)	5·4 (4·6 to 6·5)	286·0 (257·7 to 317·9)	66·5 (55·3 to 79·1)	199·9 (176·2 to 228·3)	4·3 (2·9 to 6·3)	15·3 (12·9 to 18·3)
Cambodia*	2045	102·4 (92·3 to 113·7)	23·9 (19·8 to 28·4)	71·5 (63·0 to 81·6)	1·6 (1·1 to 2·3)	5·5 (4·6 to 6·7)	289·4 (260·6 to 321·4)	67·5 (56·0 to 80·4)	201·9 (178·0 to 230·6)	4·5 (3·0 to 6·5)	15·5 (13·1 to 18·8)
Cambodia*	2046	103·6 (93·3 to 115·2)	24·3 (20·1 to 28·9)	72·1 (63·6 to 82·4)	1·6 (1·1 to 2·4)	5·6 (4·7 to 6·8)	292·8 (263·5 to 325·4)	68·5 (56·7 to 81·6)	203·7 (179·7 to 232·7)	4·6 (3·1 to 6·7)	15·9 (13·2 to 19·2)
Cambodia*	2047	104·8 (94·3 to 116·7)	24·6 (20·3 to 29·3)	72·7 (64·1 to 83·1)	1·7 (1·1 to 2·5)	5·7 (4·8 to 7·0)	296·0 (266·5 to 329·7)	69·5 (57·5 to 82·9)	205·4 (181·2 to 234·7)	4·8 (3·2 to 7·0)	16·2 (13·5 to 19·9)

Cambodia*	2048	105·9 (95·4 to 117·9)	25·0 (20·6 to 29·8)	73·3 (64·6 to 83·7)	1·8 (1·2 to 2·6)	5·9 (4·9 to 7·3)	299·3 (269·5 to 333·2)	70·6 (58·3 to 84·2)	207·0 (182·6 to 236·6)	4·9 (3·3 to 7·2)	16·7 (13·9 to 20·6)
Cambodia*	2049	107·0 (96·3 to 119·0)	25·4 (20·9 to 30·3)	73·7 (65·0 to 84·2)	1·8 (1·2 to 2·7)	6·1 (5·0 to 7·6)	302·2 (272·0 to 336·1)	71·7 (59·2 to 85·5)	208·2 (183·6 to 238·0)	5·1 (3·4 to 7·5)	17·2 (14·3 to 21·5)
Cambodia*	2050	108·1 (97·3 to 120·3)	25·8 (21·3 to 30·7)	74·2 (65·4 to 84·8)	1·9 (1·2 to 2·7)	6·3 (5·2 to 7·8)	305·4 (274·9 to 340·0)	72·8 (60·1 to 86·8)	209·5 (184·7 to 239·5)	5·3 (3·4 to 7·8)	17·8 (14·7 to 22·2)
Kiribati*	1995	200·7 (180·4 to 222·0)	146·0 (130·9 to 163·0)	51·0 (40·0 to 64·1)	0·6 (0·4 to 0·8)	3·1 (3·1 to 3·1)	260·5 (234·2 to 288·1)	189·5 (169·9 to 211·6)	66·3 (51·9 to 83·2)	0·7 (0·5 to 1·1)	4·1 (4·1 to 4·1)
Kiribati*	1996	191·0 (172·9 to 208·9)	141·2 (128·5 to 154·9)	49·3 (38·9 to 61·5)	0·6 (0·4 to 0·8)	0·0 (0·0 to 0·0)	247·9 (224·4 to 271·1)	183·2 (166·8 to 201·1)	63·9 (50·5 to 79·9)	0·7 (0·5 to 1·0)	0·0 (0·0 to 0·0)
Kiribati*	1997	189·7 (172·7 to 206·8)	139·5 (126·7 to 152·4)	48·3 (38·3 to 59·8)	0·5 (0·4 to 0·7)	1·3 (1·3 to 1·3)	246·2 (224·2 to 268·4)	181·1 (164·4 to 197·8)	62·7 (49·7 to 77·6)	0·7 (0·5 to 1·0)	1·7 (1·7 to 1·7)
Kiribati*	1998	189·4 (172·8 to 205·6)	139·9 (127·1 to 153·2)	48·2 (37·9 to 59·2)	0·4 (0·3 to 0·6)	0·9 (0·9 to 0·9)	245·8 (224·2 to 266·9)	181·6 (165·0 to 198·8)	62·6 (49·2 to 76·8)	0·5 (0·4 to 0·8)	1·1 (1·1 to 1·1)
Kiribati*	1999	192·1 (176·3 to 208·9)	141·9 (129·4 to 155·9)	47·8 (37·4 to 58·6)	0·2 (0·2 to 0·3)	2·2 (2·2 to 2·2)	249·3 (228·8 to 271·2)	184·1 (167·9 to 202·3)	62·0 (48·5 to 76·1)	0·3 (0·2 to 0·4)	2·9 (2·9 to 2·9)
Kiribati*	2000	225·2 (210·9 to 240·9)	135·2 (124·3 to 147·5)	45·8 (35·9 to 57·1)	0·0 (0·0 to 0·1)	44·1 (44·1 to 44·1)	292·2 (273·7 to 312·7)	175·4 (161·3 to 191·4)	59·4 (46·5 to 74·1)	0·1 (0·0 to 0·1)	57·3 (57·3 to 57·3)
Kiribati*	2001	221·4 (205·1 to 239·1)	169·6 (156·6 to 183·4)	48·1 (38·1 to 59·9)	0·0 (0·0 to 0·0)	3·7 (3·7 to 3·7)	287·3 (266·1 to 310·3)	220·1 (203·2 to 238·1)	62·4 (49·4 to 77·7)	0·0 (0·0 to 0·0)	4·9 (4·9 to 4·9)
Kiribati*	2002	221·1 (204·1 to 239·0)	170·4 (157·8 to 184·1)	47·3 (37·0 to 58·9)	0·0 (0·0 to 0·0)	3·4 (3·4 to 3·4)	287·0 (264·9 to 310·2)	221·1 (204·8 to 239·0)	61·4 (48·0 to 76·5)	0·0 (0·0 to 0·0)	4·5 (4·5 to 4·5)
Kiribati*	2003	211·7 (195·5 to 228·4)	160·6 (147·9 to 174·2)	45·8 (36·0 to 57·2)	0·0 (0·0 to 0·0)	5·3 (5·3 to 5·3)	274·8 (253·8 to 296·4)	208·5 (191·9 to 226·1)	59·5 (46·8 to 74·3)	0·0 (0·0 to 0·0)	6·9 (6·9 to 6·9)
Kiribati*	2004	306·1 (288·4 to 324·7)	175·9 (161·9 to 190·1)	47·2 (37·1 to 59·0)	0·0 (0·0 to 0·0)	82·9 (82·9 to 82·9)	397·2 (374·3 to 421·5)	228·3 (210·1 to 246·7)	61·3 (48·1 to 76·6)	0·0 (0·0 to 0·0)	107·6 (107·6 to 107·6)
Kiribati*	2005	250·5 (233·3 to 268·1)	169·8 (155·9 to 184·9)	46·5 (36·1 to 58·2)	0·0 (0·0 to 0·0)	34·1 (34·1 to 34·1)	325·1 (302·8 to 347·9)	220·4 (202·4 to 240·0)	60·4 (46·9 to 75·5)	0·0 (0·0 to 0·0)	44·3 (44·3 to 44·3)

Kiribati*	2006	223·4 (208·2 to 239·5)	144·4 (132·6 to 157·7)	43·6 (34·1 to 54·2)	0·0 (0·0 to 0·0)	35·4 (35·4 to 35·4)	289·9 (270·3 to 310·8)	187·4 (172·1 to 204·7)	56·6 (44·2 to 70·4)	0·0 (0·0 to 0·0)	46·0 (46·0 to 46·0)
Kiribati*	2007	243·5 (228·0 to 259·9)	142·0 (130·0 to 155·3)	43·9 (34·6 to 55·0)	0·0 (0·0 to 0·0)	57·6 (57·6 to 57·6)	316·0 (295·9 to 337·3)	184·3 (168·8 to 201·6)	56·9 (45·0 to 71·4)	0·0 (0·0 to 0·0)	74·7 (74·7 to 74·7)
Kiribati*	2008	295·0 (279·0 to 312·5)	139·2 (127·0 to 152·7)	47·3 (37·0 to 59·4)	8·7 (6·4 to 11·8)	99·8 (99·8 to 99·8)	382·9 (362·1 to 405·6)	180·7 (164·9 to 198·2)	61·4 (48·0 to 77·1)	11·3 (8·3 to 15·4)	129·5 (129·5 to 129·5)
Kiribati*	2009	244·8 (228·4 to 261·5)	128·3 (116·2 to 140·3)	45·6 (35·7 to 57·3)	8·4 (6·2 to 11·5)	62·6 (62·6 to 62·6)	317·8 (296·5 to 339·4)	166·5 (150·9 to 182·1)	59·1 (46·4 to 74·3)	10·9 (8·0 to 15·0)	81·2 (81·2 to 81·2)
Kiribati*	2010	241·1 (226·4 to 257·0)	116·7 (105·4 to 128·4)	43·7 (34·2 to 54·9)	8·0 (5·9 to 10·9)	72·7 (72·7 to 72·7)	312·9 (293·9 to 333·6)	151·4 (136·8 to 166·6)	56·7 (44·4 to 71·3)	10·4 (7·7 to 14·2)	94·4 (94·4 to 94·4)
Kiribati*	2011	229·0 (213·9 to 245·5)	114·1 (103·0 to 125·6)	43·2 (33·8 to 54·5)	7·9 (5·7 to 10·7)	63·8 (63·8 to 63·8)	297·2 (277·6 to 318·7)	148·1 (133·7 to 163·0)	56·0 (43·8 to 70·7)	10·2 (7·4 to 13·9)	82·8 (82·8 to 82·8)
Kiribati*	2012	215·4 (200·5 to 231·6)	114·4 (103·0 to 126·0)	43·1 (33·5 to 54·5)	7·8 (5·5 to 10·6)	50·1 (50·1 to 50·1)	279·5 (260·2 to 300·6)	148·5 (133·7 to 163·6)	55·9 (43·4 to 70·7)	10·1 (7·2 to 13·7)	65·0 (65·0 to 65·0)
Kiribati*	2013	200·5 (186·1 to 216·8)	109·0 (98·1 to 120·3)	42·1 (32·4 to 53·1)	7·5 (5·3 to 10·0)	41·9 (41·9 to 41·9)	260·2 (241·5 to 281·4)	141·5 (127·3 to 156·1)	54·6 (42·0 to 68·9)	9·7 (6·9 to 12·9)	54·4 (54·4 to 54·4)
Kiribati*	2014	188·2 (172·2 to 205·3)	119·2 (107·8 to 131·5)	43·5 (33·6 to 55·0)	7·4 (5·2 to 10·0)	18·0 (18·0 to 18·0)	244·2 (223·5 to 266·4)	154·8 (140·0 to 170·7)	56·5 (43·6 to 71·4)	9·6 (6·8 to 13·0)	23·3 (23·3 to 23·3)
Kiribati*	2015	183·2 (168·1 to 199·5)	114·6 (103·5 to 126·6)	42·6 (33·1 to 54·2)	7·0 (4·9 to 9·5)	19·0 (19·0 to 19·0)	237·7 (218·2 to 259·0)	148·7 (134·3 to 164·3)	55·3 (43·0 to 70·3)	9·1 (6·3 to 12·3)	24·7 (24·7 to 24·7)
Kiribati*	2016	212·3 (196·2 to 230·4)	126·3 (113·7 to 139·4)	44·1 (34·2 to 56·2)	7·1 (5·0 to 9·7)	34·8 (34·8 to 34·8)	275·6 (254·6 to 299·1)	163·9 (147·5 to 181·0)	57·2 (44·4 to 72·9)	9·2 (6·5 to 12·5)	45·2 (45·2 to 45·2)
Kiribati*	2017	219·1 (202·6 to 237·6)	128·3 (115·6 to 142·7)	43·8 (34·3 to 55·5)	7·0 (4·9 to 9·7)	40·0 (40·0 to 40·0)	284·4 (262·9 to 308·4)	166·6 (150·0 to 185·2)	56·9 (44·5 to 72·0)	9·1 (6·4 to 12·5)	51·9 (51·9 to 51·9)
Kiribati*	2018	224·4 (204·1 to 245·7)	144·6 (129·2 to 161·8)	45·8 (36·3 to 58·1)	7·8 (5·5 to 10·7)	26·2 (26·2 to 26·2)	291·2 (264·9 to 318·9)	187·6 (167·7 to 210·0)	59·5 (47·0 to 75·4)	10·1 (7·1 to 13·9)	34·0 (34·0 to 34·0)
Kiribati*	2019	199·9 (182·0 to 218·7)	118·6 (105·7 to 132·3)	46·0 (36·4 to 58·3)	8·0 (5·6 to 11·1)	27·4 (27·3 to 27·8)	259·5 (236·2 to 283·9)	153·9 (137·1 to 171·7)	59·7 (47·2 to 75·6)	10·4 (7·3 to 14·4)	35·6 (35·4 to 36·1)

Kiribati*	2020	202·0 (183·6 to 220·6)	116·5 (103·3 to 131·1)	45·4 (35·9 to 57·5)	8·1 (5·7 to 11·3)	32·1 (31·4 to 33·2)	262·2 (238·3 to 286·4)	151·1 (134·1 to 170·1)	58·9 (46·6 to 74·7)	10·5 (7·4 to 14·7)	41·6 (40·7 to 43·1)
Kiribati*	2021	197·7 (178·3 to 217·1)	116·4 (102·2 to 131·2)	45·5 (36·0 to 57·6)	8·3 (5·8 to 11·5)	27·5 (26·5 to 28·2)	256·6 (231·4 to 281·7)	151·1 (132·7 to 170·3)	59·0 (46·7 to 74·8)	10·7 (7·6 to 15·0)	35·7 (34·4 to 36·6)
Kiribati*	2022	186·5 (166·4 to 208·3)	110·8 (94·9 to 129·5)	45·2 (35·8 to 57·3)	8·4 (5·9 to 11·7)	22·1 (20·7 to 23·5)	242·1 (215·9 to 270·4)	143·7 (123·2 to 168·1)	58·7 (46·5 to 74·4)	10·9 (7·7 to 15·2)	28·7 (26·9 to 30·5)
Kiribati*	2023	185·6 (164·2 to 208·7)	108·6 (92·4 to 128·5)	45·0 (35·6 to 57·1)	8·6 (6·0 to 11·9)	23·5 (21·9 to 25·1)	240·9 (213·1 to 270·9)	140·9 (119·9 to 166·8)	58·4 (46·3 to 74·1)	11·1 (7·8 to 15·5)	30·5 (28·4 to 32·6)
Kiribati*	2024	185·9 (164·6 to 210·3)	107·5 (91·4 to 127·4)	44·8 (35·5 to 56·8)	8·7 (6·1 to 12·1)	24·9 (23·1 to 27·1)	241·3 (213·6 to 272·9)	139·5 (118·6 to 165·3)	58·1 (46·0 to 73·7)	11·3 (8·0 to 15·7)	32·3 (30·0 to 35·2)
Kiribati*	2025	186·4 (163·8 to 209·8)	106·6 (89·0 to 126·8)	44·6 (35·3 to 56·5)	8·9 (6·2 to 12·4)	26·3 (24·3 to 29·3)	242·0 (212·6 to 272·3)	138·4 (115·6 to 164·5)	57·9 (45·8 to 73·4)	11·5 (8·1 to 16·0)	34·2 (31·6 to 38·0)
Kiribati*	2026	187·2 (164·6 to 212·3)	106·0 (88·0 to 126·6)	44·4 (35·2 to 56·4)	9·1 (6·3 to 12·7)	27·7 (25·4 to 31·3)	242·9 (213·7 to 275·6)	137·6 (114·2 to 164·3)	57·7 (45·7 to 73·2)	11·8 (8·2 to 16·4)	35·9 (33·0 to 40·7)
Kiribati*	2027	188·1 (165·5 to 214·0)	105·6 (86·8 to 129·1)	44·4 (35·1 to 56·3)	9·3 (6·5 to 13·0)	28·9 (26·4 to 33·2)	244·1 (214·9 to 277·8)	137·1 (112·7 to 167·6)	57·6 (45·6 to 73·1)	12·0 (8·4 to 16·8)	37·4 (34·3 to 43·1)
Kiribati*	2028	189·4 (165·8 to 216·2)	105·5 (85·7 to 128·7)	44·3 (35·1 to 56·2)	9·5 (6·6 to 13·2)	30·0 (27·3 to 35·1)	245·8 (215·2 to 280·7)	136·9 (111·3 to 167·0)	57·5 (45·5 to 73·0)	12·3 (8·6 to 17·1)	39·0 (35·4 to 45·6)
Kiribati*	2029	191·0 (166·5 to 218·6)	105·7 (85·8 to 130·4)	44·3 (35·1 to 56·2)	9·7 (6·8 to 13·5)	31·2 (28·2 to 37·2)	247·8 (216·1 to 283·8)	137·2 (111·3 to 169·3)	57·5 (45·6 to 72·9)	12·6 (8·8 to 17·5)	40·5 (36·6 to 48·3)
Kiribati*	2030	193·5 (167·9 to 221·6)	107·0 (85·6 to 133·4)	44·4 (35·2 to 56·2)	9·9 (6·9 to 13·8)	32·2 (28·9 to 39·3)	251·1 (217·9 to 287·6)	138·8 (111·1 to 173·1)	57·6 (45·6 to 73·0)	12·9 (9·0 to 17·9)	41·8 (37·5 to 51·0)
Kiribati*	2031	196·0 (169·2 to 225·8)	108·2 (86·4 to 134·3)	44·4 (35·2 to 56·4)	10·2 (7·1 to 14·2)	33·2 (29·7 to 41·0)	254·4 (219·6 to 293·1)	140·5 (112·2 to 174·3)	57·7 (45·7 to 73·2)	13·2 (9·2 to 18·4)	43·1 (38·5 to 53·2)
Kiribati*	2032	198·5 (171·1 to 229·4)	109·4 (86·8 to 136·5)	44·5 (35·3 to 56·4)	10·5 (7·3 to 14·5)	34·2 (30·3 to 43·1)	257·7 (222·1 to 297·7)	142·0 (112·7 to 177·2)	57·8 (45·8 to 73·2)	13·6 (9·5 to 18·8)	44·4 (39·3 to 55·9)
Kiribati*	2033	200·8 (171·7 to 233·1)	110·4 (86·9 to 139·1)	44·7 (35·4 to 56·6)	10·7 (7·5 to 14·9)	35·0 (30·9 to 44·7)	260·6 (222·8 to 302·6)	143·3 (112·8 to 180·6)	58·0 (46·0 to 73·5)	13·9 (9·7 to 19·3)	45·4 (40·1 to 58·0)

Kiribati*	2034	203·0 (173·3 to 235·8)	111·3 (87·0 to 140·2)	44·8 (35·6 to 56·8)	11·0 (7·7 to 15·2)	35·8 (31·5 to 46·5)	263·5 (224·9 to 306·0)	144·5 (112·9 to 181·9)	58·2 (46·2 to 73·7)	14·3 (10·0 to 19·8)	46·5 (40·8 to 60·4)
Kiribati*	2035	205·1 (173·8 to 238·1)	112·1 (87·2 to 141·1)	45·0 (35·7 to 57·0)	11·4 (7·9 to 15·6)	36·6 (31·9 to 48·0)	266·2 (225·6 to 309·0)	145·5 (113·2 to 183·2)	58·4 (46·4 to 74·0)	14·7 (10·3 to 20·3)	47·5 (41·4 to 62·3)
Kiribati*	2036	207·2 (176·1 to 240·8)	112·8 (87·8 to 143·4)	45·1 (35·8 to 57·1)	11·7 (8·1 to 16·0)	37·6 (32·6 to 50·5)	268·9 (228·6 to 312·6)	146·4 (114·0 to 186·1)	58·6 (46·5 to 74·2)	15·1 (10·5 to 20·7)	48·8 (42·3 to 65·5)
Kiribati*	2037	209·4 (178·5 to 244·4)	113·6 (88·1 to 145·3)	45·3 (36·0 to 57·3)	12·0 (8·3 to 16·4)	38·6 (33·5 to 52·2)	271·8 (231·6 to 317·3)	147·4 (114·3 to 188·5)	58·8 (46·7 to 74·4)	15·6 (10·8 to 21·3)	50·1 (43·5 to 67·8)
Kiribati*	2038	211·9 (179·0 to 249·1)	114·5 (87·9 to 146·5)	45·5 (36·1 to 57·5)	12·3 (8·5 to 16·9)	39·6 (34·0 to 54·3)	275·0 (232·4 to 323·3)	148·6 (114·1 to 190·2)	59·0 (46·9 to 74·6)	16·0 (11·0 to 21·9)	51·3 (44·2 to 70·5)
Kiribati*	2039	214·6 (181·9 to 252·8)	115·7 (87·8 to 148·5)	45·7 (36·3 to 57·7)	12·7 (8·7 to 17·3)	40·6 (34·7 to 56·6)	278·5 (236·1 to 328·0)	150·2 (114·0 to 192·8)	59·3 (47·1 to 74·9)	16·5 (11·3 to 22·5)	52·6 (45·0 to 73·4)
Kiribati*	2040	217·4 (183·3 to 257·2)	116·8 (89·3 to 150·3)	45·8 (36·4 to 57·9)	13·0 (8·9 to 17·9)	41·7 (35·3 to 58·9)	282·2 (237·9 to 333·8)	151·6 (116·0 to 195·1)	59·5 (47·3 to 75·2)	16·9 (11·6 to 23·3)	54·1 (45·8 to 76·4)
Kiribati*	2041	220·5 (185·8 to 261·0)	118·2 (89·6 to 152·3)	46·1 (36·6 to 58·1)	13·4 (9·2 to 18·5)	42·8 (36·1 to 60·8)	286·2 (241·1 to 338·7)	153·5 (116·3 to 197·7)	59·8 (47·5 to 75·5)	17·4 (11·9 to 24·0)	55·6 (46·9 to 78·9)
Kiribati*	2042	223·9 (187·1 to 266·5)	119·8 (89·7 to 154·9)	46·3 (36·8 to 58·4)	13·8 (9·4 to 19·1)	44·1 (36·8 to 64·1)	290·6 (242·8 to 345·9)	155·5 (116·4 to 201·0)	60·0 (47·7 to 75·8)	17·9 (12·2 to 24·8)	57·2 (47·8 to 83·1)
Kiribati*	2043	227·2 (189·8 to 271·5)	121·0 (90·4 to 161·4)	46·4 (36·9 to 58·6)	14·2 (9·6 to 19·7)	45·6 (37·7 to 67·0)	294·9 (246·4 to 352·3)	157·1 (117·3 to 209·5)	60·2 (47·9 to 76·0)	18·4 (12·5 to 25·6)	59·2 (49·0 to 87·0)
Kiribati*	2044	230·8 (192·4 to 278·2)	122·4 (91·3 to 163·6)	46·5 (37·0 to 58·8)	14·5 (9·8 to 20·3)	47·3 (39·0 to 70·7)	299·5 (249·7 to 361·1)	158·9 (118·4 to 212·4)	60·4 (48·0 to 76·3)	18·9 (12·7 to 26·3)	61·4 (50·6 to 91·7)
Kiribati*	2045	234·5 (195·2 to 282·7)	123·8 (90·3 to 164·8)	46·6 (37·1 to 58·9)	14·9 (10·0 to 21·0)	49·1 (40·0 to 74·3)	304·3 (253·3 to 366·9)	160·7 (117·2 to 213·9)	60·5 (48·1 to 76·5)	19·4 (13·0 to 27·2)	63·7 (52·0 to 96·4)
Kiribati*	2046	238·3 (197·6 to 289·9)	125·2 (91·3 to 168·5)	46·7 (37·2 to 59·1)	15·3 (10·3 to 21·7)	51·1 (41·3 to 78·5)	309·3 (256·5 to 376·2)	162·5 (118·4 to 218·7)	60·7 (48·2 to 76·7)	19·9 (13·3 to 28·1)	66·3 (53·6 to 101·9)
Kiribati*	2047	242·3 (200·1 to 293·9)	126·7 (93·6 to 171·4)	46·8 (37·3 to 59·3)	15·7 (10·5 to 22·3)	53·1 (42·4 to 81·6)	314·5 (259·6 to 381·5)	164·4 (121·5 to 222·5)	60·8 (48·4 to 76·9)	20·4 (13·6 to 29·0)	68·9 (55·0 to 105·9)

Kiribati*	2048	246·3 (203·7 to 303·6)	127·9 (93·8 to 175·3)	46·9 (37·4 to 59·4)	16·1 (10·7 to 22·9)	55·3 (44·2 to 87·2)	319·7 (264·4 to 394·0)	166·1 (121·7 to 227·5)	60·9 (48·5 to 77·1)	20·9 (13·9 to 29·7)	71·8 (57·3 to 113·2)
Kiribati*	2049	250·4 (206·0 to 309·0)	129·2 (94·0 to 177·1)	47·0 (37·5 to 59·6)	16·5 (10·9 to 23·6)	57·5 (45·1 to 91·2)	324·9 (267·3 to 401·0)	167·7 (121·9 to 229·8)	61·1 (48·6 to 77·3)	21·5 (14·1 to 30·6)	74·7 (58·6 to 118·3)
Kiribati*	2050	254·9 (209·3 to 316·9)	130·5 (94·3 to 180·5)	47·1 (37·5 to 59·7)	17·0 (11·1 to 24·3)	60·3 (47·1 to 97·4)	330·8 (271·6 to 411·2)	169·4 (122·4 to 234·3)	61·1 (48·6 to 77·4)	22·0 (14·4 to 31·5)	78·3 (61·2 to 126·5)
Saint Kitts and Nevis	1995	704·0 (619·5 to 796·2)	263·9 (217·3 to 317·1)	363·5 (294·1 to 447·2)	47·9 (33·0 to 66·1)	28·8 (28·8 to 28·8)	970·5 (854·0 to 1097·6)	363·7 (299·6 to 437·2)	501·1 (405·4 to 616·5)	66·0 (45·5 to 91·1)	39·7 (39·7 to 39·7)
Saint Kitts and Nevis	1996	693·6 (608·4 to 782·3)	279·4 (232·7 to 332·8)	368·9 (301·0 to 449·3)	45·3 (31·6 to 62·9)	0·0 (0·0 to 0·0)	956·1 (838·7 to 1078·5)	385·2 (320·8 to 458·8)	508·5 (414·9 to 619·4)	62·4 (43·6 to 86·7)	0·0 (0·0 to 0·0)
Saint Kitts and Nevis	1997	686·3 (610·0 to 767·2)	278·2 (236·6 to 324·9)	366·7 (300·3 to 435·9)	41·3 (28·9 to 56·7)	0·0 (0·0 to 0·0)	946·1 (840·9 to 1057·6)	383·6 (326·2 to 447·9)	505·5 (414·0 to 600·9)	57·0 (39·9 to 78·2)	0·0 (0·0 to 0·0)
Saint Kitts and Nevis	1998	692·3 (620·5 to 761·8)	283·9 (248·8 to 322·7)	368·9 (309·1 to 432·4)	39·6 (28·2 to 53·1)	0·0 (0·0 to 0·0)	954·4 (855·4 to 1050·2)	391·3 (343·0 to 444·8)	508·5 (426·1 to 596·1)	54·6 (38·8 to 73·1)	0·0 (0·0 to 0·0)
Saint Kitts and Nevis	1999	770·4 (708·1 to 832·1)	308·1 (277·4 to 344·3)	381·2 (328·9 to 433·3)	40·5 (29·6 to 53·2)	40·5 (40·5 to 40·5)	1062·0 (976·2 to 1147·1)	424·7 (382·4 to 474·6)	525·5 (453·5 to 597·4)	55·8 (40·8 to 73·3)	55·9 (55·9 to 55·9)
Saint Kitts and Nevis	2000	769·6 (715·8 to 820·8)	320·1 (294·2 to 349·0)	385·9 (343·6 to 429·2)	40·8 (30·7 to 52·9)	22·8 (22·8 to 22·8)	1060·9 (986·7 to 1131·5)	441·2 (405·6 to 481·2)	531·9 (473·7 to 591·6)	56·2 (42·4 to 72·9)	31·5 (31·5 to 31·5)
Saint Kitts and Nevis	2001	713·3 (671·1 to 755·8)	302·0 (280·9 to 325·0)	365·1 (331·5 to 401·3)	38·1 (29·3 to 48·9)	8·1 (8·1 to 8·1)	983·3 (925·2 to 1041·9)	416·3 (387·2 to 448·0)	503·2 (456·9 to 553·2)	52·5 (40·4 to 67·5)	11·2 (11·2 to 11·2)
Saint Kitts and Nevis	2002	747·8 (705·5 to 788·9)	332·5 (310·5 to 356·1)	371·7 (338·9 to 404·4)	38·5 (29·7 to 48·9)	5·1 (5·1 to 5·1)	1030·9 (972·6 to 1087·6)	458·4 (428·0 to 490·8)	512·4 (467·1 to 557·6)	53·0 (41·0 to 67·5)	7·1 (7·1 to 7·1)
Saint Kitts and Nevis	2003	693·8 (655·2 to 732·6)	303·8 (284·9 to 323·9)	349·4 (317·5 to 379·8)	35·4 (27·0 to 44·6)	5·1 (5·1 to 5·1)	956·4 (903·2 to 1009·9)	418·9 (392·7 to 446·6)	481·7 (437·6 to 523·6)	48·8 (37·3 to 61·4)	7·0 (7·0 to 7·0)
Saint Kitts and Nevis	2004	731·2 (693·5 to 772·2)	334·2 (314·2 to 355·8)	357·6 (326·7 to 391·0)	36·0 (28·2 to 44·7)	3·5 (3·5 to 3·5)	1007·9 (956·0 to 1064·5)	460·7 (433·1 to 490·5)	492·9 (450·4 to 539·0)	49·6 (38·9 to 61·7)	4·8 (4·8 to 4·8)
Saint Kitts and Nevis	2005	806·7 (764·9 to 850·1)	371·7 (348·6 to 396·2)	394·3 (361·7 to 429·4)	38·5 (30·7 to 47·6)	2·2 (2·2 to 2·2)	1112·1 (1054·4 to 1171·9)	512·4 (480·5 to 546·1)	543·5 (498·6 to 591·9)	53·1 (42·3 to 65·6)	3·1 (3·1 to 3·1)

Saint Kitts and Nevis	2006	838·2 (798·8 to 879·7)	365·9 (343·9 to 389·5)	422·9 (390·4 to 457·7)	41·8 (33·7 to 51·2)	7·6 (7·6 to 7·6)	1155·5 (1101·1 to 1212·7)	504·4 (474·1 to 537·0)	583·0 (538·2 to 630·9)	57·6 (46·5 to 70·6)	10·5 (10·5 to 10·5)
Saint Kitts and Nevis	2007	876·6 (837·4 to 915·9)	349·7 (328·6 to 371·6)	451·9 (417·0 to 485·6)	46·2 (37·7 to 55·5)	28·8 (28·8 to 28·8)	1208·5 (1154·4 to 1262·6)	482·1 (452·9 to 512·2)	623·0 (574·9 to 669·4)	63·7 (51·9 to 76·5)	39·7 (39·7 to 39·7)
Saint Kitts and Nevis	2008	912·5 (872·4 to 954·4)	341·7 (321·1 to 364·3)	496·4 (460·4 to 532·9)	50·8 (42·1 to 60·3)	23·6 (23·6 to 23·6)	1257·9 (1202·6 to 1315·6)	471·0 (442·6 to 502·2)	684·3 (634·7 to 734·7)	70·1 (58·1 to 83·2)	32·6 (32·6 to 32·6)
Saint Kitts and Nevis	2009	906·8 (864·8 to 949·7)	336·8 (315·8 to 359·3)	505·1 (470·5 to 539·6)	51·7 (44·5 to 59·4)	13·2 (13·2 to 13·2)	1250·1 (1192·2 to 1309·3)	464·3 (435·4 to 495·3)	696·3 (648·6 to 743·9)	71·3 (61·3 to 81·9)	18·2 (18·2 to 18·2)
Saint Kitts and Nevis	2010	901·7 (858·7 to 941·2)	339·5 (317·9 to 362·6)	504·6 (469·7 to 539·5)	51·7 (45·9 to 57·7)	5·9 (5·9 to 5·9)	1243·0 (1183·7 to 1297·5)	468·0 (438·2 to 499·8)	695·6 (647·5 to 743·7)	71·3 (63·3 to 79·5)	8·1 (8·1 to 8·1)
Saint Kitts and Nevis	2011	869·5 (829·7 to 910·8)	327·7 (307·7 to 347·5)	490·9 (457·4 to 525·2)	50·9 (46·8 to 55·4)	0·0 (0·0 to 0·0)	1198·6 (1143·8 to 1255·6)	451·8 (424·2 to 479·1)	676·7 (630·5 to 724·0)	70·2 (64·5 to 76·3)	0·0 (0·0 to 0·0)
Saint Kitts and Nevis	2012	829·4 (792·9 to 869·5)	315·2 (297·5 to 332·9)	468·2 (436·7 to 502·8)	45·9 (42·7 to 49·3)	0·0 (0·0 to 0·0)	1143·3 (1093·1 to 1198·6)	434·6 (410·1 to 458·9)	645·4 (602·0 to 693·2)	63·3 (58·8 to 68·0)	0·0 (0·0 to 0·0)
Saint Kitts and Nevis	2013	861·1 (822·7 to 901·9)	344·1 (327·2 to 361·8)	471·6 (438·2 to 507·3)	45·5 (42·4 to 49·0)	0·0 (0·0 to 0·0)	1187·1 (1134·1 to 1243·3)	474·3 (451·0 to 498·8)	650·1 (604·1 to 699·3)	62·7 (58·4 to 67·6)	0·0 (0·0 to 0·0)
Saint Kitts and Nevis	2014	863·5 (824·9 to 903·1)	361·7 (343·8 to 380·5)	456·3 (422·9 to 491·4)	45·5 (42·3 to 48·9)	0·0 (0·0 to 0·0)	1190·4 (1137·2 to 1245·0)	498·7 (473·9 to 524·6)	629·0 (583·0 to 677·5)	62·7 (58·3 to 67·5)	0·0 (0·0 to 0·0)
Saint Kitts and Nevis	2015	893·4 (853·6 to 933·5)	376·8 (358·6 to 395·0)	468·3 (434·4 to 503·9)	48·2 (45·1 to 51·7)	0·0 (0·0 to 0·0)	1231·6 (1176·7 to 1286·9)	519·5 (494·3 to 544·5)	645·6 (598·8 to 694·6)	66·5 (62·2 to 71·3)	0·0 (0·0 to 0·0)
Saint Kitts and Nevis	2016	911·5 (870·4 to 952·7)	384·1 (366·5 to 402·2)	478·4 (443·2 to 513·5)	49·0 (45·8 to 52·4)	0·0 (0·0 to 0·0)	1256·6 (1199·9 to 1313·4)	529·5 (505·2 to 554·4)	659·5 (611·0 to 707·9)	67·5 (63·1 to 72·2)	0·0 (0·0 to 0·0)
Saint Kitts and Nevis	2017	920·7 (880·4 to 963·5)	395·1 (375·6 to 413·9)	476·1 (440·1 to 515·0)	49·5 (46·2 to 53·0)	0·0 (0·0 to 0·0)	1269·2 (1213·7 to 1328·3)	544·6 (517·7 to 570·6)	656·3 (606·6 to 710·0)	68·3 (63·7 to 73·1)	0·0 (0·0 to 0·0)
Saint Kitts and Nevis	2018	988·2 (932·8 to 1042·9)	452·9 (424·4 to 481·4)	485·6 (439·8 to 532·5)	49·7 (45·0 to 54·6)	0·0 (0·0 to 0·0)	1362·3 (1286·0 to 1437·7)	624·4 (585·1 to 663·6)	669·4 (606·3 to 734·1)	68·5 (62·1 to 75·2)	0·0 (0·0 to 0·0)
Saint Kitts and Nevis	2019	1026·2 (966·6 to 1086·2)	480·7 (447·6 to 517·3)	493·2 (446·5 to 541·1)	52·3 (47·2 to 57·6)	0·0 (0·0 to 0·0)	1414·7 (1332·5 to 1497·4)	662·7 (617·0 to 713·2)	679·9 (615·5 to 745·9)	72·0 (65·1 to 79·4)	0·0 (0·0 to 0·0)

Saint Kitts and Nevis	2020	1007·4 (947·1 to 1068·3)	495·4 (456·7 to 538·3)	461·9 (418·2 to 506·8)	50·1 (45·2 to 55·1)	0·0 (0·0 to 0·0)	1388·7 (1305·6 to 1472·7)	682·9 (629·6 to 742·1)	636·8 (576·6 to 698·7)	69·0 (62·3 to 76·0)	0·0 (0·0 to 0·0)
Saint Kitts and Nevis	2021	968·7 (910·5 to 1027·2)	476·4 (439·2 to 517·7)	443·2 (401·1 to 486·3)	49·1 (44·3 to 54·2)	0·0 (0·0 to 0·0)	1335·4 (1255·2 to 1416·0)	656·7 (605·4 to 713·6)	610·9 (552·9 to 670·4)	67·8 (61·1 to 74·7)	0·0 (0·0 to 0·0)
Saint Kitts and Nevis	2022	939·6 (876·8 to 1003·0)	439·1 (396·2 to 486·3)	449·4 (406·8 to 493·1)	51·0 (46·0 to 56·2)	0·0 (0·0 to 0·0)	1295·2 (1208·7 to 1382·6)	605·3 (546·1 to 670·4)	619·6 (560·8 to 679·8)	70·3 (63·4 to 77·5)	0·0 (0·0 to 0·0)
Saint Kitts and Nevis	2023	953·1 (886·0 to 1023·8)	444·4 (397·8 to 500·7)	455·7 (412·7 to 499·9)	53·0 (47·7 to 58·4)	0·0 (0·0 to 0·0)	1313·8 (1221·3 to 1411·4)	612·6 (548·4 to 690·2)	628·2 (568·9 to 689·1)	73·0 (65·8 to 80·5)	0·0 (0·0 to 0·0)
Saint Kitts and Nevis	2024	970·0 (900·9 to 1042·2)	452·9 (402·7 to 510·8)	462·1 (418·7 to 506·9)	55·0 (49·5 to 60·7)	0·0 (0·0 to 0·0)	1337·2 (1241·9 to 1436·7)	624·3 (555·1 to 704·2)	637·1 (577·2 to 698·9)	75·8 (68·2 to 83·7)	0·0 (0·0 to 0·0)
Saint Kitts and Nevis	2025	986·1 (912·3 to 1061·1)	460·5 (406·6 to 522·3)	468·5 (424·0 to 513·8)	57·1 (51·5 to 62·9)	0·0 (0·0 to 0·0)	1359·5 (1257·6 to 1462·8)	634·9 (560·4 to 720·0)	645·9 (584·5 to 708·3)	78·7 (71·0 to 86·7)	0·0 (0·0 to 0·0)
Saint Kitts and Nevis	2026	999·0 (922·7 to 1077·0)	466·2 (409·1 to 533·5)	473·7 (429·0 to 519·3)	59·1 (53·1 to 65·1)	0·0 (0·0 to 0·0)	1377·2 (1272·0 to 1484·7)	642·7 (564·0 to 735·4)	653·1 (591·3 to 715·8)	81·4 (73·1 to 89·7)	0·0 (0·0 to 0·0)
Saint Kitts and Nevis	2027	1013·0 (933·9 to 1094·0)	472·9 (411·9 to 540·5)	478·9 (433·7 to 524·8)	61·1 (54·9 to 67·4)	0·0 (0·0 to 0·0)	1396·4 (1287·4 to 1508·1)	651·9 (567·8 to 745·1)	660·2 (597·9 to 723·5)	84·3 (75·7 to 92·9)	0·0 (0·0 to 0·0)
Saint Kitts and Nevis	2028	1027·9 (945·7 to 1112·8)	480·4 (413·8 to 553·7)	484·3 (438·6 to 530·6)	63·3 (56·7 to 69·7)	0·0 (0·0 to 0·0)	1417·0 (1303·7 to 1534·0)	662·2 (570·4 to 763·4)	667·6 (604·7 to 731·4)	87·2 (78·2 to 96·1)	0·0 (0·0 to 0·0)
Saint Kitts and Nevis	2029	1042·3 (957·0 to 1131·0)	487·7 (416·7 to 561·3)	489·3 (443·0 to 536·0)	65·4 (58·7 to 72·2)	0·0 (0·0 to 0·0)	1436·9 (1319·2 to 1559·1)	672·3 (574·5 to 773·8)	674·5 (610·7 to 738·8)	90·2 (81·0 to 99·5)	0·0 (0·0 to 0·0)
Saint Kitts and Nevis	2030	1057·9 (968·4 to 1149·5)	495·6 (424·7 to 573·3)	494·6 (447·8 to 541·9)	67·7 (60·4 to 74·8)	0·0 (0·0 to 0·0)	1458·3 (1335·0 to 1584·6)	683·1 (585·5 to 790·3)	681·9 (617·3 to 747·0)	93·3 (83·3 to 103·2)	0·0 (0·0 to 0·0)
Saint Kitts and Nevis	2031	1073·0 (982·8 to 1169·0)	503·1 (427·6 to 581·8)	499·9 (452·4 to 547·8)	70·0 (62·3 to 77·7)	0·0 (0·0 to 0·0)	1479·2 (1354·8 to 1611·5)	693·5 (589·4 to 802·1)	689·2 (623·7 to 755·2)	96·5 (85·9 to 107·1)	0·0 (0·0 to 0·0)
Saint Kitts and Nevis	2032	1089·4 (997·0 to 1189·9)	511·8 (434·5 to 594·4)	505·2 (457·0 to 553·9)	72·4 (64·3 to 80·7)	0·0 (0·0 to 0·0)	1501·7 (1374·4 to 1640·4)	705·5 (599·0 to 819·4)	696·4 (629·9 to 763·6)	99·8 (88·7 to 111·2)	0·0 (0·0 to 0·0)
Saint Kitts and Nevis	2033	1107·4 (1010·9 to 1211·6)	522·1 (445·2 to 610·6)	510·4 (461·5 to 559·4)	74·9 (66·2 to 83·8)	0·0 (0·0 to 0·0)	1526·7 (1393·6 to 1670·3)	719·7 (613·7 to 841·8)	703·7 (636·3 to 771·2)	103·3 (91·3 to 115·5)	0·0 (0·0 to 0·0)

Saint Kitts and Nevis	2034	1124·7 (1025·5 to 1234·4)	531·7 (450·0 to 626·1)	515·6 (466·0 to 565·1)	77·4 (68·0 to 87·0)	0·0 (0·0 to 0·0)	1550·5 (1413·7 to 1701·6)	733·0 (620·4 to 863·2)	710·7 (642·4 to 779·1)	106·8 (93·8 to 120·0)	0·0 (0·0 to 0·0)
Saint Kitts and Nevis	2035	1143·4 (1041·2 to 1257·2)	542·5 (456·6 to 642·7)	520·8 (470·7 to 571·0)	80·1 (70·0 to 90·4)	0·0 (0·0 to 0·0)	1576·2 (1435·4 to 1733·0)	747·9 (629·4 to 886·1)	717·9 (648·9 to 787·2)	110·4 (96·5 to 124·6)	0·0 (0·0 to 0·0)
Saint Kitts and Nevis	2036	1162·5 (1055·4 to 1283·2)	553·5 (463·7 to 654·5)	526·2 (476·0 to 576·9)	82·8 (72·0 to 94·1)	0·0 (0·0 to 0·0)	1602·6 (1455·0 to 1769·0)	763·0 (639·3 to 902·3)	725·5 (656·2 to 795·2)	114·2 (99·2 to 129·8)	0·0 (0·0 to 0·0)
Saint Kitts and Nevis	2037	1182·0 (1071·6 to 1303·0)	564·6 (473·2 to 673·1)	531·8 (481·2 to 582·9)	85·7 (74·0 to 98·0)	0·0 (0·0 to 0·0)	1629·5 (1477·3 to 1796·2)	778·3 (652·3 to 927·8)	733·1 (663·3 to 803·6)	118·1 (102·0 to 135·1)	0·0 (0·0 to 0·0)
Saint Kitts and Nevis	2038	1201·7 (1086·4 to 1325·6)	575·9 (479·1 to 685·6)	537·3 (486·0 to 588·9)	88·6 (76·0 to 101·8)	0·0 (0·0 to 0·0)	1656·6 (1497·6 to 1827·5)	793·9 (660·4 to 945·2)	740·6 (669·9 to 811·8)	122·1 (104·7 to 140·4)	0·0 (0·0 to 0·0)
Saint Kitts and Nevis	2039	1222·6 (1104·3 to 1350·7)	588·0 (489·5 to 704·1)	542·9 (490·9 to 594·8)	91·6 (77·8 to 105·9)	0·0 (0·0 to 0·0)	1685·4 (1522·3 to 1862·1)	810·6 (674·9 to 970·6)	748·4 (676·7 to 820·0)	126·3 (107·3 to 146·0)	0·0 (0·0 to 0·0)
Saint Kitts and Nevis	2040	1242·6 (1121·6 to 1377·3)	599·5 (492·5 to 723·3)	548·3 (495·7 to 600·8)	94·7 (79·9 to 110·2)	0·0 (0·0 to 0·0)	1712·9 (1546·2 to 1898·7)	826·4 (679·0 to 997·1)	755·9 (683·3 to 828·2)	130·6 (110·1 to 151·9)	0·0 (0·0 to 0·0)
Saint Kitts and Nevis	2041	1263·3 (1136·6 to 1406·6)	611·3 (501·2 to 745·2)	554·0 (500·7 to 607·2)	98·0 (82·1 to 114·7)	0·0 (0·0 to 0·0)	1741·5 (1566·8 to 1939·0)	842·7 (690·9 to 1027·3)	763·6 (690·2 to 837·1)	135·1 (113·1 to 158·1)	0·0 (0·0 to 0·0)
Saint Kitts and Nevis	2042	1283·0 (1152·4 to 1430·6)	622·7 (502·0 to 758·4)	559·1 (505·3 to 613·1)	101·2 (84·3 to 119·2)	0·0 (0·0 to 0·0)	1768·7 (1588·7 to 1972·2)	858·4 (692·1 to 1045·6)	770·7 (696·6 to 845·2)	139·6 (116·2 to 164·4)	0·0 (0·0 to 0·0)
Saint Kitts and Nevis	2043	1303·2 (1168·0 to 1455·3)	633·9 (508·2 to 776·1)	564·6 (510·2 to 619·2)	104·7 (86·6 to 124·2)	0·0 (0·0 to 0·0)	1796·5 (1610·2 to 2006·2)	873·8 (700·6 to 1070·0)	778·3 (703·3 to 853·6)	144·3 (119·4 to 171·2)	0·0 (0·0 to 0·0)
Saint Kitts and Nevis	2044	1323·8 (1183·7 to 1483·4)	645·7 (520·4 to 795·3)	570·0 (514·7 to 624·8)	108·2 (89·2 to 129·3)	0·0 (0·0 to 0·0)	1825·0 (1631·7 to 2045·0)	890·1 (717·4 to 1096·4)	785·7 (709·6 to 861·3)	149·1 (122·9 to 178·3)	0·0 (0·0 to 0·0)
Saint Kitts and Nevis	2045	1345·6 (1203·0 to 1510·4)	658·2 (526·6 to 808·9)	575·6 (519·6 to 631·0)	111·8 (91·6 to 134·7)	0·0 (0·0 to 0·0)	1855·0 (1658·5 to 2082·1)	907·3 (726·0 to 1115·1)	793·6 (716·3 to 869·8)	154·2 (126·2 to 185·8)	0·0 (0·0 to 0·0)
Saint Kitts and Nevis	2046	1367·1 (1218·2 to 1537·5)	670·5 (531·8 to 823·8)	581·1 (524·3 to 636·7)	115·6 (93·9 to 140·3)	0·0 (0·0 to 0·0)	1884·6 (1679·4 to 2119·5)	924·3 (733·1 to 1135·6)	801·0 (722·8 to 877·7)	159·3 (129·5 to 193·4)	0·0 (0·0 to 0·0)
Saint Kitts and Nevis	2047	1388·9 (1232·7 to 1564·8)	682·7 (541·4 to 845·5)	586·8 (529·4 to 642·8)	119·5 (96·3 to 146·1)	0·0 (0·0 to 0·0)	1914·7 (1699·3 to 2157·2)	941·1 (746·3 to 1165·6)	808·9 (729·7 to 886·1)	164·7 (132·8 to 201·5)	0·0 (0·0 to 0·0)

Saint Kitts and Nevis	2048	1411·3<br>(1251·6 to 1594·3)	695·4<br>(548·3 to 864·2)	592·4<br>(534·5 to 649·4)	123·5<br>(98·8 to 152·2)	0·0<br>(0·0 to 0·0)	1945·6<br>(1725·4 to 2197·8)	958·6<br>(755·9 to 1191·3)	816·7<br>(736·8 to 895·3)	170·3<br>(136·1 to 209·8)	0·0<br>(0·0 to 0·0)
Saint Kitts and Nevis	2049	1433·3<br>(1272·8 to 1619·0)	707·5<br>(558·3 to 882·9)	598·0<br>(539·3 to 656·0)	127·7<br>(101·2 to 158·5)	0·0<br>(0·0 to 0·0)	1975·8<br>(1754·6 to 2231·9)	975·4<br>(769·6 to 1217·1)	824·4<br>(743·4 to 904·4)	176·0<br>(139·5 to 218·5)	0·0<br>(0·0 to 0·0)
Saint Kitts and Nevis	2050	1456·8<br>(1292·6 to 1650·9)	720·9<br>(568·5 to 905·0)	604·0<br>(544·3 to 663·0)	132·0<br>(103·8 to 165·2)	0·0<br>(0·0 to 0·0)	2008·3<br>(1781·9 to 2275·8)	993·7<br>(783·7 to 1247·5)	832·6<br>(750·4 to 913·9)	182·0<br>(143·1 to 227·7)	0·0<br>(0·0 to 0·0)
South Korea	1995	468·5<br>(438·9 to 501·7)	192·4<br>(172·0 to 215·9)	258·1<br>(236·7 to 279·9)	18·0<br>(13·3 to 23·7)	0·0<br>(0·0 to 0·0)	677·1<br>(634·4 to 725·1)	278·0<br>(248·5 to 312·1)	373·1<br>(342·1 to 404·6)	26·0<br>(19·2 to 34·3)	0·0<br>(0·0 to 0·0)
South Korea	1996	508·5<br>(483·2 to 537·4)	220·5<br>(201·3 to 243·0)	267·0<br>(250·3 to 284·5)	20·9<br>(15·9 to 27·0)	0·0<br>(0·0 to 0·0)	734·9<br>(698·5 to 776·7)	318·7<br>(290·9 to 351·2)	385·9<br>(361·8 to 411·2)	30·3<br>(22·9 to 39·0)	0·0<br>(0·0 to 0·0)
South Korea	1997	534·3<br>(510·4 to 560·9)	243·7<br>(224·6 to 264·5)	265·8<br>(249·5 to 281·8)	24·8<br>(19·5 to 30·9)	0·0<br>(0·0 to 0·0)	772·2<br>(737·8 to 810·8)	352·2<br>(324·6 to 382·3)	384·2<br>(360·7 to 407·3)	35·8<br>(28·1 to 44·6)	0·0<br>(0·0 to 0·0)
South Korea	1998	555·8<br>(532·0 to 582·2)	267·0<br>(246·5 to 286·8)	259·4<br>(244·2 to 274·4)	29·4<br>(24·2 to 35·2)	0·0<br>(0·0 to 0·0)	803·3<br>(769·0 to 841·4)	385·9<br>(356·2 to 414·5)	374·9<br>(353·0 to 396·6)	42·5<br>(35·0 to 50·9)	0·0<br>(0·0 to 0·0)
South Korea	1999	615·6<br>(591·8 to 641·8)	310·0<br>(288·1 to 332·0)	272·2<br>(259·4 to 285·0)	33·4<br>(28·8 to 38·6)	0·0<br>(0·0 to 0·0)	889·8<br>(855·3 to 927·7)	448·1<br>(416·4 to 479·8)	393·4<br>(374·9 to 411·9)	48·3<br>(41·6 to 55·8)	0·0<br>(0·0 to 0·0)
South Korea	2000	676·7<br>(653·3 to 700·7)	352·7<br>(330·5 to 375·1)	286·3<br>(276·7 to 296·7)	37·8<br>(34·2 to 41·6)	0·0<br>(0·0 to 0·0)	978·1<br>(944·2 to 1012·8)	509·7<br>(477·7 to 542·1)	413·8<br>(400·0 to 428·8)	54·6<br>(49·5 to 60·1)	0·0<br>(0·0 to 0·0)
South Korea	2001	749·6<br>(726·4 to 773·3)	408·6<br>(386·9 to 430·6)	301·8<br>(292·3 to 310·6)	39·2<br>(36·3 to 42·3)	0·0<br>(0·0 to 0·0)	1083·4<br>(1049·9 to 1117·7)	590·6<br>(559·2 to 622·3)	436·2<br>(422·5 to 448·9)	56·6<br>(52·4 to 61·1)	0·0<br>(0·0 to 0·0)
South Korea	2002	781·5<br>(759·0 to 803·7)	433·6<br>(413·7 to 454·2)	308·2<br>(298·9 to 317·9)	39·7<br>(37·0 to 42·7)	0·0<br>(0·0 to 0·0)	1129·5<br>(1097·0 to 1161·6)	626·7<br>(597·9 to 656·4)	445·4<br>(432·0 to 459·5)	57·3<br>(53·4 to 61·7)	0·0<br>(0·0 to 0·0)
South Korea	2003	875·8<br>(852·0 to 899·5)	500·0<br>(476·9 to 521·2)	331·3<br>(321·5 to 341·3)	44·5<br>(41·6 to 47·5)	0·0<br>(0·0 to 0·0)	1265·8<br>(1231·4 to 1300·1)	722·7<br>(689·3 to 753·3)	478·8<br>(464·7 to 493·3)	64·3<br>(60·2 to 68·7)	0·0<br>(0·0 to 0·0)
South Korea	2004	909·3<br>(885·7 to 932·7)	519·5<br>(496·1 to 541·7)	342·9<br>(333·5 to 352·3)	46·9<br>(44·1 to 49·8)	0·0<br>(0·0 to 0·0)	1314·3<br>(1280·1 to 1348·1)	750·9<br>(717·1 to 783·0)	495·6<br>(482·1 to 509·1)	67·8<br>(63·7 to 71·9)	0·0<br>(0·0 to 0·0)
South Korea	2005	975·9<br>(950·3 to 1001·7)	561·1<br>(538·0 to 583·8)	364·1<br>(354·5 to 374·4)	50·7<br>(47·8 to 53·7)	0·0<br>(0·0 to 0·0)	1410·6<br>(1373·5 to 1447·8)	811·0<br>(777·6 to 843·8)	526·2<br>(512·3 to 541·2)	73·3<br>(69·1 to 77·7)	0·0<br>(0·0 to 0·0)

South Korea	2006	1081·4 (1056·3 to 1105·6)	630·0 (608·6 to 651·9)	394·5 (383·9 to 405·5)	56·8 (53·8 to 60·1)	0·0 (0·0 to 0·0)	1562·9 (1526·7 to 1598·0)	910·5 (879·6 to 942·2)	570·2 (554·8 to 586·1)	82·2 (77·7 to 86·8)	0·0 (0·0 to 0·0)
South Korea	2007	1184·9 (1161·0 to 1209·4)	690·9 (668·3 to 712·3)	430·2 (420·2 to 440·8)	63·9 (60·8 to 67·1)	0·0 (0·0 to 0·0)	1712·6 (1678·1 to 1748·0)	998·6 (965·9 to 1029·5)	621·7 (607·3 to 637·1)	92·3 (87·8 to 97·0)	0·0 (0·0 to 0·0)
South Korea	2008	1294·6 (1268·3 to 1321·8)	755·4 (733·1 to 778·1)	464·8 (453·6 to 475·3)	74·3 (70·9 to 77·7)	0·0 (0·0 to 0·0)	1871·1 (1833·2 to 1910·4)	1091·9 (1059·7 to 1124·6)	671·8 (655·7 to 687·0)	107·4 (102·5 to 112·4)	0·0 (0·0 to 0·0)
South Korea	2009	1409·3 (1383·3 to 1435·9)	834·1 (810·8 to 856·9)	486·3 (475·1 to 497·5)	88·9 (85·2 to 92·5)	0·0 (0·0 to 0·0)	2037·0 (1999·4 to 2075·3)	1205·6 (1171·8 to 1238·5)	702·8 (686·7 to 719·0)	128·5 (123·2 to 133·7)	0·0 (0·0 to 0·0)
South Korea	2010	1467·2 (1443·0 to 1493·1)	861·8 (840·2 to 885·8)	505·3 (495·3 to 516·4)	100·1 (96·5 to 103·8)	0·0 (0·0 to 0·0)	2120·6 (2085·7 to 2158·1)	1245·5 (1214·4 to 1280·2)	730·4 (715·8 to 746·4)	144·7 (139·4 to 150·0)	0·0 (0·0 to 0·0)
South Korea	2011	1562·5 (1534·8 to 1588·8)	914·6 (890·7 to 940·1)	534·3 (523·1 to 545·0)	113·6 (110·0 to 117·4)	0·0 (0·0 to 0·0)	2258·3 (2218·3 to 2296·3)	1321·9 (1287·4 to 1358·8)	772·2 (756·1 to 787·7)	164·2 (159·0 to 169·7)	0·0 (0·0 to 0·0)
South Korea	2012	1628·9 (1600·8 to 1657·8)	945·9 (921·6 to 971·2)	557·9 (547·1 to 569·7)	125·1 (121·4 to 128·7)	0·0 (0·0 to 0·0)	2354·3 (2313·7 to 2396·0)	1367·1 (1332·1 to 1403·7)	806·4 (790·8 to 823·4)	180·8 (175·5 to 186·1)	0·0 (0·0 to 0·0)
South Korea	2013	1686·4 (1658·9 to 1713·0)	972·7 (948·9 to 995·7)	577·4 (566·8 to 588·8)	136·3 (132·5 to 140·1)	0·0 (0·0 to 0·0)	2437·5 (2397·8 to 2475·8)	1405·9 (1371·4 to 1439·2)	834·6 (819·2 to 851·0)	196·9 (191·4 to 202·5)	0·0 (0·0 to 0·0)
South Korea	2014	1773·4 (1745·4 to 1800·7)	1016·6 (991·7 to 1040·7)	602·3 (591·2 to 613·3)	154·5 (150·6 to 158·3)	0·0 (0·0 to 0·0)	2563·2 (2522·7 to 2602·7)	1469·4 (1433·3 to 1504·1)	870·5 (854·4 to 886·5)	223·4 (217·7 to 228·7)	0·0 (0·0 to 0·0)
South Korea	2015	1872·7 (1844·3 to 1898·2)	1074·2 (1048·5 to 1098·1)	627·7 (616·8 to 639·2)	170·7 (166·6 to 175·0)	0·0 (0·0 to 0·0)	2706·7 (2665·6 to 2743·6)	1552·7 (1515·4 to 1587·1)	907·3 (891·5 to 923·9)	246·8 (240·8 to 252·9)	0·0 (0·0 to 0·0)
South Korea	2016	1968·4 (1941·0 to 1997·2)	1131·5 (1106·0 to 1157·2)	651·1 (639·3 to 662·1)	185·8 (181·6 to 189·9)	0·0 (0·0 to 0·0)	2845·0 (2805·5 to 2886·7)	1635·4 (1598·6 to 1672·5)	941·1 (924·0 to 957·0)	268·5 (262·5 to 274·5)	0·0 (0·0 to 0·0)
South Korea	2017	2095·3 (2064·8 to 2128·1)	1213·6 (1185·9 to 1239·9)	684·3 (671·5 to 697·0)	197·4 (193·1 to 202·0)	0·0 (0·0 to 0·0)	3028·5 (2984·3 to 3075·8)	1754·1 (1714·0 to 1792·1)	989·0 (970·5 to 1007·3)	285·3 (279·1 to 291·9)	0·0 (0·0 to 0·0)
South Korea	2018	2253·2 (2214·2 to 2290·4)	1316·1 (1280·5 to 1351·0)	732·3 (718·4 to 746·5)	204·7 (199·6 to 210·1)	0·0 (0·0 to 0·0)	3256·7 (3200·3 to 3310·4)	1902·3 (1850·7 to 1952·7)	1058·5 (1038·3 to 1078·9)	295·9 (288·5 to 303·6)	0·0 (0·0 to 0·0)
South Korea	2019	2441·6 (2394·9 to 2486·6)	1483·8 (1442·1 to 1527·2)	743·2 (728·8 to 757·5)	214·6 (208·7 to 220·9)	0·0 (0·0 to 0·0)	3529·0 (3461·4 to 3594·1)	2144·6 (2084·3 to 2207·3)	1074·2 (1053·4 to 1094·8)	310·2 (301·6 to 319·3)	0·0 (0·0 to 0·0)

South Korea	2020	2599·4 (2542·3 to 2654·9)	1673·9 (1620·9 to 1728·0)	713·2 (699·3 to 726·9)	212·3 (204·1 to 220·4)	0·0 (0·0 to 0·0)	3757·1 (3674·5 to 3837·2)	2419·4 (2342·8 to 2497·5)	1030·8 (1010·7 to 1050·6)	306·8 (295·0 to 318·5)	0·0 (0·0 to 0·0)
South Korea	2021	2563·0 (2507·2 to 2618·3)	1645·3 (1593·3 to 1698·5)	702·1 (688·3 to 715·5)	215·5 (204·1 to 227·1)	0·0 (0·0 to 0·0)	3704·4 (3623·9 to 3784·4)	2378·1 (2302·8 to 2454·9)	1014·9 (994·8 to 1034·1)	311·4 (294·9 to 328·3)	0·0 (0·0 to 0·0)
South Korea	2022	2494·1 (2429·5 to 2560·4)	1560·2 (1497·8 to 1623·5)	709·4 (695·2 to 723·1)	224·5 (209·1 to 240·8)	0·0 (0·0 to 0·0)	3604·9 (3511·6 to 3700·7)	2255·1 (2164·8 to 2346·6)	1025·3 (1004·8 to 1045·1)	324·5 (302·2 to 348·0)	0·0 (0·0 to 0·0)
South Korea	2023	2578·1 (2503·8 to 2654·8)	1629·3 (1559·3 to 1703·3)	715·2 (700·9 to 729·2)	233·6 (213·7 to 255·4)	0·0 (0·0 to 0·0)	3726·3 (3618·8 to 3837·2)	2354·9 (2253·7 to 2461·9)	1033·7 (1013·1 to 1054·0)	337·6 (308·9 to 369·2)	0·0 (0·0 to 0·0)
South Korea	2024	2660·9 (2574·7 to 2751·2)	1699·0 (1618·8 to 1784·2)	719·3 (704·5 to 733·6)	242·7 (217·5 to 270·9)	0·0 (0·0 to 0·0)	3846·0 (3721·4 to 3976·4)	2455·6 (2339·7 to 2578·8)	1039·7 (1018·3 to 1060·3)	350·7 (314·4 to 391·5)	0·0 (0·0 to 0·0)
South Korea	2025	2744·3 (2644·0 to 2848·5)	1769·7 (1676·4 to 1867·3)	722·7 (708·0 to 737·8)	251·8 (220·9 to 286·6)	0·0 (0·0 to 0·0)	3966·4 (3821·6 to 4117·1)	2557·9 (2423·0 to 2698·9)	1044·6 (1023·3 to 1066·4)	364·0 (319·3 to 414·3)	0·0 (0·0 to 0·0)
South Korea	2026	2821·4 (2706·1 to 2939·0)	1834·7 (1726·8 to 1947·2)	725·7 (710·3 to 741·3)	261·0 (224·6 to 302·9)	0·0 (0·0 to 0·0)	4077·9 (3911·3 to 4247·9)	2651·8 (2495·9 to 2814·5)	1048·8 (1026·6 to 1071·5)	377·2 (324·6 to 437·8)	0·0 (0·0 to 0·0)
South Korea	2027	2898·4 (2770·6 to 3033·6)	1898·8 (1779·0 to 2030·7)	729·2 (713·8 to 745·7)	270·4 (228·5 to 319·6)	0·0 (0·0 to 0·0)	4189·3 (4004·5 to 4384·6)	2744·5 (2571·3 to 2935·2)	1053·9 (1031·7 to 1077·9)	390·9 (330·3 to 461·9)	0·0 (0·0 to 0·0)
South Korea	2028	2974·6 (2835·4 to 3122·2)	1961·6 (1834·9 to 2102·3)	733·0 (717·3 to 750·2)	280·0 (232·7 to 335·7)	0·0 (0·0 to 0·0)	4299·4 (4098·1 to 4512·7)	2835·2 (2652·0 to 3038·6)	1059·4 (1036·8 to 1084·4)	404·7 (336·3 to 485·2)	0·0 (0·0 to 0·0)
South Korea	2029	3052·3 (2893·9 to 3213·1)	2025·2 (1878·5 to 2181·4)	737·2 (721·2 to 755·1)	289·9 (237·0 to 352·5)	0·0 (0·0 to 0·0)	4411·7 (4182·8 to 4644·1)	2927·2 (2715·1 to 3152·9)	1065·5 (1042·4 to 1091·4)	419·0 (342·6 to 509·5)	0·0 (0·0 to 0·0)
South Korea	2030	3134·1 (2961·2 to 3316·0)	2091·5 (1927·2 to 2270·3)	742·2 (726·0 to 760·3)	300·4 (242·0 to 370·1)	0·0 (0·0 to 0·0)	4530·0 (4279·9 to 4792·9)	3023·0 (2785·4 to 3281·4)	1072·8 (1049·3 to 1098·9)	434·1 (349·8 to 535·0)	0·0 (0·0 to 0·0)
South Korea	2031	3215·9 (3028·0 to 3416·1)	2157·9 (1976·4 to 2353·7)	747·0 (730·2 to 765·3)	311·1 (246·4 to 388·3)	0·0 (0·0 to 0·0)	4648·2 (4376·5 to 4937·4)	3118·9 (2856·6 to 3401·9)	1079·7 (1055·5 to 1106·1)	449·6 (356·1 to 561·3)	0·0 (0·0 to 0·0)
South Korea	2032	3298·7 (3094·9 to 3520·2)	2225·1 (2029·4 to 2443·7)	751·5 (734·3 to 770·2)	322·1 (251·0 to 407·6)	0·0 (0·0 to 0·0)	4767·7 (4473·3 to 5087·9)	3216·1 (2933·2 to 3532·1)	1086·2 (1061·3 to 1113·2)	465·5 (362·8 to 589·2)	0·0 (0·0 to 0·0)
South Korea	2033	3384·0 (3162·3 to 3622·8)	2294·8 (2086·8 to 2523·3)	755·7 (738·0 to 774·6)	333·4 (255·4 to 428·0)	0·0 (0·0 to 0·0)	4891·0 (4570·7 to 5236·2)	3316·8 (3016·1 to 3647·1)	1092·3 (1066·7 to 1119·5)	481·9 (369·2 to 618·7)	0·0 (0·0 to 0·0)

South Korea	2034	3469·3 (3231·5 to 3723·7)	2365·0 (2135·6 to 2610·6)	759·2 (740·9 to 778·4)	345·0 (259·8 to 449·4)	0·0 (0·0 to 0·0)	5014·3 (4670·6 to 5382·1)	3418·3 (3086·7 to 3773·3)	1097·4 (1070·9 to 1125·1)	498·6 (375·5 to 649·5)	0·0 (0·0 to 0·0)
South Korea	2035	3552·8 (3297·7 to 3822·5)	2433·9 (2195·4 to 2690·5)	762·3 (743·1 to 781·9)	356·7 (264·0 to 471·3)	0·0 (0·0 to 0·0)	5135·1 (4766·4 to 5524·8)	3517·9 (3173·2 to 3888·7)	1101·7 (1074·0 to 1130·2)	515·5 (381·6 to 681·2)	0·0 (0·0 to 0·0)
South Korea	2036	3635·5 (3362·3 to 3919·9)	2501·7 (2247·9 to 2771·8)	765·3 (745·4 to 785·4)	368·6 (268·5 to 493·4)	0·0 (0·0 to 0·0)	5254·6 (4859·7 to 5665·6)	3615·8 (3249·0 to 4006·3)	1106·1 (1077·3 to 1135·2)	532·7 (388·1 to 713·1)	0·0 (0·0 to 0·0)
South Korea	2037	3719·4 (3431·2 to 4014·7)	2569·4 (2301·6 to 2856·7)	769·1 (748·1 to 790·0)	380·9 (274·0 to 516·2)	0·0 (0·0 to 0·0)	5375·9 (4959·3 to 5802·7)	3713·7 (3326·7 to 4129·0)	1111·6 (1081·3 to 1141·8)	550·6 (396·1 to 746·1)	0·0 (0·0 to 0·0)
South Korea	2038	3805·6 (3502·3 to 4119·3)	2637·4 (2356·4 to 2935·2)	774·2 (752·4 to 795·8)	394·0 (280·4 to 540·1)	0·0 (0·0 to 0·0)	5500·4 (5062·1 to 5953·8)	3812·0 (3405·8 to 4242·3)	1118·9 (1087·5 to 1150·2)	569·5 (405·3 to 780·7)	0·0 (0·0 to 0·0)
South Korea	2039	3893·6 (3574·4 to 4219·9)	2706·5 (2416·8 to 3014·2)	779·7 (757·6 to 802·1)	407·5 (287·4 to 564·7)	0·0 (0·0 to 0·0)	5627·7 (5166·3 to 6099·2)	3911·8 (3493·1 to 4356·6)	1127·0 (1095·0 to 1159·4)	588·9 (415·4 to 816·2)	0·0 (0·0 to 0·0)
South Korea	2040	3983·3 (3643·3 to 4329·1)	2775·7 (2467·3 to 3103·8)	786·0 (762·8 to 809·2)	421·6 (295·0 to 590·1)	0·0 (0·0 to 0·0)	5757·3 (5265·9 to 6257·1)	4011·9 (3566·1 to 4486·1)	1136·1 (1102·6 to 1169·5)	609·3 (426·4 to 852·9)	0·0 (0·0 to 0·0)
South Korea	2041	4076·3 (3723·4 to 4444·6)	2846·8 (2525·5 to 3192·2)	793·0 (768·9 to 817·0)	436·5 (302·7 to 616·7)	0·0 (0·0 to 0·0)	5891·7 (5381·6 to 6424·0)	4114·7 (3650·3 to 4613·9)	1146·2 (1111·4 to 1180·8)	630·8 (437·5 to 891·4)	0·0 (0·0 to 0·0)
South Korea	2042	4170·0 (3799·7 to 4557·3)	2918·2 (2581·0 to 3277·4)	799·9 (775·1 to 824·9)	451·8 (309·9 to 642·6)	0·0 (0·0 to 0·0)	6027·1 (5491·9 to 6586·9)	4217·9 (3730·4 to 4737·0)	1156·2 (1120·4 to 1192·2)	653·0 (448·0 to 928·8)	0·0 (0·0 to 0·0)
South Korea	2043	4261·6 (3877·0 to 4669·8)	2988·1 (2636·8 to 3373·2)	806·2 (780·7 to 832·3)	467·3 (317·1 to 668·3)	0·0 (0·0 to 0·0)	6159·5 (5603·7 to 6749·5)	4318·9 (3811·1 to 4875·5)	1165·2 (1128·4 to 1202·9)	675·4 (458·3 to 965·9)	0·0 (0·0 to 0·0)
South Korea	2044	4356·0 (3958·6 to 4780·5)	3059·9 (2691·1 to 3459·1)	812·6 (786·4 to 839·6)	483·4 (324·1 to 698·5)	0·0 (0·0 to 0·0)	6296·0 (5721·6 to 6909·5)	4422·7 (3889·6 to 4999·6)	1174·5 (1136·7 to 1213·5)	698·8 (468·5 to 1009·5)	0·0 (0·0 to 0·0)
South Korea	2045	4449·1 (4034·7 to 4895·0)	3130·1 (2759·6 to 3547·2)	819·0 (791·8 to 846·3)	500·0 (331·3 to 730·8)	0·0 (0·0 to 0·0)	6430·5 (5831·6 to 7075·0)	4524·1 (3988·6 to 5127·0)	1183·7 (1144·5 to 1223·2)	722·7 (478·8 to 1056·2)	0·0 (0·0 to 0·0)
South Korea	2046	4545·1 (4116·8 to 4999·2)	3201·3 (2819·1 to 3621·9)	826·4 (798·3 to 854·3)	517·4 (339·0 to 764·8)	0·0 (0·0 to 0·0)	6569·3 (5950·2 to 7225·7)	4627·0 (4074·6 to 5234·9)	1194·4 (1153·9 to 1234·7)	747·9 (490·0 to 1105·5)	0·0 (0·0 to 0·0)
South Korea	2047	4646·3 (4205·3 to 5112·7)	3275·2 (2877·0 to 3704·7)	835·1 (806·2 to 864·0)	535·9 (347·4 to 798·5)	0·0 (0·0 to 0·0)	6715·6 (6078·2 to 7389·7)	4733·9 (4158·3 to 5354·6)	1207·1 (1165·3 to 1248·8)	774·6 (502·2 to 1154·1)	0·0 (0·0 to 0·0)

South Korea	2048	4747·4 (4292·3 to 5229·6)	3347·4 (2939·1 to 3790·1)	844·8 (814·9 to 874·6)	555·1 (357·2 to 830·5)	0·0 (0·0 to 0·0)	6861·6 (6203·9 to 7558·6)	4838·2 (4248·1 to 5478·1)	1221·0 (1177·8 to 1264·1)	802·4 (516·3 to 1200·3)	0·0 (0·0 to 0·0)
South Korea	2049	4849·8 (4378·2 to 5361·7)	3419·5 (3004·0 to 3876·5)	855·3 (824·8 to 886·1)	575·1 (369·3 to 861·9)	0·0 (0·0 to 0·0)	7009·8 (6328·0 to 7749·5)	4942·3 (4341·9 to 5603·0)	1236·2 (1192·1 to 1280·8)	831·3 (533·7 to 1245·8)	0·0 (0·0 to 0·0)
South Korea	2050	4957·3 (4470·1 to 5477·5)	3494·0 (3065·0 to 3965·5)	866·9 (835·5 to 898·7)	596·3 (380·5 to 893·8)	0·0 (0·0 to 0·0)	7165·0 (6460·9 to 7917·0)	5050·1 (4430·1 to 5731·6)	1253·0 (1207·6 to 1298·9)	861·9 (550·0 to 1291·8)	0·0 (0·0 to 0·0)
Kuwait	1995	1341·6 (1212·5 to 1483·3)	1092·2 (971·5 to 1228·4)	234·7 (211·0 to 258·4)	14·7 (10·6 to 19·7)	0·0 (0·0 to 0·0)	2515·7 (2273·6 to 2781·5)	2048·0 (1821·7 to 2303·5)	440·1 (395·7 to 484·6)	27·6 (20·0 to 36·9)	0·0 (0·0 to 0·0)
Kuwait	1996	1277·5 (1169·9 to 1392·9)	1018·2 (915·8 to 1130·7)	244·1 (223·9 to 263·8)	15·2 (11·4 to 20·0)	0·0 (0·0 to 0·0)	2395·5 (2193·8 to 2611·8)	1909·2 (1717·2 to 2120·3)	457·7 (419·8 to 494·6)	28·6 (21·4 to 37·5)	0·0 (0·0 to 0·0)
Kuwait	1997	1284·0 (1187·6 to 1394·8)	1014·7 (919·9 to 1125·0)	253·6 (234·9 to 272·1)	15·7 (12·0 to 20·3)	0·0 (0·0 to 0·0)	2407·7 (2226·9 to 2615·5)	1902·7 (1724·9 to 2109·6)	475·5 (440·4 to 510·3)	29·5 (22·4 to 38·1)	0·0 (0·0 to 0·0)
Kuwait	1998	1384·4 (1281·0 to 1502·3)	1099·2 (997·0 to 1214·9)	267·7 (248·9 to 287·1)	17·5 (13·9 to 21·7)	0·0 (0·0 to 0·0)	2596·0 (2402·1 to 2817·0)	2061·2 (1869·5 to 2278·1)	502·0 (466·8 to 538·3)	32·8 (26·1 to 40·6)	0·0 (0·0 to 0·0)
Kuwait	1999	1195·7 (1107·3 to 1298·5)	944·7 (855·1 to 1045·5)	235·1 (219·2 to 251·4)	15·8 (13·3 to 18·8)	0·0 (0·0 to 0·0)	2242·1 (2076·3 to 2434·9)	1771·5 (1603·4 to 1960·5)	440·9 (411·0 to 471·4)	29·7 (24·9 to 35·3)	0·0 (0·0 to 0·0)
Kuwait	2000	1043·4 (962·1 to 1127·6)	821·1 (742·3 to 905·0)	207·8 (194·4 to 222·2)	14·5 (12·7 to 16·5)	0·0 (0·0 to 0·0)	1956·4 (1804·1 to 2114·5)	1539·6 (1391·9 to 1697·0)	389·7 (364·6 to 416·6)	27·1 (23·8 to 30·9)	0·0 (0·0 to 0·0)
Kuwait	2001	1142·2 (1054·9 to 1239·1)	915·2 (830·5 to 1012·8)	210·8 (197·6 to 224·3)	16·1 (14·5 to 17·9)	0·0 (0·0 to 0·0)	2141·7 (1978·0 to 2323·4)	1716·2 (1557·3 to 1899·2)	395·3 (370·6 to 420·5)	30·2 (27·3 to 33·5)	0·0 (0·0 to 0·0)
Kuwait	2002	1141·1 (1056·0 to 1243·4)	913·0 (830·0 to 1011·2)	209·9 (197·1 to 223·3)	18·2 (16·6 to 19·8)	0·0 (0·0 to 0·0)	2139·8 (1980·1 to 2331·5)	1712·0 (1556·4 to 1896·1)	393·5 (369·6 to 418·8)	34·2 (31·2 to 37·1)	0·0 (0·0 to 0·0)
Kuwait	2003	1128·6 (1035·4 to 1228·7)	902·0 (812·7 to 998·8)	205·6 (193·6 to 218·3)	21·0 (19·4 to 22·9)	0·0 (0·0 to 0·0)	2116·3 (1941·6 to 2304·0)	1691·3 (1524·0 to 1872·8)	385·5 (363·1 to 409·4)	39·5 (36·3 to 42·9)	0·0 (0·0 to 0·0)
Kuwait	2004	1075·4 (984·4 to 1172·7)	860·1 (771·5 to 956·0)	194·8 (182·8 to 207·4)	20·6 (19·0 to 22·4)	0·0 (0·0 to 0·0)	2016·6 (1845·9 to 2199·1)	1612·7 (1446·6 to 1792·7)	365·3 (342·7 to 388·9)	38·6 (35·6 to 41·9)	0·0 (0·0 to 0·0)
Kuwait	2005	958·0 (879·8 to 1044·8)	763·4 (685·9 to 846·8)	176·1 (165·2 to 188·6)	18·5 (17·0 to 20·2)	0·0 (0·0 to 0·0)	1796·4 (1649·7 to 1959·1)	1431·5 (1286·2 to 1587·9)	330·1 (309·8 to 353·7)	34·8 (32·0 to 37·9)	0·0 (0·0 to 0·0)

Kuwait	2006	1020·3 (936·2 to 1109·8)	828·4 (743·6 to 917·7)	173·4 (162·5 to 186·3)	18·5 (16·9 to 20·3)	0·0 (0·0 to 0·0)	1913·2 (1755·4 to 2081·0)	1553·4 (1394·4 to 1720·8)	325·1 (304·6 to 349·3)	34·7 (31·7 to 38·0)	0·0 (0·0 to 0·0)
Kuwait	2007	942·6 (862·6 to 1022·5)	764·1 (684·1 to 844·3)	161·5 (151·0 to 173·9)	16·9 (15·4 to 18·6)	0·0 (0·0 to 0·0)	1767·4 (1617·6 to 1917·2)	1432·9 (1282·7 to 1583·2)	302·8 (283·1 to 326·1)	31·7 (28·9 to 34·8)	0·0 (0·0 to 0·0)
Kuwait	2008	1079·5 (981·9 to 1172·8)	900·2 (805·5 to 990·9)	162·2 (150·0 to 173·9)	17·1 (15·5 to 18·8)	0·0 (0·0 to 0·0)	2024·1 (1841·3 to 2199·3)	1688·0 (1510·5 to 1858·0)	304·1 (281·3 to 326·2)	32·0 (29·1 to 35·2)	0·0 (0·0 to 0·0)
Kuwait	2009	1066·4 (974·8 to 1162·1)	902·0 (812·4 to 996·6)	148·8 (136·8 to 161·2)	15·6 (14·1 to 17·2)	0·0 (0·0 to 0·0)	1999·6 (1827·8 to 2179·0)	1691·3 (1523·4 to 1868·7)	279·1 (256·5 to 302·2)	29·2 (26·5 to 32·2)	0·0 (0·0 to 0·0)
Kuwait	2010	1009·6 (921·0 to 1100·9)	863·0 (776·7 to 955·7)	133·0 (121·8 to 144·5)	13·5 (12·1 to 15·0)	0·0 (0·0 to 0·0)	1893·1 (1727·0 to 2064·4)	1618·3 (1456·4 to 1792·0)	249·5 (228·3 to 270·9)	25·4 (22·8 to 28·2)	0·0 (0·0 to 0·0)
Kuwait	2011	953·1 (877·3 to 1041·5)	817·6 (741·5 to 907·4)	123·0 (112·3 to 134·0)	12·5 (11·1 to 13·9)	0·0 (0·0 to 0·0)	1787·2 (1645·1 to 1952·9)	1533·2 (1390·5 to 1701·5)	230·7 (210·6 to 251·2)	23·4 (20·8 to 26·0)	0·0 (0·0 to 0·0)
Kuwait	2012	962·2 (883·0 to 1051·6)	825·1 (748·4 to 913·2)	124·4 (113·6 to 136·1)	12·7 (11·3 to 14·3)	0·0 (0·0 to 0·0)	1804·2 (1655·8 to 1971·9)	1547·1 (1403·3 to 1712·4)	233·2 (213·1 to 255·3)	23·9 (21·2 to 26·8)	0·0 (0·0 to 0·0)
Kuwait	2013	982·6 (898·0 to 1069·5)	838·4 (756·0 to 925·2)	130·5 (119·1 to 142·6)	13·7 (12·2 to 15·3)	0·0 (0·0 to 0·0)	1842·6 (1684·0 to 2005·5)	1572·2 (1417·6 to 1734·8)	244·7 (223·4 to 267·4)	25·7 (22·8 to 28·7)	0·0 (0·0 to 0·0)
Kuwait	2014	1090·5 (1000·5 to 1184·0)	930·0 (841·5 to 1021·9)	145·2 (133·4 to 157·9)	15·3 (13·7 to 17·0)	0·0 (0·0 to 0·0)	2044·8 (1876·0 to 2220·1)	1743·9 (1577·9 to 1916·3)	272·3 (250·2 to 296·1)	28·6 (25·7 to 31·8)	0·0 (0·0 to 0·0)
Kuwait	2015	1241·0 (1145·3 to 1343·8)	1055·5 (957·3 to 1157·2)	167·3 (155·2 to 179·8)	18·1 (16·4 to 20·0)	0·0 (0·0 to 0·0)	2327·0 (2147·6 to 2519·8)	1979·3 (1795·1 to 2170·0)	313·8 (291·1 to 337·1)	33·9 (30·8 to 37·4)	0·0 (0·0 to 0·0)
Kuwait	2016	1313·9 (1216·3 to 1421·0)	1116·7 (1021·7 to 1224·0)	177·9 (166·2 to 191·5)	19·3 (17·6 to 21·0)	0·0 (0·0 to 0·0)	2463·7 (2280·7 to 2664·6)	2093·9 (1915·8 to 2295·2)	333·6 (311·6 to 359·1)	36·2 (33·1 to 39·4)	0·0 (0·0 to 0·0)
Kuwait	2017	1349·7 (1239·2 to 1470·4)	1159·5 (1049·2 to 1277·4)	172·2 (159·0 to 186·5)	18·1 (16·5 to 19·9)	0·0 (0·0 to 0·0)	2530·9 (2323·7 to 2757·3)	2174·1 (1967·4 to 2395·3)	322·9 (298·2 to 349·7)	33·9 (30·9 to 37·2)	0·0 (0·0 to 0·0)
Kuwait	2018	1363·2 (1237·9 to 1503·3)	1184·0 (1062·6 to 1319·1)	162·5 (146·9 to 179·4)	16·6 (14·7 to 18·7)	0·0 (0·0 to 0·0)	2556·1 (2321·2 to 2818·9)	2220·3 (1992·6 to 2473·6)	304·7 (275·5 to 336·5)	31·1 (27·6 to 35·1)	0·0 (0·0 to 0·0)
Kuwait	2019	1452·8 (1316·8 to 1606·8)	1277·3 (1143·4 to 1428·9)	158·8 (143·7 to 175·4)	16·8 (14·8 to 18·9)	0·0 (0·0 to 0·0)	2724·2 (2469·2 to 3012·9)	2395·1 (2144·0 to 2679·4)	297·7 (269·4 to 328·9)	31·4 (27·7 to 35·5)	0·0 (0·0 to 0·0)

Kuwait	2020	1584·2 (1409·3 to 1781·0)	1427·8 (1254·9 to 1624·6)	141·0 (127·3 to 155·5)	15·4 (13·5 to 17·6)	0·0 (0·0 to 0·0)	2970·6 (2642·5 to 3339·6)	2677·3 (2353·1 to 3046·4)	264·3 (238·7 to 291·7)	29·0 (25·3 to 33·0)	0·0 (0·0 to 0·0)
Kuwait	2021	1412·9 (1256·8 to 1588·5)	1272·9 (1118·8 to 1448·4)	125·7 (113·4 to 139·0)	14·3 (12·4 to 16·5)	0·0 (0·0 to 0·0)	2649·5 (2356·8 to 2978·7)	2386·9 (2097·8 to 2715·9)	235·8 (212·7 to 260·6)	26·8 (23·2 to 30·9)	0·0 (0·0 to 0·0)
Kuwait	2022	1253·2 (1089·6 to 1439·0)	1112·9 (951·3 to 1296·2)	125·5 (113·2 to 138·8)	14·8 (12·7 to 17·3)	0·0 (0·0 to 0·0)	2349·8 (2043·1 to 2698·3)	2086·8 (1783·8 to 2430·5)	235·3 (212·2 to 260·3)	27·7 (23·8 to 32·4)	0·0 (0·0 to 0·0)
Kuwait	2023	1284·2 (1112·1 to 1474·4)	1143·2 (971·5 to 1330·2)	125·6 (113·3 to 138·9)	15·4 (12·9 to 18·1)	0·0 (0·0 to 0·0)	2408·0 (2085·4 to 2764·6)	2143·6 (1821·6 to 2494·2)	235·5 (212·4 to 260·5)	28·8 (24·2 to 33·9)	0·0 (0·0 to 0·0)
Kuwait	2024	1322·2 (1136·9 to 1531·3)	1180·3 (996·9 to 1387·4)	126·0 (113·6 to 139·2)	16·0 (13·2 to 19·0)	0·0 (0·0 to 0·0)	2479·4 (2131·9 to 2871·4)	2213·3 (1869·3 to 2601·5)	236·2 (213·1 to 261·0)	29·9 (24·7 to 35·7)	0·0 (0·0 to 0·0)
Kuwait	2025	1356·6 (1157·3 to 1582·5)	1214·2 (1018·0 to 1437·7)	125·9 (113·6 to 139·1)	16·5 (13·3 to 20·1)	0·0 (0·0 to 0·0)	2543·9 (2170·2 to 2967·5)	2276·8 (1908·9 to 2696·0)	236·1 (213·1 to 260·8)	31·0 (25·0 to 37·7)	0·0 (0·0 to 0·0)
Kuwait	2026	1375·3 (1171·3 to 1611·3)	1232·3 (1028·6 to 1467·6)	125·9 (113·7 to 139·0)	17·2 (13·5 to 21·3)	0·0 (0·0 to 0·0)	2578·9 (2196·4 to 3021·4)	2310·7 (1928·8 to 2751·9)	236·0 (213·1 to 260·6)	32·2 (25·2 to 40·0)	0·0 (0·0 to 0·0)
Kuwait	2027	1395·1 (1179·1 to 1652·4)	1251·4 (1037·5 to 1505·8)	125·9 (113·6 to 139·0)	17·8 (13·6 to 22·5)	0·0 (0·0 to 0·0)	2616·0 (2211·0 to 3098·5)	2346·6 (1945·5 to 2823·5)	236·0 (212·9 to 260·7)	33·4 (25·5 to 42·2)	0·0 (0·0 to 0·0)
Kuwait	2028	1416·1 (1190·2 to 1680·7)	1271·8 (1049·5 to 1537·2)	125·7 (113·3 to 138·9)	18·5 (13·7 to 23·9)	0·0 (0·0 to 0·0)	2655·3 (2231·8 to 3151·6)	2384·9 (1968·0 to 2882·4)	235·8 (212·4 to 260·5)	34·7 (25·6 to 44·8)	0·0 (0·0 to 0·0)
Kuwait	2029	1440·1 (1204·7 to 1709·9)	1295·2 (1063·1 to 1561·6)	125·7 (113·1 to 138·9)	19·2 (13·7 to 25·5)	0·0 (0·0 to 0·0)	2700·5 (2259·0 to 3206·3)	2428·7 (1993·4 to 2928·1)	235·7 (212·1 to 260·5)	36·0 (25·8 to 47·8)	0·0 (0·0 to 0·0)
Kuwait	2030	1439·3 (1200·5 to 1719·8)	1293·7 (1056·4 to 1572·8)	125·6 (113·1 to 138·9)	19·9 (14·3 to 26·5)	0·0 (0·0 to 0·0)	2698·8 (2251·1 to 3224·9)	2425·9 (1981·0 to 2949·2)	235·5 (212·0 to 260·5)	37·4 (26·9 to 49·7)	0·0 (0·0 to 0·0)
Kuwait	2031	1431·7 (1186·8 to 1717·2)	1285·9 (1038·6 to 1568·1)	125·5 (112·9 to 138·7)	20·3 (14·6 to 27·1)	0·0 (0·0 to 0·0)	2684·6 (2225·4 to 3219·9)	2411·3 (1947·6 to 2940·3)	235·2 (211·7 to 260·1)	38·0 (27·4 to 50·9)	0·0 (0·0 to 0·0)
Kuwait	2032	1431·6 (1187·6 to 1719·1)	1285·8 (1041·8 to 1572·3)	125·1 (112·5 to 138·3)	20·7 (14·9 to 27·8)	0·0 (0·0 to 0·0)	2684·4 (2227·0 to 3223·6)	2411·0 (1953·6 to 2948·3)	234·6 (210·9 to 259·4)	38·8 (28·0 to 52·2)	0·0 (0·0 to 0·0)
Kuwait	2033	1429·0 (1186·5 to 1718·6)	1283·2 (1040·4 to 1571·5)	124·7 (112·2 to 137·9)	21·1 (15·2 to 29·0)	0·0 (0·0 to 0·0)	2679·5 (2224·9 to 3222·6)	2406·1 (1951·0 to 2946·8)	233·9 (210·4 to 258·6)	39·5 (28·4 to 54·3)	0·0 (0·0 to 0·0)

Kuwait	2034	1423·5 (1182·9 to 1712·5)	1278·0 (1039·5 to 1566·3)	124·1 (111·7 to 137·3)	21·4 (15·4 to 29·7)	0·0 (0·0 to 0·0)	2669·2 (2218·0 to 3211·1)	2396·4 (1949·2 to 2937·0)	232·7 (209·4 to 257·4)	40·2 (28·8 to 55·8)	0·0 (0·0 to 0·0)
Kuwait	2035	1417·8 (1175·0 to 1706·7)	1272·7 (1034·8 to 1558·2)	123·3 (110·9 to 136·5)	21·8 (15·5 to 30·4)	0·0 (0·0 to 0·0)	2658·6 (2203·4 to 3200·2)	2386·4 (1940·4 to 2921·9)	231·3 (207·9 to 256·0)	40·9 (29·1 to 57·1)	0·0 (0·0 to 0·0)
Kuwait	2036	1411·5 (1173·8 to 1700·0)	1266·9 (1030·2 to 1550·7)	122·5 (110·0 to 135·6)	22·2 (15·6 to 31·2)	0·0 (0·0 to 0·0)	2646·8 (2201·0 to 3187·8)	2375·7 (1931·8 to 2907·9)	229·6 (206·3 to 254·2)	41·5 (29·3 to 58·5)	0·0 (0·0 to 0·0)
Kuwait	2037	1404·8 (1169·1 to 1691·7)	1260·8 (1027·7 to 1547·5)	121·5 (109·1 to 134·5)	22·5 (15·8 to 32·5)	0·0 (0·0 to 0·0)	2634·1 (2192·3 to 3172·1)	2364·1 (1927·1 to 2901·8)	227·9 (204·5 to 252·3)	42·2 (29·6 to 60·9)	0·0 (0·0 to 0·0)
Kuwait	2038	1395·3 (1161·3 to 1682·6)	1251·9 (1021·9 to 1538·5)	120·5 (108·2 to 133·6)	22·8 (15·9 to 33·1)	0·0 (0·0 to 0·0)	2616·4 (2177·6 to 3155·1)	2347·5 (1916·3 to 2884·9)	226·0 (203·0 to 250·5)	42·8 (29·8 to 62·1)	0·0 (0·0 to 0·0)
Kuwait	2039	1383·7 (1151·1 to 1669·6)	1241·2 (1013·3 to 1525·9)	119·3 (107·2 to 132·3)	23·1 (15·9 to 34·3)	0·0 (0·0 to 0·0)	2594·6 (2158·4 to 3130·7)	2327·5 (1900·1 to 2861·3)	223·7 (200·9 to 248·2)	43·4 (29·9 to 64·3)	0·0 (0·0 to 0·0)
Kuwait	2040	1368·5 (1137·2 to 1651·2)	1227·1 (999·4 to 1508·2)	118·0 (106·0 to 131·1)	23·4 (16·0 to 35·6)	0·0 (0·0 to 0·0)	2566·0 (2132·4 to 3096·2)	2301·0 (1874·0 to 2828·1)	221·2 (198·7 to 245·8)	43·9 (30·0 to 66·7)	0·0 (0·0 to 0·0)
Kuwait	2041	1352·3 (1121·9 to 1632·2)	1212·0 (985·1 to 1490·8)	116·6 (104·8 to 129·6)	23·7 (16·0 to 37·0)	0·0 (0·0 to 0·0)	2535·7 (2103·7 to 3060·7)	2272·7 (1847·3 to 2795·4)	218·6 (196·5 to 243·0)	44·4 (30·0 to 69·4)	0·0 (0·0 to 0·0)
Kuwait	2042	1331·8 (1105·0 to 1608·6)	1193·0 (967·3 to 1469·4)	114·9 (103·3 to 127·8)	23·9 (16·0 to 38·5)	0·0 (0·0 to 0·0)	2497·4 (2072·1 to 3016·4)	2237·1 (1813·8 to 2755·4)	215·4 (193·7 to 239·7)	44·9 (30·0 to 72·2)	0·0 (0·0 to 0·0)
Kuwait	2043	1309·6 (1086·3 to 1583·7)	1172·3 (951·4 to 1445·1)	113·2 (101·8 to 125·9)	24·1 (16·0 to 39·9)	0·0 (0·0 to 0·0)	2455·7 (2037·0 to 2969·7)	2198·2 (1784·0 to 2709·8)	212·3 (190·9 to 236·2)	45·3 (29·9 to 74·8)	0·0 (0·0 to 0·0)
Kuwait	2044	1286·0 (1064·7 to 1556·6)	1150·4 (931·6 to 1419·6)	111·3 (100·1 to 123·9)	24·3 (15·9 to 41·3)	0·0 (0·0 to 0·0)	2411·4 (1996·5 to 2918·8)	2157·1 (1746·9 to 2662·0)	208·7 (187·8 to 232·3)	45·6 (29·8 to 77·5)	0·0 (0·0 to 0·0)
Kuwait	2045	1261·0 (1043·8 to 1527·9)	1127·1 (911·0 to 1392·8)	109·4 (98·4 to 121·9)	24·5 (15·8 to 42·6)	0·0 (0·0 to 0·0)	2364·6 (1957·3 to 2865·0)	2113·5 (1708·3 to 2611·7)	205·1 (184·6 to 228·6)	45·9 (29·7 to 80·0)	0·0 (0·0 to 0·0)
Kuwait	2046	1234·3 (1020·7 to 1495·7)	1102·5 (889·5 to 1363·8)	107·2 (96·5 to 119·6)	24·6 (15·8 to 43·9)	0·0 (0·0 to 0·0)	2314·5 (1913·9 to 2804·7)	2067·3 (1667·9 to 2557·3)	201·1 (180·9 to 224·3)	46·1 (29·5 to 82·2)	0·0 (0·0 to 0·0)
Kuwait	2047	1205·2 (995·5 to 1461·6)	1075·6 (866·8 to 1331·8)	104·9 (94·4 to 117·0)	24·7 (15·7 to 45·0)	0·0 (0·0 to 0·0)	2259·9 (1866·6 to 2740·7)	2016·9 (1625·3 to 2497·3)	196·7 (176·9 to 219·5)	46·2 (29·4 to 84·4)	0·0 (0·0 to 0·0)

Kuwait	2048	1172·5 (968·5 to 1421·8)	1045·5 (842·8 to 1295·3)	102·3 (92·0 to 114·3)	24·7 (15·5 to 46·1)	0·0 (0·0 to 0·0)	2198·5 (1816·1 to 2666·0)	1960·4 (1580·4 to 2428·9)	191·9 (172·6 to 214·4)	46·2 (29·0 to 86·4)	0·0 (0·0 to 0·0)
Kuwait	2049	1134·3 (936·8 to 1376·7)	1010·0 (814·5 to 1252·0)	99·7 (89·6 to 111·6)	24·6 (15·3 to 47·3)	0·0 (0·0 to 0·0)	2127·0 (1756·7 to 2581·5)	1894·0 (1527·2 to 2347·6)	186·9 (168·0 to 209·2)	46·2 (28·6 to 88·7)	0·0 (0·0 to 0·0)
Kuwait	2050	1098·3 (905·9 to 1331·6)	976·8 (787·7 to 1211·0)	96·9 (87·1 to 108·7)	24·5 (15·0 to 48·6)	0·0 (0·0 to 0·0)	2059·5 (1698·8 to 2497·0)	1831·7 (1477·1 to 2270·9)	181·8 (163·3 to 203·8)	46·0 (28·2 to 91·1)	0·0 (0·0 to 0·0)
Laos*	1995	33·2 (28·9 to 38·4)	12·1 (9·8 to 14·4)	17·2 (13·7 to 21·5)	1·7 (1·2 to 2·4)	2·3 (2·3 to 2·3)	106·3 (92·4 to 122·8)	38·6 (31·5 to 46·2)	55·0 (43·8 to 68·8)	5·5 (3·7 to 7·8)	7·3 (7·3 to 7·3)
Laos*	1996	32·7 (28·4 to 37·7)	12·1 (9·8 to 14·6)	17·3 (13·6 to 21·5)	1·8 (1·2 to 2·5)	1·5 (1·5 to 1·5)	104·7 (90·9 to 120·8)	38·9 (31·4 to 46·7)	55·4 (43·7 to 68·9)	5·6 (3·9 to 8·0)	4·8 (4·8 to 4·8)
Laos*	1997	34·1 (29·8 to 38·8)	12·3 (10·0 to 14·7)	17·5 (13·9 to 21·7)	1·9 (1·3 to 2·7)	2·4 (2·4 to 2·4)	109·1 (95·6 to 124·2)	39·3 (32·1 to 47·0)	56·1 (44·7 to 69·4)	6·0 (4·1 to 8·6)	7·6 (7·6 to 7·6)
Laos*	1998	33·5 (29·4 to 38·0)	11·9 (9·8 to 14·3)	17·6 (14·2 to 21·4)	2·0 (1·4 to 2·9)	2·0 (2·0 to 2·0)	107·2 (94·1 to 121·5)	38·2 (31·4 to 45·7)	56·3 (45·6 to 68·6)	6·4 (4·4 to 9·3)	6·3 (6·3 to 6·3)
Laos*	1999	34·6 (30·6 to 38·8)	11·7 (9·6 to 14·1)	17·8 (14·5 to 21·3)	2·3 (1·6 to 3·3)	2·8 (2·8 to 2·8)	110·8 (97·9 to 124·3)	37·5 (30·8 to 45·0)	56·9 (46·5 to 68·2)	7·3 (5·1 to 10·6)	9·1 (9·1 to 9·1)
Laos*	2000	39·6 (35·5 to 43·8)	11·7 (9·6 to 14·0)	18·1 (15·0 to 21·6)	3·3 (2·3 to 4·8)	6·5 (6·5 to 6·5)	126·7 (113·8 to 140·4)	37·3 (30·8 to 44·9)	58·1 (48·2 to 69·1)	10·7 (7·3 to 15·4)	20·7 (20·7 to 20·7)
Laos*	2001	35·9 (31·9 to 40·0)	11·9 (9·8 to 14·3)	18·7 (15·7 to 22·1)	2·7 (1·8 to 3·8)	2·6 (2·6 to 2·6)	114·8 (102·2 to 128·1)	38·0 (31·4 to 45·7)	59·9 (50·2 to 70·6)	8·5 (5·8 to 12·0)	8·4 (8·4 to 8·4)
Laos*	2002	33·9 (30·3 to 37·9)	10·4 (8·6 to 12·5)	18·5 (15·6 to 21·9)	2·7 (1·9 to 3·7)	2·4 (2·4 to 2·4)	108·7 (96·9 to 121·5)	33·3 (27·5 to 40·0)	59·3 (50·0 to 70·1)	8·6 (5·9 to 11·9)	7·6 (7·6 to 7·6)
Laos*	2003	39·1 (35·3 to 43·2)	10·4 (8·6 to 12·5)	19·5 (16·4 to 22·8)	2·9 (2·0 to 4·1)	6·3 (6·3 to 6·3)	125·2 (113·2 to 138·2)	33·3 (27·7 to 40·1)	62·4 (52·6 to 73·2)	9·3 (6·3 to 13·0)	20·3 (20·3 to 20·3)
Laos*	2004	33·4 (30·2 to 36·7)	8·8 (7·2 to 10·6)	17·3 (14·8 to 20·2)	2·8 (1·9 to 3·9)	4·5 (4·5 to 4·5)	106·9 (96·8 to 117·4)	28·0 (23·2 to 33·9)	55·5 (47·4 to 64·6)	8·9 (6·1 to 12·4)	14·4 (14·4 to 14·4)
Laos*	2005	35·6 (32·6 to 38·6)	9·2 (7·6 to 11·2)	16·5 (14·4 to 18·9)	2·8 (1·9 to 3·9)	7·1 (7·1 to 7·1)	113·9 (104·4 to 123·5)	29·4 (24·3 to 35·8)	53·0 (46·2 to 60·6)	8·8 (6·1 to 12·5)	22·7 (22·7 to 22·7)

Laos*	2006	32·0 (29·6 to 34·8)	8·4 (6·9 to 10·3)	15·0 (13·4 to 16·8)	2·3 (1·6 to 3·3)	6·3 (6·3 to 6·3)	102·4 (94·8 to 111·4)	26·8 (22·1 to 32·8)	48·0 (42·8 to 53·7)	7·4 (5·2 to 10·5)	20·2 (20·2 to 20·2)
Laos*	2007	42·4 (38·5 to 47·5)	8·3 (6·8 to 10·1)	14·5 (13·2 to 15·9)	12·1 (8·5 to 16·8)	7·5 (7·5 to 7·5)	135·9 (123·3 to 152·1)	26·7 (21·9 to 32·3)	46·5 (42·2 to 51·0)	38·8 (27·2 to 54·0)	23·9 (23·9 to 23·9)
Laos*	2008	41·0 (37·5 to 45·5)	8·3 (6·9 to 10·0)	13·9 (12·7 to 15·2)	10·6 (7·3 to 14·7)	8·2 (8·2 to 8·2)	131·4 (120·1 to 145·9)	26·6 (22·0 to 32·0)	44·6 (40·8 to 48·6)	34·0 (23·4 to 47·1)	26·2 (26·2 to 26·2)
Laos*	2009	43·8 (40·1 to 48·1)	10·2 (8·5 to 12·0)	15·3 (14·0 to 16·6)	10·3 (7·3 to 14·4)	8·0 (8·0 to 8·0)	140·3 (128·5 to 154·1)	32·7 (27·2 to 38·5)	49·0 (45·0 to 53·2)	32·9 (23·4 to 46·2)	25·7 (25·7 to 25·7)
Laos*	2010	45·3 (42·0 to 49·1)	10·0 (8·4 to 11·7)	16·6 (15·5 to 17·7)	8·9 (6·2 to 12·4)	9·8 (9·8 to 9·8)	145·0 (134·5 to 157·1)	32·1 (27·0 to 37·6)	53·1 (49·8 to 56·7)	28·5 (19·9 to 39·7)	31·3 (31·3 to 31·3)
Laos*	2011	37·3 (35·8 to 39·0)	8·9 (7·7 to 10·2)	16·8 (15·9 to 17·7)	0·7 (0·5 to 1·0)	10·9 (10·9 to 10·9)	119·6 (114·7 to 124·9)	28·6 (24·6 to 32·6)	53·8 (51·1 to 56·7)	2·3 (1·6 to 3·2)	34·9 (34·9 to 34·9)
Laos*	2012	40·0 (38·4 to 41·7)	10·2 (8·9 to 11·5)	18·8 (17·9 to 19·8)	1·0 (0·7 to 1·4)	10·0 (10·0 to 10·0)	128·1 (123·0 to 133·4)	32·5 (28·6 to 36·8)	60·2 (57·4 to 63·3)	3·2 (2·2 to 4·4)	32·1 (32·1 to 32·1)
Laos*	2013	47·7 (46·0 to 49·6)	12·7 (11·3 to 14·2)	21·6 (20·7 to 22·6)	1·2 (0·8 to 1·6)	12·3 (12·3 to 12·3)	152·9 (147·3 to 159·0)	40·8 (36·3 to 45·5)	69·1 (66·1 to 72·2)	3·7 (2·5 to 5·2)	39·3 (39·3 to 39·3)
Laos*	2014	49·8 (47·9 to 51·8)	14·7 (13·3 to 16·2)	23·2 (22·2 to 24·3)	1·2 (0·8 to 1·7)	10·6 (10·6 to 10·6)	159·4 (153·4 to 165·9)	47·1 (42·7 to 52·0)	74·4 (71·2 to 77·8)	3·9 (2·7 to 5·5)	34·0 (34·0 to 34·0)
Laos*	2015	54·1 (52·1 to 56·1)	17·3 (15·8 to 18·9)	24·6 (23·6 to 25·7)	1·3 (0·9 to 1·8)	10·9 (10·9 to 10·9)	173·2 (167·0 to 179·5)	55·4 (50·7 to 60·6)	78·9 (75·7 to 82·4)	4·1 (2·8 to 5·9)	34·9 (34·9 to 34·9)
Laos*	2016	51·7 (49·9 to 53·6)	17·9 (16·4 to 19·4)	25·2 (24·2 to 26·3)	1·2 (0·8 to 1·8)	7·3 (7·3 to 7·3)	165·5 (159·9 to 171·6)	57·2 (52·5 to 62·0)	80·8 (77·6 to 84·3)	3·9 (2·7 to 5·7)	23·5 (23·5 to 23·5)
Laos*	2017	57·1 (55·1 to 59·2)	20·6 (18·9 to 22·4)	27·2 (26·2 to 28·2)	0·9 (0·6 to 1·3)	8·5 (8·5 to 8·5)	183·0 (176·6 to 189·6)	65·9 (60·5 to 71·8)	87·0 (84·0 to 90·4)	3·0 (2·0 to 4·2)	27·1 (27·1 to 27·1)
Laos*	2018	62·5 (59·9 to 64·9)	21·9 (19·8 to 24·0)	27·7 (26·5 to 29·1)	0·4 (0·3 to 0·6)	12·4 (12·4 to 12·4)	200·0 (191·9 to 207·7)	70·0 (63·5 to 77·0)	88·9 (84·8 to 93·3)	1·4 (0·9 to 1·9)	39·8 (39·8 to 39·8)
Laos*	2019	63·0 (60·4 to 65·7)	22·0 (19·8 to 24·5)	28·7 (27·4 to 30·1)	0·4 (0·3 to 0·6)	11·9 (11·8 to 12·1)	201·8 (193·4 to 210·5)	70·5 (63·6 to 78·5)	91·8 (87·6 to 96·4)	1·4 (1·0 to 2·0)	38·1 (37·7 to 38·8)

Laos*	2020	64·7 (61·9 to 67·5)	21·7 (19·6 to 24·2)	28·0 (26·7 to 29·3)	0·4 (0·3 to 0·6)	14·6 (14·0 to 15·3)	207·2 (198·1 to 216·3)	69·5 (62·6 to 77·4)	89·5 (85·5 to 93·9)	1·4 (1·0 to 2·0)	46·7 (44·8 to 49·1)
Laos*	2021	60·4 (57·7 to 63·1)	19·3 (17·4 to 21·5)	28·1 (26·8 to 29·4)	0·5 (0·3 to 0·6)	12·6 (11·8 to 13·4)	193·5 (184·7 to 202·1)	61·7 (55·6 to 68·7)	89·8 (85·8 to 94·2)	1·5 (1·0 to 2·1)	40·5 (37·8 to 43·0)
Laos*	2022	58·2 (55·6 to 60·9)	19·5 (17·6 to 21·8)	28·6 (27·3 to 30·0)	0·5 (0·3 to 0·7)	9·6 (8·8 to 10·5)	186·5 (177·9 to 195·0)	62·6 (56·2 to 69·9)	91·6 (87·5 to 96·0)	1·5 (1·0 to 2·2)	30·8 (28·0 to 33·5)
Laos*	2023	59·2 (56·3 to 61·9)	19·8 (17·7 to 22·3)	29·2 (27·9 to 30·6)	0·5 (0·3 to 0·7)	9·6 (8·7 to 10·7)	189·4 (180·4 to 198·3)	63·5 (56·8 to 71·4)	93·5 (89·3 to 98·0)	1·6 (1·1 to 2·2)	30·9 (27·8 to 34·1)
Laos*	2024	60·1 (57·1 to 63·1)	20·1 (18·0 to 22·6)	29·8 (28·4 to 31·2)	0·5 (0·3 to 0·7)	9·7 (8·6 to 10·9)	192·4 (183·0 to 202·2)	64·4 (57·6 to 72·5)	95·3 (91·0 to 100·0)	1·7 (1·1 to 2·3)	31·0 (27·6 to 34·8)
Laos*	2025	60·9 (57·8 to 64·0)	20·4 (18·1 to 23·0)	30·3 (29·0 to 31·8)	0·5 (0·4 to 0·8)	9·6 (8·5 to 10·8)	194·9 (185·0 to 204·9)	65·3 (58·0 to 73·5)	97·1 (92·7 to 101·9)	1·7 (1·2 to 2·5)	30·8 (27·1 to 34·6)
Laos*	2026	61·6 (58·4 to 64·9)	20·7 (18·3 to 23·3)	30·9 (29·5 to 32·4)	0·6 (0·4 to 0·8)	9·5 (8·2 to 10·8)	197·3 (187·0 to 207·7)	66·2 (58·5 to 74·5)	98·9 (94·4 to 103·7)	1·8 (1·2 to 2·6)	30·4 (26·4 to 34·6)
Laos*	2027	62·4 (59·0 to 65·8)	20·9 (18·4 to 23·6)	31·5 (30·0 to 33·0)	0·6 (0·4 to 0·8)	9·4 (8·1 to 10·8)	199·8 (188·9 to 210·6)	67·0 (58·9 to 75·7)	100·8 (96·2 to 105·7)	1·9 (1·3 to 2·7)	30·0 (25·8 to 34·5)
Laos*	2028	63·2 (59·6 to 66·7)	21·2 (18·5 to 24·0)	32·1 (30·6 to 33·7)	0·6 (0·4 to 0·9)	9·2 (7·9 to 10·7)	202·3 (190·9 to 213·6)	67·9 (59·4 to 77·0)	102·7 (98·0 to 107·8)	2·0 (1·4 to 2·9)	29·6 (25·2 to 34·3)
Laos*	2029	63·9 (60·2 to 67·7)	21·5 (18·7 to 24·4)	32·7 (31·2 to 34·3)	0·7 (0·4 to 0·9)	9·1 (7·7 to 10·7)	204·8 (192·9 to 216·7)	68·8 (59·8 to 78·2)	104·6 (99·8 to 109·8)	2·1 (1·4 to 3·0)	29·3 (24·7 to 34·3)
Laos*	2030	64·7 (60·9 to 68·8)	21·8 (18·8 to 24·8)	33·3 (31·7 to 34·9)	0·7 (0·5 to 1·0)	9·0 (7·5 to 10·6)	207·3 (194·9 to 220·2)	69·7 (60·2 to 79·6)	106·6 (101·6 to 111·8)	2·2 (1·5 to 3·1)	28·8 (24·0 to 34·0)
Laos*	2031	65·5 (61·4 to 69·8)	22·0 (19·0 to 25·3)	33·9 (32·3 to 35·6)	0·7 (0·5 to 1·0)	8·9 (7·3 to 10·5)	209·9 (196·8 to 223·7)	70·6 (60·8 to 81·0)	108·6 (103·5 to 113·9)	2·3 (1·6 to 3·3)	28·4 (23·4 to 33·8)
Laos*	2032	66·4 (62·2 to 70·8)	22·4 (19·1 to 25·8)	34·6 (33·0 to 36·2)	0·7 (0·5 to 1·1)	8·7 (7·1 to 10·4)	212·6 (199·2 to 226·8)	71·6 (61·1 to 82·7)	110·7 (105·5 to 116·1)	2·4 (1·6 to 3·4)	28·0 (22·8 to 33·4)
Laos*	2033	67·3 (62·9 to 72·0)	22·7 (19·2 to 26·3)	35·2 (33·6 to 37·0)	0·8 (0·5 to 1·1)	8·6 (6·9 to 10·4)	215·6 (201·5 to 230·4)	72·7 (61·6 to 84·3)	112·9 (107·6 to 118·4)	2·5 (1·7 to 3·6)	27·5 (22·2 to 33·3)

Laos*	2034	68·3 (63·6 to 73·1)	23·1 (19·5 to 26·9)	36·0 (34·3 to 37·7)	0·8 (0·5 to 1·2)	8·4 (6·8 to 10·3)	218·8 (203·6 to 234·2)	74·0 (62·3 to 86·2)	115·2 (109·7 to 120·7)	2·6 (1·8 to 3·8)	27·0 (21·6 to 33·1)
Laos*	2035	69·4 (64·5 to 74·4)	23·5 (19·7 to 27·5)	36·7 (35·0 to 38·5)	0·9 (0·6 to 1·2)	8·3 (6·6 to 10·2)	222·2 (206·5 to 238·4)	75·3 (63·2 to 88·2)	117·6 (111·9 to 123·3)	2·7 (1·8 to 3·9)	26·5 (21·0 to 32·8)
Laos*	2036	70·5 (65·4 to 75·6)	23·9 (20·0 to 28·2)	37·5 (35·7 to 39·3)	0·9 (0·6 to 1·3)	8·1 (6·4 to 10·2)	225·7 (209·6 to 242·2)	76·7 (64·0 to 90·5)	120·1 (114·2 to 125·9)	2·9 (1·9 to 4·1)	26·0 (20·4 to 32·6)
Laos*	2037	71·6 (66·4 to 77·1)	24·4 (20·3 to 29·0)	38·3 (36·4 to 40·1)	0·9 (0·6 to 1·4)	8·0 (6·2 to 10·1)	229·3 (212·7 to 247·0)	78·2 (65·0 to 92·8)	122·6 (116·5 to 128·5)	3·0 (2·0 to 4·3)	25·6 (19·8 to 32·2)
Laos*	2038	72·8 (67·4 to 78·5)	24·9 (20·6 to 29·7)	39·1 (37·1 to 41·0)	1·0 (0·6 to 1·4)	7·9 (6·0 to 10·0)	233·1 (215·8 to 251·4)	79·7 (65·9 to 95·1)	125·1 (118·9 to 131·2)	3·1 (2·1 to 4·5)	25·2 (19·3 to 32·0)
Laos*	2039	74·0 (68·3 to 79·9)	25·4 (20·9 to 30·4)	39·8 (37·8 to 41·8)	1·0 (0·7 to 1·5)	7·8 (5·9 to 10·0)	237·0 (218·8 to 255·8)	81·3 (67·0 to 97·2)	127·6 (121·2 to 133·9)	3·3 (2·2 to 4·7)	24·9 (18·9 to 32·1)
Laos*	2040	75·3 (69·4 to 81·3)	25·9 (21·3 to 31·0)	40·6 (38·5 to 42·6)	1·1 (0·7 to 1·5)	7·7 (5·7 to 10·1)	241·0 (222·2 to 260·3)	82·8 (68·2 to 99·3)	130·0 (123·4 to 136·4)	3·4 (2·2 to 5·0)	24·7 (18·4 to 32·4)
Laos*	2041	76·5 (70·4 to 83·0)	26·4 (21·7 to 31·8)	41·4 (39·2 to 43·4)	1·1 (0·7 to 1·6)	7·7 (5·7 to 10·2)	245·1 (225·3 to 265·7)	84·5 (69·4 to 101·8)	132·5 (125·6 to 139·1)	3·6 (2·3 to 5·2)	24·6 (18·2 to 32·7)
Laos*	2042	77·8 (71·4 to 84·5)	26·9 (22·0 to 32·6)	42·1 (39·9 to 44·2)	1·2 (0·8 to 1·7)	7·7 (5·6 to 10·3)	249·2 (228·6 to 270·5)	86·2 (70·6 to 104·4)	134·8 (127·8 to 141·6)	3·7 (2·4 to 5·4)	24·5 (17·9 to 32·9)
Laos*	2043	79·2 (72·5 to 86·0)	27·5 (22·4 to 33·4)	42·8 (40·6 to 45·1)	1·2 (0·8 to 1·8)	7·6 (5·5 to 10·3)	253·5 (232·0 to 275·4)	88·0 (71·9 to 107·0)	137·2 (130·2 to 144·3)	3·9 (2·5 to 5·7)	24·5 (17·8 to 33·1)
Laos*	2044	80·6 (73·7 to 87·6)	28·0 (22·9 to 34·2)	43·6 (41·3 to 45·9)	1·3 (0·8 to 1·9)	7·7 (5·5 to 10·4)	258·0 (236·1 to 280·7)	89·8 (73·2 to 109·6)	139·6 (132·3 to 147·0)	4·1 (2·6 to 6·0)	24·5 (17·6 to 33·3)
Laos*	2045	81·9 (74·9 to 89·1)	28·6 (23·3 to 35·1)	44·3 (42·0 to 46·7)	1·3 (0·9 to 2·0)	7·7 (5·5 to 10·4)	262·4 (239·9 to 285·3)	91·7 (74·6 to 112·3)	141·9 (134·4 to 149·4)	4·2 (2·7 to 6·2)	24·6 (17·6 to 33·4)
Laos*	2046	83·4 (76·1 to 91·1)	29·2 (23·8 to 35·9)	45·0 (42·6 to 47·5)	1·4 (0·9 to 2·0)	7·7 (5·4 to 10·5)	267·0 (243·6 to 291·8)	93·7 (76·1 to 115·1)	144·2 (136·4 to 152·0)	4·4 (2·8 to 6·5)	24·8 (17·5 to 33·8)
Laos*	2047	84·8 (77·3 to 92·6)	29·9 (24·2 to 36·8)	45·7 (43·3 to 48·3)	1·4 (0·9 to 2·1)	7·8 (5·4 to 10·7)	271·7 (247·7 to 296·4)	95·7 (77·6 to 117·9)	146·4 (138·5 to 154·5)	4·6 (2·9 to 6·8)	25·0 (17·4 to 34·2)

Laos*	2048	86·3 (78·6 to 94·6)	30·5 (24·7 to 37·7)	46·4 (43·9 to 49·0)	1·5 (0·9 to 2·2)	7·9 (5·4 to 11·0)	276·4 (251·9 to 303·0)	97·8 (79·2 to 120·7)	148·6 (140·5 to 157·0)	4·8 (3·0 to 7·1)	25·2 (17·4 to 35·3)
Laos*	2049	87·8 (79·9 to 96·1)	31·2 (25·3 to 38·5)	47·1 (44·5 to 49·8)	1·6 (1·0 to 2·3)	8·0 (5·5 to 11·3)	281·2 (255·9 to 307·8)	99·9 (80·9 to 123·4)	150·7 (142·4 to 159·5)	5·0 (3·1 to 7·5)	25·6 (17·6 to 36·1)
Laos*	2050	89·3 (81·2 to 98·1)	31·9 (25·8 to 39·4)	47·7 (45·1 to 50·6)	1·6 (1·0 to 2·4)	8·1 (5·5 to 11·6)	286·1 (260·1 to 314·1)	102·1 (82·5 to 126·3)	152·8 (144·3 to 161·9)	5·2 (3·2 to 7·8)	26·0 (17·7 to 37·1)
Lebanon*	1995	503·2 (475·4 to 536·5)	181·4 (164·0 to 200·9)	257·8 (236·7 to 284·0)	57·4 (45·7 to 72·1)	6·7 (6·7 to 6·7)	2119·9 (2002·5 to 2259·9)	764·0 (690·8 to 846·3)	1085·8 (997·3 to 1196·2)	241·8 (192·3 to 303·8)	28·2 (28·2 to 28·2)
Lebanon*	1996	552·9 (525·1 to 583·9)	212·4 (194·8 to 232·0)	276·6 (258·1 to 298·4)	63·1 (51·3 to 77·5)	0·8 (0·8 to 0·8)	2329·1 (2211·9 to 2459·6)	894·8 (820·8 to 977·2)	1165·1 (1087·4 to 1257·0)	265·8 (215·9 to 326·6)	3·4 (3·4 to 3·4)
Lebanon*	1997	534·5 (506·8 to 562·9)	196·3 (180·1 to 214·2)	275·0 (257·6 to 292·9)	61·5 (50·9 to 74·0)	1·6 (1·6 to 1·6)	2251·5 (2134·8 to 2371·3)	827·0 (758·5 to 902·3)	1158·6 (1085·0 to 1233·8)	259·2 (214·5 to 311·5)	6·7 (6·7 to 6·7)
Lebanon*	1998	487·3 (463·0 to 514·4)	159·5 (145·9 to 174·1)	268·2 (250·8 to 285·3)	57·5 (47·5 to 68·5)	2·2 (2·2 to 2·2)	2052·9 (1950·3 to 2166·8)	671·8 (614·5 to 733·4)	1129·9 (1056·7 to 1201·7)	242·2 (200·0 to 288·4)	9·1 (9·1 to 9·1)
Lebanon*	1999	457·2 (433·9 to 482·1)	141·7 (128·7 to 155·3)	257·5 (240·3 to 275·0)	54·7 (45·1 to 66·0)	3·3 (3·3 to 3·3)	1925·9 (1827·8 to 2031·0)	596·9 (542·1 to 654·1)	1084·6 (1012·2 to 1158·5)	230·5 (189·9 to 278·1)	13·9 (13·9 to 13·9)
Lebanon*	2000	444·7 (420·6 to 469·3)	142·0 (128·5 to 156·1)	247·1 (229·6 to 265·2)	53·2 (43·1 to 64·5)	2·5 (2·5 to 2·5)	1873·4 (1771·9 to 1976·9)	598·0 (541·2 to 657·7)	1040·9 (967·3 to 1117·3)	224·1 (181·5 to 271·8)	10·4 (10·4 to 10·4)
Lebanon*	2001	410·7 (387·8 to 433·9)	132·4 (119·8 to 146·2)	224·9 (207·8 to 242·8)	50·5 (41·1 to 61·6)	2·9 (2·9 to 2·9)	1730·1 (1633·8 to 1827·8)	557·9 (504·6 to 615·7)	947·5 (875·4 to 1023·0)	212·7 (173·0 to 259·5)	12·0 (12·0 to 12·0)
Lebanon*	2002	380·8 (359·0 to 403·2)	126·5 (114·1 to 140·3)	202·9 (187·4 to 219·2)	48·1 (39·3 to 58·4)	3·3 (3·3 to 3·3)	1604·0 (1512·3 to 1698·7)	532·8 (480·8 to 591·0)	854·8 (789·4 to 923·3)	202·5 (165·4 to 246·0)	13·9 (13·9 to 13·9)
Lebanon*	2003	362·2 (341·7 to 384·0)	125·6 (113·3 to 138·4)	184·9 (170·6 to 199·4)	46·8 (37·8 to 56·6)	4·9 (4·9 to 4·9)	1525·9 (1439·4 to 1617·4)	529·1 (477·3 to 583·2)	779·0 (718·7 to 840·2)	197·1 (159·2 to 238·2)	20·8 (20·8 to 20·8)
Lebanon*	2004	346·7 (326·1 to 367·0)	126·6 (114·6 to 139·3)	171·1 (157·6 to 184·8)	46·2 (37·6 to 55·8)	2·8 (2·8 to 2·8)	1460·6 (1373·7 to 1546·1)	533·3 (482·9 to 587·0)	720·8 (663·9 to 778·4)	194·6 (158·2 to 235·0)	12·0 (12·0 to 12·0)
Lebanon*	2005	339·2 (319·8 to 359·3)	128·8 (116·2 to 142·4)	163·0 (151·1 to 176·5)	46·5 (38·1 to 56·0)	0·9 (0·9 to 0·9)	1429·0 (1347·1 to 1513·6)	542·5 (489·6 to 600·0)	686·7 (636·3 to 743·3)	196·0 (160·6 to 236·0)	3·9 (3·9 to 3·9)

Lebanon*	2006	366·6 (344·3 to 388·1)	145·6 (132·3 to 160·8)	168·1 (155·3 to 182·0)	50·9 (41·6 to 61·1)	2·0 (2·0 to 2·0)	1544·5 (1450·4 to 1634·7)	613·5 (557·2 to 677·5)	708·1 (654·1 to 766·7)	214·2 (175·3 to 257·2)	8·6 (8·6 to 8·6)
Lebanon*	2007	384·7 (361·1 to 407·7)	154·3 (140·3 to 169·1)	172·2 (157·8 to 187·7)	54·4 (44·8 to 65·7)	3·8 (3·8 to 3·8)	1620·5 (1521·3 to 1717·6)	649·8 (590·9 to 712·5)	725·4 (664·7 to 790·8)	229·2 (188·8 to 277·0)	16·1 (16·1 to 16·1)
Lebanon*	2008	402·5 (379·8 to 427·5)	161·0 (146·5 to 177·3)	175·1 (159·8 to 190·5)	57·8 (48·0 to 69·2)	8·6 (8·6 to 8·6)	1695·6 (1600·0 to 1800·8)	678·2 (617·1 to 746·8)	737·7 (673·2 to 802·4)	243·4 (202·3 to 291·7)	36·3 (36·3 to 36·3)
Lebanon*	2009	413·7 (389·8 to 439·3)	171·8 (156·7 to 189·4)	177·9 (162·9 to 194·2)	62·1 (51·8 to 73·4)	2·0 (2·0 to 2·0)	1742·7 (1641·9 to 1850·6)	723·7 (660·2 to 797·7)	749·3 (686·2 to 818·3)	261·4 (218·3 to 309·0)	8·3 (8·3 to 8·3)
Lebanon*	2010	428·7 (405·1 to 455·1)	181·6 (165·9 to 197·8)	177·1 (162·7 to 192·9)	67·0 (56·4 to 78·9)	2·9 (2·9 to 2·9)	1805·8 (1706·5 to 1916·9)	765·0 (698·9 to 833·1)	746·2 (685·5 to 812·4)	282·3 (237·7 to 332·3)	12·3 (12·3 to 12·3)
Lebanon*	2011	424·3 (400·7 to 449·6)	188·7 (173·5 to 205·5)	165·1 (151·5 to 178·8)	68·5 (57·8 to 81·0)	2·0 (2·0 to 2·0)	1787·3 (1687·7 to 1894·1)	794·7 (730·8 to 865·5)	695·6 (638·3 to 753·2)	288·4 (243·4 to 341·3)	8·6 (8·6 to 8·6)
Lebanon*	2012	411·4 (388·7 to 435·0)	196·1 (181·3 to 213·3)	146·6 (133·2 to 159·8)	66·3 (56·3 to 77·8)	2·3 (2·3 to 2·3)	1733·0 (1637·2 to 1832·3)	826·0 (763·8 to 898·7)	617·7 (561·3 to 673·1)	279·4 (237·1 to 327·7)	9·9 (9·9 to 9·9)
Lebanon*	2013	384·8 (363·8 to 408·0)	188·9 (173·9 to 204·3)	132·6 (120·2 to 145·2)	61·7 (52·2 to 73·0)	1·7 (1·7 to 1·7)	1621·2 (1532·4 to 1718·8)	795·7 (732·7 to 860·5)	558·6 (506·4 to 611·5)	259·9 (219·9 to 307·5)	7·0 (7·0 to 7·0)
Lebanon*	2014	386·7 (364·7 to 410·5)	190·7 (175·2 to 205·5)	129·3 (116·4 to 142·5)	60·7 (51·1 to 71·8)	6·0 (6·0 to 6·0)	1629·0 (1536·5 to 1729·3)	803·2 (738·1 to 865·5)	544·6 (490·4 to 600·4)	255·8 (215·2 to 302·5)	25·3 (25·3 to 25·3)
Lebanon*	2015	380·0 (357·2 to 402·8)	189·3 (174·3 to 204·6)	127·2 (114·7 to 140·7)	60·4 (49·8 to 71·3)	3·0 (3·0 to 3·0)	1600·8 (1504·6 to 1697·0)	797·6 (734·4 to 862·1)	536·0 (483·3 to 592·5)	254·4 (210·0 to 300·5)	12·7 (12·7 to 12·7)
Lebanon*	2016	396·7 (373·1 to 419·9)	200·0 (184·0 to 216·0)	131·0 (118·4 to 144·3)	63·0 (52·5 to 73·9)	2·8 (2·8 to 2·8)	1671·3 (1571·8 to 1768·9)	842·6 (775·1 to 909·9)	551·8 (498·6 to 608·1)	265·2 (221·1 to 311·5)	11·7 (11·7 to 11·7)
Lebanon*	2017	416·2 (390·9 to 441·6)	211·2 (195·8 to 228·7)	135·8 (122·2 to 149·0)	65·7 (54·0 to 78·9)	3·4 (3·4 to 3·4)	1753·2 (1646·7 to 1860·0)	889·7 (825·0 to 963·3)	572·2 (514·7 to 627·5)	276·9 (227·6 to 332·6)	14·3 (14·3 to 14·3)
Lebanon*	2018	422·1 (393·9 to 452·3)	213·3 (195·6 to 231·8)	137·9 (122·5 to 152·9)	66·9 (53·7 to 82·6)	4·0 (4·0 to 4·0)	1778·0 (1659·4 to 1905·2)	898·4 (824·0 to 976·4)	580·9 (516·1 to 644·1)	281·7 (226·2 to 347·9)	17·0 (17·0 to 17·0)
Lebanon*	2019	394·9 (367·3 to 424·6)	195·4 (176·8 to 213·9)	130·0 (115·5 to 144·0)	64·3 (51·6 to 79·4)	5·3 (5·2 to 5·5)	1663·4 (1547·4 to 1788·8)	822·9 (744·8 to 901·0)	547·5 (486·5 to 606·7)	270·7 (217·5 to 334·5)	22·3 (21·9 to 23·3)

Lebanon*	2020	350·6 (324·9 to 377·7)	162·5 (146·5 to 179·2)	119·6 (106·2 to 132·5)	60·7 (48·6 to 74·7)	7·8 (7·5 to 8·3)	1476·8 (1368·5 to 1590·9)	684·7 (617·0 to 754·8)	503·7 (447·2 to 558·2)	255·6 (204·9 to 314·7)	32·9 (31·8 to 35·0)
Lebanon*	2021	325·7 (301·6 to 350·6)	149·8 (135·0 to 165·2)	109·8 (97·6 to 121·7)	57·1 (46·0 to 70·4)	8·9 (8·4 to 9·5)	1371·8 (1270·6 to 1477·1)	631·1 (568·7 to 695·7)	462·7 (411·0 to 512·6)	240·4 (193·9 to 296·5)	37·6 (35·6 to 39·9)
Lebanon*	2022	327·2 (301·6 to 354·4)	151·0 (133·5 to 168·9)	110·6 (98·2 to 122·5)	58·8 (47·4 to 72·6)	6·9 (6·3 to 7·5)	1378·5 (1270·4 to 1492·8)	635·9 (562·4 to 711·5)	465·9 (413·6 to 516·1)	247·7 (199·6 to 305·8)	28·9 (26·6 to 31·4)
Lebanon*	2023	332·5 (305·6 to 361·5)	153·1 (134·0 to 172·0)	111·7 (99·1 to 123·7)	60·8 (49·1 to 74·9)	6·9 (6·3 to 7·5)	1400·5 (1287·3 to 1522·9)	645·1 (564·6 to 724·5)	470·4 (417·6 to 521·0)	256·0 (206·7 to 315·4)	29·1 (26·6 to 31·8)
Lebanon*	2024	338·0 (308·9 to 368·7)	155·7 (134·9 to 177·1)	112·6 (99·9 to 124·7)	62·7 (50·7 to 77·2)	7·0 (6·3 to 7·6)	1423·7 (1301·4 to 1553·0)	656·0 (568·2 to 745·9)	474·2 (420·8 to 525·2)	264·1 (213·4 to 325·1)	29·4 (26·7 to 32·1)
Lebanon*	2025	344·0 (314·2 to 376·1)	158·6 (136·2 to 182·1)	113·6 (100·8 to 125·9)	64·8 (52·4 to 79·9)	7·0 (6·3 to 7·7)	1449·3 (1323·6 to 1584·4)	668·2 (573·8 to 767·0)	478·6 (424·6 to 530·3)	272·9 (220·8 to 336·4)	29·5 (26·7 to 32·5)
Lebanon*	2026	350·3 (319·9 to 383·7)	161·6 (136·9 to 185·8)	114·7 (101·8 to 126·9)	67·0 (54·0 to 82·6)	7·0 (6·3 to 7·8)	1475·6 (1347·6 to 1616·3)	680·8 (576·7 to 782·7)	483·0 (428·8 to 534·8)	282·1 (227·3 to 347·9)	29·7 (26·7 to 32·7)
Lebanon*	2027	356·1 (324·2 to 389·9)	164·2 (139·4 to 189·4)	115·7 (102·7 to 128·0)	69·2 (55·7 to 85·4)	7·1 (6·4 to 7·9)	1500·0 (1365·9 to 1642·6)	691·6 (587·1 to 797·9)	487·2 (432·5 to 539·1)	291·4 (234·6 to 359·8)	29·8 (26·8 to 33·2)
Lebanon*	2028	361·6 (328·1 to 396·6)	166·6 (140·9 to 193·2)	116·5 (103·4 to 129·0)	71·4 (57·4 to 88·2)	7·2 (6·4 to 8·0)	1523·2 (1382·2 to 1670·7)	701·7 (593·5 to 813·9)	490·7 (435·6 to 543·3)	300·6 (241·9 to 371·5)	30·2 (26·8 to 33·7)
Lebanon*	2029	367·4 (332·5 to 404·9)	169·1 (142·5 to 198·1)	117·4 (104·2 to 130·0)	73·7 (59·3 to 91·1)	7·2 (6·4 to 8·1)	1547·5 (1400·7 to 1705·6)	712·1 (600·1 to 834·3)	494·6 (439·1 to 547·8)	310·3 (249·9 to 383·6)	30·5 (26·8 to 34·2)
Lebanon*	2030	373·1 (337·6 to 411·9)	171·5 (144·2 to 201·9)	118·3 (105·0 to 131·0)	76·0 (61·1 to 93·9)	7·3 (6·4 to 8·2)	1571·6 (1422·3 to 1735·2)	722·3 (607·4 to 850·4)	498·4 (442·4 to 551·7)	320·2 (257·5 to 395·7)	30·8 (27·0 to 34·7)
Lebanon*	2031	378·9 (341·8 to 419·4)	173·9 (145·1 to 205·1)	119·2 (105·8 to 132·0)	78·4 (63·1 to 96·8)	7·4 (6·4 to 8·4)	1596·2 (1439·8 to 1766·6)	732·6 (611·3 to 864·0)	502·2 (445·5 to 556·0)	330·3 (265·9 to 407·9)	31·1 (27·1 to 35·3)
Lebanon*	2032	384·6 (347·4 to 426·2)	176·2 (146·6 to 208·0)	120·1 (106·4 to 132·9)	80·9 (64·9 to 99·9)	7·5 (6·5 to 8·6)	1620·1 (1463·6 to 1795·4)	742·1 (617·7 to 876·2)	505·9 (448·3 to 559·8)	340·6 (273·4 to 420·9)	31·5 (27·4 to 36·2)
Lebanon*	2033	390·2 (351·5 to 432·3)	178·4 (147·1 to 210·7)	120·9 (107·1 to 134·0)	83·3 (66·6 to 103·5)	7·6 (6·6 to 8·8)	1643·8 (1480·7 to 1821·0)	751·4 (619·6 to 887·6)	509·2 (451·3 to 564·3)	351·1 (280·6 to 436·0)	32·0 (27·7 to 37·2)

Lebanon*	2034	395·5 (355·0 to 439·3)	180·3 (148·6 to 213·6)	121·6 (107·7 to 135·1)	85·8 (68·4 to 106·4)	7·8 (6·7 to 9·1)	1665·9 (1495·5 to 1850·6)	759·4 (625·9 to 899·7)	512·3 (453·7 to 568·9)	361·5 (288·0 to 448·1)	32·7 (28·1 to 38·4)
Lebanon*	2035	400·2 (358·4 to 445·3)	182·0 (150·0 to 216·0)	122·0 (108·0 to 135·6)	88·2 (70·0 to 109·1)	8·0 (6·8 to 9·5)	1685·8 (1509·6 to 1875·7)	766·6 (631·7 to 909·7)	514·1 (455·0 to 571·3)	371·4 (295·0 to 459·7)	33·7 (28·6 to 40·1)
Lebanon*	2036	404·7 (362·2 to 451·1)	183·6 (150·0 to 220·3)	122·3 (108·3 to 136·2)	90·5 (71·9 to 112·4)	8·3 (7·0 to 10·1)	1704·8 (1525·7 to 1900·3)	773·6 (631·9 to 928·0)	515·3 (456·3 to 573·7)	381·0 (302·8 to 473·4)	34·8 (29·4 to 42·4)
Lebanon*	2037	408·1 (364·2 to 455·7)	184·6 (150·7 to 221·1)	122·3 (108·3 to 136·3)	92·6 (72·9 to 115·8)	8·6 (7·3 to 10·7)	1719·3 (1534·4 to 1919·4)	777·7 (634·7 to 931·3)	515·3 (456·0 to 574·1)	390·0 (307·3 to 487·8)	36·3 (30·6 to 45·1)
Lebanon*	2038	411·2 (366·3 to 461·2)	185·4 (149·5 to 222·5)	122·1 (108·2 to 136·5)	94·6 (73·9 to 118·7)	9·1 (7·5 to 11·5)	1732·0 (1542·9 to 1942·8)	780·8 (629·8 to 937·1)	514·5 (455·8 to 575·0)	398·5 (311·2 to 499·9)	38·2 (31·7 to 48·5)
Lebanon*	2039	413·6 (367·8 to 465·3)	185·8 (149·0 to 225·0)	121·7 (107·9 to 136·3)	96·5 (75·4 to 121·1)	9·6 (8·0 to 12·3)	1742·2 (1549·4 to 1960·1)	782·8 (627·8 to 947·9)	512·6 (454·6 to 574·0)	406·4 (317·6 to 510·3)	40·4 (33·5 to 52·0)
Lebanon*	2040	415·4 (368·7 to 468·0)	185·9 (149·6 to 225·0)	121·1 (107·3 to 136·0)	98·2 (76·5 to 123·5)	10·2 (8·4 to 13·3)	1750·0 (1553·2 to 1971·5)	783·0 (630·2 to 947·9)	510·1 (451·8 to 572·8)	413·8 (322·2 to 520·1)	43·1 (35·4 to 56·1)
Lebanon*	2041	417·2 (370·2 to 471·0)	185·9 (149·7 to 225·0)	120·4 (106·6 to 135·2)	100·0 (77·9 to 125·8)	10·9 (8·9 to 14·5)	1757·4 (1559·5 to 1984·0)	782·9 (630·8 to 947·8)	507·2 (448·9 to 569·6)	421·1 (328·0 to 529·9)	46·1 (37·6 to 61·0)
Lebanon*	2042	418·7 (371·4 to 472·6)	185·6 (149·0 to 225·0)	119·7 (105·9 to 134·2)	101·7 (78·9 to 128·1)	11·7 (9·4 to 15·8)	1763·7 (1564·5 to 1990·7)	781·7 (627·7 to 947·8)	504·2 (445·9 to 565·5)	428·5 (332·2 to 539·6)	49·4 (39·6 to 66·6)
Lebanon*	2043	421·0 (372·6 to 476·3)	185·8 (148·3 to 227·4)	119·1 (105·5 to 133·9)	103·6 (80·1 to 131·1)	12·5 (10·0 to 17·2)	1773·3 (1569·6 to 2006·4)	782·5 (624·9 to 957·9)	501·7 (444·2 to 563·9)	436·4 (337·5 to 552·0)	52·7 (41·9 to 72·5)
Lebanon*	2044	423·2 (374·3 to 479·9)	185·8 (147·3 to 226·3)	118·5 (104·8 to 133·2)	105·5 (81·3 to 134·4)	13·4 (10·5 to 19·0)	1782·7 (1576·7 to 2021·5)	782·6 (620·4 to 953·2)	499·2 (441·5 to 561·3)	444·6 (342·6 to 566·1)	56·3 (44·2 to 80·0)
Lebanon*	2045	425·9 (376·1 to 485·4)	186·1 (147·9 to 228·3)	118·0 (104·2 to 132·9)	107·6 (83·0 to 137·6)	14·2 (11·1 to 20·6)	1794·1 (1584·3 to 2044·6)	784·0 (623·0 to 961·8)	497·0 (439·1 to 559·7)	453·2 (349·7 to 579·6)	59·9 (46·8 to 86·6)
Lebanon*	2046	428·4 (377·2 to 489·6)	186·3 (147·2 to 230·3)	117·3 (103·5 to 132·4)	109·5 (84·2 to 140·4)	15·2 (11·8 to 22·3)	1804·6 (1588·9 to 2062·5)	784·8 (620·2 to 970·0)	494·2 (436·1 to 557·9)	461·4 (354·6 to 591·6)	64·2 (49·6 to 94·0)
Lebanon*	2047	430·7 (379·3 to 493·3)	186·3 (146·6 to 227·9)	116·6 (102·8 to 132·0)	111·5 (85·5 to 143·5)	16·3 (12·5 to 24·4)	1814·4 (1598·0 to 2078·1)	784·7 (617·6 to 960·0)	491·3 (433·3 to 556·1)	469·5 (360·0 to 604·4)	68·8 (52·8 to 102·6)

Lebanon*	2048	432·6 (380·3 to 495·1)	186·1 (144·6 to 228·5)	115·7 (102·0 to 131·0)	113·2 (86·4 to 146·6)	17·6 (13·3 to 26·6)	1822·4 (1601·9 to 2085·8)	783·9 (609·3 to 962·5)	487·4 (429·5 to 552·0)	476·9 (363·9 to 617·6)	74·3 (56·1 to 112·2)
Lebanon*	2049	434·6 (382·0 to 496·8)	185·7 (145·6 to 227·8)	114·8 (101·1 to 130·2)	115·0 (87·4 to 149·1)	19·0 (14·1 to 29·1)	1830·6 (1609·0 to 2092·7)	782·4 (613·3 to 959·8)	483·7 (425·9 to 548·6)	484·4 (368·0 to 628·2)	80·2 (59·5 to 122·7)
Lebanon*	2050	436·0 (381·6 to 500·6)	185·0 (144·1 to 229·2)	113·7 (100·0 to 129·0)	116·6 (88·2 to 152·0)	20·7 (15·3 to 32·2)	1836·8 (1607·7 to 2108·7)	779·5 (606·9 to 965·6)	479·1 (421·4 to 543·3)	491·0 (371·4 to 640·5)	87·2 (64·4 to 135·5)
Liberia*	1995	13·9 (11·5 to 16·7)	2·1 (1·7 to 2·5)	10·5 (8·1 to 13·3)	1·0 (0·7 to 1·4)	0·3 (0·3 to 0·3)	32·7 (27·0 to 39·1)	4·9 (4·0 to 5·9)	24·6 (18·9 to 31·2)	2·3 (1·6 to 3·2)	0·8 (0·8 to 0·8)
Liberia*	1996	12·4 (10·5 to 14·8)	2·0 (1·6 to 2·4)	9·0 (7·0 to 11·2)	0·7 (0·5 to 1·0)	0·8 (0·8 to 0·8)	29·2 (24·6 to 34·7)	4·6 (3·7 to 5·5)	21·1 (16·5 to 26·3)	1·7 (1·1 to 2·4)	1·8 (1·8 to 1·8)
Liberia*	1997	14·0 (12·0 to 16·3)	2·4 (2·0 to 2·9)	9·0 (7·1 to 11·3)	0·6 (0·4 to 0·9)	1·9 (1·9 to 1·9)	32·9 (28·3 to 38·3)	5·7 (4·7 to 6·9)	21·2 (16·8 to 26·6)	1·5 (1·0 to 2·2)	4·5 (4·5 to 4·5)
Liberia*	1998	17·6 (15·8 to 19·8)	2·5 (2·0 to 3·0)	8·6 (6·9 to 10·8)	0·6 (0·4 to 0·8)	6·0 (6·0 to 6·0)	41·3 (37·0 to 46·6)	5·8 (4·7 to 7·0)	20·1 (16·1 to 25·4)	1·3 (0·9 to 1·9)	14·1 (14·1 to 14·1)
Liberia*	1999	14·2 (12·4 to 16·3)	2·5 (2·0 to 3·0)	8·4 (6·7 to 10·4)	0·5 (0·3 to 0·7)	2·9 (2·9 to 2·9)	33·3 (29·2 to 38·2)	5·8 (4·8 to 7·0)	19·6 (15·7 to 24·5)	1·2 (0·8 to 1·6)	6·7 (6·7 to 6·7)
Liberia*	2000	14·8 (13·1 to 16·8)	2·3 (1·9 to 2·7)	7·8 (6·2 to 9·7)	0·5 (0·3 to 0·6)	4·3 (4·3 to 4·3)	34·8 (30·7 to 39·4)	5·4 (4·4 to 6·4)	18·2 (14·5 to 22·7)	1·1 (0·7 to 1·5)	10·1 (10·1 to 10·1)
Liberia*	2001	12·6 (11·0 to 14·4)	2·1 (1·7 to 2·6)	7·5 (6·0 to 9·3)	0·5 (0·3 to 0·6)	2·5 (2·5 to 2·5)	29·5 (25·7 to 33·9)	5·0 (4·1 to 6·0)	17·5 (14·0 to 21·9)	1·1 (0·7 to 1·5)	5·9 (5·9 to 5·9)
Liberia*	2002	13·7 (11·8 to 15·8)	2·3 (1·9 to 2·8)	8·6 (6·8 to 10·6)	0·5 (0·4 to 0·8)	2·2 (2·2 to 2·2)	32·1 (27·7 to 37·1)	5·4 (4·5 to 6·5)	20·2 (16·0 to 24·9)	1·3 (0·9 to 1·8)	5·2 (5·2 to 5·2)
Liberia*	2003	13·3 (11·6 to 15·3)	1·5 (1·2 to 1·8)	8·1 (6·5 to 10·1)	0·4 (0·3 to 0·6)	3·3 (3·3 to 3·3)	31·2 (27·3 to 36·0)	3·5 (2·9 to 4·2)	19·1 (15·2 to 23·6)	1·0 (0·7 to 1·4)	7·7 (7·7 to 7·7)
Liberia*	2004	19·2 (16·9 to 21·9)	1·8 (1·5 to 2·1)	11·3 (9·0 to 14·0)	0·5 (0·4 to 0·8)	5·5 (5·5 to 5·5)	45·0 (39·7 to 51·4)	4·2 (3·4 to 5·0)	26·6 (21·1 to 32·9)	1·3 (0·9 to 1·8)	13·0 (13·0 to 13·0)
Liberia*	2005	21·7 (19·0 to 25·0)	1·8 (1·5 to 2·2)	13·1 (10·4 to 16·3)	0·6 (0·4 to 0·8)	6·2 (6·2 to 6·2)	51·0 (44·5 to 58·6)	4·3 (3·5 to 5·1)	30·8 (24·3 to 38·2)	1·4 (1·0 to 2·0)	14·5 (14·5 to 14·5)
Liberia*	2006	23·3 (20·4 to 26·8)	1·9 (1·5 to 2·2)	13·5 (10·6 to 16·9)	0·6 (0·4 to 0·8)	7·3 (7·3 to 7·3)	54·7 (47·8 to 62·9)	4·3 (3·5 to 5·2)	31·8 (24·9 to 39·7)	1·4 (1·0 to 2·0)	17·1 (17·1 to 17·1)
Liberia*	2007	26·6 (23·1 to 30·6)	2·7 (2·2 to 3·2)	16·1 (12·5 to 19·9)	0·8 (0·6 to 1·1)	7·1 (7·1 to 7·1)	62·4 (54·3 to 71·8)	6·3 (5·1 to 7·5)	37·7 (29·4 to 46·7)	1·9 (1·3 to 2·6)	16·6 (16·6 to 16·6)
Liberia*	2008	37·0 (33·5 to 41·1)	3·4 (2·7 to 4·1)	16·7 (13·2 to 20·6)	0·9 (0·6 to 1·3)	16·0 (16·0 to 16·0)	86·9 (78·6 to 96·6)	8·0 (6·5 to 9·7)	39·2 (30·9 to 48·3)	2·2 (1·5 to 3·0)	37·6 (37·6 to 37·6)

Liberia*	2009	45·3 (41·7 to 49·3)	3·5 (2·8 to 4·3)	17·2 (13·7 to 21·1)	1·0 (0·7 to 1·4)	23·5 (23·5 to 23·5)	106·3 (97·9 to 115·7)	8·3 (6·7 to 10·1)	40·3 (32·2 to 49·4)	2·4 (1·7 to 3·4)	55·3 (55·3 to 55·3)
Liberia*	2010	38·4 (34·8 to 42·4)	3·5 (2·9 to 4·3)	17·4 (13·8 to 21·4)	1·1 (0·8 to 1·6)	16·3 (16·3 to 16·3)	90·1 (81·7 to 99·6)	8·3 (6·8 to 10·2)	40·8 (32·4 to 50·2)	2·6 (1·8 to 3·7)	38·3 (38·3 to 38·3)
Liberia*	2011	41·5 (37·6 to 45·3)	4·1 (3·4 to 5·1)	18·6 (14·8 to 22·5)	1·3 (0·9 to 1·9)	17·4 (17·4 to 17·4)	97·4 (88·2 to 106·5)	9·7 (7·9 to 11·9)	43·6 (34·8 to 52·7)	3·1 (2·1 to 4·5)	40·9 (40·9 to 40·9)
Liberia*	2012	47·5 (43·9 to 51·2)	3·9 (3·2 to 4·7)	18·9 (15·5 to 22·6)	1·4 (1·0 to 2·1)	23·2 (23·2 to 23·2)	111·4 (103·0 to 120·1)	9·2 (7·6 to 11·1)	44·4 (36·5 to 53·1)	3·4 (2·3 to 4·8)	54·5 (54·5 to 54·5)
Liberia*	2013	46·7 (43·4 to 50·3)	3·9 (3·2 to 4·7)	20·4 (17·2 to 24·0)	1·7 (1·1 to 2·4)	20·7 (20·7 to 20·7)	109·7 (101·8 to 118·1)	9·2 (7·6 to 11·1)	48·0 (40·4 to 56·3)	3·9 (2·7 to 5·6)	48·6 (48·6 to 48·6)
Liberia*	2014	87·0 (83·9 to 90·1)	4·7 (3·9 to 5·7)	22·7 (19·9 to 25·6)	2·1 (1·4 to 3·1)	57·4 (57·4 to 57·4)	204·1 (196·9 to 211·6)	11·0 (9·1 to 13·3)	53·3 (46·7 to 60·2)	5·0 (3·4 to 7·2)	134·7 (134·7 to 134·7)
Liberia*	2015	129·9 (127·4 to 132·6)	6·1 (5·1 to 7·4)	25·0 (23·0 to 27·0)	3·5 (2·3 to 5·0)	95·2 (95·2 to 95·2)	304·8 (299·1 to 311·2)	14·4 (11·9 to 17·4)	58·7 (53·9 to 63·4)	8·2 (5·4 to 11·6)	223·6 (223·6 to 223·6)
Liberia*	2016	62·7 (60·5 to 65·4)	7·3 (6·1 to 8·7)	25·7 (24·4 to 27·0)	4·3 (2·9 to 6·0)	25·5 (25·5 to 25·5)	147·2 (141·9 to 153·5)	17·1 (14·2 to 20·5)	60·2 (57·3 to 63·4)	10·0 (6·7 to 14·0)	59·8 (59·8 to 59·8)
Liberia*	2017	65·6 (62·7 to 68·8)	8·5 (7·0 to 10·2)	25·7 (23·7 to 27·8)	4·5 (3·1 to 6·5)	26·8 (26·8 to 26·8)	154·0 (147·2 to 161·5)	20·0 (16·5 to 23·9)	60·3 (55·6 to 65·2)	10·6 (7·2 to 15·2)	63·0 (63·0 to 63·0)
Liberia*	2018	61·8 (57·7 to 66·0)	10·0 (8·3 to 11·9)	25·6 (22·7 to 29·0)	4·6 (3·1 to 6·6)	21·6 (21·6 to 21·6)	145·1 (135·5 to 155·0)	23·4 (19·4 to 28·0)	60·1 (53·2 to 68·1)	10·9 (7·4 to 15·5)	50·6 (50·6 to 50·6)
Liberia*	2019	65·8 (61·7 to 70·0)	10·0 (8·3 to 12·0)	24·4 (21·6 to 27·6)	4·5 (3·1 to 6·4)	26·8 (26·2 to 28·1)	154·4 (144·9 to 164·3)	23·5 (19·5 to 28·1)	57·3 (50·8 to 64·9)	10·6 (7·2 to 15·1)	62·9 (61·5 to 66·0)
Liberia*	2020	73·7 (69·4 to 79·5)	9·4 (7·7 to 11·3)	22·9 (20·3 to 26·0)	4·4 (2·9 to 6·2)	37·1 (35·8 to 41·8)	173·1 (162·9 to 186·7)	22·0 (18·2 to 26·5)	53·8 (47·7 to 61·0)	10·2 (6·9 to 14·5)	87·0 (84·1 to 98·0)
Liberia*	2021	73·0 (68·8 to 78·4)	8·9 (7·4 to 10·7)	21·7 (19·2 to 24·6)	4·2 (2·9 to 6·0)	38·1 (36·5 to 42·5)	171·3 (161·6 to 184·0)	20·9 (17·3 to 25·2)	51·0 (45·2 to 57·8)	10·0 (6·7 to 14·1)	89·4 (85·7 to 99·7)
Liberia*	2022	64·8 (60·5 to 69·8)	9·2 (7·6 to 11·1)	21·9 (19·4 to 24·8)	4·4 (2·9 to 6·2)	29·3 (27·1 to 33·5)	152·0 (142·1 to 163·7)	21·6 (17·9 to 26·0)	51·4 (45·5 to 58·1)	10·2 (6·9 to 14·5)	68·8 (63·7 to 78·6)

Liberia*	2023	65·9 (61·5 to 71·2)	9·4 (7·8 to 11·4)	22·1 (19·5 to 25·0)	4·5 (3·0 to 6·4)	29·8 (27·3 to 33·9)	154·6 (144·3 to 167·1)	22·1 (18·3 to 26·7)	51·8 (45·8 to 58·6)	10·6 (7·1 to 15·0)	70·0 (64·1 to 79·6)
Liberia*	2024	66·9 (62·2 to 72·2)	9·7 (8·0 to 11·7)	22·3 (19·7 to 25·2)	4·7 (3·1 to 6·6)	30·3 (27·4 to 34·1)	157·2 (146·0 to 169·4)	22·8 (18·7 to 27·5)	52·3 (46·2 to 59·2)	11·0 (7·3 to 15·6)	71·1 (64·3 to 80·1)
Liberia*	2025	68·0 (63·0 to 73·4)	10·0 (8·2 to 12·1)	22·5 (19·9 to 25·5)	4·8 (3·2 to 6·9)	30·7 (27·5 to 34·5)	159·7 (147·9 to 172·3)	23·4 (19·2 to 28·4)	52·8 (46·6 to 59·8)	11·3 (7·6 to 16·1)	72·1 (64·6 to 81·1)
Liberia*	2026	68·7 (63·8 to 74·4)	10·3 (8·4 to 12·5)	22·7 (20·0 to 25·7)	5·0 (3·3 to 7·1)	30·8 (27·6 to 34·9)	161·4 (149·7 to 174·7)	24·1 (19·6 to 29·2)	53·3 (47·0 to 60·4)	11·7 (7·8 to 16·6)	72·3 (64·8 to 81·9)
Liberia*	2027	69·4 (64·3 to 75·1)	10·6 (8·6 to 12·8)	22·9 (20·2 to 26·0)	5·2 (3·4 to 7·3)	30·7 (27·5 to 34·8)	162·9 (151·0 to 176·3)	24·8 (20·2 to 30·1)	53·9 (47·4 to 61·0)	12·1 (8·1 to 17·2)	72·2 (64·5 to 81·8)
Liberia*	2028	70·1 (64·7 to 76·1)	10·9 (8·9 to 13·3)	23·2 (20·4 to 26·3)	5·3 (3·6 to 7·6)	30·7 (27·4 to 34·9)	164·5 (151·8 to 178·6)	25·5 (20·8 to 31·1)	54·4 (47·9 to 61·6)	12·5 (8·4 to 17·8)	72·1 (64·3 to 81·9)
Liberia*	2029	70·8 (65·2 to 76·9)	11·2 (9·1 to 13·6)	23·4 (20·6 to 26·5)	5·5 (3·7 to 7·9)	30·7 (27·2 to 35·0)	166·2 (153·1 to 180·6)	26·2 (21·3 to 31·9)	54·9 (48·3 to 62·3)	13·0 (8·7 to 18·5)	72·0 (63·9 to 82·2)
Liberia*	2030	71·6 (65·7 to 77·9)	11·5 (9·3 to 14·0)	23·6 (20·8 to 26·8)	5·7 (3·8 to 8·1)	30·7 (27·1 to 35·3)	168·0 (154·3 to 182·8)	27·0 (21·9 to 33·0)	55·4 (48·7 to 62·8)	13·4 (8·9 to 19·1)	72·1 (63·6 to 82·9)
Liberia*	2031	72·3 (66·3 to 79·2)	11·9 (9·4 to 14·8)	23·8 (21·0 to 27·0)	5·9 (3·9 to 8·4)	30·7 (27·1 to 35·5)	169·7 (155·6 to 185·9)	27·8 (22·0 to 34·7)	56·0 (49·2 to 63·4)	13·8 (9·2 to 19·8)	72·1 (63·6 to 83·2)
Liberia*	2032	73·0 (66·8 to 80·0)	12·0 (9·4 to 15·4)	24·0 (21·1 to 27·2)	6·1 (4·1 to 8·7)	30·8 (27·1 to 35·7)	171·3 (156·8 to 187·9)	28·2 (22·1 to 36·1)	56·4 (49·6 to 63·9)	14·3 (9·5 to 20·4)	72·4 (63·5 to 83·9)
Liberia*	2033	73·8 (67·5 to 81·1)	12·2 (9·4 to 15·7)	24·2 (21·3 to 27·4)	6·3 (4·2 to 8·9)	31·1 (27·1 to 36·3)	173·2 (158·4 to 190·5)	28·7 (22·0 to 37·0)	56·9 (50·0 to 64·4)	14·7 (9·9 to 21·0)	72·9 (63·7 to 85·3)
Liberia*	2034	74·6 (67·8 to 82·4)	12·4 (9·3 to 16·3)	24·4 (21·5 to 27·7)	6·5 (4·3 to 9·2)	31·3 (27·2 to 36·9)	175·1 (159·2 to 193·3)	29·1 (21·9 to 38·2)	57·3 (50·4 to 65·0)	15·2 (10·2 to 21·7)	73·4 (63·9 to 86·7)
Liberia*	2035	75·6 (68·5 to 84·0)	12·6 (9·2 to 16·8)	24·6 (21·6 to 27·8)	6·7 (4·5 to 9·5)	31·7 (27·4 to 38·0)	177·4 (160·8 to 197·2)	29·6 (21·6 to 39·3)	57·7 (50·7 to 65·4)	15·7 (10·5 to 22·4)	74·4 (64·4 to 89·3)
Liberia*	2036	76·4 (69·1 to 85·2)	12·8 (9·3 to 17·4)	24·8 (21·8 to 28·0)	6·9 (4·6 to 9·9)	31·9 (27·7 to 38·6)	179·4 (162·2 to 199·9)	30·1 (21·8 to 40·7)	58·2 (51·1 to 65·8)	16·2 (10·8 to 23·2)	75·0 (64·9 to 90·7)

Liberia*	2037	77·4 (69·6 to 86·7)	13·0 (9·1 to 18·2)	25·0 (21·9 to 28·3)	7·1 (4·8 to 10·2)	32·3 (27·9 to 39·5)	181·6 (163·5 to 203·5)	30·5 (21·4 to 42·7)	58·6 (51·5 to 66·3)	16·7 (11·2 to 23·9)	75·8 (65·5 to 92·7)
Liberia*	2038	78·3 (70·1 to 88·2)	13·2 (9·3 to 18·4)	25·2 (22·1 to 28·5)	7·4 (4·9 to 10·5)	32·6 (28·1 to 40·1)	183·9 (164·7 to 206·9)	31·0 (21·8 to 43·1)	59·1 (51·9 to 66·9)	17·3 (11·5 to 24·7)	76·6 (65·9 to 94·1)
Liberia*	2039	79·5 (71·0 to 89·8)	13·4 (9·2 to 19·0)	25·4 (22·2 to 28·7)	7·6 (5·0 to 10·9)	33·1 (28·4 to 41·0)	186·6 (166·8 to 210·8)	31·5 (21·7 to 44·6)	59·5 (52·2 to 67·4)	17·8 (11·8 to 25·5)	77·8 (66·6 to 96·3)
Liberia*	2040	80·7 (72·1 to 91·7)	13·6 (9·3 to 19·5)	25·5 (22·4 to 28·9)	7·8 (5·2 to 11·3)	33·8 (28·5 to 42·8)	189·5 (169·4 to 215·2)	32·0 (21·9 to 45·7)	59·9 (52·5 to 67·9)	18·3 (12·2 to 26·4)	79·3 (66·9 to 100·5)
Liberia*	2041	81·9 (72·8 to 93·3)	13·9 (9·3 to 20·1)	25·7 (22·5 to 29·1)	8·1 (5·3 to 11·7)	34·2 (29·0 to 44·1)	192·2 (170·9 to 219·1)	32·5 (21·8 to 47·2)	60·3 (52·9 to 68·4)	18·9 (12·6 to 27·4)	80·4 (68·0 to 103·4)
Liberia*	2042	83·2 (73·5 to 95·2)	14·0 (9·3 to 20·5)	25·9 (22·6 to 29·3)	8·3 (5·5 to 12·0)	35·0 (29·3 to 45·8)	195·3 (172·5 to 223·5)	33·0 (21·8 to 48·1)	60·7 (53·1 to 68·8)	19·5 (12·9 to 28·2)	82·2 (68·9 to 107·6)
Liberia*	2043	84·5 (74·4 to 97·7)	14·2 (9·4 to 20·7)	26·0 (22·8 to 29·5)	8·6 (5·7 to 12·4)	35·6 (29·7 to 47·2)	198·3 (174·6 to 229·4)	33·4 (22·1 to 48·6)	61·1 (53·5 to 69·3)	20·1 (13·3 to 29·1)	83·6 (69·8 to 110·7)
Liberia*	2044	85·8 (75·6 to 100·2)	14·5 (9·4 to 21·3)	26·2 (22·9 to 29·8)	8·8 (5·8 to 12·8)	36·2 (30·1 to 48·3)	201·4 (177·5 to 235·3)	34·0 (22·2 to 50·1)	61·6 (53·8 to 69·9)	20·8 (13·7 to 30·0)	85·1 (70·7 to 113·3)
Liberia*	2045	87·1 (76·5 to 102·1)	14·7 (9·5 to 21·6)	26·4 (23·1 to 30·0)	9·1 (6·0 to 13·2)	36·9 (30·5 to 49·8)	204·5 (179·6 to 239·7)	34·5 (22·4 to 50·6)	62·0 (54·2 to 70·4)	21·4 (14·1 to 31·0)	86·7 (71·5 to 116·8)
Liberia*	2046	88·5 (77·6 to 104·4)	14·9 (9·8 to 22·1)	26·6 (23·3 to 30·2)	9·4 (6·2 to 13·7)	37·6 (30·8 to 51·3)	207·7 (182·2 to 245·0)	35·0 (23·0 to 51·9)	62·4 (54·6 to 71·0)	22·1 (14·5 to 32·1)	88·2 (72·2 to 120·3)
Liberia*	2047	89·9 (78·2 to 106·5)	15·2 (9·6 to 22·8)	26·8 (23·4 to 30·5)	9·7 (6·4 to 14·1)	38·2 (31·1 to 52·4)	211·0 (183·7 to 250·0)	35·6 (22·6 to 53·6)	62·9 (55·0 to 71·5)	22·8 (14·9 to 33·2)	89·8 (73·0 to 122·9)
Liberia*	2048	91·3 (79·4 to 108·6)	15·4 (9·7 to 23·8)	27·0 (23·6 to 30·7)	10·0 (6·5 to 14·7)	39·0 (31·8 to 54·5)	214·3 (186·5 to 255·0)	36·1 (22·8 to 55·8)	63·3 (55·3 to 72·1)	23·5 (15·4 to 34·4)	91·5 (74·6 to 128·0)
Liberia*	2049	92·8 (80·1 to 111·0)	15·6 (9·7 to 24·1)	27·1 (23·7 to 30·9)	10·3 (6·7 to 15·2)	39·8 (31·9 to 56·6)	217·8 (188·0 to 260·6)	36·6 (22·8 to 56·6)	63·7 (55·6 to 72·6)	24·2 (15·8 to 35·7)	93·4 (74·9 to 132·8)
Liberia*	2050	94·5 (81·3 to 113·6)	15·7 (9·8 to 24·4)	27·2 (23·8 to 31·1)	10·6 (6·9 to 15·7)	40·9 (32·6 to 57·7)	221·7 (190·9 to 266·7)	36·9 (22·9 to 57·3)	63·9 (55·8 to 72·9)	24·9 (16·2 to 36·8)	96·0 (76·5 to 135·3)

Libya*	1995	920·7 (849·8 to 994·0)	417·8 (357·9 to 483·7)	496·2 (464·5 to 528·1)	6·8 (4·6 to 9·5)	0·0 (0·0 to 0·0)	1331·5 (1228·9 to 1437·5)	604·1 (517·6 to 699·5)	717·6 (671·7 to 763·7)	9·8 (6·7 to 13·7)	0·0 (0·0 to 0·0)
Libya*	1996	976·6 (912·1 to 1051·5)	460·2 (399·0 to 529·8)	509·5 (485·3 to 536·9)	6·9 (4·7 to 9·6)	0·0 (0·0 to 0·0)	1412·3 (1319·0 to 1520·7)	665·6 (577·1 to 766·1)	736·8 (701·9 to 776·4)	9·9 (6·7 to 13·9)	0·0 (0·0 to 0·0)
Libya*	1997	1048·0 (983·2 to 1122·7)	502·9 (442·0 to 575·4)	537·7 (511·9 to 563·8)	7·4 (4·9 to 10·2)	0·0 (0·0 to 0·0)	1515·5 (1421·9 to 1623·7)	727·3 (639·2 to 832·1)	777·6 (740·3 to 815·3)	10·6 (7·1 to 14·7)	0·0 (0·0 to 0·0)
Libya*	1998	1082·3 (1017·0 to 1149·8)	507·3 (445·6 to 572·1)	567·1 (543·3 to 594·1)	7·9 (5·3 to 10·8)	0·0 (0·0 to 0·0)	1565·1 (1470·7 to 1662·8)	733·6 (644·3 to 827·4)	820·1 (785·7 to 859·2)	11·4 (7·7 to 15·6)	0·0 (0·0 to 0·0)
Libya*	1999	1023·2 (966·2 to 1086·3)	470·6 (417·2 to 529·5)	544·6 (522·2 to 568·2)	7·9 (5·3 to 11·0)	0·0 (0·0 to 0·0)	1479·7 (1397·2 to 1570·9)	680·6 (603·4 to 765·7)	787·6 (755·2 to 821·7)	11·5 (7·7 to 15·9)	0·0 (0·0 to 0·0)
Libya*	2000	1021·4 (964·5 to 1085·7)	503·8 (449·9 to 564·8)	509·1 (487·5 to 529·0)	8·6 (5·7 to 12·2)	0·0 (0·0 to 0·0)	1477·1 (1394·8 to 1570·0)	728·5 (650·7 to 816·9)	736·2 (705·0 to 765·1)	12·4 (8·3 to 17·7)	0·0 (0·0 to 0·0)
Libya*	2001	1146·7 (1078·4 to 1226·2)	643·7 (577·6 to 719·5)	493·2 (471·8 to 513·9)	9·9 (6·7 to 13·9)	0·0 (0·0 to 0·0)	1658·3 (1559·5 to 1773·3)	930·8 (835·3 to 1040·5)	713·2 (682·3 to 743·2)	14·3 (9·7 to 20·1)	0·0 (0·0 to 0·0)
Libya*	2002	1168·3 (1093·5 to 1247·4)	717·8 (641·8 to 796·7)	440·0 (419·0 to 459·2)	10·4 (7·0 to 14·7)	0·0 (0·0 to 0·0)	1689·5 (1581·4 to 1803·9)	1038·1 (928·2 to 1152·1)	636·3 (606·0 to 664·1)	15·1 (10·2 to 21·3)	0·0 (0·0 to 0·0)
Libya*	2003	1168·1 (1088·5 to 1250·2)	756·5 (677·8 to 835·1)	400·7 (382·3 to 420·1)	10·7 (7·3 to 15·3)	0·0 (0·0 to 0·0)	1689·2 (1574·1 to 1808·0)	1094·1 (980·2 to 1207·7)	579·5 (552·9 to 607·5)	15·5 (10·6 to 22·1)	0·0 (0·0 to 0·0)
Libya*	2004	1077·7 (1002·1 to 1155·3)	711·2 (639·1 to 788·9)	356·3 (339·3 to 374·7)	10·3 (7·1 to 14·5)	0·0 (0·0 to 0·0)	1558·6 (1449·3 to 1670·7)	1028·4 (924·2 to 1140·8)	515·2 (490·7 to 541·9)	14·9 (10·2 to 21·0)	0·0 (0·0 to 0·0)
Libya*	2005	870·7 (811·0 to 932·8)	559·6 (499·9 to 621·5)	302·1 (286·9 to 319·8)	8·9 (6·2 to 12·5)	0·1 (0·1 to 0·1)	1259·2 (1172·9 to 1349·0)	809·2 (722·9 to 898·7)	436·9 (414·9 to 462·5)	12·9 (8·9 to 18·1)	0·1 (0·1 to 0·1)
Libya*	2006	865·9 (804·5 to 929·0)	565·4 (504·0 to 626·2)	291·2 (274·9 to 308·2)	9·2 (6·3 to 12·9)	0·1 (0·1 to 0·1)	1252·3 (1163·4 to 1343·4)	817·7 (728·9 to 905·6)	421·1 (397·6 to 445·7)	13·3 (9·2 to 18·7)	0·2 (0·2 to 0·2)
Libya*	2007	869·3 (806·4 to 931·4)	575·3 (514·2 to 636·1)	284·1 (267·7 to 301·8)	9·6 (6·7 to 13·5)	0·3 (0·3 to 0·3)	1257·1 (1166·2 to 1346·9)	832·0 (743·7 to 919·9)	410·8 (387·2 to 436·4)	13·8 (9·7 to 19·5)	0·5 (0·5 to 0·5)
Libya*	2008	959·8 (890·5 to 1028·7)	651·8 (587·6 to 717·5)	291·0 (273·9 to 308·8)	10·8 (7·5 to 15·1)	6·2 (6·2 to 6·2)	1388·0 (1287·7 to 1487·6)	942·6 (849·7 to 1037·6)	420·8 (396·1 to 446·6)	15·6 (10·9 to 21·8)	9·0 (9·0 to 9·0)

Libya*	2009	1200·5 (1113·7 to 1292·4)	847·1 (760·6 to 937·5)	336·7 (318·4 to 356·7)	13·0 (9·2 to 18·4)	3·7 (3·7 to 3·7)	1736·1 (1610·5 to 1869·0)	1225·1 (1100·0 to 1355·8)	486·9 (460·5 to 515·9)	18·8 (13·3 to 26·6)	5·4 (5·4 to 5·4)
Libya*	2010	1125·9 (1043·8 to 1225·7)	785·3 (705·4 to 879·1)	327·7 (308·7 to 347·9)	12·7 (8·9 to 17·8)	0·2 (0·2 to 0·2)	1628·2 (1509·5 to 1772·6)	1135·7 (1020·1 to 1271·3)	473·9 (446·4 to 503·1)	18·4 (12·9 to 25·8)	0·3 (0·3 to 0·3)
Libya*	2011	699·2 (651·4 to 755·0)	440·3 (397·5 to 493·0)	248·1 (232·5 to 265·0)	8·7 (6·0 to 12·2)	2·1 (2·1 to 2·1)	1011·1 (942·1 to 1091·9)	636·7 (574·9 to 712·9)	358·8 (336·2 to 383·3)	12·6 (8·7 to 17·6)	3·0 (3·0 to 3·0)
Libya*	2012	1001·2 (925·5 to 1083·7)	680·2 (608·6 to 758·6)	309·1 (287·5 to 331·9)	11·5 (8·0 to 16·1)	0·3 (0·3 to 0·3)	1447·8 (1338·3 to 1567·2)	983·7 (880·2 to 1097·1)	447·0 (415·8 to 480·0)	16·6 (11·6 to 23·3)	0·5 (0·5 to 0·5)
Libya*	2013	964·4 (880·7 to 1051·5)	680·7 (602·4 to 764·0)	271·2 (246·6 to 296·2)	11·7 (8·1 to 16·6)	0·7 (0·7 to 0·7)	1394·6 (1273·6 to 1520·6)	984·4 (871·2 to 1104·8)	392·2 (356·7 to 428·3)	17·0 (11·7 to 24·1)	1·0 (1·0 to 1·0)
Libya*	2014	841·5 (761·6 to 927·5)	615·1 (536·8 to 699·9)	215·2 (191·2 to 241·6)	11·0 (7·6 to 15·4)	0·1 (0·1 to 0·1)	1216·9 (1101·3 to 1341·3)	889·6 (776·4 to 1012·2)	311·3 (276·5 to 349·4)	15·9 (11·0 to 22·3)	0·1 (0·1 to 0·1)
Libya*	2015	744·9 (659·7 to 827·6)	543·8 (466·0 to 626·5)	190·7 (164·1 to 220·9)	10·1 (7·0 to 14·1)	0·3 (0·3 to 0·3)	1077·3 (954·1 to 1196·8)	786·5 (673·9 to 906·0)	275·7 (237·3 to 319·4)	14·6 (10·2 to 20·4)	0·5 (0·5 to 0·5)
Libya*	2016	687·8 (603·3 to 780·3)	503·2 (423·2 to 588·9)	174·1 (145·6 to 206·8)	9·8 (6·8 to 13·8)	0·6 (0·6 to 0·6)	994·6 (872·4 to 1128·5)	727·8 (612·0 to 851·7)	251·8 (210·6 to 299·0)	14·2 (9·9 to 20·0)	0·9 (0·9 to 0·9)
Libya*	2017	653·6 (572·7 to 744·7)	476·8 (399·3 to 558·5)	166·0 (136·2 to 202·1)	9·7 (6·8 to 13·5)	1·1 (1·1 to 1·1)	945·2 (828·3 to 1077·0)	689·5 (577·4 to 807·7)	240·0 (196·9 to 292·3)	14·0 (9·8 to 19·5)	1·6 (1·6 to 1·6)
Libya*	2018	660·5 (574·4 to 756·3)	483·9 (403·8 to 574·9)	166·1 (132·7 to 205·4)	10·2 (7·0 to 14·3)	0·4 (0·4 to 0·4)	955·2 (830·7 to 1093·7)	699·8 (583·9 to 831·4)	240·2 (191·9 to 297·0)	14·7 (10·1 to 20·6)	0·6 (0·6 to 0·6)
Libya*	2019	798·5 (683·1 to 929·6)	613·3 (502·9 to 744·7)	173·8 (138·5 to 215·0)	11·1 (7·6 to 15·5)	0·2 (0·0 to 0·3)	1154·7 (987·9 to 1344·3)	887·0 (727·3 to 1076·9)	251·4 (200·4 to 310·9)	16·0 (11·0 to 22·5)	0·3 (0·0 to 0·5)
Libya*	2020	780·2 (652·6 to 918·0)	610·3 (486·7 to 748·3)	159·2 (126·8 to 197·1)	10·4 (7·2 to 14·6)	0·3 (0·0 to 0·5)	1128·2 (943·8 to 1327·6)	882·5 (703·8 to 1082·2)	230·2 (183·4 to 285·1)	15·1 (10·4 to 21·2)	0·5 (0·0 to 0·7)
Libya*	2021	740·6 (623·3 to 873·5)	579·5 (462·2 to 710·7)	150·7 (120·2 to 186·5)	10·1 (7·0 to 14·2)	0·2 (0·0 to 0·6)	1071·0 (901·4 to 1263·3)	838·1 (668·4 to 1027·7)	218·0 (173·8 to 269·7)	14·6 (10·1 to 20·6)	0·4 (0·0 to 0·8)
Libya*	2022	642·6 (531·7 to 771·9)	481·5 (372·4 to 612·1)	150·6 (120·0 to 186·3)	10·3 (7·1 to 14·6)	0·2 (0·0 to 0·4)	929·3 (769·0 to 1116·3)	696·3 (538·6 to 885·2)	217·9 (173·5 to 269·4)	14·9 (10·3 to 21·1)	0·3 (0·0 to 0·6)

Libya*	2023	647·2 (523·9 to 788·3)	485·8 (368·2 to 621·3)	150·6 (119·9 to 186·4)	10·5 (7·3 to 14·9)	0·2 (0·0 to 0·5)	935·9 (757·7 to 1140·0)	702·6 (532·5 to 898·6)	217·8 (173·4 to 269·6)	15·3 (10·5 to 21·6)	0·3 (0·0 to 0·7)
Libya*	2024	651·4 (524·5 to 793·1)	490·2 (365·1 to 628·5)	150·3 (119·7 to 185·9)	10·8 (7·4 to 15·2)	0·2 (0·0 to 0·5)	942·1 (758·5 to 1146·9)	708·8 (528·0 to 909·0)	217·3 (173·1 to 268·8)	15·6 (10·7 to 22·0)	0·3 (0·0 to 0·7)
Libya*	2025	655·2 (526·5 to 805·1)	493·9 (364·3 to 650·6)	150·1 (119·5 to 185·5)	11·1 (7·6 to 15·7)	0·2 (0·0 to 0·5)	947·6 (761·4 to 1164·3)	714·2 (526·9 to 940·8)	217·0 (172·8 to 268·3)	16·0 (11·0 to 22·6)	0·3 (0·0 to 0·7)
Libya*	2026	659·7 (523·0 to 818·3)	498·3 (366·3 to 657·8)	149·7 (119·2 to 185·0)	11·4 (7·9 to 16·1)	0·2 (0·0 to 0·5)	954·0 (756·3 to 1183·3)	720·6 (529·8 to 951·3)	216·5 (172·4 to 267·6)	16·5 (11·4 to 23·3)	0·3 (0·0 to 0·8)
Libya*	2027	661·8 (522·1 to 834·3)	500·9 (369·9 to 673·2)	149·0 (118·7 to 184·3)	11·7 (8·1 to 16·6)	0·2 (0·0 to 0·6)	957·0 (755·0 to 1206·6)	724·3 (535·0 to 973·6)	215·5 (171·7 to 266·5)	16·9 (11·7 to 24·0)	0·3 (0·0 to 0·8)
Libya*	2028	666·3 (522·7 to 842·0)	505·4 (366·9 to 679·8)	148·6 (118·4 to 183·6)	12·0 (8·3 to 17·1)	0·2 (0·0 to 0·6)	963·6 (755·9 to 1217·7)	730·9 (530·6 to 983·0)	215·0 (171·2 to 265·5)	17·4 (12·1 to 24·7)	0·3 (0·0 to 0·9)
Libya*	2029	669·2 (524·1 to 851·3)	508·4 (366·5 to 688·7)	148·3 (118·2 to 182·8)	12·3 (8·5 to 17·7)	0·3 (0·0 to 0·7)	967·8 (757·9 to 1231·1)	735·2 (530·0 to 995·9)	214·4 (170·9 to 264·3)	17·9 (12·3 to 25·6)	0·4 (0·0 to 1·0)
Libya*	2030	672·6 (521·7 to 853·5)	511·8 (365·7 to 690·9)	147·9 (118·0 to 181·9)	12·7 (8·7 to 18·4)	0·3 (0·0 to 0·7)	972·7 (754·4 to 1234·2)	740·1 (528·8 to 999·1)	213·9 (170·6 to 263·1)	18·4 (12·6 to 26·5)	0·4 (0·0 to 1·0)
Libya*	2031	675·5 (522·5 to 870·7)	514·7 (360·0 to 703·6)	147·4 (117·7 to 181·5)	13·1 (8·9 to 18·9)	0·3 (0·0 to 0·7)	976·8 (755·7 to 1259·2)	744·4 (520·6 to 1017·5)	213·2 (170·2 to 262·5)	18·9 (12·9 to 27·3)	0·4 (0·0 to 1·1)
Libya*	2032	678·9 (521·7 to 890·7)	518·3 (357·9 to 719·3)	146·9 (117·3 to 181·1)	13·4 (9·1 to 19·6)	0·3 (0·0 to 0·8)	981·8 (754·4 to 1288·1)	749·5 (517·6 to 1040·2)	212·4 (169·7 to 261·8)	19·4 (13·2 to 28·3)	0·4 (0·0 to 1·2)
Libya*	2033	679·8 (516·5 to 887·6)	519·6 (358·8 to 724·8)	146·1 (116·8 to 180·3)	13·8 (9·3 to 20·2)	0·3 (0·0 to 0·9)	983·1 (747·0 to 1283·7)	751·4 (518·9 to 1048·2)	211·3 (168·9 to 260·7)	19·9 (13·5 to 29·2)	0·5 (0·0 to 1·3)
Libya*	2034	680·7 (518·2 to 897·6)	520·6 (353·2 to 738·6)	145·6 (116·4 to 180·0)	14·2 (9·6 to 20·6)	0·3 (0·0 to 0·9)	984·4 (749·4 to 1298·0)	752·8 (510·8 to 1068·2)	210·6 (168·3 to 260·3)	20·5 (13·8 to 29·8)	0·5 (0·0 to 1·4)
Libya*	2035	682·1 (514·5 to 905·3)	522·4 (353·7 to 756·9)	144·8 (115·8 to 179·0)	14·6 (9·8 to 21·2)	0·4 (0·0 to 1·0)	986·5 (744·0 to 1309·2)	755·5 (511·5 to 1094·7)	209·4 (167·5 to 258·8)	21·1 (14·1 to 30·7)	0·5 (0·0 to 1·5)
Libya*	2036	680·6 (506·6 to 909·7)	521·6 (352·2 to 756·9)	143·6 (114·9 to 177·7)	15·0 (9·9 to 21·9)	0·4 (0·0 to 1·1)	984·2 (732·6 to 1315·6)	754·4 (509·3 to 1094·6)	207·6 (166·1 to 256·9)	21·6 (14·3 to 31·7)	0·6 (0·0 to 1·6)

Libya*	2037	680·7 (504·7 to 916·4)	522·6 (343·9 to 761·2)	142·4 (114·0 to 176·3)	15·3 (10·1 to 22·5)	0·4 (0·0 to 1·2)	984·4 (729·9 to 1325·3)	755·7 (497·3 to 1100·8)	205·9 (164·8 to 254·9)	22·2 (14·6 to 32·6)	0·6 (0·0 to 1·8)
Libya*	2038	677·9 (501·7 to 908·2)	520·8 (346·9 to 750·0)	141·0 (112·9 to 174·2)	15·7 (10·3 to 23·1)	0·5 (0·0 to 1·3)	980·4 (725·5 to 1313·4)	753·2 (501·6 to 1084·6)	203·9 (163·2 to 251·9)	22·7 (14·9 to 33·5)	0·7 (0·0 to 1·9)
Libya*	2039	677·0 (493·0 to 904·4)	520·5 (340·2 to 758·9)	139·9 (112·0 to 173·0)	16·1 (10·5 to 23·8)	0·5 (0·0 to 1·5)	979·1 (713·0 to 1307·9)	752·8 (491·9 to 1097·4)	202·3 (162·0 to 250·2)	23·3 (15·2 to 34·4)	0·7 (0·0 to 2·1)
Libya*	2040	677·2 (488·8 to 902·3)	521·3 (334·5 to 753·9)	138·9 (111·3 to 171·9)	16·5 (10·7 to 24·4)	0·5 (0·0 to 1·6)	979·3 (706·8 to 1304·9)	753·8 (483·7 to 1090·3)	200·8 (160·9 to 248·6)	23·8 (15·5 to 35·3)	0·8 (0·0 to 2·3)
Libya*	2041	675·2 (485·2 to 906·4)	520·0 (330·4 to 761·2)	137·6 (110·0 to 170·5)	16·9 (10·8 to 25·1)	0·6 (0·0 to 1·8)	976·4 (701·6 to 1310·8)	752·0 (477·8 to 1100·9)	199·1 (159·1 to 246·6)	24·4 (15·7 to 36·4)	0·9 (0·0 to 2·6)
Libya*	2042	673·1 (482·7 to 915·9)	518·6 (322·8 to 761·4)	136·5 (108·9 to 169·0)	17·3 (11·0 to 25·9)	0·7 (0·0 to 2·0)	973·4 (698·1 to 1324·6)	750·0 (466·9 to 1101·0)	197·5 (157·5 to 244·3)	25·0 (15·9 to 37·4)	0·9 (0·0 to 2·8)
Libya*	2043	671·2 (477·6 to 923·2)	517·4 (320·0 to 774·0)	135·4 (107·8 to 167·5)	17·7 (11·1 to 26·7)	0·7 (0·0 to 2·2)	970·6 (690·7 to 1335·2)	748·2 (462·7 to 1119·3)	195·8 (155·9 to 242·2)	25·5 (16·1 to 38·7)	1·0 (0·0 to 3·1)
Libya*	2044	668·8 (472·0 to 924·5)	515·9 (317·9 to 773·3)	134·1 (106·5 to 166·2)	18·0 (11·2 to 27·5)	0·8 (0·0 to 2·4)	967·1 (682·6 to 1336·9)	746·0 (459·7 to 1118·3)	193·9 (154·1 to 240·3)	26·1 (16·2 to 39·7)	1·1 (0·0 to 3·4)
Libya*	2045	666·1 (464·6 to 921·6)	513·9 (315·9 to 767·9)	132·9 (105·7 to 164·7)	18·4 (11·3 to 28·3)	0·9 (0·0 to 2·6)	963·3 (671·8 to 1332·7)	743·2 (456·8 to 1110·4)	192·2 (152·8 to 238·2)	26·6 (16·4 to 41·0)	1·2 (0·0 to 3·8)
Libya*	2046	662·9 (462·5 to 919·6)	511·5 (312·4 to 767·6)	131·7 (104·6 to 162·9)	18·8 (11·5 to 29·2)	0·9 (0·0 to 2·8)	958·7 (668·9 to 1329·8)	739·7 (451·8 to 1110·0)	190·4 (151·2 to 235·6)	27·2 (16·7 to 42·3)	1·4 (0·0 to 4·1)
Libya*	2047	661·0 (458·7 to 902·1)	510·2 (307·0 to 755·8)	130·6 (103·7 to 161·5)	19·2 (11·7 to 30·1)	1·0 (0·0 to 3·2)	956·0 (663·3 to 1304·6)	737·8 (444·0 to 1093·0)	188·9 (150·0 to 233·6)	27·8 (16·9 to 43·6)	1·5 (0·0 to 4·6)
Libya*	2048	658·0 (458·1 to 911·8)	508·1 (308·3 to 761·8)	129·3 (102·6 to 159·9)	19·6 (11·7 to 31·0)	1·1 (0·0 to 3·5)	951·6 (662·4 to 1318·6)	734·7 (445·9 to 1101·7)	186·9 (148·4 to 231·2)	28·3 (17·0 to 44·8)	1·6 (0·0 to 5·1)
Libya*	2049	655·8 (450·2 to 910·3)	506·7 (306·0 to 756·2)	127·9 (101·4 to 158·3)	20·0 (11·9 to 31·9)	1·2 (0·0 to 3·9)	948·4 (651·1 to 1316·5)	732·8 (442·5 to 1093·6)	185·0 (146·6 to 229·0)	28·9 (17·2 to 46·1)	1·8 (0·0 to 5·7)
Libya*	2050	653·0 (448·4 to 924·1)	504·5 (299·9 to 778·5)	126·8 (100·4 to 157·1)	20·4 (12·1 to 32·8)	1·4 (0·0 to 4·4)	944·4 (648·4 to 1336·5)	729·5 (433·7 to 1125·8)	183·4 (145·2 to 227·2)	29·5 (17·5 to 47·4)	2·0 (0·0 to 6·3)

Saint Lucia*	1995	500·1 (466·2 to 534·7)	177·4 (161·5 to 195·6)	282·4 (254·3 to 311·4)	40·3 (30·0 to 52·6)	0·1 (0·1 to 0·1)	701·1 (653·5 to 749·6)	248·7 (226·4 to 274·2)	395·8 (356·6 to 436·5)	56·4 (42·0 to 73·7)	0·1 (0·1 to 0·1)
Saint Lucia*	1996	536·4 (507·6 to 567·1)	180·0 (165·8 to 194·9)	284·2 (261·4 to 309·7)	40·5 (31·0 to 52·0)	31·8 (31·8 to 31·8)	752·0 (711·6 to 795·0)	252·3 (232·4 to 273·2)	398·5 (366·4 to 434·2)	56·7 (43·5 to 72·8)	44·5 (44·5 to 44·5)
Saint Lucia*	1997	501·4 (475·5 to 530·7)	175·8 (161·9 to 189·2)	276·5 (254·2 to 297·8)	40·0 (31·4 to 50·0)	9·2 (9·2 to 9·2)	702·9 (666·6 to 743·9)	246·4 (227·0 to 265·3)	387·6 (356·3 to 417·5)	56·1 (44·0 to 70·1)	12·8 (12·8 to 12·8)
Saint Lucia*	1998	489·8 (464·0 to 518·0)	175·4 (161·8 to 189·0)	268·5 (247·5 to 290·0)	39·7 (31·1 to 49·5)	6·2 (6·2 to 6·2)	686·6 (650·5 to 726·1)	245·9 (226·8 to 265·0)	376·4 (347·0 to 406·5)	55·7 (43·7 to 69·4)	8·7 (8·7 to 8·7)
Saint Lucia*	1999	494·2 (469·3 to 523·0)	179·5 (165·6 to 192·8)	273·3 (251·9 to 295·8)	39·6 (31·1 to 50·2)	1·7 (1·7 to 1·7)	692·7 (657·8 to 733·2)	251·7 (232·2 to 270·3)	383·1 (353·2 to 414·7)	55·5 (43·6 to 70·4)	2·4 (2·4 to 2·4)
Saint Lucia*	2000	480·5 (454·5 to 508·3)	169·5 (156·6 to 182·4)	273·7 (252·6 to 295·5)	36·9 (29·2 to 46·0)	0·4 (0·4 to 0·4)	673·5 (637·1 to 712·5)	237·6 (219·6 to 255·7)	383·6 (354·1 to 414·3)	51·8 (40·9 to 64·5)	0·6 (0·6 to 0·6)
Saint Lucia*	2001	479·3 (453·9 to 509·9)	164·0 (151·9 to 177·5)	274·7 (253·5 to 296·6)	39·4 (31·5 to 49·1)	1·1 (1·1 to 1·1)	671·9 (636·3 to 714·9)	229·9 (213·0 to 248·8)	385·2 (355·3 to 415·8)	55·2 (44·1 to 68·9)	1·6 (1·6 to 1·6)
Saint Lucia*	2002	489·2 (463·7 to 517·9)	163·7 (151·4 to 177·0)	281·1 (258·7 to 303·5)	42·8 (34·5 to 53·1)	1·5 (1·5 to 1·5)	685·8 (650·0 to 726·0)	229·5 (212·2 to 248·2)	394·1 (362·7 to 425·4)	60·0 (48·4 to 74·4)	2·1 (2·1 to 2·1)
Saint Lucia*	2003	491·8 (465·7 to 520·8)	159·9 (147·2 to 172·8)	285·5 (265·0 to 308·8)	42·9 (34·4 to 52·3)	3·6 (3·6 to 3·6)	689·5 (652·9 to 730·1)	224·1 (206·3 to 242·3)	400·2 (371·5 to 432·8)	60·2 (48·3 to 73·3)	5·0 (5·0 to 5·0)
Saint Lucia*	2004	498·5 (472·2 to 526·6)	161·0 (148·7 to 174·3)	290·1 (269·4 to 313·5)	43·0 (34·4 to 52·8)	4·3 (4·3 to 4·3)	698·8 (661·9 to 738·2)	225·7 (208·5 to 244·3)	406·7 (377·7 to 439·5)	60·3 (48·3 to 74·1)	6·0 (6·0 to 6·0)
Saint Lucia*	2005	524·2 (497·7 to 552·4)	170·2 (157·0 to 184·1)	305·7 (284·8 to 327·2)	44·0 (35·2 to 54·1)	4·4 (4·4 to 4·4)	734·9 (697·7 to 774·3)	238·5 (220·1 to 258·1)	428·5 (399·2 to 458·7)	61·7 (49·4 to 75·9)	6·1 (6·1 to 6·1)
Saint Lucia*	2006	549·3 (521·1 to 578·3)	170·6 (158·3 to 184·1)	327·8 (304·0 to 352·0)	43·4 (34·5 to 54·1)	7·5 (7·5 to 7·5)	770·1 (730·5 to 810·7)	239·2 (221·9 to 258·1)	459·6 (426·2 to 493·4)	60·8 (48·3 to 75·9)	10·5 (10·5 to 10·5)
Saint Lucia*	2007	550·3 (522·7 to 578·6)	164·5 (152·7 to 177·2)	340·4 (317·5 to 363·9)	40·6 (31·9 to 50·7)	4·8 (4·8 to 4·8)	771·4 (732·8 to 811·2)	230·6 (214·1 to 248·4)	477·2 (445·0 to 510·2)	56·9 (44·8 to 71·1)	6·7 (6·7 to 6·7)
Saint Lucia*	2008	570·3 (543·9 to 599·4)	177·9 (165·2 to 190·8)	343·1 (321·4 to 367·0)	39·0 (30·7 to 49·1)	10·3 (10·3 to 10·3)	799·5 (762·5 to 840·3)	249·4 (231·6 to 267·5)	481·0 (450·5 to 514·5)	54·6 (43·0 to 68·9)	14·4 (14·4 to 14·4)

Saint Lucia*	2009	577·4 (552·3 to 605·6)	192·3 (179·7 to 205·2)	335·7 (314·5 to 358·1)	38·0 (29·9 to 48·0)	11·5 (11·5 to 11·5)	809·5 (774·3 to 848·9)	269·5 (251·9 to 287·7)	470·5 (440·9 to 502·0)	53·3 (41·9 to 67·3)	16·1 (16·1 to 16·1)
Saint Lucia*	2010	570·8 (543·5 to 599·7)	199·9 (186·8 to 213·5)	320·2 (299·4 to 342·9)	39·0 (31·1 to 49·0)	11·7 (11·7 to 11·7)	800·2 (761·9 to 840·7)	280·3 (261·9 to 299·4)	448·9 (419·7 to 480·8)	54·7 (43·6 to 68·7)	16·4 (16·4 to 16·4)
Saint Lucia*	2011	671·2 (642·1 to 700·6)	215·7 (201·3 to 229·7)	315·1 (293·3 to 338·5)	41·4 (32·4 to 52·1)	98·9 (98·9 to 98·9)	940·9 (900·2 to 982·1)	302·4 (282·2 to 322·0)	441·7 (411·2 to 474·6)	58·1 (45·4 to 73·0)	138·7 (138·7 to 138·7)
Saint Lucia*	2012	646·6 (617·9 to 675·3)	228·5 (213·9 to 242·7)	299·0 (276·0 to 321·1)	41·7 (32·5 to 52·6)	77·4 (77·4 to 77·4)	906·5 (866·3 to 946·6)	320·3 (299·9 to 340·3)	419·1 (386·9 to 450·1)	58·5 (45·5 to 73·8)	108·5 (108·5 to 108·5)
Saint Lucia*	2013	566·6 (540·0 to 593·9)	220·0 (206·4 to 233·7)	273·8 (253·1 to 294·3)	39·3 (30·7 to 49·3)	33·6 (33·6 to 33·6)	794·4 (757·1 to 832·6)	308·4 (289·3 to 327·6)	383·8 (354·8 to 412·6)	55·1 (43·1 to 69·2)	47·1 (47·1 to 47·1)
Saint Lucia*	2014	521·8 (495·8 to 548·2)	216·4 (202·3 to 229·8)	246·0 (226·7 to 265·9)	37·2 (28·7 to 47·2)	22·3 (22·3 to 22·3)	731·6 (695·1 to 768·6)	303·4 (283·5 to 322·1)	344·9 (317·8 to 372·7)	52·1 (40·2 to 66·1)	31·2 (31·2 to 31·2)
Saint Lucia*	2015	504·4 (478·7 to 529·1)	218·5 (204·8 to 232·0)	231·7 (211·6 to 251·5)	33·1 (25·6 to 42·3)	21·1 (21·1 to 21·1)	707·1 (671·0 to 741·7)	306·3 (287·1 to 325·3)	324·8 (296·7 to 352·6)	46·4 (35·9 to 59·3)	29·6 (29·6 to 29·6)
Saint Lucia*	2016	522·6 (495·3 to 548·7)	223·0 (209·1 to 237·0)	230·6 (210·1 to 250·7)	30·8 (23·7 to 39·9)	38·2 (38·2 to 38·2)	732·6 (694·4 to 769·2)	312·6 (293·1 to 332·2)	323·2 (294·6 to 351·4)	43·2 (33·2 to 55·9)	53·6 (53·6 to 53·6)
Saint Lucia*	2017	517·3 (487·3 to 546·1)	233·0 (216·5 to 248·9)	229·9 (206·9 to 252·5)	30·1 (22·9 to 39·3)	24·3 (24·3 to 24·3)	725·2 (683·1 to 765·6)	326·6 (303·6 to 348·9)	322·3 (290·0 to 353·9)	42·2 (32·1 to 55·1)	34·1 (34·1 to 34·1)
Saint Lucia*	2018	506·2 (472·7 to 542·1)	237·3 (218·0 to 256·2)	230·7 (204·8 to 258·7)	30·8 (22·8 to 39·9)	7·5 (7·5 to 7·5)	709·6 (662·7 to 759·9)	332·6 (305·7 to 359·1)	323·3 (287·1 to 362·7)	43·1 (32·0 to 55·9)	10·5 (10·5 to 10·5)
Saint Lucia*	2019	530·9 (495·8 to 568·2)	259·0 (238·2 to 280·1)	232·4 (206·6 to 260·8)	31·7 (23·6 to 40·9)	7·8 (7·8 to 7·9)	744·2 (695·0 to 796·5)	363·0 (333·9 to 392·7)	325·8 (289·6 to 365·6)	44·5 (33·0 to 57·4)	10·9 (10·9 to 11·0)
Saint Lucia*	2020	556·6 (520·6 to 592·7)	299·5 (275·0 to 324·5)	215·8 (191·7 to 242·3)	30·1 (22·3 to 39·1)	11·1 (11·0 to 11·3)	780·3 (729·8 to 830·9)	419·9 (385·5 to 454·9)	302·6 (268·8 to 339·7)	42·2 (31·3 to 54·9)	15·6 (15·4 to 15·9)
Saint Lucia*	2021	528·9 (494·9 to 563·0)	283·8 (260·6 to 307·5)	204·4 (181·6 to 229·5)	29·1 (21·6 to 38·0)	11·6 (11·3 to 11·9)	741·5 (693·7 to 789·3)	397·9 (365·3 to 431·1)	286·5 (254·5 to 321·8)	40·8 (30·2 to 53·3)	16·2 (15·9 to 16·7)
Saint Lucia*	2022	478·6 (445·8 to 511·9)	233·5 (212·8 to 255·5)	205·9 (182·8 to 231·1)	29·9 (22·1 to 39·2)	9·2 (8·7 to 9·7)	670·9 (625·0 to 717·7)	327·4 (298·4 to 358·2)	288·7 (256·3 to 324·0)	42·0 (31·0 to 54·9)	12·9 (12·2 to 13·6)

Saint Lucia*	2023	487·1 (453·0 to 521·3)	239·1 (216·7 to 263·1)	207·5 (184·3 to 232·9)	30·8 (22·7 to 40·2)	9·6 (9·0 to 10·3)	682·8 (635·0 to 730·9)	335·2 (303·8 to 368·8)	290·9 (258·3 to 326·5)	43·2 (31·8 to 56·4)	13·5 (12·6 to 14·4)
Saint Lucia*	2024	496·2 (459·7 to 532·2)	245·5 (220·5 to 273·0)	209·1 (185·6 to 234·6)	31·7 (23·3 to 41·6)	10·0 (9·3 to 10·7)	695·6 (644·4 to 746·1)	344·1 (309·1 to 382·7)	293·1 (260·2 to 328·9)	44·4 (32·6 to 58·3)	14·0 (13·1 to 15·0)
Saint Lucia*	2025	501·4 (463·5 to 539·6)	248·6 (221·7 to 277·7)	209·9 (186·4 to 235·7)	32·5 (23·9 to 42·9)	10·4 (9·7 to 11·2)	703·0 (649·8 to 756·5)	348·5 (310·8 to 389·3)	294·3 (261·3 to 330·5)	45·6 (33·5 to 60·2)	14·6 (13·6 to 15·7)
Saint Lucia*	2026	506·8 (466·8 to 547·4)	251·9 (223·6 to 284·3)	210·7 (187·0 to 236·7)	33·4 (24·5 to 44·0)	10·8 (10·0 to 11·7)	710·4 (654·4 to 767·3)	353·1 (313·5 to 398·6)	295·3 (262·2 to 331·9)	46·8 (34·3 to 61·7)	15·2 (14·1 to 16·4)
Saint Lucia*	2027	515·4 (474·0 to 557·8)	258·6 (227·2 to 295·1)	211·2 (187·5 to 237·3)	34·2 (25·1 to 45·0)	11·3 (10·4 to 12·3)	722·5 (664·5 to 782·0)	362·5 (318·5 to 413·6)	296·1 (262·9 to 332·7)	48·0 (35·1 to 63·1)	15·9 (14·6 to 17·2)
Saint Lucia*	2028	523·5 (480·9 to 567·0)	264·9 (233·1 to 303·7)	211·7 (187·9 to 238·0)	35·1 (25·7 to 46·4)	11·8 (10·9 to 13·0)	733·8 (674·2 to 794·8)	371·3 (326·8 to 425·8)	296·8 (263·4 to 333·6)	49·2 (36·0 to 65·1)	16·6 (15·2 to 18·2)
Saint Lucia*	2029	531·3 (486·7 to 578·9)	270·9 (234·8 to 311·7)	212·1 (188·2 to 238·5)	36·0 (26·3 to 47·6)	12·4 (11·3 to 13·6)	744·9 (682·2 to 811·5)	379·7 (329·1 to 437·0)	297·4 (263·9 to 334·4)	50·4 (36·9 to 66·8)	17·3 (15·8 to 19·1)
Saint Lucia*	2030	539·2 (492·3 to 589·1)	277·1 (239·0 to 319·8)	212·3 (188·4 to 238·7)	36·8 (27·0 to 48·8)	12·9 (11·7 to 14·4)	755·9 (690·1 to 825·9)	388·5 (335·0 to 448·4)	297·6 (264·1 to 334·6)	51·6 (37·9 to 68·3)	18·1 (16·4 to 20·2)
Saint Lucia*	2031	546·9 (495·9 to 599·0)	283·3 (242·7 to 331·8)	212·3 (188·4 to 238·6)	37·7 (27·5 to 49·9)	13·6 (12·3 to 15·4)	766·7 (695·1 to 839·8)	397·2 (340·2 to 465·2)	297·7 (264·1 to 334·5)	52·8 (38·6 to 69·9)	19·1 (17·2 to 21·5)
Saint Lucia*	2032	554·5 (502·5 to 609·3)	289·4 (246·6 to 340·5)	212·3 (188·4 to 238·5)	38·5 (28·0 to 51·0)	14·3 (12·9 to 16·3)	777·4 (704·5 to 854·2)	405·7 (345·7 to 477·3)	297·6 (264·1 to 334·3)	54·0 (39·3 to 71·6)	20·1 (18·0 to 22·8)
Saint Lucia*	2033	562·4 (508·4 to 618·9)	295·6 (250·1 to 346·9)	212·2 (188·3 to 238·5)	39·4 (28·6 to 52·4)	15·1 (13·5 to 17·4)	788·3 (712·7 to 867·6)	414·4 (350·5 to 486·3)	297·5 (264·0 to 334·4)	55·3 (40·1 to 73·4)	21·2 (18·9 to 24·3)
Saint Lucia*	2034	570·2 (513·9 to 628·4)	301·9 (255·2 to 355·7)	212·1 (188·2 to 238·5)	40·3 (29·1 to 53·8)	16·0 (14·2 to 18·7)	799·4 (720·5 to 881·0)	423·2 (357·8 to 498·7)	297·4 (263·9 to 334·3)	56·5 (40·8 to 75·4)	22·4 (19·9 to 26·1)
Saint Lucia*	2035	578·1 (520·1 to 638·5)	307·9 (259·6 to 364·4)	212·1 (188·3 to 238·6)	41·3 (29·6 to 54·8)	16·8 (14·9 to 20·0)	810·5 (729·2 to 895·1)	431·7 (363·9 to 510·8)	297·4 (263·9 to 334·5)	57·8 (41·5 to 76·9)	23·6 (20·9 to 28·1)
Saint Lucia*	2036	586·1 (526·3 to 649·0)	314·1 (263·3 to 375·3)	212·0 (188·2 to 238·6)	42·2 (30·2 to 56·2)	17·8 (15·6 to 21·4)	821·6 (737·8 to 909·8)	440·3 (369·1 to 526·1)	297·2 (263·8 to 334·5)	59·1 (42·3 to 78·8)	24·9 (21·9 to 30·1)

Saint Lucia*	2037	593·6 (531·7 to 658·3)	319·7 (266·6 to 381·9)	211·9 (188·1 to 238·5)	43·1 (30·8 to 57·4)	18·8 (16·5 to 22·9)	832·1 (745·3 to 922·9)	448·2 (373·7 to 535·3)	297·0 (263·6 to 334·4)	60·5 (43·2 to 80·5)	26·4 (23·2 to 32·1)
Saint Lucia*	2038	601·3 (536·9 to 668·4)	325·4 (270·5 to 390·8)	211·9 (188·1 to 238·7)	44·1 (31·5 to 58·7)	19·9 (17·4 to 24·6)	842·9 (752·7 to 937·0)	456·1 (379·2 to 547·8)	297·1 (263·7 to 334·6)	61·9 (44·2 to 82·3)	27·9 (24·4 to 34·5)
Saint Lucia*	2039	609·2 (543·8 to 680·0)	331·0 (274·1 to 397·7)	211·9 (188·1 to 238·8)	45·2 (32·0 to 60·2)	21·1 (18·3 to 25·9)	854·0 (762·3 to 953·3)	464·0 (384·2 to 557·5)	297·1 (263·8 to 334·7)	63·3 (44·9 to 84·5)	29·5 (25·6 to 36·4)
Saint Lucia*	2040	617·7 (550·3 to 689·3)	337·0 (277·7 to 405·0)	212·2 (188·4 to 239·0)	46·3 (32·7 to 62·1)	22·2 (19·2 to 28·1)	866·0 (771·4 to 966·3)	472·5 (389·3 to 567·8)	297·5 (264·1 to 335·0)	64·9 (45·8 to 87·1)	31·2 (26·9 to 39·4)
Saint Lucia*	2041	626·2 (553·7 to 700·6)	342·9 (280·1 to 416·4)	212·5 (188·7 to 239·3)	47·4 (33·4 to 63·9)	23·4 (20·0 to 29·9)	877·9 (776·2 to 982·1)	480·7 (392·7 to 583·8)	298·0 (264·5 to 335·5)	66·5 (46·8 to 89·6)	32·8 (28·1 to 41·9)
Saint Lucia*	2042	634·8 (561·4 to 711·6)	348·6 (281·0 to 423·8)	213·0 (189·0 to 239·9)	48·6 (34·0 to 65·8)	24·7 (21·0 to 32·2)	889·9 (787·0 to 997·6)	488·7 (393·9 to 594·1)	298·5 (265·0 to 336·2)	68·1 (47·7 to 92·2)	34·6 (29·4 to 45·2)
Saint Lucia*	2043	643·7 (567·9 to 725·4)	354·4 (284·2 to 432·9)	213·4 (189·3 to 240·4)	49·8 (34·7 to 67·7)	26·0 (21·9 to 34·3)	902·3 (796·1 to 1016·9)	496·8 (398·4 to 606·9)	299·2 (265·4 to 336·9)	69·9 (48·6 to 95·0)	36·5 (30·7 to 48·1)
Saint Lucia*	2044	651·9 (573·3 to 734·6)	359·6 (287·2 to 438·6)	213·8 (189·6 to 240·8)	51·1 (35·4 to 69·8)	27·3 (22·6 to 36·7)	913·8 (803·7 to 1029·8)	504·1 (402·6 to 614·9)	299·8 (265·7 to 337·5)	71·6 (49·6 to 97·8)	38·3 (31·7 to 51·5)
Saint Lucia*	2045	660·8 (577·5 to 750·4)	365·3 (291·5 to 447·2)	214·3 (189·9 to 241·3)	52·4 (36·2 to 71·8)	28·7 (23·4 to 39·6)	926·3 (809·6 to 1051·9)	512·1 (408·7 to 626·9)	300·4 (266·2 to 338·3)	73·5 (50·7 to 100·7)	40·3 (32·8 to 55·5)
Saint Lucia*	2046	669·5 (585·9 to 760·8)	370·9 (295·6 to 456·2)	214·9 (190·3 to 242·1)	53·8 (37·0 to 73·9)	30·0 (0·0 to 42·0)	938·6 (821·4 to 1066·6)	520·0 (414·4 to 639·5)	301·3 (266·7 to 339·4)	75·4 (51·8 to 103·5)	42·0 (0·0 to 58·9)
Saint Lucia*	2047	678·4 (591·5 to 774·0)	376·4 (298·6 to 467·3)	215·5 (190·7 to 242·8)	55·2 (37·9 to 76·0)	31·4 (0·0 to 45·1)	951·1 (829·2 to 1085·0)	527·7 (418·7 to 655·1)	302·0 (267·3 to 340·3)	77·3 (53·1 to 106·5)	44·0 (0·0 to 63·2)
Saint Lucia*	2048	688·0 (598·4 to 787·2)	382·3 (300·9 to 474·4)	216·2 (191·3 to 243·7)	56·6 (38·8 to 78·4)	32·9 (0·0 to 48·1)	964·5 (838·9 to 1103·6)	535·9 (421·8 to 665·1)	303·1 (268·2 to 341·7)	79·4 (54·3 to 109·9)	46·1 (0·0 to 67·4)
Saint Lucia*	2049	697·9 (602·9 to 799·3)	388·2 (305·2 to 482·3)	216·9 (192·1 to 244·6)	58·1 (39·6 to 81·1)	34·6 (0·0 to 51·2)	978·3 (845·2 to 1120·5)	544·3 (427·9 to 676·1)	304·1 (269·3 to 343·0)	81·5 (55·5 to 113·6)	48·5 (0·0 to 71·7)
Saint Lucia*	2050	707·5 (610·2 to 809·0)	393·8 (306·4 to 487·0)	217·6 (192·9 to 245·5)	59·7 (40·4 to 83·8)	36·4 (0·0 to 55·3)	991·9 (855·4 to 1134·1)	552·1 (429·6 to 682·7)	305·0 (270·4 to 344·2)	83·7 (56·7 to 117·4)	51·1 (0·0 to 77·5)

Sri Lanka*	1995	62·5 (54·8 to 70·5)	34·2 (28·9 to 40·1)	22·0 (17·8 to 27·3)	5·4 (3·7 to 7·7)	0·8 (0·8 to 0·8)	221·5 (194·2 to 249·9)	121·5 (102·6 to 142·4)	77·9 (63·3 to 96·9)	19·1 (13·2 to 27·2)	2·9 (2·9 to 2·9)
Sri Lanka*	1996	65·2 (57·8 to 73·0)	35·4 (30·5 to 41·1)	23·3 (19·1 to 28·4)	5·5 (3·7 to 7·8)	1·0 (1·0 to 1·0)	231·2 (204·8 to 258·8)	125·7 (108·0 to 145·8)	82·7 (67·8 to 100·7)	19·5 (13·3 to 27·5)	3·4 (3·4 to 3·4)
Sri Lanka*	1997	68·4 (61·4 to 75·9)	36·6 (31·6 to 42·1)	24·6 (20·5 to 29·4)	5·5 (3·8 to 7·8)	1·7 (1·7 to 1·7)	242·4 (217·9 to 269·3)	129·6 (112·2 to 149·5)	87·2 (72·5 to 104·1)	19·7 (13·4 to 27·7)	5·9 (5·9 to 5·9)
Sri Lanka*	1998	71·8 (65·1 to 78·8)	37·7 (33·0 to 42·8)	25·8 (21·8 to 30·5)	5·4 (3·8 to 7·6)	2·9 (2·9 to 2·9)	254·7 (230·8 to 279·6)	133·6 (117·0 to 151·8)	91·6 (77·4 to 108·3)	19·2 (13·3 to 27·1)	10·3 (10·3 to 10·3)
Sri Lanka*	1999	70·6 (64·2 to 76·7)	37·6 (33·4 to 42·0)	27·0 (23·1 to 31·4)	5·0 (3·6 to 7·0)	0·9 (0·9 to 0·9)	250·2 (227·8 to 271·8)	133·5 (118·6 to 149·1)	95·8 (81·9 to 111·3)	17·8 (12·7 to 24·8)	3·2 (3·2 to 3·2)
Sri Lanka*	2000	75·6 (69·8 to 81·6)	40·2 (36·4 to 44·3)	29·1 (25·3 to 33·1)	4·6 (3·3 to 6·3)	1·7 (1·7 to 1·7)	268·1 (247·4 to 289·3)	142·6 (129·0 to 157·2)	103·1 (89·8 to 117·4)	16·3 (11·7 to 22·4)	6·0 (6·0 to 6·0)
Sri Lanka*	2001	75·1 (69·8 to 80·3)	40·2 (36·8 to 44·1)	29·6 (26·3 to 33·3)	4·4 (3·2 to 6·0)	0·8 (0·8 to 0·8)	266·3 (247·7 to 284·9)	142·7 (130·3 to 156·3)	105·1 (93·3 to 118·2)	15·6 (11·3 to 21·4)	2·8 (2·8 to 2·8)
Sri Lanka*	2002	75·0 (70·4 to 79·8)	39·9 (36·8 to 43·1)	29·7 (26·6 to 33·1)	4·4 (3·2 to 6·0)	1·0 (1·0 to 1·0)	266·1 (249·6 to 283·0)	141·4 (130·5 to 153·0)	105·5 (94·2 to 117·3)	15·7 (11·4 to 21·2)	3·5 (3·5 to 3·5)
Sri Lanka*	2003	75·9 (71·3 to 80·4)	39·9 (37·0 to 43·1)	30·0 (27·1 to 33·3)	4·5 (3·3 to 5·9)	1·5 (1·5 to 1·5)	269·1 (252·8 to 285·1)	141·6 (131·2 to 152·8)	106·3 (96·1 to 118·3)	15·9 (11·5 to 20·8)	5·3 (5·3 to 5·3)
Sri Lanka*	2004	78·8 (74·0 to 83·5)	41·4 (38·3 to 44·4)	32·2 (29·3 to 35·7)	4·7 (3·4 to 6·1)	0·5 (0·5 to 0·5)	279·4 (262·5 to 296·1)	146·7 (135·7 to 157·6)	114·2 (103·9 to 126·6)	16·6 (12·1 to 21·8)	1·9 (1·9 to 1·9)
Sri Lanka*	2005	83·9 (78·8 to 88·7)	43·1 (40·0 to 46·3)	34·7 (31·6 to 38·1)	4·9 (3·6 to 6·5)	1·1 (1·1 to 1·1)	297·4 (279·5 to 314·5)	153·0 (141·8 to 164·2)	123·1 (112·2 to 135·2)	17·3 (12·9 to 23·1)	4·1 (4·1 to 4·1)
Sri Lanka*	2006	89·6 (84·4 to 94·7)	44·4 (41·3 to 47·8)	38·3 (35·0 to 41·6)	5·0 (3·7 to 6·7)	1·9 (1·9 to 1·9)	317·7 (299·2 to 335·7)	157·4 (146·4 to 169·4)	135·7 (124·2 to 147·4)	17·7 (13·3 to 23·8)	6·8 (6·8 to 6·8)
Sri Lanka*	2007	93·1 (88·2 to 98·2)	43·6 (40·4 to 47·2)	42·8 (39·4 to 46·2)	5·1 (3·8 to 6·9)	1·7 (1·7 to 1·7)	330·2 (312·9 to 348·2)	154·6 (143·4 to 167·5)	151·6 (139·9 to 163·8)	18·0 (13·4 to 24·3)	6·0 (6·0 to 6·0)
Sri Lanka*	2008	97·2 (92·1 to 102·3)	42·0 (38·9 to 45·4)	47·7 (44·4 to 51·5)	5·1 (3·8 to 6·7)	2·4 (2·4 to 2·4)	344·7 (326·5 to 362·9)	149·0 (138·0 to 160·9)	169·1 (157·4 to 182·7)	18·0 (13·4 to 23·8)	8·6 (8·6 to 8·6)

Sri Lanka*	2009	105·1 (99·8 to 110·5)	44·3 (41·0 to 47·6)	53·1 (49·4 to 57·1)	5·3 (4·0 to 7·0)	2·4 (2·4 to 2·4)	372·9 (353·9 to 391·9)	157·1 (145·3 to 168·9)	188·5 (175·3 to 202·3)	18·8 (14·1 to 24·7)	8·5 (8·5 to 8·5)
Sri Lanka*	2010	106·4 (101·3 to 111·7)	43·6 (40·5 to 46·7)	54·5 (50·8 to 58·3)	5·4 (4·1 to 7·0)	2·9 (2·9 to 2·9)	377·2 (359·2 to 396·1)	154·5 (143·5 to 165·5)	193·3 (180·2 to 206·8)	19·1 (14·5 to 24·8)	10·2 (10·2 to 10·2)
Sri Lanka*	2011	110·2 (105·1 to 115·9)	46·5 (43·4 to 49·8)	55·9 (52·2 to 59·8)	5·8 (4·6 to 7·4)	2·0 (2·0 to 2·0)	390·9 (372·6 to 410·8)	164·9 (154·0 to 176·7)	198·3 (185·2 to 212·0)	20·7 (16·2 to 26·3)	6·9 (6·9 to 6·9)
Sri Lanka*	2012	111·0 (106·3 to 116·1)	47·6 (44·7 to 50·6)	56·1 (52·6 to 60·0)	5·9 (4·8 to 7·2)	1·4 (1·4 to 1·4)	393·6 (376·9 to 411·9)	168·7 (158·5 to 179·4)	198·9 (186·6 to 212·9)	20·8 (16·9 to 25·6)	5·1 (5·1 to 5·1)
Sri Lanka*	2013	116·0 (112·2 to 120·5)	50·5 (48·1 to 52·7)	57·6 (54·5 to 60·9)	6·2 (5·3 to 7·2)	1·8 (1·8 to 1·8)	411·5 (398·0 to 427·3)	179·0 (170·6 to 186·9)	204·4 (193·2 to 216·0)	21·9 (18·6 to 25·7)	6·2 (6·2 to 6·2)
Sri Lanka*	2014	122·5 (119·5 to 125·6)	53·6 (51·9 to 55·2)	59·9 (57·6 to 62·5)	6·7 (5·9 to 7·5)	2·3 (2·3 to 2·3)	434·5 (423·9 to 445·3)	190·1 (184·1 to 195·6)	212·6 (204·2 to 221·6)	23·6 (21·0 to 26·5)	8·2 (8·2 to 8·2)
Sri Lanka*	2015	136·6 (133·9 to 139·5)	59·8 (58·5 to 61·3)	65·9 (63·7 to 68·2)	7·7 (7·0 to 8·4)	3·3 (3·3 to 3·3)	484·6 (475·0 to 494·5)	212·1 (207·4 to 217·4)	233·5 (225·8 to 241·8)	27·2 (24·8 to 29·8)	11·7 (11·7 to 11·7)
Sri Lanka*	2016	139·7 (136·0 to 143·3)	59·9 (57·9 to 61·8)	69·4 (66·4 to 72·4)	8·2 (7·4 to 9·1)	2·2 (2·2 to 2·2)	495·4 (482·3 to 508·3)	212·3 (205·4 to 219·2)	246·0 (235·6 to 256·7)	29·1 (26·1 to 32·4)	7·9 (7·9 to 7·9)
Sri Lanka*	2017	146·9 (140·9 to 153·5)	60·9 (57·3 to 64·6)	72·6 (67·7 to 78·0)	8·7 (7·4 to 10·1)	4·7 (4·7 to 4·7)	520·9 (499·5 to 544·5)	216·0 (203·1 to 229·2)	257·3 (240·2 to 276·6)	30·8 (26·1 to 35·7)	16·7 (16·7 to 16·7)
Sri Lanka*	2018	148·3 (138·5 to 158·5)	61·7 (56·1 to 67·5)	74·9 (67·6 to 83·1)	9·1 (7·3 to 11·0)	2·6 (2·6 to 2·6)	525·9 (491·2 to 561·9)	218·9 (198·8 to 239·5)	265·7 (239·7 to 294·8)	32·2 (26·0 to 38·9)	9·1 (9·1 to 9·1)
Sri Lanka*	2019	156·4 (146·2 to 167·2)	68·2 (61·5 to 75·2)	76·2 (68·7 to 84·5)	9·4 (7·6 to 11·4)	2·6 (2·6 to 2·6)	554·7 (518·6 to 592·9)	242·0 (218·0 to 266·8)	270·2 (243·7 to 299·6)	33·4 (26·9 to 40·4)	9·2 (9·2 to 9·3)
Sri Lanka*	2020	168·4 (158·4 to 178·9)	81·6 (74·9 to 88·6)	74·2 (66·9 to 82·3)	9·4 (7·5 to 11·3)	3·3 (3·2 to 3·4)	597·3 (561·7 to 634·5)	289·5 (265·6 to 314·2)	263·0 (237·3 to 291·8)	33·2 (26·8 to 40·0)	11·6 (11·4 to 11·9)
Sri Lanka*	2021	148·0 (138·2 to 158·5)	60·9 (54·4 to 68·0)	74·6 (67·4 to 82·8)	9·6 (7·8 to 11·6)	2·8 (2·7 to 3·0)	524·8 (490·1 to 562·2)	216·0 (192·9 to 241·1)	264·7 (238·9 to 293·6)	34·0 (27·5 to 41·0)	10·1 (9·7 to 10·5)
Sri Lanka*	2022	150·0 (139·6 to 161·0)	62·3 (55·0 to 70·3)	75·5 (68·2 to 83·8)	9·9 (8·0 to 11·9)	2·2 (2·1 to 2·4)	531·8 (495·1 to 571·0)	221·0 (195·1 to 249·4)	267·9 (241·8 to 297·2)	35·1 (28·5 to 42·3)	7·8 (7·3 to 8·4)

Sri Lanka*	2023	152·4 (141·4 to 163·6)	63·4 (55·9 to 72·0)	76·5 (69·1 to 84·8)	10·2 (8·3 to 12·4)	2·3 (2·1 to 2·5)	540·6 (501·5 to 580·3)	224·9 (198·4 to 255·3)	271·4 (245·0 to 300·8)	36·3 (29·3 to 43·8)	8·0 (7·5 to 8·7)
Sri Lanka*	2024	155·0 (143·7 to 166·7)	64·6 (56·7 to 73·0)	77·5 (70·0 to 86·0)	10·6 (8·5 to 12·8)	2·3 (2·1 to 2·5)	549·7 (509·7 to 591·1)	229·0 (201·1 to 259·0)	275·0 (248·2 to 305·1)	37·5 (30·1 to 45·4)	8·2 (7·6 to 9·0)
Sri Lanka*	2025	157·7 (146·5 to 169·7)	65·8 (57·4 to 74·8)	78·6 (70·9 to 87·2)	10·9 (8·8 to 13·3)	2·4 (2·2 to 2·6)	559·2 (519·6 to 601·8)	233·2 (203·5 to 265·2)	278·8 (251·6 to 309·2)	38·7 (31·2 to 47·1)	8·4 (7·7 to 9·3)
Sri Lanka*	2026	160·5 (148·6 to 172·8)	67·1 (58·3 to 76·3)	79·7 (71·9 to 88·4)	11·3 (9·0 to 13·7)	2·4 (2·2 to 2·7)	569·0 (527·0 to 612·7)	237·8 (206·9 to 270·7)	282·6 (255·0 to 313·3)	40·1 (32·1 to 48·7)	8·6 (7·8 to 9·4)
Sri Lanka*	2027	163·4 (151·1 to 176·6)	68·5 (59·7 to 78·5)	80·8 (72·8 to 89·6)	11·7 (9·3 to 14·2)	2·5 (2·2 to 2·7)	579·5 (535·7 to 626·4)	242·9 (211·7 to 278·5)	286·4 (258·3 to 317·8)	41·4 (33·1 to 50·5)	8·7 (7·9 to 9·6)
Sri Lanka*	2028	166·5 (154·0 to 180·0)	70·1 (60·8 to 80·3)	81·9 (73·8 to 90·8)	12·1 (9·7 to 14·7)	2·5 (2·3 to 2·8)	590·6 (546·1 to 638·2)	248·6 (215·7 to 284·7)	290·3 (261·6 to 322·1)	42·8 (34·2 to 52·2)	8·9 (8·0 to 9·9)
Sri Lanka*	2029	169·5 (156·6 to 183·5)	71·5 (62·2 to 83·0)	83·0 (74·8 to 92·1)	12·5 (10·0 to 15·3)	2·5 (2·3 to 2·8)	601·1 (555·3 to 650·7)	253·5 (220·6 to 294·5)	294·3 (265·1 to 326·6)	44·3 (35·4 to 54·2)	9·0 (8·1 to 10·1)
Sri Lanka*	2030	172·5 (159·3 to 186·9)	72·9 (62·9 to 84·7)	84·1 (75·8 to 93·4)	12·9 (10·3 to 15·8)	2·6 (2·3 to 2·9)	611·8 (564·8 to 662·7)	258·5 (222·9 to 300·4)	298·4 (268·8 to 331·2)	45·9 (36·6 to 56·1)	9·1 (8·2 to 10·2)
Sri Lanka*	2031	175·6 (162·1 to 190·7)	74·3 (63·8 to 86·8)	85·3 (76·9 to 94·7)	13·4 (10·6 to 16·4)	2·6 (2·3 to 3·0)	622·6 (574·7 to 676·4)	263·5 (226·2 to 307·8)	302·4 (272·7 to 335·9)	47·5 (37·7 to 58·2)	9·2 (8·2 to 10·5)
Sri Lanka*	2032	178·6 (164·8 to 194·3)	75·7 (64·4 to 88·9)	86·4 (77·9 to 96·0)	13·8 (10·9 to 17·0)	2·6 (2·3 to 3·0)	633·2 (584·5 to 689·1)	268·5 (228·5 to 315·2)	306·3 (276·2 to 340·5)	49·1 (38·8 to 60·4)	9·4 (8·3 to 10·7)
Sri Lanka*	2033	181·6 (167·2 to 197·5)	77·1 (65·0 to 90·3)	87·4 (78·8 to 97·3)	14·3 (11·3 to 17·8)	2·7 (2·4 to 3·1)	643·8 (592·8 to 700·3)	273·5 (230·6 to 320·2)	310·1 (279·6 to 345·0)	50·7 (39·9 to 63·0)	9·6 (8·4 to 11·0)
Sri Lanka*	2034	184·6 (169·8 to 201·2)	78·5 (66·3 to 92·1)	88·5 (79·7 to 98·5)	14·8 (11·6 to 18·4)	2·7 (2·4 to 3·2)	654·5 (602·1 to 713·5)	278·5 (235·1 to 326·8)	313·8 (282·8 to 349·3)	52·4 (41·0 to 65·4)	9·7 (8·6 to 11·3)
Sri Lanka*	2035	187·4 (171·9 to 204·6)	79·8 (67·0 to 94·6)	89·5 (80·6 to 99·6)	15·3 (11·9 to 19·0)	2·8 (2·5 to 3·3)	664·6 (609·7 to 725·5)	283·1 (237·6 to 335·6)	317·3 (285·7 to 353·3)	54·1 (42·1 to 67·5)	10·0 (8·7 to 11·6)
Sri Lanka*	2036	190·1 (174·2 to 208·0)	81·1 (67·9 to 96·2)	90·4 (81·4 to 100·7)	15·8 (12·2 to 19·7)	2·9 (2·5 to 3·4)	674·3 (617·7 to 737·5)	287·5 (240·8 to 341·0)	320·7 (288·5 to 357·0)	55·9 (43·3 to 69·8)	10·2 (8·9 to 12·0)

Sri Lanka*	2037	192·7 (176·4 to 211·2)	82·3 (69·0 to 98·1)	91·2 (82·0 to 101·6)	16·2 (12·5 to 20·3)	3·0 (2·6 to 3·5)	683·5 (625·5 to 749·0)	291·8 (244·7 to 347·9)	323·5 (290·9 to 360·2)	57·6 (44·4 to 72·2)	10·6 (9·1 to 12·4)
Sri Lanka*	2038	195·4 (178·7 to 214·8)	83·6 (69·7 to 100·3)	92·1 (82·8 to 102·5)	16·7 (12·9 to 21·0)	3·1 (2·6 to 3·6)	693·1 (633·9 to 761·6)	296·4 (247·1 to 355·6)	326·4 (293·7 to 363·4)	59·4 (45·6 to 74·6)	10·9 (9·3 to 12·8)
Sri Lanka*	2039	198·5 (181·4 to 218·2)	85·1 (71·0 to 102·4)	93·0 (83·7 to 103·6)	17·3 (13·2 to 21·8)	3·2 (2·7 to 3·8)	703·9 (643·1 to 773·8)	301·7 (251·9 to 363·2)	329·7 (296·9 to 367·2)	61·3 (46·9 to 77·2)	11·2 (9·6 to 13·4)
Sri Lanka*	2040	201·6 (184·3 to 221·7)	86·6 (72·3 to 103·8)	94·0 (84·6 to 104·7)	17·8 (13·6 to 22·5)	3·3 (2·8 to 3·9)	715·1 (653·5 to 786·1)	307·0 (256·4 to 368·0)	333·3 (300·0 to 371·1)	63·3 (48·2 to 79·7)	11·6 (9·9 to 13·8)
Sri Lanka*	2041	204·8 (186·9 to 225·2)	88·0 (73·1 to 105·5)	95·0 (85·5 to 105·7)	18·4 (13·9 to 23·4)	3·4 (2·8 to 4·0)	726·2 (662·7 to 798·5)	312·1 (259·1 to 374·3)	336·9 (303·2 to 375·0)	65·3 (49·4 to 82·8)	11·9 (10·1 to 14·2)
Sri Lanka*	2042	208·0 (189·8 to 229·3)	89·5 (74·3 to 107·5)	96·0 (86·3 to 106·9)	19·0 (14·3 to 24·3)	3·5 (2·9 to 4·2)	737·5 (673·0 to 813·2)	317·5 (263·5 to 381·2)	340·3 (306·2 to 379·0)	67·5 (50·6 to 86·0)	12·3 (10·3 to 14·8)
Sri Lanka*	2043	211·1 (192·2 to 233·0)	91·0 (74·9 to 110·4)	96·9 (87·2 to 108·0)	19·6 (14·7 to 25·2)	3·6 (3·0 to 4·4)	748·7 (681·8 to 826·1)	322·7 (265·5 to 391·3)	343·7 (309·1 to 382·9)	69·6 (52·0 to 89·3)	12·7 (10·5 to 15·5)
Sri Lanka*	2044	214·2 (195·4 to 236·3)	92·4 (75·6 to 112·0)	97·8 (87·9 to 108·9)	20·2 (15·1 to 26·0)	3·7 (3·1 to 4·6)	759·5 (693·0 to 838·0)	327·7 (268·2 to 397·2)	346·9 (311·8 to 386·3)	71·7 (53·4 to 92·2)	13·2 (11·0 to 16·2)
Sri Lanka*	2045	217·2 (197·5 to 239·8)	93·8 (76·6 to 113·6)	98·7 (88·7 to 109·9)	20·8 (15·4 to 27·0)	3·9 (3·2 to 4·8)	770·3 (700·3 to 850·5)	332·6 (271·7 to 402·9)	350·1 (314·6 to 389·7)	73·9 (54·6 to 95·9)	13·7 (11·3 to 16·9)
Sri Lanka*	2046	220·4 (200·0 to 244·2)	95·3 (78·0 to 115·4)	99·6 (89·5 to 110·8)	21·5 (15·8 to 27·9)	4·0 (3·3 to 5·0)	781·5 (709·4 to 866·0)	337·9 (276·5 to 409·1)	353·2 (317·2 to 393·1)	76·2 (55·9 to 99·1)	14·3 (11·8 to 17·9)
Sri Lanka*	2047	223·7 (202·9 to 248·5)	96·8 (79·1 to 118·1)	100·5 (90·3 to 112·0)	22·2 (16·1 to 29·1)	4·2 (3·4 to 5·3)	793·1 (719·5 to 881·1)	343·2 (280·6 to 418·9)	356·5 (320·1 to 397·0)	78·6 (57·1 to 103·2)	14·8 (12·1 to 18·6)
Sri Lanka*	2048	227·0 (206·0 to 252·9)	98·3 (79·5 to 119·2)	101·5 (91·2 to 113·1)	22·9 (16·5 to 30·2)	4·3 (3·5 to 5·5)	805·0 (730·4 to 896·7)	348·6 (281·9 to 422·8)	360·0 (323·3 to 401·1)	81·1 (58·5 to 107·0)	15·4 (12·5 to 19·6)
Sri Lanka*	2049	230·5 (208·3 to 256·6)	99·9 (80·5 to 121·9)	102·5 (92·1 to 114·3)	23·6 (16·9 to 31·3)	4·5 (3·6 to 5·8)	817·4 (738·7 to 910·1)	354·2 (285·6 to 432·3)	363·6 (326·6 to 405·2)	83·7 (59·8 to 111·1)	15·9 (12·9 to 20·4)
Sri Lanka*	2050	234·1 (211·1 to 261·1)	101·5 (81·5 to 123·7)	103·5 (93·0 to 115·4)	24·3 (17·3 to 32·5)	4·7 (3·8 to 6·0)	830·1 (748·8 to 925·8)	360·1 (289·0 to 438·6)	367·1 (329·8 to 409·3)	86·3 (61·4 to 115·3)	16·6 (13·3 to 21·3)

Lesotho*	1995	33·6 (29·3 to 38·3)	16·2 (13·4 to 19·6)	14·4 (11·6 to 17·6)	1·3 (0·9 to 1·9)	1·7 (1·7 to 1·7)	105·0 (91·3 to 119·7)	50·7 (41·8 to 61·3)	45·0 (36·1 to 55·1)	4·2 (2·9 to 5·8)	5·2 (5·2 to 5·2)
Lesotho*	1996	35·3 (30·9 to 39·9)	16·9 (14·0 to 20·3)	14·6 (11·7 to 17·7)	2·3 (1·5 to 3·1)	1·5 (1·5 to 1·5)	110·3 (96·5 to 124·5)	52·8 (43·6 to 63·4)	45·6 (36·6 to 55·2)	7·1 (4·8 to 9·8)	4·7 (4·7 to 4·7)
Lesotho*	1997	37·0 (32·7 to 41·6)	17·3 (14·4 to 20·8)	14·4 (11·7 to 17·3)	3·4 (2·3 to 4·8)	1·9 (1·9 to 1·9)	115·7 (102·0 to 129·8)	54·1 (45·0 to 64·9)	44·9 (36·5 to 54·2)	10·5 (7·2 to 14·9)	6·1 (6·1 to 6·1)
Lesotho*	1998	37·9 (33·5 to 42·6)	18·5 (15·3 to 22·0)	13·9 (11·5 to 16·7)	4·2 (2·9 to 6·0)	1·2 (1·2 to 1·2)	118·2 (104·5 to 133·1)	57·6 (47·7 to 68·6)	43·5 (36·0 to 52·1)	13·2 (9·0 to 18·6)	3·9 (3·9 to 3·9)
Lesotho*	1999	37·6 (33·2 to 42·3)	19·1 (15·9 to 22·7)	13·4 (11·3 to 15·7)	4·7 (3·2 to 6·6)	0·4 (0·4 to 0·4)	117·4 (103·6 to 132·2)	59·7 (49·6 to 70·9)	41·8 (35·4 to 49·0)	14·6 (10·0 to 20·5)	1·3 (1·3 to 1·3)
Lesotho*	2000	37·9 (33·9 to 42·4)	18·4 (15·3 to 22·0)	12·5 (10·9 to 14·3)	4·7 (3·2 to 6·6)	2·3 (2·3 to 2·3)	118·3 (106·0 to 132·4)	57·5 (47·8 to 68·8)	39·1 (34·0 to 44·8)	14·6 (9·9 to 20·7)	7·1 (7·1 to 7·1)
Lesotho*	2001	41·5 (37·5 to 46·0)	20·8 (17·4 to 25·0)	12·4 (11·2 to 13·8)	4·6 (3·1 to 6·5)	3·6 (3·6 to 3·6)	129·5 (117·0 to 143·7)	65·1 (54·4 to 78·0)	38·8 (34·9 to 43·0)	14·5 (9·8 to 20·3)	11·1 (11·1 to 11·1)
Lesotho*	2002	41·5 (37·5 to 45·9)	21·2 (17·7 to 25·3)	12·1 (11·0 to 13·1)	4·2 (2·8 to 5·8)	4·1 (4·1 to 4·1)	129·6 (117·0 to 143·3)	66·1 (55·3 to 79·1)	37·6 (34·4 to 41·0)	13·1 (8·9 to 18·2)	12·7 (12·7 to 12·7)
Lesotho*	2003	43·5 (39·4 to 47·8)	21·2 (17·7 to 25·3)	11·9 (10·6 to 13·1)	3·1 (2·1 to 4·3)	7·4 (7·4 to 7·4)	135·7 (123·1 to 149·3)	66·1 (55·3 to 78·9)	37·0 (33·2 to 41·0)	9·6 (6·5 to 13·3)	23·0 (23·0 to 23·0)
Lesotho*	2004	43·5 (39·5 to 47·9)	20·9 (17·4 to 24·8)	11·7 (10·1 to 13·4)	2·4 (1·6 to 3·3)	8·6 (8·6 to 8·6)	135·9 (123·3 to 149·7)	65·2 (54·3 to 77·4)	36·6 (31·6 to 41·8)	7·4 (5·0 to 10·3)	26·7 (26·7 to 26·7)
Lesotho*	2005	44·8 (40·4 to 49·7)	22·0 (18·2 to 26·2)	12·0 (10·1 to 14·2)	2·2 (1·5 to 3·0)	8·6 (8·6 to 8·6)	140·0 (126·0 to 155·2)	68·6 (57·0 to 81·8)	37·6 (31·6 to 44·2)	6·8 (4·6 to 9·5)	27·0 (27·0 to 27·0)
Lesotho*	2006	50·2 (45·1 to 55·9)	25·4 (21·1 to 30·0)	12·7 (10·4 to 15·2)	2·2 (1·5 to 3·1)	10·0 (10·0 to 10·0)	156·8 (140·8 to 174·6)	79·2 (65·8 to 93·8)	39·5 (32·4 to 47·5)	6·9 (4·7 to 9·6)	31·1 (31·1 to 31·1)
Lesotho*	2007	61·0 (55·4 to 67·4)	29·7 (24·8 to 35·0)	13·2 (10·6 to 16·1)	2·2 (1·5 to 3·1)	15·9 (15·9 to 15·9)	190·5 (172·9 to 210·5)	92·6 (77·3 to 109·2)	41·3 (33·2 to 50·3)	6·9 (4·7 to 9·7)	49·7 (49·7 to 49·7)
Lesotho*	2008	76·8 (70·5 to 83·9)	34·6 (28·9 to 40·7)	13·9 (11·2 to 17·0)	2·0 (1·4 to 2·9)	26·3 (26·3 to 26·3)	239·9 (220·3 to 261·9)	108·1 (90·1 to 127·1)	43·4 (35·0 to 53·1)	6·3 (4·3 to 9·1)	82·0 (82·0 to 82·0)

Lesotho*	2009	79·6 (72·4 to 87·4)	41·1 (34·7 to 47·8)	14·8 (11·8 to 18·2)	1·3 (0·9 to 1·9)	22·3 (22·3 to 22·3)	248·4 (226·2 to 272·8)	128·2 (108·2 to 149·4)	46·2 (36·7 to 56·9)	4·2 (2·9 to 5·9)	69·8 (69·8 to 69·8)
Lesotho*	2010	95·1 (88·5 to 102·2)	40·5 (34·4 to 47·0)	14·7 (11·7 to 18·1)	0·8 (0·5 to 1·1)	39·1 (39·1 to 39·1)	296·8 (276·4 to 319·1)	126·4 (107·4 to 146·8)	46·0 (36·4 to 56·6)	2·4 (1·7 to 3·5)	122·0 (122·0 to 122·0)
Lesotho*	2011	112·4 (104·6 to 120·3)	47·5 (40·9 to 54·9)	15·6 (12·2 to 19·1)	0·2 (0·1 to 0·3)	49·1 (49·1 to 49·1)	351·0 (326·7 to 375·7)	148·3 (127·6 to 171·5)	48·6 (38·1 to 59·7)	0·6 (0·4 to 0·9)	153·4 (153·4 to 153·4)
Lesotho*	2012	114·0 (105·6 to 122·6)	51·2 (43·7 to 59·2)	15·7 (12·3 to 19·4)	0·0 (0·0 to 0·0)	47·1 (47·1 to 47·1)	355·9 (329·9 to 382·7)	159·9 (136·5 to 184·9)	48·9 (38·5 to 60·5)	0·0 (0·0 to 0·0)	147·0 (147·0 to 147·0)
Lesotho*	2013	135·0 (126·2 to 143·6)	56·1 (47·8 to 64·4)	15·7 (12·3 to 19·2)	0·0 (0·0 to 0·0)	63·1 (63·1 to 63·1)	421·5 (394·1 to 448·5)	175·3 (149·1 to 201·1)	49·1 (38·5 to 59·9)	0·0 (0·0 to 0·0)	197·1 (197·1 to 197·1)
Lesotho*	2014	90·9 (82·4 to 99·3)	56·3 (48·5 to 64·3)	15·4 (12·1 to 18·9)	0·0 (0·0 to 0·0)	19·2 (19·2 to 19·2)	283·9 (257·4 to 310·1)	175·7 (151·3 to 200·8)	48·1 (37·8 to 59·0)	0·0 (0·0 to 0·0)	60·1 (60·1 to 60·1)
Lesotho*	2015	94·7 (85·6 to 103·6)	60·7 (52·4 to 69·5)	15·9 (12·5 to 19·5)	0·0 (0·0 to 0·0)	18·2 (18·2 to 18·2)	295·8 (267·2 to 323·5)	189·4 (163·6 to 216·9)	49·6 (39·1 to 60·8)	0·0 (0·0 to 0·0)	56·8 (56·8 to 56·8)
Lesotho*	2016	104·2 (94·9 to 113·7)	63·0 (54·7 to 72·1)	16·4 (13·0 to 20·1)	0·0 (0·0 to 0·0)	24·8 (24·8 to 24·8)	325·4 (296·4 to 355·0)	196·6 (170·7 to 225·3)	51·2 (40·6 to 62·7)	0·0 (0·0 to 0·0)	77·5 (77·5 to 77·5)
Lesotho*	2017	116·2 (106·3 to 126·5)	62·9 (54·2 to 72·4)	16·7 (13·1 to 20·5)	0·0 (0·0 to 0·0)	36·6 (36·6 to 36·6)	362·8 (332·0 to 395·0)	196·4 (169·2 to 226·1)	52·1 (40·9 to 64·1)	0·0 (0·0 to 0·0)	114·2 (114·2 to 114·2)
Lesotho*	2018	121·0 (111·1 to 131·8)	64·4 (54·4 to 74·5)	17·1 (13·5 to 20·9)	0·0 (0·0 to 0·0)	39·5 (39·5 to 39·5)	377·9 (347·0 to 411·5)	201·1 (169·9 to 232·7)	53·3 (42·3 to 65·3)	0·0 (0·0 to 0·0)	123·5 (123·5 to 123·5)
Lesotho*	2019	125·0 (114·8 to 136·2)	65·8 (55·6 to 76·2)	17·2 (13·6 to 21·0)	0·0 (0·0 to 0·0)	42·0 (41·7 to 42·5)	390·3 (358·3 to 425·2)	205·4 (173·7 to 238·0)	53·6 (42·5 to 65·7)	0·0 (0·0 to 0·0)	131·3 (130·2 to 132·8)
Lesotho*	2020	140·2 (128·9 to 151·7)	70·8 (59·5 to 81·9)	16·6 (13·2 to 20·4)	0·0 (0·0 to 0·0)	52·7 (51·6 to 55·0)	437·7 (402·5 to 473·8)	221·1 (185·9 to 255·9)	51·9 (41·1 to 63·6)	0·0 (0·0 to 0·0)	164·7 (161·0 to 171·8)
Lesotho*	2021	134·0 (122·7 to 145·4)	70·8 (59·6 to 82·0)	16·6 (13·1 to 20·3)	0·0 (0·0 to 0·0)	46·6 (45·2 to 48·8)	418·3 (383·3 to 454·1)	221·2 (186·0 to 255·9)	51·7 (41·0 to 63·5)	0·0 (0·0 to 0·0)	145·4 (141·2 to 152·3)
Lesotho*	2022	120·9 (110·4 to 132·3)	68·1 (57·5 to 78·8)	16·8 (13·3 to 20·6)	0·0 (0·0 to 0·0)	36·0 (33·9 to 38·3)	377·5 (344·8 to 413·2)	212·8 (179·7 to 246·0)	52·4 (41·5 to 64·3)	0·0 (0·0 to 0·0)	112·3 (105·9 to 119·6)

Lesotho*	2023	123·6 (112·6 to 135·5)	70·4 (59·6 to 81·5)	17·0 (13·5 to 20·9)	0·0 (0·0 to 0·0)	36·2 (33·8 to 38·8)	386·0 (351·5 to 423·2)	220·0 (186·0 to 254·4)	53·1 (42·1 to 65·1)	0·0 (0·0 to 0·0)	112·9 (105·7 to 121·1)
Lesotho*	2024	126·4 (115·0 to 139·1)	72·8 (61·5 to 84·4)	17·2 (13·7 to 21·1)	0·0 (0·0 to 0·0)	36·3 (33·7 to 39·3)	394·6 (359·0 to 434·3)	227·4 (191·9 to 263·5)	53·8 (42·7 to 66·0)	0·0 (0·0 to 0·0)	113·4 (105·3 to 122·6)
Lesotho*	2025	129·2 (117·1 to 142·3)	75·3 (63·8 to 87·5)	17·5 (13·9 to 21·4)	0·0 (0·0 to 0·0)	36·4 (33·6 to 39·6)	403·4 (365·6 to 444·3)	235·2 (199·1 to 273·1)	54·6 (43·3 to 66·9)	0·0 (0·0 to 0·0)	113·6 (105·0 to 123·8)
Lesotho*	2026	132·0 (119·3 to 145·6)	77·9 (65·9 to 90·4)	17·7 (14·1 to 21·8)	0·0 (0·0 to 0·0)	36·4 (33·4 to 40·0)	412·1 (372·6 to 454·7)	243·2 (205·7 to 282·4)	55·4 (44·0 to 67·9)	0·0 (0·0 to 0·0)	113·6 (104·2 to 124·9)
Lesotho*	2027	134·8 (121·8 to 149·0)	80·5 (68·0 to 93·5)	18·0 (14·3 to 22·1)	0·0 (0·0 to 0·0)	36·3 (33·2 to 40·2)	421·0 (380·3 to 465·3)	251·4 (212·2 to 291·9)	56·1 (44·6 to 68·9)	0·0 (0·0 to 0·0)	113·4 (103·5 to 125·4)
Lesotho*	2028	137·7 (123·8 to 152·4)	83·3 (70·1 to 96·9)	18·2 (14·5 to 22·4)	0·0 (0·0 to 0·0)	36·2 (32·8 to 40·5)	430·0 (386·7 to 476·0)	260·0 (219·0 to 302·6)	56·9 (45·2 to 69·8)	0·0 (0·0 to 0·0)	113·1 (102·4 to 126·3)
Lesotho*	2029	140·7 (126·2 to 156·3)	86·1 (72·5 to 100·4)	18·5 (14·7 to 22·7)	0·0 (0·0 to 0·0)	36·1 (32·4 to 40·8)	439·4 (394·1 to 488·2)	269·0 (226·3 to 313·5)	57·8 (45·9 to 70·8)	0·0 (0·0 to 0·1)	112·6 (101·2 to 127·5)
Lesotho*	2030	143·7 (128·6 to 160·0)	89·1 (74·8 to 104·0)	18·8 (14·9 to 23·0)	0·0 (0·0 to 0·0)	35·8 (31·9 to 41·0)	448·8 (401·4 to 499·4)	278·2 (233·4 to 324·6)	58·7 (46·6 to 71·8)	0·0 (0·0 to 0·1)	111·9 (99·7 to 128·0)
Lesotho*	2031	146·9 (130·9 to 163·6)	92·2 (77·3 to 108·1)	19·1 (15·1 to 23·4)	0·0 (0·0 to 0·0)	35·6 (31·5 to 40·9)	458·7 (408·7 to 510·9)	287·9 (241·3 to 337·4)	59·5 (47·3 to 72·9)	0·0 (0·0 to 0·1)	111·2 (98·5 to 127·8)
Lesotho*	2032	150·1 (133·6 to 167·3)	95·4 (79·6 to 111·8)	19·4 (15·4 to 23·7)	0·0 (0·0 to 0·0)	35·4 (31·1 to 41·5)	468·8 (417·1 to 522·5)	297·8 (248·5 to 349·2)	60·5 (48·0 to 74·0)	0·0 (0·0 to 0·1)	110·5 (97·1 to 129·6)
Lesotho*	2033	153·4 (136·3 to 171·5)	98·6 (82·1 to 115·9)	19·7 (15·6 to 24·1)	0·0 (0·0 to 0·0)	35·2 (30·6 to 41·5)	479·1 (425·6 to 535·6)	307·9 (256·4 to 362·0)	61·4 (48·8 to 75·2)	0·0 (0·0 to 0·1)	109·8 (95·5 to 129·6)
Lesotho*	2034	156·9 (139·0 to 175·8)	102·0 (84·5 to 120·0)	20·0 (15·9 to 24·5)	0·0 (0·0 to 0·0)	34·9 (30·4 to 41·8)	489·8 (433·9 to 548·8)	318·4 (263·9 to 374·6)	62·4 (49·6 to 76·4)	0·0 (0·0 to 0·1)	109·0 (95·0 to 130·6)
Lesotho*	2035	160·4 (141·0 to 180·1)	105·4 (87·2 to 123·9)	20·3 (16·1 to 24·9)	0·0 (0·0 to 0·0)	34·7 (30·1 to 42·1)	500·8 (440·3 to 562·3)	329·0 (272·2 to 386·8)	63·4 (50·4 to 77·6)	0·0 (0·0 to 0·1)	108·3 (93·9 to 131·4)
Lesotho*	2036	163·9 (143·9 to 184·9)	108·8 (89·9 to 128·2)	20·6 (16·4 to 25·2)	0·0 (0·0 to 0·0)	34·5 (29·6 to 42·1)	511·9 (449·2 to 577·4)	339·8 (280·8 to 400·3)	64·4 (51·2 to 78·8)	0·0 (0·0 to 0·1)	107·6 (92·3 to 131·6)

Lesotho*	2037	167·6 (146·4 to 189·7)	112·3 (92·6 to 132·5)	20·9 (16·7 to 25·6)	0·0 (0·0 to 0·0)	34·4 (29·3 to 42·5)	523·3 (457·1 to 592·3)	350·5 (289·2 to 413·8)	65·4 (52·0 to 80·0)	0·0 (0·0 to 0·1)	107·3 (91·6 to 132·7)
Lesotho*	2038	171·3 (149·2 to 194·2)	115·8 (95·5 to 137·0)	21·3 (16·9 to 26·0)	0·0 (0·0 to 0·0)	34·2 (29·0 to 42·7)	534·9 (465·9 to 606·4)	361·7 (298·1 to 427·7)	66·4 (52·8 to 81·3)	0·0 (0·0 to 0·1)	106·7 (90·5 to 133·4)
Lesotho*	2039	175·1 (152·4 to 198·8)	119·5 (98·4 to 141·5)	21·6 (17·2 to 26·5)	0·0 (0·0 to 0·0)	34·0 (28·6 to 43·4)	546·9 (476·0 to 620·9)	373·0 (307·3 to 441·8)	67·5 (53·7 to 82·6)	0·0 (0·0 to 0·1)	106·3 (89·4 to 135·7)
Lesotho*	2040	179·1 (155·6 to 203·7)	123·1 (101·2 to 145·9)	22·0 (17·5 to 26·9)	0·0 (0·0 to 0·0)	34·0 (28·4 to 43·3)	559·1 (485·8 to 636·0)	384·3 (316·0 to 455·6)	68·6 (54·5 to 83·9)	0·0 (0·0 to 0·1)	106·1 (88·6 to 135·3)
Lesotho*	2041	183·0 (158·8 to 208·2)	126·7 (104·0 to 150·3)	22·3 (17·7 to 27·3)	0·0 (0·0 to 0·0)	34·0 (28·2 to 43·8)	571·4 (495·7 to 650·2)	395·6 (324·8 to 469·3)	69·7 (55·3 to 85·3)	0·0 (0·0 to 0·1)	106·0 (87·9 to 136·8)
Lesotho*	2042	187·0 (162·1 to 213·9)	130·4 (106·9 to 154·7)	22·7 (18·0 to 27·7)	0·0 (0·0 to 0·0)	34·0 (27·9 to 44·1)	584·0 (506·1 to 668·0)	407·1 (333·9 to 483·1)	70·8 (56·2 to 86·6)	0·0 (0·0 to 0·1)	106·1 (87·0 to 137·7)
Lesotho*	2043	191·1 (165·5 to 218·8)	134·0 (109·8 to 159·1)	23·0 (18·3 to 28·2)	0·0 (0·0 to 0·0)	34·1 (27·8 to 44·5)	596·7 (516·7 to 683·2)	418·3 (342·8 to 496·9)	71·9 (57·1 to 87·9)	0·1 (0·0 to 0·1)	106·4 (86·7 to 139·0)
Lesotho*	2044	195·2 (168·4 to 223·6)	137·5 (112·6 to 163·4)	23·4 (18·6 to 28·6)	0·0 (0·0 to 0·0)	34·3 (27·9 to 45·4)	609·5 (526·0 to 698·3)	429·4 (351·6 to 510·2)	73·0 (57·9 to 89·2)	0·1 (0·0 to 0·1)	107·0 (87·0 to 141·7)
Lesotho*	2045	199·2 (171·8 to 229·1)	141·0 (115·4 to 167·7)	23·7 (18·8 to 29·0)	0·0 (0·0 to 0·0)	34·6 (27·8 to 46·5)	622·1 (536·3 to 715·4)	440·2 (360·3 to 523·6)	74·0 (58·8 to 90·6)	0·1 (0·0 to 0·1)	107·9 (86·9 to 145·1)
Lesotho*	2046	203·4 (175·1 to 235·1)	144·5 (118·1 to 172·0)	24·0 (19·1 to 29·5)	0·0 (0·0 to 0·0)	34·9 (28·1 to 47·8)	635·0 (546·6 to 734·2)	451·0 (368·8 to 537·1)	75·1 (59·6 to 92·0)	0·1 (0·0 to 0·1)	108·9 (87·9 to 149·3)
Lesotho*	2047	207·5 (178·1 to 239·4)	147·9 (120·8 to 176·2)	24·4 (19·4 to 29·9)	0·0 (0·0 to 0·0)	35·2 (28·1 to 48·5)	647·9 (556·0 to 747·6)	461·7 (377·2 to 550·0)	76·1 (60·4 to 93·3)	0·1 (0·0 to 0·1)	110·0 (87·8 to 151·5)
Lesotho*	2048	211·6 (181·1 to 244·7)	151·2 (123·5 to 180·1)	24·7 (19·6 to 30·3)	0·0 (0·0 to 0·0)	35·6 (28·4 to 49·7)	660·6 (565·4 to 763·9)	472·1 (385·5 to 562·4)	77·1 (61·2 to 94·5)	0·1 (0·0 to 0·1)	111·3 (88·6 to 155·0)
Lesotho*	2049	215·7 (184·4 to 249·6)	154·4 (126·1 to 183·9)	25·0 (19·9 to 30·7)	0·0 (0·0 to 0·0)	36·2 (28·6 to 50·8)	673·4 (575·9 to 779·4)	482·2 (393·6 to 574·2)	78·1 (62·0 to 95·7)	0·1 (0·0 to 0·2)	113·0 (89·2 to 158·7)
Lesotho*	2050	219·8 (187·7 to 254·7)	157·6 (128·7 to 187·6)	25·3 (20·1 to 31·0)	0·0 (0·0 to 0·1)	36·8 (29·0 to 52·2)	686·2 (586·2 to 795·2)	492·1 (401·8 to 585·7)	79·1 (62·8 to 96·9)	0·1 (0·0 to 0·2)	115·0 (90·4 to 163·0)

Lithuania	1995	312·9 (286·0 to 341·7)	213·8 (189·8 to 239·5)	82·5 (69·6 to 96·7)	15·3 (10·9 to 21·6)	1·3 (1·3 to 1·3)	607·0 (554·8 to 662·8)	414·7 (368·1 to 464·5)	160·0 (135·0 to 187·6)	29·7 (21·1 to 42·0)	2·5 (2·5 to 2·5)
Lithuania	1996	330·1 (306·0 to 354·7)	225·5 (205·4 to 248·5)	88·7 (76·3 to 102·0)	15·9 (11·4 to 22·0)	0·0 (0·0 to 0·0)	640·2 (593·6 to 688·0)	437·3 (398·3 to 482·0)	172·0 (148·1 to 197·8)	30·8 (22·1 to 42·6)	0·0 (0·0 to 0·0)
Lithuania	1997	363·5 (340·1 to 387·1)	251·5 (232·3 to 274·1)	95·5 (83·4 to 108·5)	16·5 (11·9 to 23·0)	0·0 (0·0 to 0·0)	705·1 (659·6 to 750·8)	487·9 (450·5 to 531·8)	185·2 (161·7 to 210·4)	32·0 (23·1 to 44·6)	0·0 (0·0 to 0·0)
Lithuania	1998	416·9 (391·1 to 442·6)	293·5 (271·7 to 317·0)	105·5 (93·7 to 118·1)	17·9 (13·0 to 24·5)	0·0 (0·0 to 0·0)	808·6 (758·6 to 858·6)	569·3 (527·1 to 614·8)	204·7 (181·8 to 229·0)	34·6 (25·3 to 47·6)	0·0 (0·0 to 0·0)
Lithuania	1999	445·9 (420·9 to 472·7)	313·1 (291·4 to 338·8)	114·0 (102·8 to 125·7)	18·8 (13·5 to 26·3)	0·0 (0·0 to 0·0)	864·9 (816·3 to 916·8)	607·3 (565·1 to 657·1)	221·1 (199·4 to 243·7)	36·5 (26·3 to 50·9)	0·0 (0·0 to 0·0)
Lithuania	2000	467·2 (442·9 to 495·0)	325·5 (304·1 to 350·0)	122·2 (111·8 to 133·1)	19·2 (14·0 to 26·1)	0·3 (0·3 to 0·3)	906·2 (859·0 to 960·1)	631·3 (589·8 to 678·9)	237·0 (216·8 to 258·1)	37·2 (27·2 to 50·6)	0·6 (0·6 to 0·6)
Lithuania	2001	498·2 (473·6 to 524·8)	351·8 (329·6 to 376·5)	131·8 (121·8 to 142·7)	14·4 (10·5 to 19·1)	0·2 (0·2 to 0·2)	966·3 (918·7 to 1018·0)	682·4 (639·2 to 730·4)	255·6 (236·3 to 276·8)	27·9 (20·3 to 37·0)	0·4 (0·4 to 0·4)
Lithuania	2002	533·3 (509·0 to 560·2)	376·6 (354·5 to 401·2)	142·7 (132·8 to 153·4)	12·5 (9·2 to 16·4)	1·5 (1·5 to 1·5)	1034·3 (987·4 to 1086·7)	730·4 (687·7 to 778·2)	276·8 (257·5 to 297·6)	24·3 (17·8 to 31·8)	2·9 (2·9 to 2·9)
Lithuania	2003	575·1 (549·9 to 602·7)	399·2 (375·2 to 424·8)	160·1 (149·4 to 170·1)	14·3 (10·7 to 18·4)	1·5 (1·5 to 1·5)	1115·5 (1066·7 to 1169·1)	774·3 (727·8 to 823·9)	310·5 (289·8 to 329·9)	27·7 (20·7 to 35·7)	3·0 (3·0 to 3·0)
Lithuania	2004	613·7 (586·4 to 641·2)	402·1 (376·7 to 426·8)	184·8 (174·5 to 196·4)	24·3 (18·3 to 31·2)	2·5 (2·5 to 2·5)	1190·4 (1137·5 to 1243·8)	779·9 (730·6 to 827·8)	358·5 (338·5 to 380·9)	47·1 (35·4 to 60·5)	4·8 (4·8 to 4·8)
Lithuania	2005	667·7 (638·6 to 696·1)	423·5 (398·5 to 449·2)	208·5 (197·9 to 219·1)	35·0 (27·2 to 44·8)	0·7 (0·7 to 0·7)	1295·0 (1238·6 to 1350·1)	821·4 (773·0 to 871·3)	404·4 (383·9 to 425·0)	67·8 (52·7 to 86·9)	1·4 (1·4 to 1·4)
Lithuania	2006	734·5 (705·5 to 762·5)	468·9 (444·5 to 494·0)	227·3 (216·4 to 239·2)	37·7 (29·2 to 48·2)	0·7 (0·7 to 0·7)	1424·7 (1368·4 to 1479·0)	909·5 (862·1 to 958·3)	440·9 (419·8 to 464·0)	73·1 (56·7 to 93·6)	1·3 (1·3 to 1·3)
Lithuania	2007	827·2 (799·3 to 854·8)	551·4 (527·9 to 575·4)	242·8 (231·5 to 254·4)	33·0 (25·9 to 41·9)	0·0 (0·0 to 0·0)	1604·5 (1550·3 to 1658·0)	1069·5 (1023·9 to 1116·1)	470·9 (449·1 to 493·4)	64·0 (50·2 to 81·2)	0·0 (0·0 to 0·0)
Lithuania	2008	914·1 (888·7 to 940·3)	638·9 (617·1 to 663·3)	255·5 (244·4 to 267·8)	18·3 (14·3 to 22·9)	1·4 (1·4 to 1·4)	1773·1 (1723·8 to 1823·8)	1239·2 (1196·9 to 1286·5)	495·6 (474·1 to 519·4)	35·5 (27·7 to 44·4)	2·8 (2·8 to 2·8)

Lithuania	2009	926·8 (901·8 to 953·0)	656·5 (634·4 to 680·0)	251·7 (240·3 to 263·1)	14·5 (11·4 to 18·1)	4·0 (4·0 to 4·0)	1797·6 (1749·1 to 1848·4)	1273·5 (1230·5 to 1319·0)	488·2 (466·1 to 510·4)	28·2 (22·1 to 35·0)	7·7 (7·7 to 7·7)
Lithuania	2010	905·5 (879·7 to 931·5)	638·5 (615·6 to 661·7)	251·4 (239·3 to 263·2)	12·7 (10·2 to 15·4)	2·9 (2·9 to 2·9)	1756·3 (1706·3 to 1806·8)	1238·5 (1194·1 to 1283·4)	487·6 (464·1 to 510·5)	24·6 (19·8 to 30·0)	5·7 (5·7 to 5·7)
Lithuania	2011	942·0 (916·0 to 968·8)	659·6 (636·9 to 682·9)	269·2 (257·6 to 281·5)	13·0 (11·0 to 15·3)	0·2 (0·2 to 0·2)	1827·1 (1776·7 to 1879·1)	1279·4 (1235·3 to 1324·7)	522·1 (499·7 to 546·0)	25·2 (21·3 to 29·6)	0·3 (0·3 to 0·3)
Lithuania	2012	910·1 (885·8 to 934·5)	612·7 (591·2 to 635·8)	284·0 (272·5 to 297·1)	13·3 (11·7 to 15·1)	0·0 (0·0 to 0·0)	1765·3 (1718·2 to 1812·6)	1188·5 (1146·7 to 1233·3)	550·9 (528·5 to 576·2)	25·8 (22·8 to 29·4)	0·0 (0·0 to 0·0)
Lithuania	2013	953·0 (927·5 to 979·1)	632·5 (610·0 to 654·4)	306·0 (294·6 to 318·6)	14·5 (13·2 to 16·0)	0·0 (0·0 to 0·0)	1848·5 (1799·0 to 1899·2)	1226·9 (1183·2 to 1269·3)	593·6 (571·4 to 617·9)	28·1 (25·5 to 31·1)	0·0 (0·0 to 0·0)
Lithuania	2014	1007·8 (981·5 to 1031·8)	667·0 (644·2 to 688·9)	324·4 (312·8 to 337·9)	16·3 (14·9 to 17·7)	0·0 (0·0 to 0·0)	1954·7 (1903·8 to 2001·3)	1293·7 (1249·5 to 1336·2)	629·3 (606·8 to 655·3)	31·6 (28·9 to 34·4)	0·0 (0·0 to 0·0)
Lithuania	2015	1082·9 (1055·7 to 1109·4)	716·0 (691·6 to 739·5)	348·0 (334·9 to 362·0)	19·0 (17·5 to 20·4)	0·0 (0·0 to 0·0)	2100·5 (2047·6 to 2151·8)	1388·7 (1341·5 to 1434·4)	674·9 (649·6 to 702·2)	36·9 (33·9 to 39·7)	0·0 (0·0 to 0·0)
Lithuania	2016	1134·1 (1106·2 to 1162·6)	746·0 (722·1 to 770·1)	367·3 (354·2 to 380·8)	20·9 (19·4 to 22·4)	0·0 (0·0 to 0·0)	2199·7 (2145·7 to 2255·0)	1446·9 (1400·5 to 1493·7)	712·3 (687·0 to 738·7)	40·5 (37·7 to 43·4)	0·0 (0·0 to 0·0)
Lithuania	2017	1185·5 (1157·8 to 1213·0)	777·5 (753·2 to 801·9)	383·9 (370·9 to 397·3)	24·1 (22·5 to 25·8)	0·0 (0·0 to 0·0)	2299·5 (2245·6 to 2352·9)	1508·0 (1461·0 to 1555·5)	744·7 (719·5 to 770·6)	46·8 (43·7 to 50·0)	0·0 (0·0 to 0·0)
Lithuania	2018	1254·1 (1216·6 to 1292·0)	827·5 (795·2 to 860·6)	399·7 (382·5 to 418·4)	27·0 (25·0 to 29·2)	0·0 (0·0 to 0·0)	2432·6 (2359·8 to 2506·0)	1605·0 (1542·3 to 1669·2)	775·2 (741·9 to 811·6)	52·4 (48·5 to 56·7)	0·0 (0·0 to 0·0)
Lithuania	2019	1320·7 (1277·3 to 1364·1)	877·0 (838·9 to 915·5)	414·9 (397·2 to 434·7)	28·8 (26·7 to 31·2)	0·0 (0·0 to 0·0)	2561·7 (2477·5 to 2646·0)	1701·1 (1627·3 to 1775·7)	804·8 (770·4 to 843·1)	55·8 (51·7 to 60·4)	0·0 (0·0 to 0·0)
Lithuania	2020	1535·0 (1491·6 to 1578·6)	1097·6 (1059·3 to 1136·2)	408·4 (391·0 to 428·0)	29·0 (26·8 to 31·4)	0·0 (0·0 to 0·0)	2977·3 (2893·1 to 3061·9)	2128·9 (2054·6 to 2203·9)	792·2 (758·4 to 830·2)	56·2 (52·0 to 61·0)	0·0 (0·0 to 0·0)
Lithuania	2021	1424·7 (1372·9 to 1476·3)	992·9 (943·6 to 1040·1)	402·5 (385·3 to 421·6)	29·2 (27·0 to 31·7)	0·0 (0·0 to 0·0)	2763·4 (2663·0 to 2863·6)	1926·0 (1830·2 to 2017·4)	780·8 (747·4 to 817·8)	56·7 (52·3 to 61·5)	0·0 (0·0 to 0·0)
Lithuania	2022	1299·4 (1246·5 to 1354·8)	866·5 (817·1 to 916·3)	403·0 (385·7 to 422·0)	29·9 (27·5 to 32·6)	0·0 (0·0 to 0·0)	2520·4 (2417·7 to 2627·7)	1680·6 (1584·8 to 1777·2)	781·7 (748·2 to 818·6)	58·1 (53·4 to 63·2)	0·0 (0·0 to 0·0)

Lithuania	2023	1302·8 (1244·6 to 1357·7)	869·8 (817·6 to 922·8)	402·4 (385·1 to 421·6)	30·6 (28·1 to 33·3)	0·0 (0·0 to 0·0)	2526·9 (2414·1 to 2633·5)	1687·1 (1585·8 to 1789·8)	780·5 (747·0 to 817·7)	59·3 (54·5 to 64·5)	0·0 (0·0 to 0·0)
Lithuania	2024	1303·9 (1244·6 to 1360·7)	871·5 (813·4 to 925·2)	401·3 (384·0 to 420·4)	31·1 (28·6 to 33·9)	0·0 (0·0 to 0·0)	2529·1 (2414·0 to 2639·4)	1690·5 (1577·7 to 1794·5)	778·3 (744·9 to 815·5)	60·4 (55·5 to 65·8)	0·0 (0·0 to 0·0)
Lithuania	2025	1306·0 (1242·8 to 1365·7)	873·9 (814·4 to 931·3)	400·4 (383·0 to 419·6)	31·7 (29·1 to 34·6)	0·0 (0·0 to 0·0)	2533·2 (2410·6 to 2649·0)	1695·1 (1579·6 to 1806·4)	776·6 (743·0 to 813·9)	61·5 (56·5 to 67·0)	0·0 (0·0 to 0·0)
Lithuania	2026	1307·6 (1241·0 to 1367·9)	876·1 (813·9 to 935·1)	399·2 (381·9 to 418·4)	32·3 (29·6 to 35·2)	0·0 (0·0 to 0·0)	2536·4 (2407·1 to 2653·3)	1699·3 (1578·7 to 1813·8)	774·4 (740·8 to 811·5)	62·6 (57·5 to 68·3)	0·0 (0·0 to 0·0)
Lithuania	2027	1311·2 (1241·9 to 1373·8)	879·6 (816·2 to 942·8)	398·7 (381·4 to 417·8)	32·9 (30·2 to 36·0)	0·0 (0·0 to 0·0)	2543·3 (2408·8 to 2664·8)	1706·1 (1583·2 to 1828·8)	773·3 (739·8 to 810·3)	63·9 (58·6 to 69·8)	0·0 (0·0 to 0·0)
Lithuania	2028	1317·4 (1245·3 to 1384·2)	884·9 (814·0 to 952·1)	398·9 (381·5 to 417·8)	33·7 (30·8 to 36·9)	0·0 (0·0 to 0·0)	2555·4 (2415·4 to 2684·9)	1716·3 (1578·8 to 1846·8)	773·7 (740·0 to 810·5)	65·3 (59·8 to 71·5)	0·0 (0·0 to 0·0)
Lithuania	2029	1324·3 (1253·7 to 1392·0)	890·6 (819·2 to 958·3)	399·3 (381·8 to 418·4)	34·5 (31·5 to 37·8)	0·0 (0·0 to 0·0)	2568·8 (2431·7 to 2700·1)	1727·5 (1589·0 to 1858·9)	774·5 (740·6 to 811·6)	66·8 (61·0 to 73·3)	0·0 (0·0 to 0·0)
Lithuania	2030	1332·9 (1260·5 to 1404·2)	897·6 (826·1 to 967·9)	400·0 (382·4 to 419·1)	35·3 (32·1 to 38·8)	0·0 (0·0 to 0·0)	2585·3 (2445·0 to 2723·6)	1741·1 (1602·4 to 1877·4)	775·8 (741·7 to 813·0)	68·4 (62·3 to 75·2)	0·0 (0·0 to 0·0)
Lithuania	2031	1343·0 (1266·1 to 1417·4)	905·8 (829·9 to 978·6)	401·1 (383·3 to 420·4)	36·2 (32·7 to 39·8)	0·0 (0·0 to 0·0)	2605·0 (2455·8 to 2749·2)	1756·9 (1609·7 to 1898·2)	778·0 (743·5 to 815·4)	70·1 (63·5 to 77·2)	0·0 (0·0 to 0·0)
Lithuania	2032	1354·3 (1274·4 to 1433·5)	915·1 (838·9 to 992·4)	402·1 (384·2 to 421·5)	37·0 (33·4 to 40·9)	0·0 (0·0 to 0·0)	2626·8 (2471·9 to 2780·5)	1775·0 (1627·1 to 1924·9)	779·9 (745·2 to 817·6)	71·9 (64·9 to 79·3)	0·0 (0·0 to 0·0)
Lithuania	2033	1365·8 (1285·6 to 1446·6)	924·6 (844·4 to 1005·6)	403·2 (385·1 to 422·6)	38·0 (34·1 to 42·0)	0·0 (0·0 to 0·0)	2649·1 (2493·7 to 2806·0)	1793·4 (1637·9 to 1950·4)	782·0 (747·0 to 819·7)	73·6 (66·1 to 81·4)	0·0 (0·0 to 0·0)
Lithuania	2034	1378·4 (1294·4 to 1461·3)	934·9 (852·5 to 1020·5)	404·6 (386·3 to 424·1)	38·9 (34·8 to 43·2)	0·0 (0·0 to 0·0)	2673·7 (2510·7 to 2834·5)	1813·3 (1653·5 to 1979·5)	784·8 (749·4 to 822·7)	75·5 (67·5 to 83·7)	0·0 (0·0 to 0·0)
Lithuania	2035	1392·6 (1306·1 to 1480·5)	946·2 (864·6 to 1031·0)	406·4 (387·9 to 426·1)	40·0 (35·6 to 44·5)	0·0 (0·0 to 0·0)	2701·3 (2533·3 to 2871·7)	1835·4 (1677·0 to 1999·7)	788·3 (752·5 to 826·4)	77·6 (69·0 to 86·3)	0·0 (0·0 to 0·0)
Lithuania	2036	1407·3 (1318·3 to 1498·4)	957·7 (872·0 to 1046·1)	408·5 (389·9 to 428·3)	41·1 (36·4 to 45·9)	0·0 (0·0 to 0·0)	2729·7 (2557·1 to 2906·3)	1857·7 (1691·4 to 2029·1)	792·4 (756·3 to 830·8)	79·7 (70·6 to 89·0)	0·0 (0·0 to 0·0)

Lithuania	2037	1421·8 (1334·2 to 1519·6)	969·0 (881·2 to 1066·8)	410·6 (392·0 to 430·5)	42·2 (37·1 to 47·2)	0·0 (0·0 to 0·0)	2757·9 (2587·9 to 2947·4)	1879·5 (1709·2 to 2069·3)	796·5 (760·3 to 835·1)	81·9 (72·0 to 91·6)	0·0 (0·0 to 0·0)
Lithuania	2038	1436·4 (1347·1 to 1533·3)	980·3 (892·5 to 1079·0)	412·7 (394·1 to 432·6)	43·4 (38·0 to 49·0)	0·0 (0·0 to 0·0)	2786·2 (2612·9 to 2974·1)	1901·4 (1731·1 to 2092·8)	800·6 (764·4 to 839·1)	84·2 (73·7 to 95·0)	0·0 (0·0 to 0·0)
Lithuania	2039	1453·5 (1363·5 to 1555·7)	993·1 (903·9 to 1093·8)	415·7 (396·8 to 435·7)	44·7 (38·8 to 50·9)	0·0 (0·0 to 0·0)	2819·3 (2644·8 to 3017·5)	1926·2 (1753·2 to 2121·6)	806·4 (769·7 to 845·1)	86·7 (75·3 to 98·8)	0·0 (0·0 to 0·0)
Lithuania	2040	1471·0 (1376·6 to 1572·6)	1006·2 (912·4 to 1112·1)	418·7 (399·4 to 438·7)	46·1 (39·7 to 52·9)	0·0 (0·0 to 0·0)	2853·2 (2670·1 to 3050·3)	1951·8 (1769·8 to 2157·0)	812·1 (774·7 to 850·9)	89·4 (77·0 to 102·6)	0·0 (0·0 to 0·0)
Lithuania	2041	1489·0 (1391·6 to 1598·0)	1019·7 (923·9 to 1128·1)	421·9 (402·4 to 441·9)	47·5 (40·7 to 55·0)	0·0 (0·0 to 0·0)	2888·2 (2699·2 to 3099·5)	1977·9 (1792·1 to 2188·1)	818·3 (780·5 to 857·1)	92·1 (78·9 to 106·7)	0·0 (0·0 to 0·0)
Lithuania	2042	1506·8 (1403·4 to 1617·4)	1032·6 (931·6 to 1139·8)	425·2 (405·5 to 445·3)	49·0 (41·8 to 57·2)	0·0 (0·0 to 0·0)	2922·6 (2722·2 to 3137·1)	2002·9 (1807·0 to 2210·8)	824·7 (786·5 to 863·7)	95·0 (81·1 to 110·9)	0·0 (0·0 to 0·0)
Lithuania	2043	1526·5 (1423·4 to 1635·2)	1047·0 (942·8 to 1153·9)	428·9 (409·1 to 449·4)	50·6 (42·9 to 59·5)	0·0 (0·0 to 0·0)	2960·9 (2760·9 to 3171·8)	2030·8 (1828·7 to 2238·2)	832·0 (793·5 to 871·6)	98·1 (83·3 to 115·5)	0·0 (0·0 to 0·0)
Lithuania	2044	1546·3 (1439·5 to 1662·2)	1061·1 (956·5 to 1173·0)	432·9 (412·8 to 453·4)	52·2 (44·1 to 62·0)	0·0 (0·0 to 0·0)	2999·3 (2792·2 to 3224·0)	2058·3 (1855·4 to 2275·2)	839·7 (800·7 to 879·5)	101·3 (85·5 to 120·2)	0·0 (0·0 to 0·0)
Lithuania	2045	1564·7 (1457·7 to 1684·5)	1074·2 (968·4 to 1190·6)	436·6 (416·1 to 457·4)	53·9 (45·2 to 64·3)	0·0 (0·0 to 0·0)	3035·0 (2827·5 to 3267·4)	2083·6 (1878·4 to 2309·3)	846·8 (807·2 to 887·2)	104·6 (87·8 to 124·8)	0·0 (0·0 to 0·0)
Lithuania	2046	1583·4 (1470·1 to 1705·0)	1087·3 (976·4 to 1205·5)	440·3 (419·6 to 461·3)	55·7 (46·3 to 67·1)	0·0 (0·0 to 0·0)	3071·2 (2851·5 to 3307·0)	2109·0 (1893·9 to 2338·2)	854·1 (813·9 to 894·7)	108·0 (89·9 to 130·1)	0·0 (0·0 to 0·0)
Lithuania	2047	1600·6 (1484·4 to 1728·2)	1099·7 (988·0 to 1222·4)	443·4 (422·5 to 464·2)	57·4 (47·4 to 69·9)	0·0 (0·0 to 0·0)	3104·6 (2879·3 to 3352·1)	2133·1 (1916·3 to 2370·9)	860·1 (819·5 to 900·5)	111·4 (92·0 to 135·6)	0·0 (0·0 to 0·0)
Lithuania	2048	1616·9 (1493·0 to 1750·9)	1111·5 (992·5 to 1238·4)	446·2 (425·1 to 467·5)	59·1 (48·6 to 72·6)	0·0 (0·0 to 0·0)	3136·2 (2895·8 to 3396·2)	2156·0 (1925·1 to 2402·0)	865·5 (824·5 to 906·8)	114·7 (94·2 to 140·7)	0·0 (0·0 to 0·0)
Lithuania	2049	1632·5 (1511·3 to 1770·9)	1123·0 (1000·8 to 1252·3)	448·6 (427·3 to 470·4)	60·9 (49·7 to 75·2)	0·0 (0·0 to 0·0)	3166·4 (2931·3 to 3434·9)	2178·2 (1941·3 to 2429·0)	870·2 (828·9 to 912·5)	118·0 (96·4 to 145·9)	0·0 (0·0 to 0·0)
Lithuania	2050	1648·9 (1519·1 to 1786·2)	1135·2 (1008·0 to 1266·5)	451·1 (429·5 to 473·4)	62·6 (50·8 to 77·7)	0·0 (0·0 to 0·0)	3198·2 (2946·5 to 3464·6)	2201·8 (1955·2 to 2456·6)	875·0 (833·1 to 918·2)	121·5 (98·6 to 150·8)	0·0 (0·0 to 0·0)

Luxembourg	1995	2893·3 (2641·9 to 3156·8)	2546·8 (2300·3 to 2813·1)	244·4 (202·4 to 292·2)	102·1 (74·4 to 138·8)	0·0 (0·0 to 0·0)	2981·1 (2722·1 to 3252·6)	2624·1 (2370·1 to 2898·4)	251·8 (208·6 to 301·0)	105·2 (76·7 to 143·0)	0·0 (0·0 to 0·0)
Luxembourg	1996	3004·0 (2768·2 to 3231·6)	2627·3 (2394·6 to 2866·2)	271·4 (228·4 to 319·7)	105·3 (77·6 to 140·1)	0·0 (0·0 to 0·0)	3095·2 (2852·2 to 3329·6)	2707·0 (2467·3 to 2953·1)	279·6 (235·3 to 329·4)	108·5 (79·9 to 144·3)	0·0 (0·0 to 0·0)
Luxembourg	1997	3307·1 (3082·6 to 3546·1)	2882·1 (2671·1 to 3119·2)	313·4 (266·6 to 364·7)	111·5 (84·8 to 144·6)	0·0 (0·0 to 0·0)	3407·4 (3176·2 to 3653·7)	2969·6 (2752·2 to 3213·8)	322·9 (274·7 to 375·8)	114·9 (87·4 to 148·9)	0·0 (0·0 to 0·0)
Luxembourg	1998	3726·6 (3506·1 to 3975·1)	3231·8 (3012·3 to 3468·6)	376·8 (325·2 to 429·4)	118·1 (92·5 to 146·5)	0·0 (0·0 to 0·0)	3839·7 (3612·5 to 4095·7)	3329·8 (3103·7 to 3573·9)	388·2 (335·1 to 442·4)	121·6 (95·3 to 150·9)	0·0 (0·0 to 0·0)
Luxembourg	1999	4420·8 (4190·3 to 4683·7)	3804·7 (3573·3 to 4060·2)	487·6 (431·2 to 546·7)	128·5 (105·2 to 156·2)	0·0 (0·0 to 0·0)	4554·9 (4317·4 to 4825·8)	3920·1 (3681·7 to 4183·4)	502·4 (444·3 to 563·3)	132·4 (108·4 to 161·0)	0·0 (0·0 to 0·0)
Luxembourg	2000	5395·1 (5137·6 to 5667·3)	4555·4 (4302·3 to 4822·4)	698·7 (631·9 to 766·3)	141·1 (117·7 to 166·0)	0·0 (0·0 to 0·0)	5558·8 (5293·5 to 5839·3)	4693·6 (4432·8 to 4968·7)	719·9 (651·1 to 789·5)	145·3 (121·3 to 171·0)	0·0 (0·0 to 0·0)
Luxembourg	2001	6181·4 (5930·1 to 6455·4)	5177·0 (4925·2 to 5436·2)	853·1 (785·8 to 920·8)	151·2 (129·7 to 175·5)	0·0 (0·0 to 0·0)	6368·9 (6110·1 to 6651·3)	5334·1 (5074·7 to 5601·1)	879·0 (809·7 to 948·7)	155·8 (133·6 to 180·8)	0·0 (0·0 to 0·0)
Luxembourg	2002	7002·9 (6718·6 to 7283·5)	5870·9 (5597·4 to 6140·3)	964·1 (891·5 to 1033·1)	167·9 (147·0 to 191·9)	0·0 (0·0 to 0·0)	7215·4 (6922·5 to 7504·5)	6049·0 (5767·3 to 6326·6)	993·3 (918·6 to 1064·5)	173·0 (151·4 to 197·7)	0·0 (0·0 to 0·0)
Luxembourg	2003	7434·6 (7154·5 to 7718·7)	6233·3 (5968·8 to 6520·3)	1004·8 (932·7 to 1086·9)	196·6 (173·7 to 220·1)	0·0 (0·0 to 0·0)	7660·2 (7371·6 to 7952·9)	6422·4 (6149·9 to 6718·2)	1035·3 (961·0 to 1119·8)	202·5 (179·0 to 226·7)	0·0 (0·0 to 0·0)
Luxembourg	2004	7714·0 (7442·2 to 8012·6)	6482·6 (6224·8 to 6766·8)	1014·4 (934·4 to 1088·8)	217·0 (194·0 to 241·9)	0·0 (0·0 to 0·0)	7948·1 (7668·1 to 8255·7)	6679·3 (6413·6 to 6972·1)	1045·2 (962·7 to 1121·8)	223·5 (199·9 to 249·2)	0·0 (0·0 to 0·0)
Luxembourg	2005	7794·3 (7508·6 to 8087·1)	6566·2 (6294·4 to 6851·4)	991·7 (913·8 to 1068·9)	236·3 (214·0 to 260·4)	0·0 (0·0 to 0·0)	8030·8 (7736·4 to 8332·5)	6765·5 (6485·4 to 7059·3)	1021·8 (941·5 to 1101·4)	243·5 (220·5 to 268·3)	0·0 (0·0 to 0·0)
Luxembourg	2006	7529·0 (7254·5 to 7814·4)	6357·3 (6085·9 to 6625·7)	925·6 (856·7 to 999·9)	246·1 (224·1 to 271·5)	0·0 (0·0 to 0·0)	7757·5 (7474·6 to 8051·5)	6550·2 (6270·6 to 6826·7)	953·7 (882·7 to 1030·3)	253·6 (230·9 to 279·7)	0·0 (0·0 to 0·0)
Luxembourg	2007	7588·2 (7297·8 to 7875·1)	6463·0 (6189·1 to 6742·5)	848·6 (783·6 to 916·3)	276·6 (253·5 to 303·9)	0·0 (0·0 to 0·0)	7818·4 (7519·3 to 8114·1)	6659·1 (6376·9 to 6947·1)	874·3 (807·4 to 944·1)	285·0 (261·2 to 313·1)	0·0 (0·0 to 0·0)
Luxembourg	2008	7716·6 (7415·7 to 8006·1)	6657·0 (6368·1 to 6940·3)	810·5 (744·9 to 875·4)	249·1 (227·8 to 273·5)	0·0 (0·0 to 0·0)	7950·7 (7640·7 to 8249·0)	6859·0 (6561·3 to 7150·9)	835·1 (767·5 to 902·0)	256·7 (234·7 to 281·8)	0·0 (0·0 to 0·0)

Luxembourg	2009	7864·3 (7591·3 to 8134·6)	6793·5 (6534·1 to 7064·2)	796·9 (730·1 to 868·5)	274·0 (250·2 to 300·6)	0·0 (0·0 to 0·0)	8103·0 (7821·6 to 8381·4)	6999·6 (6732·4 to 7278·5)	821·0 (752·2 to 894·8)	282·3 (257·8 to 309·7)	0·0 (0·0 to 0·0)
Luxembourg	2010	7565·7 (7288·5 to 7864·8)	6512·5 (6247·9 to 6816·7)	779·0 (709·0 to 850·1)	274·2 (249·4 to 300·6)	0·0 (0·0 to 0·0)	7795·3 (7509·6 to 8103·5)	6710·1 (6437·4 to 7023·6)	802·6 (730·5 to 875·9)	282·6 (256·9 to 309·7)	0·0 (0·0 to 0·0)
Luxembourg	2011	6856·9 (6568·1 to 7142·6)	5883·8 (5614·5 to 6162·6)	741·8 (676·5 to 808·6)	231·4 (209·5 to 255·1)	0·0 (0·0 to 0·0)	7065·0 (6767·4 to 7359·3)	6062·3 (5784·9 to 6349·6)	764·3 (697·0 to 833·1)	238·4 (215·9 to 262·9)	0·0 (0·0 to 0·0)
Luxembourg	2012	6372·6 (6091·1 to 6661·0)	5447·0 (5179·1 to 5727·7)	711·1 (648·9 to 779·5)	214·4 (191·6 to 237·9)	0·0 (0·0 to 0·0)	6565·9 (6275·9 to 6863·1)	5612·3 (5336·3 to 5901·5)	732·7 (668·6 to 803·2)	220·9 (197·4 to 245·2)	0·0 (0·0 to 0·0)
Luxembourg	2013	6080·4 (5814·9 to 6369·2)	5185·9 (4925·8 to 5465·4)	687·8 (626·8 to 759·7)	206·8 (183·8 to 228·9)	0·0 (0·0 to 0·0)	6264·9 (5991·3 to 6562·5)	5343·2 (5075·3 to 5631·2)	708·6 (645·8 to 782·7)	213·0 (189·4 to 235·9)	0·0 (0·0 to 0·0)
Luxembourg	2014	5975·0 (5696·3 to 6259·1)	5099·8 (4827·0 to 5376·3)	673·7 (616·5 to 741·1)	201·5 (179·8 to 223·6)	0·0 (0·0 to 0·0)	6156·3 (5869·1 to 6449·1)	5254·6 (4973·5 to 5539·4)	694·1 (635·2 to 763·6)	207·6 (185·2 to 230·4)	0·0 (0·0 to 0·0)
Luxembourg	2015	5987·1 (5708·5 to 6258·3)	5120·8 (4847·4 to 5385·9)	663·1 (601·3 to 728·2)	203·2 (180·7 to 226·2)	0·0 (0·0 to 0·0)	6168·8 (5881·8 to 6448·2)	5276·2 (4994·4 to 5549·3)	683·2 (619·6 to 750·3)	209·4 (186·1 to 233·0)	0·0 (0·0 to 0·0)
Luxembourg	2016	5999·2 (5729·7 to 6285·2)	5136·8 (4880·1 to 5409·8)	658·0 (597·3 to 728·8)	204·4 (180·8 to 229·9)	0·0 (0·0 to 0·0)	6181·3 (5903·6 to 6475·9)	5292·7 (5028·2 to 5573·9)	678·0 (615·4 to 750·9)	210·6 (186·2 to 236·8)	0·0 (0·0 to 0·0)
Luxembourg	2017	6069·2 (5779·7 to 6359·7)	5213·3 (4945·4 to 5496·3)	656·9 (589·9 to 731·3)	199·0 (174·7 to 227·5)	0·0 (0·0 to 0·0)	6253·3 (5955·1 to 6552·6)	5371·5 (5095·5 to 5663·1)	676·8 (607·8 to 753·5)	205·1 (179·9 to 234·4)	0·0 (0·0 to 0·0)
Luxembourg	2018	6159·3 (5810·5 to 6547·1)	5300·5 (4961·0 to 5679·1)	659·1 (583·2 to 741·8)	199·7 (170·4 to 233·6)	0·0 (0·0 to 0·0)	6346·2 (5986·8 to 6745·8)	5461·3 (5111·5 to 5851·5)	679·1 (600·9 to 764·4)	205·8 (175·5 to 240·6)	0·0 (0·0 to 0·0)
Luxembourg	2019	6246·4 (5877·0 to 6654·6)	5381·4 (5017·9 to 5775·3)	660·4 (584·2 to 743·2)	204·6 (174·7 to 239·0)	0·0 (0·0 to 0·0)	6435·9 (6055·3 to 6856·5)	5544·7 (5170·1 to 5950·5)	680·4 (601·9 to 765·8)	210·9 (180·0 to 246·2)	0·0 (0·0 to 0·0)
Luxembourg	2020	6952·0 (6511·3 to 7435·5)	6128·4 (5701·0 to 6590·7)	625·4 (552·9 to 704·2)	198·3 (169·6 to 231·7)	0·0 (0·0 to 0·0)	7163·0 (6708·9 to 7661·2)	6314·3 (5874·0 to 6790·7)	644·4 (569·6 to 725·5)	204·3 (174·8 to 238·7)	0·0 (0·0 to 0·0)
Luxembourg	2021	6782·3 (6352·6 to 7253·4)	5976·0 (5559·2 to 6426·9)	608·8 (538·1 to 686·3)	197·5 (168·7 to 231·2)	0·0 (0·0 to 0·0)	6988·0 (6545·3 to 7473·5)	6157·3 (5727·9 to 6621·9)	627·3 (554·4 to 707·2)	203·5 (173·9 to 238·2)	0·0 (0·0 to 0·0)
Luxembourg	2022	5834·2 (5428·6 to 6289·3)	5020·2 (4613·4 to 5472·6)	611·2 (540·3 to 689·9)	202·8 (173·2 to 238·2)	0·0 (0·0 to 0·0)	6011·2 (5593·3 to 6480·1)	5172·5 (4753·4 to 5638·7)	629·7 (556·7 to 710·9)	209·0 (178·5 to 245·4)	0·0 (0·0 to 0·0)

Luxembourg	2023	5864·5 (5443·6 to 6327·9)	5043·2 (4627·2 to 5496·2)	613·1 (541·9 to 692·0)	208·1 (177·3 to 243·9)	0·0 (0·0 to 0·0)	6042·4 (5608·7 to 6520·0)	5196·3 (4767·6 to 5663·0)	631·8 (558·3 to 713·0)	214·4 (182·7 to 251·3)	0·0 (0·0 to 0·0)
Luxembourg	2024	5910·5 (5462·7 to 6395·2)	5082·4 (4638·0 to 5541·9)	614·7 (543·3 to 693·3)	213·4 (180·8 to 250·7)	0·0 (0·0 to 0·0)	6089·8 (5628·4 to 6589·2)	5236·6 (4778·7 to 5710·0)	633·3 (559·8 to 714·4)	219·8 (186·3 to 258·3)	0·0 (0·0 to 0·0)
Luxembourg	2025	5992·6 (5529·3 to 6499·2)	5157·3 (4704·2 to 5659·1)	616·5 (544·8 to 695·3)	218·8 (184·8 to 257·9)	0·0 (0·0 to 0·0)	6174·4 (5697·1 to 6696·4)	5313·8 (4847·0 to 5830·8)	635·2 (561·3 to 716·4)	225·4 (190·4 to 265·7)	0·0 (0·0 to 0·0)
Luxembourg	2026	6070·4 (5587·8 to 6609·5)	5229·3 (4734·2 to 5744·5)	617·2 (545·4 to 695·8)	223·9 (188·7 to 264·2)	0·0 (0·0 to 0·0)	6254·6 (5757·4 to 6810·1)	5388·0 (4877·8 to 5918·9)	635·9 (561·9 to 716·9)	230·7 (194·5 to 272·3)	0·0 (0·0 to 0·0)
Luxembourg	2027	6107·8 (5601·2 to 6668·6)	5266·1 (4766·8 to 5801·2)	614·1 (542·5 to 692·7)	227·7 (191·7 to 268·3)	0·0 (0·0 to 0·0)	6293·2 (5771·1 to 6870·9)	5425·8 (4911·5 to 5977·2)	632·7 (559·0 to 713·7)	234·6 (197·5 to 276·5)	0·0 (0·0 to 0·0)
Luxembourg	2028	6137·4 (5608·0 to 6702·8)	5296·0 (4773·6 to 5851·4)	609·4 (538·1 to 688·1)	231·9 (195·3 to 274·1)	0·0 (0·0 to 0·0)	6323·6 (5778·1 to 6906·2)	5456·7 (4918·4 to 6028·9)	627·9 (554·5 to 709·0)	239·0 (201·2 to 282·4)	0·0 (0·0 to 0·0)
Luxembourg	2029	6176·1 (5650·1 to 6770·0)	5334·6 (4808·2 to 5912·1)	605·1 (534·3 to 682·8)	236·4 (199·0 to 279·9)	0·0 (0·0 to 0·0)	6363·5 (5821·6 to 6975·4)	5496·5 (4954·1 to 6091·5)	623·5 (550·5 to 703·5)	243·5 (205·1 to 288·4)	0·0 (0·0 to 0·0)
Luxembourg	2030	6217·1 (5663·9 to 6834·9)	5374·9 (4818·1 to 5975·6)	601·1 (530·4 to 677·8)	241·0 (203·2 to 286·4)	0·0 (0·0 to 0·0)	6405·7 (5835·8 to 7042·3)	5538·0 (4964·3 to 6156·9)	619·4 (546·5 to 698·3)	248·3 (209·4 to 295·1)	0·0 (0·0 to 0·0)
Luxembourg	2031	6248·0 (5666·2 to 6887·3)	5406·0 (4822·6 to 6035·4)	596·5 (526·4 to 672·1)	245·4 (206·8 to 292·1)	0·0 (0·0 to 0·0)	6437·5 (5838·2 to 7096·3)	5570·0 (4969·0 to 6218·5)	614·6 (542·4 to 692·5)	252·9 (213·1 to 301·0)	0·0 (0·0 to 0·0)
Luxembourg	2032	6289·8 (5692·2 to 6956·0)	5446·2 (4855·9 to 6113·7)	593·2 (523·6 to 667·9)	250·4 (210·8 to 297·9)	0·0 (0·0 to 0·0)	6480·7 (5864·9 to 7167·1)	5611·5 (5003·3 to 6299·2)	611·2 (539·4 to 688·1)	258·0 (217·2 to 306·9)	0·0 (0·0 to 0·0)
Luxembourg	2033	6332·9 (5716·8 to 6997·3)	5487·0 (4885·6 to 6152·4)	590·3 (521·1 to 664·9)	255·5 (213·5 to 303·7)	0·0 (0·0 to 0·0)	6525·0 (5890·2 to 7209·7)	5653·5 (5033·8 to 6339·1)	608·2 (536·9 to 685·1)	263·3 (220·0 to 312·9)	0·0 (0·0 to 0·0)
Luxembourg	2034	6375·9 (5748·2 to 7034·6)	5527·6 (4916·7 to 6188·9)	587·5 (518·6 to 662·0)	260·8 (216·9 to 310·6)	0·0 (0·0 to 0·0)	6569·3 (5922·6 to 7248·1)	5695·3 (5065·9 to 6376·7)	605·3 (534·4 to 682·0)	268·7 (223·5 to 320·1)	0·0 (0·0 to 0·0)
Luxembourg	2035	6422·3 (5783·8 to 7116·3)	5571·2 (4929·8 to 6238·2)	584·9 (516·4 to 658·9)	266·2 (220·5 to 318·0)	0·0 (0·0 to 0·0)	6617·2 (5959·3 to 7332·2)	5740·3 (5079·4 to 6427·5)	602·6 (532·0 to 678·9)	274·3 (227·2 to 327·7)	0·0 (0·0 to 0·0)
Luxembourg	2036	6458·6 (5805·8 to 7166·1)	5605·1 (4961·4 to 6301·0)	582·1 (513·9 to 656·0)	271·5 (223·8 to 325·9)	0·0 (0·0 to 0·0)	6654·6 (5981·9 to 7383·5)	5775·2 (5112·0 to 6492·2)	599·7 (529·5 to 675·9)	279·7 (230·6 to 335·8)	0·0 (0·0 to 0·0)

Luxembourg	2037	6497·9 (5829·5 to 7209·1)	5641·2 (4962·5 to 6329·1)	579·8 (512·0 to 653·7)	277·0 (227·2 to 335·1)	0·0 (0·0 to 0·0)	6695·1 (6006·4 to 7427·9)	5812·3 (5113·1 to 6521·1)	597·4 (527·5 to 673·5)	285·4 (234·1 to 345·2)	0·0 (0·0 to 0·0)
Luxembourg	2038	6539·7 (5863·5 to 7274·5)	5678·8 (4990·5 to 6390·5)	578·1 (510·4 to 652·0)	282·8 (231·0 to 341·9)	0·0 (0·0 to 0·0)	6738·1 (6041·4 to 7495·2)	5851·1 (5141·9 to 6584·4)	595·6 (525·8 to 671·8)	291·4 (238·0 to 352·3)	0·0 (0·0 to 0·0)
Luxembourg	2039	6572·7 (5877·9 to 7352·4)	5707·9 (5017·6 to 6478·3)	576·3 (508·5 to 650·2)	288·6 (234·8 to 349·6)	0·0 (0·0 to 0·0)	6772·1 (6056·3 to 7575·5)	5881·0 (5169·8 to 6674·9)	593·8 (523·9 to 669·9)	297·3 (241·9 to 360·3)	0·0 (0·0 to 0·0)
Luxembourg	2040	6608·3 (5899·2 to 7401·5)	5738·9 (5040·3 to 6522·9)	574·9 (507·3 to 648·8)	294·5 (237·3 to 359·1)	0·0 (0·0 to 0·0)	6808·8 (6078·2 to 7626·1)	5913·0 (5193·2 to 6720·8)	592·3 (522·6 to 668·5)	303·5 (244·5 to 370·0)	0·0 (0·0 to 0·0)
Luxembourg	2041	6643·4 (5913·8 to 7443·3)	5769·2 (5047·3 to 6571·7)	573·6 (506·1 to 647·2)	300·6 (241·0 to 368·7)	0·0 (0·0 to 0·0)	6845·0 (6093·2 to 7669·2)	5944·2 (5200·4 to 6771·1)	591·0 (521·5 to 666·8)	309·8 (248·3 to 379·9)	0·0 (0·0 to 0·0)
Luxembourg	2042	6675·2 (5922·4 to 7487·0)	5796·3 (5056·2 to 6621·7)	572·1 (504·8 to 645·3)	306·8 (245·0 to 378·5)	0·0 (0·0 to 0·0)	6877·7 (6102·1 to 7714·1)	5972·2 (5209·6 to 6822·6)	589·5 (520·1 to 664·9)	316·1 (252·4 to 390·0)	0·0 (0·0 to 0·0)
Luxembourg	2043	6701·0 (5938·8 to 7541·6)	5817·5 (5067·0 to 6659·8)	570·4 (502·9 to 643·3)	313·0 (249·0 to 386·5)	0·0 (0·0 to 0·0)	6904·3 (6119·0 to 7770·5)	5994·1 (5220·7 to 6861·8)	587·8 (518·2 to 662·8)	322·5 (256·5 to 398·2)	0·0 (0·0 to 0·0)
Luxembourg	2044	6735·0 (5973·6 to 7592·3)	5846·8 (5103·4 to 6716·3)	568·9 (501·2 to 641·4)	319·3 (252·9 to 397·3)	0·0 (0·0 to 0·0)	6939·3 (6154·8 to 7822·6)	6024·2 (5258·3 to 6920·1)	586·1 (516·4 to 660·8)	329·0 (260·5 to 409·3)	0·0 (0·0 to 0·0)
Luxembourg	2045	6761·8 (5986·4 to 7634·3)	5868·8 (5106·5 to 6745·1)	567·2 (499·3 to 639·3)	325·8 (256·2 to 407·8)	0·0 (0·0 to 0·0)	6966·9 (6168·1 to 7866·0)	6046·9 (5261·5 to 6949·7)	584·4 (514·5 to 658·7)	335·7 (263·9 to 420·2)	0·0 (0·0 to 0·0)
Luxembourg	2046	6789·7 (5979·9 to 7689·4)	5891·9 (5074·9 to 6774·8)	565·5 (497·4 to 637·2)	332·2 (260·2 to 417·6)	0·0 (0·0 to 0·0)	6995·7 (6161·3 to 7922·7)	6070·7 (5228·9 to 6980·4)	582·7 (512·5 to 656·6)	342·3 (268·1 to 430·2)	0·0 (0·0 to 0·0)
Luxembourg	2047	6816·0 (5988·5 to 7704·1)	5913·1 (5103·5 to 6811·3)	564·0 (495·9 to 635·7)	338·9 (263·8 to 428·5)	0·0 (0·0 to 0·0)	7022·8 (6170·2 to 7937·9)	6092·6 (5258·3 to 7018·0)	581·1 (510·9 to 654·9)	349·1 (271·8 to 441·5)	0·0 (0·0 to 0·0)
Luxembourg	2048	6846·7 (6031·5 to 7753·0)	5938·2 (5101·4 to 6847·0)	562·7 (494·6 to 634·9)	345·7 (266·7 to 438·0)	0·0 (0·0 to 0·0)	7054·4 (6214·5 to 7988·3)	6118·4 (5256·2 to 7054·7)	579·8 (509·6 to 654·1)	356·2 (274·8 to 451·3)	0·0 (0·0 to 0·0)
Luxembourg	2049	6869·2 (6018·1 to 7824·8)	5955·9 (5101·1 to 6898·6)	561·0 (492·9 to 633·7)	352·4 (270·1 to 450·0)	0·0 (0·0 to 0·0)	7077·7 (6200·7 to 8062·2)	6136·6 (5255·9 to 7107·9)	578·0 (507·9 to 652·9)	363·1 (278·3 to 463·6)	0·0 (0·0 to 0·0)
Luxembourg	2050	6892·6 (6019·5 to 7848·6)	5973·9 (5096·3 to 6917·6)	559·4 (491·4 to 632·3)	359·3 (274·4 to 460·6)	0·0 (0·0 to 0·0)	7101·8 (6202·1 to 8086·8)	6155·2 (5251·0 to 7127·5)	576·4 (506·3 to 651·4)	370·2 (282·7 to 474·6)	0·0 (0·0 to 0·0)

Latvia	1995	349·7 (320·7 to 380·4)	192·2 (171·8 to 213·6)	154·1 (133·6 to 177·0)	3·1 (2·1 to 4·3)	0·3 (0·3 to 0·3)	620·6 (569·0 to 675·0)	341·0 (304·8 to 379·1)	273·5 (237·1 to 314·1)	5·5 (3·7 to 7·6)	0·6 (0·6 to 0·6)
Latvia	1996	358·7 (332·0 to 385·3)	191·3 (173·6 to 209·8)	164·3 (146·6 to 186·1)	3·1 (2·1 to 4·2)	0·0 (0·0 to 0·0)	636·5 (589·2 to 683·6)	339·4 (308·0 to 372·2)	291·6 (260·1 to 330·2)	5·4 (3·7 to 7·5)	0·0 (0·0 to 0·0)
Latvia	1997	382·1 (356·6 to 406·9)	201·5 (184·7 to 218·7)	176·8 (159·3 to 195·9)	3·7 (2·5 to 5·2)	0·0 (0·0 to 0·0)	678·0 (632·8 to 722·0)	357·6 (327·7 to 388·0)	313·8 (282·6 to 347·7)	6·6 (4·4 to 9·3)	0·0 (0·0 to 0·0)
Latvia	1998	408·3 (381·4 to 434·4)	216·1 (199·1 to 233·2)	187·4 (169·9 to 206·9)	4·6 (3·1 to 6·5)	0·2 (0·2 to 0·2)	724·6 (676·7 to 770·8)	383·4 (353·3 to 413·7)	332·5 (301·4 to 367·1)	8·2 (5·6 to 11·5)	0·4 (0·4 to 0·4)
Latvia	1999	431·7 (406·1 to 457·5)	226·6 (209·1 to 244·6)	199·1 (180·7 to 218·8)	5·6 (3·8 to 7·8)	0·4 (0·4 to 0·4)	766·0 (720·6 to 811·8)	402·1 (371·1 to 434·0)	353·3 (320·7 to 388·2)	9·9 (6·7 to 13·9)	0·7 (0·7 to 0·7)
Latvia	2000	439·7 (415·2 to 466·9)	224·2 (208·2 to 241·4)	207·6 (188·7 to 227·8)	7·0 (4·8 to 9·7)	0·8 (0·8 to 0·8)	780·1 (736·8 to 828·4)	397·9 (369·4 to 428·4)	368·4 (334·8 to 404·2)	12·4 (8·6 to 17·2)	1·5 (1·5 to 1·5)
Latvia	2001	467·7 (442·7 to 495·2)	233·8 (218·3 to 251·2)	223·5 (204·9 to 242·8)	9·3 (6·6 to 12·8)	1·1 (1·1 to 1·1)	830·0 (785·5 to 878·7)	414·8 (387·4 to 445·8)	396·6 (363·5 to 430·8)	16·6 (11·7 to 22·6)	2·0 (2·0 to 2·0)
Latvia	2002	515·9 (488·8 to 542·3)	261·0 (244·2 to 278·6)	241·5 (222·6 to 260·6)	11·3 (8·0 to 15·4)	2·1 (2·1 to 2·1)	915·5 (867·3 to 962·3)	463·2 (433·3 to 494·4)	428·5 (395·0 to 462·4)	20·0 (14·2 to 27·2)	3·7 (3·7 to 3·7)
Latvia	2003	555·6 (529·5 to 581·3)	290·0 (273·9 to 309·6)	254·3 (234·1 to 273·5)	10·9 (7·7 to 14·6)	0·4 (0·4 to 0·4)	985·8 (939·5 to 1031·5)	514·5 (486·0 to 549·4)	451·2 (415·3 to 485·4)	19·4 (13·7 to 26·0)	0·7 (0·7 to 0·7)
Latvia	2004	633·8 (604·2 to 662·5)	348·3 (331·1 to 367·9)	270·2 (249·2 to 292·6)	14·4 (10·4 to 19·1)	0·9 (0·9 to 0·9)	1124·6 (1072·1 to 1175·5)	618·0 (587·6 to 652·8)	479·4 (442·1 to 519·2)	25·6 (18·5 to 33·9)	1·6 (1·6 to 1·6)
Latvia	2005	702·3 (671·8 to 732·9)	403·3 (383·9 to 423·8)	283·2 (261·7 to 305·0)	15·8 (11·7 to 20·8)	0·0 (0·0 to 0·0)	1246·1 (1192·0 to 1300·4)	715·5 (681·2 to 752·0)	502·5 (464·4 to 541·1)	28·0 (20·7 to 36·9)	0·0 (0·0 to 0·0)
Latvia	2006	775·3 (744·5 to 807·6)	463·3 (443·4 to 483·7)	293·8 (271·7 to 316·2)	18·1 (13·7 to 23·5)	0·0 (0·0 to 0·0)	1375·7 (1321·0 to 1432·9)	822·2 (786·7 to 858·3)	521·4 (482·2 to 561·0)	32·1 (24·4 to 41·8)	0·0 (0·0 to 0·0)
Latvia	2007	811·8 (781·8 to 843·7)	490·2 (470·3 to 512·2)	304·3 (282·2 to 325·5)	17·3 (13·3 to 21·9)	0·0 (0·0 to 0·0)	1440·5 (1387·3 to 1497·0)	869·9 (834·5 to 908·8)	540·0 (500·8 to 577·6)	30·7 (23·5 to 38·8)	0·0 (0·0 to 0·0)
Latvia	2008	823·0 (795·5 to 853·3)	499·5 (480·5 to 521·2)	307·0 (286·1 to 327·1)	15·6 (12·2 to 19·7)	0·8 (0·8 to 0·8)	1460·3 (1411·6 to 1514·1)	886·4 (852·6 to 924·8)	544·8 (507·6 to 580·4)	27·8 (21·6 to 34·9)	1·4 (1·4 to 1·4)

Latvia	2009	785·6 (758·8 to 814·7)	477·9 (458·3 to 497·2)	295·1 (276·7 to 314·0)	12·5 (9·9 to 15·5)	0·0 (0·0 to 0·0)	1394·0 (1346·4 to 1445·6)	848·0 (813·2 to 882·3)	523·7 (491·0 to 557·2)	22·2 (17·5 to 27·5)	0·0 (0·0 to 0·0)
Latvia	2010	785·5 (758·8 to 813·3)	465·0 (446·4 to 482·7)	283·2 (264·3 to 301·7)	14·8 (11·9 to 17·9)	22·5 (22·5 to 22·5)	1393·9 (1346·5 to 1443·1)	825·1 (792·2 to 856·5)	502·5 (469·0 to 535·4)	26·3 (21·1 to 31·8)	40·0 (40·0 to 40·0)
Latvia	2011	773·4 (747·4 to 799·0)	458·3 (440·5 to 475·1)	276·4 (258·6 to 294·1)	14·4 (11·8 to 17·4)	24·2 (24·2 to 24·2)	1372·3 (1326·1 to 1417·7)	813·3 (781·7 to 842·9)	490·5 (458·9 to 521·8)	25·6 (21·0 to 30·8)	42·9 (42·9 to 42·9)
Latvia	2012	756·1 (732·2 to 779·1)	457·8 (443·0 to 473·4)	285·3 (267·1 to 302·6)	13·0 (11·0 to 15·5)	0·0 (0·0 to 0·0)	1341·7 (1299·3 to 1382·5)	812·3 (786·1 to 840·1)	506·3 (474·0 to 537·0)	23·1 (19·5 to 27·5)	0·0 (0·0 to 0·0)
Latvia	2013	787·0 (763·9 to 810·0)	472·0 (456·8 to 487·9)	303·8 (286·2 to 320·0)	11·2 (9·6 to 13·1)	0·0 (0·0 to 0·0)	1396·5 (1355·5 to 1437·2)	837·5 (810·6 to 865·8)	539·0 (507·8 to 567·8)	19·9 (16·9 to 23·2)	0·0 (0·0 to 0·0)
Latvia	2014	834·7 (810·7 to 858·0)	493·6 (477·9 to 509·9)	331·7 (313·8 to 349·1)	9·4 (8·0 to 11·1)	0·0 (0·0 to 0·0)	1481·0 (1438·5 to 1522·4)	875·8 (848·1 to 904·8)	588·5 (556·8 to 619·5)	16·7 (14·2 to 19·6)	0·0 (0·0 to 0·0)
Latvia	2015	906·2 (882·3 to 932·7)	526·9 (510·8 to 543·8)	371·4 (352·5 to 390·7)	7·9 (6·6 to 9·4)	0·0 (0·0 to 0·0)	1608·0 (1565·6 to 1655·1)	935·0 (906·3 to 964·9)	659·0 (625·5 to 693·3)	14·0 (11·7 to 16·7)	0·0 (0·0 to 0·0)
Latvia	2016	963·7 (938·4 to 988·7)	548·9 (533·1 to 566·1)	407·7 (388·0 to 426·2)	7·1 (5·9 to 8·7)	0·0 (0·0 to 0·0)	1710·0 (1665·2 to 1754·4)	973·9 (946·0 to 1004·6)	723·4 (688·5 to 756·3)	12·7 (10·4 to 15·4)	0·0 (0·0 to 0·0)
Latvia	2017	1029·8 (1004·9 to 1056·2)	593·3 (577·4 to 610·8)	429·2 (410·0 to 448·2)	7·3 (5·9 to 9·0)	0·0 (0·0 to 0·0)	1827·4 (1783·0 to 1874·1)	1052·7 (1024·5 to 1083·8)	761·6 (727·5 to 795·3)	13·0 (10·4 to 16·0)	0·0 (0·0 to 0·0)
Latvia	2018	1102·7 (1068·8 to 1137·4)	651·5 (629·7 to 675·1)	443·1 (416·8 to 469·3)	8·2 (6·4 to 10·2)	0·0 (0·0 to 0·0)	1956·7 (1896·5 to 2018·2)	1156·0 (1117·3 to 1198·0)	786·2 (739·6 to 832·8)	14·5 (11·4 to 18·2)	0·0 (0·0 to 0·0)
Latvia	2019	1131·6 (1093·9 to 1169·5)	670·5 (643·9 to 698·4)	452·6 (425·5 to 479·3)	8·6 (6·8 to 10·7)	0·0 (0·0 to 0·0)	2008·0 (1941·1 to 2075·2)	1189·7 (1142·5 to 1239·3)	803·0 (755·0 to 850·5)	15·2 (12·0 to 19·0)	0·0 (0·0 to 0·0)
Latvia	2020	1232·0 (1194·7 to 1270·1)	782·8 (756·0 to 811·1)	440·7 (414·3 to 466·7)	8·6 (6·7 to 10·8)	0·0 (0·0 to 0·0)	2186·2 (2119·8 to 2253·7)	1389·0 (1341·4 to 1439·2)	781·9 (735·1 to 828·1)	15·2 (11·9 to 19·1)	0·0 (0·0 to 0·0)
Latvia	2021	1197·7 (1153·6 to 1242·7)	755·3 (721·7 to 791·6)	433·8 (407·9 to 459·2)	8·6 (6·8 to 10·9)	0·0 (0·0 to 0·0)	2125·3 (2046·9 to 2205·1)	1340·2 (1280·7 to 1404·6)	769·8 (723·7 to 814·9)	15·3 (12·0 to 19·3)	0·0 (0·0 to 0·0)
Latvia	2022	1107·1 (1061·9 to 1154·8)	664·8 (630·4 to 704·6)	433·4 (407·2 to 458·7)	8·8 (7·0 to 11·2)	0·0 (0·0 to 0·0)	1964·4 (1884·3 to 2049·1)	1179·7 (1118·5 to 1250·2)	769·0 (722·6 to 813·9)	15·7 (12·3 to 19·8)	0·0 (0·0 to 0·0)

Latvia	2023	1110·7 (1063·1 to 1162·2)	668·8 (629·6 to 714·3)	432·8 (406·8 to 457·9)	9·0 (7·1 to 11·3)	0·0 (0·0 to 0·0)	1970·8 (1886·4 to 2062·3)	1186·7 (1117·2 to 1267·5)	768·0 (721·8 to 812·5)	16·0 (12·7 to 20·1)	0·0 (0·0 to 0·0)
Latvia	2024	1115·1 (1064·3 to 1168·3)	673·6 (629·7 to 720·7)	432·3 (406·3 to 457·5)	9·2 (7·3 to 11·6)	0·0 (0·0 to 0·0)	1978·7 (1888·5 to 2073·1)	1195·3 (1117·3 to 1278·8)	767·0 (720·9 to 811·9)	16·4 (12·9 to 20·6)	0·0 (0·0 to 0·0)
Latvia	2025	1119·6 (1066·2 to 1174·6)	678·3 (631·5 to 729·3)	431·8 (406·0 to 456·9)	9·5 (7·4 to 11·9)	0·0 (0·0 to 0·0)	1986·6 (1891·9 to 2084·2)	1203·6 (1120·6 to 1294·1)	766·2 (720·3 to 810·7)	16·8 (13·2 to 21·1)	0·0 (0·0 to 0·0)
Latvia	2026	1123·9 (1069·1 to 1181·3)	682·9 (635·0 to 735·3)	431·3 (405·7 to 456·3)	9·7 (7·6 to 12·1)	0·0 (0·0 to 0·0)	1994·2 (1897·0 to 2096·2)	1211·7 (1126·8 to 1304·7)	765·4 (719·9 to 809·6)	17·1 (13·5 to 21·5)	0·0 (0·0 to 0·0)
Latvia	2027	1129·9 (1071·7 to 1191·3)	688·5 (637·0 to 743·2)	431·5 (405·9 to 456·5)	9·9 (7·8 to 12·4)	0·0 (0·0 to 0·0)	2004·9 (1901·7 to 2113·8)	1221·7 (1130·4 to 1318·7)	765·6 (720·3 to 810·0)	17·6 (13·9 to 22·0)	0·0 (0·0 to 0·0)
Latvia	2028	1136·8 (1078·6 to 1197·9)	694·6 (642·9 to 749·2)	432·1 (406·5 to 457·0)	10·1 (8·0 to 12·7)	0·0 (0·0 to 0·0)	2017·1 (1913·8 to 2125·5)	1232·4 (1140·7 to 1329·4)	766·7 (721·3 to 810·9)	18·0 (14·2 to 22·5)	0·0 (0·0 to 0·0)
Latvia	2029	1145·3 (1084·0 to 1209·2)	701·6 (646·6 to 757·7)	433·3 (407·7 to 458·4)	10·4 (8·2 to 13·0)	0·0 (0·0 to 0·0)	2032·3 (1923·4 to 2145·6)	1244·9 (1147·4 to 1344·5)	768·9 (723·4 to 813·4)	18·5 (14·5 to 23·1)	0·0 (0·0 to 0·0)
Latvia	2030	1155·0 (1090·8 to 1219·3)	709·5 (650·7 to 767·6)	434·9 (409·2 to 459·8)	10·7 (8·4 to 13·4)	0·0 (0·0 to 0·0)	2049·5 (1935·5 to 2163·5)	1258·9 (1154·6 to 1362·0)	771·6 (726·1 to 816·0)	19·0 (14·9 to 23·8)	0·0 (0·0 to 0·0)
Latvia	2031	1168·1 (1102·8 to 1232·0)	720·2 (662·0 to 777·8)	436·9 (411·1 to 462·0)	11·0 (8·6 to 13·8)	0·0 (0·0 to 0·0)	2072·6 (1956·9 to 2186·1)	1277·9 (1174·6 to 1380·2)	775·2 (729·5 to 819·9)	19·5 (15·3 to 24·4)	0·0 (0·0 to 0·0)
Latvia	2032	1181·3 (1114·2 to 1247·7)	731·3 (667·3 to 790·4)	438·7 (412·9 to 464·1)	11·3 (8·9 to 14·1)	0·0 (0·0 to 0·0)	2096·2 (1977·0 to 2214·0)	1297·6 (1184·1 to 1402·5)	778·4 (732·7 to 823·5)	20·1 (15·8 to 25·0)	0·0 (0·0 to 0·0)
Latvia	2033	1195·1 (1124·5 to 1265·1)	742·7 (674·6 to 806·6)	440·8 (415·0 to 466·3)	11·6 (9·1 to 14·5)	0·0 (0·0 to 0·0)	2120·6 (1995·3 to 2244·8)	1317·8 (1197·1 to 1431·2)	782·1 (736·3 to 827·4)	20·6 (16·2 to 25·7)	0·0 (0·0 to 0·0)
Latvia	2034	1210·3 (1136·1 to 1282·7)	755·1 (684·6 to 822·7)	443·2 (417·3 to 468·9)	12·0 (9·3 to 15·0)	0·0 (0·0 to 0·0)	2147·5 (2016·0 to 2276·0)	1339·8 (1214·7 to 1459·9)	786·5 (740·4 to 832·1)	21·2 (16·5 to 26·5)	0·0 (0·0 to 0·0)
Latvia	2035	1225·6 (1147·9 to 1303·7)	767·6 (694·4 to 839·5)	445·6 (419·5 to 471·6)	12·3 (9·5 to 15·4)	0·0 (0·0 to 0·0)	2174·7 (2036·8 to 2313·3)	1362·0 (1232·2 to 1489·6)	790·8 (744·4 to 836·8)	21·9 (16·9 to 27·3)	0·0 (0·0 to 0·0)
Latvia	2036	1240·4 (1160·8 to 1320·8)	779·7 (704·4 to 855·9)	447·9 (421·6 to 474·1)	12·7 (9·8 to 15·9)	0·0 (0·0 to 0·0)	2200·9 (2059·7 to 2343·6)	1383·6 (1249·9 to 1518·6)	794·8 (748·1 to 841·2)	22·5 (17·4 to 28·3)	0·0 (0·0 to 0·0)

Latvia	2037	1256·2 (1174·6 to 1342·0)	792·7 (716·9 to 871·2)	450·4 (423·9 to 476·6)	13·0 (10·1 to 16·5)	0·0 (0·0 to 0·0)	2229·0 (2084·2 to 2381·2)	1406·6 (1272·1 to 1545·8)	799·2 (752·1 to 845·7)	23·1 (17·9 to 29·3)	0·0 (0·0 to 0·0)
Latvia	2038	1269·4 (1186·6 to 1357·9)	804·1 (721·5 to 884·3)	451·9 (425·2 to 478·2)	13·4 (10·3 to 17·0)	0·0 (0·0 to 0·0)	2252·4 (2105·6 to 2409·6)	1426·8 (1280·2 to 1569·0)	801·8 (754·5 to 848·5)	23·8 (18·3 to 30·1)	0·0 (0·0 to 0·0)
Latvia	2039	1282·4 (1190·4 to 1373·8)	815·3 (727·2 to 897·3)	453·3 (426·7 to 479·8)	13·8 (10·6 to 17·5)	0·0 (0·0 to 0·0)	2275·5 (2112·3 to 2437·7)	1446·7 (1290·3 to 1592·2)	804·4 (757·2 to 851·3)	24·4 (18·7 to 31·1)	0·0 (0·0 to 0·0)
Latvia	2040	1294·9 (1200·4 to 1390·7)	826·3 (735·7 to 912·0)	454·5 (428·1 to 481·1)	14·1 (10·8 to 18·0)	0·0 (0·0 to 0·0)	2297·7 (2130·0 to 2467·7)	1466·2 (1305·5 to 1618·3)	806·4 (759·6 to 853·6)	25·0 (19·2 to 32·0)	0·0 (0·0 to 0·0)
Latvia	2041	1308·1 (1212·5 to 1407·4)	837·8 (747·4 to 929·0)	455·9 (429·2 to 482·8)	14·5 (11·0 to 18·6)	0·0 (0·0 to 0·0)	2321·2 (2151·4 to 2497·2)	1486·5 (1326·2 to 1648·4)	808·9 (761·6 to 856·7)	25·7 (19·6 to 33·0)	0·0 (0·0 to 0·0)
Latvia	2042	1319·0 (1222·0 to 1422·5)	847·4 (754·3 to 942·9)	456·7 (429·8 to 483·7)	14·9 (11·2 to 19·2)	0·0 (0·0 to 0·0)	2340·4 (2168·3 to 2524·1)	1503·6 (1338·4 to 1673·1)	810·4 (762·6 to 858·3)	26·4 (19·9 to 34·0)	0·0 (0·0 to 0·0)
Latvia	2043	1331·1 (1233·0 to 1436·5)	857·9 (763·3 to 955·6)	457·9 (430·9 to 485·3)	15·3 (11·5 to 19·8)	0·0 (0·0 to 0·0)	2361·9 (2187·8 to 2548·9)	1522·3 (1354·5 to 1695·6)	812·5 (764·6 to 861·1)	27·1 (20·4 to 35·1)	0·0 (0·0 to 0·0)
Latvia	2044	1342·8 (1241·7 to 1453·8)	868·0 (769·6 to 969·8)	459·1 (432·0 to 487·0)	15·7 (11·7 to 20·4)	0·0 (0·0 to 0·0)	2382·7 (2203·2 to 2579·6)	1540·2 (1365·6 to 1720·9)	814·7 (766·6 to 864·1)	27·8 (20·8 to 36·3)	0·0 (0·0 to 0·0)
Latvia	2045	1352·9 (1246·0 to 1466·0)	877·1 (773·0 to 982·7)	459·8 (432·6 to 487·8)	16·1 (12·0 to 21·0)	0·0 (0·0 to 0·0)	2400·6 (2211·0 to 2601·3)	1556·3 (1371·6 to 1743·8)	815·8 (767·7 to 865·5)	28·5 (21·3 to 37·2)	0·0 (0·0 to 0·0)
Latvia	2046	1361·9 (1253·1 to 1479·9)	885·5 (782·8 to 994·5)	459·9 (432·8 to 488·0)	16·4 (12·3 to 21·7)	0·0 (0·0 to 0·0)	2416·6 (2223·5 to 2625·9)	1571·3 (1389·0 to 1764·6)	816·1 (768·0 to 866·0)	29·2 (21·7 to 38·5)	0·0 (0·0 to 0·0)
Latvia	2047	1369·1 (1254·7 to 1490·0)	893·0 (785·4 to 1003·1)	459·3 (432·4 to 487·6)	16·8 (12·5 to 22·2)	0·0 (0·0 to 0·0)	2429·4 (2226·4 to 2644·0)	1584·5 (1393·7 to 1779·9)	815·1 (767·3 to 865·2)	29·8 (22·2 to 39·4)	0·0 (0·0 to 0·0)
Latvia	2048	1376·2 (1261·7 to 1498·5)	900·6 (788·2 to 1020·0)	458·4 (431·4 to 486·7)	17·2 (12·7 to 22·9)	0·0 (0·0 to 0·0)	2441·9 (2238·8 to 2658·9)	1598·1 (1398·7 to 1809·9)	813·3 (765·5 to 863·6)	30·5 (22·5 to 40·6)	0·0 (0·0 to 0·0)
Latvia	2049	1381·2 (1259·0 to 1508·6)	907·1 (792·9 to 1028·6)	456·5 (429·6 to 484·8)	17·5 (12·9 to 23·5)	0·0 (0·0 to 0·0)	2450·8 (2234·0 to 2676·9)	1609·6 (1406·9 to 1825·1)	810·1 (762·3 to 860·3)	31·1 (22·9 to 41·7)	0·0 (0·0 to 0·0)
Latvia	2050	1384·4 (1258·7 to 1517·4)	913·0 (793·4 to 1041·1)	453·6 (426·8 to 481·8)	17·8 (13·0 to 24·0)	0·0 (0·0 to 0·0)	2456·5 (2233·4 to 2692·5)	1620·0 (1407·8 to 1847·4)	804·9 (757·3 to 855·0)	31·6 (23·1 to 42·6)	0·0 (0·0 to 0·0)

Morocco*	1995	47·9 (41·3 to 55·3)	14·6 (11·9 to 17·5)	29·8 (23·9 to 36·6)	2·0 (1·4 to 2·9)	1·5 (1·5 to 1·5)	116·8 (100·7 to 134·7)	35·5 (29·1 to 42·6)	72·7 (58·3 to 89·3)	4·9 (3·4 to 7·1)	3·6 (3·6 to 3·6)
Morocco*	1996	50·8 (44·0 to 58·2)	14·7 (12·1 to 17·6)	31·3 (25·4 to 37·8)	2·7 (1·9 to 3·8)	2·0 (2·0 to 2·0)	123·8 (107·3 to 141·9)	35·9 (29·5 to 42·9)	76·2 (61·9 to 92·2)	6·6 (4·6 to 9·3)	5·0 (5·0 to 5·0)
Morocco*	1997	52·7 (45·7 to 60·6)	15·0 (12·5 to 18·0)	32·9 (26·8 to 39·5)	3·8 (2·6 to 5·2)	0·9 (0·9 to 0·9)	128·4 (111·4 to 147·7)	36·7 (30·5 to 43·9)	80·3 (65·3 to 96·4)	9·1 (6·4 to 12·7)	2·3 (2·3 to 2·3)
Morocco*	1998	59·1 (51·8 to 66·9)	16·4 (13·5 to 19·6)	35·4 (28·9 to 42·1)	5·0 (3·5 to 6·8)	2·2 (2·2 to 2·2)	144·0 (126·4 to 163·0)	40·0 (33·0 to 47·7)	86·3 (70·5 to 102·7)	12·2 (8·5 to 16·5)	5·4 (5·4 to 5·4)
Morocco*	1999	60·2 (52·8 to 68·2)	16·8 (13·8 to 20·0)	36·8 (30·2 to 43·5)	5·2 (3·7 to 7·1)	1·4 (1·4 to 1·4)	146·9 (128·8 to 166·3)	41·0 (33·7 to 48·8)	89·8 (73·7 to 106·1)	12·7 (9·0 to 17·3)	3·3 (3·3 to 3·3)
Morocco*	2000	80·4 (70·6 to 91·6)	19·2 (16·0 to 22·7)	40·5 (33·5 to 48·3)	18·4 (13·1 to 25·3)	2·3 (2·3 to 2·3)	196·0 (172·0 to 223·3)	46·8 (39·0 to 55·3)	98·7 (81·7 to 117·7)	44·9 (32·0 to 61·6)	5·5 (5·5 to 5·5)
Morocco*	2001	84·1 (73·9 to 95·2)	21·8 (18·2 to 25·8)	44·0 (36·8 to 52·4)	17·4 (12·2 to 23·9)	0·9 (0·9 to 0·9)	205·0 (180·1 to 232·0)	53·1 (44·4 to 63·0)	107·4 (89·7 to 127·7)	42·3 (29·8 to 58·2)	2·2 (2·2 to 2·2)
Morocco*	2002	91·6 (81·0 to 103·3)	24·9 (20·8 to 29·7)	48·3 (40·5 to 57·1)	17·3 (12·1 to 23·7)	1·1 (1·1 to 1·1)	223·3 (197·5 to 251·8)	60·7 (50·6 to 72·4)	117·7 (98·7 to 139·2)	42·2 (29·5 to 57·7)	2·7 (2·7 to 2·7)
Morocco*	2003	96·4 (85·5 to 108·1)	26·4 (22·1 to 31·2)	51·4 (43·2 to 60·2)	17·0 (11·9 to 23·2)	1·6 (1·6 to 1·6)	235·1 (208·5 to 263·5)	64·3 (53·9 to 76·0)	125·4 (105·3 to 146·7)	41·6 (29·0 to 56·5)	3·8 (3·8 to 3·8)
Morocco*	2004	103·3 (92·1 to 115·4)	29·1 (24·3 to 34·3)	55·9 (47·7 to 64·8)	17·2 (12·1 to 23·6)	1·0 (1·0 to 1·0)	251·7 (224·6 to 281·3)	70·8 (59·3 to 83·6)	136·3 (116·3 to 157·9)	42·0 (29·4 to 57·5)	2·6 (2·6 to 2·6)
Morocco*	2005	119·8 (107·7 to 132·8)	34·8 (29·5 to 40·7)	62·3 (53·5 to 71·6)	17·9 (12·5 to 25·0)	4·8 (4·8 to 4·8)	292·1 (262·6 to 323·7)	84·8 (72·0 to 99·1)	151·8 (130·5 to 174·6)	43·7 (30·5 to 60·8)	11·7 (11·7 to 11·7)
Morocco*	2006	113·8 (102·6 to 125·1)	40·1 (34·0 to 46·1)	66·1 (57·5 to 75·9)	4·6 (3·2 to 6·6)	3·0 (3·0 to 3·0)	277·4 (250·0 to 305·0)	97·7 (82·9 to 112·3)	161·2 (140·1 to 185·1)	11·3 (7·9 to 16·0)	7·2 (7·2 to 7·2)
Morocco*	2007	124·5 (112·5 to 136·7)	46·6 (39·8 to 53·4)	71·0 (61·8 to 80·8)	5·1 (3·5 to 7·2)	1·9 (1·9 to 1·9)	303·5 (274·2 to 333·3)	113·6 (97·1 to 130·2)	173·0 (150·7 to 196·9)	12·4 (8·7 to 17·6)	4·6 (4·6 to 4·6)
Morocco*	2008	140·3 (127·3 to 153·4)	53·9 (46·4 to 61·3)	76·3 (67·2 to 86·6)	5·4 (3·8 to 7·6)	4·7 (4·7 to 4·7)	342·0 (310·4 to 374·0)	131·3 (113·1 to 149·4)	185·9 (163·9 to 211·1)	13·2 (9·3 to 18·6)	11·6 (11·6 to 11·6)

Morocco*	2009	150·4 (137·7 to 164·4)	58·8 (51·0 to 66·4)	80·6 (71·3 to 91·7)	5·7 (4·0 to 8·1)	5·3 (5·3 to 5·3)	366·7 (335·7 to 400·8)	143·3 (124·3 to 161·9)	196·4 (173·8 to 223·6)	14·0 (9·7 to 19·7)	13·0 (13·0 to 13·0)
Morocco*	2010	153·7 (140·1 to 168·4)	61·5 (53·4 to 69·7)	83·7 (74·2 to 94·9)	6·1 (4·3 to 8·5)	2·5 (2·5 to 2·5)	374·7 (341·5 to 410·4)	149·8 (130·2 to 170·0)	203·9 (180·8 to 231·3)	14·8 (10·4 to 20·7)	6·1 (6·1 to 6·1)
Morocco*	2011	162·8 (148·9 to 177·6)	66·9 (58·0 to 75·8)	86·9 (76·8 to 98·2)	6·1 (4·3 to 8·5)	2·9 (2·9 to 2·9)	396·8 (363·0 to 433·0)	163·2 (141·5 to 184·7)	211·8 (187·2 to 239·4)	14·8 (10·4 to 20·7)	7·0 (7·0 to 7·0)
Morocco*	2012	165·9 (151·8 to 180·4)	69·9 (61·3 to 79·0)	88·1 (78·0 to 99·5)	6·2 (4·4 to 8·8)	1·7 (1·7 to 1·7)	404·4 (370·1 to 439·8)	170·4 (149·4 to 192·5)	214·7 (190·2 to 242·6)	15·2 (10·8 to 21·4)	4·2 (4·2 to 4·2)
Morocco*	2013	169·0 (155·2 to 183·9)	69·0 (60·6 to 78·4)	87·3 (76·9 to 98·2)	6·5 (4·5 to 9·1)	6·2 (6·2 to 6·2)	412·0 (378·3 to 448·2)	168·3 (147·7 to 191·1)	212·8 (187·5 to 239·4)	15·8 (11·1 to 22·2)	15·1 (15·1 to 15·1)
Morocco*	2014	167·5 (154·6 to 182·0)	68·3 (60·3 to 77·7)	87·2 (77·0 to 98·3)	6·3 (4·4 to 8·9)	5·8 (5·8 to 5·8)	408·4 (376·8 to 443·7)	166·5 (147·0 to 189·4)	212·5 (187·6 to 239·7)	15·3 (10·8 to 21·6)	14·1 (14·1 to 14·1)
Morocco*	2015	160·2 (148·2 to 173·5)	66·8 (59·9 to 74·6)	85·9 (76·2 to 96·2)	5·8 (4·2 to 8·2)	1·6 (1·6 to 1·6)	390·4 (361·3 to 422·9)	163·0 (146·0 to 181·8)	209·4 (185·7 to 234·4)	14·2 (10·2 to 19·9)	3·8 (3·8 to 3·8)
Morocco*	2016	167·1 (156·6 to 179·5)	68·1 (61·6 to 75·1)	85·8 (77·1 to 95·1)	6·5 (4·7 to 8·9)	6·7 (6·7 to 6·7)	407·2 (381·7 to 437·7)	166·0 (150·2 to 183·0)	209·1 (187·9 to 231·9)	15·8 (11·5 to 21·7)	16·2 (16·2 to 16·2)
Morocco*	2017	168·3 (158·4 to 179·1)	69·9 (63·1 to 76·6)	85·6 (78·0 to 94·0)	8·0 (6·0 to 10·6)	4·7 (4·7 to 4·7)	410·4 (386·1 to 436·5)	170·5 (153·9 to 186·9)	208·8 (190·2 to 229·1)	19·6 (14·5 to 25·8)	11·5 (11·5 to 11·5)
Morocco*	2018	171·9 (161·0 to 182·4)	71·3 (64·7 to 78·3)	84·4 (76·9 to 92·5)	14·8 (11·0 to 19·3)	1·4 (1·4 to 1·4)	419·1 (392·6 to 444·5)	173·8 (157·8 to 191·0)	205·9 (187·5 to 225·4)	36·0 (26·8 to 47·1)	3·5 (3·5 to 3·5)
Morocco*	2019	175·8 (164·6 to 186·7)	73·4 (66·6 to 81·2)	85·4 (77·8 to 93·4)	15·5 (11·5 to 20·4)	1·5 (1·4 to 1·5)	428·5 (401·2 to 455·0)	178·9 (162·4 to 197·9)	208·2 (189·7 to 227·6)	37·8 (28·0 to 49·8)	3·5 (3·5 to 3·6)
Morocco*	2020	198·8 (187·9 to 209·4)	100·2 (93·5 to 107·9)	81·5 (74·2 to 89·0)	15·2 (11·3 to 20·0)	1·9 (1·9 to 2·1)	484·6 (458·1 to 510·5)	244·3 (227·9 to 263·1)	198·6 (180·9 to 217·0)	37·0 (27·4 to 48·8)	4·7 (4·6 to 5·1)
Morocco*	2021	176·5 (165·4 to 187·7)	80·6 (73·1 to 89·0)	78·9 (71·9 to 86·1)	15·0 (11·1 to 19·9)	1·9 (1·8 to 2·1)	430·1 (403·1 to 457·7)	196·6 (178·2 to 216·9)	192·3 (175·2 to 209·9)	36·6 (27·1 to 48·5)	4·6 (4·5 to 5·2)
Morocco*	2022	169·2 (158·3 to 180·3)	72·6 (65·8 to 80·1)	79·6 (72·5 to 87·0)	15·5 (11·5 to 20·5)	1·5 (1·4 to 1·7)	412·6 (385·8 to 439·5)	177·0 (160·3 to 195·3)	194·2 (176·8 to 212·0)	37·8 (28·0 to 49·9)	3·6 (3·4 to 4·1)

Morocco*	2023	172·8 (161·7 to 184·2)	74·7 (67·6 to 82·7)	80·5 (73·3 to 87·9)	16·1 (11·9 to 21·2)	1·5 (1·4 to 1·7)	421·1 (394·1 to 449·0)	182·1 (164·7 to 201·6)	196·2 (178·6 to 214·2)	39·2 (29·0 to 51·8)	3·7 (3·4 to 4·2)
Morocco*	2024	176·4 (164·9 to 188·2)	76·9 (69·4 to 85·3)	81·3 (74·0 to 88·8)	16·6 (12·3 to 22·0)	1·6 (1·4 to 1·8)	429·9 (401·9 to 458·7)	187·5 (169·1 to 208·0)	198·1 (180·4 to 216·5)	40·5 (29·9 to 53·6)	3·8 (3·5 to 4·4)
Morocco*	2025	180·0 (168·1 to 192·2)	79·1 (71·2 to 88·2)	82·1 (74·6 to 89·8)	17·2 (12·7 to 22·9)	1·6 (1·5 to 1·9)	438·9 (409·9 to 468·6)	192·9 (173·7 to 215·0)	200·1 (182·0 to 218·8)	42·0 (31·0 to 55·8)	3·9 (3·6 to 4·6)
Morocco*	2026	183·8 (171·5 to 196·4)	81·4 (73·1 to 91·2)	82·9 (75·4 to 90·7)	17·8 (13·2 to 23·6)	1·6 (1·5 to 1·9)	448·1 (418·2 to 478·9)	198·6 (178·1 to 222·4)	202·1 (183·9 to 221·1)	43·5 (32·1 to 57·6)	4·0 (3·6 to 4·7)
Morocco*	2027	187·6 (175·0 to 200·6)	83·8 (74·8 to 94·0)	83·7 (76·2 to 91·5)	18·4 (13·6 to 24·6)	1·7 (1·5 to 1·9)	457·4 (426·6 to 489·0)	204·3 (182·5 to 229·1)	204·1 (185·8 to 223·2)	45·0 (33·1 to 59·9)	4·0 (3·7 to 4·7)
Morocco*	2028	191·6 (178·5 to 205·2)	86·3 (76·7 to 97·4)	84·6 (77·0 to 92·5)	19·1 (14·0 to 25·2)	1·7 (1·5 to 2·0)	467·1 (435·1 to 500·3)	210·3 (187·1 to 237·4)	206·2 (187·8 to 225·6)	46·5 (34·1 to 61·5)	4·1 (3·7 to 4·8)
Morocco*	2029	195·8 (182·0 to 209·9)	88·8 (78·9 to 100·6)	85·5 (77·8 to 93·5)	19·8 (14·4 to 26·1)	1·7 (1·5 to 2·0)	477·2 (443·7 to 511·8)	216·5 (192·3 to 245·3)	208·4 (189·7 to 227·9)	48·2 (35·2 to 63·7)	4·2 (3·8 to 4·9)
Morocco*	2030	199·9 (185·5 to 214·9)	91·4 (80·8 to 103·7)	86·3 (78·7 to 94·6)	20·4 (15·0 to 27·1)	1·7 (1·6 to 2·0)	487·4 (452·2 to 523·8)	222·8 (197·0 to 252·8)	210·5 (191·7 to 230·5)	49·8 (36·5 to 66·0)	4·2 (3·8 to 5·0)
Morocco*	2031	204·2 (189·1 to 219·8)	94·1 (82·8 to 106·8)	87·2 (79·4 to 95·6)	21·1 (15·4 to 27·9)	1·8 (1·6 to 2·1)	497·7 (461·0 to 535·8)	229·3 (201·8 to 260·4)	212·6 (193·6 to 233·2)	51·5 (37·6 to 68·0)	4·3 (3·9 to 5·1)
Morocco*	2032	208·7 (193·4 to 224·6)	97·1 (85·6 to 109·6)	88·0 (80·1 to 96·5)	21·8 (15·9 to 28·8)	1·8 (1·6 to 2·1)	508·7 (471·5 to 547·6)	236·6 (208·6 to 267·3)	214·6 (195·3 to 235·3)	53·1 (38·7 to 70·2)	4·4 (3·9 to 5·1)
Morocco*	2033	213·3 (197·7 to 229·9)	100·1 (88·2 to 113·0)	88·9 (80·8 to 97·4)	22·5 (16·3 to 29·8)	1·8 (1·6 to 2·2)	519·9 (482·0 to 560·4)	244·0 (215·1 to 275·5)	216·7 (197·0 to 237·4)	54·8 (39·7 to 72·7)	4·5 (4·0 to 5·3)
Morocco*	2034	217·8 (201·6 to 234·9)	103·0 (90·7 to 116·4)	89·7 (81·5 to 98·3)	23·2 (16·8 to 30·8)	1·9 (1·7 to 2·2)	530·9 (491·4 to 572·6)	251·2 (221·1 to 283·6)	218·6 (198·7 to 239·5)	56·5 (41·0 to 75·0)	4·6 (4·1 to 5·4)
Morocco*	2035	222·4 (205·7 to 240·1)	106·1 (93·0 to 120·4)	90·5 (82·2 to 99·1)	23·9 (17·4 to 31·8)	1·9 (1·7 to 2·3)	542·1 (501·4 to 585·4)	258·5 (226·8 to 293·6)	220·6 (200·4 to 241·6)	58·2 (42·4 to 77·6)	4·7 (4·1 to 5·5)
Morocco*	2036	226·9 (209·4 to 245·2)	109·0 (95·2 to 124·2)	91·3 (82·9 to 100·0)	24·6 (17·9 to 32·9)	2·0 (1·7 to 2·3)	553·2 (510·5 to 597·7)	265·8 (232·1 to 302·7)	222·5 (202·1 to 243·8)	60·0 (43·6 to 80·1)	4·8 (4·2 to 5·7)

Morocco*	2037	231·4 (213·1 to 250·4)	112·0 (97·6 to 127·6)	92·0 (83·7 to 100·9)	25·4 (18·4 to 33·8)	2·0 (1·8 to 2·4)	564·2 (519·5 to 610·4)	273·0 (238·0 to 311·2)	224·4 (203·9 to 246·0)	61·8 (45·0 to 82·4)	5·0 (4·3 to 5·9)
Morocco*	2038	235·9 (217·1 to 255·5)	114·9 (99·5 to 131·1)	92·8 (84·3 to 101·7)	26·1 (18·9 to 34·9)	2·1 (1·8 to 2·5)	575·1 (529·2 to 622·8)	280·2 (242·5 to 319·6)	226·1 (205·4 to 248·0)	63·7 (46·1 to 85·2)	5·1 (4·4 to 6·1)
Morocco*	2039	240·7 (220·9 to 261·4)	118·0 (102·0 to 135·6)	93·6 (85·0 to 102·7)	26·9 (19·4 to 36·2)	2·1 (1·9 to 2·6)	586·8 (538·6 to 637·3)	287·8 (248·7 to 330·6)	228·2 (207·2 to 250·3)	65·7 (47·2 to 88·2)	5·2 (4·5 to 6·3)
Morocco*	2040	245·6 (224·9 to 267·6)	121·2 (104·2 to 139·6)	94·5 (85·8 to 103·6)	27·8 (19·9 to 37·4)	2·2 (1·9 to 2·7)	598·7 (548·3 to 652·3)	295·4 (254·1 to 340·4)	230·3 (209·1 to 252·6)	67·7 (48·5 to 91·1)	5·4 (4·6 to 6·5)
Morocco*	2041	250·5 (228·8 to 273·4)	124·3 (106·5 to 144·0)	95·3 (86·5 to 104·6)	28·6 (20·5 to 38·8)	2·3 (1·9 to 2·8)	610·7 (557·7 to 666·4)	303·0 (259·7 to 351·1)	232·4 (210·9 to 255·0)	69·8 (50·0 to 94·6)	5·5 (4·7 to 6·8)
Morocco*	2042	255·6 (232·7 to 279·9)	127·5 (108·6 to 148·3)	96·2 (87·3 to 105·6)	29·5 (21·0 to 40·1)	2·3 (2·0 to 2·9)	623·1 (567·2 to 682·4)	310·8 (264·8 to 361·6)	234·6 (212·9 to 257·4)	72·0 (51·3 to 97·8)	5·7 (4·8 to 7·0)
Morocco*	2043	260·6 (236·7 to 286·5)	130·6 (110·8 to 152·6)	97·1 (88·0 to 106·6)	30·5 (21·6 to 41·3)	2·4 (2·0 to 3·0)	635·3 (577·1 to 698·3)	318·5 (270·2 to 371·9)	236·7 (214·6 to 259·9)	74·3 (52·6 to 100·8)	5·8 (4·9 to 7·3)
Morocco*	2044	265·6 (240·4 to 292·9)	133·8 (113·0 to 156·9)	98·0 (88·7 to 107·6)	31·4 (22·2 to 43·0)	2·5 (2·1 to 3·1)	647·5 (586·0 to 714·1)	326·1 (275·5 to 382·5)	238·8 (216·2 to 262·3)	76·6 (54·0 to 104·8)	6·0 (5·0 to 7·5)
Morocco*	2045	270·5 (244·3 to 299·4)	136·8 (115·0 to 161·2)	98·8 (89·4 to 108·7)	32·4 (22·7 to 44·6)	2·6 (2·1 to 3·3)	659·5 (595·6 to 729·8)	333·6 (280·4 to 393·0)	240·8 (217·9 to 264·9)	78·9 (55·3 to 108·6)	6·2 (5·2 to 7·9)
Morocco*	2046	275·5 (248·3 to 306·1)	139·9 (117·1 to 165·9)	99·6 (90·1 to 109·7)	33·4 (23·4 to 46·1)	2·6 (2·2 to 3·4)	671·7 (605·4 to 746·2)	341·1 (285·4 to 404·5)	242·9 (219·7 to 267·5)	81·3 (57·0 to 112·5)	6·4 (5·3 to 8·3)
Morocco*	2047	280·4 (251·9 to 312·7)	142·9 (119·0 to 170·3)	100·4 (90·8 to 110·7)	34·4 (24·0 to 47·8)	2·7 (2·2 to 3·5)	683·6 (614·1 to 762·3)	348·3 (290·1 to 415·2)	244·8 (221·4 to 270·0)	83·8 (58·6 to 116·4)	6·7 (5·4 to 8·6)
Morocco*	2048	285·3 (255·6 to 319·0)	145·8 (121·0 to 174·8)	101·2 (91·4 to 111·8)	35·4 (24·8 to 49·5)	2·8 (2·3 to 3·7)	695·5 (623·0 to 777·6)	355·4 (294·9 to 426·0)	246·8 (222·8 to 272·5)	86·4 (60·4 to 120·6)	6·9 (5·7 to 9·1)
Morocco*	2049	290·0 (259·3 to 324·9)	148·6 (122·8 to 179·0)	102·0 (92·0 to 112·9)	36·5 (25·3 to 51·5)	2·9 (2·4 to 3·9)	706·9 (632·1 to 791·9)	362·2 (299·4 to 436·4)	248·7 (224·3 to 275·2)	88·9 (61·7 to 125·5)	7·1 (5·8 to 9·5)
Morocco*	2050	294·9 (263·2 to 331·4)	151·5 (124·7 to 183·1)	102·8 (92·7 to 113·8)	37·6 (25·8 to 52·9)	3·0 (2·4 to 4·1)	718·9 (641·6 to 807·8)	369·2 (303·9 to 446·3)	250·6 (225·9 to 277·3)	91·6 (62·9 to 129·0)	7·4 (6·0 to 9·9)

Monaco	1995	2126·7 (1833·1 to 2467·3)	1738·9 (1449·6 to 2078·4)	144·3 (117·3 to 177·4)	243·5 (173·3 to 323·4)	0·0 (0·0 to 0·0)	2078·5 (1791·5 to 2411·3)	1699·5 (1416·7 to 2031·3)	141·1 (114·7 to 173·4)	238·0 (169·4 to 316·1)	0·0 (0·0 to 0·0)
Monaco	1996	2150·1 (1871·5 to 2491·3)	1745·8 (1459·1 to 2061·0)	146·2 (120·0 to 175·4)	258·1 (191·4 to 338·5)	0·0 (0·0 to 0·0)	2101·3 (1829·0 to 2434·8)	1706·2 (1426·0 to 2014·2)	142·9 (117·3 to 171·5)	252·2 (187·1 to 330·8)	0·0 (0·0 to 0·0)
Monaco	1997	2216·8 (1950·7 to 2516·9)	1791·4 (1527·1 to 2097·5)	149·8 (126·0 to 176·2)	275·6 (211·9 to 357·4)	0·0 (0·0 to 0·0)	2166·5 (1906·5 to 2459·8)	1750·8 (1492·5 to 2050·0)	146·4 (123·1 to 172·2)	269·3 (207·1 to 349·3)	0·0 (0·0 to 0·0)
Monaco	1998	2298·8 (2060·5 to 2560·6)	1854·9 (1616·2 to 2122·6)	154·3 (132·9 to 176·4)	289·6 (229·7 to 363·7)	0·0 (0·0 to 0·0)	2246·7 (2013·8 to 2502·5)	1812·9 (1579·5 to 2074·4)	150·8 (129·9 to 172·4)	283·0 (224·5 to 355·4)	0·0 (0·0 to 0·0)
Monaco	1999	2341·3 (2140·8 to 2564·7)	1886·3 (1685·0 to 2104·6)	158·3 (142·2 to 175·0)	296·7 (248·5 to 356·8)	0·0 (0·0 to 0·0)	2288·1 (2092·3 to 2506·5)	1843·5 (1646·8 to 2056·9)	154·7 (139·0 to 171·0)	289·9 (242·9 to 348·8)	0·0 (0·0 to 0·0)
Monaco	2000	2390·1 (2225·3 to 2553·9)	1923·6 (1759·7 to 2084·6)	163·5 (151·4 to 175·9)	303·0 (265·5 to 347·7)	0·0 (0·0 to 0·0)	2335·9 (2174·8 to 2495·9)	1880·0 (1719·8 to 2037·3)	159·8 (148·0 to 171·9)	296·1 (259·5 to 339·8)	0·0 (0·0 to 0·0)
Monaco	2001	2590·1 (2446·9 to 2733·3)	2078·5 (1943·4 to 2220·6)	180·5 (170·6 to 191·1)	331·2 (298·7 to 367·8)	0·0 (0·0 to 0·0)	2531·3 (2391·4 to 2671·3)	2031·3 (1899·3 to 2170·2)	176·4 (166·7 to 186·8)	323·7 (291·9 to 359·4)	0·0 (0·0 to 0·0)
Monaco	2002	2695·1 (2568·5 to 2831·0)	2163·4 (2040·3 to 2299·8)	183·8 (174·5 to 193·4)	347·9 (316·2 to 383·4)	0·0 (0·0 to 0·0)	2634·0 (2510·2 to 2766·8)	2114·3 (1994·0 to 2247·6)	179·6 (170·5 to 189·0)	340·0 (309·0 to 374·7)	0·0 (0·0 to 0·0)
Monaco	2003	2835·3 (2707·4 to 2964·5)	2270·2 (2143·5 to 2399·9)	181·1 (171·5 to 190·9)	384·0 (351·1 to 420·2)	0·0 (0·0 to 0·0)	2770·9 (2645·9 to 2897·2)	2218·7 (2094·9 to 2345·4)	176·9 (167·6 to 186·6)	375·3 (343·1 to 410·7)	0·0 (0·0 to 0·0)
Monaco	2004	3059·5 (2922·3 to 3189·6)	2475·7 (2345·6 to 2606·7)	180·2 (170·7 to 189·8)	403·6 (369·2 to 439·7)	0·0 (0·0 to 0·0)	2990·1 (2856·0 to 3117·2)	2419·6 (2292·4 to 2547·5)	176·1 (166·9 to 185·5)	394·5 (360·8 to 429·7)	0·0 (0·0 to 0·0)
Monaco	2005	3077·0 (2953·0 to 3223·0)	2498·3 (2372·7 to 2631·6)	176·6 (167·0 to 185·9)	402·1 (367·0 to 437·6)	0·0 (0·0 to 0·0)	3007·2 (2886·0 to 3149·9)	2441·6 (2318·8 to 2571·9)	172·6 (163·2 to 181·7)	393·0 (358·7 to 427·7)	0·0 (0·0 to 0·0)
Monaco	2006	3125·5 (2998·4 to 3265·9)	2536·3 (2407·8 to 2672·2)	178·9 (169·4 to 188·2)	410·3 (377·6 to 446·6)	0·0 (0·0 to 0·0)	3054·6 (2930·3 to 3191·8)	2478·8 (2353·1 to 2611·6)	174·9 (165·6 to 183·9)	401·0 (369·1 to 436·5)	0·0 (0·0 to 0·0)
Monaco	2007	3239·8 (3108·4 to 3387·6)	2629·8 (2502·2 to 2767·8)	191·2 (181·3 to 201·2)	418·8 (385·8 to 456·0)	0·0 (0·0 to 0·0)	3166·3 (3037·9 to 3310·7)	2570·1 (2445·4 to 2705·0)	186·9 (177·2 to 196·6)	409·3 (377·0 to 445·6)	0·0 (0·0 to 0·0)
Monaco	2008	3449·1 (3304·1 to 3591·5)	2805·2 (2664·7 to 2942·8)	198·7 (189·0 to 208·9)	445·2 (409·1 to 481·7)	0·0 (0·0 to 0·0)	3370·9 (3229·1 to 3510·1)	2741·5 (2604·2 to 2876·0)	194·2 (184·7 to 204·2)	435·1 (399·8 to 470·8)	0·0 (0·0 to 0·0)

Monaco	2009	3373·5 (3233·3 to 3512·5)	2729·1 (2594·2 to 2864·2)	193·1 (183·6 to 202·8)	451·3 (415·8 to 486·8)	0·0 (0·0 to 0·0)	3296·9 (3160·0 to 3432·8)	2667·2 (2535·4 to 2799·3)	188·7 (179·5 to 198·2)	441·0 (406·4 to 475·8)	0·0 (0·0 to 0·0)
Monaco	2010	3540·5 (3400·6 to 3679·9)	2879·5 (2748·5 to 3016·6)	194·3 (184·8 to 203·6)	466·7 (429·7 to 505·2)	0·0 (0·0 to 0·0)	3460·2 (3323·5 to 3596·4)	2814·2 (2686·2 to 2948·1)	189·9 (180·6 to 199·0)	456·1 (420·0 to 493·7)	0·0 (0·0 to 0·0)
Monaco	2011	3646·1 (3504·1 to 3781·3)	2961·8 (2832·9 to 3088·2)	199·5 (189·4 to 209·1)	484·9 (447·5 to 528·6)	0·0 (0·0 to 0·0)	3563·4 (3424·6 to 3695·5)	2894·6 (2768·7 to 3018·1)	194·9 (185·1 to 204·3)	473·9 (437·3 to 516·6)	0·0 (0·0 to 0·0)
Monaco	2012	3745·9 (3602·9 to 3886·7)	3033·3 (2896·3 to 3164·0)	213·0 (202·8 to 222·5)	499·6 (461·6 to 542·5)	0·0 (0·0 to 0·0)	3660·9 (3521·2 to 3798·5)	2964·5 (2830·6 to 3092·3)	208·1 (198·2 to 217·5)	488·2 (451·2 to 530·2)	0·0 (0·0 to 0·0)
Monaco	2013	3820·2 (3683·4 to 3964·0)	3072·7 (2940·5 to 3203·5)	225·4 (215·9 to 235·7)	522·2 (485·8 to 561·5)	0·0 (0·0 to 0·0)	3733·6 (3599·9 to 3874·1)	3003·0 (2873·8 to 3130·8)	220·3 (211·0 to 230·3)	510·3 (474·7 to 548·8)	0·0 (0·0 to 0·0)
Monaco	2014	4019·4 (3883·6 to 4165·8)	3236·3 (3104·2 to 3376·9)	244·4 (234·3 to 255·2)	538·6 (500·9 to 578·2)	0·0 (0·0 to 0·0)	3928·2 (3795·5 to 4071·3)	3162·9 (3033·8 to 3300·3)	238·8 (228·9 to 249·4)	526·4 (489·6 to 565·1)	0·0 (0·0 to 0·0)
Monaco	2015	3876·6 (3729·6 to 4019·9)	3156·9 (3020·0 to 3295·6)	249·2 (238·6 to 259·8)	470·6 (431·8 to 511·8)	0·0 (0·0 to 0·0)	3788·6 (3645·0 to 3928·7)	3085·3 (2951·5 to 3220·9)	243·5 (233·2 to 253·9)	459·9 (422·0 to 500·2)	0·0 (0·0 to 0·0)
Monaco	2016	3586·1 (3437·2 to 3721·8)	3017·3 (2873·6 to 3147·3)	248·6 (238·2 to 259·4)	320·2 (286·8 to 357·9)	0·0 (0·0 to 0·0)	3504·7 (3359·2 to 3637·4)	2948·8 (2808·4 to 3075·9)	242·9 (232·8 to 253·5)	313·0 (280·3 to 349·8)	0·0 (0·0 to 0·0)
Monaco	2017	3318·7 (3173·2 to 3463·6)	2829·1 (2686·7 to 2966·9)	243·2 (232·6 to 253·4)	246·3 (213·6 to 285·8)	0·0 (0·0 to 0·0)	3243·4 (3101·2 to 3385·0)	2764·9 (2625·8 to 2899·6)	237·7 (227·3 to 247·6)	240·7 (208·8 to 279·4)	0·0 (0·0 to 0·0)
Monaco	2018	3330·8 (3141·9 to 3518·7)	2864·6 (2683·3 to 3048·9)	244·1 (230·4 to 257·7)	222·1 (183·9 to 269·8)	0·0 (0·0 to 0·0)	3255·3 (3070·6 to 3438·9)	2799·7 (2622·5 to 2979·7)	238·6 (225·2 to 251·9)	217·0 (179·8 to 263·6)	0·0 (0·0 to 0·0)
Monaco	2019	3559·6 (3346·1 to 3778·6)	3060·9 (2858·1 to 3273·5)	258·4 (244·1 to 273·0)	240·3 (198·4 to 291·7)	0·0 (0·0 to 0·0)	3478·8 (3270·2 to 3692·9)	2991·4 (2793·2 to 3199·2)	252·5 (238·5 to 266·9)	234·9 (193·9 to 285·0)	0·0 (0·0 to 0·0)
Monaco	2020	3556·5 (3339·7 to 3793·9)	3085·2 (2878·9 to 3309·3)	241·4 (228·0 to 255·0)	229·9 (189·5 to 279·4)	0·0 (0·0 to 0·0)	3475·8 (3263·9 to 3707·8)	3015·2 (2813·6 to 3234·2)	235·9 (222·9 to 249·2)	224·7 (185·2 to 273·1)	0·0 (0·0 to 0·0)
Monaco	2021	3432·1 (3222·9 to 3660·5)	2974·1 (2775·3 to 3190·2)	232·0 (219·1 to 245·1)	225·9 (185·8 to 275·2)	0·0 (0·0 to 0·0)	3354·2 (3149·8 to 3577·4)	2906·7 (2712·4 to 3117·8)	226·8 (214·2 to 239·6)	220·8 (181·6 to 268·9)	0·0 (0·0 to 0·0)
Monaco	2022	3293·9 (3068·5 to 3530·4)	2828·4 (2616·9 to 3067·6)	233·4 (220·4 to 246·5)	232·2 (190·9 to 284·5)	0·0 (0·0 to 0·0)	3219·2 (2998·8 to 3450·3)	2764·2 (2557·5 to 2998·0)	228·1 (215·4 to 240·9)	226·9 (186·6 to 278·1)	0·0 (0·0 to 0·0)

Monaco	2023	3344·6 (3097·5 to 3606·2)	2871·1 (2633·4 to 3133·1)	234·7 (221·7 to 247·8)	238·8 (196·7 to 291·5)	0·0 (0·0 to 0·0)	3268·7 (3027·3 to 3524·4)	2806·0 (2573·7 to 3062·0)	229·3 (216·6 to 242·2)	233·4 (192·3 to 284·9)	0·0 (0·0 to 0·0)
Monaco	2024	3391·9 (3138·4 to 3675·0)	2910·2 (2664·0 to 3182·2)	235·9 (222·8 to 249·1)	245·8 (201·9 to 300·1)	0·0 (0·0 to 0·0)	3314·9 (3067·2 to 3591·6)	2844·2 (2603·5 to 3110·0)	230·5 (217·8 to 243·4)	240·2 (197·3 to 293·3)	0·0 (0·0 to 0·0)
Monaco	2025	3437·0 (3185·3 to 3730·0)	2946·8 (2691·1 to 3223·8)	237·2 (224·1 to 250·4)	253·1 (207·2 to 308·6)	0·0 (0·0 to 0·0)	3359·0 (3113·0 to 3645·3)	2880·0 (2630·0 to 3150·7)	231·8 (219·0 to 244·8)	247·3 (202·5 to 301·6)	0·0 (0·0 to 0·0)
Monaco	2026	3484·3 (3226·1 to 3772·9)	2985·2 (2733·2 to 3266·9)	238·5 (225·3 to 251·8)	260·6 (212·9 to 318·0)	0·0 (0·0 to 0·0)	3405·2 (3152·9 to 3687·3)	2917·4 (2671·2 to 3192·8)	233·1 (220·2 to 246·1)	254·7 (208·1 to 310·8)	0·0 (0·0 to 0·0)
Monaco	2027	3532·7 (3265·7 to 3829·5)	3024·5 (2771·0 to 3315·2)	239·8 (226·6 to 253·2)	268·4 (218·4 to 329·1)	0·0 (0·0 to 0·0)	3452·6 (3191·6 to 3742·6)	2955·9 (2708·2 to 3240·0)	234·4 (221·4 to 247·5)	262·3 (213·4 to 321·6)	0·0 (0·0 to 0·0)
Monaco	2028	3582·3 (3307·2 to 3887·0)	3064·1 (2795·6 to 3369·7)	241·8 (228·5 to 255·4)	276·4 (225·0 to 338·1)	0·0 (0·0 to 0·0)	3501·0 (3232·1 to 3798·8)	2994·6 (2732·2 to 3293·2)	236·3 (223·4 to 249·6)	270·1 (219·8 to 330·5)	0·0 (0·0 to 0·0)
Monaco	2029	3633·1 (3346·3 to 3935·6)	3105·8 (2831·8 to 3398·1)	243·3 (229·8 to 257·4)	284·0 (230·7 to 347·7)	0·0 (0·0 to 0·0)	3550·7 (3270·4 to 3846·3)	3035·3 (2767·6 to 3321·1)	237·8 (224·6 to 251·5)	277·6 (225·4 to 339·8)	0·0 (0·0 to 0·0)
Monaco	2030	3680·2 (3365·8 to 4002·5)	3143·5 (2845·3 to 3462·0)	244·7 (231·1 to 258·7)	291·9 (237·6 to 358·3)	0·0 (0·0 to 0·0)	3596·7 (3289·4 to 3911·7)	3072·2 (2780·7 to 3383·4)	239·2 (225·9 to 252·9)	285·3 (232·2 to 350·1)	0·0 (0·0 to 0·0)
Monaco	2031	3725·9 (3390·2 to 4073·3)	3179·7 (2870·8 to 3514·9)	246·2 (232·5 to 260·3)	300·0 (243·3 to 369·9)	0·0 (0·0 to 0·0)	3641·4 (3313·3 to 3980·9)	3107·6 (2805·6 to 3435·2)	240·6 (227·3 to 254·4)	293·2 (237·8 to 361·5)	0·0 (0·0 to 0·0)
Monaco	2032	3769·0 (3433·3 to 4126·6)	3213·2 (2889·9 to 3558·4)	247·6 (233·9 to 261·8)	308·3 (248·8 to 381·1)	0·0 (0·0 to 0·0)	3683·5 (3355·4 to 4033·0)	3140·3 (2824·4 to 3477·7)	241·9 (228·5 to 255·9)	301·3 (243·2 to 372·4)	0·0 (0·0 to 0·0)
Monaco	2033	3811·9 (3453·6 to 4201·9)	3246·4 (2885·8 to 3618·2)	248·9 (235·1 to 263·2)	316·6 (255·1 to 392·8)	0·0 (0·0 to 0·0)	3725·4 (3375·2 to 4106·6)	3172·8 (2820·4 to 3536·1)	243·2 (229·7 to 257·2)	309·4 (249·3 to 383·9)	0·0 (0·0 to 0·0)
Monaco	2034	3857·8 (3493·5 to 4252·0)	3281·9 (2923·8 to 3667·6)	250·4 (236·4 to 264·8)	325·5 (261·4 to 405·0)	0·0 (0·0 to 0·0)	3770·3 (3414·2 to 4155·6)	3207·5 (2857·5 to 3584·4)	244·7 (231·1 to 258·8)	318·1 (255·5 to 395·8)	0·0 (0·0 to 0·0)
Monaco	2035	3905·9 (3528·4 to 4318·5)	3319·6 (2949·6 to 3736·1)	251·8 (237·6 to 266·3)	334·5 (267·8 to 416·9)	0·0 (0·0 to 0·0)	3817·3 (3448·4 to 4220·5)	3244·2 (2882·7 to 3651·4)	246·1 (232·2 to 260·3)	326·9 (261·7 to 407·4)	0·0 (0·0 to 0·0)
Monaco	2036	3952·7 (3567·0 to 4390·7)	3355·7 (2979·5 to 3779·3)	253·3 (238·8 to 267·9)	343·7 (273·6 to 429·5)	0·0 (0·0 to 0·0)	3863·0 (3486·1 to 4291·0)	3279·6 (2911·9 to 3693·5)	247·5 (233·4 to 261·8)	335·9 (267·3 to 419·7)	0·0 (0·0 to 0·0)

Monaco	2037	4000·9 (3603·9 to 4466·0)	3392·8 (3008·2 to 3862·8)	254·7 (240·3 to 269·4)	353·3 (279·7 to 446·3)	0·0 (0·0 to 0·0)	3910·1 (3522·1 to 4364·7)	3315·9 (2940·0 to 3775·2)	249·0 (234·8 to 263·3)	345·3 (273·4 to 436·1)	0·0 (0·0 to 0·0)
Monaco	2038	4049·7 (3641·6 to 4538·5)	3430·2 (3037·6 to 3911·0)	256·3 (241·6 to 271·2)	363·2 (287·0 to 461·2)	0·0 (0·0 to 0·0)	3957·8 (3558·9 to 4435·5)	3352·4 (2968·7 to 3822·3)	250·5 (236·1 to 265·0)	354·9 (280·5 to 450·7)	0·0 (0·0 to 0·0)
Monaco	2039	4102·8 (3664·5 to 4621·8)	3471·6 (3052·7 to 3982·5)	257·8 (243·0 to 272·9)	373·4 (293·3 to 476·1)	0·0 (0·0 to 0·0)	4009·7 (3581·4 to 4517·0)	3392·8 (2983·5 to 3892·2)	252·0 (237·5 to 266·7)	364·9 (286·7 to 465·3)	0·0 (0·0 to 0·0)
Monaco	2040	4155·5 (3705·1 to 4690·0)	3512·3 (3080·7 to 4021·2)	259·4 (244·4 to 274·5)	383·8 (300·6 to 491·4)	0·0 (0·0 to 0·0)	4061·2 (3621·0 to 4583·6)	3432·7 (3010·8 to 3930·0)	253·5 (238·8 to 268·3)	375·1 (293·8 to 480·3)	0·0 (0·0 to 0·0)
Monaco	2041	4208·7 (3737·9 to 4755·4)	3553·3 (3107·8 to 4083·3)	260·9 (245·7 to 276·2)	394·5 (306·3 to 507·7)	0·0 (0·0 to 0·0)	4113·2 (3653·1 to 4647·5)	3472·6 (3037·3 to 3990·7)	255·0 (240·2 to 269·9)	385·6 (299·4 to 496·2)	0·0 (0·0 to 0·0)
Monaco	2042	4261·9 (3783·7 to 4822·8)	3594·0 (3141·4 to 4136·6)	262·4 (247·0 to 277·8)	405·5 (314·4 to 524·1)	0·0 (0·0 to 0·0)	4165·3 (3697·9 to 4713·3)	3512·5 (3070·1 to 4042·8)	256·5 (241·4 to 271·5)	396·3 (307·3 to 512·2)	0·0 (0·0 to 0·0)
Monaco	2043	4315·8 (3820·0 to 4901·8)	3635·2 (3160·9 to 4209·1)	263·9 (248·4 to 279·5)	416·7 (322·6 to 541·6)	0·0 (0·0 to 0·0)	4217·9 (3733·3 to 4790·6)	3552·7 (3089·2 to 4113·6)	257·9 (242·8 to 273·1)	407·3 (315·3 to 529·4)	0·0 (0·0 to 0·0)
Monaco	2044	4366·7 (3876·3 to 4968·8)	3673·1 (3173·1 to 4254·4)	265·4 (249·9 to 281·1)	428·3 (330·2 to 559·4)	0·0 (0·0 to 0·0)	4267·7 (3788·4 to 4856·1)	3589·7 (3101·2 to 4157·9)	259·4 (244·2 to 274·7)	418·5 (322·7 to 546·8)	0·0 (0·0 to 0·0)
Monaco	2045	4424·8 (3895·5 to 5054·8)	3717·4 (3201·2 to 4304·3)	267·0 (251·4 to 282·8)	440·4 (337·4 to 575·4)	0·0 (0·0 to 0·0)	4324·4 (3807·1 to 4940·2)	3633·1 (3128·6 to 4206·7)	261·0 (245·7 to 276·4)	430·4 (329·7 to 562·3)	0·0 (0·0 to 0·0)
Monaco	2046	4483·3 (3949·4 to 5123·8)	3761·8 (3242·3 to 4389·8)	268·7 (252·9 to 284·5)	452·8 (344·8 to 593·3)	0·0 (0·0 to 0·0)	4381·6 (3859·8 to 5007·6)	3676·5 (3168·7 to 4290·2)	262·6 (247·1 to 278·1)	442·5 (337·0 to 579·8)	0·0 (0·0 to 0·0)
Monaco	2047	4539·0 (3997·2 to 5180·2)	3803·3 (3258·0 to 4432·2)	270·2 (254·3 to 286·2)	465·4 (351·7 to 612·2)	0·0 (0·0 to 0·0)	4436·0 (3906·5 to 5062·7)	3717·0 (3184·0 to 4331·6)	264·1 (248·5 to 279·7)	454·9 (343·7 to 598·3)	0·0 (0·0 to 0·0)
Monaco	2048	4598·1 (4019·3 to 5267·4)	3847·5 (3290·4 to 4530·3)	271·9 (255·8 to 288·1)	478·7 (358·9 to 632·0)	0·0 (0·0 to 0·0)	4493·8 (3928·1 to 5147·9)	3760·2 (3215·8 to 4427·5)	265·8 (250·0 to 281·6)	467·8 (350·8 to 617·6)	0·0 (0·0 to 0·0)
Monaco	2049	4658·6 (4049·6 to 5372·5)	3892·6 (3300·3 to 4554·6)	273·7 (257·4 to 290·0)	492·3 (366·1 to 652·5)	0·0 (0·0 to 0·0)	4552·9 (3957·7 to 5250·7)	3804·3 (3225·4 to 4451·3)	267·5 (251·6 to 283·4)	481·2 (357·8 to 637·7)	0·0 (0·0 to 0·0)
Monaco	2050	4719·2 (4118·4 to 5445·7)	3937·7 (3352·0 to 4651·9)	275·3 (258·9 to 291·7)	506·2 (373·7 to 673·4)	0·0 (0·0 to 0·0)	4612·2 (4024·9 to 5322·2)	3848·4 (3275·9 to 4546·4)	269·1 (253·0 to 285·1)	494·7 (365·3 to 658·1)	0·0 (0·0 to 0·0)

Moldova*	1995	123·3 (110·0 to 137·7)	83·1 (72·0 to 95·7)	38·8 (31·5 to 47·6)	1·4 (0·9 to 2·0)	0·0 (0·0 to 0·0)	382·8 (341·7 to 427·7)	258·0 (223·6 to 297·2)	120·5 (97·8 to 147·9)	4·3 (2·9 to 6·1)	0·0 (0·0 to 0·0)
Moldova*	1996	117·2 (105·3 to 130·5)	78·0 (67·9 to 89·2)	37·8 (30·8 to 46·0)	1·4 (0·9 to 1·9)	0·0 (0·0 to 0·0)	363·8 (327·1 to 405·3)	242·1 (210·7 to 277·1)	117·5 (95·7 to 142·9)	4·2 (2·9 to 5·9)	0·0 (0·0 to 0·0)
Moldova*	1997	111·9 (101·0 to 124·2)	72·8 (63·5 to 83·1)	37·4 (30·6 to 45·4)	1·4 (0·9 to 2·0)	0·2 (0·2 to 0·2)	347·5 (313·6 to 385·7)	226·2 (197·3 to 258·2)	116·2 (95·1 to 140·9)	4·3 (2·9 to 6·1)	0·7 (0·7 to 0·7)
Moldova*	1998	92·1 (83·1 to 102·9)	55·3 (48·0 to 63·7)	35·0 (29·2 to 42·3)	1·3 (0·8 to 1·7)	0·5 (0·5 to 0·5)	285·9 (257·9 to 319·6)	171·8 (149·1 to 197·7)	108·7 (90·6 to 131·3)	3·9 (2·6 to 5·4)	1·5 (1·5 to 1·5)
Moldova*	1999	80·8 (72·7 to 89·8)	42·6 (36·5 to 49·2)	33·0 (27·9 to 39·5)	1·1 (0·7 to 1·5)	4·0 (4·0 to 4·0)	250·8 (225·8 to 278·8)	132·4 (113·4 to 152·7)	102·5 (86·6 to 122·8)	3·4 (2·3 to 4·7)	12·5 (12·5 to 12·5)
Moldova*	2000	81·3 (73·3 to 90·1)	42·1 (35·7 to 48·7)	35·2 (30·0 to 41·5)	1·1 (0·7 to 1·5)	2·9 (2·9 to 2·9)	252·4 (227·7 to 279·7)	130·7 (110·9 to 151·1)	109·2 (93·1 to 128·8)	3·4 (2·3 to 4·8)	9·1 (9·1 to 9·1)
Moldova*	2001	83·1 (74·9 to 92·1)	41·7 (35·6 to 48·0)	38·6 (33·3 to 44·9)	1·1 (0·7 to 1·5)	1·7 (1·7 to 1·7)	258·2 (232·4 to 286·0)	129·5 (110·5 to 149·2)	119·9 (103·5 to 139·4)	3·4 (2·3 to 4·8)	5·4 (5·4 to 5·4)
Moldova*	2002	96·5 (87·5 to 106·1)	49·3 (42·1 to 56·2)	44·9 (39·1 to 51·4)	1·2 (0·8 to 1·7)	1·2 (1·2 to 1·2)	299·7 (271·7 to 329·5)	152·9 (130·9 to 174·6)	139·5 (121·5 to 159·7)	3·7 (2·5 to 5·2)	3·6 (3·6 to 3·6)
Moldova*	2003	113·7 (103·7 to 124·3)	59·1 (51·4 to 67·3)	51·9 (45·8 to 58·4)	1·0 (0·7 to 1·4)	1·7 (1·7 to 1·7)	353·0 (322·0 to 385·9)	183·4 (159·7 to 209·1)	161·1 (142·1 to 181·4)	3·2 (2·1 to 4·4)	5·3 (5·3 to 5·3)
Moldova*	2004	139·3 (128·6 to 150·6)	73·7 (65·3 to 83·2)	61·0 (54·7 to 68·0)	0·6 (0·4 to 0·8)	4·0 (4·0 to 4·0)	432·7 (399·4 to 467·5)	228·8 (202·8 to 258·5)	189·5 (169·8 to 211·3)	1·8 (1·3 to 2·6)	12·5 (12·5 to 12·5)
Moldova*	2005	165·3 (153·7 to 177·5)	86·6 (77·4 to 96·7)	73·5 (67·0 to 80·2)	0·4 (0·2 to 0·5)	4·8 (4·8 to 4·8)	513·2 (477·2 to 551·1)	269·0 (240·5 to 300·3)	228·1 (207·9 to 249·0)	1·2 (0·8 to 1·6)	14·9 (14·9 to 14·9)
Moldova*	2006	189·5 (177·1 to 201·7)	98·5 (88·3 to 110·1)	87·8 (81·3 to 94·4)	0·3 (0·2 to 0·5)	2·8 (2·8 to 2·8)	588·5 (549·9 to 626·4)	306·0 (274·1 to 341·9)	272·7 (252·4 to 293·0)	1·0 (0·7 to 1·5)	8·8 (8·8 to 8·8)
Moldova*	2007	214·0 (200·1 to 226·9)	108·3 (97·0 to 120·2)	100·2 (93·8 to 106·9)	0·4 (0·3 to 0·5)	5·1 (5·1 to 5·1)	664·4 (621·3 to 704·6)	336·3 (301·3 to 373·4)	311·1 (291·2 to 331·9)	1·2 (0·8 to 1·7)	15·8 (15·8 to 15·8)
Moldova*	2008	228·0 (214·3 to 241·5)	112·6 (101·4 to 124·4)	110·0 (103·7 to 116·3)	0·5 (0·3 to 0·7)	4·8 (4·8 to 4·8)	708·1 (665·3 to 750·1)	349·8 (314·7 to 386·2)	341·7 (322·2 to 361·1)	1·6 (1·1 to 2·3)	15·0 (15·0 to 15·0)

Moldova*	2009	240·5 (227·6 to 255·0)	114·2 (103·1 to 125·8)	112·8 (106·4 to 119·4)	1·5 (1·0 to 2·1)	12·1 (12·1 to 12·1)	747·0 (706·8 to 791·8)	354·5 (320·2 to 390·7)	350·2 (330·4 to 370·7)	4·6 (3·1 to 6·5)	37·7 (37·7 to 37·7)
Moldova*	2010	219·4 (207·8 to 231·4)	102·7 (93·2 to 112·8)	100·4 (94·5 to 106·4)	2·3 (1·5 to 3·3)	14·0 (14·0 to 14·0)	681·4 (645·4 to 718·4)	318·9 (289·4 to 350·3)	311·9 (293·6 to 330·3)	7·1 (4·8 to 10·3)	43·5 (43·5 to 43·5)
Moldova*	2011	208·5 (198·1 to 219·0)	101·1 (92·7 to 110·2)	95·2 (89·3 to 101·9)	2·4 (1·6 to 3·4)	9·9 (9·9 to 9·9)	647·6 (615·0 to 680·1)	314·0 (287·8 to 342·1)	295·5 (277·4 to 316·5)	7·3 (5·1 to 10·5)	30·7 (30·7 to 30·7)
Moldova*	2012	206·4 (197·7 to 216·0)	101·7 (94·7 to 109·0)	91·9 (86·5 to 98·7)	2·3 (1·6 to 3·3)	10·4 (10·4 to 10·4)	640·8 (613·8 to 670·9)	315·9 (294·2 to 338·6)	285·3 (268·6 to 306·4)	7·2 (4·9 to 10·3)	32·4 (32·4 to 32·4)
Moldova*	2013	207·7 (198·9 to 216·9)	103·8 (97·5 to 110·5)	92·4 (86·8 to 98·6)	2·1 (1·4 to 3·0)	9·5 (9·5 to 9·5)	645·1 (617·8 to 673·6)	322·5 (302·9 to 343·1)	286·8 (269·5 to 306·2)	6·5 (4·4 to 9·3)	29·3 (29·3 to 29·3)
Moldova*	2014	220·0 (211·3 to 229·1)	109·4 (103·5 to 115·7)	95·2 (89·8 to 101·7)	2·2 (1·5 to 3·1)	13·2 (13·2 to 13·2)	683·3 (656·1 to 711·6)	339·7 (321·4 to 359·2)	295·7 (278·7 to 315·9)	6·8 (4·7 to 9·5)	41·0 (41·0 to 41·0)
Moldova*	2015	219·9 (211·1 to 228·8)	105·7 (99·1 to 112·7)	97·4 (91·7 to 103·8)	2·2 (1·5 to 3·2)	14·5 (14·5 to 14·5)	682·9 (655·5 to 710·4)	328·2 (307·7 to 350·0)	302·6 (284·8 to 322·2)	7·0 (4·8 to 9·8)	45·1 (45·1 to 45·1)
Moldova*	2016	209·2 (198·9 to 219·0)	104·7 (96·9 to 112·9)	95·0 (89·9 to 100·8)	2·3 (1·6 to 3·3)	7·2 (7·2 to 7·2)	649·7 (617·6 to 680·0)	325·1 (300·9 to 350·5)	295·0 (279·2 to 313·0)	7·1 (4·8 to 10·2)	22·4 (22·4 to 22·4)
Moldova*	2017	208·5 (196·7 to 220·4)	109·2 (99·0 to 120·2)	91·1 (85·8 to 97·0)	2·1 (1·4 to 3·0)	6·0 (6·0 to 6·0)	647·4 (610·9 to 684·4)	339·2 (307·4 to 373·4)	283·0 (266·6 to 301·1)	6·6 (4·5 to 9·3)	18·6 (18·6 to 18·6)
Moldova*	2018	210·1 (194·5 to 226·1)	116·6 (103·2 to 130·3)	87·8 (81·1 to 94·8)	1·8 (1·2 to 2·6)	3·9 (3·9 to 3·9)	652·4 (604·0 to 702·1)	362·2 (320·5 to 404·7)	272·5 (251·8 to 294·5)	5·6 (3·8 to 8·0)	12·2 (12·2 to 12·2)
Moldova*	2019	214·8 (198·4 to 231·9)	119·8 (106·0 to 134·6)	89·2 (82·4 to 96·4)	1·9 (1·3 to 2·7)	3·9 (3·9 to 4·0)	667·1 (616·2 to 720·2)	372·0 (329·2 to 417·8)	277·0 (256·0 to 299·4)	5·8 (4·0 to 8·3)	12·2 (12·1 to 12·3)
Moldova*	2020	240·7 (224·4 to 257·9)	147·3 (133·5 to 162·1)	86·6 (80·0 to 93·6)	1·9 (1·3 to 2·7)	5·0 (4·9 to 5·2)	747·6 (696·7 to 800·8)	457·5 (414·5 to 503·4)	268·8 (248·4 to 290·8)	5·8 (4·0 to 8·2)	15·5 (15·3 to 16·2)
Moldova*	2021	235·2 (215·9 to 255·7)	141·9 (125·2 to 160·1)	87·0 (80·3 to 94·1)	1·9 (1·3 to 2·7)	4·4 (4·3 to 4·6)	730·4 (670·5 to 793·9)	440·6 (388·9 to 497·2)	270·2 (249·4 to 292·3)	5·9 (4·1 to 8·4)	13·6 (13·3 to 14·4)
Moldova*	2022	214·6 (197·0 to 233·2)	122·2 (106·8 to 139·2)	86·8 (80·1 to 94·1)	1·9 (1·3 to 2·8)	3·6 (3·4 to 3·8)	666·4 (611·7 to 724·1)	379·6 (331·5 to 432·1)	269·6 (248·8 to 292·1)	6·0 (4·1 to 8·6)	11·1 (10·5 to 11·9)

Moldova*	2023	216·6 (198·5 to 235·6)	124·0 (108·5 to 140·9)	86·7 (79·8 to 93·9)	2·0 (1·4 to 2·8)	3·9 (3·6 to 4·2)	672·5 (616·5 to 731·6)	385·2 (336·8 to 437·5)	269·1 (247·9 to 291·7)	6·2 (4·2 to 8·7)	12·1 (11·3 to 13·1)
Moldova*	2024	218·9 (200·4 to 238·2)	126·1 (110·3 to 144·1)	86·5 (79·6 to 93·9)	2·0 (1·4 to 2·9)	4·2 (3·9 to 4·6)	679·8 (622·3 to 739·6)	391·7 (342·4 to 447·5)	268·6 (247·3 to 291·6)	6·3 (4·3 to 8·9)	13·1 (12·1 to 14·3)
Moldova*	2025	220·8 (201·5 to 241·1)	128·0 (111·0 to 146·6)	86·2 (79·4 to 93·6)	2·1 (1·4 to 2·9)	4·6 (4·2 to 5·0)	685·7 (625·8 to 748·7)	397·3 (344·6 to 455·2)	267·8 (246·6 to 290·6)	6·4 (4·4 to 9·1)	14·2 (12·9 to 15·5)
Moldova*	2026	221·9 (202·2 to 242·5)	128·9 (111·5 to 147·4)	86·0 (79·1 to 93·4)	2·1 (1·4 to 3·0)	4·9 (4·5 to 5·4)	689·0 (627·8 to 753·1)	400·2 (346·2 to 457·6)	267·0 (245·7 to 290·0)	6·6 (4·5 to 9·3)	15·2 (13·9 to 16·8)
Moldova*	2027	222·7 (203·2 to 243·7)	129·4 (111·7 to 148·2)	85·9 (79·1 to 93·3)	2·2 (1·5 to 3·1)	5·2 (4·7 to 5·8)	691·5 (631·1 to 756·7)	401·7 (346·9 to 460·1)	266·8 (245·7 to 289·6)	6·7 (4·6 to 9·5)	16·2 (14·7 to 18·1)
Moldova*	2028	223·9 (204·2 to 245·2)	130·1 (112·5 to 150·4)	86·0 (79·2 to 93·3)	2·2 (1·5 to 3·2)	5·5 (5·0 to 6·3)	695·3 (634·2 to 761·4)	404·1 (349·3 to 466·9)	267·1 (245·9 to 289·7)	6·9 (4·7 to 9·8)	17·1 (15·5 to 19·4)
Moldova*	2029	225·6 (205·0 to 247·3)	131·2 (113·0 to 151·2)	86·2 (79·4 to 93·5)	2·3 (1·5 to 3·2)	5·8 (5·2 to 6·7)	700·5 (636·5 to 767·9)	407·6 (351·0 to 469·5)	267·7 (246·4 to 290·4)	7·1 (4·8 to 10·0)	18·1 (16·3 to 20·8)
Moldova*	2030	227·4 (206·7 to 249·9)	132·5 (113·6 to 153·4)	86·5 (79·5 to 93·8)	2·3 (1·6 to 3·3)	6·1 (5·5 to 7·2)	706·3 (642·0 to 775·9)	411·5 (352·8 to 476·3)	268·5 (247·0 to 291·2)	7·3 (4·9 to 10·3)	19·0 (17·0 to 22·2)
Moldova*	2031	229·2 (207·8 to 252·0)	133·7 (113·9 to 154·7)	86·6 (79·7 to 93·9)	2·4 (1·6 to 3·4)	6·4 (5·7 to 7·6)	711·6 (645·3 to 782·7)	415·2 (353·7 to 480·4)	268·9 (247·4 to 291·5)	7·5 (5·1 to 10·5)	20·0 (17·8 to 23·7)
Moldova*	2032	230·8 (209·2 to 254·0)	134·8 (114·6 to 156·6)	86·7 (79·8 to 94·0)	2·5 (1·7 to 3·5)	6·8 (6·0 to 8·1)	716·8 (649·7 to 788·7)	418·7 (355·9 to 486·2)	269·3 (247·8 to 291·9)	7·7 (5·2 to 10·8)	21·1 (18·6 to 25·2)
Moldova*	2033	232·4 (210·2 to 255·8)	135·8 (115·1 to 158·0)	87·0 (80·0 to 94·2)	2·5 (1·7 to 3·6)	7·1 (6·3 to 8·7)	721·7 (652·9 to 794·4)	421·8 (357·3 to 490·5)	270·0 (248·5 to 292·6)	7·9 (5·3 to 11·1)	22·1 (19·4 to 26·9)
Moldova*	2034	234·1 (211·6 to 257·6)	136·9 (115·9 to 158·5)	87·2 (80·2 to 94·4)	2·6 (1·8 to 3·7)	7·5 (6·5 to 9·3)	727·1 (657·0 to 799·8)	425·1 (359·9 to 492·3)	270·6 (249·0 to 293·2)	8·1 (5·5 to 11·5)	23·3 (20·3 to 28·8)
Moldova*	2035	236·0 (213·0 to 260·1)	138·1 (116·6 to 160·5)	87·3 (80·3 to 94·6)	2·7 (1·8 to 3·8)	7·9 (6·8 to 9·9)	732·7 (661·4 to 807·6)	428·7 (362·0 to 498·3)	271·2 (249·5 to 293·7)	8·3 (5·6 to 11·8)	24·5 (21·3 to 30·7)
Moldova*	2036	237·7 (214·7 to 262·6)	139·2 (116·3 to 162·9)	87·5 (80·5 to 94·8)	2·7 (1·8 to 3·9)	8·3 (7·2 to 10·5)	738·3 (666·6 to 815·5)	432·2 (361·3 to 505·9)	271·8 (250·0 to 294·3)	8·5 (5·7 to 12·1)	25·8 (22·3 to 32·7)

Moldova*	2037	239·3 (215·2 to 264·7)	140·1 (117·5 to 164·5)	87·6 (80·6 to 94·8)	2·8 (1·9 to 4·0)	8·8 (7·6 to 11·3)	743·2 (668·4 to 822·0)	435·2 (364·8 to 511·0)	272·0 (250·2 to 294·4)	8·7 (5·9 to 12·4)	27·3 (23·6 to 35·1)
Moldova*	2038	240·5 (215·8 to 266·6)	140·8 (118·1 to 165·6)	87·5 (80·5 to 94·7)	2·9 (1·9 to 4·1)	9·4 (8·1 to 12·3)	746·9 (670·3 to 827·9)	437·2 (366·7 to 514·3)	271·7 (249·9 to 294·1)	8·9 (6·0 to 12·7)	29·2 (25·1 to 38·2)
Moldova*	2039	241·5 (216·7 to 268·7)	141·2 (117·5 to 165·9)	87·3 (80·3 to 94·5)	2·9 (2·0 to 4·2)	10·1 (8·6 to 13·3)	750·0 (672·9 to 834·3)	438·6 (364·9 to 515·2)	271·1 (249·4 to 293·6)	9·1 (6·1 to 13·0)	31·2 (26·7 to 41·4)
Moldova*	2040	242·8 (217·7 to 270·2)	141·9 (118·5 to 167·0)	87·1 (80·2 to 94·4)	3·0 (2·0 to 4·3)	10·8 (9·2 to 14·4)	753·9 (676·0 to 839·0)	440·6 (368·1 to 518·7)	270·6 (249·1 to 293·1)	9·3 (6·2 to 13·3)	33·5 (28·5 to 44·9)
Moldova*	2041	243·7 (218·3 to 272·1)	142·2 (118·4 to 168·0)	86·9 (80·0 to 94·1)	3·0 (2·0 to 4·4)	11·6 (9·8 to 15·8)	756·9 (677·8 to 845·1)	441·7 (367·6 to 521·6)	269·8 (248·4 to 292·3)	9·5 (6·3 to 13·6)	36·0 (30·3 to 49·1)
Moldova*	2042	244·8 (218·6 to 273·6)	142·6 (118·2 to 168·7)	86·6 (79·8 to 93·9)	3·1 (2·1 to 4·5)	12·5 (10·4 to 17·2)	760·2 (679·0 to 849·6)	442·8 (366·9 to 523·9)	269·0 (247·7 to 291·4)	9·7 (6·5 to 14·0)	38·7 (32·4 to 53·5)
Moldova*	2043	245·9 (219·7 to 275·3)	142·9 (118·3 to 169·2)	86·3 (79·5 to 93·5)	3·2 (2·1 to 4·6)	13·5 (11·1 to 19·0)	763·5 (682·4 to 854·9)	443·9 (367·4 to 525·3)	268·0 (246·7 to 290·4)	9·9 (6·6 to 14·3)	41·8 (34·5 to 58·9)
Moldova*	2044	246·9 (220·3 to 276·6)	143·1 (117·7 to 170·1)	85·9 (79·1 to 93·1)	3·2 (2·2 to 4·7)	14·6 (12·1 to 20·9)	766·7 (684·2 to 859·0)	444·5 (365·5 to 528·3)	266·9 (245·7 to 289·1)	10·0 (6·7 to 14·6)	45·2 (37·6 to 64·8)
Moldova*	2045	247·7 (221·0 to 278·1)	143·1 (117·9 to 171·0)	85·5 (78·7 to 92·6)	3·3 (2·2 to 4·8)	15·8 (13·0 to 22·9)	769·3 (686·4 to 863·7)	444·5 (366·0 to 531·1)	265·4 (244·3 to 287·4)	10·2 (6·8 to 15·0)	49·1 (40·4 to 71·1)
Moldova*	2046	248·7 (221·7 to 279·6)	143·1 (117·6 to 171·3)	85·0 (78·3 to 92·0)	3·4 (2·2 to 4·9)	17·2 (14·1 to 25·5)	772·2 (688·4 to 868·4)	444·4 (365·3 to 532·0)	263·9 (243·1 to 285·7)	10·4 (6·9 to 15·4)	53·5 (43·8 to 79·1)
Moldova*	2047	249·7 (223·1 to 281·3)	143·1 (117·9 to 171·7)	84·4 (77·8 to 91·4)	3·4 (2·3 to 5·1)	18·8 (15·2 to 28·3)	775·5 (692·8 to 873·6)	444·4 (366·2 to 533·0)	262·2 (241·7 to 283·9)	10·6 (7·0 to 15·7)	58·3 (47·3 to 88·0)
Moldova*	2048	250·5 (223·5 to 282·6)	142·7 (117·0 to 171·7)	83·7 (77·1 to 90·6)	3·5 (2·3 to 5·1)	20·6 (16·7 to 31·6)	777·8 (694·1 to 877·6)	443·0 (363·5 to 533·2)	259·9 (239·5 to 281·3)	10·7 (7·0 to 15·9)	64·1 (51·7 to 98·0)
Moldova*	2049	251·2 (224·1 to 282·9)	142·0 (116·6 to 170·4)	82·8 (76·3 to 89·7)	3·5 (2·3 to 5·2)	22·9 (18·3 to 35·7)	779·9 (696·0 to 878·5)	440·9 (362·2 to 529·2)	257·1 (236·9 to 278·6)	10·9 (7·0 to 16·2)	71·0 (56·8 to 110·8)
Moldova*	2050	252·1 (224·8 to 284·7)	141·3 (115·5 to 171·6)	81·9 (75·5 to 88·7)	3·5 (2·3 to 5·3)	25·3 (20·2 to 40·7)	782·8 (698·0 to 884·1)	438·8 (358·7 to 532·8)	254·3 (234·4 to 275·5)	11·0 (7·1 to 16·3)	78·7 (62·6 to 126·4)

Madagascar*	1995	22·3 (19·6 to 25·2)	8·7 (7·2 to 10·6)	9·0 (7·1 to 11·2)	1·8 (1·2 to 2·6)	2·7 (2·7 to 2·7)	71·2 (62·6 to 80·6)	27·9 (23·0 to 33·9)	28·9 (22·8 to 35·9)	5·8 (3·9 to 8·4)	8·6 (8·6 to 8·6)
Madagascar*	1996	22·0 (19·2 to 24·8)	8·6 (7·0 to 10·5)	8·8 (6·9 to 10·9)	1·8 (1·2 to 2·6)	2·8 (2·8 to 2·8)	70·3 (61·5 to 79·4)	27·4 (22·5 to 33·6)	28·1 (22·2 to 34·8)	5·7 (3·8 to 8·3)	9·1 (9·1 to 9·1)
Madagascar*	1997	21·4 (18·7 to 24·3)	8·7 (7·1 to 10·6)	8·7 (6·9 to 10·8)	1·8 (1·2 to 2·5)	2·2 (2·2 to 2·2)	68·3 (59·9 to 77·7)	27·7 (22·7 to 33·8)	27·8 (22·2 to 34·5)	5·6 (3·8 to 8·0)	7·2 (7·2 to 7·2)
Madagascar*	1998	23·3 (20·6 to 26·3)	9·2 (7·5 to 11·2)	8·7 (7·0 to 10·9)	1·8 (1·2 to 2·6)	3·6 (3·6 to 3·6)	74·7 (65·8 to 84·2)	29·5 (24·0 to 35·8)	28·0 (22·4 to 34·9)	5·7 (3·9 to 8·2)	11·5 (11·5 to 11·5)
Madagascar*	1999	21·4 (18·7 to 24·2)	9·1 (7·4 to 11·0)	8·6 (6·9 to 10·8)	1·8 (1·2 to 2·5)	2·0 (2·0 to 2·0)	68·4 (60·0 to 77·4)	29·1 (23·7 to 35·3)	27·4 (22·0 to 34·6)	5·6 (3·8 to 8·1)	6·2 (6·2 to 6·2)
Madagascar*	2000	21·6 (18·9 to 24·5)	9·4 (7·6 to 11·4)	8·5 (6·9 to 10·8)	1·8 (1·2 to 2·5)	1·8 (1·8 to 1·8)	69·0 (60·4 to 78·4)	30·1 (24·4 to 36·3)	27·3 (22·0 to 34·4)	5·7 (3·8 to 8·0)	5·9 (5·9 to 5·9)
Madagascar*	2001	22·5 (19·8 to 25·5)	9·9 (8·1 to 12·0)	8·5 (6·9 to 10·6)	1·8 (1·2 to 2·5)	2·3 (2·3 to 2·3)	71·9 (63·4 to 81·5)	31·5 (25·8 to 38·3)	27·2 (21·9 to 33·8)	5·7 (3·8 to 8·1)	7·5 (7·5 to 7·5)
Madagascar*	2002	19·4 (17·2 to 22·0)	8·0 (6·5 to 9·7)	7·6 (6·1 to 9·5)	1·6 (1·1 to 2·2)	2·2 (2·2 to 2·2)	62·1 (55·2 to 70·3)	25·5 (20·6 to 31·0)	24·5 (19·6 to 30·3)	5·0 (3·4 to 7·0)	7·2 (7·2 to 7·2)
Madagascar*	2003	23·3 (20·8 to 26·3)	9·7 (7·8 to 11·7)	8·3 (6·6 to 10·1)	1·7 (1·2 to 2·4)	3·7 (3·7 to 3·7)	74·7 (66·7 to 84·1)	31·1 (25·0 to 37·5)	26·4 (21·1 to 32·4)	5·5 (3·7 to 7·8)	11·7 (11·7 to 11·7)
Madagascar*	2004	26·5 (23·8 to 29·7)	11·2 (9·0 to 13·7)	8·7 (6·8 to 10·9)	1·8 (1·2 to 2·6)	4·8 (4·8 to 4·8)	84·9 (76·0 to 95·1)	35·9 (28·9 to 43·8)	27·9 (21·9 to 34·8)	5·9 (4·0 to 8·4)	15·2 (15·2 to 15·2)
Madagascar*	2005	25·2 (22·4 to 28·4)	10·0 (8·1 to 12·1)	8·4 (6·5 to 10·5)	1·8 (1·2 to 2·5)	5·0 (5·0 to 5·0)	80·6 (71·8 to 90·8)	32·0 (25·9 to 38·6)	27·0 (20·9 to 33·5)	5·7 (3·9 to 8·1)	16·0 (16·0 to 16·0)
Madagascar*	2006	24·1 (21·3 to 27·3)	10·0 (8·1 to 12·2)	8·5 (6·6 to 10·5)	1·8 (1·3 to 2·7)	3·8 (3·8 to 3·8)	77·2 (68·1 to 87·2)	32·1 (26·0 to 39·0)	27·2 (21·1 to 33·5)	5·9 (4·0 to 8·5)	12·0 (12·0 to 12·0)
Madagascar*	2007	23·8 (21·1 to 26·6)	9·3 (7·5 to 11·3)	8·3 (6·5 to 10·2)	1·9 (1·3 to 2·6)	4·3 (4·3 to 4·3)	76·1 (67·6 to 85·0)	29·7 (24·1 to 36·2)	26·6 (20·8 to 32·6)	6·0 (4·0 to 8·5)	13·9 (13·9 to 13·9)
Madagascar*	2008	24·0 (21·4 to 26·7)	9·0 (7·3 to 10·9)	8·4 (6·6 to 10·3)	2·0 (1·3 to 2·8)	4·7 (4·7 to 4·7)	76·9 (68·4 to 85·4)	28·7 (23·2 to 35·0)	26·9 (21·2 to 33·0)	6·3 (4·3 to 8·9)	15·0 (15·0 to 15·0)
Madagascar*	2009	20·4 (18·1 to 22·9)	7·5 (6·1 to 9·1)	7·8 (6·2 to 9·5)	1·8 (1·2 to 2·5)	3·3 (3·3 to 3·3)	65·4 (58·1 to 73·1)	24·0 (19·4 to 29·2)	24·9 (19·8 to 30·5)	5·8 (4·0 to 8·0)	10·7 (10·7 to 10·7)
Madagascar*	2010	22·3 (19·9 to 24·8)	7·9 (6·4 to 9·6)	7·6 (6·0 to 9·3)	1·7 (1·2 to 2·4)	5·1 (5·1 to 5·1)	71·4 (63·8 to 79·4)	25·4 (20·5 to 30·7)	24·2 (19·2 to 29·7)	5·6 (3·8 to 7·7)	16·3 (16·3 to 16·3)
Madagascar*	2011	19·4 (17·2 to 21·9)	7·9 (6·5 to 9·6)	6·9 (5·5 to 8·5)	1·6 (1·1 to 2·3)	3·0 (3·0 to 3·0)	62·2 (55·1 to 70·0)	25·4 (20·7 to 30·6)	22·1 (17·6 to 27·3)	5·2 (3·6 to 7·2)	9·5 (9·5 to 9·5)
Madagascar*	2012	19·2 (17·1 to 21·5)	7·6 (6·2 to 9·2)	6·4 (5·1 to 8·0)	1·6 (1·1 to 2·2)	3·6 (3·6 to 3·6)	61·5 (54·8 to 68·7)	24·5 (19·9 to 29·5)	20·6 (16·4 to 25·7)	5·0 (3·4 to 6·9)	11·5 (11·5 to 11·5)

Madagascar*	2013	20·0 (17·8 to 22·4)	8·1 (6·5 to 9·7)	6·3 (5·0 to 7·9)	1·6 (1·1 to 2·2)	4·1 (4·1 to 4·1)	64·0 (57·1 to 71·6)	25·8 (20·9 to 31·1)	20·1 (15·9 to 25·1)	5·1 (3·5 to 7·0)	13·0 (13·0 to 13·0)
Madagascar*	2014	19·8 (17·5 to 22·2)	8·6 (7·0 to 10·4)	6·1 (4·8 to 7·7)	1·5 (1·1 to 2·1)	3·5 (3·5 to 3·5)	63·2 (56·0 to 71·1)	27·6 (22·3 to 33·3)	19·6 (15·4 to 24·7)	4·9 (3·4 to 6·9)	11·1 (11·1 to 11·1)
Madagascar*	2015	20·2 (17·7 to 22·7)	9·4 (7·6 to 11·4)	6·1 (4·8 to 7·7)	1·5 (1·0 to 2·1)	3·2 (3·2 to 3·2)	64·7 (56·8 to 72·6)	30·1 (24·3 to 36·3)	19·5 (15·2 to 24·6)	4·8 (3·3 to 6·8)	10·3 (10·3 to 10·3)
Madagascar*	2016	21·1 (18·5 to 23·7)	10·3 (8·3 to 12·3)	6·1 (4·8 to 7·7)	1·4 (1·0 to 2·0)	3·4 (3·4 to 3·4)	67·6 (59·3 to 75·8)	32·8 (26·6 to 39·5)	19·5 (15·3 to 24·7)	4·5 (3·1 to 6·4)	10·8 (10·8 to 10·8)
Madagascar*	2017	21·1 (18·4 to 23·9)	10·7 (8·7 to 12·8)	6·2 (4·9 to 7·9)	1·4 (1·0 to 2·0)	2·9 (2·9 to 2·9)	67·6 (58·9 to 76·3)	34·1 (27·7 to 41·0)	19·8 (15·5 to 25·1)	4·6 (3·1 to 6·3)	9·1 (9·1 to 9·1)
Madagascar*	2018	21·2 (18·7 to 23·8)	9·6 (7·9 to 11·6)	6·1 (4·8 to 7·8)	1·4 (0·9 to 2·0)	4·0 (4·0 to 4·0)	67·7 (59·8 to 76·2)	30·9 (25·1 to 37·2)	19·5 (15·3 to 24·9)	4·4 (3·0 to 6·2)	12·9 (12·9 to 12·9)
Madagascar*	2019	21·8 (19·2 to 24·6)	10·1 (8·2 to 12·3)	6·2 (4·9 to 7·9)	1·4 (1·0 to 2·0)	4·0 (4·0 to 4·0)	69·7 (61·4 to 78·6)	32·4 (26·3 to 39·5)	19·8 (15·6 to 25·3)	4·6 (3·1 to 6·5)	12·8 (12·8 to 12·9)
Madagascar*	2020	26·1 (23·6 to 28·8)	13·4 (11·6 to 15·6)	5·9 (4·6 to 7·5)	1·4 (1·0 to 2·0)	5·4 (5·3 to 5·5)	83·5 (75·5 to 92·1)	43·0 (37·0 to 49·9)	18·7 (14·7 to 23·9)	4·5 (3·0 to 6·3)	17·3 (17·1 to 17·6)
Madagascar*	2021	23·0 (20·4 to 25·7)	10·5 (8·5 to 12·8)	5·5 (4·3 to 7·1)	1·3 (0·9 to 1·9)	5·6 (5·5 to 5·9)	73·5 (65·4 to 82·3)	33·5 (27·1 to 40·9)	17·7 (13·9 to 22·6)	4·3 (2·9 to 6·1)	18·0 (17·7 to 18·8)
Madagascar*	2022	20·4 (18·0 to 23·1)	9·2 (7·3 to 11·2)	5·5 (4·3 to 7·1)	1·4 (0·9 to 1·9)	4·4 (4·1 to 4·7)	65·3 (57·5 to 73·9)	29·3 (23·3 to 35·9)	17·7 (13·9 to 22·6)	4·4 (3·0 to 6·2)	13·9 (13·1 to 15·0)
Madagascar*	2023	20·7 (18·2 to 23·4)	9·3 (7·3 to 11·3)	5·6 (4·4 to 7·1)	1·4 (1·0 to 2·0)	4·5 (4·2 to 4·8)	66·1 (58·1 to 74·8)	29·6 (23·2 to 36·2)	17·8 (14·0 to 22·7)	4·5 (3·1 to 6·4)	14·2 (13·3 to 15·5)
Madagascar*	2024	20·9 (18·4 to 23·8)	9·3 (7·4 to 11·5)	5·6 (4·4 to 7·1)	1·5 (1·0 to 2·0)	4·5 (4·2 to 5·0)	66·9 (58·9 to 76·0)	29·9 (23·6 to 36·8)	17·8 (14·0 to 22·8)	4·6 (3·1 to 6·5)	14·5 (13·5 to 16·0)
Madagascar*	2025	21·2 (18·6 to 24·0)	9·4 (7·5 to 11·6)	5·6 (4·4 to 7·1)	1·5 (1·0 to 2·1)	4·6 (4·3 to 5·1)	67·7 (59·6 to 76·8)	30·2 (23·9 to 37·2)	17·9 (14·1 to 22·8)	4·8 (3·2 to 6·7)	14·9 (13·7 to 16·4)
Madagascar*	2026	21·4 (18·8 to 24·3)	9·6 (7·6 to 11·8)	5·6 (4·4 to 7·2)	1·5 (1·0 to 2·2)	4·7 (4·3 to 5·3)	68·6 (60·3 to 77·7)	30·6 (24·2 to 37·6)	18·0 (14·1 to 22·9)	4·9 (3·3 to 6·9)	15·1 (13·8 to 16·9)
Madagascar*	2027	21·7 (19·1 to 24·6)	9·6 (7·6 to 11·9)	5·6 (4·4 to 7·2)	1·6 (1·1 to 2·2)	4·8 (4·4 to 5·4)	69·3 (61·1 to 78·6)	30·8 (24·4 to 38·0)	18·0 (14·1 to 22·9)	5·0 (3·4 to 7·1)	15·5 (14·1 to 17·4)
Madagascar*	2028	21·9 (19·3 to 24·9)	9·8 (7·7 to 12·0)	5·6 (4·4 to 7·2)	1·6 (1·1 to 2·3)	5·0 (4·5 to 5·6)	70·2 (61·7 to 79·5)	31·2 (24·6 to 38·4)	18·0 (14·1 to 23·0)	5·2 (3·5 to 7·3)	15·8 (14·3 to 18·0)
Madagascar*	2029	22·2 (19·6 to 25·2)	9·9 (7·8 to 12·2)	5·6 (4·4 to 7·2)	1·7 (1·1 to 2·3)	5·1 (4·5 to 5·8)	71·2 (62·8 to 80·6)	31·7 (25·0 to 39·2)	18·0 (14·1 to 23·0)	5·3 (3·6 to 7·5)	16·2 (14·5 to 18·6)
Madagascar*	2030	22·5 (19·9 to 25·6)	10·1 (7·9 to 12·4)	5·7 (4·4 to 7·2)	1·7 (1·2 to 2·4)	5·1 (4·6 to 6·0)	72·1 (63·5 to 81·9)	32·2 (25·3 to 39·6)	18·1 (14·2 to 23·1)	5·4 (3·7 to 7·7)	16·4 (14·7 to 19·0)

Madagascar*	2031	22·9 (20·1 to 25·9)	10·2 (8·0 to 12·6)	5·7 (4·4 to 7·2)	1·7 (1·2 to 2·5)	5·2 (4·6 to 6·1)	73·1 (64·4 to 82·8)	32·7 (25·7 to 40·4)	18·1 (14·2 to 23·1)	5·6 (3·8 to 7·9)	16·7 (14·9 to 19·7)
Madagascar*	2032	23·2 (20·4 to 26·2)	10·4 (8·2 to 12·8)	5·7 (4·5 to 7·2)	1·8 (1·2 to 2·5)	5·3 (4·7 to 6·3)	74·2 (65·2 to 83·8)	33·2 (26·2 to 41·0)	18·2 (14·2 to 23·2)	5·7 (3·9 to 8·1)	17·0 (15·0 to 20·1)
Madagascar*	2033	23·5 (20·6 to 26·5)	10·6 (8·3 to 13·0)	5·7 (4·5 to 7·3)	1·8 (1·2 to 2·6)	5·4 (4·7 to 6·4)	75·2 (66·0 to 84·9)	33·8 (26·5 to 41·7)	18·2 (14·3 to 23·2)	5·9 (4·0 to 8·3)	17·3 (15·2 to 20·5)
Madagascar*	2034	23·8 (20·8 to 26·9)	10·7 (8·5 to 13·3)	5·7 (4·5 to 7·3)	1·9 (1·3 to 2·7)	5·5 (4·8 to 6·6)	76·2 (66·5 to 86·1)	34·3 (27·1 to 42·5)	18·3 (14·3 to 23·3)	6·0 (4·0 to 8·6)	17·6 (15·4 to 21·0)
Madagascar*	2035	24·2 (21·1 to 27·3)	10·9 (8·6 to 13·5)	5·7 (4·5 to 7·3)	1·9 (1·3 to 2·8)	5·6 (4·8 to 6·7)	77·3 (67·5 to 87·3)	34·9 (27·5 to 43·3)	18·4 (14·4 to 23·4)	6·2 (4·2 to 8·8)	17·9 (15·5 to 21·4)
Madagascar*	2036	24·5 (21·4 to 27·8)	11·1 (8·8 to 13·7)	5·8 (4·5 to 7·3)	2·0 (1·3 to 2·8)	5·7 (4·9 to 6·9)	78·4 (68·5 to 88·8)	35·5 (28·1 to 44·0)	18·4 (14·5 to 23·5)	6·3 (4·3 to 9·1)	18·2 (15·7 to 22·0)
Madagascar*	2037	24·9 (21·7 to 28·3)	11·3 (8·9 to 14·0)	5·8 (4·5 to 7·4)	2·0 (1·4 to 2·9)	5·8 (5·0 to 7·0)	79·5 (69·3 to 90·4)	36·1 (28·5 to 44·8)	18·5 (14·5 to 23·6)	6·5 (4·4 to 9·4)	18·5 (16·0 to 22·5)
Madagascar*	2038	25·2 (22·0 to 28·6)	11·5 (9·0 to 14·3)	5·8 (4·6 to 7·4)	2·1 (1·4 to 3·0)	5·9 (5·0 to 7·2)	80·7 (70·4 to 91·6)	36·7 (28·9 to 45·6)	18·6 (14·6 to 23·7)	6·7 (4·5 to 9·7)	18·7 (16·1 to 23·0)
Madagascar*	2039	25·6 (22·3 to 29·1)	11·7 (9·2 to 14·6)	5·8 (4·6 to 7·5)	2·2 (1·4 to 3·1)	5·9 (5·1 to 7·3)	81·9 (71·3 to 93·1)	37·4 (29·3 to 46·6)	18·7 (14·7 to 23·8)	6·9 (4·6 to 10·0)	19·0 (16·2 to 23·2)
Madagascar*	2040	26·0 (22·6 to 29·6)	11·9 (9·4 to 14·9)	5·9 (4·6 to 7·5)	2·2 (1·5 to 3·2)	6·0 (5·1 to 7·4)	83·2 (72·3 to 94·6)	38·1 (30·0 to 47·5)	18·8 (14·7 to 24·0)	7·1 (4·7 to 10·3)	19·2 (16·2 to 23·7)
Madagascar*	2041	26·4 (23·0 to 30·1)	12·2 (9·5 to 15·1)	5·9 (4·6 to 7·5)	2·3 (1·5 to 3·3)	6·1 (5·1 to 7·5)	84·5 (73·6 to 96·3)	38·9 (30·5 to 48·5)	18·9 (14·8 to 24·1)	7·3 (4·8 to 10·6)	19·4 (16·4 to 24·0)
Madagascar*	2042	26·8 (23·3 to 30·6)	12·4 (9·7 to 15·4)	5·9 (4·7 to 7·6)	2·4 (1·6 to 3·4)	6·1 (5·2 to 7·6)	85·9 (74·6 to 97·9)	39·7 (31·1 to 49·4)	19·0 (14·9 to 24·3)	7·6 (5·0 to 11·0)	19·6 (16·5 to 24·4)
Madagascar*	2043	27·3 (23·8 to 31·3)	12·7 (10·0 to 15·9)	6·0 (4·7 to 7·6)	2·4 (1·6 to 3·6)	6·2 (5·2 to 7·7)	87·3 (76·0 to 100·2)	40·6 (31·9 to 50·8)	19·1 (15·0 to 24·4)	7·8 (5·1 to 11·4)	19·8 (16·5 to 24·8)
Madagascar*	2044	27·7 (24·1 to 31·9)	13·0 (10·1 to 16·2)	6·0 (4·7 to 7·7)	2·5 (1·6 to 3·7)	6·3 (5·2 to 7·9)	88·8 (77·1 to 101·9)	41·5 (32·5 to 51·9)	19·2 (15·1 to 24·6)	8·0 (5·2 to 11·8)	20·0 (16·7 to 25·1)
Madagascar*	2045	28·2 (24·4 to 32·3)	13·3 (10·3 to 16·6)	6·1 (4·8 to 7·7)	2·6 (1·7 to 3·8)	6·3 (5·2 to 8·0)	90·3 (78·2 to 103·4)	42·4 (33·0 to 53·1)	19·4 (15·2 to 24·8)	8·3 (5·4 to 12·2)	20·2 (16·6 to 25·5)
Madagascar*	2046	28·7 (24·8 to 32·8)	13·6 (10·6 to 17·0)	6·1 (4·8 to 7·8)	2·7 (1·7 to 3·9)	6·4 (5·2 to 8·0)	91·8 (79·3 to 104·9)	43·4 (33·9 to 54·4)	19·5 (15·3 to 24·9)	8·5 (5·5 to 12·6)	20·3 (16·7 to 25·6)

Madagascar*	2047	29·2 (25·2 to 33·4)	13·9 (10·8 to 17·4)	6·1 (4·8 to 7·8)	2·8 (1·8 to 4·0)	6·4 (5·2 to 8·2)	93·4 (80·6 to 106·8)	44·4 (34·7 to 55·7)	19·6 (15·4 to 25·1)	8·8 (5·7 to 12·9)	20·5 (16·8 to 26·1)
Madagascar*	2048	29·7 (25·6 to 34·0)	14·2 (11·1 to 17·8)	6·2 (4·9 to 7·9)	2·8 (1·8 to 4·2)	6·4 (5·3 to 8·1)	94·9 (81·9 to 108·7)	45·4 (35·4 to 57·0)	19·8 (15·5 to 25·3)	9·1 (5·9 to 13·3)	20·6 (17·0 to 26·0)
Madagascar*	2049	30·2 (26·1 to 34·6)	14·5 (11·3 to 18·3)	6·2 (4·9 to 8·0)	2·9 (1·9 to 4·3)	6·5 (5·3 to 8·3)	96·5 (83·4 to 110·8)	46·5 (36·2 to 58·5)	19·9 (15·7 to 25·4)	9·4 (6·0 to 13·8)	20·7 (16·8 to 26·4)
Madagascar*	2050	30·7 (26·5 to 35·3)	14·9 (11·6 to 18·7)	6·3 (4·9 to 8·0)	3·0 (1·9 to 4·5)	6·5 (5·3 to 8·3)	98·1 (84·7 to 112·8)	47·5 (37·0 to 60·0)	20·0 (15·7 to 25·6)	9·7 (6·2 to 14·3)	20·9 (16·9 to 26·6)
Maldives*	1995	605·0 (562·6 to 648·2)	149·8 (129·0 to 172·3)	390·0 (356·1 to 427·6)	65·1 (49·3 to 82·8)	0·1 (0·1 to 0·1)	1114·6 (1036·4 to 1194·2)	276·1 (237·6 to 317·5)	718·4 (656·0 to 787·8)	120·0 (90·9 to 152·6)	0·2 (0·2 to 0·2)
Maldives*	1996	595·1 (559·2 to 632·4)	153·6 (134·0 to 175·0)	374·5 (347·1 to 404·5)	67·0 (52·1 to 83·5)	0·0 (0·0 to 0·0)	1096·3 (1030·2 to 1165·2)	282·9 (246·8 to 322·4)	690·0 (639·4 to 745·3)	123·4 (96·0 to 153·7)	0·1 (0·1 to 0·1)
Maldives*	1997	600·9 (566·0 to 637·4)	163·2 (144·4 to 184·9)	364·3 (337·8 to 390·7)	69·2 (55·2 to 85·3)	4·2 (4·2 to 4·2)	1107·1 (1042·7 to 1174·3)	300·6 (266·0 to 340·6)	671·2 (622·3 to 719·9)	127·4 (101·8 to 157·2)	7·8 (7·8 to 7·8)
Maldives*	1998	611·5 (577·0 to 650·5)	182·0 (162·0 to 203·6)	359·6 (334·5 to 385·6)	69·7 (55·7 to 85·1)	0·1 (0·1 to 0·1)	1126·5 (1062·9 to 1198·4)	335·4 (298·5 to 375·2)	662·5 (616·2 to 710·4)	128·5 (102·6 to 156·7)	0·1 (0·1 to 0·1)
Maldives*	1999	607·4 (573·6 to 645·7)	191·5 (171·2 to 212·5)	351·2 (325·5 to 377·5)	63·7 (50·4 to 78·6)	1·1 (1·1 to 1·1)	1119·0 (1056·7 to 1189·5)	352·8 (315·5 to 391·4)	647·0 (599·7 to 695·5)	117·3 (92·9 to 144·9)	2·0 (2·0 to 2·0)
Maldives*	2000	566·5 (535·8 to 600·5)	186·6 (166·5 to 207·3)	331·9 (308·1 to 356·4)	46·1 (36·7 to 56·7)	1·9 (1·9 to 1·9)	1043·7 (987·1 to 1106·3)	343·7 (306·7 to 381·8)	611·5 (567·5 to 656·7)	85·0 (67·7 to 104·5)	3·5 (3·5 to 3·5)
Maldives*	2001	564·2 (531·9 to 598·9)	181·0 (161·7 to 200·2)	321·3 (296·6 to 345·8)	61·3 (48·8 to 76·1)	0·6 (0·6 to 0·6)	1039·5 (979·9 to 1103·3)	333·4 (297·9 to 368·8)	592·0 (546·4 to 637·1)	113·0 (89·9 to 140·2)	1·1 (1·1 to 1·1)
Maldives*	2002	594·2 (560·6 to 634·0)	184·1 (165·1 to 203·3)	324·6 (300·6 to 348·9)	85·0 (68·6 to 104·8)	0·5 (0·5 to 0·5)	1094·7 (1032·8 to 1168·1)	339·1 (304·2 to 374·5)	598·0 (553·9 to 642·7)	156·6 (126·4 to 193·0)	1·0 (1·0 to 1·0)
Maldives*	2003	611·6 (577·9 to 650·0)	191·6 (173·0 to 210·7)	336·3 (312·0 to 360·8)	83·2 (67·5 to 103·5)	0·5 (0·5 to 0·5)	1126·8 (1064·6 to 1197·4)	353·1 (318·8 to 388·1)	619·5 (574·9 to 664·6)	153·2 (124·4 to 190·7)	1·0 (1·0 to 1·0)
Maldives*	2004	631·8 (598·9 to 668·9)	202·7 (185·4 to 221·6)	354·5 (330·9 to 379·7)	73·8 (59·5 to 90·9)	0·7 (0·7 to 0·7)	1163·9 (1103·3 to 1232·4)	373·4 (341·5 to 408·3)	653·1 (609·7 to 699·5)	136·0 (109·7 to 167·4)	1·4 (1·4 to 1·4)

Maldives*	2005	718·4 (682·9 to 760·7)	266·4 (244·1 to 290·1)	375·0 (352·0 to 402·4)	75·0 (60·2 to 92·5)	1·9 (1·9 to 1·9)	1323·5 (1258·1 to 1401·5)	490·9 (449·7 to 534·4)	690·8 (648·5 to 741·3)	138·3 (110·9 to 170·3)	3·6 (3·6 to 3·6)
Maldives*	2006	795·2 (758·5 to 837·2)	334·0 (309·7 to 359·2)	381·0 (355·9 to 406·5)	79·6 (63·6 to 97·1)	0·6 (0·6 to 0·6)	1465·0 (1397·4 to 1542·3)	615·3 (570·6 to 661·7)	702·0 (655·7 to 749·0)	146·6 (117·2 to 178·8)	1·1 (1·1 to 1·1)
Maldives*	2007	887·5 (850·3 to 928·6)	430·6 (404·0 to 458·3)	385·2 (360·5 to 413·1)	65·7 (52·3 to 80·7)	5·9 (5·9 to 5·9)	1635·1 (1566·5 to 1710·8)	793·4 (744·4 to 844·4)	709·7 (664·1 to 761·0)	121·1 (96·3 to 148·6)	10·9 (10·9 to 10·9)
Maldives*	2008	1016·3 (973·9 to 1060·3)	547·3 (516·6 to 575·6)	389·0 (362·9 to 417·1)	77·9 (61·2 to 95·6)	2·2 (2·2 to 2·2)	1872·4 (1794·3 to 1953·4)	1008·2 (951·8 to 1060·4)	716·6 (668·6 to 768·3)	143·5 (112·8 to 176·1)	4·1 (4·1 to 4·1)
Maldives*	2009	944·7 (907·3 to 983·8)	532·4 (503·7 to 562·7)	353·6 (328·7 to 378·8)	54·3 (43·2 to 66·8)	4·4 (4·4 to 4·4)	1740·4 (1671·5 to 1812·5)	980·9 (927·9 to 1036·7)	651·3 (605·6 to 697·8)	100·0 (79·6 to 123·0)	8·2 (8·2 to 8·2)
Maldives*	2010	855·8 (818·9 to 896·5)	470·5 (443·4 to 501·0)	325·4 (302·1 to 349·7)	56·1 (45·0 to 68·5)	3·8 (3·8 to 3·8)	1576·6 (1508·7 to 1651·7)	866·8 (816·9 to 923·0)	599·4 (556·5 to 644·3)	103·4 (82·8 to 126·2)	7·1 (7·1 to 7·1)
Maldives*	2011	826·9 (790·8 to 867·2)	430·7 (404·6 to 458·1)	302·9 (280·7 to 327·9)	83·5 (68·5 to 101·1)	9·9 (9·9 to 9·9)	1523·5 (1456·9 to 1597·6)	793·4 (745·5 to 844·0)	558·1 (517·2 to 604·0)	153·8 (126·2 to 186·2)	18·2 (18·2 to 18·2)
Maldives*	2012	821·0 (781·2 to 862·6)	426·6 (400·2 to 452·5)	277·0 (253·3 to 299·1)	111·2 (93·1 to 132·1)	6·2 (6·2 to 6·2)	1512·5 (1439·2 to 1589·1)	785·8 (737·3 to 833·6)	510·4 (466·7 to 551·1)	204·9 (171·5 to 243·4)	11·4 (11·4 to 11·4)
Maldives*	2013	805·0 (767·2 to 846·0)	443·6 (417·0 to 470·8)	252·0 (229·3 to 275·6)	108·1 (90·9 to 127·7)	1·4 (1·4 to 1·4)	1483·1 (1413·5 to 1558·6)	817·3 (768·3 to 867·4)	464·2 (422·5 to 507·8)	199·2 (167·5 to 235·3)	2·5 (2·5 to 2·5)
Maldives*	2014	900·5 (860·1 to 943·1)	563·9 (533·4 to 593·0)	238·7 (216·3 to 262·6)	97·2 (80·7 to 116·0)	0·7 (0·7 to 0·7)	1658·9 (1584·5 to 1737·5)	1038·8 (982·6 to 1092·6)	439·7 (398·4 to 483·7)	179·1 (148·6 to 213·6)	1·3 (1·3 to 1·3)
Maldives*	2015	948·7 (907·5 to 992·2)	643·0 (613·3 to 672·8)	214·5 (194·2 to 237·0)	89·6 (74·3 to 107·3)	1·6 (1·6 to 1·6)	1747·9 (1671·9 to 1827·9)	1184·7 (1129·9 to 1239·5)	395·2 (357·7 to 436·6)	165·1 (136·9 to 197·6)	2·9 (2·9 to 2·9)
Maldives*	2016	1063·2 (1021·9 to 1108·2)	754·0 (723·2 to 785·6)	215·0 (193·2 to 237·6)	93·3 (76·3 to 113·2)	0·9 (0·9 to 0·9)	1958·7 (1882·7 to 2041·7)	1389·1 (1332·4 to 1447·3)	396·1 (356·0 to 437·6)	171·8 (140·7 to 208·6)	1·7 (1·7 to 1·7)
Maldives*	2017	1048·7 (1006·4 to 1090·5)	751·3 (719·5 to 786·1)	211·3 (189·1 to 233·8)	85·8 (69·3 to 104·0)	0·3 (0·3 to 0·3)	1932·0 (1854·0 to 2009·0)	1384·1 (1325·6 to 1448·2)	389·2 (348·3 to 430·7)	158·1 (127·6 to 191·6)	0·5 (0·5 to 0·5)
Maldives*	2018	1072·0 (1016·8 to 1132·3)	765·3 (723·7 to 810·3)	215·7 (188·8 to 244·9)	84·9 (66·4 to 104·2)	6·0 (6·0 to 6·0)	1974·9 (1873·3 to 2086·0)	1409·8 (1333·3 to 1492·8)	397·5 (347·7 to 451·2)	156·5 (122·4 to 192·0)	11·1 (11·1 to 11·1)

Maldives*	2019	1164·5 (1085·3 to 1244·7)	847·5 (777·4 to 913·3)	223·4 (195·6 to 253·7)	89·6 (70·2 to 110·1)	4·0 (0·0 to 5·8)	2145·3 (1999·5 to 2293·1)	1561·3 (1432·1 to 1682·6)	411·6 (360·4 to 467·3)	165·1 (129·4 to 202·9)	7·4 (0·0 to 10·8)
Maldives*	2020	1341·2 (1264·6 to 1419·2)	1031·7 (962·7 to 1096·5)	216·3 (189·1 to 245·7)	88·5 (69·2 to 108·7)	4·8 (0·0 to 7·4)	2470·9 (2329·8 to 2614·5)	1900·7 (1773·6 to 2020·1)	398·4 (348·4 to 452·6)	163·0 (127·6 to 200·3)	8·8 (0·0 to 13·6)
Maldives*	2021	1260·4 (1163·7 to 1362·1)	951·7 (853·0 to 1041·3)	215·4 (188·2 to 244·6)	89·8 (70·5 to 110·8)	3·5 (0·0 to 6·6)	2322·0 (2143·8 to 2509·4)	1753·2 (1571·6 to 1918·3)	396·8 (346·7 to 450·7)	165·4 (129·8 to 204·1)	6·5 (0·0 to 12·2)
Maldives*	2022	1185·8 (1072·6 to 1301·9)	874·2 (763·1 to 987·6)	216·8 (189·3 to 246·0)	92·2 (72·4 to 113·8)	2·6 (0·0 to 5·2)	2184·6 (1976·0 to 2398·6)	1610·5 (1405·9 to 1819·5)	399·4 (348·8 to 453·3)	169·9 (133·3 to 209·6)	4·8 (0·0 to 9·6)
Maldives*	2023	1216·3 (1093·2 to 1342·0)	900·8 (785·3 to 1021·2)	218·1 (190·5 to 247·6)	94·9 (74·6 to 117·1)	2·5 (0·0 to 5·3)	2240·7 (2013·9 to 2472·4)	1659·5 (1446·8 to 1881·3)	401·8 (350·9 to 456·2)	174·8 (137·5 to 215·8)	4·7 (0·0 to 9·8)
Maldives*	2024	1247·9 (1115·3 to 1383·4)	928·3 (803·3 to 1057·3)	219·4 (191·5 to 248·9)	97·7 (77·0 to 120·9)	2·5 (0·0 to 5·5)	2299·0 (2054·7 to 2548·6)	1710·2 (1479·9 to 1947·8)	404·2 (352·8 to 458·6)	180·0 (141·8 to 222·6)	4·6 (0·0 to 10·1)
Maldives*	2025	1278·0 (1127·7 to 1427·1)	953·5 (812·7 to 1093·6)	220·9 (193·3 to 250·4)	101·1 (79·5 to 125·7)	2·4 (0·0 to 5·6)	2354·4 (2077·5 to 2629·2)	1756·7 (1497·3 to 2014·7)	407·0 (356·2 to 461·3)	186·2 (146·4 to 231·5)	4·5 (0·0 to 10·4)
Maldives*	2026	1295·6 (1129·8 to 1465·4)	965·5 (807·2 to 1123·9)	222·7 (194·5 to 252·3)	105·0 (82·7 to 130·9)	2·4 (0·0 to 5·8)	2386·9 (2081·5 to 2699·7)	1778·7 (1487·1 to 2070·5)	410·4 (358·4 to 464·7)	193·4 (152·3 to 241·1)	4·4 (0·0 to 10·6)
Maldives*	2027	1313·5 (1136·5 to 1489·9)	977·5 (801·8 to 1140·7)	224·4 (196·5 to 254·0)	109·2 (85·6 to 136·5)	2·4 (0·0 to 5·9)	2419·8 (2093·7 to 2744·9)	1800·8 (1477·2 to 2101·6)	413·4 (362·1 to 468·0)	201·2 (157·8 to 251·5)	4·4 (0·0 to 10·9)
Maldives*	2028	1333·2 (1144·3 to 1524·4)	990·6 (810·5 to 1166·1)	226·3 (198·3 to 256·8)	113·9 (89·4 to 142·5)	2·3 (0·0 to 6·1)	2456·1 (2108·1 to 2808·4)	1825·1 (1493·1 to 2148·3)	416·9 (365·3 to 473·1)	209·9 (164·7 to 262·6)	4·2 (0·0 to 11·2)
Maldives*	2029	1355·5 (1151·7 to 1563·4)	1005·9 (819·6 to 1196·9)	228·4 (200·7 to 260·0)	118·9 (93·2 to 148·2)	2·3 (0·0 to 6·2)	2497·3 (2121·8 to 2880·2)	1853·2 (1509·9 to 2205·1)	420·8 (369·8 to 479·0)	219·1 (171·8 to 273·1)	4·2 (0·0 to 11·4)
Maldives*	2030	1377·4 (1160·4 to 1593·8)	1020·3 (817·6 to 1227·9)	230·7 (203·0 to 262·8)	124·2 (97·0 to 155·7)	2·2 (0·0 to 6·3)	2537·6 (2137·8 to 2936·3)	1879·7 (1506·2 to 2262·2)	425·1 (374·0 to 484·1)	228·7 (178·7 to 286·8)	4·0 (0·0 to 11·6)
Maldives*	2031	1398·8 (1176·4 to 1633·1)	1034·1 (822·3 to 1259·4)	232·9 (205·0 to 265·4)	129·6 (101·2 to 163·4)	2·2 (0·0 to 6·4)	2577·0 (2167·3 to 3008·7)	1905·1 (1515·0 to 2320·1)	429·2 (377·6 to 489·0)	238·7 (186·5 to 301·1)	4·0 (0·0 to 11·8)
Maldives*	2032	1421·9 (1192·2 to 1671·8)	1049·3 (826·5 to 1290·5)	235·2 (207·2 to 268·3)	135·2 (105·1 to 171·6)	2·2 (0·0 to 6·5)	2619·6 (2196·3 to 3079·9)	1933·1 (1522·7 to 2377·6)	433·3 (381·8 to 494·3)	249·2 (193·6 to 316·1)	4·0 (0·0 to 12·0)

Maldives*	2033	1442·3 (1208·0 to 1698·7)	1062·1 (837·6 to 1301·0)	237·1 (208·6 to 271·1)	140·9 (108·6 to 179·8)	2·1 (0·0 to 6·7)	2657·2 (2225·5 to 3129·5)	1956·7 (1543·1 to 2396·8)	436·9 (384·3 to 499·4)	259·6 (200·1 to 331·2)	3·9 (0·0 to 12·3)
Maldives*	2034	1464·4 (1222·0 to 1730·3)	1076·0 (835·9 to 1328·1)	239·3 (210·2 to 273·4)	147·0 (112·5 to 188·3)	2·1 (0·0 to 6·8)	2697·8 (2251·3 to 3187·7)	1982·3 (1539·9 to 2446·7)	440·8 (387·2 to 503·6)	270·8 (207·3 to 347·0)	3·9 (0·0 to 12·6)
Maldives*	2035	1485·8 (1231·2 to 1759·4)	1089·3 (849·0 to 1356·3)	241·2 (211·6 to 275·6)	153·2 (116·4 to 196·8)	2·1 (0·0 to 7·0)	2737·4 (2268·2 to 3241·4)	2006·8 (1564·2 to 2498·7)	444·4 (389·8 to 507·7)	282·2 (214·4 to 362·6)	3·9 (0·0 to 13·0)
Maldives*	2036	1508·5 (1244·9 to 1805·7)	1103·9 (842·8 to 1382·9)	242·9 (212·8 to 277·4)	159·5 (119·9 to 206·8)	2·1 (0·0 to 7·2)	2779·0 (2293·5 to 3326·7)	2033·8 (1552·7 to 2547·8)	447·5 (392·0 to 511·0)	293·8 (220·9 to 381·0)	3·9 (0·0 to 13·3)
Maldives*	2037	1527·0 (1244·8 to 1852·1)	1114·6 (844·5 to 1418·7)	244·4 (213·8 to 279·7)	165·9 (123·4 to 217·6)	2·1 (0·0 to 7·4)	2813·2 (2293·3 to 3412·1)	2053·5 (1555·9 to 2613·8)	450·2 (393·9 to 515·4)	305·6 (227·4 to 400·9)	3·9 (0·0 to 13·7)
Maldives*	2038	1548·6 (1262·6 to 1870·8)	1127·8 (857·5 to 1426·9)	246·1 (215·3 to 281·6)	172·6 (127·1 to 228·9)	2·1 (0·0 to 7·7)	2853·0 (2326·1 to 3446·6)	2077·7 (1579·7 to 2628·8)	453·3 (396·7 to 518·7)	318·0 (234·2 to 421·7)	3·9 (0·0 to 14·2)
Maldives*	2039	1569·2 (1271·7 to 1901·1)	1140·3 (856·2 to 1453·8)	247·4 (216·2 to 283·3)	179·4 (130·1 to 239·7)	2·1 (0·0 to 8·1)	2890·9 (2342·9 to 3502·4)	2100·7 (1577·3 to 2678·2)	455·8 (398·3 to 521·9)	330·5 (239·8 to 441·7)	3·9 (0·0 to 14·9)
Maldives*	2040	1591·5 (1284·7 to 1933·8)	1153·9 (861·8 to 1482·0)	248·9 (217·2 to 284·9)	186·6 (133·0 to 252·9)	2·1 (0·0 to 8·5)	2932·1 (2366·7 to 3562·6)	2125·8 (1587·7 to 2730·4)	458·6 (400·1 to 524·9)	343·8 (245·1 to 466·0)	3·9 (0·0 to 15·6)
Maldives*	2041	1611·1 (1302·0 to 1975·5)	1164·8 (858·0 to 1503·5)	250·2 (217·9 to 286·4)	194·0 (135·9 to 266·6)	2·2 (0·0 to 8·8)	2968·1 (2398·6 to 3639·5)	2145·9 (1580·7 to 2769·9)	460·9 (401·5 to 527·6)	357·4 (250·3 to 491·1)	4·0 (0·0 to 16·3)
Maldives*	2042	1628·4 (1314·5 to 1997·5)	1173·9 (873·7 to 1520·0)	250·9 (218·2 to 288·1)	201·4 (138·0 to 281·4)	2·2 (0·0 to 9·3)	2999·9 (2421·7 to 3680·0)	2162·6 (1609·7 to 2800·4)	462·3 (402·1 to 530·7)	371·0 (254·2 to 518·5)	4·1 (0·0 to 17·2)
Maldives*	2043	1646·2 (1320·5 to 2032·0)	1183·2 (862·7 to 1546·2)	251·6 (218·5 to 289·7)	209·1 (141·0 to 296·7)	2·2 (0·0 to 9·7)	3032·8 (2432·8 to 3743·6)	2179·9 (1589·3 to 2848·6)	463·6 (402·6 to 533·7)	385·3 (259·8 to 546·7)	4·1 (0·0 to 17·9)
Maldives*	2044	1661·9 (1330·7 to 2045·2)	1190·4 (874·8 to 1559·9)	252·0 (218·7 to 290·7)	217·1 (143·2 to 312·8)	2·3 (0·0 to 10·4)	3061·7 (2451·5 to 3767·8)	2193·1 (1611·7 to 2873·7)	464·3 (402·8 to 535·5)	400·0 (263·9 to 576·3)	4·2 (0·0 to 19·2)
Maldives*	2045	1675·3 (1340·5 to 2081·8)	1195·6 (871·6 to 1595·8)	252·1 (218·4 to 291·0)	225·3 (145·6 to 329·7)	2·4 (0·0 to 11·1)	3086·5 (2469·6 to 3835·2)	2202·7 (1605·8 to 2939·9)	464·4 (402·4 to 536·0)	415·0 (268·2 to 607·4)	4·4 (0·0 to 20·5)
Maldives*	2046	1687·5 (1342·3 to 2101·8)	1199·6 (862·3 to 1597·3)	251·9 (218·3 to 290·9)	233·5 (147·4 to 344·9)	2·5 (0·0 to 12·0)	3108·9 (2472·9 to 3872·2)	2210·0 (1588·6 to 2942·7)	464·1 (402·2 to 535·9)	430·2 (271·6 to 635·4)	4·6 (0·0 to 22·1)

Maldives*	2047	1697·6 (1335·2 to 2114·6)	1201·8 (854·1 to 1596·2)	251·4 (217·5 to 291·0)	241·8 (148·7 to 361·9)	2·7 (0·0 to 12·8)	3127·6 (2459·8 to 3895·8)	2214·1 (1573·5 to 2940·7)	463·1 (400·7 to 536·1)	445·4 (273·9 to 666·7)	4·9 (0·0 to 23·6)
Maldives*	2048	1705·2 (1343·3 to 2127·2)	1202·3 (859·8 to 1609·0)	250·2 (216·2 to 290·0)	249·9 (149·4 to 383·1)	2·8 (0·0 to 13·9)	3141·6 (2474·8 to 3918·9)	2215·0 (1584·0 to 2964·2)	461·0 (398·3 to 534·3)	460·3 (275·3 to 705·9)	5·2 (0·0 to 25·7)
Maldives*	2049	1710·2 (1344·1 to 2155·0)	1199·8 (859·6 to 1597·7)	249·0 (214·8 to 288·9)	258·4 (149·9 to 405·9)	3·1 (0·0 to 15·2)	3150·7 (2476·2 to 3970·1)	2210·4 (1583·7 to 2943·4)	458·7 (395·8 to 532·2)	476·0 (276·1 to 747·8)	5·6 (0·0 to 28·1)
Maldives*	2050	1711·6 (1343·2 to 2145·9)	1194·4 (846·5 to 1590·3)	247·2 (212·9 to 287·1)	266·6 (151·3 to 428·8)	3·3 (0·0 to 17·0)	3153·2 (2474·6 to 3953·4)	2200·5 (1559·4 to 2929·8)	455·4 (392·3 to 529·0)	491·2 (278·8 to 789·9)	6·2 (0·0 to 31·3)
Mexico*	1995	278·6 (256·0 to 305·3)	117·4 (102·9 to 135·2)	155·9 (139·8 to 175·6)	3·6 (2·5 to 5·1)	1·7 (1·7 to 1·7)	649·2 (596·6 to 711·6)	273·5 (239·7 to 315·0)	363·3 (325·8 to 409·1)	8·4 (5·9 to 11·9)	4·0 (4·0 to 4·0)
Mexico*	1996	284·4 (263·6 to 306·6)	124·2 (110·2 to 140·8)	154·5 (140·1 to 170·8)	4·2 (2·9 to 5·9)	1·6 (1·6 to 1·6)	662·8 (614·4 to 714·6)	289·5 (256·8 to 328·0)	360·0 (326·4 to 398·1)	9·7 (6·7 to 13·7)	3·6 (3·6 to 3·6)
Mexico*	1997	299·3 (279·1 to 319·7)	135·2 (122·1 to 150·9)	157·2 (143·5 to 171·8)	5·1 (3·6 to 7·1)	1·9 (1·9 to 1·9)	697·6 (650·5 to 745·0)	315·0 (284·5 to 351·6)	366·4 (334·5 to 400·3)	11·9 (8·3 to 16·5)	4·3 (4·3 to 4·3)
Mexico*	1998	318·2 (298·4 to 338·1)	143·9 (130·3 to 158·4)	162·2 (148·4 to 177·0)	5·8 (4·1 to 8·1)	6·3 (6·3 to 6·3)	741·6 (695·4 to 787·8)	335·3 (303·6 to 369·1)	378·0 (345·8 to 412·5)	13·6 (9·7 to 18·8)	14·8 (14·8 to 14·8)
Mexico*	1999	336·9 (317·4 to 358·8)	156·4 (142·7 to 171·8)	172·4 (157·5 to 188·0)	6·8 (5·0 to 9·2)	1·3 (1·3 to 1·3)	785·2 (739·7 to 836·2)	364·5 (332·5 to 400·3)	401·7 (367·0 to 438·2)	15·9 (11·7 to 21·5)	3·1 (3·1 to 3·1)
Mexico*	2000	361·8 (342·3 to 384·0)	162·5 (147·9 to 178·1)	186·3 (171·2 to 202·0)	8·6 (6·5 to 11·1)	4·4 (4·4 to 4·4)	843·1 (797·6 to 894·9)	378·8 (344·8 to 415·1)	434·1 (399·0 to 470·7)	20·0 (15·1 to 25·9)	10·2 (10·2 to 10·2)
Mexico*	2001	381·4 (361·1 to 403·2)	167·3 (153·4 to 181·4)	202·5 (188·3 to 218·9)	10·1 (8·0 to 12·3)	1·5 (1·5 to 1·5)	888·8 (841·6 to 939·6)	389·9 (357·5 to 422·8)	472·0 (438·9 to 510·2)	23·5 (18·6 to 28·7)	3·4 (3·4 to 3·4)
Mexico*	2002	405·9 (387·5 to 425·1)	172·4 (159·4 to 185·8)	220·0 (207·3 to 234·3)	11·2 (9·5 to 13·1)	2·2 (2·2 to 2·2)	945·9 (903·1 to 990·7)	401·8 (371·6 to 432·9)	512·8 (483·2 to 546·0)	26·1 (22·1 to 30·6)	5·2 (5·2 to 5·2)
Mexico*	2003	444·7 (427·5 to 462·7)	188·5 (175·6 to 201·1)	243·2 (231·8 to 255·6)	12·5 (11·1 to 14·1)	0·5 (0·5 to 0·5)	1036·3 (996·4 to 1078·2)	439·3 (409·1 to 468·7)	566·7 (540·1 to 595·6)	29·2 (25·8 to 33·0)	1·1 (1·1 to 1·1)
Mexico*	2004	457·6 (441·9 to 473·2)	192·5 (181·2 to 203·9)	251·4 (241·0 to 262·2)	13·4 (12·1 to 14·7)	0·4 (0·4 to 0·4)	1066·5 (1029·8 to 1102·7)	448·6 (422·2 to 475·1)	585·9 (561·6 to 611·0)	31·2 (28·3 to 34·2)	0·9 (0·9 to 0·9)

Mexico*	2005	466·8 (451·8 to 481·9)	197·3 (186·8 to 208·1)	254·1 (244·3 to 264·7)	14·6 (13·4 to 15·9)	0·9 (0·9 to 0·9)	1087·9 (1053·0 to 1123·0)	459·7 (435·3 to 485·1)	592·1 (569·4 to 616·8)	34·0 (31·2 to 37·0)	2·1 (2·1 to 2·1)
Mexico*	2006	470·7 (454·8 to 485·6)	201·7 (190·3 to 213·9)	251·4 (241·5 to 262·2)	16·3 (15·1 to 17·5)	1·3 (1·3 to 1·3)	1097·0 (1059·9 to 1131·6)	470·1 (443·5 to 498·4)	585·9 (562·8 to 611·1)	37·9 (35·2 to 40·9)	3·1 (3·1 to 3·1)
Mexico*	2007	471·7 (456·6 to 486·8)	206·9 (195·3 to 218·9)	244·6 (234·9 to 254·5)	19·2 (18·0 to 20·5)	1·0 (1·0 to 1·0)	1099·4 (1064·1 to 1134·4)	482·2 (455·1 to 510·1)	570·1 (547·4 to 593·1)	44·7 (42·0 to 47·7)	2·4 (2·4 to 2·4)
Mexico*	2008	491·5 (476·1 to 507·2)	226·5 (213·7 to 239·1)	238·9 (228·7 to 248·6)	25·1 (23·8 to 26·6)	1·0 (1·0 to 1·0)	1145·4 (1109·5 to 1182·0)	527·8 (498·1 to 557·2)	556·7 (533·1 to 579·4)	58·5 (55·4 to 61·9)	2·4 (2·4 to 2·4)
Mexico*	2009	473·1 (457·7 to 488·3)	221·4 (210·2 to 233·3)	223·6 (213·7 to 234·4)	27·5 (26·1 to 29·0)	0·5 (0·5 to 0·5)	1102·5 (1066·6 to 1138·0)	516·0 (489·8 to 543·6)	521·2 (498·1 to 546·2)	64·2 (60·8 to 67·5)	1·2 (1·2 to 1·2)
Mexico*	2010	481·6 (466·0 to 497·3)	231·9 (220·3 to 242·8)	216·0 (206·0 to 226·1)	28·1 (26·7 to 29·4)	5·6 (5·6 to 5·6)	1122·4 (1086·0 to 1158·9)	540·5 (513·4 to 565·9)	503·3 (480·1 to 526·9)	65·4 (62·2 to 68·5)	13·1 (13·1 to 13·1)
Mexico*	2011	485·0 (468·6 to 500·9)	242·9 (231·1 to 255·0)	208·9 (199·1 to 218·7)	28·2 (26·8 to 29·7)	5·1 (5·1 to 5·1)	1130·4 (1092·0 to 1167·4)	566·0 (538·6 to 594·2)	486·7 (464·0 to 509·7)	65·7 (62·5 to 69·1)	11·9 (11·9 to 11·9)
Mexico*	2012	496·3 (479·6 to 512·9)	256·5 (244·0 to 268·8)	208·0 (198·3 to 218·6)	29·0 (27·6 to 30·5)	2·8 (2·8 to 2·8)	1156·5 (1117·8 to 1195·3)	597·7 (568·6 to 626·3)	484·7 (462·1 to 509·4)	67·5 (64·3 to 71·1)	6·5 (6·5 to 6·5)
Mexico*	2013	495·7 (479·4 to 512·0)	260·3 (248·2 to 273·1)	204·4 (194·8 to 215·3)	30·1 (28·8 to 31·6)	0·9 (0·9 to 0·9)	1155·2 (1117·2 to 1193·2)	606·5 (578·4 to 636·4)	476·4 (454·0 to 501·7)	70·3 (67·0 to 73·6)	2·0 (2·0 to 2·0)
Mexico*	2014	501·0 (483·8 to 517·6)	265·9 (253·2 to 278·1)	202·1 (192·4 to 212·8)	32·4 (31·0 to 33·8)	0·6 (0·6 to 0·6)	1167·5 (1127·4 to 1206·2)	619·7 (590·1 to 648·2)	470·9 (448·4 to 495·8)	75·5 (72·3 to 78·9)	1·4 (1·4 to 1·4)
Mexico*	2015	503·9 (487·3 to 520·7)	266·2 (254·3 to 278·2)	203·1 (193·5 to 213·4)	33·7 (32·2 to 35·3)	0·9 (0·9 to 0·9)	1174·3 (1135·7 to 1213·5)	620·3 (592·6 to 648·3)	473·4 (450·9 to 497·2)	78·5 (75·1 to 82·2)	2·1 (2·1 to 2·1)
Mexico*	2016	506·1 (489·5 to 523·2)	264·9 (253·0 to 277·0)	205·0 (194·8 to 215·2)	35·5 (34·0 to 37·0)	0·7 (0·7 to 0·7)	1179·5 (1140·8 to 1219·3)	617·3 (589·6 to 645·6)	477·8 (454·1 to 501·5)	82·7 (79·3 to 86·2)	1·6 (1·6 to 1·6)
Mexico*	2017	497·1 (481·4 to 514·2)	254·6 (242·1 to 267·4)	205·2 (195·5 to 216·3)	36·4 (35·0 to 37·9)	0·9 (0·9 to 0·9)	1158·4 (1121·9 to 1198·4)	593·2 (564·2 to 623·2)	478·3 (455·6 to 504·0)	84·9 (81·6 to 88·4)	2·0 (2·0 to 2·0)
Mexico*	2018	499·7 (479·9 to 521·8)	253·1 (237·7 to 270·6)	207·8 (195·0 to 221·6)	38·6 (36·7 to 40·6)	0·1 (0·1 to 0·1)	1164·5 (1118·4 to 1216·2)	589·9 (554·1 to 630·7)	484·4 (454·5 to 516·5)	89·9 (85·6 to 94·6)	0·3 (0·3 to 0·3)

Mexico*	2019	502·4 (482·3 to 524·6)	258·3 (242·2 to 275·9)	204·9 (192·3 to 218·5)	39·1 (37·2 to 41·2)	0·1 (0·1 to 0·1)	1170·9 (1124·1 to 1222·6)	601·9 (564·3 to 643·0)	477·6 (448·1 to 509·3)	91·2 (86·8 to 96·1)	0·3 (0·3 to 0·3)
Mexico*	2020	501·0 (480·5 to 523·0)	273·8 (257·0 to 292·9)	189·7 (177·9 to 202·3)	37·3 (35·4 to 39·4)	0·2 (0·2 to 0·2)	1167·6 (1119·9 to 1218·9)	638·1 (598·9 to 682·7)	442·2 (414·7 to 471·5)	86·9 (82·4 to 91·8)	0·4 (0·4 to 0·4)
Mexico*	2021	491·6 (471·6 to 513·3)	268·5 (252·0 to 287·2)	185·5 (174·0 to 197·9)	37·5 (35·4 to 39·8)	0·2 (0·2 to 0·2)	1145·8 (1099·0 to 1196·1)	625·7 (587·2 to 669·3)	432·4 (405·6 to 461·2)	87·3 (82·5 to 92·8)	0·4 (0·4 to 0·4)
Mexico*	2022	465·8 (446·0 to 487·4)	240·7 (224·3 to 258·9)	186·3 (174·8 to 198·9)	38·6 (36·4 to 41·3)	0·1 (0·1 to 0·1)	1085·5 (1039·3 to 1135·8)	560·9 (522·6 to 603·3)	434·2 (407·4 to 463·4)	90·0 (84·8 to 96·1)	0·3 (0·3 to 0·3)
Mexico*	2023	470·6 (449·5 to 493·1)	243·5 (226·1 to 262·6)	187·1 (175·6 to 199·7)	39·9 (37·3 to 42·7)	0·1 (0·1 to 0·2)	1096·8 (1047·5 to 1149·2)	567·5 (526·8 to 612·0)	436·1 (409·2 to 465·4)	93·0 (86·9 to 99·5)	0·3 (0·3 to 0·4)
Mexico*	2024	475·5 (453·7 to 499·0)	246·3 (228·4 to 265·6)	187·8 (176·3 to 200·5)	41·2 (38·3 to 44·4)	0·1 (0·1 to 0·2)	1108·0 (1057·3 to 1162·9)	574·0 (532·3 to 618·9)	437·8 (410·9 to 467·3)	95·9 (89·2 to 103·4)	0·3 (0·3 to 0·4)
Mexico*	2025	479·9 (457·0 to 504·9)	248·9 (229·9 to 269·7)	188·4 (176·9 to 201·0)	42·5 (39·3 to 46·1)	0·2 (0·1 to 0·2)	1118·4 (1065·0 to 1176·6)	580·1 (535·7 to 628·6)	439·0 (412·2 to 468·5)	99·0 (91·6 to 107·5)	0·4 (0·3 to 0·4)
Mexico*	2026	484·3 (460·7 to 510·1)	251·4 (230·6 to 272·9)	189·0 (177·5 to 201·6)	43·8 (40·1 to 47·9)	0·2 (0·1 to 0·2)	1128·7 (1073·7 to 1188·8)	585·9 (537·5 to 636·0)	440·3 (413·6 to 469·8)	102·1 (93·6 to 111·6)	0·4 (0·3 to 0·4)
Mexico*	2027	488·8 (464·3 to 515·6)	254·0 (231·7 to 276·6)	189·4 (178·0 to 202·0)	45·2 (40·9 to 49·8)	0·2 (0·2 to 0·2)	1139·1 (1082·1 to 1201·6)	592·0 (540·1 to 644·7)	441·5 (414·9 to 470·8)	105·3 (95·4 to 116·0)	0·4 (0·4 to 0·4)
Mexico*	2028	494·1 (469·1 to 521·9)	257·4 (235·7 to 281·2)	190·0 (178·4 to 202·6)	46·6 (41·9 to 51·4)	0·2 (0·2 to 0·2)	1151·6 (1093·2 to 1216·3)	599·8 (549·4 to 655·4)	442·8 (415·8 to 472·0)	108·5 (97·6 to 119·8)	0·4 (0·4 to 0·4)
Mexico*	2029	499·9 (474·1 to 527·6)	261·3 (238·8 to 285·5)	190·4 (178·8 to 202·9)	48·0 (43·2 to 53·4)	0·2 (0·2 to 0·2)	1165·0 (1105·0 to 1229·6)	608·9 (556·4 to 665·4)	443·7 (416·6 to 472·9)	111·9 (100·6 to 124·4)	0·4 (0·4 to 0·5)
Mexico*	2030	504·8 (478·6 to 533·7)	264·3 (240·3 to 290·6)	190·8 (179·2 to 203·4)	49·5 (44·5 to 55·5)	0·2 (0·2 to 0·2)	1176·4 (1115·4 to 1243·8)	615·9 (560·1 to 677·3)	444·7 (417·7 to 474·1)	115·3 (103·7 to 129·3)	0·4 (0·4 to 0·5)
Mexico*	2031	509·4 (483·0 to 538·9)	267·0 (242·9 to 292·7)	191·2 (179·6 to 203·8)	51·0 (45·5 to 57·7)	0·2 (0·2 to 0·2)	1187·1 (1125·6 to 1256·0)	622·2 (566·2 to 682·2)	445·6 (418·6 to 475·0)	118·8 (106·0 to 134·5)	0·4 (0·4 to 0·5)
Mexico*	2032	514·1 (486·5 to 544·1)	269·6 (245·2 to 297·7)	191·7 (180·2 to 204·4)	52·6 (46·8 to 60·0)	0·2 (0·2 to 0·2)	1198·2 (1133·9 to 1268·0)	628·4 (571·3 to 693·7)	446·8 (419·9 to 476·4)	122·6 (109·0 to 139·7)	0·5 (0·4 to 0·5)

Mexico*	2033	518·8 (490·8 to 549·9)	272·2 (246·6 to 300·9)	192·2 (180·6 to 205·0)	54·2 (47·9 to 61·8)	0·2 (0·2 to 0·2)	1209·2 (1143·9 to 1281·5)	634·5 (574·6 to 701·2)	447·9 (421·0 to 477·8)	126·4 (111·6 to 143·9)	0·5 (0·4 to 0·5)
Mexico*	2034	523·8 (494·1 to 556·5)	275·1 (248·0 to 305·6)	192·6 (181·1 to 205·5)	55·9 (49·2 to 64·1)	0·2 (0·2 to 0·2)	1220·8 (1151·4 to 1297·0)	641·0 (577·9 to 712·2)	448·9 (422·1 to 478·9)	130·3 (114·6 to 149·5)	0·5 (0·4 to 0·6)
Mexico*	2035	528·8 (497·9 to 562·3)	277·8 (250·8 to 307·9)	193·1 (181·5 to 206·0)	57·7 (50·2 to 66·3)	0·2 (0·2 to 0·3)	1232·5 (1160·5 to 1310·5)	647·4 (584·4 to 717·5)	450·1 (423·1 to 480·2)	134·4 (117·1 to 154·4)	0·5 (0·5 to 0·6)
Mexico*	2036	534·0 (502·3 to 568·4)	280·7 (251·8 to 312·4)	193·6 (181·8 to 206·5)	59·5 (51·5 to 68·8)	0·2 (0·2 to 0·3)	1244·4 (1170·6 to 1324·7)	654·1 (586·7 to 728·1)	451·1 (423·7 to 481·2)	138·6 (120·1 to 160·4)	0·5 (0·5 to 0·6)
Mexico*	2037	539·4 (507·6 to 575·2)	283·6 (254·7 to 316·0)	194·1 (182·2 to 207·1)	61·4 (52·9 to 71·3)	0·2 (0·2 to 0·3)	1257·0 (1182·9 to 1340·6)	661·0 (593·6 to 736·5)	452·4 (424·6 to 482·6)	143·0 (123·2 to 166·2)	0·6 (0·5 to 0·7)
Mexico*	2038	545·0 (511·8 to 581·2)	286·7 (257·5 to 319·4)	194·7 (182·6 to 207·8)	63·3 (54·1 to 74·0)	0·2 (0·2 to 0·3)	1270·1 (1192·7 to 1354·6)	668·2 (600·1 to 744·4)	453·8 (425·5 to 484·2)	147·5 (126·2 to 172·5)	0·6 (0·5 to 0·7)
Mexico*	2039	550·7 (516·5 to 588·4)	289·8 (258·4 to 324·7)	195·3 (182·9 to 208·4)	65·3 (55·4 to 77·1)	0·3 (0·2 to 0·3)	1283·3 (1203·7 to 1371·2)	675·5 (602·2 to 756·8)	455·1 (426·3 to 485·7)	152·1 (129·2 to 179·6)	0·6 (0·5 to 0·7)
Mexico*	2040	556·2 (521·5 to 594·9)	292·8 (261·4 to 329·1)	195·8 (183·3 to 209·1)	67·3 (56·7 to 80·6)	0·3 (0·2 to 0·3)	1296·1 (1215·3 to 1386·4)	682·3 (609·2 to 766·9)	456·4 (427·3 to 487·3)	156·9 (132·1 to 187·7)	0·6 (0·5 to 0·8)
Mexico*	2041	562·1 (526·2 to 602·8)	296·0 (263·7 to 331·5)	196·5 (184·0 to 209·9)	69·4 (57·9 to 83·6)	0·3 (0·2 to 0·3)	1310·0 (1226·2 to 1404·8)	689·7 (614·5 to 772·6)	457·8 (428·8 to 489·1)	161·8 (135·0 to 194·9)	0·7 (0·6 to 0·8)
Mexico*	2042	568·1 (530·3 to 609·5)	299·1 (265·9 to 337·2)	197·1 (184·7 to 210·6)	71·6 (59·2 to 87·1)	0·3 (0·3 to 0·4)	1323·8 (1235·9 to 1420·3)	697·1 (619·8 to 785·9)	459·3 (430·5 to 490·8)	166·8 (137·9 to 203·1)	0·7 (0·6 to 0·8)
Mexico*	2043	574·2 (535·9 to 616·4)	302·5 (269·1 to 342·7)	197·7 (185·5 to 211·4)	73·8 (60·5 to 90·9)	0·3 (0·3 to 0·4)	1338·3 (1248·9 to 1436·5)	704·9 (627·1 to 798·7)	460·8 (432·3 to 492·6)	171·9 (140·9 to 211·8)	0·7 (0·6 to 0·9)
Mexico*	2044	580·3 (540·9 to 624·4)	305·7 (270·7 to 346·9)	198·3 (186·0 to 211·9)	76·0 (61·8 to 94·2)	0·3 (0·3 to 0·4)	1352·5 (1260·6 to 1455·3)	712·4 (630·9 to 808·4)	462·3 (433·6 to 493·9)	177·1 (143·9 to 219·5)	0·7 (0·6 to 0·9)
Mexico*	2045	586·6 (545·7 to 632·1)	309·0 (273·8 to 352·3)	199·0 (186·6 to 212·5)	78·3 (63·1 to 97·9)	0·3 (0·3 to 0·4)	1367·0 (1271·8 to 1473·2)	720·1 (638·0 to 821·0)	463·8 (434·9 to 495·3)	182·4 (147·0 to 228·1)	0·8 (0·6 to 1·0)
Mexico*	2046	592·9 (551·2 to 639·2)	312·3 (276·6 to 355·6)	199·7 (187·3 to 213·1)	80·6 (64·5 to 101·4)	0·3 (0·3 to 0·4)	1381·8 (1284·5 to 1489·8)	727·8 (644·5 to 828·8)	465·3 (436·5 to 496·7)	187·9 (150·2 to 236·4)	0·8 (0·7 to 1·0)

Mexico*	2047	599·0 (555·7 to 647·3)	315·4 (278·8 to 359·5)	200·2 (187·9 to 213·6)	83·0 (65·8 to 105·0)	0·4 (0·3 to 0·5)	1395·9 (1295·0 to 1508·5)	735·0 (649·8 to 837·8)	466·6 (437·9 to 497·9)	193·4 (153·4 to 244·6)	0·8 (0·7 to 1·1)
Mexico*	2048	605·4 (560·8 to 655·4)	318·7 (280·7 to 362·0)	200·9 (188·5 to 214·3)	85·5 (67·2 to 108·7)	0·4 (0·3 to 0·5)	1410·9 (1306·9 to 1527·5)	742·7 (654·3 to 843·6)	468·1 (439·2 to 499·4)	199·2 (156·7 to 253·2)	0·9 (0·7 to 1·1)
Mexico*	2049	611·8 (566·7 to 663·9)	322·0 (284·6 to 365·5)	201·4 (188·9 to 214·9)	88·0 (68·5 to 112·4)	0·4 (0·3 to 0·5)	1425·7 (1320·6 to 1547·3)	750·4 (663·2 to 851·7)	469·4 (440·3 to 500·9)	205·0 (159·6 to 262·0)	0·9 (0·8 to 1·2)
Mexico*	2050	618·5 (571·6 to 671·1)	325·5 (287·0 to 370·9)	202·1 (189·5 to 215·7)	90·5 (70·0 to 116·3)	0·4 (0·3 to 0·5)	1441·5 (1332·0 to 1564·0)	758·6 (668·8 to 864·3)	470·9 (441·6 to 502·8)	211·0 (163·1 to 271·1)	1·0 (0·8 to 1·3)
Marshall Islands*	1995	341·5 (308·3 to 375·7)	244·4 (216·0 to 274·8)	62·2 (51·8 to 73·8)	24·8 (17·9 to 33·9)	10·1 (10·1 to 10·1)	317·7 (286·8 to 349·5)	227·3 (201·0 to 255·6)	57·8 (48·2 to 68·7)	23·1 (16·7 to 31·5)	9·4 (9·4 to 9·4)
Marshall Islands*	1996	332·5 (305·4 to 361·2)	231·0 (207·0 to 256·2)	59·3 (50·3 to 69·6)	24·4 (18·0 to 32·6)	17·9 (17·9 to 17·9)	309·3 (284·1 to 336·0)	214·9 (192·6 to 238·4)	55·2 (46·8 to 64·7)	22·7 (16·7 to 30·4)	16·6 (16·6 to 16·6)
Marshall Islands*	1997	333·8 (309·1 to 361·9)	232·2 (210·2 to 255·2)	57·1 (48·3 to 66·6)	24·5 (18·2 to 32·4)	20·0 (20·0 to 20·0)	310·5 (287·5 to 336·6)	216·0 (195·6 to 237·4)	53·1 (44·9 to 61·9)	22·8 (16·9 to 30·1)	18·6 (18·6 to 18·6)
Marshall Islands*	1998	339·6 (317·6 to 365·9)	229·7 (209·5 to 252·4)	54·9 (46·6 to 64·2)	24·5 (18·4 to 32·5)	30·5 (30·5 to 30·5)	315·9 (295·4 to 340·4)	213·7 (194·9 to 234·8)	51·1 (43·3 to 59·8)	22·8 (17·2 to 30·2)	28·3 (28·3 to 28·3)
Marshall Islands*	1999	425·8 (403·8 to 450·4)	231·2 (211·3 to 252·5)	53·8 (45·7 to 62·6)	24·7 (18·8 to 32·6)	116·1 (116·1 to 116·1)	396·1 (375·6 to 419·0)	215·1 (196·6 to 234·9)	50·0 (42·6 to 58·2)	23·0 (17·5 to 30·3)	108·0 (108·0 to 108·0)
Marshall Islands*	2000	382·6 (359·1 to 409·1)	265·2 (244·1 to 288·8)	56·5 (47·7 to 65·8)	27·5 (20·7 to 35·8)	33·4 (33·4 to 33·4)	355·9 (334·0 to 380·5)	246·7 (227·1 to 268·6)	52·5 (44·4 to 61·2)	25·6 (19·3 to 33·3)	31·1 (31·1 to 31·1)
Marshall Islands*	2001	363·6 (339·0 to 392·2)	269·5 (247·9 to 293·2)	57·7 (48·9 to 67·0)	28·0 (21·0 to 36·2)	8·5 (8·5 to 8·5)	338·3 (315·3 to 364·8)	250·7 (230·6 to 272·8)	53·6 (45·4 to 62·3)	26·0 (19·5 to 33·7)	7·9 (7·9 to 7·9)
Marshall Islands*	2002	343·6 (320·8 to 366·7)	238·6 (219·0 to 260·6)	56·8 (48·6 to 65·7)	26·9 (20·0 to 35·0)	21·2 (21·2 to 21·2)	319·6 (298·4 to 341·1)	222·0 (203·7 to 242·4)	52·8 (45·2 to 61·1)	25·0 (18·6 to 32·5)	19·7 (19·7 to 19·7)
Marshall Islands*	2003	352·5 (330·6 to 374·8)	206·3 (187·7 to 225·8)	56·1 (48·2 to 65·2)	25·6 (19·1 to 33·9)	64·5 (64·5 to 64·5)	327·9 (307·5 to 348·7)	191·9 (174·6 to 210·0)	52·2 (44·8 to 60·7)	23·8 (17·7 to 31·5)	60·0 (60·0 to 60·0)
Marshall Islands*	2004	541·7 (520·8 to 562·7)	196·2 (177·9 to 214·5)	58·4 (50·3 to 67·8)	25·0 (18·8 to 32·7)	262·1 (262·1 to 262·1)	503·9 (484·4 to 523·4)	182·6 (165·5 to 199·5)	54·3 (46·8 to 63·0)	23·2 (17·5 to 30·5)	243·8 (243·8 to 243·8)

Marshall Islands*	2005	706·1 (680·0 to 731·6)	238·7 (216·9 to 262·8)	70·2 (60·5 to 81·5)	27·1 (20·5 to 35·8)	370·0 (370·0 to 370·0)	656·8 (632·6 to 680·6)	222·1 (201·7 to 244·4)	65·3 (56·3 to 75·8)	25·2 (19·1 to 33·3)	344·2 (344·2 to 344·2)
Marshall Islands*	2006	458·1 (435·8 to 479·6)	190·2 (172·2 to 208·7)	67·7 (58·6 to 77·7)	23·8 (17·7 to 31·4)	176·4 (176·4 to 176·4)	426·2 (405·3 to 446·1)	177·0 (160·2 to 194·1)	63·0 (54·5 to 72·2)	22·1 (16·5 to 29·2)	164·1 (164·1 to 164·1)
Marshall Islands*	2007	498·4 (473·1 to 524·1)	218·9 (198·8 to 240·6)	72·0 (61·9 to 82·8)	24·7 (18·2 to 32·5)	182·9 (182·9 to 182·9)	463·6 (440·1 to 487·5)	203·6 (184·9 to 223·9)	66·9 (57·6 to 77·0)	23·0 (17·0 to 30·2)	170·1 (170·1 to 170·1)
Marshall Islands*	2008	501·1 (478·8 to 525·5)	197·5 (179·7 to 217·5)	69·2 (59·6 to 79·2)	22·9 (17·1 to 29·9)	211·5 (211·5 to 211·5)	466·1 (445·4 to 488·9)	183·7 (167·1 to 202·4)	64·4 (55·4 to 73·7)	21·3 (15·9 to 27·8)	196·7 (196·7 to 196·7)
Marshall Islands*	2009	514·3 (491·2 to 539·6)	196·4 (178·5 to 217·0)	69·8 (60·1 to 80·8)	22·7 (16·6 to 29·8)	225·4 (225·4 to 225·4)	478·4 (456·9 to 501·9)	182·7 (166·0 to 201·8)	64·9 (55·9 to 75·2)	21·1 (15·4 to 27·7)	209·6 (209·6 to 209·6)
Marshall Islands*	2010	313·3 (289·8 to 338·4)	196·4 (178·4 to 217·1)	70·3 (60·3 to 81·0)	22·1 (16·3 to 29·5)	24·4 (24·4 to 24·4)	291·4 (269·6 to 314·8)	182·7 (165·9 to 202·0)	65·4 (56·1 to 75·4)	20·6 (15·2 to 27·4)	22·7 (22·7 to 22·7)
Marshall Islands*	2011	326·0 (303·3 to 351·2)	205·6 (186·5 to 227·3)	70·7 (60·7 to 81·6)	21·3 (15·4 to 28·2)	28·5 (28·5 to 28·5)	303·3 (282·2 to 326·7)	191·3 (173·5 to 211·4)	65·8 (56·5 to 75·9)	19·8 (14·4 to 26·2)	26·5 (26·5 to 26·5)
Marshall Islands*	2012	486·0 (463·9 to 510·9)	209·5 (191·6 to 229·6)	65·4 (56·4 to 75·1)	20·1 (14·6 to 26·9)	191·0 (191·0 to 191·0)	452·1 (431·5 to 475·2)	194·9 (178·2 to 213·6)	60·8 (52·5 to 69·9)	18·7 (13·6 to 25·0)	177·7 (177·7 to 177·7)
Marshall Islands*	2013	331·2 (307·7 to 354·7)	231·1 (211·1 to 251·6)	64·8 (56·3 to 74·9)	20·5 (14·7 to 27·3)	14·9 (14·9 to 14·9)	308·1 (286·3 to 330·0)	215·0 (196·4 to 234·0)	60·3 (52·4 to 69·6)	19·0 (13·7 to 25·4)	13·9 (13·9 to 13·9)
Marshall Islands*	2014	423·7 (401·9 to 445·4)	215·8 (197·5 to 234·7)	62·2 (53·7 to 71·5)	19·3 (14·0 to 25·7)	126·4 (126·4 to 126·4)	394·1 (373·9 to 414·3)	200·8 (183·8 to 218·4)	57·8 (50·0 to 66·5)	18·0 (13·1 to 23·9)	117·6 (117·6 to 117·6)
Marshall Islands*	2015	403·2 (380·1 to 427·2)	240·0 (219·8 to 260·1)	65·0 (56·0 to 74·3)	19·7 (14·1 to 26·2)	78·4 (78·4 to 78·4)	375·1 (353·6 to 397·4)	223·3 (204·5 to 242·0)	60·5 (52·1 to 69·1)	18·3 (13·1 to 24·3)	73·0 (73·0 to 73·0)
Marshall Islands*	2016	342·4 (318·8 to 367·6)	249·3 (228·0 to 270·3)	68·5 (58·6 to 78·4)	19·1 (13·6 to 25·6)	5·6 (5·6 to 5·6)	318·5 (296·5 to 342·0)	231·9 (212·1 to 251·5)	63·7 (54·5 to 72·9)	17·7 (12·7 to 23·8)	5·2 (5·2 to 5·2)
Marshall Islands*	2017	412·6 (387·8 to 441·0)	272·9 (250·3 to 298·4)	71·9 (61·4 to 83·2)	19·3 (13·4 to 25·7)	48·5 (48·5 to 48·5)	383·8 (360·7 to 410·2)	253·8 (232·8 to 277·6)	66·9 (57·2 to 77·4)	17·9 (12·5 to 23·9)	45·1 (45·1 to 45·1)
Marshall Islands*	2018	494·2 (463·9 to 528·9)	288·9 (260·5 to 318·6)	73·3 (61·9 to 85·8)	19·5 (13·5 to 25·9)	112·4 (112·4 to 112·4)	459·7 (431·5 to 492·0)	268·8 (242·3 to 296·4)	68·2 (57·6 to 79·8)	18·2 (12·5 to 24·1)	104·5 (104·5 to 104·5)

Marshall Islands*	2019	536·2 (499·3 to 579·8)	334·9 (299·7 to 374·4)	75·9 (64·0 to 88·7)	21·0 (14·5 to 28·1)	104·5 (102·8 to 106·1)	498·8 (464·5 to 539·4)	311·6 (278·8 to 348·3)	70·6 (59·5 to 82·5)	19·5 (13·5 to 26·1)	97·2 (95·6 to 98·7)
Marshall Islands*	2020	621·1 (571·6 to 675·8)	399·4 (350·4 to 453·1)	74·3 (62·7 to 87·1)	21·2 (14·7 to 28·2)	126·2 (124·0 to 129·2)	577·8 (531·7 to 628·7)	371·5 (325·9 to 421·5)	69·1 (58·3 to 81·0)	19·7 (13·7 to 26·2)	117·4 (115·4 to 120·2)
Marshall Islands*	2021	605·0 (555·2 to 659·2)	396·4 (347·8 to 449·7)	73·6 (62·0 to 86·2)	21·6 (15·1 to 28·7)	113·4 (110·9 to 117·1)	562·8 (516·5 to 613·2)	368·8 (323·5 to 418·3)	68·5 (57·7 to 80·2)	20·1 (14·0 to 26·7)	105·5 (103·2 to 108·9)
Marshall Islands*	2022	526·9 (476·9 to 583·8)	342·3 (293·7 to 397·1)	74·2 (62·5 to 86·8)	22·3 (15·6 to 29·5)	88·2 (83·1 to 93·7)	490·2 (443·7 to 543·1)	318·4 (273·2 to 369·4)	69·1 (58·1 to 80·8)	20·7 (14·6 to 27·5)	82·0 (77·3 to 87·2)
Marshall Islands*	2023	536·3 (483·2 to 600·1)	348·2 (296·7 to 410·2)	74·9 (63·1 to 87·6)	23·0 (16·1 to 30·7)	90·2 (84·6 to 96·2)	498·8 (449·5 to 558·2)	323·9 (276·0 to 381·6)	69·7 (58·7 to 81·5)	21·4 (15·0 to 28·5)	83·9 (78·7 to 89·5)
Marshall Islands*	2024	545·5 (486·7 to 613·5)	354·1 (297·2 to 421·5)	75·7 (63·6 to 88·5)	23·7 (16·7 to 31·6)	92·1 (86·1 to 98·8)	507·4 (452·7 to 570·7)	329·4 (276·5 to 392·1)	70·4 (59·2 to 82·3)	22·0 (15·5 to 29·4)	85·6 (80·1 to 91·9)
Marshall Islands*	2025	554·2 (492·6 to 627·5)	359·6 (299·6 to 430·5)	76·4 (64·4 to 89·4)	24·4 (17·2 to 32·5)	93·7 (87·5 to 100·7)	515·5 (458·2 to 583·7)	334·5 (278·7 to 400·5)	71·1 (59·9 to 83·1)	22·7 (16·0 to 30·3)	87·2 (81·4 to 93·7)
Marshall Islands*	2026	563·2 (496·7 to 641·0)	365·5 (300·2 to 441·4)	77·2 (65·0 to 90·3)	25·2 (17·8 to 33·6)	95·2 (88·5 to 102·8)	523·9 (462·0 to 596·3)	340·0 (279·3 to 410·6)	71·8 (60·5 to 84·0)	23·4 (16·5 to 31·3)	88·6 (82·4 to 95·6)
Marshall Islands*	2027	574·7 (506·1 to 655·1)	374·1 (306·0 to 453·3)	78·0 (65·7 to 91·3)	26·0 (18·3 to 34·7)	96·6 (89·2 to 105·0)	534·6 (470·8 to 609·4)	348·0 (284·6 to 421·7)	72·6 (61·1 to 84·9)	24·2 (17·0 to 32·3)	89·9 (83·0 to 97·7)
Marshall Islands*	2028	586·4 (514·9 to 668·9)	382·9 (310·6 to 463·1)	78·8 (66·4 to 92·2)	26·8 (19·0 to 35·7)	97·9 (90·1 to 106·7)	545·5 (478·9 to 622·2)	356·2 (288·9 to 430·8)	73·3 (61·7 to 85·8)	24·9 (17·6 to 33·2)	91·1 (83·8 to 99·3)
Marshall Islands*	2029	597·6 (522·5 to 685·2)	391·2 (318·2 to 477·1)	79·6 (67·1 to 93·2)	27·7 (19·6 to 37·0)	99·1 (90·5 to 108·5)	555·9 (486·1 to 637·4)	363·9 (296·0 to 443·8)	74·1 (62·4 to 86·7)	25·7 (18·2 to 34·4)	92·2 (84·2 to 100·9)
Marshall Islands*	2030	608·1 (531·4 to 698·8)	399·0 (324·6 to 489·3)	80·5 (67·8 to 94·3)	28·6 (20·2 to 38·2)	100·1 (90·9 to 109·8)	565·7 (494·3 to 650·0)	371·2 (302·0 to 455·2)	74·9 (63·1 to 87·7)	26·6 (18·8 to 35·5)	93·1 (84·6 to 102·2)
Marshall Islands*	2031	616·7 (536·9 to 711·4)	404·6 (325·6 to 499·4)	81·2 (68·5 to 95·2)	29·5 (20·8 to 39·4)	101·4 (91·7 to 112·0)	573·7 (499·4 to 661·7)	376·4 (302·9 to 464·6)	75·6 (63·7 to 88·6)	27·4 (19·3 to 36·7)	94·4 (85·3 to 104·2)
Marshall Islands*	2032	626·5 (541·8 to 730·3)	411·2 (326·6 to 514·8)	82·0 (69·1 to 96·1)	30·4 (21·3 to 40·7)	102·9 (92·9 to 113·5)	582·8 (504·0 to 679·3)	382·5 (303·8 to 478·9)	76·3 (64·3 to 89·4)	28·3 (19·8 to 37·9)	95·7 (86·4 to 105·6)

Marshall Islands*	2033	635·5 (549·5 to 742·2)	416·7 (332·5 to 524·7)	82·7 (69·7 to 96·9)	31·3 (22·0 to 42·1)	104·7 (94·0 to 116·0)	591·1 (511·1 to 690·4)	387·6 (309·3 to 488·1)	76·9 (64·9 to 90·1)	29·1 (20·4 to 39·2)	97·4 (87·4 to 107·9)
Marshall Islands*	2034	644·6 (554·4 to 750·3)	422·0 (334·1 to 527·7)	83·4 (70·3 to 97·7)	32·2 (22·5 to 43·4)	107·0 (95·8 to 119·1)	599·6 (515·7 to 698·0)	392·5 (310·8 to 490·8)	77·6 (65·4 to 90·9)	30·0 (20·9 to 40·4)	99·5 (89·1 to 110·8)
Marshall Islands*	2035	655·1 (561·1 to 773·6)	428·3 (338·2 to 540·5)	84·0 (70·8 to 98·4)	33·2 (23·1 to 44·8)	109·6 (98·1 to 122·6)	609·4 (521·9 to 719·7)	398·4 (314·6 to 502·8)	78·1 (65·8 to 91·6)	30·9 (21·5 to 41·7)	101·9 (91·3 to 114·0)
Marshall Islands*	2036	664·8 (565·9 to 786·8)	433·8 (338·5 to 553·6)	84·6 (71·3 to 99·2)	34·2 (23·6 to 46·1)	112·2 (99·9 to 126·3)	618·4 (526·4 to 731·9)	403·5 (314·9 to 515·0)	78·7 (66·3 to 92·3)	31·8 (22·0 to 42·9)	104·4 (92·9 to 117·5)
Marshall Islands*	2037	674·8 (569·6 to 799·9)	439·4 (335·7 to 564·7)	85·3 (71·8 to 99·9)	35·2 (24·3 to 47·7)	115·0 (102·5 to 130·0)	627·7 (529·9 to 744·1)	408·7 (312·3 to 525·3)	79·3 (66·8 to 93·0)	32·7 (22·6 to 44·3)	107·0 (95·3 to 121·0)
Marshall Islands*	2038	685·7 (578·2 to 822·9)	445·5 (340·9 to 572·8)	85·9 (72·3 to 100·7)	36·2 (24·9 to 49·4)	118·1 (105·0 to 134·6)	637·8 (537·9 to 765·5)	414·4 (317·1 to 532·8)	79·9 (67·3 to 93·6)	33·6 (23·2 to 46·0)	109·9 (97·7 to 125·2)
Marshall Islands*	2039	696·6 (586·0 to 839·6)	451·0 (340·2 to 593·4)	86·4 (72·7 to 101·3)	37·2 (25·7 to 51·0)	122·0 (107·8 to 139·8)	648·0 (545·1 to 781·0)	419·5 (316·5 to 552·0)	80·4 (67·7 to 94·2)	34·6 (23·9 to 47·4)	113·5 (100·2 to 130·1)
Marshall Islands*	2040	707·9 (591·9 to 862·1)	456·5 (343·0 to 603·1)	86·9 (73·1 to 101·9)	38·2 (26·3 to 52·2)	126·3 (110·6 to 145·4)	658·6 (550·6 to 801·9)	424·7 (319·1 to 561·1)	80·9 (68·0 to 94·8)	35·5 (24·5 to 48·6)	117·5 (102·9 to 135·2)
Marshall Islands*	2041	718·8 (600·1 to 873·9)	461·4 (344·4 to 613·0)	87·5 (73·6 to 102·6)	39·3 (26·8 to 53·8)	130·7 (114·1 to 151·0)	668·6 (558·3 to 813·0)	429·2 (320·4 to 570·2)	81·4 (68·4 to 95·4)	36·5 (25·0 to 50·1)	121·6 (106·1 to 140·5)
Marshall Islands*	2042	730·3 (604·5 to 892·4)	466·3 (342·3 to 618·3)	88·0 (73·9 to 103·2)	40·3 (27·6 to 55·6)	135·8 (117·2 to 159·2)	679·4 (562·3 to 830·1)	433·7 (318·4 to 575·2)	81·8 (68·8 to 96·0)	37·5 (25·6 to 51·7)	126·3 (109·1 to 148·1)
Marshall Islands*	2043	742·2 (612·4 to 906·2)	471·4 (344·9 to 630·1)	88·5 (74·4 to 104·0)	41·4 (28·4 to 57·3)	140·8 (121·6 to 166·4)	690·4 (569·7 to 843·0)	438·5 (320·9 to 586·1)	82·4 (69·2 to 96·7)	38·5 (26·4 to 53·3)	131·0 (113·1 to 154·8)
Marshall Islands*	2044	755·1 (624·3 to 926·4)	477·0 (345·3 to 640·5)	89·1 (74·9 to 104·7)	42·6 (29·1 to 58·8)	146·3 (126·0 to 172·9)	702·4 (580·7 to 861·8)	443·8 (321·2 to 595·8)	82·9 (69·7 to 97·4)	39·6 (27·1 to 54·7)	136·1 (117·2 to 160·8)
Marshall Islands*	2045	767·2 (632·7 to 938·8)	481·6 (348·8 to 645·2)	89·7 (75·4 to 105·4)	43·7 (29·6 to 60·5)	152·2 (130·3 to 182·7)	713·7 (588·5 to 873·3)	448·0 (324·5 to 600·2)	83·4 (70·1 to 98·1)	40·7 (27·6 to 56·3)	141·6 (121·2 to 170·0)
Marshall Islands*	2046	780·6 (645·4 to 957·8)	487·1 (351·4 to 653·1)	90·3 (75·8 to 106·1)	45·0 (30·4 to 62·6)	158·3 (134·5 to 190·6)	726·2 (600·4 to 891·0)	453·1 (326·9 to 607·5)	84·0 (70·5 to 98·7)	41·8 (28·2 to 58·2)	147·3 (125·1 to 177·3)

Marshall Islands*	2047	794·3 (652·0 to 967·8)	492·5 (349·5 to 659·9)	90·8 (76·2 to 106·9)	46·2 (31·3 to 64·8)	164·7 (139·0 to 200·0)	738·9 (606·5 to 900·3)	458·2 (325·1 to 613·9)	84·5 (70·9 to 99·4)	43·0 (29·1 to 60·3)	153·2 (129·3 to 186·0)
Marshall Islands*	2048	807·7 (661·5 to 995·7)	497·5 (348·2 to 675·9)	91·4 (76·7 to 107·7)	47·5 (32·0 to 66·7)	171·3 (144·4 to 208·4)	751·4 (615·4 to 926·2)	462·8 (323·9 to 628·8)	85·1 (71·3 to 100·2)	44·2 (29·8 to 62·1)	159·3 (134·3 to 193·9)
Marshall Islands*	2049	822·0 (671·3 to 1013·0)	502·6 (359·8 to 685·4)	92·0 (77·2 to 108·4)	48·8 (32·9 to 68·5)	178·5 (149·0 to 221·3)	764·6 (624·5 to 942·4)	467·5 (334·7 to 637·6)	85·6 (71·8 to 100·9)	45·4 (30·6 to 63·7)	166·0 (138·6 to 205·9)
Marshall Islands*	2050	836·6 (682·6 to 1036·4)	507·4 (356·3 to 701·3)	92·6 (77·7 to 109·2)	50·1 (33·6 to 71·1)	186·5 (153·5 to 233·4)	778·3 (635·0 to 964·1)	472·0 (331·5 to 652·4)	86·1 (72·3 to 101·5)	46·6 (31·3 to 66·1)	173·5 (142·8 to 217·1)
North Macedonia*	1995	305·4 (281·7 to 330·6)	175·4 (158·4 to 193·1)	121·3 (104·0 to 138·7)	2·2 (1·4 to 3·1)	6·5 (6·5 to 6·5)	842·9 (777·3 to 912·4)	484·1 (437·0 to 532·8)	334·8 (287·1 to 382·7)	6·0 (4·0 to 8·4)	18·0 (18·0 to 18·0)
North Macedonia*	1996	308·3 (286·7 to 329·7)	179·8 (165·3 to 194·8)	122·3 (108·1 to 137·8)	2·3 (1·6 to 3·3)	3·9 (3·9 to 3·9)	850·7 (791·3 to 909·8)	496·1 (456·2 to 537·5)	337·4 (298·3 to 380·3)	6·4 (4·3 to 9·1)	10·9 (10·9 to 10·9)
[truncated: 640,272 more chars]
